# Identification of key genes involved in the recurrence of glioblastoma multiforme using weighted gene co-expression network analysis and differential expression analysis

**DOI:** 10.1080/21655979.2021.1943986

**Published:** 2021-07-08

**Authors:** Peng Ren, JingYa Wang, Lei Li, XiaoWan Lin, GuangHan Wu, JiaYi Chen, ZhiRui Zeng, HongMei Zhang

**Affiliations:** a Department of Anesthesiology, The First Affiliated Hospital of Shandong First Medical University & Shandong Provincial Qianfoshan Hospital, Jinan, Shandong, China; b Department of Gastroenterology, The Affiliated Hospital of Weifang Medical University, Weifang, Shandong, China; c Department of Physiology of Basic Medicine College, Guizhou Medical University, Guiyang, Guizhou, China; d Department of Anesthesiology, Beijing Shijitan Hospital, Capital Medical University, Beijing, China

**Keywords:** Glioblastoma multiforme, wgcna, deg analysis, recurrence

## Abstract

Glioblastoma multiforme (GBM) is the most fatal malignancy, and despite extensive treatment, tumors inevitably recur. This study aimed to identify recurrence-associated molecules in GBM. The gene expression profile GSE139533, containing 70 primary and 47 recurrent GBM tissues and their corresponding clinical traits, was downloaded from the Gene Expression Omnibus (GEO) database and used for weighted gene co-expression network analysis (WGCNA) and differentially expressed gene (DEG) analysis. After identifying the hub genes which differentially expressed in recurrent GBM tissues and in the gene modules correlated with recurrence, data from the Chinese Glioma Genome Atlas (CCGA) and The Cancer Genome Atlas (TCGA) databases were analyzed with GSE43378 to determine the relationship between hub genes and patient prognosis. The diagnostic value of the identified hub genes was verified using 52 GBM tissues. Three gene modules were correlated with recurrence and 2623 genes were clustered in these clinically significant modules. Among these, 13 genes – *EHF, TRPM1, FXYD4, CDH15, LHX5, TP73, FBN3, TLX1, C1QL4, COL2A, SEC61G, NEUROD4* and *GPR139* – were differentially expressed in recurrent GBM samples; low *LHX5* and *TLX1* expression predicted poor outcomes. *LHX5* and *TLX1* expression showed weak positive relationships with Karnofsky performance scale scores. Additionally, *LHX5* and *TLX1* expression was found to be decreased in our recurrent GBM samples compared with that in primary samples; these genes exhibited high diagnostic value in distinguishing recurrent samples from primary samples. Our findings indicate that *LHX5* and *TLX1* might be involved in GBM recurrence and act as potential biomarkers for this condition.

## Graphical abstract

**Figure:**
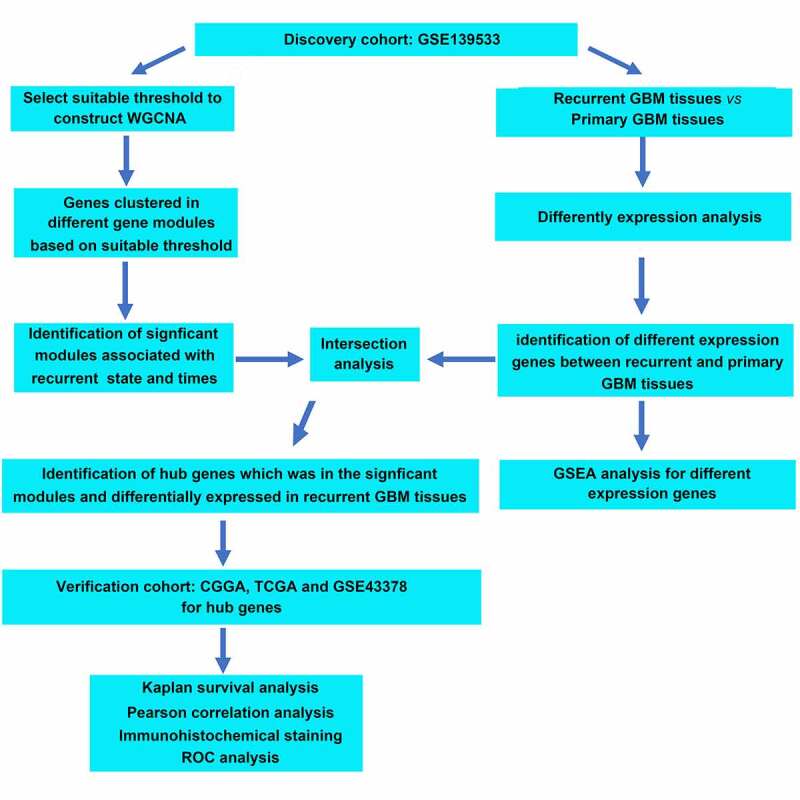

## Introduction

Glioblastoma multiforme (GBM) is a common malignancy in the brain, with a distinctly low life expectancy after diagnosis [1]. Despite considerable efforts being made in molecular understanding and disease therapy, patient survival remains at a dismally low rate, with a median survival time is 14–16 months with a 5-year survival of 9.8% [2,3]. Particularly, as GBM is a highly malignant brain tumor and lacks an effective prevention strategy, it inevitably progresses or recurs after the first-line standard of care. There is no consensus regarding the best treatment to offer people upon disease progression or recurrence [4]. Tumor recurrence affects approximately 90% of patients, and it is a significant barrier to the increase in overall survival following effective surgery [5]. Hence, a more intensive understanding of the mechanisms underlying GBM may contribute to the diagnosis and therapy of GBM recurrence.

Bioinformatic methods, including analysis of GBM microarray and high-throughput sequencing, are effective strategies for the exploration of therapeutic targets in GBM [6–8]. Weighted gene co-expression network analysis (WGCNA) is a potent tool for uncovering complex mechanisms and multigene analysis of high-throughput data, particularly for exploring the relationship between genes and traits of samples [9–11]. In the WGCNA network, the average expression level of genes in the significant modules was determined and modules containing highly related genes were explored; thus, clusters can be generalized by a module eigengene or module core gene, module relationships with the traits of samples can be determined, and module membership measures can be calculated [12–14]. For instance, Zhang *et al*. [15] identified 24 genes, including vimentin (*VIM*), chloride intracellular channel 1 (*CLIC1*), and tubulin beta 6 (*TUBB6*), which were associated with the tumor grade and prognosis in GBM. Similarly, utilizing WGCNA, Chen et al. [16] identified 22 genes, including *BUB1*, cyclin B2 (*CCNB2*), kinesin family member 20A (*KIF20A*), and nucleolar and spindle associated protein 1 (*NUSAP1*), which are potential biomarkers in glioma.

LIM homeobox 5 (LHX5), a member of the LIM homeobox (LHX) family of transcription factors identified in 1995 [17], is highly expressed in the caudal hypothalamus and acts as an important differentiation mediator [18]. LHX5 also plays a key role in development of the forebrain and hippocampus, while deficiency of LHX5 induces a variety of brain abnormalities [19]. Moreover, low expression of LHX5 has been observed in breast cancer [20]. T cell leukemia homeobox 1 (TLX1), a nuclear transcription factor of the NK-linked or NK-like (NKL) subfamily of homeobox genes, was identified in 1993 [21] and is involved in the specification of neuronal cell fates [22]. TLX1 is also required for normal development of the spleen during embryogenesis [23]. However, the roles of LHX5 and TLX1 in GBM are still unknown.

The present study aimed to identify novel recurrence-associated molecular markers in GBM. By combining WGCNA, differentially expressed genes (DEGs), and experimental verification, it was demonstrated that LHX5 and TLX1 were clustered in the gene modules associated with recurrence and showed decreased expression in recurrent samples compared with that in primary GBM tissues. Lower LHX5 and TLX1 expression predicted poor outcomes, and both LHX5 and TLX1 had high diagnostic value in distinguishing recurrent samples. Therefore, we suggest that LHX5 and TLX1 may be recurrence-associated molecules in GBM, as well as effective biomarkers for predicting the recurrence of GBM.

## Materials and methods

### Preconditioned data profile used for WGCNA

The gene expression profile GSE139533, including the corresponding clinical traits (recurrence state and recurrence time), was downloaded from the Gene Expression Omnibus (GEO) database (https://www.ncbi.nlm.nih.gov/gds). The data profile was contributed by Ella *et al*. [24] and performed on the GPL18573 platform. Before performing WGCNA, the data profile was pre-processed as follows: the data were converted to log_2_ values; the probes of the profile were mapped to the gene names according to the information provided by the GPL18573 platform; and the null probes and low-abundance genes with a mean expression < 0.5 were removed. The gene expression data of 16,043 genes were then analyzed with WGCNA.

### WGCNA

To perform WGCNA [12], the gene expression data of 16,043 genes in 117 GBM tissue samples, along with their corresponding clinical characteristics (recurrence state and recurrence time; recurrence state: 0 for GBM without recurrence and 1 for GBM with recurrence; recurrence time: 0–2 times), were imported into R software v.4.0.2 (https://www.r-project.org/). First, all the samples were clustered via hclust to identify the outliers with a cutoff value of 300. The outliers need to be removed before performing WGCNA. Then, Pearson’s correlation analysis was employed to assess the relationships between the gene pairs, the results of which were used to construct a matrix of similarity. Subsequently, WGCNA was performed to cluster the genes in co-expression modules using an appropriate soft power that ensured that the scale independence was greater than 0.85 and mean connectivity was close to 0. The dynamic tree cutting algorithm was used to define modules by cutting the clustering tree into branches, following which the modules were assigned to different colors for visualization.

### Identification of clinically significant modules

The relationship between different module eigengenes and patient characteristics (recurrence state and recurrence time) was assessed using Pearson’s correlation analysis. Modules were considered clinically significant if they were correlated with the above-mentioned two clinical characteristics (r > 0.3, *P* < 0.05). In the clinically significant modules, gene significance (GS) was calculated based on the relationship between each gene and the characteristics of interest, while the module membership (MM) was measured by analyzing the correlation between the module eigengenes to confirm that they were significant modules. If *P* < 0.05, the correlations between GS and MM in the clinically significant modules were regarded as real clinically significant modules and subjected to further analysis. Moreover, the correlation intensity was analyzed using the cutoff values as follows: very strong relationship (0.80 < r ≤ 1.0), strong relationship (0.60 < r ≤ 0.80), moderate relationship (0.4 < r ≤ 0.6), weak relationship (0.2 < r ≤ 0.4), and very weak or no relationship (0 < r ≤ 0.20).

### Screening of DEGs

For DEG analysis, gene expression data were imported into the R software, and the limma package (https://www.bioconductor.org/) [25] was employed to identify DEGs between the 70 primary and 47 recurrent GBM tissues. *P* < 0.05, and |log-fold change (LogFC)|>2 were used to consider differentially expressed genes.

### Gene set enrichment analysis (GSEA)

In the gene expression profile GSE139533, the GBM samples were divided into primary and recurrent tissue groups. To identify and distinguish the functions of DEGs between the primary and recurrent tissues, GSEA (https://software.broad institute. org/gsea/index.jsp) [26] was performed to determine the biological processes that were enriched in the gene rank derived from DEGs between the two groups. Enriched terms with false discovery rate (FDR) < 0.05 were significant.

### Survival analysis of hub genes

To verify the value of predicting the prognosis of hub genes, Kaplan–Meier survival analysis was performed according to the data obtained from the Chinese Glioma Genome Atlas (CCGA) (http://www.cgga.org.cn/) [27]. Based on the median gene expression values, patients in the CCGA database were divided into high and low expression groups. *P* < 0.05 was set as the cutoff value to determine the significant differences between the high and low expression groups.

### Analysis of the relationship between the expression levels of hub genes and the Karnofsky performance scale (KPS) scores in GBM patients

Two gene expression profiles containing KPS scores for GBM patients were downloaded from The Cancer Genome Atlas (TCGA) (https://xenabrowser.net/datapages/) [28] and GEO (https://www.ncbi.nlm.nih.gov/gds; Accession: GSE43378) databases [29]. After normalization, the two gene profiles were merged using sva package v.3.12 (https://www.bioconductor.org/) [30]. Thereafter, the expression levels of LHX5 and TLX1 and the corresponding KPS scores for GBM patients were extracted. Finally, Pearson’s correlation analysis was performed to determine the relationship between the expression levels of *LHX5* and *TLX1* and the KPS scores in GBM patients. Statistical significance was set at *P* < 0.05.

### Tissue collection

GBM tissues were obtained from the samples of 52 patients with GBM, aged 39–72 years, which were collected from the Affiliated Hospital of Weifang Medical University (Shandong, China). Among these, 36 were primary and 16 were recurrent GBM samples. All samples were diagnosed as GBM by two pathologists according to the following criteria: (1) the cells in tissues exhibited high heteromorphosis; (2) the tissues were disordered and exhibited high heteromorphosis; (3) no normal neurons with long spindle type nerve fibers were found in the tissues; and (4) there was a high density of cells in the tissues. The tissues were collected between June 2017 and July 2020. None of the patients received chemotherapy or radiotherapy prior to tissue collection. All patients signed the informed consent forms, and the Human Research Ethics Review Committee of the Affiliated Hospital of Weifang Medical University approved and administered the use of collected tissues.

### Immunohistochemistry (IHC)

All GBM tissues were fixed with 4% paraformaldehyde (Cat no. 30,525–89-4, Sigma-Aldrich, USA). The tissues were then embedded in paraffin and cut into 4 μm thick sections. After heating, the tissues were deparaffinized and rehydrated using graded xylene and ethanol. After antigen retrieval using sodium citrate (Servicebio, Wuhan, China), the endogenous peroxidases in the tissue sections were blocked using hydrogen peroxide (H_2_O_2_). Next, 5% bovine serum albumin (Wuhan Boster Biological Technology Ltd.) was added to block nonspecific binding. The sections were then incubated with the following primary antibodies overnight at 4°C: LHX5 (1:20 dilution; cat no: ab187975; Abcam, USA) and TLX1 (1:50 dilution; cat no: 26,877-1-AP; Proteintech, Wuhan, China). Subsequently, the sections were incubated for 2 h with anti-mouse and anti-rabbit horseradish peroxidase (HRP)-conjugated goat secondary antibodies (Servicebio, Wuhan, China). The sections were then stained with 3,3ʹ-diaminobenzidine (DAB) and hematoxylin (ZSGB-BIO, Beijing, China), and images were obtained using an orthophoto microscope (Version: BX53; Olympus, Japan). Protein levels of the targets were calculated based on the product of the intensity scores (0, no staining; 1, +; 2, ++; 3, +++) and percentage of positive cells (0, 0–1%; 1, 1–33%; 2, 34–66%; 3, 67–100%).

### Verification of diagnostic value of genes

Diagnostic value of the genes was analyzed using receiver operating characteristic (ROC) curve analysis according to IHC-based protein levels. The protein expression level scores of tissues were imported into SPSS software v.20.0 (https://www.ibm.com/analytics/spss-statistics-software) and an ROC curve analysis was performed. Genes with area under the curve (AUC) > 0.7 were considered to possess high diagnostic value.

### Statistical analysis

The IHC results were analyzed in SPSS (version 20.0) using the chi-square test. Differences among the IHC scores in each group were determined using a cutoff value of *P* < 0.05.

## Results

After performing WGCNA based on the gene expression profile GSE139533 and its corresponding traits, the genes were clustered into 17 co-expression modules; three gene modules (blue, royal blue, and dark turquoise) were significantly associated with GBM recurrence; a total of 2613 genes were present in these three modules. Then, through DEG analysis, a total of 232 genes were found to be differentially expressed between GBM recurrent tissues and primary tissues. After intersection analysis, we found that 13 genes, including ETS homologous factor (*EHF*), transient receptor potential cation channel subfamily M member 1 (*TRPM1*), FXYD domain containing ion transport regulator 4 (*FXYD4*), cadherin 15 (*CDH15*), LHX5, tumor protein p73 (*TP73*), fibrillin 3 (*FBN3*), TLX1, complement C1q-like 4 (*C1QL4*), collagen type II alpha chain (*COL2A*), SEC61 translocon subunit gamma (*SEC61G*), neuronal differentiation 4 (*NEUROD4*), and G protein-coupled receptor 139 (*GPR139*), were simultaneously presented in the gene modules associated with GBM recurrence and were differentially expressed in the recurrent GBM tissues. Among them, low expression levels of *TLX1* and *LHX5* predicted poor overall survival rate of GBM patients and low KPS scores; both two genes showed high diagnostic value in distinguishing the recurrent GBM samples from the primary GBM samples.

### WGCNA

**Figure 1.**
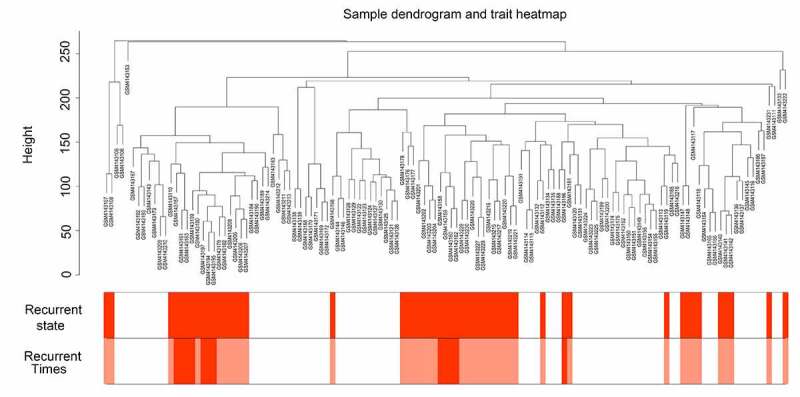
Sample tree clustering and clinical trait heatmap of 70 primary and 47 recurrent glioblastoma multiforme (GBM) samples. For sample tree clustering, there were no samples with height > 300, and all samples were used for the weighted gene co-expression network analysis (WGCNA). For the construction of the clinical trait heatmap, the recurrence state contained two parts classified as with or without recurrence (with recurrence is shown in red, and without recurrence is shown in white); recurrence time contained three parts classified as non-recurrence, recurrence once, and recurrence twice (non-recurrence is shown in white, recurrence once is shown in pink, and recurrence twice is shown in red). The clinical trait heatmap shows the information of all the traits. All the traits could be used for WGCNA

**Figure 2.**
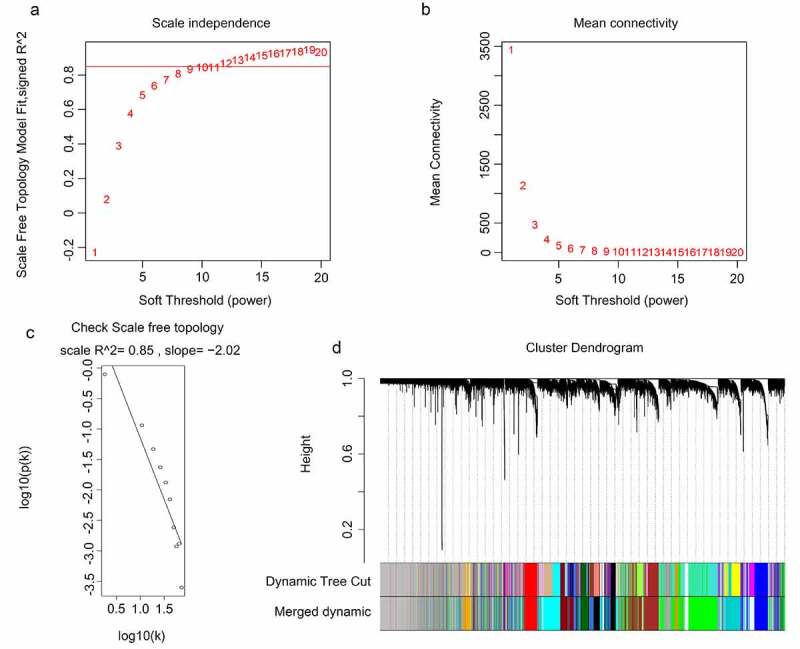
WGCNA for the gene expression profiles of 117 GBM tissues. (a-b) Scale independence and mean connectivity of various soft-threshold values (β). Red numbers indicate the different soft threshold values (1–20), while the red lines indicate the selected cutoff values, as the scale independence > 0.85. (c) Gene sets with the corresponding log_10_ and log_10_ *P*-values when the scale-free topology is set as β = 10. (d) Clustering dendrograms of all genes with dissimilarities based on topological overlap, together with their assigned module colors

The gene expression data of 117 GBM tissues and the corresponding clinical characteristic data were used to perform the WGCNA. The sample dendrogram revealed that there were no outliers (samples with height > 300); the trait heatmap showed that the clinical data of all patients were completely documented, and that these traits could be used for WGCNA (Figure 1). Then, the soft power β was selected as 10 to perform WGCNA, which ensured scale independence > 0.85, as shown in Figure 2a, and mean connectivity close to 0 (Figure 2(b)). Furthermore, the results revealed that when β = 10, the topological overlap matrix met the scale-free topology criterion, with R^2^ = 0.85 (Figure 2(c)). These results all indicated that β = 10 was suitable, and we used β = 10 to construct WGCNA. Finally, a total of 17 gene co-expression modules (blue, light cyan, dark red, cyan, dark magenta, orange, dark gray, brown, dark olive green, red, royalblue, black, darkgreen, darkturquoise, lightgreen, darkorange, and green) were identified, while the genes without co-expression relationships were all clustered together in the gray module (Figure 2d). Detailed information about the genes in each module is available in Supplementary STable 1.

### Identification of clinically significant modules

**Figure 3.**
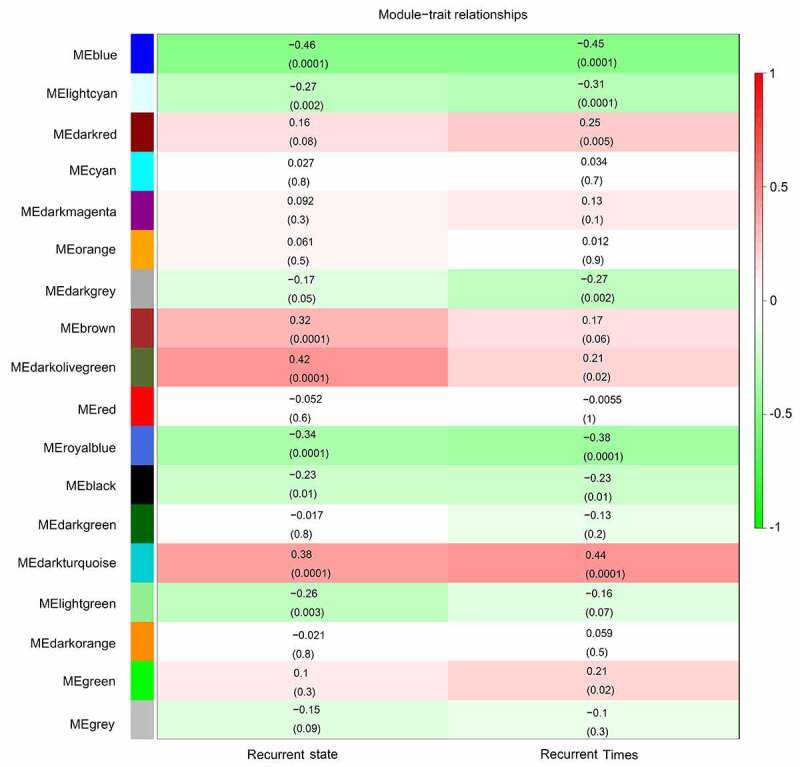
Identification of significant modules associated with the clinical traits (recurrence state and recurrence time). Each cell in the heat map contains the corresponding correlation score and *P*-value between gene modules and clinical traits. Red indicates positive correlation, and green indicates negative correlation

**Figure 4.**
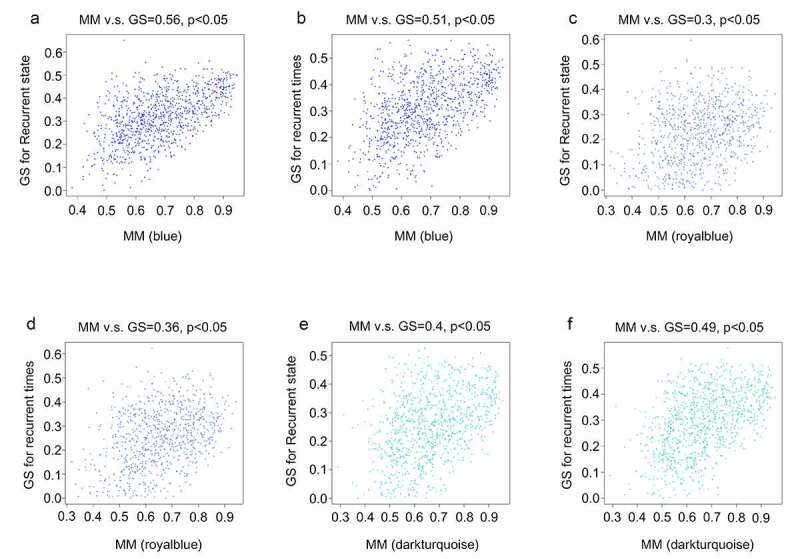
Relationship between gene significance (GS) and module membership (MM) in the significant modules. (a) Relationship between the MM in blue and GS for recurrence state; (b) Relationship between the MM in blue and GS for recurrence time; (c) Relationship between MM in royal blue and GS for recurrence state; (d) Relationship between the MM in royal blue and GS for recurrence time; (e) Relationship between the MM in dark turquoise and GS for recurrence state; (f) Relationship between the MM in dark turquoise and GS for recurrence time

After obtaining information on gene modules, we determined which gene modules were associated with recurrence state and recurrence times. It was found that three gene co-expression modules (blue, royalblue, and darkturquoise module) were simultaneously and significantly correlated with two clinical characteristics (recurrence state and recurrence times) in patients with GBM. The gene of the blue module was negatively and moderately correlated with the recurrence state (R = – 0.46, *P* = 0.0001) and recurrence times (R = – 0.45, *P* = 0.0001) (Figure 3). Genes of the royalblue module were negatively and weakly correlated with the recurrence state (R = – 0.34, *P* = 0.0001) and recurrence times (R = – 0.38, *P* = 0.0001) (Figure 3). Additionally, genes in the darkturquoise module had a weak positive relationship with recurrence (R = 0.38, *P* = 0.0001) and a moderate positive relationship with recurrence times (R = 0.44, *P* = 0.0001) (Figure 3). Correlations between GS and MM were subsequently calculated in the three aforementioned modules to further confirm that they were real clinically significant modules. The MM of the blue module was moderately correlated with the GS for recurrence state (correlation = 0.56, *P* < 0.05) and recurrence times (correlation = 0.51, *P* < 0.05) (Figure 4(a, b)). The MM of the royalblue module was weakly correlated with the GS for recurrence state (correlation = 0.3, *P* < 0.05) and recurrence times (correlation = 0.36, *P* < 0.05) (Figure 4(c, d)). The MM of the darkturquoise module was moderately correlated with the GS for recurrence state (correlation = 0.4, *P* < 0.05) and recurrence times (correlation = 0.49, *P* < 0.05) (Figure 4(e, f)). Accordingly, these three modules (i.e., blue, royalblue, and darkturquoise modules) were set as real clinically significant modules and were analyzed further.

### Identification of DEGs in the primary and recurrent GBM samples

**Figure 5.**
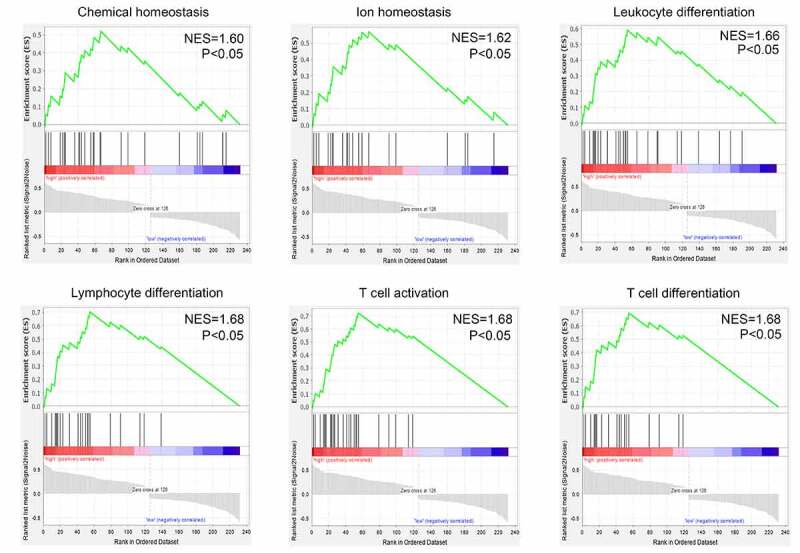
Gene set enrichment analysis (GSEA) for the enriched biological terms of differentially expressed genes between 70 primary and 47 recurrent GBM tissues

After performing the DEG analysis, a total of 126 upregulated and 106 downregulated genes were identified in the recurrent GBM tissues compared with those in the primary GBM tissues (Supplementary Table 2). To identify the biological functions of DEGs, GSEA was conducted; we found that the DEGs were enriched in ‘chemical homeostasis,’ ‘ion homeostasis,’ ‘leukocyte differentiation,’ ‘lymphocyte differentiation,’ ‘T cell activation,’ and ‘T cell differentiation’ (FDR < 0.05; Figure 5). Genes in each enriched term of biological function are shown in Supplementary Table 3.

### Identification of hub genes involved in the recurrence of GBM

**Figure 6.**
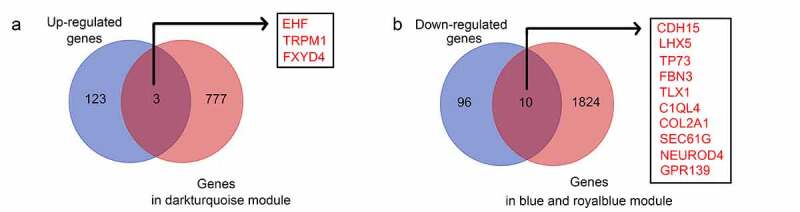
Intersection analysis for the genes in significant modules and differentially expressed genes. (a) Intersection analysis for the genes in significant modules positively associated with recurrence and upregulated genes in recurrent GBM tissues; (b) Intersection analysis for the genes in significant modules negatively associated with recurrence and downregulated genes in recurrent GBM tissues

To identify hub genes that were differentially expressed in recurrent GBM tissues and presented in the modules significantly associated with recurrence state and recurrence times, intersection analysis was performed. We found that three genes (*EHF, TRPM1*, and *FXYD4*) were upregulated in recurrent GBM tissues and in the module (dark turquoise) positively associated with recurrence state and recurrence times (Figure 6(a)), whereas 10 genes (*CDH15, LHX5, TP73, FBN3, TLX1, C1QL4, COL2A, SEC61G, NEUROD4*, and *GPR139*) showed low expression in recurrent GBM tissues and in the modules (blue and royalblue) negatively associated with recurrence state and recurrence times (Figure 6(b)). Therefore, these 13 genes were identified as hub genes for GBM recurrence.

### *Low expression levels of* LHX5 *and* TLX1 *predicted a poor prognosis*

**Figure 7.**
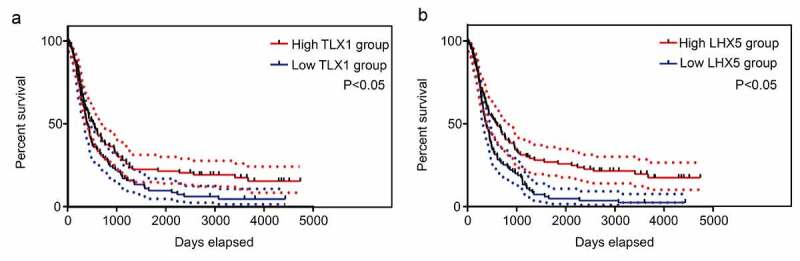
Kaplan–Meier survival analysis of the relationship between the expression of hub genes and the survival of GBM patients according to the data from the Chinese Glioma Genome Atlas (CGGA). The two red imaginary lines indicate 95% confidence interval (CI) for the high expression group, while the two blue imaginary lines indicate 95% CI for the low expression group

**Figure 8.**
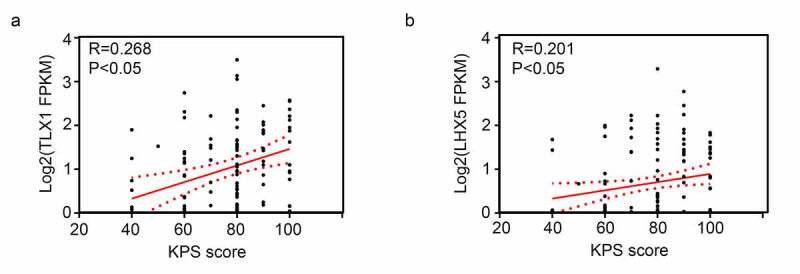
Pearson correlation analysis showed the expression levels of LIM homeobox 5 (LHX5) and T cell leukemia homeobox 1 (TLX1) were weakly and positively associated with the Karnofsky performance scale (KPS) scores

We then determined the relationship between the expression levels of 13 hub genes and patient survival rates, according to the data obtained from the CCGA database. The results showed that only *LHX5* and *TLX1* had significant value in predicting the survival rates of patients with GBM; patients with low expression levels of *LHX5* and *TLX1* had a lower survival rate than those with high expression levels of *LHX5* and *TLX1* (Figure 7). Similarly, using a merged verification cohort of data from TCGA and GSE43378, we found that the expression levels of *LHX5* and *TLX1* were weakly and positively associated with the KPS scores (Figure 8). Taken together, low expression levels of LHX5 and *TLX1* predicted a poor prognosis in GBM patients.

### LHX5 and TLX1 expression was decreased in the recurrent GBM tissues and exhibited high diagnostic value

**Figure 9.**
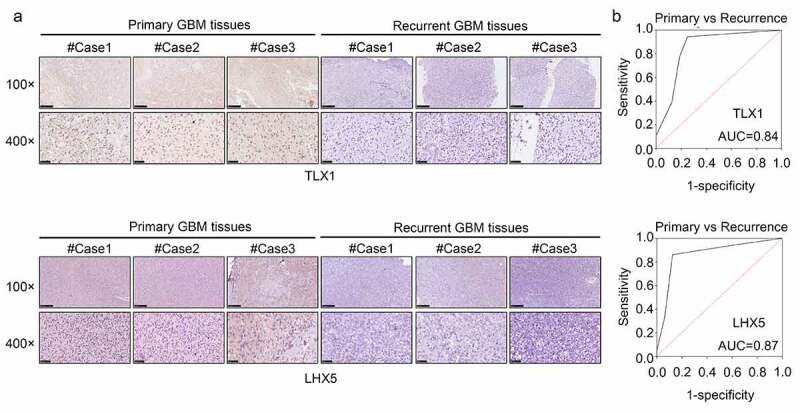
LIM homeobox 5 (LHX5) and T cell leukemia homeobox 1 (TLX1) were downregulated in the recurrent GBM tissues and exhibited high diagnostic value. (a) Immunohistochemical (IHC) staining was used to determine the expression of LHX5 and TLX1 in the recurrent GBM tissues and primary GBM tissues. (b) Receiver operating characteristic (ROC) analysis was performed to determine the diagnostic value of LHX5 and TLX1 in distinguishing between the recurrent and primary GBM tissues

We then determined the expression levels of LHX5 and TLX1 in 52 GBM samples by IHC. The results indicated that the levels of LHX5 and TLX1 were reduced in the recurrent GBM tissues compared to those in the primary GBM tissues (Figure 9(a)). The IHC scores of all samples are shown in Table 1. Based on the IHC scores, we found that both LHX5 and TLX1 exhibited high diagnostic value for distinguishing between recurrent and primary GBM tissues (Figure 9(b)).

**Table 1.** Detail scores of T cell leukemia homeobox 1 (TLX1) and LIM homeobox 5 (LHX5) expression in the primary and recurrent glioblastoma multiforme (GBM) tissues

Genes	Tissues	Expression score						
		0	1	2	3	4	5	6
TLX1	Primary tumor	2	3	19	10	1	0	1
	Recurrence tumor	6	8	1	1	0	0	0
LHX5	Primary tumor	1	1	6	14	10	2	2
	Recurrence tumor	6	6	1	1	2	0	0

## Discussion

Since recurrence has become a major obstacle in the treatment of GBM, our primary goal was to gain molecular insights into and provide clinical signatures that can accurately identify and predict the candidate gene groups associated with the recurrence risk of GBM. Research on GBM pathogenesis is important and as an exploratory research, this study aimed to discover key genes related to GBM recurrence, which could serve as potential biomarkers for predicting GBM recurrence and aid in future investigations.

In the current study, through analyzing the gene expression profile of 70 primary and 47 recurrent GBM tissues using bioinformatic technology, we identified three gene co-expression modules associated with the recurrence state and recurrence time of the patients with GBM. Additionally, we performed DEG analysis and found that 232 genes were differentially expressed between the recurrent GBM tissues and primary tissues. These genes were enriched in ‘chemical homeostasis,’ ‘ionic homeostasis,’ ‘leukocyte differentiation,’ ‘lymphocyte differentiation,’ ‘T cell activation,’ and “T cell differentiation. Previous studies have shown that dysfunction of immune cells, particularly T cells, is an important factor in cancer recurrence [31]. Based on the evidence that DEGs between the recurrent and primary GBM tissues are enriched in terms of their immune cell biology, we speculated that these DEGs may be linked to the process of immune escape and GBM recurrence.

Thereafter, we performed intersection analysis and found that 13 genes (*EHF, TRPM1, FXYD4, TP73, CDH15, LHX5, FBN3, TLX1, C1QL4, COL2A1, SEC61G, NEUROD4*, and *GPR139*) were in the modules associated with GBM recurrence and were also differentially expressed between the recurrent and primary tissues. Among these genes, the expression levels of *LHX5* and *TLX1* were correlated with patient survival rates and KPS scores. Furthermore, we found that *LHX5* and *TLX1* were downregulated in recurrent GBM tissues compared with those in the primary tissues.

Homeobox genes contain Lin-11, Isl-1, and Mec-3 domains (LIM) subfamilies, which are involved in a series of diseases [20,32], including various types of cancer [33]. *LHX5*, a vital member of the LIM family, has been reported to be involved in the regulation of neuronal differentiation and migration during development of the central nervous system [34]. *LHX5* also has the potential to promote nerve precursor cell proliferation, neuronal differentiation, and migration during development of the hippocampus [34]. Dysregulated *LHX5* has also been observed in urothelial carcinoma of the bladder [33]. *TLX1*, a key member of the *HOX* gene family, was first identified in the T-lineage leukemia cells. Under normal conditions, *TLX1* expression is widely detected during embryonic life in the branchial arches, hindbrain, and splenic primordia of mice [35]. TLX1 protein is a DNA-binding homeodomain protein [36], and it functionally synergizes with NOTCH1 activation during malignant T-cell transformation [37]. One study conducted by Andreiuolo *et al*. [38] reported that *TLX1* was upregulated in supratentorial ependymoma. Interestingly, *TLX1* was found to be enriched in the biological functions of ‘T cell activation,’ and ‘T cell differentiation.’ Therefore, we speculated that *TLX1* may regulate immune cell functions, thereby influencing the recurrence of GBM. Moreover, GBM recurrence is primarily due to the presence of cancer stem cells (CSCs) [39]. Previous studies have also demonstrated that inducing tumor stem cell differentiation is an important therapeutic method to overcome tumor stemness, thereby reducing the recurrence and drug resistance of cancer [40,41]. Based on the evidence that *LHX5* and *TLX1* have the potential to promote neuronal differentiation, we speculated that they may also be linked to cancer stemness and recurrence of GBM. Collectively, these findings reinforce the analysis that *LHX5* and *TLX1* are recurrence-associated hub genes in GBM patients.

However, the specific roles and underlying molecular mechanisms of these genes need to be studied further in future experiments. Furthermore, once the suppressive roles of *LHX5* and *TLX1* in the recurrence of GBM are confirmed, we hope that a series of therapeutic strategies, including the development of *LHX5*/*TLX1*-specific agonists, agonists for their upstream transcription factors, and inhibitors of their repressors, may be developed to enhance their expression levels and/or activities to inhibit the recurrence of GBM.

## Conclusion

Overall, the findings of our study indicated that *LHX5* and *TLX1* are reliable recurrence-associated genes in GBM patients and may serve as viable molecular biomarkers for the recurrence of GBM.

## Supplementary Material

Supplemental Material

## Data Availability

The datasets used and/or analyzed in the current study are available from the corresponding author upon reasonable request. GEO database (https://www.ncbi.nlm.nih.gov/gds) GSEA (https://software.broad institute. org/gsea/index.jsp) CCGA (http://www.cgga.org.cn/) TCGA (https://xenabrowser.net/datapages/)

## Research highlights

1. *TLX1* and *LHX5* were present in the gene modules associated with recurrence.

2. *TLX1* and *LHX5* expression was decreased in the recurrent GBM tissues.

3. Low expression levels of *TLX1* and *LHX5* were associated with poor outcomes.

4. *TLX1* and *LHX5* have potential to distinguish recurrent from primary GBM tissues.
