# Supplementary material for: Identification of key genes involved in the recurrence of glioblastoma multiforme using weighted gene co-expression network analysis and differential expression analysis
Source: Bioengineered. 2021 Jul 8;12(1):3188–200. doi: 10.1080/21655979.2021.1943986 (PMC8806787; doi:10.1080/21655979.2021.1943986)
Supplement: Supplemental Material [file KBIE_A_1943986_SM7710.zip › Supplement Table 2.docx]

|  |  |  |  |
| --- | --- | --- | --- |
| Gene | logFC | PValue | FDR |
| CCL3L3 | 7.149601 | 5.04E-09 | 2.71E-07 |
| NPAS4 | 6.52188 | 1.48E-22 | 5.93E-19 |
| ITGAD | 4.962431 | 2.30E-22 | 7.34E-19 |
| MEOX1 | 4.59444 | 7.81E-11 | 9.91E-09 |
| CCL18 | 4.223769 | 1.25E-10 | 1.40E-08 |
| COMP | 4.182446 | 3.69E-14 | 1.97E-11 |
| SERPINB2 | 4.05684 | 6.61E-10 | 5.38E-08 |
| EGR4 | 3.757231 | 1.44E-11 | 2.49E-09 |
| IL6 | 3.631542 | 3.04E-12 | 7.33E-10 |
| JCHAIN | 3.567335 | 3.04E-12 | 7.33E-10 |
| IGLL5 | 3.453597 | 6.63E-10 | 5.38E-08 |
| COL6A5 | 3.44327 | 1.65E-09 | 1.14E-07 |
| PLA2G2D | 3.354182 | 2.09E-11 | 3.41E-09 |
| RAB44 | 3.29436 | 9.45E-12 | 1.85E-09 |
| LILRB5 | 3.258635 | 2.40E-17 | 3.48E-14 |
| ABRA | 3.174336 | 6.39E-09 | 3.33E-07 |
| CCL21 | 3.159203 | 3.57E-08 | 1.38E-06 |
| DPT | 3.145704 | 1.93E-13 | 7.90E-11 |
| SCX | 3.109809 | 3.13E-07 | 7.27E-06 |
| COL6A6 | 3.000103 | 3.54E-07 | 7.93E-06 |
| CDHR4 | 2.897257 | 3.03E-06 | 4.44E-05 |
| TRPM1 | 2.878319 | 1.13E-11 | 2.15E-09 |
| PCDH11Y | 2.875714 | 2.46E-08 | 1.00E-06 |
| CCL19 | 2.837452 | 2.04E-11 | 3.39E-09 |
| CARTPT | 2.837375 | 5.79E-06 | 7.53E-05 |
| IL1B | 2.822959 | 7.86E-13 | 2.51E-10 |
| HLA-DQA2 | 2.822128 | 1.12E-10 | 1.32E-08 |
| IFNG | 2.812558 | 6.58E-10 | 5.38E-08 |
| GPR174 | 2.790027 | 5.42E-13 | 1.84E-10 |
| CRTAM | 2.771005 | 5.14E-18 | 9.59E-15 |
| CCR7 | 2.75858 | 2.42E-12 | 6.23E-10 |
| HTRA4 | 2.728604 | 1.95E-16 | 1.83E-13 |
| PAGE2 | 2.724901 | 9.52E-05 | 0.000706 |
| CXCR3 | 2.684947 | 6.76E-14 | 3.28E-11 |
| TNMD | 2.682794 | 7.75E-07 | 1.49E-05 |
| TACSTD2 | 2.662739 | 2.32E-16 | 1.95E-13 |
| SPATA18 | 2.645422 | 3.58E-12 | 8.16E-10 |
| PKD2L1 | 2.63574 | 1.61E-26 | 8.60E-23 |
| RIPPLY1 | 2.617104 | 2.19E-08 | 9.08E-07 |
| KERA | 2.611548 | 0.00041428 | 0.002255 |
| SLAMF7 | 2.595114 | 7.87E-17 | 8.99E-14 |
| SCGB3A2 | 2.586973 | 2.26E-06 | 3.44E-05 |
| PTPN20 | 2.579392 | 1.07E-10 | 1.27E-08 |
| TESPA1 | 2.575386 | 2.92E-09 | 1.80E-07 |
| CCL5 | 2.574299 | 1.69E-17 | 2.70E-14 |
| HBG1 | 2.57173 | 6.36E-05 | 0.000513 |
| MAS1 | 2.568417 | 2.39E-06 | 3.62E-05 |
| SIGLEC1 | 2.562496 | 1.41E-16 | 1.41E-13 |
| GZMK | 2.555517 | 2.01E-13 | 8.04E-11 |
| HLA-DQB2 | 2.527361 | 3.83E-10 | 3.40E-08 |
| CD5L | 2.525918 | 1.53E-08 | 6.81E-07 |
| ACP5 | 2.512434 | 1.85E-13 | 7.76E-11 |
| MZB1 | 2.485462 | 3.75E-08 | 1.44E-06 |
| OASL | 2.480581 | 4.76E-19 | 1.09E-15 |
| FASLG | 2.438223 | 1.80E-12 | 5.03E-10 |
| ABCB5 | 2.414148 | 1.22E-08 | 5.70E-07 |
| CTXN3 | 2.410809 | 1.02E-05 | 0.000119 |
| STAP1 | 2.400583 | 8.33E-10 | 6.50E-08 |
| UBASH3A | 2.398563 | 7.93E-11 | 9.98E-09 |
| FCRL5 | 2.38689 | 3.53E-06 | 4.98E-05 |
| PRSS16 | 2.385779 | 5.42E-11 | 7.43E-09 |
| NPFFR2 | 2.370342 | 4.84E-07 | 1.02E-05 |
| EHF | 2.364748 | 4.47E-15 | 3.10E-12 |
| MCHR2 | 2.338336 | 0.000148575 | 0.000995 |
| CXCL9 | 2.329032 | 9.53E-15 | 6.09E-12 |
| CCL8 | 2.328136 | 2.92E-11 | 4.38E-09 |
| ZBP1 | 2.320444 | 1.92E-14 | 1.14E-11 |
| FAM71A | 2.313693 | 5.55E-06 | 7.25E-05 |
| EPYC | 2.298353 | 0.000571002 | 0.002915 |
| DNASE2B | 2.291071 | 3.11E-10 | 2.84E-08 |
| CBLN2 | 2.285831 | 3.52E-06 | 4.98E-05 |
| LRRC66 | 2.28078 | 3.10E-14 | 1.71E-11 |
| CXCL10 | 2.270285 | 8.88E-13 | 2.74E-10 |
| LYPD8 | 2.262812 | 5.31E-06 | 6.98E-05 |
| GZMA | 2.261904 | 3.08E-10 | 2.83E-08 |
| GPR18 | 2.261158 | 2.22E-11 | 3.54E-09 |
| CXCR5 | 2.246284 | 2.15E-07 | 5.55E-06 |
| CD3E | 2.243839 | 8.92E-13 | 2.74E-10 |
| LY9 | 2.243351 | 4.41E-12 | 9.66E-10 |
| HNF4A | 2.23293 | 4.23E-06 | 5.81E-05 |
| DLK1 | 2.2276 | 0.001401863 | 0.005977 |
| ICOS | 2.224875 | 1.57E-08 | 6.95E-07 |
| GABRR1 | 2.199394 | 0.000133339 | 0.000911 |
| GPR22 | 2.195727 | 4.25E-05 | 0.00037 |
| APOBEC3H | 2.188208 | 6.84E-16 | 5.20E-13 |
| ANKRD34C | 2.178158 | 1.07E-06 | 1.90E-05 |
| FXYD4 | 2.177968 | 7.09E-08 | 2.37E-06 |
| XCL2 | 2.177464 | 1.06E-08 | 5.09E-07 |
| LGALS2 | 2.172991 | 3.81E-08 | 1.46E-06 |
| PAX7 | 2.165835 | 9.03E-07 | 1.65E-05 |
| CD40LG | 2.162672 | 1.08E-09 | 8.14E-08 |
| SLITRK6 | 2.155489 | 6.07E-05 | 0.000492 |
| THEMIS | 2.154873 | 7.44E-13 | 2.45E-10 |
| ZNF683 | 2.15384 | 1.15E-09 | 8.46E-08 |
| AKR1B10 | 2.145726 | 1.99E-05 | 0.0002 |
| FABP4 | 2.139557 | 4.80E-09 | 2.62E-07 |
| ANKRD1 | 2.134883 | 6.56E-07 | 1.30E-05 |
| ABHD12B | 2.122003 | 1.83E-13 | 7.76E-11 |
| CD27 | 2.119134 | 2.05E-11 | 3.39E-09 |
| RSAD2 | 2.113908 | 5.40E-18 | 9.59E-15 |
| IKZF3 | 2.112283 | 2.93E-13 | 1.12E-10 |
| PVRIG | 2.111301 | 2.51E-11 | 3.97E-09 |
| LCK | 2.109065 | 1.16E-11 | 2.16E-09 |
| KCNV1 | 2.107876 | 0.00055594 | 0.002856 |
| LTB | 2.099527 | 2.69E-11 | 4.21E-09 |
| HSH2D | 2.098231 | 2.28E-14 | 1.30E-11 |
| CD3D | 2.095333 | 1.63E-09 | 1.14E-07 |
| TIMD4 | 2.09528 | 4.00E-09 | 2.30E-07 |
| ASTL | 2.092024 | 7.15E-07 | 1.40E-05 |
| IFIT2 | 2.086368 | 3.23E-30 | 5.17E-26 |
| TNF | 2.077357 | 1.41E-09 | 9.99E-08 |
| CD8A | 2.076973 | 1.14E-16 | 1.21E-13 |
| CIDEA | 2.076464 | 5.33E-05 | 0.000441 |
| CD2 | 2.076133 | 8.43E-11 | 1.05E-08 |
| FOSB | 2.065665 | 3.92E-07 | 8.61E-06 |
| MKRN2OS | 2.063827 | 2.04E-05 | 0.000205 |
| FGF5 | 2.056151 | 8.94E-07 | 1.63E-05 |
| CD8B | 2.048928 | 1.88E-12 | 5.15E-10 |
| SIT1 | 2.04288 | 2.39E-10 | 2.32E-08 |
| SOST | 2.042518 | 5.71E-05 | 0.000468 |
| FAM163A | 2.038046 | 5.98E-06 | 7.69E-05 |
| CLLU1OS | 2.024165 | 0.000391313 | 0.002161 |
| SKAP1 | 2.015705 | 3.80E-10 | 3.39E-08 |
| GZMH | 2.012373 | 1.41E-08 | 6.42E-07 |
| KRT14 | 2.01062 | 0.000560608 | 0.002878 |
| SH2D3A | 2.005313 | 1.73E-08 | 7.51E-07 |
| AGXT | -2.00445 | 4.09E-05 | 0.000358 |
| SLC18A1 | -2.00955 | 3.34E-07 | 7.60E-06 |
| KCNE5 | -2.01983 | 2.91E-07 | 6.89E-06 |
| ZAR1 | -2.02874 | 6.38E-09 | 3.33E-07 |
| NPW | -2.03482 | 2.89E-07 | 6.87E-06 |
| GRM6 | -2.03638 | 0.001262793 | 0.005518 |
| TNNI1 | -2.06692 | 0.000393412 | 0.002167 |
| OR51E1 | -2.0753 | 1.18E-07 | 3.59E-06 |
| FBN3 | -2.07671 | 5.71E-09 | 3.02E-07 |
| ASIC4 | -2.08559 | 1.25E-11 | 2.25E-09 |
| NEU4 | -2.09923 | 9.94E-09 | 4.86E-07 |
| MAB21L1 | -2.10278 | 1.23E-07 | 3.69E-06 |
| PRAC2 | -2.10702 | 0.000439991 | 0.002358 |
| SEC61G | -2.10935 | 6.10E-12 | 1.30E-09 |
| CATSPERD | -2.11679 | 0.000407851 | 0.002229 |
| GPR15 | -2.14175 | 0.005762571 | 0.018477 |
| NEUROD4 | -2.1567 | 3.39E-05 | 0.000308 |
| TNNC1 | -2.16041 | 8.08E-06 | 9.88E-05 |
| TP73 | -2.16456 | 1.43E-08 | 6.48E-07 |
| FAM81B | -2.16703 | 0.001380195 | 0.005914 |
| KCNK17 | -2.18346 | 0.000252301 | 0.001524 |
| LKAAEAR1 | -2.19184 | 6.95E-07 | 1.36E-05 |
| RSPO1 | -2.21027 | 8.57E-05 | 0.000651 |
| BARX1 | -2.21589 | 8.65E-07 | 1.60E-05 |
| TSHR | -2.22473 | 5.29E-09 | 2.82E-07 |
| SLC6A2 | -2.22476 | 2.43E-05 | 0.000234 |
| XKR3 | -2.25116 | 0.00134714 | 0.005797 |
| HCRTR1 | -2.26121 | 5.13E-06 | 6.78E-05 |
| C6orf15 | -2.27644 | 0.000300427 | 0.001752 |
| SLC10A4 | -2.27693 | 1.42E-10 | 1.56E-08 |
| VGLL2 | -2.28561 | 1.61E-06 | 2.63E-05 |
| LHX3 | -2.28915 | 2.33E-05 | 0.000226 |
| CA4 | -2.32849 | 1.53E-07 | 4.35E-06 |
| TMPRSS7 | -2.34059 | 4.63E-05 | 0.000395 |
| VEPH1 | -2.34435 | 2.83E-11 | 4.34E-09 |
| GPR139 | -2.34751 | 1.05E-05 | 0.000122 |
| AQP5 | -2.36217 | 8.51E-09 | 4.26E-07 |
| DMRT1 | -2.3622 | 0.000102739 | 0.000744 |
| SLC1A7 | -2.36343 | 0.000151242 | 0.001009 |
| PITX2 | -2.36552 | 0.000126067 | 0.000872 |
| IGFN1 | -2.36788 | 5.53E-08 | 1.95E-06 |
| POU4F1 | -2.38518 | 4.89E-07 | 1.02E-05 |
| SPIB | -2.41683 | 3.19E-05 | 0.000293 |
| UGT2B17 | -2.43153 | 4.96E-08 | 1.80E-06 |
| ANKUB1 | -2.45042 | 2.99E-05 | 0.000276 |
| IGFBPL1 | -2.46841 | 2.37E-10 | 2.31E-08 |
| CHST8 | -2.47583 | 1.44E-14 | 8.83E-12 |
| IL24 | -2.50048 | 0.000281693 | 0.001664 |
| NTF3 | -2.50739 | 0.000123794 | 0.00086 |
| GDF3 | -2.52574 | 5.88E-05 | 0.000479 |
| LRRC38 | -2.5346 | 4.24E-05 | 0.00037 |
| GPR17 | -2.53564 | 1.30E-06 | 2.21E-05 |
| MMP9 | -2.54747 | 7.13E-07 | 1.39E-05 |
| COL2A1 | -2.5621 | 1.01E-12 | 2.98E-10 |
| PRAME | -2.56727 | 0.000252048 | 0.001524 |
| MET | -2.6049 | 3.25E-08 | 1.29E-06 |
| EPHA8 | -2.61956 | 1.11E-05 | 0.000127 |
| NOS2 | -2.64652 | 8.59E-10 | 6.63E-08 |
| MRLN | -2.65591 | 0.000221052 | 0.001373 |
| KLF1 | -2.68254 | 4.05E-07 | 8.84E-06 |
| NODAL | -2.6893 | 6.03E-14 | 3.01E-11 |
| DPEP1 | -2.76284 | 3.13E-10 | 2.84E-08 |
| LEMD1 | -2.77504 | 3.96E-08 | 1.49E-06 |
| C1QL4 | -2.79914 | 4.86E-14 | 2.50E-11 |
| TLX1 | -2.80954 | 4.67E-09 | 2.57E-07 |
| MAGEA1 | -2.81355 | 0.000307744 | 0.001789 |
| CXCL5 | -2.84801 | 1.30E-09 | 9.27E-08 |
| PIH1D3 | -2.85308 | 0.000270775 | 0.001615 |
| AGR3 | -2.86148 | 4.93E-06 | 6.57E-05 |
| PI3 | -2.89141 | 2.86E-05 | 0.000267 |
| USH1G | -2.93419 | 9.81E-07 | 1.76E-05 |
| CSF3 | -2.96228 | 1.19E-05 | 0.000135 |
| GPR87 | -2.96556 | 0.00029463 | 0.001725 |
| LHX5 | -2.97333 | 5.72E-09 | 3.02E-07 |
| PRR35 | -3.0816 | 4.27E-05 | 0.000371 |
| ZPLD1 | -3.13862 | 3.58E-07 | 8.01E-06 |
| OTOR | -3.15806 | 1.22E-05 | 0.000137 |
| ITIH6 | -3.23691 | 6.90E-06 | 8.68E-05 |
| CPLX4 | -3.26781 | 2.12E-05 | 0.000211 |
| LHX4 | -3.39203 | 1.96E-10 | 2.02E-08 |
| OTOG | -3.47689 | 1.13E-09 | 8.42E-08 |
| SLC22A31 | -3.59913 | 1.96E-09 | 1.32E-07 |
| SIX6 | -3.6094 | 1.84E-11 | 3.13E-09 |
| C1orf189 | -3.63093 | 2.83E-07 | 6.79E-06 |
| SCGB2A1 | -3.63498 | 1.90E-05 | 0.000193 |
| APOBEC4 | -3.63832 | 3.36E-06 | 4.78E-05 |
| REG1A | -3.6676 | 2.72E-06 | 4.04E-05 |
| CSN1S1 | -3.71434 | 2.02E-06 | 3.15E-05 |
| CDH15 | -3.74444 | 3.70E-11 | 5.33E-09 |
| LMX1A | -3.86352 | 2.05E-06 | 3.19E-05 |
| PDX1 | -3.87098 | 3.20E-08 | 1.27E-06 |
| LHX1 | -3.87875 | 6.17E-10 | 5.16E-08 |
| REG1B | -3.93616 | 4.77E-07 | 1.01E-05 |
| CXCL13 | -3.95482 | 4.74E-08 | 1.73E-06 |
| LBX1 | -4.09825 | 4.37E-09 | 2.43E-07 |
| UNC93A | -4.36666 | 1.06E-06 | 1.88E-05 |
| LDHAL6B | -4.48544 | 1.09E-08 | 5.20E-07 |
| CR2 | -5.18039 | 3.88E-09 | 2.26E-07 |
| FDCSP | -5.27814 | 1.07E-07 | 3.31E-06 |
| PPBP | -5.64476 | 5.05E-17 | 6.21E-14 |
| PRL | -6.68114 | 1.01E-08 | 4.93E-07 |
| UNCX | -6.93686 | 4.82E-11 | 6.75E-09 |
| SLC6A3 | -7.09309 | 5.54E-11 | 7.50E-09 |
| OTX2 | -7.61914 | 1.90E-12 | 5.15E-10 |
| BARHL1 | -8.40058 | 4.06E-15 | 2.95E-12 |
| NEUROG1 | -9.1039 | 1.92E-11 | 3.22E-09 |
|  |  |  |  |
