# Supplementary material for: Identification of key genes involved in the recurrence of glioblastoma multiforme using weighted gene co-expression network analysis and differential expression analysis
Source: Bioengineered. 2021 Jul 8;12(1):3188–200. doi: 10.1080/21655979.2021.1943986 (PMC8806787; doi:10.1080/21655979.2021.1943986)
Supplement: Supplemental Material [file KBIE_A_1943986_SM7710.zip › Supplement Table 3.docx]

| GO terms | Genes |
| --- | --- |
| Chemical homeostasis | GPR18 FASLG CCL8 CXCR3 CCL5 HSH2D GPR174 HNF4A IFNG TRPM1 LCK CXCL9 CCL19 FABP4 CXCL10 CARTPT CXCR5 CCR7 CCL21 IL6 HCRTR1 CXCL13 REG1A MET PDX1 GPR17 |
| Ion homeostasis | GPR18 FASLG CCL8 CXCR3 CCL5 HSH2D GPR174 HNF4A IFNG TRPM1 LCK CXCL9 CCL19 CXCL10 CXCR5 CCR7 CCL21 HCRTR1 CXCL13 REG1A GPR17 |
| Leukocyte differentiation | GPR18 THEMIS CD8A CRTAM ZNF683 RSAD2 CD2 IKZF3 LY9 CD3E IFNG LCK CD3D CD27 TESPA1 CD40LG CCL19 CARTPT PLA2G2D TNF CCR7 IL1B IL6 CR2 MMP9 CSF3 POU4F1 |
| lymphocyte differentiation | GPR18 THEMIS CD8A CRTAM ZNF683 RSAD2 CD2 IKZF3 LY9 CD3E IFNG LCK CD3D CD27 TESPA1 CD40LG CCL19 PLA2G2D CCR7 IL1B IL6 CR2 |
| T cell activation | GPR18 THEMIS CD8A CRTAM ZNF683 RSAD2 CD2 CCL5 LY9 HSH2D ICOS SIT1 LTX1 CD3E CD8B IFNG LCK CD3D CD27 TESPA1 CD40LG CCL19 PLA2G2D CCR7 CCL21 IL1B IL6 |
| T cell differentiation | GPR18 THEMIS CD8A CRTAM ZNF683 RSAD2 CD2 LY9 CD3E IFNG LCK LTX1 CD3D CD27 TESPA1 CCL19 PLA2G2D CCR7 IL1B IL6 |
