# Supplementary material for: Identification of key genes involved in the recurrence of glioblastoma multiforme using weighted gene co-expression network analysis and differential expression analysis
Source: Bioengineered. 2021 Jul 8;12(1):3188–200. doi: 10.1080/21655979.2021.1943986 (PMC8806787; doi:10.1080/21655979.2021.1943986)
Supplement: Supplemental Material [file suppl material.pdf › suppl material.pdf]

probes	AAMP	ABCB8	ABHD17A	ACADS	ACD	ACHE	ACSF3	ACTL10
moduleCo	black	black	black	black	black	black	black	black
GS.Recurre	-0.09628	-0.20255	-0.13278	0.08329	-0.16446	-0.13879	-0.22571	-0.18148
p.GS.Recu	0.281552	0.022386	0.136707	0.351869	0.064659	0.119658	0.010728	0.041158
GS.Times	-0.03639	-0.20358	-0.13418	0.093409	-0.11955	-0.05294	-0.22826	-0.2628
p.GS.Time:	0.684579	0.021697	0.132575	0.296233	0.180639	0.554471	0.00985	0.002836
MMblue	-0.14017	-0.05807	-0.24212	-0.40867	0.088999	0.02419	0.163757	0.002778
p.MMblue	0.116013	0.51665	0.006098	1.84E-06	0.319723	0.787194	0.065822	0.975271
MMlightcy	-0.48269	-0.29735	-0.62348	-0.44096	-0.47012	-0.22387	-0.2253	-0.10628
p.MMlight	9.09E-09	0.000686	4.92E-15	2.12E-07	2.45E-08	0.011402	0.010875	0.234328
MMdarkre	0.099338	-0.31085	-0.02759	0.35902	0.066711	-0.00686	0.000417	-0.20483
p.MMdark	0.266499	0.000375	0.758179	3.4E-05	0.456159	0.938994	0.996284	0.020888
MMcyan	-0.46207	-0.54107	-0.61577	-0.0988	-0.54541	-0.30381	-0.36439	-0.25842
p.MMcyan	4.54E-08	5.13E-11	1.31E-14	0.269087	3.35E-11	0.000516	2.54E-05	0.003352
MMdarkm	-0.11198	-0.34541	-0.52393	-0.12074	-0.40313	-0.19174	-0.26895	-0.28684
p.MMdark	0.210053	6.97E-05	2.6E-10	0.176331	2.61E-06	0.030809	0.002232	0.001077
MMorange	-0.14989	-0.051	-0.26357	-0.01981	-0.35854	-0.18557	-0.30818	-0.03271
p.MMoran	0.09256	0.569084	0.002753	0.825083	3.49E-05	0.036734	0.000424	0.715069
MMdarkgr	-0.14512	0.086706	-0.10364	-0.09259	-0.14728	-0.16823	0.109766	0.231459
p.MMdark	0.103562	0.332398	0.246239	0.300506	0.098453	0.058674	0.219265	0.008838
MMbrown	-0.10423	-0.00997	0.019329	0.341868	-0.30994	-0.36535	0.031831	0.185658
p.MMbrow	0.243545	0.91138	0.829223	8.36E-05	0.000391	2.4E-05	0.722391	0.036638
MMdarkol	-0.24992	-0.08337	-0.23812	0.142975	-0.46123	-0.40584	-0.20283	0.137115
p.MMdark	0.004601	0.351405	0.00702	0.108809	4.83E-08	2.2E-06	0.022193	0.124245
MMred	0.25349	0.316961	0.160647	-0.03959	0.241641	-0.02372	0.148887	0.256874
p.MMred	0.004033	0.000283	0.071194	0.658581	0.006202	0.791223	0.0948	0.003554
MMroyalb	0.301567	0.532269	0.334682	-0.18818	0.481991	0.191934	0.531566	0.368466
p.MMroya	0.00057	1.19E-10	0.00012	0.034122	9.61E-09	0.030638	1.27E-10	2.02E-05
MMblack	0.582695	0.674473	0.884999	0.543652	0.779585	0.443263	0.764865	0.546625
p.MMblacl	6.63E-13	3.54E-18	2.54E-43	3.98E-11	3.6E-27	1.8E-07	1.24E-25	2.97E-11
MMdarkgr	0.332769	0.574126	0.600228	0.35786	0.344989	0.021789	0.56515	0.55283
p.MMdark	0.000132	1.71E-12	8.77E-14	3.62E-05	7.13E-05	0.807887	4.46E-12	1.59E-11
MMdarktu	0.177974	0.097546	0.39922	0.227115	0.07817	0.019699	-0.03088	-0.0028
p.MMdark	0.045301	0.275261	3.33E-06	0.010235	0.382359	0.826007	0.730352	0.975076
MMlightgr	0.348563	0.239551	0.355123	-0.18118	0.483086	0.425909	0.177573	-0.02451
p.MMlight	5.92E-05	0.006677	4.19E-05	0.041503	8.8E-09	5.97E-07	0.045797	0.784467
MMdarkor	-0.28088	-0.37591	-0.32759	-0.32664	-0.23505	0.122039	-0.49274	-0.4267
p.MMdark	0.00138	1.33E-05	0.00017	0.000178	0.007811	0.171679	3.99E-09	5.66E-07
MMgreen	0.167022	-0.07578	0.080857	-0.09902	0.168394	0.27037	-0.25241	-0.33303
p.MMgree	0.060541	0.397152	0.366161	0.268055	0.058427	0.00211	0.004198	0.00013
MMgrey	0.049035	-0.25785	-0.03733	-0.4451	0.381867	0.087072	0.226691	-0.21448
p.MMgrey	0.584064	0.003425	0.676923	1.58E-07	9.43E-06	0.330356	0.010381	0.015462

ACY1	ADAT3	ADCK5	ADRM1	AFF4	AFTPH	AGPAT2	AGRN	AIP
black	black	black	black	black	black	black	black	black
-0.36655	-0.21926	-0.27686	-0.13272	-0.14388	0.199949	0.035469	-0.31824	0.082675
2.25E-05	0.013261	0.001627	0.136901	0.106554	0.024206	0.69219	0.000266	0.355448
-0.37177	-0.25156	-0.30925	-0.11164	-0.20709	0.182705	-0.02519	-0.3862	0.115508
1.68E-05	0.004332	0.000404	0.211462	0.019485	0.039786	0.778599	7.31E-06	0.195952
0.140268	0.066652	0.05155	-0.21879	0.435388	-0.13316	-0.16513	0.189172	-0.31533
0.115741	0.456554	0.564899	0.013463	3.13E-07	0.135593	0.063554	0.033167	0.000305
-0.14524	-0.33956	-0.35536	-0.59296	0.737608	0.30666	-0.35179	-0.22545	-0.65455
0.103252	9.4E-05	4.14E-05	2.06E-13	4.61E-23	0.000454	5E-05	0.010819	7.06E-17
-0.06306	-0.26608	-0.01	-0.06583	-0.46585	-0.08451	0.164665	-0.47298	0.460774
0.481207	0.002498	0.911164	0.462173	3.41E-08	0.344824	0.064316	1.96E-08	5E-08
-0.2793	-0.58383	-0.4618	-0.60874	0.362933	0.363239	-0.21473	-0.61616	-0.32828
0.001473	5.83E-13	4.63E-08	3.14E-14	2.75E-05	2.7E-05	0.015336	1.25E-14	0.000165
-0.27234	-0.52439	-0.46761	-0.39366	0.21113	0.32058	-0.16024	-0.46059	-0.2629
0.001951	2.49E-10	2.98E-08	4.68E-06	0.017185	0.000238	0.07193	5.08E-08	0.002825
-0.14366	-0.30085	-0.33408	-0.31666	0.057011	0.145007	-0.20451	-0.31237	-0.29236
0.107102	0.000589	0.000124	0.000287	0.524353	0.103824	0.021092	0.00035	0.000852
0.162593	0.163549	-0.02122	0.150867	0.321909	-0.0893	0.344522	0.419469	-0.24765
0.067793	0.066169	0.812852	0.09044	0.000224	0.318076	7.3E-05	9.16E-07	0.004998
-0.07109	0.043933	-0.12464	0.156399	-0.11385	-0.04973	0.420764	0.044031	0.050911
0.427088	0.623824	0.162668	0.079102	0.202478	0.578745	8.41E-07	0.623043	0.569741
-0.23867	-0.13505	-0.28934	-0.12955	0.168372	0.124095	0.13964	-0.0964	-0.24203
0.006887	0.130069	0.000969	0.1466	0.058461	0.164519	0.117398	0.280944	0.006117
0.103071	0.362089	0.148969	0.49899	-0.07766	-0.23793	0.037498	0.367379	0.104218
0.248852	2.88E-05	0.094616	2.36E-09	0.385472	0.007069	0.675541	2.15E-05	0.243595
0.378856	0.641456	0.433695	0.423858	0.065055	-0.44311	0.179048	0.750031	-0.01365
1.12E-05	4.48E-16	3.52E-07	6.85E-07	0.467439	1.82E-07	0.043997	3.41E-24	0.878924
0.57967	0.842035	0.821357	0.834692	-0.60982	-0.72604	0.675505	0.671594	0.726052
9.28E-13	2.61E-35	2.89E-32	3.51E-34	2.75E-14	4.56E-22	3.01E-18	5.53E-18	4.56E-22
0.369035	0.73602	0.465418	0.702478	-0.31393	-0.56007	0.611506	0.725266	0.319931
1.96E-05	6.36E-23	3.52E-08	3.47E-20	0.000325	7.59E-12	2.23E-14	5.3E-22	0.000246
-0.1683	0.169649	0.06396	0.214646	-0.51042	-0.16871	-0.10459	-0.00881	0.342763
0.058565	0.056547	0.474981	0.015379	8.77E-10	0.057944	0.241902	0.921664	7.99E-05
0.165103	0.304543	0.316257	0.182861	-0.21788	-0.29673	-0.24327	0.315659	0.194517
0.0636	0.000499	0.000292	0.039615	0.013866	0.000705	0.005853	0.0003	0.028423
-0.29347	-0.49878	-0.26031	-0.51973	0.316203	0.532624	-0.57644	-0.48003	-0.26018
0.000812	2.4E-09	0.003119	3.81E-10	0.000293	1.15E-10	1.33E-12	1.13E-08	0.003136
-0.12879	-0.21199	-0.02248	-0.05091	-0.22206	0.209435	-0.31856	-0.21094	0.142839
0.149005	0.016727	0.801885	0.56975	0.012103	0.018121	0.000262	0.017289	0.10915
0.061698	0.113108	0.212334	-0.03922	-0.00792	-0.11245	-0.11267	0.228829	0.157271
0.490773	0.205466	0.016547	0.661503	0.929602	0.208129	0.207241	0.009662	0.077424

AKAP10	AKR7A2	AKT1S1	ALDH16A1	ALKBH4	ALKBH7	AMACR	AMD1	AMN
black	black	black	black	black	black	black	black	black
0.168272	-0.06558	-0.23701	-0.17932	-0.41237	-0.15788	-0.20528	0.337506	-0.05823
0.058613	0.463828	0.007298	0.043674	1.45E-06	0.076269	0.020602	0.000104	0.515508
0.168961	-0.02438	-0.23131	-0.24	-0.32978	-0.14865	-0.27345	0.386248	-0.09219
0.057571	0.785546	0.008884	0.006572	0.000153	0.095325	0.001866	7.29E-06	0.302604
-0.0393	-0.25481	0.095676	0.008758	0.283263	-0.19198	-0.04454	-0.2069	-0.05991
0.660889	0.003839	0.284614	0.92215	0.001251	0.030595	0.619014	0.019601	0.503421
0.411286	-0.43708	-0.32652	-0.31114	-0.13148	-0.55054	-0.317	0.179814	-0.25393
1.56E-06	2.78E-07	0.000179	0.00037	0.140632	2.01E-11	0.000282	0.043085	0.003968
0.199236	0.477057	-0.23956	-0.18887	0.125029	0.318616	-0.18753	0.179396	-0.02368
0.024727	1.43E-08	0.006676	0.033455	0.161339	0.000261	0.034753	0.043581	0.791558
0.615123	-0.10386	-0.58617	-0.47015	-0.23843	-0.36881	-0.44953	0.405212	-0.26919
1.42E-14	0.245215	4.48E-13	2.45E-08	0.006946	1.98E-05	1.15E-07	2.29E-06	0.002211
0.394713	-0.14152	-0.35601	-0.45908	-0.23474	-0.36439	-0.38326	0.340179	-0.28187
4.39E-06	0.112493	4E-05	5.68E-08	0.007895	2.54E-05	8.69E-06	9.11E-05	0.001325
0.196474	0.231642	-0.32455	-0.20785	-0.34844	-0.24557	-0.21991	0.003616	-0.19084
0.026837	0.008782	0.000197	0.019036	5.96E-05	0.00539	0.012981	0.967818	0.031615
-0.02298	-0.22605	0.242587	0.338824	-0.14085	-0.14924	-0.03587	-0.17305	0.113245
0.79757	0.010604	0.005997	9.76E-05	0.114235	0.094005	0.688877	0.051707	0.204916
0.095284	0.164291	0.080313	0.327349	-0.42242	0.030852	-0.11314	0.127389	0.132062
0.286604	0.064933	0.369407	0.000172	7.54E-07	0.730597	0.205323	0.153514	0.138862
0.215601	-0.06767	-0.19506	0.084723	-0.4044	-0.22898	-0.13085	0.160247	0.013905
0.014917	0.449662	0.027972	0.343614	2.41E-06	0.009613	0.142564	0.071911	0.876691
-0.2934	-0.11698	0.333158	0.247371	0.280022	0.081949	0.182755	-0.18704	0.150997
0.000815	0.190274	0.00013	0.005049	0.00143	0.359704	0.039731	0.035235	0.09016
-0.58991	-0.09365	0.72235	0.558998	0.321143	0.139434	0.308531	-0.51979	0.256235
2.93E-13	0.295007	9.27E-22	8.48E-12	0.000232	0.117947	0.000417	3.79E-10	0.00364
-0.67028	0.58138	0.810741	0.850608	0.623988	0.871697	0.562115	-0.50353	0.508698
6.77E-18	7.68E-13	7.48E-31	1.06E-36	4.61E-15	1.55E-40	6.13E-12	1.6E-09	1.02E-09
-0.56875	0.296533	0.772283	0.862183	0.081574	0.435241	0.330009	-0.39371	0.432231
3.04E-12	0.000711	2.15E-26	9.97E-39	0.361916	3.16E-07	0.000151	4.66E-06	3.89E-07
-0.20942	0.16811	0.13302	0.080242	-0.08784	0.220023	0.058622	0.205206	0.03767
0.018129	0.05886	0.135994	0.36983	0.326102	0.012935	0.512681	0.020647	0.67414
-0.44752	-0.02567	0.292313	0.043783	0.493256	0.23999	0.320657	-0.2628	0.020294
1.33E-07	0.774479	0.000854	0.625012	3.82E-09	0.006575	0.000237	0.002836	0.820843
0.401383	-0.37405	-0.62782	-0.68478	-0.00156	-0.32003	-0.05918	0.307624	-0.33887
2.91E-06	1.48E-05	2.8E-15	6.85E-19	0.986089	0.000245	0.5087	0.000435	9.74E-05
-0.00077	-0.04986	-0.24893	-0.40302	0.098577	0.032976	0.110909	0.059176	-0.12874
0.993123	0.577738	0.004771	2.63E-06	0.270198	0.712837	0.214473	0.508696	0.149158
-0.21515	-0.21527	0.156284	-0.07465	0.178173	0.026156	0.021821	-0.17761	-0.06111
0.015133	0.015078	0.079326	0.404202	0.045057	0.770366	0.807609	0.045755	0.494933

ANAPC11	ANAPC2	ANKRD13I	ANKS3	AP1G1	AP1M1	AP4E1	AP5Z1	APBA3
black	black	black	black	black	black	black	black	black
-0.23579	-0.18345	0.131134	-0.17201	-0.1337	-0.14312	-0.04642	-0.20556	-0.33511
0.007614	0.038972	0.141684	0.053145	0.133975	0.108444	0.604305	0.020423	0.000118
-0.1814	-0.16849	0.050577	-0.20907	-0.13178	-0.10574	-0.07916	-0.25045	-0.36707
0.041248	0.058285	0.572275	0.018329	0.139702	0.236731	0.376313	0.004513	2.19E-05
0.070367	-0.05505	-0.45847	0.069519	0.419776	-0.00587	0.167382	0.065421	0.250293
0.431792	0.538733	5.94E-08	0.437376	8.98E-07	0.947756	0.059979	0.46493	0.004538
-0.3278	-0.49089	-0.70614	-0.29504	0.632916	-0.43756	0.511891	-0.27706	-0.09241
0.000169	4.65E-09	1.82E-20	0.000759	1.43E-15	2.69E-07	7.7E-10	0.001614	0.301444
0.502513	-0.22814	0.086651	-0.03848	-0.4778	-0.39761	-0.42824	-0.15948	-0.06444
1.75E-09	0.00989	0.332709	0.667562	1.34E-08	3.68E-06	5.1E-07	0.073301	0.471683
-0.11933	-0.69132	-0.52666	-0.4365	0.232222	-0.73195	0.261305	-0.45485	-0.2929
0.181473	2.33E-19	2.02E-10	2.89E-07	0.00861	1.44E-22	0.003003	7.78E-08	0.000832
-0.19638	-0.45985	-0.38483	-0.31601	0.210258	-0.4415	0.156968	-0.36638	-0.35014
0.026911	5.36E-08	7.92E-06	0.000295	0.017661	2.04E-07	0.078003	2.27E-05	5.45E-05
-0.18318	-0.18342	-0.06623	-0.14012	0.037149	-0.37569	0.052613	-0.21889	-0.31802
0.039268	0.039004	0.459391	0.11612	0.678396	1.35E-05	0.556891	0.01342	0.000269
-0.04941	-0.02535	-0.13148	0.065324	0.209867	0.122445	0.300266	0.309998	0.259953
0.581207	0.777221	0.140631	0.465594	0.017878	0.170246	0.000604	0.00039	0.003163
-0.03453	-0.0082	0.269458	0.05781	-0.17821	-0.14198	0.199076	0.230177	0.049962
0.699934	0.927138	0.002188	0.518547	0.045016	0.111326	0.024845	0.009231	0.576966
-0.26688	-0.25098	-0.00093	-0.09524	0.082715	-0.25137	0.31181	0.018622	-0.07964
0.002421	0.004425	0.991721	0.28682	0.355218	0.004361	0.000359	0.835384	0.37347
-0.00391	0.251963	-0.04714	0.252837	0.15748	0.556281	0.022076	0.281128	0.233985
0.965229	0.004268	0.598718	0.004132	0.077026	1.12E-11	0.805404	0.001366	0.008105
0.065383	0.643245	0.116922	0.558752	0.181459	0.657903	0.039988	0.580846	0.54616
0.465192	3.5E-16	0.190496	8.7E-12	0.041181	4.33E-17	0.655331	8.15E-13	3.11E-11
0.763424	0.841442	0.642049	0.801899	-0.63908	0.670859	-0.49133	0.865851	0.833348
1.73E-25	3.24E-35	4.13E-16	9.67E-30	6.21E-16	6.19E-18	4.48E-09	2.08E-39	5.57E-34
0.248095	0.719766	0.539548	0.650191	-0.28258	0.558936	-0.03467	0.799522	0.59874
0.004918	1.51E-21	5.94E-11	1.32E-16	0.001287	8.53E-12	0.698765	1.88E-29	1.05E-13
-0.00883	0.31467	0.333781	0.077654	-0.26685	0.173425	-0.18513	0.009374	-0.17327
0.92155	0.000314	0.000126	0.385516	0.002424	0.051191	0.037187	0.916696	0.051406
0.187107	0.400034	0.066875	0.207806	-0.02263	0.480763	-0.35288	0.091342	0.136694
0.035171	3.17E-06	0.455048	0.01906	0.800629	1.06E-08	4.72E-05	0.307096	0.125416
-0.2675	-0.49009	-0.36297	-0.57031	0.23092	-0.30813	0.045466	-0.64392	-0.48866
0.002363	4.97E-09	2.74E-05	2.58E-12	0.009001	0.000425	0.611753	3.19E-16	5.59E-09
-0.04404	-0.05417	0.116801	-0.2608	-0.14087	0.103561	-0.33714	-0.36581	-0.3596
0.622998	0.545256	0.190959	0.003063	0.114165	0.246597	0.000106	2.34E-05	3.29E-05
0.141495	0.113586	-0.07968	0.196269	0.178899	0.070046	-0.13859	-0.04044	0.077137
0.11256	0.203545	0.37323	0.026999	0.044176	0.433902	0.120203	0.651696	0.388701

APRT	ARFGAP1	ARFGEF1	ARFGEF2	ARFRP1	ARHGDIA	ARID5A	ARL15	ARL16
black	black	black	black	black	black	black	black	black
-0.1028	-0.07124	-0.03204	0.14409	-0.03128	-0.1244	0.044041	0.027446	-0.24853
0.25009	0.426118	0.720687	0.106051	0.727007	0.163471	0.622972	0.759376	0.004841
-0.11593	-0.1035	-0.10815	0.161706	-0.05717	-0.14985	-0.04153	0.005751	-0.26028
0.194325	0.246891	0.22616	0.069327	0.523167	0.092665	0.642979	0.948836	0.003124
0.029667	-0.09249	0.324227	-0.24191	-0.31134	-0.0763	-0.05034	0.066367	0.190733
0.74057	0.301007	0.0002	0.006142	0.000367	0.39391	0.574114	0.458484	0.031717
-0.18656	-0.36975	0.643887	0.157208	-0.54279	-0.5366	-0.22217	0.218685	-0.12124
0.035719	1.88E-05	3.2E-16	0.077543	4.33E-11	7.89E-11	0.012059	0.013509	0.174534
0.580286	-0.35611	-0.39636	-0.32542	-0.06931	-0.37705	-0.07237	-0.06344	0.477593
8.67E-13	3.97E-05	3.97E-06	0.000189	0.43873	1.25E-05	0.418744	0.478607	1.37E-08
0.115659	-0.60436	0.344453	0.109292	-0.49287	-0.79944	-0.27778	0.238311	0.050729
0.195364	5.35E-14	7.33E-05	0.221273	3.95E-09	1.92E-29	0.001567	0.006974	0.571123
0.080795	-0.367	0.217227	0.159769	-0.31565	-0.52211	-0.19343	0.096218	-0.11197
0.366535	2.19E-05	0.01416	0.072773	0.0003	3.07E-10	0.029334	0.281883	0.210106
-0.02918	-0.24103	-0.00573	0.083363	-0.14935	-0.44695	-0.21472	0.07736	-0.06334
0.744716	0.006337	0.949012	0.351443	0.093763	1.38E-07	0.015342	0.387323	0.479279
0.283169	0.188891	0.248329	-0.09863	0.077492	0.227479	0.399517	-0.12072	-0.05929
0.001256	0.033434	0.004876	0.269961	0.386511	0.010111	3.27E-06	0.17641	0.507879
0.439548	0.170145	-0.18467	-0.11488	0.293931	0.033905	0.42581	-0.19879	-0.16679
2.34E-07	0.055819	0.037665	0.198408	0.000796	0.705118	6.01E-07	0.025061	0.0609
0.089639	-0.00561	0.137724	0.009103	0.072922	-0.19975	0.143878	-0.09414	-0.25291
0.31624	0.950098	0.122566	0.919094	0.415218	0.024353	0.10657	0.292474	0.004121
0.002995	0.286821	0.04573	-0.173	0.112919	0.562549	0.221975	-0.50635	0.046003
0.97334	0.001078	0.609681	0.051775	0.20623	5.86E-12	0.012136	1.25E-09	0.607549
-0.00252	0.630252	-0.04963	-0.32176	0.292159	0.712325	0.341882	-0.34659	-0.01619
0.977593	2.03E-15	0.57951	0.000225	0.000859	5.99E-21	8.36E-05	6.56E-05	0.856652
0.590483	0.652137	-0.7213	-0.67012	0.682221	0.809227	0.512796	-0.53568	0.427408
2.74E-13	9.99E-17	1.13E-21	6.94E-18	1.04E-18	1.17E-30	7.11E-10	8.62E-11	5.4E-07
0.425807	0.773599	-0.46077	-0.44495	0.679541	0.782212	0.621003	-0.53233	-0.02311
6.01E-07	1.57E-26	5.01E-08	1.6E-07	1.59E-18	1.86E-27	6.77E-15	1.19E-10	0.796472
-0.17984	0.18949	-0.43316	0.020774	0.24724	0.227153	-0.07263	-0.10451	-0.14941
0.043057	0.032867	3.65E-07	0.816674	0.005073	0.010222	0.417062	0.242284	0.093637
-0.28579	0.142343	-0.14372	-0.18574	-0.01852	0.400384	-0.08608	-0.01331	0.15278
0.001126	0.110398	0.106952	0.036558	0.836267	3.1E-06	0.335919	0.881954	0.086382
-0.62742	-0.56606	0.451333	0.57749	-0.50807	-0.5148	-0.56897	0.606389	-0.08913
2.95E-15	4.05E-12	1.01E-07	1.18E-12	1.08E-09	5.94E-10	2.97E-12	4.18E-14	0.319006
-0.4406	-0.21711	-0.03673	0.345723	-0.11177	-0.02905	-0.32994	0.302788	-0.00296
2.17E-07	0.014212	0.681811	6.86E-05	0.21092	0.745801	0.000152	0.00054	0.973639
0.070701	0.002077	0.081607	-0.2413	-0.2835	0.154768	0.102691	0.058394	0.270163
0.429604	0.981511	0.361718	0.006276	0.001238	0.082319	0.250612	0.514323	0.002128

ARL8B	ARMC5	ARMC6	ARMC7	ARMC8	ARMCX5	ARRDC1	ARVCF	ASL
black	black	black	black	black	black	black	black	black
0.346429	-0.20601	-0.30357	-0.27394	-0.15811	0.154704	0.019641	-0.29795	-0.06438
6.61E-05	0.020146	0.000521	0.00183	0.075827	0.082447	0.826512	0.000669	0.472076
0.403541	-0.20139	-0.2543	-0.25986	-0.09636	0.206186	-0.04641	-0.31372	-0.10368
2.55E-06	0.023185	0.003913	0.003173	0.281145	0.020036	0.604354	0.000329	0.24605
-0.33219	-0.03097	0.383677	0.201487	0.197805	0.176572	-0.35466	0.138067	-0.13346
0.000136	0.729601	8.48E-06	0.023114	0.025802	0.047053	4.29E-05	0.121628	0.134682
0.128902	-0.4835	-0.14646	-0.2126	0.428344	0.300024	-0.60976	-0.37913	-0.1431
0.148649	8.51E-09	0.100379	0.016408	5.07E-07	0.00061	2.77E-14	1.1E-05	0.108509
0.11355	-0.21064	0.064032	-0.00574	0.197626	0.344336	0.081638	-0.29897	0.117698
0.203691	0.017452	0.474487	0.948894	0.025939	7.37E-05	0.361536	0.000639	0.187549
0.401143	-0.68399	-0.31883	-0.36239	0.533749	0.474279	-0.47962	-0.69766	-0.04009
2.96E-06	7.79E-19	0.000259	2.83E-05	1.04E-10	1.77E-08	1.16E-08	7.99E-20	0.654483
0.32067	-0.54585	-0.23864	-0.36653	0.386296	0.301181	-0.31937	-0.56438	0.054589
0.000237	3.2E-11	0.006895	2.25E-05	7.27E-06	0.00058	0.000252	4.84E-12	0.542152
0.272274	-0.3363	-0.44165	-0.4317	0.098651	-0.07668	-0.16274	-0.1995	-0.1337
0.001956	0.000111	2.02E-07	4.03E-07	0.269836	0.391527	0.067536	0.024533	0.133997
-0.27958	0.046548	-0.0335	0.053205	-0.05312	-0.21795	0.034658	-0.18016	0.392012
0.001456	0.603298	0.708516	0.552454	0.553109	0.013836	0.698879	0.042677	5.17E-06
0.012235	-0.08859	-0.38162	-0.1715	-0.21097	-0.2804	0.375116	-0.38749	0.349493
0.89141	0.32197	9.56E-06	0.053868	0.017275	0.001408	1.39E-05	6.77E-06	5.64E-05
0.17846	-0.28508	-0.38253	-0.26767	-0.06097	-0.14385	0.051575	-0.43591	0.199746
0.044707	0.00116	9.07E-06	0.002347	0.495907	0.106645	0.56471	3.02E-07	0.024353
-0.36962	0.342809	0.201258	0.339224	-0.2367	-0.28883	0.144	0.253886	0.414374
1.9E-05	7.97E-05	0.023274	9.56E-05	0.007377	0.000991	0.106271	0.003974	1.28E-06
-0.66447	0.615986	0.456596	0.456843	-0.43051	-0.4451	0.226906	0.661831	0.053622
1.64E-17	1.28E-14	6.84E-08	6.71E-08	4.37E-07	1.58E-07	0.010307	2.43E-17	0.549344
-0.71176	0.889168	0.686231	0.685708	-0.60726	-0.52857	0.751157	0.733036	0.344263
6.64E-21	2.89E-44	5.4E-19	5.89E-19	3.76E-14	1.69E-10	2.68E-24	1.16E-22	7.4E-05
-0.57531	0.664636	0.183125	0.359252	-0.68961	-0.70036	0.698662	0.43043	0.350182
1.5E-12	1.6E-17	0.039325	3.36E-05	3.1E-19	5.02E-20	6.72E-20	4.4E-07	5.44E-05
0.117425	0.170368	-0.08968	-0.043	-0.37431	-0.14735	0.285462	0.202794	-0.24538
0.188582	0.055494	0.316032	0.631203	1.46E-05	0.098299	0.001141	0.02222	0.005425
-0.29308	0.412951	0.533967	0.325435	-0.16141	-0.04052	0.008211	0.697404	-0.31283
0.000826	1.4E-06	1.01E-10	0.000189	0.069851	0.651075	0.926997	8.34E-20	0.000342
0.514292	-0.42187	-0.08918	-0.22845	0.511697	0.552914	-0.53772	-0.17331	-0.39873
6.22E-10	7.82E-07	0.318725	0.009787	7.84E-10	1.58E-11	7.09E-11	0.051351	3.43E-06
0.275933	-0.02769	0.059642	-0.06114	0.162418	0.231149	-0.10753	0.166916	-0.25849
0.001689	0.757328	0.505361	0.494699	0.068093	0.008932	0.228876	0.060706	0.003343
-0.36266	0.138034	0.359537	0.23008	0.097134	0.419832	-0.07335	0.289085	-0.23086
2.79E-05	0.121719	3.3E-05	0.009262	0.277305	8.95E-07	0.412482	0.00098	0.009019

ASPSCR1	ATAD3A	ATAD3B	ATG101	ATG2A	ATG2B	ATG4B	ATG4D	ATMIN
black	black	black	black	black	black	black	black	black
-0.10495	-0.39881	-0.29826	-0.16014	-0.22646	0.212258	-0.1682	-0.25208	0.210768
0.240303	3.41E-06	0.00066	0.072099	0.01046	0.016587	0.058723	0.00425	0.017382
-0.09753	-0.39524	-0.31772	-0.06467	-0.25618	0.254363	-0.13544	-0.19364	0.285577
0.275327	4.25E-06	0.000273	0.470112	0.003647	0.003904	0.128954	0.029159	0.001136
-0.08615	0.38864	0.319339	0.052296	-0.11634	-0.05027	-0.04762	0.157113	-0.00142
0.335545	6.32E-06	0.000253	0.559274	0.192721	0.574641	0.59498	0.077725	0.98733
-0.52616	-0.12387	-0.05625	-0.32631	-0.48441	0.179791	-0.42669	-0.35908	0.194477
2.11E-10	0.165288	0.529895	0.000181	7.91E-09	0.043112	5.67E-07	3.39E-05	0.028456
0.194065	-0.10241	-0.01108	0.321738	-0.49741	-0.35189	-0.11503	-0.17009	-0.12848
0.028801	0.251924	0.901616	0.000226	2.7E-09	4.97E-05	0.197821	0.055892	0.149996
-0.46312	-0.38732	-0.23919	-0.21271	-0.8089	-0.00371	-0.5932	-0.61932	0.157605
4.19E-08	6.84E-06	0.006764	0.016353	1.29E-30	0.966997	2E-13	8.39E-15	0.076787
-0.44873	-0.38848	-0.29752	-0.13653	-0.5722	0.04019	-0.25679	-0.35808	0.238025
1.22E-07	6.39E-06	0.000681	0.125864	2.1E-12	0.653702	0.003565	3.58E-05	0.007045
-0.27624	-0.2986	-0.11846	-0.44194	-0.2428	-0.0901	-0.37705	-0.6004	-0.05858
0.001668	0.00065	0.184677	1.98E-07	0.005952	0.31373	1.25E-05	8.6E-14	0.512968
-0.1734	0.201862	0.170336	-0.04287	0.102982	-0.18129	-0.07286	0.027585	-0.35286
0.051223	0.022854	0.05554	0.632282	0.249262	0.041378	0.415629	0.758192	4.72E-05
-0.03597	-0.14409	0.077879	-0.21342	-0.02895	-0.29923	-0.14393	-0.32293	-0.33663
0.688053	0.106046	0.384136	0.015989	0.746668	0.000632	0.106444	0.000213	0.000109
-0.23519	-0.22913	-0.05355	-0.32656	-0.18218	-0.10383	-0.3046	-0.41827	-0.08144
0.007774	0.009565	0.549863	0.000179	0.040369	0.245346	0.000498	9.91E-07	0.362717
0.131219	0.190767	0.007475	0.415723	0.430031	0.095947	0.438233	0.633128	-0.01248
0.141424	0.031686	0.933525	1.17E-06	4.52E-07	0.283245	2.57E-07	1.39E-15	0.889243
0.330493	0.646994	0.537548	0.114029	0.71327	-0.07149	0.544038	0.649274	-0.24298
0.000148	2.07E-16	7.2E-11	0.201777	5.04E-21	0.424467	3.83E-11	1.5E-16	0.005913
0.913509	0.827878	0.71293	0.552275	0.764764	-0.56363	0.673355	0.732727	-0.66467
1.16E-50	3.5E-33	5.36E-21	1.69E-11	1.27E-25	5.24E-12	4.21E-18	1.23E-22	1.59E-17
0.520455	0.534678	0.56987	0.085122	0.738698	-0.40217	0.479942	0.419335	-0.58214
3.57E-10	9.48E-11	2.7E-12	0.341339	3.69E-23	2.77E-06	1.13E-08	9.25E-07	7.05E-13
0.270583	-0.16469	-0.12987	-0.06823	0.184179	0.171469	0.188916	0.087414	0.192632
0.002093	0.064282	0.145593	0.445923	0.038187	0.053911	0.033411	0.328451	0.030026
0.319987	0.332505	0.13201	0.267071	0.379614	0.093216	0.4403	0.567445	0.170602
0.000245	0.000134	0.13902	0.002403	1.07E-05	0.297239	2.22E-07	3.5E-12	0.055154
-0.38721	-0.41205	-0.53115	-0.08285	-0.41521	0.509383	-0.3461	-0.24332	0.571811
6.88E-06	1.49E-06	1.33E-10	0.354448	1.21E-06	9.61E-10	6.73E-05	0.005841	2.19E-12
-0.0145	-0.24116	-0.39135	0.145473	0.008669	0.315614	0.076831	0.099641	0.37407
0.871495	0.006309	5.38E-06	0.102704	0.922943	0.000301	0.390587	0.265033	1.48E-05
0.100048	0.287325	0.237083	0.312273	-0.02452	0.14491	0.289991	0.413984	0.051615
0.263079	0.001055	0.00728	0.000351	0.784338	0.104055	0.000943	1.31E-06	0.564413

ATP2C1	ATXN1	AURKAIP1	AXIN1	AZI2	B3GALT6	B3GAT3	B4GALT2	B9D2
black	black	black	black	black	black	black	black	black
0.058776	0.07004	-0.1507	-0.38773	0.123948	-0.41582	0.091182	-0.27424	-0.08367
0.511575	0.433942	0.090809	6.68E-06	0.165022	1.16E-06	0.30795	0.001808	0.349684
0.018374	0.01398	-0.10519	-0.43875	0.128377	-0.44049	0.141341	-0.17584	-0.0573
0.837545	0.876034	0.239201	2.47E-07	0.150324	2.19E-07	0.112953	0.047987	0.522231
-0.07671	-0.05929	-0.16239	0.346154	-0.0806	0.355737	-0.25582	0.32301	-0.04719
0.391334	0.507897	0.068148	6.71E-05	0.367676	4.05E-05	0.003697	0.000212	0.5983
0.36625	0.348055	-0.56943	-0.07662	0.25651	-0.13154	-0.59797	-0.25027	-0.22621
2.29E-05	6.08E-05	2.83E-12	0.391895	0.003603	0.140443	1.15E-13	0.004542	0.010548
-0.25771	-0.25321	0.387964	-0.27075	0.130907	-0.15228	0.362751	0.181606	0.241675
0.003443	0.004075	6.58E-06	0.002079	0.142382	0.087419	2.77E-05	0.041015	0.006194
0.33386	0.268218	-0.34815	-0.4335	0.403286	-0.40345	-0.32691	-0.33721	-0.12845
0.000125	0.002297	6.05E-05	3.56E-07	2.59E-06	2.56E-06	0.000176	0.000106	0.1501
0.223693	0.151594	-0.37001	-0.35282	0.289564	-0.45521	-0.2161	-0.19055	-0.02065
0.011469	0.08888	1.86E-05	4.73E-05	0.00096	7.57E-08	0.014683	0.031881	0.81779
0.23491	0.372391	-0.12722	-0.35304	0.118433	-0.35649	-0.21928	-0.4184	0.218433
0.007851	1.62E-05	0.154062	4.68E-05	0.184789	3.89E-05	0.013252	9.83E-07	0.01362
0.13111	-0.00112	-0.22555	0.319281	0.176959	0.26577	-0.10689	-0.20545	0.070775
0.141757	0.990061	0.010784	0.000253	0.046564	0.002528	0.231664	0.020491	0.429121
0.063111	-0.09739	-0.00634	-0.05798	0.123349	-0.14303	0.182075	-0.5506	0.027354
0.480876	0.276019	0.943579	0.517343	0.16709	0.108675	0.040486	1.99E-11	0.76016
0.25193	0.196744	-0.24742	-0.21172	0.278546	-0.26328	-0.13285	-0.61434	-0.14706
0.004273	0.026624	0.00504	0.016869	0.001519	0.002784	0.136492	1.57E-14	0.098961
-0.10333	-0.21416	-0.02583	0.33391	0.197505	0.195939	0.011234	0.129864	0.047907
0.247637	0.015619	0.773168	0.000125	0.026032	0.027263	0.900241	0.145615	0.592749
-0.33306	-0.28072	0.100534	0.768258	-0.38898	0.60865	0.071646	0.369454	0.121082
0.00013	0.00139	0.260753	5.61E-26	6.2E-06	3.18E-14	0.423444	1.91E-05	0.175089
-0.70216	-0.53608	0.844458	0.762487	-0.4803	0.734346	0.771533	0.584609	0.490589
3.66E-20	8.29E-11	1.08E-35	2.14E-25	1.1E-08	8.9E-23	2.58E-26	5.35E-13	4.77E-09
-0.37678	-0.3952	0.381325	0.655288	-0.34945	0.487871	0.477957	0.024815	0.230565
1.26E-05	4.26E-06	9.73E-06	6.34E-17	5.65E-05	5.96E-09	1.33E-08	0.781838	0.009111
-0.19468	-0.21883	0.24934	-0.18364	-0.29029	-0.13086	0.351464	0.018099	-0.17294
0.028288	0.013447	0.0047	0.038767	0.000931	0.142512	5.09E-05	0.839942	0.051854
-0.38105	-0.29115	0.256843	0.242193	-0.42865	0.337768	0.093424	0.661888	-0.02416
9.88E-06	0.000897	0.003558	0.006081	4.96E-07	0.000103	0.296155	2.41E-17	0.787462
0.36907	0.440042	-0.30219	-0.53655	0.217305	-0.3517	-0.41604	0.015593	-0.27704
1.96E-05	2.26E-07	0.000555	7.93E-11	0.014124	5.02E-05	1.15E-06	0.86187	0.001615
0.038262	0.083394	0.079966	-0.35427	-0.02346	-0.18686	-0.02609	0.251728	-0.06691
0.66932	0.351266	0.371487	4.38E-05	0.793459	0.035414	0.77095	0.004305	0.454834
-0.31888	-0.37031	-0.00349	0.312131	-0.07782	0.284158	0.010903	0.499085	0.004754
0.000258	1.83E-05	0.968915	0.000354	0.384525	0.001205	0.903167	2.34E-09	0.957694

BANF1	BANP	BARHL1	BBC3	BCAR1	BCAT2	BCKDK	BCL7C	BDP1
black	black	black	black	black	black	black	black	black
-0.30623	-0.28088	-0.27604	-0.05296	-0.18795	-0.22283	-0.01697	-0.31013	-0.10076
0.000463	0.00138	0.001682	0.554253	0.034338	0.011798	0.849813	0.000388	0.259686
-0.27886	-0.3483	-0.28092	-0.04562	-0.19722	-0.25337	0.008908	-0.29548	-0.17039
0.0015	6E-05	0.001378	0.61057	0.026251	0.004051	0.920821	0.000745	0.055462
0.38749	0.343164	0.23144	-0.27271	-0.11365	0.097339	0.157545	0.25873	0.299064
6.77E-06	7.83E-05	0.008843	0.001923	0.203298	0.276285	0.076902	0.003313	0.000637
0.017701	0.083651	-0.1187	-0.55885	-0.57515	-0.02702	-0.12031	-0.13017	0.550596
0.843414	0.349781	0.183778	8.61E-12	1.53E-12	0.763007	0.177867	0.144662	2E-11
0.497116	0.135713	-0.03911	0.032014	-0.26463	-0.0686	0.165465	0.40541	-0.34788
2.76E-09	0.128176	0.662452	0.720861	0.002643	0.4435	0.063013	2.26E-06	6.13E-05
0.103156	-0.01554	-0.27917	-0.47427	-0.75475	-0.16219	-0.11143	-0.0471	0.250309
0.24846	0.862307	0.001481	1.78E-08	1.22E-24	0.068486	0.212318	0.599018	0.004536
0.050252	-0.18366	-0.24945	-0.35517	-0.59062	-0.10359	-0.0259	-0.12794	0.077406
0.574751	0.038741	0.004681	4.18E-05	2.7E-13	0.246478	0.772571	0.151737	0.387043
-0.11699	-0.06341	-0.13358	-0.21851	-0.38839	0.09901	-0.38929	-0.14894	-0.00997
0.190233	0.478774	0.134328	0.013585	6.42E-06	0.268086	6.08E-06	0.094679	0.911403
0.010584	0.249533	0.14685	-0.02985	0.00779	0.254695	0.365018	0.153385	0.010032
0.905987	0.004667	0.099457	0.738992	0.930735	0.003856	2.45E-05	0.085129	0.910873
-0.12612	0.1141	-0.11762	0.120423	-0.07997	0.265605	0.150255	0.100225	-0.3218
0.157674	0.201494	0.187834	0.177469	0.371488	0.002544	0.091771	0.262228	0.000225
-0.15937	-0.0088	-0.2178	-0.15295	-0.30423	0.061428	-0.06021	-0.1113	-0.02434
0.073504	0.921751	0.013902	0.086024	0.000506	0.492677	0.50133	0.212846	0.785881
0.148605	-0.01645	0.174543	0.022473	0.348537	0.066891	0.340412	0.034232	-0.05382
0.095433	0.854353	0.049687	0.801979	5.93E-05	0.454936	9.01E-05	0.702403	0.547855
0.154823	0.394842	0.441512	0.225636	0.58075	0.432059	0.334491	0.257292	-0.05363
0.082209	4.35E-06	2.04E-07	0.010753	8.24E-13	3.93E-07	0.000121	0.003498	0.54931
0.556037	0.635982	0.549832	0.717199	0.839053	0.591166	0.491242	0.794546	-0.58562
1.15E-11	9.45E-16	2.16E-11	2.44E-21	7.62E-35	2.53E-13	4.52E-09	7.37E-29	4.77E-13
0.102652	0.438786	0.34163	0.520706	0.663748	0.615618	0.417076	0.423047	-0.51061
0.250791	2.47E-07	8.46E-05	3.49E-10	1.82E-17	1.34E-14	1.07E-06	7.23E-07	8.63E-10
-0.22599	-0.33197	-0.12974	0.230578	0.297292	-0.10154	-0.23139	-0.20596	-0.32438
0.010628	0.000137	0.145997	0.009106	0.000688	0.256007	0.008857	0.020173	0.000199
0.19342	-0.06795	0.254315	0.110043	0.442346	-0.14385	-0.07195	0.052666	0.00563
0.029346	0.447782	0.003911	0.218098	1.92E-07	0.106647	0.421457	0.556496	0.949906
-0.26738	-0.50411	-0.30833	-0.32217	-0.37159	-0.60848	-0.54988	-0.5036	0.581158
0.002374	1.52E-09	0.000421	0.000221	1.7E-05	3.24E-14	2.15E-11	1.59E-09	7.87E-13
-0.22088	-0.47705	-0.12555	0.038334	0.071163	-0.4856	-0.33803	-0.35431	0.113551
0.012579	1.43E-08	0.159586	0.668732	0.426584	7.18E-09	0.000102	4.37E-05	0.203687
0.323096	0.156172	0.174315	-0.07101	0.117342	-0.07666	0.276818	0.155462	0.176462
0.000211	0.079543	0.049992	0.427579	0.188898	0.391666	0.00163	0.080938	0.047192

BOP1	BRAT1	BRCC3	BRICD5	BTBD2	C11orf98	C12orf4	C12orf57	C16orf86
black	black	black	black	black	black	black	black	black
-0.38655	-0.3719	0.111175	-0.2103	-0.24117	-0.15932	0.080457	0.024203	0.004527
7.16E-06	1.67E-05	0.21337	0.017638	0.006307	0.073588	0.368545	0.787083	0.959718
-0.38757	-0.39516	0.136055	-0.13705	-0.24476	-0.13056	0.115933	0.114468	0.016242
6.74E-06	4.27E-06	0.127207	0.124421	0.005548	0.143446	0.194301	0.200033	0.856178
0.244249	0.303129	0.190672	0.132597	-0.01202	0.097504	0.225517	-0.16609	-0.30178
0.005651	0.000532	0.031773	0.137257	0.893309	0.27547	0.010796	0.06201	0.000565
-0.35444	-0.1169	0.406929	-0.27059	-0.51023	-0.33154	0.387227	-0.47174	-0.48416
4.34E-05	0.190582	2.06E-06	0.002092	8.92E-10	0.00014	6.88E-06	2.16E-08	8.07E-09
-0.14398	-0.14173	0.095079	0.117962	-0.38444	-0.09807	0.38153	0.41515	0.402839
0.106311	0.111951	0.287647	0.186557	8.11E-06	0.27267	9.61E-06	1.22E-06	2.66E-06
-0.6138	-0.38211	0.47519	-0.31479	-0.82106	-0.50089	0.601574	-0.20803	-0.17938
1.68E-14	9.29E-06	1.65E-08	0.000313	3.17E-32	2.01E-09	7.47E-14	0.018931	0.043598
-0.48978	-0.35712	0.314345	-0.23767	-0.59395	-0.383	0.318393	-0.14972	-0.1466
5.1E-09	3.76E-05	0.000319	0.007132	1.84E-13	8.82E-06	0.000264	0.092946	0.100035
-0.4509	-0.21069	0.20149	-0.24353	-0.46541	-0.48702	0.019221	-0.26381	0.042294
1.04E-07	0.017425	0.023112	0.005798	3.52E-08	6.39E-09	0.830168	0.002728	0.636832
-0.00267	0.261517	-0.01212	-0.25784	-0.03469	-0.02671	-0.13009	-0.12234	-0.20481
0.976203	0.002979	0.892453	0.003427	0.698652	0.765638	0.144899	0.170618	0.020898
-0.31811	-0.00123	-0.09089	-0.18528	-0.33658	-0.22445	-0.00498	-0.02609	0.276325
0.000268	0.989065	0.309494	0.037024	0.000109	0.011186	0.955717	0.770969	0.001663
-0.41716	-0.10173	0.072368	-0.30523	-0.4171	-0.30729	0.148123	-0.1751	-0.06736
1.07E-06	0.255074	0.418776	0.000484	1.07E-06	0.000441	0.096526	0.04895	0.451765
0.300583	0.237338	-0.30077	-0.081	0.473266	0.464135	-0.26882	0.426812	-0.20725
0.000595	0.007216	0.000591	0.365297	1.92E-08	3.88E-08	0.002244	5.62E-07	0.019391
0.658061	0.644385	-0.34379	0.346593	0.663507	0.521401	-0.46957	-0.02561	-0.03545
4.23E-17	2.99E-16	7.58E-05	6.56E-05	1.89E-17	3.27E-10	2.56E-08	0.775037	0.692362
0.850514	0.861639	-0.65918	0.673189	0.772358	0.722844	-0.52991	0.486673	0.613732
1.1E-36	1.25E-38	3.59E-17	4.32E-18	2.11E-26	8.44E-22	1.49E-10	6.58E-09	1.69E-14
0.472889	0.645232	-0.5532	0.309355	0.486246	0.424072	-0.57479	0.148947	0.408944
1.98E-08	2.65E-16	1.53E-11	0.000402	6.81E-09	6.75E-07	1.59E-12	0.094665	1.81E-06
0.049667	-0.15931	-0.27533	0.200779	0.168506	0.124194	-0.21382	0.102965	0.296883
0.579222	0.073616	0.00173	0.023611	0.058258	0.164179	0.015785	0.249341	0.000701
0.598789	0.244983	-0.25371	0.358602	0.634416	0.415819	-0.21034	0.14258	-0.06493
1.04E-13	0.005504	0.004	3.48E-05	1.17E-15	1.16E-06	0.017617	0.109801	0.468297
-0.25756	-0.51964	0.386824	-0.20695	-0.14696	-0.2542	0.338574	-0.18007	-0.39628
0.003462	3.84E-10	7.04E-06	0.019573	0.099207	0.003928	9.88E-05	0.042779	3.99E-06
0.057054	-0.33911	0.068109	-0.00235	0.263841	0.009982	0.001762	0.146729	-0.08707
0.524042	9.62E-05	0.446752	0.979074	0.002724	0.911309	0.984313	0.099738	0.330343
0.401758	0.057384	0.034982	0.265187	0.195002	0.335043	0.070246	0.104224	-0.02021
2.85E-06	0.521641	0.696209	0.002586	0.028023	0.000118	0.432586	0.243569	0.821559

C18orf32	C19orf24	C19orf25	C19orf47	C19orf71	C19orf73	C19orf81	C1orf122	C1orf159
black	black	black	black	black	black	black	black	black
-0.2287	-0.12291	-0.14154	-0.26046	-0.16991	-0.24513	-0.18031	0.080394	-0.2221
0.009705	0.168612	0.112436	0.003102	0.056165	0.005476	0.042499	0.368923	0.012087
-0.30169	-0.14255	-0.10591	-0.23317	-0.20131	-0.19441	-0.19852	0.158025	-0.19212
0.000567	0.109877	0.235975	0.008335	0.023238	0.028515	0.025264	0.075996	0.030472
-0.14048	0.020943	-0.14804	0.188972	0.008257	0.126283	-0.03073	-0.31048	0.092992
0.115187	0.81521	0.096717	0.033357	0.926588	0.157147	0.731649	0.000381	0.298407
-0.38401	-0.30693	-0.54409	-0.29828	-0.31459	-0.24945	-0.32091	-0.50901	-0.3907
8.31E-06	0.000448	3.81E-11	0.000659	0.000316	0.004681	0.000235	9.93E-10	5.59E-06
-0.07975	0.262447	0.1766	-0.1414	-0.31319	-0.03436	0.159523	0.550345	-0.00888
0.372772	0.002875	0.047017	0.112803	0.000337	0.701336	0.073221	2.05E-11	0.921035
-0.4232	-0.20843	-0.45278	-0.51964	-0.58869	-0.41623	-0.28894	-0.10601	-0.50787
7.16E-07	0.018694	9.06E-08	3.84E-10	3.37E-13	1.13E-06	0.000986	0.235545	1.1E-09
-0.52246	-0.22006	-0.39091	-0.4378	-0.38513	-0.33216	-0.25884	-0.19219	-0.44001
2.97E-10	0.01292	5.52E-06	2.64E-07	7.78E-06	0.000136	0.0033	0.030413	2.26E-07
-0.10393	-0.14919	-0.20878	-0.48672	-0.26298	-0.32066	-0.14516	-0.03992	-0.3848
0.244914	0.094119	0.018493	6.55E-09	0.002817	0.000237	0.103447	0.655909	7.94E-06
-0.08385	0.186532	-0.23653	0.021534	-0.08616	-0.08829	0.054384	-0.31326	-0.0903
0.348616	0.035747	0.007422	0.810095	0.335482	0.323585	0.543671	0.000336	0.312665
-0.00402	0.265958	-0.06586	-0.24354	-0.19871	-0.27029	0.066468	0.197864	-0.20415
0.964207	0.00251	0.461959	0.005796	0.025119	0.002117	0.457798	0.025756	0.021322
-0.14822	0.006345	-0.2467	-0.33852	-0.27382	-0.27733	-0.14314	-0.10197	-0.34616
0.096303	0.943559	0.005174	9.91E-05	0.001839	0.001596	0.108399	0.253957	6.71E-05
-0.07112	0.042652	0.011819	0.452054	0.168319	0.479423	0.080755	-0.09521	0.191331
0.426886	0.633982	0.895078	9.55E-08	0.058541	1.18E-08	0.366769	0.286999	0.031175
0.230765	0.274353	0.212811	0.591995	0.580787	0.460767	0.279757	-0.1674	0.48992
0.009049	0.0018	0.0163	2.3E-13	8.2E-13	5.01E-08	0.001446	0.059956	5.04E-09
0.738617	0.857609	0.853275	0.738736	0.606341	0.625856	0.792798	0.560756	0.851782
3.75E-23	6.61E-38	3.74E-37	3.66E-23	4.21E-14	3.62E-15	1.18E-28	7.07E-12	6.71E-37
0.449099	0.610397	0.411218	0.433121	0.462713	0.292125	0.481548	0.283292	0.464542
1.18E-07	2.56E-14	1.57E-06	3.66E-07	4.32E-08	0.000861	9.96E-09	0.001249	3.76E-08
0.182536	-0.03505	0.296122	0.027889	0.169654	0.089515	0.054313	0.471684	0.133741
0.039973	0.69561	0.000724	0.755616	0.056541	0.316916	0.544204	2.17E-08	0.133867
0.234215	-0.03533	0.35484	0.455453	0.382652	0.449199	0.146975	0.040735	0.399563
0.008041	0.693296	4.25E-05	7.44E-08	9E-06	1.18E-07	0.099166	0.649321	3.26E-06
-0.16708	-0.59343	-0.22926	-0.2615	-0.21823	-0.18922	-0.42123	-0.33244	-0.27542
0.060444	1.95E-13	0.009521	0.002981	0.013708	0.033121	8.16E-07	0.000134	0.001724
0.020921	-0.34618	0.118879	0.009579	0.054865	0.046661	-0.15237	-0.01769	0.013026
0.8154	6.7E-05	0.18313	0.914884	0.540111	0.60242	0.087248	0.843541	0.884436
-0.14481	-0.00762	-0.0823	0.272174	0.20142	0.210125	0.017209	-0.11669	0.282116
0.104308	0.932252	0.357647	0.001964	0.023161	0.017735	0.847718	0.191394	0.001312

C1orf35	C20orf27	C2orf42	C2orf49	C4orf48	C5orf22	C5orf51	C6orf226	C7orf43
black	black	black	black	black	black	black	black	black
-0.18632	-0.15278	0.049576	-0.15665	-0.19039	0.106697	-0.0995	-0.15792	-0.25824
0.035962	0.086391	0.579912	0.078624	0.032027	0.232502	0.265724	0.076194	0.003375
-0.22117	-0.19648	0.138229	-0.07357	-0.1728	0.168761	-0.11233	-0.17241	-0.31555
0.012458	0.026835	0.121186	0.411103	0.052049	0.057872	0.208617	0.052591	0.000302
0.020543	-0.01606	0.15966	0.290864	-0.18109	0.080366	0.44563	0.049649	0.094145
0.818683	0.85778	0.072972	0.000908	0.041605	0.369091	1.52E-07	0.579354	0.292426
-0.35339	-0.48311	0.432982	0.576277	-0.58629	0.438172	0.753148	-0.2369	-0.28192
4.59E-05	8.78E-09	3.69E-07	1.35E-12	4.42E-13	2.58E-07	1.73E-24	0.007326	0.001322
0.130814	-0.08442	0.158151	0.214112	0.032124	0.126109	-0.35604	0.314733	-0.39904
0.142669	0.345334	0.075759	0.015642	0.719949	0.157723	3.99E-05	0.000314	3.37E-06
-0.36987	-0.58734	0.462976	0.622258	-0.57373	0.570922	0.45327	-0.12947	-0.62454
1.87E-05	3.93E-13	4.24E-08	5.76E-15	1.78E-12	2.41E-12	8.74E-08	0.14684	4.29E-15
-0.43073	-0.43941	0.382687	0.373062	-0.49578	0.379986	0.249869	-0.27874	-0.46066
4.31E-07	2.36E-07	8.99E-06	1.56E-05	3.09E-09	1.05E-05	0.004609	0.001507	5.05E-08
-0.07543	-0.40426	0.088428	0.110484	-0.20118	0.213104	0.187273	-0.07193	-0.29945
0.399312	2.44E-06	0.322848	0.216245	0.023331	0.01615	0.035006	0.421616	0.000626
-0.13169	0.071484	-0.04611	-0.19569	-0.11717	-0.14525	0.200705	-0.00114	0.161475
0.139987	0.424496	0.606676	0.027465	0.189556	0.103238	0.023664	0.989832	0.069731
-0.01998	0.025046	-0.08638	-0.35338	0.045092	-0.24048	-0.07575	0.117468	-0.01522
0.823548	0.779853	0.334203	4.6E-05	0.61469	0.006462	0.397333	0.188421	0.865097
-0.13643	-0.2055	0.06374	-0.10243	-0.2231	0.034612	0.168777	-0.01002	-0.09075
0.126159	0.020465	0.476502	0.251825	0.011694	0.699263	0.057848	0.91101	0.31024
0.006232	0.250981	0.124406	-0.25084	0.216052	-0.34829	-0.21474	0.159974	0.382956
0.94456	0.004425	0.163456	0.004449	0.014704	6E-05	0.015331	0.072402	8.85E-06
0.358194	0.513852	-0.19728	-0.42771	0.36872	-0.5886	0.000618	0.196626	0.706784
3.55E-05	6.47E-10	0.026205	5.29E-07	1.99E-05	3.4E-13	0.994496	0.026717	1.62E-20
0.836369	0.780865	-0.45656	-0.57667	0.886454	-0.77935	-0.62483	0.751365	0.713533
1.96E-34	2.61E-27	6.85E-08	1.29E-12	1.2E-43	3.82E-27	4.14E-15	2.56E-24	4.8E-21
0.503646	0.602291	-0.40247	-0.765	0.621893	-0.81601	-0.3316	0.430003	0.66048
1.58E-09	6.86E-14	2.72E-06	1.2E-25	6.04E-15	1.53E-31	0.00014	4.53E-07	2.97E-17
0.165595	0.12582	-0.19016	-0.35993	0.357111	-0.26596	-0.37662	-0.00191	0.055946
0.062803	0.158684	0.032242	3.23E-05	3.77E-05	0.002509	1.28E-05	0.983	0.532148
0.256229	0.339733	-0.23553	-0.08456	0.355711	-0.23236	-0.24526	0.064472	0.35197
0.003641	9.32E-05	0.007683	0.344526	4.06E-05	0.00857	0.00545	0.471447	4.95E-05
-0.39141	-0.41661	0.164866	0.600913	-0.4219	0.699326	0.275042	-0.45477	-0.41364
5.36E-06	1.1E-06	0.063988	8.08E-14	7.8E-07	5.99E-20	0.001751	7.82E-08	1.34E-06
-0.1216	-0.01369	-0.1306	0.093754	0.003155	0.249414	-0.27551	-0.27869	-0.12493
0.173229	0.878597	0.143313	0.294445	0.971915	0.004687	0.001718	0.00151	0.161686
0.078186	0.222259	0.073281	0.060771	0.024091	-0.05385	-0.00555	-0.00176	0.053467
0.382263	0.012023	0.412915	0.497319	0.788049	0.547655	0.950641	0.984339	0.550503

C7orf50	C8orf44	C8orf82	CAB39	CACFD1	CACTIN	CACUL1	CADM1	CAMK2D
black	black	black	black	black	black	black	black	black
-0.31817	0.170616	-0.40346	0.22264	-0.18609	-0.3038	0.361642	-0.10096	-0.08268
0.000267	0.055134	2.56E-06	0.011874	0.036194	0.000516	2.95E-05	0.258708	0.355391
-0.29374	0.202928	-0.4157	0.247845	-0.21554	-0.32704	0.339434	-0.12009	-0.10217
0.000803	0.02213	1.17E-06	0.004963	0.014947	0.000175	9.46E-05	0.178684	0.253028
0.073117	0.182533	0.32556	-0.23348	0.260755	0.10534	-0.40275	0.131302	0.246267
0.413966	0.039976	0.000188	0.008246	0.003067	0.238529	2.68E-06	0.141169	0.005255
-0.30789	0.314612	-0.15638	0.136325	-0.19311	-0.33454	-0.0482	0.399607	0.54418
0.000429	0.000315	0.079148	0.126449	0.029616	0.000121	0.590519	3.25E-06	3.78E-11
0.276732	0.290313	-0.10242	-0.11179	-0.06464	-0.45167	-0.11096	0.041994	-0.15509
0.001635	0.00093	0.25185	0.210833	0.470306	9.82E-08	0.214272	0.639232	0.081674
-0.22531	0.425293	-0.41209	0.241289	-0.35671	-0.72914	0.122653	0.405371	0.385772
0.01087	6.22E-07	1.48E-06	0.00628	3.85E-05	2.5E-22	0.169518	2.27E-06	7.5E-06
-0.27085	0.281329	-0.43556	0.102267	-0.36707	-0.55803	-0.00574	0.254595	0.365314
0.00207	0.001355	3.09E-07	0.252583	2.19E-05	9.37E-12	0.948911	0.003871	2.41E-05
-0.18499	-0.01052	-0.39775	0.074952	-0.21784	-0.35456	0.180023	0.386386	-0.07884
0.037327	0.906527	3.64E-06	0.402316	0.013883	4.32E-05	0.042839	7.23E-06	0.378252
-0.12056	-0.32908	0.189618	-0.30634	0.037706	0.186243	-0.0838	-0.20005	0.152033
0.176959	0.000158	0.032747	0.000461	0.673848	0.03604	0.348925	0.024134	0.087948
-0.1413	-0.26467	-0.15911	-0.04178	0.022872	-0.11975	0.331042	-0.27756	-0.18334
0.11305	0.002639	0.073971	0.640975	0.798538	0.179907	0.000144	0.001582	0.039091
-0.19941	-0.12101	-0.24816	0.17348	-0.08212	-0.24887	0.314163	0.045468	0.095794
0.024599	0.17535	0.004906	0.051116	0.358688	0.004781	0.000322	0.611735	0.284019
0.031023	-0.26298	0.293958	-0.34554	0.083286	0.470168	-0.17883	-0.24836	-0.03576
0.729167	0.002817	0.000795	6.93E-05	0.351894	2.44E-08	0.044255	0.00487	0.689828
0.140914	-0.285	0.627227	-0.51895	0.531744	0.807585	-0.4049	-0.37225	-0.1566
0.114057	0.001164	3.03E-15	4.09E-10	1.25E-10	1.89E-30	2.34E-06	1.64E-05	0.078721
0.750086	-0.41688	0.835916	-0.6813	0.705036	0.835848	-0.4847	-0.49848	-0.49842
3.37E-24	1.09E-06	2.3E-34	1.2E-18	2.21E-20	2.35E-34	7.72E-09	2.46E-09	2.48E-09
0.239222	-0.54987	0.52079	-0.53766	0.573169	0.711421	-0.10634	-0.6085	-0.46817
0.006755	2.15E-11	3.46E-10	7.13E-11	1.89E-12	7.06E-21	0.23406	3.23E-14	2.85E-08
-0.0024	-0.04197	-0.17066	0.164432	0.116149	0.053899	0.314073	-0.22052	-0.45478
0.978593	0.639458	0.055072	0.0647	0.193465	0.547276	0.000323	0.012727	7.82E-08
0.297475	-0.02314	0.35269	-0.09176	0.306874	0.438117	-0.33937	-0.04705	-0.14757
0.000683	0.796186	4.77E-05	0.304905	0.00045	2.59E-07	9.49E-05	0.599407	0.097799
-0.14661	0.374094	-0.39461	0.637916	-0.46183	-0.4282	0.165988	0.547853	0.431048
0.100015	1.47E-05	4.42E-06	7.27E-16	4.62E-08	5.11E-07	0.062174	2.63E-11	4.22E-07
0.036046	0.080196	-0.20645	0.361445	-0.27467	-0.08812	0.067969	0.169958	-0.00726
0.687438	0.370109	0.019873	2.98E-05	0.001777	0.324538	0.447687	0.056093	0.935443
-0.02603	0.423649	0.266094	-0.3136	0.160898	0.136035	-0.33426	-0.19123	-0.03316
0.771469	6.95E-07	0.002496	0.00033	0.070748	0.127264	0.000123	0.031263	0.711345

CAPN1	CAPN10	CAPN15	CARF	CASC10	CCDC12	CCDC124	CCDC130	CCDC137
black	black	black	black	black	black	black	black	black
-0.17476	-0.24882	-0.27429	-0.08105	-0.1314	-0.07571	-0.21672	-0.26635	-0.30726
0.049406	0.00479	0.001805	0.365039	0.14087	0.397556	0.014392	0.002471	0.000442
-0.20578	-0.26166	-0.3392	-0.05592	-0.11582	-0.0782	-0.23712	-0.26793	-0.36711
0.020286	0.002963	9.58E-05	0.532362	0.194735	0.382186	0.007272	0.002324	2.18E-05
-0.17394	-0.05687	0.073439	0.173085	-0.08101	-0.02569	-0.05002	0.17734	0.44124
0.050494	0.525398	0.411909	0.051655	0.365261	0.774329	0.576525	0.046087	2.08E-07
-0.53784	-0.5148	-0.3475	0.483754	-0.37928	-0.44476	-0.54109	-0.06389	0.044698
7.01E-11	5.94E-10	6.26E-05	8.34E-09	1.1E-05	1.62E-07	5.11E-11	0.475477	0.617786
-0.2828	-0.15205	-0.4218	-0.03152	0.023867	0.349352	-0.19218	-0.06648	-0.06227
0.001275	0.087915	7.85E-07	0.724975	0.78997	5.68E-05	0.030418	0.457735	0.486754
-0.69836	-0.6672	-0.69402	0.39188	-0.37829	-0.31104	-0.7199	-0.27381	-0.19318
7.07E-20	1.08E-17	1.48E-19	5.21E-06	1.16E-05	0.000372	1.47E-21	0.00184	0.029549
-0.46755	-0.46072	-0.56031	0.282473	-0.34948	-0.2177	-0.58766	-0.08145	-0.28551
2.99E-08	5.03E-08	7.4E-12	0.001293	5.64E-05	0.013944	3.78E-13	0.362618	0.001139
-0.2634	-0.25985	-0.35112	0.204667	-0.09281	-0.18883	-0.32125	-0.14877	-0.16881
0.002771	0.003175	5.18E-05	0.02099	0.29937	0.03349	0.000231	0.095056	0.057803
0.141102	-0.06856	0.253316	-0.3003	-0.26761	-0.13685	-0.00876	0.315358	0.336688
0.113571	0.443716	0.004059	0.000603	0.002353	0.12497	0.922117	0.000305	0.000109
0.084341	-0.10273	0.020493	-0.3346	-0.22496	0.028669	-0.07533	0.090144	0.071181
0.345806	0.250439	0.819115	0.000121	0.010999	0.749	0.399955	0.313509	0.42647
-0.11327	-0.32469	-0.14399	-0.10812	-0.28202	-0.18219	-0.2708	-0.11845	-0.01394
0.204808	0.000196	0.106284	0.22631	0.001317	0.040352	0.002075	0.184721	0.876382
0.476355	0.101461	0.405952	-0.29677	-0.05985	0.007768	0.2921	0.311625	0.107967
1.51E-08	0.256361	2.19E-06	0.000704	0.503873	0.930929	0.000862	0.000362	0.226957
0.518973	0.543412	0.759256	-0.25517	0.2133	0.253178	0.600991	0.575379	0.591549
4.08E-10	4.07E-11	4.47E-25	0.003789	0.01605	0.00408	8.01E-14	1.49E-12	2.42E-13
0.72119	0.856179	0.835117	-0.53358	0.60775	0.804232	0.940235	0.684639	0.6955
1.15E-21	1.18E-37	3.03E-34	1.05E-10	3.54E-14	4.98E-30	2.5E-60	7.01E-19	1.15E-19
0.66122	0.591782	0.785264	-0.60124	0.240002	0.447277	0.677914	0.590052	0.566197
2.66E-17	2.36E-13	8.53E-28	7.78E-14	0.006572	1.35E-07	2.06E-18	2.88E-13	3.99E-12
0.106142	0.197905	0.069301	-0.07865	0.178583	0.210582	0.234564	-0.16725	-0.24151
0.234956	0.025725	0.438819	0.379412	0.044558	0.017483	0.007945	0.060178	0.006232
0.299661	0.436217	0.322379	-0.00568	0.322453	0.268826	0.459985	0.052744	0.097642
0.00062	2.95E-07	0.000219	0.949497	0.000218	0.002243	5.31E-08	0.555907	0.274786
-0.40967	-0.34786	-0.50317	0.576446	-0.06571	-0.42739	-0.40287	-0.59378	-0.56177
1.73E-06	6.14E-05	1.65E-09	1.32E-12	0.462949	5.4E-07	2.66E-06	1.87E-13	6.36E-12
0.045155	0.065873	-0.17504	0.135307	0.161623	-0.08789	0.029594	-0.38611	-0.47555
0.614196	0.461848	0.049028	0.129334	0.069472	0.325822	0.741189	7.35E-06	1.61E-08
-0.07016	0.145503	0.153053	0.096819	0.010437	0.242142	0.075378	0.130863	0.191304
0.433161	0.102634	0.085814	0.278875	0.90729	0.006092	0.399638	0.142515	0.0312

CCDC154	CCDC171	CCDC22	CCDC61	CCDC85B	CCDC9	CCHCR1	CCNYL1	CCR10
black	black	black	black	black	black	black	black	black
-0.1274	0.095719	0.024552	-0.31604	-0.18439	-0.24517	-0.11309	0.141595	-0.20196
0.153493	0.284396	0.784092	0.000295	0.037962	0.005468	0.20555	0.112301	0.022784
-0.18382	0.040988	-0.01592	-0.31979	-0.20278	-0.34632	-0.14024	0.129054	-0.2616
0.038569	0.647283	0.859011	0.000247	0.022232	6.65E-05	0.115804	0.148165	0.00297
0.091918	0.087754	-0.13479	0.198733	-0.27147	0.088342	0.258029	0.215136	-0.02211
0.304045	0.326566	0.130804	0.0251	0.00202	0.323324	0.003402	0.015141	0.805136
-0.1027	0.373757	-0.53127	-0.06206	-0.64815	-0.23698	0.009565	0.516189	-0.23807
0.250574	1.5E-05	1.31E-10	0.488194	1.76E-16	0.007307	0.915008	5.25E-10	0.007034
-0.09787	-0.17243	0.048411	-0.0212	-0.05655	-0.38824	0.038002	-0.11245	-0.1821
0.273654	0.052554	0.588858	0.812991	0.527714	6.48E-06	0.671435	0.208115	0.040458
-0.26271	0.299893	-0.50459	-0.21989	-0.64606	-0.55573	-0.0737	0.441296	-0.39679
0.002846	0.000614	1.46E-09	0.012992	2.36E-16	1.19E-11	0.410274	2.07E-07	3.87E-06
-0.2502	0.144797	-0.44081	-0.25314	-0.58847	-0.47732	-0.21018	0.329026	-0.40594
0.004553	0.104329	2.14E-07	0.004086	3.45E-13	1.4E-08	0.017706	0.000159	2.19E-06
-0.00769	0.330757	-0.20824	-0.23693	-0.25659	-0.28511	0.164743	-0.03912	-0.06012
0.931617	0.000146	0.018803	0.00732	0.003592	0.001158	0.064188	0.662319	0.501932
0.058895	0.028622	0.032513	0.172367	-0.14966	0.338179	0.171324	0.292783	0.179076
0.510719	0.749403	0.716699	0.052647	0.093072	0.000101	0.054117	0.000837	0.043963
0.092249	-0.06115	0.226285	0.019839	-0.08232	0.036147	0.130341	0.115905	0.152665
0.3023	0.494668	0.010523	0.824792	0.357535	0.686611	0.144129	0.194407	0.08662
0.019918	0.074542	-0.04412	-0.05463	-0.27573	-0.07127	0.096573	0.309294	0.032837
0.824107	0.404902	0.622375	0.541863	0.001703	0.425906	0.280101	0.000403	0.713993
0.202452	-0.3121	0.17969	0.351898	0.150742	0.331112	-0.1399	-0.03554	0.224793
0.022451	0.000354	0.043232	4.97E-05	0.09071	0.000143	0.116715	0.691633	0.011058
0.446033	-0.16659	0.423048	0.47079	0.287932	0.685105	0.346422	-0.19836	0.435828
1.48E-07	0.061215	7.23E-07	2.33E-08	0.001029	6.5E-19	6.62E-05	0.02538	3.03E-07
0.552344	-0.53027	0.840007	0.669739	0.827439	0.746883	0.512024	-0.55301	0.752144
1.67E-11	1.44E-10	5.43E-35	7.36E-18	4.05E-33	6.69E-24	7.61E-10	1.57E-11	2.16E-24
0.501295	-0.33685	0.717754	0.491754	0.486239	0.69061	0.42173	-0.29441	0.634269
1.94E-09	0.000108	2.2E-21	4.33E-09	6.81E-09	2.62E-19	7.89E-07	0.00078	1.19E-15
-0.05526	-0.22808	0.235331	-0.12485	0.301865	-0.14022	-0.23245	-0.26273	0.033917
0.537197	0.00991	0.007737	0.161939	0.000563	0.115871	0.008545	0.002844	0.705015
0.018438	-0.25113	0.118999	0.093048	0.394213	0.154918	-0.11582	-0.30393	0.09431
0.836989	0.0044	0.182684	0.298112	4.52E-06	0.082018	0.194721	0.000513	0.291579
-0.42106	0.25559	-0.5966	-0.45343	-0.18697	-0.47688	-0.45445	0.21955	-0.43987
8.25E-07	0.003729	1.35E-13	8.64E-08	0.035304	1.45E-08	8.01E-08	0.013135	2.29E-07
-0.29355	-0.02384	-0.16424	-0.34358	0.228216	-0.22758	-0.398	-0.19992	-0.25794
0.00081	0.790229	0.065013	7.66E-05	0.009863	0.010075	3.59E-06	0.024224	0.003414
0.133958	-0.06124	0.046904	0.078371	-0.02624	0.051983	-0.07568	-0.02213	-0.18049
0.133231	0.493972	0.600526	0.381133	0.769648	0.561631	0.397728	0.804925	0.042293

CCS	CD2BP2	CD320	CD82	CDC14A	CDC27	CDC34	CDC37	CDK10
black	black	black	black	black	black	black	black	black
0.069041	-0.1782	-0.45898	-0.13198	-0.0542	-0.03876	-0.29721	-0.15275	-0.23105
0.440543	0.045023	5.73E-08	0.139121	0.545063	0.665244	0.000691	0.086437	0.00896
0.036208	-0.11033	-0.43114	-0.1423	-0.06518	0.007493	-0.23562	-0.16514	-0.20913
0.686107	0.216908	4.19E-07	0.110519	0.466579	0.933367	0.007659	0.063545	0.018295
-0.30229	0.049474	0.318538	-0.18098	0.172654	0.263996	0.107443	-0.03075	0.202043
0.000552	0.580695	0.000262	0.041733	0.052249	0.002708	0.229234	0.731485	0.02273
-0.53633	-0.34177	-0.06214	-0.38037	0.503586	0.537569	-0.42816	-0.40796	-0.17116
8.1E-11	8.4E-05	0.487682	1.03E-05	1.59E-09	7.19E-11	5.13E-07	1.93E-06	0.05435
0.234545	0.253462	0.107295	-0.2149	0.079046	0.101225	0.059848	-0.20031	0.073824
0.00795	0.004037	0.229881	0.015254	0.377034	0.257474	0.503885	0.023946	0.409456
-0.34338	-0.26636	-0.18647	-0.47235	0.532984	0.576937	-0.52362	-0.61205	-0.28684
7.74E-05	0.002471	0.035805	2.06E-08	1.11E-10	1.25E-12	2.67E-10	2.09E-14	0.001077
-0.2532	-0.10758	-0.16305	-0.21325	0.297888	0.358735	-0.38955	-0.30088	-0.11464
0.004076	0.228637	0.067005	0.016074	0.00067	3.45E-05	5.99E-06	0.000588	0.199359
-0.04316	-0.04826	-0.27282	-0.13891	0.25732	0.019511	-0.38813	-0.39855	-0.12843
0.629912	0.589999	0.001914	0.119355	0.003494	0.827644	6.52E-06	3.47E-06	0.150143
-0.03653	-0.2704	0.248505	0.051785	-0.06551	0.139469	-0.19228	0.254963	-0.01609
0.683431	0.002108	0.004845	0.563124	0.464322	0.117853	0.030335	0.003818	0.857525
0.386132	-0.12751	-0.03987	0.221639	-0.05293	-0.13418	-0.35987	0.06228	-0.15429
7.34E-06	0.153125	0.65631	0.01227	0.554553	0.132596	3.25E-05	0.486682	0.083279
0.092566	-0.29229	-0.21776	0.016867	0.137405	0.008687	-0.46291	-0.17012	-0.27798
0.300636	0.000855	0.013918	0.850709	0.123442	0.922785	4.26E-08	0.055854	0.001554
0.043897	-0.15687	0.142116	0.032824	-0.36866	-0.26803	0.225299	0.465663	0.038064
0.624102	0.078197	0.110974	0.714107	2E-05	0.002314	0.010874	3.46E-08	0.670929
0.180265	0.201498	0.447358	0.386608	-0.37437	-0.42433	0.445338	0.615246	0.428679
0.042556	0.023107	1.34E-07	7.14E-06	1.45E-05	6.64E-07	1.55E-07	1.4E-14	4.95E-07
0.722007	0.568569	0.777296	0.588738	-0.51519	-0.65002	0.841601	0.785802	0.533607
9.89E-22	3.1E-12	6.35E-27	3.35E-13	5.74E-10	1.35E-16	3.06E-35	7.43E-28	1.05E-10
0.652276	0.231038	0.444383	0.637528	-0.47989	-0.63069	0.315425	0.678902	0.300446
9.8E-17	0.008965	1.66E-07	7.67E-16	1.14E-08	1.92E-15	0.000304	1.76E-18	0.000599
0.243075	0.218227	-0.26082	0.248608	-0.31396	-0.30605	0.103158	0.082502	-0.05461
0.005893	0.013711	0.003059	0.004827	0.000325	0.000467	0.248452	0.356461	0.541986
-0.04658	0.326279	0.15441	0.123185	-0.23935	-0.23643	0.585183	0.282449	0.241005
0.603033	0.000181	0.083039	0.167662	0.006726	0.007449	5.01E-13	0.001294	0.006343
-0.60578	-0.21005	-0.43154	-0.44138	0.398606	0.508613	-0.14507	-0.50108	-0.29
4.5E-14	0.017777	4.08E-07	2.06E-07	3.46E-06	1.03E-09	0.103673	1.97E-09	0.000942
-0.20237	0.060349	-0.29999	-0.10681	-0.07534	0.025745	0.18928	-0.09667	-0.06789
0.022504	0.500318	0.000611	0.232021	0.399908	0.773871	0.033065	0.279614	0.448223
-0.07837	0.143378	0.13196	-0.18635	-0.03682	0.064537	0.179702	0.151832	0.324424
0.381145	0.107806	0.139171	0.035932	0.681119	0.471	0.043217	0.088375	0.000198

CDK11B	CDK2AP2	CENPB	CENPC	CEP131	CERS1	CFP	CHCHD5	CHID1
black	black	black	black	black	black	black	black	black
-0.18535	-0.07475	-0.15293	0.064354	-0.27996	0.007866	-0.20436	-0.09949	-0.04141
0.036961	0.403602	0.086065	0.472261	0.001434	0.930061	0.021188	0.265749	0.643906
-0.16014	-0.06494	-0.16861	0.025702	-0.34086	0.034493	-0.20519	-0.0682	-0.04949
0.072096	0.468242	0.058105	0.774239	8.8E-05	0.700245	0.020657	0.446142	0.580552
0.092516	-0.18805	-0.02512	0.307529	0.159532	-0.33227	-0.02178	-0.04701	-0.15984
0.300896	0.034241	0.77926	0.000436	0.073205	0.000135	0.807937	0.599729	0.072646
-0.26106	-0.53186	-0.37018	0.560684	-0.30311	-0.63486	-0.25247	-0.37619	-0.35986
0.003031	1.24E-10	1.84E-05	7.12E-12	0.000532	1.1E-15	0.004188	1.31E-05	3.25E-05
-0.16765	0.147979	-0.15328	0.117669	-0.38361	-0.24863	0.069345	0.565863	0.386561
0.059558	0.096854	0.08534	0.18766	8.51E-06	0.004824	0.438524	4.14E-12	7.15E-06
-0.48357	-0.42983	-0.51012	0.592285	-0.65826	-0.71674	-0.23851	-0.06128	-0.10776
8.46E-09	4.58E-07	9.01E-10	2.23E-13	4.11E-17	2.66E-21	0.006925	0.49375	0.227839
-0.07017	-0.33776	-0.39835	0.358844	-0.59974	-0.54737	-0.07802	-0.12771	0.025679
0.433077	0.000103	3.51E-06	3.43E-05	9.3E-14	2.76E-11	0.383299	0.152488	0.77444
-0.15988	-0.15738	-0.15836	0.110405	-0.23519	-0.04796	-0.21322	-0.09591	-0.1288
0.072573	0.077211	0.075363	0.216576	0.007774	0.592371	0.016091	0.283432	0.148982
0.151302	-0.01434	0.106937	0.053336	0.147454	-0.26865	0.207929	-0.09031	0.250178
0.089504	0.872834	0.231446	0.551477	0.098057	0.002258	0.018988	0.312609	0.004558
0.033191	0.232551	0.216775	0.030014	-0.05368	0.024421	0.233059	0.150499	0.315726
0.711052	0.008514	0.014367	0.737644	0.548928	0.78521	0.008367	0.091239	0.000299
-0.11979	-0.09568	-0.04224	0.144754	-0.17967	-0.13386	-0.0555	-0.10553	0.036401
0.179777	0.284618	0.637272	0.104435	0.04326	0.133523	0.535397	0.237675	0.684526
0.335923	0.043874	0.144004	-0.46091	0.349599	0.169197	0.102486	-0.08927	0.103051
0.000113	0.62429	0.106262	4.95E-08	5.61E-05	0.057219	0.251564	0.318233	0.248946
0.636686	0.298929	0.540497	-0.29063	0.776501	0.397574	0.271515	-0.03253	0.031329
8.6E-16	0.000641	5.42E-11	0.000917	7.72E-27	3.69E-06	0.002017	0.716515	0.7266
0.689751	0.85433	0.817181	-0.61638	0.819615	0.682165	0.553655	0.727167	0.592688
3.03E-19	2.47E-37	1.06E-31	1.22E-14	5E-32	1.05E-18	1.47E-11	3.67E-22	2.12E-13
0.653847	0.673275	0.792913	-0.44605	0.74053	0.604747	0.496455	0.311153	0.413735
7.81E-17	4.26E-18	1.14E-28	1.48E-07	2.53E-23	5.1E-14	2.92E-09	0.00037	1.33E-06
0.127205	0.295088	0.294436	-0.28582	0.088178	0.555047	-0.01551	0.044183	-0.1001
0.154114	0.000757	0.000779	0.001124	0.324226	1.27E-11	0.862611	0.621845	0.262821
0.268434	0.125865	0.19569	-0.33316	0.384991	0.409232	-0.00838	0.02901	-0.19514
0.002278	0.158534	0.027463	0.00013	7.85E-06	1.78E-06	0.925493	0.746121	0.02791
-0.56196	-0.5794	-0.60288	0.263225	-0.4896	-0.31993	-0.449	-0.36769	-0.4887
6.23E-12	9.57E-13	6.39E-14	0.00279	5.17E-09	0.000246	1.19E-07	2.11E-05	5.57E-09
-0.24827	-0.16134	-0.28082	-0.19063	-0.18851	0.151038	-0.23726	-0.12939	-0.2016
0.004886	0.069964	0.001384	0.031809	0.0338	0.090072	0.007236	0.147107	0.023032
0.202309	-0.02992	-0.05991	0.236697	0.19975	-0.28351	0.132213	0.012321	-0.09899
0.022548	0.738404	0.50345	0.007379	0.024351	0.001238	0.138407	0.890645	0.268185

CHM	CHMP6	CHST12	CHUK	CHURC1	CITED4	CLCN7	CLEC11A	CLOCK
black	black	black	black	black	black	black	black	black
0.116518	-0.28214	-0.28544	0.139837	0.222105	-0.01241	-0.00844	-0.27464	0.148241
0.192045	0.001311	0.001142	0.116878	0.012084	0.889861	0.92498	0.001779	0.096257
0.143145	-0.28241	-0.33128	0.184028	0.188581	-0.02516	-0.0783	-0.37633	0.120175
0.108386	0.001296	0.000142	0.038349	0.033731	0.778871	0.381581	1.3E-05	0.17837
-0.0547	0.077156	0.139255	-0.02711	-0.15451	-0.37177	-0.28626	0.179862	-0.13605
0.541353	0.388583	0.118423	0.762212	0.082846	1.68E-05	0.001104	0.043028	0.127213
0.2589	-0.30934	-0.11084	0.344171	0.30828	-0.68045	-0.59326	-0.1132	0.152204
0.003292	0.000402	0.214757	7.43E-05	0.000422	1.38E-18	1.99E-13	0.205104	0.087589
0.063598	0.181971	-0.00302	0.179276	0.056818	-0.04032	-0.47066	0.006175	-0.12547
0.477492	0.040603	0.97311	0.043723	0.525759	0.652643	2.35E-08	0.945065	0.159847
0.377192	-0.28809	-0.1996	0.548939	0.475479	-0.61126	-0.81733	-0.19519	0.142497
1.24E-05	0.001022	0.024461	2.36E-11	1.61E-08	2.3E-14	1.02E-31	0.027869	0.11001
0.234196	-0.34481	-0.24167	0.335537	0.2583	-0.49499	-0.56943	-0.3028	0.097105
0.008046	7.19E-05	0.006195	0.000115	0.003367	3.31E-09	2.83E-12	0.00054	0.277452
0.191328	-0.28459	-0.3285	0.198732	0.378655	-0.09792	-0.21148	-0.31542	0.430519
0.031179	0.001184	0.000163	0.025101	1.14E-05	0.273421	0.016998	0.000304	4.37E-07
-0.26222	0.133457	0.453426	-0.08783	-0.00025	-0.11308	0.088142	0.516885	-0.25695
0.0029	0.134703	8.64E-08	0.326157	0.99778	0.205586	0.324424	4.93E-10	0.003544
-0.32114	0.050696	0.222603	0.164832	0.313655	0.102283	0.152718	0.225661	-0.1574
0.000232	0.57137	0.011888	0.064043	0.00033	0.252508	0.08651	0.010744	0.077171
-0.10198	-0.18988	0.049611	0.2044	0.420724	-0.11072	-0.04284	0.010308	0.020805
0.25393	0.032506	0.579647	0.021162	8.44E-07	0.215262	0.632463	0.908428	0.816408
-0.32867	0.263896	0.265323	-0.47721	-0.24938	0.202932	0.41908	0.151617	-0.1696
0.000161	0.002719	0.002572	1.41E-08	0.004693	0.022127	9.4E-07	0.088831	0.056626
-0.55808	0.32332	0.377605	-0.54279	-0.50028	0.2653	0.607028	0.37656	-0.26039
9.32E-12	0.000209	1.21E-05	4.33E-11	2.11E-09	0.002575	3.87E-14	1.28E-05	0.003111
-0.718	0.834337	0.709972	-0.5692	-0.59685	0.675714	0.713937	0.679612	-0.53782
2.1E-21	3.97E-34	9.17E-21	2.9E-12	1.31E-13	2.91E-18	4.46E-21	1.57E-18	7.02E-11
-0.80182	0.505502	0.604615	-0.43755	-0.30154	0.555231	0.811727	0.580513	-0.4003
9.88E-30	1.35E-09	5.19E-14	2.69E-07	0.000571	1.25E-11	5.58E-31	8.45E-13	3.12E-06
-0.1365	-0.01778	-0.18527	-0.023	0.001027	0.362734	0.348553	-0.26535	0.106708
0.125955	0.842732	0.037041	0.79743	0.990855	2.78E-05	5.92E-05	0.00257	0.232455
-0.08644	0.155991	-0.03662	-0.37537	-0.42745	0.255428	0.267397	-0.06907	-0.04955
0.333883	0.079897	0.682693	1.37E-05	5.38E-07	0.003752	0.002372	0.440353	0.580109
0.781691	-0.42576	-0.53004	0.32542	0.194262	-0.37825	-0.46782	-0.5042	0.393988
2.12E-27	6.03E-07	1.47E-10	0.000189	0.028635	1.16E-05	2.93E-08	1.51E-09	4.59E-06
0.449452	-0.19754	-0.42019	-0.04731	-0.10473	0.127572	0.001974	-0.41046	0.248214
1.15E-07	0.026004	8.74E-07	0.597351	0.241292	0.152921	0.982429	1.64E-06	0.004897
-0.03218	0.073783	-0.02918	-0.21399	-0.47886	-0.1175	-0.10895	0.107571	-0.13401
0.719487	0.409718	0.744722	0.015703	1.24E-08	0.188302	0.222712	0.228675	0.133087

CLPP	CLTC	CLUH	COASY	COG3	COG6	COMMD4	COMMD5	COMT
black	black	black	black	black	black	black	black	black
-0.29786	-0.00784	-0.28463	0.057413	-0.02692	-0.0111	-0.13521	-0.23773	-0.06719
0.000671	0.930323	0.001182	0.521432	0.763852	0.901399	0.129604	0.007119	0.452935
-0.267	-0.01135	-0.28734	0.080966	-0.03364	-0.00917	-0.11958	-0.21234	-0.07933
0.00241	0.899237	0.001055	0.365517	0.707328	0.918524	0.180561	0.016544	0.375303
0.17349	0.12715	0.041438	-0.18183	0.250254	0.084421	0.167537	0.152191	-0.11049
0.051103	0.154295	0.643679	0.040763	0.004545	0.345348	0.05974	0.087615	0.21623
-0.30538	0.322001	-0.45947	-0.37328	0.617966	0.423297	-0.11145	-0.11311	-0.40211
0.000481	0.000223	5.52E-08	1.54E-05	9.96E-15	7.11E-07	0.212244	0.205444	2.78E-06
0.120871	-0.42742	-0.479	0.295373	0.029364	0.350357	0.452495	0.544696	-0.12357
0.17585	5.39E-07	1.22E-08	0.000748	0.743124	5.39E-05	9.25E-08	3.59E-11	0.166326
-0.38296	0.078586	-0.83205	-0.21387	0.571609	0.631262	0.059544	0.11346	-0.5093
8.84E-06	0.379822	8.68E-34	0.015764	2.24E-12	1.78E-15	0.50606	0.204052	9.68E-10
-0.27031	0.004798	-0.63771	-0.08408	0.474126	0.483452	-0.00062	-0.01159	-0.31575
0.002115	0.957304	7.49E-16	0.347309	1.8E-08	8.54E-09	0.994474	0.897114	0.000299
-0.38939	0.059713	-0.37745	-0.11261	0.052995	0.102212	-0.01209	-0.15663	-0.26272
6.05E-06	0.504856	1.22E-05	0.207497	0.554028	0.252841	0.89268	0.078645	0.002845
0.133529	0.13635	-0.04218	0.145482	0.214236	-0.08992	0.06995	-0.03221	0.142145
0.13449	0.126378	0.637713	0.102682	0.01558	0.314732	0.434533	0.719268	0.110901
-0.10222	-0.20885	-0.28533	0.249104	0.049271	-0.13159	0.331064	0.032103	0.199501
0.25279	0.018451	0.001147	0.00474	0.582254	0.140304	0.000144	0.720117	0.024532
-0.32676	0.031938	-0.36771	-0.03798	0.170069	0.056485	0.079321	-0.09874	-0.01019
0.000177	0.721496	2.11E-05	0.671576	0.055931	0.528196	0.375368	0.269401	0.909508
0.226225	0.007076	0.352413	0.289325	-0.04073	-0.26405	-0.0085	-0.03418	0.435602
0.010544	0.93707	4.84E-05	0.00097	0.649372	0.002702	0.924423	0.702823	3.08E-07
0.473399	-0.05366	0.7444	0.16973	-0.23788	-0.60183	0.140856	-0.0439	0.505343
1.9E-08	0.54905	1.13E-23	0.056429	0.00708	7.25E-14	0.114209	0.624098	1.37E-09
0.874295	-0.55434	0.776528	0.624696	-0.61996	-0.58441	0.59918	0.581981	0.744638
4.7E-41	1.37E-11	7.67E-27	4.21E-15	7.74E-15	5.47E-13	9.93E-14	7.18E-13	1.08E-23
0.483967	-0.36169	0.576005	0.47973	-0.41067	-0.7224	0.437884	0.130592	0.72371
8.2E-09	2.94E-05	1.39E-12	1.15E-08	1.62E-06	9.18E-22	2.63E-07	0.143353	7.15E-22
-0.02617	-0.1423	0.19453	0.083612	-0.31839	-0.27117	0.0054	-0.13239	0.226187
0.770244	0.110515	0.028412	0.350006	0.000264	0.002045	0.951954	0.137889	0.010557
0.328951	0.010184	0.604796	-0.11042	-0.32992	-0.21356	-0.10111	0.075665	0.226411
0.000159	0.909524	5.07E-14	0.216517	0.000152	0.015917	0.257995	0.397843	0.010479
-0.41675	0.449723	-0.21904	-0.54433	0.21632	0.542553	-0.58232	-0.22823	-0.60615
1.1E-06	1.13E-07	0.013356	3.72E-11	0.014578	4.43E-11	6.91E-13	0.00986	4.31E-14
-0.12169	0.201983	0.167603	-0.20723	-0.22639	0.136618	-0.43677	-0.16859	-0.20959
0.172901	0.022771	0.059638	0.019404	0.010485	0.125627	2.84E-07	0.058132	0.018031
0.290291	-0.15933	0.137318	0.061887	0.144276	-0.07337	0.097354	0.116599	-0.00966
0.000931	0.073576	0.123683	0.489439	0.105595	0.412353	0.276212	0.191732	0.914205

COMTD1	COPE	COQ4	COX5B	COX8A	CPTP	CRACR2B	CRELD2	CRIP2
black	black	black	black	black	black	black	black	black
-0.12738	-0.10988	0.020097	0.119138	-0.14245	-0.25349	-0.07918	-0.19875	-0.22584
0.153552	0.218774	0.82255	0.182172	0.110122	0.004033	0.376212	0.025091	0.010682
-0.13372	-0.05754	0.001346	0.208309	-0.04691	-0.23617	-0.06514	-0.27847	-0.17717
0.133922	0.520474	0.988013	0.018766	0.600503	0.007516	0.466866	0.001524	0.046296
-0.18162	-0.15133	-0.14989	-0.50601	-0.13892	-0.0714	-0.13702	0.085736	-0.12957
0.040994	0.089451	0.092569	1.29E-09	0.119314	0.425072	0.124499	0.337856	0.146551
-0.48188	-0.57056	-0.31339	-0.65143	-0.5902	-0.5539	-0.28735	-0.09139	-0.61401
9.7E-09	2.51E-12	0.000334	1.11E-16	2.83E-13	1.43E-11	0.001054	0.306858	1.64E-14
0.205466	0.115049	0.461478	0.65512	0.333633	-0.10993	0.156457	-0.06306	-0.06454
0.020484	0.197748	4.75E-08	6.5E-17	0.000127	0.218588	0.078989	0.481215	0.470959
-0.34252	-0.52197	-0.06939	-0.11474	-0.41595	-0.66353	-0.18913	-0.20406	-0.6823
8.09E-05	3.11E-10	0.438252	0.198955	1.15E-06	1.88E-17	0.033204	0.02138	1.02E-18
-0.37051	-0.33432	0.089912	-0.08918	-0.20682	-0.59037	-0.1445	-0.18959	-0.51203
1.8E-05	0.000122	0.314765	0.31873	0.019648	2.78E-13	0.105055	0.032771	7.61E-10
-0.23534	-0.27648	0.279876	0.077575	-0.30169	-0.31595	0.123053	-0.17487	-0.21541
0.007734	0.001652	0.001439	0.386005	0.000567	0.000296	0.16812	0.049257	0.015008
-0.02213	0.033033	-0.13176	-0.50476	-0.07655	-0.04592	-0.00884	0.505964	-0.15924
0.804922	0.712364	0.139783	1.44E-09	0.392329	0.608166	0.921398	1.3E-09	0.07373
0.112788	0.105128	0.246548	0.064447	-0.02701	-0.14134	0.191919	0.37281	-0.19678
0.206762	0.239483	0.005202	0.471615	0.763042	0.11295	0.030651	1.59E-05	0.026592
-0.12443	-0.1649	0.005126	-0.18194	-0.30678	-0.31119	-0.02509	0.13219	-0.37033
0.16339	0.063936	0.954391	0.040643	0.000451	0.000369	0.779439	0.138476	1.82E-05
0.142772	0.433695	-0.11682	-0.25123	0.332823	0.289636	-0.09215	0.290397	0.211735
0.109318	3.52E-07	0.190885	0.004384	0.000132	0.000957	0.302828	0.000927	0.016862
0.159537	0.374135	0.045134	-0.41766	0.190321	0.496705	0.169012	0.449633	0.407894
0.073196	1.47E-05	0.614355	1.03E-06	0.032094	2.86E-09	0.057496	1.14E-07	1.94E-06
0.79065	0.86594	0.492712	0.454568	0.760344	0.916371	0.613974	0.651916	0.832124
2.09E-28	2E-39	4E-09	7.94E-08	3.49E-25	1.56E-51	1.64E-14	1.03E-16	8.46E-34
0.474347	0.616702	0.352773	0.029362	0.38744	0.573715	0.476793	0.724293	0.461889
1.76E-08	1.17E-14	4.75E-05	0.743141	6.79E-06	1.78E-12	1.46E-08	6.39E-22	4.6E-08
0.111839	0.215478	0.182693	0.477054	0.23629	0.180505	0.118717	-0.15716	0.294391
0.210632	0.014976	0.039799	1.43E-08	0.007484	0.042276	0.183732	0.077628	0.000781
0.078765	0.273085	-0.0566	0.097174	0.325376	0.426622	-0.09113	-0.17111	0.548909
0.378738	0.001894	0.527368	0.277105	0.000189	5.69E-07	0.308235	0.054424	2.36E-11
-0.34435	-0.51193	-0.49886	-0.03645	-0.34121	-0.31743	-0.45067	-0.71812	-0.20286
7.37E-05	7.67E-10	2.39E-09	0.684089	8.65E-05	0.000276	1.06E-07	2.06E-21	0.022174
-0.0529	-0.03572	-0.19139	0.322589	0.11319	0.092268	-0.22946	-0.54764	0.253355
0.554716	0.690111	0.031119	0.000217	0.205137	0.302198	0.009458	2.68E-11	0.004053
-0.05787	-0.01582	0.059778	-0.18204	0.220804	0.031549	-0.08648	-0.01615	0.049086
0.518146	0.859875	0.504386	0.04053	0.01261	0.724755	0.333653	0.857002	0.583673

CROCC	CRYAA	CSNK1G2	CTBP1	CTDP1	CTU1	CTU2	CUEDC1	CXXC1
black	black	black	black	black	black	black	black	black
-0.14159	-0.20663	-0.24099	-0.11963	-0.17208	-0.38424	-0.3642	-0.16773	-0.23493
0.112318	0.019767	0.006346	0.180372	0.053047	8.2E-06	2.56E-05	0.059438	0.007844
-0.21922	-0.18031	-0.3133	-0.12064	-0.25972	-0.38721	-0.34201	-0.18733	-0.26543
0.013276	0.042507	0.000335	0.176694	0.003191	6.89E-06	8.3E-05	0.034952	0.002562
-0.19223	0.072632	0.005001	-0.23341	0.028411	0.274765	0.239925	0.077844	0.146242
0.030381	0.41708	0.955504	0.008268	0.751186	0.001771	0.00659	0.384353	0.100879
-0.49418	-0.23301	-0.42523	-0.66116	-0.33115	-0.13091	-0.26944	-0.35371	-0.32498
3.54E-09	0.008382	6.25E-07	2.68E-17	0.000143	0.142381	0.00219	4.52E-05	0.000193
-0.29652	-0.17744	-0.33209	-0.2412	-0.31993	-0.15604	0.130379	-0.17158	-0.14993
0.000712	0.045962	0.000137	0.006299	0.000246	0.079802	0.144011	0.053748	0.092481
-0.66116	-0.41623	-0.68996	-0.80058	-0.58002	-0.40854	-0.34807	-0.5327	-0.56721
2.68E-17	1.13E-06	2.92E-19	1.4E-29	8.94E-13	1.86E-06	6.08E-05	1.15E-10	3.59E-12
-0.53708	-0.46801	-0.56023	-0.60861	-0.54214	-0.47362	-0.37576	-0.46713	-0.41772
7.54E-11	2.89E-08	7.46E-12	3.19E-14	4.61E-11	1.87E-08	1.34E-05	3.09E-08	1.03E-06
0.061079	-0.23443	-0.31915	-0.27073	-0.29326	-0.32407	-0.31916	-0.21783	-0.18152
0.495138	0.007982	0.000255	0.002081	0.00082	0.000202	0.000255	0.013887	0.041115
0.013138	-0.10935	0.227406	-0.0629	0.292295	0.103488	-0.07638	-0.05921	-0.02294
0.883446	0.221033	0.010135	0.482329	0.000854	0.246932	0.393371	0.508462	0.797925
0.120656	-0.15222	0.016492	-0.05981	0.207098	-0.21384	-0.28586	0.042467	-0.09362
0.176625	0.087557	0.853991	0.504181	0.019481	0.015776	0.001122	0.635456	0.295114
-0.08449	-0.25791	-0.16698	-0.2524	-0.03088	-0.27877	-0.37726	-0.16826	-0.28678
0.344938	0.003418	0.060602	0.0042	0.730358	0.001505	1.23E-05	0.058632	0.00108
0.129898	0.162617	0.308277	0.428247	0.333586	0.267324	0.146074	0.137027	0.242588
0.14551	0.067752	0.000422	5.1E-07	0.000127	0.002379	0.101276	0.124488	0.005996
0.523144	0.408227	0.661513	0.531105	0.667167	0.613478	0.388976	0.563407	0.676823
2.79E-10	1.9E-06	2.54E-17	1.33E-10	1.09E-17	1.75E-14	6.2E-06	5.36E-12	2.45E-18
0.723254	0.485413	0.861565	0.828588	0.810236	0.787382	0.811736	0.756873	0.787459
7.8E-22	7.29E-09	1.29E-38	2.77E-33	8.69E-31	4.93E-28	5.56E-31	7.62E-25	4.83E-28
0.724605	0.337562	0.73081	0.648003	0.840021	0.47053	0.28061	0.663643	0.607586
6.02E-22	0.000104	1.8E-22	1.8E-16	5.4E-35	2.38E-08	0.001396	1.85E-17	3.62E-14
0.276869	0.199578	0.052305	0.386669	0.100755	-0.08366	-0.07109	0.350445	0.132003
0.001626	0.024476	0.559207	7.11E-06	0.2597	0.349725	0.427063	5.37E-05	0.13904
0.213084	0.364969	0.31648	0.462722	0.164501	0.339912	0.439255	0.385476	0.393793
0.01616	2.46E-05	0.000289	4.32E-08	0.064586	9.24E-05	2.39E-07	7.63E-06	4.64E-06
-0.46415	-0.18287	-0.44409	-0.34932	-0.62109	-0.31794	-0.16806	-0.46032	-0.45601
3.88E-08	0.039603	1.7E-07	5.69E-05	6.69E-15	0.00027	0.058934	5.18E-08	7.14E-08
-0.05781	0.016766	-0.07728	0.162235	-0.31299	-0.17219	0.03143	-0.15475	-0.10898
0.518542	0.851593	0.387843	0.068407	0.00034	0.0529	0.725749	0.082347	0.22262
-0.06783	0.075948	0.073163	0.025229	0.108296	0.197305	0.245157	0.120279	0.323694
0.448595	0.396072	0.413672	0.778288	0.225537	0.026186	0.00547	0.177989	0.000205

CYC1	CYHR1	DAPK3	DAZAP1	DCTN5	DCUN1D4	DCXR	DDAH2	DDRGK1
black	black	black	black	black	black	black	black	black
-0.27976	-0.03248	-0.2366	-0.16885	-0.20868	0.244034	-0.20634	-0.01047	0.047244
0.001445	0.716967	0.007404	0.057743	0.018552	0.005695	0.019939	0.906991	0.597884
-0.18805	-0.01287	-0.29598	-0.23827	-0.20361	0.277794	-0.05409	-0.0243	0.018464
0.034242	0.885787	0.000729	0.006984	0.021677	0.001566	0.54588	0.786246	0.836756
-0.0202	-0.28586	0.08113	0.161667	0.372802	-0.01296	-0.03884	0.095594	-0.40763
0.821664	0.001123	0.364542	0.069393	1.59E-05	0.88499	0.664609	0.285032	1.97E-06
-0.36412	-0.6186	-0.30463	-0.24669	0.431818	0.279247	-0.46018	-0.31571	-0.66162
2.57E-05	9.19E-15	0.000497	0.005175	4E-07	0.001476	5.23E-08	0.0003	2.5E-17
0.518855	0.180685	-0.33025	-0.15421	-0.1953	0.189755	0.41804	0.083818	-0.21049
4.13E-10	0.042068	0.00015	0.083435	0.027782	0.032619	1.01E-06	0.348813	0.017532
-0.11602	-0.49576	-0.5897	-0.45731	0.205469	0.410251	-0.27016	-0.34945	-0.68877
0.193977	3.1E-09	3E-13	6.48E-08	0.020482	1.67E-06	0.002127	5.65E-05	3.56E-19
-0.0831	-0.25837	-0.48735	-0.41179	0.308814	0.178021	-0.24039	-0.28158	-0.49516
0.352946	0.003359	6.22E-09	1.51E-06	0.000412	0.045244	0.006483	0.001341	3.26E-09
-0.12625	-0.00389	-0.31226	-0.29165	0.012411	0.412016	-0.3651	-0.11398	-0.25475
0.15725	0.965416	0.000352	0.000878	0.889853	1.49E-06	2.44E-05	0.201957	0.003848
-0.35919	-0.23288	0.3375	0.265549	0.072268	-0.38728	-0.46078	0.052389	0.005997
3.37E-05	0.008417	0.000104	0.00255	0.419424	6.86E-06	5E-08	0.558579	0.946646
-0.32611	0.035038	0.047747	0.119211	-0.33357	-0.14777	-0.40331	0.203754	0.176128
0.000183	0.695742	0.593988	0.181901	0.000127	0.097338	2.58E-06	0.021582	0.047619
-0.44668	-0.23061	-0.11957	-0.10161	-0.13091	0.048136	-0.50476	0.018385	-0.07
1.41E-07	0.009097	0.180563	0.255642	0.142358	0.590979	1.44E-09	0.837451	0.434179
-0.02513	0.110974	0.374914	0.186412	-0.05405	-0.29049	0.035271	0.097175	0.271743
0.779134	0.214203	1.41E-05	0.035868	0.546134	0.000923	0.693817	0.277102	0.001998
-0.09102	0.26248	0.666765	0.632621	0.019581	-0.42138	0.031894	0.467115	0.370569
0.30881	0.002871	1.16E-17	1.48E-15	0.827037	8.08E-07	0.721866	3.09E-08	1.8E-05
0.534501	0.764942	0.811427	0.766456	-0.52599	-0.51637	0.599913	0.729234	0.733584
9.65E-11	1.22E-25	6.1E-31	8.57E-26	2.15E-10	5.16E-10	9.11E-14	2.45E-22	1.04E-22
-0.09371	0.513477	0.719794	0.715736	-0.45573	-0.53184	-0.00836	0.650644	0.681666
0.294674	6.69E-10	1.5E-21	3.2E-21	7.29E-08	1.24E-10	0.925639	1.24E-16	1.13E-18
0.067232	0.329397	-0.05972	0.049027	-0.31955	0.138549	0.257359	0.108924	0.389147
0.452638	0.000156	0.504821	0.584124	0.00025	0.120321	0.003489	0.222844	6.14E-06
0.386476	0.237609	0.240007	0.162284	0.12779	-0.07827	0.506291	0.166356	0.178377
7.19E-06	0.007148	0.006571	0.068324	0.152212	0.381776	1.26E-09	0.061589	0.044808
0.066822	-0.43154	-0.49593	-0.57346	0.374095	0.410231	0.047891	-0.63101	-0.4028
0.455407	4.08E-07	3.05E-09	1.83E-12	1.47E-05	1.67E-06	0.592871	1.84E-15	2.67E-06
0.298184	0.057102	-0.19102	-0.32252	0.12873	0.129369	0.335159	-0.303	0.071263
0.000662	0.523689	0.031459	0.000217	0.149194	0.147172	0.000117	0.000535	0.425935
0.162816	0.011582	0.021823	0.296343	0.189792	-0.00925	0.237289	0.110535	-0.22686
0.067411	0.89717	0.807597	0.000717	0.032585	0.917774	0.007228	0.216032	0.010321

DDX49	DDX5	DDX54	DDX6	DECR2	DENND4A	DENND4C	DGAT1	DGCR6L
black	black	black	black	black	black	black	black	black
-0.32028	-0.01785	-0.20855	-0.05849	-0.24056	0.224046	-0.07206	-0.28207	-0.01545
0.000242	0.842117	0.018628	0.513662	0.006445	0.011336	0.420747	0.001314	0.863102
-0.29559	-0.09233	-0.23348	-0.04746	-0.22361	0.15641	-0.18529	-0.23631	0.005071
0.000741	0.301885	0.008246	0.59621	0.011501	0.079081	0.037014	0.007479	0.954873
0.322339	0.299885	-0.05239	0.336587	0.114869	-0.32422	0.055723	0.110319	-0.20113
0.000219	0.000614	0.558602	0.000109	0.198455	0.0002	0.533785	0.21694	0.023363
-0.19628	0.475552	-0.48505	0.364427	-0.21196	0.212765	0.430111	-0.40818	-0.60038
0.02699	1.6E-08	7.5E-09	2.53E-05	0.016741	0.016324	4.49E-07	1.9E-06	8.61E-14
0.092621	-0.41899	-0.27504	-0.53139	0.202698	-0.20397	-0.43712	-0.07025	0.431024
0.300344	9.46E-07	0.001751	1.3E-10	0.022285	0.021439	2.77E-07	0.432551	4.22E-07
-0.33144	0.125842	-0.68846	-0.06854	-0.20128	0.268414	0.200838	-0.58789	-0.31538
0.000141	0.158613	3.75E-19	0.443846	0.02326	0.00228	0.02357	3.69E-13	0.000304
-0.24664	0.128242	-0.59636	-0.02946	-0.24843	0.185883	0.069134	-0.34107	-0.29419
0.005185	0.150758	1.39E-13	0.742333	0.004858	0.036407	0.439927	8.71E-05	0.000788
-0.3838	0.157893	-0.30754	-0.17086	-0.18549	0.397474	0.277673	-0.44079	-0.19292
8.42E-06	0.076243	0.000436	0.054775	0.036815	3.71E-06	0.001574	2.14E-07	0.029779
0.086734	0.397752	0.141588	0.057529	0.068442	-0.09802	0.407634	-0.01493	-0.34373
0.332243	3.65E-06	0.11232	0.520587	0.444525	0.272908	1.97E-06	0.867679	7.6E-05
-0.17903	0.145759	-0.00925	-0.31634	-0.02904	0.227001	0.337602	-0.30013	-0.08408
0.044021	0.102024	0.917755	0.000291	0.74583	0.010274	0.000104	0.000607	0.347299
-0.30408	0.214597	-0.19383	-0.0566	-0.19112	0.403838	0.373273	-0.44624	-0.28151
0.00051	0.015403	0.028998	0.52736	0.031367	2.5E-06	1.54E-05	1.45E-07	0.001345
0.214867	0.085418	0.364164	0.26636	0.117108	-0.18314	-0.01279	0.459684	-0.15359
0.015271	0.339657	2.57E-05	0.002471	0.189787	0.03931	0.886516	5.43E-08	0.084712
0.502325	0.269352	0.5985	0.332163	0.271847	-0.4112	0.02332	0.591174	-0.02291
1.77E-09	0.002197	1.08E-13	0.000136	0.00199	1.57E-06	0.794682	2.53E-13	0.798247
0.799904	-0.34175	0.866929	-0.39987	0.754516	-0.64167	-0.40122	0.726981	0.721657
1.69E-29	8.41E-05	1.3E-39	3.2E-06	1.28E-24	4.35E-16	2.94E-06	3.81E-22	1.06E-21
0.398762	0.09295	0.700097	-0.16147	0.37651	-0.27665	0.088409	0.425086	0.211738
3.43E-06	0.298626	5.25E-20	0.069746	1.28E-05	0.001641	0.322954	6.31E-07	0.016861
-0.0756	-0.16163	0.106559	-0.04973	-0.0789	0.022149	-0.27937	0.077439	0.292994
0.398257	0.069458	0.233111	0.578709	0.377927	0.804779	0.001469	0.386836	0.000829
0.404763	-0.16263	0.316739	0.267907	0.165128	-0.38244	-0.41833	0.510581	0.312126
2.36E-06	0.067732	0.000286	0.002325	0.063559	9.11E-06	9.88E-07	8.65E-10	0.000354
-0.34673	-0.1127	-0.4237	0.251032	-0.36319	0.280099	-0.03164	-0.28729	-0.1268
6.51E-05	0.207136	6.92E-07	0.004417	2.71E-05	0.001426	0.72403	0.001057	0.155425
-0.11728	-0.35538	-0.03802	0.03194	-0.18292	0.058878	-0.3463	0.093885	0.236792
0.189128	4.13E-05	0.671269	0.721486	0.039554	0.510841	6.66E-05	0.293767	0.007354
0.248915	0.120885	0.012735	0.285196	0.086675	-0.54534	-0.25714	0.423289	0.045641
0.004773	0.175798	0.887001	0.001154	0.332571	3.37E-11	0.003518	7.12E-07	0.610385

DHPS	DHRS4	DHX30	DKK2	DMAP1	DMGDH	DMXL1	DNAJC16	DNAJC4
black	black	black	black	black	black	black	black	black
-0.24221	0.064678	-0.26175	0.191472	-0.03226	0.255749	0.118577	0.203716	0.080579
0.006078	0.470029	0.002952	0.031049	0.71881	0.003707	0.184255	0.021607	0.367819
-0.11641	0.036334	-0.27289	0.156728	0.034913	0.370721	0.049929	0.210134	0.079667
0.192446	0.685075	0.001909	0.078466	0.696773	1.78E-05	0.577217	0.01773	0.373286
0.273401	-0.06119	0.171723	-0.00489	-0.02335	-0.23213	0.024287	-0.20953	-0.34537
0.00187	0.49434	0.053552	0.956485	0.794444	0.008638	0.786367	0.018067	6.99E-05
-0.13053	-0.26324	-0.38144	0.222237	-0.51536	0.065448	0.436355	0.218778	-0.49302
0.143541	0.002788	9.66E-06	0.012032	5.66E-10	0.464744	2.92E-07	0.013468	3.9E-09
0.314179	0.217388	-0.28101	0.031273	0.188895	0.270405	-0.44153	-0.2179	0.38229
0.000322	0.014086	0.001373	0.727063	0.033431	0.002107	2.04E-07	0.013854	9.2E-06
-0.16008	-0.0975	-0.69493	0.296731	-0.50246	0.33261	0.212761	0.196295	-0.17436
0.072208	0.275507	1.27E-19	0.000705	1.75E-09	0.000133	0.016326	0.026979	0.049932
0.029622	-0.24648	-0.43926	0.182481	-0.31619	0.36946	0.030929	0.152246	-0.0925
0.740951	0.005214	2.39E-07	0.040034	0.000293	1.91E-05	0.729956	0.0875	0.300968
-0.36175	-0.19989	-0.25957	-0.09277	-0.23189	0.229279	0.254993	0.40477	0.012137
2.93E-05	0.024246	0.003209	0.299561	0.00871	0.009516	0.003813	2.36E-06	0.892276
-0.14851	0.107571	0.011973	0.140534	-0.34423	-0.26405	-0.10298	-0.0334	-0.01229
0.095637	0.228676	0.893719	0.115046	7.41E-05	0.002703	0.249282	0.709333	0.890912
-0.47272	0.379453	-0.28862	-0.06363	-0.24579	-0.04989	-0.12305	0.000575	0.398296
2E-08	1.08E-05	0.000999	0.477237	0.005346	0.577524	0.168113	0.994884	3.53E-06
-0.51138	0.249762	-0.38673	0.020835	-0.36557	0.085729	0.140313	0.148422	0.11911
8.06E-10	0.004628	7.09E-06	0.816145	2.38E-05	0.337896	0.115626	0.095847	0.182275
0.288061	-0.03181	0.337763	-0.08723	0.185801	-0.03852	-0.26656	-0.05924	-0.02732
0.001023	0.72259	0.000103	0.329491	0.036491	0.667239	0.002452	0.50827	0.760418
0.271455	0.089137	0.734145	-0.37077	0.38972	-0.48036	-0.15151	-0.27696	-0.0124
0.002021	0.318972	9.27E-23	1.78E-05	5.93E-06	1.1E-08	0.089069	0.00162	0.889967
0.507839	0.566571	0.703449	-0.4961	0.704862	-0.46599	-0.63522	-0.58298	0.656921
1.1E-09	3.84E-12	2.93E-20	3.01E-09	2.28E-20	3.37E-08	1.05E-15	6.42E-13	5E-17
-0.04965	0.506949	0.501877	-0.43975	0.325512	-0.44334	-0.37772	-0.33807	0.532373
0.579335	1.19E-09	1.84E-09	2.31E-07	0.000188	1.79E-07	1.2E-05	0.000101	1.18E-10
-0.07196	0.07777	0.099149	-0.1616	0.327075	0.112283	-0.06837	0.004578	0.187817
0.421381	0.384807	0.267414	0.06951	0.000175	0.208814	0.445009	0.959264	0.034471
0.486185	-0.14221	0.562364	-0.16858	0.511593	-0.20569	-0.14544	-0.29848	-0.18998
6.85E-09	0.110724	5.98E-12	0.058143	7.91E-10	0.020343	0.102784	0.000653	0.032413
-0.0302	-0.4903	-0.3204	0.425395	-0.26933	0.292871	0.497493	0.330479	-0.55483
0.736111	4.88E-09	0.00024	6.18E-07	0.002199	0.000833	2.68E-09	0.000148	1.3E-11
0.148433	-0.33722	0.08877	0.145068	0.172916	0.172679	0.081228	0.123663	-0.2135
0.095823	0.000106	0.320974	0.103676	0.051887	0.052214	0.363963	0.166005	0.015947
0.511478	-0.15015	0.39813	0.03971	0.333768	-0.14331	-0.14676	-0.2348	-0.20673
7.99E-10	0.092002	3.56E-06	0.657577	0.000126	0.107985	0.099657	0.007882	0.019706

DNASE1L2	DNPH1	DPM3	DPP7	DPP8	DRAP1	DRD4	DUS1L	DUS3L
black	black	black	black	black	black	black	black	black
-0.208	-0.17247	-0.23575	-0.02104	-0.08857	-0.06179	-0.27389	-0.12992	-0.30177
0.018945	0.052506	0.007626	0.814406	0.322079	0.490155	0.001834	0.145426	0.000565
-0.14577	-0.16667	-0.21407	-0.03557	-0.15345	-0.09895	-0.26836	-0.16282	-0.32727
0.102005	0.061089	0.015665	0.691319	0.085001	0.268355	0.002285	0.067403	0.000173
0.131761	-0.21889	-0.03534	-0.31292	-0.01879	-0.18744	0.064173	0.004241	0.235543
0.139774	0.013421	0.693262	0.000341	0.833878	0.034844	0.473509	0.962257	0.00768
-0.26113	-0.45497	-0.31605	-0.57602	0.218842	-0.56929	-0.25136	-0.39655	-0.22074
0.003023	7.71E-08	0.000295	1.39E-12	0.01344	2.87E-12	0.004364	3.92E-06	0.012638
0.037793	0.281797	0.431764	0.07742	-0.47884	0.29501	-0.0811	-0.03244	-0.17879
0.673135	0.001329	4.01E-07	0.386953	1.24E-08	0.00076	0.364744	0.717288	0.044313
-0.36862	-0.25918	-0.10813	-0.47313	-0.01113	-0.37118	-0.39124	-0.48217	-0.49139
2.01E-05	0.003257	0.22625	1.94E-08	0.901187	1.74E-05	5.41E-06	9.48E-09	4.46E-09
-0.24946	-0.32643	-0.23278	-0.32176	-0.04596	-0.35258	-0.40071	-0.39172	-0.4496
0.004678	0.00018	0.008447	0.000225	0.607891	4.8E-05	3.04E-06	5.26E-06	1.14E-07
-0.1406	0.006972	-0.00927	-0.0358	0.10079	-0.24296	-0.01028	-0.27038	-0.37285
0.114881	0.937993	0.917642	0.689424	0.259538	0.005918	0.908695	0.00211	1.58E-05
-0.22005	-0.18973	-0.04817	-0.02745	-0.12226	-0.11968	-0.09937	0.227949	0.202328
0.012925	0.032645	0.590692	0.759354	0.170917	0.180194	0.266359	0.009952	0.022536
-0.24599	0.048609	0.084951	0.31046	-0.21499	0.112928	-0.11946	0.184451	-0.03168
0.005308	0.587334	0.342314	0.000382	0.01521	0.206193	0.180998	0.037899	0.723647
-0.39304	-0.06569	-0.13498	-0.00182	0.015558	-0.0742	-0.23805	-0.09654	-0.15616
4.86E-06	0.463079	0.130266	0.983788	0.862172	0.407094	0.007038	0.280284	0.079569
0.091173	-0.0545	-0.03909	-0.00074	-0.28219	-0.01971	0.084232	0.176389	0.36011
0.307997	0.542839	0.662609	0.993391	0.001308	0.825937	0.346432	0.047285	3.2E-05
0.406636	-0.00032	0.047422	0.25867	-0.07216	0.126112	0.432846	0.528445	0.686671
2.1E-06	0.997182	0.596508	0.003321	0.420121	0.157714	3.73E-07	1.71E-10	5.03E-19
0.622373	0.709806	0.766737	0.820238	-0.48234	0.835188	0.663668	0.826705	0.882645
5.68E-15	9.45E-21	8.02E-26	4.11E-32	9.35E-09	2.96E-34	1.85E-17	5.15E-33	8.37E-43
0.297824	0.321025	0.345398	0.696906	-0.32892	0.457176	0.437135	0.739049	0.668456
0.000672	0.000233	6.98E-05	9.08E-20	0.00016	6.55E-08	2.77E-07	3.43E-23	8.95E-18
0.155503	0.189792	0.01197	0.31753	-0.07894	0.199555	0.070827	0.114282	-0.04097
0.080856	0.032585	0.893744	0.000275	0.377696	0.024493	0.428782	0.200771	0.647401
0.400922	0.170062	0.051615	0.091776	0.070536	0.187959	0.286052	0.12535	0.333337
3E-06	0.055941	0.564411	0.304797	0.430686	0.034332	0.001113	0.16026	0.000128
-0.19379	-0.20563	-0.33684	-0.54875	0.528294	-0.34163	-0.29408	-0.63011	-0.5048
0.029031	0.02038	0.000108	2.4E-11	1.73E-10	8.47E-05	0.000791	2.07E-15	1.43E-09
0.025402	0.051615	-0.14617	-0.11633	0.289937	0.02997	-0.08055	-0.26235	-0.25777
0.776804	0.564406	0.101047	0.192764	0.000945	0.738015	0.367975	0.002885	0.003435
0.325444	-0.27807	-0.02916	-0.2228	-0.28266	0.011789	0.135538	0.172776	0.194429
0.000189	0.001549	0.744846	0.011811	0.001282	0.89534	0.128673	0.052081	0.028496

DUSP14	DUSP15	DVL1	DYRK1A	E4F1	ECH1	ECI1	EDF1	EEF1D
black	black	black	black	black	black	black	black	black
0.165064	-0.14799	-0.18742	0.077072	-0.27049	-0.04331	-0.23748	0.091888	-0.18918
0.063663	0.096834	0.034858	0.389102	0.0021	0.628751	0.007179	0.304203	0.033163
0.207147	-0.11165	-0.16883	0.094494	-0.26948	0.019401	-0.17833	0.142402	-0.22154
0.019452	0.211412	0.057768	0.290633	0.002186	0.828604	0.04486	0.11025	0.01231
-0.08353	0.13795	-0.14509	0.065048	0.061219	-0.06252	0.079699	-0.32586	0.0651
0.350456	0.121946	0.103618	0.467488	0.494149	0.485005	0.373088	0.000185	0.467126
0.063357	-0.15437	-0.60371	0.414694	-0.38402	-0.53332	-0.20714	-0.68425	-0.2993
0.479162	0.083125	5.79E-14	1.25E-06	8.31E-06	1.08E-10	0.019454	7.46E-19	0.00063
0.331363	0.288014	-0.10009	-0.0167	-0.05643	-0.00733	0.534766	0.484834	0.056881
0.000142	0.001025	0.262854	0.85213	0.528605	0.934789	9.41E-11	7.64E-09	0.525301
0.322741	-0.08296	-0.68366	0.416267	-0.51284	-0.61372	0.018193	-0.33934	-0.38226
0.000215	0.353798	8.21E-19	1.13E-06	7.08E-10	1.7E-14	0.839128	9.51E-05	9.21E-06
0.348298	-0.14344	-0.5789	0.443869	-0.50041	-0.36401	-0.12805	-0.2052	-0.24007
6E-05	0.107656	1.01E-12	1.72E-07	2.09E-09	2.59E-05	0.151367	0.020649	0.006556
0.266722	-0.07557	-0.23355	0.153856	-0.24966	-0.38554	-0.0296	-0.09991	-0.27003
0.002436	0.398407	0.008226	0.084163	0.004645	7.6E-06	0.741108	0.263726	0.002138
-0.15651	-0.06953	-0.23111	-0.1041	0.001508	0.008417	-0.19306	-0.1925	0.267124
0.078889	0.437273	0.008942	0.244138	0.986574	0.925177	0.029653	0.030139	0.002398
-0.1054	0.066967	-0.24699	-0.21486	-0.09014	-0.0199	-0.06239	0.211579	0.223864
0.238279	0.454426	0.005119	0.015275	0.313515	0.824267	0.485888	0.016945	0.011404
-0.03046	-0.09342	-0.41856	-0.01465	-0.26937	-0.24899	-0.19111	-0.133	-0.02336
0.733921	0.296178	9.73E-07	0.870112	0.002195	0.004761	0.031376	0.136041	0.794295
-0.14656	0.034929	0.06959	-0.21168	0.132415	0.598162	-0.08451	0.05018	0.542913
0.100141	0.696644	0.436907	0.016893	0.137801	1.12E-13	0.344823	0.575301	4.28E-11
-0.45689	0.215953	0.419327	-0.3729	0.499258	0.555723	-0.01827	0.028406	0.525501
6.69E-08	0.01475	9.25E-07	1.58E-05	2.31E-09	1.19E-11	0.838468	0.751228	2.25E-10
-0.41576	0.647669	0.836467	-0.74899	0.896308	0.803172	0.67742	0.724461	0.794775
1.17E-06	1.89E-16	1.9E-34	4.27E-24	5.66E-46	6.74E-30	2.23E-18	6.19E-22	6.93E-29
-0.52262	0.395006	0.457054	-0.6611	0.569969	0.621734	0.152489	0.454655	0.721154
2.93E-10	4.31E-06	6.61E-08	2.71E-17	2.67E-12	6.16E-15	0.08699	7.89E-08	1.16E-21
-0.15632	0.137073	0.3234	-0.25773	0.087432	0.269533	-0.02028	0.40151	0.026627
0.07925	0.124361	0.000208	0.00344	0.328354	0.002181	0.82098	2.89E-06	0.766345
-0.10214	0.126417	0.507834	-0.16999	0.327072	0.412119	0.119929	0.133318	0.130148
0.253178	0.156702	1.1E-09	0.056052	0.000175	1.48E-06	0.179267	0.135112	0.144729
0.310811	-0.44036	-0.17894	0.564271	-0.36401	-0.52452	-0.20907	-0.45885	-0.70084
0.000376	2.21E-07	0.044131	4.89E-12	2.59E-05	2.46E-10	0.01833	5.78E-08	4.61E-20
0.260788	-0.30412	0.243972	0.214248	-0.07387	-0.03217	-0.09631	0.04514	-0.35983
0.003063	0.000509	0.005707	0.015575	0.409176	0.719578	0.281445	0.61431	3.25E-05
-0.03267	0.066272	0.105345	0.07534	0.11585	0.247672	0.008827	0.013857	0.222523
0.715428	0.459131	0.23851	0.399875	0.194624	0.004994	0.921541	0.877118	0.011919

EFCAB14	EFCAB7	EHD1	EIF3F	EIF3G	EIF5	EIF5A	ELL	ELOF1
black	black	black	black	black	black	black	black	black
0.086999	-0.04069	-0.09821	0.02284	-0.10308	0.280848	-0.29604	-0.12188	-0.05403
0.330763	0.649676	0.271999	0.798817	0.248827	0.001382	0.000727	0.172241	0.546298
0.061817	0.020213	-0.10805	0.065924	-0.1459	0.3088	-0.24779	-0.1743	-0.0174
0.489932	0.821547	0.226588	0.461499	0.1017	0.000412	0.004974	0.050005	0.846055
-0.10753	0.220058	-0.01865	0.103394	-0.01966	-0.05846	0.381262	-0.01544	-0.00805
0.228874	0.01292	0.835106	0.247366	0.826386	0.513817	9.76E-06	0.863203	0.928409
0.259625	0.411013	-0.45767	-0.1157	-0.37368	0.303321	-0.09729	-0.21541	-0.46634
0.003202	1.59E-06	6.31E-08	0.195191	1.51E-05	0.000527	0.276525	0.01501	3.28E-08
-0.52043	0.363251	-0.16343	0.525287	0.150414	0.112154	0.09703	-0.34963	0.153981
3.58E-10	2.7E-05	0.066369	2.29E-10	0.091424	0.209341	0.277822	5.6E-05	0.083909
0.018216	0.574015	-0.59928	0.126031	-0.37365	0.421596	-0.23658	-0.44067	-0.4497
0.838922	1.73E-12	9.82E-14	0.157984	1.51E-05	7.96E-07	0.00741	2.16E-07	1.13E-07
0.078784	0.349112	-0.46315	0.1382	-0.19748	0.271754	-0.12213	-0.26628	-0.19195
0.378619	5.75E-05	4.18E-08	0.121266	0.026052	0.001997	0.171353	0.002478	0.03062
0.326251	0.354564	-0.38492	-0.18581	-0.21019	0.144597	-0.51747	-0.3062	-0.19974
0.000182	4.32E-05	7.88E-06	0.036484	0.017699	0.104813	4.68E-10	0.000463	0.024361
0.161303	-0.35124	0.034049	0.182125	0.170548	-0.17525	0.323539	0.437457	-0.06343
0.070031	5.15E-05	0.703928	0.04043	0.055233	0.048752	0.000207	2.71E-07	0.478667
0.242873	-0.38463	0.044862	0.285444	0.238335	0.02323	-0.07549	0.238995	-0.00549
0.005936	8.02E-06	0.616495	0.001142	0.006969	0.795457	0.398946	0.006809	0.95111
0.398077	-0.17436	-0.16812	0.017864	0.004445	0.24357	-0.24085	0.056772	-0.21711
3.57E-06	0.049933	0.058849	0.841997	0.960442	0.00579	0.006379	0.526095	0.014215
0.08499	-0.48305	0.293703	0.098288	0.300547	-0.16799	0.390283	0.411821	0.085851
0.342092	8.83E-09	0.000804	0.271612	0.000596	0.059039	5.73E-06	1.51E-06	0.337205
0.106777	-0.49753	0.544342	0.061408	0.435789	-0.43736	0.491063	0.528048	0.410091
0.232153	2.67E-09	3.72E-11	0.492814	3.04E-07	2.73E-07	4.58E-09	1.77E-10	1.68E-06
-0.38175	-0.52136	0.727506	0.355852	0.861968	-0.56061	0.66424	0.540742	0.772659
9.49E-06	3.29E-10	3.44E-22	4.03E-05	1.09E-38	7.17E-12	1.69E-17	5.29E-11	1.97E-26
0.158176	-0.79019	0.660585	0.296077	0.69005	-0.4671	0.384003	0.647915	0.499366
0.075712	2.36E-28	2.92E-17	0.000726	2.88E-19	3.1E-08	8.32E-06	1.82E-16	2.28E-09
0.11785	-0.20929	0.311865	-0.07186	0.065676	0.024228	-0.24588	-0.1844	0.181641
0.186979	0.018201	0.000358	0.422053	0.463193	0.786868	0.005328	0.037954	0.040975
-0.22794	-0.0191	0.386431	-0.15555	0.120515	-0.19311	0.286937	-0.02311	0.31801
0.009955	0.831221	7.21E-06	0.080765	0.177133	0.029611	0.001073	0.796463	0.000269
-0.09823	0.591231	-0.47678	-0.56224	-0.65644	0.365099	-0.40362	-0.49812	-0.46145
0.271905	2.51E-13	1.46E-08	6.05E-12	5.37E-17	2.44E-05	2.53E-06	2.54E-09	4.76E-08
-0.23891	0.226344	-0.08956	-0.37802	-0.28333	0.075668	-0.20909	-0.24959	-0.02863
0.006829	0.010502	0.316687	1.18E-05	0.001247	0.397819	0.018319	0.004657	0.74932
-0.46802	0.096946	0.25483	0.3124	0.043631	-0.26847	0.404601	-0.04302	0.165475
2.89E-08	0.278242	0.003837	0.000349	0.626209	0.002275	2.38E-06	0.631058	0.062998

EMC10	ENKD1	ERCC6	ESRRA	ETV2	EVA1B	EXD3	EXOC1	EXOSC4
black	black	black	black	black	black	black	black	black
-0.3813	-0.26757	0.04654	-0.16781	-0.22775	-0.29901	-0.11893	0.263802	-0.21313
9.74E-06	0.002356	0.603358	0.059312	0.01002	0.000638	0.182943	0.002728	0.016138
-0.37207	-0.28708	0.042326	-0.13446	-0.1352	-0.35784	-0.12338	0.337104	-0.17644
1.65E-05	0.001066	0.636582	0.131773	0.129651	3.62E-05	0.166993	0.000106	0.047219
0.134553	0.066328	-0.01009	-0.11917	0.229942	0.109062	-0.09351	-0.00268	0.007359
0.131501	0.458749	0.910367	0.182065	0.009305	0.222252	0.295721	0.976166	0.934555
-0.28472	-0.10192	0.177824	-0.47907	-0.06455	-0.13291	-0.50476	0.401895	-0.41668
0.001177	0.25421	0.045486	1.21E-08	0.470923	0.136308	1.44E-09	2.82E-06	1.1E-06
-0.15041	0.256337	-0.10478	0.163206	0.441524	0.090513	-0.06074	0.29458	0.348658
0.091432	0.003626	0.241032	0.066748	2.04E-07	0.311526	0.497512	0.000774	5.89E-05
-0.48574	-0.03288	0.155812	-0.39332	0.054205	-0.13334	-0.59031	0.629999	-0.27896
7.1E-09	0.713625	0.080248	4.77E-06	0.545	0.135044	2.79E-13	2.1E-15	0.001493
-0.39545	-0.07889	0.079865	-0.37334	0.053667	-0.29631	-0.44549	0.403912	-0.29959
4.2E-06	0.377962	0.372092	1.54E-05	0.549004	0.000718	1.54E-07	2.49E-06	0.000622
-0.27952	0.378126	0.073688	-0.24764	-0.1684	-0.1627	-0.20344	0.30862	-0.27965
0.00146	1.17E-05	0.410319	0.005	0.058426	0.067614	0.021791	0.000415	0.001452
0.231776	0.096845	-0.28102	-0.0057	-0.02746	0.435674	-0.25243	-0.15794	-0.0244
0.008743	0.278744	0.001373	0.949301	0.75929	3.07E-07	0.004194	0.076157	0.785387
0.048112	0.27115	-0.21715	0.096784	-0.13525	0.337603	-0.10966	0.117407	0.007376
0.591165	0.002046	0.014196	0.27905	0.129487	0.000104	0.219696	0.188653	0.93441
-0.17676	0.045966	-0.1091	-0.19908	-0.28321	0.08582	-0.31059	0.219936	-0.2249
0.046809	0.607836	0.22208	0.02484	0.001254	0.337382	0.00038	0.012972	0.01102
0.407755	-0.09671	-0.25904	0.09403	0.037014	0.015977	0.089207	-0.23006	0.160112
1.95E-06	0.279406	0.003274	0.293023	0.679498	0.858502	0.318589	0.009267	0.072153
0.580257	0.230162	-0.22037	0.261019	0.094301	0.273784	0.464508	-0.56142	0.208771
8.7E-13	0.009236	0.012791	0.003037	0.291625	0.001842	3.77E-08	6.6E-12	0.018498
0.793198	0.617373	-0.52224	0.840029	0.498365	0.739524	0.731272	-0.67573	0.852256
1.06E-28	1.07E-14	3.03E-10	5.38E-35	2.49E-09	3.11E-23	1.64E-22	2.91E-18	5.58E-37
0.665467	0.503158	-0.45434	0.548604	0.076502	0.629024	0.508906	-0.52561	0.417001
1.41E-17	1.65E-09	8.08E-08	2.44E-11	0.392625	2.39E-15	1E-09	2.22E-10	1.08E-06
0.067372	-0.11096	-0.01045	0.200382	-0.13494	-0.17267	0.337836	0.028339	0.061877
0.451693	0.214267	0.907188	0.023895	0.130384	0.052232	0.000103	0.7518	0.489512
0.332137	-0.14165	-0.01709	0.181733	0.129965	-0.13965	0.411583	-0.36322	0.217347
0.000136	0.112151	0.848758	0.040871	0.1453	0.117365	1.53E-06	2.7E-05	0.014105
-0.51756	-0.61251	0.47492	-0.41441	-0.18464	-0.56002	-0.31943	0.295951	-0.37204
4.64E-10	1.97E-14	1.69E-08	1.28E-06	0.037695	7.63E-12	0.000252	0.00073	1.66E-05
-0.22101	-0.43395	0.262875	-0.08809	-0.12787	-0.46867	0.078102	-0.06189	-0.08228
0.012523	3.45E-07	0.002828	0.324719	0.151957	2.74E-08	0.382775	0.489431	0.357739
0.072037	-0.09773	0.164917	0.077417	0.201421	-0.09568	0.232181	-0.12346	0.163772
0.420915	0.274336	0.063904	0.386977	0.02316	0.284585	0.008622	0.1667	0.065796

EXOSC5	F12	FAAP100	FAAP20	FAM110A	FAM110D	FAM122B	FAM173A	FAM184A
black	black	black	black	black	black	black	black	black
-0.09294	-0.22976	-0.36311	-0.31239	-0.28702	-0.22913	0.042444	-0.18464	0.0773
0.298668	0.009363	2.72E-05	0.000349	0.001069	0.009564	0.635641	0.0377	0.387697
-0.00753	-0.18168	-0.41745	-0.34512	-0.37199	-0.2898	0.071271	-0.19365	0.135297
0.93304	0.040934	1.05E-06	7.08E-05	1.66E-05	0.00095	0.425884	0.029147	0.129362
-0.08488	0.220359	0.347235	0.168906	0.370832	-0.03003	0.092567	-0.1947	-0.04216
0.342736	0.012794	6.34E-05	0.057654	1.77E-05	0.737527	0.300626	0.028272	0.637884
-0.4824	-0.25916	-0.14085	-0.26709	-0.11052	-0.28916	0.377308	-0.60801	0.233042
9.3E-09	0.003259	0.114221	0.002401	0.216099	0.000977	1.23E-05	3.43E-14	0.008372
0.402419	-0.01994	-0.30807	0.04597	-0.21562	-0.22163	-0.0457	-0.04129	-0.02398
2.73E-06	0.823891	0.000426	0.607809	0.014909	0.012276	0.609933	0.644851	0.789032
-0.293	-0.43167	-0.51494	-0.37636	-0.40259	-0.45046	0.378283	-0.63458	0.246854
0.000829	4.04E-07	5.87E-10	1.3E-05	2.7E-06	1.07E-07	1.16E-05	1.14E-15	0.005145
-0.18108	-0.22369	-0.46098	-0.42777	-0.41465	-0.31073	0.204043	-0.51916	0.292895
0.041609	0.011471	4.93E-08	5.27E-07	1.26E-06	0.000377	0.021393	4.01E-10	0.000833
-0.33303	-0.46654	-0.38769	-0.21403	-0.43798	-0.24866	0.242006	-0.1912	0.288566
0.00013	3.23E-08	6.69E-06	0.015681	2.61E-07	0.004818	0.006122	0.031293	0.001002
-0.2953	0.022668	0.347618	0.172985	0.316247	0.138844	-0.20688	-0.14531	-0.4304
0.00075	0.800298	6.22E-05	0.051793	0.000292	0.119523	0.019613	0.103096	4.41E-07
-0.26938	-0.33982	-0.02572	0.042207	0.052343	0.090179	-0.19026	-0.03905	-0.37369
0.002194	9.28E-05	0.774096	0.637527	0.558918	0.313325	0.032149	0.662952	1.51E-05
-0.41514	-0.43473	-0.16099	-0.16134	-0.08393	-0.11132	-0.02096	-0.27262	-0.12104
1.22E-06	3.27E-07	0.070579	0.069968	0.348162	0.212751	0.815028	0.00193	0.175226
0.107295	0.267509	0.348993	0.178829	0.23459	0.08499	-0.42941	0.126687	-0.35611
0.229879	0.002362	5.79E-05	0.04426	0.007938	0.342092	4.71E-07	0.155811	3.97E-05
0.068253	0.438025	0.804631	0.513635	0.6786	0.401549	-0.36645	0.37066	-0.32754
0.44579	2.6E-07	4.45E-30	6.6E-10	1.85E-18	2.88E-06	2.26E-05	1.79E-05	0.000171
0.673097	0.589382	0.828253	0.917278	0.684714	0.48323	-0.64667	0.86983	-0.5773
4.38E-18	3.11E-13	3.1E-33	8.1E-52	6.92E-19	8.7E-09	2.17E-16	3.6E-40	1.21E-12
0.088393	0.19334	0.727781	0.635718	0.629293	0.484943	-0.57441	0.568611	-0.62122
0.32304	0.029415	3.26E-22	9.8E-16	2.31E-15	7.57E-09	1.65E-12	3.09E-12	6.59E-15
0.149822	-0.06344	-0.1226	-0.06034	-0.12733	0.171869	-0.01892	0.351703	0.077225
0.092721	0.478584	0.169686	0.50038	0.153713	0.053346	0.832766	5.02E-05	0.388156
0.371536	0.550002	0.311416	0.20628	0.243212	0.251495	-0.12076	0.401258	0.086813
1.7E-05	2.12E-11	0.000365	0.019978	0.005865	0.004342	0.176254	2.94E-06	0.331799
-0.04139	-0.0649	-0.55474	-0.53704	-0.50017	-0.30338	0.573331	-0.29537	0.57794
0.644043	0.468474	1.31E-11	7.57E-11	2.13E-09	0.000526	1.86E-12	0.000748	1.12E-12
0.273285	0.183554	-0.33535	-0.28466	-0.34059	-0.05279	0.174379	0.117733	0.348178
0.001879	0.038859	0.000116	0.00118	8.93E-05	0.555566	0.049906	0.187418	6.04E-05
0.231396	0.410596	0.256669	0.173908	0.406552	0.108771	0.019796	0.018423	-0.06906
0.008857	1.63E-06	0.003581	0.050537	2.11E-06	0.223495	0.825165	0.837119	0.440403

FAM207A	FAM214A	FAM229A	FAM3A	FAM83E	FAM8A1	FAM98B	FAM98C	FAR2
black	black	black	black	black	black	black	black	black
-0.30334	0.095415	-0.31009	0.060721	-0.17454	0.034043	-0.04305	-0.05548	0.170096
0.000527	0.285938	0.000388	0.497678	0.049697	0.703971	0.630817	0.535545	0.055891
-0.31689	0.10751	-0.32576	0.120605	-0.18573	0.098009	-0.01908	0.044854	0.129609
0.000284	0.228943	0.000186	0.17681	0.036563	0.272981	0.831403	0.616562	0.146414
0.109592	0.187138	0.076612	-0.30926	0.303573	0.006799	0.269584	-0.157	-0.12451
0.220001	0.035141	0.391946	0.000403	0.000521	0.939526	0.002177	0.077932	0.163093
-0.35134	0.515561	-0.32311	-0.5222	-0.03842	0.380483	0.388684	-0.44476	0.208543
5.12E-05	5.55E-10	0.000211	3.04E-10	0.667997	1.02E-05	6.31E-06	1.62E-07	0.01863
0.121999	0.004363	-0.05485	0.462329	0.037577	0.024865	-0.23556	0.34004	-0.04772
0.17182	0.961175	0.540212	4.45E-08	0.674897	0.781404	0.007677	9.18E-05	0.594222
-0.38956	0.497403	-0.45317	-0.19174	-0.19774	0.453463	0.214804	-0.26802	0.293052
5.99E-06	2.7E-09	8.8E-08	0.030813	0.025848	8.61E-08	0.015301	0.002315	0.000827
-0.39547	0.27218	-0.51076	-0.18181	-0.13864	0.215371	0.112471	-0.13875	0.181443
4.19E-06	0.001964	8.51E-10	0.040781	0.120064	0.015027	0.208047	0.119779	0.0412
-0.37037	0.102695	-0.1609	0.004085	-0.14755	0.346706	-0.12137	-0.21424	0.050435
1.82E-05	0.250593	0.070743	0.963647	0.097825	6.52E-05	0.174073	0.015577	0.573357
0.022993	0.024348	0.031511	-0.2196	0.058814	-0.29495	-0.06432	-0.37218	0.001342
0.7975	0.785839	0.725073	0.013112	0.511298	0.000762	0.472484	1.64E-05	0.988057
-0.09815	0.065444	-0.05096	0.204856	-0.13787	-0.29913	-0.36097	-0.27453	0.011989
0.27227	0.464776	0.569368	0.020869	0.122153	0.000635	3.06E-05	0.001787	0.893579
-0.29261	0.115212	-0.19193	-0.04078	-0.19969	0.024021	-0.06686	-0.37547	0.21534
0.000843	0.197106	0.030638	0.648959	0.024393	0.788652	0.455172	1.36E-05	0.015042
0.15598	-0.22213	0.122292	-0.06383	0.196713	-0.32799	-0.05394	0.004143	-0.16962
0.079918	0.012073	0.170785	0.4759	0.026648	0.000167	0.54699	0.963133	0.056586
0.394766	-0.22922	0.472135	-0.04761	0.438939	-0.51719	-0.08674	0.025244	-0.44891
4.37E-06	0.009537	2.1E-08	0.59507	2.44E-07	4.8E-10	0.332194	0.778156	1.2E-07
0.85757	-0.56951	0.871876	0.732064	0.499353	-0.67635	-0.54159	0.607211	-0.54206
6.72E-38	2.81E-12	1.43E-40	1.4E-22	2.29E-09	2.64E-18	4.87E-11	3.79E-14	4.65E-11
0.452397	-0.33725	0.569649	0.410935	0.286826	-0.72543	-0.50368	0.049157	-0.45153
9.31E-08	0.000106	2.77E-12	1.59E-06	0.001078	5.14E-22	1.58E-09	0.583123	9.92E-08
-0.01205	-0.15014	0.058998	0.344941	-0.12908	-0.09358	-0.1715	0.172842	-0.06188
0.893056	0.092022	0.509979	7.14E-05	0.148075	0.295356	0.05387	0.051989	0.489482
0.295025	-0.348	0.283486	0.011794	0.275428	-0.11589	0.081156	0.316832	-0.18726
0.000759	6.1E-05	0.001239	0.895299	0.001724	0.194462	0.364389	0.000284	0.035021
-0.32412	0.214056	-0.37662	-0.41442	-0.28797	0.691957	0.519083	0.006409	0.477201
0.000201	0.01567	1.28E-05	1.27E-06	0.001027	2.1E-19	4.04E-10	0.942994	1.41E-08
-0.07062	-0.21798	-0.14019	-0.07032	-0.17677	0.272415	0.170574	0.282649	0.197985
0.430112	0.013821	0.115937	0.432076	0.046804	0.001945	0.055195	0.001283	0.025664
0.239095	0.158971	0.045214	-0.18444	0.379064	-0.31704	0.117143	0.047697	-0.35128
0.006785	0.074233	0.613732	0.037914	1.11E-05	0.000282	0.189655	0.594377	5.13E-05

FASTK	FASTKD5	FBRS	FBXL8	FBXO46	FBXW2	FBXW5	FBXW9	FDXR
black	black	black	black	black	black	black	black	black
-0.24224	0.159004	-0.12878	-0.19692	-0.37629	-0.07643	-0.11576	-0.34964	-0.01843
0.006071	0.074172	0.149047	0.026485	1.3E-05	0.39306	0.194955	5.6E-05	0.837057
-0.22518	0.193056	-0.20696	-0.25654	-0.36516	-0.10862	-0.12901	-0.35351	-0.03059
0.010919	0.029659	0.019564	0.003599	2.43E-05	0.224154	0.148309	4.56E-05	0.732818
0.122374	0.211776	-0.08117	0.177383	0.317391	0.419782	-0.25689	0.281758	-0.15906
0.170496	0.01684	0.364294	0.046033	0.000277	8.98E-07	0.003551	0.001331	0.07407
-0.1817	0.429637	-0.42844	-0.02265	-0.15273	0.567902	-0.65281	0.014797	-0.37595
0.040913	4.64E-07	5.03E-07	0.800452	0.086477	3.33E-12	9.07E-17	0.868852	1.33E-05
0.238336	0.013814	-0.40895	0.005847	-0.2727	-0.44067	-0.08761	0.097747	0.083828
0.006968	0.877494	1.81E-06	0.94798	0.001924	2.16E-07	0.32737	0.274272	0.348759
-0.15964	0.392868	-0.70257	-0.1183	-0.51382	0.174853	-0.66116	-0.09042	-0.31197
0.073001	4.91E-06	3.41E-20	0.185287	6.49E-10	0.049278	2.68E-17	0.312035	0.000356
-0.14664	0.292437	-0.46146	-0.18382	-0.46395	0.19429	-0.53048	-0.08308	-0.21033
0.099938	0.000849	4.75E-08	0.038567	3.94E-08	0.028612	1.41E-10	0.353102	0.01762
-0.05712	-0.14617	-0.16232	-0.17353	-0.36945	0.061539	-0.19193	0.115779	-0.07305
0.52359	0.101055	0.068256	0.051041	1.92E-05	0.49189	0.030641	0.194896	0.414412
0.151494	0.181247	0.221712	0.398834	0.224945	0.41257	0.039765	0.223394	0.099664
0.089094	0.041423	0.012241	3.41E-06	0.011003	1.44E-06	0.657136	0.011582	0.264926
0.130098	-0.02995	0.167046	0.233741	-0.16364	0.043854	0.125767	-0.02295	0.272714
0.144885	0.73818	0.060503	0.008173	0.066019	0.624448	0.158863	0.797887	0.001922
-0.03655	0.111161	-0.06755	-0.04834	-0.30589	0.192689	-0.18299	-0.09965	0.033539
0.68328	0.213429	0.45048	0.589428	0.00047	0.029976	0.039469	0.264992	0.708156
0.061241	0.167794	0.285272	0.020017	0.370658	0.224534	0.179589	0.205242	0.064216
0.493997	0.059343	0.00115	0.823242	1.79E-05	0.011154	0.043352	0.020624	0.473209
0.306991	-0.14041	0.711933	0.364821	0.775078	0.30265	0.405581	0.350238	0.269166
0.000447	0.115367	6.43E-21	2.48E-05	1.09E-26	0.000543	2.24E-06	5.42E-05	0.002213
0.771511	-0.52011	0.776094	0.615366	0.795816	-0.47088	0.873872	0.576643	0.698896
2.59E-26	3.68E-10	8.54E-27	1.38E-14	5.22E-29	2.31E-08	5.72E-41	1.3E-12	6.46E-20
0.496863	-0.36264	0.85994	0.551316	0.599025	-0.02432	0.70981	0.306907	0.621762
2.82E-09	2.79E-05	2.54E-38	1.86E-11	1.01E-13	0.786043	9.44E-21	0.000449	6.14E-15
-0.10986	-0.36172	0.175266	-0.16492	-0.10748	-0.30955	0.295184	-0.39387	0.207533
0.218889	2.94E-05	0.048735	0.063899	0.229055	0.000398	0.000754	4.62E-06	0.019222
0.018989	-0.23868	0.225105	-0.13966	0.366701	-0.1386	0.242825	0.041492	-0.02338
0.832186	0.006884	0.010945	0.117353	2.23E-05	0.120172	0.005946	0.643244	0.794149
-0.5155	0.190646	-0.62629	-0.5057	-0.45067	-0.04434	-0.46239	-0.35567	-0.54687
5.59E-10	0.031796	3.42E-15	1.33E-09	1.06E-07	0.620571	4.43E-08	4.07E-05	2.9E-11
-0.31515	-0.0995	-0.20455	-0.46384	-0.22853	-0.33506	0.018416	-0.26907	-0.26511
0.000308	0.265734	0.021067	3.97E-08	0.00976	0.000118	0.837183	0.002221	0.002594
-0.06821	0.172763	0.040607	0.162538	0.347319	0.199127	-0.06072	0.01179	-0.08743
0.44607	0.052098	0.65035	0.067886	6.32E-05	0.024807	0.497695	0.895333	0.328375

FIBP	FIG4	FIZ1	FKBP2	FKTN	FLYWCH2	FOXJ3	FPGS	FSCN1
black	black	black	black	black	black	black	black	black
-0.08532	0.318752	-0.25353	0.036082	-0.22052	-0.23695	-0.02218	-0.1906	-0.26776
0.340193	0.00026	0.004027	0.687145	0.012728	0.007314	0.804532	0.031842	0.002339
-0.0143	0.389755	-0.24172	0.048904	-0.19696	-0.19355	-0.01063	-0.21408	-0.28557
0.873232	5.92E-06	0.006183	0.585067	0.026454	0.029233	0.905568	0.015658	0.001136
-0.02071	-0.36422	0.263518	-0.31779	0.556147	0.012823	0.392739	0.146079	0.237566
0.817232	2.56E-05	0.002759	0.000272	1.14E-11	0.886226	4.95E-06	0.101264	0.007159
-0.50522	-0.17403	-0.24173	-0.63751	0.711209	-0.3572	0.515432	-0.23097	-0.24478
1.38E-09	0.050377	0.006182	7.68E-16	7.33E-21	3.75E-05	5.62E-10	0.008986	0.005544
0.354919	0.086919	-0.14866	0.204341	-0.38028	0.426676	-0.40935	0.297286	-0.38632
4.23E-05	0.331206	0.095308	0.0212	1.03E-05	5.67E-07	1.76E-06	0.000688	7.26E-06
-0.34256	0.057493	-0.52582	-0.45305	0.342091	-0.16634	0.122695	-0.14848	-0.63241
8.07E-05	0.520847	2.18E-10	8.88E-08	8.27E-05	0.061614	0.169371	0.095714	1.53E-15
-0.14967	0.193133	-0.33482	-0.33225	0.18068	-0.18734	0.200399	-0.15192	-0.50896
0.093052	0.029593	0.000119	0.000135	0.042074	0.034944	0.023882	0.088194	9.98E-10
-0.38836	0.02418	-0.45041	-0.09179	0.165011	-0.14324	-0.07361	-0.052	-0.52634
6.43E-06	0.787281	1.08E-07	0.304703	0.063749	0.108138	0.410831	0.561517	2.08E-10
-0.13713	-0.33457	0.013485	-0.08217	0.213642	-0.12569	0.238476	0.132019	0.08978
0.124191	0.000121	0.880389	0.358399	0.015877	0.159118	0.006934	0.138993	0.315477
-0.14	-0.11655	-0.25324	0.243092	-0.22576	-0.08552	-0.30428	0.261423	-0.26064
0.116439	0.191922	0.00407	0.00589	0.01071	0.339067	0.000505	0.00299	0.003081
-0.33151	-0.06478	-0.34428	-0.09383	0.061302	-0.31558	-0.10396	0.007779	-0.3011
0.000141	0.469308	7.39E-05	0.294036	0.493561	0.000301	0.244782	0.93083	0.000582
0.209073	-0.09435	0.295846	-0.02364	-0.05035	-0.07942	0.175268	-0.06085	0.482642
0.018326	0.291352	0.000733	0.791899	0.573987	0.374766	0.048732	0.496774	9.12E-09
0.167748	-0.44301	0.702846	0.194777	0.123165	0.088628	0.28048	0.282419	0.723364
0.059415	1.83E-07	3.25E-20	0.028208	0.16773	0.321751	0.001403	0.001295	7.64E-22
0.630998	-0.5483	0.742476	0.810853	-0.57716	0.753364	-0.44273	0.762878	0.704204
1.84E-15	2.51E-11	1.69E-23	7.24E-31	1.22E-12	1.65E-24	1.87E-07	1.96E-25	2.56E-20
0.203289	-0.46921	0.489631	0.626581	-0.34429	0.240321	-0.24882	0.549514	0.520096
0.021889	2.63E-08	5.16E-09	3.29E-15	7.39E-05	0.006498	0.00479	2.23E-11	3.69E-10
0.105263	0.27868	0.103608	0.375506	-0.36354	-0.02575	-0.3707	-0.07105	0.160254
0.238875	0.001511	0.246382	1.36E-05	2.66E-05	0.773815	1.79E-05	0.427316	0.071898
0.380526	0.035928	0.52435	0.094586	-0.06039	0.174634	0.012192	0.024032	0.613817
1.02E-05	0.68841	2.5E-10	0.290162	0.499993	0.049566	0.891785	0.788558	1.68E-14
-0.20496	0.429198	-0.37671	-0.51262	0.287999	-0.23513	0.197363	-0.58897	-0.25586
0.020802	4.78E-07	1.27E-05	7.22E-10	0.001026	0.00779	0.026141	3.26E-13	0.003691
0.20097	0.501566	-0.07792	-0.06285	-0.18966	-0.00287	-0.08044	-0.35252	-0.01077
0.023477	1.89E-09	0.38388	0.482717	0.032713	0.974466	0.368634	4.81E-05	0.904363
0.307656	-0.08357	0.421513	-0.06745	0.099111	0.126611	0.383141	0.080323	0.318885
0.000434	0.350227	8.01E-07	0.451198	0.267599	0.156062	8.75E-06	0.369347	0.000258

FUK	FUZ	FZR1	G6PC3	GADD45G	GADD45G	GAK	GALE	GALK1
black	black	black	black	black	black	black	black	black
-0.16299	-0.20415	-0.25342	-0.16235	-0.10806	-0.18836	-0.08367	-0.15805	-0.27928
0.067108	0.021325	0.004044	0.06821	0.226567	0.03394	0.349648	0.075946	0.001474
-0.15722	-0.19211	-0.2683	-0.13113	-0.0828	-0.15847	-0.13611	-0.06596	-0.28742
0.077522	0.030485	0.00229	0.141697	0.354698	0.075154	0.127047	0.461238	0.001051
-0.09371	-0.07661	0.143784	0.020893	-0.05971	-0.1	-0.22846	0.312166	0.196872
0.294664	0.391974	0.106802	0.815644	0.504871	0.263287	0.009782	0.000353	0.026524
-0.36878	-0.42507	-0.39783	-0.18178	-0.52354	-0.47587	-0.58376	-0.02275	-0.00987
1.99E-05	6.32E-07	3.63E-06	0.040823	2.69E-10	1.56E-08	5.89E-13	0.799613	0.912271
-0.01427	-0.03346	-0.35959	0.060715	-0.00274	0.332725	-0.49331	0.518328	0.246056
0.873468	0.708822	3.3E-05	0.497718	0.975584	0.000132	3.8E-09	4.33E-10	0.005295
-0.42722	-0.50649	-0.74612	-0.18112	-0.562	-0.31063	-0.87173	0.135823	0.034115
5.47E-07	1.24E-09	7.87E-24	0.041565	6.21E-12	0.000379	1.53E-40	0.127864	0.703374
-0.26138	-0.39054	-0.49334	-0.03924	-0.54413	-0.37045	-0.53977	0.116974	-0.14018
0.002995	5.65E-06	3.8E-09	0.661355	3.8E-11	1.81E-05	5.81E-11	0.190298	0.115967
0.062429	0.032938	-0.46603	0.026143	-0.30701	-0.17718	-0.2998	-0.29658	-0.17561
0.485636	0.713151	3.36E-08	0.770473	0.000447	0.046291	0.000616	0.00071	0.048286
-0.05547	-0.19181	0.075427	0.280414	-0.23804	-0.13817	0.110842	0.004802	0.312571
0.535626	0.030746	0.399332	0.001407	0.00704	0.121337	0.214754	0.957273	0.000347
0.157568	-0.07973	-0.24247	0.295085	-0.17419	0.009273	0.069694	-0.12451	0.29283
0.076858	0.372912	0.006021	0.000757	0.050162	0.917589	0.436219	0.163111	0.000835
-0.06949	-0.25497	-0.35061	0.070106	-0.32775	-0.22003	-0.16122	-0.20739	0.099523
0.437548	0.003817	5.32E-05	0.433508	0.000169	0.012934	0.070176	0.019309	0.265604
0.113791	0.11546	0.428452	0.228786	0.258316	-0.00167	0.509253	-0.03385	0.061466
0.202727	0.196138	5.03E-07	0.009676	0.003365	0.9851	9.72E-10	0.705601	0.492403
0.451982	0.431799	0.771403	0.283385	0.358154	0.137535	0.693809	0.030179	0.221238
9.6E-08	4E-07	2.66E-26	0.001245	3.56E-05	0.123086	1.54E-19	0.736258	0.012432
0.772227	0.798077	0.818038	0.560009	0.646086	0.845729	0.700208	0.446516	0.692473
2.18E-26	2.81E-29	8.17E-32	7.64E-12	2.36E-16	6.72E-36	5.15E-20	1.43E-07	1.92E-19
0.682003	0.512299	0.588983	0.558144	0.34524	0.37652	0.779352	-0.01372	0.5083
1.07E-18	7.43E-10	3.25E-13	9.26E-12	7.03E-05	1.28E-05	3.81E-27	0.878316	1.06E-09
0.255853	0.228094	0.069469	0.033675	0.22033	0.101889	0.34157	-0.22025	-0.25203
0.003693	0.009904	0.437705	0.707029	0.012806	0.254351	8.49E-05	0.012839	0.004258
0.152973	0.330422	0.564647	-0.04042	0.518997	0.219086	0.392812	0.109713	-0.15921
0.085979	0.000148	4.7E-12	0.651872	4.08E-10	0.013335	4.92E-06	0.21949	0.073797
-0.61161	-0.36805	-0.32553	-0.58575	-0.17533	-0.28994	-0.46402	-0.14225	-0.55473
2.2E-14	2.07E-05	0.000188	4.7E-13	0.048651	0.000945	3.92E-08	0.110634	1.31E-11
-0.24028	0.000124	0.060197	-0.3499	0.229178	0.030291	0.062771	-0.0956	-0.52346
0.006509	0.998893	0.501402	5.52E-05	0.009549	0.735317	0.483249	0.285017	2.71E-10
-0.06594	-0.0718	0.271032	-0.23013	0.165425	-0.04685	0.058526	0.32694	-0.06144
0.461368	0.422453	0.002056	0.009246	0.063078	0.600972	0.513376	0.000176	0.49257

GAMT	GAPVD1	GAS2L1	GCDH	GFER	GGA1	GGN	GIPC1	GLI4
black	black	black	black	black	black	black	black	black
-0.01749	-0.16958	-0.21384	-0.28895	-0.25595	0.065603	-0.3088	-0.13394	-0.24249
0.845257	0.056648	0.015778	0.000985	0.003679	0.463687	0.000412	0.133283	0.006018
0.045249	-0.17567	-0.21585	-0.22119	-0.18836	-0.01012	-0.36229	-0.11565	-0.25296
0.613455	0.048215	0.0148	0.012453	0.033939	0.910093	2.84E-05	0.195401	0.004113
-0.31393	0.375831	-0.13125	-0.01197	0.318318	-0.12922	0.267974	-0.18216	0.04731
0.000326	1.34E-05	0.141337	0.893789	0.000265	0.147638	0.002319	0.040388	0.597374
-0.62444	0.514758	-0.50552	-0.4068	-0.19371	-0.41979	0.018148	-0.6004	-0.36329
4.35E-15	5.97E-10	1.35E-09	2.07E-06	0.029104	8.97E-07	0.839515	8.59E-14	2.69E-05
0.163118	-0.53958	-0.29634	0.07542	0.214458	-0.10327	-0.23863	0.018085	-0.05796
0.066898	5.92E-11	0.000717	0.399374	0.015471	0.247919	0.006896	0.840064	0.517484
-0.46661	0.072496	-0.70599	-0.45441	-0.27247	-0.5298	-0.26051	-0.58712	-0.51355
3.21E-08	0.417954	1.87E-20	8.03E-08	0.001942	1.51E-10	0.003096	4.03E-13	6.65E-10
-0.37945	0.081806	-0.56758	-0.26464	-0.1671	-0.33883	-0.18804	-0.40988	-0.39174
1.08E-05	0.360548	3.45E-12	0.002641	0.060421	9.76E-05	0.034257	1.71E-06	5.25E-06
-0.16012	0.01254	-0.25341	-0.26811	-0.34283	-0.19396	-0.24967	-0.31423	-0.30737
0.072138	0.888717	0.004045	0.002307	7.96E-05	0.028891	0.004644	0.000321	0.000439
-0.32778	0.296803	-0.10142	-0.19923	-0.17765	0.029825	0.41726	-0.09996	-0.00135
0.000169	0.000703	0.256555	0.024731	0.0457	0.739241	1.06E-06	0.263496	0.98796
-0.06591	-0.09915	-0.18737	-0.15697	-0.34817	0.238777	0.006456	0.040048	-0.11901
0.461612	0.267416	0.034906	0.077994	6.04E-05	0.006862	0.942568	0.65485	0.182639
-0.20886	0.081638	-0.20228	-0.32207	-0.41557	0.015269	-0.11813	-0.24358	-0.26524
0.01845	0.361536	0.02257	0.000222	1.18E-06	0.864713	0.185924	0.005787	0.002581
0.096608	0.337183	0.404137	0.204191	0.302674	0.311712	0.270428	0.155034	0.308624
0.279927	0.000106	2.45E-06	0.021297	0.000543	0.000361	0.002105	0.081788	0.000415
0.099006	0.317551	0.52193	0.381244	0.423849	0.535687	0.548052	0.356195	0.540679
0.268107	0.000275	3.12E-10	9.77E-06	6.85E-07	8.61E-11	2.58E-11	3.96E-05	5.32E-11
0.719209	-0.428	0.767788	0.752553	0.662223	0.709333	0.584876	0.879036	0.869935
1.68E-21	5.19E-07	6.27E-26	1.98E-24	2.29E-17	1.03E-20	5.19E-13	4.95E-42	3.44E-40
0.351791	-0.05795	0.522239	0.397862	0.197011	0.740965	0.501371	0.601158	0.563159
5E-05	0.517516	3.03E-10	3.62E-06	0.026415	2.31E-23	1.92E-09	7.85E-14	5.5E-12
0.488658	-0.20326	0.187268	0.200051	0.008992	0.298645	-0.26473	0.39949	0.120548
5.59E-09	0.02191	0.035011	0.024132	0.920077	0.000649	0.002632	3.28E-06	0.177017
0.335745	0.022066	0.472818	0.43113	0.481116	0.192582	0.061478	0.377809	0.339897
0.000114	0.805497	1.99E-08	4.19E-07	1.03E-08	0.030069	0.492324	1.19E-05	9.24E-05
-0.17727	0.079081	-0.23572	-0.26063	-0.21563	-0.63159	-0.40734	-0.38008	-0.39366
0.046172	0.376817	0.007634	0.003082	0.014904	1.7E-15	2.01E-06	1.05E-05	4.68E-06
0.2127	-0.20614	0.154019	0.057266	-0.00227	-0.21616	-0.34859	0.042051	-0.09047
0.016357	0.020067	0.083832	0.522501	0.979749	0.014651	5.91E-05	0.638774	0.311757
-0.1798	0.155804	-0.1073	0.078275	0.46779	0.183743	0.169122	0.013768	0.218645
0.043107	0.080263	0.229837	0.381717	2.94E-08	0.038655	0.05733	0.877897	0.013526

GMEB2	GNA11	GNB1L	GNB2	GNPTG	GPAA1	GPATCH1	GPATCH3	GPBP1
black	black	black	black	black	black	black	black	black
-0.3438	-0.30089	-0.27946	-0.42135	0.139192	-0.11736	-0.20374	-0.02248	-0.04547
7.57E-05	0.000588	0.001463	8.09E-07	0.118593	0.188848	0.021593	0.801939	0.61176
-0.33425	-0.3197	-0.25108	-0.39971	0.14834	-0.14427	-0.13564	-0.02458	-0.03618
0.000123	0.000248	0.004409	3.23E-06	0.096032	0.105602	0.128378	0.783854	0.68631
0.188244	0.077915	0.275338	0.37898	-0.57823	-0.2159	0.251101	-0.12304	0.503819
0.034055	0.383916	0.00173	1.11E-05	1.09E-12	0.014775	0.004406	0.16818	1.56E-09
-0.13275	-0.42058	-0.22429	-0.02653	-0.6554	-0.59721	0.473584	-0.46147	0.63602
0.136788	8.52E-07	0.011245	0.767135	6.24E-17	1.25E-13	1.87E-08	4.75E-08	9.41E-16
-0.2854	-0.44628	0.003586	0.088877	0.32318	-0.09543	0.036181	0.161715	0.166338
0.001144	1.45E-07	0.968082	0.320389	0.000211	0.28584	0.686332	0.06931	0.061617
-0.42931	-0.78699	-0.4091	-0.15514	-0.29295	-0.62743	0.439955	-0.38916	0.569599
4.74E-07	5.46E-28	1.79E-06	0.08158	0.000831	2.94E-15	2.27E-07	6.13E-06	2.78E-12
-0.25279	-0.57683	-0.35342	-0.16921	-0.19146	-0.48327	0.253753	-0.28199	0.332316
0.004139	1.27E-12	4.59E-05	0.057197	0.031063	8.67E-09	0.003994	0.001318	0.000135
-0.3029	-0.41223	-0.55316	-0.16365	0.041047	-0.14696	0.209265	-0.30181	0.171557
0.000537	1.47E-06	1.54E-11	0.066005	0.646813	0.099208	0.018217	0.000564	0.053785
0.205942	0.10176	0.002215	0.337458	-0.20637	0.123914	-0.17947	-0.22667	-0.13327
0.020187	0.254955	0.980282	0.000105	0.019922	0.16514	0.043493	0.010387	0.135247
-0.13092	-0.25757	-0.34378	0.069072	0.419112	0.225009	-0.23901	-0.04298	-0.27273
0.142338	0.003461	7.58E-05	0.440332	9.38E-07	0.010979	0.006806	0.63139	0.001921
-0.20284	-0.35018	-0.42048	-0.05139	0.094699	-0.08086	-0.09738	-0.19717	-0.02124
0.022187	5.44E-05	8.57E-07	0.566129	0.289587	0.366173	0.276096	0.026295	0.812629
0.274454	0.417446	0.376705	0.162875	-0.04652	0.289287	-0.32817	0.094655	-0.31692
0.001793	1.05E-06	1.27E-05	0.067311	0.603481	0.000971	0.000165	0.289809	0.000283
0.609884	0.741554	0.510695	0.466597	-0.11845	0.46424	-0.28535	0.249215	-0.15973
2.73E-14	2.05E-23	8.56E-10	3.22E-08	0.184723	3.85E-08	0.001147	0.004721	0.072841
0.600186	0.79374	0.745384	0.732537	0.60887	0.889551	-0.47382	0.710011	-0.50753
8.81E-14	9.16E-29	9.2E-24	1.28E-22	3.09E-14	2.36E-44	1.84E-08	9.11E-21	1.13E-09
0.469968	0.582184	0.295572	0.503776	0.511463	0.798261	-0.54076	0.386383	-0.56208
2.48E-08	7.02E-13	0.000742	1.57E-09	8E-10	2.67E-29	5.28E-11	7.23E-06	6.16E-12
-0.21567	0.112526	-0.04502	-0.31306	0.47954	0.252455	-0.17429	0.289313	-0.29487
0.014884	0.207824	0.615238	0.000339	1.17E-08	0.004191	0.050026	0.00097	0.000765
0.220899	0.561901	0.486394	0.107762	-0.08065	0.169962	0.008402	0.261716	-0.03324
0.012571	6.27E-12	6.73E-09	0.227848	0.367384	0.056086	0.925305	0.002957	0.710675
-0.3431	-0.25752	-0.15558	-0.52579	-0.44947	-0.5987	0.445705	-0.26186	0.344215
7.85E-05	0.003468	0.080702	2.19E-10	1.15E-07	1.05E-13	1.51E-07	0.002941	7.42E-05
-0.13682	0.112084	0.021522	-0.39195	-0.01617	-0.11903	0.068836	0.043179	-0.11223
0.125052	0.209628	0.8102	5.19E-06	0.856841	0.182566	0.441903	0.629796	0.209044
0.093524	0.1619	0.4545	0.051914	-0.37963	-0.10073	-0.14346	0.126082	0.441651
0.29564	0.068987	7.98E-08	0.562149	1.07E-05	0.259823	0.107603	0.157814	2.02E-07

GPER1	GPR137	GPR180	GPS1	GPT	GPX4	GRHPR	GRSF1	GSG1
black	black	black	black	black	black	black	black	black
-0.35929	0.006834	0.012397	-0.20496	-0.1583	0.219691	0.085273	0.089214	-0.04816
3.35E-05	0.939219	0.889982	0.020801	0.075472	0.013075	0.340483	0.318553	0.590787
-0.33141	0.060778	0.044694	-0.14565	-0.12845	0.214693	0.138288	0.13838	-0.01272
0.000141	0.497271	0.617816	0.102283	0.150088	0.015356	0.121026	0.120777	0.887126
-0.08862	-0.30735	0.051415	-0.04792	-0.21346	-0.48095	-0.26436	-0.0277	0.076989
0.321819	0.00044	0.565921	0.592615	0.015971	1.05E-08	0.00267	0.757219	0.389615
-0.39138	-0.66947	0.333153	-0.57629	-0.40991	-0.71122	-0.58149	0.267079	0.208832
5.37E-06	7.67E-18	0.00013	1.35E-12	1.7E-06	7.32E-21	7.58E-13	0.002402	0.018463
-0.20452	-0.09829	0.035571	0.09508	-0.10627	0.355209	0.433613	0.182945	0.296285
0.021087	0.271583	0.691345	0.287639	0.234383	4.17E-05	3.53E-07	0.039522	0.000719
-0.54867	-0.66775	0.411272	-0.6018	-0.47004	-0.39797	-0.29015	0.44599	0.394792
2.42E-11	9.97E-18	1.56E-06	7.27E-14	2.47E-08	3.6E-06	0.000936	1.48E-07	4.37E-06
-0.19414	-0.40052	0.329543	-0.42881	-0.36957	-0.21729	-0.24706	0.315021	0.323544
0.028738	3.07E-06	0.000155	4.91E-07	1.9E-05	0.014133	0.005107	0.000309	0.000207
-0.34256	-0.1801	0.100368	-0.29605	0.094462	-0.05819	-0.06908	0.412146	0.218336
8.07E-05	0.042747	0.261548	0.000726	0.290801	0.515808	0.440299	1.48E-06	0.013662
0.174277	-0.16379	-0.06071	-0.25278	-0.38629	-0.1906	-0.40051	-0.33882	-0.0695
0.050042	0.065761	0.497736	0.00414	7.27E-06	0.031837	3.08E-06	9.76E-05	0.437526
-0.06422	-0.01082	-0.135	-0.23421	-0.14931	0.276685	0.0659	-0.11539	-0.09982
0.473171	0.903908	0.130217	0.008042	0.093864	0.001639	0.461659	0.196427	0.264177
-0.29204	-0.2115	-0.02094	-0.42533	-0.20555	-0.03754	-0.16641	0.034574	0.050149
0.000864	0.016986	0.815225	6.21E-07	0.020432	0.675207	0.061511	0.69958	0.575537
0.295345	0.195697	-0.46275	0.286295	0.023931	0.061192	-0.00555	-0.46772	-0.21086
0.000749	0.027457	4.31E-08	0.001102	0.789425	0.494337	0.950602	2.95E-08	0.017333
0.431705	0.361243	-0.45	0.426397	0.282076	0.033832	-0.01495	-0.506	-0.36416
4.03E-07	3.01E-05	1.11E-07	5.78E-07	0.001314	0.70572	0.867505	1.29E-09	2.57E-05
0.576869	0.762316	-0.59062	0.85343	0.572407	0.700205	0.588844	-0.6112	-0.36934
1.26E-12	2.23E-25	2.7E-13	3.52E-37	2.06E-12	5.15E-20	3.31E-13	2.32E-14	1.93E-05
0.474916	0.566053	-0.5688	0.432088	0.339448	0.50842	0.287949	-0.58182	-0.45618
1.69E-08	4.05E-12	3.03E-12	3.93E-07	9.46E-05	1.05E-09	0.001028	7.31E-13	7.05E-08
0.132856	0.382625	-0.0502	0.324146	0.363041	0.453296	0.534704	0.034159	-0.31295
0.136483	9.02E-06	0.575166	0.000201	2.73E-05	8.72E-08	9.46E-11	0.703008	0.000341
0.345916	0.311245	-0.16533	0.549121	0.361706	0.052996	0.306977	-0.14351	-0.20914
6.79E-05	0.000368	0.063239	2.31E-11	2.94E-05	0.554018	0.000447	0.107491	0.018287
-0.27825	-0.33984	0.557316	-0.26565	-0.15581	-0.46302	-0.28293	0.475407	0.234488
0.001538	9.27E-05	1.01E-11	0.00254	0.080257	4.23E-08	0.001268	1.62E-08	0.007966
0.071777	0.146256	0.172639	0.187996	0.139538	0.056596	0.136977	0.184221	0.082771
0.4226	0.100847	0.05227	0.034297	0.11767	0.527387	0.124627	0.038143	0.354889
0.050345	-0.09483	0.000348	0.220489	-0.13474	-0.10131	0.087791	-0.12786	-0.02935
0.574041	0.28889	0.996899	0.01274	0.130947	0.257086	0.326364	0.152001	0.74326

GSTP1	GTF3C5	GTPBP6	GUK1	GZF1	H1FX	H2AFX	HAGHL	HBA1
black	black	black	black	black	black	black	black	black
-0.12519	-0.08444	-0.11691	0.031353	0.07818	-0.2655	-0.45436	-0.12133	-0.16893
0.160808	0.345218	0.190561	0.7264	0.3823	0.002555	8.06E-08	0.174213	0.057624
-0.13082	-0.0176	-0.06107	0.091172	0.099801	-0.28964	-0.40432	-0.09152	-0.13417
0.142634	0.844282	0.495178	0.308005	0.264262	0.000957	2.43E-06	0.30613	0.132617
-0.08677	0.25979	-0.26403	-0.45038	0.102417	0.078674	0.366642	-0.22306	-0.16635
0.332031	0.003182	0.002704	1.08E-07	0.251882	0.379288	2.24E-05	0.01171	0.061596
-0.36348	-0.26518	-0.49779	-0.68813	0.383653	-0.39797	-0.17332	-0.55701	-0.52371
2.67E-05	0.002587	2.61E-09	3.96E-19	8.49E-06	3.6E-06	0.051334	1.04E-11	2.65E-10
0.360398	0.1721	0.304077	0.483352	-0.10779	-0.12528	-0.0479	0.090726	0.043176
3.15E-05	0.05302	0.00051	8.61E-09	0.227708	0.160502	0.592824	0.310385	0.62982
-0.16128	-0.33548	-0.29941	-0.27805	0.318212	-0.57905	-0.38773	-0.49096	-0.51678
0.070081	0.000115	0.000627	0.00155	0.000267	9.94E-13	6.67E-06	4.62E-09	4.98E-10
-0.0981	-0.20968	0.045684	-0.22247	0.314577	-0.48278	-0.47221	-0.35279	-0.30232
0.27254	0.017982	0.610045	0.011939	0.000316	9.02E-09	2.09E-08	4.74E-05	0.000551
0.078003	-0.37142	-0.07484	-0.00899	0.050445	-0.34014	-0.25566	-0.01778	-0.34608
0.383378	1.71E-05	0.403012	0.920125	0.573286	9.13E-05	0.003719	0.842736	6.73E-05
0.079023	-0.19644	-0.14601	-0.39596	-0.07273	0.042647	0.006134	-0.33053	-0.00828
0.377174	0.026865	0.101421	4.07E-06	0.416473	0.634021	0.94543	0.000147	0.926387
0.309531	-0.24669	0.076292	-0.005	-0.10581	-0.18111	-0.23377	-0.10169	-0.02394
0.000398	0.005176	0.393928	0.955537	0.236414	0.041575	0.008164	0.255305	0.789324
-0.03417	-0.41095	-0.21727	-0.23204	0.04555	-0.29972	-0.33128	-0.3142	-0.31268
0.702911	1.59E-06	0.014138	0.008663	0.611093	0.000619	0.000142	0.000321	0.000345
-0.14107	0.217128	-0.06731	-0.18875	-0.22839	0.436378	0.216449	-0.0976	0.209722
0.113649	0.014205	0.452087	0.033573	0.009807	2.92E-07	0.014518	0.274996	0.017959
0.12912	0.453797	0.076363	-0.25356	-0.18096	0.541895	0.546585	0.196939	0.318736
0.147956	8.4E-08	0.39349	0.004022	0.041747	4.73E-11	2.98E-11	0.026471	0.00026
0.747093	0.588298	0.688026	0.582634	-0.55356	0.780533	0.788246	0.71029	0.627615
6.4E-24	3.52E-13	4.03E-19	6.67E-13	1.48E-11	2.84E-27	3.94E-28	8.66E-21	2.88E-15
0.546471	0.272802	0.388604	0.130643	-0.38403	0.481861	0.422954	0.367395	0.446539
3.01E-11	0.001916	6.34E-06	0.143194	8.31E-06	9.71E-09	7.28E-07	2.15E-05	1.42E-07
0.079482	0.173299	0.319652	0.39482	-0.14369	-0.00641	0.01126	0.427995	0.26408
0.374397	0.051362	0.000249	4.36E-06	0.107023	0.943012	0.900012	5.19E-07	0.002699
-0.05889	0.480179	0.164834	0.198502	-0.12881	0.447407	0.48744	0.404644	0.308616
0.510728	1.11E-08	0.064039	0.025273	0.148945	1.34E-07	6.18E-09	2.38E-06	0.000415
-0.56745	-0.2746	-0.32286	-0.04134	0.320703	-0.29516	-0.28962	-0.16005	-0.31324
3.5E-12	0.001782	0.000214	0.644481	0.000237	0.000755	0.000958	0.072262	0.000336
-0.27169	0.009714	0.02972	0.351608	0.016706	0.05154	-0.11594	0.190762	0.102926
0.002003	0.913689	0.740128	5.05E-05	0.852115	0.564973	0.194265	0.031691	0.249522
-0.07048	0.564586	-0.04531	-0.15863	0.079614	0.287448	0.222446	0.052987	0.221351
0.43105	4.73E-12	0.613003	0.074866	0.373603	0.00105	0.01195	0.554092	0.012386

HCFC1R1	HELZ	HEPN1	HERC4	HES4	HES6	HEXDC	HEXIM2	HGFAC
black	black	black	black	black	black	black	black	black
0.216013	-0.11506	-0.28028	0.288278	-0.42372	-0.28201	-0.05258	-0.06061	-0.33407
0.014722	0.197701	0.001415	0.001014	6.91E-07	0.001317	0.557149	0.498428	0.000124
0.214089	-0.19957	-0.33256	0.308893	-0.42003	-0.20466	-0.10282	-0.04162	-0.26233
0.015653	0.02448	0.000133	0.00041	8.83E-07	0.020996	0.249993	0.642247	0.002888
-0.49291	0.416825	0.115758	-0.29081	0.408921	0.167849	0.070939	0.03708	0.414432
3.93E-09	1.09E-06	0.194979	0.00091	1.81E-06	0.05926	0.428048	0.678963	1.27E-06
-0.71389	0.64578	-0.16009	0.056672	-0.10919	-0.2684	-0.23855	-0.38792	0.117776
4.49E-21	2.46E-16	0.072187	0.526825	0.221697	0.002281	0.006917	6.6E-06	0.187256
0.392551	-0.50096	-0.32322	-0.04443	-0.14503	0.13785	0.013583	0.297676	-0.0245
5E-06	1.99E-09	0.00021	0.619901	0.103771	0.122222	0.879525	0.000677	0.784531
-0.33839	0.20658	-0.43231	0.186506	-0.39213	-0.31216	-0.33145	-0.30118	-0.11044
9.98E-05	0.019795	3.87E-07	0.035773	5.13E-06	0.000353	0.000141	0.00058	0.216428
-0.29448	0.187838	-0.53019	0.138465	-0.47033	-0.36478	-0.35909	-0.19208	-0.13724
0.000778	0.034451	1.45E-10	0.120547	2.41E-08	2.48E-05	3.39E-05	0.030511	0.123911
-0.05972	-0.00562	-0.14316	0.018561	-0.45389	-0.01129	-0.22879	-0.31078	-0.14051
0.504827	0.949996	0.108343	0.835918	8.34E-08	0.899775	0.009673	0.000376	0.11512
-0.42706	0.383016	-0.00692	-0.07318	0.108849	-0.40455	0.118228	-0.07904	-0.04984
5.53E-07	8.81E-06	0.938468	0.413548	0.223165	2.39E-06	0.185558	0.377066	0.577899
0.0303	-0.08271	-0.11767	0.108902	-0.28304	-0.3258	0.211931	-0.06405	-0.28758
0.73524	0.355232	0.187663	0.222938	0.001262	0.000186	0.016758	0.47436	0.001044
-0.18757	0.126876	-0.05438	0.0387	-0.29203	-0.35526	0.02634	-0.23021	-0.16085
0.034718	0.155191	0.543715	0.665757	0.000864	4.16E-05	0.768795	0.00922	0.070838
-0.21592	0.172808	0.171939	-0.18781	0.206796	-0.0377	0.173949	0.286532	0.120104
0.014766	0.052036	0.053246	0.034481	0.019664	0.673856	0.050481	0.001091	0.178628
-0.17679	0.247381	0.467095	-0.36005	0.579544	0.268413	0.494794	0.292634	0.415101
0.046775	0.005047	3.1E-08	3.21E-05	9.41E-13	0.00228	3.36E-09	0.000842	1.22E-06
0.54042	-0.52542	0.620931	-0.49144	0.711089	0.584898	0.742098	0.7701	0.465904
5.46E-11	2.26E-10	6.83E-15	4.44E-09	7.49E-21	5.18E-13	1.83E-23	3.63E-26	3.39E-08
0.186615	-0.16494	0.447816	-0.2509	0.371328	0.1568	0.668784	0.367932	0.168471
0.035663	0.063868	1.3E-07	0.004438	1.72E-05	0.078327	8.52E-18	2.08E-05	0.058311
0.423516	-0.43683	0.093432	0.233063	-0.11556	0.213841	0.083418	0.03874	-0.1671
7.01E-07	2.83E-07	0.296112	0.008366	0.195743	0.015777	0.351128	0.665432	0.060416
0.165582	-0.12115	0.346855	-0.28149	0.505136	0.596937	0.055592	0.21465	0.340241
0.062825	0.174839	6.47E-05	0.001346	1.39E-09	1.29E-13	0.53475	0.015377	9.08E-05
-0.08873	0.136145	-0.17299	0.271447	-0.20919	-0.06749	-0.60316	-0.37584	-0.13461
0.32117	0.126954	0.051787	0.002022	0.01826	0.450924	6.18E-14	1.33E-05	0.131328
0.332021	-0.24312	-0.08435	0.113314	-0.085	0.150693	-0.35884	-0.05135	-0.1698
0.000137	0.005885	0.345742	0.204637	0.342008	0.090817	3.43E-05	0.566396	0.056321
-0.07155	0.201426	-0.10356	-0.04836	0.298603	0.137775	0.170376	0.26157	0.146103
0.424095	0.023157	0.246585	0.589256	0.00065	0.122425	0.055482	0.002973	0.101208

HIC1	HIGD2A	HIRIP3	HIST1H4C	HIST2H2A	HIST2H3D	HIST3H2A	HMG20B	HOMER3
black	black	black	black	black	black	black	black	black
-0.24264	0.05682	-0.14284	-0.19866	-0.20076	-0.17201	-0.00831	-0.30872	-0.13121
0.005986	0.525748	0.109136	0.025156	0.023624	0.053146	0.926149	0.000413	0.141455
-0.36438	0.109505	-0.1038	-0.2331	-0.23924	-0.20849	0.016324	-0.3899	-0.18664
2.54E-05	0.220369	0.245495	0.008355	0.006752	0.018663	0.855457	5.87E-06	0.035636
0.195011	-0.30991	0.311365	0.113024	0.401284	0.091469	0.001176	0.284842	-0.00491
0.028016	0.000392	0.000366	0.205805	2.93E-06	0.306425	0.989531	0.001171	0.95627
-0.03205	-0.62872	-0.19008	-0.18209	0.08023	-0.10764	-0.3472	-0.0298	-0.23373
0.720587	2.49E-15	0.032314	0.040468	0.369902	0.228386	6.36E-05	0.73947	0.008175
-0.29862	0.471035	0.074758	0.200512	0.081985	-0.13692	0.343039	-0.07511	-0.09559
0.000649	2.29E-08	0.403536	0.023802	0.359491	0.124788	7.88E-05	0.401326	0.285058
-0.28366	-0.29748	-0.32681	-0.14528	-0.05954	-0.23857	-0.18357	-0.238	-0.36096
0.00123	0.000683	0.000177	0.103175	0.506098	0.006911	0.038842	0.00705	3.06E-05
-0.38718	-0.17308	-0.29326	-0.28434	-0.10484	-0.29075	-0.18211	-0.30051	-0.33181
6.9E-06	0.051657	0.000819	0.001196	0.24076	0.000913	0.040447	0.000597	0.000138
-0.32203	-0.23484	-0.2123	-0.15996	-0.21998	-0.0975	-0.19004	-0.13165	-0.14231
0.000222	0.007871	0.016562	0.072426	0.012951	0.275469	0.032353	0.140097	0.110493
0.614266	-0.12728	-0.13607	0.067414	0.31965	0.21548	-0.24564	0.368577	0.212422
1.59E-14	0.15387	0.127176	0.451413	0.000249	0.014975	0.005375	2.01E-05	0.016502
0.184183	0.199021	-0.19026	-0.02995	0.067045	0.153621	-0.20011	0.227921	0.240498
0.038183	0.024886	0.032152	0.738217	0.453901	0.084644	0.024086	0.009962	0.006458
0.040379	-0.11355	-0.22445	-0.1303	-0.00224	0.033912	-0.33795	0.031144	0.103516
0.652179	0.2037	0.011186	0.144272	0.980056	0.70506	0.000102	0.728149	0.246803
0.190185	0.269512	0.253573	0.085837	0.147294	0.038619	-0.19568	0.101956	0.426016
0.03222	0.002183	0.004021	0.337285	0.098426	0.66642	0.027469	0.254037	5.93E-07
0.504559	0.025366	0.507433	0.174473	0.416695	0.340775	0.02982	0.580793	0.496925
1.46E-09	0.777118	1.14E-09	0.049781	1.1E-06	8.84E-05	0.73928	8.2E-13	2.81E-09
0.559491	0.643133	0.671609	0.569364	0.56168	0.469585	0.486789	0.792071	0.763801
8.06E-12	3.55E-16	5.52E-18	2.85E-12	6.42E-12	2.56E-08	6.52E-09	1.43E-28	1.59E-25
0.603089	0.403337	0.38109	0.26121	0.390258	0.464292	0.064855	0.732529	0.734651
6.23E-14	2.58E-06	9.86E-06	0.003014	5.74E-06	3.84E-08	0.468808	1.28E-22	8.38E-23
-0.31193	0.297284	0.147853	-0.10027	-0.34668	-0.06536	0.171697	-0.14725	0.047351
0.000357	0.000688	0.09714	0.26203	6.53E-05	0.465365	0.053588	0.098521	0.597056
-0.0755	0.103678	0.440827	0.15141	0.055969	0.009243	0.304264	0.047255	0.062348
0.398848	0.24606	2.14E-07	0.089274	0.531972	0.917854	0.000506	0.597798	0.486202
-0.44844	-0.43858	-0.3635	-0.23075	-0.43121	-0.35258	-0.04506	-0.67198	-0.64845
1.24E-07	2.5E-07	2.66E-05	0.009052	4.17E-07	4.79E-05	0.614903	5.21E-18	1.69E-16
-0.4038	0.037985	-0.14263	-0.1202	-0.41791	-0.2374	0.170576	-0.54872	-0.40002
2.51E-06	0.671574	0.10968	0.178272	1.02E-06	0.0072	0.055192	2.41E-11	3.17E-06
0.040929	0.091303	0.313052	0.155072	0.304889	-0.02086	0.330785	0.089599	-0.05658
0.647763	0.307304	0.000339	0.081712	0.000492	0.815894	0.000146	0.316456	0.527514

HPS6	HRAS	HSD17B10	HSD17B14	HSF1	HSPA4	HSPB1	HSPB6	HSPBP1
black	black	black	black	black	black	black	black	black
-0.07823	0.007952	-0.30678	0.180502	-0.17541	-0.00992	-0.10238	-0.09405	-0.21819
0.381966	0.929293	0.000451	0.04228	0.048542	0.911832	0.252047	0.292917	0.013726
-0.02864	0.065444	-0.26275	0.132651	-0.18168	-0.08681	-0.11449	-0.10805	-0.1793
0.74924	0.464771	0.002841	0.137093	0.040935	0.331832	0.199935	0.22662	0.043697
-0.12566	-0.30301	0.297552	-0.48512	0.031022	0.217982	-0.28701	-0.19476	-0.1241
0.15921	0.000535	0.00068	7.47E-09	0.729174	0.01382	0.00107	0.028222	0.164503
-0.44174	-0.73702	0.019428	-0.54614	-0.39539	0.538051	-0.37795	-0.3483	-0.60398
2.01E-07	5.19E-23	0.828363	3.11E-11	4.21E-06	6.86E-11	1.18E-05	6E-05	5.6E-14
0.177279	0.294815	0.513431	0.123203	-0.20238	-0.16286	0.040943	-0.47456	0.018224
0.046163	0.000766	6.72E-10	0.167596	0.022497	0.067332	0.64765	1.73E-08	0.838852
-0.33259	-0.50797	0.180556	-0.33406	-0.62489	0.464262	-0.27367	-0.60406	-0.63292
0.000133	1.09E-09	0.042217	0.000124	4.1E-15	3.85E-08	0.00185	5.54E-14	1.43E-15
-0.3362	-0.35585	0.057259	-0.21943	-0.39907	0.245691	-0.23658	-0.38608	-0.48754
0.000111	4.03E-05	0.522553	0.013185	3.36E-06	0.005366	0.007408	7.36E-06	6.13E-09
-0.3279	-0.24207	0.065814	0.087422	-0.42586	0.347465	0.003305	-0.0903	-0.19677
0.000168	0.006108	0.462248	0.328407	5.99E-07	6.27E-05	0.970579	0.312685	0.026603
-0.07739	-0.39553	0.08608	-0.3229	0.150282	0.217342	0.191303	0.196659	-0.24928
0.387113	4.17E-06	0.335914	0.000213	0.091712	0.014107	0.031201	0.026691	0.004711
-0.05581	-0.12652	0.143464	0.277045	-0.1065	0.072272	0.382616	0.101931	-0.23094
0.53313	0.156346	0.107592	0.001615	0.233383	0.419399	9.02E-06	0.254155	0.008994
-0.233	-0.32077	-0.00163	0.132943	-0.24313	0.342114	0.143828	0.035096	-0.40696
0.008384	0.000236	0.985533	0.136224	0.005882	8.26E-05	0.106695	0.695262	2.05E-06
0.088639	0.035491	-0.04967	-0.0971	0.624152	-0.37395	0.108246	0.460143	0.145113
0.321693	0.69201	0.579186	0.277484	4.51E-15	1.49E-05	0.225752	5.25E-08	0.103568
0.113558	0.082406	0.073588	-0.01224	0.671704	-0.24734	0.15483	0.521989	0.375101
0.203658	0.357023	0.410958	0.891382	5.44E-18	0.005054	0.082195	3.1E-10	1.39E-05
0.629788	0.703587	0.550618	0.537842	0.751977	-0.62305	0.662969	0.571103	0.860932
2.16E-15	2.86E-20	1.99E-11	7E-11	2.24E-24	5.2E-15	2.05E-17	2.37E-12	1.68E-38
0.259417	0.281926	0.253014	0.44073	0.614763	-0.35155	0.633234	0.664637	0.410773
0.003228	0.001322	0.004105	2.15E-07	1.49E-14	5.06E-05	1.37E-15	1.6E-17	1.61E-06
0.134521	0.478377	-0.18218	0.412131	0.057487	-0.34709	0.052296	0.191499	0.27058
0.131592	1.28E-08	0.040365	1.48E-06	0.520888	6.39E-05	0.559275	0.031025	0.002093
0.211628	0.400898	-0.02317	0.017922	0.385133	-0.34776	-0.17134	0.144892	0.531887
0.016919	3E-06	0.795983	0.841484	7.78E-06	6.17E-05	0.0541	0.104101	1.24E-10
-0.13529	-0.14589	-0.44966	-0.30258	-0.48723	0.256907	-0.54055	-0.40072	-0.18808
0.129375	0.10172	1.14E-07	0.000545	6.28E-09	0.003549	5.39E-11	3.04E-06	0.034215
0.132076	0.334271	-0.39478	0.040666	-0.06495	-0.15202	-0.25851	-0.10697	0.258875
0.13882	0.000123	4.37E-06	0.649873	0.468167	0.087976	0.00334	0.231317	0.003295
0.002454	0.05694	0.032695	-0.41673	0.328419	-0.28195	-0.44049	-0.34582	0.047645
0.978155	0.52487	0.715183	1.1E-06	0.000164	0.001321	2.19E-07	6.83E-05	0.594779

IARS	IBTK	ICE1	ID1	ID3	IDH2	IER2	IER5L	IFNAR1
black	black	black	black	black	black	black	black	black
-0.01874	0.051988	-0.14871	-0.15756	-0.25386	-0.21158	-0.09217	-0.15544	-0.03427
0.83435	0.561593	0.095193	0.076878	0.003979	0.016943	0.302732	0.080978	0.702105
-0.00192	0.018935	-0.16565	-0.20683	-0.31758	-0.16488	-0.18294	-0.21914	-0.09565
0.982886	0.832655	0.062723	0.019646	0.000275	0.063968	0.03953	0.013311	0.284739
0.476361	0.234972	0.430889	-0.06466	0.216057	0.06013	-0.23986	0.073593	0.206019
1.51E-08	0.007834	4.26E-07	0.470165	0.014701	0.501879	0.006605	0.410928	0.020139
0.536823	0.653283	0.676445	-0.31709	-0.01148	-0.41895	-0.41342	-0.23989	0.532284
7.72E-11	8.48E-17	2.6E-18	0.000281	0.898034	9.48E-07	1.36E-06	0.006598	1.19E-10
-0.25274	-0.04398	-0.41373	-0.13672	-0.20305	-0.03224	-0.31648	-0.26137	-0.42187
0.004146	0.623448	1.33E-06	0.125336	0.022049	0.718946	0.000289	0.002996	7.82E-07
0.23244	0.630419	0.291877	-0.43087	-0.23413	-0.48431	-0.5444	-0.48657	0.278348
0.008546	1.99E-15	0.00087	4.27E-07	0.008064	7.97E-09	3.7E-11	6.63E-09	0.001531
0.230031	0.397118	0.269687	-0.32005	-0.32553	-0.49471	-0.37501	-0.43036	0.253269
0.009277	3.79E-06	0.002168	0.000244	0.000188	3.38E-09	1.4E-05	4.42E-07	0.004066
-0.03539	0.158639	0.196992	-0.20488	-0.13369	-0.13702	-0.08641	-0.41229	0.061143
0.692833	0.074847	0.02643	0.020856	0.134028	0.124515	0.334086	1.46E-06	0.494688
0.402073	0.246009	0.203932	0.173929	0.344268	-0.10233	0.309228	0.390074	0.605661
2.79E-06	0.005304	0.021466	0.050509	7.4E-05	0.252282	0.000404	5.81E-06	4.57E-14
-0.15989	0.145281	-0.23799	0.246256	0.26755	-0.0435	0.352082	0.125258	0.304491
0.072562	0.103165	0.007054	0.005257	0.002358	0.62725	4.92E-05	0.160568	0.0005
0.073451	0.232538	-0.00097	-0.04124	0.108848	-0.19023	0.078013	-0.03636	0.317231
0.41183	0.008518	0.991397	0.645276	0.223168	0.032182	0.383321	0.684838	0.000279
0.287001	-0.37395	-0.12152	0.159297	0.114267	0.093121	0.248778	0.542063	0.013863
0.00107	1.49E-05	0.173539	0.073635	0.20083	0.297734	0.004797	4.65E-11	0.877068
0.215751	-0.31398	0.131105	0.421089	0.451491	0.407645	0.422107	0.610955	0.078295
0.014846	0.000325	0.141774	8.23E-07	9.95E-08	1.97E-06	7.7E-07	2.39E-14	0.381597
-0.4134	-0.69983	-0.57222	0.613896	0.510153	0.720769	0.542905	0.741114	-0.48832
1.36E-06	5.5E-20	2.1E-12	1.66E-14	8.98E-10	1.25E-21	4.28E-11	2.24E-23	5.75E-09
-0.22112	-0.38132	-0.34364	0.638521	0.589409	0.500382	0.721204	0.694219	0.040155
0.012481	9.73E-06	7.64E-05	6.7E-16	3.1E-13	2.09E-09	1.15E-21	1.43E-19	0.653984
-0.47116	-0.27233	-0.41917	0.140561	-0.06454	0.218048	0.127883	-0.09262	-0.33833
2.26E-08	0.001952	9.35E-07	0.114975	0.470999	0.01379	0.151912	0.300343	0.0001
-0.08222	-0.49461	-0.08714	0.146845	0.088297	0.380987	0.054226	0.15509	-0.42766
0.358117	3.41E-09	0.330001	0.099467	0.323571	9.92E-06	0.544847	0.081675	5.31E-07
0.072839	0.226797	0.300291	-0.50102	-0.48565	-0.33836	-0.53775	-0.52358	-0.08769
0.415749	0.010344	0.000603	1.98E-09	7.15E-09	9.99E-05	7.07E-11	2.68E-10	0.326937
-0.17815	-0.30752	-0.12263	-0.2003	-0.39335	-0.00499	-0.15516	-0.27831	-0.40318
0.045085	0.000437	0.16961	0.023955	4.77E-06	0.955586	0.081532	0.001534	2.61E-06
0.231506	0.006815	0.157833	0.038935	-0.10014	-0.0122	-0.13675	0.181544	-0.1661
0.008823	0.939385	0.076358	0.663852	0.262644	0.891686	0.125249	0.041085	0.061993

IFT80	IGFALS	IL17RC	IL6ST	ILVBL	IMP3	IMP4	IMPAD1	INAFM1
black	black	black	black	black	black	black	black	black
0.095451	-0.05463	-0.22666	-0.05368	-0.06098	0.093426	-0.12616	-0.15405	-0.14512
0.285753	0.541846	0.010391	0.548919	0.495824	0.296144	0.157556	0.083762	0.103547
0.091017	-0.03636	-0.29905	-0.05277	-0.04114	0.200776	-0.06639	-0.15732	-0.15566
0.30883	0.684892	0.000637	0.555702	0.646091	0.023613	0.458313	0.077321	0.080549
0.074782	-0.01899	-0.03191	0.093899	-0.1472	-0.36359	-0.05176	0.277553	0.03261
0.403385	0.832208	0.721762	0.293695	0.098634	2.65E-05	0.563333	0.001582	0.715891
0.410603	-0.30358	-0.04686	0.512418	-0.46486	-0.46258	-0.44224	0.552728	-0.20208
1.63E-06	0.000521	0.600875	7.35E-10	3.67E-08	4.37E-08	1.94E-07	1.61E-11	0.022704
-0.20343	0.025041	-0.07376	-0.13435	0.189783	0.415549	0.325648	-0.28615	0.038172
0.021796	0.7799	0.409831	0.132098	0.032593	1.18E-06	0.000187	0.001109	0.670049
0.30137	-0.36709	-0.11797	0.448548	-0.3648	-0.13713	-0.29074	0.352195	-0.22988
0.000575	2.18E-05	0.186521	1.23E-07	2.48E-05	0.124194	0.000913	4.89E-05	0.009325
0.151356	-0.24384	-0.11261	0.311641	-0.24853	-0.03864	-0.1693	0.246392	-0.21184
0.089389	0.005734	0.207467	0.000362	0.004842	0.666225	0.057071	0.005231	0.016806
0.392896	-0.20168	0.19892	0.175744	-0.18598	-0.23599	-0.24837	0.002228	-0.18111
4.9E-06	0.02298	0.024961	0.048113	0.036303	0.007563	0.004868	0.980167	0.041584
-0.13354	-0.20445	0.31476	0.139273	-0.00885	-0.11483	-0.31552	0.234229	0.429651
0.134451	0.02113	0.000313	0.118377	0.921378	0.198587	0.000302	0.008037	4.64E-07
-0.10752	-0.22249	0.24665	0.088779	0.152469	0.043613	-0.19536	-0.13922	0.240433
0.228891	0.011931	0.005183	0.320928	0.08703	0.626357	0.027729	0.118511	0.006473
0.131648	-0.21932	0.154243	0.20894	-0.12595	-0.0766	-0.36282	0.062896	0.008183
0.140116	0.013234	0.083376	0.018402	0.158258	0.392007	2.76E-05	0.482373	0.927246
-0.11301	0.147123	0.029138	-0.17318	0.115151	0.425674	-0.20374	-0.02487	0.256538
0.205871	0.09882	0.745035	0.051522	0.197346	6.07E-07	0.021589	0.781325	0.003599
-0.18849	0.331038	0.239343	-0.33045	0.259689	-0.1613	0.092916	-0.13588	0.342855
0.033818	0.000144	0.006726	0.000148	0.003195	0.07004	0.298802	0.127704	7.95E-05
-0.6015	0.606176	0.478876	-0.61245	0.8328	0.410206	0.651082	-0.58195	0.656153
7.53E-14	4.29E-14	1.23E-08	1.99E-14	6.72E-34	1.67E-06	1.16E-16	7.2E-13	5.6E-17
-0.37736	0.286909	0.480204	-0.39824	0.570718	0.116365	0.150206	-0.39416	0.550408
1.22E-05	0.001074	1.11E-08	3.54E-06	2.47E-12	0.192634	0.091877	4.54E-06	2.03E-11
0.026819	0.170545	-0.23287	-0.21014	0.271998	0.206111	0.21127	-0.30113	-0.27669
0.764711	0.055237	0.008421	0.017725	0.001978	0.020082	0.01711	0.000581	0.001638
-0.13322	0.321443	-0.22954	-0.28757	0.148297	0.031726	0.398472	-0.11299	-0.15749
0.135406	0.000229	0.009431	0.001045	0.096129	0.72327	3.49E-06	0.205924	0.077007
0.344251	-0.17427	-0.43664	0.381617	-0.48601	-0.16521	-0.08756	0.401984	-0.54516
7.4E-05	0.050056	2.87E-07	9.56E-06	6.94E-09	0.063422	0.327652	2.81E-06	3.43E-11
0.013309	0.046166	-0.34677	-0.06453	-0.13604	0.151238	0.218102	-0.06016	-0.32162
0.881942	0.606274	6.5E-05	0.471064	0.127251	0.089641	0.013766	0.501664	0.000227
-0.07465	0.169493	-0.32489	-0.25278	-0.04218	-0.10934	0.131541	-0.03962	-0.07887
0.404205	0.056779	0.000194	0.004141	0.637774	0.221062	0.140441	0.658323	0.37809

INO80E	INPP4B	INTS1	INTU	IRF2BP1	IRF2BPL	IRF3	ISOC2	ISYNA1
black	black	black	black	black	black	black	black	black
-0.24798	0.011119	-0.25555	0.121724	-0.30972	-0.21877	-0.03377	-0.14748	-0.34745
0.004939	0.901263	0.003734	0.172798	0.000395	0.013471	0.706258	0.097992	6.27E-05
-0.215	0.016046	-0.29484	0.147329	-0.32072	-0.29455	-0.07304	-0.13582	-0.3963
0.015207	0.857895	0.000766	0.098344	0.000237	0.000775	0.414435	0.127868	3.98E-06
0.113264	-0.00815	0.06303	0.001816	-0.00915	0.187113	-0.10563	-0.15265	0.247306
0.204838	0.927526	0.481438	0.983837	0.918675	0.035165	0.237227	0.08666	0.005061
-0.28408	0.237849	-0.39317	0.441819	-0.44048	-0.09195	-0.23007	-0.59934	-0.14986
0.001209	0.007088	4.82E-06	1.99E-07	2.19E-07	0.303862	0.009264	9.75E-14	0.092631
0.358424	-0.05853	-0.51844	0.224474	-0.3529	-0.39179	0.144638	0.187688	-0.00175
3.51E-05	0.51335	4.29E-10	0.011176	4.71E-05	5.24E-06	0.104714	0.034597	0.984433
-0.19826	0.267896	-0.79278	0.600817	-0.7352	-0.43932	-0.18889	-0.50773	-0.30735
0.025458	0.002326	1.18E-28	8.18E-14	7.5E-23	2.38E-07	0.033438	1.11E-09	0.00044
-0.21232	0.153309	-0.58954	0.392458	-0.62327	-0.48692	-0.00612	-0.39359	-0.19513
0.016554	0.085286	3.06E-13	5.03E-06	5.06E-15	6.45E-09	0.945577	4.7E-06	0.027914
-0.18652	0.078884	-0.36867	0.538181	-0.33837	-0.36206	-0.02819	-0.29678	-0.17263
0.035757	0.378016	2E-05	6.78E-11	9.99E-05	2.88E-05	0.753072	0.000704	0.052286
-0.05061	0.000602	0.150505	-0.17574	-0.00414	0.265967	0.284339	-0.14031	0.319642
0.572018	0.994638	0.091226	0.048123	0.963168	0.002509	0.001196	0.115627	0.000249
-0.05023	-0.19127	-0.15887	0.019856	-0.23585	-0.07432	0.4187	-0.06968	-0.06724
0.574934	0.031232	0.074413	0.824647	0.0076	0.406303	9.64E-07	0.436281	0.452562
-0.26583	-0.00629	-0.23875	0.188903	-0.33756	-0.07057	0.151349	-0.29576	-0.22078
0.002522	0.944085	0.006868	0.033423	0.000104	0.430432	0.089405	0.000736	0.012621
0.218858	-0.37452	0.452787	-0.36999	0.358139	0.364724	0.096309	0.091525	0.141001
0.013434	1.44E-05	9.05E-08	1.86E-05	3.56E-05	2.49E-05	0.281424	0.306127	0.113831
0.266392	-0.36192	0.786904	-0.4759	0.642925	0.578112	0.311539	0.256116	0.482322
0.002467	2.9E-05	5.58E-28	1.56E-08	3.66E-16	1.1E-12	0.000363	0.003656	9.36E-09
0.795557	-0.51428	0.769427	-0.59738	0.846135	0.582561	0.664298	0.877344	0.680577
5.6E-29	6.23E-10	4.26E-26	1.23E-13	5.77E-36	6.73E-13	1.68E-17	1.12E-41	1.35E-18
0.367209	-0.50232	0.678002	-0.50249	0.568584	0.469351	0.672147	0.446029	0.453169
2.17E-05	1.77E-09	2.03E-18	1.75E-09	3.1E-12	2.6E-08	5.08E-18	1.48E-07	8.8E-08
0.008656	-0.20879	0.111051	-0.12936	0.150056	-0.20599	-0.01008	0.249315	-0.24077
0.923054	0.018487	0.213884	0.147184	0.092205	0.020157	0.91046	0.004704	0.006396
0.20628	-0.13832	0.472462	-0.39196	0.512107	0.275271	-0.20591	0.379537	0.209544
0.019978	0.120949	2.04E-08	5.18E-06	7.56E-10	0.001735	0.020206	1.08E-05	0.018059
-0.39994	0.53178	-0.35202	0.284191	-0.24632	-0.2417	-0.70796	-0.27325	-0.39863
3.19E-06	1.25E-10	4.94E-05	0.001204	0.005246	0.00619	1.32E-20	0.001882	3.45E-06
-0.14675	0.237312	0.012308	-0.07615	0.104119	-0.13183	-0.4452	0.132628	-0.20712
0.099692	0.007222	0.890765	0.394829	0.244046	0.139554	1.57E-07	0.137163	0.019466
0.208331	-0.14167	0.088209	-0.19385	0.048374	0.082157	-0.04282	0.073146	0.326014
0.018753	0.112111	0.324052	0.028979	0.58915	0.358481	0.632652	0.413782	0.000184

JAZF1	JOSD2	JUNB	JUP	KATNAL1	KBTBD8	KCMF1	KCNG2	KDM7A
black	black	black	black	black	black	black	black	black
0.213565	-0.0358	-0.02864	0.092525	0.101355	0.053079	0.023488	-0.44478	-0.12518
0.015916	0.689466	0.749284	0.300851	0.256859	0.5534	0.793236	1.62E-07	0.160839
0.19249	-0.06353	-0.09669	0.083355	0.124186	0.051818	0.108505	-0.44344	-0.14447
0.03015	0.477988	0.279502	0.351494	0.164209	0.562873	0.224637	1.78E-07	0.105132
-0.15432	-0.28928	-0.33827	-0.27141	0.023841	0.188615	0.172384	-0.1316	0.322585
0.083219	0.000972	0.0001	0.002025	0.790193	0.033698	0.052624	0.140254	0.000217
0.065828	-0.55202	-0.47604	-0.46691	0.363016	0.399135	0.39488	-0.26413	0.579349
0.462153	1.73E-11	1.54E-08	3.14E-08	2.73E-05	3.35E-06	4.34E-06	0.002694	9.62E-13
0.099964	0.016217	-0.25837	-0.14181	-0.27815	0.144039	0.268138	-0.35858	-0.22749
0.263483	0.856394	0.003358	0.111744	0.001544	0.106176	0.002304	3.48E-05	0.010106
0.251687	-0.49098	-0.52089	-0.48203	0.240431	0.498355	0.535924	-0.46414	0.339924
0.004312	4.61E-09	3.43E-10	9.58E-09	0.006473	2.49E-09	8.42E-11	3.88E-08	9.23E-05
0.056769	-0.47767	-0.41111	-0.2097	0.199475	0.275884	0.460207	-0.34857	0.275453
0.526119	1.36E-08	1.58E-06	0.017973	0.024552	0.001693	5.22E-08	5.92E-05	0.001722
0.106499	-0.16009	-0.08124	-0.32682	0.312091	-0.04235	-0.04982	-0.13201	-0.08309
0.233377	0.072197	0.363902	0.000177	0.000354	0.636368	0.578079	0.139023	0.353003
-0.13667	0.066906	0.22614	0.234989	-0.09839	-0.01829	0.07184	0.208713	0.263137
0.125485	0.454834	0.010574	0.007829	0.271094	0.838285	0.422186	0.018532	0.002799
0.029463	0.291829	0.34239	0.156906	-0.12995	-0.02125	-0.22266	-0.00411	-0.09158
0.742292	0.000872	8.14E-05	0.078121	0.145352	0.812537	0.011867	0.963383	0.305822
0.147876	-0.01557	0.0585	-0.02849	0.056022	0.107288	-0.13037	-0.19941	0.067259
0.097089	0.862087	0.513564	0.75052	0.531584	0.229909	0.144026	0.024598	0.452453
-0.12652	0.194587	0.145164	0.352066	-0.22678	-0.38687	-0.0945	0.130082	0.045918
0.156375	0.028365	0.103445	4.93E-05	0.010349	7.03E-06	0.290594	0.144935	0.608214
-0.49623	0.29848	0.27484	0.310116	-0.19008	-0.42738	-0.42407	0.350189	0.020245
2.98E-09	0.000653	0.001765	0.000388	0.032317	5.41E-07	6.76E-07	5.44E-05	0.821263
-0.50826	0.847806	0.542595	0.502502	-0.60302	-0.52466	-0.58006	0.428187	-0.49282
1.06E-09	3.08E-36	4.41E-11	1.75E-09	6.29E-14	2.43E-10	8.89E-13	5.12E-07	3.96E-09
-0.46589	0.733403	0.628222	0.559569	-0.38398	-0.54215	-0.66526	0.405849	-0.2887
3.4E-08	1.08E-22	2.66E-15	7.99E-12	8.33E-06	4.61E-11	1.45E-17	2.2E-06	0.000996
0.008689	0.334966	0.132172	0.26297	0.012337	-0.26384	-0.44557	-0.03108	-0.43829
0.922766	0.000118	0.138532	0.002818	0.890512	0.002725	1.53E-07	0.728675	2.55E-07
-0.12676	0.062786	0.014593	0.06697	-0.08474	-0.15656	-0.19717	0.147541	-0.17106
0.155555	0.48314	0.87065	0.454402	0.343521	0.078788	0.026288	0.097857	0.05449
0.46206	-0.54737	-0.39905	-0.41294	0.396852	0.456005	0.434433	-0.14498	0.20285
4.54E-08	2.76E-11	3.37E-06	1.4E-06	3.85E-06	7.14E-08	3.34E-07	0.103882	0.022182
0.296376	-0.16358	-0.04033	-0.05303	0.063556	0.057851	0.131539	0.05385	-0.17623
0.000716	0.066112	0.652541	0.553761	0.477784	0.518248	0.140447	0.547644	0.047488
-0.2397	-0.14814	-0.28519	-0.05562	-0.12868	0.093422	0.198415	-0.11564	0.154127
0.006643	0.096496	0.001155	0.534522	0.149359	0.296165	0.025339	0.195438	0.083611

KEAP1	KIAA1328	KIAA1468	KIFC3	KLF2	KLF9	KLHDC4	KLHL12	KLHL15
black	black	black	black	black	black	black	black	black
-0.15369	0.062682	0.134839	-0.16755	-0.16687	-0.11681	-0.06397	0.058654	-0.01612
0.084494	0.483869	0.130674	0.059726	0.06078	0.190932	0.474885	0.51245	0.857222
-0.1659	0.077923	0.152364	-0.19867	-0.23092	-0.02272	-0.07792	0.054629	-0.01907
0.06232	0.383871	0.087252	0.025148	0.009001	0.799886	0.383895	0.541859	0.831456
0.088763	0.215491	-0.04176	-0.17396	-0.35206	-0.04301	-0.02543	0.016498	0.141601
0.321014	0.01497	0.641116	0.050467	4.93E-05	0.631137	0.776587	0.85394	0.112286
-0.30294	0.444053	0.263688	-0.49962	-0.52866	0.323069	-0.40275	0.394995	0.409674
0.000536	1.7E-07	0.002741	2.23E-09	1.67E-10	0.000212	2.68E-06	4.31E-06	1.73E-06
-0.23946	0.04872	0.035626	-0.23906	-0.20725	0.009083	-0.01259	-0.03465	-0.05872
0.006698	0.586481	0.690892	0.006794	0.019393	0.91927	0.888286	0.69894	0.511992
-0.55277	0.412221	0.344615	-0.62563	-0.56247	0.407565	-0.45088	0.420638	0.384958
1.6E-11	1.47E-06	7.26E-05	3.73E-15	5.91E-12	1.98E-06	1.04E-07	8.48E-07	7.86E-06
-0.36988	0.359117	0.205004	-0.41286	-0.45617	0.294722	-0.34198	0.249106	0.174735
1.87E-05	3.38E-05	0.020775	1.41E-06	7.06E-08	0.000769	8.32E-05	0.00474	0.049433
-0.29898	0.042609	0.153127	-0.30321	-0.21281	0.078402	-0.18855	0.438468	0.109875
0.000639	0.634324	0.085662	0.00053	0.0163	0.380943	0.033765	2.52E-07	0.218805
0.199286	-0.15063	-0.37341	0.227632	0.172137	0.133199	-0.14395	0.017153	-0.14191
0.02469	0.090957	1.53E-05	0.010059	0.052968	0.135465	0.106396	0.848205	0.111509
0.077861	-0.22368	-0.24143	0.183013	0.315331	-0.0305	0.005202	-0.05513	-0.2639
0.38425	0.011472	0.006249	0.039448	0.000305	0.733568	0.953716	0.538161	0.002718
-0.08412	0.00147	-0.0298	-0.14361	0.011491	0.013947	-0.19645	0.23069	-0.0232
0.347056	0.986915	0.739459	0.107237	0.897978	0.876329	0.026856	0.009072	0.795679
0.37366	-0.01747	-0.34045	0.275882	0.301899	-0.20707	-0.06003	-0.15873	-0.40117
1.51E-05	0.845405	8.99E-05	0.001693	0.000562	0.019499	0.502559	0.074682	2.95E-06
0.677856	-0.17038	-0.46032	0.488196	0.303886	-0.39706	0.393083	-0.33844	-0.35248
2.08E-18	0.055482	5.18E-08	5.81E-09	0.000514	3.8E-06	4.84E-06	9.95E-05	4.82E-05
0.805886	-0.52099	-0.69874	0.732016	0.579585	-0.4845	0.649206	-0.61825	-0.5471
3.1E-30	3.4E-10	6.63E-20	1.42E-22	9.37E-13	7.85E-09	1.52E-16	9.61E-15	2.83E-11
0.742702	-0.48812	-0.67563	0.721823	0.675905	-0.42008	0.484998	-0.46919	-0.61237
1.61E-23	5.84E-09	2.95E-18	1.02E-21	2.83E-18	8.8E-07	7.54E-09	2.64E-08	2.01E-14
0.156803	-0.15878	0.099225	0.221615	0.264061	-0.30047	0.245045	-0.29031	-0.32206
0.07832	0.074593	0.267045	0.01228	0.002701	0.000598	0.005492	0.00093	0.000222
0.290322	-0.10141	-0.0393	0.222926	0.113976	-0.37247	0.289756	-0.32358	-0.11025
0.00093	0.256615	0.6609	0.011762	0.201988	1.62E-05	0.000952	0.000207	0.217241
-0.58546	0.305382	0.654723	-0.49154	-0.45198	0.369086	-0.35322	0.332692	0.627539
4.86E-13	0.000481	6.89E-17	4.41E-09	9.6E-08	1.95E-05	4.64E-05	0.000133	2.9E-15
-0.24876	-0.02086	0.340636	-0.07034	-0.04881	0.033906	-0.04069	0.032617	0.186468
0.0048	0.815969	8.9E-05	0.431989	0.585818	0.705106	0.6497	0.715831	0.035812
0.036279	0.252271	0.042709	0.049484	-0.16294	-0.30253	0.197215	-0.25778	-0.03099
0.685527	0.00422	0.633529	0.580619	0.067196	0.000546	0.026256	0.003434	0.729402

KLHL17	KPNA1	KPTN	KXD1	L3MBTL3	LAGE3	LENG1	LENG9	LGALS8
black	black	black	black	black	black	black	black	black
-0.33756	0.001717	-0.22673	-0.03641	0.04452	0.108577	-0.23164	-0.05877	0.279984
0.000104	0.984714	0.010366	0.68442	0.619189	0.224328	0.008783	0.511622	0.001432
-0.38149	0.040279	-0.20819	-0.01644	0.004574	0.17592	-0.21913	-0.06294	0.252894
9.63E-06	0.652988	0.018832	0.854414	0.959296	0.047886	0.013316	0.482068	0.004123
0.327196	0.079988	0.169352	0.056065	0.131038	-0.38235	0.085703	-0.00387	-0.43331
0.000174	0.371353	0.056988	0.531273	0.14198	9.17E-06	0.338045	0.965595	3.61E-07
-0.1603	0.336552	-0.12864	-0.24957	0.433316	-0.58785	-0.29433	-0.16462	0.019431
0.071812	0.000109	0.14949	0.00466	3.61E-07	3.7E-13	0.000783	0.064389	0.828342
-0.1859	-0.35387	0.0092	0.022387	-0.00214	0.615943	0.180284	-0.14572	-0.13285
0.036389	4.48E-05	0.918235	0.802722	0.980943	1.29E-14	0.042534	0.102123	0.136495
-0.47411	0.138266	-0.26236	-0.33828	0.424884	-0.10288	-0.286	-0.27656	0.160128
1.8E-08	0.121086	0.002885	0.0001	6.4E-07	0.249727	0.001116	0.001647	0.072125
-0.397	0.198381	-0.1826	-0.13984	0.226902	-0.20585	-0.30578	-0.19828	0.228461
3.82E-06	0.025364	0.039897	0.11687	0.010308	0.020247	0.000472	0.025437	0.009782
-0.41004	0.101413	-0.175	-0.32055	-0.09103	0.076667	-0.29795	-0.28168	0.197907
1.69E-06	0.256586	0.049084	0.000239	0.308739	0.391607	0.000668	0.001336	0.025724
0.193002	0.040649	-0.02431	0.309081	0.199048	-0.45005	0.01358	0.381458	0.141354
0.029705	0.650011	0.786184	0.000407	0.024866	1.11E-07	0.879554	9.65E-06	0.112921
-0.13782	-0.19224	-0.10858	0.160767	-0.05946	0.043896	0.020279	0.293974	0.256404
0.122309	0.030372	0.224335	0.07098	0.506672	0.624111	0.820975	0.000795	0.003617
-0.29258	0.070804	-0.14242	-0.05032	0.106479	-0.14542	-0.20614	0.114981	0.339996
0.000844	0.428926	0.110194	0.574257	0.233462	0.10284	0.020066	0.198013	9.2E-05
0.29314	0.270529	0.190451	0.625625	0.03588	-0.27471	0.095237	0.285036	0.066618
0.000824	0.002097	0.031975	3.73E-15	0.688803	0.001774	0.286842	0.001162	0.456782
0.724686	-0.07078	0.430721	0.504481	-0.26188	-0.34715	0.348405	0.393926	-0.41469
5.93E-22	0.429088	4.31E-07	1.47E-09	0.002938	6.37E-05	5.97E-05	4.6E-06	1.25E-06
0.799886	-0.60403	0.602509	0.655494	-0.5475	0.506531	0.832826	0.589956	-0.5295
1.7E-29	5.56E-14	6.68E-14	6.16E-17	2.72E-11	1.23E-09	6.66E-34	2.91E-13	1.55E-10
0.576525	-0.3643	0.354161	0.606465	-0.41713	0.047111	0.496898	0.633311	-0.18924
1.31E-12	2.55E-05	4.41E-05	4.15E-14	1.07E-06	0.598916	2.82E-09	1.35E-15	0.033106
-0.11405	-0.03683	-0.03683	0.00959	-0.37569	0.32974	0.119601	0.029596	0.009375
0.201699	0.681022	0.681009	0.914785	1.35E-05	0.000153	0.180465	0.741166	0.91669
0.359558	0.011049	0.228662	0.111355	-0.32054	0.055542	0.225095	-0.08708	-0.41714
3.3E-05	0.901879	0.009716	0.212626	0.000239	0.535114	0.010948	0.33029	1.07E-06
-0.42078	0.384683	-0.3239	-0.6516	0.328775	-0.05154	-0.42493	-0.52635	0.18503
8.4E-07	7.99E-06	0.000203	1.08E-16	0.000161	0.565003	6.37E-07	2.08E-10	0.037289
-0.21371	0.133829	-0.13822	-0.31305	-0.06373	0.264693	-0.20171	-0.39872	0.114529
0.015845	0.133609	0.121219	0.000339	0.476587	0.002636	0.022957	3.44E-06	0.199795
0.44032	-0.02184	0.034317	0.181161	0.07776	-0.20666	0.180923	-0.10798	-0.54649
2.22E-07	0.807472	0.701705	0.04152	0.384865	0.019748	0.041793	0.22691	3.01E-11

LIG4	LIMD2	LIME1	LKAAEAR1	LMF2	LMLN	LONP1	LONP2	LPIN2
black	black	black	black	black	black	black	black	black
0.091697	-0.03246	-0.19884	-0.29896	-0.19761	0.071706	-0.15949	-0.03468	0.022932
0.305212	0.717115	0.025017	0.00064	0.025952	0.423059	0.073279	0.698739	0.798021
0.08464	-0.10075	-0.22872	-0.28861	-0.29958	0.07965	-0.18236	-0.05434	-0.02198
0.34409	0.259706	0.009697	0.001	0.000622	0.373384	0.040173	0.544008	0.806232
-0.05255	0.021762	-0.0644	0.062933	0.029005	0.037848	0.102627	-0.00151	-0.1331
0.557378	0.808123	0.471912	0.482117	0.74616	0.672691	0.250906	0.986551	0.13575
0.204753	-0.39101	-0.43946	-0.36604	-0.32437	0.480956	-0.25967	0.282368	0.120232
0.020935	5.49E-06	2.35E-07	2.32E-05	0.000199	1.04E-08	0.003197	0.001298	0.178161
-0.0464	0.19866	-0.24343	0.086546	-0.2201	-0.22183	-0.29479	-0.41536	-0.42428
0.604422	0.025155	0.005819	0.333294	0.012903	0.012193	0.000767	1.2E-06	6.66E-07
0.225934	-0.31857	-0.6422	-0.40156	-0.51672	0.378401	-0.54966	0.049279	-0.04637
0.010646	0.000262	4.04E-16	2.88E-06	5E-10	1.15E-05	2.19E-11	0.582193	0.604701
0.148847	-0.29402	-0.51209	-0.42844	-0.46068	0.387898	-0.35616	0.165476	0.014164
0.094889	0.000793	7.57E-10	5.03E-07	5.04E-08	6.61E-06	3.96E-05	0.062996	0.874415
0.210236	-0.30456	-0.33912	-0.17682	-0.20602	0.42998	-0.31499	0.189546	0.243354
0.017674	0.000499	9.61E-05	0.046738	0.020136	4.53E-07	0.00031	0.032815	0.005835
-0.35091	0.01956	0.063184	-0.24603	0.306202	0.22281	0.341211	-0.03964	-0.14843
5.24E-05	0.827217	0.48037	0.005301	0.000463	0.011807	8.65E-05	0.65816	0.095819
-0.18682	0.120688	0.00806	-0.33785	0.240777	0.035104	0.067453	-0.20237	-0.19565
0.035458	0.176509	0.928335	0.000103	0.006395	0.695198	0.451147	0.022509	0.027497
-0.01637	-0.14898	-0.15017	-0.41394	0.039071	0.201803	-0.13021	0.042411	0.087086
0.85506	0.0946	0.091949	1.31E-06	0.662752	0.022895	0.144537	0.635905	0.330276
-0.39453	0.028841	0.402985	-0.02979	0.218128	-0.05244	0.361396	-0.03478	0.015847
4.44E-06	0.747548	2.64E-06	0.739541	0.013755	0.558155	2.99E-05	0.697868	0.85964
-0.34743	0.232959	0.601828	0.248791	0.592514	-0.18001	0.717606	-0.00833	-0.10934
6.28E-05	0.008396	7.25E-14	0.004795	2.17E-13	0.04286	2.26E-21	0.925915	0.221083
-0.54314	0.570468	0.846185	0.647326	0.846043	-0.64773	0.789235	-0.50617	-0.49585
4.19E-11	2.53E-12	5.67E-36	1.98E-16	5.98E-36	1.87E-16	3.04E-28	1.27E-09	3.07E-09
-0.50906	0.394922	0.697862	0.160988	0.80932	-0.32124	0.749677	-0.291	-0.3052
9.89E-10	4.33E-06	7.71E-20	0.070589	1.14E-30	0.000231	3.68E-24	0.000903	0.000485
0.194712	0.159909	0.167609	0.137303	-0.01547	-0.31494	-0.01367	-0.09637	0.017544
0.028262	0.072519	0.059629	0.123723	0.862931	0.000311	0.878759	0.281103	0.844787
0.0615	0.206015	0.286501	0.498354	0.093418	-0.43501	0.191617	-0.00398	0.070138
0.492163	0.020142	0.001093	2.49E-09	0.296189	3.21E-07	0.03092	0.964555	0.433296
0.556528	-0.32282	-0.44291	-0.00101	-0.61885	0.174194	-0.5989	0.337932	0.457976
1.09E-11	0.000214	1.85E-07	0.991019	8.9E-15	0.050152	1.03E-13	0.000102	6.17E-08
0.287236	-0.03404	-0.0926	0.253829	-0.31388	-0.12786	-0.28633	0.153438	0.309674
0.001059	0.703997	0.300478	0.003983	0.000326	0.151984	0.0011	0.08502	0.000396
0.030696	0.36518	0.063136	0.216209	-0.008	-0.15129	0.142981	-0.14064	-0.33765
0.731912	2.43E-05	0.480698	0.01463	0.928829	0.089527	0.108795	0.114774	0.000104

LRFN3	LRFN4	LRRC14	LRRC24	LRRC26	LRRC29	LRRC45	LRRC57	LRRC75B
black	black	black	black	black	black	black	black	black
-0.21265	-0.31609	-0.29615	-0.28165	-0.27024	0.034544	-0.34433	-0.04521	-0.26653
0.016384	0.000294	0.000723	0.001337	0.002121	0.699827	7.37E-05	0.613752	0.002454
-0.24606	-0.3417	-0.30545	-0.25159	-0.24543	-0.01732	-0.42308	-0.04413	-0.23827
0.005294	8.44E-05	0.000479	0.004326	0.005417	0.846775	7.21E-07	0.622226	0.006985
0.06508	0.096445	0.083275	0.030704	0.086807	-0.21718	0.220194	0.343013	0.42159
0.46727	0.280745	0.351959	0.731838	0.331836	0.014183	0.012863	7.89E-05	7.97E-07
-0.37391	-0.42147	-0.35448	-0.42185	-0.25013	-0.40527	-0.1654	0.497521	0.027107
1.49E-05	8.03E-07	4.34E-05	7.83E-07	0.004565	2.28E-06	0.063116	2.67E-09	0.762256
-0.44967	-0.31346	-0.48598	-0.33467	-0.05968	0.314379	-0.1985	0.017071	0.149238
1.14E-07	0.000333	6.96E-09	0.00012	0.505077	0.000319	0.025273	0.848922	0.094014
-0.74913	-0.71132	-0.75513	-0.72843	-0.37474	-0.1575	-0.42282	0.436361	-0.12416
4.14E-24	7.18E-21	1.12E-24	2.87E-22	1.42E-05	0.076991	7.34E-07	2.92E-07	0.164298
-0.52712	-0.64024	-0.59984	-0.54176	-0.36277	-0.18903	-0.4895	0.229686	-0.12065
1.93E-10	5.3E-16	9.18E-14	4.79E-11	2.77E-05	0.033302	5.22E-09	0.009386	0.176655
-0.50363	-0.29748	-0.32574	-0.47477	-0.22335	-0.08796	-0.04824	-0.00665	-0.26429
1.59E-09	0.000683	0.000186	1.71E-08	0.011597	0.325452	0.590152	0.940862	0.002678
0.138941	-0.01316	-0.02766	-0.00932	-0.1518	0.014876	0.121322	-0.08227	-0.04754
0.119265	0.883225	0.757585	0.917204	0.088438	0.868157	0.174232	0.357801	0.595592
-0.24163	-0.23045	-0.30498	-0.35517	-0.32881	0.349465	-0.01328	-0.30859	-0.34455
0.006205	0.009146	0.00049	4.18E-05	0.00016	5.65E-05	0.882234	0.000416	7.29E-05
-0.28137	-0.32062	-0.30184	-0.41452	-0.39482	0.094786	-0.13137	-0.00919	-0.33309
0.001353	0.000238	0.000563	1.27E-06	4.36E-06	0.28914	0.140975	0.918322	0.00013
0.57659	0.359895	0.467613	0.689949	0.139536	0.013568	0.050226	-0.20024	0.200369
1.3E-12	3.24E-05	2.98E-08	2.93E-19	0.117674	0.879663	0.574951	0.023999	0.023904
0.725152	0.663992	0.726735	0.65565	0.316274	0.034707	0.602989	-0.21395	0.416676
5.42E-22	1.76E-17	3.99E-22	6.02E-17	0.000292	0.698477	6.31E-14	0.015723	1.1E-06
0.71398	0.852186	0.71763	0.713409	0.462221	0.618192	0.787994	-0.51849	0.491805
4.42E-21	5.73E-37	2.25E-21	4.91E-21	4.49E-08	9.68E-15	4.2E-28	4.26E-10	4.31E-09
0.546579	0.571527	0.520175	0.44898	0.154113	0.471568	0.618327	-0.58771	0.110715
2.98E-11	2.26E-12	3.66E-10	1.19E-07	0.083641	2.19E-08	9.51E-15	3.76E-13	0.215283
0.063088	0.115512	0.145424	0.147269	0.137913	0.119035	-0.04902	-0.4126	-0.24139
0.481038	0.195937	0.10282	0.098483	0.122049	0.182554	0.584196	1.43E-06	0.006257
0.520697	0.55032	0.585312	0.598809	0.453057	-0.14368	0.227317	-0.04054	0.344161
3.49E-10	2.05E-11	4.94E-13	1.04E-13	8.87E-08	0.107068	0.010166	0.650863	7.44E-05
-0.24754	-0.27651	-0.18946	-0.18254	-0.01672	-0.45733	-0.44592	0.478125	-0.1748
0.005017	0.00165	0.032895	0.039966	0.852003	6.47E-08	1.49E-07	1.31E-08	0.04935
0.093277	0.069072	0.121726	0.182961	0.174616	-0.20885	-0.27096	0.071118	-0.10852
0.296918	0.440332	0.172792	0.039505	0.049591	0.018452	0.002061	0.426877	0.224555
0.172149	0.141727	0.08436	0.295144	0.295824	-0.09592	0.071223	0.112779	0.450521
0.052952	0.111965	0.345695	0.000756	0.000734	0.283404	0.426194	0.206799	1.07E-07

LRRC8C	LRWD1	LSM4	LSM7	LYPLA2	LZTFL1	MACROD1	MAD1L1	MAF1
black	black	black	black	black	black	black	black	black
0.215396	-0.51188	-0.19564	-0.39595	-0.22465	0.123797	-0.28978	-0.21462	-0.10728
0.015015	7.71E-10	0.027503	4.07E-06	0.011111	0.165544	0.000951	0.015389	0.229954
0.153501	-0.48567	-0.07661	-0.43576	-0.24317	0.108702	-0.23762	-0.25426	-0.1206
0.084889	7.14E-09	0.391965	3.05E-07	0.005874	0.223792	0.007146	0.003919	0.176844
-0.28887	0.326896	0.190295	0.383702	0.212946	-0.14529	0.187052	0.177911	-0.08484
0.000989	0.000176	0.032119	8.47E-06	0.01623	0.103131	0.035226	0.045379	0.342966
0.026836	-0.0218	-0.28961	-0.07224	-0.26889	0.263013	-0.2584	-0.3008	-0.49365
0.764562	0.807789	0.000958	0.419635	0.002238	0.002813	0.003354	0.00059	3.7E-09
-0.18738	-0.03861	0.464015	0.193082	0.17134	0.150762	0.201421	-0.28138	0.34804
0.034905	0.666453	3.92E-08	0.029636	0.054094	0.090668	0.02316	0.001352	6.08E-05
0.119171	-0.23819	-0.14541	-0.17537	-0.28376	0.460803	-0.2999	-0.59211	-0.34752
0.182048	0.007003	0.102855	0.048599	0.001225	4.99E-08	0.000614	2.27E-13	6.25E-05
0.030607	-0.22051	-0.09922	-0.26547	-0.26499	0.306144	-0.29224	-0.52817	-0.26542
0.732659	0.012731	0.267088	0.002558	0.002606	0.000465	0.000856	1.75E-10	0.002563
0.132753	-0.06337	-0.22486	-0.23502	-0.1663	0.54779	-0.39329	-0.51007	-0.20484
0.136791	0.479065	0.011034	0.00782	0.061675	2.64E-11	4.78E-06	9.04E-10	0.02088
-0.00142	0.075461	-0.20537	0.223987	0.170081	-0.18834	-0.26251	0.201815	-0.12882
0.987392	0.399118	0.020543	0.011358	0.055912	0.033964	0.002868	0.022887	0.148902
0.291856	-0.2294	-0.24427	-0.02332	0.120591	-0.1222	-0.42241	-0.07921	0.065852
0.000871	0.009478	0.005647	0.794648	0.17686	0.171126	7.54E-07	0.376047	0.461992
0.306239	-0.24958	-0.40229	-0.16789	-0.10546	0.095933	-0.45342	-0.15587	-0.18767
0.000463	0.004658	2.75E-06	0.059192	0.237987	0.283319	8.64E-08	0.080128	0.034617
-0.33669	0.226001	-0.02008	0.139103	0.066602	-0.32767	0.168951	0.49011	0.184205
0.000109	0.010623	0.822739	0.118829	0.456895	0.00017	0.057587	4.96E-09	0.03816
-0.36224	0.450259	0.121815	0.455479	0.410848	-0.60939	0.304193	0.676784	0.243833
2.85E-05	1.09E-07	0.172473	7.43E-08	1.6E-06	2.9E-14	0.000507	2.46E-18	0.005736
-0.49368	0.668445	0.643485	0.819341	0.755822	-0.69587	0.708233	0.797666	0.811353
3.69E-09	8.97E-18	3.39E-16	5.44E-32	9.62E-25	1.08E-19	1.25E-20	3.14E-29	6.24E-31
-0.12842	0.292944	0.082423	0.459063	0.528741	-0.69399	0.122028	0.63437	0.465011
0.150175	0.000831	0.356922	5.69E-08	1.66E-10	1.49E-19	0.171718	1.17E-15	3.63E-08
0.184958	-0.22011	0.066174	-0.22842	-0.08423	-0.22569	0.016067	0.061139	0.187273
0.037364	0.0129	0.459801	0.009796	0.346451	0.010735	0.857712	0.494716	0.035006
-0.26083	0.339393	0.390462	0.222886	0.159246	-0.27848	0.511166	0.411217	0.253153
0.003058	9.48E-05	5.67E-06	0.011778	0.073729	0.001523	8.21E-10	1.57E-06	0.004084
0.240647	-0.21872	-0.13184	-0.46018	-0.51126	0.571992	-0.03183	-0.40651	-0.43503
0.006424	0.013495	0.139523	5.23E-08	8.15E-10	2.15E-12	0.72237	2.11E-06	3.21E-07
0.083887	-0.12542	0.104434	-0.32811	-0.24828	0.295757	0.132784	-0.13055	-0.04219
0.348416	0.160023	0.242618	0.000166	0.004884	0.000736	0.136698	0.143492	0.637701
-0.27954	0.016477	0.273573	0.281428	0.232374	-0.26741	0.361517	0.191159	0.197903
0.001459	0.854117	0.001857	0.00135	0.008566	0.002371	2.97E-05	0.03133	0.025727

MAGEF1	MAGIX	MAN1B1	MAP1S	MAP2K2	MAPK12	MAPK14	MBD3	MBLAC1
black	black	black	black	black	black	black	black	black
-0.2672	0.007089	0.111793	-0.1318	-0.1482	-0.28925	0.046578	-0.24594	-0.13892
0.002391	0.936952	0.210822	0.139664	0.096341	0.000973	0.603063	0.005318	0.119308
-0.20143	0.044253	0.084423	-0.12752	-0.16124	-0.16942	0.071612	-0.2626	-0.03558
0.023152	0.621298	0.345335	0.153095	0.070146	0.056884	0.423666	0.002858	0.691299
0.429784	-0.27464	-0.07606	-0.19195	-0.11025	0.336101	0.125512	0.036555	0.003457
4.59E-07	0.001779	0.395406	0.030622	0.217218	0.000112	0.159716	0.683263	0.969229
-0.03572	-0.57021	-0.19876	-0.62321	-0.61374	-0.15324	0.475052	-0.45388	-0.33608
0.690094	2.6E-12	0.025078	5.1E-15	1.69E-14	0.085433	1.67E-08	8.36E-08	0.000112
-0.00291	0.193698	0.301593	-0.41436	-0.16276	0.099487	0.150875	-0.22697	0.189457
0.974119	0.02911	0.000569	1.28E-06	0.067508	0.265779	0.090424	0.010283	0.032898
-0.23	-0.42242	0.00349	-0.87238	-0.76224	-0.29428	0.571102	-0.66474	-0.28816
0.009287	7.54E-07	0.968935	1.14E-40	2.27E-25	0.000784	2.37E-12	1.57E-17	0.001019
-0.35816	-0.29419	0.152054	-0.62651	-0.552	-0.21834	0.425547	-0.53232	-0.38016
3.56E-05	0.000787	0.087905	3.32E-15	1.73E-11	0.013662	6.12E-07	1.19E-10	1.04E-05
-0.23053	0.034334	-0.02214	-0.4445	-0.42457	-0.40657	0.072121	-0.29644	-0.23727
0.009121	0.701563	0.804858	1.65E-07	6.53E-07	2.11E-06	0.42037	0.000714	0.007232
-0.09909	-0.30901	0.348431	-0.04315	-0.02109	-0.17874	0.281128	0.139513	-0.30332
0.267689	0.000408	5.96E-05	0.630012	0.813923	0.044369	0.001366	0.117735	0.000527
-0.40454	-0.09226	0.377975	-0.17494	-0.11993	-0.48997	0.049429	-0.0016	-0.38914
2.39E-06	0.302263	1.18E-05	0.049166	0.179248	5.02E-09	0.581041	0.985792	6.14E-06
-0.33285	-0.29361	0.145724	-0.29967	-0.29915	-0.56359	0.231675	-0.20096	-0.39202
0.000132	0.000808	0.102106	0.00062	0.000634	5.25E-12	0.008773	0.023482	5.17E-06
0.12672	-0.0431	0.070342	0.497987	0.435488	0.063526	0.017117	0.274957	0.192784
0.155704	0.630431	0.431958	2.57E-09	3.11E-07	0.47799	0.848518	0.001757	0.029893
0.418735	0.133314	0.032146	0.601161	0.574776	0.403161	-0.38716	0.639469	0.129224
9.62E-07	0.135123	0.719759	7.85E-14	1.59E-12	2.61E-06	6.91E-06	5.89E-16	0.147629
0.470209	0.610069	0.329237	0.76799	0.898155	0.532176	-0.57399	0.918497	0.567154
2.44E-08	2.67E-14	0.000157	5.98E-26	1.95E-46	1.2E-10	1.73E-12	3.33E-52	3.61E-12
0.100804	0.287185	0.351461	0.590608	0.61894	0.055193	-0.47292	0.728102	0.023018
0.259472	0.001062	5.09E-05	2.7E-13	8.8E-15	0.53769	1.97E-08	3.06E-22	0.797281
-0.10138	0.315295	-0.17216	0.319079	0.268097	-0.04375	-0.48655	0.141944	0.0253
0.256719	0.000305	0.052934	0.000256	0.002308	0.625239	6.64E-09	0.111411	0.777683
0.458621	0.274376	-0.31126	0.545292	0.504174	0.552115	-0.47679	0.37268	0.369307
5.88E-08	0.001799	0.000368	3.39E-11	1.51E-09	1.71E-11	1.46E-08	1.6E-05	1.93E-05
-0.03929	-0.18364	-0.5087	-0.21588	-0.35751	-0.01116	0.263839	-0.47733	0.085172
0.660983	0.038765	1.02E-09	0.014784	3.69E-05	0.900918	0.002725	1.39E-08	0.341056
0.019543	0.207674	-0.28432	0.252541	0.138584	0.157432	-0.11468	-0.091	0.266983
0.827363	0.019138	0.001197	0.004177	0.120226	0.077117	0.199198	0.308924	0.002411
0.377718	0.052651	-0.02688	0.044725	0.112642	0.481183	-0.14236	0.063026	0.032883
1.2E-05	0.556611	0.764228	0.617573	0.207354	1.03E-08	0.110353	0.48147	0.713609

MCAT	MED16	MED25	MED4	MEGF6	MESP1	METRN	MFSD10	MFSD3
black	black	black	black	black	black	black	black	black
-0.19065	-0.27221	-0.27587	0.070836	-0.08899	-0.29577	-0.25242	-0.29292	-0.20354
0.031791	0.001961	0.001694	0.42872	0.319768	0.000735	0.004196	0.000832	0.021725
-0.16349	-0.29198	-0.28246	0.140006	-0.15323	-0.1711	-0.24581	-0.35615	-0.18299
0.06627	0.000866	0.001293	0.116431	0.085443	0.054443	0.005343	3.97E-05	0.039471
0.087596	0.031359	0.017235	0.176027	-0.16577	0.066096	-0.07292	0.144328	-0.23391
0.327443	0.726347	0.847491	0.047749	0.062529	0.460327	0.41522	0.105468	0.008126
-0.23143	-0.44375	-0.43126	0.420565	-0.35917	-0.32551	-0.46197	-0.24997	-0.59329
0.008845	1.74E-07	4.15E-07	8.53E-07	3.37E-05	0.000188	4.57E-08	0.004593	1.98E-13
0.326634	-0.37899	-0.43947	0.284235	-0.22652	0.242008	0.042737	-0.20773	0.038281
0.000178	1.11E-05	2.35E-07	0.001201	0.010439	0.006122	0.633306	0.019108	0.669162
-0.13643	-0.7635	-0.79049	0.57644	-0.48589	-0.27131	-0.457	-0.50387	-0.54764
0.126145	1.7E-25	2.18E-28	1.33E-12	7.01E-09	0.002033	6.63E-08	1.55E-09	2.68E-11
-0.18091	-0.57653	-0.54659	0.468689	-0.40642	-0.23537	-0.41024	-0.40373	-0.48935
0.041806	1.31E-12	2.98E-11	2.74E-08	2.13E-06	0.007726	1.67E-06	2.52E-06	5.28E-09
-0.10433	-0.39402	-0.2945	-0.01418	0.072039	-0.24338	-0.00161	-0.23312	-0.27301
0.243107	4.58E-06	0.000777	0.874277	0.4209	0.005829	0.985698	0.00835	0.0019
-0.246	0.104357	-0.02336	0.121553	0.085462	-0.39483	-0.11426	0.361917	-0.23595
0.005305	0.242964	0.794346	0.173405	0.33941	4.36E-06	0.200874	2.9E-05	0.007572
-0.36225	-0.15274	-0.22565	-0.00433	0.114954	-0.5367	0.062604	0.129846	-0.13803
2.85E-05	0.086474	0.010746	0.96144	0.198119	7.82E-11	0.484417	0.145671	0.121742
-0.34408	-0.28493	-0.32475	0.040187	-0.01285	-0.56917	-0.20253	-0.09689	-0.30914
7.47E-05	0.001167	0.000195	0.653728	0.886019	2.91E-12	0.022399	0.278535	0.000406
0.162534	0.441017	0.384553	-0.05601	0.263919	-0.01059	-0.09042	0.359662	0.053576
0.067893	2.11E-07	8.05E-06	0.531669	0.002716	0.905919	0.312046	3.28E-05	0.549686
0.047966	0.719937	0.740074	-0.41479	0.399158	0.105139	0.314989	0.701336	0.238537
0.592296	1.46E-21	2.78E-23	1.24E-06	3.34E-06	0.239435	0.00031	4.23E-20	0.00692
0.443544	0.854844	0.785086	-0.58831	0.448001	0.565808	0.798393	0.8745	0.803154
1.76E-07	2.01E-37	8.93E-28	3.52E-13	1.28E-07	4.16E-12	2.57E-29	4.27E-41	6.78E-30
-0.09544	0.656338	0.59986	-0.52787	0.535723	-0.0902	0.557323	0.797855	0.401215
0.285826	5.45E-17	9.16E-14	1.8E-10	8.58E-11	0.313227	1.01E-11	2.98E-29	2.94E-06
-0.08666	0.104014	0.167783	-0.21494	0.137428	0.059405	0.288184	-0.09536	0.267204
0.332662	0.244526	0.059361	0.015238	0.12338	0.507056	0.001018	0.286237	0.00239
0.344517	0.490025	0.56848	-0.27473	0.133073	0.48525	0.307656	0.160626	0.366481
7.3E-05	5E-09	3.13E-12	0.001773	0.135839	7.39E-09	0.000434	0.071231	2.26E-05
0.071623	-0.3496	-0.29096	0.302201	-0.38619	0.190999	-0.3782	-0.66494	-0.15978
0.423598	5.61E-05	0.000905	0.000554	7.31E-06	0.031475	1.17E-05	1.52E-17	0.072759
0.239676	0.034857	0.104335	-0.04113	-0.0596	0.371145	-0.04567	-0.35735	0.206837
0.006648	0.697236	0.243067	0.64611	0.505648	1.74E-05	0.610144	3.72E-05	0.019639
0.249441	0.118449	0.12469	0.193414	0.001327	0.183574	-0.03795	0.153434	-0.04513
0.004682	0.184732	0.162488	0.029352	0.988183	0.038837	0.671891	0.085027	0.614414

MIB2	MIEN1	MIIP	MKNK2	MLST8	MMGT1	MOB2	MORC3	MOSPD3
black	black	black	black	black	black	black	black	black
-0.25507	0.159072	-0.24077	-0.14741	-0.09614	0.245175	0.071915	0.073615	-0.38179
0.003802	0.074048	0.006396	0.098158	0.28226	0.005466	0.421702	0.410782	9.47E-06
-0.29546	0.243691	-0.24458	-0.20075	-0.01761	0.303829	0.149367	0.027035	-0.32909
0.000745	0.005765	0.005584	0.02363	0.844215	0.000515	0.093727	0.762875	0.000158
-0.08302	-0.35427	0.214955	-0.08282	-0.11248	0.03595	-0.30219	0.277166	0.338395
0.353439	4.38E-05	0.015228	0.35458	0.208023	0.688227	0.000554	0.001607	9.97E-05
-0.48917	-0.67179	-0.28075	-0.34535	-0.60481	0.329942	-0.55211	0.658233	-0.11684
5.36E-09	5.37E-18	0.001388	6.99E-05	5.06E-14	0.000152	1.71E-11	4.13E-17	0.190796
-0.2548	0.5423	0.20812	-0.34883	0.289726	0.154135	0.499727	-0.01582	0.133609
0.003842	4.54E-11	0.018876	5.84E-05	0.000953	0.083595	2.21E-09	0.859867	0.134256
-0.68426	-0.28459	-0.31946	-0.57132	-0.46213	0.509	-0.19768	0.614461	-0.23792
7.45E-19	0.001183	0.000251	2.31E-12	4.52E-08	9.94E-10	0.025894	1.55E-14	0.007071
-0.58746	-0.16904	-0.35609	-0.36371	-0.28908	0.254454	-0.10486	0.392333	-0.25301
3.87E-13	0.057454	3.98E-05	2.63E-05	0.00098	0.003891	0.24067	5.07E-06	0.004105
-0.16293	-0.18177	-0.28534	-0.25811	-0.22235	0.088546	-0.22231	0.066252	-0.33392
0.067217	0.04083	0.001147	0.003391	0.011986	0.322204	0.012004	0.459268	0.000125
0.007011	-0.39315	-0.07735	0.297724	-0.37858	-0.0256	-0.37272	0.097767	0.133973
0.937643	4.82E-06	0.387382	0.000675	1.14E-05	0.775132	1.59E-05	0.274171	0.133186
-0.07347	0.00776	-0.06142	0.139773	-0.29986	0.000906	-0.03752	-0.01057	-0.14474
0.411724	0.930998	0.492757	0.117046	0.000615	0.991936	0.675399	0.906102	0.104472
-0.2448	-0.30906	-0.19235	-0.07417	-0.44412	0.13676	-0.25953	0.189508	-0.23982
0.005541	0.000407	0.030271	0.407235	1.69E-07	0.125232	0.003214	0.03285	0.006614
0.176306	0.148035	0.15128	0.250045	0.059441	-0.27752	-0.15857	-0.35185	0.351063
0.047391	0.096725	0.089551	0.00458	0.506799	0.001584	0.074972	4.98E-05	5.19E-05
0.566354	-0.11152	0.42042	0.583965	0.164813	-0.50164	-0.14269	-0.3304	0.479696
3.93E-12	0.21196	8.61E-07	5.75E-13	0.064074	1.88E-09	0.109513	0.000148	1.16E-08
0.874146	0.554033	0.836026	0.681985	0.741451	-0.62667	0.530485	-0.72385	0.786618
5.03E-41	1.41E-11	2.21E-34	1.08E-18	2.09E-23	3.25E-15	1.41E-10	6.96E-22	6.01E-28
0.654761	0.180164	0.44692	0.710208	0.18693	-0.53822	0.115713	-0.54041	0.405687
6.85E-17	0.042674	1.39E-07	8.79E-21	0.035347	6.75E-11	0.195156	5.46E-11	2.23E-06
0.188938	0.537231	0.002779	-0.03023	0.233954	-0.13997	0.363124	-0.36158	-0.19939
0.03339	7.43E-11	0.975261	0.735848	0.008114	0.116513	2.72E-05	2.96E-05	0.024612
0.371897	0.293498	0.361135	0.083363	0.503323	-0.31089	0.184616	-0.34355	0.299224
1.67E-05	0.000811	3.03E-05	0.351448	1.63E-09	0.000374	0.037724	7.67E-05	0.000632
-0.36903	-0.21439	-0.39781	-0.4976	-0.07154	0.421559	-0.09881	0.420812	-0.39418
1.96E-05	0.015505	3.63E-06	2.65E-09	0.424109	7.98E-07	0.26907	8.39E-07	4.53E-06
0.014263	0.252959	-0.13322	-0.16827	0.344643	0.064397	0.229028	-0.13319	-0.22771
0.873544	0.004113	0.135413	0.058614	7.25E-05	0.471962	0.009597	0.13549	0.010033
0.00692	0.146037	0.284359	-0.01531	0.142804	-0.07345	0.022721	0.138201	0.224236
0.938451	0.101364	0.001195	0.864345	0.109239	0.411823	0.799842	0.121263	0.011265

MPG	MPST	MPV17L2	MROH6	MRPL28	MRPL34	MRPL4	MRPL41	MRPL55
black	black	black	black	black	black	black	black	black
-0.14207	-0.27204	-0.0148	-0.17131	-0.06982	0.079312	-0.26063	-0.09947	0.008893
0.11108	0.001975	0.868863	0.054132	0.435386	0.375426	0.003082	0.26584	0.92096
-0.09297	-0.23836	0.109475	-0.21269	0.017942	0.105045	-0.21531	-0.074	0.068309
0.298528	0.006963	0.220495	0.016364	0.84131	0.239857	0.015057	0.408308	0.445413
-0.16349	0.196012	0.149794	0.239411	-0.23983	-0.39196	0.068285	-0.36243	-0.22801
0.066275	0.027205	0.092782	0.00671	0.006612	5.18E-06	0.445577	2.82E-05	0.00993
-0.50307	-0.24297	-0.23262	-0.08098	-0.65958	-0.58993	-0.4252	-0.71103	-0.4548
1.66E-09	0.005915	0.008494	0.365456	3.39E-17	2.92E-13	6.26E-07	7.57E-21	7.81E-08
0.331577	0.232121	0.264701	-0.15156	0.454504	0.398124	-0.03062	0.194105	0.605133
0.00014	0.00864	0.002635	0.088949	7.98E-08	3.56E-06	0.732534	0.028767	4.87E-14
-0.30621	-0.25847	-0.18756	-0.33157	-0.34544	-0.24143	-0.55709	-0.53129	-0.07046
0.000463	0.003346	0.034723	0.00014	6.96E-05	0.00625	1.03E-11	1.31E-10	0.431155
-0.24334	-0.30729	0.063157	-0.16928	-0.23257	-0.34499	-0.48018	-0.47002	-0.05835
0.005838	0.000441	0.480554	0.05709	0.008508	7.13E-05	1.11E-08	2.47E-08	0.514647
-0.34179	-0.33869	-0.36641	-0.26805	-0.18001	-0.11976	-0.42822	-0.14226	-0.01396
8.4E-05	9.83E-05	2.27E-05	0.002313	0.042859	0.17988	5.11E-07	0.110616	0.876229
0.0012	-0.06626	-0.08714	0.263528	-0.38589	-0.15386	0.000412	-0.28949	-0.20088
0.989316	0.459237	0.329969	0.002758	7.45E-06	0.084145	0.996335	0.000963	0.023539
0.021263	-0.07774	-0.08653	0.042577	-0.12404	0.176923	-0.18016	-0.01009	0.129123
0.812441	0.384991	0.333364	0.634578	0.164692	0.046609	0.042683	0.910364	0.147948
-0.21216	-0.24783	-0.22486	-0.05617	-0.35788	-0.09865	-0.3168	-0.27729	-0.10179
0.016639	0.004966	0.011033	0.530489	3.61E-05	0.269861	0.000285	0.001599	0.254824
0.326182	0.210924	0.148581	0.328207	-0.10328	-0.00905	0.397508	0.099422	-0.11986
0.000182	0.017297	0.095489	0.000165	0.247911	0.919557	3.7E-06	0.26609	0.179499
0.092779	0.37246	0.267859	0.638564	-0.06164	-0.12743	0.508146	0.115721	-0.14084
0.299516	1.62E-05	0.00233	6.66E-16	0.491179	0.153384	1.07E-09	0.195122	0.114252
0.76967	0.807768	0.535126	0.620578	0.660063	0.679723	0.879494	0.793234	0.641417
4.02E-26	1.79E-30	9.09E-11	7.15E-15	3.15E-17	1.55E-18	3.96E-42	1.05E-28	4.51E-16
0.354578	0.426203	0.24487	0.587749	0.153334	0.330966	0.479225	0.425106	0.226162
4.31E-05	5.85E-07	0.005526	3.75E-13	0.085235	0.000144	1.2E-08	6.3E-07	0.010566
0.074216	0.085309	0.142275	-0.14258	0.326259	0.297239	0.06456	0.436146	0.124756
0.406968	0.340279	0.11057	0.109802	0.000182	0.00069	0.47084	2.97E-07	0.162264
0.157578	0.336397	0.287878	0.1057	0.334669	0.012027	0.455475	0.332609	-0.00858
0.07684	0.00011	0.001031	0.236921	0.00012	0.893246	7.43E-08	0.000133	0.923706
-0.27561	-0.38727	-0.29715	-0.53825	-0.07152	-0.24436	-0.29053	-0.22964	-0.29347
0.001711	6.86E-06	0.000692	6.73E-11	0.424288	0.005629	0.000921	0.009402	0.000812
0.043543	-0.18193	-0.05872	-0.37085	0.335138	0.083047	0.057313	0.224089	0.001572
0.626908	0.040647	0.511952	1.77E-05	0.000117	0.353283	0.52216	0.011319	0.986008
0.050852	0.297948	0.240029	0.374225	0.040973	-0.21847	0.153743	-0.02813	-0.07392
0.570192	0.000669	0.006566	1.46E-05	0.647403	0.013603	0.084393	0.75353	0.408812

MRPS12	MRPS24	MRPS26	MRPS27	MRPS34	MTG2	MTHFS	MTM1	MUS81
black	black	black	black	black	black	black	black	black
-0.14278	-0.36132	-0.29497	0.093423	-0.25115	-0.14397	-0.22131	0.071921	-0.27403
0.109298	3E-05	0.000761	0.296163	0.004397	0.106347	0.012404	0.421663	0.001824
-0.08194	-0.33416	-0.23408	0.184443	-0.2158	-0.20177	-0.28079	0.029749	-0.29703
0.35976	0.000123	0.008079	0.037906	0.014821	0.022918	0.001386	0.739878	0.000696
0.073989	0.272596	0.060706	0.076592	-0.04677	0.017919	-0.1052	-0.07496	0.325747
0.408406	0.001932	0.497781	0.392072	0.601534	0.841516	0.239144	0.402264	0.000186
-0.26583	0.001475	-0.35069	0.20212	-0.46896	-0.03357	-0.33266	0.379751	-0.08936
0.002522	0.986872	5.3E-05	0.022677	2.68E-08	0.707887	0.000133	1.07E-05	0.317737
0.450492	0.151806	0.327698	0.324256	0.341166	-0.146	-0.0974	-0.13655	0.19816
1.07E-07	0.08843	0.000169	0.0002	8.67E-05	0.101444	0.275997	0.125821	0.025531
-0.08459	-0.05025	-0.27323	0.368733	-0.31187	-0.14588	-0.38056	0.396635	-0.1741
0.344351	0.574747	0.001883	1.99E-05	0.000358	0.101724	1.02E-05	3.9E-06	0.050284
-0.10479	-0.11447	-0.3221	0.384216	-0.33032	-0.036	-0.48441	0.235592	-0.18722
0.241	0.200043	0.000222	8.22E-06	0.000149	0.687801	7.91E-09	0.007667	0.035059
-0.26427	-0.11416	-0.21133	0.077515	-0.15534	0.114237	-0.12233	0.56391	-0.29468
0.00268	0.201267	0.017079	0.386373	0.081171	0.20095	0.170637	5.08E-12	0.000771
0.062178	-0.00991	-0.28162	-0.29806	-0.17177	0.21621	0.159432	-0.14066	0.075316
0.487399	0.911924	0.001339	0.000665	0.05348	0.014629	0.073387	0.114727	0.400026
0.012161	-0.25079	-0.28725	-0.37907	-0.03715	0.271877	0.20021	0.134963	-0.04352
0.892063	0.004457	0.001059	1.11E-05	0.678352	0.001988	0.024018	0.130319	0.627065
-0.18329	-0.28082	-0.36951	-0.17477	-0.27677	0.224472	0.014666	0.411331	-0.14026
0.039143	0.001384	1.91E-05	0.04938	0.001633	0.011177	0.870001	1.56E-06	0.115753
0.064248	-0.0251	0.187182	0.060677	0.007884	-0.04025	0.167233	-0.33807	0.109291
0.472994	0.779416	0.035097	0.497986	0.9299	0.653205	0.060212	0.000101	0.22128
0.086178	0.206063	0.182721	-0.38817	0.168076	0.31403	0.324854	-0.36858	0.445615
0.335365	0.020112	0.039769	6.5E-06	0.058911	0.000324	0.000194	2.01E-05	1.52E-07
0.704212	0.490788	0.737463	-0.5158	0.860491	0.424245	0.746575	-0.5907	0.740868
2.56E-20	4.69E-09	4.74E-23	5.44E-10	2.02E-38	6.68E-07	7.15E-24	2.67E-13	2.36E-23
0.244891	0.0687	0.155755	-0.71203	0.388258	0.505899	0.631077	-0.30549	0.405611
0.005522	0.442804	0.080359	6.32E-21	6.47E-06	1.3E-09	1.82E-15	0.000478	2.24E-06
-0.09347	-0.27589	0.062259	-0.15205	0.174399	-0.09535	0.077238	-0.03211	-0.16435
0.295914	0.001692	0.486826	0.087906	0.049879	0.286247	0.388075	0.720045	0.064833
0.089081	0.179772	0.403408	0.060027	0.271225	-0.18184	0.041003	-0.27245	0.214358
0.319275	0.043135	2.57E-06	0.502612	0.00204	0.040747	0.647163	0.001943	0.01552
-0.28277	-0.06264	-0.09736	0.494247	-0.30245	-0.45925	-0.4391	0.267484	-0.43111
0.001277	0.484191	0.276199	3.52E-09	0.000548	5.61E-08	2.41E-07	0.002364	4.2E-07
-0.09302	-0.05092	0.146601	0.346245	0.001876	-0.37153	-0.2156	-0.0737	-0.27731
0.298268	0.569645	0.100038	6.68E-05	0.983296	1.7E-05	0.014916	0.410259	0.001598
0.098637	0.158101	0.148467	0.176019	0.065546	-0.29995	-0.18805	-0.51452	0.345925
0.269904	0.075853	0.095745	0.047759	0.464075	0.000612	0.034247	6.1E-10	6.79E-05

MVB12A	MYLK4	MZT2A	MZT2B	NAA10	NAA35	NAA60	NAPRT	NARFL
black	black	black	black	black	black	black	black	black
-0.25435	0.132487	-0.17656	-0.21306	0.017957	-0.06451	-0.15987	-0.0106	-0.20184
0.003906	0.137586	0.04707	0.016174	0.841184	0.47121	0.072586	0.905817	0.022873
-0.30001	0.156906	-0.19426	-0.20295	0.109387	-0.03196	-0.15556	-0.09482	-0.15707
0.000611	0.078123	0.028635	0.022118	0.220871	0.721276	0.080737	0.288976	0.077799
0.34608	-0.08978	-0.19126	-0.18785	-0.08304	0.350643	0.014218	-0.32474	-0.09633
6.74E-05	0.315458	0.031237	0.034436	0.353333	5.31E-05	0.873941	0.000195	0.281325
0.017239	0.174669	-0.56412	-0.58141	-0.45532	0.585581	-0.50099	-0.43696	-0.52782
0.847455	0.04952	4.97E-12	7.65E-13	7.51E-08	4.79E-13	1.99E-09	2.8E-07	1.81E-10
0.150949	-0.13485	0.139677	0.08841	0.534538	-0.05584	-0.24154	0.114016	0.267623
0.090263	0.130644	0.117301	0.322948	9.61E-11	0.532954	0.006224	0.201828	0.002352
-0.07078	0.187021	-0.46733	-0.53113	-0.19201	0.45649	-0.7148	-0.27886	-0.41922
0.42911	0.035257	3.04E-08	1.33E-10	0.03057	6.89E-08	3.8E-21	0.0015	9.32E-07
-0.12844	0.174939	-0.50907	-0.52468	-0.05423	0.327485	-0.47663	-0.21988	-0.35435
0.150118	0.049165	9.88E-10	2.42E-10	0.544817	0.000171	1.47E-08	0.012997	4.36E-05
-0.20033	0.165992	-0.2567	-0.24514	-0.15543	0.279724	-0.39274	-0.07076	-0.2368
0.02393	0.062167	0.003577	0.005473	0.080995	0.001448	4.94E-06	0.429211	0.007352
0.379475	-0.15737	-0.23467	-0.12382	-0.15077	-0.07517	0.041996	0.117769	-0.289
1.08E-05	0.077233	0.007915	0.165477	0.090649	0.40092	0.639212	0.187282	0.000983
0.242897	-0.12459	-0.11668	-0.06502	0.034769	-0.25142	-0.06268	0.376554	-0.1502
0.005931	0.16282	0.191422	0.467653	0.697961	0.004354	0.483906	1.28E-05	0.0919
0.055029	0.027037	-0.30391	-0.28149	-0.19425	0.031434	-0.23422	0.178263	-0.36191
0.5389	0.762852	0.000514	0.001346	0.028648	0.725721	0.008041	0.044947	2.91E-05
0.05969	-0.20275	-0.04981	0.206272	0.349862	-0.09966	0.437984	0.082695	0.09139
0.505015	0.022251	0.578097	0.019983	5.53E-05	0.264961	2.61E-07	0.355331	0.306842
0.456616	-0.28266	0.154935	0.266673	0.086528	-0.15202	0.666856	0.085476	0.206138
6.83E-08	0.001283	0.081985	0.002441	0.333398	0.087979	1.14E-17	0.339329	0.020065
0.785094	-0.58463	0.789791	0.879642	0.592626	-0.67724	0.802999	0.60813	0.846628
8.91E-28	5.33E-13	2.62E-28	3.69E-42	2.14E-13	2.29E-18	7.08E-30	3.38E-14	4.8E-36
0.622729	-0.42327	0.335327	0.468945	0.268582	-0.56207	0.685126	0.563335	0.328795
5.42E-15	7.12E-07	0.000116	2.69E-08	0.002265	6.16E-12	6.47E-19	5.4E-12	0.000161
-0.25837	-0.02118	0.210428	0.195698	0.196919	-0.25448	0.27313	0.121309	0.233226
0.003359	0.813191	0.017567	0.027457	0.026487	0.003887	0.001891	0.174277	0.008319
-0.04921	-0.16452	0.343872	0.324046	0.198142	-0.07081	0.482975	-0.13565	0.391416
0.582738	0.064561	7.55E-05	0.000202	0.025545	0.428891	8.88E-09	0.128367	5.36E-06
-0.67089	0.448688	-0.11326	-0.25468	-0.39901	0.39078	-0.46477	-0.44972	-0.19301
6.16E-18	1.22E-07	0.204864	0.003859	3.37E-06	5.56E-06	3.7E-08	1.13E-07	0.029697
-0.58926	0.224527	0.212802	0.128047	-0.00309	-0.01046	-0.01799	-0.16982	0.15557
3.15E-13	0.011156	0.016304	0.151384	0.972537	0.90711	0.840855	0.056301	0.080724
0.133654	-0.12651	-0.00131	-0.02093	0.272604	0.142524	0.196783	-0.21344	0.134396
0.134123	0.156397	0.988381	0.815293	0.001931	0.109942	0.026594	0.015978	0.131956

NARS	NAT14	NCAPH2	NCK2	NCLN	NDOR1	NDUFA3	NDUFAF3	NDUFB10
black	black	black	black	black	black	black	black	black
-0.00037	-0.32445	-0.17015	-0.12054	-0.23372	-0.16474	0.045076	0.137538	0.069285
0.996725	0.000198	0.055808	0.17704	0.00818	0.064188	0.614816	0.123077	0.438921
-0.00711	-0.24163	-0.13541	-0.18518	-0.27292	-0.17522	0.153484	0.157091	0.14154
0.936797	0.006205	0.129036	0.03713	0.001907	0.0488	0.084925	0.077767	0.112443
0.230632	0.09279	0.241137	-0.04886	0.093991	0.077346	-0.24648	-0.44155	-0.33048
0.00909	0.299463	0.006314	0.585435	0.293221	0.387409	0.005215	2.03E-07	0.000148
0.452105	-0.3414	-0.25804	-0.39265	-0.35373	-0.33754	-0.5936	-0.65521	-0.56126
9.52E-08	8.56E-05	0.003401	4.97E-06	4.51E-05	0.000104	1.91E-13	6.41E-17	6.7E-12
-0.29958	0.21849	0.188455	-0.28612	-0.36039	-0.39646	0.526504	0.396887	0.670609
0.000622	0.013595	0.033851	0.00111	3.16E-05	3.95E-06	2.05E-10	3.84E-06	6.44E-18
0.264095	-0.32784	-0.31844	-0.56709	-0.66314	-0.67999	-0.27425	-0.2946	-0.1055
0.002698	0.000168	0.000264	3.63E-12	2E-17	1.48E-18	0.001807	0.000773	0.237825
0.149362	-0.20134	-0.28462	-0.52729	-0.47826	-0.44698	-0.06186	-0.23625	-0.11228
0.093739	0.023217	0.001182	1.9E-10	1.3E-08	1.38E-07	0.489614	0.007495	0.208835
0.265645	-0.11789	-0.25762	-0.39576	-0.42766	-0.28689	-0.22769	0.046163	-0.00529
0.00254	0.186814	0.003455	4.12E-06	5.31E-07	0.001075	0.010038	0.606301	0.952896
0.037771	-0.30789	-0.26979	0.233993	0.324013	0.200451	-0.50511	-0.20806	-0.34787
0.673313	0.000429	0.00216	0.008103	0.000202	0.023845	1.39E-09	0.018913	6.14E-05
-0.14737	-0.38476	-0.36118	0.051391	0.03789	0.045488	-0.18607	0.308368	0.026371
0.098241	7.96E-06	3.02E-05	0.566106	0.672345	0.611579	0.036215	0.00042	0.768527
0.089259	-0.45654	-0.38539	-0.10992	-0.15517	-0.10576	-0.35453	-0.00608	-0.21124
0.318303	6.86E-08	7.67E-06	0.218634	0.081507	0.236658	4.32E-05	0.94592	0.017127
-0.23136	-0.06995	0.088199	0.35311	0.369954	0.577351	0.038469	-0.02276	-0.06146
0.008869	0.43451	0.324108	4.66E-05	1.86E-05	1.2E-12	0.667632	0.799518	0.49246
-0.06777	0.22542	0.359777	0.485466	0.735292	0.807331	-0.09819	-0.05857	-0.24649
0.449008	0.010831	3.26E-05	7.26E-09	7.36E-23	2.04E-30	0.272097	0.513074	0.005213
-0.5602	0.673888	0.649212	0.62633	0.837086	0.756411	0.487362	0.639409	0.533164
7.48E-12	3.88E-18	1.52E-16	3.4E-15	1.53E-34	8.44E-25	6.22E-09	5.94E-16	1.1E-10
-0.34827	0.102767	0.157487	0.603118	0.767683	0.801012	0.003897	0.475794	0.100371
6.01E-05	0.25026	0.077013	6.21E-14	6.43E-26	1.24E-29	0.965319	1.57E-08	0.261531
-0.16898	0.048489	0.071387	0.129658	0.039783	0.150367	0.394012	0.485895	0.324679
0.05754	0.58826	0.425126	0.146261	0.656988	0.091525	4.58E-06	7.01E-09	0.000196
-0.11301	0.506146	0.52567	0.2706	0.315394	0.315806	0.421612	0.012616	0.102279
0.205865	1.28E-09	2.21E-10	0.002091	0.000304	0.000298	7.95E-07	0.888046	0.252529
0.344221	-0.02484	-0.12849	-0.34749	-0.5243	-0.61058	-0.01663	-0.46014	-0.17831
7.41E-05	0.781596	0.149963	6.26E-05	2.51E-10	2.51E-14	0.852753	5.25E-08	0.044891
-0.00289	0.230952	0.120743	-0.05946	-0.17245	-0.22041	0.397605	-0.01374	0.180966
0.974258	0.008991	0.176309	0.506676	0.052533	0.012772	3.68E-06	0.878164	0.041745
-0.09821	0.12332	0.38193	0.108028	0.121592	0.188372	0.154617	-0.1086	0.025539
0.271977	0.167194	9.39E-06	0.226694	0.173268	0.033931	0.082623	0.224241	0.775638

NDUFB11	NDUFB7	NDUFS6	NDUFS7	NDUFS8	NEK10	NELFA	NELFB	NEU4
black	black	black	black	black	black	black	black	black
0.03104	-0.08732	-0.11094	-0.07247	-0.05767	0.053694	-0.28763	-0.25015	-0.31601
0.729025	0.32897	0.214359	0.418133	0.519577	0.548803	0.001042	0.004563	0.000295
0.111558	-0.05303	-0.13754	-0.05021	-0.00653	0.028955	-0.30039	-0.25681	-0.29151
0.211786	0.553763	0.123085	0.575041	0.941872	0.746585	0.000601	0.003563	0.000883
-0.14614	-0.25425	-0.15855	-0.27179	-0.23297	-0.10916	0.145944	0.095046	0.111086
0.101125	0.003921	0.075016	0.001995	0.008394	0.22184	0.101585	0.287814	0.213739
-0.51828	-0.57469	-0.48659	-0.66677	-0.6196	0.148888	-0.31024	-0.47742	-0.32908
4.35E-10	1.6E-12	6.62E-09	1.16E-17	8.09E-15	0.094796	0.000386	1.39E-08	0.000158
0.710373	0.460184	0.267635	-0.12351	0.43863	-0.04504	-0.20277	-0.17488	-0.28041
8.53E-21	5.23E-08	0.00235	0.16654	2.5E-07	0.615076	0.022234	0.049245	0.001407
-0.08256	-0.28229	-0.31543	-0.72128	-0.3271	0.198313	-0.57734	-0.68689	-0.62098
0.356115	0.001302	0.000304	1.14E-21	0.000174	0.025416	1.2E-12	4.85E-19	6.79E-15
-0.105	-0.21095	-0.30655	-0.50963	-0.26835	0.263284	-0.46956	-0.53202	-0.44147
0.240048	0.017284	0.000456	9.4E-10	0.002286	0.002784	2.56E-08	1.22E-10	2.04E-07
-0.12773	-0.09691	-0.07205	-0.37841	-0.20952	0.471928	-0.29852	-0.34633	-0.25463
0.152422	0.2784	0.420803	1.15E-05	0.018072	2.13E-08	0.000652	6.65E-05	0.003865
-0.27889	-0.22848	0.021022	-0.05206	-0.21734	-0.15689	0.104047	-0.00635	-0.14846
0.001498	0.009775	0.814531	0.561015	0.014107	0.078147	0.244374	0.943483	0.095769
-0.00413	0.052245	0.245607	-0.01302	0.076723	-0.18847	-0.06594	-0.18103	-0.26231
0.963222	0.559659	0.005382	0.884461	0.391257	0.033839	0.461357	0.041672	0.00289
-0.24949	-0.19986	-0.05479	-0.22605	-0.18795	0.017036	-0.22916	-0.33434	-0.34136
0.004673	0.024271	0.540635	0.010605	0.034337	0.84923	0.009555	0.000122	8.58E-05
-0.11435	-0.01079	-0.01161	0.522523	0.04703	-0.21824	0.399726	0.280765	0.210892
0.200502	0.904159	0.896918	2.96E-10	0.599545	0.013706	3.23E-06	0.001387	0.017314
-0.17852	-0.02067	0.169377	0.445145	0.034115	-0.35704	0.709892	0.634829	0.585483
0.044634	0.817615	0.056951	1.57E-07	0.703374	3.78E-05	9.3E-21	1.1E-15	4.84E-13
0.552482	0.786081	0.7867	0.852271	0.776076	-0.53971	0.871902	0.869538	0.659738
1.65E-11	6.91E-28	5.89E-28	5.54E-37	8.57E-27	5.84E-11	1.41E-40	4.11E-40	3.31E-17
0.089544	0.312096	0.563674	0.636232	0.374665	-0.51849	0.686177	0.579086	0.439266
0.316759	0.000354	5.21E-12	9.14E-16	1.43E-05	4.27E-10	5.45E-19	9.9E-13	2.39E-07
0.196619	0.227214	0.187727	0.396114	0.27753	-0.14037	0.094678	0.136773	0.263993
0.026723	0.010201	0.034559	4.03E-06	0.001583	0.115479	0.289693	0.125196	0.002708
0.153045	0.188719	0.044527	0.39479	0.200093	-0.08681	0.398268	0.53004	0.662894
0.08583	0.033598	0.619132	4.37E-06	0.024102	0.331793	3.53E-06	1.47E-10	2.07E-17
-0.1934	-0.24753	-0.50102	-0.37375	-0.32083	0.465924	-0.52192	-0.34506	-0.17397
0.029366	0.005021	1.98E-09	1.5E-05	0.000236	3.39E-08	3.12E-10	7.1E-05	0.050459
0.1496	0.114629	-0.15321	0.112274	0.094035	0.326291	-0.19128	0.068843	0.122511
0.093211	0.199398	0.085494	0.208852	0.292994	0.000181	0.031222	0.441857	0.170016
0.187799	-0.08212	0.003284	0.034461	0.029606	-0.15705	0.234426	0.203568	0.213471
0.034489	0.358714	0.970766	0.70051	0.741083	0.077846	0.007983	0.021705	0.015963

NFKBIB	NFKBIL1	NFYA	NHS	NHSL2	NME1	NME2	NME3	NME4
black	black	black	black	black	black	black	black	black
-0.04743	-0.14293	0.000199	0.17738	0.172103	-0.14237	-0.32446	-0.08837	-0.40244
0.596413	0.10892	0.998229	0.046037	0.053016	0.110331	0.000198	0.323163	2.73E-06
-0.06422	-0.16081	0.029699	0.131936	0.129578	-0.02289	-0.33357	-0.07581	-0.35434
0.473152	0.070899	0.740297	0.139243	0.146513	0.798413	0.000127	0.396956	4.37E-05
-0.12302	-0.00304	0.344077	-0.0635	-0.17463	0.311481	0.280547	-0.32703	0.369734
0.168225	0.972978	7.47E-05	0.478181	0.049574	0.000364	0.001399	0.000175	1.88E-05
-0.45319	-0.40359	0.504987	0.371345	0.094136	0.017277	-0.08673	-0.52167	-0.13326
8.79E-08	2.54E-06	1.41E-09	1.72E-05	0.292475	0.847122	0.332277	3.19E-10	0.135288
-0.10364	0.139647	-0.1383	-0.00433	-0.07896	0.643676	-0.10596	0.267032	0.128755
0.246251	0.11738	0.121001	0.961431	0.377539	3.3E-16	0.235767	0.002407	0.149115
-0.53986	-0.3723	0.331684	0.473658	0.164893	0.248028	-0.31346	-0.28712	-0.25989
5.76E-11	1.63E-05	0.000139	1.86E-08	0.063943	0.00493	0.000333	0.001064	0.00317
-0.38707	-0.44217	0.35614	0.449897	0.172518	0.098622	-0.24708	-0.29106	-0.15805
6.94E-06	1.94E-07	3.97E-05	1.12E-07	0.052438	0.269975	0.005103	0.000901	0.075939
-0.20804	-0.24513	0.055257	0.312038	0.279337	-0.28891	-0.26504	-0.02389	-0.2579
0.01892	0.005476	0.537213	0.000355	0.001471	0.000987	0.002601	0.78979	0.003419
0.002632	0.006036	0.251916	0.221786	-0.07796	-0.04664	0.214676	-0.06947	0.091247
0.976572	0.946299	0.004275	0.012211	0.38364	0.602591	0.015364	0.437703	0.307603
0.187226	0.027545	-0.12325	0.180027	-0.00346	-0.23796	-0.14405	0.240165	-0.14214
0.035053	0.758536	0.167425	0.042834	0.96924	0.00706	0.106146	0.006534	0.110913
-0.02838	-0.12167	0.010196	0.310789	0.134819	-0.30512	-0.26221	-0.02675	-0.33517
0.751488	0.17299	0.909417	0.000376	0.130733	0.000486	0.002901	0.76531	0.000117
0.226474	0.176389	0.01743	-0.15497	-0.38853	0.148484	0.207819	-0.03829	-0.02793
0.010457	0.047286	0.845788	0.081911	6.36E-06	0.095707	0.019053	0.669076	0.75527
0.49233	0.330392	0.025902	-0.43756	-0.30606	-0.14245	0.525468	0.018341	0.493883
4.13E-09	0.000148	0.77253	2.69E-07	0.000466	0.110132	2.25E-10	0.83783	3.63E-09
0.81291	0.85556	-0.52071	-0.71302	-0.51219	0.227151	0.606391	0.772259	0.760613
3.91E-31	1.51E-37	3.49E-10	5.27E-21	7.5E-10	0.010223	4.18E-14	2.17E-26	3.29E-25
0.740837	0.513899	-0.2728	-0.40123	-0.33221	-0.25888	0.389082	0.501527	0.409743
2.38E-23	6.44E-10	0.001916	2.94E-06	0.000136	0.003294	6.16E-06	1.9E-09	1.72E-06
0.313609	0.038237	-0.21516	-0.31949	-0.02624	-0.1718	-0.12513	0.253731	-0.17109
0.00033	0.669524	0.01513	0.000251	0.769655	0.053438	0.161014	0.003997	0.054447
0.230286	0.184497	-0.1394	-0.52525	-0.23251	0.162807	0.287841	-0.00862	0.345258
0.009197	0.037849	0.11804	2.3E-10	0.008525	0.067426	0.001033	0.923368	7.03E-05
-0.57282	-0.40994	0.099172	0.255634	0.31724	0.03126	-0.31378	-0.38452	-0.40007
1.97E-12	1.7E-06	0.267303	0.003723	0.000279	0.727173	0.000328	8.07E-06	3.16E-06
-0.18272	-0.12075	-0.19864	-0.05442	0.17871	0.024933	-0.17061	-0.07253	-0.19878
0.03977	0.176281	0.025168	0.543383	0.044403	0.780823	0.055146	0.417756	0.025068
-0.05316	0.03709	0.242394	-0.17604	-0.20305	0.456273	0.283479	-0.28035	0.348046
0.552828	0.678877	0.006038	0.047728	0.022048	7E-08	0.00124	0.001411	6.08E-05

NOC2L	NOC4L	NOSIP	NPB	NPW	NR1H2	NR2F6	NRCAM	NT5C
black	black	black	black	black	black	black	black	black
-0.38839	-0.2377	-0.17208	-0.23256	-0.27503	0.002261	-0.30449	-0.1166	-0.3458
6.42E-06	0.007126	0.05305	0.008512	0.001752	0.979869	0.0005	0.191714	6.83E-05
-0.37938	-0.22163	-0.14523	-0.28545	-0.23749	-0.04312	-0.26075	-0.10262	-0.33417
1.09E-05	0.012275	0.103275	0.001142	0.007177	0.630228	0.003068	0.250941	0.000123
0.359278	0.098743	-0.04195	-0.04157	0.398267	-0.31031	0.099927	0.019148	0.247959
3.35E-05	0.269388	0.63961	0.642622	3.53E-06	0.000385	0.263658	0.8308	0.004943
-0.2137	-0.37968	-0.42788	-0.39289	0.014633	-0.59021	-0.29623	0.392068	-0.08948
0.015848	1.07E-05	5.23E-07	4.9E-06	0.870293	2.83E-13	0.000721	5.15E-06	0.317107
-0.21126	-0.13805	0.479983	-0.16283	-0.1037	-0.07754	-0.06721	-0.31381	0.210532
0.017118	0.121688	1.13E-08	0.067389	0.24595	0.386226	0.452778	0.000327	0.017511
-0.54585	-0.5679	-0.203	-0.53548	-0.25954	-0.59704	-0.43004	0.239053	-0.14783
3.2E-11	3.33E-12	0.022081	8.79E-11	0.003213	1.28E-13	4.52E-07	0.006795	0.097201
-0.44902	-0.45751	-0.22712	-0.53506	-0.32337	-0.39074	-0.36026	0.28182	-0.2013
1.19E-07	6.39E-08	0.010235	9.15E-11	0.000209	5.58E-06	3.18E-05	0.001328	0.023244
-0.39438	-0.401	-0.22484	-0.2756	-0.30998	-0.15915	-0.30912	0.150476	-0.18592
4.48E-06	2.98E-06	0.011042	0.001712	0.00039	0.073907	0.000406	0.091287	0.03637
0.140056	0.011942	-0.1676	0.042711	0.128999	0.098526	0.220623	0.085755	0.127986
0.1163	0.893991	0.059637	0.633512	0.148342	0.270447	0.012685	0.337748	0.151581
-0.33852	-0.14314	0.000304	-0.04054	-0.27082	0.245947	-0.15899	-0.09517	0.037697
9.91E-05	0.108399	0.997292	0.650877	0.002073	0.005316	0.074195	0.28718	0.673917
-0.42948	-0.17931	-0.2415	-0.24244	-0.28612	-0.01135	-0.30261	0.120036	-0.12931
4.69E-07	0.043677	0.006234	0.006029	0.00111	0.899214	0.000544	0.178875	0.147346
0.347674	0.544929	-0.01948	0.263696	0.173045	0.231544	0.296759	-0.00343	0.297626
6.2E-05	3.51E-11	0.827956	0.00274	0.05171	0.008812	0.000704	0.969484	0.000678
0.706788	0.578861	0.068681	0.472698	0.511286	0.40534	0.457853	-0.17432	0.405911
1.62E-20	1.02E-12	0.442934	2.01E-08	8.13E-10	2.28E-06	6.23E-08	0.049981	2.19E-06
0.75312	0.73908	0.79861	0.796583	0.535534	0.868709	0.768086	-0.56167	0.784533
1.75E-24	3.41E-23	2.42E-29	4.23E-29	8.74E-11	5.94E-40	5.85E-26	6.42E-12	1.03E-27
0.425316	0.508073	0.293909	0.575528	0.27799	0.760957	0.436981	-0.37608	0.486874
6.21E-07	1.08E-09	0.000797	1.46E-12	0.001554	3.04E-25	2.8E-07	1.32E-05	6.47E-09
-0.09187	0.08988	0.122306	0.149724	-0.12689	0.256035	-0.14016	-0.28062	-0.08727
0.304305	0.314937	0.170737	0.092937	0.155132	0.003668	0.116018	0.001395	0.329234
0.533266	0.427791	0.215804	0.281254	0.384238	0.128347	0.2187	-0.17846	0.14788
1.08E-10	5.26E-07	0.014821	0.001359	8.21E-06	0.150421	0.013503	0.044711	0.097078
-0.26249	-0.34569	-0.27074	-0.35088	-0.20668	-0.57005	-0.28824	0.382092	-0.51701
0.00287	6.87E-05	0.00208	5.24E-05	0.019735	2.65E-12	0.001015	9.3E-06	4.88E-10
-0.00231	-0.00922	0.010736	-0.07224	-0.12355	-0.09993	-0.05147	0.086289	-0.3813
0.979396	0.91809	0.904648	0.419589	0.166404	0.263636	0.56549	0.334737	9.74E-06
0.42691	0.257053	0.107228	0.080917	0.351055	-0.15886	0.083064	-0.35471	0.173359
5.58E-07	0.00353	0.230173	0.365806	5.2E-05	0.074435	0.353183	4.28E-05	0.05128

NTHL1	NTMT1	NUBP2	NUBPL	NUDC	NUDT16L1	NUDT22	NUDT3	NUDT8
black	black	black	black	black	black	black	black	black
-0.25089	-0.03116	-0.28967	0.207426	0.044014	-0.25152	-0.09351	-0.19444	-0.10328
0.004441	0.728035	0.000956	0.019285	0.623182	0.004337	0.295711	0.02849	0.247897
-0.14962	0.07225	-0.27091	0.265971	0.06769	-0.16427	-0.07754	-0.24514	-0.06696
0.093168	0.41954	0.002065	0.002508	0.44956	0.06496	0.386239	0.005473	0.454497
0.135002	0.129537	0.083324	0.056798	-0.24272	-0.12394	-0.03811	-0.00297	0.0326
0.130206	0.146641	0.351672	0.525905	0.005968	0.165063	0.670547	0.973561	0.715968
-0.30739	-0.28313	-0.36525	0.395629	-0.62363	-0.34416	-0.29235	-0.40951	-0.23989
0.000439	0.001258	2.42E-05	4.15E-06	4.83E-15	7.44E-05	0.000852	1.75E-06	0.006598
0.367777	0.53687	0.257169	0.254094	0.0062	0.494178	0.371048	-0.3608	0.35878
2.1E-05	7.69E-11	0.003514	0.003943	0.944847	3.54E-09	1.75E-05	3.08E-05	3.44E-05
-0.22322	-0.06535	-0.30999	0.594386	-0.60386	-0.04809	-0.1049	-0.6799	-0.08449
0.011649	0.465446	0.00039	1.74E-13	5.68E-14	0.591361	0.240509	1.5E-18	0.344975
-0.17447	-0.00401	-0.33976	0.356169	-0.37026	-0.07024	-0.11026	-0.64785	-0.12534
0.049791	0.964347	9.31E-05	3.96E-05	1.83E-05	0.43262	0.217193	1.84E-16	0.160293
-0.32586	-0.39418	-0.23681	0.148325	0.071918	-0.00419	-0.16776	-0.36402	-0.1414
0.000185	4.53E-06	0.00735	0.096067	0.421685	0.962747	0.059402	2.59E-05	0.112792
-0.29818	-0.05103	-0.0404	-0.14443	-0.27324	-0.3633	0.20259	-0.04176	0.008919
0.000662	0.568844	0.652024	0.10521	0.001882	2.69E-05	0.022357	0.641097	0.920728
-0.31321	-0.10575	-0.03756	-0.05504	-0.11809	-0.1721	0.312626	-0.20679	0.144172
0.000336	0.236696	0.675058	0.538805	0.186081	0.053017	0.000346	0.019668	0.10585
-0.43316	-0.29475	-0.24436	0.108357	-0.22805	-0.28302	0.084807	-0.21469	-0.08934
3.65E-07	0.000769	0.005628	0.225273	0.00992	0.001264	0.343135	0.015358	0.317886
0.094643	0.298595	0.063489	-0.29149	0.116914	-0.09073	0.180448	0.349633	-0.06757
0.289872	0.00065	0.478247	0.000884	0.190528	0.31036	0.042343	5.6E-05	0.450375
0.184642	0.081684	0.287957	-0.5137	0.326868	-0.19939	0.144154	0.565922	0.157589
0.037697	0.361267	0.001028	6.56E-10	0.000176	0.024615	0.105893	4.11E-12	0.076819
0.726278	0.541883	0.904009	-0.62971	0.626536	0.500981	0.764088	0.739478	0.693583
4.36E-22	4.73E-11	5.81E-48	2.18E-15	3.31E-15	1.99E-09	1.48E-25	3.14E-23	1.6E-19
0.130598	0.105689	0.446074	-0.59679	0.416216	-0.03952	0.532335	0.502953	0.416559
0.143335	0.236972	1.47E-07	1.32E-13	1.13E-06	0.659126	1.19E-10	1.68E-09	1.11E-06
0.132326	-0.03828	0.034896	-0.17261	0.407219	0.135463	-0.05429	0.166967	-0.0053
0.138067	0.669191	0.696912	0.05231	2.02E-06	0.128888	0.54437	0.060626	0.952873
0.440789	0.215516	0.2692	-0.32898	0.391354	0.217025	-0.13106	0.485399	-0.043
2.14E-07	0.014958	0.00221	0.000159	5.38E-06	0.014252	0.141926	7.3E-09	0.631232
-0.07891	-0.23793	-0.35006	0.409713	-0.2964	0.038577	-0.57789	-0.1691	-0.45479
0.377827	0.007067	5.47E-05	1.72E-06	0.000715	0.666756	1.13E-12	0.057357	7.81E-08
0.108264	-0.00485	-0.09151	0.016929	0.224192	0.203921	-0.35325	0.108249	-0.28026
0.225676	0.95688	0.30622	0.850161	0.011281	0.021473	4.63E-05	0.225739	0.001416
0.304405	0.449866	0.100297	-0.06229	0.060178	-0.1153	-0.04881	0.059526	0.078968
0.000502	1.12E-07	0.261885	0.486584	0.501533	0.196753	0.585824	0.506193	0.377504

OAZ1	ODF3L2	OGFOD3	OGFR	OPLAH	OTUD4	OVCA2	OXLD1	PAFAH1B3
black	black	black	black	black	black	black	black	black
0.107966	0.067008	-0.25654	-0.02323	-0.04671	-0.11199	-0.28348	-0.10293	-0.34168
0.226961	0.454148	0.003599	0.795455	0.602056	0.210013	0.00124	0.249502	8.44E-05
0.062732	0.1221	-0.31251	-0.14098	-0.0666	-0.19531	-0.36075	-0.09323	-0.25122
0.48352	0.171463	0.000348	0.113897	0.456899	0.027775	3.09E-05	0.297148	0.004387
-0.51965	-0.00519	0.161884	-0.2704	-0.42803	0.254854	0.022341	-0.06307	0.379059
3.84E-10	0.953789	0.069016	0.002108	5.17E-07	0.003833	0.80312	0.481135	1.11E-05
-0.68344	-0.30111	-0.20377	-0.5547	-0.52223	0.579922	-0.30817	-0.37719	-0.23291
8.51E-19	0.000582	0.021571	1.32E-11	3.03E-10	9.03E-13	0.000424	1.24E-05	0.008411
0.18812	-0.08196	-0.19663	-0.31363	-0.27984	-0.40412	-0.30648	0.413285	0.084731
0.034176	0.359654	0.026711	0.00033	0.001441	2.46E-06	0.000457	1.37E-06	0.343573
-0.42719	-0.38524	-0.44104	-0.67315	-0.5591	0.277663	-0.55739	-0.17307	-0.41202
5.48E-07	7.73E-06	2.11E-07	4.35E-18	8.39E-12	0.001575	1E-11	0.051671	1.49E-06
-0.3005	-0.1955	-0.40011	-0.54282	-0.37848	0.320381	-0.59523	-0.22937	-0.3147
0.000598	0.02762	3.15E-06	4.32E-11	1.15E-05	0.000241	1.58E-13	0.009487	0.000314
-0.11285	-0.37045	-0.28317	-0.18847	0.06242	0.010376	-0.26269	-0.1188	-0.43073
0.206513	1.81E-05	0.001256	0.033842	0.485701	0.907831	0.002848	0.183421	4.31E-07
-0.0621	-0.08958	0.329931	0.125763	-0.00284	0.307999	0.178556	-0.06453	-0.2389
0.487926	0.316558	0.000152	0.158876	0.974735	0.000427	0.04459	0.471071	0.006832
0.289149	-0.08373	0.043042	0.252149	0.248232	-0.00346	0.00242	0.130265	-0.59472
0.000977	0.349332	0.630886	0.004239	0.004893	0.969182	0.978455	0.144367	1.68E-13
0.030227	-0.30331	-0.07696	0.105919	0.018098	0.173362	-0.11139	-0.10022	-0.61721
0.735852	0.000528	0.389788	0.235944	0.839956	0.051276	0.212491	0.262257	1.1E-14
0.11681	0.196478	0.288252	0.190998	0.095282	-0.0177	0.302911	-0.00195	0.239171
0.190926	0.026833	0.001015	0.031477	0.28661	0.84346	0.000537	0.98265	0.006767
-0.04025	0.379866	0.610335	0.469377	0.297575	0.04481	0.561379	0.101316	0.47897
0.653227	1.06E-05	2.58E-14	2.6E-08	0.00068	0.616907	6.62E-12	0.257043	1.22E-08
0.617415	0.465081	0.734889	0.741239	0.584534	-0.49724	0.801831	0.790711	0.629638
1.07E-14	3.61E-08	7.98E-23	2.19E-23	5.39E-13	2.74E-09	9.85E-30	2.06E-28	2.2E-15
0.435002	0.381853	0.639148	0.774034	0.670684	-0.20948	0.646421	0.414147	0.068515
3.21E-07	9.43E-06	6.15E-16	1.41E-26	6.36E-18	0.018092	2.25E-16	1.3E-06	0.44404
0.32955	0.300497	-0.09932	0.236094	0.363985	-0.44837	0.045138	0.135724	0.032502
0.000155	0.000598	0.266588	0.007535	2.59E-05	1.25E-07	0.614327	0.128144	0.716788
0.046713	0.292444	0.155477	0.123007	0.01838	-0.21769	0.255903	0.062122	0.755407
0.602011	0.000849	0.080909	0.168282	0.837492	0.01395	0.003686	0.487793	1.06E-24
-0.25763	-0.26737	-0.51116	-0.47902	-0.40263	0.221076	-0.36955	-0.39768	0.021074
0.003454	0.002375	8.21E-10	1.22E-08	2.7E-06	0.012498	1.9E-05	3.66E-06	0.814076
0.178158	-0.02831	-0.26832	-0.06855	-0.04035	-0.1777	-0.13962	-0.15801	0.247531
0.045076	0.752036	0.002288	0.443774	0.652406	0.045637	0.117448	0.076024	0.00502
-0.27352	0.351789	0.108719	-0.18951	-0.41496	-0.00522	5.84E-05	0.009038	0.555084
0.001862	5E-05	0.22372	0.032847	1.23E-06	0.953582	0.99948	0.919671	1.27E-11

PAK4	PANO1	PAPOLG	PARP11	PCBP4	PCM1	PCSK4	PCYOX1	PDF
black	black	black	black	black	black	black	black	black
-0.2551	-0.35943	-0.01199	-0.04365	-0.06419	-0.07709	-0.18399	0.088057	-0.35037
0.003799	3.32E-05	0.893555	0.626072	0.473377	0.388977	0.038391	0.324893	5.39E-05
-0.28193	-0.32439	0.046889	-0.03799	-0.02494	-0.17408	-0.23256	0.062999	-0.40482
0.001322	0.000199	0.600645	0.671565	0.780737	0.050308	0.008512	0.481653	2.35E-06
0.095977	0.338536	0.007909	0.535464	0.057295	0.356571	-0.03697	-0.11322	0.095583
0.283095	9.9E-05	0.929681	8.8E-11	0.522288	3.88E-05	0.679854	0.205016	0.285085
-0.38895	0.094402	0.321629	0.572323	-0.38024	0.670408	-0.24851	0.23239	-0.2737
6.21E-06	0.291104	0.000227	2.07E-12	1.04E-05	6.64E-18	0.004844	0.008561	0.001848
-0.35941	-0.12206	0.024739	0.031669	0.046839	-0.35523	-0.14501	-0.47288	-0.31892
3.33E-05	0.171615	0.782482	0.723751	0.601032	4.17E-05	0.103818	1.98E-08	0.000258
-0.71406	-0.13469	0.399978	0.454119	-0.4676	0.368239	-0.40676	0.043992	-0.54987
4.36E-21	0.131119	3.18E-06	8.21E-08	2.98E-08	2.05E-05	2.08E-06	0.623357	2.15E-11
-0.54186	-0.19847	0.209565	0.20634	-0.23085	0.176862	-0.28667	0.096343	-0.60694
4.74E-11	0.0253	0.018047	0.019941	0.009022	0.046686	0.001085	0.281256	3.91E-14
-0.32531	-0.14576	0.02664	-0.12227	-0.27723	0.341585	0.027011	0.079602	-0.20434
0.00019	0.102013	0.766237	0.170855	0.001603	8.48E-05	0.763078	0.373672	0.021201
0.100517	0.070002	0.044667	0.110011	-0.23659	0.165564	0.11875	0.144264	0.158228
0.260836	0.43419	0.61803	0.218233	0.007406	0.062854	0.183609	0.105625	0.075615
-0.15297	-0.21166	0.024257	-0.09404	-0.20476	-0.00228	0.097524	-0.09637	-0.06442
0.085994	0.0169	0.786625	0.29299	0.020929	0.979675	0.275371	0.2811	0.471807
-0.24649	-0.14955	0.105195	0.184291	-0.37053	0.234682	-0.03361	0.048793	-0.20402
0.005212	0.093311	0.239183	0.038068	1.8E-05	0.007913	0.707585	0.585921	0.021409
0.374665	-0.04909	-0.11395	-0.06486	0.18646	-0.19055	0.21603	-0.00041	0.21504
1.43E-05	0.583672	0.202088	0.468745	0.03582	0.031888	0.014714	0.996346	0.015187
0.739591	0.357515	-0.38024	-0.04503	0.464516	0.061828	0.482055	-0.10045	0.593119
3.07E-23	3.69E-05	1.04E-05	0.615154	3.77E-08	0.489857	9.56E-09	0.26114	2.02E-13
0.851601	0.44131	-0.53216	-0.44381	0.601684	-0.57762	0.729796	-0.60308	0.788117
7.2E-37	2.07E-07	1.2E-10	1.73E-07	7.37E-14	1.16E-12	2.2E-22	6.24E-14	4.07E-28
0.663516	0.175069	-0.44006	-0.37742	0.377779	-0.21107	0.607952	-0.29346	0.61677
1.89E-17	0.048993	2.26E-07	1.22E-05	1.19E-05	0.017218	3.46E-14	0.000813	1.16E-14
0.116674	-0.20643	-0.23725	-0.41404	0.39382	-0.24816	-0.04439	-0.17072	0.021011
0.191444	0.019887	0.007238	1.31E-06	4.63E-06	0.004907	0.620251	0.054979	0.814627
0.476411	0.238256	-0.32974	-0.07517	0.505766	-0.2244	0.055642	-0.15138	0.307011
1.5E-08	0.006988	0.000153	0.400942	1.32E-09	0.011203	0.53438	0.089344	0.000447
-0.40195	-0.07569	0.385391	0.202599	-0.31481	0.139996	-0.48261	0.392787	-0.34816
2.81E-06	0.397711	7.67E-06	0.022351	0.000312	0.116458	9.15E-09	4.93E-06	6.04E-05
-0.03857	-0.12137	0.050497	-0.19085	0.045301	-0.30161	-0.23793	0.196347	-0.12757
0.66681	0.174057	0.572886	0.031606	0.613044	0.000569	0.007069	0.026938	0.152916
0.156773	0.063098	-0.11426	0.231525	0.481083	0.017036	-0.08304	-0.23656	0.028152
0.078378	0.480966	0.200861	0.008818	1.03E-08	0.849226	0.353331	0.007415	0.753379

PDLIM2	PEMT	PET117	PEX10	PEX11G	PEX6	PFDN2	PFKL	PGLS
black	black	black	black	black	black	black	black	black
0.017931	-0.24449	-0.1782	-0.20441	-0.21634	-0.19931	0.011698	-0.13207	-0.17695
0.841413	0.005602	0.04502	0.021153	0.014569	0.024675	0.896143	0.13884	0.046577
-0.00843	-0.15121	-0.12922	-0.15694	-0.1942	-0.18629	0.062569	-0.17571	-0.20151
0.925027	0.089693	0.147647	0.078057	0.028684	0.035989	0.484657	0.04816	0.023101
-0.17431	0.231371	0.085244	-0.02351	0.020046	0.007803	-0.14668	-0.1682	0.10946
0.049991	0.008864	0.340647	0.793006	0.822997	0.930617	0.099854	0.058728	0.220562
-0.3369	-0.23273	-0.12714	-0.26343	-0.23455	-0.1085	-0.48142	-0.5329	-0.22084
0.000108	0.008462	0.15433	0.002768	0.007948	0.224658	1.01E-08	1.12E-10	0.012596
0.254274	0.043634	-0.08405	-0.0367	0.356774	0.064276	0.471328	-0.32639	0.132043
0.003917	0.626184	0.347488	0.682035	3.83E-05	0.472798	2.23E-08	0.00018	0.138922
-0.16618	-0.37912	-0.20877	-0.31205	-0.07849	-0.14032	-0.20461	-0.73254	-0.23338
0.061874	1.11E-05	0.018499	0.000355	0.380412	0.115594	0.021024	1.28E-22	0.008275
-0.17261	-0.26048	-0.39791	-0.27871	-0.23918	-0.05004	-0.1456	-0.49845	-0.21898
0.052308	0.0031	3.61E-06	0.001509	0.006766	0.576359	0.102399	2.47E-09	0.013382
-0.27073	-0.59395	-0.30221	-0.06981	-0.05182	0.191048	-0.20011	-0.21291	-0.15038
0.00208	1.83E-13	0.000554	0.435434	0.562857	0.031431	0.024089	0.016248	0.091498
0.285807	0.045582	0.039587	0.01855	-0.11323	0.121565	-0.23606	0.173775	0.319306
0.001125	0.610844	0.658572	0.836011	0.204978	0.173364	0.007545	0.050716	0.000253
0.344833	-0.29588	-0.11912	0.008323	0.144369	0.151669	-0.06389	0.129409	0.344098
7.18E-05	0.000732	0.182248	0.92601	0.105369	0.08872	0.47547	0.147043	7.46E-05
0.100638	-0.40156	-0.1788	-0.00769	-0.07568	0.087365	-0.28493	-0.11938	0.0582
0.260257	2.88E-06	0.044299	0.931629	0.397773	0.328721	0.001167	0.181291	0.515723
0.323554	0.511949	0.025001	0.402285	-0.06904	0.169258	-0.00619	0.341887	0.131428
0.000207	7.66E-10	0.780242	2.75E-06	0.440531	0.057128	0.944942	8.35E-05	0.140786
0.105909	0.49663	0.243528	0.291547	0.097476	0.27155	-0.01412	0.621239	0.431947
0.235989	2.88E-09	0.005799	0.000882	0.275608	0.002014	0.874768	6.57E-15	3.96E-07
0.596225	0.713506	0.47458	0.588244	0.683333	0.537521	0.56291	0.849981	0.833472
1.41E-13	4.83E-21	1.73E-08	3.54E-13	8.66E-19	7.22E-11	5.64E-12	1.35E-36	5.34E-34
0.507913	0.320047	0.242445	0.421886	0.351723	0.435807	0.18048	0.818245	0.745859
1.09E-09	0.000244	0.006027	7.81E-07	5.02E-05	3.04E-07	0.042306	7.66E-32	8.32E-24
-0.02596	-0.02216	-0.05468	0.091083	0.033203	-0.06188	0.314829	0.202485	-0.02395
0.772041	0.804706	0.541451	0.308476	0.710948	0.489516	0.000312	0.022428	0.789297
-0.12661	0.469747	0.088185	0.182889	0.039918	-0.08757	0.254952	0.243872	-0.02434
0.156062	2.53E-08	0.324189	0.039584	0.655896	0.32759	0.003819	0.005728	0.785943
-0.51322	-0.24966	-0.07992	-0.35061	-0.36374	-0.45934	-0.21154	-0.55483	-0.75883
6.84E-10	0.004644	0.37177	5.32E-05	2.63E-05	5.57E-08	0.016967	1.3E-11	4.92E-25
-0.25709	-0.0071	-0.06872	-0.10828	-0.21804	-0.2944	0.117543	-0.09684	-0.49431
0.003525	0.936861	0.442691	0.225594	0.013793	0.00078	0.188137	0.278786	3.5E-09
-0.00776	0.358334	0.070944	-0.26442	-0.04666	-0.29787	0.347759	-0.097	-0.02632
0.931034	3.53E-05	0.428013	0.002664	0.602398	0.000671	6.17E-05	0.277986	0.768948

PHB	PHF3	PHRF1	PIAS4	PIDD1	PIH1D1	PIK3R4	PKN1	PLEKHH3
black	black	black	black	black	black	black	black	black
-0.25739	-0.037	-0.24223	-0.26411	-0.2621	-0.09855	-0.07824	-0.23404	-0.38207
0.003485	0.679618	0.006074	0.002696	0.002913	0.270337	0.381933	0.008089	9.32E-06
-0.19053	-0.11158	-0.29798	-0.27991	-0.27051	-0.09775	-0.1067	-0.27501	-0.43443
0.031906	0.211689	0.000668	0.001436	0.002099	0.274265	0.232508	0.001753	3.34E-07
0.334931	0.200151	0.05339	0.092743	0.05905	-0.16572	0.091167	0.150148	0.261202
0.000119	0.02406	0.551074	0.299707	0.509604	0.062607	0.308028	0.092003	0.003015
0.070897	0.519156	-0.34596	-0.38427	-0.28275	-0.49158	0.487159	-0.29023	-0.15883
0.428325	4.02E-10	6.78E-05	8.19E-06	0.001278	4.39E-09	6.32E-09	0.000933	0.074492
0.524669	-0.44022	-0.56693	-0.37759	-0.14818	0.333735	-0.30165	-0.20684	-0.26112
2.43E-10	2.23E-07	3.69E-12	1.21E-05	0.0964	0.000126	0.000568	0.019638	0.003024
0.199947	0.220468	-0.76378	-0.72392	-0.46568	-0.32361	0.33247	-0.54592	-0.4691
0.024208	0.012749	1.59E-25	6.87E-22	3.45E-08	0.000206	0.000134	3.18E-11	2.65E-08
0.178133	0.117073	-0.59464	-0.60505	-0.3523	-0.22617	0.257507	-0.4119	-0.45305
0.045106	0.18992	1.69E-13	4.92E-14	4.87E-05	0.010563	0.00347	1.5E-06	8.88E-08
-0.16186	0.274078	-0.28802	-0.45754	-0.07554	-0.14335	0.328961	-0.27681	-0.19601
0.069063	0.00182	0.001025	6.38E-08	0.398654	0.107874	0.000159	0.00163	0.027209
0.150754	0.305076	0.204681	-0.0002	0.081664	-0.15586	0.03453	0.295222	0.289619
0.090685	0.000487	0.020981	0.998259	0.361381	0.080163	0.699941	0.000753	0.000958
-0.019	0.175427	-0.038	-0.2311	0.065374	0.049658	-0.15536	0.143948	-0.02552
0.832132	0.048525	0.671489	0.008947	0.465252	0.579287	0.081141	0.106399	0.775758
-0.1507	0.30024	-0.14044	-0.3165	-0.12848	-0.2191	0.166248	-0.09154	-0.15176
0.090808	0.000605	0.115302	0.000289	0.150006	0.013328	0.06176	0.306023	0.088533
0.193204	0.015081	0.402616	0.391346	0.111468	-0.00748	-0.1027	0.340481	0.208685
0.029531	0.866357	2.7E-06	5.38E-06	0.212159	0.933505	0.25056	8.97E-05	0.018548
0.046124	0.153048	0.810522	0.723713	0.573265	0.144458	-0.21399	0.730053	0.679687
0.606607	0.085825	7.98E-31	7.15E-22	1.87E-12	0.105153	0.015701	2.09E-22	1.55E-18
0.424924	-0.46564	0.748494	0.831725	0.802972	0.731472	-0.69107	0.881866	0.835485
6.38E-07	3.46E-08	4.75E-24	9.69E-34	7.13E-30	1.58E-22	2.43E-19	1.23E-42	2.67E-34
0.056976	0.0221	0.76149	0.605459	0.683759	0.351991	-0.48547	0.826764	0.66486
0.524607	0.8052	2.69E-25	4.68E-14	8.08E-19	4.95E-05	7.25E-09	5.05E-33	1.54E-17
-0.22666	-0.30194	0.102451	0.214487	0.118153	0.130727	-0.30037	0.020399	-0.15815
0.010392	0.000561	0.251726	0.015457	0.185838	0.142935	0.000601	0.819928	0.075757
0.041674	-0.34949	0.36976	0.542381	0.181343	0.190692	-0.2076	0.243159	0.25307
0.641786	5.64E-05	1.88E-05	4.51E-11	0.041313	0.031755	0.019182	0.005876	0.004097
-0.26008	-0.0718	-0.44513	-0.29113	-0.52998	-0.313	0.456675	-0.6868	-0.48002
0.003148	0.422465	1.57E-07	0.000898	1.48E-10	0.00034	6.8E-08	4.93E-19	1.13E-08
-0.24932	-0.3763	-0.10616	0.020347	-0.2589	0.064083	0.078145	-0.33902	-0.28331
0.004704	1.3E-05	0.23488	0.820377	0.003292	0.47413	0.382512	9.66E-05	0.001249
0.282084	-0.11615	0.048421	0.190075	0.093562	0.039556	-0.21291	0.141076	0.090518
0.001314	0.193456	0.588785	0.032322	0.295438	0.658819	0.01625	0.113639	0.3115

PLEKHJ1	PLEKHO1	PLSCR3	PMF1	PMPCA	PMVK	PNKP	PNPLA2	PNPLA6
black	black	black	black	black	black	black	black	black
-0.32782	0.124181	-0.12059	-0.0873	-0.29687	0.101836	-0.27982	-0.11857	-0.00166
0.000168	0.164226	0.176871	0.329076	0.000701	0.254597	0.001442	0.184293	0.985243
-0.30675	0.03191	-0.1787	-0.01072	-0.21588	0.173971	-0.32864	-0.16046	-0.03585
0.000452	0.72173	0.044419	0.90482	0.014786	0.050452	0.000162	0.071523	0.689026
0.229692	-0.14495	0.060168	0.257186	0.321848	-0.36047	0.113783	-0.3073	-0.38821
0.009384	0.103971	0.501604	0.003512	0.000224	3.14E-05	0.202758	0.000441	6.49E-06
-0.27107	-0.4556	-0.23595	-0.10583	-0.14272	-0.63818	-0.21155	-0.55596	-0.69513
0.002052	7.36E-08	0.007572	0.236362	0.109447	7.02E-16	0.016959	1.16E-11	1.23E-19
0.231491	0.224667	-0.18666	0.531268	0.068343	0.392677	-0.26521	-0.24035	-0.39796
0.008828	0.011105	0.03562	1.31E-10	0.445186	4.96E-06	0.002584	0.006493	3.6E-06
-0.28852	-0.30609	-0.39546	0.078482	-0.29945	-0.29886	-0.49642	-0.64353	-0.85408
0.001003	0.000466	4.19E-06	0.380454	0.000626	0.000643	2.93E-09	3.37E-16	2.73E-37
-0.33189	-0.22406	-0.40224	-0.08068	-0.15135	-0.35871	-0.36141	-0.52816	-0.54252
0.000138	0.011331	2.76E-06	0.367198	0.089397	3.46E-05	2.99E-05	1.75E-10	4.44E-11
-0.33406	-0.18438	-0.25608	-0.11746	-0.3927	0.085365	-0.11215	-0.14479	-0.28879
0.000124	0.037976	0.003661	0.188458	4.96E-06	0.339959	0.209363	0.104354	0.000992
-0.05708	-0.00523	0.220857	-0.02726	-0.05305	-0.47446	0.320392	0.103893	0.03122
0.523855	0.953466	0.012588	0.760949	0.553651	1.75E-08	0.00024	0.245079	0.727512
-0.16032	0.188965	0.1322	0.057399	-0.42132	0.020993	0.133161	0.201076	0.119537
0.071779	0.033363	0.138448	0.521532	8.11E-07	0.81478	0.135576	0.023402	0.1807
-0.29101	0.031372	-0.03362	-0.08554	-0.4871	-0.13909	-0.0741	-0.0781	-0.11032
0.000903	0.726234	0.70752	0.338946	6.35E-09	0.118871	0.407694	0.382774	0.216935
0.065352	0.085016	0.190122	0.082914	0.33309	-0.15961	0.335211	0.249054	0.410188
0.465403	0.341943	0.032278	0.354058	0.00013	0.073062	0.000117	0.004749	1.67E-06
0.347221	0.166941	0.464728	0.130031	0.404549	-0.11019	0.69019	0.461806	0.539129
6.35E-05	0.060667	3.71E-08	0.145095	2.39E-06	0.217479	2.81E-19	4.63E-08	6.19E-11
0.843838	0.529753	0.627601	0.526152	0.471685	0.583284	0.774663	0.811059	0.741266
1.35E-35	1.51E-10	2.88E-15	2.11E-10	2.17E-08	6.21E-13	1.21E-26	6.81E-31	2.17E-23
0.343073	0.412058	0.581712	0.22154	0.077296	0.240426	0.754288	0.784491	0.767168
7.86E-05	1.48E-06	7.4E-13	0.01231	0.387718	0.006474	1.35E-24	1.04E-27	7.25E-26
-0.03891	0.103154	-0.0371	-0.10715	-0.09932	0.465826	-0.0638	0.260859	0.454732
0.664016	0.248472	0.678818	0.230515	0.2666	3.41E-08	0.4761	0.003055	7.85E-08
0.367147	0.10123	0.112569	0.066846	0.54642	0.194389	0.129751	0.113737	0.332187
2.18E-05	0.257449	0.207648	0.45524	3.03E-11	0.028529	0.14597	0.202941	0.000136
-0.26212	-0.40706	-0.44132	-0.42369	-0.03605	-0.17056	-0.6196	-0.53463	-0.43455
0.002911	2.04E-06	2.07E-07	6.93E-07	0.687424	0.05521	8.1E-15	9.53E-11	3.31E-07
-0.05437	-0.03203	-0.21843	-0.28807	0.146203	0.212609	-0.33022	-0.10642	0.114251
0.54379	0.720705	0.013622	0.001023	0.100972	0.016404	0.00015	0.233704	0.200894
0.229581	0.191638	0.008649	0.274313	0.494783	-0.22656	0.056323	-0.18153	-0.14331
0.009419	0.030902	0.923116	0.001803	3.36E-09	0.010426	0.529378	0.041106	0.107972

POLR2E	POLR2L	POLR3K	POLRMT	POR	PPDPF	PPID	PPP1CA	PPP1R11
black	black	black	black	black	black	black	black	black
-0.15006	-0.16097	-0.06183	-0.20824	-0.28762	-0.12925	0.316667	-0.14411	0.32952
0.092199	0.070613	0.489822	0.018807	0.001042	0.147535	0.000287	0.106011	0.000155
-0.13586	-0.16284	0.049338	-0.22649	-0.28564	-0.08571	0.355332	-0.16835	0.414987
0.127767	0.067378	0.581737	0.01045	0.001133	0.338024	4.14E-05	0.058494	1.23E-06
-0.06462	-0.17261	-0.09618	-0.01928	0.059521	-0.2018	-0.18076	0.104934	-0.17999
0.47044	0.052313	0.282054	0.829688	0.506228	0.022896	0.041983	0.240357	0.04288
-0.60995	-0.49183	-0.38881	-0.44304	-0.17148	-0.63212	0.079599	-0.32742	-0.02685
2.71E-14	4.3E-09	6.26E-06	1.83E-07	0.053898	1.59E-15	0.373692	0.000172	0.764448
-0.12224	0.348257	0.544657	-0.26123	-0.01788	-0.03805	0.097908	0.27651	0.415129
0.170961	6.02E-05	3.61E-11	0.003012	0.841896	0.671045	0.273476	0.00165	1.22E-06
-0.74035	-0.24968	-0.04835	-0.6788	-0.2572	-0.64845	0.247886	-0.23512	0.332239
2.63E-23	0.004642	0.589355	1.79E-18	0.003511	1.69E-16	0.004956	0.007794	0.000136
-0.49344	-0.2767	-0.09813	-0.50624	-0.12936	-0.50651	0.247355	-0.23135	0.429125
3.76E-09	0.001638	0.272403	1.26E-09	0.147208	1.24E-09	0.005052	0.008872	4.81E-07
-0.38492	-0.0947	-0.23354	-0.26957	-0.14558	-0.31912	-0.01323	-0.29269	0.12354
7.88E-06	0.289586	0.008229	0.002178	0.102449	0.000255	0.882621	0.00084	0.16643
-0.1221	-0.04733	-0.38849	0.070692	0.206004	-0.05954	-0.1368	0.205679	-0.12308
0.171451	0.597183	6.38E-06	0.429665	0.020148	0.506086	0.12511	0.02035	0.168026
-0.18548	0.189145	-0.28424	-0.03659	0.039511	0.011956	-0.20175	0.240163	0.030527
0.036823	0.033192	0.001201	0.683002	0.659189	0.893866	0.022933	0.006535	0.733331
-0.36239	-0.03662	-0.40114	-0.21189	-0.00964	-0.25981	-0.04233	-0.05171	0.094087
2.83E-05	0.68272	2.96E-06	0.016778	0.914337	0.00318	0.636543	0.563717	0.292728
0.253547	-0.04736	-0.07068	0.265304	0.303571	0.276285	0.109683	0.204629	-0.04849
0.004025	0.596949	0.429708	0.002574	0.000521	0.001665	0.219614	0.021015	0.588224
0.537556	0.036235	-0.249	0.658004	0.33798	0.429297	-0.43251	0.274309	-0.48381
7.2E-11	0.685883	0.004758	4.27E-17	0.000102	4.75E-07	3.81E-07	0.001803	8.3E-09
0.871122	0.762374	0.336994	0.89583	0.639532	0.887286	-0.56233	0.725198	-0.42341
2.01E-40	2.2E-25	0.000107	7.43E-46	5.84E-16	7.8E-44	6E-12	5.37E-22	7.06E-07
0.532452	0.430809	-0.21392	0.710712	0.422868	0.652764	-0.57531	0.52415	-0.41535
1.17E-10	4.28E-07	0.01574	8.02E-21	7.32E-07	9.13E-17	1.5E-12	2.54E-10	1.2E-06
0.279545	0.112708	0.182955	0.173597	-0.19864	0.354864	-0.05614	0.020995	0.020674
0.001458	0.207084	0.039511	0.050957	0.02517	4.25E-05	0.530744	0.814764	0.817547
0.576828	0.058114	0.25812	0.368464	0.06667	0.335676	-0.11549	0.105579	-0.2102
1.27E-12	0.51635	0.00339	2.02E-05	0.456435	0.000114	0.196019	0.237461	0.017696
-0.27163	-0.35761	0.20767	-0.46644	-0.35302	-0.43154	0.491147	-0.5242	0.150655
0.002007	3.67E-05	0.01914	3.26E-08	4.69E-05	4.08E-07	4.55E-09	2.53E-10	0.0909
0.211481	-0.05148	0.360477	-0.07022	-0.16926	0.033554	0.35465	-0.2386	0.179577
0.016997	0.56543	3.14E-05	0.43276	0.057125	0.708034	4.3E-05	0.006904	0.043366
0.153998	-0.20915	0.221539	0.070085	-0.16785	0.025346	0.058666	0.184751	-0.12378
0.083875	0.018284	0.01231	0.433644	0.059263	0.777288	0.512362	0.037581	0.165592

PPP1R16A	PPP1R35	PPP2R2A	PPP4C	PPP6C	PQBP1	PQLC2	PRCC	PRDM4
black	black	black	black	black	black	black	black	black
-0.28365	-0.32799	0.255982	-0.33778	0.166525	0.074269	-0.16839	-0.36375	0.033532
0.001231	0.000167	0.003675	0.000103	0.061321	0.406631	0.058431	2.63E-05	0.708214
-0.30199	-0.24682	0.289226	-0.32856	0.23546	0.135568	-0.18221	-0.33188	0.048631
0.000559	0.005151	0.000974	0.000162	0.007702	0.128589	0.040333	0.000138	0.587166
-0.09486	0.243449	0.001067	0.393625	0.051681	-0.20458	0.139915	0.257954	0.277519
0.288775	0.005815	0.990502	4.69E-06	0.563911	0.021043	0.116671	0.003412	0.001584
-0.45567	-0.04468	0.169555	0.040792	0.335678	-0.6192	-0.18793	-0.30507	0.442399
7.32E-08	0.617902	0.056687	0.648857	0.000114	8.52E-15	0.034358	0.000488	1.91E-07
-0.20752	0.306436	0.193962	0.380341	0.145025	0.499001	0.056331	-0.10649	-0.15729
0.01923	0.000459	0.028888	1.03E-05	0.103779	2.36E-09	0.529326	0.233395	0.077384
-0.62885	-0.02448	0.319959	0.092915	0.48065	-0.32927	-0.23192	-0.5347	0.288778
2.45E-15	0.78472	0.000245	0.298805	1.07E-08	0.000157	0.0087	9.47E-11	0.000993
-0.53427	-0.09519	0.245157	-0.07112	0.361545	-0.26048	-0.17228	-0.49433	0.357553
9.86E-11	0.287057	0.00547	0.42689	2.96E-05	0.0031	0.052765	3.49E-09	3.68E-05
-0.01608	-0.2269	-0.02934	-0.1141	0.068113	-0.16955	-0.18182	-0.27715	-0.1742
0.857628	0.010308	0.743343	0.201512	0.446725	0.056694	0.04077	0.001608	0.050146
0.014447	0.171857	-0.13922	0.32265	-0.1434	-0.51402	0.195142	-0.02944	0.362142
0.871927	0.053361	0.118516	0.000216	0.107743	6.37E-10	0.027908	0.742471	2.87E-05
-0.08922	-0.01225	-0.04401	0.197105	-0.06816	-0.16593	0.19146	-0.28344	-0.13182
0.318526	0.891233	0.623201	0.026342	0.446441	0.062268	0.03106	0.001242	0.139595
-0.24397	-0.12903	0.060206	-0.01535	0.077303	-0.33841	-0.01502	-0.33599	-0.00622
0.005708	0.148229	0.501335	0.86401	0.387675	9.96E-05	0.866875	0.000113	0.944645
0.325275	0.376551	0.039239	0.024879	-0.11247	0.015211	0.04602	0.34719	0.087432
0.00019	1.28E-05	0.661387	0.781288	0.208047	0.865221	0.607414	6.36E-05	0.32835
0.518276	0.258308	-0.35239	0.256838	-0.48168	0.008824	0.411291	0.619872	-0.00621
4.35E-10	0.003366	4.84E-05	0.003559	9.85E-09	0.921569	1.56E-06	7.82E-15	0.94476
0.831543	0.673314	-0.48015	0.683188	-0.72017	0.626788	0.729168	0.817061	-0.50433
1.03E-33	4.24E-18	1.11E-08	8.86E-19	1.4E-21	3.2E-15	2.49E-22	1.11E-31	1.49E-09
0.594444	0.315981	-0.40819	0.416735	-0.59731	0.130468	0.611318	0.452165	-0.33984
1.73E-13	0.000296	1.9E-06	1.1E-06	1.24E-13	0.143736	2.29E-14	9.47E-08	9.27E-05
0.11439	-0.23801	0.222426	-0.33071	0.06144	0.389201	-0.02317	-0.01307	-0.4778
0.200343	0.007049	0.011958	0.000146	0.49259	6.12E-06	0.795948	0.884023	1.34E-08
0.339019	0.040708	0.003875	-0.05861	-0.10741	0.419502	0.001373	0.53201	-0.23073
9.66E-05	0.649533	0.965514	0.512736	0.229389	9.14E-07	0.987777	1.22E-10	0.009058
-0.3612	-0.39776	0.281693	-0.55983	0.457418	-0.11928	-0.58889	-0.29878	0.204273
3.02E-05	3.64E-06	0.001335	7.78E-12	6.43E-08	0.181661	3.29E-13	0.000645	0.021244
0.01434	-0.31935	0.096415	-0.50542	0.132103	0.307384	-0.39404	-0.00219	-0.06076
0.872867	0.000253	0.280897	1.36E-09	0.13874	0.000439	4.57E-06	0.980539	0.497427
-0.06146	0.12153	0.251792	0.154668	0.018352	0.217791	0.001792	0.247924	0.256547
0.492441	0.173487	0.004295	0.08252	0.837735	0.013905	0.984045	0.004949	0.003598

PRDX5	PRKAB2	PRMT1	PRMT7	PRMT9	PROB1	PRPF31	PRPF6	PRR14
black	black	black	black	black	black	black	black	black
0.054907	0.139026	-0.39142	-0.26385	0.154141	-0.22212	-0.31472	-0.01981	-0.19523
0.539797	0.119036	5.35E-06	0.002723	0.083583	0.012079	0.000314	0.825008	0.027838
0.096743	0.158724	-0.33461	-0.24094	0.154339	-0.32874	-0.33256	-0.03669	-0.22098
0.279253	0.07469	0.000121	0.006357	0.083182	0.000161	0.000133	0.682131	0.012536
-0.34471	0.056729	0.43743	0.175213	0.108349	-0.03105	0.19933	-0.17668	0.231605
7.23E-05	0.526412	2.71E-07	0.048805	0.225307	0.728921	0.024658	0.046916	0.008794
-0.5	0.337581	-0.07479	-0.24279	0.495374	-0.15979	-0.23732	-0.4527	-0.22033
2.16E-09	0.000104	0.403316	0.005954	3.2E-09	0.072738	0.007219	9.11E-08	0.012805
0.622816	0.072057	0.172728	0.008963	0.159051	-0.40742	-0.24727	-0.27356	-0.05836
5.36E-15	0.420783	0.052146	0.920338	0.074086	1.99E-06	0.005068	0.001859	0.514604
-0.02908	0.366692	-0.22988	-0.39794	0.610684	-0.41616	-0.55653	-0.63134	-0.41558
0.745513	2.23E-05	0.009324	3.6E-06	2.47E-14	1.14E-06	1.09E-11	1.76E-15	1.18E-06
-0.09791	0.336521	-0.05216	-0.2267	0.4201	-0.42843	-0.33586	-0.36735	-0.33463
0.273484	0.00011	0.560301	0.01038	8.79E-07	5.04E-07	0.000113	2.15E-05	0.00012
0.206154	0.021436	-0.43681	-0.18498	0.174453	0.220809	-0.36053	-0.16857	-0.23138
0.020056	0.810946	2.83E-07	0.037339	0.049807	0.012608	3.13E-05	0.058154	0.008861
-0.41687	-0.03568	0.129398	-0.26328	-0.04203	0.080355	0.31167	0.049801	0.168434
1.09E-06	0.690435	0.147079	0.002785	0.638918	0.369159	0.000361	0.578194	0.058366
0.069562	-0.12746	-0.30041	-0.35769	-0.06076	0.104108	-0.00486	0.100013	0.098503
0.437088	0.153283	0.0006	3.65E-05	0.49741	0.244097	0.95678	0.263244	0.270557
-0.115	0.017659	-0.43479	-0.35334	0.189559	0.085183	-0.18572	-0.01887	-0.10476
0.197953	0.843782	3.26E-07	4.61E-05	0.032803	0.340991	0.036572	0.833242	0.241149
-0.38333	-0.02011	0.249558	0.195492	-0.31013	-0.07364	0.556258	0.462484	0.24749
8.66E-06	0.822448	0.004662	0.027623	0.000388	0.410645	1.12E-11	4.4E-08	0.005027
-0.36456	-0.25688	0.490311	0.435721	-0.51978	0.447293	0.766907	0.570437	0.664723
2.51E-05	0.003553	4.88E-09	3.06E-07	3.8E-10	1.35E-07	7.71E-26	2.54E-12	1.58E-17
0.450819	-0.51238	0.666062	0.55083	-0.74134	0.516419	0.812172	0.642413	0.80252
1.05E-07	7.37E-10	1.29E-17	1.95E-11	2.14E-23	5.14E-10	4.88E-31	3.93E-16	8.11E-30
0.022458	-0.43961	0.219933	0.192859	-0.66844	0.561944	0.716901	0.690712	0.705034
0.802105	2.33E-07	0.012973	0.029829	8.98E-18	6.24E-12	2.58E-21	2.58E-19	2.21E-20
0.245098	-0.17253	-0.22283	0.089848	-0.31022	0.06182	-0.02708	0.262086	-0.01051
0.005481	0.052414	0.0118	0.315108	0.000386	0.489912	0.762516	0.002915	0.90668
0.006555	-0.13779	0.43578	0.534191	-0.2851	0.104887	0.327507	0.207234	0.214359
0.941692	0.122382	3.04E-07	9.94E-11	0.001159	0.240568	0.000171	0.0194	0.01552
-0.05549	0.291517	-0.24904	-0.09946	0.495771	-0.31853	-0.57555	-0.51471	-0.65532
0.535516	0.000883	0.004751	0.265916	3.1E-09	0.000263	1.46E-12	5.99E-10	6.32E-17
0.207014	0.045987	-0.07123	0.145343	0.05993	-0.196	-0.25735	-0.09939	-0.36011
0.019532	0.607678	0.426124	0.103016	0.503301	0.027212	0.00349	0.266229	3.2E-05
-0.17681	0.073816	0.509422	0.333311	-0.06879	-0.29226	0.239816	-0.06273	0.280153
0.046749	0.409508	9.58E-10	0.000129	0.442204	0.000856	0.006615	0.483543	0.001422

PRR22	PRR7	PRSS27	PRX	PSMC4	PTCD3	PTDSS2	PTGER1	PTMA
black	black	black	black	black	black	black	black	black
-0.15313	-0.31962	-0.1876	-0.05473	-0.00297	-0.00055	-0.15777	-0.0727	0.041152
0.085649	0.000249	0.034688	0.541101	0.973601	0.995079	0.076481	0.416671	0.645966
-0.14705	-0.34739	-0.15404	-0.05531	0.036557	0.055942	-0.08171	-0.07324	2.47E-05
0.099	6.29E-05	0.083781	0.536814	0.683247	0.532172	0.361128	0.413165	0.99978
0.048717	-0.05761	0.387488	-0.19752	-0.05456	0.25809	-0.09797	-0.05967	-0.00339
0.586507	0.520007	6.77E-06	0.02602	0.542376	0.003394	0.273182	0.50519	0.969812
-0.30944	-0.3771	-0.07256	-0.38263	-0.38454	0.523925	-0.4779	-0.30125	-0.23992
0.0004	1.24E-05	0.417527	9.02E-06	8.06E-06	2.6E-10	1.33E-08	0.000578	0.00659
-0.21172	-0.17309	0.025944	-0.1766	-0.02821	0.251489	0.05669	-0.10651	0.051818
0.016868	0.051646	0.772174	0.047017	0.752872	0.004343	0.526693	0.233334	0.562878
-0.54272	-0.53721	-0.25339	-0.46155	-0.44025	0.605403	-0.49961	-0.38535	-0.25311
4.36E-11	7.45E-11	0.004048	4.72E-08	2.23E-07	4.71E-14	2.24E-09	7.69E-06	0.004091
-0.31622	-0.52058	-0.11074	-0.26065	-0.18661	0.411411	-0.27102	-0.34495	-0.12133
0.000293	3.53E-10	0.215177	0.003079	0.035665	1.55E-06	0.002057	7.14E-05	0.174216
-0.2721	-0.16445	-0.32687	-0.26557	-0.27074	0.228549	-0.26772	-0.2827	-0.07538
0.00197	0.064669	0.000176	0.002548	0.00208	0.009753	0.002342	0.00128	0.399604
0.100956	-0.05802	0.07516	0.049176	0.141368	-0.18373	-0.33449	0.119093	0.098397
0.258747	0.517012	0.40101	0.582977	0.112885	0.038671	0.000121	0.182336	0.271078
0.079301	-0.19857	-0.20443	-0.03197	0.095848	-0.0963	-0.20038	0.131633	0.199961
0.37549	0.025223	0.021145	0.721205	0.283747	0.281467	0.023895	0.140163	0.024198
-0.09733	-0.31935	-0.22677	-0.13833	-0.04042	0.118334	-0.29465	-0.10101	-0.01475
0.276348	0.000253	0.010353	0.120913	0.651887	0.185162	0.000772	0.258509	0.8693
0.366788	0.192601	0.250663	0.279489	0.423	-0.24068	0.319609	0.225004	0.051866
2.22E-05	0.030052	0.004477	0.001462	7.25E-07	0.006416	0.00025	0.010981	0.562517
0.655245	0.415979	0.510605	0.316272	0.394248	-0.35686	0.350095	0.371234	0.323282
6.38E-17	1.15E-06	8.63E-10	0.000292	4.51E-06	3.82E-05	5.46E-05	1.73E-05	0.00021
0.790775	0.768557	0.590371	0.415771	0.587872	-0.55892	0.707626	0.609559	0.538621
2.02E-28	5.23E-26	2.78E-13	1.17E-06	3.69E-13	8.54E-12	1.4E-20	2.84E-14	6.5E-11
0.729624	0.440828	0.29677	0.371799	0.500784	-0.52899	0.365884	0.557429	0.549127
2.27E-22	2.14E-07	0.000704	1.68E-05	2.02E-09	1.62E-10	2.34E-05	9.97E-12	2.31E-11
0.181413	0.137467	-0.18319	0.115037	0.190779	-0.10358	0.412923	0.194074	0.324383
0.041233	0.123272	0.03925	0.197792	0.031676	0.246501	1.4E-06	0.028793	0.000199
0.27385	0.391542	0.31987	0.097598	0.264316	-0.15593	0.499149	0.125837	0.104844
0.001837	5.32E-06	0.000246	0.275006	0.002675	0.080013	2.33E-09	0.15863	0.240763
-0.54972	-0.17693	-0.2909	-0.22207	-0.44136	0.277878	-0.23544	-0.40531	-0.51373
2.18E-11	0.046604	0.000907	0.012099	2.06E-07	0.001561	0.007708	2.28E-06	6.54E-10
-0.24069	0.07541	-0.14356	0.069632	-0.03958	-0.10485	0.136737	-0.17388	-0.2949
0.006415	0.399436	0.107358	0.436627	0.658646	0.240742	0.125296	0.05058	0.000764
0.132533	0.019382	0.368873	0.020467	-0.01798	0.04089	0.064371	0.046233	0.158016
0.137446	0.828765	1.98E-05	0.819342	0.841011	0.648068	0.472144	0.605753	0.076013

PTMS	PTPDC1	PUF60	PUS1	PUSL1	QTRT1	R3HCC1	R3HDM4	RAB11A
black	black	black	black	black	black	black	black	black
-0.15444	0.245684	-0.20492	-0.15748	-0.39159	-0.29461	-0.04109	-0.20666	0.166055
0.082985	0.005367	0.020828	0.077034	5.3E-06	0.000773	0.646434	0.019744	0.062066
-0.11854	0.288935	-0.17564	-0.1614	-0.33528	-0.29795	0.030123	-0.20722	0.258918
0.184373	0.000986	0.048249	0.069864	0.000117	0.000669	0.736725	0.019408	0.003289
0.225273	-0.22277	0.060028	0.126569	0.342088	0.123945	-0.01829	0.098241	-0.17321
0.010884	0.011824	0.502607	0.1562	8.27E-05	0.165033	0.83825	0.27184	0.051486
-0.36002	0.075431	-0.40599	-0.17372	-0.13477	-0.24655	-0.40355	-0.39075	0.088729
3.22E-05	0.399309	2.18E-06	0.050795	0.130881	0.005201	2.55E-06	5.57E-06	0.321198
0.026255	0.11607	0.214751	-0.0367	0.077254	-0.17568	0.454955	-0.23041	0.069252
0.769516	0.193769	0.015327	0.682099	0.387978	0.048191	7.72E-08	0.009158	0.439144
-0.48025	0.248519	-0.39419	-0.28215	-0.27431	-0.50057	-0.19316	-0.63339	0.297719
1.11E-08	0.004843	4.53E-06	0.00131	0.001803	2.06E-09	0.029567	1.34E-15	0.000675
-0.3331	0.256291	-0.22235	-0.23549	-0.38618	-0.36484	-0.12851	-0.39976	0.207948
0.00013	0.003632	0.011988	0.007693	7.32E-06	2.47E-05	0.149894	3.22E-06	0.018977
-0.41645	0.164307	-0.14166	-0.24522	-0.27396	-0.19961	-0.27567	-0.5077	0.098393
1.12E-06	0.064906	0.11213	0.005458	0.001829	0.024451	0.001707	1.11E-09	0.271095
-0.09266	-0.24126	-0.21998	0.280825	0.093837	0.009183	-0.36171	0.221407	-0.26168
0.300156	0.006287	0.012953	0.001384	0.294016	0.91839	2.94E-05	0.012364	0.002961
-0.25758	-0.0373	-0.09117	0.238033	-0.15551	-0.08385	-0.15109	-0.1027	-0.07699
0.00346	0.677184	0.307998	0.007043	0.080844	0.348617	0.089966	0.25056	0.389582
-0.33392	-0.03807	-0.23129	0.015824	-0.30602	-0.16679	-0.30582	-0.24001	0.054746
0.000125	0.670911	0.00889	0.859838	0.000467	0.060907	0.000471	0.006571	0.540989
0.186922	-0.13225	0.219245	0.218503	0.175013	0.299378	0.131287	0.395397	-0.24477
0.035356	0.138289	0.013266	0.013589	0.049067	0.000628	0.141215	4.21E-06	0.005546
0.492555	-0.39707	0.353155	0.462604	0.493161	0.607776	0.056396	0.634583	-0.53621
4.05E-09	3.8E-06	4.65E-05	4.36E-08	3.85E-09	3.53E-14	0.528846	1.14E-15	8.2E-11
0.639619	-0.49415	0.764732	0.623077	0.809978	0.785327	0.573109	0.794658	-0.66999
5.77E-16	3.55E-09	1.28E-25	5.19E-15	9.38E-31	8.39E-28	1.91E-12	7.15E-29	7.08E-18
0.31759	-0.36217	0.407331	0.610843	0.427596	0.561428	0.136968	0.57743	-0.56146
0.000274	2.86E-05	2.01E-06	2.43E-14	5.33E-07	6.59E-12	0.124653	1.19E-12	6.56E-12
0.025567	0.32925	0.245902	-0.02783	-0.09188	0.072748	0.367145	-0.00873	0.122779
0.7754	0.000157	0.005325	0.75612	0.304251	0.416336	2.18E-05	0.92243	0.169076
0.514697	-0.14436	0.466256	-0.00372	0.300397	0.368767	0.409267	0.409791	-0.09882
6E-10	0.105387	3.3E-08	0.966905	0.0006	1.99E-05	1.77E-06	1.72E-06	0.269031
-0.21625	0.296357	-0.35392	-0.57429	-0.36573	-0.38825	-0.16527	-0.36461	0.525202
0.014611	0.000717	4.47E-05	1.67E-12	2.36E-05	6.47E-06	0.063324	2.5E-05	2.31E-10
0.113196	0.140406	0.006864	-0.35506	-0.2132	-0.11819	0.11495	-0.00347	0.349201
0.205113	0.115381	0.938948	4.2E-05	0.016099	0.185699	0.198137	0.969143	5.73E-05
0.343093	0.069781	0.169377	0.211933	0.29495	0.061177	0.235791	0.16617	-0.22521
7.85E-05	0.435643	0.056951	0.016758	0.000762	0.494444	0.007615	0.061884	0.010906

RAB11B	RAB14	RAB22A	RAB3GAP1	RAB43	RAB5A	RAB5C	RABAC1	RABEP2
black	black	black	black	black	black	black	black	black
-0.14736	0.015367	0.036247	0.229536	-0.2857	-0.07535	-0.09592	-0.10121	-0.15881
0.098269	0.863847	0.685784	0.009434	0.00113	0.399797	0.283386	0.25754	0.074539
-0.14994	0.05337	0.028285	0.234497	-0.38342	0.01535	-0.09899	-0.09196	-0.21554
0.092458	0.551224	0.752257	0.007963	8.61E-06	0.863998	0.26819	0.30381	0.014945
-0.12695	0.154879	0.089673	-0.24001	0.041296	0.27587	-0.09017	-0.31219	-0.08519
0.154962	0.082097	0.316055	0.00657	0.644814	0.001693	0.313372	0.000353	0.340966
-0.59346	0.325767	0.535049	-0.02368	-0.27306	0.458684	-0.43879	-0.66554	-0.40086
1.94E-13	0.000186	9.16E-11	0.791553	0.001896	5.85E-08	2.47E-07	1.39E-17	3.01E-06
-0.30962	-0.17723	-0.00751	-0.44701	-0.39358	-0.18614	-0.06303	0.160624	-0.19333
0.000397	0.046218	0.933196	1.38E-07	4.7E-06	0.036142	0.481406	0.071236	0.029425
-0.82588	0.243978	0.552731	-0.14819	-0.5512	0.282908	-0.50591	-0.53256	-0.53524
6.75E-33	0.005706	1.61E-11	0.096376	1.88E-11	0.001269	1.3E-09	1.16E-10	8.99E-11
-0.54422	0.174741	0.432013	0.008196	-0.53809	0.406009	-0.27023	-0.42451	-0.43633
3.76E-11	0.049425	3.95E-07	0.927132	6.84E-11	2.18E-06	0.002122	6.56E-07	2.93E-07
-0.37622	-0.06108	0.232812	0.087814	-0.25874	-0.04194	-0.34102	-0.22032	-0.27236
1.31E-05	0.49512	0.008438	0.326234	0.003312	0.639659	8.73E-05	0.012811	0.00195
-0.00813	0.015118	0.057507	-0.14736	0.224593	0.155207	0.190121	-0.15707	0.22634
0.927746	0.866033	0.520747	0.098266	0.011132	0.081444	0.032279	0.077813	0.010504
-0.1082	-0.13928	-0.0559	-0.22082	0.061605	-0.07744	0.220754	0.049095	0.223586
0.225964	0.11836	0.532485	0.012602	0.491428	0.386846	0.012631	0.583602	0.011509
-0.30661	0.003636	0.191195	-0.00814	-0.02879	0.022657	-0.04954	-0.22308	-0.05473
0.000455	0.967642	0.031298	0.927673	0.74798	0.800395	0.580227	0.011705	0.54114
0.481236	-0.10078	-0.27517	0.096653	0.254316	-0.00464	0.506386	0.126913	0.169117
1.02E-08	0.25958	0.001742	0.279704	0.003911	0.958688	1.25E-09	0.155068	0.057338
0.678403	-0.18964	-0.4077	-0.10715	0.575155	0.024836	0.438664	0.188966	0.521515
1.91E-18	0.03273	1.96E-06	0.230517	1.53E-12	0.781658	2.49E-07	0.033363	3.24E-10
0.850432	-0.57557	-0.63151	-0.62285	0.718368	-0.47047	0.692723	0.858696	0.833512
1.13E-36	1.46E-12	1.72E-15	5.34E-15	1.96E-21	2.39E-08	1.84E-19	4.24E-38	5.27E-34
0.703109	-0.40314	-0.52958	-0.35352	0.664785	-0.22533	0.650496	0.508883	0.801579
3.11E-20	2.61E-06	1.54E-10	4.56E-05	1.56E-17	0.010861	1.26E-16	1E-09	1.06E-29
0.3466	0.011471	-0.34565	0.110741	-0.00269	-0.12196	0.201018	0.366817	0.206197
6.56E-05	0.898153	6.89E-05	0.215172	0.976059	0.171967	0.023443	2.22E-05	0.020029
0.526662	0.019493	-0.38369	0.090237	0.212956	-0.02124	0.253686	0.319658	0.115083
2.02E-10	0.827802	8.47E-06	0.313013	0.016225	0.812673	0.004004	0.000249	0.197612
-0.4166	0.437813	0.437745	0.465239	-0.38099	0.122092	-0.53513	-0.31195	-0.57663
1.11E-06	2.64E-07	2.65E-07	3.57E-08	9.92E-06	0.171491	9.09E-11	0.000357	1.3E-12
0.084646	0.120016	-0.04127	0.472706	-0.16247	-0.15112	-0.12092	0.134692	-0.26529
0.344058	0.178947	0.645058	2.01E-08	0.068006	0.089901	0.175684	0.131098	0.002576
0.16508	0.061228	-0.19798	-0.14138	-0.05514	0.166585	0.024669	-0.10403	-0.00418
0.063638	0.494086	0.025666	0.112842	0.53806	0.061227	0.78309	0.244464	0.962832

RABL3	RAD23A	RAD9A	RADIL	RALGAPB	RALY	RAMP1	RAMP2	RANBP2
black	black	black	black	black	black	black	black	black
0.088843	0.001982	-0.14916	-0.38441	-0.06396	-0.15269	-0.20431	-0.26547	0.279225
0.320574	0.982358	0.094184	8.12E-06	0.474975	0.086571	0.021219	0.002558	0.001477
0.08574	0.018764	-0.20701	-0.32545	-0.0765	-0.14534	-0.12103	-0.21261	0.18506
0.337833	0.834142	0.019535	0.000189	0.392626	0.103017	0.17528	0.016404	0.037258
0.047465	-0.17126	0.37663	0.166986	0.305994	0.336917	-0.19292	0.185711	-0.13857
0.596175	0.054209	1.28E-05	0.060596	0.000468	0.000107	0.029774	0.036584	0.120276
0.424765	-0.55694	0.054007	-0.26513	0.547005	-0.09096	-0.3382	-0.21549	0.212634
6.45E-07	1.05E-11	0.546473	0.002592	2.86E-11	0.309121	0.000101	0.014969	0.016392
0.162556	-0.24969	0.039109	-0.22288	-0.51691	0.285417	0.302138	0.123967	-0.28863
0.067855	0.00464	0.662438	0.011779	4.92E-10	0.001143	0.000556	0.164959	0.000999
0.570219	-0.73259	-0.11742	-0.52093	0.156419	-0.09399	-0.13904	-0.29705	0.178722
2.6E-12	1.26E-22	0.188597	3.42E-10	0.079063	0.293248	0.118992	0.000696	0.044389
0.34272	-0.42997	-0.0841	-0.48895	0.213794	-0.07916	-0.0664	-0.2257	0.032404
8.01E-05	4.54E-07	0.347196	5.46E-09	0.015801	0.376347	0.458267	0.010729	0.717606
0.421331	-0.38277	-0.11925	-0.22838	0.095651	-0.29415	0.064051	-0.24979	0.291295
8.1E-07	8.94E-06	0.181741	0.00981	0.28474	0.000789	0.474355	0.004622	0.000892
-0.13008	0.054245	0.193101	-0.10741	0.296639	0.248949	-0.25486	-0.02548	0.120117
0.144933	0.544707	0.02962	0.229382	0.000708	0.004767	0.003832	0.776145	0.178579
-0.00214	-0.06856	0.076014	-0.41708	-0.14327	0.150818	0.078084	-0.20947	0.273497
0.980945	0.443725	0.395659	1.07E-06	0.108069	0.090546	0.382881	0.018102	0.001863
0.234656	-0.20714	-0.00546	-0.36993	0.088286	-0.09191	-0.1048	-0.40266	0.362861
0.00792	0.019457	0.951433	1.86E-05	0.32363	0.304095	0.240959	2.69E-06	2.76E-05
-0.37484	0.63325	0.152548	0.255132	0.130815	0.137001	0.022101	0.148604	-0.26605
1.41E-05	1.36E-15	0.086865	0.003794	0.142663	0.124562	0.805191	0.095435	0.002501
-0.48707	0.576109	0.510741	0.522559	0.201904	0.419634	-0.06927	0.373052	-0.20565
6.37E-09	1.37E-12	8.53E-10	2.95E-10	0.022826	9.07E-07	0.43905	1.56E-05	0.020367
-0.61296	0.748904	0.528559	0.673335	-0.5703	0.71012	0.457303	0.563119	-0.57468
1.87E-14	4.35E-24	1.69E-10	4.22E-18	2.58E-12	8.93E-21	6.49E-08	5.52E-12	1.61E-12
-0.52531	0.599542	0.449156	0.303607	-0.21227	0.523993	0.176379	0.261613	-0.1045
2.29E-10	9.52E-14	1.18E-07	0.000521	0.016579	2.58E-10	0.047298	0.002968	0.242299
-0.17047	0.237858	-0.22827	0.03558	-0.33037	-0.1285	0.209684	0.092173	-0.00463
0.055342	0.007086	0.009847	0.691276	0.000149	0.149937	0.01798	0.302701	0.958786
-0.31265	0.402565	0.023175	0.52999	-0.15849	0.034812	0.152143	0.408169	-0.36607
0.000345	2.71E-06	0.795925	1.48E-10	0.075132	0.697609	0.087717	1.9E-06	2.31E-05
0.343265	-0.38609	-0.49956	-0.08705	0.200562	-0.62321	-0.15753	-0.22877	0.122151
7.79E-05	7.36E-06	2.25E-09	0.330492	0.023766	5.1E-15	0.076922	0.009682	0.171285
-0.03657	0.100812	-0.42186	0.131725	-0.12226	-0.45273	0.07771	-0.00471	-0.06813
0.683104	0.259432	7.83E-07	0.139882	0.170908	9.09E-08	0.385171	0.958079	0.446642
-0.24012	0.071327	0.408281	0.067954	0.072163	0.355074	-0.22733	0.355447	-0.19385
0.006544	0.425521	1.89E-06	0.44779	0.420102	4.2E-05	0.01016	4.12E-05	0.028979

RANBP3	RANBP6	RANBP9	RAPGEF6	RARB	RASAL2	RASSF7	RBM18	RBM38
black	black	black	black	black	black	black	black	black
-0.17502	0.080992	-0.02897	0.178961	0.053789	-0.0146	-0.00054	-0.0165	-0.17187
0.049059	0.36536	0.746496	0.044101	0.548099	0.870565	0.995182	0.853926	0.053348
-0.19803	0.09499	-0.01371	0.14689	0.038542	-0.04712	-0.01287	0.058496	-0.23369
0.025628	0.288096	0.878387	0.099363	0.667038	0.598817	0.885824	0.513591	0.008187
-0.02053	0.07153	0.148617	-0.16016	0.129935	0.056028	-0.17316	0.296737	0.073124
0.818786	0.4242	0.095406	0.072059	0.145394	0.531542	0.051556	0.000705	0.41392
-0.50492	0.401356	0.427275	0.230204	0.323031	0.1683	-0.27247	0.479422	-0.11534
1.42E-09	2.92E-06	5.45E-07	0.009223	0.000212	0.058569	0.001941	1.18E-08	0.196592
-0.30986	0.088703	-0.08159	-0.15622	0.149069	-0.37719	0.404915	0.332772	-0.04613
0.000392	0.321341	0.361826	0.079458	0.094391	1.23E-05	2.34E-06	0.000132	0.606578
-0.77597	0.50054	0.412076	0.228701	0.434788	-0.05345	0.039239	0.638984	-0.22226
8.8E-27	2.07E-09	1.48E-06	0.009703	3.26E-07	0.550626	0.661391	6.29E-16	0.012024
-0.41152	0.162934	0.258173	0.10513	0.435288	0.050214	-0.14342	0.44749	-0.2435
1.54E-06	0.06721	0.003384	0.239472	3.15E-07	0.575042	0.107704	1.33E-07	0.005804
-0.34156	0.190651	0.177889	0.107346	0.020303	0.015001	0.073997	0.003036	0.068959
8.49E-05	0.031792	0.045406	0.229657	0.820764	0.867065	0.408356	0.972975	0.441086
0.036558	-0.11311	0.101229	-0.24901	0.201216	0.124963	-0.08713	-0.08074	0.110689
0.683238	0.205455	0.257456	0.004757	0.023303	0.161564	0.330013	0.366846	0.215389
-0.09093	-0.12015	-0.07868	-0.07849	-0.08162	-0.20467	0.150575	-0.31353	0.201496
0.30931	0.178454	0.379281	0.380425	0.361648	0.020989	0.091072	0.000332	0.023108
-0.28181	0.108247	0.142137	0.133071	0.027775	-0.1375	0.023691	-0.13358	0.067875
0.001328	0.225749	0.11092	0.135843	0.756582	0.123184	0.791485	0.134342	0.448314
0.456505	-0.36915	-0.31782	-0.07097	-0.21932	0.020441	-0.28953	-0.29516	0.038358
6.88E-08	1.95E-05	0.000271	0.427864	0.013232	0.819563	0.000961	0.000755	0.668535
0.734453	-0.51122	-0.32821	-0.35954	-0.36968	0.051575	-0.22129	-0.49694	0.404272
8.71E-23	8.18E-10	0.000165	3.3E-05	1.89E-05	0.564709	0.012413	2.8E-09	2.43E-06
0.812452	-0.56054	-0.60301	-0.60453	-0.56104	-0.48244	0.414845	-0.65424	0.660278
4.49E-31	7.23E-12	6.29E-14	5.24E-14	6.86E-12	9.27E-09	1.24E-06	7.39E-17	3.06E-17
0.691919	-0.60597	-0.47746	-0.43716	-0.52396	-0.25995	0.115747	-0.80659	0.606163
2.11E-19	4.4E-14	1.38E-08	2.77E-07	2.59E-10	0.003163	0.195022	2.53E-30	4.3E-14
0.244747	-0.16131	-0.28306	0.15551	-0.38485	-0.15732	-0.044	-0.34882	-0.03499
0.005551	0.070018	0.001262	0.080843	7.92E-06	0.077326	0.623266	5.84E-05	0.696162
0.511712	-0.18393	-0.23361	-0.12567	-0.26879	0.064334	-0.23154	-0.11305	0.022639
7.83E-10	0.038451	0.008211	0.159183	0.002246	0.4724	0.008813	0.205702	0.800551
-0.46062	0.555179	0.423006	0.499683	0.371285	0.32362	-0.1076	0.584158	-0.53544
5.06E-08	1.26E-11	7.25E-07	2.22E-09	1.73E-05	0.000206	0.228558	5.63E-13	8.82E-11
0.023114	0.120711	0.046743	0.198309	0.090347	0.230391	-0.02887	0.160928	-0.40255
0.79645	0.176425	0.601776	0.025419	0.312421	0.009164	0.747339	0.070694	2.71E-06
0.224258	-0.2261	-0.1851	-0.18137	0.140286	0.254779	-0.2139	0.203473	-0.0714
0.011256	0.010586	0.03722	0.041285	0.115696	0.003844	0.015746	0.021767	0.42505

RBM42	RBM48	RBMXL1	REPIN1	REXO4	RFNG	RFXAP	RHBDD3	RHOBTB1
black	black	black	black	black	black	black	black	black
-0.14051	0.093252	0.13017	-0.38411	-0.16062	-0.22025	-0.09477	-0.19205	0.265598
0.115117	0.297053	0.144663	8.27E-06	0.071243	0.012841	0.289247	0.030537	0.002545
-0.15561	0.09535	0.155411	-0.35698	-0.14998	-0.19339	-0.03807	-0.23759	0.211675
0.080637	0.286268	0.081039	3.79E-05	0.092382	0.029373	0.670885	0.007152	0.016894
0.009903	0.170683	0.208267	0.304646	0.324898	0.127967	0.242737	0.049686	-0.0159
0.912011	0.055037	0.01879	0.000497	0.000194	0.151642	0.005965	0.579074	0.85916
-0.42015	0.506579	0.466095	-0.10666	-0.16007	-0.31542	0.443417	-0.31899	0.270441
8.76E-07	1.23E-09	3.34E-08	0.232676	0.072224	0.000304	1.78E-07	0.000257	0.002104
-0.12572	0.126541	0.170224	-0.25461	0.115767	0.017921	0.177629	0.066916	-0.00292
0.159028	0.156292	0.055704	0.003868	0.194943	0.841494	0.045727	0.454767	0.97398
-0.57886	0.54837	0.530694	-0.4445	-0.24563	-0.45293	0.501389	-0.35341	0.316702
1.02E-12	2.49E-11	1.38E-10	1.65E-07	0.005378	8.95E-08	1.92E-09	4.59E-05	0.000286
-0.49351	0.408997	0.269111	-0.37857	-0.22519	-0.31707	0.186738	-0.36811	0.236433
3.74E-09	1.81E-06	0.002218	1.14E-05	0.010914	0.000281	0.03554	2.06E-05	0.007447
-0.31952	0.020546	0.08175	-0.21024	-0.32024	-0.35852	0.226385	-0.17311	0.367429
0.000251	0.818653	0.360878	0.017673	0.000242	3.49E-05	0.010488	0.051622	2.14E-05
0.128029	-0.02287	-0.20357	0.138032	0.051136	0.110218	-0.35503	0.104904	0.099737
0.151443	0.798536	0.021704	0.121723	0.568034	0.217362	4.21E-05	0.240491	0.26457
0.085072	-0.17146	-0.17356	-0.13788	-0.09338	-0.06492	-0.31093	0.170521	0.346249
0.341626	0.053921	0.051013	0.122126	0.296406	0.468369	0.000374	0.055271	6.68E-05
-0.09608	0.128086	0.204619	-0.18643	-0.18794	-0.27589	-0.04012	-0.02747	0.291836
0.2826	0.151259	0.021021	0.035852	0.034352	0.001692	0.654292	0.759187	0.000871
0.405202	-0.0345	-0.13761	0.320702	0.278777	0.327606	-0.37752	0.119556	-0.26546
2.29E-06	0.700211	0.122868	0.000237	0.001505	0.00017	1.21E-05	0.180631	0.002558
0.603593	-0.35301	-0.37472	0.690854	0.455308	0.579922	-0.39913	0.41818	-0.15618
5.87E-14	4.69E-05	1.42E-05	2.52E-19	7.52E-08	9.03E-13	3.35E-06	9.97E-07	0.079528
0.908652	-0.58216	-0.5757	0.732929	0.606527	0.870898	-0.48614	0.868317	-0.39788
3.04E-49	7.04E-13	1.44E-12	1.18E-22	4.11E-14	2.23E-40	6.87E-09	7.07E-40	3.62E-06
0.750465	-0.59675	-0.58189	0.569604	0.365538	0.590551	-0.66606	0.660053	0.006564
3.11E-24	1.32E-13	7.25E-13	2.78E-12	2.38E-05	2.72E-13	1.29E-17	3.16E-17	0.941616
0.191474	-0.30265	-0.18923	-0.00155	-0.07721	0.006433	-0.11452	0.064478	0.058744
0.031048	0.000543	0.033117	0.986192	0.388253	0.942778	0.199825	0.471405	0.511804
0.307226	-0.25307	-0.09175	0.409942	0.266202	0.279482	0.028495	0.125544	-0.39145
0.000442	0.004097	0.30491	1.7E-06	0.002486	0.001462	0.750477	0.159608	5.35E-06
-0.57272	0.409052	0.417964	-0.43309	-0.39157	-0.50617	0.589705	-0.55526	-0.11975
1.99E-12	1.8E-06	1.01E-06	3.66E-07	5.31E-06	1.27E-09	3E-13	1.24E-11	0.179937
-0.19452	0.012165	-0.0008	-0.23833	-0.17763	-0.17825	0.145308	-0.29898	-0.33083
0.028424	0.892024	0.992893	0.00697	0.045732	0.04496	0.103099	0.000639	0.000145
0.021766	0.005913	0.05295	0.107786	0.357656	0.269676	-0.04026	0.018987	0.043204
0.808086	0.947395	0.554367	0.227743	3.66E-05	0.002169	0.653144	0.832204	0.629598

RHOT2	RIN1	RLIM	RNASEH2(	RNF126	RNPC3	ROBO3	RPL13	RPL18
black	black	black	black	black	black	black	black	black
-0.25765	-0.15239	0.148341	-0.15388	-0.22638	0.045783	-0.24371	-0.08853	-0.13045
0.003452	0.087193	0.096031	0.084105	0.010491	0.609274	0.005761	0.322295	0.143785
-0.27703	-0.12885	0.12143	-0.02826	-0.23435	0.00983	-0.20431	-0.05939	-0.0679
0.001616	0.148803	0.173844	0.752437	0.008003	0.912654	0.021217	0.50715	0.448137
0.041694	-0.13447	0.001413	0.176243	-0.06952	0.126578	0.230623	-0.0031	0.164702
0.641626	0.131739	0.987422	0.047472	0.437345	0.156171	0.009092	0.97242	0.064256
-0.39851	-0.31617	0.338951	-0.10096	-0.55068	0.38019	-0.03793	-0.34521	-0.26555
3.48E-06	0.000293	9.7E-05	0.258746	1.98E-11	1.04E-05	0.671983	7.04E-05	0.00255
-0.10444	-0.08301	-0.17317	0.472219	-0.18754	-0.19011	0.141279	0.477685	0.395731
0.242587	0.35347	0.051534	2.08E-08	0.03474	0.03229	0.113114	1.36E-08	4.12E-06
-0.5546	-0.35979	0.284131	0.047299	-0.72064	0.195961	-0.11352	-0.14656	-0.1875
1.33E-11	3.26E-05	0.001207	0.597461	1.28E-21	0.027245	0.203794	0.100136	0.034786
-0.42445	-0.21429	0.149547	0.002331	-0.56932	0.236372	-0.10495	-0.20757	-0.16972
6.58E-07	0.015556	0.093327	0.979248	2.86E-12	0.007462	0.240287	0.019199	0.056447
-0.24058	-0.23944	0.217808	-0.07214	-0.41793	0.170805	-0.08484	-0.1909	-0.34432
0.00644	0.006702	0.013897	0.420259	1.01E-06	0.05486	0.342922	0.031568	7.38E-05
0.077619	0.130785	-0.2151	-0.08476	-0.05693	-0.11887	-0.0632	0.013849	0.049862
0.385732	0.142757	0.015157	0.343382	0.524963	0.183149	0.480263	0.877186	0.577725
0.005845	-0.11096	-0.27373	-0.03267	-0.18731	-0.20174	-0.08073	0.159663	0.019231
0.948002	0.214272	0.001846	0.715417	0.034967	0.02294	0.366941	0.072966	0.830082
-0.20619	-0.19994	0.068353	-0.16392	-0.33756	-0.00956	-0.13747	-0.12621	-0.26235
0.020034	0.024215	0.445121	0.065548	0.000104	0.915058	0.12327	0.157384	0.002885
0.166726	0.213246	-0.24675	-0.0262	0.354354	-0.09306	0.041374	0.203291	0.184621
0.061004	0.016077	0.005165	0.770012	4.36E-05	0.298034	0.644192	0.021888	0.037719
0.57172	0.21443	-0.35559	0.094272	0.55285	-0.01875	0.353281	0.160907	0.316277
2.21E-12	0.015485	4.08E-05	0.291776	1.59E-11	0.834296	4.62E-05	0.070732	0.000292
0.881561	0.476644	-0.69561	0.560124	0.890613	-0.50627	0.569035	0.696367	0.73003
1.44E-42	1.47E-08	1.13E-19	7.55E-12	1.33E-44	1.26E-09	2.95E-12	9.95E-20	2.1E-22
0.667773	0.239436	-0.64407	0.186323	0.557589	-0.35617	0.308774	0.416125	0.397907
9.93E-18	0.006704	3.12E-16	0.035959	9.81E-12	3.96E-05	0.000412	1.14E-06	3.61E-06
0.101656	-0.14505	-0.19734	0.038939	0.205311	-0.09555	-0.11279	0.069802	0.013309
0.255443	0.103729	0.02616	0.663821	0.020581	0.285251	0.206761	0.435507	0.881938
0.282118	0.057567	-0.05639	0.096392	0.514564	-0.03835	0.151981	0.073426	0.230568
0.001312	0.520308	0.528915	0.281008	6.07E-10	0.668581	0.088059	0.411991	0.00911
-0.47335	-0.08789	0.669498	-0.31451	-0.27405	0.316875	-0.36782	-0.51328	-0.48576
1.91E-08	0.325812	7.63E-18	0.000317	0.001823	0.000284	2.1E-05	6.81E-10	7.08E-09
-0.13429	0.131143	0.32734	-0.21011	0.146131	0.068489	-0.23271	-0.19845	-0.1942
0.132253	0.141658	0.000172	0.017741	0.101142	0.444209	0.008467	0.025315	0.028687
0.149375	-0.03975	-0.07086	0.169084	0.141966	0.214527	0.130274	0.229828	0.388279
0.09371	0.657288	0.428554	0.057388	0.111354	0.015437	0.144338	0.009341	6.46E-06

RPL18A	RPL28	RPL36	RPL8	RPLP1	RPLP2	RPS15	RPS16	RPS19
black	black	black	black	black	black	black	black	black
-0.1746	-0.06979	-0.05313	-0.27543	-0.13913	-0.03165	-0.18308	-0.09913	-0.01464
0.049618	0.43556	0.553014	0.001724	0.118757	0.723895	0.039377	0.267488	0.870246
-0.16068	-0.09619	-0.00389	-0.32358	-0.14378	-0.01127	-0.18801	-0.12344	0.008079
0.071141	0.282008	0.965393	0.000207	0.106804	0.899912	0.034281	0.16676	0.928169
0.18506	0.000697	0.102727	0.204731	0.097882	0.093105	-0.03083	0.149073	0.213484
0.037258	0.993798	0.250443	0.020949	0.273603	0.297818	0.730776	0.094383	0.015957
-0.24443	-0.38875	-0.20139	-0.19856	-0.23485	-0.14217	-0.44248	-0.18574	-0.00487
0.005614	6.28E-06	0.02318	0.025232	0.007866	0.110826	1.9E-07	0.036553	0.956641
0.247118	0.139802	0.563554	0.201237	0.345878	0.575901	0.092528	0.31766	0.447839
0.005096	0.11697	5.28E-12	0.023289	6.81E-05	1.41E-12	0.300834	0.000274	1.3E-07
-0.24927	-0.38505	0.017666	-0.24008	-0.12074	0.124882	-0.46205	-0.11904	0.135359
0.004711	7.82E-06	0.843728	0.006553	0.176324	0.161839	4.54E-08	0.182521	0.129184
-0.19602	-0.26612	0.048099	-0.28331	-0.13757	0.008521	-0.37855	-0.15155	0.164831
0.027198	0.002494	0.591265	0.001249	0.122981	0.924254	1.14E-05	0.088984	0.064044
-0.33577	-0.30575	-0.21276	-0.26666	-0.13971	-0.0367	-0.27168	-0.16272	-0.14532
0.000114	0.000473	0.016324	0.002441	0.117221	0.68205	0.002003	0.067571	0.103077
0.181707	0.199126	0.088483	0.209939	0.212293	0.134212	0.118523	0.242287	0.272543
0.0409	0.024808	0.32255	0.017838	0.016568	0.132491	0.184454	0.006061	0.001936
0.073393	0.265362	0.129059	0.091174	0.33656	0.359763	0.13975	0.280593	0.365204
0.412204	0.002568	0.148152	0.307994	0.000109	3.26E-05	0.117107	0.001397	2.42E-05
-0.15258	-0.00625	-0.09574	-0.11676	0.028186	0.11019	-0.12087	0.000112	0.115882
0.086795	0.944364	0.284294	0.19111	0.753092	0.217481	0.175862	0.999005	0.194496
0.284444	0.259684	0.089367	0.197527	0.071647	-0.06268	0.278423	0.104016	0.004889
0.001191	0.003195	0.317715	0.026015	0.423441	0.483894	0.001527	0.244515	0.956494
0.39984	0.435255	0.086432	0.419712	0.257106	0.041405	0.44001	0.321525	0.172844
3.21E-06	3.16E-07	0.333936	9.02E-07	0.003523	0.643943	2.26E-07	0.000228	0.051986
0.750155	0.813883	0.612376	0.830207	0.714716	0.540444	0.902024	0.716881	0.450392
3.32E-24	2.92E-31	2.01E-14	1.62E-33	3.87E-21	5.44E-11	1.96E-47	2.59E-21	1.08E-07
0.481686	0.702844	0.269701	0.55043	0.586484	0.397842	0.676328	0.57311	0.415838
9.85E-09	3.25E-20	0.002167	2.03E-11	4.33E-13	3.63E-06	2.65E-18	1.9E-12	1.16E-06
-0.09329	0.125345	-0.11015	-0.10253	0.000299	-0.04044	0.139879	-0.05914	-0.10345
0.296841	0.160276	0.217647	0.251371	0.997338	0.651666	0.116766	0.508944	0.247123
0.187911	0.130708	0.010741	0.163029	-0.03494	-0.17647	0.225522	-0.00983	-0.16802
0.03438	0.142994	0.904599	0.067049	0.696536	0.047178	0.010794	0.912667	0.058993
-0.50919	-0.66735	-0.43662	-0.5372	-0.65591	-0.61744	-0.56257	-0.65688	-0.63978
9.77E-10	1.06E-17	2.87E-07	7.45E-11	5.8E-17	1.06E-14	5.85E-12	5.03E-17	5.64E-16
-0.23273	-0.29311	-0.22375	-0.31694	-0.40508	-0.44214	-0.18079	-0.42749	-0.51588
0.008463	0.000825	0.011448	0.000283	2.31E-06	1.95E-07	0.041941	5.37E-07	5.4E-10
0.318374	0.158923	0.221749	0.252663	0.14214	0.122845	0.105612	0.220127	0.23287
0.000264	0.074322	0.012226	0.004159	0.110914	0.168844	0.237313	0.012891	0.008421

RPS19BP1	RPS28	RPS5	RPS6KB2	RPS6KC1	RPS9	RPUSD1	RRNAD1	RRP1
black	black	black	black	black	black	black	black	black
0.028562	-0.0646	-0.21489	-0.17857	0.200503	-0.16095	-0.28052	-0.06184	-0.2061
0.749908	0.470575	0.015261	0.044569	0.023808	0.070652	0.001401	0.489765	0.020087
0.031596	-0.04028	-0.15641	-0.20887	0.296038	-0.17947	-0.24854	-0.02354	-0.21046
0.724358	0.652999	0.079081	0.018444	0.000727	0.04349	0.004839	0.792813	0.017551
-0.28353	7.92E-05	0.414462	0.08993	-0.22501	-0.03037	0.087501	-0.03649	0.103108
0.001237	0.999295	1.27E-06	0.314668	0.01098	0.734646	0.327969	0.683834	0.248683
-0.49229	-0.40673	0.130123	-0.18093	0.105594	-0.31701	-0.45021	-0.15829	-0.39292
4.14E-09	2.08E-06	0.144808	0.041791	0.237395	0.000282	1.09E-07	0.0755	4.89E-06
0.505562	0.294135	0.521365	0.332811	-0.09997	0.502451	0.018952	0.3272	-0.0052
1.34E-09	0.000789	3.29E-10	0.000132	0.263446	1.75E-09	0.832507	0.000173	0.953749
-0.13478	-0.30773	0.22678	-0.06186	0.187744	-0.08996	-0.53299	-0.02	-0.52346
0.130849	0.000432	0.01035	0.489621	0.034542	0.31453	1.11E-10	0.82343	2.71E-10
-0.24719	-0.1727	0.122679	-0.10413	0.113312	-0.09845	-0.46331	0.006404	-0.39739
0.005082	0.052179	0.169425	0.243988	0.204646	0.2708	4.13E-08	0.943035	3.73E-06
-0.08036	-0.28225	-0.19539	-0.0981	0.288756	-0.1089	-0.38894	0.216736	-0.39544
0.369111	0.001305	0.02771	0.272521	0.000993	0.222931	6.21E-06	0.014385	4.2E-06
-0.18265	0.102811	0.231783	0.199755	-0.24549	0.032651	-0.05356	-0.23074	0.041806
0.039842	0.250055	0.00874	0.024347	0.005405	0.71555	0.549815	0.009057	0.640728
0.204887	0.209413	0.036447	0.302553	-0.12905	0.220719	-0.17351	0.064996	-0.06947
0.02085	0.018133	0.684144	0.000546	0.148186	0.012645	0.051078	0.467841	0.437716
-0.01433	-0.12127	-0.14266	0.019584	0.045547	-0.07302	-0.37167	0.012924	-0.27981
0.872915	0.17441	0.109597	0.82701	0.61112	0.414566	1.69E-05	0.885329	0.001442
-0.02174	0.122933	0.043043	0.063013	-0.09072	0.038237	0.122982	-0.02222	0.344332
0.808324	0.168537	0.630878	0.481559	0.310431	0.66952	0.168369	0.804126	7.37E-05
-0.13323	0.328241	0.143973	0.237909	-0.40241	0.082405	0.451944	0.082612	0.564868
0.135364	0.000165	0.106338	0.007073	2.73E-06	0.35703	9.63E-08	0.355818	4.6E-12
0.665553	0.765969	0.467258	0.715651	-0.6885	0.742878	0.891876	0.469757	0.839845
1.39E-17	9.59E-26	3.06E-08	3.25E-21	3.72E-19	1.56E-23	6.72E-45	2.52E-08	5.75E-35
0.316255	0.577318	0.134611	0.54225	-0.50268	0.410829	0.471646	0.25175	0.581752
0.000292	1.2E-12	0.131333	4.57E-11	1.72E-09	1.61E-06	2.18E-08	0.004301	7.37E-13
0.256153	0.105901	-0.37542	-0.06044	0.186083	0.004608	0.125997	0.122666	0.153915
0.003651	0.236027	1.37E-05	0.499702	0.036202	0.958993	0.158095	0.169472	0.084044
0.011439	0.127983	-0.02652	-0.12037	-0.09104	0.014297	0.443433	0.022853	0.356527
0.89843	0.151589	0.767295	0.177657	0.308707	0.873244	1.78E-07	0.798702	3.89E-05
-0.31896	-0.59008	-0.37497	-0.62884	0.471585	-0.47037	-0.25637	-0.36764	-0.45419
0.000257	2.87E-13	1.4E-05	2.45E-15	2.19E-08	2.41E-08	0.003621	2.12E-05	8.16E-08
-0.02202	-0.21445	-0.36825	-0.44337	0.3385	-0.222	0.057626	-0.20449	-0.09619
0.805915	0.015476	2.05E-05	1.79E-07	9.92E-05	0.012126	0.519882	0.021102	0.282017
-0.15005	0.255815	0.309981	0.133086	-0.23171	0.097667	0.224469	-0.09388	0.343596
0.092213	0.003698	0.00039	0.135801	0.008761	0.274663	0.011178	0.29377	7.66E-05

RRP9	RUVBL2	SAC3D1	SACM1L	SAMD1	SART1	SCAF1	SCAF8	SCAMP4
black	black	black	black	black	black	black	black	black
-0.26307	-0.37333	-0.22604	-0.07879	-0.34374	-0.18767	-0.22723	-0.21203	-0.1486
0.002806	1.54E-05	0.01061	0.378583	7.6E-05	0.034613	0.010195	0.016705	0.095446
-0.22441	-0.30803	-0.17237	-0.05276	-0.35554	-0.2377	-0.24203	-0.27201	-0.20358
0.011199	0.000427	0.052638	0.555778	4.1E-05	0.007124	0.006118	0.001977	0.021698
0.477908	0.26938	0.261782	0.158539	0.303463	0.027778	-0.0736	0.359679	-0.07342
1.33E-08	0.002195	0.002949	0.075034	0.000524	0.756559	0.410864	3.28E-05	0.412053
0.158635	-0.21144	-0.20675	0.55568	-0.22113	-0.46309	-0.53737	0.486155	-0.39404
0.074856	0.01702	0.01969	1.19E-11	0.012475	4.2E-08	7.33E-11	6.86E-09	4.57E-06
0.469248	0.272407	0.358536	0.039958	-0.2339	-0.30687	-0.46164	-0.40919	-0.4571
2.62E-08	0.001946	3.49E-05	0.655574	0.00813	0.00045	4.69E-08	1.78E-06	6.59E-08
0.216408	-0.23894	-0.14312	0.56777	-0.55188	-0.73419	-0.86575	0.139846	-0.69705
0.014537	0.006822	0.108456	3.38E-12	1.75E-11	9.19E-23	2.17E-39	0.116854	8.86E-20
0.155738	-0.19074	-0.18019	0.336854	-0.50841	-0.57295	-0.67024	0.099104	-0.46942
0.080393	0.031714	0.042639	0.000108	1.05E-09	1.94E-12	6.81E-18	0.26763	2.59E-08
-0.13776	-0.18124	-0.28412	0.31065	-0.35715	-0.38333	-0.35236	-0.05403	-0.19054
0.122473	0.04143	0.001207	0.000379	3.76E-05	8.65E-06	4.85E-05	0.54634	0.031892
0.127624	-0.01767	-0.02159	-0.08949	-0.01618	0.127756	-0.01796	0.372282	0.201329
0.152751	0.843652	0.809632	0.317025	0.856763	0.152323	0.841141	1.63E-05	0.023225
-0.08146	-0.23735	-0.0685	-0.15229	-0.29212	0.012809	-0.21923	-0.10906	0.152079
0.362596	0.007212	0.444167	0.087398	0.000861	0.886349	0.013272	0.222278	0.087852
-0.1206	-0.39546	-0.25352	0.134031	-0.3983	-0.18664	-0.34383	0.074988	-0.015
0.176816	4.19E-06	0.004029	0.133018	3.52E-06	0.035642	7.56E-05	0.402088	0.867032
-0.02841	0.129685	0.023504	-0.35346	0.117067	0.41012	0.389397	0.306436	0.39771
0.75117	0.146176	0.793098	4.58E-05	0.189944	1.68E-06	6.04E-06	0.000458	3.65E-06
0.156311	0.350322	0.286072	-0.4417	0.707475	0.734368	0.708498	0.226056	0.690772
0.079272	5.4E-05	0.001113	2.01E-07	1.44E-20	8.87E-23	1.2E-20	0.010603	2.55E-19
0.481845	0.77726	0.766858	-0.60685	0.802671	0.850582	0.810386	-0.37777	0.751912
9.73E-09	6.41E-27	7.8E-26	3.95E-14	7.77E-30	1.07E-36	8.31E-31	1.19E-05	2.27E-24
0.078323	0.252188	0.337268	-0.62053	0.493847	0.7765	0.626022	-0.12212	0.826066
0.381428	0.004232	0.000106	7.19E-15	3.64E-09	7.73E-27	3.54E-15	0.171398	6.35E-33
-0.3524	-0.14166	-0.01439	-0.23607	0.03996	0.212085	0.256482	-0.48522	0.236958
4.84E-05	0.112126	0.872435	0.007542	0.655559	0.016677	0.003606	7.4E-09	0.007312
0.040614	0.367612	0.206387	-0.22089	0.538055	0.419455	0.568086	-0.08448	0.287276
0.650289	2.12E-05	0.019913	0.012576	6.86E-11	9.17E-07	3.27E-12	0.344992	0.001058
-0.28799	-0.25866	-0.36339	0.554506	-0.2835	-0.52905	-0.26558	0.087847	-0.55395
0.001026	0.003321	2.68E-05	1.34E-11	0.001239	1.61E-10	0.002546	0.326051	1.42E-11
-0.33279	-0.03231	-0.19745	0.033246	-0.05704	-0.10409	0.175546	-0.18404	-0.17627
0.000132	0.718366	0.026078	0.710588	0.524134	0.244192	0.048371	0.038338	0.047438
0.393458	0.2351	0.30047	-0.16624	0.326794	0.251423	0.093765	0.198373	-0.11613
4.74E-06	0.007799	0.000598	0.061769	0.000177	0.004354	0.294389	0.025371	0.193555

SCAND1	SCAP	SCARB2	SCRIB	SCRN2	SCRN3	SCYL1	SDAD1	SDF2L1
black	black	black	black	black	black	black	black	black
-0.18571	-0.18325	0.017369	-0.29198	-0.02719	0.004408	-0.10327	0.131159	-0.15968
0.036589	0.03919	0.846317	0.000866	0.761564	0.960775	0.247945	0.141609	0.072937
-0.19474	-0.21386	-0.00627	-0.34042	-0.06388	0.072577	-0.05846	0.09551	-0.23967
0.028234	0.015768	0.944235	9E-05	0.475523	0.417433	0.513866	0.285458	0.006648
-0.17855	-0.00709	0.307719	0.000128	-0.07689	0.145486	-0.14778	0.107693	0.124431
0.044599	0.936962	0.000433	0.998861	0.390194	0.102674	0.097304	0.228145	0.163371
-0.55507	-0.44936	0.430466	-0.42522	-0.3379	0.405864	-0.58351	0.513376	-0.09764
1.27E-11	1.16E-07	4.39E-07	6.25E-07	0.000102	2.2E-06	6.05E-13	6.75E-10	0.274806
0.116685	-0.38532	-0.40033	-0.48743	0.220777	0.082409	0.091506	-0.15959	0.060297
0.191403	7.7E-06	3.11E-06	6.18E-09	0.012621	0.357005	0.306225	0.073097	0.500687
-0.48408	-0.74709	0.113763	-0.78451	-0.26234	0.478231	-0.54291	0.421036	-0.13502
8.12E-09	6.41E-24	0.202837	1.03E-27	0.002886	1.3E-08	4.28E-11	8.26E-07	0.130142
-0.46841	-0.47625	0.129091	-0.63439	-0.08651	0.225866	-0.30402	0.375144	-0.24585
2.8E-08	1.52E-08	0.148049	1.17E-15	0.333509	0.010671	0.000511	1.39E-05	0.005335
-0.23698	-0.15272	-0.10041	-0.23088	-0.08609	0.240315	-0.40774	0.129384	-0.13984
0.007305	0.086509	0.261334	0.009012	0.335875	0.0065	1.96E-06	0.147123	0.11687
-0.02711	0.098654	0.269919	0.051459	-0.00425	-0.31696	-0.01691	0.274632	0.426474
0.762245	0.269819	0.002148	0.565589	0.962199	0.000283	0.850344	0.00178	5.75E-07
0.073968	-0.05277	0.060895	-0.08959	0.155169	-0.24568	-0.1269	0.062148	0.411367
0.408536	0.555727	0.496446	0.316505	0.081518	0.005367	0.155099	0.487605	1.55E-06
-0.18321	-0.17202	0.140547	-0.2084	-0.06997	-0.00546	-0.36439	0.204891	0.161851
0.039231	0.053138	0.115011	0.018714	0.434388	0.951376	2.54E-05	0.020847	0.069073
0.172606	0.357049	0.1443	0.365847	0.069106	-0.4543	0.323599	0.037252	0.105941
0.052316	3.78E-05	0.105536	2.34E-05	0.440107	8.1E-08	0.000206	0.677552	0.23585
0.268949	0.732648	0.258612	0.739246	0.257694	-0.45238	0.303273	-0.1715	0.375184
0.002232	1.25E-22	0.003328	3.3E-23	0.003446	9.32E-08	0.000528	0.053869	1.39E-05
0.903852	0.746049	-0.33309	0.808184	0.583635	-0.6061	0.703889	-0.67374	0.711788
6.41E-48	7.99E-24	0.00013	1.59E-30	5.97E-13	4.33E-14	2.71E-20	3.97E-18	6.6E-21
0.562017	0.72022	0.061854	0.729224	0.454078	-0.64829	0.360684	-0.34909	0.717309
6.2E-12	1.39E-21	0.489675	2.46E-22	8.23E-08	1.73E-16	3.11E-05	5.76E-05	2.39E-21
0.176149	0.207922	-0.00277	0.229875	0.147335	0.005895	0.094918	-0.3293	-0.12102
0.047592	0.018992	0.975384	0.009326	0.098332	0.947557	0.288465	0.000157	0.175322
0.22437	0.410079	-0.03604	0.484543	0.083251	-0.05987	0.331901	-0.40986	-0.16217
0.011214	1.68E-06	0.687478	7.82E-09	0.352097	0.503733	0.000138	1.71E-06	0.068515
-0.36561	-0.49111	-0.06953	-0.38879	-0.47386	0.599319	-0.22166	0.202209	-0.72619
2.37E-05	4.57E-09	0.43727	6.27E-06	1.83E-08	9.77E-14	0.012262	0.022616	4.44E-22
-0.00053	-0.04333	-0.32404	-0.01884	-0.15762	0.167256	0.22556	-0.13799	-0.58683
0.995248	0.628562	0.000202	0.833454	0.076752	0.060176	0.01078	0.121829	4.16E-13
-0.04791	0.10746	0.225791	0.027699	0.202566	-0.09745	0.222052	-0.02937	-0.01675
0.592709	0.229161	0.010697	0.757223	0.022373	0.275736	0.012105	0.743043	0.851719

SDHAF1	SENP2	SENP5	SERAC1	SETX	SGTA	SH3GL1	SH3YL1	SHARPIN
black	black	black	black	black	black	black	black	black
-0.15316	0.152039	0.116056	-0.00998	0.04739	-0.19855	-0.29947	0.058948	-0.10954
0.085585	0.087936	0.193826	0.911316	0.596752	0.025235	0.000626	0.510338	0.220235
-0.05323	0.200771	0.199422	0.058414	-0.07071	-0.16532	-0.31502	0.075476	-0.13751
0.552302	0.023617	0.024591	0.514184	0.429513	0.063254	0.000309	0.399027	0.12315
-0.0759	0.032173	-0.10819	0.107706	0.190851	0.024549	0.238485	0.122022	-0.15373
0.396346	0.719534	0.226	0.22809	0.03161	0.78412	0.006932	0.171741	0.084415
-0.43411	0.25058	0.115496	0.426459	0.498494	-0.50161	-0.26897	0.4253	-0.56327
3.41E-07	0.004491	0.195997	5.75E-07	2.46E-09	1.89E-09	0.00223	6.22E-07	5.44E-12
0.4435	-0.26997	0.124702	0.071283	-0.54353	-0.20916	-0.21676	-0.08289	0.080452
1.77E-07	0.002144	0.162448	0.425804	4.03E-11	0.018279	0.014372	0.354166	0.368579
-0.22505	0.125322	0.242186	0.470244	0.146468	-0.7314	-0.54396	0.340986	-0.53282
0.010963	0.160354	0.006083	2.43E-08	0.10035	1.6E-22	3.86E-11	8.75E-05	1.13E-10
-0.24659	0.199692	0.268862	0.250109	0.186224	-0.39845	-0.42288	0.197777	-0.4219
0.005194	0.024393	0.00224	0.004569	0.036059	3.49E-06	7.31E-07	0.025823	7.8E-07
-0.22688	0.112673	0.080281	0.065078	0.125289	-0.42406	-0.43315	0.128698	-0.19371
0.010315	0.207227	0.369601	0.467277	0.160465	6.76E-07	3.65E-07	0.149298	0.029103
-0.30636	-0.08075	-0.25328	-0.12592	0.467184	-0.03839	0.294941	-0.11719	-0.02326
0.00046	0.366776	0.004064	0.158355	3.08E-08	0.668285	0.000762	0.189465	0.795173
-0.1762	-0.20586	-0.18573	-0.19217	0.328056	-0.17474	-0.08708	-0.10818	0.158956
0.047526	0.02024	0.03656	0.030426	0.000166	0.049431	0.330316	0.226039	0.074261
-0.36796	-0.02283	-0.12185	0.042795	0.443911	-0.34092	-0.2421	0.140429	-0.09671
2.08E-05	0.798885	0.172352	0.632847	1.72E-07	8.78E-05	0.006101	0.11532	0.279409
0.048529	-0.06829	-0.13136	-0.0668	0.151266	0.441047	0.390685	-0.0836	0.181721
0.587953	0.445563	0.140991	0.455573	0.089582	2.11E-07	5.6E-06	0.35008	0.040884
0.06274	-0.09887	-0.36159	-0.39729	0.228986	0.660013	0.681896	-0.14833	0.420234
0.483463	0.268792	2.96E-05	3.75E-06	0.009611	3.18E-17	1.09E-18	0.096055	8.71E-07
0.726978	-0.62926	-0.52861	-0.56094	-0.46964	0.838002	0.825682	-0.44486	0.921296
3.81E-22	2.32E-15	1.68E-10	6.93E-12	2.55E-08	1.11E-34	7.2E-33	1.61E-07	4.09E-53
0.169555	-0.40507	-0.51209	-0.60361	0.156462	0.601818	0.607491	-0.3277	0.711604
0.056687	2.31E-06	7.57E-10	5.86E-14	0.07898	7.26E-14	3.66E-14	0.000169	6.83E-21
0.209085	0.011564	0.075214	-0.175	-0.22195	0.308668	-0.14464	0.017083	0.225253
0.018319	0.897326	0.400667	0.049083	0.012145	0.000414	0.10472	0.848818	0.010891
0.306428	0.028398	-0.06291	-0.15414	-0.32124	0.612092	0.350862	-0.1306	0.215223
0.000459	0.7513	0.482254	0.083595	0.000231	2.08E-14	5.25E-05	0.143312	0.015098
-0.15324	0.379324	0.428436	0.512118	-0.16515	-0.37031	-0.42652	0.279189	-0.5837
0.085423	1.09E-05	5.04E-07	7.55E-10	0.063521	1.82E-05	5.73E-07	0.00148	5.92E-13
0.12797	0.202517	0.286835	0.117877	-0.43307	0.079877	-0.1378	-0.07936	-0.13037
0.151631	0.022407	0.001077	0.186875	3.67E-07	0.372018	0.122367	0.375146	0.144043
0.099743	0.070925	0.255632	-0.16745	-0.03422	0.211405	0.198498	-0.01534	-0.01291
0.264542	0.428137	0.003723	0.059877	0.702522	0.017038	0.025276	0.864096	0.88542

SHC2	SHISA3	SIGMAR1	SIRT6	SIRT7	SIVA1	SLC19A1	SLC25A1	SLC25A10
black	black	black	black	black	black	black	black	black
-0.25088	-0.0112	-0.25807	-0.21625	-0.11569	-0.12188	-0.39627	-0.26717	-0.24047
0.004441	0.900551	0.003397	0.014611	0.195226	0.172244	3.99E-06	0.002394	0.006464
-0.24166	0.00948	-0.19571	-0.21364	-0.14033	-0.13466	-0.43621	-0.3432	-0.16568
0.006197	0.915758	0.027444	0.015879	0.115574	0.131195	2.95E-07	7.81E-05	0.06266
-0.01462	-0.01802	0.251551	0.157492	0.135784	-0.06561	0.252908	0.101818	0.153912
0.870398	0.840676	0.004333	0.077003	0.127975	0.463651	0.004121	0.254681	0.084048
-0.43744	-0.29711	-0.15522	-0.31332	-0.10707	-0.34273	-0.24101	-0.20352	-0.33164
2.71E-07	0.000694	0.081422	0.000335	0.230854	8E-05	0.006343	0.021733	0.00014
-0.27075	-0.18082	0.2121	0.079892	0.188488	0.456463	-0.30464	-0.11825	-0.14863
0.002079	0.041918	0.016669	0.37193	0.03382	6.9E-08	0.000497	0.185482	0.09538
-0.6956	-0.42854	-0.16822	-0.39878	-0.10059	-0.09113	-0.57394	-0.35688	-0.55322
1.13E-19	5E-07	0.058693	3.42E-06	0.260467	0.308239	1.74E-12	3.81E-05	1.53E-11
-0.41991	-0.27999	-0.21034	-0.40285	-0.16249	-0.23862	-0.45748	-0.38772	-0.41796
8.9E-07	0.001432	0.017618	2.66E-06	0.067962	0.006899	6.4E-08	6.68E-06	1.01E-06
-0.34883	-0.21877	-0.13866	-0.36435	-0.07077	-0.07899	-0.46844	-0.1997	-0.40796
5.84E-05	0.013474	0.120012	2.54E-05	0.429146	0.37736	2.79E-08	0.02439	1.93E-06
-0.0627	-0.11283	-0.02781	0.05952	0.137482	0.000989	0.099363	0.253925	-0.05078
0.483735	0.206585	0.756288	0.506233	0.123231	0.991198	0.266376	0.003968	0.570765
-0.22386	-0.1067	-0.18833	-0.0294	0.23831	0.21792	-0.22314	0.202166	-0.30867
0.011406	0.232492	0.033972	0.742836	0.006975	0.013847	0.01168	0.022646	0.000414
-0.38794	-0.20299	-0.28387	-0.2194	-0.00148	-0.02356	-0.32074	0.034702	-0.36565
6.59E-06	0.022091	0.00122	0.013201	0.98679	0.792575	0.000236	0.69852	2.37E-05
0.299356	0.105959	0.024136	0.157862	-0.05797	-0.10253	0.182351	0.291192	0.623818
0.000629	0.235768	0.787659	0.076303	0.51742	0.251382	0.040179	0.000896	4.71E-15
0.630937	0.338131	0.241104	0.481848	0.326082	0.010078	0.675285	0.505746	0.581428
1.86E-15	0.000101	0.006321	9.72E-09	0.000183	0.910466	3.12E-18	1.32E-09	7.64E-13
0.760281	0.425761	0.52658	0.907315	0.652607	0.763099	0.734921	0.712047	0.649566
3.54E-25	6.03E-07	2.03E-10	7.22E-49	9.34E-17	1.86E-25	7.93E-23	6.3E-21	1.44E-16
0.540917	0.321179	0.171912	0.551879	0.537372	0.386951	0.49376	0.684635	0.389102
5.2E-11	0.000232	0.053285	1.75E-11	7.33E-11	6.99E-06	3.66E-09	7.01E-19	6.15E-06
0.229832	0.340861	-0.01724	0.045792	-0.00404	-0.00356	-0.04068	-0.00726	0.172052
0.00934	8.8E-05	0.847467	0.6092	0.964007	0.968311	0.649747	0.935415	0.053088
0.547371	0.404968	0.353242	0.303813	-0.08371	-0.03072	0.490838	0.100202	0.510215
2.76E-11	2.33E-06	4.63E-05	0.000516	0.349462	0.731695	4.67E-09	0.26234	8.93E-10
-0.29534	-0.15475	-0.20433	-0.42812	-0.58427	-0.38147	-0.25048	-0.59891	-0.23513
0.000749	0.082361	0.021204	5.14E-07	5.55E-13	9.64E-06	0.004508	1.03E-13	0.007792
0.091294	0.086098	-0.01595	-0.17084	-0.43727	-0.17334	-0.01831	-0.3603	0.054112
0.307353	0.335814	0.858696	0.054809	2.74E-07	0.051306	0.838143	3.17E-05	0.545696
0.248393	0.104474	0.245148	0.211963	0.128087	-0.06179	0.328557	0.027176	0.357928
0.004865	0.242433	0.005471	0.016742	0.151254	0.49014	0.000162	0.761672	3.6E-05

SLC25A11	SLC25A29	SLC25A30	SLC25A32	SLC25A39	SLC2A4RG	SLC2A8	SLC35A1	SLC35B4
black	black	black	black	black	black	black	black	black
-0.00545	0.02627	0.212645	0.114694	-0.12307	-0.16573	0.177306	0.030339	-0.04148
0.951528	0.769393	0.016386	0.199141	0.16806	0.062583	0.046129	0.734914	0.643324
0.13557	0.032857	0.137247	0.060654	-0.11371	-0.15743	0.179557	0.083763	0.010491
0.128582	0.713829	0.123878	0.498148	0.203038	0.077123	0.043389	0.349131	0.906812
-0.24813	-0.02001	-0.07126	0.048853	0.101943	0.086942	-0.30852	0.101072	0.219281
0.004911	0.823284	0.425982	0.585458	0.254098	0.331079	0.000417	0.258197	0.013251
-0.63736	-0.29414	0.417108	0.343219	-0.31452	-0.07557	-0.54653	0.454959	0.551168
7.84E-16	0.000789	1.07E-06	7.8E-05	0.000317	0.398424	3E-11	7.72E-08	1.88E-11
0.45352	-0.0979	0.035327	-0.0028	0.24775	0.129928	0.301849	0.387632	-0.05039
8.58E-08	0.273516	0.69336	0.975087	0.00498	0.145416	0.000563	6.72E-06	0.573668
-0.32885	-0.41804	0.540059	0.401236	-0.25876	-0.0342	-0.31032	0.671338	0.487145
0.00016	1.01E-06	5.65E-11	2.94E-06	0.00331	0.702668	0.000384	5.75E-18	6.33E-09
-0.20257	-0.33086	0.417608	0.158129	-0.15127	-0.09344	-0.17712	0.543446	0.398121
0.022373	0.000145	1.04E-06	0.075799	0.089567	0.296092	0.046365	4.06E-11	3.56E-06
-0.28707	-0.09287	0.235222	-0.03097	-0.14478	-0.05184	-0.27119	0.151426	0.335078
0.001067	0.299062	0.007766	0.729622	0.10436	0.562743	0.002043	0.08924	0.000118
-0.48708	-0.09315	0.180468	0.166835	0.165071	0.294739	-0.2595	0.002415	0.087869
6.36E-09	0.297567	0.04232	0.060832	0.063653	0.000769	0.003218	0.978505	0.325931
-0.26479	0.114281	0.345666	0.000879	0.273996	0.256913	0.10282	-0.08526	-0.10041
0.002627	0.200777	6.88E-05	0.992177	0.001826	0.003548	0.250015	0.340548	0.261336
-0.4624	0.006174	0.489456	0.061987	-0.08057	0.166614	-0.13035	0.050113	0.18115
4.43E-08	0.945075	5.24E-09	0.488739	0.367844	0.06118	0.144093	0.575816	0.041533
0.010483	0.194348	-0.19001	-0.23986	0.073792	0.007594	0.046177	-0.09842	-0.13479
0.906885	0.028564	0.032385	0.006605	0.409657	0.932476	0.606188	0.270971	0.130822
-0.10446	0.496145	-0.4311	-0.39841	0.359317	0.207862	0.039435	-0.5455	-0.27168
0.242498	3E-09	4.2E-07	3.5E-06	3.34E-05	0.019027	0.659799	3.32E-11	0.002003
0.51352	0.621491	-0.63326	-0.52223	0.769696	0.57093	0.565835	-0.60287	-0.6299
6.66E-10	6.36E-15	1.36E-15	3.03E-10	3.99E-26	2.41E-12	4.15E-12	6.4E-14	2.13E-15
-0.02259	0.59758	-0.27131	-0.46082	0.619764	0.484079	0.353642	-0.6611	-0.50492
0.800942	1.2E-13	0.002033	4.99E-08	7.93E-15	8.12E-09	4.53E-05	2.71E-17	1.42E-09
0.391398	0.271893	-0.21091	-0.29225	0.070179	-0.2313	0.490015	-0.32947	-0.33177
5.36E-06	0.001986	0.017303	0.000856	0.433023	0.008886	5E-09	0.000155	0.000139
0.441258	0.172477	-0.55739	-0.28172	0.088185	-0.23949	0.194713	-0.33018	-0.24475
2.07E-07	0.052494	1E-11	0.001333	0.324184	0.006691	0.02826	0.00015	0.005551
0.069601	-0.52985	0.135671	0.461596	-0.65228	-0.51302	-0.29137	0.387896	0.347002
0.436832	1.5E-10	0.128294	4.7E-08	9.79E-17	6.97E-10	0.000889	6.61E-06	6.42E-05
0.474565	-0.22244	-0.23495	0.065896	-0.31971	-0.42912	0.057626	0.017364	-0.05138
1.73E-08	0.011953	0.00784	0.46169	0.000248	4.81E-07	0.519884	0.846365	0.566191
0.110564	0.091853	-0.24129	0.042453	0.135901	-0.24439	0.149207	0.047325	-0.232
0.215911	0.304387	0.00628	0.635567	0.127644	0.005622	0.094083	0.597257	0.008677

SLC39A13	SLC39A3	SLC52A2	SLC6A8	SLMAP	SMARCB1	SMIM1	SNAPC2	SNRPB
black	black	black	black	black	black	black	black	black
-0.12955	-0.26363	-0.35067	-0.16405	0.028578	-0.25754	0.176942	-0.18315	-0.2135
0.146587	0.002746	5.3E-05	0.065325	0.749776	0.003465	0.046586	0.039293	0.015946
-0.1281	-0.27007	-0.39771	-0.07458	-0.02376	-0.23774	0.190407	-0.19215	-0.20016
0.151197	0.002135	3.66E-06	0.404683	0.790931	0.007116	0.032015	0.030445	0.024052
-0.0685	0.02868	0.254735	-0.06502	0.059272	0.406492	-0.42199	0.062796	0.471574
0.444153	0.748911	0.00385	0.467682	0.508011	2.12E-06	7.76E-07	0.483071	2.19E-08
-0.32543	-0.41059	-0.14718	-0.32829	0.489904	-0.15996	-0.52114	-0.33964	0.135486
0.000189	1.63E-06	0.098698	0.000165	5.05E-09	0.072422	3.35E-10	9.37E-05	0.128822
0.08146	-0.26584	-0.18712	-0.2449	-0.20711	-0.005	0.338714	0.045979	0.341441
0.362586	0.002521	0.035159	0.00552	0.019476	0.955512	9.81E-05	0.607734	8.55E-05
-0.29833	-0.64426	-0.40563	-0.54022	0.423168	-0.39144	-0.19834	-0.40762	0.13351
0.000658	3.04E-16	2.23E-06	5.56E-11	7.17E-07	5.35E-06	0.025399	1.97E-06	0.134547
-0.12393	-0.53573	-0.31797	-0.30008	0.364878	-0.32338	-0.12206	-0.24643	-0.01053
0.16508	8.57E-11	0.00027	0.000609	2.47E-05	0.000209	0.1716	0.005224	0.90646
-0.24722	-0.37155	-0.33477	-0.09572	0.33736	-0.47769	-0.02891	-0.43441	-0.35834
0.005077	1.7E-05	0.00012	0.284402	0.000105	1.36E-08	0.746936	3.35E-07	3.52E-05
0.380644	0.098451	0.478721	-0.13831	0.34559	-0.03052	-0.17386	0.183166	0.336895
1.01E-05	0.270813	1.25E-08	0.120959	6.91E-05	0.733383	0.050604	0.03928	0.000108
0.323732	-0.16189	0.162631	-0.23475	0.107167	-0.4152	0.29011	-0.03156	-0.01444
0.000205	0.069006	0.067726	0.007893	0.230439	1.21E-06	0.000938	0.724642	0.871997
0.006037	-0.22843	-0.04294	-0.3439	0.255505	-0.44794	0.063579	-0.2597	-0.10832
0.946293	0.009791	0.631683	7.54E-05	0.003741	1.29E-07	0.477621	0.003194	0.225444
0.217056	0.568211	0.368043	0.303213	-0.20612	0.412861	0.027678	0.258146	0.303222
0.014238	3.22E-12	2.07E-05	0.00053	0.020075	1.41E-06	0.757402	0.003387	0.00053
0.352987	0.569342	0.673779	0.489468	-0.28794	0.572131	-0.11206	0.44615	0.208955
4.69E-05	2.86E-12	3.94E-18	5.23E-09	0.001029	2.12E-12	0.20971	1.46E-07	0.018393
0.747596	0.780919	0.834733	0.545377	-0.6341	0.592425	0.482552	0.812188	0.446214
5.75E-24	2.58E-27	3.46E-34	3.36E-11	1.22E-15	2.19E-13	9.19E-09	4.86E-31	1.46E-07
0.69201	0.529671	0.769354	0.414757	-0.32669	0.216437	0.357882	0.506982	0.127384
2.08E-19	1.52E-10	4.33E-26	1.25E-06	0.000178	0.014523	3.61E-05	1.19E-09	0.153531
-0.0399	0.095267	-0.17524	0.350514	-0.33593	-0.034	0.392641	-0.07739	-0.4445
0.656022	0.286689	0.048767	5.35E-05	0.000113	0.704337	4.98E-06	0.387122	1.65E-07
-0.12542	0.445171	0.134109	0.467638	-0.43025	0.636095	-0.07642	0.220579	0.022191
0.160024	1.57E-07	0.132792	2.97E-08	4.45E-07	9.31E-16	0.393147	0.012703	0.804413
-0.65123	-0.26931	-0.65426	-0.26692	0.279452	-0.17238	-0.32263	-0.42105	-0.26805
1.14E-16	0.002201	7.36E-17	0.002417	0.001464	0.052627	0.000216	8.26E-07	0.002313
-0.34979	0.059763	-0.42959	0.066831	-0.13213	0.054224	0.000749	-0.12593	-0.30241
5.55E-05	0.504496	4.66E-07	0.455347	0.138644	0.544863	0.993336	0.158317	0.000549
-0.02679	0.033853	0.140048	-0.0065	-0.25603	0.564185	-0.23927	0.240343	0.397141
0.764979	0.705549	0.116322	0.942142	0.003669	4.94E-12	0.006744	0.006493	3.78E-06

SOCS5	SOD3	SOX15	SOX17	SOX18	SPATA2L	SPATC1L	SPEG	SPHK2
black	black	black	black	black	black	black	black	black
-0.0025	0.0335	-0.23595	-0.2253	-0.24287	-0.24764	-0.13862	-0.07631	-0.17236
0.97778	0.708482	0.007573	0.010873	0.005936	0.005	0.120131	0.393849	0.052662
-0.01147	0.009275	-0.16134	-0.26359	-0.28891	-0.24683	-0.17248	-0.10308	-0.16649
0.898191	0.91757	0.069965	0.002751	0.000987	0.00515	0.052486	0.24882	0.061376
0.112312	-0.2988	0.068133	-0.14123	-0.13132	0.071369	0.004547	-0.16229	-0.16449
0.208696	0.000644	0.446587	0.113249	0.141116	0.425247	0.959539	0.068305	0.064604
0.414813	-0.48828	-0.20624	-0.33988	-0.44734	-0.37628	-0.33417	-0.29085	-0.59071
1.24E-06	5.77E-09	0.020006	9.25E-05	1.34E-07	1.3E-05	0.000123	0.000909	2.67E-13
0.000168	-0.21858	-0.06629	-0.2372	-0.26333	-0.01585	-0.10989	-0.38216	-0.36794
0.998507	0.013554	0.458999	0.007249	0.002779	0.859586	0.218754	9.26E-06	2.08E-05
0.455256	-0.53135	-0.34582	-0.46121	-0.61141	-0.46601	-0.49184	-0.51042	-0.83681
7.55E-08	1.3E-10	6.83E-05	4.84E-08	2.26E-14	3.37E-08	4.3E-09	8.77E-10	1.68E-34
0.280756	-0.40594	-0.31318	-0.3147	-0.48215	-0.45442	-0.25005	-0.36348	-0.59127
0.001388	2.19E-06	0.000337	0.000314	9.49E-09	8.03E-08	0.004578	2.67E-05	2.5E-13
-0.04559	-0.13617	-0.24759	-0.3012	-0.24853	-0.38066	-0.50887	-0.01193	-0.36366
0.610772	0.126889	0.005009	0.000579	0.00484	1.01E-05	1E-09	0.894087	2.64E-05
0.042692	0.258949	-0.30879	0.177039	0.189012	-0.05938	0.120622	0.047358	-0.06917
0.633664	0.003286	0.000412	0.046464	0.033319	0.507243	0.176747	0.597002	0.43968
-0.15514	0.271611	-0.46578	0.073643	0.130504	-0.25064	-0.1908	0.088626	-0.15601
0.081568	0.002009	3.43E-08	0.410604	0.143626	0.004482	0.03166	0.321763	0.079852
-0.04365	0.016051	-0.37855	-0.12908	-0.0787	-0.34818	-0.28827	0.029442	-0.30417
0.626032	0.857855	1.14E-05	0.148083	0.379137	6.04E-05	0.001014	0.742469	0.000508
-0.41905	0.210808	0.18788	0.236113	0.349533	0.069637	0.518103	0.198292	0.482901
9.42E-07	0.01736	0.034409	0.00753	5.63E-05	0.436593	4.42E-10	0.025432	8.93E-09
-0.42237	0.342198	0.272263	0.320409	0.496698	0.371714	0.44986	0.44831	0.632019
7.56E-07	8.22E-05	0.001957	0.00024	2.86E-09	1.69E-05	1.12E-07	1.25E-07	1.61E-15
-0.60238	0.628174	0.480987	0.443993	0.721818	0.772106	0.577159	0.482345	0.827021
6.78E-14	2.67E-15	1.04E-08	1.71E-07	1.03E-21	2.25E-26	1.22E-12	9.34E-09	4.65E-33
-0.58844	0.686368	0.020646	0.465916	0.708223	0.324706	0.351197	0.536843	0.640599
3.46E-13	5.29E-19	0.817789	3.39E-08	1.26E-20	0.000196	5.16E-05	7.71E-11	5.04E-16
-0.26719	0.177904	0.005484	0.210762	0.259114	-0.01796	0.072899	0.098972	0.342436
0.002392	0.045387	0.951211	0.017385	0.003265	0.841166	0.415365	0.268274	8.12E-05
-0.23733	0.009489	0.470028	0.221221	0.285314	0.398459	0.341909	0.127654	0.526326
0.007217	0.91568	2.47E-08	0.012439	0.001148	3.49E-06	8.35E-05	0.152652	2.08E-10
0.588903	-0.4839	0.131648	-0.24933	-0.45638	-0.12329	-0.19779	-0.34725	-0.3064
3.28E-13	8.24E-09	0.140115	0.004701	6.95E-08	0.16729	0.02581	6.34E-05	0.000459
0.120246	-0.1175	0.27631	0.005463	-0.09252	0.116869	0.093082	-0.05707	0.160923
0.17811	0.188285	0.001664	0.951397	0.300868	0.190699	0.297933	0.523907	0.070703
0.081991	-0.19413	-0.00647	0.007256	-0.00848	0.193054	0.365006	-0.2058	0.039814
0.35946	0.02875	0.94244	0.935472	0.924597	0.02966	2.45E-05	0.020273	0.656738

SPSB2	SPTSSB	SRM	SSBP4	SSNA1	SSSCA1	ST3GAL2	ST6GALNA	STARD13
black	black	black	black	black	black	black	black	black
-0.1227	0.407465	-0.35036	-0.17729	0.100454	0.088881	-0.38913	-0.15225	0.291806
0.169358	1.99E-06	5.39E-05	0.046143	0.261135	0.320365	6.14E-06	0.087487	0.000872
-0.11189	0.360242	-0.27251	-0.22901	0.161792	0.138988	-0.44043	-0.15255	0.280534
0.210409	3.18E-05	0.001938	0.009604	0.069176	0.119137	2.2E-07	0.08686	0.0014
0.014198	-0.308	0.36241	-0.11948	-0.24645	-0.24009	0.312073	-0.1338	-0.28655
0.874119	0.000427	2.83E-05	0.180902	0.00522	0.006552	0.000355	0.133707	0.001091
-0.10974	0.045081	-0.21664	-0.5208	-0.56047	-0.52661	-0.15747	-0.36502	0.0875
0.219361	0.614772	0.014428	3.46E-10	7.28E-12	2.03E-10	0.077047	2.45E-05	0.327971
0.515932	0.104815	0.03833	-0.14627	0.594491	0.488291	-0.30702	0.095508	0.024446
5.37E-10	0.240892	0.668763	0.100803	1.72E-13	5.76E-09	0.000447	0.285464	0.784996
0.173889	0.239013	-0.38847	-0.64765	-0.16044	-0.17774	-0.48564	-0.28736	0.258065
0.050562	0.006805	6.39E-06	1.89E-16	0.071558	0.045595	7.16E-09	0.001054	0.003398
0.021066	0.256084	-0.29802	-0.51786	-0.06313	-0.14726	-0.44905	-0.18602	0.392159
0.814143	0.003661	0.000667	4.51E-10	0.480755	0.0985	1.19E-07	0.036268	5.12E-06
0.253144	0.28394	-0.47129	-0.09666	-0.11386	-0.18451	-0.44639	-0.13273	0.030678
0.004085	0.001216	2.24E-08	0.279663	0.202452	0.037838	1.44E-07	0.136869	0.732064
-0.09502	-0.16668	0.180439	0.013273	-0.28463	-0.24482	0.332942	0.151478	0.225106
0.287948	0.061079	0.042353	0.882259	0.001181	0.005537	0.000131	0.089128	0.010944
0.349852	0.105881	-0.25598	-0.09511	0.1555	-0.06497	-0.23484	0.252358	0.275575
5.53E-05	0.236113	0.003675	0.287477	0.080863	0.468007	0.007868	0.004206	0.001714
0.162692	0.236851	-0.34443	-0.22807	-0.15231	-0.22571	-0.30143	-0.02431	0.219101
0.067623	0.007339	7.33E-05	0.009912	0.087365	0.010728	0.000573	0.786161	0.013328
-0.23732	-0.20783	0.379978	0.238235	-0.10364	-0.09341	0.265254	-0.05467	0.035401
0.00722	0.019046	1.05E-05	0.006993	0.246218	0.296252	0.002579	0.541556	0.692749
-0.09355	-0.46385	0.554469	0.479431	-0.10059	-0.14898	0.601564	0.222797	-0.38234
0.2955	3.97E-08	1.35E-11	1.18E-08	0.260499	0.094586	7.48E-14	0.011812	9.17E-06
0.477694	-0.46989	0.736546	0.84523	0.661722	0.567698	0.623929	0.719561	-0.49104
1.35E-08	2.5E-08	5.71E-23	8.09E-36	2.47E-17	3.41E-12	4.65E-15	1.57E-21	4.59E-09
0.294781	-0.3716	0.37131	0.549244	0.294343	0.082166	0.398953	0.559232	-0.17714
0.000768	1.7E-05	1.73E-05	2.29E-11	0.000782	0.35843	3.39E-06	8.28E-12	0.046339
-0.00054	0.024584	-0.12882	0.077816	0.390059	0.148599	-0.27137	0.005758	0.085461
0.995189	0.783813	0.148914	0.384524	5.81E-06	0.095447	0.002028	0.948775	0.339416
-0.20447	-0.21336	0.43971	0.354293	0.123621	0.119381	0.379948	-0.05068	-0.42827
0.021116	0.016017	2.31E-07	4.38E-05	0.166151	0.181275	1.05E-05	0.57148	5.09E-07
-0.43822	0.316281	-0.32289	-0.31779	-0.33435	-0.06315	-0.17415	-0.46521	0.116308
2.57E-07	0.000292	0.000213	0.000272	0.000122	0.480633	0.05021	3.58E-08	0.19285
-0.36335	0.208706	-0.06106	0.103271	0.029815	0.226955	-0.01663	-0.18909	-0.02219
2.68E-05	0.018536	0.495265	0.247933	0.739327	0.01029	0.85278	0.033249	0.804453
-0.16296	-0.2573	0.388918	-0.00804	0.026965	0.065177	0.241883	-0.14606	-0.19742
0.06717	0.003498	6.22E-06	0.928553	0.763469	0.4666	0.006149	0.101311	0.026101

STARD3	STARD4	STBD1	STK11	STK19	STK4	STX10	STX12	STX17
black	black	black	black	black	black	black	black	black
-0.09965	0.002309	-0.22065	-0.29871	-0.16666	0.137484	-0.28874	0.130029	0.07622
0.26498	0.979449	0.012672	0.000647	0.061109	0.123224	0.000994	0.1451	0.394381
-0.16221	0.04742	-0.29556	-0.38215	-0.20545	-0.00488	-0.30191	0.198671	0.041002
0.068459	0.596518	0.000742	9.27E-06	0.020493	0.956582	0.000561	0.025146	0.64717
-0.16855	-0.05744	0.225895	0.078143	0.184448	0.025538	0.395351	-0.05073	0.16309
0.058189	0.521198	0.010661	0.382525	0.037901	0.775647	4.22E-06	0.571128	0.066944
-0.49861	0.223861	0.136791	-0.33566	-0.03197	0.392188	-0.0142	0.255739	0.393259
2.43E-09	0.011405	0.125144	0.000114	0.721223	5.11E-06	0.874108	0.003708	4.79E-06
-0.11873	0.047601	-0.30853	-0.30796	0.244187	-0.38473	0.312223	0.014107	-0.37444
0.183673	0.595119	0.000417	0.000428	0.005663	7.97E-06	0.000352	0.874915	1.45E-05
-0.58451	0.363335	-0.11857	-0.61491	0.02476	0.194592	-0.04654	0.346003	0.149626
5.41E-13	2.69E-05	0.184297	1.46E-14	0.782305	0.028361	0.603384	6.76E-05	0.093154
-0.40764	0.226373	-0.24913	-0.53045	-0.1805	0.2029	-0.05964	0.259701	0.117705
1.97E-06	0.010492	0.004736	1.42E-10	0.042283	0.022149	0.505374	0.003193	0.187523
-0.13712	0.009212	-0.13286	-0.24451	0.144749	0.148214	-0.25321	0.030713	0.075024
0.124243	0.918132	0.13647	0.005598	0.104446	0.096318	0.004075	0.731765	0.401864
0.156557	0.001102	0.390101	0.219063	-0.0184	0.398331	0.258803	-0.00871	0.287179
0.078796	0.990192	5.8E-06	0.013345	0.837332	3.52E-06	0.003304	0.922533	0.001062
0.233603	-0.0779	0.114989	-0.01127	0.074195	0.411761	0.106799	0.039139	0.062094
0.008212	0.384004	0.197983	0.899894	0.4071	1.51E-06	0.232056	0.662194	0.487987
-0.08585	0.104482	0.083351	-0.14176	0.021212	0.449426	-0.12494	0.115394	0.316195
0.337197	0.242398	0.351517	0.111877	0.812883	1.16E-07	0.161625	0.196398	0.000293
0.3152	-0.23692	0.002026	0.234057	-0.25355	-0.03104	0.131029	-0.04765	0.425888
0.000307	0.007321	0.981964	0.008085	0.004024	0.729053	0.142008	0.594735	5.98E-07
0.512097	-0.52188	0.370606	0.662985	0.128865	0.047499	0.418102	-0.37037	0.113296
7.56E-10	3.14E-10	1.79E-05	2.04E-17	0.148765	0.595905	1E-06	1.82E-05	0.204709
0.825764	-0.59001	0.357678	0.873413	0.518022	-0.44783	0.737528	-0.58404	-0.47451
7.01E-33	2.89E-13	3.65E-05	7.07E-41	4.45E-10	1.3E-07	4.68E-23	5.7E-13	1.74E-08
0.792656	-0.54573	0.391248	0.702045	0.256454	0.095506	0.474661	-0.39209	-0.06032
1.23E-28	3.24E-11	5.41E-06	3.74E-20	0.00361	0.285476	1.72E-08	5.14E-06	0.500557
0.245477	-0.16346	-0.25727	-0.02273	-0.13116	-0.2192	-0.20866	0.056544	-0.16722
0.005407	0.066311	0.003501	0.799722	0.1416	0.013286	0.018565	0.527765	0.060234
0.165758	-0.16592	-0.08908	0.29293	-0.03921	-0.45773	0.074527	-0.16332	-0.20613
0.062543	0.062286	0.319256	0.000831	0.661602	6.28E-08	0.404994	0.066563	0.02007
-0.6264	0.54437	-0.28163	-0.44464	-0.2917	-0.10033	-0.59311	0.310013	0.023743
3.37E-15	3.71E-11	0.001339	1.63E-07	0.000876	0.261706	2.02E-13	0.00039	0.791039
-0.18852	0.242819	-0.36855	-0.14136	-0.26821	-0.35316	-0.46062	0.070087	-0.22644
0.033789	0.005947	2.01E-05	0.112918	0.002298	4.65E-05	5.06E-08	0.433632	0.01047
-0.00716	-0.1951	-0.09507	0.006274	-0.09353	-0.0594	0.383058	-0.1593	-0.03094
0.936281	0.027939	0.287667	0.944185	0.295616	0.507096	8.79E-06	0.07363	0.72983

STX19	SUGP1	SUPT7L	SURF2	SURF6	SYCP2	TAB1	TAB3	TAF3
black	black	black	black	black	black	black	black	black
0.176421	-0.18658	0.411196	-0.07128	-0.16968	0.007968	-0.18449	-0.04839	-0.019
0.047245	0.035695	1.57E-06	0.425855	0.056506	0.929151	0.037854	0.588995	0.832065
0.279182	-0.16386	0.458076	-0.01613	-0.20297	0.063846	-0.19358	-0.05319	-0.11411
0.00148	0.065654	6.12E-08	0.857174	0.0221	0.47577	0.029212	0.552603	0.201441
0.046368	0.063497	-0.17126	-0.12265	0.032518	0.031786	0.071469	0.258874	-0.05075
0.604703	0.478193	0.054213	0.169519	0.716655	0.722774	0.424594	0.003295	0.570927
0.258744	-0.39461	0.184119	-0.45339	-0.4045	0.489678	-0.33633	0.480818	-0.36004
0.003311	4.42E-06	0.038251	8.66E-08	2.4E-06	5.14E-09	0.000111	1.06E-08	3.22E-05
0.199808	-0.13838	0.095273	0.547453	-0.16013	0.141683	-0.19423	-0.25294	-0.17829
0.024308	0.120777	0.28666	2.73E-11	0.072128	0.112077	0.028658	0.004117	0.044919
0.387312	-0.62974	0.318708	-0.1512	-0.57316	0.617259	-0.58968	0.281345	-0.51183
6.84E-06	2.18E-15	0.00026	0.089733	1.89E-12	1.09E-14	3.01E-13	0.001354	7.74E-10
0.311155	-0.32856	0.355068	-0.18975	-0.47813	0.484495	-0.44433	0.166944	-0.54246
0.00037	0.000162	4.2E-05	0.032621	1.31E-08	7.85E-09	1.67E-07	0.060663	4.47E-11
0.091835	-0.39415	0.205532	-0.0058	-0.18466	0.150539	-0.40672	0.024583	-0.31651
0.304483	4.54E-06	0.020442	0.948385	0.037683	0.091151	2.09E-06	0.783821	0.000289
-0.19674	-0.09794	-0.08309	-0.44817	0.031428	0.030045	-0.17984	-0.047	-0.08588
0.026623	0.2733	0.353041	1.27E-07	0.725767	0.737385	0.04306	0.599747	0.337067
-0.13955	-0.2488	0.135941	-0.10417	0.044563	0.079866	-0.30143	-0.35788	-0.0217
0.117643	0.004793	0.127532	0.243808	0.618852	0.372087	0.000574	3.61E-05	0.808632
0.01701	-0.36581	0.190429	-0.2487	-0.15029	0.260083	-0.31947	-0.12141	-0.18539
0.849456	2.34E-05	0.031995	0.004812	0.091687	0.003147	0.000251	0.17392	0.036919
-0.24857	0.488383	0.031284	-0.0694	0.09906	-0.27231	0.469419	-0.18905	0.204961
0.004834	5.72E-09	0.726979	0.438172	0.267845	0.001954	2.59E-08	0.033279	0.020803
-0.34842	0.631551	-0.30666	-0.07881	0.589626	-0.5155	0.584364	-0.13756	0.44881
5.97E-05	1.71E-15	0.000454	0.37848	3.02E-13	5.58E-10	5.5E-13	0.123009	1.21E-07
-0.47018	0.733938	-0.52719	0.583427	0.756535	-0.69966	0.660575	-0.6598	0.517757
2.44E-08	9.66E-23	1.92E-10	6.11E-13	8.22E-25	5.66E-20	2.92E-17	3.28E-17	4.56E-10
-0.4976	0.456333	-0.27447	0.08592	0.665228	-0.52422	0.393382	-0.58384	0.464603
2.65E-09	6.97E-08	0.001792	0.336817	1.46E-17	2.53E-10	4.76E-06	5.83E-13	3.75E-08
-0.00953	0.199126	0.071642	0.261665	0.183485	-0.1942	0.232979	-0.29634	0.282556
0.91529	0.024808	0.423471	0.002962	0.038933	0.028688	0.00839	0.000717	0.001288
-0.09892	0.560515	-0.31846	0.28205	0.298781	-0.40978	0.595907	-0.00929	0.310735
0.26854	7.25E-12	0.000263	0.001315	0.000645	1.72E-06	1.46E-13	0.917477	0.000377
0.339246	-0.29113	0.104863	-0.11734	-0.50105	0.366866	-0.20678	0.616007	-0.26869
9.55E-05	0.000898	0.240674	0.188912	1.98E-09	2.21E-05	0.019675	1.27E-14	0.002255
0.113407	0.099365	-0.00787	0.173109	-0.12156	-0.0715	0.104213	0.203723	-0.00454
0.204264	0.266368	0.930057	0.051623	0.173371	0.424404	0.243619	0.021602	0.959578
-0.01099	0.346644	-0.06426	0.118222	0.128656	-0.16738	0.318232	0.232078	0.342778
0.902401	6.54E-05	0.472906	0.185579	0.149432	0.059985	0.000266	0.008653	7.98E-05

TAF4B	TAF6L	TAPT1	TBC1D8B	TBCK	TBK1	TBL3	TCAP	TELO2
black	black	black	black	black	black	black	black	black
0.272693	-0.30811	0.249033	0.321748	-0.02607	0.243024	-0.18208	-0.11372	-0.33086
0.001924	0.000425	0.004753	0.000225	0.771063	0.005904	0.040484	0.203013	0.000145
0.263664	-0.31792	0.344437	0.240229	-0.04562	0.213122	-0.18	-0.06571	-0.35937
0.002743	0.00027	7.33E-05	0.00652	0.610517	0.01614	0.042861	0.462961	3.33E-05
-0.1471	0.265734	-0.2202	-0.15687	0.315612	0.015929	-0.00825	-0.0234	0.07718
0.098879	0.002532	0.012861	0.078193	0.000301	0.858918	0.926697	0.79399	0.388435
0.083079	-0.18732	0.030842	0.212277	0.57719	0.476805	-0.45634	-0.34538	-0.38769
0.353093	0.034958	0.730687	0.016577	1.22E-12	1.45E-08	6.97E-08	6.98E-05	6.69E-06
-0.02823	-0.10558	0.018148	0.030109	-0.18366	0.121299	0.026367	0.160128	-0.20237
0.752714	0.23746	0.839516	0.736849	0.038744	0.174311	0.768565	0.072124	0.022505
0.16331	-0.44332	0.157918	0.377372	0.343667	0.656029	-0.51698	-0.33294	-0.60393
0.066573	1.79E-07	0.076197	1.22E-05	7.63E-05	5.7E-17	4.89E-10	0.000131	5.63E-14
0.134992	-0.36389	0.090234	0.307013	0.346858	0.441218	-0.40375	-0.20412	-0.56087
0.130235	2.61E-05	0.313025	0.000447	6.47E-05	2.08E-07	2.51E-06	0.021342	6.99E-12
-0.08286	-0.21754	0.16316	0.072435	0.076943	0.273034	-0.29772	0.002126	-0.22363
0.354364	0.014018	0.066827	0.418343	0.389896	0.001898	0.000675	0.981076	0.011494
-0.09128	0.01785	-0.45251	0.35618	0.098187	0.112928	0.03512	-0.28806	0.10257
0.307446	0.842117	9.24E-08	3.96E-05	0.272106	0.206193	0.695069	0.001023	0.251175
-0.09873	-0.19471	-0.20371	0.401028	-0.12927	0.238969	0.036177	-0.14667	-0.02053
0.269443	0.028264	0.021612	2.98E-06	0.147496	0.006815	0.686367	0.099864	0.818808
-0.02125	-0.23215	-0.04422	0.349859	0.007987	0.3558	-0.18893	-0.32994	-0.20264
0.812594	0.008632	0.621526	5.53E-05	0.928987	4.04E-05	0.033399	0.000152	0.022327
-0.11223	0.297589	-0.25263	-0.15737	0.037112	-0.56586	0.287874	0.033904	0.264841
0.209019	0.000679	0.004163	0.077227	0.6787	4.14E-12	0.001031	0.705123	0.002621
-0.32846	0.62582	-0.39743	-0.37763	0.006249	-0.54889	0.499588	0.280304	0.616918
0.000163	3.64E-15	3.72E-06	1.2E-05	0.94441	2.37E-11	2.24E-09	0.001414	1.14E-14
-0.52494	0.795588	-0.4833	-0.52618	-0.58614	-0.67894	0.938125	0.59128	0.919203
2.37E-10	5.55E-29	8.65E-09	2.11E-10	4.5E-13	1.75E-18	2.05E-59	2.5E-13	1.98E-52
-0.45984	0.491005	-0.45908	-0.13455	-0.35657	-0.43533	0.659137	0.272336	0.697793
5.37E-08	4.61E-09	5.68E-08	0.131502	3.88E-05	3.14E-07	3.62E-17	0.001952	7.8E-20
-0.08102	-0.03566	0.391912	-0.06653	-0.29006	-0.22224	0.117033	0.174909	0.07245
0.365175	0.690591	5.2E-06	0.457374	0.00094	0.012031	0.190072	0.049204	0.418251
-0.11142	0.402942	0.110699	-0.55637	-0.08086	-0.58742	0.260827	0.31948	0.338859
0.212349	2.64E-06	0.21535	1.11E-11	0.366138	3.89E-13	0.003059	0.000251	9.74E-05
0.43795	-0.36394	0.479249	0.017028	0.22902	0.282358	-0.51199	-0.21749	-0.45363
2.62E-07	2.6E-05	1.2E-08	0.849299	0.0096	0.001299	7.64E-10	0.014042	8.51E-08
0.321444	-0.14498	0.326569	-0.19369	-0.11094	-0.16475	-0.14276	0.066514	-0.12903
0.000229	0.103899	0.000179	0.029118	0.214339	0.064176	0.109339	0.457491	0.148236
0.101081	0.232884	-0.16549	-0.10865	0.199767	-0.2408	0.128817	0.209302	0.050779
0.258157	0.008417	0.062978	0.224025	0.024338	0.00639	0.148918	0.018196	0.570745

TFAP4	TGOLN2	THAP4	THAP7	THAP8	THOC3	THOP1	THUMPD1	TICAM1
black	black	black	black	black	black	black	black	black
-0.28738	0.35498	-0.11497	-0.04423	-0.11097	-0.24125	-0.28544	0.23738	-0.06019
0.001053	4.22E-05	0.198059	0.621491	0.214237	0.006287	0.001142	0.007205	0.501426
-0.28973	0.24267	-0.04512	-0.02318	-0.09934	-0.29635	-0.2785	0.242733	-0.11024
0.000953	0.005979	0.6145	0.795877	0.266465	0.000717	0.001522	0.005966	0.217282
0.301133	-0.42157	-0.13985	-0.06116	0.218991	0.093502	0.055335	-0.02623	-0.16853
0.000581	7.98E-07	0.116844	0.4946	0.013376	0.295754	0.53664	0.76972	0.058217
-0.11865	-0.0543	-0.53413	-0.38665	-0.17236	-0.13904	-0.46945	0.351617	-0.29886
0.183991	0.544275	1E-10	7.12E-06	0.052657	0.118998	2.58E-08	5.04E-05	0.000642
-0.08463	-0.40164	0.156237	0.456965	0.112908	0.18416	-0.19202	0.086346	-0.25506
0.344163	2.87E-06	0.079418	6.65E-08	0.206275	0.038208	0.030566	0.334421	0.003805
-0.35948	-0.07674	-0.46224	-0.1641	-0.25261	-0.08464	-0.68993	0.483	-0.40613
3.31E-05	0.391124	4.48E-08	0.065257	0.004167	0.344115	2.94E-19	8.86E-09	2.16E-06
-0.36026	0.129839	-0.21429	-0.20348	-0.17352	-0.31724	-0.57743	0.314246	-0.2715
3.18E-05	0.145696	0.015555	0.02176	0.051063	0.000279	1.19E-12	0.000321	0.002018
-0.39577	0.273019	-0.35571	-0.11212	-0.30315	-0.1603	-0.30928	0.231948	-0.22773
4.12E-06	0.001899	4.06E-05	0.209493	0.000531	0.071819	0.000403	0.008691	0.010024
-0.04123	0.288642	-0.0962	-0.20833	0.081139	-0.12481	-0.11715	-0.08551	0.250867
0.645341	0.000998	0.281998	0.018753	0.364487	0.162092	0.18961	0.339121	0.004444
-0.33664	0.513043	-0.13869	-0.04446	0.023293	-0.25142	-0.29722	-0.04207	0.215521
0.000109	6.95E-10	0.119948	0.619672	0.794912	0.004354	0.00069	0.638662	0.014955
-0.36358	0.467366	-0.40322	-0.23731	-0.12562	-0.27069	-0.42472	0.124288	0.112612
2.65E-05	3.03E-08	2.6E-06	0.007222	0.159354	0.002084	6.47E-07	0.163857	0.207477
0.210251	0.061586	0.24134	-0.0823	0.308578	-0.25971	0.203358	-0.34732	0.283585
0.017665	0.491559	0.006269	0.357668	0.000416	0.003192	0.021843	6.32E-05	0.001234
0.549711	-0.08562	0.248775	0.044588	0.494779	0.012329	0.577606	-0.50173	0.34518
2.18E-11	0.33852	0.004798	0.618656	3.36E-09	0.890577	1.17E-12	1.87E-09	7.06E-05
0.660182	-0.42314	0.639115	0.726744	0.700782	0.414215	0.858112	-0.74432	0.515204
3.1E-17	7.19E-07	6.18E-16	3.99E-22	4.66E-20	1.29E-06	5.39E-38	1.15E-23	5.73E-10
0.306378	0.217382	0.286783	0.236899	0.508005	-0.04505	0.473225	-0.59584	0.544849
0.00046	0.014089	0.00108	0.007327	1.08E-09	0.615005	1.93E-08	1.47E-13	3.54E-11
-0.04428	0.14892	0.050412	0.110407	0.002372	-0.15486	0.197756	-0.09058	-0.10038
0.621063	0.094725	0.57353	0.21657	0.978883	0.082135	0.02584	0.311152	0.261496
0.414174	-0.43265	0.325664	0.173855	0.170863	0.159754	0.598315	-0.32223	-0.02751
1.29E-06	3.78E-07	0.000187	0.050608	0.054777	0.072801	1.1E-13	0.00022	0.758803
-0.17586	-0.1569	-0.20736	-0.23681	-0.54932	0.134497	-0.19722	0.4997	-0.37681
0.047959	0.078126	0.019325	0.00735	2.27E-11	0.131663	0.026252	2.22E-09	1.26E-05
-0.05282	-0.10065	0.232592	0.006294	-0.30528	0.163192	0.177947	0.127696	-0.13296
0.555343	0.260207	0.008502	0.944008	0.000483	0.066772	0.045335	0.152516	0.136182
0.434161	-0.38633	0.200805	0.109629	0.24185	0.082928	0.154502	-0.01509	-0.13606
3.4E-07	7.26E-06	0.023593	0.219844	0.006156	0.353974	0.082853	0.866243	0.127202

TICAM2	TIGD5	TIMM13	TIMM17B	TIMM44	TIMM50	TKFC	TM9SF3	TMED1
black	black	black	black	black	black	black	black	black
-0.17262	-0.4412	-0.29094	-0.03043	-0.37425	-0.29089	-0.01694	0.098207	-0.15576
0.052297	2.08E-07	0.000905	0.734131	1.46E-05	0.000907	0.850109	0.272006	0.080352
-0.24388	-0.4884	-0.27028	0.00838	-0.355	-0.20297	0.037757	0.104712	-0.16628
0.005725	5.71E-09	0.002118	0.925498	4.22E-05	0.022099	0.673434	0.241359	0.061714
-0.02459	0.268579	0.112373	-0.10639	0.384302	0.305565	-0.28335	0.052837	0.07
0.783763	0.002265	0.208447	0.233857	8.17E-06	0.000477	0.001246	0.555213	0.434205
-0.16262	-0.1513	-0.43453	-0.46144	0.015713	-0.19137	-0.56089	0.403445	-0.08928
0.067741	0.0895	3.32E-07	4.76E-08	0.860812	0.031144	6.97E-12	2.56E-06	0.31821
-0.32811	-0.06289	0.087777	0.58804	0.103075	0.214961	0.082919	-0.18457	0.152228
0.000166	0.482384	0.326438	3.62E-13	0.248834	0.015225	0.354026	0.037777	0.087537
-0.37232	-0.34166	-0.50631	-0.11163	-0.16988	-0.25055	-0.46647	0.372747	-0.06348
1.63E-05	8.45E-05	1.26E-09	0.211489	0.05621	0.004496	3.25E-08	1.59E-05	0.47829
-0.43592	-0.44717	-0.42259	-0.1362	-0.15409	-0.24161	-0.25199	0.21716	-0.01864
3.01E-07	1.36E-07	7.45E-07	0.126809	0.083695	0.00621	0.004263	0.01419	0.835265
-0.06236	-0.25296	-0.41674	-0.11626	-0.34216	-0.40251	0.016575	0.094328	-0.16148
0.486128	0.004113	1.1E-06	0.193029	8.24E-05	2.72E-06	0.85326	0.291488	0.069715
0.142064	0.092093	0.01181	-0.37784	0.125502	-0.20896	-0.17542	0.153128	0.39618
0.111106	0.30312	0.895159	1.19E-05	0.159748	0.018392	0.048531	0.085659	4.01E-06
0.090215	-0.1124	-0.13781	-0.11385	-0.23528	-0.38447	0.065762	0.106702	0.258271
0.313128	0.208349	0.122333	0.202481	0.00775	8.1E-06	0.462601	0.232478	0.003371
0.074377	-0.22488	-0.32983	-0.29551	-0.27106	-0.41966	-0.15546	0.181268	0.141019
0.405944	0.011027	0.000153	0.000744	0.002053	9.05E-07	0.080947	0.041398	0.113786
0.145865	0.002572	0.258722	-0.26826	0.311195	0.091797	0.073976	-0.35256	0.454318
0.10177	0.977102	0.003314	0.002294	0.000369	0.304685	0.408487	4.8E-05	8.09E-08
0.371361	0.495301	0.473843	-0.15608	0.463583	0.306578	0.223993	-0.29673	0.248799
1.72E-05	3.22E-09	1.84E-08	0.079729	4.05E-08	0.000456	0.011355	0.000705	0.004794
0.479428	0.821325	0.926354	0.561508	0.693586	0.641627	0.699056	-0.60229	0.580863
1.18E-08	2.92E-32	7.55E-55	6.53E-12	1.59E-19	4.38E-16	6.28E-20	6.86E-14	8.13E-13
0.459967	0.482087	0.501766	0.019401	0.269653	0.083167	0.473676	-0.30183	0.474743
5.32E-08	9.54E-09	1.86E-09	0.828604	0.002171	0.352586	1.86E-08	0.000563	1.71E-08
-4.7E-05	-0.10245	0.090649	0.201947	-0.25262	0.00993	0.27896	-0.02977	-0.25449
0.999584	0.251735	0.310798	0.022796	0.004166	0.911769	0.001494	0.739708	0.003885
0.118088	0.295833	0.463933	0.24335	0.321741	0.558863	0.166809	-0.30831	-0.15142
0.186081	0.000733	3.94E-08	0.005836	0.000226	8.6E-12	0.060875	0.000421	0.089246
-0.23082	-0.30998	-0.34508	-0.04239	-0.26401	-0.0279	-0.35745	0.309652	-0.54975
0.009031	0.00039	7.09E-05	0.636039	0.002707	0.75548	3.7E-05	0.000396	2.17E-11
-0.11812	-0.18732	0.000328	0.216185	-0.18979	0.139438	0.064994	-0.08889	-0.4094
0.185975	0.034958	0.997077	0.014641	0.032582	0.117936	0.467856	0.320296	1.76E-06
-0.24293	0.129727	0.217071	0.169431	0.309229	0.305287	-0.1419	-0.19692	-0.0933
0.005924	0.146045	0.014231	0.056871	0.000404	0.000483	0.111528	0.026482	0.296805

TMED3	TMEFF1	TMEM115	TMEM121	TMEM129	TMEM132/	TMEM134	TMEM160	TMEM161/
black	black	black	black	black	black	black	black	black
0.142004	-0.24259	-0.1546	-0.32566	-0.17894	-0.27204	-0.09127	-0.31255	-0.29307
0.111258	0.005997	0.082665	0.000187	0.044121	0.001975	0.307467	0.000347	0.000826
0.103456	-0.27209	-0.11733	-0.30059	-0.16498	-0.24264	-0.08069	-0.27319	-0.28948
0.24708	0.001971	0.188953	0.000595	0.063796	0.005985	0.367187	0.001887	0.000963
-0.30942	0.01481	-0.01321	0.061848	-0.11348	-0.00272	-0.05379	-0.11829	0.176119
0.0004	0.868737	0.882782	0.489716	0.203983	0.975781	0.548108	0.185311	0.047631
-0.26717	-0.39084	-0.28437	-0.38568	-0.33095	-0.39126	-0.38796	-0.55721	-0.26611
0.002393	5.54E-06	0.001195	7.54E-06	0.000144	5.41E-06	6.59E-06	1.02E-11	0.002495
0.42885	-0.27296	0.083292	-0.03389	0.137586	-0.21782	0.411753	0.00517	-0.22146
4.9E-07	0.001904	0.35186	0.705236	0.122944	0.013892	1.51E-06	0.953999	0.012343
0.142123	-0.62198	-0.28046	-0.49376	-0.2638	-0.54539	-0.17758	-0.59594	-0.54627
0.110955	5.97E-15	0.001404	3.66E-09	0.002729	3.35E-11	0.045784	1.46E-13	3.07E-11
0.161781	-0.57534	-0.14737	-0.4596	-0.16543	-0.46619	-0.20148	-0.56346	-0.35875
0.069195	1.5E-12	0.098248	5.47E-08	0.063072	3.32E-08	0.023118	5.33E-12	3.45E-05
0.137508	-0.29342	-0.16441	-0.35996	0.074107	-0.32822	-0.20267	-0.3101	-0.3878
0.12316	0.000814	0.064739	3.23E-05	0.407655	0.000165	0.022306	0.000388	6.65E-06
0.33451	-0.19769	0.350852	-0.12037	-0.11135	-0.05099	-0.07397	-0.24073	0.238749
0.000121	0.02589	5.25E-05	0.177654	0.212645	0.569147	0.408546	0.006406	0.006868
0.616203	-0.31074	0.251865	-0.21546	0.118067	-0.21584	0.160189	-0.26621	-0.0714
1.24E-14	0.000377	0.004283	0.014984	0.186162	0.014805	0.072015	0.002485	0.425021
0.269116	-0.34072	-0.01684	-0.34167	-0.04225	-0.24832	-0.11697	-0.44437	-0.21688
0.002217	8.87E-05	0.850926	8.45E-05	0.637162	0.004877	0.190305	1.66E-07	0.014318
-0.0598	0.194959	0.33473	0.17844	-0.06897	0.181727	-0.06574	0.169115	0.415714
0.504233	0.028058	0.00012	0.044731	0.440991	0.040878	0.462777	0.057341	1.17E-06
-0.28038	0.505242	0.368439	0.397726	0.165895	0.389915	0.12381	0.318406	0.69662
0.001409	1.38E-09	2.03E-05	3.65E-06	0.062323	5.86E-06	0.165498	0.000264	9.53E-20
0.165504	0.675722	0.715923	0.812237	0.632952	0.662474	0.818275	0.824033	0.849048
0.062951	2.91E-18	3.09E-21	4.79E-31	1.42E-15	2.21E-17	7.59E-32	1.23E-32	1.92E-36
0.294037	0.399477	0.631448	0.393503	0.413301	0.36603	0.458544	0.348427	0.648294
0.000793	3.28E-06	1.73E-15	4.72E-06	1.37E-06	2.32E-05	5.91E-08	5.96E-05	1.73E-16
-0.09024	0.329163	-0.05301	0.189584	0.172735	0.072941	0.12952	0.262812	-0.03668
0.313002	0.000158	0.553887	0.032779	0.052137	0.415097	0.146695	0.002835	0.68222
-0.5492	0.590598	-0.05535	0.466748	0.123772	0.418011	0.069418	0.534493	0.315679
2.3E-11	2.7E-13	0.536538	3.18E-08	0.16563	1.01E-06	0.438045	9.65E-11	0.0003
-0.46066	-0.10653	-0.63839	-0.15111	-0.33261	-0.10178	-0.44347	-0.08302	-0.47736
5.05E-08	0.233229	6.82E-16	0.089911	0.000133	0.254848	1.77E-07	0.353412	1.39E-08
-0.26884	0.165877	-0.33224	0.086745	-0.07527	0.165817	-0.18979	0.272403	-0.18412
0.002242	0.062351	0.000136	0.332181	0.400318	0.062447	0.032582	0.001946	0.038248
-0.24522	0.187877	-0.02928	0.171207	-0.28573	-0.06829	0.073919	0.08587	0.131436
0.005458	0.034413	0.743857	0.054284	0.001129	0.445518	0.408852	0.337099	0.14076

TMEM167	TMEM181	TMEM184	TMEM191	TMEM201	TMEM222	TMEM238	TMEM254	TMEM259
black	black	black	black	black	black	black	black	black
0.244827	-0.08313	-0.30679	-0.07822	-0.34711	0.022529	-0.1729	0.181606	-0.25658
0.005535	0.352794	0.000451	0.382033	6.39E-05	0.801499	0.051914	0.041015	0.003593
0.258951	-0.02899	-0.3496	-0.10186	-0.34073	0.086481	-0.19608	0.268859	-0.30508
0.003285	0.746325	5.61E-05	0.254486	8.86E-05	0.333661	0.027151	0.00224	0.000487
-0.09893	0.215394	0.268428	-0.16764	0.256529	-0.13506	0.072204	-0.02405	0.066736
0.268486	0.015016	0.002278	0.059587	0.0036	0.130029	0.419835	0.788429	0.455987
0.42739	0.437614	0.056214	-0.43577	-0.24063	-0.46072	-0.18095	0.236629	-0.32417
5.4E-07	2.68E-07	0.530179	3.05E-07	0.006429	5.03E-08	0.041764	0.007396	0.000201
0.207167	-0.13606	-0.31407	-0.02492	-0.34747	0.403328	0.126335	0.10905	-0.37788
0.01944	0.1272	0.000323	0.780904	6.27E-05	2.58E-06	0.156973	0.222305	1.19E-05
0.673385	0.345167	-0.25009	-0.42846	-0.60881	-0.24201	-0.16496	0.369733	-0.66927
4.19E-18	7.06E-05	0.004572	5.03E-07	3.11E-14	0.006121	0.06383	1.89E-05	7.91E-18
0.509311	0.153684	-0.39541	-0.46055	-0.38146	-0.05575	-0.19368	0.289478	-0.45905
9.67E-10	0.084514	4.21E-06	5.09E-08	9.65E-06	0.533549	0.029124	0.000964	5.7E-08
0.331357	-0.13931	-0.27285	-0.13609	-0.28744	-0.10515	-0.01435	0.414347	-0.33027
0.000142	0.118282	0.001912	0.127102	0.00105	0.239374	0.872817	1.28E-06	0.000149
0.068888	0.019386	0.263484	-0.32099	0.028802	-0.31288	0.126612	-0.2352	0.246111
0.441559	0.828728	0.002762	0.000234	0.747875	0.000342	0.156059	0.007772	0.005285
0.213188	-0.29529	-0.06505	-0.28702	-0.33683	-0.05532	0.098167	-0.09784	0.038934
0.016107	0.000751	0.467503	0.001069	0.000108	0.536784	0.272204	0.273835	0.663863
0.355603	-0.07043	-0.06667	-0.33012	-0.40601	-0.16242	-0.08596	0.075222	-0.12908
4.08E-05	0.431374	0.456407	0.00015	2.18E-06	0.068085	0.336589	0.400619	0.148074
-0.30054	-0.08415	0.262244	0.011099	0.220769	0.112744	-0.00095	-0.07461	0.481445
0.000597	0.346891	0.002897	0.901439	0.012624	0.206939	0.991538	0.4045	1E-08
-0.62138	-0.26689	0.464843	0.116988	0.725748	0.075518	0.230472	-0.34864	0.783325
6.45E-15	0.00242	3.68E-08	0.190244	4.83E-22	0.39876	0.009139	5.9E-05	1.4E-27
-0.76661	-0.60245	0.385582	0.476452	0.66756	0.601583	0.574438	-0.58537	0.856443
8.27E-26	6.73E-14	7.58E-06	1.49E-08	1.03E-17	7.46E-14	1.65E-12	4.9E-13	1.06E-37
-0.50575	-0.59183	0.368843	0.100711	0.460803	0.20572	0.391215	-0.46725	0.806286
1.32E-09	2.35E-13	1.98E-05	0.259911	4.99E-08	0.020325	5.42E-06	3.06E-08	2.76E-30
-0.18632	-0.18505	-0.07156	0.250961	0.06797	0.20822	-0.05202	-0.03971	0.091584
0.035957	0.037273	0.424032	0.004428	0.447682	0.018817	0.561378	0.657602	0.305815
-0.55306	-0.0373	0.193218	0.397398	0.555743	0.249627	0.022602	-0.20304	0.3261
1.56E-11	0.677166	0.02952	3.73E-06	1.18E-11	0.004651	0.800865	0.022055	0.000183
0.261395	0.580983	-0.21686	0.123321	-0.24896	-0.25515	-0.36897	0.271365	-0.56867
0.002993	8.02E-13	0.014326	0.167189	0.004766	0.003792	1.97E-05	0.002029	3.07E-12
-0.10577	0.165989	-0.24324	0.360447	0.022289	0.113266	-0.21267	0.092726	-0.22222
0.236622	0.062173	0.005858	3.15E-05	0.803568	0.20483	0.016372	0.299794	0.012037
-0.32164	-0.00333	0.191383	0.053145	0.383086	0.006439	-0.02407	-0.13023	0.118169
0.000227	0.970386	0.03113	0.552902	8.78E-06	0.942728	0.788186	0.144466	0.185779

TMEM30A	TMEM54	TMEM91	TMUB1	TNFRSF18	TNFRSF4	TNKS2	TOMM40	TOR2A
black	black	black	black	black	black	black	black	black
0.07943	-0.08038	0.07027	-0.26352	0.052789	-0.20826	0.090813	-0.39897	-0.07381
0.374712	0.368995	0.432431	0.002758	0.555569	0.018792	0.309917	3.38E-06	0.409556
0.070586	-0.01244	0.092319	-0.27816	-0.00912	-0.31784	0.036507	-0.31633	-0.10009
0.430359	0.889631	0.301929	0.001543	0.918908	0.000271	0.683651	0.000291	0.262862
-0.12692	-0.2121	-0.26863	0.046423	-0.20857	0.124511	-0.03057	0.317474	0.057121
0.155046	0.016668	0.00226	0.604273	0.018612	0.163097	0.732991	0.000276	0.523555
0.230316	-0.37563	-0.47867	-0.36347	-0.38663	-0.0303	0.26302	-0.19072	-0.29695
0.009188	1.35E-05	1.25E-08	2.67E-05	7.13E-06	0.735202	0.002812	0.031733	0.000698
-0.34133	0.388684	0.148256	-0.079	0.091699	-0.07199	-0.47729	-0.03221	0.066888
8.59E-05	6.31E-06	0.096224	0.377295	0.305202	0.421207	1.4E-08	0.719228	0.454958
0.166509	-0.1226	-0.38404	-0.483	-0.28796	-0.13469	0.055048	-0.40967	-0.32444
0.061346	0.169692	8.3E-06	8.86E-09	0.001028	0.131091	0.538762	1.73E-06	0.000198
0.139102	-0.11663	-0.20149	-0.46274	-0.14397	-0.28114	-0.04265	-0.19688	-0.27181
0.118831	0.191602	0.023111	4.31E-08	0.106354	0.001366	0.634028	0.026514	0.001993
0.186296	0.161239	0.009276	-0.25552	-0.18534	-0.16368	-0.05104	-0.39521	-0.33109
0.035986	0.070145	0.917567	0.00374	0.036969	0.065945	0.568768	4.26E-06	0.000143
-0.0399	-0.21959	-0.06849	0.138285	0.211376	0.439741	0.028151	0.111606	0.098644
0.656033	0.013116	0.444199	0.121034	0.017053	2.31E-07	0.753393	0.21159	0.269871
-0.03376	0.033129	0.11272	0.062958	0.264434	0.19979	-0.02913	-0.29284	0.16859
0.706346	0.711563	0.207036	0.481941	0.002663	0.024321	0.745103	0.000834	0.05813
0.117677	-0.07956	-0.11425	-0.10905	0.033622	0.079515	0.146103	-0.47802	-0.04095
0.187631	0.373934	0.200916	0.222317	0.707465	0.374199	0.101208	1.32E-08	0.647563
-0.36604	0.07691	0.247953	0.192762	0.197243	0.087696	-0.14448	0.204743	0.200004
2.31E-05	0.390098	0.004944	0.029913	0.026234	0.326885	0.105097	0.020941	0.024166
-0.30813	-0.06145	0.242245	0.505406	0.14384	0.285196	-0.08899	0.583938	0.389759
0.000425	0.492532	0.00607	1.36E-09	0.106666	0.001154	0.319745	5.77E-13	5.92E-06
-0.65597	0.539066	0.564489	0.917174	0.536606	0.516907	-0.50349	0.691848	0.651961
5.74E-17	6.22E-11	4.78E-12	8.74E-52	7.89E-11	4.92E-10	1.61E-09	2.13E-19	1.02E-16
-0.37509	0.185852	0.43596	0.671719	0.458815	0.469772	-0.21278	0.345591	0.540868
1.39E-05	0.036438	3.01E-07	5.43E-18	5.8E-08	2.52E-08	0.016316	6.91E-05	5.22E-11
0.024567	0.064645	0.144781	0.017227	0.019358	-0.26785	0.035296	-0.10653	0.053522
0.783959	0.470253	0.104369	0.847556	0.828971	0.002331	0.693614	0.233244	0.550089
-0.13702	0.038174	0.036332	0.22445	-0.09186	-0.09745	-0.06867	0.438811	0.110785
0.124505	0.670031	0.685092	0.011185	0.304357	0.275746	0.442999	2.46E-07	0.214989
0.498323	-0.24457	-0.41897	-0.49311	-0.38227	-0.3699	0.403089	-0.28185	-0.45901
2.5E-09	0.005586	9.47E-07	3.87E-09	9.21E-06	1.87E-05	2.62E-06	0.001326	5.71E-08
0.215236	0.048495	0.0031	-0.1941	-0.08357	-0.35902	0.100207	-0.02455	-0.21499
0.015092	0.588217	0.972405	0.028774	0.350261	3.4E-05	0.262318	0.784082	0.015212
-0.35118	-0.29322	0.047814	-0.05529	-0.04646	-0.00356	-0.18018	0.393829	0.174643
5.16E-05	0.000821	0.593473	0.536971	0.603992	0.968334	0.04265	4.63E-06	0.049554

TP53I13	TPGS1	TRABD	TRAF2	TRAPPC11	TRAPPC12	TRAPPC5	TRAPPC6A	TRAPPC8
black	black	black	black	black	black	black	black	black
-0.26291	-0.24788	-0.22885	-0.05305	-0.15553	-0.18288	-0.1296	-0.02784	0.050262
0.002824	0.004957	0.009654	0.553586	0.080796	0.03959	0.146441	0.756039	0.574676
-0.33755	-0.30541	-0.33922	-0.08874	-0.1525	-0.09049	-0.12207	0.000348	0.06641
0.000104	0.00048	9.57E-05	0.321163	0.086957	0.311661	0.171581	0.996902	0.458197
0.172226	-0.10099	0.123269	0.263626	0.401271	-0.01242	-0.21817	-0.16903	0.014987
0.052844	0.25859	0.16737	0.002747	2.93E-06	0.88976	0.013734	0.05747	0.867185
-0.04631	-0.43718	-0.1942	-0.1789	0.596525	-0.39124	-0.58678	-0.45796	0.463183
0.605165	2.76E-07	0.028686	0.04418	1.36E-13	5.41E-06	4.18E-13	6.18E-08	4.17E-08
0.122917	-0.17275	-0.25713	0.013657	-0.22301	-0.04911	-0.11005	0.459867	0.048515
0.168595	0.052121	0.00352	0.878876	0.01173	0.583489	0.218048	5.36E-08	0.588057
-0.07478	-0.56984	-0.45934	-0.30544	0.383262	-0.4831	-0.66184	-0.19759	0.557332
0.40338	2.71E-12	5.57E-08	0.00048	8.69E-06	8.79E-09	2.42E-17	0.025963	1.01E-11
-0.22075	-0.57723	-0.43219	-0.2888	0.126011	-0.48037	-0.49241	-0.05151	0.288775
0.012634	1.21E-12	3.9E-07	0.000992	0.158048	1.09E-08	4.1E-09	0.565193	0.000993
-0.0112	-0.23469	-0.25334	-0.3449	0.031832	-0.24789	-0.36498	-0.12405	0.324206
0.900509	0.007911	0.004056	7.16E-05	0.722389	0.004954	2.45E-05	0.164676	0.0002
0.381225	0.096825	0.358459	0.151316	0.201233	-0.08266	0.043266	-0.17454	-0.18987
9.78E-06	0.278843	3.5E-05	0.089475	0.023291	0.355564	0.629104	0.049687	0.032513
0.366698	0.022893	0.259369	0.086673	-0.15723	-0.01163	-0.01422	0.123024	-0.10963
2.23E-05	0.798358	0.003234	0.332585	0.077499	0.896766	0.873959	0.168221	0.219835
0.142845	-0.15658	0.082424	-0.03919	0.036073	-0.17838	-0.20962	-0.16851	0.107923
0.109135	0.078742	0.356918	0.661777	0.68722	0.044802	0.018019	0.05825	0.22715
0.015066	0.284996	0.402205	0.212316	-0.08482	0.303663	0.589373	-0.05967	-0.31778
0.866489	0.001164	2.77E-06	0.016557	0.343068	0.000519	3.11E-13	0.505176	0.000272
0.33266	0.448543	0.67247	0.537469	-0.0592	0.456114	0.438873	0.053501	-0.55991
0.000133	1.23E-07	4.83E-18	7.26E-11	0.508512	7.08E-08	2.45E-07	0.550247	7.71E-12
0.748427	0.858676	0.787748	0.660321	-0.61218	0.728897	0.803331	0.710127	-0.79415
4.82E-24	4.28E-38	4.48E-28	3.04E-17	2.05E-14	2.62E-22	6.44E-30	8.92E-21	8.19E-29
0.65405	0.630397	0.828988	0.556221	-0.42374	0.562973	0.589015	0.362445	-0.70158
7.59E-17	1.99E-15	2.43E-33	1.13E-11	6.9E-07	5.61E-12	3.24E-13	2.82E-05	4.05E-20
-0.22957	0.137071	-0.06093	-0.0409	-0.41029	0.280249	0.240012	0.215569	-0.12242
0.009422	0.124367	0.496179	0.648008	1.66E-06	0.001417	0.00657	0.014933	0.170353
-0.19348	0.273486	0.087233	0.139839	-0.13906	0.344648	0.354603	0.111274	-0.2733
0.029298	0.001864	0.329458	0.116873	0.118932	7.25E-05	4.31E-05	0.212962	0.001878
-0.65237	-0.35914	-0.67739	-0.53501	0.319675	-0.41536	-0.36789	-0.37725	0.617701
9.67E-17	3.38E-05	2.24E-18	9.19E-11	0.000249	1.2E-06	2.09E-05	1.23E-05	1.03E-14
-0.57111	-0.06109	-0.43263	-0.31597	-0.07579	-0.08794	0.089182	-0.05476	0.158439
2.36E-12	0.495097	3.78E-07	0.000296	0.397038	0.32553	0.318723	0.5409	0.075221
-0.07793	-0.04671	0.132797	0.351084	0.142225	0.036856	0.046458	0.129102	-0.18949
0.383804	0.602042	0.13666	5.19E-05	0.110696	0.680798	0.603996	0.148013	0.032871

TREX2	TRIM11	TRIM44	TRIM52	TRMT1	TRMT2A	TRMT61A	TRPC1	TSC22D4
black	black	black	black	black	black	black	black	black
-0.11364	-0.08502	0.207273	-0.18612	-0.21194	-0.12367	-0.07961	0.157506	-0.10976
0.203348	0.34191	0.019377	0.03617	0.016753	0.165966	0.373613	0.076975	0.219278
-0.15693	-0.11503	0.18458	-0.2443	-0.2062	-0.1431	-0.06921	0.185063	-0.08905
0.078081	0.197805	0.037761	0.005641	0.020027	0.1085	0.439407	0.037255	0.319441
0.076598	-0.03727	-0.38531	0.35784	0.178416	0.030066	-0.01798	-0.14168	-0.29627
0.392029	0.67744	7.7E-06	3.62E-05	0.04476	0.737212	0.840965	0.112091	0.00072
-0.17134	-0.48675	-0.13777	0.659437	-0.16595	-0.32303	-0.4356	0.27302	-0.56164
0.054089	6.54E-09	0.122438	3.46E-17	0.062229	0.000212	3.08E-07	0.001899	6.44E-12
-0.16896	-0.07072	-0.45981	-0.3541	-0.10176	0.101522	0.113464	0.070161	-0.12546
0.057576	0.429452	5.38E-08	4.42E-05	0.254962	0.256075	0.204036	0.433141	0.159891
-0.3647	-0.59631	-0.22473	0.320893	-0.40762	-0.36929	-0.42793	0.412086	-0.58643
2.49E-05	1.39E-13	0.011082	0.000235	1.97E-06	1.93E-05	5.21E-07	1.48E-06	4.35E-13
-0.28255	-0.47862	-0.1086	0.26561	-0.16703	-0.24594	-0.40356	0.335967	-0.43707
0.001289	1.26E-08	0.224245	0.002544	0.060521	0.005318	2.54E-06	0.000113	2.78E-07
-0.16055	-0.30818	0.080112	0.283591	-0.376	-0.16409	-0.28645	0.308316	-0.0992
0.071366	0.000424	0.370609	0.001234	1.32E-05	0.065272	0.001095	0.000421	0.267175
0.178351	0.003914	-0.07569	0.183299	0.121384	-0.0134	-0.12009	-0.18605	-0.2492
0.044839	0.965162	0.397661	0.039135	0.17401	0.881126	0.178662	0.03624	0.004725
0.06531	-0.10312	0.00099	-0.11325	-0.06533	0.015953	0.00691	-0.0662	0.063806
0.465689	0.248607	0.991186	0.204901	0.465586	0.858715	0.93854	0.459646	0.476047
-0.09424	-0.26068	0.112859	0.116375	-0.224	-0.14845	-0.14249	0.06214	-0.08053
0.291929	0.003076	0.206474	0.192596	0.011352	0.095795	0.110032	0.487667	0.368117
0.132076	0.320136	0.113346	-0.11984	0.315267	0.224009	0.114473	-0.33359	0.120719
0.138822	0.000243	0.204511	0.179597	0.000306	0.01135	0.200015	0.000127	0.176398
0.514848	0.498972	-0.09713	0.112133	0.620009	0.42487	0.370371	-0.50917	0.310334
5.92E-10	2.36E-09	0.277341	0.209426	7.68E-15	6.4E-07	1.82E-05	9.79E-10	0.000384
0.580813	0.743145	-0.42993	-0.4935	0.709926	0.785617	0.857587	-0.63196	0.704692
8.18E-13	1.47E-23	4.55E-07	3.74E-09	9.25E-21	7.79E-28	6.67E-38	1.62E-15	2.35E-20
0.552671	0.510122	-0.09119	-0.25267	0.522712	0.528952	0.522898	-0.55331	0.576477
1.62E-11	9E-10	0.307925	0.004157	2.9E-10	1.63E-10	2.86E-10	1.52E-11	1.32E-12
0.041635	0.141581	0.359155	-0.45139	-0.00994	0.124842	0.199712	-0.00184	0.527368
0.642103	0.112339	3.37E-05	1E-07	0.91168	0.161973	0.024378	0.9836	1.89E-10
0.109814	0.349705	-0.01922	-0.21511	0.277986	0.23692	0.283509	-0.27755	0.382605
0.219063	5.58E-05	0.830209	0.015151	0.001554	0.007322	0.001238	0.001582	9.03E-06
-0.43964	-0.34537	0.28354	0.185889	-0.43831	-0.47165	-0.39503	0.49015	-0.32311
2.33E-07	6.99E-05	0.001237	0.036401	2.55E-07	2.18E-08	4.3E-06	4.94E-09	0.000211
-0.24784	0.044986	0.309834	-0.22494	-0.19019	-0.17245	-0.07304	0.171797	0.073795
0.004963	0.615523	0.000393	0.011006	0.032214	0.052534	0.414452	0.053446	0.40964
0.19675	0.366461	-0.30256	0.011465	0.273996	0.165046	0.05459	-0.10389	-0.29103
0.026619	2.26E-05	0.000546	0.898206	0.001826	0.063693	0.542143	0.2451	0.000902

TSEN34	TSEN54	TSR3	TSSC4	TSSK6	TSTA3	TTBK2	TTC36	TTPAL
black	black	black	black	black	black	black	black	black
-0.36795	-0.25499	-0.10662	-0.12582	-0.35417	-0.25792	0.172449	-0.35427	-0.09394
2.08E-05	0.003814	0.232835	0.158701	4.41E-05	0.003416	0.052533	4.38E-05	0.2935
-0.32248	-0.20801	-0.10299	-0.10025	-0.40196	-0.27253	0.149496	-0.38618	-0.08849
0.000218	0.018943	0.24924	0.262108	2.81E-06	0.001937	0.093442	7.32E-06	0.322496
0.421817	0.299914	-0.19918	-0.1126	0.266825	0.101547	-0.20762	0.183322	0.266886
7.85E-07	0.000613	0.024771	0.207505	0.002426	0.255957	0.01917	0.03911	0.00242
0.070703	-0.03402	-0.62458	-0.50095	0.059801	-0.26587	0.080026	-0.13876	0.496785
0.429589	0.704159	4.27E-15	2E-09	0.504227	0.002519	0.371126	0.119742	2.84E-09
0.277984	0.20997	0.176526	0.256402	-0.21912	0.468396	-0.36246	-0.08735	-0.3375
0.001554	0.017821	0.047111	0.003617	0.013319	2.8E-08	2.82E-05	0.328829	0.000104
0.01341	-0.0994	-0.515	-0.37527	-0.23438	-0.07439	-0.04425	-0.33291	0.242118
0.88105	0.266213	5.84E-10	1.38E-05	0.007995	0.405884	0.6213	0.000131	0.006098
-0.00574	-0.06868	-0.41041	-0.26621	-0.22763	-0.07107	0.056141	-0.29238	0.231695
0.948963	0.442953	1.65E-06	0.002485	0.01006	0.427209	0.530713	0.000851	0.008767
-0.21658	-0.14775	-0.15791	-0.15652	-0.2044	-0.19903	0.073395	-0.16386	0.15697
0.014459	0.097388	0.076218	0.078861	0.021164	0.024882	0.41219	0.065644	0.078
0.330006	-0.04768	-0.06711	-0.0165	0.3369	0.05444	-0.33061	-0.10461	0.22489
0.000151	0.59451	0.453445	0.853955	0.000108	0.543261	0.000147	0.241828	0.011023
0.019069	-0.07238	0.14736	0.152455	0.058725	0.040292	-0.27004	-0.3254	-0.06744
0.83149	0.418695	0.098273	0.08706	0.511938	0.652879	0.002138	0.000189	0.451229
-0.15301	-0.20908	-0.16194	-0.11289	-0.03183	-0.17366	-0.07698	-0.30853	0.14787
0.085905	0.018319	0.068913	0.206368	0.722439	0.050875	0.389666	0.000417	0.097102
0.133924	0.065919	0.161564	0.053081	0.342313	-0.00555	-0.21981	0.06826	0.051744
0.133332	0.46153	0.069574	0.553384	8.17E-05	0.950636	0.013026	0.445739	0.563436
0.381962	0.390874	0.322893	0.277378	0.569828	0.073725	-0.17111	0.401103	0.063996
9.37E-06	5.53E-06	0.000213	0.001593	2.71E-12	0.410084	0.054421	2.96E-06	0.47473
0.656177	0.592719	0.904971	0.90528	0.65088	0.637938	-0.57085	0.58321	-0.49349
5.58E-17	2.12E-13	3.19E-48	2.63E-48	1.2E-16	7.25E-16	2.43E-12	6.26E-13	3.75E-09
0.354527	0.346572	0.652999	0.596986	0.562443	0.244225	-0.42149	0.217735	-0.23812
4.32E-05	6.57E-05	8.83E-17	1.29E-13	5.93E-12	0.005656	8.02E-07	0.01393	0.007021
-0.4069	0.062914	0.294991	0.173073	-0.20728	-0.13389	0.165509	-0.04557	-0.41604
2.06E-06	0.482248	0.000761	0.051672	0.019373	0.13344	0.062943	0.610944	1.15E-06
0.03342	0.189294	0.217646	0.154429	0.051693	0.119122	0.116115	0.400053	-0.21163
0.709141	0.033052	0.01397	0.083001	0.563824	0.182229	0.193597	3.16E-06	0.016916
-0.49896	-0.41818	-0.53437	-0.53353	-0.458	-0.30629	0.565652	-0.07023	0.185924
2.37E-09	9.97E-07	9.77E-11	1.06E-10	6.16E-08	0.000462	4.23E-12	0.432668	0.036365
-0.4185	-0.30793	-0.05755	-0.14566	-0.42642	-0.093	0.433474	0.045764	-0.11723
9.76E-07	0.000429	0.52047	0.102264	5.77E-07	0.298339	3.57E-07	0.609416	0.189312
0.27985	0.335487	0.013656	-0.02277	0.024563	0.193429	-0.06504	0.184236	-0.06919
0.00144	0.000115	0.878887	0.799443	0.783995	0.029339	0.467573	0.038127	0.43954

TUBGCP2	TUBGCP5	TUFM	TWF2	TXNDC5	TXNRD2	TYSND1	TYW3	UBALD2
black	black	black	black	black	black	black	black	black
0.096298	0.084841	-0.34455	0.01898	-0.29315	-0.22842	0.037642	0.314568	-0.35543
0.28148	0.342945	7.29E-05	0.832263	0.000824	0.009796	0.674372	0.000316	4.12E-05
0.117247	0.133505	-0.28316	-0.03814	-0.35606	-0.22543	0.050943	0.315595	-0.31419
0.189257	0.134562	0.001256	0.670291	3.98E-05	0.010826	0.569498	0.000301	0.000322
-0.36396	0.186052	0.242781	-0.23991	0.142562	0.131315	-0.08925	-0.04599	0.389231
2.6E-05	0.036234	0.005955	0.006594	0.109846	0.14113	0.318374	0.607665	6.11E-06
-0.72513	0.419199	-0.2125	-0.54171	-0.07354	-0.36714	-0.33034	0.347758	0.019048
5.44E-22	9.33E-07	0.016461	4.81E-11	0.411261	2.18E-05	0.000149	6.17E-05	0.831674
-0.06431	-0.08272	0.110244	0.105886	-0.30589	-0.00789	0.155019	0.372493	0.17449
0.47258	0.355189	0.217254	0.236094	0.00047	0.929814	0.081817	1.61E-05	0.049757
-0.6806	0.341262	-0.3128	-0.43235	-0.31603	-0.488	-0.26066	0.652058	-0.10668
1.34E-18	8.62E-05	0.000343	3.86E-07	0.000295	5.9E-09	0.003079	1.01E-16	0.232597
-0.43509	0.22593	-0.13912	-0.35107	-0.42951	-0.44912	-0.19139	0.435021	-0.0572
3.19E-07	0.010648	0.118797	5.19E-05	4.68E-07	1.18E-07	0.031121	3.21E-07	0.52296
-0.33493	0.208852	-0.14992	-0.23617	-0.23813	-0.44748	-0.21756	0.320044	-0.33341
0.000119	0.018452	0.092513	0.007514	0.007018	1.33E-07	0.014008	0.000244	0.000128
-0.01114	0.03862	0.042438	0.221212	0.375575	-0.14844	-0.04788	-0.19293	0.237388
0.901106	0.666411	0.635685	0.012443	1.35E-05	0.0958	0.592953	0.029768	0.007203
0.13926	-0.22157	-0.04748	0.318565	0.062029	-0.30911	0.143941	0.01742	-0.03406
0.118411	0.012298	0.596049	0.000262	0.488443	0.000406	0.106417	0.845875	0.703802
-0.18666	0.006981	-0.23986	0.043245	-0.04871	-0.39299	-0.10089	0.186025	-0.21779
0.03562	0.937915	0.006606	0.629269	0.586534	4.87E-06	0.259081	0.036262	0.013904
0.386009	-0.03654	0.253652	0.356959	0.177561	0.264217	-0.00404	-0.5694	0.198603
7.39E-06	0.683416	0.004009	3.8E-05	0.045812	0.002685	0.964071	2.84E-12	0.025197
0.360729	-0.16277	0.479485	0.250448	0.454459	0.450556	0.244323	-0.64145	0.461369
3.1E-05	0.067489	1.18E-08	0.004513	8.01E-08	1.07E-07	0.005636	4.49E-16	4.79E-08
0.673253	-0.60118	0.729009	0.757125	0.527025	0.684757	0.603055	-0.66448	0.69819
4.28E-18	7.83E-14	2.56E-22	7.2E-25	1.95E-10	6.87E-19	6.26E-14	1.63E-17	7.29E-20
0.624701	-0.5058	0.476452	0.644565	0.503477	0.288699	0.462664	-0.6203	0.417758
4.2E-15	1.31E-09	1.49E-08	2.91E-16	1.61E-09	0.000996	4.34E-08	7.41E-15	1.03E-06
0.383632	-0.30775	0.032621	0.094585	-0.19907	0.041961	0.225813	-0.09254	-0.19008
8.5E-06	0.000432	0.715793	0.290167	0.024851	0.639493	0.01069	0.300746	0.032316
0.203714	-0.18208	0.332107	0.010893	0.052894	0.489931	0.026765	-0.43166	0.191705
0.021608	0.04048	0.000136	0.90326	0.554789	5.03E-09	0.765173	4.04E-07	0.030842
-0.44722	0.351814	-0.47049	-0.53382	-0.3338	-0.14473	-0.42542	0.392297	-0.46656
1.36E-07	4.99E-05	2.38E-08	1.03E-10	0.000126	0.104482	6.17E-07	5.08E-06	3.23E-08
0.126507	0.082668	-0.18837	-0.12965	-0.2484	0.146571	-0.17124	0.039145	-0.37466
0.156407	0.355493	0.033935	0.146298	0.004864	0.100107	0.054242	0.66215	1.43E-05
0.06673	0.063363	0.163508	-0.00569	-0.02027	0.358535	0.099995	-0.05704	0.398895
0.45603	0.479122	0.06624	0.949358	0.821051	3.49E-05	0.263332	0.524163	3.4E-06

UBE2I	UBE2J2	UBE2S	UBLCP1	UBR1	UBR2	UBTD1	UBXN1	UBXN2B
black	black	black	black	black	black	black	black	black
-0.24514	-0.36103	-0.43493	0.260203	0.0555	0.20009	-0.09136	0.055539	-0.23291
0.005473	3.05E-05	3.23E-07	0.003133	0.535428	0.024104	0.307015	0.535138	0.008409
-0.23084	-0.36031	-0.37301	0.256988	0.032274	0.145783	-0.09136	0.056408	-0.27494
0.009024	3.17E-05	1.57E-05	0.003538	0.718696	0.101966	0.306999	0.528758	0.001758
0.271663	0.155733	0.523788	0.02035	0.131932	-0.01044	-0.25351	-0.26721	0.453174
0.002005	0.080404	2.63E-10	0.820356	0.139255	0.907269	0.00403	0.00239	8.8E-08
-0.18595	-0.30217	-0.09321	0.319582	0.540317	0.456236	-0.51187	-0.62932	0.615813
0.03634	0.000555	0.297284	0.00025	5.51E-11	7.02E-08	7.71E-10	2.3E-15	1.31E-14
-0.00482	0.018281	0.049439	0.191483	-0.21154	-0.25067	0.151967	0.300789	-0.35996
0.957126	0.838359	0.58096	0.031039	0.016963	0.004476	0.088089	0.00059	3.23E-05
-0.34645	-0.42961	-0.30625	0.520803	0.427474	0.351691	-0.36009	-0.42215	0.296074
6.61E-05	4.65E-07	0.000462	3.46E-10	5.37E-07	5.02E-05	3.21E-05	7.68E-07	0.000726
-0.26396	-0.44962	-0.31513	0.207271	0.314706	0.295858	-0.3201	-0.27884	0.082631
0.002712	1.14E-07	0.000308	0.019378	0.000314	0.000733	0.000244	0.001501	0.355707
-0.30987	-0.26472	-0.49594	0.146823	0.046549	0.254352	-0.16752	-0.15562	-0.00833
0.000392	0.002634	3.05E-09	0.099519	0.603286	0.003906	0.059766	0.080629	0.925932
0.261627	0.139253	0.007362	-0.21594	0.067356	0.111575	0.01758	-0.16621	0.13117
0.002967	0.11843	0.934529	0.014757	0.451804	0.211718	0.844473	0.061812	0.141573
-0.09799	-0.10214	-0.47946	-0.0427	-0.10593	0.168836	0.280522	0.244095	-0.25798
0.27309	0.253154	1.18E-08	0.633641	0.235894	0.057759	0.001401	0.005682	0.003409
-0.27231	-0.27758	-0.5135	0.168838	0.189118	0.414995	-0.03513	-0.08271	0.005697
0.001954	0.00158	6.68E-10	0.057757	0.033218	1.23E-06	0.694982	0.355246	0.949313
0.514927	0.26509	0.144278	-0.43009	-0.04715	-0.07065	0.060784	0.064867	0.005359
5.88E-10	0.002596	0.10559	4.5E-07	0.59864	0.42995	0.497228	0.468731	0.952319
0.538422	0.490467	0.511685	-0.59656	-0.28131	-0.18092	0.173459	0.213017	0.017436
6.62E-11	4.82E-09	7.84E-10	1.35E-13	0.001356	0.041801	0.051145	0.016194	0.845736
0.579353	0.882579	0.643728	-0.68136	-0.76302	-0.63034	0.793339	0.784493	-0.55717
9.62E-13	8.65E-43	3.27E-16	1.19E-18	1.9E-25	2.01E-15	1.02E-28	1.04E-27	1.02E-11
0.42437	0.5249	0.140898	-0.62909	-0.54111	-0.20689	0.625178	0.604995	-0.43179
6.62E-07	2.37E-10	0.1141	2.37E-15	5.1E-11	0.019606	3.95E-15	4.95E-14	4.01E-07
-0.12373	-0.00994	-0.18223	0.025947	-0.28608	-0.06416	0.253319	0.412803	-0.34431
0.165766	0.911641	0.040315	0.772147	0.001112	0.473574	0.004059	1.41E-06	7.38E-05
0.262063	0.331236	0.597745	-0.19981	-0.24302	-0.4036	0.04578	0.178209	0.042181
0.002918	0.000142	1.18E-13	0.024305	0.005904	2.54E-06	0.609291	0.045013	0.63774
-0.41838	-0.39246	-0.07852	0.567744	0.484773	0.153131	-0.49891	-0.55493	0.466183
9.84E-07	5.03E-06	0.380232	3.39E-12	7.68E-09	0.085652	2.37E-09	1.29E-11	3.32E-08
-0.16243	-0.12358	0.05499	0.163532	0.037702	-0.20075	-0.16144	-0.07246	-0.01612
0.068066	0.16629	0.539183	0.066199	0.673881	0.02363	0.06979	0.418214	0.857241
0.52051	0.160417	0.532501	-0.11845	-0.06813	-0.26719	-0.18643	0.126627	0.121448
3.55E-10	0.071606	1.17E-10	0.184738	0.446621	0.002391	0.035847	0.156008	0.173781

UCN	UFSP1	UHMK1	UQCC3	URM1	USE1	USP14	USP25	USP33
black	black	black	black	black	black	black	black	black
-0.05024	-0.45253	0.066551	-0.25969	-0.12982	0.028761	0.076456	0.31529	0.032212
0.574843	9.22E-08	0.457236	0.003194	0.14575	0.748224	0.392912	0.000306	0.719211
-0.05455	-0.4503	0.042734	-0.21074	-0.0846	0.043075	0.096	0.239508	0.065456
0.542463	1.09E-07	0.633331	0.017399	0.344339	0.630622	0.282978	0.006687	0.464689
0.052097	0.406072	-0.21683	0.125467	0.262831	-0.1547	0.026188	-0.40596	0.099483
0.560771	2.17E-06	0.014343	0.159865	0.002833	0.082465	0.770088	2.19E-06	0.265795
-0.25318	0.254714	0.206466	-0.27691	-0.06246	-0.45991	0.342692	0.011608	0.392613
0.00408	0.003853	0.019864	0.001624	0.485391	5.34E-08	8.02E-05	0.896937	4.98E-06
-0.02957	0.233357	-0.28	0.357325	0.496543	0.311452	0.122994	-0.09147	0.071126
0.74137	0.008282	0.001431	3.72E-05	2.9E-09	0.000365	0.168326	0.306439	0.426825
-0.38169	0.213927	0.19975	-0.19136	0.088512	-0.30968	0.483734	0.19155	0.434228
9.52E-06	0.015734	0.024351	0.031148	0.322386	0.000396	8.35E-09	0.030979	3.39E-07
-0.22528	0.005222	0.048664	-0.26917	0.144452	-0.1921	0.374871	0.107175	0.234382
0.010883	0.953532	0.586916	0.002213	0.105168	0.030493	1.41E-05	0.230407	0.007995
-0.21033	-0.04339	0.378941	-0.26382	-0.27492	-0.27195	0.258153	0.277807	0.168043
0.017621	0.628104	1.12E-05	0.002726	0.00176	0.001982	0.003386	0.001566	0.058962
-0.00537	0.247225	-0.10879	-0.03441	0.061662	-0.17159	-0.07167	-0.08281	-0.32572
0.952225	0.005076	0.223413	0.700908	0.491021	0.053734	0.423278	0.354647	0.000186
0.034346	-0.02579	0.012307	-0.12876	0.0862	-0.05949	-0.08399	0.120682	-0.4114
0.701467	0.773459	0.890774	0.149094	0.335238	0.50645	0.34783	0.176529	1.55E-06
-0.06139	-0.02492	0.182542	-0.2905	-0.11862	-0.22485	0.028129	0.263887	-0.06642
0.492923	0.780942	0.039967	0.000923	0.184087	0.011037	0.753577	0.00272	0.458148
0.322618	-0.02233	-0.39953	0.291434	0.131212	0.168042	-0.35833	-0.26587	-0.2295
0.000216	0.803175	3.27E-06	0.000886	0.141446	0.058963	3.53E-05	0.002518	0.009446
0.49382	0.141862	-0.41607	0.226181	0.12079	0.160067	-0.53311	-0.53585	-0.5007
3.65E-09	0.111621	1.14E-06	0.010559	0.17614	0.072235	1.1E-10	8.48E-11	2.04E-09
0.678441	0.437766	-0.5856	0.787197	0.436603	0.703275	-0.75571	-0.65737	-0.67898
1.89E-18	2.65E-07	4.78E-13	5.17E-28	2.87E-07	3.02E-20	9.86E-25	4.68E-17	1.74E-18
0.542621	0.117064	-0.39586	0.282394	0.200241	0.298162	-0.67214	-0.41225	-0.8361
4.4E-11	0.189955	4.09E-06	0.001297	0.023995	0.000662	5.09E-18	1.47E-06	2.15E-34
0.109693	-0.47279	0.009771	-0.06828	-0.01215	0.187692	-0.12689	0.012847	-0.10736
0.219572	1.99E-08	0.913178	0.445598	0.892153	0.034593	0.155142	0.886008	0.229611
0.204788	-0.09943	-0.23488	0.224995	0.109949	0.236565	-0.22716	-0.33981	0.036553
0.020913	0.266057	0.007859	0.010984	0.218494	0.007413	0.010218	9.29E-05	0.683274
-0.47554	-0.20247	0.553238	-0.27665	-0.38549	-0.2678	0.548063	0.459844	0.821392
1.61E-08	0.022438	1.53E-11	0.001641	7.62E-06	0.002335	2.57E-11	5.37E-08	2.85E-32
-0.20394	-0.39189	0.230132	-0.08305	-0.27148	0.075767	0.223182	0.324955	0.386894
0.021461	5.21E-06	0.009245	0.353284	0.002019	0.397205	0.011664	0.000193	7.02E-06
0.199411	-0.02586	-0.48273	0.22843	0.380735	0.062098	-0.0325	-0.36108	-0.03074
0.024598	0.772885	9.05E-09	0.009792	1.01E-05	0.487962	0.716813	3.04E-05	0.731554

USP46	USP47	USP9X	VAPA	VDAC1	VEGFB	VEZT	VPS13A	VPS28
black	black	black	black	black	black	black	black	black
0.258693	-0.01781	-0.09611	0.008555	0.163205	0.118492	-0.01169	-0.07814	-0.01445
0.003318	0.842482	0.282442	0.923954	0.06675	0.184571	0.896223	0.382564	0.871917
0.281627	-0.00668	-0.13788	0.089375	0.239828	0.12436	0.043983	-0.11727	0.033646
0.001339	0.94057	0.122139	0.317676	0.006612	0.163611	0.623428	0.189187	0.707266
-0.39671	0.161473	0.324785	0.096296	-0.22248	-0.14355	0.123965	0.011406	-0.20111
3.88E-06	0.069734	0.000195	0.28149	0.011937	0.107369	0.164967	0.898724	0.02338
-0.21469	0.460519	0.50138	0.436247	0.030446	-0.33194	0.347213	0.372543	-0.61011
0.015357	5.1E-08	1.92E-09	2.95E-07	0.734009	0.000138	6.35E-05	1.61E-05	2.65E-14
-0.12815	-0.26505	-0.59468	0.338364	0.075939	0.376007	0.128098	-0.29338	0.420917
0.151062	0.0026	1.69E-13	9.99E-05	0.396128	1.32E-05	0.15122	0.000815	8.33E-07
-0.15525	0.291089	0.067103	0.678465	0.232764	-0.1122	0.462534	0.239876	-0.35853
0.081363	0.0009	0.453508	1.89E-18	0.008452	0.209145	4.38E-08	0.006601	3.49E-05
-0.02718	0.213608	0.012166	0.445288	0.310718	-0.05611	0.286703	0.145292	-0.2373
0.761621	0.015894	0.89202	1.56E-07	0.000377	0.530921	0.001083	0.103139	0.007226
0.259293	0.271537	0.187071	0.170631	0.088839	-0.05246	0.061495	0.293122	-0.17862
0.003243	0.002015	0.035208	0.055111	0.3206	0.558068	0.492199	0.000825	0.044516
-0.52469	0.038319	0.340911	-0.24313	-0.00511	0.02571	-0.10858	0.016381	-0.18715
2.42E-10	0.668856	8.78E-05	0.005882	0.954525	0.774176	0.224335	0.854959	0.035129
-0.21334	0.086099	-0.05264	-0.17397	-0.04997	0.329093	-0.19859	-0.10069	0.114353
0.016031	0.335806	0.556688	0.050452	0.576909	0.000158	0.025204	0.259988	0.20049
-0.10601	0.223445	0.121999	-0.00846	0.024233	0.093287	-0.06511	0.198738	-0.21287
0.235533	0.011563	0.171821	0.924785	0.786832	0.296867	0.46709	0.025097	0.016268
-0.02391	-0.32326	0.104407	-0.376	-0.03463	0.028257	-0.40067	-0.23729	0.023066
0.789609	0.00021	0.242739	1.32E-05	0.69915	0.75249	3.04E-06	0.007227	0.796864
-0.22298	-0.01638	0.280672	-0.65515	-0.46501	0.114691	-0.47239	-0.25143	0.132657
0.011743	0.854985	0.001392	6.47E-17	3.63E-08	0.199156	2.06E-08	0.004353	0.137077
-0.39434	-0.49506	-0.46947	-0.6911	-0.57954	0.66362	-0.58249	-0.61096	0.799999
4.49E-06	3.29E-09	2.58E-08	2.42E-19	9.42E-13	1.86E-17	6.79E-13	2.39E-14	1.65E-29
-0.29405	-0.11347	-0.0539	-0.77667	-0.47041	0.532987	-0.64004	-0.44381	0.453354
0.000792	0.203995	0.547291	7.41E-27	2.4E-08	1.11E-10	5.44E-16	1.73E-07	8.68E-08
0.492061	0.019237	-0.29868	-0.11056	-0.01174	0.176746	-0.19639	-0.25652	0.31038
4.22E-09	0.830025	0.000648	0.215908	0.895794	0.046833	0.026903	0.003601	0.000383
0.220913	-0.21525	-0.11391	-0.19295	-0.19788	-0.09241	-0.13131	-0.17048	0.235727
0.012565	0.015084	0.20224	0.029746	0.025742	0.301464	0.141159	0.055331	0.007631
0.381388	0.05005	0.090207	0.579844	0.372668	-0.61323	0.599759	0.505927	-0.42932
9.69E-06	0.57629	0.31317	9.11E-13	1.6E-05	1.8E-14	9.27E-14	1.3E-09	4.74E-07
0.478846	-0.26052	-0.1811	0.137193	0.309481	-0.3373	0.203133	0.17734	0.031022
1.24E-08	0.003095	0.041586	0.124029	0.000399	0.000105	0.021993	0.046086	0.729169
-0.09692	-0.17261	-0.00386	-0.08838	-0.24701	-0.02233	0.007964	-0.38803	0.102596
0.278361	0.05231	0.965634	0.323119	0.005115	0.803231	0.929191	6.56E-06	0.25105

VPS35	VPS37C	VPS41	VPS45	VPS51	VPS72	VPS8	VWCE	WDR13
black	black	black	black	black	black	black	black	black
0.130018	0.082948	0.065118	-0.10399	-0.16566	-0.25419	0.123619	-0.0868	-0.08637
0.145136	0.353855	0.467004	0.244631	0.062694	0.00393	0.166155	0.331895	0.334266
0.104649	0.044338	0.092187	0.013117	-0.13368	-0.1671	0.162126	-0.05064	-0.06123
0.241642	0.620622	0.302626	0.883628	0.13406	0.060411	0.068595	0.571783	0.494057
0.035458	-0.26674	0.064625	0.421774	-0.15654	0.267886	0.175734	-0.0359	-0.17354
0.692276	0.002434	0.470391	7.87E-07	0.078834	0.002327	0.048127	0.688676	0.051032
0.314584	-0.37542	0.388172	0.455422	-0.65875	-0.1314	0.354176	-0.31514	-0.59293
0.000316	1.37E-05	6.5E-06	7.46E-08	3.83E-17	0.140871	4.41E-05	0.000308	2.07E-13
0.055702	-0.06593	-0.01702	0.351959	-0.10045	0.503038	-0.24109	-0.1866	0.127997
0.53394	0.461457	0.849412	4.95E-05	0.261165	1.67E-09	0.006323	0.035678	0.151546
0.410115	-0.31627	0.423073	0.514567	-0.75426	-0.01366	0.133321	-0.44369	-0.54778
1.68E-06	0.000292	7.22E-07	6.07E-10	1.36E-24	0.878836	0.135103	1.75E-07	2.64E-11
0.200935	-0.24302	0.315878	0.375401	-0.59377	-0.0471	0.254763	-0.24325	-0.3389
0.023501	0.005905	0.000297	1.37E-05	1.87E-13	0.599006	0.003846	0.005857	9.72E-05
0.408386	-0.23941	0.171804	0.172896	-0.39118	-0.14021	0.008199	0.023145	-0.09923
1.88E-06	0.00671	0.053437	0.051914	5.43E-06	0.11589	0.927107	0.796183	0.267004
-0.17903	0.284426	-0.18681	-0.23625	-0.18587	-0.30549	-0.09419	-0.10167	-0.2105
0.044015	0.001192	0.035463	0.007494	0.036415	0.000478	0.292182	0.255393	0.017526
-0.16029	0.386885	-0.20568	-0.39375	-0.21594	-0.42532	-0.20147	0.04837	-0.07019
0.071832	7.02E-06	0.020348	4.65E-06	0.014755	6.21E-07	0.023123	0.589178	0.432969
0.076357	0.150581	0.126113	-0.14333	-0.38483	-0.42588	0.009896	-0.06316	-0.31011
0.39353	0.091061	0.157708	0.107916	7.92E-06	5.98E-07	0.912079	0.480508	0.000388
-0.36634	0.334187	-0.22691	-0.19084	0.386755	0.086939	0.075224	0.020761	0.071343
2.28E-05	0.000123	0.010306	0.031621	7.07E-06	0.331094	0.400604	0.81679	0.425412
-0.46776	0.216886	-0.44416	-0.25468	0.484404	0.068652	0.117434	0.425327	0.353385
2.94E-08	0.014316	1.69E-07	0.003858	7.91E-09	0.443124	0.18855	6.21E-07	4.59E-05
-0.68594	0.495008	-0.72699	-0.43487	0.873309	0.497244	-0.46428	0.488831	0.840373
5.67E-19	3.3E-09	3.8E-22	3.24E-07	7.42E-41	2.73E-09	3.84E-08	5.51E-09	4.76E-35
-0.64992	0.598944	-0.66031	-0.65355	0.520557	-0.13656	-0.23031	0.491593	0.505809
1.37E-16	1.02E-13	3.04E-17	8.15E-17	3.54E-10	0.125785	0.009191	4.39E-09	1.31E-09
-0.1806	0.11569	-0.09351	-0.23211	0.334265	-0.06479	0.011906	0.245618	0.332019
0.042172	0.195245	0.295705	0.008645	0.000123	0.469289	0.894308	0.00538	0.000137
-0.15398	-0.1632	-0.08764	0.105269	0.575626	0.428519	0.049023	0.261991	0.360399
0.083914	0.066757	0.327204	0.238848	1.45E-12	5.01E-07	0.584156	0.002926	3.15E-05
0.555849	-0.53235	0.612296	0.375456	-0.24632	0.029032	0.146918	-0.36514	-0.36666
1.17E-11	1.18E-10	2.03E-14	1.36E-05	0.005245	0.745933	0.099298	2.43E-05	2.24E-05
0.259948	-0.23839	0.228217	0.08817	0.239926	0.147244	-0.02686	-0.07501	0.089185
0.003163	0.006955	0.009863	0.324268	0.00659	0.098542	0.764362	0.401959	0.318708
-0.14133	-0.11907	-0.17123	0.217626	0.168265	0.389067	0.252119	0.002183	0.102443
0.112988	0.182414	0.054255	0.013979	0.058623	6.17E-06	0.004243	0.980568	0.251764

WDR18	WDR24	WDR31	WDR34	WDR44	WDR83	WDR86	WFIKKN1	WNT6
black	black	black	black	black	black	black	black	black
-0.27406	-0.28697	-0.04242	-0.37169	0.288726	-0.11559	0.034217	-0.20811	-0.16862
0.001821	0.001071	0.635835	1.69E-05	0.000995	0.195646	0.702532	0.018881	0.058083
-0.30738	-0.29856	-0.02629	-0.38182	0.177982	-0.08212	0.014791	-0.22038	-0.14581
0.000439	0.000651	0.769191	9.45E-06	0.045292	0.358686	0.868903	0.012787	0.101895
0.135166	0.098817	0.265394	0.445818	-0.2013	-0.17052	-0.09795	0.015543	0.046896
0.129735	0.269026	0.002565	1.5E-07	0.023248	0.05527	0.273248	0.862305	0.600587
-0.39308	-0.26916	0.458535	0.05122	0.345431	-0.55195	-0.40973	-0.4291	-0.29099
4.84E-06	0.002214	5.92E-08	0.567396	6.97E-05	1.74E-11	1.72E-06	4.81E-07	0.000903
-0.18411	-0.18483	0.080533	0.16845	-0.04329	-0.13892	0.055188	-0.19519	0.000479
0.038264	0.037502	0.368095	0.058342	0.628941	0.119308	0.537724	0.027871	0.995738
-0.62695	-0.49139	0.429614	-0.04428	0.498163	-0.68774	-0.38181	-0.62956	-0.3657
3.14E-15	4.46E-09	4.65E-07	0.621114	2.53E-09	4.22E-19	9.46E-06	2.23E-15	2.36E-05
-0.51242	-0.41871	0.247992	-0.18636	0.227463	-0.42269	-0.30606	-0.50588	-0.20896
7.35E-10	9.63E-07	0.004936	0.035918	0.010116	7.4E-07	0.000466	1.3E-09	0.01839
-0.33258	-0.37181	0.43068	0.161715	0.428526	-0.40334	-0.25342	-0.25537	-0.33033
0.000133	1.68E-05	4.32E-07	0.06931	5E-07	2.58E-06	0.004043	0.00376	0.000149
0.078823	0.164068	-0.1119	0.135496	-0.04197	-0.09657	0.008734	-0.21641	0.096738
0.378383	0.065302	0.210399	0.128794	0.639417	0.280141	0.922363	0.014534	0.279276
-0.11591	-0.06577	-0.24493	-0.02102	0.284425	-0.16573	-0.00561	-0.30528	-0.06866
0.194389	0.462563	0.005515	0.814517	0.001192	0.062579	0.950089	0.000483	0.44304
-0.22015	-0.21001	-0.11685	-0.09216	0.445554	-0.31972	-0.23193	-0.3848	-0.23557
0.012882	0.017796	0.19077	0.302777	1.53E-07	0.000248	0.008697	7.94E-06	0.007674
0.325656	0.376081	-0.42825	-0.09025	-0.54	0.659449	-0.08153	0.156049	0.261251
0.000187	1.32E-05	5.1E-07	0.312915	5.69E-11	3.45E-17	0.362189	0.079783	0.00301
0.652574	0.585494	-0.21722	0.368398	-0.57308	0.5117	0.252679	0.520289	0.35993
9.39E-17	4.84E-13	0.014162	2.03E-05	1.91E-12	7.83E-10	0.004156	3.62E-10	3.23E-05
0.902341	0.828621	-0.46789	0.617682	-0.6903	0.745636	0.516807	0.740496	0.570602
1.62E-47	2.74E-33	2.91E-08	1.03E-14	2.77E-19	8.72E-24	4.97E-10	2.55E-23	2.5E-12
0.620719	0.5946	-0.51418	0.335312	-0.37816	0.498696	0.350698	0.419046	0.378775
7.02E-15	1.7E-13	6.28E-10	0.000116	1.17E-05	2.42E-09	5.29E-05	9.42E-07	1.13E-05
0.044721	-0.044	-0.32743	-0.29959	-0.02583	0.27732	0.110852	0.245523	0.024257
0.617609	0.623314	0.000172	0.000622	0.773138	0.001597	0.214711	0.005398	0.786625
0.448198	0.238717	-0.14712	0.118682	-0.53106	0.461048	0.169236	0.546441	0.270294
1.26E-07	0.006876	0.098827	0.183864	1.34E-10	4.9E-08	0.057161	3.02E-11	0.002117
-0.40789	-0.40241	0.370174	-0.40009	0.342225	-0.28924	-0.23668	-0.14332	-0.26253
1.94E-06	2.73E-06	1.84E-05	3.16E-06	8.21E-05	0.000973	0.007383	0.107959	0.002865
-0.0521	-0.1743	0.016413	-0.33809	-0.05456	0.164877	0.048143	0.173569	-0.04173
0.560734	0.050006	0.85468	0.000101	0.542378	0.063969	0.590931	0.050995	0.641362
0.144743	0.12887	0.098989	0.061847	-0.457	0.131479	0.29994	0.174014	0.369092
0.10446	0.148749	0.26819	0.489726	6.63E-08	0.140631	0.000613	0.050394	1.95E-05

XAB2	YIF1A	YIF1B	YIPF2	YIPF6	YTHDC2	ZACN	ZBTB11	ZBTB17
black	black	black	black	black	black	black	black	black
-0.12514	-0.22219	-0.09948	-0.12386	-0.05751	0.057007	-0.17213	-0.02888	-0.21388
0.160953	0.01205	0.265825	0.165343	0.520753	0.524387	0.052981	0.747198	0.015756
-0.11373	-0.24502	-0.087	-0.16248	-0.04523	-0.0261	-0.26145	-0.03604	-0.21934
0.202974	0.005496	0.330754	0.067984	0.613642	0.770817	0.002987	0.687513	0.013224
-0.00795	0.068259	-0.1755	-0.09405	0.149163	0.180354	0.079407	0.192029	0.144005
0.929339	0.445745	0.048427	0.292908	0.094182	0.042453	0.374853	0.030554	0.106258
-0.47732	-0.22282	-0.59887	-0.26188	0.581901	0.472309	-0.22238	0.495411	-0.36608
1.4E-08	0.011803	1.03E-13	0.002938	7.24E-13	2.07E-08	0.011975	3.19E-09	2.31E-05
-0.18189	0.287175	0.123914	0.152229	-0.02219	-0.39387	-0.18189	-0.15327	-0.03474
0.040694	0.001062	0.16514	0.087536	0.804434	4.62E-06	0.04069	0.085363	0.698181
-0.69386	-0.11169	-0.51096	-0.17672	0.587646	0.210876	-0.38173	0.389125	-0.52384
1.52E-19	0.211233	8.36E-10	0.046862	3.79E-13	0.017323	9.5E-06	6.14E-06	2.62E-10
-0.46808	-0.28564	-0.44426	-0.13569	0.408148	0.191385	-0.48142	0.223193	-0.39756
2.87E-08	0.001133	1.68E-07	0.128228	1.91E-06	0.031128	1.01E-08	0.011659	3.69E-06
-0.43205	-0.08497	-0.21804	-0.19278	0.476281	0.249474	-0.21489	0.133622	-0.36125
3.94E-07	0.342198	0.013792	0.029901	1.52E-08	0.004677	0.015261	0.134218	3.01E-05
0.041169	0.229809	-0.08348	0.297818	0.023995	0.147768	0.108377	-0.10048	0.018607
0.64583	0.009347	0.350757	0.000672	0.78887	0.097336	0.225189	0.26099	0.835515
-0.09836	0.292195	0.098055	0.200305	0.007918	-0.02442	-0.03478	-0.34636	-0.0518
0.271235	0.000858	0.272753	0.023949	0.929599	0.785196	0.697907	6.64E-05	0.562996
-0.27139	0.055619	-0.18792	0.043082	0.252308	0.169107	-0.09945	-0.08323	-0.30219
0.002027	0.534548	0.034367	0.630562	0.004214	0.057353	0.265938	0.352232	0.000555
0.508729	0.070449	0.105894	0.240049	-0.25774	-0.22074	0.182535	-0.32694	0.177797
1.02E-09	0.431252	0.236059	0.006561	0.00344	0.012637	0.039974	0.000176	0.045519
0.684948	0.218813	0.290201	0.154591	-0.4059	0.007243	0.395479	-0.31123	0.628186
6.66E-19	0.013453	0.000934	0.082674	2.2E-06	0.935584	4.19E-06	0.000369	2.67E-15
0.85557	0.801149	0.890239	0.647568	-0.7394	-0.58859	0.560127	-0.66541	0.839331
1.5E-37	1.19E-29	1.63E-44	1.91E-16	3.2E-23	3.4E-13	7.54E-12	1.42E-17	6.91E-35
0.667013	0.571899	0.586082	0.429693	-0.51342	-0.25081	0.431493	-0.68943	0.631191
1.11E-17	2.17E-12	4.53E-13	4.62E-07	6.72E-10	0.004453	4.09E-07	3.19E-19	1.8E-15
0.223724	-0.11175	0.296013	-0.18082	-0.21818	-0.20007	0.038805	-0.28146	0.150864
0.011457	0.211016	0.000728	0.041915	0.013731	0.02412	0.664906	0.001348	0.090448
0.454043	-0.08715	0.259445	-0.10871	-0.29412	-0.19991	0.204259	-0.07264	0.362665
8.25E-08	0.329894	0.003224	0.223776	0.00079	0.024237	0.021253	0.417034	2.79E-05
-0.48597	-0.58951	-0.41001	-0.38848	0.360759	0.272125	-0.22965	0.708152	-0.52145
6.97E-09	3.06E-13	1.69E-06	6.39E-06	3.09E-05	0.001968	0.009398	1.27E-20	3.26E-10
-0.0336	-0.39636	0.008691	-0.19419	-0.10127	-0.0694	-0.07831	0.226403	-0.15296
0.70762	3.97E-06	0.922747	0.028696	0.257279	0.438177	0.381517	0.010481	0.085997
0.2818	-0.10507	-0.06178	-0.14945	-0.26249	0.049252	0.092071	0.090744	0.409424
0.001329	0.239725	0.490182	0.093554	0.00287	0.582399	0.303237	0.310287	1.76E-06

ZBTB22	ZBTB24	ZBTB43	ZBTB45	ZBTB48	ZC3H3	ZDHHC24	ZDHHC8	ZFPM1
black	black	black	black	black	black	black	black	black
-0.03083	-0.08984	0.040549	-0.28928	-0.26231	-0.28065	-0.14017	-0.16791	-0.31202
0.730818	0.315179	0.650817	0.000972	0.002889	0.001394	0.115996	0.059173	0.000355
-0.06827	-0.1113	0.026556	-0.28745	-0.30924	-0.33261	-0.16223	-0.21138	-0.32465
0.445661	0.212866	0.766953	0.00105	0.000404	0.000133	0.068422	0.017052	0.000196
-0.10899	0.303441	0.128338	0.068811	0.255774	0.047471	-0.17613	-0.00312	0.04191
0.222568	0.000524	0.150448	0.44207	0.003704	0.596122	0.047613	0.972205	0.639898
-0.3995	0.533736	0.309208	-0.41648	-0.03419	-0.37613	-0.43915	-0.47513	-0.36466
3.27E-06	1.04E-10	0.000404	1.11E-06	0.702717	1.31E-05	2.41E-07	1.66E-08	2.5E-05
0.077307	-0.21247	-0.36546	-0.26999	0.170051	-0.51089	0.155987	-0.44187	-0.08162
0.387649	0.016475	2.39E-05	0.002143	0.055957	8.42E-10	0.079905	1.99E-07	0.361621
-0.35888	0.334436	0.063945	-0.68658	-0.11007	-0.77258	-0.3005	-0.82373	-0.50987
3.42E-05	0.000122	0.475088	5.11E-19	0.217994	2E-26	0.000598	1.35E-32	9.21E-10
-0.24768	0.258809	0.236272	-0.5448	-0.15218	-0.56364	-0.31193	-0.58609	-0.46419
0.004993	0.003303	0.007489	3.55E-11	0.087641	5.23E-12	0.000357	4.52E-13	3.87E-08
-0.09201	-0.08957	-0.09808	-0.41256	-0.0153	-0.44378	-0.11529	-0.40539	-0.25756
0.303576	0.316613	0.272642	1.44E-06	0.864399	1.73E-07	0.196819	2.27E-06	0.003464
-0.16892	0.071743	0.109038	0.010833	0.181942	0.16099	0.062665	0.015135	0.095842
0.057635	0.422815	0.222356	0.903785	0.040635	0.070584	0.483988	0.865889	0.283778
-0.10541	-0.15049	-0.12682	-0.26798	0.123274	-0.12177	0.301117	-0.19068	-0.0484
0.238199	0.091259	0.155374	0.002319	0.167352	0.172634	0.000582	0.031763	0.588948
-0.17224	-0.00211	-0.03619	-0.3828	-0.04897	-0.23076	-0.00284	-0.32223	-0.24815
0.052826	0.981248	0.686244	8.93E-06	0.584582	0.009049	0.9747	0.00022	0.004909
-0.14284	-0.1058	0.075243	0.327872	0.064662	0.549447	-0.14117	0.388662	0.269165
0.109138	0.236464	0.400483	0.000168	0.470139	2.24E-11	0.113402	6.32E-06	0.002213
0.167134	-0.0425	0.11005	0.638225	0.409833	0.779085	0.156407	0.759398	0.534019
0.060366	0.635197	0.218067	6.98E-16	1.71E-06	4.08E-27	0.079087	4.33E-25	1.01E-10
0.580429	-0.51801	-0.44453	0.846398	0.666179	0.734901	0.776323	0.7893	0.889724
8.53E-13	4.45E-10	1.64E-07	5.23E-36	1.26E-17	7.97E-23	8.07E-27	2.99E-28	2.15E-44
0.24966	-0.35896	-0.18167	0.518989	0.482032	0.671974	0.592308	0.64725	0.617114
0.004645	3.41E-05	0.040945	4.08E-10	9.58E-09	5.22E-18	2.22E-13	2E-16	1.11E-14
0.107551	-0.25683	-0.1743	0.096975	-0.21893	0.086903	0.148745	0.228523	0.037875
0.228766	0.00356	0.050008	0.278097	0.013402	0.331299	0.095118	0.009762	0.672468
0.257281	-0.12093	-0.00507	0.532196	0.01459	0.47089	-0.00357	0.551421	0.285571
0.0035	0.17563	0.954926	1.2E-10	0.870671	2.31E-08	0.968198	1.84E-11	0.001136
-0.1145	0.318878	0.186593	-0.24104	-0.54498	-0.35715	-0.47107	-0.32701	-0.45446
0.199927	0.000258	0.035686	0.006334	3.49E-11	3.76E-05	2.28E-08	0.000175	8.01E-08
0.141304	-0.10251	0.009318	0.093338	-0.40494	-0.00198	-0.18538	0.090666	-0.14092
0.113049	0.251431	0.917189	0.2966	2.33E-06	0.98239	0.036926	0.310707	0.114052
-0.10122	0.280769	0.219132	0.180967	0.216249	0.204709	-0.18906	0.247334	0.109339
0.257481	0.001387	0.013315	0.041744	0.014611	0.020963	0.033278	0.005056	0.221073

ZMYM2	ZNF10	ZNF189	ZNF205	ZNF213	ZNF219	ZNF280D	ZNF296	ZNF329
black	black	black	black	black	black	black	black	black
-0.09465	-0.02956	0.334131	-0.29237	-0.18579	-0.35076	0.119605	-0.28515	0.020981
0.289839	0.741433	0.000123	0.000852	0.036503	5.28E-05	0.180451	0.001156	0.81488
-0.03565	0.020109	0.416969	-0.31734	-0.20502	-0.34285	0.136386	-0.27647	0.100096
0.690728	0.822448	1.08E-06	0.000278	0.020763	7.95E-05	0.126277	0.001653	0.262847
0.296262	0.249185	-0.2076	0.169414	0.029345	0.168471	-0.00714	0.109633	0.152909
0.00072	0.004726	0.019182	0.056896	0.74329	0.05831	0.936459	0.219829	0.086114
0.417069	0.516522	0.012619	-0.30285	-0.45425	-0.36235	0.288773	-0.23628	0.336886
1.07E-06	5.1E-10	0.888018	0.000538	8.13E-08	2.84E-05	0.000993	0.007487	0.000108
0.036924	0.099806	0.237372	-0.17624	-0.38223	-0.19718	0.087024	-0.24466	0.263395
0.680237	0.264242	0.007207	0.047471	9.23E-06	0.026283	0.330624	0.005568	0.002772
0.32757	0.495029	0.265018	-0.55687	-0.76723	-0.63195	0.372215	-0.50788	0.475672
0.00017	3.29E-09	0.002603	1.06E-11	7.14E-26	1.62E-15	1.64E-05	1.1E-09	1.59E-08
0.30149	0.420788	0.263161	-0.43607	-0.54932	-0.56146	0.213609	-0.32945	0.386923
0.000572	8.4E-07	0.002797	2.98E-07	2.27E-11	6.57E-12	0.015894	0.000155	7E-06
-0.08289	0.285442	0.125182	-0.36264	-0.33481	-0.40371	0.008413	-0.17702	0.110165
0.354198	0.001142	0.160826	2.79E-05	0.000119	2.52E-06	0.925212	0.04649	0.217585
-0.15704	-0.17661	-0.25156	0.157354	-0.05402	-0.01154	-0.23213	0.053633	-0.07865
0.077867	0.047004	0.004332	0.077264	0.546385	0.89756	0.008637	0.549263	0.379455
-0.32894	-0.2333	0.103882	-0.0838	-0.24446	-0.24883	-0.12336	-0.10046	-0.17553
0.000159	0.008298	0.245128	0.348912	0.005608	0.004789	0.167065	0.261108	0.048388
-0.20809	-0.01396	0.045545	-0.22677	-0.31841	-0.38823	-0.00206	-0.22481	-0.09037
0.018894	0.876198	0.611136	0.010354	0.000264	6.48E-06	0.981674	0.011053	0.312271
-0.10304	-0.24733	-0.20408	0.403211	0.343231	0.317399	-0.28061	0.209403	-0.10885
0.248986	0.005058	0.021369	2.6E-06	7.8E-05	0.000277	0.001396	0.018139	0.223146
-0.14454	-0.27914	-0.37567	0.695234	0.704436	0.67364	-0.3655	0.634949	-0.37871
0.104943	0.001483	1.35E-05	1.21E-19	2.46E-20	4.03E-18	2.39E-05	1.09E-15	1.13E-05
-0.54478	-0.6285	-0.43185	0.895861	0.780723	0.886151	-0.48676	0.73039	-0.55835
3.56E-11	2.56E-15	3.99E-07	7.3E-46	2.71E-27	1.41E-43	6.53E-09	1.95E-22	9.06E-12
-0.55169	-0.60672	-0.26314	0.661351	0.575518	0.555424	-0.46331	0.57736	-0.59813
1.79E-11	4.02E-14	0.002799	2.61E-17	1.47E-12	1.22E-11	4.13E-08	1.2E-12	1.12E-13
-0.07425	-0.19217	0.398021	-0.03113	0.189444	0.074704	0.056666	0.067417	-0.28896
0.406731	0.030427	3.59E-06	0.728247	0.032911	0.40388	0.52687	0.451393	0.000985
0.130708	-0.09455	-0.21339	0.330905	0.534472	0.537207	-0.16137	0.318271	-0.1623
0.142993	0.290349	0.016002	0.000145	9.67E-11	7.44E-11	0.069909	0.000266	0.068293
0.456232	0.418685	0.119016	-0.49421	-0.28164	-0.31614	0.43146	-0.4091	0.358284
7.02E-08	9.65E-07	0.182621	3.53E-09	0.001338	0.000294	4.1E-07	1.79E-06	3.54E-05
0.141434	0.042908	0.051999	-0.19376	0.109642	0.002973	0.091118	-0.1557	0.113421
0.112716	0.631951	0.561512	0.029059	0.219788	0.973539	0.308289	0.08046	0.204207
0.468344	0.165502	0.115299	0.247732	0.186659	0.28362	0.100692	0.169185	0.188563
2.81E-08	0.062953	0.196768	0.004983	0.035619	0.001232	0.26	0.057238	0.033748

ZNF358	ZNF408	ZNF414	ZNF428	ZNF444	ZNF449	ZNF451	ZNF467	ZNF471
black	black	black	black	black	black	black	black	black
-0.10108	-0.18851	-0.25707	-0.35976	-0.24245	0.055225	0.059532	-0.10207	0.030298
0.25814	0.033803	0.003528	3.27E-05	0.006026	0.537448	0.506151	0.253511	0.735257
-0.08139	-0.20668	-0.2817	-0.30095	-0.25871	0.072893	0.038133	-0.20124	0.039538
0.363021	0.019733	0.001335	0.000586	0.003316	0.415401	0.670371	0.023285	0.658971
-0.16562	0.252183	0.062664	0.155552	-0.0639	0.333727	0.033773	-0.22746	0.172731
0.062764	0.004233	0.483996	0.080759	0.475364	0.000126	0.706215	0.010116	0.052143
-0.67135	-0.06261	-0.36456	-0.42531	-0.51523	0.639262	0.432616	-0.51305	0.368213
5.75E-18	0.484348	2.51E-05	6.22E-07	5.72E-10	6.06E-16	3.79E-07	6.95E-10	2.05E-05
-0.24265	-0.0774	-0.2694	-0.16979	-0.19918	0.139303	-0.00097	-0.33794	-0.34163
0.005984	0.387072	0.002193	0.056338	0.024766	0.118295	0.991403	0.000102	8.46E-05
-0.84572	-0.24567	-0.64021	-0.68824	-0.71181	0.652895	0.443405	-0.67537	0.06987
6.73E-36	0.005369	5.32E-16	3.89E-19	6.58E-21	8.96E-17	1.78E-07	3.07E-18	0.43506
-0.6393	-0.23669	-0.50616	-0.47921	-0.53198	0.461298	0.289287	-0.53635	0.204937
6.02E-16	0.00738	1.27E-09	1.2E-08	1.23E-10	4.81E-08	0.000971	8.08E-11	0.020818
-0.44615	-0.32811	-0.49182	-0.48948	-0.3119	0.149064	0.312205	-0.25342	0.160209
1.46E-07	0.000166	4.3E-09	5.23E-09	0.000357	0.094402	0.000352	0.004043	0.071979
-0.15546	0.266049	0.076945	-0.10424	-0.11389	0.107716	-0.16379	0.132535	-0.00584
0.080934	0.002501	0.389885	0.243486	0.202342	0.228046	0.065768	0.137441	0.948052
-0.23533	0.066544	-0.14661	-0.41564	-0.16312	0.048506	0.030507	0.189678	-0.20997
0.007737	0.457285	0.100018	1.18E-06	0.066895	0.588133	0.733494	0.032691	0.017819
-0.38908	-0.03416	-0.24718	-0.52083	-0.3162	0.213038	0.160719	-0.0163	-0.02496
6.16E-06	0.702984	0.005084	3.45E-10	0.000293	0.016184	0.071067	0.855659	0.780595
0.445615	0.257557	0.459624	0.568284	0.246968	-0.27963	-0.29308	0.325508	-0.03702
1.52E-07	0.003463	5.46E-08	3.2E-12	0.005123	0.001453	0.000826	0.000188	0.67948
0.586157	0.536321	0.664984	0.648688	0.592468	-0.27971	-0.32071	0.493639	0.193623
4.49E-13	8.11E-11	1.51E-17	1.63E-16	2.18E-13	0.001449	0.000237	3.7E-09	0.029174
0.8386	0.70081	0.837231	0.764132	0.903683	-0.64134	-0.50867	0.679223	-0.39171
8.96E-35	4.64E-20	1.45E-34	1.47E-25	7.11E-48	4.55E-16	1.02E-09	1.67E-18	5.26E-06
0.559891	0.572308	0.609692	0.389277	0.598858	-0.44682	-0.34848	0.728855	-0.1842
7.73E-12	2.08E-12	2.79E-14	6.09E-06	1.03E-13	1.4E-07	5.94E-05	2.64E-22	0.038162
0.360572	-0.16803	0.128338	0.154543	0.245356	-0.33747	0.041856	0.2717	-0.07642
3.12E-05	0.058976	0.150449	0.082772	0.005431	0.000104	0.640331	0.002002	0.393151
0.602217	0.086775	0.447644	0.729029	0.496439	-0.37687	-0.2428	0.21404	0.026003
6.92E-14	0.332013	1.32E-07	2.55E-22	2.93E-09	1.26E-05	0.005951	0.015678	0.771673
-0.25513	-0.52048	-0.35757	-0.18017	-0.32227	0.211145	0.284857	-0.42264	0.117084
0.003793	3.56E-10	3.67E-05	0.042661	0.00022	0.017178	0.001171	7.43E-07	0.189878
0.264804	-0.41044	-0.04951	0.207834	0.082204	-0.27424	-0.11422	-0.04999	-0.02756
0.002625	1.65E-06	0.580398	0.019043	0.358206	0.001808	0.201017	0.576787	0.758434
0.179688	0.108097	0.199938	0.415808	0.110542	0.194674	-0.04047	0.019601	0.071227
0.043233	0.226395	0.024214	1.16E-06	0.216006	0.028293	0.651476	0.826865	0.426168

ZNF500	ZNF503	ZNF513	ZNF514	ZNF524	ZNF579	ZNF580	ZNF597	ZNF628
black	black	black	black	black	black	black	black	black
-0.39882	-0.48012	-0.13943	0.152231	-0.12774	-0.32329	-0.2124	0.079635	-0.26831
3.41E-06	1.12E-08	0.117946	0.087532	0.152366	0.000209	0.016513	0.373473	0.002289
-0.47123	-0.55507	-0.12885	0.106592	-0.14784	-0.35571	-0.19201	0.144065	-0.27899
2.25E-08	1.27E-11	0.148826	0.232967	0.097175	4.06E-05	0.030571	0.106112	0.001492
0.229479	0.277279	0.092437	0.175863	-0.13231	0.126529	0.058794	0.349038	0.050968
0.009452	0.001599	0.301308	0.04796	0.138122	0.156332	0.511441	5.78E-05	0.56931
-0.09707	0.048315	-0.32052	0.318446	-0.48954	-0.35787	-0.35461	0.597015	-0.39145
0.277609	0.589604	0.000239	0.000264	5.2E-09	3.62E-05	4.3E-05	1.28E-13	5.35E-06
-0.28473	-0.23266	0.05044	-0.35251	0.096184	-0.25928	-0.04022	0.140696	-0.32497
0.001177	0.008483	0.57332	4.81E-05	0.282055	0.003245	0.653487	0.114625	0.000193
-0.42375	-0.22279	-0.39081	0.02997	-0.4565	-0.63699	-0.50297	0.616665	-0.68172
6.9E-07	0.011817	5.56E-06	0.738018	6.89E-08	8.25E-16	1.68E-09	1.17E-14	1.12E-18
-0.32749	-0.37233	-0.23784	0.107997	-0.41711	-0.63216	-0.37463	0.450185	-0.4336
0.000171	1.63E-05	0.007091	0.22683	1.07E-06	1.58E-15	1.43E-05	1.09E-07	3.54E-07
-0.18565	-0.29373	-0.15356	0.077389	-0.25749	-0.39019	-0.24299	-0.07176	-0.41003
0.036651	0.000803	0.084771	0.387147	0.003472	5.77E-06	0.005912	0.422691	1.69E-06
0.154304	0.386098	0.026712	0.032561	0.03113	0.089074	-0.05191	0.073843	0.127285
0.083253	7.35E-06	0.765625	0.716298	0.728262	0.319315	0.562185	0.409331	0.153854
-0.13125	0.021439	0.045551	-0.12466	0.180635	-0.12293	-0.11738	-0.1972	-0.08369
0.141341	0.810919	0.611087	0.162591	0.042126	0.168561	0.18876	0.026269	0.349552
-0.24896	-0.07458	-0.11136	0.038711	-0.1134	-0.2708	-0.30847	0.05517	-0.26166
0.004766	0.40468	0.212615	0.665671	0.20428	0.002074	0.000418	0.537858	0.002963
0.198341	0.200717	0.136556	0.065751	0.201505	0.295676	0.190433	-0.2642	0.378236
0.025395	0.023655	0.1258	0.462677	0.023101	0.000738	0.031992	0.002686	1.16E-05
0.648542	0.44537	0.47448	0.245492	0.376902	0.688669	0.558611	-0.31785	0.690437
1.67E-16	1.55E-07	1.75E-08	0.005404	1.26E-05	3.62E-19	8.82E-12	0.000271	2.7E-19
0.634391	0.576785	0.776128	-0.38507	0.915413	0.89466	0.772517	-0.56904	0.831921
1.17E-15	1.28E-12	8.46E-27	7.81E-06	3.08E-51	1.44E-45	2.04E-26	2.95E-12	9.06E-34
0.504333	0.432945	0.565209	-0.08589	0.681691	0.656144	0.535482	-0.61021	0.664678
1.49E-09	3.7E-07	4.43E-12	0.336984	1.13E-18	5.6E-17	8.78E-11	2.62E-14	1.59E-17
-0.12548	-0.34559	0.039413	-0.00971	0.204286	0.091799	0.208602	-0.41907	0.101151
0.159826	6.91E-05	0.659977	0.913737	0.021235	0.304673	0.018596	9.41E-07	0.257823
0.246205	0.080401	0.243883	-0.023	0.153945	0.436129	0.388917	-0.26461	0.422075
0.005267	0.368884	0.005726	0.797444	0.083982	2.97E-07	6.22E-06	0.002645	7.71E-07
-0.35185	-0.2761	-0.51168	0.04262	-0.54065	-0.40059	-0.43113	0.427347	-0.39582
4.98E-05	0.001678	7.85E-10	0.634238	5.33E-11	3.06E-06	4.19E-07	5.42E-07	4.1E-06
-0.19018	-0.28767	-0.18589	-0.10262	-0.17832	-0.09862	-0.0828	-0.08417	-0.05544
0.032223	0.00104	0.036401	0.250957	0.044871	0.269977	0.354709	0.346806	0.53583
0.238764	0.043757	0.082257	0.361675	0.036974	0.221402	0.262997	0.179111	0.167872
0.006865	0.625215	0.357896	2.94E-05	0.679829	0.012366	0.002815	0.043921	0.059224

ZNF653	ZNF668	ZNF672	ZNF688	ZNF703	ZNF747	ZNF768	ZNF771	ZNF775
black	black	black	black	black	black	black	black	black
-0.25535	-0.17791	-0.17988	-0.06838	-0.20844	0.084998	-0.27613	-0.29653	-0.39419
0.003763	0.045378	0.043005	0.444939	0.01869	0.342044	0.001676	0.000711	4.53E-06
-0.22574	-0.18289	-0.22678	-0.11084	-0.25674	0.106097	-0.32351	-0.26216	-0.35697
0.010715	0.039587	0.01035	0.214743	0.003571	0.235154	0.000207	0.002907	3.79E-05
0.146985	0.043893	0.152895	-0.11622	-0.13087	-0.15657	0.193987	0.011668	0.255519
0.099142	0.624138	0.086143	0.19318	0.142494	0.078778	0.028866	0.896411	0.003739
-0.31901	-0.39054	-0.21427	-0.39876	-0.45187	-0.45439	-0.27508	-0.39788	-0.24725
0.000257	5.64E-06	0.015562	3.43E-06	9.68E-08	8.05E-08	0.001748	3.62E-06	0.005071
-0.23422	-0.30184	-0.06403	0.343712	-0.25682	0.027232	-0.23885	0.094865	-0.07076
0.008039	0.000563	0.474506	7.61E-05	0.003561	0.761194	0.006844	0.288737	0.429245
-0.59836	-0.66229	-0.36322	-0.21419	-0.57835	-0.41618	-0.57401	-0.40365	-0.46946
1.1E-13	2.27E-17	2.7E-05	0.015601	1.07E-12	1.14E-06	1.73E-12	2.53E-06	2.58E-08
-0.43532	-0.50802	-0.34417	-0.29642	-0.50973	-0.36176	-0.36921	-0.44431	-0.46561
3.14E-07	1.08E-09	7.43E-05	0.000715	9.32E-10	2.93E-05	1.94E-05	1.67E-07	3.47E-08
-0.36564	-0.48677	-0.02423	0.035917	-0.12269	-0.24432	-0.28037	-0.28315	-0.3319
2.37E-05	6.53E-09	0.786879	0.688498	0.169371	0.005636	0.00141	0.001257	0.000138
-0.01434	0.115863	0.132263	-0.06775	0.015543	-0.06753	0.178199	-0.22871	-0.0811
0.872889	0.194572	0.138257	0.449135	0.86231	0.450615	0.045026	0.0097	0.364744
-0.36504	-0.14194	0.159331	0.23834	0.038646	0.024573	-0.06115	-0.33797	-0.28115
2.45E-05	0.111431	0.073573	0.006967	0.666193	0.783913	0.49461	0.000102	0.001365
-0.38879	-0.22821	-0.01596	-0.02655	-0.12395	-0.10428	-0.21423	-0.44529	-0.38167
6.27E-06	0.009867	0.85865	0.766991	0.165033	0.243323	0.015583	1.56E-07	9.53E-06
0.451078	0.635853	0.036448	-0.01922	0.158528	0.310152	0.353793	-0.04427	0.250415
1.03E-07	9.62E-16	0.684141	0.830196	0.075054	0.000387	4.5E-05	0.621176	0.004518
0.634305	0.643802	0.551389	0.155262	0.424972	0.272522	0.757266	0.225325	0.583207
1.18E-15	3.24E-16	1.84E-11	0.081334	6.36E-07	0.001937	6.98E-25	0.010865	6.26E-13
0.733987	0.749045	0.752279	0.807817	0.660961	0.569577	0.82258	0.732061	0.83733
9.57E-23	4.22E-24	2.1E-24	1.77E-30	2.76E-17	2.79E-12	1.96E-32	1.4E-22	1.4E-34
0.388209	0.57322	0.689956	0.537261	0.600103	0.419509	0.687308	0.18632	0.452827
6.49E-06	1.88E-12	2.93E-19	7.41E-11	8.9E-14	9.14E-07	4.53E-19	0.035961	9.03E-08
0.00588	0.089742	0.072314	0.163266	0.224533	0.242255	-0.01972	0.050943	0.100769
0.947687	0.315681	0.419128	0.066648	0.011154	0.006068	0.825847	0.569496	0.259634
0.515197	0.432787	0.175279	-0.00043	0.295163	0.14832	0.334325	0.408823	0.51145
5.74E-10	3.74E-07	0.048718	0.996152	0.000755	0.096077	0.000122	1.83E-06	8.01E-10
-0.19738	-0.32867	-0.6237	-0.51519	-0.33911	-0.31766	-0.54202	0.014806	-0.28067
0.026127	0.000161	4.79E-15	5.74E-10	9.62E-05	0.000274	4.67E-11	0.868773	0.001392
0.102164	0.002997	-0.36797	-0.22782	-0.02404	0.019025	-0.20506	0.244928	-0.0679
0.253066	0.973324	2.08E-05	0.009995	0.788491	0.831874	0.020738	0.005515	0.448166
0.278721	0.227935	0.105402	-0.05791	-0.06928	0.042944	0.228662	0.083692	0.255664
0.001508	0.009957	0.238254	0.517811	0.438926	0.631664	0.009716	0.34954	0.003719

ZNF784	ZNF787	ZNF835	ZNF837	ZNF84	ZNF860	ZNF865	ZNHIT2	ABCA5
black	black	black	black	black	black	black	black	blue
-0.35948	-0.27781	-0.32432	-0.33242	-0.03976	-0.01861	-0.25752	-0.22675	0.314554
3.32E-05	0.001565	0.000199	0.000134	0.657134	0.83552	0.003468	0.01036	0.000316
-0.38736	-0.31256	-0.34264	-0.37945	0.029602	-0.05164	-0.31508	-0.22054	0.303644
6.83E-06	0.000347	8.04E-05	1.08E-05	0.741122	0.564221	0.000308	0.012719	0.00052
0.394812	0.07566	0.31779	0.178411	0.390018	0.066955	0.061942	-0.06415	-0.56056
4.36E-06	0.397875	0.000272	0.044767	5.82E-06	0.454504	0.489054	0.473669	7.21E-12
0.033434	-0.38739	-0.06066	-0.19756	0.523783	0.349255	-0.34337	-0.3756	-0.28653
0.709029	6.81E-06	0.49813	0.025989	2.63E-10	5.71E-05	7.74E-05	1.35E-05	0.001091
-0.04557	-0.13385	-0.23808	-0.20878	0.071949	-0.00013	-0.48277	0.18351	-0.05525
0.61093	0.133554	0.00703	0.018491	0.421481	0.998878	9.03E-09	0.038907	0.537302
-0.21747	-0.56677	-0.40386	-0.4333	0.430404	0.399821	-0.7353	-0.29257	-0.08382
0.014049	3.76E-12	2.5E-06	3.61E-07	4.4E-07	3.21E-06	7.34E-23	0.000844	0.348826
-0.24022	-0.54081	-0.3899	-0.4441	0.33477	0.253339	-0.58657	-0.36611	-0.04788
0.006521	5.26E-11	5.87E-06	1.69E-07	0.00012	0.004056	4.28E-13	2.31E-05	0.592955
-0.23907	-0.37387	-0.36648	-0.21512	0.151809	0.224758	-0.38721	-0.0483	0.405172
0.006792	1.49E-05	2.26E-05	0.015149	0.088424	0.011071	6.89E-06	0.589742	2.3E-06
0.259415	0.101857	-0.03367	0.222784	-0.16185	0.133661	0.183199	-0.01575	-0.19632
0.003228	0.254499	0.707063	0.011818	0.06908	0.134103	0.039244	0.860497	0.026955
-0.05864	-0.04608	-0.40569	-0.00126	-0.29953	-0.01813	-0.07688	0.060707	0.113545
0.512548	0.606945	2.23E-06	0.988793	0.000624	0.839704	0.390304	0.497775	0.203711
-0.10982	-0.22795	-0.30997	-0.13915	-0.01484	0.196614	-0.17469	-0.11902	0.101389
0.219017	0.009951	0.000391	0.118696	0.868485	0.026726	0.049498	0.182606	0.2567
0.259093	0.224926	0.355347	0.184622	-0.15997	-0.21536	0.46232	0.036598	-0.25327
0.003268	0.01101	4.14E-05	0.037717	0.072416	0.015033	4.45E-08	0.682906	0.004067
0.599513	0.581907	0.590537	0.569874	-0.11028	-0.37528	0.783203	0.206953	-0.36891
9.55E-14	7.24E-13	2.72E-13	2.7E-12	0.217113	1.38E-05	1.45E-27	0.019569	1.97E-05
0.696185	0.931208	0.586771	0.803995	-0.49893	-0.53728	0.808504	0.830879	-0.19641
1.03E-19	1.24E-56	4.19E-13	5.33E-30	2.37E-09	7.39E-11	1.45E-30	1.29E-33	0.026884
0.4795	0.648115	0.247372	0.606379	-0.51156	-0.46591	0.727014	0.472983	-0.07966
1.17E-08	1.77E-16	0.005049	4.19E-14	7.93E-10	3.39E-08	3.78E-22	1.96E-08	0.373334
-0.25161	0.053593	-0.06315	-0.08443	-0.17888	-0.39266	0.082084	0.062558	0.352021
0.004323	0.549556	0.480588	0.345302	0.0442	4.97E-06	0.358908	0.484737	4.94E-05
0.188902	0.357361	0.474103	0.210815	0.009205	-0.34771	0.410987	0.112655	-0.20588
0.033423	3.72E-05	1.8E-08	0.017356	0.918196	6.19E-05	1.59E-06	0.207301	0.020227
-0.48197	-0.42667	-0.06978	-0.40918	0.328379	0.383891	-0.41722	-0.35433	0.199565
9.63E-09	5.67E-07	0.435636	1.78E-06	0.000164	8.37E-06	1.06E-06	4.37E-05	0.024486
-0.39385	-0.11687	0.015256	-0.22449	-0.02248	0.075649	-0.09124	-0.12319	0.291556
4.62E-06	0.19071	0.864827	0.011169	0.801938	0.397943	0.307621	0.167636	0.000882
0.284674	0.143526	0.26358	0.045705	0.217185	-0.23803	0.116613	-0.08395	-0.5676
0.00118	0.107438	0.002752	0.609883	0.014179	0.007043	0.191681	0.348079	3.44E-12

ABHD12	ABLIM3	ACAD8	ACBD6	ACLY	ACOT4	ACTR8	ADAMTS6	ADH1B
blue	blue	blue	blue	blue	blue	blue	blue	blue
0.428495	0.444974	-0.39126	-0.28094	-0.38639	0.482928	-0.26774	-0.3211	0.227738
5.02E-07	1.59E-07	5.41E-06	0.001377	7.23E-06	8.91E-09	0.002341	0.000232	0.010023
0.379988	0.443749	-0.33545	-0.18552	-0.38028	0.533325	-0.23936	-0.38837	0.280727
1.05E-05	1.74E-07	0.000116	0.036777	1.03E-05	1.08E-10	0.006723	6.43E-06	0.001389
-0.70869	-0.70254	0.563053	0.4746	0.677246	-0.52105	0.588807	0.672375	-0.49644
1.15E-20	3.43E-20	5.56E-12	1.73E-08	2.29E-18	3.38E-10	3.32E-13	4.9E-18	2.93E-09
-0.68299	-0.6646	0.420258	0.072584	0.397747	-0.36008	0.510752	0.691989	-0.35445
9.16E-19	1.6E-17	8.7E-07	0.417388	3.65E-06	3.21E-05	8.52E-10	2.09E-19	4.34E-05
0.029303	0.002501	-0.08319	0.292518	-0.3296	0.29625	-0.15295	-0.37016	0.134715
0.743644	0.977741	0.352438	0.000846	0.000154	0.00072	0.08602	1.84E-05	0.131032
-0.40789	-0.41818	0.133366	0.03539	-0.08715	0.010746	0.218059	0.234213	-0.10024
1.94E-06	9.98E-07	0.13497	0.692843	0.32994	0.904554	0.013785	0.008041	0.262136
-0.23628	-0.16464	0.062441	-0.12209	-0.01911	0.066545	0.180502	0.148681	0.029095
0.007487	0.064359	0.485555	0.171486	0.831175	0.457282	0.04228	0.095262	0.745402
-0.0848	-0.02631	-0.10278	-0.28192	-0.3274	-0.18727	-0.06009	-0.05082	0.043268
0.343179	0.76905	0.250206	0.001322	0.000172	0.03501	0.502192	0.570415	0.629084
-0.18898	-0.14189	-0.04038	0.047033	0.511031	-0.33562	0.128743	0.523476	-0.32813
0.033351	0.111544	0.652195	0.599521	8.31E-10	0.000115	0.149156	2.71E-10	0.000166
0.482203	0.427525	-0.42885	-0.31208	-0.24086	0.049644	-0.35485	0.011841	0.083072
9.45E-09	5.36E-07	4.9E-07	0.000354	0.006376	0.579396	4.25E-05	0.894886	0.353136
0.237738	0.160355	-0.2175	-0.40279	-0.21437	0.106622	-0.1553	0.225994	0.004676
0.007116	0.071716	0.014035	2.67E-06	0.015513	0.232831	0.081268	0.010625	0.958388
0.078865	0.173862	0.006282	0.004273	0.403772	0.158639	-0.07764	0.217943	-0.05927
0.37813	0.050599	0.944116	0.961977	2.51E-06	0.074848	0.38559	0.013837	0.508005
-0.1149	-0.0755	0.251906	0.278749	0.659831	-0.46406	0.204948	0.447908	-0.32451
0.198326	0.398872	0.004277	0.001506	3.26E-17	3.9E-08	0.020811	1.29E-07	0.000198
0.302425	0.117856	-0.02446	0.369629	0.167556	-0.19872	-0.28505	-0.17464	-0.11973
0.000549	0.186955	0.784847	1.9E-05	0.059711	0.02511	0.001161	0.049553	0.179979
0.478895	0.398502	-0.24615	0.011587	0.171515	-0.25525	-0.30332	0.097867	-0.09227
1.23E-08	3.48E-06	0.005277	0.897127	0.053846	0.003777	0.000527	0.27368	0.30217
0.690298	0.619369	-0.27656	-0.23148	-0.5105	0.358596	-0.36015	-0.60579	0.481873
2.76E-19	8.34E-15	0.001647	0.00883	8.71E-10	3.48E-05	3.2E-05	4.5E-14	9.7E-09
-0.08075	-0.07707	0.316918	0.276611	0.226153	-0.0677	0.187268	-0.10845	0.012124
0.36681	0.389139	0.000283	0.001643	0.010569	0.449465	0.035011	0.224886	0.892386
-0.30191	-0.32112	0.206251	-0.163	-0.22871	0.240101	0.223632	-0.20035	0.140233
0.000561	0.000232	0.019996	0.067089	0.009701	0.006549	0.011492	0.023914	0.115835
0.101777	0.173905	-0.03372	-0.12759	-0.30893	0.391186	-0.07984	-0.54655	0.31039
0.254874	0.050541	0.706666	0.152868	0.00041	5.43E-06	0.372266	2.99E-11	0.000383
-0.31767	-0.24265	0.2831	0.592096	0.475009	-0.15206	0.445984	0.177805	-0.16502
0.000273	0.005983	0.001259	2.28E-13	1.68E-08	0.087889	1.48E-07	0.045509	0.063728

ADNP2	AFAP1	AGBL5	AHCY	AK1	AKNAD1	AKR1C1	ALDH18A1	ALDH1A1
blue	blue	blue	blue	blue	blue	blue	blue	blue
-0.28922	-0.38723	-0.35167	-0.39499	0.092649	0.001848	0.257138	-0.26013	0.501295
0.000974	6.88E-06	5.03E-05	4.32E-06	0.300196	0.983545	0.003518	0.003142	1.94E-09
-0.26448	-0.43051	-0.31524	-0.31557	0.086175	0.001595	0.310578	-0.25882	0.462484
0.002658	4.37E-07	0.000306	0.000302	0.335382	0.985798	0.00038	0.003302	4.4E-08
0.641764	0.557312	0.680237	0.5372	-0.55636	0.494069	-0.60797	0.67869	-0.67247
4.3E-16	1.01E-11	1.42E-18	7.45E-11	1.11E-11	3.57E-09	3.45E-14	1.82E-18	4.83E-18
0.499703	0.404534	0.31552	0.347732	-0.6608	0.482207	-0.56176	0.366617	-0.50907
2.22E-09	2.39E-06	0.000302	6.18E-05	2.83E-17	9.45E-09	6.37E-12	2.24E-05	9.88E-10
-0.17875	-0.4476	0.146708	0.284271	-0.01997	0.096757	0.346735	-0.18707	0.072305
0.04435	1.32E-07	0.099787	0.0012	0.823654	0.279181	6.51E-05	0.035205	0.419183
0.16679	-0.0582	0.099568	0.301142	-0.51492	0.363686	-0.12373	0.022777	-0.17968
0.060903	0.515744	0.265384	0.000581	5.88E-10	2.64E-05	0.16579	0.799358	0.043245
0.153324	-4.9E-05	0.10536	0.236074	-0.37328	0.233566	-0.01332	-0.07588	-0.22164
0.085255	0.999568	0.23844	0.00754	1.54E-05	0.008223	0.88188	0.396474	0.01227
-0.26555	-0.34191	-0.09086	-0.25491	0.437896	0.101954	0.173728	-0.35753	0.246592
0.002549	8.35E-05	0.309685	0.003825	2.63E-07	0.254044	0.05078	3.68E-05	0.005194
0.056451	0.540126	0.151566	0.382282	-0.35725	0.051922	-0.44051	0.548854	-0.23678
0.528446	5.62E-11	0.088939	9.2E-06	3.74E-05	0.562095	2.19E-07	2.38E-11	0.007358
-0.43638	-0.23402	-0.37319	-0.01227	0.102405	-0.09541	0.237333	-0.10954	0.398891
2.92E-07	0.008095	1.55E-05	0.891079	0.251941	0.285986	0.007217	0.220241	3.4E-06
-0.2136	-0.10173	-0.32574	-0.10787	-0.01793	0.104397	-0.0091	-0.11965	0.250934
0.015896	0.255109	0.000186	0.227397	0.841395	0.242783	0.91911	0.180304	0.004433
0.087345	0.371922	0.125605	0.155275	0.24791	-0.18561	-0.25138	0.306846	-0.15999
0.328835	1.67E-05	0.159404	0.081308	0.004951	0.036691	0.00436	0.00045	0.072368
0.293513	0.519103	0.406732	0.170727	0.039425	0.111784	-0.42737	0.510777	-0.36786
0.000811	4.04E-10	2.08E-06	0.054974	0.65988	0.210858	5.41E-07	8.5E-10	2.09E-05
-0.18043	-0.03205	0.23965	0.231028	0.363207	-0.07916	0.020196	0.106989	-0.01669
0.04236	0.720552	0.006654	0.008968	2.71E-05	0.376371	0.821688	0.231218	0.852268
-0.29068	0.078524	-0.04181	0.0239	0.338132	-0.12131	-0.01474	0.164109	0.166274
0.000916	0.380201	0.640738	0.789687	0.000101	0.174272	0.869334	0.065234	0.061718
-0.43674	-0.54718	-0.4849	-0.51645	0.477887	-0.341	0.488365	-0.47748	0.580863
2.85E-07	2.81E-11	7.6E-09	5.13E-10	1.33E-08	8.74E-05	5.73E-09	1.38E-08	8.13E-13
0.232856	0.142972	0.258206	-0.09433	0.163429	-0.06797	-0.04465	0.08081	-0.2
0.008426	0.108817	0.003379	0.29149	0.066372	0.447658	0.618199	0.366442	0.024171
0.234288	-0.0725	-0.14722	-0.23967	-0.20237	-0.07285	0.024068	-0.24115	-0.03679
0.008021	0.417924	0.098593	0.006648	0.022508	0.415666	0.788243	0.006311	0.681348
-0.07655	-0.23591	-0.2147	-0.39529	0.282756	-0.35589	0.359264	-0.3694	0.200328
0.39234	0.007582	0.015351	4.24E-06	0.001278	4.02E-05	3.35E-05	1.92E-05	0.023933
0.545237	0.331994	0.604861	0.275268	-0.3416	0.14127	-0.30112	0.51392	-0.53711
3.4E-11	0.000137	5.03E-14	0.001735	8.48E-05	0.113137	0.000581	6.43E-10	7.52E-11

ALDH1B1	ALKBH2	ALYREF	AMMECR1	ANKH	ANKRD35	ANKRD50	ANP32E	ANXA11
blue	blue	blue	blue	blue	blue	blue	blue	blue
-0.34147	-0.29963	-0.39249	-0.34243	0.492192	0.117861	-0.35864	-0.38291	0.466288
8.54E-05	0.000621	5.02E-06	8.13E-05	4.17E-09	0.186937	3.47E-05	8.87E-06	3.29E-08
-0.33208	-0.2036	-0.40556	-0.35728	0.484465	0.128542	-0.41037	-0.39585	0.46027
0.000137	0.021685	2.24E-06	3.73E-05	7.87E-09	0.149794	1.65E-06	4.09E-06	5.2E-08
0.546123	0.556644	0.637741	0.799826	-0.59517	-0.52196	0.645365	0.671918	-0.84005
3.12E-11	1.08E-11	7.45E-16	1.73E-29	1.59E-13	3.11E-10	2.61E-16	5.26E-18	5.34E-35
0.239839	0.276184	0.20447	0.710727	-0.52185	-0.38144	0.512011	0.512073	-0.81058
0.00661	0.001672	0.021116	8E-21	3.14E-10	9.66E-06	7.62E-10	7.58E-10	7.86E-31
0.0079	0.444901	0.208953	-0.34873	0.035665	0.004725	-0.41368	-0.21077	0.109137
0.929759	1.6E-07	0.018394	5.87E-05	0.690576	0.957955	1.34E-06	0.017378	0.221935
0.015607	0.288114	0.060923	0.218	-0.24642	-0.18224	0.044588	0.150145	-0.42526
0.861746	0.001021	0.496241	0.013811	0.005227	0.040298	0.61865	0.092011	6.24E-07
-0.10265	0.165885	-0.12302	0.117534	-0.10558	-0.15396	-0.08439	-0.1123	-0.17745
0.250808	0.062338	0.16825	0.188169	0.23746	0.083959	0.345509	0.208761	0.04595
-0.37494	-0.19056	-0.24065	-0.33119	0.060196	0.241696	-0.2138	-0.01656	0.05056
1.41E-05	0.031872	0.006423	0.000143	0.501407	0.00619	0.015795	0.853372	0.572404
0.275018	0.037298	0.325875	0.474774	-0.02032	-0.13044	0.306553	0.073388	-0.23163
0.001753	0.677179	0.000185	1.71E-08	0.820609	0.143828	0.000456	0.412232	0.008785
-0.22662	-0.20334	-0.07701	-0.21613	0.333154	0.231462	-0.45015	-0.39556	0.391904
0.010407	0.021858	0.389489	0.014667	0.00013	0.008837	1.1E-07	4.17E-06	5.2E-06
-0.1995	-0.21877	-0.15379	-0.04414	0.048928	0.196279	-0.25049	-0.15674	0.097112
0.024532	0.013471	0.084294	0.622199	0.584885	0.026992	0.004505	0.078448	0.277417
0.281191	0.027301	0.028524	0.255113	-0.01387	-0.0725	0.006498	-0.25526	-0.01109
0.001363	0.76061	0.750227	0.003796	0.876996	0.417937	0.942198	0.003776	0.901509
0.353081	0.165889	0.422358	0.500198	-0.23043	-0.19359	0.356256	0.314438	-0.2778
4.67E-05	0.062331	7.57E-07	2.13E-09	0.009153	0.029205	3.94E-05	0.000318	0.001566
0.216608	0.405377	0.613596	-0.13262	-0.04749	0.205467	-0.17818	0.025058	0.133801
0.014444	2.27E-06	1.72E-14	0.137195	0.595943	0.020483	0.045051	0.77975	0.133691
0.027752	-0.01749	0.284843	-0.02215	0.182699	0.230251	-0.22977	-0.14865	0.288829
0.756779	0.845229	0.001171	0.80474	0.039793	0.009208	0.009359	0.095322	0.00099
-0.36577	-0.34294	-0.45084	-0.61604	0.495974	0.316679	-0.54597	-0.4304	0.632775
2.35E-05	7.92E-05	1.04E-07	1.27E-14	3.04E-09	0.000286	3.17E-11	4.41E-07	1.45E-15
0.250026	0.1152	0.119488	0.058451	-0.18634	-0.19133	0.245293	0.204952	-0.08854
0.004583	0.197155	0.18088	0.513915	0.03594	0.031181	0.005443	0.020808	0.32226
-0.11324	-0.20753	-0.38549	-0.04903	-0.10817	-0.09687	0.337854	0.191759	-0.1493
0.204933	0.019225	7.62E-06	0.584107	0.226083	0.27861	0.000102	0.030794	0.093883
-0.17566	-0.33911	-0.44315	-0.45012	0.20747	0.087815	-0.0282	-0.18877	0.369253
0.04822	9.62E-05	1.81E-07	1.1E-07	0.019259	0.32623	0.752965	0.03355	1.94E-05
0.491017	0.390865	0.427268	0.487898	-0.17225	-0.74246	0.405591	0.258623	-0.34095
4.6E-09	5.54E-06	5.45E-07	5.95E-09	0.052815	1.7E-23	2.24E-06	0.003326	8.76E-05

ANXA7	AP3M1	APEX1	ARHGAP1:	ARHGAP1:	ARHGAP4:	ARHGEF39	ARL5B	ARMC3
blue	blue	blue	blue	blue	blue	blue	blue	blue
0.525981	-0.1719	-0.22832	-0.405	-0.39625	-0.46374	-0.41966	-0.13567	0.269696
2.15E-10	0.053303	0.009829	2.32E-06	4E-06	4E-08	9.05E-07	0.128285	0.002167
0.566557	-0.12267	-0.19953	-0.38774	-0.41838	-0.46584	-0.44791	-0.11869	0.234741
3.84E-12	0.169455	0.024508	6.67E-06	9.85E-07	3.41E-08	1.29E-07	0.183829	0.007896
-0.772	0.573075	0.587293	0.911342	0.755382	0.809194	0.863627	0.462449	-0.58683
2.31E-26	1.91E-12	3.95E-13	5.11E-50	1.06E-24	1.18E-30	5.41E-39	4.41E-08	4.16E-13
-0.48931	0.47162	0.172506	0.647229	0.648439	0.738918	0.562068	0.424337	-0.34266
5.3E-09	2.18E-08	0.052454	2.01E-16	1.69E-16	3.53E-23	6.16E-12	6.63E-07	8.03E-05
0.223561	-0.28401	0.122033	-0.17507	-0.14551	-0.20559	0.008486	0.042799	0.055512
0.011519	0.001213	0.1717	0.048991	0.102606	0.020406	0.92456	0.632811	0.535334
-0.02815	0.134836	-0.02214	0.246843	0.325606	0.315222	0.257115	0.320628	-0.0975
0.753371	0.130683	0.804859	0.005147	0.000187	0.000307	0.003522	0.000238	0.275472
0.057663	-0.00078	-0.08777	-0.06342	0.115757	0.169931	0.024337	0.068251	0.048613
0.519611	0.993058	0.326481	0.478695	0.194982	0.056132	0.785936	0.445799	0.587304
0.136662	-0.10458	-0.41138	-0.31889	-0.03755	-0.10383	-0.11118	-0.16377	0.635489
0.125505	0.241941	1.55E-06	0.000258	0.67511	0.245349	0.213356	0.065803	1.01E-15
-0.36639	0.119585	0.147525	0.377672	0.333752	0.44581	0.378145	-0.08341	-0.20576
2.27E-05	0.180527	0.097893	1.2E-05	0.000126	1.5E-07	1.17E-05	0.351196	0.0203
0.259386	-0.21568	-0.19387	-0.25006	-0.10857	-0.24715	-0.15325	-0.35186	0.293114
0.003232	0.014881	0.028966	0.004578	0.224364	0.00509	0.085402	4.98E-05	0.000825
0.192412	-0.06653	-0.21077	-0.12862	0.010635	-0.1109	-0.0073	-0.23579	0.159601
0.030218	0.457405	0.017381	0.149552	0.905536	0.214511	0.935067	0.007614	0.073079
-0.05769	0.027499	0.311285	-0.01167	-0.30968	0.00848	-0.02628	-0.41222	-0.12842
0.519452	0.758924	0.000368	0.896373	0.000396	0.924612	0.769341	1.47E-06	0.150185
-0.63255	0.295775	0.462108	0.431636	0.304529	0.379166	0.433237	-0.08444	-0.28502
1.5E-15	0.000735	4.53E-08	4.05E-07	0.0005	1.1E-05	3.63E-07	0.34524	0.001163
-0.30008	-0.25117	0.417056	-0.00524	-0.03494	-0.13188	0.188048	-0.29725	-0.16302
0.000609	0.004394	1.07E-06	0.953406	0.696575	0.139421	0.034246	0.000689	0.067066
-0.18317	-0.11501	0.170107	-0.04724	-0.03695	-0.12857	0.06975	-0.49154	0.084666
0.039274	0.197906	0.055874	0.597908	0.680058	0.1497	0.435848	4.41E-09	0.343943
0.567259	-0.13961	-0.27925	-0.55712	-0.59117	-0.71937	-0.60878	-0.36681	0.453487
3.57E-12	0.117476	0.001476	1.03E-11	2.53E-13	1.63E-21	3.13E-14	2.22E-05	8.6E-08
-0.17464	0.180521	0.319685	0.11554	-0.05493	0.018649	0.043209	0.124602	-0.17728
0.049563	0.042258	0.000249	0.195826	0.539629	0.835151	0.629559	0.162787	0.046162
0.218504	0.097191	-0.25373	-0.02845	-0.05084	0.025098	-0.23439	0.456397	-0.09473
0.013588	0.277023	0.003997	0.750895	0.570292	0.779413	0.007992	6.94E-08	0.289412
0.438475	-0.18809	-0.27289	-0.42047	-0.45504	-0.40657	-0.5167	0.082774	0.133705
2.52E-07	0.034202	0.001909	8.58E-07	7.67E-08	2.11E-06	5.01E-10	0.354871	0.133972
-0.513	0.292041	0.529699	0.513862	0.280647	0.45409	0.415931	0.428982	-0.35284
6.98E-10	0.000864	1.52E-10	6.46E-10	0.001394	8.23E-08	1.16E-06	4.85E-07	4.73E-05

ASB2	ASF1B	ASPM	ASS1	ATAD2	ATAD5	ATF7IP	ATG7	ATP6V1B1
blue	blue	blue	blue	blue	blue	blue	blue	blue
0.454325	-0.48401	-0.47771	0.306685	-0.47661	-0.40658	-0.30082	0.418091	-0.44947
8.09E-08	8.17E-09	1.35E-08	0.000453	1.48E-08	2.1E-06	0.000589	1E-06	1.15E-07
0.420291	-0.47622	-0.47554	0.316769	-0.48038	-0.40553	-0.32847	0.405373	-0.4932
8.68E-07	1.52E-08	1.61E-08	0.000285	1.09E-08	2.25E-06	0.000163	2.27E-06	3.84E-09
-0.57029	0.896209	0.903198	-0.53913	0.887088	0.896066	0.589711	-0.64585	0.782977
2.58E-12	5.99E-46	9.59E-48	6.18E-11	8.65E-44	6.49E-46	2.99E-13	2.43E-16	1.53E-27
-0.57974	0.589207	0.613433	-0.41423	0.686165	0.73378	0.480167	-0.36878	0.51867
9.21E-13	3.17E-13	1.76E-14	1.29E-06	5.46E-19	9.97E-23	1.11E-08	1.99E-05	4.2E-10
0.108218	-0.03865	-0.21976	0.279209	-0.21863	-0.2293	-0.53063	0.188417	-0.1656
0.225872	0.666202	0.013047	0.001478	0.013531	0.009509	1.39E-10	0.033889	0.062793
-0.30298	0.260813	0.179953	-0.05966	0.267574	0.280893	-0.05082	0.021438	0.096597
0.000535	0.003061	0.042922	0.505211	0.002356	0.00138	0.570468	0.810928	0.279985
-0.08356	0.016327	-0.09203	-0.0299	-0.02345	0.003563	-0.12268	0.118102	-0.01981
0.350331	0.85543	0.303473	0.73861	0.79354	0.968285	0.169418	0.186029	0.825064
0.013017	-0.15301	-0.14891	0.117954	-0.08944	-0.17284	-0.07543	0.125388	-0.18825
0.884515	0.085908	0.094752	0.186585	0.317313	0.051996	0.399315	0.160131	0.034053
-0.24367	0.475171	0.366033	-0.19064	0.237791	0.313941	0.242779	-0.14079	0.473725
0.00577	1.65E-08	2.32E-05	0.031805	0.007103	0.000325	0.005956	0.114384	1.85E-08
0.152681	-0.12347	-0.2957	0.140482	-0.40599	-0.29903	-0.11948	0.424954	-0.10851
0.086589	0.166669	0.000738	0.115183	2.18E-06	0.000638	0.180922	6.37E-07	0.224623
0.084541	-0.0585	-0.11263	0.026837	-0.13932	-0.09818	0.022152	0.325142	-0.06516
0.34466	0.513563	0.20741	0.764556	0.118241	0.27214	0.804751	0.000192	0.466742
-0.09157	0.028636	-0.05488	0.036401	-0.09496	-0.02601	0.120452	0.05661	0.296584
0.305861	0.749281	0.539998	0.684521	0.28826	0.771613	0.177363	0.527283	0.00071
-0.25625	0.4532	0.466854	-0.37646	0.344364	0.425535	0.596008	-0.48179	0.620483
0.003638	8.78E-08	3.15E-08	1.29E-05	7.36E-05	6.12E-07	1.44E-13	9.77E-09	7.23E-15
0.061328	0.182822	0.044529	0.023424	-0.12495	-0.12054	-0.05077	-0.23826	0.223929
0.49338	0.039658	0.619115	0.793784	0.161601	0.177041	0.57081	0.006986	0.01138
0.05131	0.093148	-0.0344	-0.07178	-0.24643	-0.13413	0.203363	-0.00428	0.234953
0.566715	0.29759	0.701006	0.422577	0.005224	0.132743	0.02184	0.961948	0.007839
0.411912	-0.65992	-0.61044	0.187793	-0.64368	-0.56837	-0.26186	0.391384	-0.5931
1.5E-06	3.22E-17	2.55E-14	0.034495	3.29E-16	3.17E-12	0.00294	5.37E-06	2.03E-13
0.033353	0.012957	0.158706	-0.17317	0.169459	0.099165	0.173001	-0.32386	0.081324
0.7097	0.885044	0.074724	0.051536	0.056829	0.267335	0.051771	0.000204	0.36339
0.085356	-0.23905	-0.01184	0.047128	0.204031	0.053009	-0.14183	-0.02163	-0.3534
0.34001	0.006795	0.894902	0.598789	0.021401	0.553922	0.111714	0.809263	4.59E-05
0.416437	-0.54002	-0.36292	0.295484	-0.24319	-0.39263	-0.37236	0.165856	-0.56102
1.12E-06	5.67E-11	2.75E-05	0.000744	0.00587	4.98E-06	1.63E-05	0.062384	6.88E-12
-0.15898	0.375716	0.400383	-0.3862	0.363771	0.488721	0.287587	-0.38794	0.510717
0.074221	1.34E-05	3.1E-06	7.31E-06	2.62E-05	5.56E-09	0.001044	6.59E-06	8.54E-10

AUNIP	AURKA	AURKB	AVPI1	BAG5	BAZ1B	BCL2L1	BEND3	BEND7
blue	blue	blue	blue	blue	blue	blue	blue	blue
-0.3299	-0.42083	-0.47462	0.438028	0.060453	-0.29234	0.20633	-0.36727	-0.28294
0.000152	8.38E-07	1.73E-08	2.6E-07	0.499577	0.000853	0.019947	2.16E-05	0.001268
-0.23618	-0.38009	-0.44212	0.51751	0.111407	-0.33207	0.209683	-0.35606	-0.21332
0.007513	1.05E-05	1.95E-07	4.66E-10	0.212411	0.000137	0.017981	3.99E-05	0.016038
0.645738	0.910797	0.892116	-0.69287	0.422627	0.621172	-0.55442	0.86048	0.578079
2.47E-16	7.36E-50	5.89E-45	1.8E-19	7.44E-07	6.62E-15	1.36E-11	2.03E-38	1.11E-12
0.30109	0.681572	0.486372	-0.71042	0.526781	0.610602	-0.55208	0.57162	0.321221
0.000582	1.15E-18	6.74E-09	8.46E-21	1.99E-10	2.5E-14	1.72E-11	2.24E-12	0.000231
0.027632	0.052582	-0.10189	0.204165	-0.1805	-0.47223	0.033701	-0.21195	0.050592
0.757792	0.557123	0.254338	0.021314	0.042284	2.08E-08	0.706811	0.01675	0.572163
0.082195	0.415685	0.108123	-0.33683	0.275538	0.076071	-0.34435	0.112053	0.078344
0.358257	1.17E-06	0.226282	0.000108	0.001716	0.395304	7.36E-05	0.209753	0.381295
0.001043	0.156637	-0.11757	-0.19495	0.19051	0.03785	-0.05128	-0.03122	0.11019
0.990715	0.07864	0.188024	0.028065	0.031921	0.672675	0.566912	0.727538	0.217479
-0.34393	-0.27072	-0.35474	-0.17186	-0.34476	-0.01307	-0.18793	-0.26438	-0.26548
7.52E-05	0.002081	4.27E-05	0.053362	7.21E-05	0.884043	0.034363	0.002668	0.002557
-0.13869	0.468822	0.362334	-0.50878	0.091742	0.433943	0.039176	0.320921	-0.13938
0.119929	2.71E-08	2.84E-05	1.01E-09	0.304974	3.46E-07	0.661896	0.000234	0.118078
-0.6994	-0.19407	-0.29128	0.036681	-0.30425	-0.09166	0.385716	-0.33879	-0.5029
5.92E-20	0.028798	0.000892	0.682227	0.000506	0.305401	7.52E-06	9.78E-05	1.69E-09
-0.42319	-0.09557	-0.18791	-0.08375	0.013873	0.130849	0.139783	-0.12426	-0.44946
7.16E-07	0.285137	0.034376	0.349203	0.876975	0.142559	0.117021	0.163956	1.15E-07
0.011465	-0.02658	0.121104	-0.00519	0.126408	0.265037	0.286154	0.224722	0.043574
0.898202	0.766761	0.175013	0.9538	0.156734	0.002601	0.001109	0.011084	0.626663
0.172244	0.296255	0.527409	-0.30545	0.084742	0.546085	-0.0099	0.586056	0.323855
0.052819	0.00072	1.88E-10	0.000479	0.34351	3.13E-11	0.912	4.54E-13	0.000204
0.001272	-0.00265	0.231799	0.124434	-0.40658	-0.07623	0.338034	0.162119	0.026287
0.988674	0.976439	0.008736	0.163361	2.1E-06	0.394321	0.000102	0.068608	0.769244
-0.45441	-0.11296	0.059439	0.010681	-0.38699	0.135759	0.453049	0.046363	-0.24045
8.03E-08	0.206083	0.506817	0.905134	6.98E-06	0.128045	8.88E-08	0.604743	0.00647
-0.34075	-0.68023	-0.57311	0.617763	-0.32708	-0.45252	0.348453	-0.55233	-0.29366
8.85E-05	1.43E-18	1.91E-12	1.02E-14	0.000174	9.23E-08	5.95E-05	1.68E-11	0.000806
0.497234	-0.03353	0.220708	0.097534	0.057775	0.012549	-0.13241	0.20464	0.39266
2.74E-09	0.708204	0.01265	0.275324	0.518802	0.888638	0.137816	0.021008	4.97E-06
0.452199	-0.07501	-0.12738	0.105284	0.319433	-0.1556	-0.35619	-0.11686	0.133387
9.45E-08	0.401945	0.153547	0.238783	0.000252	0.080659	3.96E-05	0.190719	0.13491
0.225208	-0.47858	-0.372	0.498039	-0.05058	-0.44988	0.029337	-0.41777	-0.00131
0.010907	1.26E-08	1.66E-05	2.56E-09	0.572222	1.12E-07	0.743358	1.02E-06	0.9883
0.467418	0.476408	0.496774	-0.15329	0.277553	0.127251	-0.25466	0.506049	0.674568
3.02E-08	1.5E-08	2.84E-09	0.085334	0.001582	0.153964	0.003861	1.29E-09	3.49E-18

BHLHE41	BHMT2	BIRC5	BLM	BLOC1S2	BORA	BRCA1	BRCA2	BRD7
blue	blue	blue	blue	blue	blue	blue	blue	blue
0.368714	0.32934	-0.49636	-0.35919	0.47941	-0.25802	-0.43395	-0.33937	-0.2082
2E-05	0.000156	2.95E-09	3.37E-05	1.18E-08	0.003403	3.45E-07	9.49E-05	0.01883
0.275947	0.338862	-0.44384	-0.306	0.547625	-0.26127	-0.44442	-0.38967	-0.21732
0.001688	9.74E-05	1.73E-07	0.000468	2.69E-11	0.003008	1.66E-07	5.95E-06	0.014119
-0.62263	-0.54504	0.885462	0.775667	-0.63115	0.81475	0.74496	0.862692	0.558912
5.49E-15	3.47E-11	2.01E-43	9.48E-27	1.81E-15	2.24E-31	1.01E-23	8.04E-39	8.55E-12
-0.25274	-0.34402	0.525789	0.649803	-0.43349	0.760791	0.686704	0.8002	0.36845
0.004147	7.49E-05	2.19E-10	1.39E-16	3.57E-07	3.16E-25	5E-19	1.56E-29	2.03E-05
-0.11467	0.091764	0.046892	-0.12191	0.42639	0.06412	-0.13024	-0.16196	-0.05536
0.199229	0.304857	0.600622	0.172121	5.78E-07	0.473875	0.144454	0.068877	0.536461
-0.06945	-0.08777	0.252264	0.297596	0.103364	0.530158	0.342881	0.417552	0.104166
0.437862	0.326457	0.004221	0.000679	0.247503	1.45E-10	7.94E-05	1.04E-06	0.243833
-0.03148	0.09206	-0.00953	0.124234	0.141636	0.321067	0.058945	0.168587	0.094579
0.725372	0.303297	0.915339	0.164044	0.112198	0.000233	0.510357	0.058135	0.290196
0.339438	0.099539	-0.27314	-0.28389	0.110846	-0.13462	0.208928	-0.10029	-0.06492
9.46E-05	0.265527	0.00189	0.001219	0.214737	0.131299	0.018409	0.261909	0.468334
-0.10445	-0.08464	0.328421	0.23428	-0.38674	0.443772	0.364068	0.446843	0.136983
0.242546	0.34409	0.000163	0.008023	7.08E-06	1.74E-07	2.58E-05	1.39E-07	0.12461
0.536828	0.255693	-0.27209	-0.31048	0.338622	-0.033	-0.09185	-0.07183	-0.07223
7.72E-11	0.003715	0.001971	0.000381	9.86E-05	0.712642	0.304407	0.422241	0.419668
0.475321	0.162432	-0.17563	-0.07489	0.146538	0.073854	-0.00497	0.123593	-0.02582
1.63E-08	0.068068	0.048258	0.402711	0.100184	0.409266	0.955773	0.166247	0.773213
-0.03196	0.198209	0.007291	0.050567	-0.3795	0.035339	-0.00771	-0.03238	0.15506
0.721339	0.025494	0.935158	0.57235	1.08E-05	0.693262	0.931409	0.717793	0.081735
-0.25357	-0.29853	0.354752	0.30252	-0.63491	0.21565	0.338638	0.329471	0.403148
0.004021	0.000652	4.27E-05	0.000546	1.09E-15	0.014894	9.85E-05	0.000155	2.61E-06
-0.14001	-0.20908	0.168905	-0.12556	-0.18519	-0.20083	-0.05414	-0.18053	-0.03753
0.116411	0.018322	0.057656	0.15954	0.037121	0.023575	0.545483	0.042251	0.675306
0.264504	0.020332	-0.05088	-0.20637	-0.13341	-0.12504	-0.00478	-0.09647	0.093614
0.002655	0.820508	0.569983	0.019923	0.134827	0.161315	0.957486	0.280615	0.295169
0.438359	0.406823	-0.5661	-0.46321	0.561894	-0.60065	-0.53731	-0.70962	-0.1437
2.54E-07	2.07E-06	4.03E-12	4.17E-08	6.28E-12	8.35E-14	7.37E-11	9.78E-21	0.107013
-0.34073	-0.16699	0.175105	0.166617	-0.22495	-0.18892	-0.08967	-0.10187	0.157086
8.86E-05	0.060595	0.048946	0.061175	0.011	0.033405	0.316078	0.254446	0.077777
-0.19573	-0.03013	-0.07362	0.118763	0.096987	-0.13697	-0.15053	-0.04712	-0.25089
0.027428	0.736689	0.410721	0.183559	0.278037	0.12466	0.091177	0.59886	0.00444
-0.05729	0.190284	-0.36617	-0.28529	0.276463	-0.58377	-0.52516	-0.52829	-0.35206
0.522317	0.032129	2.3E-05	0.00115	0.001653	5.88E-13	2.32E-10	1.73E-10	4.93E-05
-0.63106	-0.23929	0.450891	0.365949	-0.32675	0.518274	0.176392	0.384656	0.499875
1.83E-15	0.006739	1.04E-07	2.33E-05	0.000177	4.35E-10	0.047282	8.01E-06	2.19E-09

BRIP1	BTN1A1	BUB1	BUB1B	BUD13	BYSL	BZW2	C10orf142	C10orf67
blue	blue	blue	blue	blue	blue	blue	blue	blue
-0.47925	-0.2765	-0.42526	-0.51207	-0.27006	-0.30616	-0.16402	0.291712	0.296405
1.2E-08	0.001651	6.24E-07	7.58E-10	0.002136	0.000464	0.065388	0.000876	0.000715
-0.50902	-0.25972	-0.38534	-0.51672	-0.27029	-0.24965	-0.06102	0.348421	0.306963
9.92E-10	0.003191	7.69E-06	5.01E-10	0.002117	0.004647	0.495533	5.96E-05	0.000448
0.892676	0.567403	0.924048	0.903111	0.67416	0.753881	0.594754	-0.54531	-0.55251
4.34E-45	3.51E-12	4.82E-54	1.01E-47	3.71E-18	1.48E-24	1.67E-13	3.38E-11	1.65E-11
0.602894	0.39318	0.683962	0.599155	0.541139	0.396562	0.417661	-0.41406	-0.29346
6.38E-14	4.82E-06	7.82E-19	9.96E-14	5.09E-11	3.92E-06	1.03E-06	1.3E-06	0.000813
-0.28072	-0.10222	-0.02941	-0.16329	0.356863	0.11824	0.237115	0.006413	0.140519
0.00139	0.252801	0.742731	0.066604	3.82E-05	0.185511	0.007272	0.942958	0.115086
0.12267	0.087481	0.367209	0.200759	0.481369	0.186514	0.35416	-0.22932	0.00059
0.169458	0.328078	2.17E-05	0.023626	1.01E-08	0.035766	4.41E-05	0.009504	0.994748
-0.10241	0.005677	0.082077	-0.06079	0.254547	0.071623	0.302727	-0.02426	0.124424
0.251904	0.949492	0.358954	0.497215	0.003877	0.423598	0.000541	0.786599	0.163394
-0.26737	-0.24576	-0.22073	-0.26915	-0.0308	-0.38364	-0.38598	0.020455	0.378184
0.002375	0.005352	0.012639	0.002215	0.731063	8.5E-06	7.4E-06	0.819443	1.17E-05
0.368003	0.189441	0.380403	0.374273	0.276632	0.302172	0.291497	-0.28691	-0.26354
2.08E-05	0.032913	1.03E-05	1.46E-05	0.001642	0.000555	0.000884	0.001074	0.002756
-0.33444	-0.30197	-0.2329	-0.30968	-0.01393	-0.22899	-0.27821	0.088595	0.19963
0.000122	0.00056	0.008412	0.000396	0.876442	0.009609	0.00154	0.321934	0.024438
-0.15625	-0.19408	-0.0746	-0.13908	0.01171	-0.14276	-0.23205	-0.04415	0.13073
0.079398	0.028786	0.404508	0.118889	0.896044	0.109339	0.008661	0.622145	0.142925
0.099223	0.177295	-0.05751	0.055018	0.03676	0.182965	0.124531	0.056109	-0.05106
0.267054	0.046142	0.520734	0.538982	0.681583	0.039501	0.163031	0.53095	0.568609
0.523976	0.374865	0.319462	0.43002	0.161797	0.368896	0.028382	-0.19581	-0.40314
2.59E-10	1.41E-05	0.000251	4.52E-07	0.069167	1.98E-05	0.751431	0.02737	2.61E-06
0.04688	0.080346	-0.03791	0.042967	0.097466	0.175642	-0.15343	0.035237	-0.25202
0.600711	0.369213	0.672167	0.631475	0.275659	0.048246	0.085031	0.694099	0.004259
-0.0349	-0.05926	-0.13092	-0.08021	-0.05642	-0.02743	-0.351	0.083441	-0.10992
0.696922	0.508071	0.142344	0.370008	0.528641	0.759526	5.21E-05	0.350997	0.21862
-0.60322	-0.38111	-0.62564	-0.65905	-0.57829	-0.50523	-0.45152	0.57761	0.417816
6.14E-14	9.85E-06	3.72E-15	3.66E-17	1.08E-12	1.38E-09	9.93E-08	1.17E-12	1.02E-06
0.19444	0.179512	0.054166	0.163794	-0.16427	0.163712	0.09792	0.01551	-0.1548
0.028487	0.043443	0.545296	0.065759	0.064963	0.065896	0.273417	0.862593	0.082261
0.015585	-0.01885	-0.02022	0.013443	-0.26459	-0.15291	0.142802	0.057541	0.076345
0.86194	0.833428	0.821465	0.880763	0.002646	0.086106	0.109243	0.520498	0.393601
-0.33229	-0.20637	-0.42784	-0.32957	-0.54531	-0.3206	-0.10994	0.260944	0.257239
0.000135	0.019921	5.24E-07	0.000155	3.38E-11	0.000238	0.218528	0.003045	0.003505
0.479825	0.552905	0.438072	0.452073	0.4252	0.515578	0.568853	-0.23676	-0.31218
1.14E-08	1.58E-11	2.59E-07	9.54E-08	6.26E-07	5.55E-10	3.01E-12	0.007362	0.000353

C16orf87	C16orf89	C17orf53	C18orf54	C19orf48	C19orf57	C1QL4	C1QTNF2	C20orf194
blue	blue	blue	blue	blue	blue	blue	blue	blue
-0.29637	0.345323	-0.32523	-0.40631	-0.36706	-0.35691	-0.40963	-0.13166	0.339088
0.000716	7E-05	0.000191	2.14E-06	2.19E-05	3.81E-05	1.73E-06	0.140068	9.63E-05
-0.32847	0.360822	-0.29725	-0.36786	-0.32846	-0.27535	-0.35813	-0.11873	0.306404
0.000163	3.08E-05	0.000689	2.09E-05	0.000163	0.00173	3.57E-05	0.183672	0.000459
0.632711	-0.67296	0.873349	0.840254	0.684018	0.669439	0.60114	0.436472	-0.64751
1.47E-15	4.47E-18	7.28E-41	4.97E-35	7.75E-19	7.7E-18	7.87E-14	2.9E-07	1.93E-16
0.469074	-0.55019	0.562959	0.682293	0.278755	0.2918	0.342215	0.205808	-0.44263
2.66E-08	2.08E-11	5.62E-12	1.02E-18	0.001506	0.000873	8.22E-05	0.02027	1.88E-07
0.133495	0.129749	0.015234	-0.1458	0.215726	-0.06661	0.014044	0.163246	-0.42766
0.13459	0.145977	0.865019	0.10192	0.014858	0.456868	0.875472	0.06668	5.31E-07
0.360454	-0.21577	0.24916	0.309811	0.121147	-0.03064	0.107708	0.157042	-0.462
3.14E-05	0.014837	0.004731	0.000393	0.174859	0.73236	0.228079	0.077861	4.56E-08
0.078833	-0.08767	0.114497	0.06401	0.006678	-0.09471	-0.06901	-0.09792	-0.15356
0.378321	0.327033	0.199922	0.474638	0.940599	0.289543	0.440755	0.273413	0.084775
-0.18886	0.268473	-0.27138	-0.26228	-0.27259	-0.23321	-0.05263	-0.04314	0.072724
0.033468	0.002274	0.002028	0.002894	0.001932	0.008324	0.556762	0.630074	0.416489
0.154871	-0.33934	0.484093	0.214442	0.29589	0.128798	-0.00286	-0.02511	-0.19269
0.082112	9.51E-05	8.11E-09	0.015479	0.000731	0.148979	0.974506	0.779283	0.029972
-0.20495	0.259934	-0.14495	-0.38064	-0.07264	-0.36174	-0.38167	-0.29455	0.215637
0.020808	0.003165	0.103952	1.01E-05	0.41701	2.93E-05	9.53E-06	0.000775	0.0149
-0.13424	0.141522	-0.09695	-0.20847	-0.12215	-0.30583	-0.19035	-0.07443	0.125722
0.13241	0.11249	0.278201	0.018675	0.171296	0.000471	0.032068	0.405595	0.159011
-0.39503	-0.18906	0.12875	-0.0698	0.057107	0.067081	0.042234	-0.0747	0.164772
4.3E-06	0.033271	0.149132	0.435501	0.523656	0.453655	0.637313	0.403922	0.064141
0.068941	-0.33629	0.490394	0.319836	0.431287	0.523468	0.307601	0.058249	0.041811
0.441205	0.000111	4.85E-09	0.000247	4.15E-07	2.71E-10	0.000435	0.515375	0.640689
-0.0566	0.055469	0.190486	-0.10716	0.529748	0.364783	0.220678	0.171786	-0.11512
0.527344	0.535653	0.031943	0.230475	1.51E-10	2.48E-05	0.012662	0.053463	0.19747
-0.23439	0.126201	0.092737	-0.22136	0.242325	0.085723	-0.0766	-0.18999	0.277766
0.007993	0.157417	0.299736	0.012382	0.006053	0.33793	0.392021	0.032399	0.001568
-0.51686	0.545439	-0.66173	-0.43881	-0.4463	-0.33817	-0.37604	-0.3454	0.572282
4.94E-10	3.34E-11	2.47E-17	2.46E-07	1.45E-07	0.000101	1.32E-05	6.98E-05	2.08E-12
0.034934	-0.08853	0.013162	0.153807	0.106288	0.288184	0.266652	0.148301	-0.01838
0.696599	0.322272	0.883236	0.084263	0.234307	0.001018	0.002443	0.09612	0.837501
0.114225	-0.02573	-0.28337	0.151944	-0.38669	-0.13799	0.017041	0.140152	-0.06208
0.200999	0.774044	0.001245	0.088137	7.1E-06	0.121847	0.849185	0.116047	0.488104
-0.23055	0.27727	-0.54107	-0.30729	-0.47684	-0.23263	-0.16408	-0.03773	0.244571
0.009115	0.0016	5.12E-11	0.000441	1.45E-08	0.00849	0.065284	0.673621	0.005586
0.460927	-0.52999	0.545102	0.535866	0.361829	0.402347	0.216559	0.052174	-0.33646
4.95E-08	1.48E-10	3.45E-11	8.47E-11	2.92E-05	2.74E-06	0.014467	0.560195	0.00011

C21orf58	C2CD2	C4orf46	C5orf34	C5orf67	C6orf106	CACNG1	CACNG4	CAPZA2
blue	blue	blue	blue	blue	blue	blue	blue	blue
-0.46479	0.195708	-0.28255	-0.21897	-0.29463	0.472783	-0.37601	-0.508	-0.24707
3.69E-08	0.027448	0.001288	0.013386	0.000772	1.99E-08	1.32E-05	1.08E-09	0.005105
-0.44983	0.186853	-0.26443	-0.13139	-0.25417	0.459332	-0.32931	-0.49678	-0.2399
1.12E-07	0.035425	0.002663	0.140897	0.003932	5.58E-08	0.000157	2.84E-09	0.006595
0.731193	-0.5222	0.843129	0.739342	0.58073	-0.67063	0.498611	0.521287	0.459402
1.67E-22	3.05E-10	1.75E-35	3.23E-23	8.25E-13	6.42E-18	2.44E-09	3.31E-10	5.55E-08
0.420858	-0.25317	0.771639	0.669511	0.46103	-0.55731	0.233017	0.186851	0.447139
8.36E-07	0.004081	2.51E-26	7.62E-18	4.91E-08	1.01E-11	0.008379	0.035427	1.36E-07
-0.17025	-0.29611	0.179121	0.199255	0.002514	-0.07359	-0.17238	-0.36895	0.290996
0.055667	0.000725	0.043908	0.024713	0.977621	0.410943	0.052631	1.97E-05	0.000903
0.038099	-0.22424	0.586404	0.57612	0.215822	-0.3218	-0.07868	-0.24673	0.458476
0.670647	0.011263	4.37E-13	1.37E-12	0.014812	0.000225	0.379224	0.005168	5.94E-08
-0.10729	0.027002	0.236135	0.314174	0.165399	-0.12837	-0.03935	-0.21886	0.182287
0.229902	0.763151	0.007524	0.000322	0.063121	0.150342	0.660518	0.013432	0.040249
-0.09281	0.158223	-0.19182	-0.15127	-0.18244	0.130285	-0.33626	-0.32725	0.015343
0.299332	0.075625	0.030742	0.089573	0.040076	0.144304	0.000111	0.000173	0.864065
0.232141	0.225719	0.221738	0.160635	0.239786	-0.28772	-0.06955	-0.00867	0.038309
0.008634	0.010723	0.012231	0.071216	0.006622	0.001038	0.437198	0.922904	0.668935
-0.27747	0.441975	-0.25823	-0.36941	-0.22107	0.142351	-0.51028	-0.57086	-0.16385
0.001587	1.97E-07	0.003376	1.92E-05	0.012499	0.110378	8.88E-10	2.43E-12	0.065668
-0.14467	0.196392	-0.06845	-0.13625	-0.15621	0.157606	-0.39015	-0.41088	-0.10772
0.104647	0.026902	0.44445	0.126647	0.079478	0.076787	5.78E-06	1.6E-06	0.228045
0.030537	0.199786	-0.13127	-0.2006	0.114544	-0.04701	0.221563	0.273458	-0.27262
0.733244	0.024324	0.14127	0.023738	0.199736	0.599741	0.012301	0.001866	0.00193
0.540569	-0.03284	0.091662	-0.06089	0.271386	-0.28176	0.302221	0.45628	-0.16133
5.38E-11	0.713996	0.305398	0.496483	0.002027	0.001331	0.000554	7E-08	0.069985
0.286571	-0.17163	-0.14458	-0.33086	0.001295	-0.13835	-0.03972	0.055125	-0.12111
0.001089	0.053681	0.104852	0.000145	0.988475	0.120848	0.657476	0.538188	0.175007
0.141807	0.315367	-0.33715	-0.54263	-0.10824	0.007774	-0.24021	-0.12839	-0.37785
0.11176	0.000304	0.000106	4.4E-11	0.225763	0.930875	0.006525	0.150281	1.19E-05
-0.44954	0.294704	-0.6066	-0.60979	-0.4105	0.451857	-0.11231	-0.19295	-0.43539
1.15E-07	0.00077	4.08E-14	2.76E-14	1.64E-06	9.69E-08	0.208684	0.029754	3.13E-07
0.157696	-0.30323	-0.04358	-0.02476	0.105112	-0.06647	0.556802	0.649006	-0.02255
0.076617	0.00053	0.626646	0.782276	0.239555	0.457808	1.06E-11	1.56E-16	0.801346
-0.16879	-0.23613	0.129638	0.306061	-0.0116	0.132438	0.287446	0.259727	0.244795
0.057834	0.007525	0.146325	0.000466	0.89698	0.137732	0.00105	0.00319	0.005541
-0.37381	-0.0324	-0.41823	-0.16262	-0.26548	0.460896	0.15916	0.197613	-0.11394
1.5E-05	0.717623	9.94E-07	0.067751	0.002557	4.96E-08	0.073885	0.025949	0.202134
0.278653	-0.28882	0.484113	0.452292	0.464225	-0.48882	0.513916	0.451378	0.33046
0.001512	0.000991	8.1E-09	9.39E-08	3.86E-08	5.51E-09	6.43E-10	1E-07	0.000148

CARHSP1	CASK	CASP2	CASP3	CASQ2	CBX1	CBX2	CBX3	CCDC138
blue	blue	blue	blue	blue	blue	blue	blue	blue
-0.26758	-0.49454	-0.3784	-0.40882	0.242299	-0.15812	-0.36074	-0.18684	-0.28241
0.002355	3.43E-09	1.15E-05	1.83E-06	0.006059	0.075816	3.09E-05	0.035441	0.001296
-0.21978	-0.47121	-0.40792	-0.39084	0.280113	-0.10594	-0.30421	-0.15373	-0.26771
0.013037	2.25E-08	1.93E-06	5.55E-06	0.001425	0.235859	0.000507	0.084418	0.002343
0.575996	0.730073	0.831305	0.655096	-0.4979	0.460712	0.817662	0.76003	0.68703
1.39E-12	2.08E-22	1.12E-33	6.52E-17	2.59E-09	5.03E-08	9.18E-32	3.75E-25	4.74E-19
0.270892	0.397264	0.721347	0.734321	-0.26103	0.080075	0.355067	0.775085	0.72949
0.002067	3.76E-06	1.12E-21	8.95E-23	0.003035	0.370833	4.2E-05	1.09E-26	2.33E-22
0.125584	-0.40061	-0.35649	0.213558	0.124152	-0.20219	-0.14122	0.282045	0.242337
0.159474	3.06E-06	3.89E-05	0.015919	0.164325	0.022627	0.113266	0.001316	0.00605
0.112982	-0.1273	0.218196	0.681885	0.019571	-0.23537	-0.04976	0.707647	0.655967
0.205978	0.153792	0.013724	1.09E-18	0.827125	0.007726	0.578515	1.39E-20	5.75E-17
0.12239	-0.22106	0.096618	0.279281	0.201823	-0.25857	-0.12759	0.366103	0.165687
0.170442	0.012503	0.279877	0.001474	0.022882	0.003334	0.152872	2.31E-05	0.062655
-0.33224	-0.17557	-0.09687	0.057159	0.388798	-0.17744	-0.32659	-0.00051	0.232615
0.000136	0.04834	0.278596	0.523275	6.26E-06	0.045965	0.000179	0.995479	0.008495
0.232033	0.108499	0.644968	0.401741	-0.2245	0.006632	0.157121	0.37145	0.025439
0.008666	0.224664	2.75E-16	2.85E-06	0.011166	0.941009	0.077709	1.71E-05	0.776492
-0.17955	-0.52831	-0.04359	0.042218	0.157127	-0.40424	-0.45416	-0.00333	-0.27979
0.043401	1.73E-10	0.626558	0.637439	0.077698	2.44E-06	8.18E-08	0.970396	0.001443
-0.13673	-0.30735	0.098327	0.126858	0.076176	-0.35465	-0.34201	0.148995	-0.0275
0.125315	0.00044	0.27142	0.155249	0.394655	4.3E-05	8.3E-05	0.094557	0.75892
0.345178	0.146027	0.21826	-0.28455	-0.2387	0.191666	0.104316	-0.21995	-0.30401
7.06E-05	0.101389	0.013696	0.001186	0.006879	0.030876	0.243154	0.012967	0.000511
0.352962	0.601746	0.574403	-0.11344	-0.40451	0.508344	0.600293	-0.03527	-0.13618
4.7E-05	7.32E-14	1.66E-12	0.204151	2.4E-06	1.05E-09	8.7E-14	0.693847	0.12685
0.265952	0.090737	-0.02556	-0.1868	-0.23024	0.169247	0.242363	-0.2445	-0.25268
0.00251	0.310326	0.775495	0.035483	0.009211	0.057145	0.006045	0.0056	0.004157
0.08656	-0.02924	0.174441	-0.23886	-0.10605	0.078184	0.026814	-0.28381	-0.49649
0.333215	0.744133	0.049823	0.006841	0.235357	0.382275	0.76475	0.001223	2.91E-09
-0.33508	-0.34034	-0.64334	-0.64365	0.402624	-0.02343	-0.38181	-0.65288	-0.56371
0.000118	9.04E-05	3.45E-16	3.31E-16	2.7E-06	0.793764	9.45E-06	8.98E-17	5.19E-12
0.126911	0.525566	-0.0938	-0.30895	-0.15451	0.432251	0.418092	-0.27369	-0.08581
0.155076	2.23E-10	0.294203	0.000409	0.082839	3.88E-07	1E-06	0.001849	0.337432
-0.25268	0.084795	-0.28601	0.022614	0.112793	-0.05729	-0.05693	-0.03893	0.277682
0.004156	0.343204	0.001115	0.800763	0.20674	0.522346	0.524934	0.663871	0.001573
-0.34271	-0.08333	-0.64519	-0.50944	0.223582	0.000662	-0.19567	-0.52995	-0.27806
8.01E-05	0.351622	2.67E-16	9.56E-10	0.011511	0.994106	0.027476	1.48E-10	0.00155
0.44615	0.4273	0.329805	0.054964	-0.31312	0.463186	0.65593	0.256885	0.219867
1.46E-07	5.44E-07	0.000153	0.53938	0.000338	4.17E-08	5.78E-17	0.003552	0.013001

CCDC14	CCDC140	CCDC15	CCDC150	CCDC168	CCDC18	CCDC28B	CCDC82	CCL28
blue	blue	blue	blue	blue	blue	blue	blue	blue
-0.38521	-0.11252	-0.34695	-0.40909	-0.24404	-0.24597	-0.26909	-0.15244	0.368838
7.75E-06	0.207852	6.44E-05	1.79E-06	0.005692	0.005312	0.00222	0.087099	1.98E-05
-0.41385	-0.15408	-0.30359	-0.36307	-0.20251	-0.30557	-0.1897	-0.14454	0.432885
1.32E-06	0.083716	0.000521	2.73E-05	0.022408	0.000477	0.03267	0.104954	3.72E-07
0.712036	0.46157	0.780906	0.855588	0.562244	0.730777	0.499521	0.441646	-0.52705
6.31E-21	4.71E-08	2.58E-27	1.49E-37	6.05E-12	1.81E-22	2.25E-09	2.02E-07	1.95E-10
0.769507	0.441483	0.709956	0.676407	0.390178	0.743711	-0.0191	0.445074	-0.3132
4.18E-26	2.04E-07	9.2E-21	2.61E-18	5.77E-06	1.31E-23	0.831263	1.58E-07	0.000337
-0.19394	0.17091	0.1881	-0.08321	-0.24425	-0.02058	0.326498	0.191577	0.173735
0.028904	0.054709	0.034196	0.352352	0.005652	0.818324	0.000179	0.030956	0.05077
0.39972	0.39092	0.552126	0.305858	-0.00247	0.494505	-0.06191	0.429588	-0.01228
3.23E-06	5.52E-06	1.71E-11	0.000471	0.97804	3.44E-09	0.48929	4.66E-07	0.890989
0.189596	0.100109	0.221804	0.138331	0.038735	0.225547	-0.14665	0.063567	0.071046
0.032768	0.262784	0.012204	0.120911	0.665472	0.010785	0.099922	0.477706	0.42735
0.164574	-0.18038	0.014596	-0.15902	-0.2665	0.027113	-0.4214	0.041943	0.045888
0.064466	0.042419	0.870619	0.074145	0.002457	0.762206	8.07E-07	0.639635	0.608448
0.293874	0.286165	0.237683	0.326461	0.168299	0.4621	-0.15993	-0.11122	-0.39184
0.000798	0.001108	0.007129	0.00018	0.058572	4.53E-08	0.07249	0.213188	5.22E-06
-0.10221	-0.10271	-0.10293	-0.23251	-0.3355	0.119835	-0.45403	-0.13488	-0.06305
0.252834	0.250501	0.24952	0.008525	0.000115	0.179608	8.26E-08	0.130552	0.481321
0.147186	0.132676	-0.00452	-0.15249	-0.20481	0.203489	-0.44165	-0.09128	-0.02496
0.098676	0.13702	0.959802	0.086988	0.020897	0.021757	2.02E-07	0.30743	0.780604
-0.02761	0.098866	-0.21393	0.038618	0.167464	-0.12986	-0.00209	-0.35729	-0.07154
0.758002	0.268786	0.015734	0.666427	0.059852	0.145614	0.981362	3.73E-05	0.424166
0.293378	-0.02508	0.137197	0.435767	0.472911	0.232762	0.265751	-0.11946	-0.4424
0.000815	0.779575	0.124018	3.05E-07	1.97E-08	0.008453	0.00253	0.180989	1.91E-07
-0.21071	-0.03998	-0.05469	-0.0102	-0.00958	-0.18712	0.494831	-0.25533	-0.22783
0.017411	0.655413	0.541378	0.909408	0.914858	0.035156	3.35E-09	0.003766	0.009992
-0.08298	-0.19787	-0.15434	-0.0429	-0.03436	0.008827	-0.04674	-0.34154	-0.29272
0.353664	0.025753	0.083178	0.63203	0.701316	0.921543	0.601765	8.5E-05	0.000839
-0.52355	-0.51215	-0.5749	-0.52606	-0.34113	-0.58636	-0.14608	-0.1882	0.345933
2.69E-10	7.53E-10	1.57E-12	2.13E-10	8.68E-05	4.39E-13	0.101273	0.034098	6.79E-05
-0.10742	-0.13814	-0.14702	0.061785	0.219305	-0.28814	0.50299	-0.03442	-0.05446
0.229345	0.12143	0.099055	0.490156	0.01324	0.00102	1.68E-09	0.700843	0.543086
-0.05833	0.041674	-0.0921	-0.13253	0.004352	-0.2326	0.009945	0.221813	0.320729
0.514792	0.641787	0.303087	0.137449	0.961274	0.008499	0.911638	0.012201	0.000237
-0.51158	-0.2728	-0.54253	-0.48972	-0.15487	-0.66366	0.028642	-0.14555	0.426159
7.92E-10	0.001916	4.44E-11	5.12E-09	0.082123	1.85E-17	0.749235	0.102525	5.87E-07
0.209077	0.118746	0.336392	0.544465	0.473428	0.378095	0.597528	0.436527	-0.21455
0.018323	0.183623	0.00011	3.67E-11	1.9E-08	1.17E-05	1.21E-13	2.89E-07	0.015425

CCNA2	CCNB1	CCNB2	CCND3	CCNE1	CCNE2	CCNF	CCNJ	CCNJL
blue	blue	blue	blue	blue	blue	blue	blue	blue
-0.45605	-0.44775	-0.4011	0.18271	-0.31817	-0.20678	-0.43274	-0.19723	-0.38967
7.12E-08	1.31E-07	2.97E-06	0.03978	0.000267	0.019676	3.75E-07	0.026243	5.95E-06
-0.44473	-0.39913	-0.34018	0.255546	-0.27401	-0.15595	-0.37634	-0.22899	-0.3967
1.62E-07	3.35E-06	9.11E-05	0.003735	0.001825	0.079984	1.3E-05	0.00961	3.89E-06
0.934211	0.880503	0.877917	-0.65645	0.700167	0.529388	0.708641	0.686979	0.818807
8.37E-58	2.42E-42	8.49E-42	5.36E-17	5.18E-20	1.56E-10	1.16E-20	4.78E-19	6.43E-32
0.699576	0.567936	0.49164	-0.62342	0.584344	0.441148	0.23363	0.622155	0.549997
5.74E-20	3.32E-12	4.37E-09	4.96E-15	5.51E-13	2.09E-07	0.008204	5.84E-15	2.12E-11
-0.08895	0.049306	0.023861	0.322463	0.084889	-0.09071	-0.21214	-0.22125	-0.01007
0.319992	0.581983	0.790025	0.000218	0.34267	0.310452	0.016648	0.012429	0.910539
0.342285	0.306457	0.205289	-0.17077	0.411481	0.244243	-0.16245	0.257439	0.238562
8.19E-05	0.000458	0.020595	0.054907	1.54E-06	0.005652	0.068033	0.003479	0.006913
0.031188	0.022293	-0.03258	-0.00952	0.232387	-0.01626	-0.20673	0.136468	0.092673
0.727781	0.803537	0.716171	0.915381	0.008562	0.856054	0.019706	0.126047	0.300072
-0.19126	-0.23141	-0.29206	0.088436	-0.142	-0.07156	-0.40768	-0.27974	-0.30829
0.03124	0.008853	0.000863	0.322808	0.111281	0.424031	1.96E-06	0.001446	0.000422
0.342009	0.264592	0.308306	-0.23674	0.249704	0.04132	0.155445	0.403802	0.386439
8.3E-05	0.002646	0.000421	0.007369	0.004637	0.644624	0.080972	2.51E-06	7.21E-06
-0.29204	-0.42243	-0.30839	0.247566	-0.27009	-0.17497	-0.58585	-0.04634	-0.32832
0.000864	7.53E-07	0.00042	0.005014	0.002133	0.049128	4.65E-13	0.604922	0.000164
-0.10137	-0.25008	-0.18797	0.048484	-0.10814	-0.018	-0.41129	0.079634	-0.18028
0.256783	0.004574	0.034324	0.5883	0.226229	0.840772	1.56E-06	0.37348	0.042541
-0.03374	0.00466	0.037215	-0.08596	-0.10035	0.061414	0.164673	0.123809	0.176994
0.706502	0.95853	0.677855	0.336576	0.261613	0.492773	0.064303	0.165502	0.04652
0.34389	0.258753	0.387694	-0.41721	0.089011	0.126526	0.5195	0.369876	0.394282
7.54E-05	0.00331	6.69E-06	1.06E-06	0.319655	0.156342	3.89E-10	1.87E-05	4.51E-06
-0.04569	-0.0064	0.129477	0.152478	-0.15431	-0.10269	0.232494	-0.13426	0.080986
0.609976	0.943051	0.146829	0.087013	0.083248	0.250618	0.008531	0.132344	0.365394
-0.15561	-0.27598	-0.07705	0.100528	-0.32375	-0.12482	-0.09469	0.016471	-0.09737
0.080644	0.001686	0.389226	0.260781	0.000205	0.162049	0.289623	0.854171	0.276119
-0.61079	-0.62048	-0.59469	0.452574	-0.57556	-0.05575	-0.43441	-0.459	-0.6891
2.44E-14	7.23E-15	1.68E-13	9.19E-08	1.46E-12	0.533601	3.35E-07	5.72E-08	3.37E-19
0.094847	0.194223	0.193599	-0.13987	-0.01823	0.09784	0.472894	-0.02158	0.110073
0.288826	0.028668	0.029194	0.116794	0.838804	0.273811	1.98E-08	0.809667	0.217972
0.042946	0.140744	-0.05292	-0.05313	0.181447	0.093286	0.155801	-0.11652	-0.04275
0.631644	0.114498	0.554609	0.553019	0.041194	0.296875	0.08027	0.192047	0.633216
-0.39345	-0.20363	-0.30129	0.292699	-0.22398	-0.24978	0.054648	-0.47122	-0.34887
4.74E-06	0.021663	0.000577	0.00084	0.011362	0.004625	0.541717	2.25E-08	5.82E-05
0.457563	0.513321	0.531316	-0.40214	0.321545	0.151879	0.528162	0.422168	0.589352
6.36E-08	6.78E-10	1.3E-10	2.78E-06	0.000228	0.088274	1.75E-10	7.67E-07	3.12E-13

CCT2	CCT3	CCT5	CCT6A	CCT7	CD101	CD207	CDC20	CDC25A
blue	blue	blue	blue	blue	blue	blue	blue	blue
-0.30307	-0.30477	-0.22968	-0.23173	-0.40641	-0.36874	-0.2687	-0.45121	-0.48846
0.000533	0.000494	0.009387	0.008756	2.13E-06	1.99E-05	0.002254	1.02E-07	5.68E-09
-0.26813	-0.19812	-0.17521	-0.19945	-0.3302	-0.40816	-0.28642	-0.40034	-0.41513
0.002305	0.025563	0.048805	0.024566	0.00015	1.9E-06	0.001096	3.11E-06	1.22E-06
0.693004	0.534053	0.712342	0.66592	0.478845	0.738932	0.638542	0.892517	0.912788
1.76E-19	1.01E-10	5.97E-21	1.32E-17	1.24E-08	3.52E-23	6.68E-16	4.73E-45	1.91E-50
0.679875	0.266932	0.422209	0.73769	0.065803	0.702492	0.414325	0.518693	0.549184
1.51E-18	0.002416	7.65E-07	4.53E-23	0.462326	3.46E-20	1.28E-06	4.19E-10	2.3E-11
0.366514	0.427288	0.014879	0.070479	0.292616	-0.16875	0.043167	0.028438	-0.04321
2.26E-05	5.44E-07	0.868135	0.431055	0.000843	0.057883	0.629887	0.750958	0.629514
0.682153	0.289567	0.180346	0.586346	0.013874	0.357181	0.184855	0.226023	0.200175
1.05E-18	0.00096	0.042462	4.39E-13	0.876967	3.75E-05	0.037472	0.010615	0.024043
0.338838	0.200423	0.09497	0.36661	-0.02436	0.132138	0.074484	0.017081	0.003905
9.75E-05	0.023865	0.288198	2.24E-05	0.785728	0.138633	0.40527	0.848834	0.965244
0.021437	-0.03639	-0.27541	0.029911	-0.23047	-0.12992	-0.02867	-0.29519	-0.23608
0.810936	0.684622	0.001725	0.738517	0.009139	0.145452	0.748992	0.000754	0.007538
0.143767	-0.10894	0.316067	0.38244	3.25E-05	0.370094	0.094589	0.309835	0.198387
0.106845	0.222788	0.000295	9.12E-06	0.99971	1.85E-05	0.290149	0.000393	0.02536
-0.24202	-0.32587	-0.2949	-0.06224	-0.36832	-0.18294	-0.23717	-0.28293	-0.41539
0.006119	0.000185	0.000764	0.486961	2.04E-05	0.039525	0.007257	0.001268	1.2E-06
-0.06208	-0.27231	-0.24131	0.123781	-0.39037	0.093584	-0.19414	-0.19118	-0.24178
0.488058	0.001954	0.006275	0.165599	5.7E-06	0.295327	0.028738	0.031316	0.006172
-0.32365	-0.08744	0.259723	-0.21325	0.101301	0.132106	-0.04025	0.008591	-0.02537
0.000206	0.328292	0.00319	0.016074	0.257115	0.13873	0.653227	0.923628	0.777041
-0.19857	0.027974	0.336014	-0.00782	0.211054	0.258989	0.311137	0.410903	0.418491
0.025223	0.754895	0.000112	0.930439	0.017226	0.003281	0.00037	1.6E-06	9.77E-07
-0.2635	0.131965	-0.02771	-0.3084	0.361044	-0.15174	0.059229	0.197321	0.085071
0.00276	0.139157	0.757171	0.000419	3.04E-05	0.088563	0.508315	0.026174	0.341633
-0.53291	-0.26275	-0.12512	-0.28632	-0.08804	-0.15335	-0.06761	-0.01136	-0.13953
1.12E-10	0.002842	0.161024	0.001101	0.325007	0.08521	0.450094	0.899132	0.11769
-0.61111	-0.20582	-0.40788	-0.54611	-0.21017	-0.64295	-0.38197	-0.51223	-0.50673
2.35E-14	0.020263	1.94E-06	3.12E-11	0.017712	3.64E-16	9.37E-06	7.47E-10	1.21E-09
-0.08723	0.27862	0.188485	-0.19493	0.381077	-0.00024	0.165754	0.177875	0.293288
0.329453	0.001514	0.033823	0.028081	9.87E-06	0.997877	0.062548	0.045423	0.000819
0.270093	0.05478	-0.04022	0.064558	-0.01367	0.046018	-0.07203	-0.11009	0.040986
0.002134	0.540741	0.653458	0.470856	0.878802	0.607437	0.420976	0.217881	0.647303
-0.22425	-0.07573	-0.21761	-0.41752	0.01466	-0.40053	-0.27038	-0.39111	-0.26302
0.011258	0.397408	0.013987	1.04E-06	0.870059	3.07E-06	0.002109	5.46E-06	0.002812
0.291624	0.383306	0.671134	0.138851	0.469681	0.236188	0.576334	0.495721	0.528443
0.000879	8.67E-06	5.94E-18	0.119506	2.54E-08	0.007511	1.34E-12	3.11E-09	1.71E-10

CDC25C	CDC45	CDC6	CDC7	CDCA2	CDCA3	CDCA4	CDCA5	CDCA7
blue	blue	blue	blue	blue	blue	blue	blue	blue
-0.47056	-0.45071	-0.34502	-0.29661	-0.44196	-0.35452	-0.27505	-0.46691	-0.52665
2.37E-08	1.05E-07	7.11E-05	0.000709	1.97E-07	4.32E-05	0.00175	3.14E-08	2.02E-10
-0.4541	-0.45638	-0.32196	-0.24942	-0.46319	-0.28454	-0.30193	-0.40242	-0.53094
8.22E-08	6.95E-08	0.000223	0.004686	4.17E-08	0.001186	0.000561	2.73E-06	1.35E-10
0.902026	0.920223	0.893972	0.72254	0.933221	0.820075	0.802766	0.865318	0.863651
1.96E-47	9.23E-53	2.11E-45	8.94E-22	2.06E-57	4.32E-32	7.56E-30	2.62E-39	5.35E-39
0.668519	0.615431	0.660458	0.50455	0.677408	0.474979	0.606409	0.421666	0.66084
8.87E-18	1.37E-14	2.98E-17	1.46E-09	2.23E-18	1.68E-08	4.17E-14	7.93E-07	2.81E-17
0.066858	-0.02271	-0.05613	0.033166	-0.07337	0.092824	0.248529	-0.03935	-0.16393
0.45516	0.799912	0.530808	0.711261	0.412343	0.299284	0.004841	0.660522	0.065529
0.417063	0.28051	0.325387	0.295335	0.324936	0.261933	0.468378	0.092435	0.246487
1.07E-06	0.001402	0.000189	0.000749	0.000194	0.002932	2.81E-08	0.301322	0.005214
0.089089	0.04729	0.114612	0.037544	0.050874	-0.00285	0.186119	-0.08447	-0.06812
0.319232	0.597527	0.199465	0.675171	0.570021	0.974664	0.036166	0.34505	0.446706
-0.15454	-0.21164	-0.2391	-0.02955	-0.21357	-0.294	-0.23149	-0.37637	-0.03079
0.082777	0.01691	0.006783	0.741521	0.015914	0.000794	0.008828	1.29E-05	0.731104
0.312217	0.430736	0.397533	0.01538	0.454395	0.193121	0.476133	0.213017	0.33812
0.000352	4.31E-07	3.69E-06	0.863737	8.04E-08	0.029603	1.53E-08	0.016194	0.000101
-0.27601	-0.19983	-0.31198	-0.36225	-0.18631	-0.30611	-0.00517	-0.41113	-0.2301
0.001684	0.024294	0.000356	2.85E-05	0.03597	0.000465	0.953989	1.57E-06	0.009254
-0.07596	-0.07385	-0.1514	-0.19274	-0.03081	-0.15943	0.102067	-0.26929	-0.01386
0.395983	0.409294	0.089296	0.029928	0.730933	0.073397	0.253518	0.002202	0.87705
-0.10063	0.055616	0.028155	-0.20409	0.038877	-0.02157	0.082351	0.009574	0.049209
0.260276	0.53457	0.753357	0.021366	0.664326	0.809747	0.357347	0.914926	0.58273
0.228789	0.426065	0.343184	0.142684	0.397887	0.285863	0.228727	0.443462	0.446351
0.009675	5.91E-07	7.82E-05	0.109539	3.62E-06	0.001122	0.009695	1.77E-07	1.44E-07
-0.01801	0.103578	-0.04955	-0.15129	0.017135	0.050076	0.167508	0.206358	0.120865
0.840682	0.246519	0.580093	0.08952	0.848365	0.576093	0.059785	0.019931	0.175872
-0.21289	-0.01394	-0.18879	-0.32725	-0.04486	-0.16408	0.003681	-0.07254	0.007898
0.01626	0.876377	0.033526	0.000173	0.616491	0.065289	0.967241	0.417701	0.929776
-0.69748	-0.65933	-0.68511	-0.35252	-0.69383	-0.52841	-0.7008	-0.48571	-0.63639
8.23E-20	3.51E-17	6.49E-19	4.81E-05	1.53E-19	1.71E-10	4.65E-20	7.11E-09	8.94E-16
0.037242	0.077893	0.055295	0.208801	0.028939	0.162626	-0.16478	0.339065	0.096541
0.677634	0.38405	0.536935	0.018481	0.746721	0.067736	0.064123	9.64E-05	0.280261
0.051284	-0.14958	0.041197	0.230598	-0.10143	0.010036	-0.24686	0.025753	-0.07848
0.566914	0.093244	0.645607	0.0091	0.256519	0.910836	0.005144	0.773806	0.380471
-0.37384	-0.46567	-0.34851	-0.10682	-0.48643	-0.25424	-0.60719	-0.20985	-0.46774
1.5E-05	3.45E-08	5.94E-05	0.231977	6.71E-09	0.003922	3.79E-14	0.01789	2.95E-08
0.382756	0.483236	0.487175	0.457162	0.466834	0.457844	0.410734	0.53207	0.243271
8.95E-06	8.69E-09	6.31E-09	6.55E-08	3.16E-08	6.23E-08	1.62E-06	1.22E-10	0.005852

CDCA7L	CDCA8	CDH24	CDK1	CDK2	CDK4	CDK6	CDK8	CDKN2C
blue	blue	blue	blue	blue	blue	blue	blue	blue
-0.33515	-0.49569	-0.48034	-0.44551	-0.32467	-0.18543	-0.22997	-0.21015	-0.33203
0.000117	3.12E-09	1.1E-08	1.53E-07	0.000196	0.036875	0.009296	0.017719	0.000137
-0.30231	-0.45235	-0.43827	-0.42777	-0.35069	-0.16706	-0.22127	-0.19575	-0.34894
0.000552	9.34E-08	2.56E-07	5.27E-07	5.3E-05	0.060488	0.01242	0.027413	5.81E-05
0.761927	0.926467	0.724844	0.904219	0.86621	0.575748	0.774551	0.49328	0.761138
2.44E-25	6.88E-55	5.75E-22	5.1E-48	1.78E-39	1.43E-12	1.24E-26	3.81E-09	2.92E-25
0.709512	0.584784	0.312816	0.646134	0.66827	0.276012	0.713038	0.493307	0.544889
9.96E-21	5.24E-13	0.000343	2.34E-16	9.21E-18	0.001684	5.26E-21	3.8E-09	3.52E-11
-0.06676	-0.03484	-0.14984	0.031508	-0.08903	0.227886	-0.13413	0.197358	0.072735
0.455805	0.697343	0.092682	0.725095	0.319555	0.009973	0.132734	0.026146	0.416414
0.394795	0.249181	-0.07916	0.379076	0.32518	0.191433	0.341743	0.438873	0.331239
4.37E-06	0.004727	0.376337	1.11E-05	0.000191	0.031084	8.42E-05	2.45E-07	0.000142
0.210099	-0.01449	-0.17341	0.040764	0.125103	0.100197	0.197445	0.369149	-0.00861
0.017749	0.871512	0.051211	0.649087	0.16109	0.262362	0.026078	1.95E-05	0.923455
-0.11793	-0.25438	-0.34651	-0.17452	-0.10963	-0.21113	-0.3328	-0.06579	-0.22797
0.186684	0.003902	6.59E-05	0.049715	0.219847	0.017185	0.000132	0.462384	0.009947
0.353522	0.338972	0.03532	0.326575	0.604612	0.304946	0.348823	-0.07816	0.256904
4.56E-05	9.69E-05	0.693417	0.000179	5.19E-14	0.00049	5.84E-05	0.382395	0.00355
-0.17227	-0.31351	-0.53705	-0.26609	0.014355	0.033825	-0.28289	-0.30646	-0.15872
0.052787	0.000332	7.56E-11	0.002496	0.872739	0.70578	0.001271	0.000458	0.074691
0.031321	-0.17061	-0.34473	-0.10882	0.125916	0.031119	-0.04328	-0.17678	-0.01847
0.726663	0.055141	7.22E-05	0.223303	0.158363	0.728355	0.628989	0.046794	0.836689
0.090045	0.015995	0.175513	-0.17121	0.075321	0.240792	0.149364	-0.36071	-0.1056
0.314047	0.858345	0.048413	0.054273	0.399993	0.006391	0.093735	3.1E-05	0.237388
0.286515	0.406979	0.524276	0.252901	0.456985	0.300922	0.326403	-0.10547	0.25621
0.001092	2.05E-06	2.52E-10	0.004122	6.64E-08	0.000587	0.00018	0.237941	0.003643
-0.04306	0.115638	0.291307	-0.00503	0.096282	0.22429	-0.12061	-0.3496	0.175075
0.63073	0.195445	0.000891	0.955284	0.281563	0.011245	0.176797	5.61E-05	0.048985
-0.08127	-0.07034	-0.04051	-0.18606	0.149409	0.146749	-0.16392	-0.5145	-0.01907
0.363712	0.431962	0.651158	0.036228	0.093634	0.099691	0.06555	6.11E-10	0.831487
-0.53718	-0.59747	-0.39266	-0.67258	-0.72128	-0.3646	-0.51429	-0.36649	-0.4656
7.46E-11	1.22E-13	4.97E-06	4.75E-18	1.13E-21	2.51E-05	6.22E-10	2.26E-05	3.47E-08
-0.0005	0.165604	0.482515	0.058601	-0.13615	0.037997	0.086367	0.086602	0.050924
0.995553	0.062789	9.22E-09	0.512832	0.12694	0.671479	0.334301	0.332983	0.56964
-0.06714	-0.03737	0.071107	0.049316	-0.33763	-0.37501	0.046877	0.354028	-0.08655
0.453273	0.676612	0.426953	0.581902	0.000104	1.4E-05	0.600738	4.44E-05	0.333294
-0.50304	-0.37498	-0.08087	-0.36021	-0.6649	-0.42881	-0.40388	0.011668	-0.45507
1.67E-09	1.4E-05	0.366058	3.19E-05	1.53E-17	4.91E-07	2.49E-06	0.896408	7.65E-08
0.249596	0.469654	0.455599	0.414019	0.340376	0.395478	0.485356	0.396495	0.248945
0.004656	2.54E-08	7.36E-08	1.31E-06	9.02E-05	4.19E-06	7.32E-09	3.94E-06	0.004768

CDKN3	CDT1	CENPA	CENPE	CENPF	CENPH	CENPI	CENPJ	CENPK
blue	blue	blue	blue	blue	blue	blue	blue	blue
-0.33326	-0.50345	-0.43659	-0.5146	-0.48473	-0.36263	-0.37332	-0.31726	-0.48459
0.000129	1.61E-09	2.88E-07	6.06E-10	7.71E-09	2.79E-05	1.54E-05	0.000279	7.79E-09
-0.27562	-0.51146	-0.39927	-0.53241	-0.46851	-0.34378	-0.37703	-0.29771	-0.51195
0.001711	8E-10	3.32E-06	1.18E-10	2.78E-08	7.58E-05	1.25E-05	0.000676	7.66E-10
0.776651	0.71916	0.900636	0.919264	0.885132	0.927767	0.895534	0.672887	0.906263
7.44E-27	1.69E-21	4.52E-47	1.89E-52	2.38E-43	2.35E-55	8.79E-46	4.53E-18	1.41E-48
0.512724	0.229271	0.64168	0.713962	0.531222	0.653462	0.759193	0.43933	0.76586
7.15E-10	0.009519	4.35E-16	4.44E-21	1.32E-10	8.26E-17	4.53E-25	2.38E-07	9.84E-26
0.316341	-0.07789	0.106789	-0.15412	-0.30771	0.053268	-0.10325	-0.10414	-0.00727
0.000291	0.384062	0.2321	0.083631	0.000433	0.551987	0.248004	0.243936	0.935351
0.447477	-0.09213	0.412459	0.319812	0.034628	0.372886	0.410437	0.107565	0.461467
1.33E-07	0.302918	1.45E-06	0.000247	0.699127	1.58E-05	1.65E-06	0.228701	4.75E-08
0.153731	-0.28216	0.074114	0.02274	-0.15251	0.058764	0.114998	0.116231	0.092067
0.084419	0.001309	0.407613	0.79968	0.086945	0.511657	0.197948	0.193148	0.303255
-0.20945	-0.28504	-0.17936	-0.20512	-0.17314	-0.19713	-0.03982	-0.30336	-0.082
0.018113	0.001162	0.043621	0.020704	0.05158	0.026319	0.656702	0.000526	0.359389
0.23976	0.242388	0.355199	0.445081	0.333313	0.315275	0.388311	0.07872	0.459648
0.006628	0.006039	4.17E-05	1.58E-07	0.000129	0.000306	6.45E-06	0.37901	5.45E-08
-0.29865	-0.23593	-0.17884	-0.21984	-0.35873	-0.2884	-0.18544	-0.44303	-0.1388
0.000649	0.007577	0.044245	0.013012	3.45E-05	0.001009	0.036862	1.83E-07	0.119654
-0.15529	-0.21716	-0.06408	-0.09021	-0.19266	-0.09654	0.032147	-0.34342	-0.00181
0.081282	0.014189	0.47415	0.313178	0.030006	0.280261	0.719752	7.73E-05	0.983917
-0.17339	0.047467	-0.08385	-0.08484	0.072525	-0.01358	-0.14205	0.02425	-0.11263
0.051239	0.596158	0.348623	0.342924	0.417764	0.879515	0.111152	0.786682	0.207397
0.068868	0.575674	0.264673	0.37557	0.594134	0.305509	0.302252	0.340958	0.267224
0.441689	1.44E-12	0.002638	1.36E-05	1.8E-13	0.000478	0.000553	8.76E-05	0.002389
0.042489	0.563036	0.083962	-0.02544	0.110933	0.000623	-0.1258	-0.167	-0.02323
0.63528	5.57E-12	0.347983	0.776464	0.214376	0.994453	0.158759	0.060569	0.795484
-0.30146	0.276877	-0.09051	-0.07857	0.043482	-0.17765	-0.13872	-0.2567	-0.10948
0.000573	0.001626	0.311566	0.379918	0.627393	0.045704	0.11985	0.003577	0.220458
-0.67602	-0.39537	-0.63688	-0.65417	-0.50875	-0.63938	-0.66642	-0.30354	-0.75128
2.78E-18	4.22E-06	8.38E-16	7.46E-17	1.02E-09	5.96E-16	1.22E-17	0.000522	2.61E-24
0.009721	0.283972	0.011967	0.040502	0.281621	0.090889	-0.04178	0.349507	-0.08976
0.91362	0.001215	0.893776	0.651195	0.001339	0.309511	0.640947	5.64E-05	0.315599
0.083244	-0.24912	-0.07835	-0.00481	-0.05638	0.001062	-0.01065	0.169076	-0.04477
0.352134	0.004737	0.38126	0.957157	0.52896	0.990547	0.905363	0.0574	0.617258
-0.2476	-0.34508	-0.4844	-0.44888	-0.31813	-0.38733	-0.47534	-0.04291	-0.53548
0.005008	7.09E-05	7.92E-09	1.2E-07	0.000268	6.84E-06	1.63E-08	0.631919	8.78E-11
0.416127	0.388983	0.386113	0.415927	0.466032	0.516308	0.347489	0.761901	0.345773
1.14E-06	6.2E-06	7.35E-06	1.16E-06	3.36E-08	5.19E-10	6.26E-05	2.45E-25	6.84E-05

CENPL	CENPM	CENPN	CENPO	CENPP	CENPU	CENPW	CEP128	CEP152
blue	blue	blue	blue	blue	blue	blue	blue	blue
-0.34518	-0.49594	-0.31355	-0.42517	-0.12294	-0.33623	-0.25871	-0.25311	-0.35602
7.06E-05	3.05E-09	0.000331	6.28E-07	0.168516	0.000111	0.003316	0.004091	3.99E-05
-0.34558	-0.52947	-0.27381	-0.39418	-0.05516	-0.31459	-0.26872	-0.32945	-0.42542
6.91E-05	1.55E-10	0.00184	4.53E-06	0.53796	0.000316	0.002253	0.000155	6.17E-07
0.796383	0.809322	0.812964	0.777205	0.651942	0.87514	0.700312	0.724348	0.775059
4.47E-29	1.14E-30	3.85E-31	6.49E-27	1.03E-16	3.17E-41	5.05E-20	6.33E-22	1.1E-26
0.783897	0.397868	0.567336	0.350781	0.570435	0.615496	0.427907	0.681036	0.723162
1.21E-27	3.62E-06	3.54E-12	5.27E-05	2.54E-12	1.36E-14	5.22E-07	1.25E-18	7.94E-22
0.125243	-0.00359	0.241753	-0.38174	0.292388	0.16645	0.351764	-0.16086	-0.18828
0.160621	0.968024	0.006177	9.5E-06	0.000851	0.06144	5.01E-05	0.070817	0.034017
0.617691	0.097648	0.456502	-0.17977	0.563123	0.437675	0.420074	0.346636	0.344962
1.03E-14	0.274758	6.88E-08	0.043133	5.52E-12	2.67E-07	8.81E-07	6.54E-05	7.14E-05
0.238405	-0.14214	0.159997	-0.2009	0.293038	0.08559	0.117482	0.035133	0.14327
0.006951	0.110925	0.072361	0.023527	0.000828	0.338684	0.188368	0.694962	0.108076
0.047348	-0.2612	-0.23619	-0.34518	-0.16193	-0.18749	-0.1948	-0.06482	-0.12193
0.597081	0.003015	0.00751	7.06E-05	0.06893	0.034795	0.02819	0.469079	0.172061
0.286661	0.352272	0.189058	0.232064	0.303805	0.323552	0.297404	0.456671	0.479111
0.001085	4.87E-05	0.033275	0.008657	0.000516	0.000207	0.000685	6.8E-08	1.21E-08
-0.14816	-0.17156	-0.30532	-0.47743	-0.13532	-0.14212	-0.06148	0.024292	-0.06738
0.096448	0.053783	0.000482	1.38E-08	0.129297	0.110956	0.492317	0.786322	0.451632
0.055263	-0.10464	-0.18276	-0.28614	-0.01535	-0.04586	-0.03308	0.168048	0.080706
0.537174	0.241688	0.03972	0.001109	0.863991	0.608689	0.711936	0.058954	0.367065
-0.22078	0.101698	-0.12636	0.396525	-0.09353	-0.15181	-0.19657	-0.08174	-0.00723
0.01262	0.255247	0.156891	3.93E-06	0.295628	0.088423	0.02676	0.36092	0.935734
0.016821	0.484503	0.084972	0.691902	-0.06412	0.202577	0.099326	0.361111	0.370968
0.851114	7.85E-09	0.342195	2.12E-19	0.473894	0.022366	0.266557	3.03E-05	1.76E-05
-0.20579	0.388928	-0.05012	0.187194	-0.1982	0.026443	0.110249	-0.03788	-0.13337
0.020281	6.22E-06	0.575773	0.035085	0.025504	0.767919	0.217232	0.6724	0.134969
-0.32609	0.165958	-0.30326	0.064718	-0.3494	-0.12629	-0.08181	0.078167	-0.0035
0.000183	0.062222	0.000529	0.46975	5.67E-05	0.15711	0.360538	0.38238	0.96887
-0.72137	-0.54403	-0.46413	-0.37545	-0.59167	-0.5924	-0.51927	-0.57135	-0.62473
1.12E-21	3.84E-11	3.88E-08	1.36E-05	2.39E-13	2.2E-13	3.98E-10	2.3E-12	4.19E-15
-0.16168	0.173937	0.074238	0.480395	-0.19544	-0.00742	-0.08937	-0.1876	-0.13428
0.069371	0.050498	0.406827	1.09E-08	0.027665	0.934048	0.317721	0.034687	0.132279
0.116646	-0.22563	0.099963	-0.01521	0.08143	-0.07252	-0.16412	-0.20391	-0.13599
0.191552	0.010756	0.263483	0.865206	0.362765	0.417771	0.065221	0.021481	0.127402
-0.42521	-0.43123	-0.29193	-0.14049	-0.32215	-0.45084	-0.42744	-0.62794	-0.5565
6.26E-07	4.16E-07	0.000868	0.115163	0.000221	1.04E-07	5.39E-07	2.76E-15	1.1E-11
0.191704	0.426047	0.521591	0.504663	0.358721	0.430185	0.49535	0.339558	0.444989
0.030843	5.92E-07	3.22E-10	1.45E-09	3.45E-05	4.47E-07	3.21E-09	9.4E-05	1.59E-07

CEP170	CEP55	CEP72	CEP78	CHAC2	CHAF1B	CHCHD3	CHEK1	CHST11
blue	blue	blue	blue	blue	blue	blue	blue	blue
-0.32158	-0.35257	-0.34635	-0.37645	-0.42111	-0.39691	-0.39366	-0.38573	-0.40157
0.000227	4.8E-05	6.64E-05	1.29E-05	8.22E-07	3.84E-06	4.68E-06	7.52E-06	2.88E-06
-0.34013	-0.37228	-0.31394	-0.35378	-0.41253	-0.36157	-0.37657	-0.37609	-0.45643
9.13E-05	1.63E-05	0.000325	4.5E-05	1.44E-06	2.96E-05	1.28E-05	1.32E-05	6.92E-08
0.613316	0.843158	0.583897	0.723518	0.641348	0.722921	0.675969	0.899586	0.575814
1.79E-14	1.74E-35	5.79E-13	7.42E-22	4.55E-16	8.31E-22	2.8E-18	8.44E-47	1.42E-12
0.607745	0.696339	0.226595	0.703522	0.560281	0.469998	0.677067	0.670092	0.467972
3.55E-14	1E-19	0.010414	2.89E-20	7.42E-12	2.48E-08	2.35E-18	6.97E-18	2.9E-08
-0.36701	-0.02103	-0.07596	0.120666	0.193586	-0.05753	0.381941	-0.0904	-0.29732
2.19E-05	0.814481	0.395995	0.176587	0.029205	0.520561	9.38E-06	0.312152	0.000687
0.170325	0.439198	-0.07216	0.538105	0.479573	0.182373	0.694632	0.325979	0.100748
0.055557	2.4E-07	0.420115	6.83E-11	1.17E-08	0.040153	1.34E-19	0.000184	0.259735
-0.06165	0.111193	-0.18838	0.212224	0.086812	-0.00483	0.348325	0.048056	0.057046
0.491101	0.213297	0.03392	0.016605	0.331807	0.957029	5.99E-05	0.591601	0.524096
-0.05822	-0.07465	-0.47818	0.216859	-0.00612	-0.21839	0.104753	-0.13004	-0.29082
0.515584	0.404229	1.3E-08	0.014328	0.945582	0.013637	0.241173	0.145065	0.00091
0.037015	0.568708	0.009214	0.05066	0.061702	0.124706	0.351657	0.416765	0.493429
0.679495	3.06E-12	0.918114	0.571649	0.490741	0.162436	5.03E-05	1.09E-06	3.77E-09
-0.42389	0.028298	-0.53801	-0.23416	-0.23131	-0.54183	-0.00081	-0.1538	-0.06031
6.84E-07	0.752149	6.89E-11	0.008056	0.008883	4.76E-11	0.992796	0.084282	0.500627
-0.14094	0.10511	-0.37627	-0.00231	-0.07249	-0.3253	0.076644	-0.03443	-0.01673
0.113991	0.239565	1.3E-05	0.979418	0.418018	0.00019	0.391745	0.700744	0.851863
0.014395	-0.11803	0.326223	-0.42101	-0.31179	0.136285	-0.30917	-0.09	0.168167
0.872387	0.186303	0.000182	8.28E-07	0.000359	0.12656	0.000405	0.314263	0.058773
0.277539	0.258575	0.401946	0.025715	-0.03584	0.242486	-0.10675	0.386263	0.394189
0.001583	0.003333	2.81E-06	0.774132	0.689098	0.006018	0.232254	7.28E-06	4.53E-06
-0.27617	-0.07272	0.247179	-0.21796	-0.00319	-0.10447	-0.07723	0.00754	-0.03803
0.001673	0.416482	0.005085	0.013827	0.971575	0.242467	0.388129	0.932954	0.671205
-0.28995	-0.00759	-0.13985	-0.34313	-0.31087	-0.38084	-0.25204	-0.02704	0.093599
0.000944	0.932546	0.116852	7.84E-05	0.000375	1E-05	0.004256	0.762867	0.295251
-0.30894	-0.70918	-0.38927	-0.48652	-0.50873	-0.53122	-0.71299	-0.64258	-0.493
0.000409	1.06E-20	6.09E-06	6.66E-09	1.02E-09	1.32E-10	5.3E-21	3.83E-16	3.9E-09
0.232866	-0.20469	0.390578	-0.04335	-0.01317	0.245775	-0.29541	0.001661	0.050432
0.008423	0.020976	5.63E-06	0.628404	0.883187	0.005349	0.000747	0.98521	0.57338
0.338631	-0.1794	0.179405	0.150551	0.207738	0.305929	-0.05208	-0.11469	-0.11886
9.85E-05	0.043579	0.04357	0.091126	0.0191	0.000469	0.56091	0.199159	0.183205
-0.09592	-0.58851	0.030418	-0.31943	-0.24345	0.010216	-0.49936	-0.49506	-0.35679
0.283364	3.44E-13	0.73425	0.000252	0.005814	0.909247	2.28E-09	3.29E-09	3.83E-05
0.335643	0.281621	0.589	0.157141	0.103303	0.494177	0.122412	0.390223	0.386374
0.000114	0.001339	3.25E-13	0.077672	0.247782	3.54E-09	0.170364	5.75E-06	7.23E-06

CKAP2	CKAP2L	CKS1B	CKS2	CLEC3B	CLIP4	CLSPN	CLUL1	CNOT6
blue	blue	blue	blue	blue	blue	blue	blue	blue
-0.44375	-0.45394	-0.20431	-0.149	0.115524	0.338409	-0.46318	0.355038	-0.28031
1.74E-07	8.32E-08	0.021221	0.094553	0.19589	9.97E-05	4.17E-08	4.21E-05	0.001413
-0.42251	-0.42265	-0.13849	-0.01722	-0.00684	0.330094	-0.49711	0.313001	-0.32107
7.5E-07	7.43E-07	0.120467	0.84764	0.939202	0.000151	2.77E-09	0.00034	0.000233
0.816172	0.922761	0.724892	0.706453	-0.49286	-0.62988	0.814627	-0.54559	0.651709
1.45E-31	1.32E-53	5.7E-22	1.72E-20	3.95E-09	2.13E-15	2.33E-31	3.29E-11	1.06E-16
0.57261	0.637607	0.555695	0.500087	-0.43245	-0.26907	0.41789	-0.21921	0.565441
2.01E-12	7.59E-16	1.19E-11	2.15E-09	3.83E-07	0.002222	1.02E-06	0.013282	4.33E-12
-0.09452	-0.09848	0.477416	0.414231	0.021313	0.064587	-0.33497	0.258489	-0.20261
0.290525	0.270661	1.39E-08	1.29E-06	0.812009	0.470654	0.000118	0.003343	0.022347
0.245666	0.280576	0.603341	0.540597	-0.27031	0.049381	-0.07557	0.17037	0.240906
0.00537	0.001398	6.05E-14	5.36E-11	0.002115	0.581406	0.398426	0.055491	0.006366
0.013335	0.014727	0.310046	0.268413	-0.21981	-0.06805	-0.26944	0.217673	0.031224
0.881712	0.86947	0.000389	0.00228	0.013025	0.447121	0.002189	0.013958	0.72748
-0.17965	-0.24213	-0.0076	-0.16993	0.434683	0.355266	-0.3103	0.449009	0.00477
0.043283	0.006094	0.932391	0.056138	3.28E-07	4.16E-05	0.000385	1.19E-07	0.957552
0.116284	0.365863	0.253776	0.13029	-0.09657	-0.45504	0.417122	-0.18002	0.227274
0.192945	2.34E-05	0.00399	0.144289	0.280124	7.67E-08	1.07E-06	0.042848	0.01018
-0.48768	-0.31591	-0.09301	-0.25576	0.312257	0.138971	-0.28492	0.324436	-0.21402
6.06E-09	0.000297	0.2983	0.003705	0.000352	0.119185	0.001167	0.000198	0.015689
-0.27897	-0.13353	-0.05651	-0.14772	0.2474	0.117802	-0.13989	0.263406	-0.08375
0.001493	0.134488	0.527981	0.097443	0.005044	0.187159	0.116725	0.002771	0.34921
-0.18635	0.003785	-0.22516	-0.20111	0.012798	-0.40971	0.226365	-0.35252	-0.23551
0.035931	0.966313	0.010925	0.02338	0.886441	1.72E-06	0.010495	4.81E-05	0.007688
0.229476	0.375094	-0.02491	-0.08518	-0.06719	-0.57315	0.645399	-0.55311	0.254977
0.009453	1.39E-05	0.781057	0.341019	0.452931	1.9E-12	2.59E-16	1.55E-11	0.003816
-0.09812	-0.02374	-0.01175	-0.15492	0.268058	-0.31383	0.241355	-0.19256	-0.21619
0.272426	0.791092	0.895651	0.082008	0.002312	0.000327	0.006265	0.03009	0.014641
-0.34985	-0.1375	-0.23296	-0.42114	0.335391	-0.2333	0.162642	-0.11723	-0.16189
5.54E-05	0.123176	0.008396	8.21E-07	0.000116	0.008297	0.067708	0.189327	0.069011
-0.49859	-0.64325	-0.5706	-0.53389	0.225198	0.464059	-0.52293	0.275025	-0.4005
2.44E-09	3.5E-16	2.5E-12	1.02E-10	0.010911	3.9E-08	2.85E-10	0.001752	3.08E-06
0.297632	0.128896	-0.159	-0.00796	-0.10351	-0.23654	0.250137	-0.3686	0.021921
0.000678	0.148669	0.074181	0.929204	0.246825	0.007418	0.004565	2.01E-05	0.806745
0.337979	0.022833	-0.10498	0.138322	-0.25094	0.367471	-0.1217	0.068028	0.121387
0.000102	0.798878	0.240153	0.120936	0.004431	2.14E-05	0.172876	0.447289	0.173996
-0.06856	-0.34554	-0.45092	-0.19696	0.040874	0.353966	-0.31919	0.144098	-0.25257
0.44375	6.93E-05	1.04E-07	0.026452	0.648198	4.45E-05	0.000254	0.106032	0.004172
0.453064	0.45793	0.453003	0.467517	-0.38953	-0.45485	0.461253	-0.52673	0.434184
8.87E-08	6.19E-08	8.91E-08	3E-08	6E-06	7.78E-08	4.83E-08	2.01E-10	3.4E-07

COL2A1	COX10	CPSF3	CPXM1	CREBL2	CRLS1	CRYL1	CSE1L	CSTF2
blue	blue	blue	blue	blue	blue	blue	blue	blue
-0.4626	-0.28511	-0.09014	-0.2641	0.464069	-0.26713	0.141798	-0.14587	-0.15257
4.36E-08	0.001158	0.313541	0.002697	3.9E-08	0.002397	0.111784	0.101748	0.086811
-0.43934	-0.22454	-0.07499	-0.31617	0.371144	-0.1996	0.233974	-0.07839	-0.15118
2.37E-07	0.011152	0.402099	0.000293	1.74E-05	0.024461	0.008108	0.380991	0.089758
0.677151	0.624203	0.679254	0.623375	-0.71395	0.512853	-0.48452	0.620866	0.72267
2.32E-18	4.48E-15	1.67E-18	4.99E-15	4.45E-21	7.07E-10	7.84E-09	6.89E-15	8.72E-22
0.42831	0.381388	0.584694	0.299727	-0.23996	0.702728	-0.46069	0.625733	0.727826
5.08E-07	9.69E-06	5.3E-13	0.000618	0.006583	3.32E-20	5.04E-08	3.68E-15	3.23E-22
-0.1116	0.03808	0.384533	-0.19549	0.16942	0.358461	0.384671	0.147572	0.028398
0.211626	0.670801	8.06E-06	0.027623	0.056886	3.5E-05	8E-06	0.097785	0.751298
0.083143	0.188379	0.598886	-0.0587	0.189891	0.771755	-0.09826	0.548427	0.500967
0.352726	0.033925	1.03E-13	0.512129	0.032493	2.44E-26	0.271755	2.48E-11	1.99E-09
-0.05465	0.094452	0.293951	-0.10144	0.132209	0.433837	-0.01263	0.271256	0.282305
0.541677	0.290851	0.000796	0.256457	0.138419	3.48E-07	0.887963	0.002037	0.001302
-0.17442	-0.45548	0.000675	-0.45214	0.401461	0.145598	-0.13774	0.05965	0.106052
0.04985	7.43E-08	0.993988	9.49E-08	2.9E-06	0.102405	0.122535	0.505306	0.235354
0.208513	0.286106	0.31785	0.535585	-0.11994	0.113639	-0.41447	0.048876	0.182903
0.018647	0.001111	0.000271	8.7E-11	0.179216	0.203332	1.27E-06	0.585285	0.039568
-0.45407	-0.27986	0.075773	-0.05277	0.608784	-0.0518	0.11576	-0.22767	-0.19777
8.24E-08	0.001439	0.397164	0.555693	3.12E-14	0.563005	0.19497	0.010047	0.025832
-0.30625	-0.21408	0.102031	-0.06339	0.477569	0.084844	-0.0439	-0.07852	0.074959
0.000462	0.015656	0.253684	0.478945	1.37E-08	0.342926	0.62407	0.380234	0.402272
0.133918	0.170876	-0.10143	0.329417	-0.38459	-0.28555	0.044318	-0.42109	-0.05505
0.133349	0.054759	0.256507	0.000156	8.04E-06	0.001137	0.620782	8.23E-07	0.538763
0.370208	0.262738	0.015227	0.555848	-0.65244	-0.31308	-0.30747	-0.08727	0.140086
1.84E-05	0.002843	0.865076	1.17E-11	9.56E-17	0.000339	0.000438	0.329279	0.116221
0.108328	0.050457	-0.11903	0.212815	-0.36626	-0.40623	0.272418	-0.37394	-0.27563
0.2254	0.573194	0.182566	0.016298	2.29E-05	2.15E-06	0.001945	1.49E-05	0.00171
-0.1494	-0.14111	-0.13974	0.260218	-0.02832	-0.49819	0.038166	-0.44054	-0.28887
0.093665	0.113538	0.117138	0.003131	0.75196	2.52E-09	0.670102	2.18E-07	0.000989
-0.5049	-0.50533	-0.49093	-0.50048	0.308495	-0.54496	0.417803	-0.32682	-0.56715
1.42E-09	1.37E-09	4.63E-09	2.08E-09	0.000418	3.5E-11	1.02E-06	0.000177	3.61E-12
0.283928	0.079496	-0.24981	0.135527	-0.58754	-0.29711	0.069173	-0.09283	-0.109
0.001217	0.374311	0.004619	0.128704	3.83E-13	0.000694	0.439668	0.299257	0.222509
0.109495	0.025293	-0.21475	-0.28584	0.036658	0.210073	-0.00808	0.242815	0.045196
0.220411	0.777737	0.015326	0.001124	0.682415	0.017764	0.928148	0.005948	0.613872
-0.11485	-0.20202	-0.5458	-0.37841	0.063847	-0.3121	0.232067	-0.21385	-0.37267
0.198521	0.022746	3.22E-11	1.15E-05	0.47576	0.000354	0.008656	0.015775	1.6E-05
0.365549	0.506641	0.462756	0.53244	-0.61001	0.040853	-0.2738	0.318539	0.253251
2.38E-05	1.22E-09	4.31E-08	1.17E-10	2.69E-14	0.648369	0.001841	0.000262	0.004069

CSTF3	CTBP2	CTDSPL2	CTPS1	CTTNBP2N	CXCL14	CYB561	CYB5R1	CYLD
blue	blue	blue	blue	blue	blue	blue	blue	blue
-0.2552	-0.33949	-0.32059	-0.42872	-0.40888	0.324844	0.328676	0.317217	0.483846
0.003784	9.44E-05	0.000238	4.94E-07	1.82E-06	0.000194	0.000161	0.000279	8.28E-09
-0.1662	-0.2914	-0.33414	-0.3746	-0.45412	0.305296	0.299165	0.305767	0.405713
0.061833	0.000888	0.000123	1.43E-05	8.21E-08	0.000483	0.000634	0.000473	2.22E-06
0.643765	0.482233	0.698575	0.812814	0.630956	-0.48675	-0.64856	-0.61801	-0.70115
3.26E-16	9.43E-09	6.82E-20	4.03E-31	1.85E-15	6.54E-09	1.66E-16	9.91E-15	4.37E-20
0.504481	0.274185	0.610494	0.478921	0.61183	-0.11514	-0.40896	-0.30085	-0.2244
1.47E-09	0.001812	2.53E-14	1.23E-08	2.15E-14	0.197401	1.81E-06	0.000589	0.011205
0.166358	-0.07337	0.09082	-0.11873	-0.38437	0.172977	0.060095	0.287527	-0.07383
0.061585	0.412376	0.309879	0.183685	8.14E-06	0.051803	0.502124	0.001046	0.409443
0.384179	0.073028	0.407291	0.123143	0.181863	0.197135	-0.10147	0.119202	0.053859
8.23E-06	0.414538	2.01E-06	0.167805	0.040725	0.026319	0.256297	0.181937	0.547577
0.150513	-0.03745	0.122795	0.00743	0.060079	0.153381	0.022998	0.145113	0.150866
0.091208	0.675915	0.169019	0.933927	0.502244	0.085137	0.797448	0.103567	0.090443
-0.02515	-0.22513	-0.16893	-0.25004	-0.2026	0.107082	0.393401	0.634824	0.313995
0.778969	0.010934	0.057622	0.004581	0.022353	0.23081	4.75E-06	1.1E-15	0.000324
-0.05924	-0.0069	0.057872	0.257504	0.564276	0.040709	0.021202	-0.06496	-0.08877
0.50827	0.938628	0.518101	0.00347	4.89E-12	0.649526	0.812966	0.468099	0.320967
-0.439	-0.32751	-0.41087	-0.42197	0.021946	0.30789	0.340778	0.549954	0.512996
2.43E-07	0.000171	1.6E-06	7.77E-07	0.806531	0.000429	8.84E-05	2.13E-11	6.98E-10
-0.23255	-0.33631	-0.24228	-0.32009	0.05039	0.291548	0.212104	0.428522	0.447175
0.008515	0.000111	0.006063	0.000244	0.5737	0.000882	0.016667	5.01E-07	1.36E-07
-0.10495	-0.14484	-0.18155	-0.00491	0.081724	0.027595	-0.1042	-0.1235	-0.21804
0.240277	0.104214	0.04108	0.956335	0.361028	0.758108	0.243662	0.166561	0.013792
0.029854	0.243768	0.051545	0.415469	0.417199	-0.48845	-0.39179	-0.46543	-0.49877
0.738992	0.005749	0.564937	1.19E-06	1.06E-06	5.69E-09	5.24E-06	3.52E-08	2.4E-09
-0.23793	0.030294	-0.18898	0.014771	-0.14435	-0.24545	-0.15529	-0.04672	-0.45182
0.007067	0.735285	0.03335	0.869081	0.105415	0.005412	0.081269	0.60197	9.72E-08
-0.45981	-0.08367	-0.46577	-0.15407	0.100961	-0.12093	0.029674	0.147692	-0.00591
5.38E-08	0.349649	3.43E-08	0.083725	0.258724	0.175626	0.740514	0.097509	0.947456
-0.26555	-0.15315	-0.58355	-0.48216	-0.55455	0.009026	0.166419	0.221074	0.367481
0.00255	0.085618	6.02E-13	9.48E-09	1.34E-11	0.919777	0.061489	0.012499	2.14E-05
0.179343	0.213594	0.137528	0.27508	-0.05859	-0.4712	-0.34167	-0.49612	-0.46281
0.043644	0.015901	0.123105	0.001748	0.51292	2.26E-08	8.45E-05	3E-09	4.29E-08
0.281036	0.100678	0.363839	0.085393	-0.14036	0.066549	-0.01781	-0.26164	0.050109
0.001372	0.260069	2.61E-05	0.339799	0.115501	0.457253	0.84247	0.002965	0.575839
-0.06893	-0.10997	-0.09108	-0.16418	-0.47015	0.060967	0.253482	-0.0617	0.118054
0.441291	0.218414	0.308468	0.065124	2.45E-08	0.495935	0.004034	0.490777	0.186212
0.506903	0.420083	0.599035	0.568267	0.318933	-0.47586	-0.56188	-0.71774	-0.57092
1.19E-09	8.8E-07	1.01E-13	3.21E-12	0.000258	1.57E-08	6.29E-12	2.21E-21	2.41E-12

CYP11A1	CYP27B1	CYP27C1	CYP4F11	DARS2	DAW1	DAXX	DBF4	DCAF13
blue	blue	blue	blue	blue	blue	blue	blue	blue
0.441258	-0.21524	-0.23503	0.309055	-0.33098	0.320176	-0.13009	-0.30404	-0.14483
2.07E-07	0.015092	0.007817	0.000407	0.000144	0.000243	0.144906	0.000511	0.104248
0.472044	-0.24837	-0.1927	0.332725	-0.33981	0.319485	-0.11114	-0.23677	-0.17003
2.11E-08	0.004869	0.029971	0.000132	9.28E-05	0.000251	0.213516	0.007361	0.055987
-0.6199	0.696417	0.54882	-0.63103	0.750541	-0.62758	0.504505	0.86307	0.723811
7.79E-15	9.87E-20	2.39E-11	1.83E-15	3.06E-24	2.89E-15	1.47E-09	6.85E-39	7.01E-22
-0.50746	0.512406	0.308433	-0.39387	0.753895	-0.37793	0.143828	0.731348	0.719664
1.14E-09	7.36E-10	0.000419	4.62E-06	1.47E-24	1.18E-05	0.106693	1.62E-22	1.54E-21
0.247706	-0.15718	-0.20393	-0.10369	-0.07328	0.120384	0.179086	0.115406	0.260646
0.004988	0.077595	0.021464	0.245994	0.412914	0.177608	0.04395	0.196348	0.00308
-0.13275	0.149079	-0.04137	-0.22007	0.49557	-0.05686	0.029321	0.518303	0.660371
0.1368	0.09437	0.644227	0.012914	3.15E-09	0.52544	0.743492	4.34E-10	3.01E-17
-0.05608	0.103175	-0.05263	-0.09242	0.146716	0.056127	0.041827	0.262517	0.381055
0.531141	0.248372	0.556779	0.301393	0.099768	0.530819	0.640563	0.002867	9.88E-06
0.158189	-0.21342	-0.3404	0.284722	0.09302	0.492918	-0.19658	-0.26202	0.045362
0.075688	0.01599	9.01E-05	0.001177	0.298257	3.93E-09	0.026756	0.002922	0.612568
-0.2555	0.48219	0.048722	-0.31812	0.415236	-0.22963	0.189306	0.335523	0.351548
0.003741	9.46E-09	0.58647	0.000268	1.21E-06	0.009405	0.03304	0.000115	5.06E-05
0.338106	0.003936	-0.39854	0.247173	-0.05316	0.249957	-0.02253	-0.2612	0.070925
0.000101	0.964973	3.47E-06	0.005086	0.552793	0.004595	0.801493	0.003015	0.428138
0.115129	0.058621	-0.29568	0.225824	0.08476	0.10417	-0.06142	-0.07454	0.117786
0.197433	0.512686	0.000738	0.010686	0.343407	0.243816	0.492708	0.404889	0.18722
-0.05025	0.199496	0.318828	-0.11422	-0.1326	-0.1526	0.138618	-0.10727	-0.25497
0.57478	0.024536	0.000259	0.201001	0.137258	0.086766	0.120133	0.229989	0.003816
-0.30492	0.518744	0.364988	-0.21005	0.162242	-0.43496	0.416083	0.140229	-0.00318
0.000491	4.17E-10	2.45E-05	0.017776	0.068395	3.22E-07	1.14E-06	0.115846	0.971736
0.062574	0.101906	-0.08857	-0.00769	-0.24629	-0.26965	0.413727	-0.16667	-0.25249
0.484622	0.254273	0.322048	0.931578	0.005251	0.002171	1.33E-06	0.061097	0.004186
0.168245	0.196122	-0.15863	0.146437	-0.17117	-0.08515	0.255204	-0.3179	-0.20388
0.058654	0.027116	0.074858	0.100422	0.054332	0.341193	0.003783	0.00027	0.021501
0.538569	-0.51	-0.19032	0.426049	-0.69266	0.410622	-0.29088	-0.66301	-0.56227
6.53E-11	9.1E-10	0.032098	5.92E-07	1.86E-19	1.63E-06	0.000907	2.04E-17	6.04E-12
-0.23916	0.004238	0.449037	-0.13975	-0.22722	-0.16863	0.092357	-0.01194	-0.24733
0.006771	0.962284	1.19E-07	0.117116	0.010198	0.058077	0.301729	0.893986	0.005057
-0.15959	-0.3381	0.16706	-0.0358	-0.02185	0.073448	-0.42396	0.109954	-0.11429
0.073104	0.000101	0.060482	0.689491	0.807322	0.411852	6.8E-07	0.21847	0.200722
0.149858	-0.52478	0.013775	0.201809	-0.48171	0.307441	-0.38337	-0.36819	-0.54738
0.092641	2.4E-10	0.877841	0.022891	9.83E-09	0.000438	8.63E-06	2.06E-05	2.75E-11
-0.3433	0.460145	0.575292	-0.64158	0.189466	-0.35848	0.378431	0.467219	0.37878
7.77E-05	5.25E-08	1.5E-12	4.4E-16	0.032889	3.5E-05	1.15E-05	3.07E-08	1.13E-05

DCLRE1B	DDIAS	DDX20	DDX23	DDX52	DDX56	DEK	DENND2A	DEPDC1
blue	blue	blue	blue	blue	blue	blue	blue	blue
-0.35535	-0.33728	-0.14807	-0.33729	-0.21615	-0.23763	-0.23937	-0.47143	-0.45019
4.14E-05	0.000105	0.096657	0.000105	0.014657	0.007143	0.00672	2.22E-08	1.09E-07
-0.40314	-0.26237	-0.12533	-0.30294	-0.21057	-0.17192	-0.3148	-0.42554	-0.42478
2.61E-06	0.002883	0.160325	0.000536	0.017488	0.05328	0.000313	6.12E-07	6.44E-07
0.770341	0.905304	0.725182	0.613407	0.69137	0.564425	0.651809	0.501392	0.913511
3.43E-26	2.59E-48	5.39E-22	1.76E-14	2.31E-19	4.81E-12	1.05E-16	1.92E-09	1.16E-50
0.636174	0.7225	0.761597	0.396442	0.714562	0.454588	0.61488	0.407171	0.695869
9.21E-16	9.01E-22	2.63E-25	3.95E-06	3.98E-21	7.93E-08	1.47E-14	2.03E-06	1.08E-19
-0.10112	0.07075	0.220848	-0.0798	0.05062	0.150481	-0.02596	-0.23592	0.022064
0.257986	0.429285	0.012592	0.3725	0.571951	0.091276	0.772023	0.007581	0.805515
0.291411	0.46668	0.666372	0.063114	0.485111	0.295021	0.431777	0.045668	0.41427
0.000887	3.2E-08	1.23E-17	0.480856	7.47E-09	0.00076	4.01E-07	0.61017	1.29E-06
0.184504	0.232919	0.391588	0.108041	0.30916	0.328941	0.004109	0.141392	0.067373
0.037842	0.008407	5.3E-06	0.226638	0.000405	0.000159	0.963433	0.112823	0.451685
-0.03609	-0.20512	0.07762	-0.11996	-0.12002	-0.04987	0.214403	-0.05983	-0.13079
0.687061	0.020704	0.385727	0.179156	0.178941	0.577639	0.015498	0.504027	0.142736
0.475982	0.355393	0.141788	0.383108	0.390264	0.373076	0.303741	0.307536	0.321841
1.55E-08	4.13E-05	0.111809	8.77E-06	5.74E-06	1.56E-05	0.000517	0.000436	0.000224
-0.01187	-0.25139	-0.22554	-0.1336	-0.08594	-0.0007	0.084479	-0.17402	-0.30139
0.894599	0.004359	0.010789	0.134293	0.336707	0.993771	0.345016	0.050389	0.000575
0.034547	-0.07831	-0.01054	-0.11073	0.030008	0.076951	0.19063	-0.05451	-0.11818
0.699802	0.381502	0.906421	0.215222	0.737694	0.389845	0.031811	0.542739	0.185746
0.07389	-0.05496	-0.34523	0.288651	0.144861	0.244567	-0.42684	0.234991	-0.1533
0.409033	0.539388	7.04E-05	0.000998	0.104176	0.005587	5.61E-07	0.007829	0.08531
0.422041	0.235999	-0.07986	0.517965	0.143582	0.286412	0.121552	0.431646	0.233104
7.73E-07	0.00756	0.372126	4.47E-10	0.1073	0.001097	0.173408	4.05E-07	0.008354
0.019763	-0.09386	-0.39425	0.231909	-0.26186	0.116088	-0.10028	0.131685	-0.041
0.825454	0.293885	4.51E-06	0.008703	0.002941	0.193699	0.261949	0.140003	0.64718
0.096664	-0.22361	-0.49808	0.180155	-0.2297	0.074291	-0.02468	0.124244	-0.22871
0.279648	0.0115	2.55E-09	0.042685	0.009381	0.406488	0.783004	0.16401	0.0097
-0.64431	-0.65033	-0.59443	-0.4083	-0.53182	-0.46124	-0.50269	-0.38719	-0.65866
3.02E-16	1.29E-16	1.74E-13	1.89E-06	1.24E-10	4.83E-08	1.72E-09	6.89E-06	3.88E-17
-0.06148	0.016286	-0.12479	0.065292	-0.10686	-0.10519	-0.2537	0.135217	0.042649
0.492332	0.855797	0.162163	0.465812	0.231777	0.239186	0.004001	0.129588	0.634009
-0.27864	0.013152	0.218992	-0.3038	-0.00339	-0.32377	-0.07838	-0.15234	0.093922
0.001513	0.883325	0.013375	0.000516	0.969821	0.000205	0.381071	0.087292	0.293576
-0.57197	-0.42095	-0.2948	-0.41852	-0.41586	-0.48768	-0.51106	-0.3201	-0.36822
2.16E-12	8.31E-07	0.000767	9.75E-07	1.16E-06	6.06E-09	8.29E-10	0.000244	2.05E-05
0.376405	0.441587	0.40547	0.390059	0.427736	0.189558	0.088292	0.000921	0.401793
1.29E-05	2.03E-07	2.26E-06	5.81E-06	5.28E-07	0.032803	0.323596	0.991802	2.84E-06

DEPDC1B	DEPTOR	DHFR	DHRS13	DHX15	DHX33	DHX8	DHX9	DIAPH3
blue	blue	blue	blue	blue	blue	blue	blue	blue
-0.48135	0.324945	-0.3578	-0.30221	-0.33209	-0.29802	-0.2117	-0.43409	-0.41033
1.01E-08	0.000193	3.63E-05	0.000554	0.000137	0.000666	0.016882	3.42E-07	1.66E-06
-0.44718	0.327235	-0.35232	-0.2523	-0.32753	-0.27939	-0.23688	-0.44796	-0.38274
1.36E-07	0.000173	4.86E-05	0.004215	0.000171	0.001467	0.007333	1.29E-07	8.96E-06
0.9045	-0.69687	0.803964	0.589065	0.798108	0.607372	0.62438	0.815415	0.695023
4.29E-48	9.13E-20	5.38E-30	3.22E-13	2.78E-29	3.71E-14	4.38E-15	1.83E-31	1.25E-19
0.631151	-0.57088	0.633834	0.280509	0.821151	0.495051	0.4326	0.672835	0.60346
1.8E-15	2.42E-12	1.26E-15	0.001402	3.08E-32	3.29E-09	3.79E-07	4.56E-18	5.96E-14
-0.0136	0.067207	0.020752	-0.06569	0.115198	-0.56805	-0.33021	-0.37728	0.069585
0.879423	0.452808	0.81687	0.463098	0.197163	3.28E-12	0.00015	1.23E-05	0.436941
0.314506	-0.23931	0.361616	-0.03587	0.603328	-0.08072	-0.00945	0.137733	0.418697
0.000317	0.006733	2.95E-05	0.688871	6.06E-14	0.366959	0.916047	0.122541	9.64E-07
0.024634	-0.25333	0.151968	-0.03906	0.351098	-0.0679	0.09267	0.100759	0.090826
0.78339	0.004058	0.088087	0.662829	5.18E-05	0.448162	0.300088	0.259683	0.309851
-0.28924	0.117583	-0.03764	-0.41281	0.006772	-0.27368	-0.18825	-0.0381	0.219748
0.000973	0.187986	0.674355	1.41E-06	0.939767	0.001849	0.03405	0.670674	0.013051
0.266495	-0.35982	0.144011	0.325428	0.24702	0.230781	0.505579	0.24967	0.104369
0.002458	3.25E-05	0.106244	0.000189	0.005114	0.009044	1.34E-09	0.004643	0.242913
-0.35139	0.312008	-0.31321	-0.34186	-0.22636	-0.41439	-0.10235	-0.39473	-0.31818
5.1E-05	0.000356	0.000336	8.37E-05	0.010498	1.28E-06	0.252212	4.39E-06	0.000267
-0.20109	0.060242	-0.13419	-0.29064	-0.03129	-0.18468	-0.05567	-0.10872	-0.16433
0.02339	0.501078	0.13256	0.000917	0.72691	0.037659	0.53415	0.223701	0.064866
-0.04139	-0.14737	-0.07921	0.560374	-0.12636	0.259683	0.324762	0.147333	-0.29634
0.64406	0.098255	0.376047	7.35E-12	0.156875	0.003195	0.000195	0.098335	0.000717
0.315232	-0.35718	0.239953	0.507652	0.062546	0.559145	0.584607	0.501083	0.05355
0.000306	3.75E-05	0.006583	1.12E-09	0.484816	8.35E-12	5.35E-13	1.97E-09	0.549882
-0.00044	0.003206	-0.09514	0.305736	-0.29323	-0.14842	-0.04558	-0.16901	-0.16744
0.99606	0.971461	0.287355	0.000473	0.000821	0.095858	0.610896	0.057502	0.059894
-0.19646	0.138899	-0.21603	0.079436	-0.3929	-0.0746	0.147477	-0.15016	-0.36231
0.026849	0.119377	0.014715	0.374674	4.9E-06	0.404538	0.098004	0.091983	2.84E-05
-0.58324	0.667169	-0.43668	-0.41175	-0.63805	-0.36899	-0.44796	-0.50095	-0.55853
6.24E-13	1.09E-17	2.86E-07	1.51E-06	7.14E-16	1.97E-05	1.29E-07	1.99E-09	8.9E-12
0.1656	-0.10553	0.122226	0.239436	-0.05946	0.294065	0.064672	0.275512	0.050841
0.062795	0.23767	0.171021	0.006704	0.506659	0.000792	0.470066	0.001718	0.57027
0.085749	0.027436	0.062571	-0.16654	0.160004	0.145848	-0.25677	0.101035	0.232956
0.337784	0.759459	0.484647	0.061294	0.072347	0.101811	0.003567	0.258372	0.008397
-0.31288	0.294596	-0.3211	-0.24204	-0.3726	-0.10661	-0.35296	-0.2382	-0.18262
0.000342	0.000774	0.000233	0.006114	1.6E-05	0.2329	4.7E-05	0.007001	0.039875
0.540175	-0.41246	0.424043	0.576659	0.484154	0.355378	0.45971	0.393288	0.266855
5.59E-11	1.45E-06	6.77E-07	1.29E-12	8.07E-09	4.13E-05	5.42E-08	4.78E-06	0.002423

DKC1	DKKL1	DLEU7	DLGAP5	DNA2	DNASE1	DNMT3B	DONSON	DPH2
blue	blue	blue	blue	blue	blue	blue	blue	blue
-0.32543	-0.20812	0.470856	-0.40826	-0.25719	-0.25498	-0.30401	-0.31184	-0.12601
0.000189	0.018878	2.32E-08	1.89E-06	0.003512	0.003815	0.000511	0.000358	0.158047
-0.36657	-0.18801	0.443043	-0.40492	-0.21806	-0.31651	-0.28128	-0.28705	-0.04515
2.25E-05	0.034285	1.83E-07	2.34E-06	0.013785	0.000289	0.001358	0.001068	0.614231
0.743626	0.51989	-0.62014	0.906937	0.733473	0.600834	0.724156	0.689893	0.519419
1.33E-23	3.76E-10	7.56E-15	9.2E-49	1.06E-22	8.16E-14	6.57E-22	2.96E-19	3.92E-10
0.541762	0.248013	-0.33838	0.662375	0.594177	0.47896	0.473609	0.76538	0.332264
4.79E-11	0.004933	9.98E-05	2.24E-17	1.79E-13	1.23E-08	1.87E-08	1.1E-25	0.000135
-0.05134	0.150734	0.151192	-0.02797	-0.02497	-0.07114	-0.31049	0.021074	0.170207
0.566495	0.090728	0.089739	0.754906	0.780526	0.426756	0.000381	0.814074	0.055728
0.224529	0.095824	0.037353	0.356042	0.324615	0.196367	0.018958	0.555678	0.205848
0.011156	0.283865	0.676729	3.99E-05	0.000197	0.026922	0.832459	1.19E-11	0.020245
0.08248	0.01511	0.002074	0.066864	0.068165	0.091694	-0.02105	0.31557	0.256604
0.356589	0.866108	0.981536	0.455121	0.446372	0.305232	0.814289	0.000302	0.00359
0.012883	-0.06757	0.131367	-0.20195	-0.2293	-0.05494	-0.32771	0.23169	-0.13808
0.885694	0.450354	0.140971	0.022794	0.009508	0.539583	0.000169	0.008768	0.121595
0.400932	0.093016	-0.33555	0.45259	0.099209	0.218716	0.30826	0.354279	0.111268
3E-06	0.298278	0.000115	9.18E-08	0.267124	0.013496	0.000422	4.38E-05	0.212985
0.004275	-0.24817	0.363092	-0.17786	-0.29475	-0.15666	-0.34881	-0.04606	-0.25445
0.961953	0.004905	2.72E-05	0.045438	0.000769	0.078602	5.84E-05	0.607141	0.003892
0.08532	-0.11763	0.268769	-0.02458	-0.14229	-0.12862	-0.19009	0.067423	-0.17208
0.340216	0.187789	0.002248	0.783855	0.110538	0.149544	0.03231	0.451354	0.053052
0.061109	0.013134	-0.25503	-0.07146	-0.08327	-0.2862	0.265022	-0.16847	-0.00492
0.494927	0.883481	0.003809	0.424652	0.351987	0.001107	0.002603	0.05831	0.956186
0.461215	0.325841	-0.56173	0.329736	0.238868	0.359526	0.572825	0.124859	0.225879
4.84E-08	0.000185	6.38E-12	0.000153	0.006839	3.31E-05	1.96E-12	0.161916	0.010666
0.106349	0.368207	-0.32382	0.010831	-0.09705	0.053997	0.044581	-0.26489	0.077047
0.234037	2.05E-05	0.000204	0.903809	0.27772	0.546547	0.618705	0.002616	0.389254
0.17688	0.058198	-0.13834	-0.07545	-0.19592	0.02287	0.02023	-0.18205	-0.12293
0.046664	0.515743	0.120898	0.399196	0.027278	0.798552	0.821395	0.04051	0.16855
-0.45769	-0.38689	0.485624	-0.70331	-0.32268	-0.42678	-0.4529	-0.62573	-0.28104
6.3E-08	7.02E-06	7.16E-09	3E-20	0.000216	5.63E-07	8.97E-08	3.68E-15	0.001371
-0.01902	0.13941	-0.20868	-0.01087	0.083834	-0.03849	0.204454	-0.30087	0.124099
0.831882	0.118009	0.018553	0.90347	0.348724	0.667476	0.021127	0.000588	0.164507
-0.34777	-0.18021	0.190829	-0.07025	0.096926	-0.10909	-0.06102	-0.07635	-0.0689
6.17E-05	0.042623	0.03163	0.432566	0.278341	0.222126	0.495574	0.393587	0.441483
-0.58106	-0.27489	0.298264	-0.46272	-0.31355	-0.38443	-0.28804	-0.52838	-0.18287
7.96E-13	0.001762	0.000659	4.32E-08	0.000331	8.11E-06	0.001024	1.72E-10	0.039607
0.429876	0.272891	-0.36083	0.392804	0.505006	0.413174	0.573726	0.236044	0.423394
4.57E-07	0.001909	3.08E-05	4.93E-06	1.41E-09	1.38E-06	1.78E-12	0.007548	7.07E-07

DRAXIN	DROSHA	DSCC1	DSEL	DSN1	DTL	DTNBP1	DTYMK	DUSP22
blue	blue	blue	blue	blue	blue	blue	blue	blue
-0.5004	-0.18411	-0.35312	-0.33587	-0.21225	-0.50183	0.435747	-0.42844	0.387062
2.09E-09	0.03826	4.66E-05	0.000113	0.016589	1.85E-09	3.05E-07	5.03E-07	6.95E-06
-0.52853	-0.15438	-0.30277	-0.33406	-0.20917	-0.49316	0.458795	-0.41565	0.35487
1.69E-10	0.083091	0.00054	0.000124	0.01827	3.85E-09	5.8E-08	1.18E-06	4.25E-05
0.727405	0.558396	0.853822	0.712245	0.715458	0.94767	-0.61352	0.677711	-0.69355
3.51E-22	9.02E-12	3.02E-37	6.07E-21	3.37E-21	7.81E-64	1.74E-14	2.13E-18	1.6E-19
0.405733	0.354266	0.755545	0.478538	0.702459	0.675415	-0.57786	0.43899	-0.34634
2.22E-06	4.38E-05	1.02E-24	1.27E-08	3.48E-20	3.05E-18	1.13E-12	2.43E-07	6.64E-05
-0.16312	-0.36126	0.149068	-0.35334	0.294697	-0.12617	0.480162	0.341692	0.090878
0.066889	3.01E-05	0.094394	4.61E-05	0.00077	0.157508	1.11E-08	8.44E-05	0.30957
0.029778	-0.08483	0.560536	-0.02763	0.673699	0.280458	-0.06885	0.383247	-0.00856
0.739634	0.342995	7.23E-12	0.757808	3.99E-18	0.001405	0.441813	8.7E-06	0.923879
-0.13986	-0.02092	0.125883	-0.11048	0.295868	0.028034	-0.01913	0.082421	0.036637
0.116815	0.815437	0.158475	0.216273	0.000732	0.754386	0.830933	0.356936	0.682588
-0.27773	-0.05625	-0.09592	-0.39606	0.075287	-0.14638	0.056729	-0.05739	0.401048
0.00157	0.529908	0.283377	4.04E-06	0.400207	0.100557	0.52641	0.5216	2.97E-06
0.274588	0.096493	0.25934	0.132746	0.289307	0.404526	-0.53733	0.324742	-0.30748
0.001783	0.280501	0.003237	0.136811	0.000971	2.39E-06	7.36E-11	0.000195	0.000437
-0.37842	-0.38747	-0.22074	-0.48665	-0.04297	-0.25866	0.062706	0.047808	0.410571
1.15E-05	6.78E-06	0.012638	6.59E-09	0.63142	0.003322	0.483702	0.593516	1.63E-06
-0.28214	-0.24234	-0.07504	-0.29505	0.095603	-0.09637	0.058997	-0.00098	0.352944
0.00131	0.00605	0.401763	0.000759	0.284983	0.281135	0.509982	0.991251	4.7E-05
-0.01907	0.139455	-0.16376	0.280098	-0.28815	-0.0465	-0.14478	-0.10084	-0.25071
0.831443	0.117891	0.065815	0.001426	0.001019	0.603639	0.104373	0.25931	0.004469
0.454089	0.526377	0.115607	0.518919	-0.08843	0.435174	-0.55464	0.200275	-0.47166
8.23E-08	2.07E-10	0.195567	4.1E-10	0.322823	3.17E-07	1.33E-11	0.023971	2.18E-08
0.206392	-0.1243	-0.0954	0.003003	-0.23012	0.031802	-0.0023	0.390675	-0.19129
0.01991	0.163804	0.286005	0.973268	0.00925	0.722636	0.979507	5.6E-06	0.031216
-0.0305	-0.05595	-0.26648	-0.10283	-0.32935	-0.05732	-0.21816	0.136994	0.04627
0.733576	0.532103	0.002459	0.249974	0.000156	0.522089	0.013739	0.124581	0.605465
-0.55794	-0.26014	-0.59285	-0.27786	-0.64956	-0.67016	0.502513	-0.51878	0.517097
9.46E-12	0.003141	2.09E-13	0.001562	1.44E-16	6.9E-18	1.75E-09	4.16E-10	4.84E-10
0.282116	0.282314	-0.05536	0.441627	-0.32055	0.104268	0.027657	-0.10002	-0.30148
0.001312	0.001301	0.536492	2.02E-07	0.000239	0.243369	0.757584	0.263196	0.000572
0.017174	0.023465	0.070713	0.172044	0.052241	-0.06135	0.212617	-0.35052	0.014217
0.848019	0.793433	0.429526	0.053099	0.559689	0.493249	0.0164	5.34E-05	0.873951
-0.18805	-0.08008	-0.4536	-0.11367	-0.44019	-0.43718	0.490286	-0.59045	0.147129
0.034239	0.370823	8.53E-08	0.203215	2.24E-07	2.76E-07	4.89E-09	2.75E-13	0.098809
0.397026	0.543064	0.346884	0.558856	0.261506	0.42763	-0.2595	0.209263	-0.61456
3.81E-06	4.22E-11	6.46E-05	8.6E-12	0.002981	5.32E-07	0.003218	0.018218	1.53E-14

DUSP3	E2F1	E2F2	E2F5	E2F7	E2F8	ECT2	EDA	EEF1A1
blue	blue	blue	blue	blue	blue	blue	blue	blue
0.511714	-0.32148	-0.43962	-0.27054	-0.42198	-0.46175	-0.39194	0.403488	-0.32654
7.82E-10	0.000228	2.33E-07	0.002096	7.76E-07	4.65E-08	5.19E-06	2.56E-06	0.000179
0.505432	-0.2454	-0.42485	-0.25928	-0.44588	-0.50621	-0.36415	0.367838	-0.28307
1.36E-09	0.005422	6.41E-07	0.003245	1.49E-07	1.27E-09	2.57E-05	2.1E-05	0.001261
-0.61348	0.69147	0.850788	0.683775	0.807588	0.846821	0.875711	-0.53879	0.626824
1.75E-14	2.27E-19	9.86E-37	8.06E-19	1.89E-30	4.47E-36	2.42E-41	6.39E-11	3.19E-15
-0.24699	0.209454	0.451848	0.764241	0.774058	0.71332	0.687994	-0.33365	0.563782
0.00512	0.01811	9.7E-08	1.43E-25	1.4E-26	4.99E-21	4.05E-19	0.000126	5.15E-12
-0.00973	0.069483	-0.17348	0.225985	-0.16655	-0.18809	0.003641	0.09442	0.202214
0.913524	0.437616	0.051122	0.010629	0.061281	0.034203	0.96759	0.291015	0.022613
0.011101	-0.00011	0.04669	0.670613	0.419862	0.337192	0.414287	-0.04669	0.454489
0.901419	0.998978	0.602192	6.43E-18	8.93E-07	0.000106	1.29E-06	0.602226	7.99E-08
0.143919	-0.17061	-0.14985	0.447023	0.087961	0.047209	0.083823	0.00162	0.277531
0.106471	0.055146	0.092655	1.38E-07	0.325422	0.598156	0.348788	0.98558	0.001583
0.209161	-0.25165	-0.29042	0.094153	0.072175	0.017127	-0.16483	0.390042	-0.09175
0.018275	0.004318	0.000926	0.292387	0.420024	0.848433	0.064047	5.82E-06	0.304956
-0.06759	0.056072	0.331552	0.327094	0.505056	0.50711	0.185867	-0.15046	0.129202
0.450261	0.531222	0.00014	0.000174	1.4E-09	1.17E-09	0.036423	0.091316	0.147699
0.253493	-0.30184	-0.21437	-0.10192	-0.06394	-0.08563	-0.41898	0.464576	-0.39371
0.004033	0.000563	0.015515	0.254207	0.475118	0.338436	9.46E-07	3.75E-08	4.66E-06
0.274403	-0.2655	-0.11884	0.052982	0.123582	0.090251	-0.18332	0.305375	-0.23478
0.001797	0.002555	0.183286	0.554126	0.166286	0.312934	0.039116	0.000481	0.007885
-0.03669	-0.08073	0.088131	-0.0407	-0.08368	-0.02682	-0.21629	-0.18661	0.028547
0.682189	0.36693	0.324482	0.649638	0.349594	0.76467	0.014593	0.035669	0.750035
-0.45155	0.424276	0.570914	-0.04007	0.315067	0.385237	0.143551	-0.27268	-0.01511
9.91E-08	6.66E-07	2.41E-12	0.654658	0.000309	7.74E-06	0.107378	0.001925	0.866089
-0.39076	0.401901	0.228613	-0.29886	-0.02475	-0.01041	-0.16717	-0.10151	-0.22098
5.57E-06	2.82E-06	0.009732	0.000643	0.78242	0.907561	0.060305	0.256135	0.01254
-0.16121	0.091244	0.148453	-0.34515	0.001657	0.02536	-0.40203	0.197239	-0.47127
0.070198	0.307617	0.095776	7.07E-05	0.985251	0.777167	2.8E-06	0.026238	2.24E-08
0.249451	-0.24024	-0.48419	-0.65294	-0.71572	-0.72634	-0.60954	0.446635	-0.56681
0.004681	0.006517	8.05E-09	8.91E-17	3.21E-21	4.31E-22	2.85E-14	1.41E-07	3.74E-12
-0.32238	0.301509	0.228643	-0.19378	-0.21585	-0.11322	0.143062	-0.30785	0.0662
0.000219	0.000572	0.009723	0.02904	0.014801	0.205026	0.108593	0.00043	0.459618
0.164106	-0.13082	-0.19326	0.030891	-0.11804	-0.14724	0.310007	-0.22617	0.275492
0.06524	0.142662	0.029483	0.730271	0.186269	0.098555	0.00039	0.010562	0.00172
0.28416	-0.2296	-0.40184	-0.45418	-0.61074	-0.56044	-0.19072	-0.01691	-0.10786
0.001205	0.009413	2.83E-06	8.17E-08	2.46E-14	7.3E-12	0.031728	0.850368	0.227413
-0.5074	0.428287	0.472067	0.296531	0.124052	0.179591	0.455934	-0.38908	0.456785
1.14E-09	5.09E-07	2.11E-08	0.000711	0.164666	0.043349	7.18E-08	6.16E-06	6.74E-08

EFCAB1	EFNA2	EI24	EIF4A3	ELAVL1	EME1	EN1	ENAH	ENPP4
blue	blue	blue	blue	blue	blue	blue	blue	blue
0.307341	-0.40679	-0.31418	-0.13058	-0.44909	-0.50869	-0.23015	-0.33	0.332414
0.00044	2.08E-06	0.000322	0.143401	1.19E-07	1.02E-09	0.009239	0.000151	0.000134
0.282092	-0.36461	-0.30676	-0.11625	-0.40586	-0.45898	-0.24716	-0.37777	0.348703
0.001313	2.5E-05	0.000452	0.193078	2.2E-06	5.72E-08	0.005087	1.19E-05	5.88E-05
-0.66352	0.703696	0.593388	0.488963	0.708688	0.916359	0.583732	0.630884	-0.72192
1.89E-17	2.8E-20	1.96E-13	5.45E-09	1.16E-20	1.57E-51	5.9E-13	1.87E-15	1.01E-21
-0.47702	0.264569	0.567941	0.240576	0.368167	0.625619	0.374879	0.46247	-0.51601
1.43E-08	0.002649	3.32E-12	0.00644	2.06E-05	3.73E-15	1.41E-05	4.4E-08	5.33E-10
0.019569	-0.29106	0.113488	0.236481	-0.42787	0.007538	-0.11432	-0.31059	0.023477
0.827142	0.000901	0.203941	0.007434	5.23E-07	0.932973	0.200622	0.00038	0.79333
-0.22083	-0.22631	0.446625	0.234395	-0.16575	0.290346	0.074832	0.019394	-0.20342
0.0126	0.010515	1.42E-07	0.007991	0.062549	0.000929	0.403069	0.828662	0.021799
-0.07321	-0.23564	0.43035	0.006832	-0.11405	0.057816	0.019186	-0.11777	-0.04212
0.413385	0.007656	4.42E-07	0.939232	0.201688	0.518502	0.83047	0.187287	0.638209
0.50969	-0.52191	-0.0407	-0.25432	-0.31838	-0.12831	-0.26567	-0.09113	0.246116
9.35E-10	3.12E-10	0.649603	0.003911	0.000264	0.15053	0.002538	0.308235	0.005284
-0.40044	0.173144	0.349478	0.330832	0.322608	0.320735	0.330339	0.228428	-0.37977
3.09E-06	0.051574	5.64E-05	0.000145	0.000216	0.000237	0.000149	0.009793	1.06E-05
0.127204	-0.52051	-0.10187	-0.03773	-0.46863	-0.28079	-0.19492	-0.14201	0.335962
0.154117	3.55E-10	0.25446	0.67362	2.75E-08	0.001386	0.028093	0.111248	0.000113
0.122842	-0.37757	0.012548	-0.05526	-0.36747	-0.14887	-0.05063	-0.0097	0.109554
0.168855	1.21E-05	0.888651	0.537212	2.14E-05	0.094847	0.571845	0.913791	0.220162
-0.06295	0.37364	-0.14868	0.118615	0.352701	0.080394	0.309427	0.056383	-0.18898
0.482012	1.51E-05	0.095263	0.18411	4.76E-05	0.368923	0.0004	0.528939	0.033345
-0.33867	0.686744	0.043752	0.126546	0.659264	0.426056	0.364959	0.515622	-0.38403
9.84E-05	4.97E-19	0.625251	0.156276	3.55E-17	5.91E-07	2.46E-05	5.52E-10	8.31E-06
-0.13574	0.350318	-0.18799	0.094202	0.118845	0.127965	0.041257	0.127965	-0.1351
0.128108	5.4E-05	0.034302	0.292134	0.183258	0.151647	0.645123	0.151648	0.129925
-0.03433	0.091258	-0.2262	-0.03088	0.040188	-0.04279	-0.0029	0.173302	0.074685
0.701615	0.307547	0.010552	0.730344	0.65372	0.632869	0.974191	0.051359	0.403996
0.517477	-0.323	-0.49446	-0.45785	-0.39279	-0.59054	-0.44214	-0.28967	0.68309
4.68E-10	0.000212	3.45E-09	6.23E-08	4.93E-06	2.72E-13	1.95E-07	0.000956	9.01E-19
-0.00116	0.580511	-0.08245	-0.03673	0.425908	0.138462	0.186531	0.25468	-0.11282
0.989636	8.46E-13	0.356757	0.681861	5.97E-07	0.120555	0.035748	0.003858	0.206625
0.138453	-0.02094	0.01542	-0.1862	0.023685	-0.12733	-0.07889	-0.15464	0.049829
0.120578	0.81524	0.863386	0.036083	0.791542	0.153695	0.377962	0.082578	0.577981
0.408115	-0.10576	-0.3172	-0.27922	-0.12261	-0.45687	-0.23891	-0.39267	0.330214
1.91E-06	0.236666	0.000279	0.001478	0.169662	6.7E-08	0.00683	4.97E-06	0.00015
-0.44129	0.60189	0.225886	0.384183	0.546773	0.466202	0.409079	0.258789	-0.4063
2.07E-07	7.19E-14	0.010664	8.23E-06	2.92E-11	3.32E-08	1.8E-06	0.003306	2.14E-06

ENPP5	EPB41L3	EPC2	EPHB3	EPHX1	EPHX2	EPS15	ERCC6L	ERICH5
blue	blue	blue	blue	blue	blue	blue	blue	blue
0.314368	0.387607	-0.31769	-0.30495	0.428626	0.231417	0.39661	-0.37094	0.430464
0.000319	6.73E-06	0.000273	0.00049	4.97E-07	0.00885	3.91E-06	1.76E-05	4.39E-07
0.348418	0.397617	-0.37074	-0.28177	0.460505	0.233579	0.396541	-0.36422	0.455254
5.97E-05	3.68E-06	1.78E-05	0.001331	5.11E-08	0.008219	3.93E-06	2.56E-05	7.55E-08
-0.73975	-0.68654	0.604356	0.619107	-0.76723	-0.55463	-0.61129	0.911936	-0.50733
2.97E-23	5.13E-19	5.35E-14	8.62E-15	7.14E-26	1.33E-11	2.29E-14	3.42E-50	1.15E-09
-0.56648	-0.55589	0.637546	0.116946	-0.69625	-0.24872	-0.23976	0.718076	-0.32169
3.87E-12	1.17E-11	7.65E-16	0.190406	1.02E-19	0.004808	0.006629	2.07E-21	0.000226
-0.03311	-0.04449	-0.23037	-0.17251	0.107175	0.208331	-0.07759	0.02768	0.058709
0.711705	0.619446	0.009171	0.052442	0.230403	0.018753	0.385881	0.757388	0.512057
-0.31059	-0.30202	0.289667	-0.25397	-0.35255	0.100459	0.006162	0.433989	-0.08835
0.00038	0.000559	0.000956	0.003962	4.8E-05	0.261111	0.945182	3.44E-07	0.3233
-0.06617	-0.16968	0.102808	-0.25304	-0.18369	0.101366	-0.10812	0.14465	0.005011
0.459791	0.056495	0.25007	0.004101	0.03871	0.256809	0.226314	0.104686	0.955412
0.195989	0.024905	-0.00388	-0.47494	0.225706	0.531683	0.220864	-0.13678	0.121502
0.027222	0.781066	0.965477	1.68E-08	0.010728	1.26E-10	0.012585	0.125166	0.173586
-0.3644	-0.30402	0.216607	-0.087	-0.42584	-0.31635	-0.3925	0.397059	-0.34355
2.53E-05	0.000511	0.014445	0.330756	6E-07	0.000291	5.02E-06	3.8E-06	7.67E-05
0.185998	0.240293	-0.33668	-0.60678	0.299779	0.234638	0.0619	-0.20232	-0.04953
0.036289	0.006505	0.000109	3.99E-14	0.000617	0.007925	0.489352	0.022541	0.580233
0.057944	0.0218	-0.12207	-0.39562	0.110721	0.267692	0.21013	0.002717	0.014995
0.517578	0.80779	0.17157	4.15E-06	0.215257	0.002345	0.017732	0.975818	0.86712
0.07244	-0.02645	-0.04919	0.262202	-0.01762	-0.22105	-0.15574	-0.04717	0.005362
0.418312	0.767886	0.582893	0.002902	0.84415	0.012509	0.080399	0.598444	0.952296
-0.28813	-0.31304	0.158592	0.535764	-0.2572	-0.46486	-0.53964	0.280343	-0.36879
0.00102	0.000339	0.074935	8.55E-11	0.003511	3.67E-08	5.88E-11	0.001411	1.99E-05
-0.11659	-0.15356	-0.34507	0.291469	0.150307	-0.19079	-0.43653	-0.04064	-0.24788
0.191752	0.084768	7.1E-05	0.000885	0.091656	0.031661	2.89E-07	0.650099	0.004957
0.040757	0.047495	-0.38391	-0.04759	0.261394	-0.07472	-0.28103	-0.1467	-0.24964
0.649138	0.595942	8.36E-06	0.595191	0.002993	0.403795	0.001372	0.099807	0.004649
0.563592	0.681018	-0.63314	-0.17377	0.736895	0.320478	0.475966	-0.71491	0.366271
5.26E-12	1.26E-18	1.39E-15	0.05072	5.32E-23	0.000239	1.55E-08	3.73E-21	2.29E-05
-0.01995	0.000433	0.063853	0.702766	-0.00902	-0.29966	-0.13765	-0.03513	-0.01663
0.823819	0.996146	0.475719	3.3E-20	0.91981	0.00062	0.122763	0.69496	0.852797
0.065064	0.118835	0.337414	0.135883	-0.14565	0.018639	0.419315	-0.03812	0.323882
0.467373	0.183295	0.000105	0.127695	0.102289	0.835233	9.26E-07	0.670493	0.000204
0.446818	0.425332	-0.08185	0.116298	0.289838	0.135833	0.406297	-0.4727	0.434889
1.4E-07	6.21E-07	0.360301	0.192889	0.000949	0.127836	2.14E-06	2.01E-08	3.24E-07
-0.4222	-0.18546	0.309232	0.644795	-0.44728	-0.65084	-0.55046	0.396328	-0.20882
7.65E-07	0.036841	0.000404	2.82E-16	1.35E-07	1.2E-16	2.02E-11	3.98E-06	0.018468

ESCO2	ESPL1	ETS2	ETV1	ETV4	EXO1	EZH2	FABP7	FAHD2B
blue	blue	blue	blue	blue	blue	blue	blue	blue
-0.47869	-0.43397	0.185816	-0.30595	-0.22331	-0.5037	-0.40902	-0.23772	0.392658
1.25E-08	3.45E-07	0.036475	0.000469	0.011616	1.58E-09	1.8E-06	0.00712	4.97E-06
-0.50957	-0.41992	0.183339	-0.23378	-0.17411	-0.48833	-0.38954	-0.1993	0.431153
9.46E-10	8.9E-07	0.039092	0.008161	0.050271	5.74E-09	5.99E-06	0.024677	4.19E-07
0.913321	0.865068	-0.62597	0.722425	0.618095	0.936366	0.928899	0.553243	-0.5667
1.33E-50	2.92E-39	3.56E-15	9.14E-22	9.8E-15	1.12E-58	9.08E-56	1.53E-11	3.79E-12
0.672158	0.487371	-0.53355	0.519278	0.504322	0.663603	0.664172	0.559766	-0.31577
5.07E-18	6.21E-09	1.06E-10	3.97E-10	1.49E-09	1.86E-17	1.71E-17	7.83E-12	0.000299
-0.11835	-0.23941	-0.09868	-0.0803	0.107101	-0.09608	-0.02508	0.158642	0.31011
0.185114	0.00671	0.269688	0.369474	0.230727	0.282558	0.779528	0.074843	0.000388
0.303219	0.037281	-0.33708	0.203732	0.327271	0.285739	0.324888	0.481999	0.099483
0.00053	0.677319	0.000107	0.021596	0.000173	0.001128	0.000194	9.61E-09	0.265797
-0.0319	-0.08557	-0.11153	0.119575	0.277486	-0.02278	0.083335	0.387331	0.10647
0.721817	0.338794	0.211887	0.180561	0.001586	0.799297	0.351609	6.84E-06	0.233503
-0.2228	-0.32257	-0.0375	-0.41405	-0.27712	-0.20014	-0.17485	-0.08039	0.227788
0.01181	0.000217	0.675536	1.31E-06	0.00161	0.02407	0.049281	0.36893	0.010006
0.318023	0.43621	-0.02707	0.082225	0.408704	0.317034	0.410573	0.137931	-0.38944
0.000269	2.95E-07	0.762576	0.358081	1.84E-06	0.000282	1.63E-06	0.122001	6.03E-06
-0.29519	-0.23489	0.274856	-0.48195	-0.14343	-0.3749	-0.15526	-0.24244	0.147946
0.000754	0.007855	0.001764	9.65E-09	0.107681	1.41E-05	0.081341	0.006028	0.096929
-0.07589	-0.0965	-0.00591	-0.22557	-0.11425	-0.14406	-0.03131	-0.04376	0.18413
0.396447	0.280465	0.947398	0.010776	0.200917	0.106115	0.726723	0.625178	0.038239
-0.07327	0.149588	0.083305	0.051311	0.057945	0.017436	0.012542	-0.25435	-0.1654
0.412958	0.093238	0.351782	0.566708	0.51757	0.845735	0.888701	0.003906	0.063124
0.336597	0.613687	-0.21294	0.26342	0.258481	0.378253	0.422908	-0.06663	-0.54327
0.000109	1.7E-14	0.016234	0.002769	0.003345	1.16E-05	7.3E-07	0.456707	4.13E-11
-0.01207	0.207995	-0.11226	-0.12554	0.115857	0.029137	0.072706	-0.24209	-0.16314
0.892827	0.018949	0.208904	0.15961	0.194596	0.745043	0.4166	0.006104	0.066859
-0.15433	0.149056	0.125727	-0.30883	-0.01361	-0.16833	0.008881	-0.37786	-0.17619
0.083204	0.09442	0.158996	0.000411	0.879322	0.058519	0.921059	1.19E-05	0.047545
-0.63926	-0.57012	0.390936	-0.35492	-0.51101	-0.67986	-0.64129	-0.39608	0.445113
6.06E-16	2.63E-12	5.51E-06	4.23E-05	8.33E-10	1.51E-18	4.59E-16	4.04E-06	1.58E-07
0.135454	0.204751	-0.03117	0.362018	-0.09497	0.169847	0.049609	0.019365	-0.2073
0.128914	0.020936	0.727914	2.89E-05	0.288205	0.056256	0.579663	0.828915	0.019359
0.0943	-0.19788	0.007216	0.258449	-0.16247	0.070318	-0.18095	0.194627	0.13662
0.291632	0.025741	0.935825	0.003349	0.067997	0.432114	0.041758	0.028332	0.125622
-0.34998	-0.40723	0.380174	-0.07514	-0.45177	-0.33229	-0.52774	-0.20374	0.243335
5.5E-05	2.02E-06	1.04E-05	0.401104	9.75E-08	0.000135	1.82E-10	0.021593	0.005839
0.39461	0.444525	-0.20421	0.486646	0.418492	0.425584	0.450799	0.260779	-0.55602
4.42E-06	1.64E-07	0.021283	6.59E-09	9.77E-07	6.1E-07	1.05E-07	0.003065	1.15E-11

FAM110C	FAM111B	FAM133A	FAM136A	FAM208B	FAM57A	FAM72B	FAM83D	FANCA
blue	blue	blue	blue	blue	blue	blue	blue	blue
0.201406	-0.40174	0.415373	-0.24601	-0.17486	-0.38704	-0.20337	-0.41065	-0.44529
0.023171	2.85E-06	1.2E-06	0.005305	0.049265	6.95E-06	0.021837	1.62E-06	1.56E-07
0.241899	-0.44067	0.414575	-0.24736	-0.19274	-0.37798	-0.24097	-0.3858	-0.44485
0.006145	2.16E-07	1.26E-06	0.005051	0.029931	1.18E-05	0.006352	7.49E-06	1.61E-07
-0.47984	0.848255	-0.67338	0.73079	0.63776	0.729495	0.640141	0.787598	0.838519
1.14E-08	2.6E-36	4.19E-18	1.81E-22	7.43E-16	2.33E-22	5.37E-16	4.66E-28	9.21E-35
-0.13047	0.630995	-0.59192	0.639203	0.58759	0.667261	0.604453	0.427218	0.411665
0.143738	1.84E-15	2.32E-13	6.1E-16	3.82E-13	1.07E-17	5.29E-14	5.47E-07	1.52E-06
0.00644	-0.12737	0.190492	0.326656	-0.2766	-0.10144	-0.00405	-0.13537	-0.23334
0.942715	0.153568	0.031937	0.000178	0.001644	0.256441	0.963992	0.129151	0.008288
0.07752	0.292211	-0.24735	0.582652	0.194454	0.339622	0.415574	0.092216	-0.04347
0.386342	0.000857	0.005053	6.66E-13	0.028475	9.37E-05	1.18E-06	0.302473	0.627471
0.247785	-0.00798	-0.15586	0.324507	0.119488	0.28336	0.260039	-0.1206	-0.17921
0.004974	0.929035	0.080159	0.000198	0.180881	0.001246	0.003152	0.176837	0.043801
0.255616	-0.04737	0.127443	-0.07183	-0.11547	-0.18114	-0.02354	-0.34794	-0.32436
0.003725	0.596908	0.153338	0.422264	0.196107	0.041549	0.792747	6.12E-05	0.000199
-0.07571	0.464931	-0.6206	0.362957	0.173847	0.613002	0.279195	0.256563	0.382891
0.397562	3.65E-08	7.13E-15	2.74E-05	0.050619	1.86E-14	0.001479	0.003596	8.88E-06
0.253824	-0.0472	0.056581	-0.05868	-0.28833	-0.08095	-0.09323	-0.3772	-0.27987
0.003983	0.598246	0.527491	0.512277	0.001012	0.365617	0.297182	1.23E-05	0.001439
0.194746	0.09392	0.007313	0.045486	-0.11155	-0.04457	0.032485	-0.24632	-0.1719
0.028233	0.29359	0.934966	0.611594	0.211824	0.618822	0.716929	0.005244	0.053301
-0.12461	-0.1346	-0.1104	-0.09992	0.005991	0.137224	-0.32744	0.025506	0.211868
0.162745	0.131374	0.216611	0.263669	0.946703	0.123942	0.000171	0.775917	0.016792
-0.34101	0.393121	-0.38406	0.058819	0.325577	0.366628	0.127179	0.407552	0.662767
8.74E-05	4.83E-06	8.29E-06	0.511264	0.000188	2.24E-05	0.154197	1.98E-06	2.11E-17
-0.22209	0.067497	-0.04975	-0.09916	-0.33045	-0.02547	-0.22514	0.117168	0.293222
0.012089	0.450856	0.578611	0.267364	0.000148	0.776229	0.01093	0.18956	0.000821
-0.04077	0.082004	-0.08627	-0.22022	-0.22659	0.013728	-0.18412	-0.0717	0.177284
0.649065	0.359382	0.334831	0.012851	0.010416	0.878256	0.038255	0.423115	0.046156
0.137618	-0.64249	0.709858	-0.69407	-0.41651	-0.73898	-0.51707	-0.46243	-0.55746
0.122856	3.88E-16	9.36E-21	1.47E-19	1.11E-06	3.48E-23	4.85E-10	4.42E-08	9.94E-12
-0.30841	-0.08113	0.154098	-0.2293	0.110499	-0.13967	-0.08909	0.227623	0.234204
0.000419	0.364525	0.083671	0.00951	0.216184	0.117319	0.319251	0.010062	0.008044
0.019109	-0.19483	0.191892	-0.11032	0.156594	-0.21164	0.005993	0.059836	-0.19915
0.831144	0.028165	0.030675	0.216915	0.078724	0.016913	0.946687	0.503977	0.024788
0.081381	-0.54898	0.505803	-0.4754	-0.17528	-0.53399	-0.35639	-0.21509	-0.35962
0.363055	2.35E-11	1.31E-09	1.62E-08	0.048714	1.01E-10	3.92E-05	0.015162	3.29E-05
-0.53758	0.247431	-0.25985	0.470893	0.559085	0.335328	0.378907	0.480001	0.514494
7.18E-11	0.005038	0.003175	2.31E-08	8.4E-12	0.000116	1.12E-05	1.13E-08	6.11E-10

FANCB	FANCC	FANCD2	FANCE	FANCG	FANCI	FANCM	FAT1	FBL
blue	blue	blue	blue	blue	blue	blue	blue	blue
-0.19197	-0.40216	-0.40495	-0.33167	-0.35384	-0.45011	-0.14343	-0.25626	-0.20233
0.030607	2.78E-06	2.33E-06	0.000139	4.49E-05	1.1E-07	0.10767	0.003637	0.022531
-0.15321	-0.39102	-0.41358	-0.30453	-0.33843	-0.4083	-0.09735	-0.31909	-0.16871
0.085493	5.48E-06	1.35E-06	0.0005	9.96E-05	1.89E-06	0.276229	0.000256	0.057949
0.760518	0.91744	0.910668	0.805841	0.755007	0.924505	0.619901	0.56015	0.579693
3.36E-25	7.21E-52	8.03E-50	3.14E-30	1.15E-24	3.36E-54	7.79E-15	7.53E-12	9.26E-13
0.796534	0.635254	0.688418	0.537891	0.444387	0.626194	0.688562	0.497922	0.16259
4.29E-29	1.04E-15	3.77E-19	6.97E-11	1.66E-07	3.46E-15	3.69E-19	2.58E-09	0.067797
0.040122	-0.07125	-0.14424	0.091428	0.215705	-0.15855	0.089659	-0.50701	0.2402
0.654254	0.426033	0.105677	0.306638	0.014868	0.075019	0.316131	1.18E-09	0.006526
0.585264	0.269194	0.277334	0.282898	0.270263	0.215229	0.54873	0.007636	0.028797
4.97E-13	0.002211	0.001596	0.00127	0.002119	0.015096	2.41E-11	0.932097	0.747916
0.256627	0.042249	0.053852	0.11305	0.046261	0.029769	0.257271	0.032245	-0.01293
0.003587	0.637193	0.547627	0.205703	0.605532	0.739712	0.003501	0.718934	0.885299
-0.03763	-0.08135	-0.15863	-0.18861	-0.08433	-0.28025	-0.17725	-0.22785	-0.33475
0.674467	0.363259	0.074872	0.033698	0.34589	0.001417	0.046198	0.009986	0.00012
0.248794	0.348367	0.434627	0.293995	0.199148	0.384554	0.125106	0.511633	0.243906
0.004794	5.98E-05	3.3E-07	0.000794	0.024792	8.05E-06	0.161081	7.88E-10	0.005721
-0.17738	-0.2372	-0.17122	-0.25476	-0.13589	-0.32199	-0.23144	-0.10331	-0.12048
0.046033	0.007251	0.05427	0.003847	0.127677	0.000223	0.008843	0.247752	0.17725
0.040081	-0.08651	-0.00854	-0.12213	-0.08305	-0.15247	-0.04292	0.029726	-0.17137
0.65458	0.333478	0.924098	0.171344	0.35329	0.087037	0.631862	0.740069	0.054052
-0.18942	0.009323	0.119245	0.039447	-0.028	0.119022	-0.23652	0.257251	0.19177
0.032933	0.917143	0.181776	0.6597	0.754664	0.1826	0.007423	0.003504	0.030783
0.062059	0.460271	0.48028	0.376657	0.338296	0.456444	-0.06784	0.505143	0.444603
0.488233	5.2E-08	1.1E-08	1.27E-05	0.0001	6.91E-08	0.448541	1.39E-09	1.64E-07
-0.28632	0.055769	-0.02287	0.167241	0.261489	-0.00444	-0.37849	-0.13594	0.501885
0.001101	0.533442	0.798536	0.060199	0.002982	0.960492	1.15E-05	0.127528	1.84E-09
-0.32124	-0.02102	0.014096	-0.0381	0.058022	-0.08453	-0.45856	0.125529	0.224232
0.000231	0.814519	0.875015	0.670612	0.517016	0.344743	5.91E-08	0.159657	0.011266
-0.51767	-0.62104	-0.57354	-0.5839	-0.42091	-0.57363	-0.45464	-0.4668	-0.35266
4.59E-10	6.74E-15	1.82E-12	5.79E-13	8.33E-07	1.8E-12	7.9E-08	3.17E-08	4.77E-05
-0.16199	0.091855	0.069952	0.063795	0.101141	0.184353	-0.1043	0.069987	0.171565
0.068826	0.304379	0.434522	0.476126	0.25787	0.038003	0.243231	0.434289	0.053774
0.134379	-0.14769	-0.17368	-0.15435	-0.28259	-0.02448	0.306661	-0.11556	-0.37763
0.132006	0.097514	0.050838	0.083169	0.001287	0.784738	0.000454	0.195737	1.2E-05
-0.43881	-0.46573	-0.52371	-0.41881	-0.46733	-0.36151	-0.22058	-0.35548	-0.36598
2.46E-07	3.44E-08	2.65E-10	9.57E-07	3.04E-08	2.97E-05	0.012701	4.11E-05	2.32E-05
0.34949	0.456372	0.5308	0.469176	0.495277	0.51635	0.35616	0.305101	0.552989
5.64E-05	6.95E-08	1.37E-10	2.64E-08	3.23E-09	5.17E-10	3.96E-05	0.000487	1.57E-11

FBN3	FBXO43	FBXO5	FBXW8	FEN1	FGFBP3	FHDC1	FIGNL1	FLT3
blue	blue	blue	blue	blue	blue	blue	blue	blue
-0.46474	-0.32628	-0.37975	-0.38943	-0.29556	-0.36663	-0.28481	-0.23585	0.292276
3.71E-08	0.000181	1.07E-05	6.03E-06	0.000742	2.24E-05	0.001173	0.007599	0.000855
-0.45703	-0.33536	-0.41907	-0.38928	-0.23696	-0.29283	-0.25328	-0.19852	0.328296
6.62E-08	0.000116	9.41E-07	6.09E-06	0.007311	0.000835	0.004065	0.025263	0.000164
0.559007	0.719458	0.912368	0.705455	0.699253	0.5706	0.688736	0.709197	-0.53728
8.47E-12	1.6E-21	2.55E-50	2.05E-20	6.07E-20	2.5E-12	3.58E-19	1.05E-20	7.39E-11
0.130845	0.629408	0.756954	0.621383	0.302236	0.197438	0.346346	0.716824	-0.31038
0.142571	2.27E-15	7.48E-25	6.45E-15	0.000553	0.026084	6.64E-05	2.62E-21	0.000383
-0.40737	0.066462	0.017175	-0.35578	0.154828	-0.14264	-0.19795	-0.02535	0.138711
2E-06	0.457842	0.848016	4.04E-05	0.082199	0.109651	0.025693	0.777272	0.119883
-0.35965	0.429384	0.481276	0.128176	0.164018	-0.15298	-0.04217	0.486657	0.017109
3.28E-05	4.72E-07	1.02E-08	0.150967	0.065386	0.085964	0.637786	6.59E-09	0.84859
-0.26922	0.104307	0.110514	0.116406	-0.03691	-0.13058	-0.12507	0.218034	0.052884
0.002208	0.243192	0.21612	0.192474	0.680336	0.143385	0.161218	0.013796	0.554858
-0.36158	0.115153	-0.11104	-0.19806	-0.18271	-0.49145	-0.41371	0.067332	0.048377
2.96E-05	0.197339	0.213927	0.02561	0.039776	4.44E-09	1.33E-06	0.451962	0.58912
0.080522	0.285854	0.367194	0.547688	0.123886	-0.06789	0.268706	0.216814	-0.1059
0.368156	0.001123	2.17E-05	2.67E-11	0.165236	0.448228	0.002254	0.014349	0.236014
-0.4574	-0.0807	-0.19279	-0.12802	-0.25229	-0.55262	-0.23531	-0.25348	0.299661
6.44E-08	0.367111	0.029891	0.151472	0.004217	1.63E-11	0.007742	0.004035	0.00062
-0.36196	0.003348	0.043694	0.002363	-0.2439	-0.41481	-0.24529	0.004698	0.068333
2.9E-05	0.970199	0.625711	0.978965	0.005722	1.24E-06	0.005443	0.958191	0.445253
0.299285	-0.12809	-0.16179	0.412397	-0.18447	0.335868	0.250781	-0.26612	-0.15028
0.000631	0.151258	0.069181	1.45E-06	0.03788	0.000113	0.004458	0.002493	0.091717
0.653698	0.158534	0.209219	0.575769	0.263256	0.480972	0.590881	0.07964	-0.45055
7.98E-17	0.075044	0.018243	1.43E-12	0.002787	1.04E-08	2.62E-13	0.373448	1.07E-07
0.248658	-0.08531	-0.09585	-0.01938	0.191698	0.169846	0.221145	-0.24977	-0.25448
0.004818	0.340264	0.283714	0.828755	0.030848	0.056257	0.01247	0.004626	0.003887
0.128984	-0.12923	-0.21212	0.13035	-0.06377	-0.08805	0.169559	-0.32994	-0.08456
0.148388	0.147605	0.016657	0.1441	0.476319	0.324926	0.05668	0.000152	0.344569
-0.11167	-0.49771	-0.70654	-0.61868	-0.33437	-0.12804	-0.33401	-0.51196	0.3489
0.211338	2.63E-09	1.7E-20	9.1E-15	0.000122	0.1514	0.000124	7.65E-10	5.82E-05
0.669307	-0.1167	-0.03873	0.001428	0.176154	0.592545	0.244308	-0.07961	-0.26723
7.86E-18	0.19135	0.665522	0.987286	0.047587	2.16E-13	0.005639	0.373605	0.002388
0.013188	-0.04753	0.047627	-0.23078	-0.04985	0.114059	-0.21078	0.19171	0.106193
0.883011	0.595656	0.594917	0.009045	0.57785	0.201658	0.017375	0.030837	0.23473
0.033906	-0.44791	-0.44963	-0.50575	-0.21444	0.053835	-0.33278	-0.31276	0.216916
0.705113	1.29E-07	1.14E-07	1.32E-09	0.015482	0.547758	0.000132	0.000344	0.014302
0.49705	0.246016	0.333313	0.343415	0.503902	0.55964	0.626242	0.194214	-0.22167
2.78E-09	0.005303	0.000129	7.73E-05	1.55E-09	7.93E-12	3.44E-15	0.028676	0.012259

FNBP1L	FOXD3	FOXM1	FOXP1	FREM2	FUBP3	G3BP1	GABPB1	GART
blue	blue	blue	blue	blue	blue	blue	blue	blue
-0.40584	-0.28001	-0.43745	0.393473	-0.38681	-0.48476	-0.34021	-0.16779	-0.41207
2.2E-06	0.001431	2.71E-07	4.73E-06	7.05E-06	7.68E-09	9.1E-05	0.059352	1.48E-06
-0.40636	-0.23477	-0.37045	0.342026	-0.38238	-0.5672	-0.41348	-0.17815	-0.42779
2.13E-06	0.007887	1.81E-05	8.3E-05	9.15E-06	3.59E-12	1.35E-06	0.045089	5.26E-07
0.678772	0.671328	0.830762	-0.61308	0.773581	0.625884	0.707534	0.664791	0.761126
1.8E-18	5.76E-18	1.34E-33	1.84E-14	1.57E-26	3.61E-15	1.42E-20	1.56E-17	2.93E-25
0.586324	0.277645	0.411947	-0.43946	0.586499	0.535193	0.693611	0.752123	0.707289
4.41E-13	0.001576	1.49E-06	2.35E-07	4.32E-13	9.03E-11	1.59E-19	2.17E-24	1.48E-20
-0.15027	-0.21502	-0.21674	0.043943	-0.35206	-0.5135	-0.15116	0.282616	0.027191
0.091739	0.015197	0.014381	0.623744	4.93E-05	6.68E-10	0.089819	0.001285	0.761543
0.250473	-0.11424	-0.00105	-0.13543	0.065496	-0.00297	0.397821	0.730252	0.439914
0.004509	0.200939	0.99063	0.128984	0.464419	0.973569	3.63E-06	2.01E-22	2.28E-07
0.009265	-0.27773	-0.13671	-0.02298	0.00093	-0.07983	0.108007	0.423803	0.282049
0.917665	0.00157	0.125363	0.797647	0.991725	0.372313	0.226786	6.88E-07	0.001315
0.019477	-0.3613	-0.38491	0.077269	-0.13839	-0.02979	0.141991	-0.04808	0.029309
0.827941	3E-05	7.89E-06	0.387884	0.120743	0.739564	0.111291	0.59143	0.74359
0.033482	0.301957	0.314034	-0.10244	0.21039	0.380716	0.35726	0.326996	0.391631
0.708627	0.00056	0.000324	0.251775	0.017589	1.01E-05	3.74E-05	0.000175	5.29E-06
-0.43752	-0.28365	-0.34622	0.309844	-0.4312	-0.07822	-0.0017	-0.01124	-0.1031
2.7E-07	0.001231	6.69E-05	0.000393	4.17E-07	0.382038	0.984823	0.900157	0.248725
-0.14177	-0.21845	-0.20623	0.140245	-0.20503	0.062129	0.161131	0.09451	0.048946
0.111858	0.013612	0.02001	0.115804	0.020756	0.487739	0.070336	0.290553	0.584742
-0.02307	0.264668	0.213335	-0.16226	0.0848	0.057484	-0.36804	-0.35746	0.08981
0.796862	0.002639	0.016032	0.068357	0.343177	0.520915	2.07E-05	3.7E-05	0.315315
0.239368	0.593662	0.54139	-0.35473	0.521877	0.573934	0.205895	-0.14906	0.229156
0.00672	1.9E-13	4.97E-11	4.28E-05	3.14E-10	1.74E-12	0.020216	0.094409	0.009556
-0.1722	0.286947	0.153145	-0.15389	-0.00907	0.128837	-0.11029	-0.3162	-0.14188
0.052884	0.001072	0.085625	0.084091	0.919357	0.148857	0.21706	0.000293	0.111586
-0.30085	0.183221	0.034165	0.054182	-0.08892	0.264678	-0.05198	-0.38273	-0.15444
0.000589	0.03922	0.702966	0.545176	0.320156	0.002638	0.56167	8.96E-06	0.08297
-0.51964	-0.30625	-0.47008	0.330049	-0.42355	-0.37947	-0.57688	-0.69507	-0.67086
3.85E-10	0.000462	2.46E-08	0.000151	6.99E-07	1.08E-05	1.26E-12	1.24E-19	6.19E-18
0.195378	0.325914	0.318069	-0.28379	0.32973	0.13641	-0.13347	-0.32002	-0.0855
0.027716	0.000185	0.000268	0.001224	0.000153	0.12621	0.13465	0.000245	0.339196
0.251211	-0.16699	-0.05105	0.041941	0.093604	-0.15396	-0.01897	0.10308	-0.07809
0.004388	0.060596	0.568719	0.639654	0.29522	0.083955	0.832339	0.248813	0.382876
-0.1211	-0.27577	-0.2375	0.239877	-0.24221	-0.4659	-0.47669	-0.42921	-0.44831
0.175026	0.0017	0.007175	0.006601	0.006078	3.39E-08	1.47E-08	4.78E-07	1.25E-07
0.258131	0.506042	0.450573	-0.19522	0.457599	0.08842	0.071482	0.316664	0.339696
0.003389	1.29E-09	1.06E-07	0.027843	6.35E-08	0.322894	0.42451	0.000287	9.34E-05

GAS1	GAS2L3	GBX2	GEMIN4	GEN1	GGH	GINS1	GINS2	GINS3
blue	blue	blue	blue	blue	blue	blue	blue	blue
-0.33561	-0.36835	-0.26284	-0.3606	-0.37811	-0.33167	-0.43586	-0.48263	-0.43636
0.000115	2.04E-05	0.002831	3.12E-05	1.17E-05	0.000139	3.03E-07	9.13E-09	2.92E-07
-0.3148	-0.35722	-0.31109	-0.34699	-0.3818	-0.30425	-0.40819	-0.41933	-0.41161
0.000313	3.74E-05	0.000371	6.42E-05	9.46E-06	0.000506	1.9E-06	9.25E-07	1.53E-06
0.738808	0.870353	0.649655	0.765838	0.811807	0.688968	0.912181	0.889712	0.729639
3.61E-23	2.85E-40	1.42E-16	9.89E-26	5.45E-31	3.45E-19	2.89E-50	2.16E-44	2.27E-22
0.533307	0.74321	0.416203	0.46935	0.699444	0.605715	0.674662	0.55368	0.5959
1.08E-10	1.45E-23	1.13E-06	2.6E-08	5.87E-20	4.54E-14	3.43E-18	1.46E-11	1.46E-13
-0.14886	-0.07403	-0.18152	-0.15029	-0.16225	0.264525	-0.01122	0.168215	0.091747
0.094857	0.408173	0.041108	0.091692	0.068374	0.002653	0.900393	0.0587	0.304947
0.147332	0.422364	0.041772	0.063384	0.333435	0.568597	0.361533	0.332248	0.389431
0.098337	7.57E-07	0.641001	0.478973	0.000128	3.09E-12	2.96E-05	0.000136	6.03E-06
0.017134	0.129674	-0.00023	0.069127	0.126993	0.155415	0.035046	0.082355	0.114097
0.848377	0.146212	0.997925	0.439971	0.154806	0.08103	0.695676	0.357322	0.201507
-0.29472	-0.23255	-0.14186	-0.37746	-0.13105	0.099753	-0.24425	-0.2124	0.200081
0.00077	0.008514	0.111614	1.22E-05	0.141942	0.264496	0.005652	0.016512	0.024111
0.260659	0.406146	0.266166	0.437527	0.37997	0.147166	0.296105	0.230704	0.313023
0.003079	2.16E-06	0.002489	2.7E-07	1.05E-05	0.098721	0.000725	0.009068	0.000339
-0.33134	-0.20043	-0.26479	-0.31067	-0.18182	-0.31967	-0.33612	-0.32469	-0.00497
0.000142	0.023859	0.002627	0.000378	0.040775	0.000249	0.000112	0.000196	0.955733
-0.17622	-0.04047	-0.10036	-0.20522	-0.04766	-0.13749	-0.1254	-0.2026	0.065212
0.047496	0.651471	0.261596	0.020638	0.594681	0.123221	0.160075	0.022348	0.46636
0.051633	-0.1255	0.306418	0.314222	-0.09107	-0.31481	-0.09197	-0.029	-0.04997
0.564275	0.159767	0.000459	0.000321	0.308571	0.000312	0.30375	0.746242	0.576866
0.460097	0.256668	0.464357	0.553515	0.325556	-0.14288	0.278912	0.298124	0.281219
5.27E-08	0.003581	3.82E-08	1.49E-11	0.000188	0.109047	0.001497	0.000664	0.001361
0.141789	-0.07077	0.053504	0.169747	-0.15391	-0.20829	0.018241	0.132246	0.066644
0.111807	0.429177	0.55022	0.056404	0.084062	0.018778	0.838707	0.138309	0.456612
-0.01885	-0.14709	0.004198	0.021786	-0.10886	-0.50776	-0.21503	-0.15409	0.052784
0.833395	0.098903	0.962643	0.807915	0.223119	1.11E-09	0.015192	0.083684	0.555613
-0.50889	-0.61175	-0.42084	-0.68677	-0.52723	-0.64513	-0.64827	-0.60031	-0.53277
1E-09	2.17E-14	8.37E-07	4.95E-19	1.91E-10	2.69E-16	1.73E-16	8.68E-14	1.14E-10
0.174421	0.005407	0.26383	0.160925	-0.0087	-0.05599	0.112259	0.151646	-0.07967
0.049849	0.951893	0.002726	0.070699	0.922646	0.531804	0.208914	0.088769	0.373283
-0.00747	0.053645	-0.06193	-0.12391	-0.00475	0.314119	0.122669	-0.03907	-0.28717
0.93361	0.549171	0.489115	0.165171	0.957698	0.000323	0.169461	0.662792	0.001062
-0.32638	-0.45943	-0.23572	-0.30809	-0.42295	-0.14458	-0.33495	-0.34633	-0.57448
0.00018	5.54E-08	0.007634	0.000425	7.28E-07	0.104865	0.000119	6.65E-05	1.64E-12
0.47867	0.36	0.488985	0.549415	0.481468	0.223117	0.405076	0.485693	0.161013
1.25E-08	3.22E-05	5.44E-09	2.25E-11	1E-08	0.011688	2.31E-06	7.12E-09	0.070544

GINS4	GJC1	GLCCI1	GLDC	GLE1	GMNN	GOLM1	GPR139	GPR143
blue	blue	blue	blue	blue	blue	blue	blue	blue
-0.20883	-0.39344	-0.2882	-0.35889	-0.03991	-0.34234	-0.36369	-0.28379	0.358212
0.018464	4.74E-06	0.001017	3.42E-05	0.655982	8.16E-05	2.63E-05	0.001224	3.55E-05
-0.23705	-0.45011	-0.22013	-0.28845	-0.04522	-0.23453	-0.39049	-0.29574	0.440525
0.007288	1.1E-07	0.012891	0.001007	0.613692	0.007955	5.66E-06	0.000736	2.18E-07
0.708577	0.772463	0.625079	0.555404	0.623389	0.535831	0.527889	0.648353	-0.60549
1.18E-20	2.06E-26	4E-15	1.23E-11	4.98E-15	8.5E-11	1.8E-10	1.71E-16	4.66E-14
0.566458	0.734153	0.26567	0.505291	0.516316	0.47962	0.347558	0.518524	-0.46054
3.88E-12	9.25E-23	0.002538	1.37E-09	5.19E-10	1.16E-08	6.24E-05	4.25E-10	5.09E-08
-0.19348	-0.25732	-0.23907	-0.11219	0.031426	0.399263	-0.47329	-0.05329	0.259473
0.029295	0.003494	0.006792	0.209177	0.725785	3.32E-06	1.92E-08	0.551858	0.003221
0.186323	0.325336	-0.1274	0.226916	0.328592	0.572558	-0.13785	0.214743	-0.07038
0.035959	0.00019	0.153468	0.010303	0.000162	2.02E-12	0.122213	0.015331	0.431703
0.077777	0.120907	-0.22104	0.194274	0.216389	0.173084	-0.08865	0.14416	0.132921
0.384764	0.175721	0.012514	0.028626	0.014546	0.051657	0.321627	0.105879	0.136291
-0.25043	-0.08293	-0.3798	-0.10247	0.043311	0.125991	-0.05753	-0.24299	0.143141
0.004515	0.353951	1.06E-05	0.251619	0.628745	0.158116	0.520561	0.005912	0.108395
0.530082	0.678879	-0.15662	0.102574	0.331778	0.071996	0.147552	0.166224	-0.34892
1.47E-10	1.77E-18	0.078683	0.251156	0.000139	0.421183	0.097831	0.061798	5.81E-05
-0.03663	-0.01627	-0.58994	-0.35167	0.053576	-0.12858	-0.39243	-0.32559	0.115633
0.68261	0.855902	2.92E-13	5.03E-05	0.549688	0.149664	5.04E-06	0.000188	0.195465
0.088304	0.110883	-0.39637	-0.13863	0.118458	-0.08594	-0.15647	-0.18078	-0.03395
0.323531	0.214584	3.97E-06	0.120109	0.184698	0.33671	0.078967	0.041964	0.704759
0.411717	0.069105	0.014031	0.065455	0.087941	-0.28168	0.287694	0.154623	-0.18464
1.52E-06	0.440113	0.875589	0.464701	0.325534	0.001336	0.001039	0.082611	0.037694
0.485991	0.380347	0.425704	0.173393	0.282476	-0.1952	0.498063	0.277059	-0.44079
6.95E-09	1.03E-05	6.05E-07	0.051235	0.001292	0.027859	2.55E-09	0.001614	2.14E-07
0.064564	-0.09551	0.06516	-0.16521	-0.16838	-0.0992	-0.11142	-0.06304	-0.11998
0.470811	0.285456	0.466716	0.063433	0.058456	0.267154	0.212348	0.481386	0.179088
0.164375	0.057353	-0.18106	-0.27049	0.008741	-0.35504	-0.05731	-0.22238	-0.11116
0.064794	0.521867	0.041641	0.0021	0.922301	4.21E-05	0.522186	0.011974	0.213439
-0.5305	-0.68212	-0.09237	-0.30532	-0.43093	-0.48317	-0.35376	-0.4523	0.50599
1.41E-10	1.05E-18	0.301664	0.000482	4.25E-07	8.74E-09	4.5E-05	9.38E-08	1.29E-09
-0.03162	-0.16464	0.62291	0.264422	-0.16546	-0.18543	0.391239	0.214626	-0.12231
0.72414	0.064354	5.3E-15	0.002664	0.063027	0.036877	5.41E-06	0.015388	0.170718
-0.28083	-0.16589	0.292742	0.19049	-0.27347	0.110744	0.118367	0.123257	0.128791
0.001383	0.062335	0.000838	0.031939	0.001865	0.215163	0.185037	0.167412	0.149001
-0.55846	-0.58734	0.120371	-0.11414	-0.4877	-0.23689	-0.00122	-0.17697	0.363212
8.96E-12	3.92E-13	0.177657	0.201324	6.05E-09	0.007328	0.989154	0.046555	2.71E-05
0.375164	0.228266	0.586612	0.268012	0.42564	0.029786	0.273481	0.581366	-0.30871
1.39E-05	0.009847	4.26E-13	0.002316	6.08E-07	0.739565	0.001864	7.69E-13	0.000414

GPR156	GPSM2	GSTO2	GSX1	GTPBP8	GTSE1	H2AFV	H2AFZ	HAND2
blue	blue	blue	blue	blue	blue	blue	blue	blue
-0.30094	-0.28156	0.449569	-0.36907	-0.0225	-0.5235	-0.40188	-0.28744	-0.23373
0.000586	0.001342	1.14E-07	1.96E-05	0.801722	2.7E-10	2.83E-06	0.00105	0.008176
-0.2963	-0.25515	0.535177	-0.33705	-0.04188	-0.54905	-0.35538	-0.23128	-0.25216
0.000719	0.003791	9.04E-11	0.000107	0.640161	2.33E-11	4.13E-05	0.008892	0.004237
0.643193	0.58427	-0.52924	0.553869	0.489349	0.897469	0.729677	0.696656	0.53768
3.52E-16	5.55E-13	1.59E-10	1.43E-11	5.28E-09	2.91E-46	2.25E-22	9.48E-20	7.11E-11
0.546145	0.33016	-0.45795	0.081241	0.456431	0.580403	0.63141	0.469696	0.154241
3.11E-11	0.00015	6.18E-08	0.363885	6.92E-08	8.56E-13	1.74E-15	2.54E-08	0.08338
-0.19036	-0.14981	0.423389	-0.30049	0.258425	-0.19143	-0.09678	0.401067	-0.29115
0.032056	0.092753	7.07E-07	0.000598	0.003352	0.031086	0.279063	2.97E-06	0.000897
0.222437	0.021434	-0.01086	-0.34609	0.515027	0.152889	0.32134	0.498107	-0.23394
0.011953	0.810961	0.903555	6.73E-05	5.83E-10	0.086156	0.00023	2.54E-09	0.008116
0.026673	-0.11741	0.130415	-0.29239	0.145011	-0.08031	0.118059	0.203943	-0.27147
0.765952	0.188637	0.1439	0.000851	0.103813	0.369408	0.186192	0.021458	0.00202
0.103247	-0.15576	-0.11695	-0.33734	-0.1447	-0.26526	-0.07209	-0.11019	-0.33801
0.248041	0.080351	0.190384	0.000105	0.104566	0.002579	0.420556	0.217459	0.000102
0.251734	-0.15981	-0.49807	-0.05557	0.190159	0.471856	0.018596	0.192144	0.224652
0.004304	0.072694	2.55E-09	0.534893	0.032244	2.14E-08	0.835608	0.030453	0.01111
-0.26719	-0.48589	0.007628	-0.45007	-0.06201	-0.19188	-0.52618	-0.2149	-0.24648
0.002392	7.01E-09	0.932167	1.1E-07	0.488564	0.030685	2.11E-10	0.015253	0.005216
-0.01032	-0.25823	-0.07711	-0.31997	0.032385	-0.07846	-0.21352	-0.18755	-0.19266
0.908288	0.003376	0.388872	0.000245	0.717765	0.380617	0.015937	0.034733	0.030002
0.084192	-0.005	-0.10683	0.241265	-0.40344	0.144667	-0.03463	-0.35417	0.249324
0.346661	0.95553	0.231912	0.006285	2.56E-06	0.104644	0.699086	4.41E-05	0.004702
0.298485	0.321721	-0.49641	0.614041	-0.16939	0.545617	0.118438	-0.05725	0.578805
0.000653	0.000226	2.93E-09	1.63E-14	0.056936	3.28E-11	0.184773	0.522636	1.02E-12
-0.07913	0.040895	-0.03323	0.345713	-0.14985	0.180958	-0.28632	-0.02624	0.253287
0.37655	0.648035	0.710694	6.86E-05	0.092662	0.041753	0.001101	0.769619	0.004064
-0.11371	-0.15225	-0.23216	0.132801	-0.30881	0.119775	-0.47981	-0.33598	0.225436
0.203069	0.087491	0.00863	0.136646	0.000412	0.179827	1.14E-08	0.000113	0.010825
-0.49754	-0.04508	0.497001	-0.05964	-0.43498	-0.6217	-0.4004	-0.56701	-0.14819
2.67E-09	0.614784	2.79E-09	0.505349	3.22E-07	6.19E-15	3.1E-06	3.67E-12	0.096372
0.067631	0.488878	-0.01201	0.678701	-0.16013	0.123463	0.286887	-0.03479	0.444118
0.449954	5.49E-09	0.893393	1.82E-18	0.07212	0.166695	0.001075	0.697764	1.69E-07
0.004265	0.171008	0.208896	0.003897	0.152613	-0.18975	0.42085	0.102736	-0.15953
0.962041	0.054568	0.018427	0.965316	0.086729	0.032624	8.37E-07	0.250403	0.073205
-0.30382	-0.03274	0.417398	0.045225	-0.24008	-0.48911	-0.02761	-0.2214	-0.16042
0.000516	0.714819	1.05E-06	0.61364	0.006554	5.38E-09	0.757952	0.012368	0.071603
0.144529	0.374595	-0.15636	0.468113	0.296642	0.430628	0.280108	0.423461	0.448952
0.104978	1.43E-05	0.07917	2.86E-08	0.000708	4.34E-07	0.001425	7.03E-07	1.2E-07

HAS1	HAUS8	HDAC2	HDHD3	HDX	HEBP1	HELLS	HENMT1	HEPH
blue	blue	blue	blue	blue	blue	blue	blue	blue
0.259879	-0.31674	-0.25551	0.206576	-0.12284	0.431705	-0.37478	0.393034	0.319885
0.003172	0.000286	0.00374	0.019797	0.168849	4.03E-07	1.42E-05	4.86E-06	0.000246
0.267863	-0.33937	-0.25117	0.219543	-0.07658	0.423394	-0.4184	0.449553	0.295635
0.002329	9.5E-05	0.004395	0.013138	0.392166	7.07E-07	9.83E-07	1.15E-07	0.00074
-0.56143	0.845056	0.709944	-0.54964	0.588232	-0.6617	0.865441	-0.64881	-0.639
6.59E-12	8.63E-36	9.22E-21	2.2E-11	3.55E-13	2.48E-17	2.48E-39	1.6E-16	6.27E-16
-0.54224	0.501848	0.506945	-0.31928	0.656929	-0.33253	0.646213	-0.49051	-0.34776
4.57E-11	1.85E-09	1.19E-09	0.000253	5E-17	0.000134	2.31E-16	4.8E-09	6.17E-05
0.054017	0.016243	0.295731	0.317664	0.108342	0.37999	-0.08347	0.225178	-0.0332
0.546403	0.856169	0.000737	0.000273	0.225341	1.05E-05	0.350842	0.010918	0.710975
-0.2853	0.194763	0.460942	0.08228	0.562214	0.190404	0.316775	-0.07652	-0.07769
0.001149	0.028219	4.94E-08	0.357759	6.07E-12	0.032018	0.000285	0.392534	0.385282
-0.23894	0.020326	0.141359	0.010425	0.233556	0.119489	-0.01759	-0.0172	0.079381
0.006822	0.820559	0.112908	0.907393	0.008225	0.180876	0.844375	0.847801	0.375008
-0.10907	-0.3824	-0.08983	0.386676	-0.05241	0.272524	-0.14767	0.040338	0.345387
0.222223	9.14E-06	0.315222	7.11E-06	0.558393	0.001937	0.097565	0.652511	6.98E-05
-0.22448	0.443434	0.138633	-0.20243	-0.02597	-0.06141	0.44532	-0.41183	0.051183
0.011173	1.78E-07	0.120093	0.022465	0.771958	0.492788	1.55E-07	1.51E-06	0.567679
0.069678	-0.08517	-0.24588	0.311768	-0.39111	0.614289	-0.06544	0.140839	0.480047
0.436325	0.341069	0.005329	0.00036	5.46E-06	1.58E-14	0.464771	0.114252	1.12E-08
-0.05737	-0.03013	-0.19919	0.256814	-0.11701	0.461743	0.0479	0.014138	0.33281
0.521774	0.736676	0.024763	0.003562	0.190148	4.65E-08	0.592804	0.874642	0.000132
0.00344	0.122163	-0.32862	-0.18963	-0.27862	-0.15637	-0.13513	-0.20853	-0.11981
0.969378	0.171241	0.000162	0.032734	0.001515	0.079153	0.129836	0.018638	0.179695
-0.25776	0.512248	0.018027	-0.45981	-0.15832	-0.58273	0.385707	-0.5365	-0.30607
0.003437	7.46E-10	0.840576	5.38E-08	0.075445	6.61E-13	7.53E-06	7.97E-11	0.000466
0.123909	0.282914	-0.10667	0.108153	-0.46585	-0.04782	0.079624	-0.21731	-0.15997
0.165159	0.001269	0.232611	0.226153	3.41E-08	0.593421	0.373541	0.014123	0.072419
0.026347	0.178627	-0.31279	0.054556	-0.64079	0.141402	0.054993	-0.18911	0.213434
0.768737	0.044504	0.000343	0.542397	4.91E-16	0.112798	0.539166	0.033228	0.015982
0.339984	-0.55308	-0.41803	0.272117	-0.47325	0.348238	-0.66163	0.559154	0.274317
9.2E-05	1.55E-11	1.01E-06	0.001969	1.92E-08	6.02E-05	2.5E-17	8.34E-12	0.001803
-0.02634	0.06577	0.034958	-0.32911	-0.02809	-0.57303	-0.06968	-0.06769	-0.40962
0.768751	0.462546	0.696399	0.000158	0.753913	1.92E-12	0.436339	0.449551	1.73E-06
0.133766	-0.31903	0.094859	-0.07003	0.484722	-0.23123	-0.16825	0.27104	-0.20382
0.133795	0.000256	0.288766	0.434033	7.71E-09	0.008907	0.05864	0.002055	0.021538
0.407104	-0.56316	-0.25093	0.050557	-0.04244	-0.10277	-0.55739	0.460108	0.012381
2.04E-06	5.5E-12	0.004433	0.572428	0.635686	0.25024	1E-11	5.26E-08	0.890122
-0.25296	0.583199	0.581226	-0.7263	0.371944	-0.65317	0.371892	-0.19001	-0.55902
0.004113	6.26E-13	7.81E-13	4.35E-22	1.66E-05	8.61E-17	1.67E-05	0.032381	8.46E-12

HIP1	HIST1H2BI	HJURP	HMGB1	HMGB2	HMGB3	HMGN1	HMGN2	HMGXB4
blue	blue	blue	blue	blue	blue	blue	blue	blue
-0.29796	-0.16525	-0.43933	-0.35948	-0.45146	-0.35599	-0.32928	-0.28175	-0.29805
0.000668	0.063362	2.38E-07	3.31E-05	9.98E-08	4E-05	0.000157	0.001332	0.000666
-0.23589	-0.20684	-0.41023	-0.30848	-0.43986	-0.23917	-0.32347	-0.24769	-0.35739
0.007588	0.019637	1.67E-06	0.000418	2.29E-07	0.006767	0.000208	0.004991	3.71E-05
0.5563	0.619128	0.90267	0.732063	0.801158	0.70082	0.740951	0.755219	0.632328
1.12E-11	8.6E-15	1.32E-47	1.4E-22	1.19E-29	4.63E-20	2.32E-23	1.1E-24	1.54E-15
0.472112	0.626317	0.602706	0.622253	0.464972	0.245348	0.552923	0.52786	0.493554
2.1E-08	3.41E-15	6.52E-14	5.77E-15	3.64E-08	0.005432	1.58E-11	1.8E-10	3.73E-09
-0.38357	0.226629	-0.04777	0.091755	0.146375	0.017789	0.202332	0.264451	-0.13146
8.53E-06	0.010403	0.593779	0.304906	0.100567	0.842654	0.022533	0.002661	0.1407
0.026223	0.594603	0.272224	0.388095	0.287756	-0.00489	0.415719	0.409824	0.135316
0.769796	1.7E-13	0.00196	6.53E-06	0.001036	0.956463	1.17E-06	1.71E-06	0.129307
0.117547	0.31942	0.055489	0.261514	-0.05492	-0.12822	0.199018	0.162235	0.034678
0.188121	0.000252	0.535507	0.00298	0.539735	0.15083	0.024889	0.068408	0.698718
-0.22878	0.094124	-0.22484	-0.09129	-0.09098	-0.31187	0.030619	0.044124	-0.26651
0.009677	0.292536	0.011042	0.307394	0.309003	0.000358	0.732553	0.622309	0.002456
0.267451	0.332893	0.415188	-0.00774	0.25167	-0.1143	0.330311	0.23209	0.30837
0.002367	0.000131	1.21E-06	0.931167	0.004314	0.20072	0.000149	0.008649	0.00042
-0.1734	-0.02829	-0.21252	-0.38891	-0.16387	-0.61637	0.036394	-0.1266	-0.23402
0.051229	0.752192	0.016453	6.22E-06	0.065634	1.22E-14	0.684578	0.156113	0.008095
-0.06784	0.192988	-0.08315	-0.19594	-0.13149	-0.47012	0.02938	-0.01361	-0.07542
0.448579	0.029717	0.352705	0.027264	0.14061	2.45E-08	0.742996	0.879285	0.399349
0.343612	-0.31484	-0.02798	-0.11355	-0.24625	-0.01593	-0.18535	-0.00618	0.327679
7.65E-05	0.000312	0.75485	0.203677	0.005258	0.858944	0.036953	0.944991	0.000169
0.495181	-0.08051	0.409497	0.136432	0.275684	0.322927	0.239856	0.20053	0.41979
3.25E-09	0.368224	1.75E-06	0.126149	0.001706	0.000213	0.006606	0.023788	8.97E-07
-0.10114	-0.17002	0.082056	-0.17565	0.213349	0.132435	0.045638	0.061546	0.076072
0.25787	0.056005	0.359077	0.048229	0.016025	0.137742	0.610405	0.491842	0.395303
0.092803	-0.25575	-0.01428	-0.38149	-0.00069	-0.24436	0.044942	-0.07617	0.008796
0.299392	0.003707	0.873422	9.64E-06	0.993876	0.005629	0.615868	0.394698	0.921819
-0.11793	-0.63968	-0.63637	-0.374	-0.52135	-0.19887	-0.49261	-0.44926	-0.42665
0.186658	5.72E-16	8.97E-16	1.48E-05	3.29E-10	0.024999	4.03E-09	1.17E-07	5.68E-07
0.205827	-0.27989	0.079604	0.234912	0.067984	0.575737	-0.12372	-0.00838	0.150401
0.020258	0.001438	0.373664	0.00785	0.447589	1.43E-12	0.165818	0.925484	0.091451
-0.12574	0.001633	-0.12627	0.248659	-0.13179	0.243175	-0.28959	-0.18248	-0.08751
0.15895	0.985459	0.157175	0.004818	0.139687	0.005872	0.000959	0.04004	0.327902
-0.32777	-0.42929	-0.44572	-0.16376	-0.41049	0.093317	-0.57313	-0.45751	-0.35655
0.000169	4.75E-07	1.51E-07	0.065821	1.64E-06	0.296712	1.9E-12	6.39E-08	3.88E-05
0.296459	0.068914	0.436141	0.448482	0.406117	0.56146	0.421149	0.398567	0.523809
0.000714	0.441386	2.97E-07	1.24E-07	2.17E-06	6.57E-12	8.2E-07	3.47E-06	2.63E-10

HMMR	HNRNPA0	HNRNPA2	HNRNPA3	HNRNPAB	HNRNPD	HNRNPDL	HNRNPH1	HNRNPK
blue	blue	blue	blue	blue	blue	blue	blue	blue
-0.3364	-0.2959	-0.41685	-0.31372	-0.39633	-0.36704	-0.25433	-0.37732	-0.39573
0.00011	0.000731	1.09E-06	0.000329	3.98E-06	2.19E-05	0.003908	1.23E-05	4.13E-06
-0.3196	-0.30396	-0.45568	-0.3622	-0.44096	-0.43872	-0.26822	-0.48742	-0.43462
0.00025	0.000512	7.32E-08	2.86E-05	2.12E-07	2.48E-07	0.002297	6.19E-09	3.3E-07
0.843485	0.592483	0.706064	0.740148	0.734326	0.615497	0.547258	0.74996	0.70414
1.54E-35	2.18E-13	1.84E-20	2.74E-23	8.94E-23	1.36E-14	2.79E-11	3.47E-24	2.59E-20
0.722997	0.18702	0.577225	0.483949	0.335737	0.280779	0.497903	0.622747	0.660273
8.19E-22	0.035258	1.22E-12	8.21E-09	0.000114	0.001386	2.59E-09	5.41E-15	3.06E-17
0.186235	-0.35267	-0.56072	-0.45288	-0.24156	-0.41495	-0.31611	-0.46458	-0.38714
0.036047	4.77E-05	7.1E-12	8.99E-08	0.006219	1.23E-06	0.000294	3.75E-08	6.91E-06
0.584553	-0.22403	-0.02629	-0.06692	-0.0818	-0.20898	0.083024	0.070824	0.189895
5.38E-13	0.011342	0.769218	0.454728	0.36058	0.018378	0.353413	0.4288	0.032488
0.206945	-0.3326	-0.04335	-0.07267	-0.21641	-0.25768	0.064559	-0.05322	0.0844
0.019574	0.000133	0.62846	0.416833	0.014537	0.003448	0.470848	0.552345	0.345468
-0.07236	-0.42765	-0.03996	-0.22306	-0.24693	-0.18856	-0.02042	-0.04154	0.047321
0.418804	5.31E-07	0.655556	0.011711	0.005131	0.033755	0.819717	0.642877	0.59729
0.340177	0.24862	0.372188	0.324146	0.508843	0.423171	0.076823	0.516793	0.297495
9.11E-05	0.004825	1.64E-05	0.000201	1.01E-09	7.17E-07	0.390638	4.97E-10	0.000682
-0.13607	-0.36773	-0.20369	-0.30344	-0.02631	-0.07919	-0.2412	-0.01681	-0.31891
0.127173	2.11E-05	0.021625	0.000525	0.769043	0.376155	0.0063	0.851198	0.000258
-0.01404	-0.26399	-0.02882	-0.15194	-0.03644	-0.0707	-0.1211	0.10136	-0.0401
0.875486	0.002709	0.747706	0.088151	0.684168	0.429588	0.175042	0.256835	0.654405
-0.25061	0.179025	0.120214	0.101948	0.236882	0.225789	-0.03679	0.049064	0.07346
0.004486	0.044024	0.178228	0.254076	0.007331	0.010698	0.681301	0.583842	0.411776
0.040639	0.553232	0.665289	0.643009	0.696203	0.739393	0.407053	0.638465	0.3602
0.65009	1.53E-11	1.45E-17	3.62E-16	1.02E-19	3.2E-23	2.04E-06	6.75E-16	3.19E-05
-0.14803	0.248837	0.016153	-0.01088	0.397641	0.312057	-0.15922	0.062254	-0.24664
0.096731	0.004787	0.856958	0.903364	3.67E-06	0.000355	0.073777	0.486866	0.005184
-0.2886	0.103611	0.192202	0.0849	0.429404	0.43546	-0.04222	0.266344	-0.1869
0.001	0.24637	0.030402	0.342606	4.71E-07	3.11E-07	0.637408	0.002472	0.035374
-0.69619	-0.33985	-0.33367	-0.33549	-0.41708	-0.25491	-0.13583	-0.52733	-0.50077
1.03E-19	9.27E-05	0.000126	0.000115	1.07E-06	0.003826	0.127836	1.9E-10	2.03E-09
-0.14204	0.401325	0.178227	0.301647	0.148071	0.23189	0.209603	-0.00279	0.149617
0.111159	2.92E-06	0.044991	0.000568	0.096644	0.008709	0.018026	0.975192	0.093173
0.075936	0.036308	-0.12503	-0.04513	-0.44657	-0.37127	0.035366	-0.24996	0.176623
0.396151	0.685288	0.16133	0.614368	1.42E-07	1.73E-05	0.693036	0.004595	0.046989
-0.41997	-0.04876	-0.42219	-0.27151	-0.54141	-0.43231	-0.228	-0.56631	-0.21173
8.87E-07	0.586213	7.66E-07	0.002017	4.96E-11	3.87E-07	0.009934	3.95E-12	0.016864
0.371298	0.330177	0.305155	0.616328	0.455971	0.475338	0.452385	0.316536	0.199184
1.73E-05	0.00015	0.000486	1.22E-14	7.16E-08	1.63E-08	9.32E-08	0.000288	0.024766

HNRNPR	HOXA1	HOXA10	HOXA11	HOXA13	HOXA2	HOXA3	HOXA4	HOXA5
blue	blue	blue	blue	blue	blue	blue	blue	blue
-0.3869	-0.04761	-0.22283	-0.0692	-0.25478	-0.1183	-0.14496	-0.07059	-0.14589
7.01E-06	0.595069	0.0118	0.439478	0.003844	0.185277	0.103934	0.430321	0.1017
-0.40101	-0.06933	-0.25655	-0.03196	-0.29266	-0.10124	-0.14228	-0.05124	-0.12696
2.98E-06	0.43864	0.003597	0.721355	0.000841	0.257394	0.110554	0.567216	0.154928
0.819559	0.421886	0.617454	0.526429	0.613192	0.547314	0.549461	0.528115	0.519665
5.08E-32	7.81E-07	1.06E-14	2.06E-10	1.81E-14	2.77E-11	2.24E-11	1.76E-10	3.84E-10
0.558985	0.568471	0.413875	0.338554	0.488569	0.526277	0.530443	0.517693	0.480415
8.49E-12	3.14E-12	1.32E-06	9.89E-05	5.63E-09	2.09E-10	1.42E-10	4.59E-10	1.09E-08
-0.39028	0.144574	-0.00521	0.129566	-0.14934	0.147175	0.105308	0.053086	0.162736
5.74E-06	0.10487	0.953638	0.146551	0.093785	0.098701	0.238675	0.55335	0.067547
0.03531	0.532421	0.210268	0.241296	0.186741	0.436687	0.417946	0.367896	0.395786
0.693498	1.18E-10	0.017656	0.006278	0.035537	2.86E-07	1.01E-06	2.09E-05	4.11E-06
-0.0854	0.270755	-0.05969	0.008092	0.054906	0.093541	0.104142	0.185567	0.146046
0.33977	0.002078	0.504987	0.928059	0.539805	0.295546	0.243943	0.036731	0.101342
-0.14114	0.138675	-0.07487	-0.16502	-0.16865	0.032184	0.036977	-0.06965	0.089658
0.11348	0.119979	0.402833	0.063738	0.058034	0.719447	0.679804	0.436491	0.316137
0.225955	0.339542	0.213463	0.194788	0.265432	0.151	0.238427	0.342535	0.245866
0.010639	9.41E-05	0.015967	0.028199	0.002561	0.090152	0.006946	8.08E-05	0.005332
-0.39401	0.188271	-0.12143	-0.06764	-0.17246	-0.10109	-0.01732	-0.039	0.036032
4.58E-06	0.034029	0.173847	0.449877	0.052514	0.258118	0.846747	0.663303	0.687556
-0.17709	0.291918	0.034159	0.021096	0.059756	0.100459	0.134188	0.162352	0.167431
0.046395	0.000868	0.703013	0.813888	0.504547	0.26111	0.132561	0.068206	0.059904
-0.05653	-0.08139	0.022762	0.042532	0.163908	-0.06099	-0.0757	0.252418	-0.05822
0.527845	0.36301	0.799489	0.634942	0.065569	0.495776	0.397647	0.004197	0.515585
0.550574	-0.07385	0.210638	0.139793	0.263524	-0.00469	0.048217	0.112329	0.066281
2E-11	0.409315	0.017452	0.116993	0.002758	0.958248	0.590357	0.208628	0.459074
-0.01359	-0.13202	0.037763	0.039434	-0.09503	-0.06483	-0.02967	-0.09432	0.029747
0.879437	0.138983	0.67338	0.659808	0.287889	0.468996	0.740548	0.291516	0.739899
-0.04205	-0.07703	-0.05927	-0.04151	-0.10795	-0.19457	-0.10423	-0.1036	-0.04404
0.638788	0.389368	0.507995	0.643113	0.227018	0.028381	0.243564	0.246399	0.622952
-0.42062	-0.43574	-0.39149	-0.22681	-0.4127	-0.36572	-0.42336	-0.45735	-0.40915
8.5E-07	3.05E-07	5.33E-06	0.010339	1.42E-06	2.36E-05	7.08E-07	6.47E-08	1.79E-06
0.323782	-0.33726	0.114112	0.074136	0.15112	-0.05505	-0.11342	-0.10868	-0.10515
0.000205	0.000106	0.201448	0.407471	0.089896	0.538748	0.204213	0.22389	0.239398
0.094496	-0.13419	-0.04274	-0.1045	0.027018	0.039421	-0.05186	-0.09195	-0.14881
0.290623	0.132557	0.633244	0.242338	0.763013	0.659912	0.562568	0.303891	0.094967
-0.23074	-0.52596	-0.26883	-0.28668	-0.2458	-0.34179	-0.41263	-0.39555	-0.42662
0.009057	2.15E-10	0.002243	0.001085	0.005344	8.4E-05	1.43E-06	4.17E-06	5.69E-07
0.452314	-0.08049	0.191126	0.225649	0.210167	0.097866	0.066829	0.164935	0.026675
9.37E-08	0.368365	0.031361	0.010748	0.017711	0.273684	0.455355	0.063874	0.765941

HOXA6	HOXA7	HOXC10	HOXC11	HOXC13	HOXC4	HOXC5	HOXC6	HOXC9
blue	blue	blue	blue	blue	blue	blue	blue	blue
-0.24787	-0.16772	-0.16432	-0.1958	-0.26735	-0.25765	-0.21427	-0.20513	-0.26773
0.004959	0.059461	0.064881	0.027378	0.002377	0.003451	0.015564	0.020695	0.002341
-0.24646	-0.10229	-0.14126	-0.11638	-0.21598	-0.22948	-0.16173	-0.17518	-0.26222
0.005218	0.252456	0.113162	0.192565	0.014736	0.009453	0.069281	0.048842	0.0029
0.535828	0.598299	0.471412	0.510379	0.589173	0.670914	0.573951	0.543026	0.608302
8.5E-11	1.1E-13	2.22E-08	8.8E-10	3.18E-13	6.14E-18	1.74E-12	4.23E-11	3.31E-14
0.410268	0.370493	0.317737	0.402753	0.387334	0.551844	0.37015	0.436714	0.421119
1.66E-06	1.81E-05	0.000273	2.68E-06	6.83E-06	1.76E-11	1.84E-05	2.85E-07	8.22E-07
-0.16979	-0.00381	0.071841	0.145194	-0.01496	0.068263	0.008185	0.103801	-0.062
0.056344	0.966072	0.422186	0.103373	0.867459	0.445719	0.927232	0.245499	0.48862
0.102009	0.147145	0.172075	0.288984	0.14672	0.357682	0.134669	0.295696	0.143992
0.253787	0.09877	0.053056	0.000984	0.099758	3.65E-05	0.131166	0.000738	0.106292
-0.07183	-0.03443	0.001029	0.127227	0.007361	0.083012	0.093425	0.110706	-0.05291
0.422286	0.700785	0.990844	0.154043	0.934543	0.353487	0.296148	0.215319	0.554682
-0.0376	-0.14827	-0.15706	-0.22053	-0.29593	-0.05786	-0.24032	-0.04208	-0.20241
0.674736	0.096193	0.07783	0.012722	0.00073	0.518184	0.006499	0.638537	0.022479
0.256544	0.172723	0.16942	0.169078	0.172486	0.370477	0.286276	0.294368	0.319854
0.003598	0.052153	0.056886	0.057396	0.052482	1.81E-05	0.001103	0.000781	0.000247
-0.11794	-0.21936	-0.11364	-0.19262	-0.29655	0.040516	-0.19214	-0.02371	-0.15836
0.186629	0.013218	0.203346	0.030034	0.000711	0.651076	0.030457	0.791315	0.075376
0.058226	-0.03101	-0.01045	-0.05226	-0.10583	0.083707	-0.11709	0.060389	-0.01267
0.51554	0.729258	0.907201	0.55957	0.236326	0.349455	0.189845	0.500034	0.887614
0.132815	0.27155	0.089637	0.023339	0.104263	0.041735	0.257004	0.008596	0.222704
0.136604	0.002014	0.316254	0.794516	0.243393	0.641298	0.003536	0.923584	0.011849
0.340259	0.280624	0.203779	0.102257	0.285259	0.262289	0.348844	0.175901	0.361887
9.08E-05	0.001395	0.021565	0.252628	0.001151	0.002892	5.83E-05	0.047911	2.91E-05
0.156551	0.059008	0.134372	0.067258	0.139572	0.060615	0.174894	0.101298	0.152234
0.078806	0.509905	0.132025	0.452466	0.11758	0.498425	0.049223	0.25713	0.087525
0.078453	-0.06309	0.024261	-0.13039	-0.05449	0.085785	0.044468	0.020398	0.072045
0.380632	0.481025	0.786589	0.143973	0.542894	0.337579	0.619599	0.81994	0.420863
-0.38274	-0.32026	-0.21354	-0.3041	-0.3028	-0.44188	-0.41035	-0.37868	-0.41518
8.96E-06	0.000242	0.015929	0.000509	0.00054	1.98E-07	1.66E-06	1.13E-05	1.21E-06
0.093116	0.186054	0.155773	0.117293	0.265471	-0.07917	0.137257	-0.02294	0.108219
0.297759	0.036232	0.080324	0.189084	0.002558	0.376277	0.123851	0.797915	0.225868
-0.10528	-0.05687	-0.13777	-0.01431	-0.01527	-0.29143	-0.17142	-0.18798	-0.17343
0.238791	0.525386	0.122444	0.873111	0.864735	0.000886	0.053985	0.034309	0.05118
-0.33256	-0.23696	-0.3039	-0.265	-0.24542	-0.58609	-0.33793	-0.4228	-0.37734
0.000133	0.007311	0.000514	0.002605	0.005417	4.52E-13	0.000102	7.35E-07	1.22E-05
0.031788	0.299022	0.265933	0.245131	0.348466	0.279836	0.397882	0.194719	0.342682
0.722756	0.000638	0.002512	0.005475	5.95E-05	0.001441	3.62E-06	0.028256	8.02E-05

HOXD10	HOXD11	HOXD13	HOXD3	HOXD4	HOXD8	HOXD9	HP1BP3	HPS1
blue	blue	blue	blue	blue	blue	blue	blue	blue
-0.20998	-0.27773	-0.20878	-0.23679	-0.29503	-0.16502	-0.14621	-0.26689	0.351793
0.017816	0.00157	0.018495	0.007355	0.000759	0.063743	0.100955	0.00242	5E-05
-0.26991	-0.31884	-0.2581	-0.19343	-0.31157	-0.1977	-0.20092	-0.29119	0.32694
0.002149	0.000259	0.003393	0.029337	0.000363	0.025885	0.023514	0.000896	0.000176
0.547827	0.54797	0.523212	0.539124	0.704901	0.589171	0.513238	0.617831	-0.68034
2.63E-11	2.6E-11	2.77E-10	6.19E-11	2.27E-20	3.19E-13	6.83E-10	1.01E-14	1.4E-18
0.358048	0.296956	0.316948	0.114744	0.422093	0.489152	0.255453	0.531392	-0.58841
3.58E-05	0.000698	0.000283	0.198947	7.7E-07	5.37E-09	0.003748	1.3E-10	3.47E-13
-0.18598	-0.17806	-0.02413	-0.11147	-0.29406	0.115809	-0.15077	-0.52249	0.036922
0.036305	0.045191	0.787746	0.212138	0.000792	0.194781	0.090656	2.97E-10	0.680254
0.021021	-0.03747	0.090453	-0.17681	-0.03083	0.346711	-0.05235	-0.02447	-0.3002
0.814532	0.675776	0.311849	0.046747	0.730795	6.52E-05	0.558864	0.784829	0.000606
-0.01735	-0.10152	-0.01933	-0.2183	-0.13257	0.163186	-0.07011	-0.02184	-0.12914
0.846481	0.256105	0.829231	0.01368	0.137346	0.066782	0.433461	0.807427	0.14791
-0.11476	-0.21262	-0.05065	-0.27614	-0.13443	-0.07328	-0.13957	-0.06381	0.112925
0.198866	0.0164	0.571704	0.001675	0.131851	0.412909	0.117577	0.47603	0.206207
0.262872	0.225477	0.171255	0.026793	0.277051	0.182832	0.228333	0.177145	-0.15352
0.002828	0.01081	0.054215	0.764933	0.001614	0.039647	0.009824	0.046331	0.08485
-0.15152	-0.2185	-0.22917	-0.37849	-0.26737	-0.17331	-0.11648	-0.35165	0.482223
0.089048	0.013592	0.00955	1.15E-05	0.002375	0.051345	0.192186	5.04E-05	9.44E-09
-0.04272	-0.12754	-0.08968	-0.27601	-0.05897	-0.01412	-0.03779	-0.10661	0.241926
0.633458	0.153009	0.316014	0.001684	0.510156	0.874795	0.673123	0.232867	0.006139
0.207472	0.290484	0.207875	0.321075	0.275329	0.055653	0.242755	0.269935	-0.05985
0.019258	0.000923	0.01902	0.000233	0.001731	0.5343	0.005961	0.002147	0.50385
0.422648	0.447455	0.267553	0.490482	0.578398	0.105684	0.456121	0.54315	-0.18094
7.43E-07	1.33E-07	0.002358	4.81E-09	1.07E-12	0.236994	7.08E-08	4.18E-11	0.041778
0.102339	0.214547	0.032321	0.283356	0.144988	-0.16202	0.161524	-0.19889	0.194466
0.252247	0.015427	0.718302	0.001246	0.103867	0.068784	0.069645	0.024986	0.028466
0.1047	0.119047	-0.0651	0.102451	0.124723	-0.23378	0.172507	-0.04161	0.400619
0.241409	0.182509	0.4671	0.251725	0.162376	0.008164	0.052453	0.642301	3.05E-06
-0.30414	-0.28414	-0.26904	-0.08936	-0.37634	-0.49294	-0.23012	-0.23988	0.570611
0.000508	0.001206	0.002224	0.317735	1.3E-05	3.92E-09	0.00925	0.0066	2.49E-12
0.19831	0.257244	0.251673	0.499429	0.282722	0.042838	0.241305	0.284251	-0.18687
0.025418	0.003504	0.004314	2.27E-09	0.001279	0.6325	0.006276	0.001201	0.035409
-0.15807	-0.1229	-0.04147	-0.10745	-0.15115	0.018673	-0.23217	0.086244	-0.29392
0.075908	0.168645	0.643434	0.229195	0.089838	0.834937	0.008625	0.334994	0.000797
-0.29699	-0.25761	-0.17082	-0.09253	-0.32536	-0.25424	-0.28226	-0.18371	0.075992
0.000697	0.003456	0.054846	0.300847	0.00019	0.003922	0.001304	0.038686	0.395802
0.388493	0.418591	0.449022	0.535432	0.422895	0.484475	0.431327	0.384334	-0.51324
6.38E-06	9.71E-07	1.19E-07	8.83E-11	7.3E-07	7.87E-09	4.14E-07	8.16E-06	6.83E-10

HPX	HSD11B1	HSPA14	HSPB8	HVCN1	IFT81	IGF2BP3	IL17RD	ILDR2
blue	blue	blue	blue	blue	blue	blue	blue	blue
-0.18034	0.229342	0.012305	0.266247	0.16799	-0.48718	-0.20246	-0.3395	-0.261
0.042465	0.009496	0.890791	0.002482	0.059042	6.31E-09	0.022448	9.43E-05	0.003039
-0.17622	0.254967	0.11089	0.26566	0.176748	-0.53065	-0.15741	-0.33614	-0.20534
0.047507	0.003817	0.214552	0.002539	0.04683	1.39E-10	0.077164	0.000112	0.02056
0.589255	-0.54813	0.384759	-0.69196	-0.49317	0.722576	0.788728	0.707953	0.579374
3.15E-13	2.56E-11	7.96E-06	2.1E-19	3.85E-09	8.88E-22	3.47E-28	1.32E-20	9.59E-13
0.422518	-0.22751	0.224793	-0.57559	-0.30611	0.715287	0.65834	0.684252	0.551013
7.49E-07	0.010101	0.011058	1.45E-12	0.000465	3.48E-21	4.06E-17	7.46E-19	1.91E-11
0.018907	0.256482	0.359601	-0.08695	0.075855	-0.17666	-0.13878	-0.25501	-0.26526
0.832897	0.003606	3.29E-05	0.331046	0.396652	0.04694	0.119708	0.003811	0.002578
0.185961	0.148844	0.311924	-0.36817	-0.08043	0.363166	0.300572	0.257692	0.17546
0.036327	0.094897	0.000357	2.06E-05	0.368715	2.71E-05	0.000596	0.003446	0.048482
0.229221	0.267336	0.179995	-0.23709	-0.07774	0.093243	0.142739	0.100652	0.181436
0.009535	0.002378	0.042872	0.007279	0.38499	0.297095	0.109402	0.260194	0.041208
-0.26258	0.05728	-0.19165	0.424188	0.248171	0.368115	-0.33188	0.064604	-0.10564
0.00286	0.522393	0.030893	6.7E-07	0.004905	2.06E-05	0.000138	0.470535	0.237185
0.270818	-0.06226	-0.24677	-0.32338	-0.01389	0.288216	0.407942	0.129309	0.025612
0.002073	0.4868	0.005161	0.000209	0.876784	0.001016	1.93E-06	0.147359	0.77501
-0.13865	0.217483	-0.3433	0.366549	0.432755	-0.2	-0.16616	-0.3552	-0.53669
0.120052	0.014043	7.77E-05	2.25E-05	3.75E-07	0.024167	0.061894	4.17E-05	7.83E-11
-0.05743	0.135046	-0.32048	0.217221	0.326418	-0.04102	0.001469	-0.0679	-0.21248
0.521312	0.13008	0.000239	0.014162	0.00018	0.647049	0.986925	0.448169	0.016472
0.147452	-0.05088	-0.1806	-0.13354	0.223271	-0.2036	0.241083	0.077487	0.036517
0.09806	0.569968	0.042164	0.13446	0.01163	0.02168	0.006326	0.386542	0.683575
0.379607	-0.53023	-0.15504	-0.1453	-0.15008	0.258308	0.352553	0.31593	0.201792
1.07E-05	1.45E-10	0.081772	0.103113	0.092161	0.003366	4.8E-05	0.000297	0.022903
0.101763	-0.24524	-0.21593	0.14878	0.132718	-0.15891	-0.14474	-0.24384	-0.37615
0.25494	0.005453	0.01476	0.09504	0.136893	0.074355	0.104455	0.005734	1.31E-05
0.0506	-0.20151	-0.49426	0.346842	0.345345	-0.14405	-0.08868	-0.24339	-0.43216
0.572101	0.023097	3.51E-09	6.47E-05	7E-05	0.106141	0.32147	0.005828	3.91E-07
-0.38166	0.102926	-0.11124	0.575362	0.273857	-0.59384	-0.47921	-0.50612	-0.31025
9.54E-06	0.249521	0.213099	1.49E-12	0.001836	1.86E-13	1.2E-08	1.28E-09	0.000386
0.04139	-0.35773	0.210399	-0.06016	-0.28643	-0.03868	0.08962	0.183591	0.338811
0.644063	3.64E-05	0.017583	0.501684	0.001096	0.665925	0.316346	0.038819	9.77E-05
-0.22388	0.13277	0.321607	-0.19277	-0.35242	0.028478	-0.05146	0.169605	0.39785
0.011397	0.136739	0.000227	0.029908	4.84E-05	0.750624	0.565547	0.056613	3.62E-06
-0.40354	0.229274	0.143561	0.191153	-0.13514	-0.39647	-0.42057	-0.23433	0.05315
2.55E-06	0.009518	0.107354	0.031337	0.129809	3.94E-06	8.52E-07	0.008008	0.552868
0.513076	-0.39562	0.642155	-0.67021	-0.58398	0.173074	0.484318	0.300938	0.370704
6.93E-10	4.15E-06	4.07E-16	6.84E-18	5.74E-13	0.05167	7.97E-09	0.000586	1.79E-05

ILF2	INTS4	INTS7	IP6K2	IPO9	IQCA1	IQCC	IQGAP3	IRX5
blue	blue	blue	blue	blue	blue	blue	blue	blue
-0.28944	-0.17654	-0.2354	-0.33857	-0.40939	0.223226	-0.16993	-0.52591	-0.35894
0.000965	0.047094	0.007717	9.89E-05	1.76E-06	0.011647	0.056129	2.16E-10	3.41E-05
-0.24864	-0.19328	-0.21734	-0.26402	-0.42857	0.23688	-0.15294	-0.53418	-0.35866
0.00482	0.029465	0.014106	0.002706	4.99E-07	0.007332	0.086051	9.94E-11	3.46E-05
0.781579	0.703399	0.797844	0.598239	0.655964	-0.69004	0.59249	0.848436	0.60733
2.18E-27	2.95E-20	2.99E-29	1.11E-13	5.75E-17	2.89E-19	2.17E-13	2.43E-36	3.73E-14
0.598694	0.630339	0.684031	0.369568	0.466456	-0.47685	0.549191	0.539172	0.440308
1.05E-13	2.01E-15	7.73E-19	1.9E-05	3.25E-08	1.45E-08	2.3E-11	6.16E-11	2.22E-07
0.412749	0.063607	-0.15521	0.148331	-0.60791	0.109208	0.317501	-0.25505	0.120225
1.42E-06	0.477426	0.081442	0.096052	3.48E-14	0.221631	0.000276	0.003805	0.178188
0.552586	0.415499	0.328564	0.162929	-0.15582	-0.15832	0.514328	0.093715	0.274905
1.63E-11	1.19E-06	0.000162	0.067218	0.080239	0.075444	6.2E-10	0.294647	0.001761
0.311888	0.242439	0.083188	0.272043	-0.16015	-0.08599	0.337834	-0.08127	0.17526
0.000358	0.006028	0.352464	0.001975	0.072084	0.336438	0.000103	0.363718	0.048743
-0.03128	-0.09374	-0.01825	-0.12374	-0.06712	0.438482	0.202922	-0.24135	0.039709
0.727024	0.294542	0.838662	0.165743	0.453361	2.52E-07	0.022134	0.006267	0.657584
0.221369	0.347819	0.204732	0.165543	0.234956	-0.52038	0.053506	0.460363	0.211268
0.012379	6.15E-05	0.020948	0.062887	0.007838	3.59E-10	0.550211	5.16E-08	0.017112
-0.17857	-0.01395	-0.30973	-0.32926	-0.40628	0.049039	-0.21499	-0.20272	-0.12903
0.044579	0.876281	0.000395	0.000157	2.14E-06	0.584027	0.015212	0.022268	0.14825
-0.09241	0.082677	-0.02686	-0.25763	-0.14037	0.045434	-0.06253	-0.08903	-0.04107
0.301473	0.35544	0.764344	0.003454	0.115475	0.612001	0.484912	0.319565	0.646616
-0.07251	0.012195	-0.08874	0.159187	0.324705	-0.10784	-0.18292	0.06454	-0.02722
0.417863	0.891759	0.32113	0.073837	0.000196	0.227529	0.039547	0.470978	0.761301
0.060103	0.279964	0.29605	0.355529	0.661477	-0.44733	0.023987	0.561929	0.243259
0.502071	0.001433	0.000726	4.1E-05	2.56E-17	1.35E-07	0.788944	6.25E-12	0.005855
-0.02349	-0.13038	-0.22909	0.139693	-0.0487	-0.18934	-0.06162	0.146862	0.09526
0.793199	0.144022	0.009579	0.117258	0.586638	0.033008	0.491331	0.099428	0.286726
-0.2679	-0.04415	-0.2398	-0.07988	0.038746	-0.1517	-0.29011	0.125833	-0.01131
0.002327	0.622088	0.006619	0.37202	0.665385	0.088656	0.000938	0.158641	0.899548
-0.57977	-0.52987	-0.47546	-0.40388	-0.37184	0.547153	-0.50587	-0.61679	-0.50993
9.18E-13	1.49E-10	1.62E-08	2.49E-06	1.67E-05	2.82E-11	1.31E-09	1.16E-14	9.16E-10
-0.00263	-0.15148	0.096188	0.190183	0.38451	0.000415	-0.11076	0.127159	0.017138
0.976632	0.089126	0.282034	0.032222	8.08E-06	0.996308	0.215113	0.154264	0.848339
-0.06076	-0.23117	0.105553	-0.1287	0.036328	0.231768	-0.01017	-0.179	-0.1827
0.497392	0.008926	0.237577	0.149301	0.685127	0.008745	0.909608	0.044051	0.039789
-0.3921	-0.5413	-0.30071	-0.23593	-0.14619	0.468422	-0.33443	-0.42816	-0.39147
5.14E-06	5.01E-11	0.000592	0.007578	0.101003	2.8E-08	0.000122	5.13E-07	5.34E-06
0.492682	0.484837	0.387591	0.577809	0.261357	-0.4558	0.307709	0.37912	0.364407
4.01E-09	7.64E-09	6.73E-06	1.14E-12	0.002998	7.25E-08	0.000433	1.11E-05	2.53E-05

ISCU	ISL2	ITM2B	ITPRIPL1	JADE2	JMJD1C	KDM1A	KDM4D	KDM5B
blue	blue	blue	blue	blue	blue	blue	blue	blue
0.422121	-0.2833	0.268139	-0.34291	0.466499	-0.10243	-0.35314	-0.18648	-0.27187
7.69E-07	0.001249	0.002304	7.93E-05	3.24E-08	0.251814	4.66E-05	0.035801	0.001988
0.456629	-0.26615	0.205876	-0.35	0.423862	-0.16069	-0.31371	-0.09928	-0.2793
6.82E-08	0.002491	0.020228	5.49E-05	6.85E-07	0.071125	0.000329	0.266801	0.001473
-0.79936	0.568138	-0.4838	0.786601	-0.83988	0.42309	0.78685	0.592672	0.627848
1.97E-29	3.25E-12	8.31E-09	6.04E-28	5.68E-35	7.21E-07	5.66E-28	2.13E-13	2.79E-15
-0.57634	0.272267	-0.1713	0.711638	-0.60033	0.446743	0.350436	0.372667	0.418148
1.34E-12	0.001957	0.054145	6.78E-21	8.66E-14	1.4E-07	5.37E-05	1.6E-05	9.99E-07
0.362808	-0.17862	-0.04198	-0.09329	-0.04202	-0.21492	-0.04291	-0.00259	-0.3241
2.77E-05	0.044517	0.639345	0.296866	0.639062	0.015243	0.631925	0.976987	0.000201
-0.05046	-0.06721	0.020294	0.378776	-0.30801	0.158572	-0.00521	0.155122	-0.03127
0.573174	0.452796	0.820841	1.13E-05	0.000427	0.074973	0.953637	0.081613	0.727114
0.090475	-0.10294	0.083771	0.210306	-0.0469	0.133574	-0.10709	0.11226	-0.02714
0.31173	0.249449	0.349084	0.017635	0.600534	0.134357	0.230767	0.20891	0.76199
0.190003	-0.30395	0.160877	-0.04637	0.114547	-0.20135	-0.26901	-0.24868	-0.33915
0.032388	0.000513	0.070785	0.604662	0.199722	0.023207	0.002226	0.004815	9.6E-05
-0.41526	0.363696	-0.13408	0.514406	-0.25006	0.101097	0.118419	0.193906	0.321612
1.21E-06	2.63E-05	0.132871	6.16E-10	0.004578	0.258082	0.184843	0.028934	0.000227
0.249902	-0.16245	0.338008	-0.02639	0.343766	-0.19392	-0.53507	-0.35343	-0.46205
0.004604	0.068039	0.000102	0.768373	7.59E-05	0.02892	9.13E-11	4.58E-05	4.54E-08
0.072763	-0.11381	0.267944	0.077858	0.231496	-0.08881	-0.39686	-0.22253	-0.30518
0.416237	0.202634	0.002322	0.384267	0.008826	0.320779	3.85E-06	0.011919	0.000485
-0.26166	0.377573	-0.21092	-0.01739	0.023095	-0.04062	0.238411	0.253991	0.280517
0.002963	1.21E-05	0.017297	0.846098	0.796615	0.650267	0.00695	0.003959	0.001401
-0.62023	0.541344	-0.36672	0.378312	-0.33168	0.183654	0.519283	0.301642	0.520451
7.47E-15	4.99E-11	2.23E-05	1.16E-05	0.000139	0.03875	3.97E-10	0.000568	3.57E-10
-0.13307	0.300525	-0.26061	0.030695	-0.09458	-0.32124	0.162908	0.004542	-0.04525
0.135848	0.000597	0.003085	0.73192	0.290193	0.000231	0.067254	0.959583	0.613414
-0.1184	0.256241	-0.00536	0.053399	0.130939	-0.20721	-0.10574	-0.15575	-0.08972
0.18493	0.003639	0.952278	0.551008	0.142284	0.019416	0.236762	0.080379	0.315812
0.561905	-0.27894	0.406566	-0.70372	0.589599	-0.281	-0.41745	-0.41285	-0.52989
6.27E-12	0.001495	2.11E-06	2.79E-20	3.03E-13	0.001373	1.05E-06	1.41E-06	1.49E-10
-0.19825	0.186062	-0.18414	-0.18567	-0.19341	0.096674	0.455073	0.150207	0.257923
0.02546	0.036224	0.038226	0.036627	0.029358	0.279595	7.65E-08	0.091875	0.003416
0.147135	-0.28747	0.141155	-0.23485	0.027262	0.193332	0.011947	0.017592	0.069029
0.098795	0.001049	0.113435	0.007867	0.760941	0.029421	0.893947	0.844371	0.440622
0.425124	-0.36944	0.119335	-0.62099	0.357443	-0.09147	-0.10036	-0.16837	-0.11871
6.29E-07	1.92E-05	0.181445	6.78E-15	3.7E-05	0.306435	0.261589	0.058457	0.183768
-0.38507	0.426398	-0.34379	0.295642	-0.43014	0.481651	0.740151	0.524893	0.644272
7.81E-06	5.78E-07	7.58E-05	0.000739	4.49E-07	9.88E-09	2.74E-23	2.38E-10	3.03E-16

KHDRBS1	KIAA0586	KIAA1958	KIF11	KIF14	KIF15	KIF16B	KIF18A	KIF18B
blue	blue	blue	blue	blue	blue	blue	blue	blue
-0.32163	-0.13232	-0.29104	-0.44639	-0.49786	-0.49761	0.318175	-0.40279	-0.50911
0.000227	0.138076	0.000902	1.44E-07	2.6E-09	2.65E-09	0.000267	2.67E-06	9.84E-10
-0.30027	-0.14379	-0.27446	-0.44602	-0.48785	-0.46535	0.261272	-0.39161	-0.49806
0.000604	0.106781	0.001792	1.48E-07	5.97E-09	3.54E-08	0.003007	5.3E-06	2.55E-09
0.853298	0.652843	0.650101	0.91092	0.911946	0.94848	-0.56002	0.877443	0.865471
3.71E-37	9.03E-17	1.34E-16	6.78E-50	3.4E-50	3.02E-64	7.63E-12	1.07E-41	2.45E-39
0.568794	0.661232	0.602656	0.64122	0.652275	0.674122	-0.29724	0.763347	0.449927
3.03E-12	2.65E-17	6.56E-14	4.63E-16	9.8E-17	3.74E-18	0.00069	1.76E-25	1.12E-07
0.06629	-0.29414	-0.10498	-0.16939	-0.20766	-0.01857	-0.19406	0.061157	-0.24683
0.459013	0.000789	0.240149	0.056936	0.019145	0.83583	0.028804	0.49459	0.005149
0.285731	0.264916	0.315405	0.250384	0.223025	0.333353	-0.18689	0.52659	-0.01403
0.001129	0.002613	0.000304	0.004523	0.011724	0.000128	0.035391	2.03E-10	0.875603
0.081745	0.122435	0.139051	-0.02531	-0.0377	0.057495	0.016565	0.204186	-0.16229
0.360908	0.170283	0.118968	0.77761	0.673901	0.520833	0.853353	0.0213	0.068318
-0.1933	-0.26574	0.135434	-0.18717	-0.19132	-0.1807	0.22927	-0.0199	-0.30485
0.02945	0.002531	0.12897	0.035108	0.031188	0.04205	0.009519	0.824277	0.000492
0.27136	0.245472	-0.03592	0.321159	0.420273	0.277039	0.03635	0.348391	0.349106
0.002029	0.005408	0.688447	0.000232	8.69E-07	0.001615	0.684941	5.97E-05	5.75E-05
-0.29257	-0.25958	-0.32797	-0.31737	-0.23805	-0.3479	0.495385	-0.1595	-0.29528
0.000844	0.003208	0.000167	0.000277	0.007038	6.13E-05	3.2E-09	0.073271	0.000751
-0.14652	0.015143	-0.14459	-0.13665	-0.11474	-0.15053	0.29644	0.033618	-0.21952
0.100237	0.865817	0.104825	0.125548	0.198951	0.091172	0.000714	0.7075	0.013146
0.047339	0.098278	-0.29077	-0.07333	-0.00746	-0.07443	-0.15651	-0.21934	0.121379
0.597152	0.27166	0.000912	0.412599	0.933703	0.405625	0.078886	0.013226	0.174027
0.386134	0.310256	0.178606	0.388145	0.47397	0.347038	-0.08564	0.173986	0.627264
7.34E-06	0.000385	0.04453	6.51E-06	1.82E-08	6.41E-05	0.33841	0.050431	3.01E-15
0.061545	-0.30958	-0.21033	-0.04079	0.047885	0.007794	-0.04576	-0.10828	0.227961
0.491846	0.000397	0.01762	0.648911	0.592919	0.9307	0.609485	0.225625	0.009948
-0.09457	-0.20757	-0.25993	-0.13171	0.008362	-0.17659	0.366489	-0.19186	0.134164
0.290224	0.019197	0.003165	0.139921	0.925658	0.047028	2.26E-05	0.030708	0.13263
-0.52735	-0.42558	-0.27227	-0.60132	-0.6018	-0.61705	0.39306	-0.66184	-0.48507
1.89E-10	6.11E-07	0.001957	7.7E-14	7.27E-14	1.12E-14	4.85E-06	2.42E-17	7.49E-09
0.15022	0.042012	0.1804	0.147359	0.105538	0.175674	-0.34057	-0.09191	0.307611
0.091848	0.639083	0.042399	0.098277	0.237646	0.048204	8.93E-05	0.304107	0.000435
-0.10181	0.122844	0.18176	0.062059	-0.07584	0.036356	-0.25318	-0.00963	-0.15088
0.254708	0.16885	0.040841	0.488231	0.396778	0.68489	0.00408	0.914448	0.090422
-0.38731	-0.28289	-0.22458	-0.32907	-0.45169	-0.36038	-0.07611	-0.50134	-0.34074
6.85E-06	0.00127	0.011137	0.000158	9.8E-08	3.16E-05	0.395036	1.93E-09	8.86E-05
0.620688	0.377737	0.319527	0.440763	0.439155	0.487201	-0.3941	0.290313	0.503713
7.05E-15	1.2E-05	0.00025	2.15E-07	2.4E-07	6.3E-09	4.56E-06	0.00093	1.57E-09

KIF20A	KIF20B	KIF22	KIF23	KIF24	KIF2C	KIF4A	KIFC1	KLF12
blue	blue	blue	blue	blue	blue	blue	blue	blue
-0.43092	-0.31154	-0.32748	-0.42333	-0.37079	-0.50472	-0.48385	-0.45797	-0.36959
4.25E-07	0.000363	0.000171	7.09E-07	1.78E-05	1.44E-09	8.27E-09	6.17E-08	1.9E-05
-0.44539	-0.39166	-0.22383	-0.41627	-0.37366	-0.47643	-0.48296	-0.42216	-0.35183
1.55E-07	5.28E-06	0.011418	1.13E-06	1.51E-05	1.5E-08	8.89E-09	7.67E-07	4.99E-05
0.872715	0.741253	0.673238	0.880074	0.670345	0.925254	0.872855	0.859318	0.691697
9.74E-41	2.18E-23	4.29E-18	2.99E-42	6.7E-18	1.84E-54	9.14E-41	3.29E-38	2.19E-19
0.612581	0.625283	0.108856	0.619802	0.561722	0.570509	0.622036	0.455321	0.458087
1.96E-14	3.9E-15	0.223134	7.89E-15	6.39E-12	2.52E-12	5.93E-15	7.51E-08	6.12E-08
-0.14963	-0.14506	0.184471	-0.10351	-0.13374	-0.03056	-0.11425	-0.15287	-0.26722
0.093135	0.103693	0.037877	0.246845	0.133881	0.733047	0.200898	0.0862	0.002389
0.247603	0.321126	-0.07686	0.275778	0.231645	0.23568	0.267492	0.073537	0.036508
0.005007	0.000232	0.390429	0.0017	0.008782	0.007644	0.002364	0.411282	0.683646
0.003268	-0.03301	-0.11871	0.027419	0.02725	-0.05062	-0.01946	-0.13849	0.003895
0.97091	0.71258	0.183762	0.759607	0.76104	0.571918	0.828115	0.120471	0.965337
-0.19816	-0.09499	-0.38911	-0.19674	0.369035	-0.24047	0.019795	-0.30798	-0.20343
0.025528	0.288079	6.15E-06	0.026626	1.96E-05	0.006464	0.825174	0.000428	0.021798
0.511352	0.410784	-0.01728	0.432537	0.198576	0.337361	0.335501	0.316195	0.019479
8.08E-10	1.61E-06	0.847074	3.81E-07	0.025218	0.000105	0.000115	0.000293	0.827923
-0.15529	-0.01706	-0.44532	-0.23483	-0.1898	-0.3019	-0.2348	-0.31178	-0.62237
0.081286	0.849063	1.55E-07	0.007871	0.032581	0.000562	0.007879	0.000359	5.68E-15
-0.01018	0.06128	-0.42973	-0.05892	0.009465	-0.17104	-0.0621	-0.17975	-0.35727
0.909589	0.493722	4.61E-07	0.510548	0.915887	0.054517	0.487947	0.043158	3.73E-05
0.034082	-0.23233	0.230226	-0.03603	0.04361	0.00122	-0.05752	0.082055	0.00742
0.703651	0.008578	0.009216	0.68757	0.626376	0.989139	0.52065	0.35908	0.934017
0.414961	0.277741	0.465406	0.370451	0.348176	0.414471	0.371813	0.510453	0.348509
1.23E-06	0.00157	3.52E-08	1.81E-05	6.04E-05	1.27E-06	1.68E-05	8.75E-10	5.94E-05
0.032963	-0.07898	0.448593	-0.0022	-0.07301	0.136953	0.008409	0.192111	-0.18838
0.712945	0.377445	1.23E-07	0.980412	0.414664	0.124694	0.925245	0.030482	0.033923
0.014752	-0.00219	0.035794	-0.08258	-0.04649	-0.05777	-0.06513	0.043483	-0.36347
0.869249	0.980492	0.689511	0.356018	0.603711	0.518818	0.466894	0.62738	2.67E-05
-0.68743	-0.55051	-0.22774	-0.67585	-0.46075	-0.63584	-0.60531	-0.51473	-0.38945
4.44E-19	2.01E-11	0.010021	2.85E-18	5.01E-08	9.63E-16	4.77E-14	5.98E-10	6.03E-06
0.009898	-0.11455	0.471701	0.049897	0.047654	0.1736	0.104826	0.245814	0.459379
0.912057	0.199708	2.17E-08	0.577462	0.594705	0.050953	0.240844	0.005342	5.56E-08
-0.11986	-0.06428	-0.12293	-0.03087	-0.11539	-0.05518	-0.04262	-0.08903	0.395996
0.179529	0.472798	0.168545	0.730487	0.196415	0.537804	0.634239	0.319554	4.06E-06
-0.45294	-0.4829	-0.10255	-0.38199	-0.37605	-0.37038	-0.39451	-0.31755	0.116433
8.95E-08	8.94E-09	0.251267	9.36E-06	1.32E-05	1.82E-05	4.44E-06	0.000275	0.192371
0.339742	0.32168	0.655599	0.372971	0.244334	0.463729	0.32603	0.440115	0.58019
9.32E-05	0.000226	6.06E-17	1.57E-05	0.005634	4E-08	0.000184	2.25E-07	8.76E-13

KLHDC8A	KLHL13	KLHL7	KLHL8	KNSTRN	KNTC1	KPNA2	KPNB1	LAMA1
blue	blue	blue	blue	blue	blue	blue	blue	blue
-0.35588	-0.13355	-0.32148	-0.33785	-0.28764	-0.29702	-0.29662	-0.38621	-0.34782
4.02E-05	0.134429	0.000228	0.000102	0.001042	0.000696	0.000709	7.31E-06	6.16E-05
-0.34357	-0.05382	-0.23601	-0.37342	-0.19933	-0.28066	-0.23262	-0.46351	-0.31421
7.67E-05	0.547845	0.007556	1.53E-05	0.024658	0.001393	0.008495	4.07E-08	0.000321
0.65667	0.52934	0.715264	0.593145	0.696728	0.860246	0.803947	0.618308	0.574372
5.19E-17	1.57E-10	3.49E-21	2.02E-13	9.36E-20	2.24E-38	5.41E-30	9.54E-15	1.66E-12
0.480423	0.390782	0.656112	0.506314	0.402875	0.745911	0.45963	0.49278	0.499563
1.09E-08	5.56E-06	5.63E-17	1.26E-09	2.65E-06	8.23E-24	5.45E-08	3.98E-09	2.25E-09
-0.05601	0.128252	0.151624	-0.22083	0.213536	-0.10227	-0.06571	-0.56597	-0.17836
0.531701	0.150725	0.088817	0.0126	0.01593	0.252585	0.462958	4.09E-12	0.044832
0.171598	0.281122	0.509608	0.147066	0.318411	0.40335	0.162978	-0.06049	0.169834
0.053728	0.001367	9.42E-10	0.098953	0.000264	2.58E-06	0.067135	0.499337	0.056275
0.110307	0.144741	0.258108	-0.08546	0.004599	0.117579	-0.03572	-0.14682	0.133075
0.21699	0.104465	0.003392	0.339397	0.959073	0.188	0.690084	0.099516	0.135832
0.007497	0.024194	-0.12721	-0.19992	-0.22734	-0.02884	-0.27219	-0.03544	-0.03433
0.933329	0.787162	0.154086	0.024225	0.010157	0.747579	0.001963	0.692439	0.701602
0.238392	-0.19538	0.010998	0.098713	-0.08216	0.41408	0.32493	0.566895	0.12643
0.006955	0.02771	0.902328	0.269532	0.358439	1.3E-06	0.000194	3.71E-12	0.156659
-0.2197	-0.45882	-0.51101	-0.4289	-0.47205	-0.10547	-0.30312	-0.01161	-0.27199
0.013074	5.79E-08	8.33E-10	4.88E-07	2.11E-08	0.237956	0.000532	0.896882	0.001979
-0.02734	-0.27527	-0.2555	-0.26363	-0.3368	0.051488	-0.22054	0.080091	-0.07988
0.760256	0.001735	0.003742	0.002747	0.000108	0.565369	0.012719	0.370734	0.372006
0.079824	-0.22941	-0.17572	-0.09158	-0.1632	-0.05085	0.005463	0.1938	0.022227
0.372339	0.009473	0.048148	0.305812	0.066763	0.570192	0.951396	0.029024	0.804107
0.412049	-0.00112	-0.08315	0.2175	0.037798	0.338416	0.351653	0.63953	0.302001
1.48E-06	0.990034	0.35271	0.014036	0.673093	9.96E-05	5.03E-05	5.84E-16	0.000559
0.184517	-0.23628	-0.3363	-0.12904	-0.13104	-0.13635	0.024598	0.048341	-0.07655
0.037829	0.007485	0.000111	0.148223	0.141966	0.126388	0.783697	0.5894	0.392338
0.054126	-0.47204	-0.63813	-0.32304	-0.45742	-0.07966	-0.10329	0.309472	-0.1053
0.545594	2.11E-08	7.06E-16	0.000212	6.43E-08	0.373319	0.247825	0.0004	0.2387
-0.45466	-0.26187	-0.51023	-0.42829	-0.37034	-0.6347	-0.42463	-0.42603	-0.27655
7.89E-08	0.00294	8.92E-10	5.09E-07	1.82E-05	1.12E-15	6.5E-07	5.92E-07	0.001648
0.140696	0.317834	0.199711	0.229385	0.244809	-0.12336	0.227901	0.024191	0.184966
0.114625	0.000271	0.024379	0.009482	0.005538	0.167056	0.009968	0.787186	0.037356
-0.17524	0.362194	0.487434	0.39547	0.302435	-0.10655	0.014091	-0.26409	0.025735
0.048772	2.86E-05	6.18E-09	4.19E-06	0.000549	0.233165	0.875058	0.002698	0.773962
-0.40301	0.096913	-0.00433	-0.00218	0.017349	-0.52894	-0.23074	-0.46485	-0.25038
2.63E-06	0.278406	0.961505	0.980611	0.846491	1.63E-10	0.009057	3.68E-08	0.004523
0.240322	0.420705	0.368795	0.405527	0.546236	0.367174	0.488467	0.153095	0.313878
0.006498	8.45E-07	1.99E-05	2.25E-06	3.08E-11	2.17E-05	5.68E-09	0.085728	0.000326

LAPTM4B	LBR	LCLAT1	LCORL	LDHD	LFNG	LIG3	LIMA1	LIN9
blue	blue	blue	blue	blue	blue	blue	blue	blue
-0.31858	-0.35301	-0.33141	-0.34671	0.143773	-0.3691	-0.28185	-0.26118	-0.37573
0.000262	4.69E-05	0.000141	6.52E-05	0.10683	1.95E-05	0.001326	0.003018	1.34E-05
-0.30846	-0.392	-0.25479	-0.34318	0.201409	-0.36408	-0.2437	-0.2634	-0.33144
0.000418	5.17E-06	0.003842	7.82E-05	0.023169	2.58E-05	0.005763	0.002771	0.000141
0.741125	0.744736	0.623606	0.670007	-0.66908	0.526484	0.570812	0.781644	0.814685
2.24E-23	1.05E-23	4.84E-15	7.06E-18	8.14E-18	2.05E-10	2.44E-12	2.15E-27	2.29E-31
0.582483	0.648243	0.690335	0.589432	-0.68488	0.346325	0.305642	0.680529	0.69529
6.79E-13	1.74E-16	2.75E-19	3.09E-13	6.74E-19	6.65E-05	0.000475	1.36E-18	1.2E-19
0.003541	-0.10314	0.030243	-0.01406	0.128288	-0.20764	-0.25102	-0.11559	0.237405
0.96848	0.248538	0.73572	0.875375	0.150609	0.01916	0.004418	0.195616	0.007199
0.341555	0.34996	0.519421	0.366771	-0.40087	-0.01261	-0.12475	0.344086	0.566306
8.5E-05	5.5E-05	3.92E-10	2.22E-05	3.01E-06	0.888085	0.162289	7.47E-05	3.95E-12
0.131938	0.034827	0.182946	0.050297	-0.21602	0.006156	0.05751	0.177277	0.190672
0.139239	0.697486	0.039521	0.574407	0.014717	0.945233	0.520728	0.046165	0.031773
-0.20643	0.02192	0.033331	-0.07253	0.068459	-0.23945	-0.30732	-0.36214	-0.0554
0.019886	0.806759	0.709887	0.417746	0.444415	0.006701	0.000441	2.87E-05	0.53616
0.295208	0.407609	0.150664	0.017911	-0.62187	0.269165	0.194859	0.512626	0.079621
0.000753	1.97E-06	0.090881	0.841583	6.06E-15	0.002213	0.02814	7.22E-10	0.37356
-0.2558	0.040958	-0.29287	-0.34566	-0.03769	-0.21336	-0.47675	-0.10612	-0.39883
0.0037	0.647529	0.000833	6.88E-05	0.674	0.016021	1.46E-08	0.235062	3.41E-06
-0.14073	0.051568	-0.03116	-0.19007	-0.15395	-0.02929	-0.37402	0.00483	-0.16376
0.114539	0.564765	0.727983	0.032322	0.083962	0.743734	1.48E-05	0.957024	0.065818
-0.04907	-0.26134	-0.1481	-0.4029	-0.08075	0.258956	0.426875	0.196448	-0.31735
0.583811	0.003	0.096573	2.65E-06	0.366826	0.003285	5.6E-07	0.026858	0.000277
0.186087	0.308966	-0.07319	0.083071	-0.22494	0.462059	0.55937	0.331331	-0.01772
0.036199	0.000409	0.413523	0.353145	0.011003	4.54E-08	8.16E-12	0.000142	0.84324
-0.1275	-0.00603	-0.34308	-0.16874	0.257215	0.227644	0.070603	-0.16134	-0.15994
0.153165	0.946379	7.86E-05	0.05791	0.003508	0.010055	0.430243	0.06997	0.07247
-0.20795	0.085817	-0.48533	-0.33436	0.070892	0.134388	-0.05459	-0.06686	-0.49562
0.018978	0.3374	7.34E-09	0.000122	0.428356	0.13198	0.54218	0.455157	3.13E-09
-0.44605	-0.47552	-0.61064	-0.38108	0.667659	-0.40317	-0.32224	-0.5427	-0.62083
1.47E-07	1.61E-08	2.49E-14	9.87E-06	1.01E-17	2.61E-06	0.00022	4.37E-11	6.92E-15
0.143296	-0.1282	-0.04712	0.135915	0.264828	0.20126	0.377664	-0.02302	0.053117
0.108009	0.150889	0.598859	0.127604	0.002622	0.023273	1.2E-05	0.797291	0.553118
0.090713	-0.18266	0.350294	0.295334	0.092154	-0.12457	0.012441	-0.08455	0.304984
0.310456	0.039832	5.41E-05	0.000749	0.302798	0.162905	0.88959	0.34458	0.000489
-0.28561	-0.59163	-0.16232	-0.17427	0.489867	-0.26556	-0.04691	-0.47277	-0.20574
0.001134	2.4E-13	0.068254	0.050055	5.06E-09	0.002549	0.600496	2E-08	0.020311
0.458713	0.32153	0.134811	0.404344	-0.33193	0.130786	0.564084	0.574495	0.386669
5.84E-08	0.000228	0.130756	2.42E-06	0.000138	0.142753	4.99E-12	1.64E-12	7.11E-06

LIX1L	LMNB1	LPL	LRR1	LRRK1	LRRN1	LSM14A	LSM8	LVRN
blue	blue	blue	blue	blue	blue	blue	blue	blue
-0.31329	-0.45991	-0.35981	-0.15235	0.39004	-0.41996	-0.2259	-0.29026	-0.21383
0.000335	5.34E-08	3.26E-05	0.087279	5.82E-06	8.87E-07	0.010657	0.000932	0.015782
-0.31833	-0.47333	-0.35517	-0.1294	0.273717	-0.36722	-0.28331	-0.24406	-0.2495
0.000265	1.91E-08	4.18E-05	0.147073	0.001847	2.17E-05	0.001248	0.005689	0.004672
0.720282	0.879167	0.659412	0.733733	-0.62136	0.675404	0.52202	0.713979	0.705176
1.37E-21	4.64E-42	3.47E-17	1.01E-22	6.47E-15	3.06E-18	3.09E-10	4.43E-21	2.16E-20
0.66191	0.525173	0.62131	0.678354	-0.44673	0.421765	0.504067	0.805873	0.69069
2.4E-17	2.32E-10	6.51E-15	1.92E-18	1.4E-07	7.87E-07	1.53E-09	3.11E-30	2.59E-19
-0.00471	-0.26302	-0.08945	0.332406	-0.24789	-0.17707	-0.57201	0.111009	-0.1183
0.958078	0.002812	0.317288	0.000134	0.004955	0.046431	2.15E-12	0.214057	0.185276
0.397256	0.072264	0.324398	0.648892	-0.32484	0.03815	-0.03142	0.620491	0.372914
3.76E-06	0.419452	0.000199	1.59E-16	0.000194	0.670226	0.725851	7.23E-15	1.58E-05
0.259201	-0.13959	0.088072	0.305906	-0.24347	-0.02658	-0.05618	0.430547	0.166381
0.003254	0.117524	0.324811	0.00047	0.005811	0.766763	0.530429	4.36E-07	0.061549
-0.19384	-0.25194	-0.2572	-0.1184	0.043684	-0.18816	-0.01205	-0.00662	-0.21263
0.028988	0.004271	0.00351	0.184927	0.625789	0.034136	0.893007	0.9411	0.016392
0.39972	0.438368	0.262553	0.355533	-0.08718	-0.12093	0.505757	0.267385	0.436093
3.23E-06	2.54E-07	0.002863	4.1E-05	0.329733	0.175621	1.32E-09	0.002373	2.98E-07
-0.15393	-0.2087	-0.20794	0.088755	0.406878	-0.62004	0.016702	-0.14145	-0.13839
0.084017	0.018538	0.018983	0.321058	2.06E-06	7.66E-15	0.852154	0.112686	0.120747
0.022516	-0.07466	-0.02702	0.127837	0.335965	-0.32777	0.108219	0.031879	0.075827
0.801608	0.40417	0.762983	0.152061	0.000113	0.000169	0.225868	0.721997	0.396828
0.214762	0.076591	0.079455	-0.21249	0.051895	0.045088	0.200825	-0.11006	-0.00576
0.015322	0.392075	0.374562	0.016464	0.562297	0.614721	0.023579	0.218023	0.948775
0.262754	0.576592	0.244116	0.026504	-0.16867	0.343245	0.625845	0.046477	0.260688
0.002841	1.3E-12	0.005678	0.767396	0.058008	7.79E-05	3.62E-15	0.603853	0.003075
-0.14985	0.163036	-0.03347	-0.07825	-0.1495	-0.03409	0.008753	-0.30436	-0.14242
0.092653	0.067037	0.708707	0.38186	0.093437	0.703556	0.922199	0.000503	0.110217
-0.11953	0.132109	-0.11099	-0.12266	0.222143	-0.29998	0.326173	-0.32051	-0.13067
0.180737	0.138722	0.214144	0.169483	0.012069	0.000612	0.000182	0.000239	0.143103
-0.5877	-0.56073	-0.42493	-0.56713	0.456024	-0.246	-0.35182	-0.55303	-0.65799
3.77E-13	7.09E-12	6.38E-07	3.62E-12	7.13E-08	0.005305	4.99E-05	1.56E-11	4.28E-17
-0.05607	0.180915	0.055355	-0.30476	-0.13189	0.575174	-0.01516	-0.18333	-0.11218
0.531243	0.041803	0.53649	0.000494	0.139377	1.52E-12	0.865707	0.039104	0.209231
-0.09237	-0.16145	0.024005	-0.1766	-0.01964	0.315088	-0.29035	0.071997	0.000868
0.301664	0.069767	0.788791	0.04702	0.826504	0.000308	0.000929	0.421172	0.992271
-0.46709	-0.42193	-0.39824	-0.62428	0.228341	0.064478	-0.50604	-0.45729	-0.43986
3.1E-08	7.79E-07	3.54E-06	4.44E-15	0.009822	0.471401	1.29E-09	6.49E-08	2.29E-07
0.440345	0.3967	0.288611	0.347632	-0.37858	0.428611	0.089878	0.309878	0.349426
2.21E-07	3.89E-06	0.001	6.21E-05	1.14E-05	4.98E-07	0.314948	0.000392	5.66E-05

LY6G5C	MAD2L1	MAD2L2	MAGED1	MAML2	MANSC1	MAOA	MAP3K1	44257
blue	blue	blue	blue	blue	blue	blue	blue	blue
-0.12369	-0.4091	-0.36621	-0.2903	-0.34502	0.243888	0.013575	-0.26161	0.187149
0.165906	1.79E-06	2.29E-05	0.00093	7.11E-05	0.005724	0.879596	0.002969	0.035129
-0.06658	-0.35265	-0.31893	-0.16882	-0.43386	0.285512	0.127604	-0.30596	0.229702
0.457046	4.78E-05	0.000258	0.057781	3.47E-07	0.001139	0.152815	0.000468	0.009381
0.537445	0.812478	0.664856	0.426393	0.72022	-0.61305	-0.58514	0.695433	-0.63099
7.28E-11	4.45E-31	1.54E-17	5.78E-07	1.39E-21	1.85E-14	5.04E-13	1.17E-19	1.84E-15
0.443737	0.552509	0.184445	0.050498	0.634854	-0.29339	-0.46437	0.583813	-0.49741
1.74E-07	1.65E-11	0.037905	0.572878	1.1E-15	0.000815	3.81E-08	5.85E-13	2.7E-09
0.326981	0.224914	0.408292	-0.14364	-0.28862	-0.03135	0.022529	-0.15016	0.287706
0.000175	0.011014	1.89E-06	0.107169	0.000999	0.726396	0.801499	0.091976	0.001039
0.427033	0.429041	0.145677	-0.20382	0.20819	-0.05595	-0.24368	0.233046	-0.08911
5.54E-07	4.83E-07	0.102219	0.021542	0.018835	0.532111	0.005768	0.008371	0.319097
0.288823	0.098213	-0.00425	-0.09489	0.047095	0.204763	-0.0503	0.089721	-0.05955
0.000991	0.27198	0.962188	0.288613	0.59904	0.020929	0.57438	0.315795	0.506003
0.04307	-0.17751	-0.30125	-0.2054	-0.03827	0.379681	0.283654	-0.21112	0.211479
0.630664	0.045871	0.000578	0.020524	0.669214	1.07E-05	0.001231	0.017189	0.016998
0.104315	0.085856	0.124274	-0.0278	0.453779	-0.28156	-0.42158	0.206935	-0.3878
0.243157	0.337177	0.163907	0.756378	8.42E-08	0.001342	7.97E-07	0.01958	6.65E-06
-0.00648	-0.41793	-0.20446	-0.50236	0.037272	0.083021	-0.03185	-0.21172	0.221326
0.9424	1.01E-06	0.021125	1.77E-09	0.677389	0.353435	0.722267	0.01687	0.012397
0.016141	-0.25266	-0.29779	-0.39235	0.111032	0.058491	-0.16018	-0.07309	0.099561
0.857062	0.00416	0.000673	5.06E-06	0.213964	0.513627	0.072035	0.414153	0.265421
0.011603	-0.27449	0.015503	0.157353	0.078872	-0.12689	-0.13363	-0.0664	-0.30445
0.896987	0.001791	0.862658	0.077266	0.378085	0.155137	0.1342	0.45829	0.000501
0.064145	0.040337	0.297928	0.380017	0.454652	-0.45122	-0.25291	0.288624	-0.43645
0.473702	0.652521	0.000669	1.05E-05	7.89E-08	1.02E-07	0.00412	0.000999	2.9E-07
-0.02741	-0.06131	0.469608	0.109829	-0.10148	-0.42862	-0.0202	-0.10689	0.163997
0.759688	0.493517	2.55E-08	0.218999	0.256251	4.97E-07	0.821671	0.231655	0.065421
-0.11023	-0.42572	0.074139	-0.07764	0.119251	-0.24049	-0.03856	-0.1386	0.052492
0.217317	6.05E-07	0.407455	0.385576	0.181754	0.006459	0.666857	0.120183	0.557801
-0.34808	-0.56869	-0.32708	-0.1041	-0.5515	0.404342	0.418273	-0.40158	0.440638
6.07E-05	3.06E-12	0.000175	0.24412	1.82E-11	2.42E-06	9.91E-07	2.88E-06	2.17E-07
-0.0888	0.170886	0.245717	0.505899	-0.01452	-0.09176	0.058813	0.168259	-0.10952
0.3208	0.054744	0.00536	1.3E-09	0.871323	0.304859	0.511307	0.058633	0.2203
-0.16687	0.2838	-0.27275	0.113575	-0.21672	0.295147	0.130338	0.120036	0.023297
0.060779	0.001223	0.00192	0.203589	0.014393	0.000755	0.144138	0.178874	0.794874
-0.41376	-0.12181	-0.30228	0.152921	-0.52826	0.432818	0.433713	-0.26507	0.249587
1.33E-06	0.172508	0.000552	0.086089	1.74E-10	3.73E-07	3.51E-07	0.002597	0.004657
0.349356	0.45022	0.611718	0.320215	0.363288	-0.40998	-0.41079	0.535712	-0.60822
5.68E-05	1.09E-07	2.18E-14	0.000242	2.69E-05	1.7E-06	1.61E-06	8.59E-11	3.35E-14

MARCKS	MASTL	MBTD1	MCM10	MCM2	MCM3	MCM4	MCM6	MCM7
blue	blue	blue	blue	blue	blue	blue	blue	blue
-0.34452	-0.04812	-0.25674	-0.4269	-0.47277	-0.23668	-0.49742	-0.29465	-0.38788
7.3E-05	0.591071	0.003572	5.59E-07	2E-08	0.007383	2.69E-09	0.000772	6.62E-06
-0.40542	-0.04968	-0.24777	-0.39524	-0.51085	-0.25148	-0.5138	-0.27441	-0.36922
2.26E-06	0.579156	0.004977	4.25E-06	8.45E-10	0.004344	6.5E-10	0.001796	1.94E-05
0.632076	0.64913	0.690568	0.893482	0.853585	0.79711	0.819227	0.816591	0.828923
1.6E-15	1.53E-16	2.64E-19	2.78E-45	3.31E-37	3.66E-29	5.64E-32	1.28E-31	2.48E-33
0.490297	0.420321	0.610471	0.502661	0.487132	0.570813	0.545179	0.632588	0.439354
4.88E-09	8.66E-07	2.54E-14	1.72E-09	6.34E-09	2.44E-12	3.42E-11	1.49E-15	2.37E-07
-0.12966	0.105495	-0.16107	-0.15142	-0.10688	-0.06533	-0.2806	0.147211	-0.08192
0.146256	0.237837	0.070439	0.08925	0.231681	0.465534	0.001396	0.098617	0.35988
0.217698	0.284708	0.248171	0.100133	0.113354	0.266822	0.085783	0.466694	0.050195
0.013946	0.001178	0.004904	0.26267	0.204479	0.002426	0.337591	3.19E-08	0.575185
-0.08105	0.064667	0.121737	-0.13833	-0.10399	0.063771	-0.14236	0.123954	-0.04017
0.364996	0.470103	0.17275	0.120906	0.24463	0.476288	0.110363	0.165004	0.653872
-0.11709	-0.34922	-0.24313	-0.30242	-0.07917	0.043519	-0.08307	-0.03122	-0.21409
0.189868	5.72E-05	0.005882	0.000549	0.376303	0.6271	0.353166	0.727517	0.015651
0.193378	0.162953	0.143461	0.262311	0.381272	0.400947	0.257609	0.297741	0.245245
0.029382	0.067178	0.107601	0.00289	9.76E-06	2.99E-06	0.003457	0.000675	0.005452
-0.12177	-0.21634	-0.3282	-0.39181	-0.18809	-0.02183	-0.43126	-0.22245	-0.25942
0.172643	0.014568	0.000165	5.23E-06	0.034208	0.80754	4.15E-07	0.011947	0.003228
0.043099	-0.11794	-0.19348	-0.23469	-0.05782	0.131761	-0.18952	-0.01722	-0.15462
0.63043	0.186641	0.029294	0.007911	0.5185	0.139775	0.032844	0.847584	0.082617
-0.1542	0.016594	0.00173	0.079072	0.080957	0.018116	0.110819	-0.12441	0.168401
0.083465	0.853097	0.984595	0.376877	0.36557	0.839799	0.214848	0.163454	0.058416
0.23272	0.149305	0.299635	0.495595	0.538959	0.414008	0.452473	0.121057	0.610566
0.008465	0.093866	0.000621	3.14E-09	6.29E-11	1.31E-06	9.26E-08	0.175181	2.51E-14
0.042945	-0.14838	-0.21443	0.102423	0.295439	0.064717	0.052505	-0.10281	0.319407
0.631657	0.095949	0.015487	0.251858	0.000746	0.469758	0.557705	0.250065	0.000252
-0.02195	-0.24275	-0.22397	-0.07169	0.154864	0.11179	-0.12428	-0.27826	0.161298
0.80653	0.005962	0.011365	0.423168	0.082127	0.210833	0.163903	0.001537	0.070042
-0.38836	-0.41844	-0.31405	-0.54302	-0.59269	-0.50526	-0.54886	-0.68125	-0.39889
6.43E-06	9.8E-07	0.000324	4.23E-11	2.12E-13	1.38E-09	2.38E-11	1.21E-18	3.4E-06
0.100769	0.075785	0.170775	0.293586	0.137052	-0.04876	0.282444	-0.05698	0.263119
0.259635	0.397089	0.054904	0.000808	0.124418	0.58619	0.001294	0.524582	0.002801
0.054652	0.09094	0.164882	0.016571	-0.24045	-0.28133	0.134461	0.055564	-0.25988
0.541686	0.309242	0.06396	0.853298	0.006469	0.001355	0.131768	0.53495	0.003172
-0.32804	-0.17847	-0.22603	-0.25017	-0.45995	-0.55464	-0.18311	-0.3363	-0.41986
0.000167	0.044693	0.010612	0.004559	5.33E-08	1.33E-11	0.039345	0.000111	8.93E-07
0.16754	0.717534	0.616357	0.566756	0.382278	0.247786	0.312801	0.325677	0.486558
0.059736	2.29E-21	1.22E-14	3.76E-12	9.2E-06	0.004974	0.000343	0.000187	6.64E-09

MCM8	MDFI	ME1	MELK	MEST	METTL1	METTL3	MEX3A	MFSD9
blue	blue	blue	blue	blue	blue	blue	blue	blue
-0.28106	-0.43763	0.362498	-0.41783	-0.11402	-0.05233	-0.27763	-0.42203	-0.23578
0.00137	2.67E-07	2.81E-05	1.02E-06	0.201797	0.559015	0.001577	7.74E-07	0.007617
-0.33905	-0.42339	0.372734	-0.4068	-0.07524	-0.04195	-0.22106	-0.3516	-0.30576
9.65E-05	7.07E-07	1.59E-05	2.08E-06	0.400507	0.639619	0.012506	5.05E-05	0.000473
0.861859	0.714502	-0.72376	0.893639	0.640087	0.500806	0.662204	0.833727	0.706622
1.14E-38	4.02E-21	7.09E-22	2.54E-45	5.41E-16	2.02E-09	2.3E-17	4.9E-34	1.67E-20
0.750819	0.351358	-0.47791	0.673563	0.672541	0.47835	0.553388	0.412908	0.670594
2.88E-24	5.11E-05	1.33E-08	4.08E-18	4.78E-18	1.29E-08	1.51E-11	1.41E-06	6.45E-18
-0.26064	0.063302	0.124684	0.003617	-0.00978	0.319127	0.001547	-0.13654	-0.26947
0.00308	0.479545	0.16251	0.967804	0.913118	0.000255	0.986225	0.125858	0.002187
0.312274	0.112628	-0.102	0.384081	0.449102	0.49546	0.264203	-0.00405	0.297751
0.000351	0.207411	0.25384	8.28E-06	1.18E-07	3.18E-09	0.002687	0.963967	0.000674
0.099353	-0.03899	0.056837	0.085108	0.290876	0.366482	0.202023	-0.12362	0.198535
0.266423	0.663394	0.525622	0.341418	0.000908	2.26E-05	0.022744	0.166149	0.025249
-0.11771	-0.36547	0.108511	-0.10865	-0.10608	-0.12224	-0.15892	-0.35724	-0.17431
0.187513	2.39E-05	0.224614	0.224006	0.235242	0.170954	0.074324	3.74E-05	0.050004
0.429726	0.432262	-0.19295	0.451822	0.438152	0.387222	0.161596	0.13104	0.56115
4.61E-07	3.88E-07	0.029746	9.71E-08	2.58E-07	6.88E-06	0.069518	0.141972	6.78E-12
-0.15233	-0.08236	0.27114	-0.10246	-0.06952	0.110251	-0.21694	-0.48915	-0.05645
0.087317	0.357308	0.002047	0.251705	0.437397	0.217221	0.01429	5.37E-09	0.528418
0.045122	-0.13552	0.122927	0.047066	0.142353	0.225101	-0.08585	-0.34188	0.080281
0.614449	0.128735	0.168558	0.599268	0.110373	0.010946	0.3372	8.36E-05	0.369596
-0.04947	0.115046	-0.07436	-0.05888	0.071744	0.15267	0.226281	0.157931	-0.09435
0.58074	0.197759	0.406077	0.510799	0.422813	0.086611	0.010524	0.076173	0.291369
0.438172	0.422352	-0.51262	0.324432	0.144441	0.00379	0.360381	0.555356	0.331527
2.58E-07	7.57E-07	7.22E-10	0.000198	0.105192	0.966265	3.16E-05	1.23E-11	0.00014
-0.15102	0.34656	-0.22582	0.03322	-0.18658	-0.08945	-0.02771	0.192403	-0.2038
0.090104	6.57E-05	0.010686	0.710806	0.035696	0.317243	0.757105	0.030226	0.021551
-0.04039	0.198301	-0.08511	-0.02362	-0.14764	-0.11681	-0.06455	-0.03416	-0.05092
0.652054	0.025425	0.341401	0.792114	0.097627	0.190928	0.470923	0.703028	0.569639
-0.60963	-0.46151	0.465775	-0.65899	-0.5159	-0.5718	-0.34771	-0.35757	-0.68769
2.82E-14	4.73E-08	3.43E-08	3.69E-17	5.39E-10	2.2E-12	6.19E-05	3.67E-05	4.26E-19
-0.05961	0.166078	-0.19542	-0.04148	-0.13496	-0.26142	0.101861	0.494177	-0.13895
0.505591	0.06203	0.027684	0.64331	0.130333	0.002991	0.254482	3.54E-09	0.119238
-0.0717	-0.29613	0.16516	-0.15495	-0.04571	-0.20863	-0.11202	-0.00097	-0.05476
0.423069	0.000724	0.063508	0.081951	0.60983	0.018581	0.2099	0.991322	0.540852
-0.50299	-0.43856	0.405838	-0.53641	-0.4462	-0.44638	-0.38567	-0.18495	-0.44008
1.68E-09	2.51E-07	2.2E-06	8.03E-11	1.46E-07	1.44E-07	7.54E-06	0.037371	2.25E-07
0.426397	0.454338	-0.44098	0.352669	0.133771	0.203284	0.470336	0.630534	0.3438
5.78E-07	8.08E-08	2.11E-07	4.77E-05	0.13378	0.021892	2.41E-08	1.96E-15	7.58E-05

MIB1	MIS18A	MKI67	MKRN1	MKRN3	MKS1	MMP16	MMS22L	MND1
blue	blue	blue	blue	blue	blue	blue	blue	blue
-0.41025	-0.28134	-0.47782	-0.20406	-0.28136	-0.26804	-0.40419	-0.33802	-0.41493
1.67E-06	0.001355	1.34E-08	0.021381	0.001353	0.002314	2.44E-06	0.000102	1.23E-06
-0.43129	-0.27946	-0.49002	-0.14884	-0.23025	-0.26218	-0.44247	-0.37399	-0.40832
4.15E-07	0.001463	5E-09	0.094911	0.00921	0.002905	1.9E-07	1.48E-05	1.89E-06
0.662815	0.764761	0.795441	0.548738	0.561112	0.647476	0.561692	0.798543	0.893802
2.1E-17	1.27E-25	5.78E-29	2.4E-11	6.81E-12	1.94E-16	6.41E-12	2.47E-29	2.32E-45
0.572956	0.575609	0.423088	0.563839	0.297786	0.537831	0.314709	0.790512	0.692622
1.94E-12	1.45E-12	7.21E-07	5.12E-12	0.000673	7.01E-11	0.000314	2.17E-28	1.88E-19
-0.31335	0.065311	-0.39434	-0.01773	-0.0421	0.109564	-0.37918	-0.08739	0.062924
0.000334	0.465683	4.49E-06	0.843146	0.638421	0.220119	1.1E-05	0.328593	0.482179
0.161624	0.361655	-0.08504	0.370613	0.052837	0.320171	-0.12556	0.489679	0.436577
0.069469	2.95E-05	0.341813	1.79E-05	0.555213	0.000243	0.15956	5.14E-09	2.88E-07
-0.06654	0.080464	-0.20243	0.294557	-0.08436	0.26295	-0.13283	0.112224	0.084826
0.45732	0.368503	0.022464	0.000775	0.345692	0.00282	0.136573	0.209054	0.34303
-0.10984	-0.2048	-0.22634	-0.13045	-0.06623	0.15232	-0.17735	0.108163	-0.1339
0.218958	0.020902	0.010503	0.143793	0.459395	0.087344	0.046076	0.226112	0.13341
0.020553	0.396563	0.416018	0.362528	-0.1512	0.325699	0.02154	0.355713	0.306633
0.818593	3.92E-06	1.15E-06	2.81E-05	0.089724	0.000187	0.810043	4.06E-05	0.000454
-0.5354	-0.06144	-0.22736	-0.12529	-0.36598	-0.0301	-0.49568	-0.02028	-0.21952
8.86E-11	0.492582	0.01015	0.160455	2.32E-05	0.736903	3.12E-09	0.820945	0.01315
-0.28987	-0.04376	-0.11294	0.008947	-0.29202	0.000297	-0.38084	0.126234	-0.09845
0.000948	0.625194	0.206139	0.920474	0.000865	0.997352	1E-05	0.157309	0.270841
-0.18167	-0.10944	0.16394	0.215619	-0.12863	0.131077	-0.06951	-0.24449	-0.14598
0.040941	0.22065	0.065515	0.014909	0.149509	0.141858	0.437434	0.005603	0.101508
0.230645	0.296097	0.653666	0.136618	0.324623	0.348505	0.392878	0.243268	0.262249
0.009086	0.000725	8.02E-17	0.125629	0.000196	5.94E-05	4.9E-06	0.005853	0.002897
-0.23159	0.054429	0.169426	-0.28439	0.094379	0.055826	-0.09839	-0.10111	-0.00125
0.008799	0.543343	0.056877	0.001194	0.291224	0.533023	0.271106	0.257999	0.988828
-0.38455	0.025027	0.191157	-0.19362	-0.0902	0.054068	-0.19482	-0.03589	-0.13218
8.06E-06	0.78002	0.031333	0.029174	0.31319	0.546025	0.028174	0.688722	0.138507
-0.4579	-0.51343	-0.50226	-0.45983	-0.17062	-0.48934	-0.24144	-0.54505	-0.57043
6.21E-08	6.72E-10	1.78E-09	5.37E-08	0.055124	5.29E-09	0.006247	3.47E-11	2.54E-12
0.270824	-0.08645	0.247541	-0.07758	0.306633	-0.10022	0.521922	-0.1896	0.013923
0.002073	0.33382	0.005018	0.385971	0.000454	0.262235	3.12E-10	0.032761	0.876536
0.482649	-0.215	-0.16249	0.000711	0.049391	-0.34417	0.320527	-0.10095	-0.04498
9.12E-09	0.015206	0.067973	0.993668	0.581334	7.43E-05	0.000239	0.258797	0.615548
0.020496	-0.5502	-0.32726	-0.31884	-0.11041	-0.53756	0.140028	-0.62935	-0.47877
0.81909	2.08E-11	0.000173	0.000259	0.216557	7.19E-11	0.116372	2.29E-15	1.24E-08
0.366139	0.484238	0.374279	0.283311	0.456669	0.376541	0.494248	0.195459	0.45805
2.3E-05	8.02E-09	1.46E-05	0.001248	6.8E-08	1.28E-05	3.52E-09	0.02765	6.14E-08

MPL	MPO	MPP1	MRO	MRPL42	MSH2	MSH6	MSTO1	MTFR2
blue	blue	blue	blue	blue	blue	blue	blue	blue
0.222399	0.377566	0.552798	0.131818	-0.26474	-0.32404	-0.33378	-0.1953	-0.25647
0.011968	1.21E-05	1.6E-11	0.139601	0.002631	0.000202	0.000126	0.027778	0.003608
0.248019	0.455006	0.514058	0.203767	-0.28061	-0.27866	-0.33065	-0.21637	-0.22779
0.004932	7.69E-08	6.35E-10	0.021573	0.001396	0.001512	0.000147	0.014552	0.010004
-0.4704	-0.68058	-0.81702	-0.62184	0.677997	0.630864	0.801095	0.612075	0.833812
2.4E-08	1.35E-18	1.12E-31	6.08E-15	2.03E-18	1.87E-15	1.21E-29	2.08E-14	4.76E-34
-0.31716	-0.61436	-0.6321	-0.47656	0.758816	0.521631	0.673129	0.565717	0.78002
0.00028	1.57E-14	1.59E-15	1.48E-08	4.93E-25	3.21E-10	4.36E-18	4.2E-12	3.23E-27
-0.22984	0.13864	0.199523	0.032787	0.274437	0.066886	-0.12226	0.114172	0.195944
0.009338	0.120073	0.024516	0.714416	0.001794	0.454975	0.17091	0.201208	0.027259
-0.29396	-0.28404	-0.1884	-0.2194	0.737089	0.359947	0.327747	0.423846	0.637932
0.000795	0.001211	0.033909	0.013199	5.12E-23	3.23E-05	0.000169	6.86E-07	7.26E-16
-0.12767	0.004938	-0.05524	-0.13655	0.311424	0.022076	0.077486	0.25967	0.310256
0.152594	0.956057	0.537369	0.125808	0.000365	0.805411	0.386552	0.003197	0.000385
0.299381	-0.0331	0.082975	0.231617	0.083444	0.096104	0.018184	0.112328	-0.05758
0.000628	0.711812	0.3537	0.00879	0.350979	0.282454	0.839205	0.20863	0.520224
-0.30149	-0.42454	-0.15026	-0.53567	0.294939	-0.1634	0.385812	0.1941	0.400263
0.000572	6.55E-07	0.091762	8.63E-11	0.000762	0.066415	7.48E-06	0.028771	3.12E-06
0.008667	0.07436	0.594792	0.076512	-0.03862	-0.51288	-0.0942	-0.07506	-0.04014
0.92296	0.40605	1.66E-13	0.392563	0.666384	7.06E-10	0.29217	0.401614	0.654135
0.063962	-0.0636	0.313027	0.021188	0.123092	-0.25437	0.026154	0.067022	0.069001
0.474964	0.477491	0.000339	0.813091	0.167985	0.003903	0.770384	0.454056	0.440805
0.222221	-0.00379	-0.06107	-0.13173	-0.39753	-0.35404	0.047101	-0.44662	-0.16438
0.012038	0.966299	0.495213	0.139865	3.7E-06	4.44E-05	0.598994	1.42E-07	0.064792
-0.03174	-0.32427	-0.4036	-0.34527	-0.17527	-0.02451	0.388538	0.130078	0.09214
0.723144	0.0002	2.54E-06	7.02E-05	0.048731	0.784444	6.36E-06	0.144947	0.302872
-0.01931	-0.03339	0.018765	-0.05358	-0.2406	-0.21901	-0.03897	-0.02568	-0.17547
0.829392	0.709372	0.834135	0.549639	0.006435	0.013367	0.663539	0.774468	0.048471
0.111302	-0.02766	0.266642	-0.03497	-0.36519	-0.53591	0.017335	-0.12113	-0.19812
0.212843	0.757577	0.002444	0.69634	2.43E-05	8.43E-11	0.846614	0.174921	0.025558
0.483481	0.639471	0.552373	0.532703	-0.66157	-0.37269	-0.50926	-0.52534	-0.64871
8.52E-09	5.88E-16	1.67E-11	1.14E-10	2.52E-17	1.6E-05	9.72E-10	2.28E-10	1.63E-16
0.042239	0.080411	-0.31513	0.074007	-0.28566	0.195185	-0.07494	-0.15864	-0.23141
0.637271	0.368819	0.000308	0.408291	0.001132	0.027873	0.402417	0.074851	0.008852
0.027347	0.130352	-0.21147	0.172497	0.135676	0.467514	-0.18653	-0.07174	-0.09536
0.760221	0.144095	0.017001	0.052467	0.128282	3E-08	0.035752	0.422828	0.286238
0.215122	0.479957	0.162323	0.421417	-0.44265	0.05434	-0.54787	-0.41	-0.58485
0.015147	1.13E-08	0.068256	8.06E-07	1.88E-07	0.544001	2.62E-11	1.69E-06	5.2E-13
-0.37527	-0.19795	-0.40445	-0.55455	0.16788	0.283644	0.369166	0.231095	0.399816
1.38E-05	0.025691	2.41E-06	1.34E-11	0.059212	0.001231	1.95E-05	0.008948	3.21E-06

MTHFD1	MXD3	MYB	MYBL1	MYBL2	MYC	MYLIP	NAA16	NAA40
blue	blue	blue	blue	blue	blue	blue	blue	blue
-0.27885	-0.48845	-0.25074	-0.32473	-0.46107	-0.19433	0.650808	-0.23968	-0.3285
0.0015	5.69E-09	0.004464	0.000195	4.9E-08	0.028581	1.21E-16	0.006648	0.000163
-0.24679	-0.50877	-0.34682	-0.33337	-0.43694	-0.25181	0.557521	-0.26051	-0.36143
0.005156	1.01E-09	6.48E-05	0.000128	2.81E-07	0.004292	9.87E-12	0.003096	2.98E-05
0.636139	0.619066	0.605068	0.625653	0.876975	0.482473	-0.5632	0.57241	0.571375
9.26E-16	8.66E-15	4.91E-14	3.72E-15	1.33E-41	9.25E-09	5.48E-12	2.05E-12	2.3E-12
0.390695	0.148529	0.356607	0.47791	0.478136	0.382017	-0.33032	0.503637	0.315733
5.59E-06	0.095604	3.87E-05	1.33E-08	1.31E-08	9.34E-06	0.000149	1.58E-09	0.000299
-0.36079	0.038588	-0.1166	-0.18335	-0.11484	-0.16138	0.131951	-0.27454	-0.22266
3.09E-05	0.666667	0.191725	0.039075	0.198558	0.069895	0.139197	0.001787	0.011865
-0.06863	-0.09565	0.093504	0.177289	0.102708	0.121379	0.007498	0.086387	-0.06265
0.443296	0.284748	0.295739	0.04615	0.250533	0.174026	0.933324	0.334187	0.484061
-0.14013	-0.26245	-0.13779	-0.06841	-0.0807	0.047429	0.036763	0.108177	-0.04963
0.116097	0.002875	0.122376	0.444731	0.367069	0.596448	0.681553	0.22605	0.57949
-0.37804	-0.25128	-0.00537	0.007672	-0.28829	-0.28571	0.17795	-0.27486	0.008047
1.18E-05	0.004376	0.952238	0.931781	0.001013	0.001129	0.04533	0.001764	0.928453
0.317919	0.200088	0.286276	0.187507	0.404673	0.579702	-0.10551	0.204839	0.101468
0.00027	0.024105	0.001103	0.034775	2.37E-06	9.25E-13	0.237791	0.02088	0.256327
-0.33022	-0.14231	-0.07849	-0.1583	-0.23443	0.208317	0.45885	-0.21232	-0.15214
0.00015	0.110485	0.380409	0.075474	0.007982	0.018761	5.78E-08	0.016556	0.08773
-0.0802	-0.18037	0.030177	-0.08127	-0.1317	0.14871	0.359686	-0.14088	-0.10101
0.370099	0.042429	0.73627	0.363727	0.139951	0.095196	3.28E-05	0.114136	0.258497
0.432427	0.053033	-0.06181	-0.30029	0.067142	0.2257	-0.16772	0.092251	0.090534
3.84E-07	0.553744	0.489952	0.000603	0.453245	0.01073	0.059453	0.302288	0.311414
0.54692	0.539345	0.367231	0.293722	0.532749	0.364207	-0.39722	0.437079	0.567115
2.88E-11	6.06E-11	2.17E-05	0.000803	1.14E-10	2.56E-05	3.77E-06	2.78E-07	3.62E-12
0.119528	0.664147	0.078993	0.017145	0.220762	0.048935	-0.19432	-0.06452	0.174908
0.180736	1.72E-17	0.377353	0.848279	0.012627	0.584827	0.028588	0.471107	0.049204
0.048167	0.363296	0.086824	0.002829	0.096101	0.235055	0.089389	0.002104	0.213522
0.590745	2.69E-05	0.331738	0.974821	0.282471	0.007811	0.317598	0.981271	0.015938
-0.40172	-0.30535	-0.46014	-0.27812	-0.59367	-0.56398	0.448802	-0.2741	-0.22102
2.85E-06	0.000482	5.25E-08	0.001546	1.9E-13	5.04E-12	1.21E-07	0.001818	0.012521
0.275594	0.253757	0.010832	0.041072	0.191544	-0.15126	-0.33734	0.170805	0.235591
0.001712	0.003993	0.903799	0.646612	0.030985	0.089585	0.000105	0.054861	0.007668
-0.03013	-0.35141	-0.15604	0.020947	-0.17767	-0.33653	-0.07966	-0.00172	-0.24094
0.736629	5.1E-05	0.079794	0.815178	0.045682	0.00011	0.373327	0.9847	0.006358
-0.20096	-0.3861	-0.33681	-0.32869	-0.39639	-0.473	0.045221	-0.28748	-0.32418
0.023484	7.35E-06	0.000108	0.000161	3.96E-06	1.96E-08	0.613671	0.001048	0.000201
0.291428	0.382185	0.40432	0.277232	0.472236	0.301214	-0.27676	0.543085	0.467823
0.000887	9.25E-06	2.43E-06	0.001603	2.08E-08	0.000579	0.001633	4.21E-11	2.93E-08

NARS2	NASP	NCAPD2	NCAPD3	NCAPG	NCAPG2	NCAPH	NCBP1	NCOA5
blue	blue	blue	blue	blue	blue	blue	blue	blue
-0.24319	-0.30313	-0.15236	-0.52663	-0.43762	-0.44536	-0.46034	-0.19186	-0.28292
0.005869	0.000532	0.087252	2.02E-10	2.68E-07	1.55E-07	5.17E-08	0.030706	0.001269
-0.19145	-0.34683	-0.15779	-0.55437	-0.42428	-0.43826	-0.42928	-0.14639	-0.25703
0.031068	6.48E-05	0.076432	1.36E-11	6.66E-07	2.56E-07	4.75E-07	0.100533	0.003533
0.617956	0.715874	0.517581	0.83412	0.901952	0.903603	0.940075	0.60217	0.573415
9.97E-15	3.12E-21	4.63E-10	4.28E-34	2.05E-47	7.47E-48	2.94E-60	6.96E-14	1.84E-12
0.522179	0.432505	0.165385	0.594809	0.615837	0.711445	0.589762	0.671126	0.311759
3.05E-10	3.81E-07	0.063143	1.66E-13	1.3E-14	7.03E-21	2.98E-13	5.95E-18	0.00036
0.265694	-0.28085	-0.30445	-0.44278	-0.07827	-0.22975	-0.10646	0.014597	-0.29117
0.002536	0.001382	0.000501	1.86E-07	0.38175	0.009365	0.233547	0.87061	0.000897
0.47068	-0.02117	-0.18688	0.014441	0.27249	0.27411	0.200344	0.526027	-0.12686
2.35E-08	0.813231	0.035402	0.871981	0.00194	0.001818	0.023921	2.14E-10	0.155259
0.218196	-0.08039	-0.23103	-0.12797	0.000905	0.034125	-0.01724	0.231566	-0.03809
0.013724	0.368941	0.008967	0.151618	0.991943	0.703294	0.847407	0.008805	0.670724
-0.17514	-0.15248	-0.2041	-0.11649	-0.15189	-0.1461	-0.3673	0.265741	-0.19684
0.0489	0.087015	0.021357	0.192134	0.088258	0.101217	2.16E-05	0.002531	0.026552
-0.00188	0.346386	0.163176	0.379345	0.379067	0.425359	0.340473	0.037598	0.165063
0.983271	6.63E-05	0.0668	1.09E-05	1.11E-05	6.2E-07	8.98E-05	0.674727	0.063666
-0.43605	-0.05001	-0.124	-0.30925	-0.24307	-0.2775	-0.35391	-0.28994	-0.32448
2.99E-07	0.576578	0.164856	0.000403	0.005894	0.001585	4.47E-05	0.000945	0.000198
-0.18502	-0.0063	-0.07648	-0.12669	-0.06617	-0.03685	-0.19483	0.002222	-0.22661
0.037295	0.943961	0.392735	0.155809	0.459811	0.680855	0.028167	0.980222	0.01041
0.031059	0.16486	0.230621	0.16108	-0.03287	0.042598	0.078422	-0.249	0.435027
0.728864	0.063997	0.009093	0.070425	0.713747	0.634415	0.38082	0.004758	3.21E-07
-0.07766	0.680338	0.57059	0.65192	0.386278	0.422355	0.458973	-0.09304	0.601144
0.385483	1.4E-18	2.5E-12	1.03E-16	7.28E-06	7.57E-07	5.73E-08	0.298139	7.86E-14
-0.23803	0.224117	0.121171	0.080998	0.032374	-0.06304	0.058064	-0.52049	0.097996
0.007044	0.011309	0.174773	0.365326	0.717856	0.481388	0.516709	3.56E-10	0.273045
-0.55926	0.354519	0.250767	0.08184	-0.07174	-0.10095	-0.09138	-0.52661	0.071574
8.25E-12	4.33E-05	0.00446	0.360346	0.422819	0.258797	0.306916	2.03E-10	0.423914
-0.56	-0.25198	-0.10804	-0.57429	-0.65781	-0.67013	-0.58362	-0.4278	-0.26681
7.64E-12	0.004264	0.226644	1.68E-12	4.39E-17	6.93E-18	5.97E-13	5.26E-07	0.002428
0.086348	0.15855	0.245716	0.226315	0.075735	0.084631	0.242017	-0.05288	0.310443
0.334406	0.075014	0.005361	0.010512	0.397402	0.344144	0.00612	0.554885	0.000382
0.362155	-0.39776	-0.25065	-0.0736	-0.05232	0.012069	-0.00937	0.345982	-0.10049
2.87E-05	3.64E-06	0.004479	0.410854	0.559105	0.892876	0.916728	6.77E-05	0.260947
-0.02406	-0.54664	-0.22939	-0.35353	-0.39516	-0.39976	-0.32796	-0.12618	-0.15844
0.788324	2.96E-11	0.009481	4.56E-05	4.27E-06	3.22E-06	0.000167	0.157486	0.075223
0.446048	0.479639	0.356833	0.359916	0.406488	0.323876	0.563921	0.164405	0.460019
1.48E-07	1.16E-08	3.82E-05	3.24E-05	2.12E-06	0.000204	5.08E-12	0.064744	5.3E-08

NCOA7	NDC1	NDC80	NEBL	NEIL3	NEK2	NEMP1	NETO2	NEURL1B
blue	blue	blue	blue	blue	blue	blue	blue	blue
0.448186	-0.31797	-0.42651	0.282146	-0.45137	-0.43915	-0.2829	-0.21237	-0.32867
1.26E-07	0.00027	5.73E-07	0.00131	1E-07	2.41E-07	0.00127	0.016531	0.000162
0.483624	-0.28868	-0.4402	0.314646	-0.43994	-0.41477	-0.29298	-0.14474	-0.3454
8.43E-09	0.000996	2.23E-07	0.000315	2.28E-07	1.25E-06	0.00083	0.104461	6.98E-05
-0.7479	0.848925	0.896694	-0.77333	0.89557	0.85334	0.822061	0.610838	0.442319
5.39E-24	2.02E-36	4.54E-46	1.67E-26	8.61E-46	3.65E-37	2.31E-32	2.43E-14	1.92E-07
-0.52064	0.848943	0.664979	-0.45421	0.613553	0.578413	0.854296	0.525505	0.173021
3.51E-10	2E-36	1.52E-17	8.15E-08	1.73E-14	1.07E-12	2.5E-37	2.25E-10	0.051743
-0.0129	0.089262	0.061096	0.053075	0.064553	0.017404	-0.02615	-0.03169	-0.42248
0.885513	0.318292	0.495022	0.553428	0.470885	0.846016	0.770418	0.723591	7.51E-07
-0.20588	0.619768	0.416011	-0.11399	0.362943	0.317256	0.574952	0.316688	-0.22533
0.020228	7.92E-15	1.15E-06	0.201946	2.75E-05	0.000279	1.56E-12	0.000286	0.010862
-0.15532	0.334571	0.088206	-0.0487	0.020938	0.021041	0.355606	0.137475	-0.28213
0.081211	0.000121	0.32407	0.586652	0.815253	0.814362	4.08E-05	0.123249	0.001311
0.022357	-0.02355	-0.14604	0.404185	-0.16696	-0.14129	-0.01004	-0.34996	0.075096
0.802979	0.792692	0.101359	2.45E-06	0.060636	0.113094	0.910838	5.5E-05	0.401411
-0.35153	0.279703	0.430512	-0.44124	0.276498	0.229796	0.400202	0.108741	0.098118
5.07E-05	0.001449	4.37E-07	2.08E-07	0.001651	0.009351	3.13E-06	0.223627	0.272444
0.101231	-0.29539	-0.10679	0.212071	-0.26404	-0.40388	-0.08275	-0.39324	-0.29729
0.257447	0.000747	0.232089	0.016685	0.002704	2.49E-06	0.355029	4.8E-06	0.000688
-0.00713	-0.05278	-0.00428	0.135711	-0.10436	-0.1802	0.107214	-0.18573	-0.12385
0.936587	0.555614	0.961887	0.128181	0.24294	0.042629	0.230232	0.036559	0.165357
-0.14435	-0.14826	-0.11809	-0.18114	-0.10854	-0.11412	-0.12749	-0.06078	0.075544
0.105414	0.096203	0.186072	0.04154	0.224468	0.201409	0.153181	0.497282	0.398596
-0.50068	0.045678	0.272568	-0.53892	0.263914	0.223116	0.160035	0.068574	0.445759
2.04E-09	0.610094	0.001934	6.31E-11	0.002717	0.011689	0.072292	0.443647	1.51E-07
-0.28259	-0.33017	0.006701	-0.27234	0.059062	-0.03537	-0.31433	-0.18962	0.025058
0.001286	0.00015	0.9404	0.001951	0.50952	0.693	0.00032	0.032749	0.779754
-0.18921	-0.46003	-0.07259	-0.13448	-0.14964	-0.28747	-0.24552	-0.40472	0.065653
0.033131	5.29E-08	0.417374	0.131716	0.093115	0.001049	0.005399	2.37E-06	0.463344
0.58266	-0.68578	-0.67243	0.553726	-0.61971	-0.64225	-0.72898	-0.47243	-0.1247
6.65E-13	5.82E-19	4.86E-18	1.46E-11	7.98E-15	4.02E-16	2.58E-22	2.05E-08	0.162447
-0.06148	-0.06206	-0.05721	-0.14087	0.082411	0.158	-0.21188	0.124485	0.393088
0.492301	0.488205	0.522895	0.114167	0.35699	0.076043	0.016788	0.163188	4.84E-06
0.370732	0.229125	-0.11145	0.240878	0.018558	0.173969	0.008507	0.339034	0.03985
1.78E-05	0.009566	0.212217	0.006372	0.835943	0.050455	0.924376	9.66E-05	0.656445
0.582397	-0.33743	-0.50548	0.44759	-0.38225	-0.19803	-0.50522	-0.05822	-0.01658
6.85E-13	0.000105	1.35E-09	1.32E-07	9.22E-06	0.02563	1.38E-09	0.515602	0.85323
-0.34943	0.393295	0.399602	-0.66405	0.414972	0.408187	0.357902	0.274366	0.241445
5.66E-05	4.78E-06	3.25E-06	1.74E-17	1.23E-06	1.9E-06	3.61E-05	0.001799	0.006245

NFRKB	NFS1	NIFK	NIN	NKIRAS2	NKX3-2	NOL11	NONO	NOP2
blue	blue	blue	blue	blue	blue	blue	blue	blue
-0.39718	0.240211	-0.29551	-0.24428	-0.31329	-0.14607	-0.20363	-0.29722	-0.0958
3.78E-06	0.006524	0.000744	0.005645	0.000335	0.101291	0.021666	0.00069	0.284006
-0.45332	0.367141	-0.25028	-0.29895	-0.32072	-0.16996	-0.16891	-0.29067	-0.07094
8.7E-08	2.18E-05	0.00454	0.00064	0.000237	0.056092	0.057642	0.000916	0.428067
0.614707	-0.52483	0.662842	0.592666	0.714629	0.539653	0.727035	0.806215	0.486903
1.5E-14	2.39E-10	2.09E-17	2.13E-13	3.93E-21	5.88E-11	3.77E-22	2.82E-30	6.46E-09
0.343222	-0.25332	0.771757	0.544092	0.27445	0.320342	0.800259	0.445027	0.265256
7.8E-05	0.004058	2.44E-26	3.81E-11	0.001793	0.000241	1.53E-29	1.59E-07	0.002579
-0.38481	0.090017	0.371791	-0.48722	-0.13453	-0.22068	0.283054	-0.20107	-0.13701
7.93E-06	0.314195	1.68E-05	6.29E-09	0.131559	0.012662	0.001262	0.023409	0.124529
-0.17142	0.004499	0.783058	0.061557	-0.0905	-0.02542	0.742158	0.00545	-0.02028
0.053976	0.959961	1.5E-27	0.491761	0.311607	0.776659	1.81E-23	0.951509	0.821003
-0.10673	0.142112	0.458578	-0.15318	-0.0662	-0.07951	0.428776	-0.02965	0.094349
0.232375	0.110984	5.9E-08	0.085546	0.45963	0.37422	4.92E-07	0.740673	0.291378
-0.12657	0.090852	0.08826	-0.11629	-0.42049	-0.18045	0.033673	-0.1893	-0.22996
0.156185	0.30971	0.323774	0.192908	8.57E-07	0.042346	0.707043	0.033042	0.0093
0.225054	-0.36498	0.250225	0.143823	0.555654	0.33833	0.309818	0.375149	0.232886
0.010963	2.45E-05	0.00455	0.106707	1.2E-11	0.0001	0.000393	1.39E-05	0.008417
-0.35535	-0.01096	-0.05086	-0.26974	-0.04712	-0.07567	-0.08202	-0.22606	-0.00095
4.14E-05	0.902708	0.570123	0.002164	0.59881	0.397801	0.359299	0.010603	0.991552
-0.20319	0.017348	0.124729	-0.02468	-0.13508	-0.0462	0.037851	-0.13193	0.062196
0.021957	0.846498	0.162358	0.782984	0.129974	0.605992	0.672669	0.139271	0.487274
0.295135	0.084404	-0.17487	-0.10657	0.342602	0.222663	-0.25219	0.227421	0.326091
0.000756	0.345444	0.049249	0.233067	8.05E-05	0.011865	0.004232	0.01013	0.000183
0.672127	-0.40677	-0.18273	0.386093	0.68895	0.514519	-0.10546	0.652761	0.497422
5.09E-18	2.08E-06	0.039758	7.36E-06	3.46E-19	6.1E-10	0.238008	9.14E-17	2.69E-09
0.140193	-0.28227	-0.28412	-0.21033	0.403544	0.249036	-0.38253	0.201624	0.091027
0.11594	0.001303	0.001207	0.017621	2.55E-06	0.004752	9.07E-06	0.023019	0.308777
0.098846	-0.2442	-0.41072	-0.09936	0.403277	0.282048	-0.41963	0.184955	0.217524
0.268887	0.005661	1.62E-06	0.266385	2.59E-06	0.001316	9.07E-07	0.037368	0.014025
-0.51055	0.354149	-0.65394	-0.34614	-0.41824	-0.22439	-0.61866	-0.42927	-0.2485
8.67E-10	4.41E-05	7.71E-17	6.71E-05	9.93E-07	0.011208	9.12E-15	4.76E-07	0.004847
0.313292	-0.14905	-0.28576	0.176055	0.181467	0.134052	-0.27221	0.249993	0.096581
0.000335	0.094444	0.001127	0.047713	0.041172	0.132956	0.001962	0.004589	0.280063
-0.09015	0.253918	0.073478	0.200341	-0.4873	-0.30413	0.087262	-0.27175	-0.34646
0.313469	0.003969	0.41166	0.023924	6.25E-09	0.000509	0.329294	0.001998	6.6E-05
-0.16592	0.341929	-0.44538	-0.18225	-0.49143	-0.43257	-0.42984	-0.41127	-0.34642
0.062282	8.34E-05	1.55E-07	0.040296	4.45E-09	3.8E-07	4.58E-07	1.56E-06	6.62E-05
0.514455	-0.36282	0.16318	0.324775	0.561773	0.343327	0.345387	0.553954	0.372832
6.13E-10	2.76E-05	0.066792	0.000195	6.36E-12	7.76E-05	6.98E-05	1.42E-11	1.58E-05

NOP56	NR1D1	NR2C2AP	NREP	NRF1	NRM	NT5DC2	NUDT16	NUF2
blue	blue	blue	blue	blue	blue	blue	blue	blue
-0.16873	0.282375	-0.17587	-0.19689	-0.31511	-0.17772	-0.45375	0.143777	-0.41851
0.057918	0.001298	0.047947	0.02651	0.000308	0.04562	8.44E-08	0.106821	9.76E-07
-0.14338	0.284115	-0.09055	-0.16776	-0.26058	-0.16866	-0.39862	0.136141	-0.3683
0.107806	0.001207	0.311324	0.059398	0.003088	0.058019	3.46E-06	0.126966	2.04E-05
0.577374	-0.65678	0.542195	0.578648	0.620722	0.77196	0.810616	-0.51938	0.902931
1.2E-12	5.11E-17	4.59E-11	1.04E-12	7.02E-15	2.33E-26	7.77E-31	3.94E-10	1.13E-47
0.267226	-0.57486	0.30795	0.350419	0.591213	0.522818	0.390231	-0.27318	0.630685
0.002388	1.57E-12	0.000428	5.37E-05	2.52E-13	2.88E-10	5.75E-06	0.001887	1.92E-15
-0.04043	-0.11588	0.356223	-0.13628	0.032674	0.080516	0.04075	-0.32034	0.11284
0.651741	0.194495	3.95E-05	0.126571	0.715352	0.368193	0.649194	0.000241	0.20655
-0.02805	-0.40984	0.277619	0.056594	0.343023	0.300577	0.087681	-0.25055	0.404828
0.754244	1.71E-06	0.001578	0.527399	7.88E-05	0.000596	0.326972	0.004495	2.35E-06
0.062165	-0.20222	0.198651	-0.01229	0.368569	0.15051	0.032992	-0.02329	0.05198
0.487491	0.02261	0.025162	0.89096	2.01E-05	0.091215	0.712701	0.794911	0.561658
-0.25671	0.081547	-0.15298	-0.3192	-0.09473	-0.24127	-0.29898	0.257227	-0.17724
0.003575	0.362075	0.085971	0.000254	0.289434	0.006284	0.000639	0.003507	0.046211
0.171217	-0.25526	0.199257	0.169101	0.224971	0.51589	0.448091	0.077428	0.268699
0.054269	0.003775	0.024711	0.057363	0.010993	5.39E-10	1.27E-07	0.386907	0.002254
-0.2343	0.010008	-0.10445	-0.53686	-0.18403	0.109362	-0.26276	0.405649	-0.27856
0.008017	0.911083	0.242561	7.7E-11	0.038347	0.220975	0.00284	2.23E-06	0.001518
-0.19606	-0.0154	-0.07941	-0.24358	-0.04565	0.098641	-0.31048	0.330279	-0.14115
0.027166	0.863518	0.374854	0.005787	0.610322	0.269886	0.000381	0.000149	0.113457
0.294891	0.170327	0.227681	0.238495	0.077252	0.104085	0.215413	0.102082	-0.15753
0.000764	0.055554	0.010042	0.00693	0.387992	0.244201	0.015007	0.253445	0.076936
0.506535	-0.11925	0.201969	0.248122	0.272545	0.423517	0.536189	-0.02589	0.23966
1.23E-09	0.181763	0.022781	0.004913	0.001935	7.01E-07	8.21E-11	0.772649	0.006652
0.141014	0.08953	0.295566	-0.11653	-0.04071	0.187973	0.304132	-0.06823	0.012717
0.113798	0.316835	0.000742	0.192006	0.649557	0.034319	0.000509	0.445916	0.887158
0.063004	0.100834	0.005589	-0.32413	-0.09209	0.23467	0.116877	0.367229	-0.18521
0.481619	0.259328	0.950272	0.000201	0.30315	0.007916	0.19067	2.17E-05	0.037099
-0.26896	0.285764	-0.39201	-0.44798	-0.42543	-0.53799	-0.55315	0.37871	-0.58044
0.002231	0.001127	5.17E-06	1.28E-07	6.17E-07	6.91E-11	1.54E-11	1.13E-05	8.52E-13
0.251034	0.018652	0.008966	0.348987	-0.02444	-0.13885	0.211559	-0.23984	0.072461
0.004417	0.835122	0.920311	5.79E-05	0.785046	0.119514	0.016956	0.006611	0.418178
-0.18712	0.045583	-0.26535	0.313873	-0.1133	-0.46866	-0.26721	-0.23563	0.010565
0.035162	0.610834	0.002569	0.000326	0.204682	2.75E-08	0.00239	0.007657	0.906154
-0.20434	0.419849	-0.37843	0.097751	-0.43425	-0.69828	-0.38512	-0.03942	-0.38493
0.021202	8.94E-07	1.15E-05	0.274248	3.38E-07	7.18E-20	7.79E-06	0.659934	7.88E-06
0.588478	-0.3349	0.29457	0.385344	0.38129	0.431501	0.653958	-0.60285	0.46236
3.45E-13	0.000119	0.000775	7.69E-06	9.75E-06	4.09E-07	7.69E-17	6.42E-14	4.44E-08

NUP35	NUP50	NUP85	NUP93	NUPL2	NUSAP1	NVL	NWD1	OAZ2
blue	blue	blue	blue	blue	blue	blue	blue	blue
-0.17382	-0.22414	-0.18882	-0.2474	-0.20286	-0.44534	-0.31582	0.253128	0.22801
0.050661	0.011301	0.033502	0.005044	0.022179	1.55E-07	0.000298	0.004088	0.009932
-0.09492	-0.3016	-0.14565	-0.24615	-0.14694	-0.44946	-0.27828	0.243964	0.271101
0.28843	0.000569	0.102293	0.005278	0.099249	1.15E-07	0.001536	0.005709	0.00205
0.669878	0.615892	0.680196	0.716042	0.647169	0.890106	0.74902	-0.61545	-0.61322
7.2E-18	1.29E-14	1.43E-18	3.03E-21	2.02E-16	1.75E-44	4.24E-24	1.37E-14	1.81E-14
0.69077	0.5975	0.58012	0.298664	0.68434	0.618716	0.607522	-0.34481	-0.49081
2.56E-19	1.21E-13	8.83E-13	0.000648	7.36E-19	9.06E-15	3.64E-14	7.19E-05	4.68E-09
0.389532	-0.23	0.351323	-0.01754	0.350754	-0.13628	0.208049	-0.10784	0.155754
6E-06	0.009287	5.12E-05	0.844848	5.28E-05	0.126586	0.018917	0.227493	0.080363
0.723934	0.264418	0.519142	0.001908	0.673347	0.255622	0.442099	-0.16413	-0.15429
6.85E-22	0.002664	4.02E-10	0.983017	4.22E-18	0.003725	1.95E-07	0.065204	0.083272
0.429629	0.062834	0.339783	0.017101	0.421303	-0.0411	0.190984	-0.10814	0.046871
4.64E-07	0.482805	9.3E-05	0.848659	8.12E-07	0.646374	0.031489	0.226204	0.600784
-0.06047	-0.11504	-0.03494	-0.34777	0.016649	-0.17827	0.075146	0.603048	0.162398
0.499484	0.197781	0.696568	6.17E-05	0.852617	0.044941	0.401096	6.26E-14	0.068127
0.202362	0.304376	0.316036	0.345324	0.071788	0.359934	0.196591	-0.34898	-0.32994
0.022512	0.000503	0.000295	7E-05	0.422525	3.23E-05	0.026744	5.79E-05	0.000152
-0.12219	-0.19827	-0.00265	-0.24105	-0.22139	-0.23918	-0.18067	0.185848	0.195052
0.171133	0.025449	0.976385	0.006334	0.012369	0.006766	0.042081	0.036443	0.027982
-0.01077	0.024632	0.009444	-0.19589	-0.02073	-0.05316	-0.04255	0.183114	0.122656
0.904364	0.783401	0.916074	0.027303	0.817091	0.552778	0.634775	0.039338	0.169509
-0.27185	-0.0207	-0.01834	0.316252	-0.24183	-0.00567	0.084109	-0.21675	0.08965
0.00199	0.817309	0.837815	0.000292	0.00616	0.94953	0.347136	0.01438	0.316183
-0.16085	0.215511	0.134127	0.534759	-0.20046	0.387292	0.2219	-0.28936	-0.39369
0.070839	0.01496	0.132737	9.41E-11	0.023838	6.85E-06	0.012166	0.000968	4.67E-06
-0.29466	-0.31564	0.031252	0.185843	-0.35528	0.007214	-0.01774	-0.15274	-0.09988
0.000772	0.000301	0.727239	0.036448	4.15E-05	0.935848	0.843055	0.086474	0.263884
-0.43869	-0.23285	-0.09238	0.085633	-0.55553	-0.06804	-0.1394	0.028081	-0.01674
2.48E-07	0.008426	0.3016	0.338438	1.21E-11	0.447195	0.118036	0.753987	0.851797
-0.57817	-0.49317	-0.56137	-0.50372	-0.47947	-0.63666	-0.48938	0.4307	0.542327
1.1E-12	3.85E-09	6.63E-12	1.57E-09	1.18E-08	8.63E-16	5.27E-09	4.32E-07	4.53E-11
-0.20061	0.021339	-0.20202	0.231962	-0.06093	0.093038	-0.0274	-0.17744	0.008677
0.023735	0.811783	0.022748	0.008687	0.496159	0.298166	0.759791	0.045957	0.922871
0.09995	0.170898	-0.23562	-0.2249	0.285038	-0.02523	-0.15549	0.082074	0.068071
0.26355	0.054728	0.007661	0.01102	0.001162	0.778286	0.08088	0.358967	0.447005
-0.3576	-0.21912	-0.53906	-0.26536	-0.2058	-0.38339	-0.46033	0.264939	0.351818
3.67E-05	0.01332	6.23E-11	0.002568	0.020274	8.62E-06	5.17E-08	0.002611	4.99E-05
0.33453	0.421416	0.390013	0.7189	0.320655	0.347784	0.449165	-0.68848	-0.48448
0.000121	8.06E-07	5.83E-06	1.78E-21	0.000237	6.17E-05	1.18E-07	3.73E-19	7.86E-09

ODC1	ODF2	OIP5	ONECUT1	OPTN	ORC1	ORC6	OSBPL2	OTOS
blue	blue	blue	blue	blue	blue	blue	blue	blue
-0.39934	-0.3428	-0.44625	-0.29505	0.527755	-0.39385	-0.33043	0.24365	0.135665
3.31E-06	7.97E-05	1.45E-07	0.000759	1.82E-10	4.63E-06	0.000148	0.005773	0.128312
-0.34656	-0.3935	-0.3979	-0.30453	0.532918	-0.3847	-0.24737	0.254528	0.169877
6.57E-05	4.72E-06	3.61E-06	0.0005	1.12E-10	7.99E-06	0.005048	0.00388	0.056212
0.841527	0.740188	0.8911	0.538881	-0.75582	0.930401	0.829028	-0.55005	-0.62013
3.14E-35	2.72E-23	1.03E-44	6.34E-11	9.64E-25	2.51E-56	2.39E-33	2.11E-11	7.57E-15
0.536873	0.56083	0.69512	0.366511	-0.70262	0.630449	0.523898	-0.2779	-0.55714
7.69E-11	7.01E-12	1.23E-19	2.26E-05	3.38E-20	1.98E-15	2.6E-10	0.001559	1.03E-11
0.140859	-0.31185	0.049954	-0.37458	0.110812	-0.08377	0.152814	-0.19049	0.245044
0.114202	0.000358	0.577022	1.43E-05	0.214877	0.349089	0.086311	0.03194	0.005492
0.31424	0.098441	0.428776	-0.09672	-0.35453	0.252234	0.333377	-0.17473	-0.21659
0.000321	0.270863	4.92E-07	0.279349	4.32E-05	0.004225	0.000128	0.049441	0.014455
0.129776	0.101587	0.088055	-0.04727	-0.21674	0.060222	0.047724	0.015694	-0.0147
0.145892	0.255769	0.324904	0.597663	0.014385	0.501224	0.594164	0.860978	0.869692
-0.08056	0.025097	-0.20352	-0.09241	-0.01202	-0.24869	-0.2103	0.238568	0.39869
0.367931	0.779415	0.021737	0.301459	0.893281	0.004812	0.017638	0.006912	3.44E-06
0.218125	0.534655	0.338777	0.119263	-0.51009	0.439981	0.05536	-0.21213	-0.32309
0.013756	9.51E-11	9.78E-05	0.181709	9.03E-10	2.27E-07	0.536459	0.016651	0.000211
-0.30217	-0.05142	-0.25087	-0.32311	0.133415	-0.19668	-0.48397	0.15248	0.242651
0.000555	0.565864	0.004443	0.000211	0.134828	0.026678	8.19E-09	0.087008	0.005983
-0.18876	0.024812	-0.07294	-0.13071	0.016699	-0.04977	-0.27099	0.197816	0.04616
0.033558	0.78186	0.415102	0.142991	0.852179	0.578453	0.002059	0.025793	0.60632
-0.03478	0.200428	0.084686	0.159159	-0.04978	0.137867	-0.15758	0.059173	-0.08965
0.697847	0.023861	0.343829	0.073887	0.578355	0.122174	0.076836	0.508717	0.316205
0.284968	0.612364	0.256982	0.548828	-0.34798	0.490055	0.157725	-0.16129	-0.28065
0.001165	2.01E-14	0.003539	2.38E-11	6.1E-05	4.98E-09	0.076562	0.07005	0.001393
0.038561	0.01163	-0.00501	0.078874	-0.03344	0.06477	-0.04652	-0.19495	0.266463
0.666886	0.896743	0.95544	0.378072	0.708993	0.469397	0.603501	0.028063	0.002461
-0.15665	0.214225	-0.16787	0.075959	0.021173	0.013927	-0.38401	0.075231	0.185772
0.07862	0.015586	0.059226	0.396002	0.813219	0.876502	8.32E-06	0.400564	0.036521
-0.51438	-0.54051	-0.64919	-0.29165	0.748128	-0.65706	-0.48403	0.355954	0.487345
6.17E-10	5.41E-11	1.52E-16	0.000878	5.13E-24	4.9E-17	8.16E-09	4.01E-05	6.23E-09
0.205658	-0.00193	0.016308	0.277557	0.10311	0.078561	0.250916	-0.16669	-0.05188
0.020363	0.982789	0.855604	0.001582	0.248675	0.379974	0.004436	0.06106	0.56241
-0.04914	-0.34833	-0.00828	-0.03997	0.125084	-0.17937	0.246491	0.004879	-0.127
0.583221	5.99E-05	0.92636	0.655497	0.161156	0.043615	0.005213	0.956581	0.154772
-0.30876	-0.53798	-0.44564	-0.1832	0.527961	-0.48979	-0.10999	0.181293	0.227521
0.000413	6.91E-11	1.52E-07	0.039239	1.79E-10	5.09E-09	0.218338	0.04137	0.010096
0.521696	0.41336	0.394731	0.346433	-0.21691	0.530936	0.532518	-0.56459	-0.53432
3.19E-10	1.36E-06	4.38E-06	6.61E-05	0.014303	1.35E-10	1.16E-10	4.73E-12	9.82E-11

P2RY2	PAICS	PAMR1	PARD6G	PARPBP	PAX3	PBK	PBRM1	PBXIP1
blue	blue	blue	blue	blue	blue	blue	blue	blue
0.260791	-0.32516	0.338222	-0.30497	-0.33774	-0.21568	-0.47795	-0.23936	0.137399
0.003063	0.000191	0.000101	0.00049	0.000103	0.014878	1.33E-08	0.006723	0.12346
0.257319	-0.26994	0.311727	-0.3458	-0.29505	-0.24003	-0.42838	-0.3486	0.115544
0.003495	0.002146	0.00036	6.83E-05	0.000759	0.006565	5.05E-07	5.91E-05	0.195812
-0.55494	0.754766	-0.69692	0.623415	0.828888	0.556942	0.880436	0.570378	-0.49322
1.29E-11	1.22E-24	9.06E-20	4.96E-15	2.51E-33	1.05E-11	2.5E-42	2.56E-12	3.83E-09
-0.47503	0.78145	-0.42026	0.409857	0.784773	0.477814	0.60718	0.495169	-0.36724
1.67E-08	2.25E-27	8.7E-07	1.71E-06	9.68E-28	1.34E-08	3.8E-14	3.26E-09	2.17E-05
-0.10775	-0.00493	-0.07858	0.137803	0.196855	-0.00514	-0.01413	-0.46394	-0.14331
0.227878	0.956096	0.37984	0.12235	0.026537	0.954259	0.874746	3.94E-08	0.107982
-0.34403	0.525902	-0.1932	0.282034	0.646825	0.291995	0.314386	0.008773	-0.27504
7.49E-05	2.16E-10	0.029537	0.001316	2.12E-16	0.000865	0.000319	0.922019	0.001751
-0.12795	0.333243	-0.04138	0.016472	0.266279	-0.02688	-0.003	-0.14679	-0.19201
0.151698	0.000129	0.644163	0.854167	0.002478	0.76416	0.973286	0.099607	0.030569
0.26756	-0.02628	0.446726	-0.08131	-0.12573	-0.12142	-0.18126	0.054042	0.429953
0.002357	0.769305	1.41E-07	0.363474	0.158998	0.173873	0.041411	0.546215	4.54E-07
-0.10935	0.271215	-0.11472	0.36262	0.379309	0.316249	0.258535	0.427498	-0.19702
0.221013	0.002041	0.199032	2.79E-05	1.09E-05	0.000292	0.003338	5.36E-07	0.026409
0.313637	-0.31789	0.436724	-0.0208	-0.17445	-0.12114	-0.30278	0.030852	0.287678
0.00033	0.000271	2.85E-07	0.816446	0.049811	0.174874	0.00054	0.730599	0.00104
0.164074	-0.08376	0.295901	-0.07585	-0.03071	0.110346	-0.1308	0.11835	0.251651
0.065292	0.349171	0.000731	0.396672	0.731825	0.216824	0.142702	0.1851	0.004317
0.157199	-0.05659	-0.10119	-0.13074	-0.13559	0.036775	-0.08724	0.071178	-0.08567
0.077561	0.527446	0.257642	0.142883	0.12852	0.681457	0.329417	0.426489	0.338216
-0.0377	0.088025	-0.2716	0.225168	0.003705	0.130599	0.283806	0.559225	-0.0231
0.673869	0.325067	0.002009	0.010922	0.96702	0.14333	0.001223	8.28E-12	0.79657
0.154498	-0.32939	-0.03133	0.131141	-0.21314	0.019145	0.01265	-0.02994	0.168315
0.082862	0.000156	0.726597	0.141661	0.016132	0.830832	0.887749	0.738282	0.058547
0.354589	-0.4117	0.232542	0.062733	-0.35991	-0.10532	-0.16266	0.264471	0.35329
4.31E-05	1.52E-06	0.008517	0.483514	3.24E-05	0.238639	0.067682	0.002659	4.62E-05
0.451846	-0.63789	0.466524	-0.44261	-0.7311	-0.50831	-0.54606	-0.37643	0.306894
9.7E-08	7.3E-16	3.24E-08	1.88E-07	1.7E-22	1.06E-09	3.14E-11	1.29E-05	0.000449
-0.0853	-0.03887	-0.2594	-0.07006	-0.17869	-0.06398	0.15361	-0.0115	-0.136
0.340304	0.664366	0.00323	0.433817	0.044426	0.474857	0.084665	0.897886	0.127377
-0.24953	0.203504	-0.14549	-0.19766	0.12912	0.01015	0.064113	-0.23844	-0.23048
0.004668	0.021747	0.102654	0.025912	0.147958	0.909824	0.473921	0.006944	0.009137
0.110455	-0.29053	0.124521	-0.46463	-0.42944	-0.29277	-0.3285	-0.48508	0.029831
0.216366	0.000921	0.163064	3.74E-08	4.7E-07	0.000837	0.000163	7.49E-09	0.739191
-0.52068	0.255382	-0.69838	0.510813	0.379267	0.086184	0.384798	0.346341	-0.65059
3.5E-10	0.003758	7.06E-20	8.47E-10	1.1E-05	0.335327	7.94E-06	6.65E-05	1.25E-16

PCBD1	PCNA	PDCL	PDE9A	PDIK1L	PGAP1	PGR	PHF13	PHF19
blue	blue	blue	blue	blue	blue	blue	blue	blue
0.31131	-0.24197	-0.2435	-0.29139	-0.21505	-0.47515	0.135389	-0.31153	-0.37216
0.000367	0.00613	0.005805	0.000888	0.015183	1.66E-08	0.129097	0.000364	1.64E-05
0.410613	-0.25746	-0.31764	-0.23642	-0.18852	-0.46649	0.191232	-0.33795	-0.31129
1.63E-06	0.003476	0.000274	0.00745	0.033788	3.24E-08	0.031265	0.000102	0.000368
-0.6002	0.729759	0.536132	0.534482	0.63402	0.704015	-0.53253	0.68258	0.655766
8.8E-14	2.21E-22	8.25E-11	9.66E-11	1.23E-15	2.65E-20	1.16E-10	9.78E-19	5.92E-17
-0.48955	0.606179	0.538945	0.254189	0.57556	0.500549	-0.33788	0.49816	0.361057
5.2E-09	4.29E-14	6.3E-11	0.00393	1.46E-12	2.06E-09	0.000102	2.53E-09	3.04E-05
0.543324	0.245486	0.189774	0.054803	0.103354	-0.33876	-0.07015	-0.22918	-0.21046
4.11E-11	0.005405	0.032601	0.54057	0.247547	9.79E-05	0.433248	0.009546	0.01755
0.065127	0.528907	0.495981	0.040462	0.426742	0.01395	-0.15746	0.118864	-0.04697
0.466941	1.64E-10	3.04E-09	0.651516	5.65E-07	0.876302	0.077058	0.183187	0.600051
0.141157	0.153438	0.195454	0.072303	0.051801	-0.00667	0.093987	0.037334	0.037142
0.113428	0.08502	0.027654	0.419198	0.563004	0.940638	0.293243	0.676882	0.67845
0.112593	0.045083	-0.01458	-0.19389	-0.08419	-0.27181	0.034331	-0.12693	-0.22555
0.207554	0.61476	0.870723	0.02895	0.346662	0.001993	0.701584	0.154998	0.010784
-0.39531	0.383516	0.074548	-0.05333	-0.15754	0.114661	-0.11719	0.318351	0.313827
4.23E-06	8.56E-06	0.404864	0.551516	0.076917	0.199273	0.189492	0.000265	0.000327
0.141495	0.074986	-0.09388	-0.32346	-0.4938	-0.61435	0.189675	-0.32316	-0.17491
0.112559	0.4021	0.293811	0.000208	3.65E-09	1.57E-14	0.032694	0.000211	0.049199
0.006031	0.142435	0.044431	-0.19036	-0.23808	-0.40887	0.047427	-0.15844	-0.13862
0.946348	0.110166	0.619892	0.032062	0.007031	1.82E-06	0.59647	0.075222	0.120125
-0.13531	-0.1344	-0.37976	0.108926	-0.3686	0.045065	-0.0437	0.077813	0.275726
0.129322	0.131932	1.07E-05	0.222834	2.01E-05	0.614903	0.625687	0.38454	0.001703
-0.57223	0.095759	-0.13812	0.373292	-0.04828	0.40902	-0.28019	0.444663	0.63854
2.1E-12	0.284195	0.121486	1.54E-05	0.589851	1.8E-06	0.00142	1.63E-07	6.68E-16
-0.00142	0.018991	-0.18342	0.20746	-0.25197	-0.07577	-0.2429	0.006839	0.266627
0.987399	0.832165	0.039008	0.019265	0.004267	0.397197	0.00593	0.939169	0.002445
-0.15356	-0.05056	-0.31778	0.019519	-0.55864	-0.29767	-0.02465	-0.04976	0.267164
0.084773	0.572405	0.000272	0.827577	8.8E-12	0.000677	0.783279	0.578516	0.002394
0.384371	-0.55982	-0.3525	-0.16635	-0.34298	-0.46788	0.413348	-0.59488	-0.29733
8.14E-06	7.79E-12	4.82E-05	0.061601	7.9E-05	2.92E-08	1.37E-06	1.65E-13	0.000687
-0.1617	-0.24918	-0.01369	0.352606	0.162146	0.448982	-0.06478	0.079517	0.246624
0.069338	0.004728	0.878556	4.79E-05	0.068561	1.19E-07	0.469355	0.374184	0.005188
0.043334	-0.1888	0.230706	-0.12715	0.492879	0.381753	0.111779	-0.02178	-0.33535
0.628568	0.03352	0.009067	0.1543	3.94E-09	9.49E-06	0.210877	0.807932	0.000116
0.30897	-0.56946	-0.23527	-0.19706	0.009916	0.054894	0.315185	-0.29049	-0.41927
0.000409	2.82E-12	0.007752	0.026376	0.911897	0.539894	0.000307	0.000923	9.28E-07
-0.34355	0.195714	0.223815	0.429986	0.376305	0.52185	-0.30481	0.48976	0.366114
7.67E-05	0.027444	0.011423	4.53E-07	1.3E-05	3.14E-10	0.000493	5.11E-09	2.31E-05

PHF6	PHLDA1	PHYHD1	PI4K2A	PIF1	PIK3IP1	PIM2	PKMYT1	PKN3
blue	blue	blue	blue	blue	blue	blue	blue	blue
-0.31048	-0.31164	0.134272	0.534906	-0.40522	0.319436	0.319568	-0.50455	-0.49693
0.000382	0.000362	0.132316	9.28E-11	2.29E-06	0.000252	0.00025	1.46E-09	2.81E-09
-0.28649	-0.3466	0.197572	0.484184	-0.3622	0.335974	0.291947	-0.47772	-0.46833
0.001093	6.56E-05	0.02598	8.05E-09	2.86E-05	0.000113	0.000867	1.35E-08	2.82E-08
0.726401	0.668313	-0.56505	-0.69364	0.833968	-0.68729	-0.53371	0.67675	0.700572
4.26E-22	9.15E-18	4.51E-12	1.58E-19	4.51E-34	4.54E-19	1.04E-10	2.48E-18	4.83E-20
0.679868	0.511062	-0.53734	-0.60432	0.46047	-0.57683	-0.27541	0.131129	0.301292
1.51E-18	8.29E-10	7.35E-11	5.38E-14	5.12E-08	1.27E-12	0.001725	0.1417	0.000577
-0.13974	-0.19354	0.011508	-0.03506	0.015619	0.168856	0.162136	-0.22557	-0.12375
0.117134	0.029247	0.897827	0.695579	0.861637	0.057729	0.068578	0.010775	0.165706
0.369368	0.168779	-0.36618	-0.3472	0.16417	-0.22046	0.033003	-0.28315	-0.05277
1.92E-05	0.057845	2.3E-05	6.36E-05	0.065133	0.012753	0.712617	0.001257	0.555724
0.049801	0.042477	-0.11811	-0.09427	0.034605	-0.11147	0.131723	-0.37714	-0.22057
0.578197	0.635374	0.185992	0.29179	0.699323	0.212162	0.139887	1.24E-05	0.012706
-0.05105	-0.35274	0.267677	-0.04685	-0.3666	-0.01425	0.211893	-0.41723	-0.35694
0.568659	4.75E-05	0.002347	0.600927	2.24E-05	0.873644	0.016779	1.06E-06	3.8E-05
-0.05575	0.637172	-0.43698	-0.1457	0.353379	-0.31565	-0.15971	0.244977	0.312201
0.533554	8.05E-16	2.8E-07	0.10217	4.6E-05	0.0003	0.072875	0.005505	0.000353
-0.48053	0.035579	0.080956	0.444302	-0.23879	0.405435	0.145884	-0.29926	-0.26315
1.08E-08	0.691283	0.365574	1.67E-07	0.006857	2.26E-06	0.101727	0.000631	0.002798
-0.16818	-0.0575	-0.00554	0.237465	-0.17894	0.111776	0.207592	-0.28513	-0.21897
0.05875	0.520765	0.950737	0.007184	0.04412	0.210889	0.019187	0.001157	0.013387
-0.25531	0.174817	-0.05193	0.130368	0.032666	-0.02982	0.024344	0.275376	0.225926
0.003768	0.049324	0.562063	0.144047	0.715421	0.739267	0.785871	0.001728	0.01065
0.08544	0.418196	-0.0586	-0.16736	0.453898	-0.30075	-0.44827	0.712841	0.579413
0.339532	9.96E-07	0.512864	0.06002	8.34E-08	0.000591	1.26E-07	5.45E-21	9.55E-13
-0.32527	0.008288	0.239583	-0.01911	0.229736	0.068313	-0.25166	0.586066	0.444222
0.00019	0.926316	0.00667	0.831127	0.00937	0.445389	0.004316	4.54E-13	1.68E-07
-0.48559	0.147294	0.210748	0.294859	0.051596	0.227631	-0.20864	0.352947	0.227583
7.19E-09	0.098425	0.017392	0.000765	0.564554	0.010059	0.018575	4.7E-05	0.010076
-0.379	-0.57261	0.50532	0.553876	-0.54349	0.63748	0.051078	-0.29363	-0.4184
1.11E-05	2.01E-12	1.37E-09	1.43E-11	4.04E-11	7.72E-16	0.56847	0.000807	9.83E-07
0.230895	-0.04014	0.085979	-0.12359	0.148646	-0.10462	-0.2514	0.439711	0.236528
0.009009	0.654139	0.336483	0.166242	0.095341	0.241753	0.004358	2.31E-07	0.007422
0.484068	-0.24685	-0.10118	-0.20265	-0.16746	-0.13182	0.181152	-0.24992	-0.23265
8.13E-09	0.005146	0.257672	0.022316	0.059852	0.139602	0.041531	0.004601	0.008486
-0.05449	-0.48395	0.282798	0.203566	-0.38939	0.192333	0.316099	-0.25048	-0.35625
0.54289	8.21E-09	0.001275	0.021706	6.05E-06	0.030287	0.000294	0.004507	3.94E-05
0.351085	0.495251	-0.42611	-0.21061	0.532988	-0.28447	-0.36612	0.433574	0.417549
5.19E-05	3.23E-09	5.89E-07	0.017467	1.11E-10	0.00119	2.3E-05	3.54E-07	1.04E-06

PKNOX1	PKP2	PLA1A	PLA2G4C	PLCXD3	PLK1	PLK4	PNN	POC1A
blue	blue	blue	blue	blue	blue	blue	blue	blue
-0.12131	0.2136	0.389647	0.472357	0.279971	-0.48962	-0.44393	-0.28046	-0.42723
0.174288	0.015898	5.96E-06	2.06E-08	0.001433	5.17E-09	1.72E-07	0.001404	5.46E-07
-0.0486	0.196052	0.293968	0.485918	0.306493	-0.46927	-0.44338	-0.3234	-0.37454
0.587413	0.027172	0.000795	7E-09	0.000457	2.62E-08	1.78E-07	0.000208	1.44E-05
0.556942	-0.52967	-0.53971	-0.74311	-0.66747	0.833095	0.939219	0.613266	0.876948
1.05E-11	1.52E-10	5.85E-11	1.48E-23	1.04E-17	6.08E-34	6.94E-60	1.8E-14	1.35E-41
0.500075	-0.24351	-0.31631	-0.56557	-0.49013	0.377244	0.726789	0.521143	0.546199
2.15E-09	0.005803	0.000291	4.27E-12	4.95E-09	1.23E-05	3.95E-22	3.35E-10	3.1E-11
0.271998	-0.00884	0.209893	0.045407	0.004755	-0.25203	-0.03572	-0.36147	0.212935
0.001978	0.921433	0.017863	0.612214	0.957688	0.004257	0.69009	2.97E-05	0.016236
0.470659	-0.01	0.045047	-0.29426	-0.22823	-0.08471	0.400499	0.043131	0.361848
2.35E-08	0.911187	0.615043	0.000785	0.00986	0.343671	3.08E-06	0.630174	2.91E-05
0.37603	0.086124	0.122067	-0.04851	-0.12008	-0.15281	0.092167	0.009641	0.130573
1.32E-05	0.335668	0.17158	0.58809	0.178732	0.086309	0.302732	0.914334	0.143411
-0.10356	0.200795	0.044725	0.175948	0.25933	-0.42747	-0.19013	-0.10862	-0.27082
0.246612	0.0236	0.617574	0.04785	0.003238	5.38E-07	0.032275	0.224152	0.002073
-0.01717	-0.09567	-0.00624	-0.29456	-0.50233	0.392727	0.313055	0.289344	0.237945
0.848039	0.284622	0.944534	0.000775	1.77E-09	4.95E-06	0.000339	0.000969	0.007064
-0.41102	0.153329	0.409018	0.390121	0.135424	-0.33917	-0.30899	-0.12654	-0.32597
1.59E-06	0.085243	1.8E-06	5.79E-06	0.128998	9.59E-05	0.000408	0.156306	0.000184
-0.20964	0.128414	0.306567	0.22542	0.060412	-0.24269	-0.10578	-0.02087	-0.18886
0.018007	0.150205	0.000456	0.010831	0.499871	0.005974	0.236547	0.815871	0.033461
-0.07103	-0.19042	-0.0761	0.107227	-0.15463	0.285221	-0.15441	0.109451	-0.01657
0.427424	0.032006	0.395113	0.230175	0.082586	0.001153	0.083044	0.220598	0.853311
-0.05612	-0.42716	-0.45557	-0.17914	-0.36605	0.664704	0.288094	0.582509	0.257401
0.530865	5.49E-07	7.38E-08	0.043888	2.31E-05	1.58E-17	0.001022	6.77E-13	0.003484
-0.2784	-0.31522	-0.18548	0.100245	-0.14025	0.324018	-0.07716	0.012373	0.118162
0.001528	0.000307	0.036821	0.262133	0.115779	0.000202	0.388534	0.890192	0.185806
-0.50807	-0.17722	0.01173	0.315785	-0.07006	0.171734	-0.22463	0.18181	-0.18494
1.08E-09	0.046234	0.89586	0.000299	0.433836	0.053536	0.011117	0.040785	0.037386
-0.41872	0.160294	0.300036	0.745481	0.531514	-0.48034	-0.67503	-0.30273	-0.56743
9.63E-07	0.071825	0.00061	9.01E-24	1.28E-10	1.1E-08	3.24E-18	0.000541	3.51E-12
-0.0104	-0.24891	-0.32015	-0.10479	-0.00278	0.343125	0.071943	0.076896	0.143915
0.907579	0.004773	0.000243	0.241022	0.975221	7.84E-05	0.421526	0.390184	0.106479
0.243082	0.256644	-0.01184	-0.24598	0.208845	-0.16704	0.093032	-0.20052	-0.01924
0.005892	0.003584	0.894874	0.00531	0.018456	0.060508	0.298199	0.023794	0.829985
-0.07093	0.312109	0.111656	0.134553	0.48157	-0.31035	-0.37675	-0.44782	-0.34443
0.428115	0.000354	0.211382	0.1315	9.94E-09	0.000384	1.27E-05	1.3E-07	7.33E-05
0.487907	-0.36339	-0.28004	-0.44616	-0.45595	0.539963	0.4698	0.447898	0.542142
5.95E-09	2.68E-05	0.001429	1.46E-07	7.17E-08	5.7E-11	2.51E-08	1.29E-07	4.61E-11

POLA1	POLA2	POLD3	POLE	POLE2	POLQ	POLR2D	POLR3D	POP1
blue	blue	blue	blue	blue	blue	blue	blue	blue
-0.34072	-0.27884	-0.39389	-0.4269	-0.28114	-0.46227	-0.16459	-0.33725	-0.30765
8.87E-05	0.001501	4.61E-06	5.59E-07	0.001366	4.47E-08	0.064442	0.000106	0.000434
-0.34506	-0.24467	-0.44901	-0.42047	-0.26169	-0.47286	-0.12735	-0.36665	-0.29356
7.1E-05	0.005567	1.19E-07	8.58E-07	0.00296	1.98E-08	0.153635	2.24E-05	0.000809
0.778	0.778708	0.785231	0.754008	0.794017	0.902486	0.721594	0.755694	0.815981
5.34E-27	4.48E-27	8.6E-28	1.44E-24	8.5E-29	1.48E-47	1.07E-21	9.9E-25	1.54E-31
0.747522	0.452801	0.767608	0.316678	0.761528	0.707903	0.746524	0.43355	0.590801
5.84E-24	9.04E-08	6.54E-26	0.000286	2.67E-25	1.33E-20	7.23E-24	3.55E-07	2.64E-13
-0.10504	0.142986	0.064523	-0.38621	0.150364	-0.17361	0.256667	-0.00514	-0.06172
0.239883	0.108781	0.471092	7.31E-06	0.091532	0.050933	0.003581	0.954255	0.490614
0.414372	0.265713	0.547796	-0.21808	0.601872	0.293435	0.690775	0.136078	0.243905
1.28E-06	0.002534	2.64E-11	0.013778	7.21E-14	0.000813	2.55E-19	0.127142	0.005721
0.169597	0.025352	0.18037	-0.22658	0.294122	0.050614	0.376292	0.018608	0.089893
0.056624	0.777232	0.042433	0.01042	0.00079	0.571995	1.3E-05	0.835502	0.314867
0.257471	-0.19659	0.19552	-0.31264	-0.15679	-0.06379	-0.05647	-0.4172	-0.22371
0.003475	0.026744	0.027601	0.000346	0.078345	0.476157	0.528299	1.06E-06	0.011463
0.297722	0.240741	0.342969	0.309508	0.420546	0.423679	0.232252	0.500499	0.259652
0.000675	0.006403	7.9E-05	0.000399	8.54E-07	6.93E-07	0.008602	2.07E-09	0.003199
-0.15641	-0.15025	-0.08285	-0.35816	-0.06904	-0.21678	-0.16578	-0.12525	-0.33421
0.079089	0.091774	0.354442	3.56E-05	0.440516	0.014367	0.062501	0.160587	0.000123
0.066434	-0.07171	0.093417	-0.24617	0.113641	-0.03175	0.019787	-0.13197	-0.19135
0.45803	0.423063	0.296191	0.005274	0.203325	0.723059	0.825249	0.139133	0.031158
-0.09027	0.035068	-0.15166	0.265358	-0.11158	0.007871	-0.24703	0.2237	0.018461
0.312857	0.695495	0.088735	0.002569	0.211711	0.930015	0.005111	0.011466	0.83679
0.28032	0.328151	0.139988	0.758579	0.079213	0.427602	-0.11985	0.496517	0.394943
0.001413	0.000166	0.116479	5.2E-25	0.376023	5.33E-07	0.179565	2.91E-09	4.33E-06
-0.16476	0.14301	-0.1679	0.294462	-0.14069	-0.04239	-0.33663	0.252889	-0.04532
0.064164	0.108722	0.059187	0.000778	0.114651	0.636089	0.000109	0.004124	0.612868
-0.12542	0.00064	-0.19733	0.210061	-0.22131	-0.06134	-0.46787	0.156574	-0.15553
0.160038	0.994298	0.026169	0.01777	0.012402	0.493289	2.92E-08	0.078762	0.080805
-0.56631	-0.40465	-0.69152	-0.42728	-0.69722	-0.6987	-0.62838	-0.57559	-0.5035
3.95E-12	2.38E-06	2.26E-19	5.44E-07	8.61E-20	6.68E-20	2.6E-15	1.45E-12	1.6E-09
-0.0947	0.077246	-0.19373	0.367586	-0.2198	0.041936	-0.16706	0.067886	0.157903
0.289572	0.388029	0.029081	2.12E-05	0.013029	0.639693	0.060483	0.448242	0.076225
-0.06338	-0.19703	-0.02063	-0.16017	-0.021	-0.05418	0.191675	-0.29766	0.010263
0.47903	0.026401	0.81794	0.072052	0.81469	0.545163	0.030868	0.000677	0.908829
-0.48485	-0.42772	-0.50832	-0.2325	-0.52414	-0.45568	-0.33617	-0.4749	-0.3137
7.63E-09	5.29E-07	1.06E-09	0.00853	2.55E-10	7.32E-08	0.000112	1.69E-08	0.000329
0.211055	0.509281	0.297718	0.512035	0.250503	0.421254	0.332168	0.625108	0.685692
0.017226	9.7E-10	0.000675	7.6E-10	0.004504	8.15E-07	0.000136	3.99E-15	5.9E-19

POU3F2	PPAT	PPIA	PPM1D	PPM1G	PPP1CC	PPP1R3E	PPP2R5E	PPP4R1
blue	blue	blue	blue	blue	blue	blue	blue	blue
-0.35644	-0.28698	-0.15045	-0.3395	-0.3328	-0.24741	0.421908	-0.11676	-0.34282
3.9E-05	0.001071	0.091354	9.43E-05	0.000132	0.005042	7.8E-07	0.191099	7.97E-05
-0.38977	-0.23087	-0.01171	-0.32383	-0.36331	-0.242	0.500625	-0.1194	-0.34742
5.91E-06	0.009017	0.896	0.000204	2.69E-05	0.006124	2.05E-09	0.181221	6.28E-05
0.714233	0.621218	0.514152	0.726509	0.614686	0.664686	-0.51326	0.579837	0.713111
4.22E-21	6.58E-15	6.3E-10	4.17E-22	1.5E-14	1.58E-17	6.82E-10	9.11E-13	5.19E-21
0.633115	0.702692	0.427956	0.63203	0.185608	0.675555	-0.35216	0.613678	0.790123
1.39E-15	3.34E-20	5.2E-07	1.61E-15	0.036689	2.99E-18	4.9E-05	1.71E-14	2.4E-28
-0.35717	-0.06193	0.338834	0.051101	-0.20828	0.12314	0.130357	-0.008	0.021918
3.75E-05	0.489136	9.75E-05	0.568302	0.018782	0.167816	0.14408	0.928914	0.806772
0.153792	0.491891	0.462409	0.399129	-0.2003	0.540237	-0.12538	0.452569	0.58422
0.084295	4.28E-09	4.42E-08	3.35E-06	0.023956	5.56E-11	0.160171	9.2E-08	5.59E-13
-0.03582	0.249455	0.401722	0.18058	-0.14256	0.176851	-0.02119	0.063242	0.408803
0.689278	0.00468	2.85E-06	0.04219	0.109851	0.0467	0.813106	0.479961	1.83E-06
-0.06918	-0.03686	-0.07757	-0.1447	-0.1759	-0.05069	0.120962	-0.24875	0.135511
0.43962	0.680727	0.386048	0.104553	0.047913	0.571383	0.175523	0.004803	0.12875
0.221464	0.080721	0.062009	0.212901	0.44577	0.293617	-0.60175	0.097237	0.444857
0.012341	0.36697	0.488587	0.016254	1.5E-07	0.000807	7.32E-14	0.276795	1.61E-07
-0.29247	-0.42958	-0.27842	-0.2498	-0.02875	-0.16279	-0.10826	-0.33611	0.016484
0.000848	4.66E-07	0.001527	0.004621	0.748323	0.06745	0.225704	0.000112	0.854058
-0.00274	-0.10879	-0.15823	-0.1278	-0.04733	0.011032	-0.08635	-0.01857	0.098604
0.975608	0.223416	0.075614	0.152174	0.597229	0.902032	0.334403	0.83582	0.270066
-0.02081	-0.22216	-0.08175	-0.07397	0.330812	-0.09615	-0.02303	-0.17934	-0.06398
0.816344	0.012062	0.36087	0.408515	0.000145	0.282207	0.79714	0.043652	0.47487
0.436733	-0.07113	-0.16163	0.177355	0.731284	-0.00466	-0.2718	-0.08867	0.09312
2.85E-07	0.426821	0.069464	0.046068	1.64E-22	0.958507	0.001994	0.321545	0.297738
0.030016	-0.42826	-0.26444	-0.07485	0.496368	-0.18645	-0.16491	-0.35834	-0.38238
0.737628	5.1E-07	0.002662	0.402975	2.94E-09	0.035831	0.063909	3.53E-05	9.15E-06
-0.03739	-0.58811	-0.47751	-0.2342	0.481438	-0.29048	-0.18068	-0.52769	-0.20948
0.676395	3.6E-13	1.37E-08	0.008046	1E-08	0.000923	0.04207	1.83E-10	0.018094
-0.50444	-0.59188	-0.28204	-0.4944	-0.37376	-0.5366	0.605758	-0.49672	-0.62885
1.48E-09	2.33E-13	0.001316	3.47E-09	1.5E-05	7.89E-11	4.52E-14	2.86E-09	2.45E-15
0.146625	0.008779	0.065687	0.046665	0.193799	-0.10472	0.080207	0.01418	-0.26622
0.09998	0.921963	0.463113	0.602383	0.029024	0.241329	0.370041	0.874281	0.002484
0.083447	0.485311	0.258265	0.10155	-0.51164	0.15015	0.194521	0.470016	-0.05805
0.350961	7.35E-09	0.003372	0.255942	7.87E-10	0.092	0.02842	2.47E-08	0.5168
-0.33541	-0.05094	-0.03493	-0.32228	-0.45385	-0.36533	0.383636	-0.05092	-0.52114
0.000116	0.569487	0.696668	0.00022	8.37E-08	2.41E-05	8.5E-06	0.569691	3.35E-10
0.178915	0.185218	0.305256	0.390927	0.370304	0.230949	-0.06886	0.228177	0.275122
0.044156	0.037093	0.000484	5.52E-06	1.83E-05	0.008992	0.441729	0.009876	0.001745

PRC1	PRELP	PRIM1	PRKCD	PRKD1	PRMT6	PROSER1	PRPF38A	PRPF4
blue	blue	blue	blue	blue	blue	blue	blue	blue
-0.48167	0.066253	-0.19347	0.36367	-0.2346	-0.28869	-0.31961	-0.37783	-0.16553
9.86E-09	0.459258	0.029306	2.64E-05	0.007935	0.000996	0.00025	1.19E-05	0.062909
-0.46271	0.120798	-0.18261	0.229611	-0.26157	-0.24352	-0.3271	-0.40021	-0.06992
4.32E-08	0.176112	0.03989	0.00941	0.002973	0.0058	0.000174	3.13E-06	0.434734
0.886502	-0.53701	0.714117	-0.71763	0.690517	0.566783	0.58219	0.784186	0.680653
1.17E-43	7.59E-11	4.31E-21	2.25E-21	2.67E-19	3.75E-12	7.01E-13	1.12E-27	1.33E-18
0.509425	-0.47748	0.593665	-0.55132	0.690533	0.530282	0.442778	0.700296	0.623711
9.57E-10	1.38E-08	1.9E-13	1.86E-11	2.66E-19	1.44E-10	1.86E-07	5.07E-20	4.78E-15
-0.25469	-0.10255	0.343576	-0.04196	-0.17005	0.168846	-0.48997	-0.13013	0.255953
0.003857	0.251249	7.66E-05	0.639486	0.05596	0.057745	5.02E-09	0.144791	0.003679
0.051526	-0.37228	0.570174	-0.2883	0.33919	0.471173	-0.07683	0.332363	0.541357
0.565083	1.63E-05	2.61E-12	0.001013	9.58E-05	2.26E-08	0.390568	0.000135	4.98E-11
-0.10175	-0.02081	0.165527	-0.13386	0.185287	0.09062	-0.01077	0.150445	0.435847
0.254992	0.816384	0.062914	0.133533	0.037021	0.310954	0.904329	0.091356	3.03E-07
-0.3465	0.103487	0.052583	0.131752	-0.13769	-0.00791	-0.12278	0.141001	-0.04468
6.59E-05	0.246938	0.557119	0.139802	0.122656	0.929629	0.169073	0.113832	0.617933
0.307593	-0.08451	0.006497	-0.07864	0.347819	0.050814	0.244423	0.205538	0.300168
0.000435	0.344818	0.94221	0.379498	6.15E-05	0.570476	0.005616	0.020438	0.000606
-0.47333	0.056745	-0.28866	0.5206	-0.13739	-0.21588	-0.25181	-0.30203	-0.14445
1.91E-08	0.526298	0.000998	3.52E-10	0.12348	0.014783	0.004292	0.000558	0.105178
-0.26983	-0.10169	-0.04365	0.350824	0.06408	-0.00975	-0.15518	-0.04056	-0.01957
0.002156	0.255277	0.626055	5.26E-05	0.474148	0.913351	0.081495	0.650738	0.827165
0.178454	0.157243	-0.25214	-0.01073	0.114972	-0.34183	0.133739	-0.08199	-0.08711
0.044714	0.077476	0.00424	0.904679	0.19805	8.38E-05	0.133872	0.359477	0.330165
0.521077	-0.07975	-0.07566	-0.25434	0.302507	-0.13646	0.556691	0.34418	0.042365
3.37E-10	0.372772	0.397853	0.003907	0.000547	0.126059	1.08E-11	7.43E-05	0.636268
0.052814	0.006416	-0.12546	-0.01601	-0.20486	-0.1034	-0.07532	-0.17014	-0.18691
0.555384	0.942932	0.159883	0.858206	0.020864	0.247356	0.400008	0.055826	0.035363
-0.10841	0.111994	-0.439	0.280311	-0.11597	-0.35136	0.069532	-0.1756	-0.30015
0.225045	0.209994	2.43E-07	0.001413	0.194143	5.11E-05	0.437291	0.048294	0.000607
-0.56609	0.414761	-0.51483	0.428691	-0.58405	-0.32083	-0.21245	-0.56039	-0.60357
4.04E-12	1.25E-06	5.93E-10	4.95E-07	5.7E-13	0.000236	0.016484	7.34E-12	5.88E-14
0.331349	0.141125	-0.02489	-0.22579	-0.00402	0.03387	0.287142	0.10469	-0.12205
0.000142	0.113512	0.781177	0.010699	0.964191	0.705409	0.001064	0.241457	0.171654
0.088036	-0.00307	0.200342	-0.15457	-0.03375	0.289099	0.002179	0.000269	-0.00676
0.325009	0.972697	0.023923	0.082709	0.706378	0.000979	0.980607	0.997609	0.939905
-0.18358	0.350571	-0.22872	0.203334	-0.42473	-0.19123	-0.21685	-0.36341	-0.35458
0.038834	5.33E-05	0.009699	0.021859	6.46E-07	0.031265	0.014333	2.68E-05	4.31E-05
0.546213	-0.19256	0.340002	-0.44306	0.333734	0.041767	0.470811	0.361689	0.351881
3.09E-11	0.030091	9.19E-05	1.83E-07	0.000126	0.641042	2.33E-08	2.94E-05	4.97E-05

PRR11	PSAP	PSMC3IP	PTGFRN	PTN	PTPRM	PTPRU	PTPRZ1	PTTG1
blue	blue	blue	blue	blue	blue	blue	blue	blue
-0.48178	0.403258	-0.38363	-0.44919	-0.38373	0.25383	0.301201	-0.47566	-0.33703
9.78E-09	2.59E-06	8.5E-06	1.18E-07	8.45E-06	0.003982	0.000579	1.59E-08	0.000107
-0.48497	0.310545	-0.34392	-0.49104	-0.34278	0.270407	0.310238	-0.4286	-0.28592
7.56E-09	0.00038	7.53E-05	4.6E-09	7.98E-05	0.002107	0.000386	4.98E-07	0.00112
0.861829	-0.60622	0.77227	0.716507	0.720322	-0.63722	-0.52756	0.746182	0.845678
1.16E-38	4.27E-14	2.16E-26	2.78E-21	1.36E-21	8E-16	1.85E-10	7.77E-24	6.85E-36
0.565105	-0.55133	0.541244	0.644265	0.608737	-0.52918	-0.48633	0.686295	0.545509
4.48E-12	1.85E-11	5.04E-11	3.04E-16	3.14E-14	1.6E-10	6.76E-09	5.35E-19	3.31E-11
-0.14945	-0.32881	-0.08863	-0.41734	-0.15848	-0.18242	-0.00727	-0.3074	0.258424
0.093534	0.00016	0.321759	1.05E-06	0.075144	0.0401	0.935341	0.000439	0.003352
0.162203	-0.5221	0.189061	0.140558	0.23214	-0.38643	-0.28173	0.215967	0.432659
0.068463	3.07E-10	0.033272	0.114984	0.008634	7.21E-06	0.001333	0.014744	3.77E-07
-0.0271	-0.27508	0.06928	0.059689	0.184101	-0.10137	-0.08176	0.174036	0.138282
0.762303	0.001748	0.438954	0.505026	0.038271	0.256786	0.360823	0.050364	0.121045
-0.2226	0.065355	-0.0334	-0.10576	-0.3079	0.055293	-0.02891	-0.15878	-0.22128
0.011888	0.465381	0.709307	0.23666	0.000429	0.536952	0.746924	0.074585	0.012416
0.414477	-0.03783	0.440923	0.598024	0.234353	-0.05629	0.016674	0.248302	0.263553
1.27E-06	0.672873	2.12E-07	1.14E-13	0.008003	0.5296	0.852401	0.004881	0.002755
-0.25425	0.42943	-0.10942	-0.10449	-0.37415	0.188078	0.250295	-0.36999	-0.23271
0.00392	4.71E-07	0.220747	0.242374	1.47E-05	0.034216	0.004538	1.86E-05	0.008469
-0.14848	0.278079	-0.04032	0.067643	-0.17994	-0.02396	-0.04029	-0.13315	-0.11984
0.095718	0.001548	0.652658	0.449874	0.042942	0.789186	0.652903	0.135596	0.179573
0.190342	0.106498	0.335138	0.169544	0.022499	0.041302	-0.00517	0.077678	-0.17039
0.032075	0.233378	0.000117	0.056703	0.801756	0.644768	0.953987	0.385368	0.055458
0.477264	0.094375	0.522239	0.528568	0.304097	-0.13868	-0.09723	0.378868	0.146006
1.4E-08	0.291243	3.03E-10	1.69E-10	0.000509	0.119955	0.276812	1.12E-05	0.101436
0.115977	0.185899	0.168614	0.028355	-0.09491	-0.12942	0.100353	-0.16426	0.014
0.194131	0.036391	0.058095	0.751665	0.288518	0.147022	0.261617	0.064983	0.875859
0.025893	0.521567	0.175875	0.147959	-0.21607	0.140035	0.229886	-0.1892	-0.21591
0.772612	3.23E-10	0.047944	0.096899	0.014696	0.116354	0.009323	0.033137	0.014769
-0.57658	0.539408	-0.5003	-0.67256	-0.42208	0.444103	0.291805	-0.46715	-0.58766
1.3E-12	6.02E-11	2.11E-09	4.76E-18	7.71E-07	1.7E-07	0.000872	3.08E-08	3.79E-13
0.178993	-0.02449	0.013916	-0.01219	0.270306	-0.01967	-0.21924	0.246675	0.048398
0.044063	0.784632	0.876598	0.891802	0.002116	0.826262	0.013267	0.005178	0.588964
-0.10443	-0.26523	-0.36042	-0.16555	0.169234	0.003939	-0.15762	0.15205	-0.01586
0.242646	0.002581	3.15E-05	0.062876	0.057164	0.964943	0.076754	0.087913	0.8595
-0.4037	0.101588	-0.57395	-0.51324	-0.21561	0.369316	0.170589	-0.25252	-0.34201
2.52E-06	0.255761	1.74E-12	6.83E-10	0.014912	1.93E-05	0.055172	0.004181	8.3E-05
0.482481	-0.42561	0.382184	0.153246	0.407647	-0.20275	-0.16469	0.32547	0.505282
9.24E-09	6.09E-07	9.25E-06	0.085415	1.97E-06	0.022252	0.064275	0.000189	1.37E-09

PYCR1	RAB39A	RACGAP1	RAD18	RAD21	RAD23B	RAD51	RAD51AP1	RAD51D
blue	blue	blue	blue	blue	blue	blue	blue	blue
-0.36622	-0.28282	-0.42542	-0.36938	-0.16929	-0.26382	-0.42844	-0.26372	-0.10733
2.29E-05	0.001274	6.17E-07	1.92E-05	0.057075	0.002727	5.03E-07	0.002737	0.229744
-0.29162	-0.37624	-0.35059	-0.30924	-0.16654	-0.23381	-0.43757	-0.244	-0.05588
0.000879	1.3E-05	5.32E-05	0.000404	0.061292	0.008155	2.69E-07	0.005701	0.532649
0.701514	0.655929	0.869738	0.836519	0.533029	0.621213	0.871733	0.799485	0.453786
4.1E-20	5.78E-17	3.76E-40	1.86E-34	1.11E-10	6.59E-15	1.53E-40	1.9E-29	8.41E-08
0.276771	0.636504	0.652488	0.816986	0.490641	0.506738	0.578371	0.642065	0.435296
0.001633	8.81E-16	9.5E-17	1.13E-31	4.75E-09	1.21E-09	1.07E-12	4.12E-16	3.15E-07
0.045413	-0.25532	0.161728	0.024579	-0.17766	-0.24453	-0.06415	0.191592	0.264319
0.612167	0.003767	0.069288	0.783855	0.045693	0.005595	0.473684	0.030942	0.002674
0.01219	0.277822	0.456275	0.550128	0.209559	0.18117	0.246673	0.517498	0.447436
0.891807	0.001565	7E-08	2.09E-11	0.018051	0.041511	0.005179	4.67E-10	1.34E-07
-0.05388	0.076566	0.18389	0.340583	-0.04348	0.071173	-0.00047	0.134679	0.177042
0.547452	0.392227	0.038496	8.93E-05	0.627434	0.42652	0.995832	0.131135	0.04646
-0.55593	-0.10165	-0.17639	-0.12632	-0.14255	-0.14648	-0.28659	-0.14105	0.005932
1.16E-11	0.255465	0.047287	0.157036	0.109875	0.100331	0.001089	0.113699	0.947231
0.388972	0.323068	0.138759	0.35573	-0.20873	0.199745	0.286484	0.181655	0.083189
6.2E-06	0.000212	0.119754	4.06E-05	0.018521	0.024354	0.001093	0.040959	0.352454
-0.3003	-0.12862	-0.44089	-0.29278	-0.51992	-0.38933	-0.34708	-0.13858	-0.10732
0.000603	0.14956	2.13E-07	0.000837	3.75E-10	6.07E-06	6.4E-05	0.120248	0.229781
-0.26922	0.11197	-0.24498	-0.07558	-0.25094	-0.22848	-0.13162	0.042948	-0.01102
0.002208	0.210095	0.005505	0.398374	0.004431	0.009776	0.140196	0.631631	0.902136
0.354531	-0.10227	-0.11353	-0.02476	-0.06437	-0.01566	0.032155	-0.21374	-0.15947
4.32E-05	0.252565	0.203774	0.782336	0.472131	0.861254	0.719685	0.015827	0.073316
0.499128	0.265388	0.120226	0.153145	0.063032	0.215404	0.356217	0.059642	-0.06661
2.33E-09	0.002566	0.178185	0.085624	0.481425	0.015011	3.95E-05	0.505357	0.456831
0.392709	-0.13477	-0.12104	-0.29257	-0.3485	-0.3154	0.02187	-0.15617	-0.09002
4.95E-06	0.13088	0.175229	0.000844	5.94E-05	0.000304	0.807192	0.079549	0.314172
0.122772	-0.13293	-0.41124	-0.35875	-0.49152	-0.32297	-0.16388	-0.27847	-0.25639
0.1691	0.136258	1.56E-06	3.45E-05	4.41E-09	0.000213	0.065608	0.001524	0.003618
-0.49915	-0.67641	-0.57699	-0.68796	-0.13907	-0.40718	-0.63087	-0.55086	-0.33491
2.33E-09	2.61E-18	1.25E-12	4.07E-19	0.118923	2.03E-06	1.87E-15	1.94E-11	0.000119
0.262622	-0.05311	0.1549	-0.06891	0.34644	0.185767	0.160571	-0.04209	-0.13528
0.002855	0.553171	0.082055	0.441442	6.61E-05	0.036525	0.071329	0.638483	0.129417
-0.21509	0.093235	0.229352	0.120007	0.551969	0.316897	0.07329	0.073862	0.023093
0.015164	0.297139	0.009493	0.178979	1.74E-11	0.000283	0.412859	0.409213	0.796638
-0.29802	-0.31007	-0.20234	-0.39089	0.123781	-0.02204	-0.28365	-0.3658	-0.26412
0.000666	0.000389	0.02253	5.53E-06	0.165598	0.805744	0.001231	2.35E-05	0.002695
0.556512	0.1867	0.500796	0.480856	0.370655	0.437047	0.483736	0.352579	0.130163
1.1E-11	0.035578	2.02E-09	1.05E-08	1.79E-05	2.79E-07	8.35E-09	4.79E-05	0.144683

RAD54L	RAF1	RAN	RANBP1	RASGEF1B	RBBP4	RBBP9	RBL1	RBM12
blue	blue	blue	blue	blue	blue	blue	blue	blue
-0.40055	-0.28519	-0.10361	-0.32461	0.337893	-0.39341	-0.20664	-0.33959	-0.26117
3.07E-06	0.001155	0.246363	0.000197	0.000102	4.75E-06	0.019761	9.39E-05	0.003019
-0.38463	-0.30241	-0.01889	-0.3222	0.306971	-0.37715	-0.17926	-0.39136	-0.24717
8.02E-06	0.000549	0.833081	0.000221	0.000448	1.24E-05	0.043744	5.37E-06	0.005087
0.888135	0.629618	0.451679	0.661625	-0.6386	0.788356	0.739162	0.837076	0.788977
5E-44	2.21E-15	9.82E-08	2.5E-17	6.63E-16	3.82E-28	3.35E-23	1.53E-34	3.25E-28
0.535566	0.455135	0.346656	0.22908	-0.50888	0.691633	0.749145	0.779051	0.753306
8.71E-11	7.62E-08	6.54E-05	0.00958	1E-09	2.21E-19	4.13E-24	4.11E-27	1.68E-24
-0.19029	-0.19459	0.355957	0.302442	0.200037	-0.03908	0.048688	-0.28003	-0.01049
0.032121	0.028361	4.01E-05	0.000548	0.024143	0.662638	0.586732	0.00143	0.906784
0.096484	0.0895	0.443577	0.124663	-0.12384	0.373741	0.519035	0.33886	0.48148
0.28055	0.316997	1.76E-07	0.162581	0.165378	1.5E-05	4.06E-10	9.74E-05	1E-08
-0.0795	0.145045	0.22871	-0.04843	-0.05174	0.125381	0.326943	0.017853	0.16481
0.374275	0.103732	0.009701	0.588727	0.563459	0.160154	0.000176	0.84209	0.064078
-0.32311	-0.12048	-0.10445	-0.26409	0.170778	0.099951	-0.24677	-0.01751	-0.18705
0.000211	0.177252	0.242533	0.002699	0.054899	0.263543	0.00516	0.845077	0.035231
0.376152	0.498649	0.059118	0.103603	-0.23985	0.144701	0.155289	0.485586	0.302838
1.31E-05	2.43E-09	0.509112	0.246407	0.006606	0.104562	0.08128	7.19E-09	0.000539
-0.25222	-0.0153	-0.22111	-0.31673	0.429394	-0.38583	-0.32809	-0.05283	-0.25058
0.004227	0.864445	0.012485	0.000286	4.72E-07	7.47E-06	0.000166	0.555284	0.004491
-0.10962	0.008198	-0.12345	-0.28959	0.167712	-0.13764	-0.05275	0.133375	-0.00038
0.219874	0.927116	0.166747	0.000959	0.05947	0.1228	0.555888	0.134943	0.996596
0.227356	0.241295	-0.20289	0.002107	-0.19407	-0.04902	-0.01582	0.011304	-0.00088
0.010152	0.006278	0.022158	0.981245	0.028795	0.584162	0.859885	0.899622	0.992163
0.609397	0.532657	-0.24438	0.313125	-0.38932	0.233045	0.073059	0.422946	0.1316
2.9E-14	1.15E-10	0.005624	0.000338	6.07E-06	0.008371	0.414338	7.28E-07	0.140261
0.181146	0.067454	-0.2585	0.408211	-0.02817	-0.14231	-0.34155	-0.0875	-0.24365
0.041538	0.451141	0.003342	1.9E-06	0.753261	0.110485	8.5E-05	0.32799	0.005773
0.12709	0.225047	-0.50004	-0.00779	0.128682	-0.2954	-0.44352	0.03477	-0.33872
0.154489	0.010965	2.16E-09	0.930735	0.149349	0.000747	1.77E-07	0.697952	9.81E-05
-0.48108	-0.43487	-0.35457	-0.35477	0.46979	-0.50129	-0.51845	-0.63088	-0.66668
1.03E-08	3.24E-07	4.31E-05	4.27E-05	2.52E-08	1.94E-09	4.28E-10	1.87E-15	1.17E-17
0.192464	0.049325	0.010508	0.29036	-0.18783	0.145061	0.034548	-0.13864	-0.02985
0.030172	0.581836	0.906659	0.000928	0.034461	0.103694	0.699788	0.120088	0.738998
-0.21766	-0.3811	0.310295	-0.15792	-0.08902	0.155672	0.25092	-0.1293	0.197801
0.013963	9.86E-06	0.000385	0.076189	0.319619	0.080523	0.004435	0.147401	0.025805
-0.46133	-0.49717	0.048011	-0.22797	0.202321	-0.2625	-0.25288	-0.59413	-0.3346
4.8E-08	2.75E-09	0.591948	0.009946	0.02254	0.002869	0.004125	1.8E-13	0.000121
0.543131	0.474358	0.272895	0.619085	-0.27701	0.339049	0.419867	0.246427	0.436945
4.19E-11	1.76E-08	0.001909	8.64E-15	0.001617	9.65E-05	8.93E-07	0.005225	2.81E-07

RBM15	RBM15B	RBM8A	RBMX	RCAN2	RCC1	RCC2	RCOR1	RDM1
blue	blue	blue	blue	blue	blue	blue	blue	blue
-0.13402	-0.38929	-0.44777	-0.31421	0.405855	-0.40017	-0.24024	-0.2908	-0.21741
0.133054	6.08E-06	1.3E-07	0.000321	2.2E-06	3.14E-06	0.006518	0.000911	0.014077
-0.12492	-0.42496	-0.43214	-0.31003	0.468211	-0.41379	-0.31998	-0.34156	-0.1839
0.161724	6.37E-07	3.91E-07	0.000389	2.84E-08	1.33E-06	0.000245	8.49E-05	0.03848
0.569187	0.710167	0.781687	0.887445	-0.77506	0.88768	0.63201	0.753718	0.556831
2.91E-12	8.85E-21	2.12E-27	7.18E-44	1.1E-26	6.35E-44	1.61E-15	1.53E-24	1.06E-11
0.61892	0.274647	0.622443	0.784115	-0.60092	0.583509	0.294767	0.715056	0.488678
8.83E-15	0.001779	5.63E-15	1.15E-27	8.08E-14	6.05E-13	0.000768	3.63E-21	5.58E-09
0.170831	-0.31679	-0.22885	0.045368	0.062366	0.073895	-0.38213	-0.32853	0.244555
0.054823	0.000285	0.009656	0.612521	0.486082	0.409002	9.28E-06	0.000163	0.005589
0.53993	-0.22076	0.161128	0.481163	-0.24184	0.301232	-0.17687	0.267478	0.475788
5.72E-11	0.012629	0.070341	1.03E-08	0.006159	0.000579	0.046671	0.002365	1.58E-08
0.23613	-0.1399	0.115792	0.245613	-0.0924	0.032841	-0.14519	0.094825	0.234421
0.007526	0.116712	0.194847	0.005381	0.3015	0.713962	0.103387	0.288939	0.007984
-0.02521	-0.2375	-0.16203	-0.05519	0.036689	-0.22607	-0.29618	-0.3079	0.082078
0.778411	0.007175	0.068763	0.537726	0.682165	0.010599	0.000722	0.000429	0.358949
0.219135	0.388259	0.266786	0.317544	-0.35189	0.47546	0.534163	0.492325	0.216412
0.013314	6.47E-06	0.00243	0.000275	4.97E-05	1.62E-08	9.96E-11	4.13E-09	0.014535
-0.04126	-0.25641	-0.38696	-0.21617	0.129737	-0.07457	0.069907	-0.15327	-0.08062
0.645129	0.003616	6.99E-06	0.01465	0.146013	0.404755	0.434814	0.085362	0.367602
0.06446	-0.20622	-0.1153	-0.04074	-0.06192	-0.06138	0.106299	0.019846	0.061181
0.471528	0.020013	0.196783	0.649276	0.489179	0.492985	0.234259	0.824734	0.494419
-0.28436	0.316954	0.363988	-0.00238	-0.04146	0.054343	0.375113	0.032818	-0.26865
0.001195	0.000283	2.59E-05	0.978853	0.643506	0.543981	1.39E-05	0.714153	0.002259
-0.00652	0.782839	0.46615	0.278462	-0.46759	0.43927	0.75198	0.399814	-0.06142
0.941969	1.59E-27	3.33E-08	0.001524	2.98E-08	2.39E-07	2.24E-24	3.21E-06	0.492707
-0.15587	0.34318	-0.0682	-0.16419	-0.20441	0.228574	0.301609	-0.197	-0.09093
0.080126	7.82E-05	0.446159	0.065099	0.021156	0.009745	0.000569	0.026426	0.309292
-0.22255	0.312141	-0.13198	-0.20913	-0.11266	0.115688	0.514681	-0.06624	-0.24108
0.011909	0.000353	0.139117	0.018295	0.20729	0.19525	6.01E-10	0.459342	0.006327
-0.59326	-0.40093	-0.53191	-0.60102	0.634333	-0.64637	-0.30201	-0.64178	-0.43259
1.99E-13	3E-06	1.23E-10	7.99E-14	1.18E-15	2.26E-16	0.000559	4.29E-16	3.79E-07
-0.24417	0.348563	0.256069	0.000382	-0.06776	-0.00667	0.147391	-0.04953	-0.157
0.005667	5.92E-05	0.003663	0.9966	0.449113	0.940644	0.098202	0.580238	0.077948
-0.00403	-0.32447	0.0278	-0.01998	0.238429	-0.30478	-0.49809	0.011602	0.024521
0.964156	0.000198	0.75637	0.823535	0.006946	0.000494	2.54E-09	0.896991	0.784352
-0.42635	-0.32086	-0.27479	-0.47693	0.558487	-0.59374	-0.5413	-0.436	-0.29972
5.8E-07	0.000235	0.001769	1.44E-08	8.94E-12	1.88E-13	5.01E-11	3E-07	0.000619
0.268548	0.593896	0.405105	0.521815	-0.36603	0.492688	0.434218	0.469098	0.092177
0.002268	1.85E-13	2.31E-06	3.15E-10	2.32E-05	4.01E-09	3.39E-07	2.65E-08	0.30268

RECQL4	REV3L	RFC2	RFC3	RFC4	RFC5	RFWD3	RGN	RHNO1
blue	blue	blue	blue	blue	blue	blue	blue	blue
-0.48465	-0.47612	-0.28084	-0.38598	-0.19216	-0.32777	-0.36305	0.141968	0.085789
7.75E-09	1.53E-08	0.001383	7.4E-06	0.03044	0.000169	2.73E-05	0.111351	0.337559
-0.49435	-0.45713	-0.24697	-0.33327	-0.16593	-0.25937	-0.40119	0.176162	0.145989
3.49E-09	6.57E-08	0.005123	0.000129	0.062269	0.003234	2.95E-06	0.047576	0.101478
0.698463	0.736435	0.748743	0.858238	0.744934	0.700414	0.844382	-0.63104	0.490189
6.95E-20	5.85E-23	4.5E-24	5.12E-38	1.01E-23	4.97E-20	1.11E-35	1.83E-15	4.93E-09
0.219371	0.525357	0.660571	0.641929	0.629596	0.708625	0.791626	-0.43172	0.370572
0.013212	2.28E-10	2.93E-17	4.2E-16	2.22E-15	1.17E-20	1.61E-28	4.03E-07	1.8E-05
-0.1662	-0.48093	0.343121	0.07275	0.322297	0.205021	-0.15786	-0.03246	0.396798
0.061839	1.05E-08	7.84E-05	0.416319	0.00022	0.020764	0.076306	0.717137	3.86E-06
-0.16991	-0.05156	0.615384	0.39433	0.583122	0.608239	0.418494	-0.21681	0.487092
0.056162	0.564811	1.38E-14	4.49E-06	6.32E-13	3.34E-14	9.77E-07	0.014348	6.36E-09
-0.2372	-0.05109	0.329041	0.1408	0.300547	0.273633	0.236993	-0.06797	0.350401
0.007251	0.568361	0.000159	0.114355	0.000596	0.001853	0.007303	0.44769	5.38E-05
-0.27611	-0.25239	-0.0459	-0.14885	0.011841	0.15669	0.018049	0.375224	-0.1881
0.001677	0.004201	0.608384	0.094883	0.894883	0.078538	0.840378	1.38E-05	0.034196
0.347458	0.206131	0.319925	0.118962	0.270839	0.132841	0.523236	-0.29451	0.080658
6.27E-05	0.02007	0.000246	0.182821	0.002071	0.136528	2.77E-10	0.000777	0.367349
-0.16061	-0.45942	-0.07727	-0.44172	-0.04016	-0.32404	-0.02023	0.17499	-0.0142
0.071267	5.54E-08	0.387886	2.01E-07	0.653909	0.000202	0.821428	0.049096	0.87409
-0.18293	-0.29659	0.07457	-0.25249	0.04375	-0.09377	0.092919	0.120952	0.055461
0.039541	0.00071	0.404723	0.004185	0.625267	0.29437	0.298788	0.175557	0.535713
0.133588	0.157859	-0.10792	-0.17083	-0.14387	-0.12416	-0.06785	-0.04519	-0.07313
0.134317	0.076307	0.227155	0.054829	0.106598	0.164306	0.448483	0.613946	0.413857
0.708665	0.604033	0.006958	0.16039	0.053587	-0.061	0.365168	-0.2475	-0.11056
1.16E-20	5.56E-14	0.938112	0.071654	0.549601	0.49572	2.43E-05	0.005026	0.215944
0.590721	-0.05238	-0.04624	-0.16539	-0.128	-0.27262	-0.14054	0.033582	-0.18934
2.67E-13	0.558643	0.605663	0.063131	0.151542	0.00193	0.11503	0.707795	0.033007
0.428708	-0.05335	-0.24162	-0.4184	-0.20367	-0.48837	0.002316	0.129004	-0.2613
4.94E-07	0.55141	0.006207	9.83E-07	0.021638	5.73E-09	0.979381	0.148325	0.003005
-0.35745	-0.37617	-0.64165	-0.5409	-0.57697	-0.63747	-0.64655	0.491619	-0.31258
3.7E-05	1.31E-05	4.37E-16	5.21E-11	1.25E-12	7.73E-16	2.21E-16	4.38E-09	0.000346
0.260101	0.394626	-0.15512	0.212335	-0.19387	-0.12194	-0.16192	-0.10678	-0.1444
0.003145	4.41E-06	0.081624	0.016546	0.028963	0.172026	0.068951	0.232134	0.105296
-0.40401	0.096347	-0.06844	0.276839	-0.11977	0.238591	-0.18475	-0.01474	-0.03044
2.47E-06	0.281235	0.444544	0.001628	0.179839	0.006906	0.037583	0.869394	0.734054
-0.43341	-0.15643	-0.47077	-0.15214	-0.48362	-0.24733	-0.62841	0.2432	-0.26543
3.58E-07	0.079038	2.33E-08	0.087724	8.43E-09	0.005056	2.59E-15	0.005867	0.002561
0.434912	0.484266	0.251981	0.554373	0.455425	0.216187	0.365582	-0.7093	0.285807
3.23E-07	8E-09	0.004265	1.36E-11	7.46E-08	0.01464	2.37E-05	1.04E-20	0.001125

RMI1	RMI2	RNASEH2/	RNF103	RNF212	RNF214	RPA1	RPP40	RRAGA
blue	blue	blue	blue	blue	blue	blue	blue	blue
-0.36558	-0.27436	-0.42327	0.325292	0.222057	-0.21916	-0.40404	-0.06495	0.319
2.38E-05	0.001799	7.12E-07	0.00019	0.012103	0.013303	2.47E-06	0.468151	0.000257
-0.34707	-0.21137	-0.37084	0.419193	0.30759	-0.22364	-0.4304	-0.00582	0.371852
6.4E-05	0.017055	1.77E-05	9.33E-07	0.000435	0.011487	4.41E-07	0.948213	1.67E-05
0.672571	0.75939	0.749195	-0.49939	-0.55049	0.54093	0.672565	0.5852	-0.68112
4.76E-18	4.33E-25	4.09E-24	2.28E-09	2.02E-11	5.19E-11	4.76E-18	5E-13	1.24E-18
0.674228	0.524973	0.297907	-0.2167	-0.51329	0.372173	0.511275	0.682774	-0.55019
3.68E-18	2.36E-10	0.00067	0.014399	6.81E-10	1.64E-05	8.13E-10	9.48E-19	2.08E-11
0.03951	0.171428	0.203842	-0.07155	0.118451	-0.19631	-0.30755	0.34916	0.265329
0.659194	0.053969	0.021524	0.424056	0.184722	0.026969	0.000436	5.74E-05	0.002572
0.513508	0.383577	0.109573	-0.06028	-0.24409	0.023047	0.065115	0.747392	-0.11649
6.67E-10	8.53E-06	0.220081	0.500795	0.005683	0.797032	0.467026	6.01E-24	0.192156
0.109938	0.149846	-0.07368	0.06578	-0.04371	0.055128	-0.08795	0.415513	-0.11703
0.21854	0.092668	0.410365	0.462483	0.62556	0.538167	0.325489	1.19E-06	0.190068
0.086816	-0.16974	-0.24063	0.034884	-0.03576	-0.26084	-0.04207	-0.0285	0.274908
0.331783	0.05642	0.006428	0.697012	0.689792	0.003058	0.63865	0.750399	0.00176
0.138201	0.146568	0.246944	-0.39059	-0.31666	0.280365	0.387963	0.209316	-0.31614
0.121263	0.100114	0.005128	5.63E-06	0.000287	0.00141	6.58E-06	0.018188	0.000294
-0.26056	-0.34567	-0.18571	-0.07317	0.025501	-0.25888	-0.1381	-0.00894	0.236257
0.00309	6.88E-05	0.036586	0.413625	0.775958	0.003294	0.121548	0.920523	0.007492
-0.01234	-0.17851	-0.20756	-0.04537	-0.12989	-0.17951	-0.02432	0.128447	0.066738
0.890499	0.04465	0.019204	0.612514	0.145543	0.043441	0.786063	0.1501	0.455975
-0.22087	-0.14449	0.100257	0.110667	0.07023	0.431239	0.160536	-0.36354	-0.09343
0.012581	0.105067	0.262078	0.215484	0.43269	4.16E-07	0.071392	2.66E-05	0.2961
-0.03634	0.123276	0.447794	-0.33935	-0.25324	0.469952	0.545445	-0.22886	-0.50342
0.685028	0.167344	1.3E-07	9.51E-05	0.004071	2.49E-08	3.34E-11	0.00965	1.61E-09
-0.25707	-0.04685	0.500279	-0.33039	-0.0135	-0.01271	0.163553	-0.33459	-0.03474
0.003527	0.600938	2.11E-09	0.000148	0.880235	0.887195	0.066164	0.000121	0.698208
-0.40641	-0.31785	0.167257	-0.23931	-0.04403	0.001486	0.182441	-0.40684	-0.03233
2.13E-06	0.000271	0.060173	0.006735	0.623017	0.986774	0.040078	2.07E-06	0.718236
-0.53122	-0.54169	-0.44297	0.492247	0.475679	-0.43003	-0.49746	-0.54458	0.490162
1.32E-10	4.82E-11	1.84E-07	4.16E-09	1.59E-08	4.52E-07	2.69E-09	3.63E-11	4.94E-09
-0.06196	0.042395	0.190471	0.024687	0.075143	0.104702	0.068221	-0.34641	-0.1146
0.488937	0.636034	0.031956	0.782931	0.401112	0.241402	0.446	6.62E-05	0.199521
0.291054	0.159449	-0.30916	0.342421	0.131399	-0.09658	-0.20906	0.112685	0.080319
0.000901	0.073357	0.000405	8.13E-05	0.140874	0.280083	0.018331	0.207177	0.369372
-0.22528	-0.20271	-0.4114	0.371805	0.420016	-0.23401	-0.44484	-0.3968	0.382801
0.01088	0.022274	1.55E-06	1.68E-05	8.84E-07	0.008098	1.61E-07	3.86E-06	8.93E-06
0.112546	0.484101	0.426569	-0.23811	-0.18313	0.59857	0.178268	0.20078	-0.47172
0.207743	8.11E-09	5.71E-07	0.007025	0.039321	1.07E-13	0.044942	0.02361	2.17E-08

RRM1	RRM2	RRP15	RRP1B	RTKN2	RUVBL1	S100A13	S100A14	SAAL1
blue	blue	blue	blue	blue	blue	blue	blue	blue
-0.31986	-0.45946	-0.27971	-0.30328	-0.41698	-0.37825	0.235692	0.261576	-0.24238
0.000247	5.52E-08	0.001449	0.000528	1.08E-06	1.16E-05	0.007641	0.002972	0.006042
-0.28115	-0.49344	-0.32383	-0.31038	-0.36422	-0.4269	0.353402	0.297903	-0.18581
0.001365	3.76E-09	0.000204	0.000383	2.56E-05	5.59E-07	4.59E-05	0.00067	0.036479
0.759772	0.852204	0.70174	0.619618	0.807449	0.597479	-0.47491	-0.47305	0.740912
3.98E-25	5.69E-37	3.94E-20	8.08E-15	1.97E-30	1.21E-13	1.69E-08	1.95E-08	2.34E-23
0.562889	0.611046	0.745447	0.455336	0.450035	0.429986	-0.24073	-0.29176	0.712609
5.66E-12	2.37E-14	9.08E-24	7.5E-08	1.11E-07	4.53E-07	0.006405	0.000874	5.69E-21
0.040474	-0.10295	-0.05736	-0.45848	-0.06946	0.143922	0.381366	0.146196	0.35278
0.651419	0.249428	0.521814	5.94E-08	0.437763	0.106463	9.7E-06	0.100988	4.74E-05
0.363068	0.280549	0.493024	-0.02121	0.125725	0.278317	0.152007	-0.05784	0.684479
2.73E-05	0.001399	3.9E-09	0.812892	0.159003	0.001533	0.088004	0.518335	7.19E-19
0.107105	0.00932	0.225221	-0.01337	-0.09387	0.166931	0.218695	0.200462	0.393858
0.230711	0.91717	0.010902	0.881409	0.293838	0.060682	0.013505	0.023837	4.62E-06
0.079338	-0.17887	-0.02369	-0.20083	-0.35138	0.291219	0.178053	0.370347	-0.03614
0.375263	0.044212	0.79151	0.023579	5.11E-05	0.000895	0.045205	1.82E-05	0.686678
0.329825	0.521733	0.368363	0.500979	0.040268	0.296973	-0.11202	-0.30472	0.321815
0.000153	3.18E-10	2.03E-05	1.99E-09	0.653075	0.000698	0.209895	0.000495	0.000225
-0.14576	-0.09814	-0.11951	-0.06034	-0.56841	-0.01225	0.279971	0.190453	-0.02965
0.10202	0.272337	0.180801	0.5004	3.16E-12	0.891306	0.001433	0.031973	0.740696
-0.05807	0.050896	0.11141	-0.0188	-0.37	0.0711	0.1722	0.161225	0.012806
0.516657	0.569858	0.212399	0.833863	1.86E-05	0.426997	0.052881	0.070169	0.886372
-0.22512	0.007129	-0.24555	0.221526	-0.02925	-0.01378	0.200301	-0.25451	-0.28749
0.010938	0.936597	0.005392	0.012316	0.744108	0.877778	0.023952	0.003882	0.001048
0.23243	0.366155	0.105727	0.580522	0.308837	0.257773	-0.45624	-0.24926	0.00264
0.008549	2.3E-05	0.236803	8.45E-13	0.000411	0.003435	7.02E-08	0.004713	0.976502
-0.05708	0.059171	-0.27017	-0.09452	-0.00806	0.149928	-0.06244	-0.02807	-0.15344
0.523854	0.508734	0.002127	0.290508	0.928313	0.092487	0.485563	0.754057	0.085005
-0.10467	0.024205	-0.26895	0.188067	-0.31699	0.072446	-0.06043	0.029537	-0.24038
0.24154	0.787065	0.002232	0.034228	0.000282	0.418274	0.499727	0.741671	0.006485
-0.55736	-0.70216	-0.67697	-0.37556	-0.45425	-0.49777	0.23632	0.42042	-0.62069
1E-11	3.67E-20	2.39E-18	1.36E-05	8.13E-08	2.62E-09	0.007476	8.61E-07	7.04E-15
-0.08585	-0.00639	-0.18935	0.05484	0.40921	-0.03635	-0.31134	-0.08915	-0.2724
0.337196	0.943188	0.033002	0.540297	1.78E-06	0.684931	0.000367	0.318878	0.001947
-0.09668	-0.13612	0.119822	-0.21283	0.307806	-0.2948	-0.06203	-0.04864	-0.11175
0.279584	0.127016	0.179656	0.016288	0.000431	0.000767	0.488458	0.587061	0.210989
-0.40618	-0.4723	-0.38388	-0.38471	-0.00123	-0.45533	0.072201	0.156421	-0.55315
2.16E-06	2.07E-08	8.38E-06	7.98E-06	0.989052	7.51E-08	0.419853	0.079058	1.54E-11
0.262103	0.277378	0.234466	0.418546	0.593285	0.279556	-0.52128	-0.35648	0.38363
0.002913	0.001593	0.007972	9.74E-07	1.98E-13	0.001458	3.31E-10	3.9E-05	8.5E-06

SAE1	SALL3	SASS6	SCML2	SDC4	SEC61G	SELENBP1	SEMA5A	SEMA5B
blue	blue	blue	blue	blue	blue	blue	blue	blue
-0.42829	-0.40954	-0.27012	-0.28399	0.210511	-0.28156	0.376336	-0.43104	-0.42172
5.08E-07	1.74E-06	0.002132	0.001214	0.017522	0.001342	1.3E-05	4.22E-07	7.9E-07
-0.36504	-0.37496	-0.28423	-0.22859	0.20494	-0.25929	0.383593	-0.42244	-0.41388
2.45E-05	1.4E-05	0.001202	0.009741	0.020816	0.003243	8.52E-06	7.53E-07	1.32E-06
0.546285	0.60839	0.765153	0.721419	-0.53315	0.483585	-0.72089	0.745911	0.576995
3.07E-11	3.28E-14	1.16E-25	1.11E-21	1.1E-10	8.45E-09	1.22E-21	8.23E-24	1.25E-12
0.096719	0.23633	0.777372	0.547166	-0.21856	0.369895	-0.68649	0.588133	0.318576
0.279374	0.007474	6.23E-27	2.81E-11	0.013566	1.87E-05	5.18E-19	3.59E-13	0.000262
-0.06851	-0.38489	-0.12277	-0.12133	0.127467	0.027233	0.109613	-0.36611	-0.3194
0.444089	7.9E-06	0.169113	0.174203	0.153261	0.76119	0.219912	2.31E-05	0.000252
-0.17009	-0.23207	0.460203	0.219962	0.06846	0.208757	-0.36604	0.08839	-0.10811
0.055903	0.008654	5.22E-08	0.012961	0.444405	0.018507	2.32E-05	0.323056	0.22636
-0.09992	-0.26567	0.122885	0.010194	0.113894	0.00854	-0.17129	0.097336	-0.03139
0.263678	0.002538	0.168707	0.909441	0.202317	0.924081	0.05417	0.276303	0.726059
-0.36763	-0.16473	-0.00121	-0.07549	0.317351	-0.10693	0.298329	-0.30719	-0.26835
2.12E-05	0.064205	0.989239	0.398957	0.000278	0.231464	0.000658	0.000443	0.002285
0.086474	-0.06479	0.237226	-0.01781	0.027625	0.437831	-0.43741	0.433654	0.055768
0.333698	0.469252	0.007244	0.84246	0.757856	2.64E-07	2.72E-07	3.52E-07	0.533452
-0.46557	-0.4658	-0.21899	-0.48697	0.466193	-0.01182	0.184349	-0.27288	-0.51915
3.48E-08	3.42E-08	0.013377	6.42E-09	3.32E-08	0.895075	0.038007	0.00191	4.02E-10
-0.40375	-0.30924	0.015607	-0.205	0.277499	-0.02731	0.035154	-0.1676	-0.24017
2.51E-06	0.000404	0.861746	0.02078	0.001585	0.760511	0.694782	0.059642	0.006532
0.079843	-0.03925	-0.12577	-0.11072	0.001475	-0.09059	-0.14044	0.132404	0.157411
0.372224	0.66131	0.158845	0.215253	0.986868	0.311099	0.115286	0.137835	0.077156
0.393644	0.576201	0.20924	0.279619	-0.3612	0.221178	-0.2283	0.510723	0.459351
4.68E-06	1.36E-12	0.018231	0.001454	3.02E-05	0.012457	0.009837	8.54E-10	5.57E-08
0.302771	0.175417	-0.20546	-0.1074	-0.10731	0.16549	0.172956	-0.07136	0.065674
0.00054	0.048538	0.020487	0.229404	0.229831	0.062973	0.051832	0.425292	0.463203
-0.05782	0.042496	-0.21631	-0.28856	0.092282	0.097341	0.208481	-0.00221	-0.10549
0.518484	0.635228	0.014581	0.001002	0.302123	0.27628	0.018666	0.980287	0.237857
-0.23492	-0.16186	-0.50602	-0.33949	0.083476	-0.41816	0.669254	-0.4603	-0.31057
0.007848	0.06905	1.29E-09	9.44E-05	0.350793	9.99E-07	7.92E-18	5.19E-08	0.00038
0.486127	0.566	-0.0989	0.246906	-0.44528	-0.08043	0.013523	0.239272	0.470919
6.88E-09	4.08E-12	0.268599	0.005135	1.56E-07	0.368729	0.880058	0.006743	2.31E-08
0.121225	0.081931	0.101407	0.237623	-0.15257	-0.20512	-0.07911	-0.02019	0.157377
0.174578	0.359812	0.256617	0.007144	0.08682	0.020703	0.37665	0.821766	0.077221
0.108432	0.000108	-0.44645	-0.12165	-0.00197	-0.39485	0.334684	-0.32105	0.016188
0.224953	0.999037	1.43E-07	0.173074	0.982465	4.35E-06	0.00012	0.000233	0.856649
0.352507	0.396474	0.324179	0.40991	-0.54044	0.104519	-0.36073	0.522513	0.307117
4.81E-05	3.94E-06	0.000201	1.7E-06	5.45E-11	0.242229	3.1E-05	2.96E-10	0.000445

SENP1	SENP8	SEPHS1	44450	SERBP1	SERPINF1	SET	SETBP1	SETD3
blue	blue	blue	blue	blue	blue	blue	blue	blue
-0.2096	-0.22376	-0.2535	-0.20523	-0.43418	0.510377	-0.2445	-0.22072	0.453576
0.018026	0.011444	0.004031	0.020632	3.4E-07	8.8E-10	0.0056	0.012642	8.54E-08
-0.19061	-0.12696	-0.20052	-0.2177	-0.50019	0.47092	-0.22202	-0.25533	0.523224
0.031829	0.154913	0.023795	0.013944	2.13E-09	2.31E-08	0.01212	0.003765	2.77E-10
0.696239	0.527491	0.544667	0.61494	0.769525	-0.6009	0.809352	0.602584	-0.68401
1.02E-19	1.87E-10	3.6E-11	1.46E-14	4.16E-26	8.1E-14	1.13E-30	6.62E-14	7.76E-19
0.655473	0.478761	0.111645	0.562304	0.712042	-0.39918	0.645136	0.491037	-0.44932
6.18E-17	1.24E-08	0.211431	6.01E-12	6.3E-21	3.34E-06	2.69E-16	4.59E-09	1.16E-07
-0.13302	0.239972	0.101682	-0.40306	-0.29464	0.283902	-0.06078	-0.33459	0.105234
0.136001	0.006579	0.25532	2.62E-06	0.000772	0.001218	0.497224	0.000121	0.239007
0.363849	0.44208	-0.0696	0.12766	0.282866	0.049109	0.340606	0.068083	-0.09417
2.61E-05	1.96E-07	0.436816	0.152635	0.001272	0.583492	8.92E-05	0.446926	0.292314
0.207824	0.271519	-0.15599	0.01886	0.012537	0.047397	0.215069	0.042933	-0.03522
0.019049	0.002016	0.079891	0.83331	0.888743	0.596696	0.015173	0.631748	0.694208
0.020046	-0.08308	-0.25858	-0.20969	0.037358	0.018153	-0.15923	-0.33273	-0.01835
0.822991	0.353089	0.003332	0.017976	0.676688	0.839469	0.073751	0.000132	0.83773
0.400908	-0.00252	-0.30015	0.273186	0.534795	0.093838	0.257018	0.179104	-0.28761
3E-06	0.977591	0.000607	0.001886	9.38E-11	0.294013	0.003534	0.04393	0.001043
-0.10126	-0.28092	-0.54653	-0.38424	-0.00454	0.535105	-0.28762	-0.45371	0.238596
0.257321	0.001378	2.99E-11	8.2E-06	0.959642	9.11E-11	0.001042	8.46E-08	0.006905
-0.00849	-0.19723	-0.49126	-0.08376	0.133085	0.336603	-0.10832	-0.14364	0.134426
0.924521	0.026248	4.51E-09	0.349172	0.135802	0.000109	0.225423	0.107156	0.131868
-0.1834	-0.04725	-0.01958	0.083678	-0.09567	-0.12686	0.006337	0.219746	-0.0324
0.039027	0.597839	0.82703	0.349624	0.284668	0.155255	0.943626	0.013052	0.717674
0.266452	-0.03547	0.30399	0.32009	0.420556	-0.48296	0.27504	0.369485	-0.45844
0.002462	0.69222	0.000512	0.000244	8.53E-07	8.89E-09	0.001751	1.91E-05	5.96E-08
-0.2195	-0.18609	0.198042	-0.33572	0.031497	-0.0825	-0.24506	-0.21439	-0.25774
0.013156	0.036198	0.025621	0.000114	0.725188	0.35646	0.005489	0.015505	0.003439
-0.10181	-0.40137	-0.19724	-0.24312	0.136949	0.098116	-0.24139	-0.25468	-0.07843
0.254721	2.92E-06	0.026239	0.005884	0.124706	0.272456	0.006257	0.003859	0.380785
-0.5274	-0.3597	-0.09103	-0.46272	-0.60135	0.195868	-0.43815	-0.52527	0.582328
1.88E-10	3.28E-05	0.308735	4.32E-08	7.67E-14	0.02732	2.58E-07	2.3E-10	6.91E-13
-0.15558	0.006046	0.572244	0.146641	-0.10133	-0.49299	0.132718	0.248737	-0.1309
0.080697	0.946215	2.09E-12	0.099943	0.256993	3.91E-09	0.136895	0.004804	0.142394
-0.05129	0.184668	0.197829	0.262533	-0.16989	-0.11573	0.084417	0.250589	0.125943
0.566854	0.037669	0.025784	0.002865	0.056188	0.19508	0.34537	0.004489	0.158276
-0.43082	-0.1342	0.14415	-0.10421	-0.60932	0.06943	-0.28772	-0.03927	0.339937
4.28E-07	0.132522	0.105904	0.243619	2.92E-14	0.437961	0.001038	0.661148	9.23E-05
0.363317	0.408	0.624737	0.317736	0.097447	-0.39675	0.526725	0.446145	-0.35593
2.69E-05	1.92E-06	4.18E-15	0.000273	0.275751	3.88E-06	2.01E-10	1.46E-07	4.01E-05

SF3B4	SFPQ	SH2B2	SH3BP4	SH3RF2	SHCBP1	SHMT2	SIM2	SIPA1L2
blue	blue	blue	blue	blue	blue	blue	blue	blue
-0.24024	-0.43732	-0.37996	-0.4139	0.253676	-0.38605	-0.2176	-0.36218	-0.28989
0.006517	2.73E-07	1.05E-05	1.32E-06	0.004005	7.38E-06	0.013991	2.86E-05	0.000947
-0.27316	-0.48418	-0.37817	-0.39852	0.253975	-0.44117	-0.19479	-0.33936	-0.34355
0.001889	8.06E-09	1.17E-05	3.48E-06	0.003961	2.09E-07	0.028194	9.5E-05	7.67E-05
0.600335	0.853817	0.627268	0.677002	-0.5938	0.814983	0.737741	0.474375	0.621022
8.66E-14	3.02E-37	3.01E-15	2.38E-18	1.87E-13	2.09E-31	4.48E-23	1.76E-08	6.75E-15
0.32935	0.59478	0.271894	0.372781	-0.37409	0.713487	0.5449	0.428039	0.599765
0.000156	1.67E-13	0.001986	1.59E-05	1.47E-05	4.84E-21	3.52E-11	5.17E-07	9.27E-14
-0.21974	-0.33257	-0.07102	-0.25996	0.008995	-0.18816	0.22174	-0.0607	-0.45072
0.013055	0.000133	0.427503	0.003162	0.920054	0.034141	0.01223	0.497817	1.05E-07
-0.05487	0.066186	-0.01758	-0.05129	-0.13228	0.356608	0.399506	0.234077	0.099584
0.540059	0.459713	0.844436	0.566863	0.138206	3.87E-05	3.27E-06	0.008079	0.265308
-0.01076	-0.08761	0.028361	-0.05605	0.086401	0.08775	0.20353	0.0742	0.183848
0.904474	0.327375	0.751608	0.531362	0.334108	0.326589	0.02173	0.407065	0.038542
-0.13383	-0.17022	-0.39563	-0.45297	0.09705	-0.04732	-0.24421	0.154117	-0.11184
0.13361	0.055705	4.15E-06	8.93E-08	0.277724	0.59729	0.005659	0.083633	0.210616
0.529973	0.345461	0.391626	0.274279	-0.15498	0.603971	0.461595	0.2243	0.508962
1.48E-10	6.95E-05	5.29E-06	0.001806	0.081902	5.6E-14	4.7E-08	0.011241	9.97E-10
0.022564	-0.29181	-0.15469	-0.36855	0.127995	0.029167	-0.05321	-0.10783	-0.13473
0.801192	0.000872	0.082466	2.01E-05	0.151551	0.744793	0.552426	0.227538	0.130984
0.077857	-0.13	-0.09858	-0.30929	0.030315	0.107919	0.016052	-0.04503	-0.0092
0.384273	0.145186	0.270162	0.000403	0.735114	0.227167	0.857842	0.615187	0.918248
0.455756	0.041206	0.279911	0.265521	0.063063	-0.039	0.086929	-0.08341	0.10688
7.27E-08	0.645535	0.001437	0.002553	0.481209	0.663345	0.331151	0.351202	0.2317
0.667102	0.601953	0.528321	0.558242	-0.33862	0.342383	0.266776	0.133414	0.505104
1.1E-17	7.14E-14	1.73E-10	9.17E-12	9.86E-05	8.15E-05	0.002431	0.13483	1.4E-09
0.284386	0.09569	0.418646	0.150377	-0.1981	-0.09926	0.185658	-0.15545	-0.17896
0.001194	0.284544	9.67E-07	0.091504	0.025577	0.266896	0.036638	0.08096	0.044107
0.390398	0.066797	0.242964	0.079821	-0.10194	0.044623	0.02648	-0.09922	0.052329
5.69E-06	0.455576	0.005917	0.372357	0.25412	0.618378	0.767598	0.267075	0.559026
-0.4741	-0.4656	-0.49086	-0.29186	0.223728	-0.65568	-0.64161	-0.30421	-0.51778
1.8E-08	3.47E-08	4.66E-09	0.000871	0.011455	5.99E-17	4.39E-16	0.000507	4.55E-10
0.056309	0.271759	0.163588	0.353334	-0.18631	-0.15754	-0.11417	0.012571	0.02186
0.52948	0.001997	0.066104	4.61E-05	0.035969	0.076904	0.201201	0.888447	0.807276
-0.51332	-0.07489	-0.31566	-0.08516	0.153201	-0.17402	-0.28351	-0.01203	-0.10205
6.79E-10	0.402696	0.0003	0.341127	0.085508	0.050387	0.001238	0.89325	0.253576
-0.52645	-0.37986	-0.37095	-0.24464	0.391609	-0.58058	-0.56653	-0.23648	-0.38578
2.06E-10	1.06E-05	1.76E-05	0.005572	5.29E-06	8.39E-13	3.86E-12	0.007434	7.49E-06
0.38593	0.488401	0.391391	0.666385	-0.32477	0.275494	0.401959	0.199865	0.389269
7.43E-06	5.71E-09	5.36E-06	1.23E-17	0.000195	0.001719	2.81E-06	0.024267	6.09E-06

SIX1	SKA1	SKA2	SKA3	SKP2	SLC14A1	SLC22A15	SLC22A6	SLC25A15
blue	blue	blue	blue	blue	blue	blue	blue	blue
-0.23447	-0.44995	-0.20101	-0.4872	-0.39619	0.033168	0.417581	0.161805	-0.43651
0.00797	1.11E-07	0.023452	6.3E-09	4.01E-06	0.711244	1.04E-06	0.069153	2.89E-07
-0.30742	-0.4321	-0.19562	-0.45773	-0.35887	0.092967	0.449514	0.163683	-0.39654
0.000439	3.92E-07	0.02752	6.28E-08	3.43E-05	0.298536	1.15E-07	0.065945	3.93E-06
0.637539	0.903728	0.58043	0.926423	0.746126	-0.53077	-0.68581	-0.61385	0.780481
7.66E-16	6.92E-48	8.53E-13	7.14E-55	7.86E-24	1.37E-10	5.79E-19	1.67E-14	2.88E-27
0.459014	0.635694	0.513448	0.61718	0.652842	-0.44943	-0.35022	-0.66089	0.618011
5.71E-08	9.83E-16	6.71E-10	1.1E-14	9.03E-17	1.16E-07	5.43E-05	2.79E-17	9.9E-15
-0.11353	-0.02667	0.236852	-0.05078	-0.12156	-0.01922	0.058899	0.017257	-0.12316
0.203754	0.765986	0.007339	0.570772	0.173387	0.830203	0.51069	0.847299	0.167758
0.176722	0.328923	0.473391	0.267482	0.318983	-0.27656	-0.00426	-0.47135	0.232576
0.046862	0.00016	1.9E-08	0.002365	0.000257	0.001647	0.962098	2.23E-08	0.008507
-0.06171	0.031041	0.068046	0.003321	0.018523	-0.26015	-0.03456	-0.26314	0.17611
0.49071	0.729016	0.447171	0.97044	0.836248	0.003138	0.699668	0.002799	0.047642
-0.20076	-0.20234	0.232071	-0.23189	0.061859	0.336217	0.259127	0.071722	-0.31454
0.023626	0.022529	0.008655	0.008707	0.48964	0.000111	0.003264	0.422952	0.000316
0.530832	0.376994	-0.09698	0.318871	0.071515	-0.51954	-0.38422	-0.57467	0.344257
1.37E-10	1.25E-05	0.278096	0.000258	0.4243	3.88E-10	8.22E-06	1.61E-12	7.4E-05
0.10725	-0.28189	-0.2242	-0.36535	-0.38752	0.030923	0.241734	-0.03499	-0.37215
0.230079	0.001324	0.011278	2.41E-05	6.76E-06	0.730006	0.006182	0.696111	1.65E-05
0.078831	-0.09013	-0.00822	-0.15926	-0.12376	-0.0156	0.218368	-0.07635	-0.22196
0.378332	0.313612	0.926927	0.07371	0.16568	0.861801	0.013648	0.393555	0.012141
0.030984	-0.00937	-0.30887	0.026924	-0.0953	-0.10789	-0.20733	0.009059	0.326224
0.72949	0.916702	0.000411	0.76382	0.286525	0.227271	0.01934	0.919481	0.000182
0.392896	0.314051	-0.11541	0.373457	0.232704	-0.19281	-0.53316	-0.10306	0.396447
4.9E-06	0.000324	0.196325	1.53E-05	0.00847	0.029868	1.1E-10	0.248914	3.95E-06
0.10941	-0.01274	-0.14976	0.02384	-0.09817	0.093218	-0.26484	0.211524	-0.07935
0.220773	0.886936	0.092847	0.790206	0.272181	0.297228	0.002621	0.016974	0.375201
0.227182	-0.15702	-0.37362	-0.16987	-0.26356	0.070291	-0.09878	0.121017	-0.17992
0.010212	0.077894	1.51E-05	0.056222	0.002754	0.432293	0.269188	0.175325	0.042964
-0.50073	-0.67781	-0.29377	-0.66472	-0.44226	0.489206	0.595607	0.631935	-0.61542
2.03E-09	2.1E-18	0.000802	1.58E-17	1.93E-07	5.34E-09	1.51E-13	1.63E-15	1.37E-14
-0.06087	0.073009	0.084547	0.181474	0.150633	0.134795	-0.24697	0.31195	0.158918
0.496612	0.414659	0.344623	0.041163	0.090947	0.130801	0.005123	0.000357	0.074332
-0.32358	0.015893	0.239357	0.053971	0.21741	0.083202	0.188218	0.056943	0.050598
0.000207	0.859239	0.006723	0.546746	0.014077	0.352382	0.034081	0.524848	0.572119
-0.56015	-0.36532	-0.18132	-0.3047	-0.23739	0.344859	0.269215	0.475968	-0.28061
7.53E-12	2.41E-05	0.041339	0.000496	0.007202	7.17E-05	0.002209	1.55E-08	0.001396
0.43375	0.411457	0.178161	0.468367	0.25932	-0.49762	-0.57125	-0.26786	0.540611
3.5E-07	1.54E-06	0.045072	2.81E-08	0.00324	2.65E-09	2.33E-12	0.00233	5.36E-11

SLC25A28	SLC39A6	SLC6A20	SLC7A2	SLC7A8	SMAD7	SMAP2	SMARCC1	SMC1A
blue	blue	blue	blue	blue	blue	blue	blue	blue
0.469283	-0.26154	0.251427	0.153301	0.485438	0.322974	0.250317	-0.27533	-0.44033
2.62E-08	0.002977	0.004353	0.085302	7.27E-09	0.000213	0.004535	0.001731	2.21E-07
0.51409	-0.2671	0.239098	0.191162	0.432164	0.222704	0.253225	-0.30757	-0.51015
6.34E-10	0.0024	0.006784	0.031328	3.91E-07	0.011848	0.004073	0.000436	8.98E-10
-0.61456	0.755783	-0.46062	-0.53015	-0.70913	-0.54554	-0.6799	0.756068	0.708933
1.53E-14	9.71E-25	5.06E-08	1.46E-10	1.07E-20	3.3E-11	1.5E-18	9.11E-25	1.11E-20
-0.55858	0.578124	-0.34925	-0.33283	-0.60218	-0.5322	-0.67558	0.536637	0.547147
8.85E-12	1.1E-12	5.71E-05	0.000132	6.95E-14	1.2E-10	2.98E-18	7.86E-11	2.82E-11
0.339631	-0.27578	0.041135	-0.11645	-0.1277	-0.04933	-0.07851	-0.25619	-0.49273
9.37E-05	0.0017	0.646109	0.192307	0.152513	0.581789	0.380306	0.003646	3.99E-09
-0.14554	0.142582	-0.16798	-0.19636	-0.38243	-0.35886	-0.47642	0.076168	-0.00822
0.102541	0.109795	0.059062	0.026924	9.12E-06	3.43E-05	1.5E-08	0.394704	0.926896
0.006074	0.046643	-0.04565	-0.08158	-0.26832	-0.20685	-0.17209	0.074981	-0.12582
0.945967	0.602554	0.610313	0.3619	0.002288	0.019632	0.053039	0.402134	0.158688
0.073476	-0.29946	-0.09326	0.177096	-0.01687	-0.10317	-0.10774	-0.20336	0.085251
0.411672	0.000626	0.297009	0.046392	0.850674	0.24841	0.227941	0.021841	0.340606
-0.43766	0.310387	-0.20565	-0.03597	-0.00849	-0.14789	-0.1605	0.456104	0.392371
2.67E-07	0.000383	0.020367	0.688091	0.924549	0.097063	0.071461	7.09E-08	5.06E-06
0.252389	-0.35753	-0.03969	0.246245	0.464974	0.257348	0.22551	-0.11763	-0.1591
0.004201	3.68E-05	0.657755	0.005259	3.64E-08	0.003491	0.010798	0.187825	0.073997
0.125927	-0.25769	-0.13154	0.136251	0.245366	0.048041	-0.06994	-0.03534	0.015838
0.158327	0.003447	0.140453	0.126656	0.005429	0.591712	0.434575	0.693231	0.859719
-0.1112	0.109681	0.03243	0.068616	0.076384	-0.01262	0.142901	0.253232	0.08906
0.213273	0.219622	0.717392	0.443367	0.393358	0.887979	0.108994	0.004072	0.319389
-0.33655	0.454179	-0.25464	-0.204	-0.14392	-0.06428	-0.12285	0.627593	0.617983
0.000109	8.17E-08	0.003864	0.021422	0.106475	0.472759	0.168821	2.88E-15	9.94E-15
0.089525	-0.13465	-0.15134	-0.097	0.114723	0.186929	0.022194	0.065074	0.033857
0.316857	0.131225	0.089426	0.277976	0.199028	0.035349	0.804389	0.46731	0.705517
0.088254	-0.12026	-0.1524	0.091553	0.39186	0.311753	0.206726	0.202209	0.180703
0.323806	0.178048	0.087168	0.305979	5.22E-06	0.00036	0.019706	0.022616	0.042047
0.608816	-0.4041	0.317271	0.333333	0.483986	0.417855	0.564627	-0.42612	-0.46076
3.11E-14	2.46E-06	0.000279	0.000128	8.18E-09	1.02E-06	4.71E-12	5.89E-07	5.01E-08
-0.08246	0.185344	0.048995	-0.12732	-0.209	-0.01564	0.070528	0.104428	0.129121
0.356695	0.036963	0.58437	0.153748	0.01837	0.86148	0.430732	0.242641	0.147952
-0.10104	0.085376	0.230557	0.004744	-0.20165	-0.14527	-0.02933	-0.31439	-0.16008
0.258336	0.339897	0.009113	0.957789	0.023003	0.103198	0.743399	0.000319	0.072216
0.245732	-0.25198	0.404884	0.22774	0.152648	0.165223	0.42631	-0.5202	-0.41164
0.005358	0.004264	2.34E-06	0.010023	0.086657	0.063405	5.81E-07	3.65E-10	1.52E-06
-0.1795	0.634042	0.022851	-0.48139	-0.4602	-0.16029	-0.10603	0.633232	0.237274
0.043455	1.23E-15	0.798718	1.01E-08	5.23E-08	0.071832	0.235434	1.37E-15	0.007232

SMC2	SMC3	SMC4	SNCAIP	SNRNP20(	SOX11	SOX2	SOX4	SOX5
blue	blue	blue	blue	blue	blue	blue	blue	blue
-0.32959	-0.12436	-0.39982	-0.25453	-0.32288	-0.37505	-0.43787	-0.29307	-0.28402
0.000154	0.163599	3.21E-06	0.00388	0.000214	1.4E-05	2.63E-07	0.000827	0.001212
-0.33812	-0.18113	-0.46987	-0.22433	-0.32639	-0.3106	-0.44444	-0.27302	-0.27204
0.000101	0.04155	2.5E-08	0.011229	0.00018	0.000379	1.65E-07	0.001899	0.001975
0.811553	0.552422	0.844727	0.520407	0.721355	0.7923	0.599627	0.69881	0.501993
5.88E-31	1.66E-11	9.74E-36	3.59E-10	1.12E-21	1.35E-28	9.42E-14	6.55E-20	1.83E-09
0.65227	0.443346	0.702839	0.410158	0.540465	0.349138	0.388106	0.262348	0.345464
9.8E-17	1.79E-07	3.26E-20	1.68E-06	5.43E-11	5.75E-05	6.53E-06	0.002886	6.95E-05
-0.11235	-0.21747	-0.18113	-0.10303	-0.43478	-0.09773	-0.42355	-0.15033	-0.33519
0.208556	0.014049	0.041553	0.249023	3.26E-07	0.27435	6.99E-07	0.091613	0.000117
0.337358	0.147592	0.301238	0.136524	-0.02001	-0.0167	-0.09875	-0.09444	-0.03582
0.000105	0.097739	0.000578	0.125891	0.82332	0.852183	0.269346	0.290937	0.689337
0.03986	-0.07722	0.034177	0.006324	0.031716	-0.15821	-0.26192	-0.29709	-0.05043
0.656365	0.388166	0.70286	0.943742	0.72336	0.075655	0.002933	0.000694	0.573396
-0.02701	0.035543	-0.09449	0.229062	-0.01789	-0.35007	-0.02426	-0.41271	-0.09378
0.763118	0.691575	0.290653	0.009586	0.84176	5.47E-05	0.786585	1.42E-06	0.294296
0.18251	0.158799	0.567616	0.031311	0.398674	0.14679	0.120709	0.153988	-0.08611
0.040002	0.074552	3.44E-12	0.726745	3.44E-06	0.099595	0.176434	0.083895	0.335737
-0.31448	-0.08736	-0.08332	-0.18489	-0.21152	-0.40213	-0.2862	-0.32341	-0.5122
0.000317	0.328731	0.351683	0.037434	0.016976	2.78E-06	0.001106	0.000208	7.5E-10
-0.08041	-0.03031	0.100069	0.004834	-0.0309	-0.31187	-0.10405	-0.25734	-0.32141
0.368811	0.735146	0.262979	0.956983	0.730215	0.000358	0.244371	0.003492	0.000229
-0.2546	-0.18281	0.152448	-0.01776	0.273211	0.047075	0.076032	0.129792	-0.1358
0.00387	0.039675	0.087076	0.842878	0.001885	0.599195	0.395551	0.145841	0.12794
0.212115	0.325163	0.431997	0.354512	0.699197	0.5251	0.543008	0.530148	0.276819
0.016662	0.000191	3.95E-07	4.33E-05	6.13E-20	2.33E-10	4.24E-11	1.46E-10	0.00163
-0.20739	-0.13159	0.019094	0.082279	0.049394	0.257685	0.229261	0.293546	-0.22675
0.019305	0.140279	0.83127	0.357764	0.581308	0.003447	0.009522	0.00081	0.010359
-0.28275	0.029276	0.072713	0.04429	0.180982	0.027035	0.15697	0.116809	-0.33454
0.001278	0.743873	0.416555	0.620998	0.041725	0.762876	0.077999	0.19093	0.000121
-0.46993	-0.21169	-0.69289	-0.28707	-0.51631	-0.35366	-0.28122	-0.2828	-0.31905
2.49E-08	0.016887	1.79E-19	0.001067	5.19E-10	4.53E-05	0.001361	0.001275	0.000256
0.110733	0.025936	-0.07761	0.112628	0.150621	0.440897	0.328489	0.405736	0.39188
0.215205	0.772242	0.385765	0.20741	0.090974	2.13E-07	0.000163	2.22E-06	5.21E-06
0.191063	-0.09393	-0.18292	-0.1477	-0.24532	-0.05375	-0.0151	-0.0812	0.404684
0.031418	0.293558	0.039554	0.097496	0.005438	0.548368	0.866196	0.36412	2.37E-06
-0.23861	-0.32987	-0.58931	-0.34035	-0.41813	-0.2061	-0.25537	-0.20295	0.210542
0.006903	0.000152	3.13E-13	9.03E-05	1E-06	0.02009	0.003759	0.022112	0.017505
0.330616	0.34206	0.352849	0.048445	0.357218	0.602007	0.081064	0.500488	0.360122
0.000147	8.28E-05	4.73E-05	0.588602	3.74E-05	7.09E-14	0.364936	2.08E-09	3.2E-05

SOX6	SOX9	SPAG5	SPATA20	SPATA5L1	SPC24	SPC25	SPDL1	SPEF2
blue	blue	blue	blue	blue	blue	blue	blue	blue
-0.40956	-0.25558	-0.35132	0.183578	-0.28218	-0.52675	-0.51017	-0.35573	0.33672
1.74E-06	0.003731	5.12E-05	0.038833	0.001308	2E-10	8.97E-10	4.05E-05	0.000108
-0.40118	-0.24809	-0.29437	0.182641	-0.2626	-0.52175	-0.48375	-0.37024	0.297806
2.95E-06	0.00492	0.000781	0.039857	0.002858	3.17E-10	8.34E-09	1.83E-05	0.000673
0.501528	0.43489	0.848172	-0.49372	0.659299	0.839331	0.910155	0.867353	-0.58918
1.9E-09	3.24E-07	2.68E-36	3.68E-09	3.53E-17	6.91E-35	1.13E-49	1.08E-39	3.18E-13
0.278819	0.344673	0.477355	-0.29134	0.559336	0.488119	0.598823	0.790987	-0.24221
0.001502	7.24E-05	1.39E-08	0.00089	8.19E-12	5.84E-09	1.04E-13	1.91E-28	0.006077
-0.4341	-0.44576	-0.12279	0.135867	0.131596	-0.01213	-0.00505	0.089524	-0.10753
3.42E-07	1.51E-07	0.169021	0.12774	0.140275	0.892334	0.955099	0.316867	0.228876
-0.20281	-0.08943	0.087948	-0.05524	0.379221	0.188729	0.286315	0.5635	-0.06326
0.02221	0.317366	0.325496	0.537325	1.1E-05	0.033588	0.001101	5.31E-12	0.479847
-0.11276	-0.13161	0.032771	0.100168	0.143411	-0.08914	-0.02319	0.240874	0.020256
0.206887	0.140227	0.714549	0.2625	0.107723	0.318942	0.795756	0.006373	0.82117
-0.08054	-0.05298	-0.31062	0.162006	-0.19558	-0.2816	-0.23441	-0.00298	0.479545
0.368081	0.554171	0.000379	0.068804	0.027553	0.00134	0.007987	0.973493	1.17E-08
-0.0443	0.091268	0.230526	0.009258	0.200811	0.338893	0.326882	0.293541	-0.17059
0.62092	0.307492	0.009123	0.917722	0.023589	9.73E-05	0.000176	0.00081	0.055171
-0.43705	-0.2673	-0.49299	0.313942	-0.20488	-0.27291	-0.30176	-0.21743	0.176742
2.79E-07	0.002382	3.91E-09	0.000325	0.020856	0.001907	0.000565	0.014067	0.046837
-0.29922	-0.01877	-0.32488	0.216608	-0.17733	-0.15008	-0.1531	-0.06429	0.187728
0.000632	0.83409	0.000194	0.014444	0.046103	0.092162	0.085709	0.47269	0.034558
0.124221	0.079877	0.148841	0.155457	0.032795	-0.11119	-0.06479	-0.22455	-0.08795
0.164089	0.372023	0.094902	0.080947	0.714348	0.213317	0.469239	0.011149	0.325469
0.514851	0.451607	0.475477	-0.20433	0.201755	0.386426	0.359956	0.13847	-0.33754
5.92E-10	9.87E-08	1.61E-08	0.021205	0.022928	7.21E-06	3.23E-05	0.120534	0.000104
-0.01402	0.093864	0.002615	0.064134	-0.01522	0.26435	0.101728	-0.22401	-0.34326
0.875699	0.293875	0.976723	0.473779	0.865158	0.002671	0.255107	0.011349	7.79E-05
-0.02108	0.083868	-0.17181	0.170195	-0.16871	0.000158	-0.09984	-0.29774	-0.0916
0.814012	0.348529	0.053423	0.055746	0.057947	0.998593	0.264068	0.000675	0.30574
-0.06076	-0.24945	-0.49159	0.080256	-0.55313	-0.59672	-0.62339	-0.61409	0.26597
0.497422	0.004681	4.39E-09	0.36975	1.55E-11	1.33E-13	4.98E-15	1.62E-14	0.002508
0.561973	0.251295	0.340239	-0.32632	-0.01244	0.150714	0.13894	-0.07662	-0.27851
6.23E-12	0.004374	9.09E-05	0.000181	0.889565	0.090771	0.119265	0.391864	0.001521
0.11796	0.02254	0.060635	-0.20442	-0.02846	-0.05241	-0.01428	0.06851	0.148161
0.186564	0.801401	0.498288	0.021152	0.750733	0.558421	0.873409	0.444075	0.09644
0.01386	-0.16381	-0.14507	0.008735	-0.3594	-0.35346	-0.37116	-0.4388	0.265404
0.877088	0.065732	0.103663	0.922359	3.33E-05	4.58E-05	1.74E-05	2.47E-07	0.002564
0.426731	-0.08149	0.666648	-0.49453	0.643106	0.364625	0.450171	0.48474	-0.51868
5.65E-07	0.362403	1.18E-17	3.43E-09	3.57E-16	2.5E-05	1.1E-07	7.7E-09	4.19E-10

SPIN4	SPRY3	SPRY4	SPSB4	SRP72	SRPK1	SRR	SRSF1	SRSF10
blue	blue	blue	blue	blue	blue	blue	blue	blue
-0.2921	-0.21809	-0.28867	-0.52	-0.12188	-0.14412	-0.37354	-0.38773	-0.29471
0.000862	0.013771	0.000997	3.72E-10	0.17226	0.105974	1.52E-05	6.68E-06	0.00077
-0.37377	-0.18122	-0.27963	-0.5064	-0.10934	-0.05569	-0.27395	-0.41331	-0.35534
1.5E-05	0.041455	0.001453	1.25E-09	0.22107	0.534019	0.00183	1.37E-06	4.14E-05
0.607098	0.542936	0.650993	0.738643	0.565786	0.557564	0.66916	0.777664	0.751226
3.84E-14	4.27E-11	1.18E-16	3.73E-23	4.17E-12	9.83E-12	8.04E-18	5.8E-27	2.64E-24
0.6142	0.480777	0.547007	0.381021	0.622356	0.513306	0.492744	0.639795	0.784772
1.6E-14	1.06E-08	2.86E-11	9.9E-06	5.69E-15	6.79E-10	3.99E-09	5.63E-16	9.68E-28
-0.07899	-0.16082	-0.2062	-0.18712	0.161266	0.030203	-0.00119	-0.38164	-0.22254
0.377368	0.07088	0.020026	0.035155	0.070098	0.736051	0.989411	9.55E-06	0.011911
0.386217	0.184647	0.187423	-0.03715	0.539501	0.369977	0.229823	0.111841	0.418674
7.3E-06	0.037691	0.034858	0.678389	5.97E-11	1.86E-05	0.009343	0.210622	9.66E-07
0.054978	0.117033	0.193998	-0.09158	0.262535	0.174756	0.061187	-0.03946	0.11808
0.539273	0.190072	0.028857	0.305854	0.002865	0.049405	0.494379	0.65956	0.186114
-0.01417	-0.08965	-0.36782	-0.25773	-0.11158	-0.17427	-0.18873	-0.11548	0.098969
0.874378	0.316156	2.1E-05	0.00344	0.211715	0.050057	0.033592	0.196065	0.268288
0.164104	0.005706	0.609466	0.091042	0.166193	-0.04158	0.00259	0.375708	0.322688
0.065242	0.949233	2.87E-14	0.308694	0.061848	0.642538	0.976941	1.34E-05	0.000216
-0.26701	-0.43027	-0.18737	-0.42311	-0.18481	-0.48767	-0.48234	-0.2242	-0.12817
0.002409	4.44E-07	0.034913	7.2E-07	0.03752	6.06E-09	9.35E-09	0.011278	0.150976
-0.06837	-0.15375	-0.10172	-0.32704	0.004863	-0.19687	-0.21116	-0.04939	0.068759
0.44498	0.084381	0.25514	0.000175	0.956728	0.026528	0.017168	0.581365	0.442413
-0.31766	0.051594	0.205101	0.051742	-0.08782	-0.07215	0.319222	0.24752	-0.31145
0.000274	0.564564	0.020714	0.563453	0.326214	0.420165	0.000254	0.005022	0.000365
0.048247	0.187263	0.370595	0.51902	-0.1418	-0.07763	0.222778	0.556995	0.228566
0.590125	0.035017	1.8E-05	4.07E-10	0.111768	0.385661	0.01182	1.04E-11	0.009748
-0.17659	-0.31664	-0.03025	0.210934	-0.40413	-0.42652	-0.04866	-0.0337	-0.23596
0.047035	0.000287	0.735639	0.017291	2.46E-06	5.73E-07	0.586952	0.70685	0.00757
-0.30271	-0.36474	-0.01615	-0.01418	-0.49366	-0.62505	-0.32971	0.053002	-0.15442
0.000542	2.49E-05	0.857015	0.874264	3.69E-09	4.02E-15	0.000154	0.553974	0.083024
-0.5427	-0.33632	-0.58385	-0.35187	-0.52754	-0.40078	-0.43821	-0.43843	-0.53557
4.37E-11	0.000111	5.83E-13	4.98E-05	1.86E-10	3.02E-06	2.57E-07	2.53E-07	8.71E-11
-0.03519	0.206177	0.004357	0.493042	-0.09323	0.11403	0.298121	0.147594	-0.10709
0.694468	0.020041	0.961229	3.89E-09	0.297178	0.201774	0.000664	0.097734	0.230781
0.29414	0.313305	-0.05169	0.032779	0.313307	0.541132	0.221397	-0.06913	0.095997
0.000789	0.000335	0.56387	0.71448	0.000335	5.09E-11	0.012368	0.43998	0.282994
-0.22271	0.000367	-0.35353	-0.14178	-0.14858	0.126794	-0.07832	-0.41838	-0.44905
0.011847	0.99673	4.56E-05	0.111842	0.095495	0.155459	0.381437	9.85E-07	1.19E-07
0.253523	0.463201	0.427384	0.522882	0.260223	0.327614	0.301681	0.398667	0.246275
0.004028	4.17E-08	5.41E-07	2.86E-10	0.00313	0.00017	0.000567	3.45E-06	0.005254

SRSF2	SRSF3	SSRP1	SSUH2	ST3GAL5	ST8SIA2	STIL	STK17A	STPG1
blue	blue	blue	blue	blue	blue	blue	blue	blue
-0.30566	-0.27389	-0.37129	-0.20335	0.319328	-0.17964	-0.3285	-0.20229	0.362405
0.000475	0.001834	1.73E-05	0.021847	0.000253	0.043292	0.000163	0.022558	2.83E-05
-0.34667	-0.25786	-0.38229	-0.1802	0.393371	-0.16611	-0.31766	-0.19884	0.390576
6.53E-05	0.003424	9.2E-06	0.042629	4.76E-06	0.061982	0.000274	0.025023	5.63E-06
0.580487	0.744522	0.697855	0.555167	-0.51205	0.492859	0.921605	0.609055	-0.64548
8.48E-13	1.1E-23	7.72E-20	1.26E-11	7.59E-10	3.95E-09	3.23E-53	3.02E-14	2.56E-16
0.424637	0.726442	0.3443	0.458147	-0.39591	0.137787	0.800505	0.734786	-0.49761
6.5E-07	4.23E-22	7.38E-05	6.09E-08	4.08E-06	0.122394	1.43E-29	8.15E-23	2.65E-09
-0.22342	0.307266	-0.31029	-0.09795	0.300412	-0.13113	-0.05697	0.22753	0.167496
0.011571	0.000442	0.000385	0.273266	0.0006	0.141702	0.524654	0.010093	0.059803
0.054014	0.675819	-0.13918	0.174178	-0.00624	-0.12786	0.461247	0.674159	-0.14496
0.546421	2.87E-18	0.118621	0.050174	0.944519	0.15198	4.83E-08	3.72E-18	0.103935
-0.10901	0.293169	-0.07893	0.123315	0.229706	-0.24881	0.195402	0.436947	0.001585
0.222467	0.000823	0.377758	0.16721	0.00938	0.004791	0.027696	2.81E-07	0.98589
-0.13079	0.003812	-0.15586	-0.27222	0.00315	-0.38845	-0.08221	-0.15128	0.376312
0.142747	0.966072	0.080161	0.001961	0.971962	6.4E-06	0.358154	0.089554	1.3E-05
0.356	0.188273	0.436131	0.245781	-0.28185	-0.0834	0.400987	0.362612	-0.30605
4E-05	0.034028	2.97E-07	0.005348	0.001326	0.351227	2.99E-06	2.8E-05	0.000467
-0.05211	-0.15353	-0.2203	-0.20673	0.226001	-0.485	-0.22559	0.004975	0.210329
0.560691	0.084835	0.012818	0.019704	0.010623	7.54E-09	0.010769	0.95573	0.017622
0.015305	0.016887	-0.15191	-0.04108	0.025172	-0.28139	-0.01008	0.144611	0.054978
0.864394	0.850536	0.088199	0.646565	0.778779	0.001352	0.910407	0.104779	0.539278
0.217095	-0.25368	0.398541	0.169095	-0.11342	0.274033	-0.06304	-0.06433	-0.14178
0.01422	0.004004	3.47E-06	0.057371	0.204228	0.001824	0.481345	0.472419	0.111828
0.471852	-0.11386	0.736682	0.343388	-0.41212	0.352313	0.288781	-0.09447	-0.38799
2.14E-08	0.202434	5.56E-23	7.74E-05	1.48E-06	4.86E-05	0.000992	0.290769	6.58E-06
0.163479	-0.24739	0.253916	-0.00764	-0.17252	0.078658	-0.17825	-0.28856	-0.08394
0.066288	0.005045	0.00397	0.932064	0.052435	0.379384	0.044964	0.001002	0.348085
0.201577	-0.43176	0.275126	-0.02695	-0.07548	-0.14195	-0.20491	-0.31041	-0.0002
0.023052	4.02E-07	0.001745	0.763638	0.399014	0.111405	0.020838	0.000383	0.998209
-0.32553	-0.6009	-0.44559	-0.38105	0.532023	-0.21726	-0.68964	-0.65636	0.551504
0.000188	8.1E-14	1.52E-07	9.88E-06	1.22E-10	0.014144	3.08E-19	5.43E-17	1.82E-11
0.06982	-0.10374	0.248858	0.073492	-0.0978	0.473622	-0.04834	-0.26557	-0.12583
0.435389	0.245798	0.004783	0.411567	0.274005	1.87E-08	0.589426	0.002548	0.158655
-0.20558	0.176793	-0.32893	-0.09152	0.00594	0.173905	0.014709	0.04452	0.018123
0.020413	0.046773	0.000159	0.306129	0.947158	0.050541	0.869631	0.61919	0.839735
-0.41848	-0.33951	-0.36439	-0.33113	0.242815	0.104812	-0.47003	-0.4557	0.316655
9.78E-07	9.43E-05	2.54E-05	0.000143	0.005948	0.240904	2.47E-08	7.3E-08	0.000287
0.31144	0.322855	0.480376	0.418267	-0.0138	0.538083	0.448715	0.265037	-0.38121
0.000365	0.000214	1.09E-08	9.92E-07	0.877652	6.84E-11	1.22E-07	0.002601	9.79E-06

STX3	STX6	SULT1A1	SUMO2	SUPT16H	SUV39H1	SUV39H2	SUZ12	SYCE2
blue	blue	blue	blue	blue	blue	blue	blue	blue
0.554544	-0.3913	0.405584	-0.28802	-0.2178	-0.32923	-0.2512	-0.35312	-0.19377
1.34E-11	5.39E-06	2.24E-06	0.001025	0.0139	0.000157	0.00439	4.66E-05	0.029046
0.516828	-0.31731	0.454624	-0.32505	-0.19865	-0.20665	-0.17966	-0.40436	-0.20691
4.96E-10	0.000278	7.91E-08	0.000192	0.025166	0.019749	0.043271	2.42E-06	0.019596
-0.63594	0.509776	-0.68308	0.549892	0.754364	0.626317	0.763135	0.805585	0.571733
9.51E-16	9.28E-10	9.02E-19	2.14E-11	1.33E-24	3.41E-15	1.85E-25	3.38E-30	2.21E-12
-0.28225	0.206796	-0.64	0.182413	0.596331	0.221988	0.443044	0.808445	0.289953
0.001304	0.019664	5.47E-16	0.040109	1.39E-13	0.012131	1.83E-07	1.47E-30	0.000944
0.234608	-0.21719	0.407944	0.325355	-0.31818	0.132537	0.092768	-0.17422	0.007388
0.007933	0.014178	1.93E-06	0.00019	0.000267	0.137436	0.299578	0.05012	0.934295
0.126593	-0.10921	-0.13477	0.156872	0.128768	0.045935	0.235605	0.439582	0.022818
0.156121	0.221612	0.130887	0.078189	0.149076	0.60808	0.007664	2.33E-07	0.799006
0.189863	-0.24607	0.001433	0.003437	0.063143	0.003419	0.0104	0.162071	0.035858
0.032518	0.005292	0.987242	0.969408	0.480652	0.969571	0.907612	0.068692	0.688984
0.281544	-0.37443	0.254224	-0.16875	-0.28618	-0.25619	-0.32832	-0.0471	-0.22112
0.001343	1.45E-05	0.003924	0.057891	0.001108	0.003646	0.000164	0.599008	0.012481
-0.01514	0.064016	-0.42365	0.117625	0.25993	-0.09377	0.006861	0.397297	0.278404
0.865852	0.474593	6.95E-07	0.187825	0.003165	0.294343	0.938978	3.75E-06	0.001528
0.500195	-0.35378	0.238066	-0.21471	-0.38763	-0.45775	-0.45709	-0.18155	-0.13531
2.13E-09	4.5E-05	0.007035	0.015349	6.71E-06	6.27E-08	6.59E-08	0.041076	0.129323
0.358948	-0.24634	-0.01433	-0.21157	-0.15158	-0.32961	-0.34046	0.023445	-0.14823
3.41E-05	0.005241	0.872981	0.016947	0.088914	0.000154	8.98E-05	0.793601	0.096291
-0.16728	0.120747	-0.38155	-0.1624	0.169064	0.055278	-0.08314	-0.23998	0.198916
0.060133	0.176295	9.6E-06	0.068125	0.057418	0.537063	0.35276	0.006577	0.024964
-0.49686	0.406912	-0.47392	0.183558	0.4814	0.323966	0.232564	0.226442	0.505884
2.82E-09	2.06E-06	1.82E-08	0.038855	1.01E-08	0.000203	0.00851	0.010468	1.3E-09
-0.23672	0.186076	0.106807	0.348659	-0.20159	0.315621	-0.03084	-0.21519	0.280955
0.007373	0.03621	0.232017	5.89E-05	0.023044	0.000301	0.730737	0.015113	0.001376
0.034168	0.044527	0.02081	-0.02846	-0.1649	-0.10839	-0.31454	-0.21513	0.187066
0.702936	0.619136	0.816363	0.750739	0.063929	0.225112	0.000316	0.015146	0.035212
0.268089	-0.10023	0.556947	-0.39769	-0.49277	-0.22235	-0.40651	-0.64445	-0.42692
0.002309	0.262198	1.05E-11	3.66E-06	3.98E-09	0.011986	2.11E-06	2.96E-16	5.58E-07
-0.5447	0.387436	-0.11791	0.157427	0.20166	0.400387	0.287658	-0.08475	0.089208
3.59E-11	6.79E-06	0.186758	0.077126	0.022994	3.1E-06	0.001041	0.343476	0.318583
-0.11322	0.050708	0.000988	-0.07624	0.091136	0.04304	0.204913	0.135395	-0.32028
0.205009	0.571283	0.991201	0.394242	0.308195	0.630896	0.020833	0.129081	0.000242
0.023807	-0.09134	0.350904	-0.19139	-0.21243	-0.0816	-0.08414	-0.42184	-0.3677
0.790489	0.307134	5.24E-05	0.031127	0.016499	0.361755	0.346976	7.84E-07	2.11E-05
-0.463	0.40964	-0.32638	0.458311	0.52905	0.438603	0.708926	0.289235	0.450925
4.23E-08	1.73E-06	0.000181	6.02E-08	1.61E-10	2.5E-07	1.11E-20	0.000974	1.04E-07

SYNE2	SYNPO	SYNPO2	TACC1	TACC3	TAF4	TAF5	TAF5L	TANGO6
blue	blue	blue	blue	blue	blue	blue	blue	blue
-0.29656	0.323349	0.320188	0.300494	-0.44307	-0.44837	-0.13667	-0.41042	-0.27764
0.00071	0.000209	0.000243	0.000598	1.82E-07	1.25E-07	0.125477	1.65E-06	0.001576
-0.40487	0.276144	0.302477	0.241343	-0.47617	-0.44947	-0.05536	-0.39382	-0.2566
2.34E-06	0.001675	0.000548	0.006268	1.53E-08	1.15E-07	0.536465	4.63E-06	0.00359
0.590783	-0.53566	-0.62328	-0.59796	0.745819	0.683629	0.510989	0.698092	0.539973
2.65E-13	8.63E-11	5.05E-15	1.15E-13	8.39E-24	8.25E-19	8.34E-10	7.41E-20	5.7E-11
0.515773	-0.48895	-0.36782	-0.24423	0.366879	0.546503	0.376406	0.622615	0.337034
5.45E-10	5.46E-09	2.1E-05	0.005656	2.21E-05	3E-11	1.29E-05	5.5E-15	0.000107
-0.57271	-0.12185	-0.01233	-0.39516	-0.16396	-0.45894	0.044963	-0.14946	-0.19637
1.99E-12	0.172338	0.890541	4.27E-06	0.065477	5.74E-08	0.615704	0.093517	0.026917
-0.05846	-0.36624	-0.10587	-0.22165	0.00591	0.002855	0.231866	0.29566	0.020392
0.513881	2.29E-05	0.236169	0.012266	0.947423	0.974588	0.008716	0.000739	0.819986
-0.15523	-0.15526	0.028472	-0.10165	-0.17999	-0.15692	0.015608	0.177576	0.055673
0.081398	0.081337	0.750674	0.255476	0.042875	0.078088	0.86174	0.045793	0.534152
-0.02008	0.053537	0.361004	0.101156	-0.29312	-0.23369	-0.17931	-0.19426	-0.23744
0.822739	0.54998	3.05E-05	0.257801	0.000825	0.008188	0.043679	0.02864	0.007191
0.42865	0.034412	0.031445	-0.04766	0.480578	0.278555	0.039282	0.322708	0.15792
4.96E-07	0.700915	0.725623	0.594673	1.08E-08	0.001518	0.661036	0.000215	0.076192
-0.09351	0.149146	0.457497	0.232953	-0.01339	-0.33021	-0.26713	-0.19736	-0.24074
0.295705	0.09422	6.39E-08	0.008397	0.881251	0.00015	0.002397	0.026147	0.006402
0.152795	0.093261	0.267538	0.227807	-0.04782	-0.16779	-0.2352	-0.13843	-0.18747
0.08635	0.297003	0.002359	0.01	0.593406	0.059353	0.007773	0.120652	0.034815
0.293544	0.112631	-0.10459	0.101758	0.093902	0.209295	-0.0073	0.043526	0.13386
0.00081	0.2074	0.24191	0.254967	0.29368	0.018199	0.935059	0.627039	0.133519
0.638238	-0.10108	-0.32674	-0.21277	0.583199	0.605659	0.164905	0.285164	0.364604
6.96E-16	0.258139	0.000177	0.016322	6.26E-13	4.57E-14	0.063923	0.001156	2.51E-05
0.033052	0.014	-0.19183	-0.37357	0.33924	0.075642	-0.11148	-0.22456	-0.08637
0.712208	0.875861	0.030729	1.52E-05	9.56E-05	0.397986	0.212128	0.011144	0.334277
0.236979	0.126851	0.157044	0.044781	0.341916	0.092422	-0.20104	-0.16407	-0.07457
0.007307	0.155273	0.077857	0.617137	8.34E-05	0.30139	0.023428	0.065294	0.40473
-0.52689	0.219586	0.387869	0.406246	-0.4424	-0.41007	-0.22564	-0.47115	-0.30385
1.97E-10	0.01312	6.62E-06	2.15E-06	1.91E-07	1.69E-06	0.010753	2.26E-08	0.000515
0.048562	-0.04786	-0.3338	-0.14585	0.100674	0.165907	0.041942	0.04864	0.242534
0.587696	0.593081	0.000125	0.101818	0.260085	0.062303	0.639646	0.587096	0.006008
-0.1825	-0.0143	-0.10614	0.160918	-0.36865	-0.0271	0.073555	0.038974	0.017063
0.040012	0.873194	0.234952	0.070712	2E-05	0.762326	0.411167	0.663535	0.848997
-0.42033	0.341371	0.100994	0.249819	-0.50981	-0.3453	-0.17446	-0.31389	-0.10712
8.66E-07	8.58E-05	0.258569	0.004618	9.26E-10	7.01E-05	0.049799	0.000326	0.230642
0.105513	-0.32355	-0.43566	-0.39686	0.38368	0.390862	0.48409	0.498381	0.457489
0.237757	0.000207	3.07E-07	3.85E-06	8.48E-06	5.54E-06	8.11E-09	2.48E-09	6.4E-08

TBC1D31	TBCCD1	TBRG4	TCF12	TCF19	TCF4	TET1	TEX10	TFAM
blue	blue	blue	blue	blue	blue	blue	blue	blue
-0.22467	-0.26625	-0.28022	-0.34928	-0.35942	-0.3587	-0.32476	-0.1856	-0.1744
0.011104	0.002481	0.001418	5.7E-05	3.33E-05	3.46E-05	0.000195	0.036701	0.04988
-0.26966	-0.26643	-0.21879	-0.36254	-0.40041	-0.42541	-0.32167	-0.13004	-0.16975
0.00217	0.002463	0.013465	2.81E-05	3.1E-06	6.18E-07	0.000226	0.14507	0.056392
0.680198	0.705237	0.538034	0.704151	0.775152	0.702292	0.662435	0.718347	0.561972
1.43E-18	2.14E-20	6.87E-11	2.59E-20	1.07E-26	3.58E-20	2.22E-17	1.97E-21	6.23E-12
0.683188	0.713544	0.422976	0.673751	0.70644	0.535052	0.481483	0.557491	0.511345
8.86E-19	4.79E-21	7.27E-07	3.96E-18	1.73E-20	9.15E-11	1E-08	9.9E-12	8.08E-10
0.007894	0.01873	0.431234	-0.33925	-0.01583	-0.45982	-0.20323	0.262925	0.155053
0.92981	0.834442	4.16E-07	9.55E-05	0.859785	5.38E-08	0.021929	0.002822	0.081749
0.481665	0.48361	0.449324	0.232932	0.456784	-0.0105	0.090245	0.47698	0.427283
9.87E-09	8.44E-09	1.16E-07	0.008404	6.74E-08	0.906736	0.312969	1.43E-08	5.44E-07
0.189838	0.284312	0.422564	0.116895	0.137957	-0.1304	-0.13134	0.285277	0.121463
0.032542	0.001198	7.47E-07	0.190599	0.121927	0.143956	0.141045	0.00115	0.173726
0.038911	-0.0915	-0.13749	-0.13416	0.109497	-0.22107	-0.28772	-0.20964	-0.15916
0.664048	0.30624	0.123207	0.13263	0.220403	0.012499	0.001038	0.018004	0.073883
0.17219	0.305637	0.309008	0.286402	0.561059	0.180153	0.153106	0.147873	-0.14701
0.052895	0.000475	0.000408	0.001097	6.85E-12	0.042687	0.085705	0.097095	0.099084
-0.22467	-0.19978	0.006035	-0.14846	0.103471	-0.48093	-0.39988	-0.2933	-0.45423
0.011102	0.024329	0.946314	0.095754	0.247011	1.05E-08	3.2E-06	0.000818	8.14E-08
-0.00219	-0.00736	0.014924	0.080985	0.233499	-0.17647	-0.22721	-0.19444	-0.26014
0.980496	0.934554	0.867737	0.365401	0.008241	0.047187	0.010203	0.02849	0.00314
-0.24462	0.114407	0.001446	0.09535	-0.11031	0.053915	0.156987	-0.08939	-0.37228
0.005576	0.200277	0.987124	0.286268	0.216961	0.547158	0.077967	0.3176	1.63E-05
0.043586	0.119651	0.060127	0.371424	0.262878	0.479674	0.45026	0.028215	-0.12511
0.626569	0.180283	0.501901	1.71E-05	0.002828	1.16E-08	1.09E-07	0.75285	0.161077
-0.38779	-0.29897	0.210407	-0.22808	0.040693	-0.08568	0.090621	-0.26481	-0.25172
6.65E-06	0.000639	0.017579	0.00991	0.649659	0.338153	0.310945	0.002624	0.004307
-0.38402	-0.31702	-0.04978	-0.05531	0.097951	-0.14591	-0.06064	-0.4218	-0.57134
8.31E-06	0.000282	0.578379	0.536804	0.273263	0.101667	0.498216	7.85E-07	2.31E-12
-0.47698	-0.64883	-0.53432	-0.38498	-0.68599	-0.41434	-0.47622	-0.49612	-0.35886
1.43E-08	1.6E-16	9.82E-11	7.86E-06	5.62E-19	1.28E-06	1.52E-08	3.01E-09	3.43E-05
-0.07714	-0.06836	-0.16135	0.108187	-0.29965	0.366201	0.170228	0.058208	0.151099
0.388677	0.445096	0.069958	0.226009	0.00062	2.29E-05	0.055698	0.51567	0.089941
0.214251	0.116969	-0.23994	0.006136	-0.28348	0.264102	0.042692	0.167354	0.518959
0.015573	0.190317	0.006587	0.945416	0.00124	0.002697	0.633664	0.060023	4.09E-10
-0.266	-0.32892	-0.45294	-0.37484	-0.70428	-0.08681	-0.26588	-0.18626	0.049302
0.002506	0.00016	8.95E-08	1.41E-05	2.53E-20	0.33181	0.002517	0.036026	0.582014
0.414508	0.411698	0.194757	0.226287	0.06422	0.396344	0.487175	0.682492	0.465108
1.27E-06	1.52E-06	0.028224	0.010522	0.473185	3.97E-06	6.31E-09	9.92E-19	3.61E-08

TFAP2A	TFDP1	TFDP2	THAP2	TICRR	TIGD1	TIMELESS	TK1	TLE6
blue	blue	blue	blue	blue	blue	blue	blue	blue
-0.07	-0.42427	-0.22056	-0.23577	-0.44488	-0.133	-0.38909	-0.45105	-0.27866
0.434223	6.66E-07	0.012709	0.007619	1.6E-07	0.136051	6.16E-06	1.03E-07	0.001512
-0.01296	-0.48764	-0.28101	-0.2416	-0.41601	-0.05919	-0.4016	-0.49549	-0.19569
0.885057	6.08E-09	0.001373	0.006211	1.15E-06	0.508615	2.87E-06	3.17E-09	0.027459
0.443992	0.693163	0.561653	0.567157	0.90527	0.633472	0.86209	0.808097	0.609534
1.71E-07	1.71E-19	6.44E-12	3.61E-12	2.65E-48	1.32E-15	1.04E-38	1.63E-30	2.85E-14
0.171844	0.537349	0.473341	0.557158	0.581728	0.643074	0.510303	0.439402	0.423787
0.053381	7.34E-11	1.91E-08	1.02E-11	7.39E-13	3.58E-16	8.86E-10	2.36E-07	6.88E-07
-0.05256	-0.05338	-0.12053	0.141887	-0.22212	0.189009	-0.18379	0.023149	0.148541
0.557265	0.551167	0.177081	0.111557	0.012079	0.033321	0.038606	0.796153	0.095577
0.003368	0.267916	0.178116	0.434663	0.123842	0.496074	0.083776	0.163701	0.245065
0.970022	0.002325	0.045127	3.29E-07	0.165389	3.02E-09	0.34906	0.065914	0.005488
-0.07447	0.09588	0.050433	0.171666	-0.02187	0.35328	-0.07675	-0.03815	0.32351
0.405345	0.283586	0.573377	0.053631	0.8072	4.62E-05	0.391062	0.670254	0.000207
-0.15978	-0.06507	0.105621	-0.08544	-0.33024	-0.04581	-0.23814	-0.31903	-0.32327
0.072761	0.467328	0.237276	0.339524	0.00015	0.609032	0.007017	0.000256	0.00021
0.254796	0.235015	0.010674	-0.08531	0.413216	0.067804	0.41816	0.547344	0.279865
0.003842	0.007822	0.905191	0.340291	1.38E-06	0.448789	9.99E-07	2.76E-11	0.001439
-0.00537	-0.1868	-0.28869	-0.37815	-0.30708	-0.26972	-0.14727	-0.06239	-0.21794
0.952205	0.035478	0.000996	1.17E-05	0.000445	0.002165	0.098491	0.485919	0.013839
-0.0447	-0.02937	-0.05731	-0.21954	-0.15865	-0.07729	-0.03548	-0.05756	-0.22596
0.617737	0.743087	0.522149	0.013138	0.07482	0.387734	0.692079	0.520346	0.010637
0.030153	-0.09354	-0.01042	-0.28523	0.099771	0.091434	0.155096	0.109856	0.09316
0.736472	0.295558	0.907444	0.001153	0.264407	0.306609	0.081663	0.218886	0.297531
0.331681	0.225417	0.265922	-0.04062	0.566761	0.096577	0.616323	0.470255	0.291107
0.000139	0.010832	0.002513	0.650225	3.76E-12	0.280081	1.23E-14	2.43E-08	0.000899
0.16889	-0.06474	-0.15981	-0.14952	0.093249	-0.26434	0.239524	0.328399	0.078732
0.057679	0.469609	0.072703	0.093385	0.297065	0.002672	0.006684	0.000164	0.378935
0.20876	-0.14432	-0.18082	-0.43188	0.029642	-0.35457	0.212602	0.197852	-0.08814
0.018505	0.105477	0.041917	3.98E-07	0.740782	4.31E-05	0.016408	0.025766	0.324419
-0.14088	-0.55365	-0.41697	-0.40085	-0.59709	-0.4743	-0.49369	-0.64255	-0.47963
0.114134	1.47E-11	1.08E-06	3.01E-06	1.27E-13	1.77E-08	3.68E-09	3.85E-16	1.16E-08
0.136089	0.071124	0.131468	0.118784	0.207372	-0.00373	0.134687	0.021693	0.050595
0.127113	0.426839	0.140665	0.183485	0.019318	0.966813	0.131113	0.808723	0.572141
-0.27548	0.080591	0.089374	0.331606	-0.10877	0.053742	-0.29131	-0.32278	-0.12607
0.00172	0.367749	0.317679	0.00014	0.223481	0.548447	0.000891	0.000215	0.157857
-0.32516	-0.25715	-0.15535	-0.11491	-0.39513	-0.30105	-0.5114	-0.51572	-0.28743
0.000192	0.003517	0.081154	0.1983	4.28E-06	0.000583	8.05E-10	5.48E-10	0.001051
0.371676	0.293814	0.377093	0.333705	0.521172	0.4809	0.437958	0.448406	0.569745
1.69E-05	0.0008	1.24E-05	0.000126	3.34E-10	1.05E-08	2.62E-07	1.24E-07	2.74E-12

TM6SF2	TMA16	TMEM100	TMEM97	TMPO	TMSB15A	TNFAIP8L3	TNFSF9	TOP1
blue	blue	blue	blue	blue	blue	blue	blue	blue
-0.35099	-0.21933	-0.21301	-0.33736	-0.41402	-0.3016	0.316095	0.409843	-0.35642
5.21E-05	0.01323	0.016198	0.000105	1.31E-06	0.000569	0.000294	1.71E-06	3.91E-05
-0.40969	-0.21976	-0.13503	-0.20586	-0.40935	-0.27582	0.325018	0.450716	-0.48511
1.73E-06	0.013044	0.13014	0.020235	1.77E-06	0.001697	0.000193	1.05E-07	7.47E-09
0.65696	0.66256	0.478492	0.730262	0.834729	0.62633	-0.51868	-0.53172	0.576995
4.97E-17	2.18E-17	1.27E-08	2E-22	3.47E-34	3.4E-15	4.19E-10	1.26E-10	1.25E-12
0.497544	0.610315	0.462225	0.468417	0.595398	0.232877	-0.28452	-0.51261	0.429688
2.67E-09	2.59E-14	4.49E-08	2.8E-08	1.55E-13	0.008419	0.001187	7.23E-10	4.62E-07
-0.30096	0.274312	0.091388	0.111791	-0.02444	-0.01926	0.136063	0.006499	-0.3961
0.000586	0.001803	0.306854	0.210827	0.785035	0.829858	0.127185	0.94219	4.03E-06
0.056376	0.588554	0.348353	0.266956	0.302553	-0.0073	0.048914	-0.29159	-0.02686
0.528991	3.42E-13	5.99E-05	0.002414	0.000546	0.935116	0.584994	0.000881	0.764383
0.0485	0.106156	0.304799	0.141498	-0.0836	-0.1738	0.049706	-0.1107	-0.17261
0.588175	0.234892	0.000494	0.112552	0.350086	0.050681	0.578916	0.215361	0.052312
-0.16633	-0.22243	-0.41204	-0.23128	-0.18495	-0.34996	0.243441	-0.16747	-0.28676
0.061628	0.011955	1.49E-06	0.008892	0.037377	5.5E-05	0.005817	0.059838	0.001081
0.552801	0.210486	0.314806	0.097254	0.137092	0.074814	-0.33928	-0.14662	0.54874
1.6E-11	0.017536	0.000313	0.276712	0.124309	0.403183	9.54E-05	0.099998	2.4E-11
-0.1308	-0.13209	-0.10603	-0.4732	-0.41681	-0.37005	0.24393	0.168767	0.023817
0.142718	0.138781	0.235437	1.93E-08	1.09E-06	1.85E-05	0.005716	0.057864	0.790401
-0.00314	0.03455	-0.06148	-0.36256	-0.17704	-0.26474	0.225142	-0.05501	0.071244
0.97208	0.699778	0.492288	2.8E-05	0.046465	0.002631	0.010931	0.539061	0.426062
0.327658	-0.24187	0.029006	0.012624	-0.18669	0.213472	-0.27857	0.154942	0.206849
0.00017	0.006152	0.746156	0.88798	0.035584	0.015963	0.001518	0.081972	0.019631
0.553975	-0.10644	0.066125	0.216214	0.207143	0.344117	-0.46812	-0.16052	0.566165
1.42E-11	0.233653	0.46013	0.014627	0.019454	7.45E-05	2.86E-08	0.071423	4.01E-12
0.083651	-0.13229	-0.16109	-0.07323	0.010551	0.08872	-0.20486	-0.00655	0.175168
0.34978	0.138191	0.070405	0.41323	0.906279	0.321248	0.020865	0.941782	0.048863
0.147434	-0.3471	-0.20049	-0.33673	-0.28415	-0.09497	-0.10898	0.120894	0.314713
0.098102	6.39E-05	0.023815	0.000108	0.001206	0.288196	0.222606	0.175767	0.000314
-0.6584	-0.56955	-0.45276	-0.48356	-0.51439	-0.27669	0.403592	0.456499	-0.5329
4.03E-17	2.79E-12	9.07E-08	8.47E-09	6.17E-10	0.001638	2.54E-06	6.89E-08	1.12E-10
-0.00565	-0.15185	-0.05672	0.1997	0.206538	0.400252	-0.18801	0.013039	-0.01821
0.949701	0.088331	0.526485	0.024387	0.01982	3.12E-06	0.034284	0.884324	0.838942
-0.22575	0.179802	0.043637	0.161633	0.273134	0.039646	0.138105	-0.02301	-0.23648
0.010712	0.0431	0.626163	0.069453	0.00189	0.658093	0.121524	0.797379	0.007433
-0.42788	-0.33115	-0.22634	-0.08663	-0.1906	-0.05979	0.232832	0.299854	-0.47177
5.23E-07	0.000143	0.010502	0.332801	0.031838	0.504331	0.008432	0.000615	2.16E-08
0.370613	0.34955	0.301061	0.500164	0.370077	0.635706	-0.44196	0.010782	0.332796
1.79E-05	5.62E-05	0.000583	2.13E-09	1.85E-05	9.81E-16	1.97E-07	0.904237	0.000132

TOP2A	TOP2B	TOPBP1	TP73	TPD52	TPX2	TRA2B	TRAF4	TRAIP
blue	blue	blue	blue	blue	blue	blue	blue	blue
-0.46786	-0.32608	-0.37177	-0.33349	0.255772	-0.42277	-0.23223	-0.42996	-0.32909
2.92E-08	0.000183	1.68E-05	0.000127	0.003704	7.36E-07	0.008608	4.54E-07	0.000158
-0.44047	-0.3429	-0.3966	-0.31722	0.295613	-0.41476	-0.21802	-0.32421	-0.27886
2.19E-07	7.93E-05	3.91E-06	0.000279	0.00074	1.25E-06	0.013803	0.0002	0.0015
0.886292	0.610768	0.825824	0.572748	-0.587	0.899558	0.717042	0.622056	0.886378
1.31E-43	2.45E-14	6.87E-33	1.98E-12	4.08E-13	8.58E-47	2.51E-21	5.91E-15	1.25E-43
0.566491	0.412286	0.740085	0.207998	-0.31592	0.526761	0.700554	0.15632	0.57786
3.87E-12	1.46E-06	2.77E-23	0.018947	0.000297	2E-10	4.85E-20	0.079254	1.13E-12
-0.09264	-0.42216	-0.2182	-0.17079	-0.1025	-0.20882	0.227844	0.047754	0.159658
0.300222	7.67E-07	0.013723	0.054882	0.251495	0.018472	0.009988	0.593935	0.072976
0.214981	-0.08284	0.347529	-0.14585	-0.13617	0.10575	0.622772	-0.0837	0.349142
0.015215	0.354474	6.25E-05	0.101807	0.126874	0.236697	5.39E-15	0.349518	5.74E-05
-0.06142	-0.12604	0.137616	-0.21271	-0.08693	-0.10232	0.370282	-0.04468	0.158183
0.4927	0.157967	0.122861	0.016353	0.331145	0.252355	1.83E-05	0.617916	0.075698
-0.20323	-0.02246	-0.08411	0.139328	0.136809	-0.29196	-0.09406	-0.44308	-0.17379
0.021927	0.802128	0.347158	0.118228	0.125093	0.000867	0.292881	1.82E-07	0.050692
0.349747	-0.06604	0.292449	0.101684	-0.20738	0.409158	0.238756	0.091367	0.241071
5.57E-05	0.460696	0.000849	0.255311	0.019312	1.79E-06	0.006866	0.306966	0.006329
-0.27321	-0.52288	-0.35844	-0.28657	0.119624	-0.28267	-0.0981	-0.38926	-0.30606
0.001884	2.86E-10	3.51E-05	0.00109	0.180381	0.001282	0.272547	6.09E-06	0.000466
-0.12094	-0.23819	-0.09835	-0.24497	0.002113	-0.13015	0.027701	-0.47943	-0.15224
0.175597	0.007003	0.271309	0.005506	0.981186	0.144723	0.757212	1.18E-08	0.087509
-0.02755	-0.06334	-0.11107	0.078739	-0.1092	0.097979	-0.24165	0.124694	0.004389
0.758514	0.479292	0.21382	0.378891	0.221647	0.273126	0.0062	0.162477	0.960945
0.396922	0.475239	0.25618	0.574147	-0.36199	0.531503	-0.05922	0.446518	0.319437
3.84E-06	1.64E-08	0.003648	1.7E-12	2.89E-05	1.28E-10	0.508387	1.43E-07	0.000252
0.076092	-0.11577	-0.33412	0.319376	-0.18338	0.116415	-0.31629	0.344788	0.059576
0.395173	0.194936	0.000124	0.000252	0.039051	0.192438	0.000292	7.2E-05	0.505832
-0.05507	-0.1487	-0.34718	0.191668	-0.10157	0.03821	-0.37228	0.056851	-0.16076
0.53856	0.095222	6.36E-05	0.030875	0.255871	0.669741	1.63E-05	0.525521	0.070993
-0.58478	-0.15655	-0.65479	-0.30159	0.402728	-0.58964	-0.58445	-0.11328	-0.57537
5.24E-13	0.078818	6.82E-17	0.00057	2.68E-06	3.02E-13	5.44E-13	0.204786	1.49E-12
0.156991	0.48153	0.082617	0.304482	-0.1331	0.188757	-0.12215	0.502099	0.118347
0.077959	9.98E-09	0.355786	0.000501	0.135746	0.033562	0.171285	1.81E-09	0.185111
-0.02715	0.237906	0.248695	-0.21337	0.271469	-0.09588	0.127077	-0.08996	-0.072
0.761883	0.007074	0.004812	0.016012	0.00202	0.283597	0.154533	0.314507	0.421133
-0.34745	0.010238	-0.21192	-0.22315	0.366682	-0.36134	-0.35479	-0.11016	-0.37456
6.27E-05	0.909051	0.016765	0.011676	2.23E-05	3E-05	4.26E-05	0.217597	1.44E-05
0.39827	0.453967	0.461935	0.40897	-0.54512	0.4805	0.431628	0.718004	0.593218
3.53E-06	8.3E-08	4.59E-08	1.81E-06	3.44E-11	1.08E-08	4.05E-07	2.1E-21	2E-13

TRIB2	TRIM24	TRIM27	TRIM45	TRIM9	TRIP13	TRIQK	TRMT5	TROAP
blue	blue	blue	blue	blue	blue	blue	blue	blue
-0.31786	-0.40146	-0.16644	-0.13917	-0.22987	-0.43399	0.429879	-0.17054	-0.46333
0.000271	2.9E-06	0.061458	0.118659	0.009327	3.44E-07	4.57E-07	0.055249	4.13E-08
-0.30083	-0.34062	-0.11476	-0.10154	-0.17883	-0.37214	0.436951	-0.1295	-0.40271
0.000589	8.91E-05	0.198866	0.25601	0.044264	1.65E-05	2.81E-07	0.14676	2.68E-06
0.796179	0.881421	0.56438	0.527498	0.549402	0.8323	-0.57258	0.680558	0.849476
4.73E-29	1.54E-42	4.84E-12	1.87E-10	2.25E-11	7.97E-34	2.02E-12	1.35E-18	1.63E-36
0.612775	0.692718	0.357275	0.486356	0.391146	0.571781	-0.37641	0.677588	0.440328
1.91E-14	1.85E-19	3.73E-05	6.75E-09	5.44E-06	2.2E-12	1.29E-05	2.17E-18	2.21E-07
-0.2595	-0.08728	0.205865	-0.03856	-0.37603	-0.01479	0.212856	0.156772	-0.0853
0.003218	0.329214	0.020234	0.666924	1.32E-05	0.868937	0.016277	0.078381	0.34035
0.144194	0.322731	0.266001	0.258278	-0.07996	0.286064	0.022833	0.538421	0.083139
0.105795	0.000215	0.002505	0.00337	0.371501	0.001113	0.798873	6.62E-11	0.352747
0.135429	0.169929	0.108444	0.18625	-0.08449	0.128113	0.017217	0.246755	-0.07561
0.128983	0.056135	0.224901	0.036032	0.344979	0.151172	0.84765	0.005163	0.398211
-0.43845	-0.22257	-0.19764	0.14903	-0.38711	-0.29146	0.264791	-0.23735	-0.30304
2.53E-07	0.011901	0.025932	0.094479	6.93E-06	0.000885	0.002626	0.007213	0.000534
0.500901	0.355399	0.222852	0.098623	0.052205	0.323248	-0.32457	0.093252	0.312154
2E-09	4.13E-05	0.011791	0.269969	0.559962	0.00021	0.000197	0.29705	0.000353
-0.29673	-0.26647	-0.09421	-0.2115	-0.49632	-0.43382	0.190543	-0.28064	-0.34966
0.000705	0.00246	0.292091	0.016988	2.96E-09	3.48E-07	0.031891	0.001394	5.59E-05
-0.15243	-0.13667	0.012322	-0.11496	-0.22696	-0.25322	0.032669	-0.01773	-0.24728
0.087114	0.125484	0.890637	0.198087	0.010287	0.004074	0.7154	0.843152	0.005066
0.223504	0.032888	0.0686	-0.0653	0.220285	0.030855	-0.38528	-0.00159	0.103619
0.01154	0.713575	0.443475	0.465788	0.012825	0.730577	7.72E-06	0.985844	0.246332
0.525412	0.385714	0.174114	0.246187	0.50045	0.267713	-0.54467	0.001972	0.507454
2.26E-10	7.52E-06	0.05026	0.00527	2.08E-09	0.002343	3.6E-11	0.982441	1.14E-09
0.004891	-0.08627	0.111921	-0.09648	0.062899	-0.04045	-0.26661	-0.27891	0.20136
0.956482	0.334837	0.210297	0.280552	0.482355	0.651633	0.002446	0.001497	0.023203
-0.01158	-0.12267	-0.04428	-0.10826	-0.077	-0.28341	-0.19295	-0.43735	0.008279
0.897206	0.169468	0.621063	0.225673	0.389517	0.001243	0.029747	2.73E-07	0.926397
-0.54705	-0.52883	-0.32867	-0.30706	-0.25269	-0.66102	0.414047	-0.53572	-0.55976
2.84E-11	1.65E-10	0.000162	0.000446	0.004155	2.74E-17	1.31E-06	8.59E-11	7.83E-12
0.172278	0.139069	0.013663	-0.00592	0.427541	0.16864	-0.20885	0.016618	0.245045
0.052772	0.118922	0.878828	0.94737	5.35E-07	0.058055	0.018454	0.852887	0.005492
-0.05443	-0.03893	-0.13308	-0.06291	0.152519	0.159171	0.241703	0.223327	-0.09443
0.543326	0.663919	0.135805	0.482286	0.086927	0.073866	0.006188	0.011608	0.290973
-0.37262	-0.41252	-0.32353	-0.2955	-0.06648	-0.17699	0.357704	-0.24342	-0.28634
1.6E-05	1.44E-06	0.000207	0.000744	0.457751	0.046529	3.65E-05	0.005821	0.0011
0.578559	0.503357	0.298902	0.346258	0.304635	0.500208	-0.25336	0.410879	0.502133
1.05E-12	1.62E-09	0.000641	6.67E-05	0.000497	2.13E-09	0.004053	1.6E-06	1.8E-09

TSPAN12	TSPAN19	TTI1	TTK	TUBB	TUBG1	TYMS	UBA2	UBE2C
blue	blue	blue	blue	blue	blue	blue	blue	blue
-0.32417	0.386726	-0.2494	-0.40966	-0.27892	-0.3842	-0.36425	-0.28661	-0.421
0.000201	7.09E-06	0.004689	1.73E-06	0.001496	8.23E-06	2.56E-05	0.001088	8.29E-07
-0.26526	0.466271	-0.22914	-0.39315	-0.27098	-0.31539	-0.36623	-0.2436	-0.36364
0.002578	3.3E-08	0.009561	4.82E-06	0.00206	0.000304	2.29E-05	0.005784	2.64E-05
0.546587	-0.57247	0.555383	0.900555	0.75616	0.552827	0.797191	0.691424	0.835547
2.98E-11	2.04E-12	1.23E-11	4.74E-47	8.93E-25	1.59E-11	3.58E-29	2.29E-19	2.61E-34
0.493033	-0.40639	0.426793	0.657624	0.257705	0.102654	0.512506	0.714053	0.435818
3.89E-09	2.13E-06	5.63E-07	4.51E-17	0.003444	0.250781	7.29E-10	4.37E-21	3.04E-07
0.087059	0.423713	-0.40134	0.0025	-0.24561	0.034021	-0.03738	0.188851	0.015821
0.330425	6.92E-07	2.92E-06	0.977748	0.00538	0.704157	0.676471	0.033473	0.859866
0.359459	0.086885	-0.02438	0.377217	-0.17728	-0.10342	0.219968	0.574761	0.165007
3.32E-05	0.3314	0.785553	1.23E-05	0.046158	0.247237	0.012958	1.59E-12	0.063756
0.083798	0.075644	0.110305	0.1029	-0.21714	-0.09747	0.023549	0.342177	-0.0465
0.348927	0.397971	0.216996	0.249644	0.0142	0.275639	0.792706	8.23E-05	0.60367
-0.02887	0.159254	-0.13962	-0.16898	-0.37034	-0.29614	-0.26184	-0.09431	-0.31332
0.747269	0.073713	0.117449	0.057549	1.82E-05	0.000724	0.002943	0.29159	0.000335
0.100952	-0.49911	0.324583	0.349033	0.268804	0.149894	0.444567	0.119705	0.321227
0.258767	2.33E-09	0.000197	5.78E-05	0.002245	0.092563	1.64E-07	0.180085	0.000231
-0.20702	0.149198	-0.29404	-0.27385	-0.4007	-0.43996	-0.1047	-0.36097	-0.29089
0.019528	0.094104	0.000792	0.001837	3.04E-06	2.27E-07	0.24139	3.06E-05	0.000907
-0.04262	-0.00181	-0.16667	-0.09068	-0.2218	-0.41586	-0.03494	-0.08089	-0.19778
0.634216	0.983908	0.0611	0.310607	0.012207	1.16E-06	0.696568	0.365983	0.025819
-0.11914	-0.37747	0.317726	-0.14064	0.312666	0.173827	0.172761	-0.06017	0.008619
0.182157	1.22E-05	0.000273	0.114773	0.000345	0.050646	0.052101	0.501627	0.92338
-0.0188	-0.60223	0.528602	0.268393	0.656305	0.377149	0.409553	-0.06242	0.393564
0.833821	6.91E-14	1.68E-10	0.002281	5.47E-17	1.24E-05	1.74E-06	0.485725	4.71E-06
-0.09825	-0.07665	-0.12296	-0.05047	0.270735	0.328221	0.141231	-0.28329	0.171347
0.271786	0.391715	0.168457	0.573083	0.00208	0.000165	0.113237	0.001249	0.054085
-0.29211	-0.19055	-0.01845	-0.19054	0.105628	-0.05621	0.095231	-0.53692	-0.03474
0.000861	0.031883	0.836891	0.031894	0.23724	0.530193	0.286869	7.65E-11	0.698177
-0.38825	0.568006	-0.4612	-0.65106	-0.40444	-0.37786	-0.46134	-0.58027	-0.57482
6.47E-06	3.3E-12	4.85E-08	1.17E-16	2.41E-06	1.19E-05	4.79E-08	8.69E-13	1.58E-12
0.129324	-0.15605	0.10918	0.049675	0.431189	0.366766	0.041287	0.035002	0.177748
0.147314	0.079786	0.221749	0.579161	4.17E-07	2.22E-05	0.644886	0.696036	0.045579
0.221221	0.198038	-0.02316	0.037718	-0.10118	0.01771	-0.22574	0.333313	-0.07811
0.012439	0.025624	0.796065	0.673748	0.257684	0.84334	0.010715	0.000129	0.382754
-0.14052	0.323292	-0.17229	-0.36384	-0.14469	0.044147	-0.4878	-0.16572	-0.29528
0.115087	0.000209	0.052757	2.61E-05	0.1046	0.622134	6E-09	0.062595	0.000751
0.043386	-0.32942	0.412144	0.420549	0.562538	0.4159	0.408914	0.292024	0.494088
0.628156	0.000156	1.48E-06	8.53E-07	5.87E-12	1.16E-06	1.81E-06	0.000864	3.56E-09

UBE2T	UBFD1	UBR7	UBTD2	UGDH	UHRF1	URB2	USP1	USP10
blue	blue	blue	blue	blue	blue	blue	blue	blue
-0.33193	-0.31135	-0.07296	-0.30137	-0.1642	-0.56116	-0.46894	-0.3108	-0.49979
0.000138	0.000367	0.414963	0.000575	0.065076	6.77E-12	2.69E-08	0.000376	2.2E-09
-0.22266	-0.27237	0.018281	-0.32291	-0.23412	-0.50979	-0.46962	-0.33565	-0.44434
0.011866	0.001949	0.838356	0.000213	0.008068	9.27E-10	2.55E-08	0.000114	1.67E-07
0.668045	0.507721	0.505906	0.860824	0.581877	0.854172	0.801102	0.777169	0.666405
9.53E-18	1.11E-09	1.3E-09	1.76E-38	7.26E-13	2.63E-37	1.21E-29	6.55E-27	1.22E-17
0.355768	0.395394	0.512761	0.777851	0.527696	0.555706	0.689881	0.763196	0.390025
4.05E-05	4.21E-06	7.13E-10	5.54E-27	1.83E-10	1.19E-11	2.96E-19	1.82E-25	5.82E-06
0.360242	-0.25011	0.007087	0.078693	-0.09899	-0.25026	-0.37841	0.01649	-0.38032
3.18E-05	0.004569	0.936969	0.379172	0.268191	0.004544	1.15E-05	0.854005	1.03E-05
0.348148	0.092411	0.363948	0.548394	0.300189	0.095397	0.169703	0.528962	-0.09485
6.05E-05	0.301447	2.6E-05	2.49E-11	0.000606	0.286026	0.056468	1.63E-10	0.288788
0.101899	0.184168	0.1453	0.222778	0.165241	-0.04109	0.063254	0.138653	-0.04793
0.254301	0.0382	0.103119	0.01182	0.063376	0.646467	0.47988	0.120038	0.592557
-0.16481	-0.15876	-0.22527	-0.12242	-0.21883	-0.24017	0.011413	0.160525	-0.3507
0.064082	0.074618	0.010886	0.170322	0.013444	0.006533	0.898661	0.071412	5.29E-05
-0.11995	0.340171	0.00388	0.38509	0.636499	0.390452	0.397167	0.217201	0.171753
0.179207	9.12E-05	0.965471	7.8E-06	8.82E-16	5.68E-06	3.78E-06	0.014171	0.053509
-0.56498	-0.35	-0.39625	-0.18719	0.161272	-0.24677	-0.2277	-0.21861	-0.52666
4.54E-12	5.49E-05	4E-06	0.035093	0.070087	0.005161	0.010037	0.013541	2.02E-10
-0.40737	-0.23059	-0.07617	-0.02844	0.120938	-0.14165	-0.01431	0.039304	-0.357
2E-06	0.009104	0.394704	0.750957	0.175607	0.112156	0.87313	0.660863	3.79E-05
-0.23932	0.201626	0.086727	-0.11454	0.041236	0.13576	0.090208	-0.37804	0.328868
0.006731	0.023018	0.332279	0.19977	0.645296	0.128041	0.313165	1.18E-05	0.00016
-0.0521	0.244896	-0.05997	0.150042	0.276802	0.5731	0.522624	0.091395	0.533064
0.560731	0.005521	0.503033	0.092238	0.001631	1.91E-12	2.93E-10	0.306818	1.11E-10
-0.05156	-0.31125	-0.35295	-0.21148	-0.13222	0.192025	-0.0528	-0.18608	-0.03515
0.564828	0.000368	4.7E-05	0.016996	0.138377	0.030558	0.555515	0.036205	0.694851
-0.53971	-0.2622	-0.5282	-0.28358	0.102942	0.120161	0.002596	-0.29569	-0.12576
5.85E-11	0.002903	1.75E-10	0.001234	0.249449	0.178419	0.976888	0.000738	0.158871
-0.39423	-0.48809	-0.36077	-0.74784	-0.60262	-0.51616	-0.61584	-0.60867	-0.37909
4.52E-06	5.86E-09	3.09E-05	5.46E-24	6.59E-14	5.26E-10	1.3E-14	3.17E-14	1.11E-05
0.295264	0.117288	0.121064	-0.11513	-0.24634	0.219105	0.082858	-0.11874	0.461119
0.000752	0.189102	0.175155	0.197416	0.005242	0.013326	0.354384	0.183648	4.88E-08
0.370746	0.19134	0.433485	0.06162	-0.28281	-0.15225	-0.05862	0.152648	0.169449
1.78E-05	0.031168	3.57E-07	0.491322	0.001274	0.087494	0.512713	0.086656	0.056844
0.103624	0.010495	0.036466	-0.43637	-0.53411	-0.42636	-0.41667	-0.38114	-0.00463
0.246307	0.906775	0.683992	2.92E-07	1E-10	5.79E-07	1.1E-06	9.83E-06	0.958793
0.508448	0.392127	0.14781	0.463878	0.430978	0.342364	0.270569	0.176752	0.5266
1.04E-09	5.13E-06	0.09724	3.96E-08	4.24E-07	8.15E-05	0.002094	0.046825	2.03E-10

USP13	USP3	USP39	USP42	USP49	USP6NL	UVRAG	VANGL2	VASH2
blue	blue	blue	blue	blue	blue	blue	blue	blue
-0.19429	-0.22483	-0.26107	-0.37073	-0.1682	-0.26286	0.452216	-0.44831	-0.31874
0.028616	0.011046	0.003031	1.78E-05	0.05872	0.002829	9.44E-08	1.25E-07	0.00026
-0.13058	-0.19713	-0.205	-0.40303	-0.16843	-0.28257	0.449077	-0.44097	-0.32145
0.14338	0.026324	0.020778	2.63E-06	0.058368	0.001287	1.19E-07	2.12E-07	0.000229
0.603393	0.566803	0.683569	0.728079	0.522255	0.557381	-0.54024	0.613796	0.761915
6.01E-14	3.75E-12	8.33E-19	3.08E-22	3.03E-10	1E-11	5.55E-11	1.68E-14	2.44E-25
0.442147	0.363424	0.619401	0.654254	0.327075	0.460052	-0.32768	0.40207	0.517699
1.95E-07	2.67E-05	8.3E-15	7.37E-17	0.000175	5.28E-08	0.000169	2.79E-06	4.58E-10
-0.1863	-0.08983	0.382684	-0.29061	-0.40336	-0.17431	0.035594	-0.42355	-0.09064
0.035978	0.315224	8.99E-06	0.000918	2.58E-06	0.05	0.69116	6.99E-07	0.310824
0.140615	0.078265	0.597304	0.194738	-0.1215	0.159358	-0.08298	-0.10691	0.200385
0.114836	0.38178	1.24E-13	0.02824	0.173587	0.073524	0.353699	0.231582	0.023892
0.073377	0.054566	0.394726	0.041064	-0.11362	0.053744	0.055365	-0.07142	-0.01818
0.412302	0.542324	4.39E-06	0.646672	0.203404	0.548433	0.536422	0.424942	0.83922
-0.2371	-0.27248	-0.06657	-0.20679	-0.18678	-0.18333	-0.10795	0.139981	-0.17933
0.007276	0.00194	0.457125	0.019666	0.035499	0.039097	0.227044	0.116498	0.043664
0.12386	0.320298	0.217605	0.41982	-0.04666	-0.02088	-0.21824	0.208666	0.293805
0.165326	0.000242	0.013988	8.95E-07	0.602424	0.815779	0.013706	0.018559	0.000801
-0.43851	-0.03583	-0.14827	-0.17664	-0.48471	-0.27953	0.187529	-0.30098	-0.36808
2.52E-07	0.689246	0.096188	0.046966	7.72E-09	0.001459	0.034753	0.000585	2.07E-05
-0.24586	-0.09357	-0.05304	0.035288	-0.24855	-0.09867	0.167517	-0.13527	-0.18439
0.005333	0.295381	0.553701	0.693682	0.004838	0.269729	0.05977	0.129429	0.03796
-0.01783	0.288955	-0.04012	0.182192	0.121462	-0.10703	0.176917	0.206702	-0.08095
0.842285	0.000985	0.654237	0.040355	0.17373	0.231059	0.046617	0.019721	0.365618
0.254682	0.471265	-0.00399	0.47696	0.464008	0.235246	-0.30955	0.626597	0.319277
0.003858	2.24E-08	0.964532	1.44E-08	3.92E-08	0.00776	0.000398	3.29E-15	0.000253
-0.21561	0.07318	-0.12458	0.018078	-0.12929	-0.15775	-0.29664	0.099593	0.025591
0.014914	0.413564	0.162869	0.840131	0.147434	0.076521	0.000708	0.265264	0.775192
-0.26275	0.181193	-0.31455	0.045444	-0.13642	-0.18785	-0.05728	0.148238	-0.1674
0.002842	0.041484	0.000316	0.611922	0.126186	0.034439	0.522402	0.096264	0.059947
-0.28296	-0.3288	-0.613	-0.59284	-0.16101	-0.20984	0.523527	-0.38334	-0.60672
0.001267	0.00016	1.86E-14	2.09E-13	0.070542	0.017891	2.7E-10	8.65E-06	4.02E-14
0.213922	0.080806	-0.10988	0.023203	0.391763	0.289233	-0.1095	0.306202	0.152094
0.015737	0.366466	0.218767	0.795688	5.25E-06	0.000974	0.220377	0.000463	0.087819
0.224508	-0.31074	-0.03536	-0.1023	0.2023	0.186926	0.079819	-0.1484	0.112599
0.011163	0.000377	0.693067	0.252413	0.022554	0.035352	0.372372	0.095903	0.20753
-0.07421	-0.41998	-0.38614	-0.47424	0.035598	-0.12375	0.24049	-0.26412	-0.21543
0.407014	8.86E-07	7.34E-06	1.78E-08	0.691129	0.165695	0.00646	0.002695	0.015
0.527254	0.657177	0.387112	0.299291	0.520554	0.438389	-0.017	0.212535	0.429965
1.91E-10	4.82E-17	6.93E-06	0.00063	3.54E-10	2.54E-07	0.849504	0.016442	4.54E-07

VAV2	VEZF1	VILL	VSX1	WDHD1	WDR27	WDR33	WDR4	WDR62
blue	blue	blue	blue	blue	blue	blue	blue	blue
-0.27829	-0.26383	0.216841	0.203079	-0.32379	-0.30202	-0.17679	-0.32427	-0.45976
0.001535	0.002726	0.014337	0.022029	0.000205	0.000559	0.046772	0.0002	5.4E-08
-0.29972	-0.30098	0.168774	0.235162	-0.29631	-0.31923	-0.1946	-0.32715	-0.4388
0.000618	0.000585	0.057853	0.007782	0.000718	0.000254	0.028355	0.000174	2.47E-07
0.710724	0.713142	-0.62635	-0.54617	0.880253	0.656382	0.535624	0.661107	0.777311
8.01E-21	5.16E-21	3.39E-15	3.1E-11	2.74E-42	5.41E-17	8.67E-11	2.7E-17	6.33E-27
0.399224	0.63248	-0.53003	-0.42699	0.756587	0.580863	0.529725	0.411854	0.27079
3.33E-06	1.51E-15	1.47E-10	5.55E-07	8.12E-25	8.13E-13	1.52E-10	1.5E-06	0.002075
-0.31935	-0.06812	0.121958	-0.10326	0.027174	-0.17032	-0.11485	-0.01271	-0.31444
0.000253	0.4467	0.171967	0.247994	0.761692	0.05556	0.198511	0.887209	0.000318
-0.06594	0.304498	-0.23488	-0.31976	0.476837	0.19978	0.272213	0.143777	-0.22857
0.461421	0.0005	0.00786	0.000248	1.45E-08	0.024328	0.001961	0.106819	0.009745
0.004203	0.152536	0.011803	-0.11422	0.165127	0.147346	0.099735	0.07483	-0.28336
0.962597	0.086891	0.895219	0.201005	0.063562	0.098306	0.26458	0.403084	0.001246
-0.35822	0.013688	0.265299	0.150939	-0.17729	-0.00142	0.036992	-0.18525	-0.46344
3.55E-05	0.878603	0.002575	0.090285	0.046151	0.987329	0.679684	0.037061	4.09E-08
0.384464	0.21499	-0.08785	-0.30792	0.312164	0.232736	0.295501	0.3561	0.224794
8.1E-06	0.015211	0.326022	0.000429	0.000353	0.00846	0.000744	3.98E-05	0.011058
-0.21329	-0.06459	0.281109	0.094993	-0.30779	-0.23484	-0.03609	-0.12921	-0.4107
0.016055	0.470649	0.001367	0.288081	0.000431	0.007869	0.687107	0.147683	1.62E-06
-0.13622	0.0258	0.115185	0.004387	-0.07276	-0.10096	0.071986	-0.0701	-0.28925
0.126748	0.773407	0.197215	0.960959	0.416255	0.258732	0.421242	0.43356	0.000973
0.207788	0.017667	-0.10258	-0.03611	-0.08652	-0.03591	0.048206	0.018066	0.260015
0.019071	0.843719	0.251122	0.686926	0.333465	0.688545	0.590439	0.840235	0.003155
0.663217	0.369471	-0.22472	-0.08095	0.199446	0.450113	0.309528	0.441688	0.73997
1.97E-17	1.91E-05	0.011086	0.365636	0.024573	1.1E-07	0.000398	2.01E-07	2.84E-23
0.138381	0.002367	0.18936	0.051143	-0.15135	-0.0271	-0.13588	0.268116	0.409674
0.120776	0.97893	0.03299	0.567985	0.089395	0.762346	0.127703	0.002306	1.73E-06
0.181591	0.043796	0.227762	0.148666	-0.31493	-0.01646	0.024396	0.133917	0.208199
0.041032	0.624909	0.010015	0.095297	0.000311	0.854299	0.785431	0.133351	0.01883
-0.50696	-0.33225	0.291972	0.511368	-0.73131	-0.46385	-0.40437	-0.51803	-0.32906
1.19E-09	0.000135	0.000866	8.07E-10	1.63E-22	3.97E-08	2.42E-06	4.45E-10	0.000158
0.195943	0.045545	-0.20034	0.05955	-0.03607	0.02204	-0.16057	0.000533	0.479342
0.027259	0.611133	0.023926	0.506015	0.687247	0.805717	0.071329	0.995251	1.19E-08
-0.21384	-0.18457	-0.15626	-0.01909	0.139463	-0.11269	-0.2151	-0.26827	-0.12376
0.01578	0.037777	0.079367	0.831344	0.117869	0.207145	0.015156	0.002293	0.165656
-0.34015	-0.52859	0.185561	0.277964	-0.35611	-0.39508	-0.45999	-0.4404	-0.20416
9.12E-05	1.69E-10	0.036737	0.001555	3.97E-05	4.29E-06	5.31E-08	2.2E-07	0.021315
0.574557	0.392139	-0.48551	-0.40227	0.418703	0.48199	0.330591	0.385682	0.510949
1.63E-12	5.13E-06	7.23E-09	2.76E-06	9.64E-07	9.61E-09	0.000147	7.54E-06	8.37E-10

WDR76	WNT2B	WRAP53	WWP1	XKR5	XPO5	XRCC2	XRCC3	XXYLT1
blue	blue	blue	blue	blue	blue	blue	blue	blue
-0.2947	0.323143	-0.31651	0.38233	-0.33985	-0.36654	-0.4589	-0.38142	-0.40744
0.00077	0.000211	0.000289	9.17E-06	9.27E-05	2.25E-05	5.76E-08	9.67E-06	1.99E-06
-0.321	0.234768	-0.28079	0.347022	-0.34766	-0.31932	-0.46012	-0.34978	-0.38788
0.000234	0.007889	0.001386	6.41E-05	6.2E-05	0.000253	5.25E-08	5.55E-05	6.62E-06
0.760371	-0.49355	0.670061	-0.55798	0.696747	0.801823	0.87405	0.595892	0.751721
3.47E-25	3.73E-09	7E-18	9.42E-12	9.33E-20	9.88E-30	5.26E-41	1.46E-13	2.37E-24
0.66936	-0.44789	0.329161	-0.22855	0.290482	0.615881	0.775641	0.171319	0.463782
7.8E-18	1.29E-07	0.000158	0.009754	0.000923	1.3E-14	9.54E-27	0.054125	3.99E-08
0.023471	-0.11625	0.270856	0.03083	-0.29597	-0.25377	-0.15937	0.063413	0.039176
0.793382	0.193071	0.00207	0.730787	0.000729	0.003992	0.073503	0.478774	0.661901
0.458004	-0.3543	0.195051	0.044552	-0.15948	0.166087	0.371429	-0.04406	0.220278
6.16E-08	4.38E-05	0.027982	0.618937	0.073298	0.062016	1.71E-05	0.622781	0.012828
0.090855	-0.25426	0.093661	0.148295	-0.14567	0.139386	0.116352	-0.25382	0.102882
0.309696	0.003919	0.294927	0.096136	0.102245	0.118073	0.19268	0.003983	0.249724
0.005	0.068353	-0.04963	0.202893	-0.3583	-0.16404	-0.02893	-0.33548	-0.36558
0.955511	0.445123	0.579476	0.022154	3.53E-05	0.065354	0.746825	0.000115	2.37E-05
0.257617	-0.19449	0.177617	-0.15738	0.279182	0.42326	0.267019	0.026721	0.371002
0.003455	0.028445	0.045742	0.077211	0.00148	7.13E-07	0.002408	0.765543	1.76E-05
-0.14403	0.251754	-0.13242	0.306795	-0.39015	-0.2964	-0.37193	-0.31368	-0.27615
0.106186	0.004301	0.137774	0.000451	5.78E-06	0.000716	1.67E-05	0.000329	0.001674
0.112586	0.10675	-0.17038	0.156283	-0.29764	-0.08055	-0.0832	-0.20753	-0.24342
0.207581	0.232269	0.055482	0.079328	0.000678	0.367975	0.352385	0.019222	0.005821
-0.03194	-0.06317	0.021776	-0.3349	0.227267	0.259545	-0.1191	0.053279	0.006344
0.721509	0.480436	0.807999	0.000119	0.010183	0.003212	0.182324	0.551905	0.943569
0.127662	0.017424	0.387676	-0.45113	0.629674	0.520206	0.295707	0.433519	0.332483
0.152627	0.845839	6.7E-06	1.02E-07	2.19E-15	3.65E-10	0.000737	3.56E-07	0.000134
-0.19213	0.115024	0.406916	-0.40114	0.208614	-0.07162	-0.17324	0.552251	0.143203
0.030469	0.197846	2.06E-06	2.96E-06	0.018589	0.423629	0.051438	1.69E-11	0.10824
-0.23748	0.307548	0.129388	-0.10487	0.104198	-0.03528	-0.27256	0.134453	-0.07356
0.007179	0.000436	0.147111	0.240653	0.243688	0.693748	0.001934	0.131791	0.411107
-0.6294	0.49551	-0.46911	0.427718	-0.35292	-0.58087	-0.61758	-0.32958	-0.61618
2.27E-15	3.16E-09	2.65E-08	5.29E-07	4.71E-05	8.13E-13	1.05E-14	0.000155	1.25E-14
-0.0758	-0.00401	0.084282	-0.26504	0.397799	0.112957	0.096822	0.289385	0.102256
0.397024	0.964343	0.346146	0.0026	3.63E-06	0.206077	0.278859	0.000967	0.252636
0.067595	-0.16557	-0.3561	0.152673	-0.06178	-0.11685	0.162117	-0.11269	-0.04502
0.450197	0.062851	3.98E-05	0.086604	0.490191	0.190772	0.068612	0.207165	0.615228
-0.37134	0.120427	-0.45263	0.207782	-0.17127	-0.3812	-0.32161	-0.21554	-0.30807
1.72E-05	0.177452	9.15E-08	0.019074	0.054188	9.8E-06	0.000227	0.014948	0.000426
0.279251	-0.09371	0.496907	-0.23059	0.581263	0.381068	0.342236	0.356797	0.546619
0.001476	0.294671	2.81E-09	0.009104	7.78E-13	9.87E-06	8.21E-05	3.83E-05	2.97E-11

YPEL2	YTHDF2	ZBED4	ZBTB5	ZBTB8A	ZBTB8B	ZC3HAV1L	ZC3HC1	ZEB1
blue	blue	blue	blue	blue	blue	blue	blue	blue
0.411534	-0.2243	-0.43022	-0.26553	-0.32128	-0.42387	-0.3081	-0.23321	-0.258
1.53E-06	0.011242	4.46E-07	0.002551	0.000231	6.84E-07	0.000425	0.008324	0.003406
0.451087	-0.21223	-0.4948	-0.24911	-0.34101	-0.37028	-0.32668	-0.14455	-0.23484
1.02E-07	0.016604	3.36E-09	0.004739	8.74E-05	1.83E-05	0.000178	0.104933	0.007869
-0.74384	0.677534	0.812851	0.644973	0.652288	0.630054	0.647743	0.668358	0.655486
1.27E-23	2.19E-18	3.98E-31	2.75E-16	9.78E-17	2.09E-15	1.87E-16	9.09E-18	6.16E-17
-0.54273	0.669161	0.540481	0.58063	0.470299	0.305536	0.610485	0.619753	0.298998
4.36E-11	8.04E-18	5.43E-11	8.35E-13	2.42E-08	0.000477	2.53E-14	7.94E-15	0.000639
0.152193	0.051921	-0.34097	-0.0947	-0.12836	-0.14728	-0.22663	0.263402	-0.37338
0.08761	0.5621	8.75E-05	0.289571	0.150374	0.098454	0.010401	0.002771	1.53E-05
-0.15083	0.494614	0.021251	0.276235	0.15322	-0.02847	0.253943	0.529791	-0.18929
0.090527	3.41E-09	0.812544	0.001669	0.085469	0.750713	0.003966	1.51E-10	0.033057
0.002092	0.198046	-0.07321	0.07301	-0.13255	-0.09579	0.024685	0.410232	-0.15674
0.981381	0.025618	0.413382	0.414649	0.137407	0.28406	0.782952	1.67E-06	0.078446
0.045901	0.007196	-0.23611	0.040459	-0.2738	-0.12495	-0.23482	-0.11431	-0.40409
0.608348	0.936003	0.007532	0.651534	0.001841	0.161605	0.007875	0.200643	2.46E-06
-0.35586	0.355354	0.520289	0.179715	0.078752	-0.10838	0.331708	0.320247	0.180241
4.03E-05	4.14E-05	3.62E-10	0.043202	0.378817	0.225158	0.000139	0.000242	0.042584
0.170116	-0.076	-0.12975	-0.1526	-0.517	-0.61713	-0.32663	-0.10105	-0.37889
0.055861	0.395754	0.145975	0.086765	4.88E-10	1.11E-14	0.000178	0.258304	1.12E-05
-0.04556	-0.00814	-0.09024	0.009696	-0.25572	-0.41715	-0.06302	0.035866	-0.32702
0.611019	0.927641	0.312973	0.913843	0.00371	1.07E-06	0.481483	0.688921	0.000175
-0.09181	-0.16809	0.203482	0.090838	0.040316	0.025912	0.13112	0.131328	0.17304
0.304617	0.058895	0.021761	0.309784	0.652692	0.772447	0.141729	0.14109	0.051718
-0.5036	0.064459	0.692045	0.298445	0.237099	0.371369	0.242672	0.091938	0.636886
1.59E-09	0.471534	2.07E-19	0.000654	0.007276	1.72E-05	0.005979	0.303939	8.37E-16
-0.22068	-0.21902	0.180887	-0.11368	-0.04491	0.076194	-0.24949	-0.1349	0.098978
0.012662	0.013362	0.041835	0.203182	0.616119	0.39454	0.004674	0.130485	0.268245
-0.12476	-0.22937	0.260171	-0.07801	-0.32312	-0.22471	-0.25436	-0.22271	0.07374
0.16226	0.009487	0.003136	0.383318	0.000211	0.011088	0.003904	0.011845	0.409988
0.65481	-0.5559	-0.52291	-0.39549	-0.4667	-0.24085	-0.54189	-0.58842	-0.24169
6.8E-17	1.17E-11	2.85E-10	4.19E-06	3.19E-08	0.006378	4.73E-11	3.47E-13	0.006191
-0.09093	-0.13979	0.14918	0.097452	0.253532	0.485669	0.093634	-0.12906	0.459419
0.309276	0.117009	0.094145	0.275729	0.004027	7.14E-09	0.295068	0.148134	5.54E-08
0.220974	0.058813	-0.30746	-0.07863	0.359531	0.227433	0.202939	-0.11114	-0.00082
0.01254	0.511303	0.000438	0.379569	3.31E-05	0.010126	0.022122	0.213501	0.992683
0.476741	-0.38563	-0.52922	-0.37898	-0.01857	0.070463	-0.19825	-0.43479	-0.10921
1.46E-08	7.56E-06	1.59E-10	1.11E-05	0.835836	0.431159	0.025464	3.26E-07	0.221636
-0.23867	0.347024	0.573174	0.360633	0.404538	0.438224	0.386038	0.276986	0.666632
0.006888	6.41E-05	1.89E-12	3.11E-05	2.39E-06	2.57E-07	7.38E-06	0.001619	1.18E-17

ZFP37	ZFP62	ZGRF1	ZIK1	ZKSCAN2	ZKSCAN7	ZMYND19	ZNF100	ZNF131
blue	blue	blue	blue	blue	blue	blue	blue	blue
-0.09595	-0.19493	-0.30448	-0.34444	-0.33672	-0.3875	-0.30699	-0.34408	-0.19339
0.283253	0.028079	0.000501	7.33E-05	0.000108	6.77E-06	0.000447	7.47E-05	0.029369
-0.03329	-0.21506	-0.29972	-0.32225	-0.25656	-0.41497	-0.22688	-0.34774	-0.2141
0.710194	0.015178	0.000619	0.00022	0.003596	1.23E-06	0.010315	6.18E-05	0.01565
0.652077	0.662934	0.792321	0.840618	0.662665	0.71526	0.519743	0.77039	0.699521
1.01E-16	2.06E-17	1.34E-28	4.36E-35	2.14E-17	3.5E-21	3.81E-10	3.39E-26	5.8E-20
0.679478	0.683621	0.771572	0.755227	0.522671	0.696873	0.054062	0.725911	0.746627
1.61E-18	8.26E-19	2.55E-26	1.1E-24	2.92E-10	9.13E-20	0.546065	4.68E-22	7.07E-24
0.321269	-0.13275	-0.13369	-0.07617	-0.23477	-0.04028	0.357532	-0.12376	0.112589
0.000231	0.136792	0.134021	0.394682	0.00789	0.652956	3.68E-05	0.165688	0.20757
0.647555	0.360468	0.415069	0.399949	0.105167	0.397043	0.042254	0.384422	0.597375
1.92E-16	3.14E-05	1.22E-06	3.18E-06	0.239308	3.81E-06	0.637156	8.12E-06	1.23E-13
0.37581	0.181479	0.191195	0.298482	0.12222	0.243702	-0.04315	0.132171	0.287771
1.34E-05	0.041158	0.031298	0.000653	0.171039	0.005763	0.629997	0.138533	0.001036
-0.00973	0.139791	-0.04157	-0.07385	-0.13262	0.027011	-0.26973	-0.12055	-0.09946
0.913524	0.116999	0.642607	0.40927	0.137197	0.763072	0.002164	0.177025	0.265929
-0.02683	0.112531	0.187521	0.393758	-0.03336	0.412056	-0.10675	0.193812	0.086431
0.76458	0.207803	0.034761	4.65E-06	0.709622	1.48E-06	0.232281	0.029013	0.33394
-0.25297	-0.22711	-0.26512	-0.22864	-0.57082	-0.06655	-0.39133	-0.2753	-0.27758
0.004112	0.010238	0.002593	0.009723	2.44E-12	0.457244	5.39E-06	0.001733	0.00158
-0.10089	0.02313	-0.02984	-0.06238	-0.35392	0.045234	-0.39871	-0.07356	-0.01931
0.259046	0.796312	0.73909	0.486006	4.47E-05	0.613571	3.44E-06	0.411158	0.829396
-0.29516	-0.14962	-0.09597	-0.00022	0.109508	0.116948	-0.07395	-0.24465	-0.35728
0.000755	0.093177	0.283125	0.998015	0.220355	0.190399	0.408649	0.00557	3.73E-05
-0.10237	0.267564	0.266452	0.348255	0.381667	0.314679	0.20673	0.233905	-0.07318
0.25211	0.002357	0.002462	6.02E-05	9.54E-06	0.000314	0.019704	0.008127	0.413594
-0.32658	-0.29288	-0.27272	-0.15169	-0.20819	-0.09109	0.417064	-0.14894	-0.37205
0.000179	0.000833	0.001922	0.088681	0.018833	0.308424	1.07E-06	0.09468	1.65E-05
-0.47732	-0.20998	-0.24455	-0.1692	-0.31662	-0.03732	-0.10145	-0.24628	-0.51801
1.4E-08	0.017816	0.00559	0.05721	0.000287	0.676996	0.256403	0.005252	4.45E-10
-0.36911	-0.40538	-0.45671	-0.69346	-0.32788	-0.58561	-0.25382	-0.54565	-0.62183
1.95E-05	2.27E-06	6.78E-08	1.63E-19	0.000168	4.78E-13	0.003983	3.27E-11	6.09E-15
-0.06224	-0.02464	0.010735	-0.01892	0.333701	-0.13624	0.347204	0.044033	-0.06356
0.486984	0.783335	0.904652	0.832796	0.000126	0.126695	6.35E-05	0.623031	0.477771
0.183896	0.037397	0.089589	-0.05461	0.251082	-0.171	-0.00096	0.184099	0.348694
0.03849	0.676372	0.316513	0.542006	0.004409	0.054585	0.99142	0.038272	5.88E-05
-0.2861	-0.35036	-0.38648	-0.44251	-0.0374	-0.53919	0.002064	-0.32903	-0.20938
0.001111	5.39E-05	7.19E-06	1.9E-07	0.676338	6.15E-11	0.981624	0.000159	0.018154
0.448101	0.371121	0.476721	0.433469	0.558874	0.375628	0.548298	0.317537	0.405867
1.27E-07	1.74E-05	1.46E-08	3.57E-07	8.59E-12	1.35E-05	2.51E-11	0.000275	2.2E-06

ZNF134	ZNF146	ZNF180	ZNF202	ZNF207	ZNF212	ZNF22	ZNF227	ZNF232
blue	blue	blue	blue	blue	blue	blue	blue	blue
-0.24894	-0.17788	-0.29683	-0.14911	-0.1917	-0.32963	-0.27642	-0.37422	-0.21918
0.00477	0.045423	0.000702	0.094299	0.030844	0.000154	0.001656	1.46E-05	0.013293
-0.27483	-0.18859	-0.28339	-0.12345	-0.15351	-0.30706	-0.25934	-0.34274	-0.16959
0.001766	0.033722	0.001244	0.166734	0.084871	0.000446	0.003237	8E-05	0.056629
0.620364	0.641817	0.661056	0.664406	0.691234	0.630658	0.758563	0.594425	0.687141
7.34E-15	4.26E-16	2.72E-17	1.65E-17	2.37E-19	1.93E-15	5.22E-25	1.74E-13	4.66E-19
0.646727	0.582036	0.733153	0.613965	0.702866	0.420136	0.586254	0.547519	0.533083
2.15E-16	7.14E-13	1.13E-22	1.65E-14	3.24E-20	8.77E-07	4.44E-13	2.72E-11	1.1E-10
-0.23175	-0.35908	0.002994	0.103814	0.177196	-0.14355	0.078916	-0.28976	0.274137
0.008751	3.39E-05	0.973346	0.245438	0.046266	0.107371	0.377818	0.000952	0.001816
0.294128	0.147846	0.506668	0.406774	0.582887	0.075381	0.356827	0.138148	0.409762
0.00079	0.097158	1.22E-09	2.08E-06	6.49E-13	0.399621	3.82E-05	0.121409	1.72E-06
0.284326	0.129386	0.296906	0.345807	0.394811	0.034443	0.141771	0.199642	0.212094
0.001197	0.147117	0.0007	6.83E-05	4.36E-06	0.700659	0.111851	0.024429	0.016673
-0.12652	-0.14713	0.068493	-0.18939	0.024626	-0.21146	-0.29543	-0.11658	-0.04014
0.156364	0.09881	0.444187	0.032957	0.783453	0.017006	0.000746	0.191794	0.654104
0.297513	0.46583	0.217176	0.230809	0.283589	0.350156	0.261906	0.391023	0.075834
0.000682	3.41E-08	0.014183	0.009035	0.001234	5.45E-05	0.002935	5.48E-06	0.396788
-0.24512	-0.07684	-0.2251	-0.18173	-0.11513	-0.13321	-0.17047	-0.15189	-0.26674
0.005478	0.390543	0.010945	0.040872	0.197437	0.13543	0.055343	0.088241	0.002434
-0.03028	0.042877	-0.05381	-0.04732	0.034996	-0.02155	-0.1495	-0.10847	-0.15486
0.735392	0.632195	0.547919	0.597315	0.69609	0.809992	0.093426	0.224802	0.08214
-0.06892	0.05345	-0.23969	0.138318	-0.0532	0.35488	-0.05261	0.186202	-0.11458
0.441347	0.550625	0.006645	0.120945	0.552507	4.24E-05	0.55695	0.036081	0.199575
0.259717	0.493357	0.09422	0.237831	-0.0041	0.541695	0.282881	0.488618	0.162824
0.003191	3.79E-09	0.29204	0.007093	0.963517	4.82E-11	0.001271	5.61E-09	0.067397
-0.30963	-0.14126	-0.28239	-0.19017	-0.35672	0.243176	0.005491	-0.05759	0.03148
0.000397	0.113164	0.001297	0.032229	3.84E-05	0.005872	0.951148	0.520135	0.725331
-0.25377	0.10152	-0.33103	-0.1969	-0.36826	0.212235	-0.08746	0.074101	-0.22356
0.003992	0.256083	0.000144	0.026503	2.05E-05	0.016599	0.328183	0.407691	0.011521
-0.58175	-0.36404	-0.57114	-0.44233	-0.56905	-0.46651	-0.48289	-0.45116	-0.45519
7.36E-13	2.58E-05	2.36E-12	1.92E-07	2.95E-12	3.24E-08	8.94E-09	1.02E-07	7.58E-08
0.006914	-0.01711	-0.13836	-0.045	-0.09661	0.062037	0.03263	0.014495	0.028426
0.938504	0.848615	0.120829	0.615431	0.279896	0.488383	0.715722	0.871508	0.751061
0.126389	-0.19272	0.137169	-0.09052	0.090758	-0.32552	-0.074	-0.20138	-0.03711
0.156794	0.02995	0.124095	0.311475	0.310211	0.000188	0.408325	0.023187	0.678722
-0.22961	-0.457	-0.33608	-0.3915	-0.32966	-0.48167	-0.43047	-0.41947	-0.32996
0.00941	6.63E-08	0.000112	5.33E-06	0.000154	9.86E-09	4.39E-07	9.16E-07	0.000152
0.333722	0.341797	0.247669	0.620759	0.325964	0.315395	0.597517	0.32498	0.419708
0.000126	8.39E-05	0.004995	6.98E-15	0.000184	0.000304	1.21E-13	0.000193	9.02E-07

ZNF250	ZNF253	ZNF254	ZNF286A	ZNF286B	ZNF300	ZNF311	ZNF326	ZNF367
blue	blue	blue	blue	blue	blue	blue	blue	blue
-0.26131	-0.26056	-0.33732	-0.2437	-0.22285	-0.26849	-0.2953	-0.2346	-0.33668
0.003003	0.003091	0.000105	0.005763	0.011793	0.002273	0.000751	0.007935	0.000109
-0.25287	-0.23139	-0.31708	-0.2235	-0.23036	-0.20974	-0.24928	-0.27159	-0.34354
0.004127	0.008857	0.000281	0.011542	0.009173	0.017951	0.00471	0.002011	7.68E-05
0.637227	0.698804	0.688331	0.75147	0.734786	0.804691	0.706872	0.463179	0.784113
7.99E-16	6.56E-20	3.83E-19	2.5E-24	8.15E-23	4.37E-30	1.6E-20	4.17E-08	1.15E-27
0.366541	0.57853	0.727849	0.563712	0.594543	0.654052	0.581787	0.366727	0.581035
2.25E-05	1.05E-12	3.22E-22	5.19E-12	1.71E-13	7.59E-17	7.34E-13	2.23E-05	7.98E-13
-0.005	-0.12043	-0.0435	-0.14635	-0.11692	0.168462	0.119553	-0.30667	-0.14259
0.955465	0.177437	0.627255	0.100632	0.19052	0.058324	0.180641	0.000454	0.109783
0.086903	0.245638	0.465719	0.196496	0.230045	0.482834	0.385997	0.015115	0.265752
0.3313	0.005376	3.44E-08	0.02682	0.009273	8.98E-09	7.4E-06	0.866062	0.00253
0.070957	0.065833	0.254029	0.013421	0.086065	0.224928	0.120486	-0.07676	-0.12219
0.42793	0.462116	0.003953	0.880952	0.335997	0.011009	0.177241	0.391006	0.17114
-0.271	-0.16306	-0.13305	-0.40046	-0.29284	-0.12508	-0.03803	-0.05139	-0.0259
0.002058	0.066997	0.135899	3.08E-06	0.000834	0.161178	0.671208	0.566106	0.772527
-0.06337	0.063229	0.184615	0.202178	0.138763	0.156506	0.213716	0.082211	0.273035
0.479051	0.480055	0.037725	0.022637	0.119743	0.078895	0.01584	0.358166	0.001898
-0.57353	-0.39898	-0.33776	-0.37469	-0.3935	-0.2603	-0.07174	-0.06665	-0.13793
1.82E-12	3.38E-06	0.000103	1.42E-05	4.72E-06	0.003121	0.422823	0.456557	0.121997
-0.37047	-0.18531	-0.15094	-0.23325	-0.13935	-0.10873	-0.03272	-0.03392	-0.00141
1.81E-05	0.036996	0.090292	0.008313	0.118183	0.223684	0.714986	0.704961	0.987476
0.09961	-0.08764	-0.2823	0.027859	0.089045	-0.14266	-0.11616	-0.16363	-0.19618
0.265184	0.327223	0.001302	0.755867	0.319474	0.109601	0.193412	0.06604	0.027068
0.334591	0.313343	0.062498	0.386734	0.360617	0.121154	0.26499	0.402723	0.323124
0.000121	0.000334	0.485155	7.08E-06	3.12E-05	0.174832	0.002606	2.68E-06	0.000211
-0.05724	-0.07013	-0.26454	-0.05032	-0.06418	-0.19008	0.088294	0.03248	0.034497
0.522701	0.433343	0.002651	0.574228	0.473431	0.032317	0.323586	0.716972	0.70021
-0.31645	-0.21441	-0.42833	-0.15029	-0.20076	-0.31191	-0.00105	0.163359	0.002618
0.000289	0.015494	5.07E-07	0.091701	0.023623	0.000357	0.990634	0.06649	0.976696
-0.38624	-0.31768	-0.59567	-0.38359	-0.41977	-0.47717	-0.45061	-0.02122	-0.42148
7.29E-06	0.000273	1.5E-13	8.52E-06	8.99E-07	1.41E-08	1.06E-07	0.812791	8.02E-07
0.379181	0.205133	0.017413	0.22153	0.212896	0.066968	-0.0881	0.158299	0.020637
1.1E-05	0.020693	0.84593	0.012314	0.016256	0.454417	0.324679	0.075482	0.817868
0.19944	0.127874	0.343067	0.059863	0.093015	0.077548	-0.19747	-0.09898	-0.04429
0.024577	0.151943	7.87E-05	0.503784	0.298282	0.386168	0.026056	0.268233	0.621008
0.040896	-0.22473	-0.19722	-0.26392	-0.25791	-0.31812	-0.52642	-0.32872	-0.44167
0.64802	0.011081	0.026254	0.002716	0.003417	0.000268	2.06E-10	0.000161	2.01E-07
0.724606	0.409883	0.270479	0.623265	0.527064	0.5368	0.273535	0.29691	0.23998
6.02E-22	1.71E-06	0.002101	5.06E-15	1.94E-10	7.74E-11	0.00186	0.0007	0.006577

ZNF398	ZNF416	ZNF43	ZNF433	ZNF454	ZNF473	ZNF486	ZNF491	ZNF530
blue	blue	blue	blue	blue	blue	blue	blue	blue
-0.40622	-0.24018	-0.39273	-0.27183	-0.44579	-0.4228	-0.34661	-0.21783	-0.43957
2.15E-06	0.00653	4.95E-06	0.001991	1.5E-07	7.35E-07	6.55E-05	0.013889	2.34E-07
-0.36663	-0.22279	-0.39559	-0.21781	-0.44667	-0.38021	-0.35214	-0.22251	-0.39772
2.24E-05	0.011814	4.16E-06	0.013898	1.41E-07	1.04E-05	4.91E-05	0.011923	3.65E-06
0.710268	0.650182	0.763147	0.653735	0.687457	0.816331	0.81368	0.645112	0.803152
8.69E-21	1.32E-16	1.84E-25	7.94E-17	4.42E-19	1.38E-31	3.1E-31	2.7E-16	6.78E-30
0.680532	0.541024	0.66822	0.72276	0.6078	0.709217	0.827879	0.603267	0.688006
1.36E-18	5.15E-11	9.28E-18	8.57E-22	3.52E-14	1.05E-20	3.5E-33	6.1E-14	4.04E-19
-0.17901	0.123604	-0.15571	0.205603	-0.2517	-0.04015	0.067194	0.041919	0.103389
0.044039	0.166208	0.080458	0.020398	0.00431	0.654031	0.45289	0.639828	0.24739
0.305757	0.332435	0.305147	0.600942	0.1964	0.375962	0.59889	0.382158	0.438683
0.000473	0.000134	0.000486	8.06E-14	0.026895	1.33E-05	1.03E-13	9.27E-06	2.49E-07
0.176805	0.294034	0.093604	0.441176	0.02837	0.286348	0.259017	0.244559	0.223782
0.046758	0.000793	0.295224	2.09E-07	0.751533	0.0011	0.003277	0.005588	0.011435
-0.05413	-0.14084	-0.11204	-0.09556	0.057518	-0.05796	-0.11004	-0.00185	-0.17485
0.545546	0.114241	0.20981	0.285192	0.520665	0.517444	0.21811	0.98355	0.04928
0.144439	0.249699	0.245821	0.179888	0.157679	0.396622	0.33597	0.319207	0.312905
0.105198	0.004638	0.00534	0.042998	0.076647	3.91E-06	0.000113	0.000254	0.000341
-0.50863	-0.21733	-0.25888	-0.32277	-0.46793	-0.23207	-0.15932	-0.07491	-0.23929
1.03E-09	0.014114	0.003295	0.000215	2.9E-08	0.008655	0.073591	0.402556	0.006739
-0.22906	-0.06377	-0.08173	-0.08347	-0.19332	-0.12995	-0.00656	0.032794	-0.10021
0.009586	0.476276	0.360988	0.350817	0.029429	0.145343	0.941627	0.714352	0.2623
0.032215	0.123874	-0.22859	-0.03738	0.079975	0.000467	-0.15952	0.029445	0.04894
0.719187	0.16528	0.009739	0.676481	0.371431	0.995843	0.073225	0.742446	0.584792
0.224885	0.290391	0.298117	-0.00776	0.341181	0.37833	0.111628	0.270266	0.256235
0.011025	0.000927	0.000664	0.930989	8.66E-05	1.16E-05	0.211499	0.002119	0.00364
-0.33295	0.046814	-0.0057	-0.26419	-0.13992	-0.05562	-0.18406	-0.05075	0.013214
0.000131	0.601229	0.949258	0.002688	0.116668	0.534522	0.038314	0.570982	0.88278
-0.45099	-0.11344	-0.1267	-0.45929	-0.25938	-0.1108	-0.27551	-0.0344	-0.17529
1.03E-07	0.204128	0.155769	5.59E-08	0.003232	0.214909	0.001718	0.701036	0.048709
-0.59254	-0.57369	-0.49207	-0.64382	-0.54056	-0.67002	-0.6772	-0.4377	-0.61352
2.16E-13	1.79E-12	4.22E-09	3.23E-16	5.39E-11	7.05E-18	2.31E-18	2.66E-07	1.74E-14
0.172546	-0.00359	0.070462	-0.10005	0.166216	-0.03938	-0.15018	-0.11622	-0.02326
0.052399	0.968059	0.431167	0.263075	0.06181	0.660267	0.091943	0.193198	0.795159
0.328569	-0.14202	0.102692	0.172908	0.201599	-0.12484	0.079883	-0.19223	-0.03116
0.000162	0.111207	0.250608	0.051899	0.023036	0.16198	0.371983	0.030375	0.728018
-0.07031	-0.38474	-0.3616	-0.28898	-0.16677	-0.47535	-0.48793	-0.51311	-0.44689
0.432192	7.96E-06	2.95E-05	0.000984	0.060933	1.63E-08	5.93E-09	6.91E-10	1.39E-07
0.403604	0.426423	0.212797	0.356035	0.31496	0.431754	0.354858	0.385501	0.404099
2.54E-06	5.77E-07	0.016307	3.99E-05	0.00031	4.02E-07	4.25E-05	7.62E-06	2.46E-06

ZNF547	ZNF548	ZNF549	ZNF551	ZNF555	ZNF566	ZNF620	ZNF649	ZNF662
blue	blue	blue	blue	blue	blue	blue	blue	blue
-0.13793	-0.0862	-0.37807	-0.38185	-0.34887	-0.33943	-0.25577	-0.41809	-0.40013
0.121996	0.335226	1.17E-05	9.44E-06	5.83E-05	9.46E-05	0.003704	1E-06	3.15E-06
-0.07286	-0.09371	-0.41545	-0.45079	-0.3571	-0.30074	-0.25622	-0.40514	-0.36617
0.415641	0.294682	1.19E-06	1.05E-07	3.77E-05	0.000591	0.003642	2.3E-06	2.3E-05
0.634994	0.643989	0.741235	0.789213	0.704698	0.650275	0.60388	0.659385	0.655819
1.08E-15	3.16E-16	2.19E-23	3.05E-28	2.35E-20	1.3E-16	5.67E-14	3.49E-17	5.87E-17
0.634473	0.693711	0.698307	0.732842	0.65183	0.580756	0.550883	0.418426	0.511804
1.16E-15	1.56E-19	7.14E-20	1.2E-22	1.04E-16	8.23E-13	1.94E-11	9.81E-07	7.76E-10
0.078122	-0.12383	-0.36774	-0.25799	-0.14411	-0.22404	-0.21775	-0.16765	-0.30822
0.382652	0.165442	2.11E-05	0.003407	0.10601	0.011339	0.013923	0.059559	0.000423
0.442552	0.388158	0.19793	0.27558	0.259298	0.189503	0.192029	0.074474	0.026656
1.89E-07	6.51E-06	0.025706	0.001713	0.003242	0.032855	0.030555	0.405329	0.766101
0.260777	0.242956	0.097065	0.120179	0.20128	-0.04783	0.073107	0.022066	0.088157
0.003065	0.005918	0.27765	0.178354	0.023259	0.593381	0.41403	0.805495	0.324342
-0.06245	-0.12613	-0.03462	-0.12146	-0.10286	-0.20392	-0.15051	-0.20694	-0.04083
0.485497	0.157661	0.699185	0.173751	0.249842	0.021474	0.091208	0.019574	0.648577
0.165538	0.390404	0.50207	0.409414	0.169171	0.026678	0.139663	0.183169	0.147742
0.062895	5.69E-06	1.81E-09	1.76E-06	0.057258	0.765909	0.117339	0.039277	0.097396
-0.24228	-0.06692	-0.16514	-0.18853	-0.40524	-0.44218	-0.25002	-0.30346	-0.33212
0.006062	0.454772	0.063542	0.03378	2.29E-06	1.94E-07	0.004585	0.000524	0.000136
-0.06637	0.091045	-0.0202	-0.0385	-0.2247	-0.23597	-0.07353	-0.233	-0.16111
0.458491	0.308682	0.82165	0.667348	0.011092	0.007567	0.411332	0.008383	0.070369
0.014915	0.070274	0.13984	0.010951	0.046813	-0.07414	0.088395	0.048887	0.198719
0.867818	0.432403	0.116871	0.902747	0.601233	0.407465	0.32303	0.585195	0.025111
0.147703	0.269851	0.523352	0.444288	0.372142	0.294092	0.35886	0.399292	0.553941
0.097485	0.002154	2.74E-10	1.67E-07	1.65E-05	0.000791	3.43E-05	3.32E-06	1.42E-11
-0.1867	-0.27115	-0.11906	-0.098	-0.0981	-0.07547	-0.08289	0.006561	-0.03929
0.035578	0.002046	0.182475	0.27303	0.272524	0.399056	0.354186	0.941643	0.660969
-0.26327	-0.13181	0.029621	-0.06414	-0.21019	-0.24642	-0.07283	-0.05454	-0.00408
0.002785	0.139637	0.74096	0.473757	0.017698	0.005226	0.415783	0.542545	0.963692
-0.41733	-0.58173	-0.60328	-0.69024	-0.46437	-0.29141	-0.28475	-0.30864	-0.29634
1.05E-06	7.38E-13	6.09E-14	2.79E-19	3.81E-08	0.000887	0.001176	0.000415	0.000717
-0.05589	-0.16355	-0.03568	-0.0189	0.140641	0.239539	0.139555	0.341019	0.279071
0.532571	0.066175	0.690467	0.83298	0.114768	0.00668	0.117623	8.73E-05	0.001487
0.042984	-0.06667	-0.13322	-0.03063	0.095334	0.296427	-0.00043	0.004357	-0.05285
0.631344	0.456406	0.13541	0.732484	0.286347	0.000715	0.996177	0.961225	0.555088
-0.34616	-0.46206	-0.47909	-0.44044	-0.27885	-0.15067	-0.31659	-0.18915	-0.25954
6.71E-05	4.54E-08	1.21E-08	2.2E-07	0.0015	0.090872	0.000288	0.033193	0.003212
0.382252	0.425171	0.427432	0.396912	0.538305	0.381354	0.440285	0.51629	0.522236
9.22E-06	6.27E-07	5.39E-07	3.84E-06	6.7E-11	9.71E-06	2.22E-07	5.2E-10	3.03E-10

ZNF669	ZNF670	ZNF678	ZNF681	ZNF682	ZNF689	ZNF695	ZNF711	ZNF713
blue	blue	blue	blue	blue	blue	blue	blue	blue
-0.16295	-0.18682	-0.36501	-0.2309	-0.20848	-0.2686	-0.2389	-0.28955	-0.36185
0.067177	0.035453	2.45E-05	0.009007	0.018664	0.002263	0.006832	0.000961	2.91E-05
-0.21664	-0.12569	-0.32396	-0.18019	-0.12544	-0.32247	-0.21404	-0.29502	-0.41981
0.014429	0.159107	0.000203	0.042641	0.159964	0.000218	0.015679	0.00076	8.96E-07
0.59767	0.657136	0.759196	0.578523	0.731683	0.60563	0.591753	0.633671	0.607798
1.19E-13	4.85E-17	4.53E-25	1.05E-12	1.51E-22	4.59E-14	2.37E-13	1.29E-15	3.52E-14
0.629899	0.575444	0.756032	0.50656	0.668954	0.437386	0.363549	0.427929	0.540713
2.13E-15	1.48E-12	9.19E-25	1.23E-09	8.3E-18	2.72E-07	2.66E-05	5.21E-07	5.3E-11
0.109239	0.161739	0.090718	0.049835	0.073836	0.10592	0.074021	-0.22866	-0.34569
0.221498	0.069269	0.310427	0.57793	0.40938	0.235939	0.408199	0.009716	6.87E-05
0.49243	0.447295	0.543226	0.339371	0.458403	0.25718	0.199575	0.04871	0.095616
4.09E-09	1.35E-07	4.15E-11	9.49E-05	5.98E-08	0.003513	0.024478	0.586559	0.284918
0.228469	0.129697	0.222487	0.085039	0.263362	0.024933	0.019961	-0.0612	-0.10064
0.00978	0.146139	0.011934	0.341814	0.002775	0.780822	0.82373	0.494267	0.260263
-0.07723	-0.19943	-0.0034	-0.23823	-0.11841	-0.08956	-0.19798	-0.04773	0.02125
0.388109	0.024581	0.969729	0.006995	0.184863	0.316684	0.025667	0.594119	0.812553
0.20109	-0.08951	0.061549	-0.11756	0.058202	0.132485	-0.00235	-0.21075	0.150949
0.023392	0.316967	0.491817	0.188071	0.515713	0.137593	0.979111	0.017393	0.090263
-0.20547	-0.51075	-0.35722	-0.45482	-0.36009	-0.09583	-0.44422	-0.61453	-0.37006
0.020481	8.52E-10	3.74E-05	7.8E-08	3.21E-05	0.283812	1.68E-07	1.53E-14	1.85E-05
-0.02141	-0.29242	-0.08295	-0.25887	-0.08788	0.019111	-0.24945	-0.33116	-0.07389
0.81113	0.00085	0.353871	0.003296	0.325843	0.831122	0.004681	0.000143	0.409066
-0.16277	-0.17605	-0.27923	-0.16337	-0.16598	0.029162	0.073018	-0.16729	0.114314
0.067485	0.04772	0.001477	0.066463	0.062188	0.744832	0.414602	0.060122	0.200644
-0.00292	-0.02098	0.030497	-0.02355	0.116205	0.260706	0.148338	0.27691	0.374856
0.974048	0.814892	0.733578	0.792687	0.193247	0.003073	0.096037	0.001624	1.41E-05
-0.24419	-0.27697	-0.24765	-0.26878	-0.23735	0.13507	-0.10138	-0.18729	-0.05333
0.005663	0.00162	0.004998	0.002247	0.007214	0.13001	0.256762	0.03499	0.55149
-0.3697	-0.57953	-0.44157	-0.52413	-0.37593	-0.00248	-0.32858	-0.39301	-0.11281
1.89E-05	9.43E-13	2.03E-07	2.55E-10	1.33E-05	0.977934	0.000162	4.87E-06	0.206668
-0.62625	-0.43612	-0.53307	-0.32401	-0.38015	-0.37866	-0.35508	-0.2503	-0.37134
3.44E-15	2.97E-07	1.11E-10	0.000202	1.04E-05	1.13E-05	4.2E-05	0.004538	1.72E-05
-0.08928	0.131726	0.052535	0.204856	0.068032	0.02642	0.235026	0.507339	0.228391
0.318195	0.139881	0.557478	0.020869	0.447265	0.76811	0.007819	1.15E-09	0.009805
0.177656	0.426091	0.28058	0.480342	0.200403	-0.14043	0.21697	0.43758	0.133011
0.045693	5.9E-07	0.001398	1.1E-08	0.023879	0.11533	0.014277	2.68E-07	0.136021
-0.24833	-0.01399	-0.27037	0.042291	-0.25631	-0.39525	-0.01316	0.155556	-0.2054
0.004877	0.875939	0.00211	0.636861	0.003629	4.25E-06	0.88325	0.080753	0.020524
0.365467	0.57322	0.321752	0.415424	0.423084	0.359182	0.591375	0.463791	0.213641
2.39E-05	1.88E-12	0.000225	1.19E-06	7.21E-07	3.37E-05	2.47E-13	3.98E-08	0.015877

ZNF726	ZNF730	ZNF738	ZNF749	ZNF77	ZNF773	ZNF782	ZNF786	ZNF8
blue	blue	blue	blue	blue	blue	blue	blue	blue
-0.31026	-0.21918	-0.355	-0.3503	-0.21437	-0.20736	-0.14496	-0.49491	-0.27813
0.000385	0.013292	4.22E-05	5.41E-05	0.015512	0.019323	0.103947	3.33E-09	0.001545
-0.27671	-0.2205	-0.2784	-0.31731	-0.20137	-0.27466	-0.18644	-0.48493	-0.29512
0.001637	0.012734	0.001528	0.000278	0.023193	0.001778	0.035843	7.58E-09	0.000756
0.772203	0.575348	0.748202	0.608918	0.665014	0.599705	0.651254	0.536851	0.588445
2.19E-26	1.49E-12	5.05E-24	3.07E-14	1.51E-17	9.33E-14	1.13E-16	7.7E-11	3.46E-13
0.629294	0.531943	0.484411	0.401449	0.543798	0.555065	0.717545	0.283509	0.346157
2.31E-15	1.23E-10	7.91E-09	2.9E-06	3.92E-11	1.27E-11	2.29E-21	0.001238	6.71E-05
0.182396	-0.04713	-0.12605	-0.27453	0.088418	-0.29851	-0.12614	-0.28557	-0.24468
0.040129	0.598791	0.157911	0.001787	0.322907	0.000653	0.157628	0.001136	0.005564
0.467604	0.32854	0.107374	-0.03962	0.364176	0.135836	0.432151	-0.109	-0.07088
2.98E-08	0.000163	0.229534	0.658334	2.57E-05	0.127827	3.91E-07	0.22252	0.428449
0.198001	0.111478	-0.0429	-0.09841	0.179306	0.145354	0.230628	-0.23991	0.030721
0.025652	0.212119	0.632022	0.270993	0.043688	0.10299	0.009091	0.006593	0.731703
-0.19943	-0.04798	-0.36039	-0.34933	-0.14906	-0.25703	0.14127	-0.32728	-0.28596
0.024587	0.592207	3.16E-05	5.69E-05	0.094404	0.003532	0.113137	0.000173	0.001118
0.090525	0.056286	0.067872	0.276035	0.292233	0.426181	0.208189	0.193217	0.149915
0.311464	0.529654	0.448335	0.001682	0.000857	5.86E-07	0.018835	0.029521	0.092516
-0.34941	-0.22491	-0.4411	-0.39873	-0.13893	-0.09474	-0.15731	-0.40053	-0.37228
5.66E-05	0.011016	2.1E-07	3.43E-06	0.119283	0.289353	0.077341	3.07E-06	1.63E-05
-0.21837	0.01636	-0.35209	-0.27473	-0.00238	0.060172	0.086066	-0.27993	-0.3023
0.013648	0.855147	4.92E-05	0.001773	0.978798	0.501577	0.335993	0.001435	0.000552
-0.27642	-0.03137	0.109788	0.442515	0.059598	0.264475	-0.08547	0.097328	0.037643
0.001656	0.726296	0.219173	1.9E-07	0.505672	0.002658	0.339382	0.276341	0.674358
0.078531	0.075791	0.424383	0.549366	0.232728	0.489234	0.176965	0.477412	0.566146
0.380157	0.397051	6.61E-07	2.26E-11	0.008462	5.33E-09	0.046557	1.39E-08	4.02E-12
-0.14269	-0.25973	0.078009	0.168203	0.012507	-0.07451	-0.38866	0.260829	0.135689
0.109528	0.00319	0.383345	0.058718	0.889007	0.405101	6.32E-06	0.003059	0.128243
-0.40019	-0.28632	-0.12098	0.015362	-0.078	0.075981	-0.25899	0.032509	0.02511
3.14E-06	0.001101	0.175441	0.863895	0.383391	0.395866	0.003281	0.716731	0.779311
-0.55401	-0.39091	-0.23549	-0.45703	-0.47142	-0.48103	-0.50879	-0.43672	-0.3966
1.41E-11	5.52E-06	0.007693	6.62E-08	2.22E-08	1.04E-08	1.01E-09	2.85E-07	3.91E-06
0.082362	0.061197	0.368409	0.253215	-0.04245	0.017119	-0.15026	0.253265	0.287834
0.357279	0.494307	2.03E-05	0.004074	0.635617	0.848504	0.091751	0.004067	0.001033
0.2257	0.214574	0.111549	-0.03097	-0.12027	-0.19594	0.082198	0.069395	-0.06476
0.01073	0.015414	0.211825	0.729607	0.17801	0.027265	0.358243	0.438196	0.469435
-0.21205	-0.16767	-0.19507	-0.19705	-0.43098	-0.4369	-0.36358	-0.10077	-0.1616
0.016696	0.059534	0.027969	0.026383	4.23E-07	2.82E-07	2.65E-05	0.25963	0.06951
0.512679	0.288756	0.557681	0.475484	0.41018	0.40977	0.284418	0.256043	0.536203
7.18E-10	0.000993	9.71E-12	1.61E-08	1.67E-06	1.72E-06	0.001192	0.003666	8.2E-11

ZNF814	ZNF823	ZNF843	ZNF85	ZNF850	ZNF90	ZNF92	ZNF93	ZSCAN2
blue	blue	blue	blue	blue	blue	blue	blue	blue
-0.25495	-0.14102	-0.38009	-0.19318	-0.27944	-0.35632	-0.24568	-0.32551	-0.3079
0.00382	0.113776	1.05E-05	0.029551	0.001464	3.93E-05	0.005368	0.000188	0.000429
-0.29276	-0.12417	-0.36855	-0.16916	-0.27995	-0.33778	-0.2022	-0.3246	-0.32569
0.000837	0.164248	2.01E-05	0.057275	0.001434	0.000103	0.022625	0.000197	0.000187
0.64504	0.581646	0.471564	0.657376	0.782816	0.837983	0.686328	0.88743	0.624718
2.73E-16	7.45E-13	2.19E-08	4.68E-17	1.59E-27	1.11E-34	5.32E-19	7.24E-44	4.2E-15
0.592121	0.624362	0.245999	0.569659	0.698894	0.701705	0.652872	0.705982	0.262261
2.27E-13	4.39E-15	0.005306	2.76E-12	6.46E-20	3.97E-20	8.99E-17	1.87E-20	0.002895
-0.01263	0.261645	-0.00871	0.250007	-0.13765	-0.13159	0.124051	0.049845	-0.0999
0.887967	0.002965	0.922566	0.004586	0.122766	0.140302	0.164669	0.577855	0.263793
0.325554	0.579004	0.049075	0.505525	0.346517	0.331022	0.500709	0.428149	-0.0811
0.000188	9.99E-13	0.583755	1.35E-09	6.58E-05	0.000144	2.04E-09	5.13E-07	0.364705
0.166571	0.431302	-0.14343	0.282344	0.174261	0.080452	0.17131	0.20884	-0.087
0.061248	4.14E-07	0.107687	0.001299	0.050063	0.368577	0.054137	0.018459	0.330749
0.03841	0.006305	-0.10428	-0.15496	-0.21066	-0.27001	-0.1626	-0.28625	-0.42513
0.668118	0.943914	0.243332	0.081934	0.017443	0.002141	0.067779	0.001104	6.29E-07
0.34147	0.261131	-0.10752	0.27442	0.521952	0.413813	0.000394	0.441511	0.315912
8.53E-05	0.003024	0.228883	0.001795	3.11E-10	1.33E-06	0.99649	2.04E-07	0.000297
-0.03361	0.070608	-0.36093	-0.05557	-0.03988	-0.17553	-0.35321	-0.16093	-0.26163
0.707592	0.43021	3.06E-05	0.534943	0.656214	0.048393	4.64E-05	0.070688	0.002966
0.044863	0.079736	-0.23931	-0.08324	0.070624	-0.07274	-0.12843	-0.05689	-0.22956
0.616489	0.372866	0.006735	0.352147	0.430107	0.416363	0.150156	0.525207	0.009426
-0.202	-0.17827	-0.25607	-0.30662	-0.03476	0.017093	-0.28164	-0.02191	0.431601
0.022761	0.044936	0.003663	0.000455	0.698042	0.848734	0.001338	0.80687	4.06E-07
0.312085	0.05193	0.215865	0.063564	0.405957	0.395687	-0.03216	0.328242	0.606887
0.000354	0.562031	0.014792	0.477722	2.19E-06	4.14E-06	0.719614	0.000165	3.94E-14
0.063652	-0.12436	0.273257	-0.06894	-0.01092	-0.01497	-0.17026	-0.02712	0.320075
0.477117	0.16361	0.001881	0.441181	0.902984	0.867308	0.055656	0.76213	0.000244
0.039645	-0.11235	-0.08045	-0.15385	0.068579	-0.02952	-0.44456	-0.0997	0.179301
0.658105	0.20856	0.368587	0.084166	0.44361	0.74184	1.64E-07	0.264763	0.043694
-0.54397	-0.45959	-0.26003	-0.42338	-0.60967	-0.56786	-0.43794	-0.68433	-0.43509
3.86E-11	5.47E-08	0.003153	7.07E-07	2.8E-14	3.35E-12	2.62E-07	7.37E-19	3.19E-07
-0.13672	-0.28187	0.299958	-0.13465	-0.15237	-0.0198	0.097474	-0.04652	0.233828
0.125345	0.001325	0.000612	0.131218	0.087229	0.825108	0.275617	0.603534	0.008149
-0.18482	-0.20436	0.14671	-0.06357	-0.22961	-0.09387	0.358148	-0.12199	-0.24768
0.037511	0.021187	0.099783	0.477702	0.00941	0.293846	3.56E-05	0.171848	0.004993
-0.52155	-0.56056	-0.07326	-0.44949	-0.62929	-0.52098	-0.18174	-0.52408	-0.30165
3.23E-10	7.22E-12	0.413062	1.15E-07	2.31E-15	3.4E-10	0.040866	2.56E-10	0.000568
0.272129	0.326247	0.105912	0.422136	0.37873	0.487417	0.271515	0.527425	0.66288
0.001968	0.000182	0.235977	7.68E-07	1.13E-05	6.19E-09	0.002017	1.88E-10	2.08E-17

ZSWIM1	ZW10	ZWILCH	ZWINT	A2M	AADAT	ABCA13	ABCC3	ABCC6
blue	blue	blue	blue	brown	brown	brown	brown	brown
-0.1558	-0.05733	-0.25981	-0.44369	0.160736	-0.17668	-0.08615	-0.04174	-0.09473
0.080278	0.522054	0.00318	1.75E-07	0.071035	0.046916	0.335512	0.641249	0.2894
-0.1197	-0.06365	-0.20839	-0.43654	0.015928	-0.13897	-0.15807	-0.1175	-0.08748
0.180112	0.477129	0.018718	2.89E-07	0.858935	0.119196	0.07591	0.188304	0.328101
0.575898	0.633317	0.789644	0.893218	-0.02317	0.495122	-0.02933	0.092617	-0.17645
1.41E-12	1.35E-15	2.73E-28	3.21E-45	0.795972	3.27E-09	0.743382	0.300368	0.047208
0.483105	0.717728	0.598692	0.522395	0.154718	0.15195	0.276364	0.42102	-0.09553
8.79E-09	2.21E-21	1.05E-13	2.99E-10	0.082418	0.088124	0.00166	8.27E-07	0.285374
0.101138	0.03896	0.054897	-0.04356	-0.18439	-0.24401	-0.10575	0.060472	0.075937
0.257888	0.66365	0.539875	0.62678	0.037958	0.0057	0.236716	0.499443	0.39614
0.303369	0.573424	0.37881	0.200664	0.103362	-0.21168	0.277734	0.471273	0.01483
0.000526	1.84E-12	1.13E-05	0.023693	0.247512	0.01689	0.00157	2.24E-08	0.868567
0.215008	0.298549	0.183836	-0.07444	0.13959	-0.25767	0.169788	0.331827	0.049393
0.015202	0.000651	0.038555	0.405567	0.11753	0.003449	0.056342	0.000138	0.581317
-0.14105	-0.04994	-0.25216	-0.19065	0.253596	-0.39245	0.379026	0.34224	0.401346
0.113709	0.57713	0.004236	0.03179	0.004017	5.03E-06	1.11E-05	8.21E-05	2.92E-06
0.087002	0.395487	0.269016	0.292213	0.643612	-0.17198	0.450424	0.641095	0.039218
0.330747	4.19E-06	0.002226	0.000857	3.33E-16	0.053188	1.08E-07	4.71E-16	0.661557
-0.31566	-0.00733	-0.32959	-0.27553	0.72851	-0.5821	0.528831	0.649896	0.472892
0.0003	0.934813	0.000154	0.001717	2.83E-22	7.08E-13	1.65E-10	1.38E-16	1.98E-08
-0.11824	0.106327	-0.22385	-0.19217	0.544574	-0.44167	0.473196	0.549834	0.306855
0.185499	0.234135	0.011409	0.030427	3.63E-11	2.01E-07	1.93E-08	2.15E-11	0.00045
0.078774	-0.12979	-0.05964	-0.11433	-0.01306	0.275385	0.003227	-0.00431	-0.07661
0.378681	0.145835	0.505398	0.200587	0.884138	0.001727	0.97128	0.961664	0.391972
0.212096	0.037184	0.167561	0.412477	0.127735	0.44031	-0.05786	-0.11071	0.017964
0.016672	0.678109	0.059703	1.44E-06	0.152391	2.22E-07	0.518188	0.215324	0.841123
-0.06209	-0.43143	-0.23647	0.119166	-0.08228	0.067184	-0.1602	-0.17821	0.267874
0.48804	4.11E-07	0.007438	0.182067	0.357747	0.452963	0.071998	0.04501	0.002329
-0.23996	-0.27285	-0.35943	-0.04118	0.514385	-0.15915	0.252874	0.231945	0.434584
0.006581	0.001912	3.32E-05	0.64574	6.17E-10	0.0739	0.004126	0.008692	3.31E-07
-0.50346	-0.56617	-0.58717	-0.54985	-0.11564	-0.01143	-0.27751	-0.45688	0.122281
1.61E-09	4E-12	4E-13	2.15E-11	0.195434	0.898542	0.001584	6.69E-08	0.170824
-0.00642	-0.33302	0.057022	0.176313	-0.55053	0.599948	-0.604	-0.72067	-0.29019
0.942873	0.00013	0.524277	0.047382	2.01E-11	9.07E-14	5.58E-14	1.27E-21	0.000935
0.053932	0.008813	0.180693	-0.05574	-0.617	0.229916	-0.37625	-0.47808	-0.48107
0.547037	0.921666	0.042058	0.533646	1.13E-14	0.009313	1.3E-05	1.31E-08	1.03E-08
-0.2304	-0.43143	-0.18657	-0.34277	-0.60972	0.176959	-0.50441	-0.68686	-0.37252
0.009161	4.11E-07	0.035712	7.98E-05	2.78E-14	0.046563	1.48E-09	4.87E-19	1.61E-05
0.428718	0.377099	0.638654	0.513668	-0.21922	0.593419	-0.45508	-0.40198	-0.3467
4.94E-07	1.24E-05	6.58E-16	6.58E-10	0.013276	1.95E-13	7.65E-08	2.81E-06	6.52E-05

ABI2	ABI3	ABI3BP	ACAA2	ACACA	ACAP1	ACP2	ACP5	ACPP
brown	brown	brown	brown	brown	brown	brown	brown	brown
-0.29937	0.0345	0.298842	0.183599	-0.22048	0.226326	0.233471	0.416375	-0.01625
0.000628	0.700186	0.000643	0.03881	0.012743	0.010509	0.00825	1.12E-06	0.85614
-0.24542	-0.11789	0.187285	0.168039	-0.16834	0.075123	0.197488	0.303911	-0.07734
0.005419	0.186838	0.034995	0.058968	0.058514	0.40124	0.026045	0.000514	0.387429
0.319797	-0.17239	-0.29674	-0.29228	0.175687	-0.34941	-0.21484	-0.2451	0.087767
0.000247	0.052609	0.000705	0.000855	0.048187	5.66E-05	0.015283	0.005481	0.326495
0.29671	-0.23982	-0.05638	-0.10193	-0.11527	-0.15139	-0.18164	-0.03995	0.36066
0.000706	0.006614	0.528958	0.254173	0.196894	0.08932	0.040979	0.655601	3.11E-05
-0.40987	-0.00142	-0.0528	0.362638	-0.54441	0.293837	0.03758	0.228155	0.124964
1.71E-06	0.987385	0.555498	2.79E-05	3.7E-11	0.000799	0.674873	0.009883	0.161561
-0.08234	-0.17719	0.073707	0.28297	-0.55274	0.178739	-0.04528	0.22989	0.47278
0.357385	0.046273	0.410196	0.001266	1.61E-11	0.044369	0.613194	0.009322	1.99E-08
0.013404	-0.21514	0.035312	0.086842	-0.27761	0.099002	0.074102	0.129609	0.361277
0.881103	0.015138	0.693483	0.331636	0.001578	0.268124	0.407691	0.146414	3.01E-05
-0.10898	0.042534	0.274225	0.415308	-0.23365	0.233415	-0.1482	0.167406	0.126262
0.222621	0.63492	0.001809	1.2E-06	0.0082	0.008266	0.096342	0.059943	0.157215
-0.23369	0.313019	0.304066	0.164612	-0.22945	0.304834	0.432613	0.336863	0.518351
0.008187	0.000339	0.00051	0.064404	0.009462	0.000493	3.79E-07	0.000108	4.32E-10
-0.7296	0.741201	0.70652	0.627364	-0.62999	0.793622	0.624861	0.778183	0.554486
2.28E-22	2.2E-23	1.7E-20	2.97E-15	2.1E-15	9.46E-29	4.12E-15	5.1E-27	1.35E-11
-0.36336	0.480339	0.558253	0.373204	-0.48217	0.535212	0.438117	0.565382	0.447408
2.68E-05	1.1E-08	9.16E-12	1.55E-05	9.47E-09	9.01E-11	2.59E-07	4.35E-12	1.34E-07
0.088628	0.052328	-0.02603	-0.23174	0.37231	-0.18031	0.268627	-0.1334	-0.15391
0.321753	0.559033	0.77141	0.008752	1.63E-05	0.042509	0.002261	0.134862	0.084052
0.158035	0.188654	-0.08459	-0.37023	0.510037	-0.25018	0.037899	-0.17243	-0.20506
0.075976	0.033661	0.344351	1.83E-05	9.07E-10	0.004557	0.67227	0.052557	0.020738
-0.38901	0.467193	-0.06282	0.039331	-0.01211	0.149455	0.134561	0.077864	-0.23896
6.19E-06	3.07E-08	0.482904	0.660643	0.892513	0.093532	0.131477	0.384231	0.006817
-0.53094	0.762355	0.410455	0.253827	-0.07167	0.413651	0.474496	0.435871	0.083292
1.35E-10	2.21E-25	1.64E-06	0.003983	0.423309	1.34E-06	1.74E-08	3.02E-07	0.351861
-0.15653	0.17981	0.01835	0.083786	0.170125	-0.02773	0.010454	0.045861	-0.43556
0.078856	0.04309	0.837755	0.349	0.055848	0.756957	0.907143	0.608661	3.09E-07
0.526136	-0.24993	-0.56404	-0.58208	0.706318	-0.64979	-0.39411	-0.64718	-0.65316
2.12E-10	0.0046	5.01E-12	7.1E-13	1.76E-20	1.4E-16	4.55E-06	2.02E-16	8.62E-17
0.659114	-0.71957	-0.4722	-0.42545	0.277598	-0.51932	-0.54075	-0.57325	-0.3054
3.63E-17	1.57E-21	2.09E-08	6.16E-07	0.001579	3.96E-10	5.28E-11	1.88E-12	0.00048
0.442791	-0.49486	-0.39911	-0.37885	0.461647	-0.43954	-0.35443	-0.56284	-0.50452
1.86E-07	3.34E-09	3.35E-06	1.12E-05	4.69E-08	2.34E-07	4.35E-05	5.69E-12	1.47E-09
0.245478	-0.26562	-0.43233	-0.50974	0.315635	-0.36821	-0.26536	-0.28415	-0.23919
0.005407	0.002543	3.86E-07	9.32E-10	0.000301	2.05E-05	0.002568	0.001206	0.006762

ACRV1	ACSF2	ACSL1	ACSL3	ACSL5	ACSM5	ACVR2A	ACVR2B	ADA
brown	brown	brown	brown	brown	brown	brown	brown	brown
0.019009	0.230897	0.235375	-0.27934	0.278334	0.272227	-0.11426	-0.16285	0.178107
0.832009	0.009008	0.007725	0.00147	0.001532	0.00196	0.200849	0.067361	0.045139
0.136447	0.215602	0.215631	-0.25332	0.211997	0.239821	-0.09466	-0.12671	0.160677
0.126105	0.014917	0.014903	0.004058	0.016723	0.006614	0.289775	0.155742	0.07114
0.086544	-0.3902	-0.51254	0.326112	-0.48073	-0.56881	0.341905	0.351896	-0.08039
0.333309	5.76E-06	7.27E-10	0.000183	1.06E-08	3.03E-12	8.35E-05	4.97E-05	0.36893
-0.21221	-0.07329	-0.25419	0.408951	-0.11777	-0.47573	0.231391	0.120958	0.115238
0.016609	0.412861	0.00393	1.81E-06	0.187276	1.58E-08	0.008858	0.175537	0.197008
0.10401	0.45831	-0.05203	-0.40625	0.186535	0.159268	0.03104	-0.35849	0.276347
0.244544	6.02E-08	0.561289	2.15E-06	0.035744	0.073687	0.729023	3.5E-05	0.001661
-0.19307	0.348754	-0.04267	0.058402	0.246688	-0.18158	0.149897	-0.26316	0.350886
0.029643	5.86E-05	0.63388	0.514265	0.005176	0.041048	0.092556	0.002797	5.24E-05
-0.06706	0.271634	0.049305	0.101358	0.250244	-0.07273	0.03915	-0.19014	0.385708
0.453805	0.002007	0.581992	0.256849	0.004547	0.416438	0.662106	0.032265	7.52E-06
-0.11953	0.240376	0.077522	-0.16223	0.316416	0.237426	-0.24441	-0.17385	-0.14701
0.180725	0.006486	0.386326	0.068416	0.00029	0.007194	0.005619	0.050614	0.099094
-0.49265	-0.01691	0.262569	0.053855	0.128339	-0.33197	-0.1192	-0.20074	0.33768
4.02E-09	0.850302	0.002861	0.547608	0.150446	0.000137	0.181947	0.023639	0.000103
-0.46518	0.588682	0.695943	-0.48112	0.688403	0.441663	-0.54147	-0.58316	0.556695
3.59E-08	3.37E-13	1.07E-19	1.03E-08	3.78E-19	2.02E-07	4.93E-11	6.29E-13	1.07E-11
-0.44374	0.480134	0.339287	-0.23607	0.553966	0.226191	-0.38929	-0.4329	0.363185
1.74E-07	1.12E-08	9.53E-05	0.00754	1.42E-11	0.010556	6.08E-06	3.71E-07	2.71E-05
-0.07717	-0.13146	-0.06071	-0.1016	-0.28457	-0.07861	-0.08035	0.101981	0.01629
0.38848	0.140679	0.497764	0.255719	0.001185	0.379674	0.369157	0.253918	0.855759
0.032095	-0.47593	-0.20033	0.137084	-0.50094	-0.23589	-0.05151	0.430016	-0.22104
0.720189	1.56E-08	0.023929	0.124331	2E-09	0.00759	0.565221	4.52E-07	0.012514
-0.01005	-0.00707	-0.07605	-0.31312	-0.30764	0.07371	-0.27084	-0.04386	-0.09709
0.910733	0.937104	0.395433	0.000338	0.000434	0.410178	0.002071	0.624407	0.277548
-0.28583	0.101609	0.38043	-0.37071	0.108811	0.256151	-0.5303	-0.14683	0.198927
0.001124	0.255667	1.02E-05	1.78E-05	0.223324	0.003652	1.44E-10	0.099513	0.024956
0.254262	0.025245	0.269804	-0.32145	0.20569	0.574245	-0.3218	0.056813	0.067652
0.003919	0.778149	0.002158	0.000229	0.020343	1.68E-12	0.000225	0.525801	0.449816
0.527958	-0.57316	-0.49277	0.188826	-0.57991	-0.06017	0.201756	0.518035	-0.5117
1.79E-10	1.9E-12	3.98E-09	0.033496	9.04E-13	0.501603	0.022928	4.45E-10	7.83E-10
0.317485	-0.26637	-0.34854	0.501188	-0.19816	-0.20451	0.507096	0.275668	-0.38254
0.000276	0.002469	5.93E-05	1.96E-09	0.02553	0.02109	1.17E-09	0.001707	9.06E-06
0.456534	-0.27139	-0.18875	0.173761	-0.15501	0.096475	0.293493	0.220649	-0.47094
6.87E-08	0.002026	0.033568	0.050735	0.081832	0.280593	0.000811	0.012674	2.3E-08
0.488374	-0.50581	-0.36386	0.082824	-0.50074	-0.29349	0.515542	0.580426	-0.03979
5.72E-09	1.31E-09	2.61E-05	0.354578	2.03E-09	0.000812	5.56E-10	8.54E-13	0.656898

ADAM28	ADAMDEC	ADAMTS3	ADAP2	ADCK2	ADGRE1	ADGRE2	ADIPOR1	ADO
brown	brown	brown	brown	brown	brown	brown	brown	brown
0.287092	0.328429	-0.12476	0.307365	0.26069	0.181038	0.082695	0.360247	0.117759
0.001066	0.000163	0.162258	0.00044	0.003075	0.041662	0.355332	3.18E-05	0.187322
0.139171	0.204853	-0.07972	0.131919	0.238306	0.035279	-0.02775	0.364428	0.217431
0.118648	0.020871	0.372989	0.139295	0.006976	0.693755	0.75676	2.53E-05	0.014067
-0.43934	-0.01536	0.463769	-0.21608	-0.54495	-0.04121	0.028639	-0.51657	0.099268
2.37E-07	0.863927	3.99E-08	0.014692	3.5E-11	0.645498	0.749254	5.07E-10	0.266835
-0.1308	0.141707	0.46514	-0.0103	-0.48217	0.082794	0.207654	-0.20679	-0.04985
0.142719	0.112015	3.6E-08	0.908527	9.48E-09	0.354754	0.01915	0.019668	0.577785
0.026564	0.2018	0.04331	0.036434	0.217803	-0.00523	-0.07998	0.219078	0.13681
0.766885	0.022897	0.628758	0.684257	0.013899	0.953456	0.371408	0.013338	0.125091
0.093918	0.316533	0.331937	0.133787	-0.13788	0.134129	0.2154	0.164563	-0.00497
0.293598	0.000288	0.000138	0.133732	0.122129	0.132731	0.015014	0.064484	0.955759
0.016196	0.163655	0.112666	0.08776	-0.12791	0.104415	0.136233	0.298221	-0.14457
0.856586	0.065992	0.207256	0.32653	0.151813	0.242702	0.126708	0.000661	0.104876
0.331522	0.253748	-0.10667	0.246294	6.97E-05	0.172363	0.12967	0.323108	-0.3062
0.00014	0.003995	0.232602	0.00525	0.999379	0.052652	0.146224	0.000211	0.000463
0.048439	0.398096	-0.01489	0.456516	-0.06069	0.499152	0.708288	0.204134	-0.42835
0.588644	3.57E-06	0.868024	6.88E-08	0.497907	2.33E-09	1.24E-20	0.021334	5.06E-07
0.788887	0.68901	-0.39374	0.914422	0.523362	0.736421	0.753245	0.650504	-0.29884
3.33E-28	3.42E-19	4.66E-06	6.18E-51	2.74E-10	5.86E-23	1.7E-24	1.26E-16	0.000643
0.590255	0.543364	-0.15593	0.69247	0.368546	0.536626	0.550426	0.40094	-0.31054
2.81E-13	4.09E-11	0.080024	1.92E-19	2.01E-05	7.87E-11	2.03E-11	2.99E-06	0.00038
-0.26067	-0.2584	-0.10469	-0.06387	0.021557	-0.01185	0.058001	-0.07764	-0.1581
0.003078	0.003355	0.241479	0.47562	0.809892	0.894766	0.517165	0.385604	0.075852
-0.26697	-0.12395	-0.04282	-0.04149	-0.24462	0.041149	0.047015	-0.36967	-0.06934
0.002413	0.165031	0.632626	0.643227	0.005575	0.645991	0.599663	1.89E-05	0.438542
-0.09001	0.030751	-0.18571	0.037175	0.36691	0.044624	-0.04235	-0.15232	0.021221
0.314223	0.73145	0.036583	0.678184	2.21E-05	0.618369	0.636425	0.08735	0.812805
0.365522	0.332154	-0.45313	0.583274	0.399861	0.471463	0.460136	0.234722	-0.21972
2.38E-05	0.000136	8.83E-08	6.21E-13	3.2E-06	2.21E-08	5.25E-08	0.007901	0.013064
0.326076	-0.10969	-0.42379	0.083611	0.374981	-0.16516	-0.32601	0.186043	0.436514
0.000183	0.219574	6.88E-07	0.350012	1.4E-05	0.063504	0.000184	0.036243	2.89E-07
-0.48725	-0.588	0.065472	-0.58781	-0.25074	-0.5007	-0.64568	-0.595	0.371152
6.27E-09	3.64E-13	0.464583	3.72E-13	0.004464	2.04E-09	2.49E-16	1.62E-13	1.74E-05
-0.35165	-0.48692	0.407151	-0.6987	-0.32745	-0.56867	-0.59198	-0.40423	0.29283
5.03E-05	6.45E-09	2.03E-06	6.68E-20	0.000171	3.07E-12	2.31E-13	2.44E-06	0.000835
-0.30494	-0.58934	-0.00435	-0.62401	-0.07299	-0.55723	-0.65518	-0.23352	0.211493
0.00049	3.13E-13	0.961245	4.6E-15	0.414766	1.02E-11	6.44E-17	0.008236	0.016991
-0.48206	-0.16351	0.210332	-0.31804	-0.57581	-0.21922	-0.18821	-0.44048	0.369189
9.56E-09	0.066239	0.017621	0.000269	1.42E-12	0.013278	0.034087	2.19E-07	1.94E-05

ADORA3	ADPGK	ADPRH	ADRB2	AFF3	AHR	AHRR	AIF1	AIM2
brown	brown	brown	brown	brown	brown	brown	brown	brown
0.205873	0.120965	0.209361	0.225514	-0.15551	0.223755	-0.13667	0.379478	0.307882
0.02023	0.175512	0.018162	0.010797	0.080849	0.011445	0.125489	1.08E-05	0.000429
0.187081	0.029005	0.091225	0.13722	-0.10742	0.108852	-0.11357	0.265973	0.204998
0.035198	0.746159	0.307721	0.123955	0.229324	0.22315	0.203602	0.002508	0.020779
-0.30696	0.038413	-0.04515	-0.34879	0.278425	0.141164	0.12066	-0.42107	-0.14737
0.000448	0.668091	0.614199	5.85E-05	0.001527	0.113411	0.176609	8.24E-07	0.09824
-0.05413	0.258614	0.297462	-0.16722	0.096508	0.457121	0.083568	-0.1669	0.006787
0.545537	0.003328	0.000683	0.060238	0.280428	6.57E-08	0.350257	0.060735	0.939637
0.256226	0.175639	0.015827	0.069347	-0.17986	0.083595	-0.45754	0.340625	0.392656
0.003641	0.04825	0.859816	0.438514	0.043029	0.350103	6.37E-08	8.91E-05	4.97E-06
0.258219	0.388715	0.391891	0.031816	-0.12028	0.515597	-0.25122	0.242749	0.354844
0.003378	6.3E-06	5.21E-06	0.722524	0.177991	5.54E-10	0.004387	0.005962	4.25E-05
0.134604	0.25749	0.335936	-0.01181	-0.14786	0.33783	-0.05984	0.212719	0.162816
0.131353	0.003472	0.000113	0.895131	0.097118	0.000103	0.503977	0.016347	0.067411
0.299154	0.208046	0.270959	0.211502	-0.23708	0.025672	-0.28666	0.313332	0.360219
0.000634	0.018919	0.002062	0.016986	0.00728	0.774501	0.001085	0.000335	3.18E-05
0.133794	0.517623	0.607142	-0.15968	-0.24226	0.485771	0.04845	0.208609	0.098269
0.133713	4.61E-10	3.82E-14	0.072937	0.006066	7.08E-09	0.588562	0.018592	0.271703
0.774684	0.767184	0.835099	0.522531	-0.68927	0.413276	-0.44477	0.901451	0.595681
1.2E-26	7.22E-26	3.05E-34	2.95E-10	3.28E-19	1.37E-06	1.62E-07	2.77E-47	1.5E-13
0.484778	0.538233	0.724233	0.410853	-0.39297	0.405476	-0.26696	0.610594	0.512394
7.67E-09	6.74E-11	6.47E-22	1.6E-06	4.88E-06	2.26E-06	0.002414	2.5E-14	7.37E-10
-0.21954	-0.11515	-0.09258	-0.26365	0.099019	-0.17864	0.155155	-0.22945	-0.32034
0.013139	0.197338	0.300579	0.002744	0.268043	0.044484	0.081547	0.009463	0.000241
-0.31697	-0.08265	-0.1248	-0.18442	0.09348	-0.26602	0.30217	-0.37922	-0.35385
0.000283	0.355609	0.16213	0.03793	0.295865	0.002504	0.000555	1.1E-05	4.48E-05
-0.04476	-0.05952	-0.22232	0.020875	-0.18137	-0.40458	-0.0522	-0.07666	-0.08473
0.617307	0.506236	0.011998	0.815802	0.041279	2.39E-06	0.559979	0.391618	0.343556
0.315731	0.345637	0.366473	0.284592	-0.48023	-0.1052	-0.10885	0.346684	0.103061
0.000299	6.89E-05	2.26E-05	0.001184	1.11E-08	0.239179	0.223159	6.53E-05	0.248898
0.263927	-0.28123	-0.17133	0.326074	-0.15778	-0.31977	-0.17007	0.189294	-0.0985
0.002715	0.001361	0.054102	0.000183	0.076457	0.000248	0.055931	0.033052	0.270566
-0.53753	-0.70845	-0.73404	-0.30422	0.547411	-0.50304	0.262137	-0.6372	-0.53879
7.22E-11	1.21E-20	9.47E-23	0.000507	2.74E-11	1.67E-09	0.002909	8.02E-16	6.39E-11
-0.43364	-0.56285	-0.56451	-0.25725	0.571839	-0.0501	0.27454	-0.52356	-0.30772
3.53E-07	5.68E-12	4.77E-12	0.003504	2.19E-12	0.575909	0.001787	2.69E-10	0.000433
-0.41447	-0.67666	-0.69925	-0.18835	0.492848	-0.41113	0.227852	-0.38537	-0.32112
1.27E-06	2.51E-18	6.07E-20	0.033955	3.95E-09	1.58E-06	0.009985	7.67E-06	0.000232
-0.43645	-0.08009	-0.37576	-0.38418	0.319954	0.063831	0.175822	-0.38951	-0.17072
2.91E-07	0.37075	1.34E-05	8.23E-06	0.000245	0.475877	0.048013	6E-06	0.054987

AKAP7	AKIP1	AKNA	AKR1A1	AKR1B1	ALDH3B1	ALOX15B	ALOX5	ALOX5AP
brown	brown	brown	brown	brown	brown	brown	brown	brown
-0.17768	-0.03182	0.200129	0.336461	0.221046	0.172648	0.063895	0.211791	0.086946
0.045664	0.722468	0.024076	0.00011	0.012511	0.052257	0.475431	0.016832	0.331057
-0.10559	-0.08728	0.045301	0.264738	0.19695	0.072188	0.132109	0.105095	0.033776
0.237434	0.329221	0.613047	0.002631	0.026463	0.419938	0.138721	0.239629	0.706191
0.449318	-0.01721	-0.32146	-0.19271	-0.32774	-0.22987	-0.38863	-0.40715	-0.15147
1.17E-07	0.847683	0.000229	0.029954	0.000169	0.009327	6.33E-06	2.03E-06	0.08915
0.431436	0.095842	-0.26313	-0.04093	-0.32033	-0.08645	-0.32453	-0.15869	0.145955
4.1E-07	0.283776	0.0028	0.647717	0.000241	0.333809	0.000197	0.074748	0.101558
0.241378	0.578796	-0.19627	0.653443	0.41466	0.057667	0.117312	0.073565	0.295512
0.00626	1.02E-12	0.027001	8.28E-17	1.25E-06	0.519582	0.18901	0.411101	0.000744
0.402751	0.467117	-0.24585	0.435209	0.053566	0.067178	-0.10289	0.079806	0.434972
2.68E-06	3.09E-08	0.005334	3.17E-07	0.549757	0.452999	0.249702	0.372449	3.22E-07
0.233996	0.202535	-0.16078	0.317807	0.105409	0.105734	-0.10009	0.063065	0.339061
0.008102	0.022395	0.070964	0.000272	0.238222	0.236769	0.262878	0.481195	9.64E-05
-0.15643	0.249871	0.316725	0.134096	0.185167	0.140839	0.117639	0.234957	0.303184
0.079051	0.004609	0.000286	0.132828	0.037146	0.114251	0.187772	0.007838	0.000531
-0.24621	0.141109	0.230858	0.132121	0.003547	0.449924	0.113436	0.350902	0.44718
0.005265	0.113553	0.00902	0.138686	0.968428	1.12E-07	0.204146	5.24E-05	1.36E-07
-0.51004	0.413722	0.665165	0.678931	0.61592	0.859996	0.479433	0.914466	0.813041
9.07E-10	1.33E-06	1.47E-17	1.75E-18	1.29E-14	2.48E-38	1.18E-08	5.99E-51	3.76E-31
-0.2831	0.372444	0.483869	0.51148	0.242056	0.51847	0.055745	0.620537	0.563475
0.001259	1.62E-05	8.26E-09	7.99E-10	0.006111	4.27E-10	0.533624	7.18E-15	5.32E-12
-0.23596	-0.29528	0.043242	-0.1678	-0.19601	-0.08528	-0.01741	-0.04976	-0.23415
0.00757	0.000751	0.629296	0.059328	0.027205	0.340447	0.845965	0.578519	0.008059
-0.1761	-0.34817	0.153122	-0.41841	-0.23432	0.024922	-0.08336	-0.15853	-0.30487
0.047649	6.04E-05	0.085671	9.83E-07	0.008012	0.780918	0.351481	0.075059	0.000492
-0.29323	0.215063	0.155712	0.068252	0.171955	0.209502	0.211713	0.065443	-0.08127
0.000821	0.015176	0.080443	0.445792	0.053224	0.018083	0.016874	0.464782	0.363738
-0.63371	0.09093	0.622131	0.174118	0.324853	0.657358	0.400178	0.544445	0.286556
1.28E-15	0.309296	5.86E-15	0.050255	0.000194	4.69E-17	3.14E-06	3.68E-11	0.00109
-0.24791	-0.22152	0.177898	0.01995	0.322683	0.045769	0.293225	0.103328	-0.14416
0.004951	0.012318	0.045395	0.823831	0.000216	0.609379	0.000821	0.247666	0.10589
0.218508	-0.49152	-0.30649	-0.54958	-0.29889	-0.6033	-0.32273	-0.63205	-0.73722
0.013586	4.41E-09	0.000457	2.21E-11	0.000642	6.07E-14	0.000215	1.6E-15	4.99E-23
0.533835	-0.3272	-0.56692	-0.45839	-0.44732	-0.74377	-0.39547	-0.61635	-0.52323
1.03E-10	0.000173	3.7E-12	5.98E-08	1.35E-07	1.29E-23	4.19E-06	1.22E-14	2.77E-10
0.149168	-0.44341	-0.30833	-0.43841	-0.19691	-0.63111	-0.18299	-0.4669	-0.58173
0.094172	1.78E-07	0.000421	2.53E-07	0.026496	1.81E-15	0.039469	3.14E-08	7.38E-13
0.433373	-0.25925	-0.27014	-0.21866	-0.14953	-0.24833	-0.19673	-0.49874	-0.39922
3.59E-07	0.003248	0.002129	0.01352	0.09336	0.004876	0.026635	2.41E-09	3.33E-06

ALPK1	AMIGO2	AMPD3	ANAPC1	ANGEL2	ANGPT1	ANKRD1	ANKRD13/	ANKRD16
brown	brown	brown	brown	brown	brown	brown	brown	brown
0.249711	0.332565	0.447641	-0.11043	-0.15971	-0.01348	0.189742	0.182893	-0.00233
0.004636	0.000133	1.32E-07	0.216458	0.072875	0.880477	0.032631	0.039579	0.979255
0.123321	0.230268	0.433055	-0.05709	-0.07028	-0.08105	0.187459	0.114343	0.116483
0.167189	0.009203	3.67E-07	0.523766	0.43236	0.365026	0.034823	0.200531	0.192179
-0.24522	-0.44595	-0.60713	0.457473	0.311054	-0.1156	-0.09817	-0.06749	0.101953
0.005457	1.49E-07	3.82E-14	6.4E-08	0.000372	0.195579	0.272205	0.450871	0.254049
0.197595	-0.14957	-0.24636	0.513183	0.507709	0.256643	0.142347	0.120325	-0.16956
0.025963	0.093273	0.005237	6.87E-10	1.11E-09	0.003585	0.11039	0.177824	0.056677
-0.05952	0.05451	0.136035	-0.31516	-0.14147	0.067522	0.264306	0.025478	0.265174
0.506215	0.542736	0.127264	0.000307	0.112625	0.450685	0.002676	0.776161	0.002587
0.313872	0.121082	0.103777	0.166604	0.32587	0.401767	0.37383	0.182417	-0.10503
0.000326	0.175092	0.245609	0.061196	0.000185	2.84E-06	1.5E-05	0.040105	0.239927
0.248142	0.120352	0.183442	0.206669	0.146686	0.144691	0.402629	0.190476	-0.06858
0.00491	0.177725	0.03898	0.019741	0.099839	0.104585	2.7E-06	0.031952	0.443597
0.445368	0.536738	0.31964	-0.16026	0.15858	0.432051	0.105101	0.157169	-0.32381
1.55E-07	7.79E-11	0.000249	0.071887	0.074959	3.94E-07	0.239606	0.077618	0.000204
0.405319	0.099109	0.063142	0.222937	-0.20822	0.248969	0.441609	0.205829	-0.48486
2.28E-06	0.267607	0.480659	0.011758	0.018817	0.004764	2.02E-07	0.020257	7.63E-09
0.835675	0.581569	0.71298	-0.4034	-0.51563	0.42721	0.481403	0.548595	-0.60036
2.5E-34	7.52E-13	5.31E-21	2.57E-06	5.52E-10	5.47E-07	1.01E-08	2.44E-11	8.63E-14
0.746294	0.454594	0.440275	-0.11482	-0.22004	0.473663	0.271068	0.350387	-0.55023
7.59E-24	7.93E-08	2.22E-07	0.19864	0.012929	1.86E-08	0.002053	5.38E-05	2.07E-11
-0.1326	-0.39114	-0.13182	0.347738	-0.20691	-0.23407	-0.10874	-0.09974	0.118276
0.137234	5.45E-06	0.13959	6.18E-05	0.019595	0.008082	0.223644	0.264562	0.185379
-0.18854	-0.35677	-0.39064	0.201538	-0.14849	-0.35049	-0.26481	-0.00018	0.004962
0.033767	3.84E-05	5.61E-06	0.023079	0.095685	5.35E-05	0.002624	0.998419	0.955844
-0.24453	-0.15522	-0.17592	-0.46627	-0.53091	-0.16434	-0.14895	-0.1001	0.115938
0.005594	0.081421	0.047882	3.3E-08	1.36E-10	0.064851	0.094657	0.262813	0.194282
0.36118	0.169162	0.251384	-0.38627	-0.6337	0.016289	0.067185	0.332255	-0.38695
3.02E-05	0.057271	0.00436	7.28E-06	1.28E-15	0.855765	0.452957	0.000135	6.99E-06
-0.04946	0.088701	0.400158	-0.43667	-0.12677	-0.26024	-0.31098	0.170253	0.057824
0.580818	0.321353	3.14E-06	2.86E-07	0.155526	0.003128	0.000373	0.055662	0.51845
-0.77991	-0.53657	-0.54892	0.077373	0.119118	-0.57168	-0.60344	-0.39811	0.449961
3.32E-27	7.91E-11	2.36E-11	0.387245	0.182245	2.22E-12	5.97E-14	3.57E-06	1.11E-07
-0.48098	-0.19426	-0.33161	0.305818	0.637764	-0.06878	-0.29124	-0.46118	0.353895
1.04E-08	0.028634	0.00014	0.000471	7.43E-16	0.442263	0.000894	4.85E-08	4.47E-05
-0.58869	-0.14262	-0.17222	0.008456	0.182251	-0.34622	-0.35285	-0.48339	0.456154
3.37E-13	0.109696	0.052847	0.924824	0.04029	6.69E-05	4.73E-05	8.59E-09	7.06E-08
-0.57062	-0.55569	-0.50448	0.388511	0.14708	-0.66652	-0.13548	-0.02509	0.544832
2.49E-12	1.19E-11	1.47E-09	6.37E-06	0.098921	1.2E-17	0.128848	0.779512	3.54E-11

ANKRD22	ANO9	ANXA1	ANXA13	ANXA2	ANXA4	AOAH	AOX1	APBB1IP
brown	brown	brown	brown	brown	brown	brown	brown	brown
0.259598	0.272301	0.143409	0.215133	0.094459	0.316412	0.464391	-0.05572	0.126199
0.003206	0.001954	0.107729	0.015142	0.290812	0.00029	3.81E-08	0.533837	0.157423
0.174376	0.101158	0.048183	0.22246	0.008392	0.223522	0.322606	-0.10118	-0.00823
0.049909	0.257789	0.590619	0.011944	0.925395	0.011533	0.000216	0.257667	0.926833
-0.04201	-0.19866	-0.00034	-0.28175	0.088361	-0.41215	-0.43399	-0.02188	-0.342
0.639069	0.025151	0.996961	0.001332	0.323218	1.48E-06	3.45E-07	0.807098	8.3E-05
0.108302	-0.07437	0.442125	-0.09255	0.390346	0.04473	-0.18487	0.165421	-0.24005
0.22551	0.406001	1.95E-07	0.300738	5.71E-06	0.617535	0.037459	0.063084	0.00656
0.152495	-0.031	0.170895	0.277696	0.176171	0.167411	0.20774	0.044583	-0.16527
0.086977	0.729337	0.054732	0.001573	0.047564	0.059935	0.019099	0.61869	0.063331
0.280381	0.001394	0.61074	0.211831	0.528361	0.369864	0.15151	0.257558	-0.1965
0.001409	0.987587	2.46E-14	0.016811	1.72E-10	1.87E-05	0.08906	0.003463	0.026817
0.125142	-0.07436	0.406681	0.080813	0.331893	0.22171	0.131217	0.096611	-0.17483
0.160961	0.40603	2.09E-06	0.366427	0.000138	0.012242	0.141429	0.279912	0.049306
0.227088	0.117035	0.582552	0.44566	0.287476	0.421403	0.239172	0.1325	0.126024
0.010244	0.190065	6.74E-13	1.52E-07	0.001049	8.07E-07	0.006767	0.137547	0.158005
0.185007	0.448979	0.459331	0.018664	0.655589	0.300018	0.13515	0.553124	0.148597
0.037313	1.19E-07	5.58E-08	0.835021	6.07E-17	0.000611	0.129782	1.55E-11	0.095452
0.548869	0.754376	0.70207	0.553471	0.758761	0.818302	0.852044	0.479129	0.682108
2.37E-11	1.32E-24	3.72E-20	1.49E-11	4.99E-25	7.53E-32	6.06E-37	1.21E-08	1.05E-18
0.511367	0.617959	0.634507	0.369844	0.616294	0.662209	0.652016	0.357746	0.477692
8.07E-10	9.97E-15	1.15E-15	1.87E-05	1.23E-14	2.29E-17	1.02E-16	3.64E-05	1.36E-08
-0.13462	0.058511	-0.2946	-0.28359	-0.10948	-0.23465	-0.19874	-0.0587	0.114574
0.131319	0.513479	0.000774	0.001234	0.220462	0.007922	0.025092	0.512139	0.199615
-0.21682	0.074943	-0.31227	-0.25093	-0.17785	-0.46632	-0.32227	-0.12818	0.066139
0.014348	0.402372	0.000351	0.004433	0.045452	3.29E-08	0.00022	0.15097	0.460039
-0.16943	0.167342	-0.28403	0.041181	-0.14811	-0.21393	-0.08276	-0.01747	0.117227
0.056872	0.060042	0.001212	0.645741	0.096557	0.01573	0.354937	0.845419	0.189334
0.11521	0.586142	0.135283	0.272852	0.272795	0.215995	0.347979	0.189921	0.567679
0.197115	4.5E-13	0.1294	0.001912	0.001916	0.014731	6.1E-05	0.032464	3.41E-12
-0.08919	-0.05398	-0.30236	0.231964	-0.36114	0.059849	0.22307	-0.3896	0.342618
0.31869	0.546674	0.00055	0.008687	3.03E-05	0.503885	0.011707	5.97E-06	8.05E-05
-0.42556	-0.54984	-0.81152	-0.45998	-0.78538	-0.77963	-0.56704	-0.59161	-0.21768
6.11E-07	2.15E-11	5.93E-31	5.31E-08	8.27E-28	3.56E-27	3.65E-12	2.41E-13	0.013956
-0.24631	-0.62831	-0.39612	-0.42977	-0.53303	-0.31555	-0.43286	-0.2932	-0.49446
0.005248	2.63E-15	4.03E-06	4.6E-07	1.11E-10	0.000302	3.72E-07	0.000822	3.46E-09
-0.31979	-0.52715	-0.68183	-0.35653	-0.75289	-0.41487	-0.32399	-0.39442	-0.27214
0.000247	1.93E-10	1.1E-18	3.89E-05	1.83E-24	1.24E-06	0.000203	4.47E-06	0.001967
-0.20131	-0.30956	-0.50895	-0.36527	-0.37291	-0.69056	-0.34783	-0.38453	-0.35487
0.023239	0.000398	9.98E-10	2.42E-05	1.58E-05	2.65E-19	6.15E-05	8.07E-06	4.25E-05

APOBEC3(	APOBEC3F	APOBEC3(	APOBEC3H	APOC1	APOL1	APOL2	APOL3	APPBP2
brown	brown	brown	brown	brown	brown	brown	brown	brown
0.093433	0.189872	0.322842	0.519205	0.259502	0.279849	0.354271	0.476526	-0.11313
0.296111	0.03251	0.000214	4E-10	0.003217	0.00144	4.38E-05	1.49E-08	0.20537
-0.09711	0.051553	0.170826	0.462245	0.221028	0.11923	0.259585	0.297882	-0.07243
0.277431	0.56488	0.05483	4.48E-08	0.012518	0.181831	0.003207	0.000671	0.418366
-0.02472	0.034873	-0.20287	-0.37383	-0.31744	-0.15001	-0.30792	-0.3864	0.363137
0.782624	0.697107	0.022168	1.5E-05	0.000276	0.092318	0.000429	7.23E-06	2.72E-05
0.256202	0.379906	0.190918	-0.13129	-0.36313	0.160023	-0.21	-0.15462	0.47934
0.003645	1.06E-05	0.031549	0.141213	2.72E-05	0.072315	0.017801	0.082617	1.19E-08
0.146974	0.242606	0.378294	0.29098	0.274906	0.17413	-0.15516	0.078352	-0.23245
0.099168	0.005993	1.16E-05	0.000904	0.001761	0.050239	0.081544	0.381248	0.008543
0.398501	0.561373	0.545515	0.226697	-0.09503	0.384919	-0.17845	0.080311	0.227494
3.48E-06	6.63E-12	3.31E-11	0.010379	0.287882	7.88E-06	0.044715	0.369421	0.010105
0.226948	0.297851	0.345112	0.173676	-0.06827	0.207924	-0.05247	0.055017	-0.00032
0.010292	0.000672	7.08E-05	0.05085	0.445688	0.018991	0.558002	0.538985	0.997131
0.216128	0.261018	0.304622	0.072999	0.054577	0.177539	0.027133	0.211231	-0.12168
0.014668	0.003037	0.000497	0.414721	0.542245	0.045839	0.762035	0.017131	0.172966
0.554342	0.4443	0.320688	0.135177	-0.00174	0.47316	0.28077	0.289642	-0.12669
1.37E-11	1.67E-07	0.000237	0.129706	0.984476	1.94E-08	0.001387	0.000957	0.155789
0.837458	0.674855	0.817508	0.704417	0.638153	0.737711	0.560492	0.850612	-0.46939
1.34E-34	3.33E-18	9.63E-32	2.47E-20	7.04E-16	4.51E-23	7.26E-12	1.06E-36	2.6E-08
0.726803	0.715077	0.788508	0.604901	0.328606	0.752005	0.520035	0.768692	-0.13704
3.94E-22	3.62E-21	3.68E-28	5.01E-14	0.000162	2.23E-24	3.71E-10	5.07E-26	0.124457
-0.15365	-0.12371	-0.1816	-0.12734	0.009813	-0.08631	0.194479	-0.049	-0.03677
0.084586	0.165855	0.041025	0.153664	0.912809	0.334595	0.028454	0.584354	0.681461
-0.13494	-0.26803	-0.46373	-0.33087	-0.03515	-0.30479	0.105508	-0.16708	-0.05502
0.130376	0.002315	4E-08	0.000145	0.694798	0.000494	0.237778	0.060444	0.538965
0.034736	-0.12732	-0.19503	-0.043	0.364023	-0.14323	0.139997	0.002233	-0.41441
0.698236	0.153732	0.028001	0.631196	2.59E-05	0.108175	0.116455	0.980122	1.28E-06
0.423368	0.173782	0.143197	0.276373	0.547634	0.207064	0.497712	0.47423	-0.52683
7.08E-07	0.050706	0.108257	0.001659	2.68E-11	0.019502	2.63E-09	1.78E-08	1.99E-10
-0.2352	-0.32367	-0.12487	0.253712	0.406769	-0.25975	0.112352	0.119155	-0.10528
0.007773	0.000206	0.161869	0.004	2.08E-06	0.003187	0.208532	0.182108	0.238783
-0.73316	-0.76121	-0.78852	-0.57042	-0.20396	-0.74036	-0.32385	-0.59481	0.206048
1.13E-22	2.87E-25	3.67E-28	2.55E-12	0.021445	2.62E-23	0.000204	1.66E-13	0.020121
-0.58338	-0.39808	-0.37878	-0.39693	-0.58639	-0.35538	-0.45761	-0.55611	0.614341
6.14E-13	3.57E-06	1.13E-05	3.83E-06	4.37E-13	4.13E-05	6.34E-08	1.14E-11	1.57E-14
-0.75238	-0.68829	-0.54484	-0.34987	-0.30966	-0.50025	-0.25779	-0.42308	0.169833
2.05E-24	3.85E-19	3.54E-11	5.53E-05	0.000396	2.12E-09	0.003433	7.22E-07	0.056277
-0.37558	-0.37948	-0.43428	-0.33691	-0.20029	-0.41166	-0.39597	-0.4495	0.068424
1.35E-05	1.08E-05	3.38E-07	0.000107	0.023963	1.52E-06	4.07E-06	1.15E-07	0.444647

AQP9	ARHGAP1!	ARHGAP1!	ARHGAP2	ARHGAP2!	ARHGAP2	ARHGAP3(	ARHGAP4	ARHGAP9
brown	brown	brown	brown	brown	brown	brown	brown	brown
0.106336	0.308418	-0.04132	0.403173	0.330553	0.183099	0.256782	0.119077	0.185521
0.234094	0.000419	0.644586	2.61E-06	0.000147	0.039353	0.003566	0.182398	0.036779
0.153685	0.177466	-0.17101	0.242435	0.147116	0.056892	0.077775	-0.00962	0.020762
0.084512	0.045929	0.054568	0.006029	0.098838	0.525223	0.384778	0.914517	0.816782
-0.18713	-0.17563	0.09997	-0.44632	-0.28602	-0.39279	-0.26123	-0.35307	-0.31084
0.035151	0.048255	0.263451	1.45E-07	0.001115	4.93E-06	0.003012	4.67E-05	0.000375
0.032588	0.152672	0.509258	-0.09874	-0.08449	-0.3576	-0.1004	-0.41433	-0.18747
0.716069	0.086606	9.72E-10	0.269399	0.344938	3.67E-05	0.261415	1.28E-06	0.03481
0.170967	0.385234	0.085915	0.028365	0.038862	-0.10653	-0.06515	-0.05519	0.083005
0.054628	7.74E-06	0.336844	0.75158	0.664447	0.233228	0.466759	0.537736	0.353527
0.259208	0.521216	0.61075	0.13874	0.090428	-0.27821	-0.00347	-0.31628	-0.0062
0.003253	3.33E-10	2.45E-14	0.119805	0.311984	0.00154	0.96909	0.000292	0.944874
0.14909	0.35331	0.368531	0.108142	0.07073	-0.17249	-0.02938	-0.28884	-0.05408
0.094345	4.61E-05	2.02E-05	0.226201	0.429415	0.052471	0.743032	0.00099	0.545969
0.019304	0.321503	0.449303	0.271526	0.110671	0.155037	0.218368	0.147584	0.16688
0.829444	0.000228	1.17E-07	0.002016	0.215466	0.081781	0.013648	0.097758	0.060763
0.325097	0.337739	0.559736	0.267423	0.286166	0.252481	0.442589	0.249692	0.403132
0.000192	0.000103	7.86E-12	0.00237	0.001108	0.004187	1.89E-07	0.00464	2.61E-06
0.434947	0.850006	0.586032	0.712927	0.784072	0.748943	0.922246	0.736805	0.889609
3.22E-07	1.33E-36	4.55E-13	5.36E-21	1.16E-27	4.31E-24	1.97E-53	5.42E-23	2.29E-44
0.193323	0.665315	0.600971	0.593195	0.656011	0.462797	0.664963	0.401564	0.606276
0.02943	1.44E-17	8.03E-14	2E-13	5.71E-17	4.3E-08	1.52E-17	2.88E-06	4.24E-14
-0.15271	-0.28397	-0.27747	-0.07453	-0.01283	0.022877	-0.06841	-0.02793	0.023529
0.08653	0.001215	0.001587	0.404995	0.886185	0.798492	0.444721	0.75529	0.792881
-0.3044	-0.42676	-0.30834	-0.28029	-0.12451	0.151236	0.047247	0.185383	-0.01126
0.000503	5.64E-07	0.000421	0.001414	0.163101	0.089645	0.59786	0.036922	0.899993
-0.14972	-0.23265	-0.3307	-0.2176	-0.06443	0.366542	0.124333	0.526378	0.257881
0.092942	0.008486	0.000146	0.01399	0.471762	2.25E-05	0.163706	2.07E-10	0.003421
0.025805	0.1889	0.027624	0.291104	0.436279	0.7624	0.681277	0.824423	0.667702
0.773361	0.033425	0.757864	0.000899	2.94E-07	2.19E-25	1.21E-18	1.08E-32	1E-17
-0.1764	-0.05927	-0.50037	0.147726	0.200426	0.294173	0.105599	0.245614	0.081864
0.047273	0.508044	2.1E-09	0.097431	0.023863	0.000788	0.23737	0.00538	0.360207
-0.52032	-0.76129	-0.77583	-0.59676	-0.46777	-0.30714	-0.56846	-0.29096	-0.54288
3.61E-10	2.82E-25	9.1E-27	1.32E-13	2.94E-08	0.000444	3.14E-12	0.000904	4.29E-11
-0.13984	-0.4435	-0.21849	-0.35751	-0.48799	-0.68616	-0.71907	-0.72197	-0.71359
0.116863	1.77E-07	0.013592	3.69E-05	5.91E-09	5.47E-19	1.72E-21	9.96E-22	4.75E-21
-0.18173	-0.56693	-0.59886	-0.31437	-0.42901	-0.3723	-0.58634	-0.38974	-0.53354
0.040875	3.69E-12	1.03E-13	0.000319	4.84E-07	1.63E-05	4.4E-13	5.92E-06	1.06E-10
-0.18336	-0.27623	-0.45543	-0.39811	-0.24778	-0.33606	-0.34532	-0.33631	-0.34032
0.039073	0.001669	7.45E-08	3.57E-06	0.004976	0.000112	7.01E-05	0.000111	9.05E-05

ARHGDIB	ARHGEF35	ARHGEF5	ARL11	ARPC2	ARPC3	ARRB2	ARRDC4	ARRDC5
brown	brown	brown	brown	brown	brown	brown	brown	brown
0.229597	0.072116	0.233089	0.206475	0.129257	0.342518	0.283884	0.237503	0.02021
0.009414	0.420401	0.008358	0.019859	0.147525	8.09E-05	0.001219	0.007174	0.821573
0.106103	0.035696	0.224986	0.092381	0.03595	0.330537	0.197074	0.20326	-0.07643
0.235128	0.690315	0.010988	0.301605	0.688228	0.000147	0.026366	0.021908	0.393045
-0.2591	-0.23668	-0.33333	-0.1077	0.066364	-0.22554	-0.55692	-0.07324	-0.32508
0.003267	0.007384	0.000128	0.228133	0.458504	0.010788	1.05E-11	0.413165	0.000192
-0.09743	-0.11914	-0.24533	0.162802	0.156865	0.045184	-0.6102	0.128449	-0.32697
0.275819	0.182146	0.005437	0.067435	0.078201	0.613967	2.63E-14	0.150092	0.000175
0.166471	0.129185	0.079469	0.318253	0.37073	0.589828	0.05351	-0.04749	-0.1166
0.061406	0.147753	0.374477	0.000266	1.78E-05	2.95E-13	0.550178	0.595941	0.19172
0.141284	0.071682	-0.06773	0.436914	0.421659	0.547917	-0.38744	0.154677	-0.29127
0.113101	0.423216	0.449263	2.81E-07	7.93E-07	2.61E-11	6.79E-06	0.082503	0.000893
0.182412	0.206846	-0.00363	0.269701	0.340973	0.448866	-0.21995	0.058858	-0.23311
0.04011	0.019634	0.967688	0.002167	8.75E-05	1.2E-07	0.012965	0.510982	0.008352
0.181689	0.055301	0.008089	0.244996	0.07103	0.244286	0.004638	0.194305	0.025728
0.040921	0.536888	0.92808	0.005501	0.427455	0.005643	0.958729	0.028599	0.774017
0.506699	0.415333	0.249448	0.329744	0.575974	0.232883	-0.04988	0.334977	0.16554
1.22E-09	1.2E-06	0.004681	0.000153	1.39E-12	0.008418	0.577607	0.000118	0.062893
0.904364	0.61575	0.527972	0.835519	0.628548	0.70774	0.61632	0.606922	0.511382
4.66E-48	1.32E-14	1.79E-10	2.64E-34	2.54E-15	1.37E-20	1.23E-14	3.92E-14	8.06E-10
0.597459	0.356912	0.280666	0.617137	0.411197	0.454668	0.248887	0.441114	0.195422
1.22E-13	3.81E-05	0.001393	1.11E-14	1.57E-06	7.88E-08	0.004778	2.1E-07	0.02768
-0.01244	0.111175	0.250525	-0.26462	-0.24956	-0.21474	-0.02677	-0.10732	0.11812
0.889566	0.213371	0.0045	0.002643	0.004663	0.015332	0.765144	0.229768	0.185962
-0.12022	-0.06752	-0.00513	-0.2656	-0.23292	-0.54488	0.039246	0.01404	0.141525
0.178194	0.450666	0.954389	0.002545	0.008408	3.53E-11	0.661331	0.875509	0.112483
0.0483	0.059006	0.106319	-0.01587	-0.1224	-0.27086	0.392934	-0.01938	0.34879
0.589716	0.509922	0.234172	0.859471	0.17039	0.00207	4.89E-06	0.828765	5.85E-05
0.540945	0.396862	0.434218	0.326791	0.153118	0.026124	0.635142	0.404348	0.604852
5.19E-11	3.85E-06	3.39E-07	0.000177	0.08568	0.77064	1.06E-15	2.42E-06	5.04E-14
0.077548	-0.00884	0.199338	-0.0698	-0.33898	0.014222	0.504467	0.126615	0.292404
0.38617	0.921416	0.024652	0.435494	9.68E-05	0.873906	1.47E-09	0.156048	0.00085
-0.59048	-0.50374	-0.41033	-0.68325	-0.59777	-0.69225	-0.12562	-0.44008	-0.19144
2.74E-13	1.57E-09	1.66E-06	8.78E-19	1.17E-13	1.99E-19	0.159338	2.25E-07	0.031076
-0.68776	-0.49916	-0.50615	-0.53909	-0.42878	-0.36319	-0.54208	-0.48608	-0.45442
4.21E-19	2.32E-09	1.27E-09	6.21E-11	4.92E-07	2.71E-05	4.64E-11	6.9E-09	8.03E-08
-0.5462	-0.37668	-0.29531	-0.64303	-0.47428	-0.39234	-0.08571	-0.53176	-0.238
3.1E-11	1.27E-05	0.00075	3.61E-16	1.77E-08	5.07E-06	0.338027	1.25E-10	0.00705
-0.30303	-0.19864	-0.14064	-0.29726	0.007905	-0.18441	-0.20237	-0.32155	-0.17562
0.000534	0.02517	0.114783	0.000689	0.929713	0.037945	0.02251	0.000228	0.048276

ASAH1	ASGR2	ASXL3	ATP2A3	ATP6V0E1	ATP8B4	ATP9B	AUTS2	AXIN2
brown	brown	brown	brown	brown	brown	brown	brown	brown
0.248518	0.385304	0.000571	0.109305	0.184137	0.300543	-0.11637	-0.29555	-0.2692
0.004843	7.71E-06	0.994913	0.221217	0.038232	0.000597	0.192596	0.000742	0.00221
0.102752	0.315512	0.038779	-0.06552	0.134674	0.164564	-0.12102	-0.2583	-0.196
0.250327	0.000302	0.665121	0.464245	0.13115	0.064482	0.175299	0.003367	0.027216
-0.34268	-0.35996	-0.00267	-0.16476	-0.02208	-0.31949	-0.08368	0.41621	0.129562
8.02E-05	3.23E-05	0.976231	0.064165	0.805386	0.000251	0.349602	1.13E-06	0.146562
-0.04011	-0.13015	-0.25439	-0.25869	0.27456	0.08434	-0.32499	0.277869	-0.07119
0.654324	0.144715	0.0039	0.003318	0.001785	0.345811	0.000193	0.001562	0.426391
-0.03618	0.274592	-0.3021	-0.13891	0.530152	0.067541	-0.40395	-0.23257	-0.31516
0.686371	0.001783	0.000557	0.119352	1.46E-10	0.450556	2.48E-06	0.008509	0.000307
0.110194	0.206285	-0.47866	-0.28566	0.657418	0.321667	-0.56642	-0.00755	-0.36037
0.217463	0.019975	1.25E-08	0.001132	4.65E-17	0.000226	3.9E-12	0.932858	3.16E-05
0.07167	0.169618	-0.26145	-0.18857	0.475617	0.131877	-0.39137	-0.16449	-0.16895
0.423291	0.056593	0.002987	0.033737	1.6E-08	0.139423	5.37E-06	0.06461	0.057591
0.344439	0.193287	-0.11972	-0.09264	0.360685	0.411225	-0.23436	0.011969	-0.08889
7.33E-05	0.02946	0.180033	0.300259	3.1E-05	1.57E-06	0.008001	0.893755	0.320319
0.123177	0.217895	-0.48724	0.505758	0.385023	0.136174	-0.28177	-0.16113	-0.05971
0.167688	0.013858	6.28E-09	1.32E-09	7.84E-06	0.126872	0.001331	0.070343	0.504904
0.726483	0.735882	-0.56989	0.554395	0.770956	0.779461	-0.4511	-0.51113	-0.57606
4.19E-22	6.54E-23	2.7E-12	1.36E-11	2.96E-26	3.71E-27	1.02E-07	8.24E-10	1.38E-12
0.569237	0.518367	-0.46862	0.375885	0.578217	0.675747	-0.42312	-0.19081	-0.4653
2.89E-12	4.31E-10	2.75E-08	1.33E-05	1.09E-12	2.9E-18	7.2E-07	0.03165	3.55E-08
-0.23586	-0.18673	0.103165	0.198098	-0.25547	-0.35588	0.144354	0.005365	0.301916
0.007598	0.035552	0.248419	0.025579	0.003746	4.02E-05	0.105406	0.952262	0.000561
-0.1912	-0.31788	0.307655	0.299641	-0.41618	-0.38829	0.317215	0.208668	0.305539
0.031298	0.000271	0.000434	0.000621	1.14E-06	6.46E-06	0.000279	0.018558	0.000477
-0.0991	-0.05786	0.084127	0.388178	-0.17982	-0.30984	0.145512	-0.03416	0.026948
0.267656	0.518213	0.347034	6.5E-06	0.043076	0.000393	0.102612	0.703016	0.763611
0.37094	0.269112	-0.11093	0.697959	0.133266	0.18328	-0.00768	-0.25783	-0.15307
1.76E-05	0.002218	0.214404	7.58E-20	0.135266	0.039156	0.931698	0.003428	0.085779
0.272254	0.126448	0.289696	-0.01582	-0.2007	0.131908	0.211345	-0.25628	-0.08184
0.001958	0.1566	0.000955	0.859846	0.023668	0.13933	0.01707	0.003634	0.360345
-0.42296	-0.5749	0.628329	-0.26181	-0.77987	-0.6289	0.515902	0.302173	0.525469
7.27E-07	1.57E-12	2.62E-15	0.002946	3.35E-27	2.43E-15	5.39E-10	0.000555	2.25E-10
-0.35067	-0.40584	0.310017	-0.64702	-0.50669	-0.2475	0.302716	0.34092	0.285255
5.3E-05	2.2E-06	0.00039	2.07E-16	1.22E-09	0.005025	0.000542	8.78E-05	0.001151
-0.35493	-0.33526	0.511201	-0.38856	-0.66138	-0.36936	0.499591	0.130713	0.399151
4.23E-05	0.000117	8.19E-10	6.35E-06	2.6E-17	1.92E-05	2.24E-09	0.14298	3.34E-06
-0.50194	-0.33148	0.219515	-0.03435	-0.27439	-0.49003	0.244182	-0.01946	0.247452
1.83E-09	0.000141	0.01315	0.701466	0.001797	4.99E-09	0.005665	0.828094	0.005034

AXL	B3GNT7	B4GALNT3	B4GALT1	BATF	BATF2	BCL2A1	BEND5	BHLHE40
brown	brown	brown	brown	brown	brown	brown	brown	brown
0.354492	0.041678	-0.14662	0.229135	0.205972	0.315766	0.184703	-0.0361	0.271108
4.33E-05	0.641758	0.099995	0.009563	0.020168	0.000299	0.037633	0.686973	0.002049
0.235095	-0.06686	-0.09881	0.08154	0.044077	0.173822	0.098598	0.017765	0.201559
0.0078	0.455139	0.269073	0.362118	0.622687	0.050653	0.270093	0.842861	0.023064
-0.38738	-0.16329	0.241535	-0.1517	-0.15136	0.022559	-0.10274	0.198619	-0.35683
6.81E-06	0.066606	0.006225	0.08866	0.089372	0.801236	0.250384	0.025186	3.82E-05
-0.14609	-0.1002	0.115518	0.113018	-0.08618	0.236801	0.157547	0.007594	-0.01244
0.101234	0.262331	0.195911	0.205831	0.335362	0.007352	0.076897	0.93247	0.889593
-0.07943	-0.27257	-0.09264	-0.07907	0.090016	0.090269	0.393798	0.433515	-0.18312
0.374721	0.001933	0.300244	0.376878	0.3142	0.312839	4.64E-06	3.56E-07	0.039325
-0.00907	-0.20335	-0.0535	0.206704	0.05223	0.309991	0.479877	0.13987	0.065073
0.919352	0.021846	0.550242	0.01972	0.559769	0.00039	1.14E-08	0.116791	0.467316
0.072567	-0.07672	0.069901	0.095098	0.055279	0.285998	0.313257	0.016837	0.139976
0.417498	0.391274	0.434854	0.287547	0.537051	0.001116	0.000336	0.850971	0.11651
0.100045	-0.06246	-0.29744	0.126574	0.102954	0.065892	0.181607	-0.06841	0.21635
0.263093	0.485436	0.000684	0.156184	0.249393	0.461718	0.041014	0.444759	0.014564
0.30231	0.344282	-0.12606	0.62017	0.473107	0.349003	0.367162	-0.31828	0.356721
0.000552	7.39E-05	0.157882	7.53E-15	1.94E-08	5.79E-05	2.18E-05	0.000266	3.85E-05
0.722906	0.441276	-0.60484	0.780918	0.822521	0.584284	0.753351	-0.45227	0.429302
8.34E-22	2.07E-07	5.05E-14	2.58E-27	1.99E-32	5.55E-13	1.66E-24	9.4E-08	4.75E-07
0.647138	0.307577	-0.46242	0.65151	0.560583	0.813342	0.541075	-0.37392	0.403354
2.03E-16	0.000435	4.42E-08	1.09E-16	7.2E-12	3.44E-31	5.12E-11	1.49E-05	2.58E-06
0.085813	0.13408	0.062199	-0.05657	-0.11856	-0.03671	-0.20052	-0.01727	0.040469
0.337421	0.132876	0.487253	0.527557	0.184313	0.681996	0.023795	0.847144	0.651456
-0.07271	0.2108	0.056417	-0.08475	0.069287	-0.06384	-0.35289	-0.10503	-0.17773
0.416566	0.017364	0.528693	0.343488	0.438908	0.475783	4.72E-05	0.23994	0.045603
-0.03392	0.215657	-0.1974	-0.11667	0.295099	-0.0082	-0.11663	0.084364	-0.24744
0.704969	0.014891	0.026109	0.191459	0.000757	0.927111	0.191621	0.345673	0.005037
0.482821	0.550306	-0.46268	0.413298	0.659871	0.270618	0.164723	-0.40026	0.136343
8.99E-09	2.06E-11	4.33E-08	1.37E-06	3.25E-17	0.00209	0.064221	3.12E-06	0.126398
0.195671	0.235105	-0.12787	-0.26822	-0.03283	-0.22175	-0.21384	-0.19525	-0.18723
0.027479	0.007798	0.151942	0.002297	0.714016	0.012225	0.015779	0.02782	0.035047
-0.49318	-0.13179	0.38872	-0.72356	-0.54457	-0.51517	-0.66217	0.265621	-0.50263
3.84E-09	0.139677	6.29E-06	7.36E-22	3.63E-11	5.75E-10	2.31E-17	0.002543	1.73E-09
-0.48669	-0.44321	0.500353	-0.5062	-0.73951	-0.41414	-0.43129	0.238321	-0.14933
6.57E-09	1.81E-07	2.1E-09	1.27E-09	3.13E-23	1.3E-06	4.15E-07	0.006972	0.093801
-0.38216	-0.32831	0.41425	-0.56322	-0.62653	-0.55355	-0.51674	0.252776	-0.14861
9.27E-06	0.000164	1.29E-06	5.46E-12	3.32E-15	1.48E-11	5E-10	0.004141	0.095425
-0.39379	-0.23742	0.395167	-0.35315	-0.2376	-0.3103	-0.23015	0.414875	-0.47185
4.64E-06	0.007194	4.27E-06	4.65E-05	0.007149	0.000385	0.009241	1.24E-06	2.15E-08

BIN2	BIRC3	BLNK	BLVRA	BMF	BMP2K	BRI3	BTG1	BTK
brown	brown	brown	brown	brown	brown	brown	brown	brown
0.299084	0.228339	0.250577	0.11328	0.231539	0.128233	0.222023	0.063917	0.280768
0.000636	0.009823	0.004491	0.204773	0.008814	0.150785	0.012117	0.47528	0.001387
0.139161	0.171033	0.109739	0.122297	0.042401	-0.01742	0.184611	0.030963	0.12327
0.118674	0.054533	0.219381	0.170767	0.635979	0.845857	0.037729	0.729669	0.167365
-0.37833	-0.26593	-0.1601	-0.01319	0.008943	-0.21968	-0.13059	0.142117	-0.27117
1.16E-05	0.002512	0.07217	0.883023	0.920515	0.013081	0.143349	0.11097	0.002044
-0.11534	0.200191	0.069432	0.160741	0.081819	0.105121	-0.01487	0.353782	0.02269
0.196624	0.024032	0.437952	0.071028	0.360467	0.239515	0.868189	4.5E-05	0.800106
0.012744	0.10479	0.100284	0.518478	-0.00176	-0.40692	0.57102	0.146212	0.108227
0.886915	0.241005	0.26195	4.27E-10	0.984376	2.06E-06	2.39E-12	0.100952	0.225833
0.057387	0.432819	0.21873	0.525901	0.101681	-0.00933	0.389256	0.43668	0.244279
0.52162	3.73E-07	0.013489	2.16E-10	0.255324	0.917107	6.1E-06	2.86E-07	0.005645
0.039989	0.271106	0.121213	0.334267	0.021951	-0.00812	0.283506	0.323238	0.152482
0.655323	0.00205	0.174622	0.000123	0.806488	0.927771	0.001238	0.00021	0.087003
0.243082	0.264686	0.21148	0.136976	0.085416	0.388689	0.072621	0.002082	0.284976
0.005892	0.002637	0.016998	0.12463	0.339667	6.31E-06	0.417151	0.981463	0.001165
0.21294	0.308983	0.087292	0.404741	0.43947	0.23807	0.30928	0.647183	0.282868
0.016234	0.000409	0.329127	2.36E-06	2.35E-07	0.007034	0.000403	2.02E-16	0.001272
0.850701	0.546193	0.59192	0.553384	0.715476	0.600085	0.714153	0.509934	0.889464
1.02E-36	3.1E-11	2.32E-13	1.51E-11	3.36E-21	8.92E-14	4.29E-21	9.15E-10	2.47E-44
0.647323	0.558353	0.548722	0.426823	0.547363	0.553268	0.473323	0.349743	0.72825
1.98E-16	9.06E-12	2.41E-11	5.61E-07	2.76E-11	1.52E-11	1.91E-08	5.57E-05	2.97E-22
-0.06802	-0.17082	-0.09686	-0.19926	-0.00107	-0.03579	-0.0899	-0.05224	-0.13291
0.447326	0.054844	0.278694	0.024706	0.990455	0.689532	0.314815	0.55973	0.13633
-0.1265	-0.4421	-0.20499	-0.38885	0.131171	0.044181	-0.27832	-0.09321	-0.21358
0.156438	1.95E-07	0.020787	6.25E-06	0.141572	0.621864	0.001533	0.297292	0.015909
-0.0272	-0.33455	-0.15244	-0.08411	0.086675	-0.2208	0.20967	-0.16924	-0.10537
0.761427	0.000121	0.087101	0.347147	0.332573	0.012614	0.017988	0.05716	0.238385
0.491284	-0.03253	0.200041	0.027819	0.537955	0.376166	0.325293	0.138448	0.412991
4.5E-09	0.716546	0.02414	0.756209	6.93E-11	1.31E-05	0.00019	0.120593	1.4E-06
0.242506	-0.23305	0.182485	-0.35001	-0.08872	0.090903	-0.11124	-0.53769	0.110583
0.006014	0.00837	0.04003	5.49E-05	0.321265	0.309439	0.21311	7.11E-11	0.215832
-0.49989	-0.72602	-0.34641	-0.66896	-0.46124	-0.37886	-0.61561	-0.64185	-0.62797
2.18E-09	4.59E-22	6.62E-05	8.29E-18	4.83E-08	1.12E-05	1.34E-14	4.24E-16	2.75E-15
-0.53092	-0.10526	-0.28232	-0.33326	-0.62673	-0.30899	-0.59249	-0.3766	-0.52728
1.35E-10	0.238907	0.001301	0.000129	3.23E-15	0.000408	2.17E-13	1.28E-05	1.9E-10
-0.419	-0.29662	-0.32731	-0.43515	-0.62312	-0.34933	-0.57372	-0.56639	-0.52404
9.45E-07	0.000709	0.000173	3.18E-07	5.16E-15	5.69E-05	1.78E-12	3.91E-12	2.57E-10
-0.37283	-0.58156	-0.27123	-0.19736	0.014617	-0.44462	-0.2119	-0.01823	-0.40517
1.58E-05	7.52E-13	0.00204	0.026146	0.870433	1.63E-07	0.016776	0.838763	2.3E-06

BTN3A3	C11orf21	C11orf45	C11orf80	C11orf95	C15orf61	C16orf54	C17orf100	C17orf51
brown	brown	brown	brown	brown	brown	brown	brown	brown
0.422731	-0.02558	0.311378	-0.00132	-0.29271	0.036772	0.22701	-0.18356	-0.10897
7.39E-07	0.775281	0.000366	0.988265	0.000839	0.681485	0.010271	0.038853	0.222644
0.255565	-0.1433	0.195746	0.07	-0.22335	0.187778	0.049765	-0.06795	-0.01538
0.003733	0.107991	0.027418	0.434203	0.011599	0.034509	0.578472	0.447841	0.863726
-0.09833	-0.29375	-0.23542	0.345287	0.518271	-0.10224	-0.22166	0.444975	-0.03363
0.271397	0.000803	0.007712	7.02E-05	4.35E-10	0.252714	0.012262	1.59E-07	0.70743
0.238949	-0.1171	0.121487	0.18922	0.048331	-0.25082	-0.11141	0.205025	-0.27951
0.00682	0.189806	0.173641	0.033122	0.589479	0.004451	0.212383	0.020761	0.00146
0.148322	0.035749	-0.02457	0.203678	-0.15924	0.375193	0.069647	0.214271	-0.43166
0.096073	0.689886	0.783914	0.021632	0.073736	1.38E-05	0.436531	0.015563	4.04E-07
0.440916	0.01663	0.223262	0.16071	-0.27793	-0.02583	0.044158	0.159473	-0.5785
2.12E-07	0.852781	0.011633	0.071083	0.001558	0.773129	0.622043	0.073312	1.06E-12
0.326084	-0.09536	0.211027	0.016906	-0.23593	-0.06736	-0.00587	0.071259	-0.2782
0.000183	0.286228	0.017241	0.850371	0.007577	0.451789	0.947741	0.425963	0.001541
0.286857	0.145329	0.096143	-0.12653	-0.39824	-0.31332	0.076306	-0.33426	-0.31275
0.001076	0.103049	0.282259	0.156321	3.54E-06	0.000335	0.393844	0.000123	0.000344
0.353534	0.041863	0.245086	-0.19243	-0.08882	-0.41492	0.399316	-0.17675	-0.29415
4.56E-05	0.640274	0.005484	0.030206	0.320704	1.23E-06	3.31E-06	0.046826	0.000789
0.690149	0.528004	0.533839	-0.55411	-0.68138	-0.48181	0.783108	-0.55389	-0.69168
2.83E-19	1.78E-10	1.03E-10	1.4E-11	1.19E-18	9.76E-09	1.48E-27	1.43E-11	2.2E-19
0.827924	0.42211	0.551787	-0.39206	-0.56388	-0.38453	0.593101	-0.49957	-0.59506
3.45E-33	7.7E-07	1.77E-11	5.15E-06	5.1E-12	8.06E-06	2.03E-13	2.24E-09	1.61E-13
-0.17878	-0.13026	0.146463	0.022857	0.131521	0.201891	-0.0493	-0.00533	0.284657
0.044317	0.144371	0.100361	0.798666	0.140501	0.022834	0.582049	0.95262	0.00118
-0.29205	-0.12356	-0.20662	-0.04268	0.476615	-0.21956	-0.0208	0.073776	0.330246
0.000863	0.166353	0.019773	0.633794	1.48E-08	0.013132	0.816431	0.409761	0.00015
-0.28873	0.128811	-0.28364	-0.15126	0.228846	0.133985	0.175761	0.03908	0.038019
0.000995	0.148939	0.001231	0.089598	0.009656	0.133154	0.048092	0.66268	0.671299
0.148583	0.32844	0.121841	-0.5299	-0.12993	-0.39464	0.563075	-0.36925	-0.17937
0.095483	0.000163	0.17238	1.49E-10	0.145397	4.41E-06	5.55E-12	1.94E-05	0.043613
-0.20247	0.130562	-0.0054	-0.30556	-0.18169	0.02532	0.024756	-0.19282	0.223227
0.022441	0.143444	0.951988	0.000477	0.040923	0.777513	0.782338	0.029867	0.011646
-0.68074	-0.31717	-0.49312	0.242141	0.658127	0.252883	-0.53009	0.34801	0.68272
1.31E-18	0.00028	3.86E-09	0.006093	4.19E-17	0.004125	1.46E-10	6.09E-05	9.56E-19
-0.34816	-0.28483	-0.19881	0.420653	0.206379	0.371381	-0.60013	0.258404	0.449664
6.04E-05	0.001172	0.025044	8.48E-07	0.019918	1.72E-05	8.87E-14	0.003354	1.14E-07
-0.52059	-0.23836	-0.28366	0.281252	0.236047	0.455236	-0.51076	0.112485	0.659843
3.53E-10	0.006963	0.00123	0.001359	0.007547	7.56E-08	8.51E-10	0.207993	3.26E-17
-0.37284	-0.41046	-0.3491	0.510289	0.676679	0.171543	-0.23867	0.55024	0.299624
1.58E-05	1.64E-06	5.76E-05	8.87E-10	2.5E-18	0.053806	0.006887	2.07E-11	0.000621

C19orf38	C1orf127	C1orf162	C1orf21	C1QA	C1QB	C1QC	C1R	C1RL
brown	brown	brown	brown	brown	brown	brown	brown	brown
0.216649	0.230119	0.264762	-0.15439	0.308252	0.286277	0.199036	0.125785	0.054761
0.014425	0.009249	0.002629	0.083078	0.000422	0.001103	0.024875	0.1588	0.540883
0.180405	0.149687	0.182471	-0.05793	0.186336	0.143394	0.043957	-0.00285	-0.05609
0.042393	0.093019	0.040045	0.517715	0.035945	0.107766	0.623631	0.974643	0.53108
-0.23336	-0.1382	-0.26257	0.11353	-0.38388	-0.36684	-0.35951	-0.00283	0.074969
0.008281	0.121262	0.002861	0.203769	8.38E-06	2.21E-05	3.31E-05	0.97484	0.40221
-0.13761	-0.16314	0.029628	-0.04806	-0.28492	-0.28722	-0.37515	0.237721	0.428104
0.122884	0.06686	0.7409	0.591597	0.001167	0.00106	1.39E-05	0.00712	5.15E-07
0.437747	-0.04091	0.269612	-0.11086	0.281126	0.129233	-0.00342	0.028616	-0.1059
2.65E-07	0.647951	0.002174	0.214696	0.001367	0.147598	0.969545	0.749451	0.236041
0.216497	-0.13894	0.341338	-0.22211	0.056241	-0.05161	-0.23812	0.292316	0.385251
0.014496	0.119277	8.59E-05	0.012081	0.529978	0.564436	0.007021	0.000854	7.73E-06
0.220274	-0.06979	0.231823	-0.03465	0.031017	-0.0362	-0.1954	0.284738	0.302265
0.01283	0.435571	0.008728	0.698936	0.729215	0.686213	0.027698	0.001176	0.000553
-0.03712	0.003465	0.303972	-0.15587	0.164262	0.169148	0.078204	0.165367	0.265589
0.678663	0.969162	0.000512	0.080139	0.06498	0.057292	0.382152	0.063173	0.002546
0.101714	0.150805	0.372883	-0.49278	0.298518	0.317022	0.323099	0.711905	0.669697
0.255172	0.090574	1.58E-05	3.98E-09	0.000652	0.000282	0.000211	6.46E-21	7.4E-18
0.607195	0.454018	0.857866	-0.69541	0.88964	0.883666	0.802938	0.776434	0.678378
3.79E-14	8.27E-08	5.96E-38	1.17E-19	2.25E-44	5.01E-43	7.2E-30	7.85E-27	1.91E-18
0.418426	0.262623	0.583294	-0.50435	0.5652	0.579975	0.51244	0.655943	0.648456
9.81E-07	0.002855	6.2E-13	1.49E-09	4.44E-12	8.98E-13	7.34E-10	5.77E-17	1.69E-16
-0.03472	0.087662	-0.17896	-0.00632	-0.10567	-0.04289	0.07174	0.10476	0.013389
0.698376	0.327078	0.044107	0.94381	0.237068	0.632095	0.422835	0.241142	0.881234
-0.25044	0.172113	-0.28987	0.132032	-0.1863	-0.04959	0.103186	0.012686	-0.00474
0.004515	0.053003	0.000948	0.138953	0.035985	0.579811	0.248324	0.887433	0.957797
0.194626	0.249284	-0.10166	-0.06	0.228687	0.258672	0.439422	-0.00909	-0.17745
0.028333	0.004709	0.255409	0.502813	0.009708	0.00332	2.36E-07	0.919199	0.045951
0.279658	0.493791	0.339439	-0.37999	0.553465	0.652263	0.764033	0.441231	0.313116
0.001452	3.65E-09	9.46E-05	1.05E-05	1.49E-11	9.81E-17	1.5E-25	2.08E-07	0.000338
0.087033	0.21988	-0.02633	0.098589	0.156179	0.20466	0.230367	-0.40905	-0.45887
0.330572	0.012995	0.768918	0.270139	0.07953	0.020994	0.009172	1.8E-06	5.77E-08
-0.37988	-0.16077	-0.73505	0.62301	-0.54181	-0.47611	-0.32893	-0.73329	-0.74116
1.06E-05	0.070979	7.73E-23	5.23E-15	4.76E-11	1.54E-08	0.000159	1.1E-22	2.22E-23
-0.45447	-0.46435	-0.55069	0.481361	-0.65089	-0.69394	-0.70192	-0.6374	-0.48137
8E-08	3.82E-08	1.98E-11	1.01E-08	1.19E-16	1.5E-19	3.82E-20	7.8E-16	1.01E-08
-0.34496	-0.33182	-0.55521	0.513922	-0.42341	-0.44196	-0.39139	-0.70112	-0.70175
7.14E-05	0.000138	1.25E-11	6.43E-10	7.06E-07	1.97E-07	5.36E-06	4.39E-20	3.94E-20
-0.17805	-0.06766	-0.28456	0.352788	-0.34284	-0.33381	-0.35412	-0.35987	-0.42178
0.045204	0.449781	0.001185	4.74E-05	7.96E-05	0.000125	4.42E-05	3.25E-05	7.86E-07

C1S	C2	C20orf203	C22orf39	C2orf80	C3	C3AR1	C3orf52	C4BPB
brown	brown	brown	brown	brown	brown	brown	brown	brown
0.215135	0.391488	-0.01769	0.051398	-0.10436	0.242632	0.335634	0.040839	0.164245
0.015141	5.33E-06	0.84353	0.56605	0.24293	0.005987	0.000115	0.648484	0.065009
0.090222	0.215763	0.050562	0.15903	0.028284	0.11485	0.23617	-0.02202	0.106728
0.313092	0.01484	0.572394	0.074124	0.752262	0.19853	0.007515	0.80585	0.232365
-0.19455	-0.3187	-0.07496	0.071777	0.231227	-0.36946	-0.45022	-0.03644	-0.17114
0.028393	0.00026	0.402251	0.422594	0.008908	1.91E-05	1.09E-07	0.684205	0.054386
0.175493	-0.16661	-0.33213	-0.01744	0.07739	-0.23423	-0.13509	0.217884	-0.04508
0.048439	0.061192	0.000136	0.845685	0.38714	0.008036	0.129943	0.013863	0.614803
0.066126	-0.06626	-0.06123	0.346732	0.133376	-0.15008	0.222668	0.046983	0.131741
0.460122	0.459244	0.494088	6.51E-05	0.13494	0.092144	0.011863	0.599916	0.139835
0.349915	-0.04451	-0.43987	0.114064	0.069047	-0.17338	0.226528	0.352781	0.122885
5.52E-05	0.619284	2.29E-07	0.201637	0.440499	0.051257	0.010438	4.74E-05	0.168705
0.230396	-0.03685	-0.13076	0.139948	0.082744	-0.04213	0.094143	0.289647	0.291186
0.009163	0.680851	0.142822	0.116584	0.355049	0.638148	0.292437	0.000957	0.000896
0.306266	0.146336	-0.35396	-0.04387	-0.19141	0.253038	0.33031	0.399328	0.180242
0.000462	0.100659	4.46E-05	0.624356	0.031101	0.004101	0.000149	3.31E-06	0.042583
0.572081	0.447106	-0.5207	-0.51856	-0.44538	0.286533	0.163433	0.365921	0.382957
2.13E-12	1.37E-07	3.49E-10	4.24E-10	1.55E-07	0.001091	0.066365	2.33E-05	8.84E-06
0.821407	0.855239	-0.52716	-0.53336	-0.66277	0.764591	0.861118	0.447258	0.557786
2.84E-32	1.72E-37	1.93E-10	1.07E-10	2.11E-17	1.32E-25	1.56E-38	1.35E-07	9.61E-12
0.718606	0.701474	-0.48181	-0.39116	-0.43355	0.555439	0.617325	0.39367	0.305178
1.88E-21	4.13E-20	9.75E-09	5.44E-06	3.55E-07	1.22E-11	1.08E-14	4.68E-06	0.000485
-0.00834	0.162131	0.251833	-0.08847	-0.23598	0.067502	-0.18789	-0.26233	-0.15132
0.925898	0.068588	0.004288	0.322614	0.007564	0.450818	0.0344	0.002888	0.089469
-0.22256	0.0193	0.258476	-0.2169	-0.17772	0.087441	-0.40881	-0.26334	-0.11013
0.011905	0.829483	0.003345	0.014309	0.045618	0.328304	1.83E-06	0.002777	0.217722
-0.16929	0.094056	0.197557	-0.17522	-0.23478	0.172457	-0.14937	-0.23478	7.99E-05
0.057078	0.292887	0.025992	0.048795	0.007887	0.052522	0.093726	0.007886	0.999288
0.321919	0.617961	-0.10159	-0.56112	-0.65209	0.647731	0.288756	0.02842	0.291404
0.000224	9.97E-15	0.25575	6.8E-12	1.01E-16	1.87E-16	0.000993	0.751112	0.000888
-0.2568	0.084583	0.319326	0.131645	0.065072	0.233334	0.22891	-0.35858	-0.08346
0.003564	0.34442	0.000253	0.140126	0.467323	0.008289	0.009635	3.48E-05	0.350858
-0.79961	-0.54062	0.612238	0.382212	0.555982	-0.38366	-0.60793	-0.57895	-0.45966
1.84E-29	5.35E-11	2.04E-14	9.24E-06	1.16E-11	8.49E-06	3.47E-14	1.01E-12	5.44E-08
-0.49419	-0.65295	0.223419	0.434691	0.667378	-0.63108	-0.39773	-0.15674	-0.42862
3.54E-09	8.89E-17	0.011573	3.28E-07	1.05E-17	1.82E-15	3.65E-06	0.078442	4.97E-07
-0.57696	-0.48056	0.493518	0.438526	0.546722	-0.37244	-0.33097	-0.32455	-0.32543
1.25E-12	1.08E-08	3.74E-09	2.51E-07	2.94E-11	1.62E-05	0.000144	0.000197	0.000189
-0.56109	-0.3412	0.436981	0.317173	0.299683	-0.49146	-0.49651	-0.35613	-0.16581
6.83E-12	8.65E-05	2.8E-07	0.00028	0.00062	4.44E-09	2.91E-09	3.97E-05	0.062463

C4orf3	C5AR1	C5AR2	C7	C9orf3	C9orf64	C9orf66	CABLES2	CACNA2D
brown	brown	brown	brown	brown	brown	brown	brown	brown
0.380392	0.199158	0.100213	0.253859	0.337019	-0.08415	0.108481	-0.22203	0.159593
1.03E-05	0.024784	0.262288	0.003978	0.000107	0.346902	0.224741	0.012113	0.073094
0.373634	0.106637	0.030593	0.174363	0.350275	-0.13398	0.047955	-0.1379	0.060029
1.51E-05	0.232767	0.732776	0.049927	5.41E-05	0.13318	0.592381	0.122084	0.5026
-0.43642	-0.21733	-0.36571	-0.35049	-0.48635	-0.05058	-0.28241	0.361497	-0.034
2.91E-07	0.014114	2.36E-05	5.35E-05	6.75E-09	0.572221	0.001296	2.97E-05	0.70435
-0.10117	0.021912	-0.19238	-0.04911	-0.24804	0.278538	-0.17952	0.18891	0.227298
0.257714	0.806821	0.030248	0.583492	0.004928	0.00152	0.043433	0.033416	0.010172
0.271268	0.004964	0.081437	0.155081	0.355653	-0.11645	0.097942	-0.11318	0.264392
0.002036	0.95583	0.362722	0.081694	4.07E-05	0.192317	0.27331	0.205191	0.002667
0.287761	0.16857	0.032328	0.220173	0.186935	0.29582	-0.01336	-0.00258	0.453233
0.001036	0.05816	0.718237	0.012872	0.035343	0.000734	0.881449	0.977049	8.76E-08
0.220279	0.054363	-0.04239	0.245014	0.069543	0.148261	0.072881	0.078383	0.310098
0.012827	0.543829	0.636043	0.005498	0.437215	0.096212	0.415478	0.381057	0.000388
0.221068	0.322662	0.118274	0.116435	0.406932	0.419073	0.086942	-0.26937	0.264365
0.012501	0.000216	0.185384	0.192364	2.06E-06	9.41E-07	0.331081	0.002195	0.00267
0.08084	0.484829	0.169208	0.366405	-0.11395	0.366694	0.157448	-0.04646	0.52858
0.366265	7.64E-09	0.057202	2.27E-05	0.202109	2.23E-05	0.077086	0.604002	1.69E-10
0.538601	0.746097	0.593147	0.622076	0.498705	0.394813	0.534884	-0.48081	0.811539
6.51E-11	7.91E-24	2.01E-13	5.9E-15	2.42E-09	4.36E-06	9.3E-11	1.06E-08	5.9E-31
0.421291	0.522544	0.262052	0.449855	0.276449	0.435362	0.266828	-0.37909	0.578048
8.13E-07	2.95E-10	0.002919	1.12E-07	0.001654	3.13E-07	0.002426	1.11E-05	1.11E-12
-0.24282	-0.13436	-0.31343	-0.08526	-0.21303	-0.2107	-0.07831	0.103367	-0.18479
0.005947	0.132056	0.000333	0.340555	0.01619	0.017416	0.381496	0.247487	0.037545
-0.49382	-0.1193	-0.22058	-0.34186	-0.45152	-0.1559	-0.05694	0.206317	-0.21115
3.64E-09	0.181576	0.012701	8.37E-05	9.93E-08	0.080066	0.524907	0.019956	0.017175
-0.23563	-0.03656	0.050716	-0.20278	-0.06683	-0.14367	0.182318	-0.10692	-0.07692
0.007658	0.683236	0.571225	0.022229	0.45533	0.107074	0.040216	0.23153	0.390011
0.038051	0.384891	0.295902	0.142845	0.112738	0.137399	0.392625	-0.27248	0.315833
0.671038	7.9E-06	0.000731	0.109136	0.206963	0.123458	4.98E-06	0.00194	0.000298
0.144276	-0.1953	0.063095	-0.04106	0.387914	-0.21838	0.199476	-0.24123	-0.25556
0.105595	0.027779	0.480987	0.646687	6.6E-06	0.013644	0.024551	0.006292	0.003734
-0.56927	-0.65346	-0.42625	-0.58392	-0.46103	-0.53745	-0.30918	0.197625	-0.75859
2.88E-12	8.27E-17	5.84E-07	5.78E-13	4.91E-08	7.27E-11	0.000405	0.02594	5.19E-25
-0.1427	-0.48951	-0.25954	-0.23756	-0.24186	-0.17119	-0.41617	0.246793	-0.57309
0.10951	5.21E-09	0.003213	0.00716	0.006155	0.054301	1.14E-06	0.005156	1.91E-12
-0.15375	-0.47746	-0.16067	-0.24071	-0.11208	-0.40147	-0.26703	0.149518	-0.67506
0.084382	1.38E-08	0.071156	0.00641	0.209661	2.9E-06	0.002407	0.093392	3.23E-18
-0.46097	-0.37821	-0.36365	-0.28262	-0.45383	-0.69231	-0.21238	0.337416	-0.23165
4.93E-08	1.17E-05	2.64E-05	0.001285	8.38E-08	1.98E-19	0.016523	0.000105	0.008782

CALCOCO	CAMK2N2	CAMP	CAMSAP1	CAMSAP2	CAND2	CAP1	CAPG	CAPZB
brown	brown	brown	brown	brown	brown	brown	brown	brown
0.214736	-0.20766	0.025081	-0.08307	-0.27893	0.02454	0.089169	0.231768	0.174331
0.015335	0.019148	0.779553	0.353176	0.001496	0.784194	0.318794	0.008745	0.04997
0.2215	-0.04862	0.064131	-0.03033	-0.19786	0.094719	0.026222	0.174026	0.076581
0.012326	0.587277	0.473797	0.734964	0.025762	0.289481	0.7698	0.050378	0.392136
-0.34909	0.104962	-0.17156	0.164428	0.382982	0.085729	-0.01134	-0.28423	-0.39381
5.76E-05	0.240228	0.053788	0.064707	8.83E-06	0.337899	0.899329	0.001202	4.64E-06
0.009377	-0.28005	0.019097	-0.17858	0.321704	-0.16488	0.287488	-0.16259	-0.44998
0.916668	0.001429	0.831249	0.044565	0.000226	0.063957	0.001048	0.06779	1.11E-07
0.024797	-0.1166	0.290154	-0.35477	-0.30508	-0.36545	0.148197	0.354975	-0.05955
0.78199	0.191716	0.000936	4.27E-05	0.000487	2.39E-05	0.096357	4.22E-05	0.506004
0.192659	-0.43686	0.298848	-0.48797	-0.01014	-0.46457	0.436892	0.178835	-0.35296
0.030002	2.82E-07	0.000643	5.91E-09	0.909948	3.76E-08	2.82E-07	0.044253	4.7E-05
0.173609	-0.21053	0.216089	-0.24264	-0.0413	-0.21303	0.34031	0.052072	-0.21161
0.050941	0.017512	0.014686	0.005986	0.644762	0.016187	9.05E-05	0.560962	0.016929
0.28745	-0.39982	0.259401	-0.3538	-0.20274	-0.35681	0.225178	0.27041	-0.0155
0.00105	3.21E-06	0.00323	4.49E-05	0.022258	3.83E-05	0.010918	0.002107	0.862671
0.353322	-0.405	0.270779	-0.34351	-0.29573	-0.28368	0.548404	0.119044	0.293742
4.61E-05	2.32E-06	0.002076	7.69E-05	0.000737	0.001229	2.49E-11	0.182519	0.000803
0.634586	-0.75805	0.555944	-0.66739	-0.78847	-0.62319	0.660003	0.757457	0.626089
1.14E-15	5.86E-25	1.16E-11	1.05E-17	3.72E-28	5.11E-15	3.18E-17	6.69E-25	3.51E-15
0.414109	-0.62823	0.265256	-0.57776	-0.45403	-0.37277	0.482764	0.512943	0.297353
1.3E-06	2.65E-15	0.002579	1.15E-12	8.26E-08	1.59E-05	9.03E-09	7.02E-10	0.000686
0.046921	0.373477	-0.21366	0.232027	-0.0686	0.432073	-0.03967	-0.21702	0.331359
0.600396	1.53E-05	0.015868	0.008668	0.443467	3.93E-07	0.657865	0.014253	0.000142
-0.20198	0.224143	-0.26054	0.384973	0.078904	0.360758	-0.22326	-0.23369	0.197631
0.022774	0.011299	0.003092	7.86E-06	0.37789	3.09E-05	0.011635	0.008187	0.025935
-0.21717	0.176063	-0.01586	0.017863	-0.33298	0.047205	-0.26822	0.269502	0.365121
0.014186	0.047703	0.859541	0.842	0.000131	0.598186	0.002297	0.002184	2.44E-05
0.288962	-0.28665	0.192043	-0.17892	-0.6099	-0.14318	0.162129	0.456205	0.71868
0.000985	0.001086	0.030542	0.044146	2.72E-14	0.108295	0.068592	7.04E-08	1.85E-21
0.115816	0.165549	-0.07849	0.184541	-0.16192	0.217661	-0.18594	0.183846	0.297403
0.194755	0.062879	0.380422	0.037804	0.06895	0.013963	0.036346	0.038543	0.000685
-0.61332	0.760508	-0.59333	0.742744	0.55132	0.644488	-0.63058	-0.49014	-0.18324
1.78E-14	3.37E-25	1.97E-13	1.6E-23	1.86E-11	2.94E-16	1.95E-15	4.95E-09	0.039202
-0.42156	0.44688	-0.36703	0.379062	0.764832	0.319869	-0.38984	-0.56194	-0.685
7.98E-07	1.39E-07	2.19E-05	1.11E-05	1.25E-25	0.000246	5.89E-06	6.25E-12	6.61E-19
-0.38648	0.64146	-0.38127	0.54168	0.461097	0.469411	-0.53111	-0.46825	-0.26669
7.19E-06	4.48E-16	9.76E-06	4.83E-11	4.88E-08	2.59E-08	1.33E-10	2.83E-08	0.002439
-0.38292	0.380596	-0.3064	0.544013	0.290205	0.355459	-0.21111	-0.47572	-0.16435
8.86E-06	1.01E-05	0.000459	3.84E-11	0.000934	4.11E-05	0.017194	1.58E-08	0.064832

CARD11	CARD16	CARD6	CASP1	CASP10	CASP4	CASP5	CASP8	CAST
brown	brown	brown	brown	brown	brown	brown	brown	brown
0.151854	0.213415	0.207643	0.211607	0.262074	0.135406	0.030147	0.026138	0.171991
0.088327	0.015992	0.019156	0.01693	0.002916	0.129049	0.736525	0.770514	0.053173
0.017957	0.090754	0.11766	0.056122	0.11152	-0.00106	-0.09743	-0.1024	0.079568
0.841182	0.310237	0.187694	0.530856	0.211945	0.990582	0.275841	0.251977	0.373878
-0.35914	0.02696	-0.13797	-0.00863	-0.31818	0.028542	0.249351	0.209351	-0.28879
3.38E-05	0.763511	0.121892	0.923246	0.000267	0.750081	0.004698	0.018168	0.000992
-0.36332	0.37909	0.207844	0.40643	0.044359	0.382605	0.506529	0.620743	0.214604
2.69E-05	1.11E-05	0.019038	2.12E-06	0.620455	9.03E-06	1.23E-09	7E-15	0.015399
-0.17574	0.377907	0.072651	0.307641	0.041481	0.330252	0.116242	0.16847	-0.06546
0.048123	1.19E-05	0.416954	0.000434	0.643332	0.000149	0.193106	0.058313	0.464653
-0.33691	0.658398	0.356286	0.662958	0.228933	0.637555	0.535884	0.708317	0.347176
0.000107	4.03E-17	3.94E-05	2.05E-17	0.009628	7.64E-16	8.45E-11	1.23E-20	6.36E-05
-0.2537	0.428965	0.246875	0.395317	0.239063	0.425168	0.277365	0.403135	0.279565
0.004002	4.86E-07	0.005141	4.23E-06	0.006793	6.28E-07	0.001594	2.61E-06	0.001457
0.083466	0.31107	0.199357	0.3588	0.2554	0.313263	0.137377	0.369588	0.46249
0.350851	0.000371	0.024638	3.44E-05	0.003756	0.000336	0.12352	1.9E-05	4.4E-08
0.195609	0.41041	0.522251	0.385632	0.404807	0.56212	0.467412	0.548944	0.509886
0.027528	1.65E-06	3.03E-10	7.56E-06	2.35E-06	6.13E-12	3.02E-08	2.36E-11	9.19E-10
0.685813	0.758823	0.773914	0.779435	0.823777	0.791329	0.507376	0.65366	0.762363
5.79E-19	4.92E-25	1.45E-26	3.74E-27	1.33E-32	1.75E-28	1.15E-09	8.03E-17	2.21E-25
0.414458	0.73187	0.629842	0.809886	0.695248	0.702015	0.534286	0.684783	0.633246
1.27E-06	1.46E-22	2.15E-15	9.64E-31	1.2E-19	3.76E-20	9.85E-11	6.84E-19	1.37E-15
0.141154	-0.30023	-0.00202	-0.28609	-0.08522	-0.25917	-0.21044	-0.2557	-0.05611
0.113437	0.000605	0.982028	0.001112	0.340763	0.003259	0.01756	0.003714	0.530944
0.20419	-0.37593	-0.19686	-0.41534	-0.22012	-0.34131	-0.14668	-0.2556	-0.27059
0.021298	1.33E-05	0.026534	1.2E-06	0.012893	8.61E-05	0.099857	0.003727	0.002092
0.348592	-0.22632	-0.23197	-0.29336	-0.16505	-0.21333	-0.22103	-0.31715	-0.30947
5.91E-05	0.01051	0.008685	0.000816	0.063691	0.016034	0.012516	0.00028	0.000399
0.739465	0.11008	0.290798	0.08172	0.375317	0.166436	0.051112	0.069716	0.252337
3.15E-23	0.217939	0.000911	0.361055	1.37E-05	0.061461	0.568219	0.436077	0.004209
0.334911	-0.30383	-0.10524	-0.28271	0.054048	-0.39381	-0.42746	-0.48504	-0.17967
0.000119	0.000515	0.238981	0.00128	0.546168	4.64E-06	5.38E-07	7.52E-09	0.043256
-0.21651	-0.81021	-0.68076	-0.80307	-0.64509	-0.83496	-0.54729	-0.79556	-0.82989
0.014491	8.76E-31	1.31E-18	6.94E-30	2.71E-16	3.2E-34	2.78E-11	5.59E-29	1.79E-33
-0.62001	-0.41412	-0.45482	-0.34534	-0.47355	-0.4597	-0.26238	-0.33178	-0.37943
7.68E-15	1.3E-06	7.8E-08	7E-05	1.88E-08	5.43E-08	0.002882	0.000139	1.09E-05
-0.32107	-0.67651	-0.57041	-0.64505	-0.49391	-0.71039	-0.58116	-0.76834	-0.51304
0.000233	2.57E-18	2.55E-12	2.72E-16	3.62E-09	8.5E-21	7.87E-13	5.5E-26	6.95E-10
-0.27642	-0.29806	-0.29967	-0.40338	-0.36505	-0.32135	-0.16173	-0.30893	-0.63626
0.001656	0.000665	0.00062	2.57E-06	2.45E-05	0.00023	0.069278	0.000409	9.11E-16

CAT	CAV2	CC2D1B	CCDC144A	CCDC69	CCL13	CCL18	CCL2	CCL20
brown	brown	brown	brown	brown	brown	brown	brown	brown
0.115908	0.174508	0.245576	-0.01307	0.39733	0.134101	0.376038	0.304597	-0.07969
0.194395	0.049734	0.005388	0.884031	3.74E-06	0.132814	1.32E-05	0.000498	0.373165
0.031322	0.069013	0.179614	-0.02257	0.307578	0.029123	0.304721	0.180363	-0.13537
0.726657	0.440729	0.043321	0.801181	0.000435	0.745166	0.000495	0.042441	0.129155
-0.15035	-0.15496	-0.25478	0.132824	-0.73374	-0.16668	-0.26515	-0.26372	0.051938
0.091571	0.081935	0.003844	0.136578	1E-22	0.061079	0.002589	0.002737	0.561974
0.23777	0.112832	-0.19803	0.09607	-0.43209	0.023663	-0.11728	0.118958	0.325953
0.007108	0.206582	0.025632	0.282628	3.93E-07	0.791725	0.189123	0.182838	0.000184
0.241456	0.081557	-0.10654	-0.47238	0.239098	0.095067	0.158414	0.111896	0.157603
0.006243	0.362015	0.233194	2.06E-08	0.006785	0.287704	0.075266	0.210397	0.076792
0.490648	0.270149	-0.16684	-0.27806	0.013018	0.196493	0.111563	0.356809	0.4258
4.74E-09	0.002129	0.060827	0.00155	0.8845	0.026822	0.211766	3.83E-05	6.02E-07
0.238918	0.187392	-0.08646	-0.0712	0.109383	0.135243	0.073258	0.271795	0.333395
0.006828	0.034889	0.333771	0.42633	0.220886	0.129514	0.413062	0.001994	0.000128
0.468515	0.016588	0.148937	-0.30216	0.384753	0.0874	0.049002	0.314323	0.159757
2.78E-08	0.853145	0.094687	0.000555	7.96E-06	0.328529	0.584312	0.00032	0.072795
0.181351	0.447999	0.270695	-0.16702	-0.0986	0.501102	0.290781	0.401804	0.510498
0.041303	1.28E-07	0.002083	0.060545	0.270067	1.97E-09	0.000912	2.84E-06	8.71E-10
0.587308	0.533401	0.662262	-0.5297	0.696299	0.646757	0.693519	0.793797	0.533982
3.94E-13	1.07E-10	2.28E-17	1.52E-10	1.01E-19	2.14E-16	1.61E-19	9.02E-29	1.01E-10
0.514695	0.405102	0.448798	-0.27688	0.465125	0.453109	0.49638	0.727846	0.342254
6E-10	2.31E-06	1.21E-07	0.001625	3.6E-08	8.84E-08	2.94E-09	3.22E-22	8.2E-05
-0.13852	-0.04084	0.202595	0.30202	-0.24638	-0.069	-0.11386	-0.16612	-0.12865
0.120395	0.648458	0.022354	0.000559	0.005234	0.44078	0.202457	0.061965	0.149461
-0.40188	-0.2253	0.229701	0.286821	-0.48665	-0.1309	-0.12919	-0.31305	-0.14609
2.82E-06	0.010873	0.009381	0.001078	6.59E-09	0.142413	0.147732	0.000339	0.101241
-0.26575	-0.14605	0.302982	-0.22235	-0.11278	0.009972	0.095579	-0.19069	-0.08978
0.00253	0.101338	0.000535	0.011988	0.206805	0.911406	0.285104	0.031758	0.31546
0.043819	0.193466	0.740952	-0.25042	0.210584	0.31691	0.400238	0.251963	0.139708
0.624722	0.029307	2.32E-23	0.004518	0.017482	0.000283	3.13E-06	0.004268	0.117218
-0.09434	-0.04281	0.31745	0.020256	0.409196	-0.26475	0.067984	-0.14223	-0.49181
0.291445	0.632726	0.000276	0.821168	1.78E-06	0.002631	0.447585	0.110694	4.31E-09
-0.59646	-0.48695	-0.3539	0.469307	-0.46867	-0.65289	-0.52714	-0.73715	-0.62189
1.37E-13	6.43E-09	4.47E-05	2.61E-08	2.74E-08	8.97E-17	1.93E-10	5.06E-23	6.04E-15
-0.24635	-0.28992	-0.7612	0.42991	-0.2384	-0.43145	-0.47687	-0.41886	-0.34905
0.00524	0.000946	2.87E-25	4.56E-07	0.006953	4.1E-07	1.45E-08	9.54E-07	5.77E-05
-0.44247	-0.40417	-0.53494	0.374587	0.019682	-0.42599	-0.40405	-0.48675	-0.51616
1.9E-07	2.45E-06	9.25E-11	1.43E-05	0.82616	5.94E-07	2.47E-06	6.54E-09	5.26E-10
-0.43042	-0.18193	-0.27295	0.24424	-0.54629	-0.26274	-0.25979	-0.57395	-0.16985
4.4E-07	0.040654	0.001904	0.005653	3.07E-11	0.002842	0.003182	1.74E-12	0.056256

CCL22	CCL23	CCL5	CCL7	CCL8	CCNG2	CCR1	CCR2	CCR4
brown	brown	brown	brown	brown	brown	brown	brown	brown
0.133589	0.154565	0.431877	0.058968	0.452448	-0.04757	0.338254	0.22827	0.292787
0.134314	0.082726	3.98E-07	0.510196	9.28E-08	0.595386	0.0001	0.009845	0.000836
0.086832	0.09384	0.250368	0.056466	0.292581	-0.00471	0.139249	0.060568	0.114115
0.331694	0.294	0.004526	0.528335	0.000844	0.958048	0.118439	0.498762	0.201434
-0.17375	-0.12244	-0.23547	-0.04098	-0.24785	0.127209	-0.23357	-0.06908	-0.14322
0.050756	0.170256	0.007701	0.647335	0.004963	0.154101	0.008223	0.44027	0.108204
-0.06567	-0.01922	-0.01191	0.183618	0.101368	-0.001	0.074223	0.258359	0.078322
0.463202	0.830151	0.894256	0.03879	0.256801	0.991087	0.40692	0.00336	0.381433
0.077625	0.214158	0.205798	0.194482	0.205643	0.117406	0.004487	0.20817	0.102119
0.385693	0.015619	0.020276	0.028452	0.020373	0.188654	0.96007	0.018847	0.253275
0.055119	0.19765	0.249507	0.367103	0.372847	0.020238	0.214764	0.486548	0.262801
0.538234	0.025921	0.004671	2.18E-05	1.58E-05	0.821327	0.015321	6.65E-09	0.002836
0.111332	0.273093	0.194559	0.35646	0.300818	-0.09654	0.101445	0.230402	0.114638
0.212719	0.001894	0.028389	3.9E-05	0.000589	0.28025	0.256436	0.009161	0.199365
0.112211	-0.02881	0.13611	0.072034	0.320499	-0.38714	0.246199	0.252967	0.118524
0.209107	0.747825	0.127053	0.420931	0.000239	6.91E-06	0.005268	0.004112	0.18445
0.179474	0.436507	0.443527	0.491695	0.354993	-0.30779	0.387551	0.450596	0.471247
0.043488	2.89E-07	1.77E-07	4.35E-09	4.22E-05	0.000431	6.75E-06	1.06E-07	2.25E-08
0.528749	0.60246	0.870051	0.501153	0.764799	-0.50003	0.885099	0.748448	0.728072
1.66E-10	6.72E-14	3.26E-40	1.96E-09	1.26E-25	2.16E-09	2.42E-43	4.8E-24	3.08E-22
0.300674	0.340617	0.693148	0.294777	0.765372	-0.43911	0.795293	0.658724	0.610403
0.000593	8.91E-05	1.72E-19	0.000768	1.1E-25	2.41E-07	6.02E-29	3.84E-17	2.56E-14
-0.03015	-0.04377	-0.13436	-0.13612	-0.1689	-0.09102	-0.13217	-0.30639	-0.11884
0.736471	0.625082	0.132047	0.127017	0.057657	0.308788	0.138544	0.000459	0.183289
-0.02546	-0.10579	-0.1978	-0.19797	-0.34095	-0.11905	-0.1347	-0.33178	-0.20898
0.776271	0.236522	0.025806	0.025677	8.76E-05	0.182491	0.131088	0.000139	0.018377
0.083934	0.065707	-0.03363	-0.12499	-0.24834	-0.10741	-0.06966	-0.21323	-0.11911
0.348146	0.462977	0.70739	0.161456	0.004875	0.22937	0.436424	0.016086	0.182274
0.349865	0.323205	0.418599	0.108518	0.169086	-0.44168	0.449655	0.153074	0.270255
5.53E-05	0.00021	9.7E-07	0.224584	0.057385	2.01E-07	1.14E-07	0.085771	0.00212
0.037002	-0.12695	-0.02388	-0.39765	-0.12796	-0.01698	0.011742	-0.27274	-0.20338
0.679601	0.154955	0.789877	3.67E-06	0.151669	0.849693	0.895758	0.00192	0.021832
-0.37655	-0.52439	-0.6943	-0.6365	-0.69586	0.333587	-0.62913	-0.78517	-0.6883
1.28E-05	2.49E-10	1.41E-19	8.82E-16	1.08E-19	0.000127	2.36E-15	8.74E-28	3.85E-19
-0.39486	-0.48894	-0.58311	-0.33084	-0.3934	0.519543	-0.54757	-0.33225	-0.37676
4.35E-06	5.46E-09	6.33E-13	0.000145	4.75E-06	3.88E-10	2.7E-11	0.000136	1.27E-05
-0.34003	-0.43526	-0.5552	-0.3986	-0.42741	0.398799	-0.56727	-0.55308	-0.44586
9.18E-05	3.15E-07	1.25E-11	3.46E-06	5.4E-07	3.42E-06	3.56E-12	1.55E-11	1.5E-07
-0.08546	-0.08701	-0.25572	-0.15249	-0.35886	0.426911	-0.41738	-0.3363	-0.25382
0.339446	0.330692	0.003711	0.08699	3.43E-05	5.58E-07	1.05E-06	0.000111	0.003984

CCR5	CCR7	CCRL2	CD109	CD14	CD163	CD163L1	CD180	CD19
brown	brown	brown	brown	brown	brown	brown	brown	brown
0.248747	0.268022	0.301259	0.156335	0.237327	0.119534	0.38052	0.276819	0.094692
0.004803	0.002315	0.000578	0.079227	0.007219	0.180713	1.02E-05	0.00163	0.289621
0.016746	0.139172	0.202116	0.073823	0.094175	0.072889	0.382441	0.126209	0.031251
0.851768	0.118644	0.02268	0.409462	0.292273	0.415426	9.12E-06	0.15739	0.727248
-0.06985	-0.09913	-0.17823	-0.19329	-0.30727	-0.10258	-0.19436	-0.01	-0.09602
0.435169	0.267499	0.044987	0.029462	0.000442	0.251129	0.028556	0.911145	0.282867
0.219184	0.057987	0.035464	0.208363	-0.21005	0.107107	-0.0371	0.169857	-0.13614
0.013293	0.517268	0.692229	0.018734	0.017775	0.230704	0.678814	0.056241	0.126974
0.016388	0.232332	0.214343	-0.08416	0.076741	0.054196	0.382161	0.128225	0.000457
0.854904	0.008578	0.015528	0.346833	0.391143	0.545071	9.27E-06	0.150812	0.995929
0.311746	0.264021	0.27582	0.305124	-0.02972	0.229999	0.312563	0.292433	-0.11251
0.00036	0.002706	0.001697	0.000486	0.740093	0.009287	0.000347	0.000849	0.207884
0.168865	0.274687	0.240709	0.152146	-0.04534	0.125255	0.251487	0.107082	-0.03657
0.057715	0.001776	0.00641	0.087711	0.612778	0.160578	0.004343	0.230811	0.683131
0.2465	0.123591	0.042331	0.569322	0.168792	0.177915	0.069539	0.162633	-0.23393
0.005211	0.166252	0.636539	2.86E-12	0.057826	0.045373	0.43724	0.067724	0.008121
0.538829	0.400416	0.332053	0.422569	0.452782	0.604533	0.212687	0.268383	0.172093
6.37E-11	3.09E-06	0.000137	7.47E-07	9.06E-08	5.24E-14	0.016364	0.002282	0.05303
0.881485	0.727436	0.775287	0.657741	0.874147	0.719996	0.551479	0.704985	0.431594
1.49E-42	3.49E-22	1.04E-26	4.44E-17	5.03E-41	1.45E-21	1.83E-11	2.23E-20	4.06E-07
0.798263	0.541423	0.56636	0.575562	0.565258	0.412061	0.3071	0.634582	0.226332
2.67E-29	4.95E-11	3.93E-12	1.46E-12	4.41E-12	1.48E-06	0.000445	1.14E-15	0.010506
-0.14398	-0.18127	-0.07042	-0.22801	0.012188	-0.06351	-0.15605	-0.15892	0.063072
0.106324	0.041394	0.431467	0.009931	0.891821	0.478084	0.079785	0.074322	0.481145
-0.08744	-0.1326	-0.18831	-0.21273	-0.0049	-0.08695	-0.33156	-0.07372	0.163832
0.328296	0.137239	0.033996	0.016344	0.956357	0.331009	0.00014	0.410121	0.065695
-0.09694	0.032578	-0.05536	-0.24928	0.298381	-0.00225	-0.18738	-0.01271	0.216109
0.278251	0.716154	0.536434	0.004711	0.000656	0.979981	0.034905	0.887226	0.014677
0.436323	0.355264	0.348859	0.22744	0.67417	0.395456	0.070817	0.364759	0.44212
2.93E-07	4.16E-05	5.83E-05	0.010124	3.71E-18	4.19E-06	0.428842	2.48E-05	1.95E-07
-0.18725	-0.15146	0.035042	-0.20861	0.069328	-0.192	0.033655	0.016466	0.082866
0.035034	0.089157	0.695706	0.018594	0.438639	0.030578	0.707195	0.854219	0.354334
-0.70373	-0.57607	-0.56128	-0.74884	-0.51675	-0.64533	-0.52424	-0.49963	-0.15841
2.78E-20	1.38E-12	6.7E-12	4.41E-24	4.99E-10	2.62E-16	2.52E-10	2.23E-09	0.075271
-0.58057	-0.53361	-0.50748	-0.33201	-0.71783	-0.51987	-0.29323	-0.49923	-0.42561
8.4E-13	1.05E-10	1.14E-09	0.000137	2.17E-21	3.77E-10	0.000821	2.31E-09	6.09E-07
-0.68961	-0.54245	-0.52351	-0.46826	-0.51653	-0.56244	-0.25841	-0.61206	-0.28121
3.1E-19	4.48E-11	2.7E-10	2.83E-08	5.09E-10	5.93E-12	0.003354	2.09E-14	0.001362
-0.31209	-0.13858	-0.13781	-0.62903	-0.38066	-0.25702	0.012697	-0.20102	0.061542
0.000354	0.120237	0.122329	2.39E-15	1.01E-05	0.003535	0.887337	0.023444	0.49187

CD1C	CD1D	CD2	CD200R1	CD209	CD244	CD247	CD27	CD300A
brown	brown	brown	brown	brown	brown	brown	brown	brown
0.171229	0.08127	0.343514	0.352893	0.298796	0.281896	0.349232	0.310411	0.241348
0.054253	0.36371	7.69E-05	4.72E-05	0.000644	0.001324	5.72E-05	0.000383	0.006267
0.043925	-0.06508	0.171028	0.178316	0.17392	0.177292	0.156401	0.149202	0.114564
0.623884	0.467286	0.05454	0.044882	0.050521	0.046146	0.079098	0.094094	0.199655
-0.16074	0.01786	-0.12053	-0.04642	-0.17861	-0.20314	-0.21559	-0.16728	-0.17201
0.071033	0.842029	0.177097	0.604274	0.044526	0.021985	0.014922	0.060134	0.053151
0.049735	0.201353	0.180515	0.326349	0.082611	0.068896	-0.04917	-0.12288	-0.02145
0.578696	0.023208	0.042265	0.000181	0.35582	0.441502	0.582997	0.168716	0.810783
0.09314	0.122557	0.228778	0.196631	0.026793	0.280831	0.185497	0.092445	0.257894
0.297633	0.169855	0.009678	0.026713	0.764935	0.001383	0.036804	0.30127	0.00342
0.203893	0.315771	0.419872	0.543761	0.239361	0.361969	0.17406	0.035057	0.222613
0.021491	0.000299	8.92E-07	3.94E-11	0.006722	2.9E-05	0.050332	0.695589	0.011884
0.220824	0.119173	0.258349	0.334307	0.170825	0.278149	0.03898	0.025128	0.146232
0.012601	0.182041	0.003361	0.000122	0.054833	0.001544	0.66349	0.779151	0.100903
0.113519	0.228743	0.196465	0.333085	0.165594	0.146381	0.025333	0.030666	0.193594
0.203815	0.00969	0.026844	0.00013	0.062805	0.100553	0.777397	0.732164	0.029198
0.290983	0.584778	0.468568	0.391879	0.532367	0.205977	0.276474	0.361894	0.436937
0.000904	5.25E-13	2.77E-08	5.21E-06	1.18E-10	0.020165	0.001653	2.91E-05	2.81E-07
0.604086	0.817634	0.830731	0.676081	0.7093	0.534849	0.66204	0.653507	0.855287
5.53E-14	9.26E-32	1.35E-33	2.75E-18	1.03E-20	9.33E-11	2.35E-17	8.21E-17	1.69E-37
0.382804	0.587867	0.731447	0.662031	0.537421	0.475179	0.592439	0.528719	0.585562
8.92E-06	3.7E-13	1.59E-22	2.36E-17	7.29E-11	1.65E-08	2.19E-13	1.67E-10	4.8E-13
-0.1816	-0.10181	-0.12794	-0.37423	-0.1248	-0.15593	-0.06202	0.040861	-0.1284
0.041023	0.254706	0.151718	1.46E-05	0.162112	0.080024	0.488503	0.648303	0.15024
-0.16532	-0.05682	-0.25431	-0.37507	-0.16303	-0.44791	-0.16889	0.008984	-0.12259
0.063248	0.525718	0.003912	1.39E-05	0.067046	1.29E-07	0.057674	0.920146	0.169754
-0.11187	0.00233	-0.14996	-0.33917	-0.15974	-0.23134	0.010982	0.155141	0.155388
0.210494	0.979257	0.092425	9.59E-05	0.072832	0.008873	0.902474	0.081575	0.081085
0.226706	0.433063	0.294218	0.03026	0.293781	-0.0309	0.31551	0.47989	0.492203
0.010376	3.67E-07	0.000786	0.735572	0.000801	0.730186	0.000302	1.14E-08	4.17E-09
-0.04961	-0.24258	-0.15704	-0.32691	-0.1831	-0.09954	-0.04888	-0.04566	-0.08165
0.579663	0.005998	0.077868	0.000176	0.039353	0.265498	0.585259	0.610272	0.361472
-0.47002	-0.66612	-0.76809	-0.78704	-0.69373	-0.50044	-0.5519	-0.49676	-0.61166
2.47E-08	1.28E-17	5.84E-26	5.39E-28	1.56E-19	2.08E-09	1.75E-11	2.85E-09	2.19E-14
-0.32099	-0.60412	-0.50321	-0.22456	-0.42341	-0.07444	-0.41779	-0.55724	-0.67348
0.000234	5.51E-14	1.64E-09	0.011145	7.06E-07	0.40555	1.02E-06	1.02E-11	4.13E-18
-0.36105	-0.69809	-0.62823	-0.5009	-0.47224	-0.18227	-0.40702	-0.44034	-0.59308
3.04E-05	7.41E-20	2.65E-15	2E-09	2.08E-08	0.040267	2.05E-06	2.21E-07	2.03E-13
-0.1489	-0.20452	-0.26769	-0.3002	-0.2891	-0.36287	-0.11655	-0.12438	-0.26488
0.094781	0.021084	0.002345	0.000606	0.000979	2.76E-05	0.19191	0.163548	0.002617

CD300C	CD300E	CD300LB	CD300LF	CD33	CD36	CD37	CD3D	CD3E
brown	brown	brown	brown	brown	brown	brown	brown	brown
0.195776	0.127984	0.242862	0.270611	0.27223	0.042465	0.231523	0.332587	0.340955
0.027394	0.151587	0.005938	0.00209	0.00196	0.635471	0.008818	0.000133	8.76E-05
0.070767	-0.00643	0.132806	0.131231	0.138102	-0.01802	0.086002	0.168203	0.149094
0.429172	0.942845	0.136632	0.141388	0.121534	0.840617	0.336353	0.058718	0.094337
-0.09154	-0.03551	-0.10482	-0.23172	-0.1728	0.043922	-0.49436	-0.18521	-0.21132
0.306063	0.691889	0.240868	0.00876	0.052044	0.623908	3.48E-09	0.037097	0.017084
0.089106	0.214246	0.013887	-0.06818	0.108761	0.235868	-0.40246	0.033239	-0.01085
0.319137	0.015576	0.876851	0.446278	0.223542	0.007594	2.73E-06	0.710654	0.903619
0.189892	-0.02413	-0.02142	0.279534	0.274748	0.121905	-0.07772	0.291445	0.118042
0.032492	0.787726	0.811071	0.001459	0.001772	0.172153	0.3851	0.000886	0.186254
0.290083	0.285769	0.099899	0.200691	0.386236	0.354975	-0.2621	0.328459	0.182909
0.000939	0.001127	0.263793	0.023674	7.29E-06	4.22E-05	0.002914	0.000163	0.039562
0.23399	0.224508	0.075971	0.133687	0.232351	0.250872	-0.19506	0.215749	0.149045
0.008104	0.011163	0.39593	0.134027	0.008573	0.004443	0.027975	0.014847	0.094445
0.157574	0.161793	0.137724	0.176021	0.270724	0.400361	0.108911	0.122413	0.102464
0.076846	0.069175	0.122567	0.047757	0.002081	3.1E-06	0.222899	0.170362	0.251665
0.44027	0.61541	0.392003	0.251777	0.379431	0.482981	0.150016	0.419358	0.468869
2.22E-07	1.37E-14	5.17E-06	0.004297	1.09E-05	8.88E-09	0.092294	9.23E-07	2.7E-08
0.753367	0.611729	0.595996	0.814254	0.888763	0.50593	0.79779	0.832172	0.826744
1.65E-24	2.17E-14	1.45E-13	2.61E-31	3.59E-44	1.3E-09	3.04E-29	8.32E-34	5.09E-33
0.557342	0.538453	0.408049	0.554618	0.654372	0.357966	0.507404	0.652122	0.671184
1.01E-11	6.6E-11	1.92E-06	1.33E-11	7.24E-17	3.6E-05	1.14E-09	1E-16	5.89E-18
-0.11426	-0.12937	-0.10141	-0.10218	-0.23369	-0.27446	0.071026	-0.12823	-0.0158
0.200866	0.147166	0.256619	0.252973	0.008186	0.001793	0.42748	0.150781	0.860059
-0.1417	-0.11305	-0.03143	-0.1689	-0.26797	-0.18907	0.051547	-0.24839	-0.11432
0.112023	0.205704	0.725773	0.057662	0.00232	0.03326	0.564921	0.004865	0.200631
-0.021	-0.2064	-0.07547	0.12493	-0.06023	-0.16828	0.300894	-0.05168	0.011504
0.814753	0.019904	0.399054	0.161674	0.501153	0.058605	0.000587	0.563887	0.897858
0.334732	0.213659	0.312528	0.44938	0.354849	0.116225	0.723995	0.345865	0.458491
0.00012	0.015869	0.000347	1.16E-07	4.25E-05	0.193173	6.77E-22	6.81E-05	5.94E-08
-0.24852	-0.41208	-0.16043	0.144618	-0.07749	-0.25301	0.402218	-0.06036	-0.03876
0.004843	1.48E-06	0.071574	0.104764	0.386544	0.004105	2.77E-06	0.500238	0.665258
-0.64578	-0.65213	-0.47428	-0.54281	-0.71713	-0.51335	-0.3154	-0.70227	-0.64806
2.46E-16	1E-16	1.77E-08	4.32E-11	2.47E-21	6.77E-10	0.000304	3.6E-20	1.78E-16
-0.52296	-0.37429	-0.40171	-0.58338	-0.56089	-0.26939	-0.62458	-0.54417	-0.58244
2.84E-10	1.46E-05	2.86E-06	6.14E-13	6.97E-12	0.002193	4.27E-15	3.78E-11	6.82E-13
-0.54261	-0.45765	-0.36528	-0.52119	-0.60978	-0.42179	-0.32421	-0.55832	-0.55937
4.41E-11	6.32E-08	2.41E-05	3.34E-10	2.76E-14	7.86E-07	0.0002	9.09E-12	8.16E-12
-0.20298	-0.25656	-0.13095	-0.25279	-0.36415	-0.18079	-0.40092	-0.239	-0.25898
0.022094	0.003596	0.142251	0.004139	2.57E-05	0.041948	3E-06	0.006807	0.003281

CD3G	CD4	CD40	CD40LG	CD48	CD5	CD52	CD53	CD55
brown	brown	brown	brown	brown	brown	brown	brown	brown
0.284311	0.276899	0.320511	0.343782	0.389374	0.416056	0.487478	0.288017	0.442353
0.001198	0.001624	0.000239	7.58E-05	6.05E-06	1.15E-06	6.16E-09	0.001025	1.92E-07
0.085985	0.100103	0.184274	0.165613	0.200846	0.266425	0.382638	0.168613	0.43409
0.336453	0.262815	0.038086	0.062775	0.023564	0.002464	9.01E-06	0.058095	3.42E-07
-0.17676	-0.34634	-0.12994	-0.14616	-0.19064	-0.4008	-0.29941	-0.37191	-0.67132
0.04682	6.65E-05	0.145375	0.101077	0.031802	3.02E-06	0.000627	1.67E-05	5.77E-18
0.108438	-0.1591	0.042174	0.002736	0.129483	-0.20717	-0.08733	-0.06904	-0.36382
0.224926	0.074005	0.63779	0.975649	0.146812	0.019436	0.32892	0.440558	2.62E-05
0.093091	-0.07413	0.209638	0.031782	0.28354	0.086233	0.477017	0.24162	0.190925
0.297887	0.407529	0.018006	0.722804	0.001237	0.335051	1.43E-08	0.006207	0.031542
0.292235	-0.03129	0.259194	0.106625	0.415917	0.051236	0.352964	0.270085	0.041471
0.000857	0.726918	0.003255	0.232819	1.16E-06	0.567279	4.7E-05	0.002134	0.643416
0.100924	0.035817	0.297459	0.170237	0.270078	0.067596	0.298186	0.176104	0.129543
0.258899	0.689321	0.000683	0.055684	0.002135	0.45019	0.000662	0.047651	0.146622
0.12451	0.197497	0.018095	0.012388	0.301488	0.1617	0.079508	0.273556	0.141832
0.163102	0.026038	0.839977	0.890055	0.000572	0.069336	0.37424	0.001859	0.111698
0.380617	0.486646	0.577042	0.384553	0.369774	0.277756	0.229346	0.283393	0.081379
1.01E-05	6.59E-09	1.24E-12	8.05E-06	1.88E-05	0.001569	0.009495	0.001244	0.363065
0.743315	0.912199	0.816591	0.661195	0.845016	0.719117	0.70737	0.925011	0.546694
1.42E-23	2.86E-50	1.28E-31	2.67E-17	8.76E-36	1.71E-21	1.46E-20	2.24E-54	2.95E-11
0.655833	0.652116	0.618041	0.622442	0.78132	0.601198	0.495212	0.662176	0.24782
5.86E-17	1E-16	9.87E-15	5.63E-15	2.33E-27	7.81E-14	3.24E-09	2.31E-17	0.004967
-0.16464	0.002895	-0.01715	0.043701	-0.10756	-0.06036	-0.1451	-0.18975	-0.10634
0.064356	0.974233	0.84825	0.62566	0.228741	0.500257	0.103587	0.032626	0.234093
-0.22586	-0.01914	-0.12289	-0.02723	-0.30885	-0.24144	-0.44138	-0.35613	-0.47135
0.010674	0.830903	0.168683	0.761171	0.000411	0.006247	2.06E-07	3.97E-05	2.23E-08
-0.1317	0.05163	0.000368	-0.01696	-0.12008	-0.05359	-0.11729	-0.09949	-0.17493
0.139959	0.564296	0.996726	0.849891	0.178697	0.549606	0.189105	0.265743	0.04917
0.274985	0.625044	0.43169	0.382984	0.276103	0.32947	0.124294	0.358531	0.116784
0.001755	4.02E-15	4.03E-07	8.83E-06	0.001678	0.000155	0.163838	3.49E-05	0.191023
-0.10821	0.110549	-0.09785	-0.10575	-0.1365	0.040337	0.024721	0.137239	0.39607
0.225904	0.215977	0.273749	0.236719	0.125969	0.65252	0.782639	0.1239	4.04E-06
-0.67263	-0.55093	-0.63189	-0.50584	-0.7489	-0.55094	-0.63368	-0.65836	-0.46784
4.71E-18	1.93E-11	1.64E-15	1.31E-09	4.35E-24	1.93E-11	1.29E-15	4.05E-17	2.93E-08
-0.36758	-0.65087	-0.6256	-0.45973	-0.48843	-0.3659	-0.34775	-0.49333	-0.13903
2.13E-05	1.2E-16	3.74E-15	5.41E-08	5.7E-09	2.33E-05	6.18E-05	3.8E-09	0.119017
-0.48276	-0.47846	-0.60201	-0.43385	-0.58854	-0.24345	-0.31628	-0.44394	0.041143
9.03E-09	1.28E-08	7.09E-14	3.48E-07	3.42E-13	0.005816	0.000292	1.72E-07	0.646038
-0.30411	-0.38273	-0.12544	-0.17686	-0.34965	-0.37853	-0.14931	-0.44422	-0.33037
0.000509	8.96E-06	0.159971	0.046694	5.59E-05	1.14E-05	0.093844	1.68E-07	0.000149

CD59	CD5L	CD6	CD69	CD7	CD72	CD74	CD79A	CD79B
brown	brown	brown	brown	brown	brown	brown	brown	brown
0.425151	0.271991	0.393847	0.129148	0.097014	0.267696	0.327729	0.162085	0.200689
6.28E-07	0.001979	4.63E-06	0.14787	0.2779	0.002345	0.000169	0.068667	0.023675
0.43849	0.16134	0.254507	-0.00297	-0.03964	0.146446	0.154857	0.036677	0.123772
2.52E-07	0.069968	0.003883	0.97356	0.658115	0.1004	0.08214	0.682257	0.165629
-0.56582	-0.22068	-0.40653	-0.1468	-0.27517	0.003258	-0.3732	-0.1107	-0.25923
4.16E-12	0.012661	2.11E-06	0.099571	0.001742	0.970997	1.55E-05	0.215341	0.003251
-0.21095	-0.27305	-0.32113	0.185919	-0.30673	0.246134	-0.20004	-0.16844	-0.29232
0.017285	0.001897	0.000232	0.03637	0.000453	0.00528	0.024141	0.058355	0.000853
0.200674	-0.06058	0.123831	0.219864	-0.00415	0.268114	-0.07665	0.095496	0.247724
0.023686	0.498682	0.165427	0.013002	0.963035	0.002307	0.391694	0.285529	0.004985
0.18825	-0.22068	-0.07438	0.437775	-0.20169	0.446989	-0.08089	-0.08867	-0.03946
0.03405	0.012663	0.405901	2.65E-07	0.022974	1.38E-07	0.365948	0.321513	0.659582
0.256788	-0.17039	0.025161	0.25698	-0.10937	0.246936	0.007939	0.017931	0.059962
0.003565	0.055465	0.778867	0.003539	0.220962	0.005129	0.929412	0.841411	0.503075
0.192771	-0.16496	0.032188	0.291097	-0.06814	0.200823	0.25326	-0.16057	0.03724
0.029905	0.06383	0.71941	0.000899	0.446543	0.02358	0.004068	0.071339	0.677652
0.099849	-0.06747	0.238014	0.262229	0.42132	0.521302	0.306122	0.366588	0.296229
0.264031	0.451014	0.007047	0.002899	8.11E-07	3.3E-10	0.000465	2.25E-05	0.000721
0.599375	0.434827	0.711999	0.630321	0.669084	0.788367	0.84556	0.5061	0.592645
9.71E-14	3.25E-07	6.35E-21	2.01E-15	8.13E-18	3.81E-28	7.16E-36	1.28E-09	2.14E-13
0.401973	0.344677	0.506694	0.590679	0.433624	0.574416	0.649769	0.267932	0.381013
2.81E-06	7.24E-05	1.22E-09	2.68E-13	3.53E-07	1.65E-12	1.4E-16	0.002323	9.91E-06
-0.09599	0.007786	-0.04016	-0.26996	0.192383	-0.10819	0.019661	0.124149	-0.07065
0.283051	0.93077	0.653971	0.002145	0.030243	0.225993	0.826341	0.164335	0.429913
-0.52671	0.09369	-0.11229	-0.43339	0.134095	-0.1582	-0.02075	0.127964	-0.07741
2.01E-10	0.294779	0.2088	3.59E-07	0.132832	0.075666	0.816905	0.15165	0.387
-0.37711	0.181315	0.166739	-0.28404	0.429235	-0.05269	0.074855	0.288125	0.290728
1.24E-05	0.041344	0.060984	0.001211	4.77E-07	0.556309	0.402929	0.00102	0.000914
0.039362	0.417455	0.477211	0.014454	0.673148	0.330481	0.594986	0.472128	0.44597
0.660393	1.05E-06	1.41E-08	0.871864	4.35E-18	0.000148	1.63E-13	2.1E-08	1.48E-07
0.321919	0.388027	0.187197	-0.18478	-0.03768	-0.28204	0.222023	-0.06108	0.04129
0.000224	6.56E-06	0.035082	0.037549	0.674092	0.001316	0.012117	0.495124	0.644865
-0.50491	-0.01556	-0.44475	-0.55047	-0.3719	-0.76185	-0.4391	-0.28186	-0.36076
1.42E-09	0.86217	1.62E-07	2.02E-11	1.67E-05	2.48E-25	2.41E-07	0.001326	3.09E-05
-0.13709	-0.30205	-0.4777	-0.16128	-0.62542	-0.58074	-0.58598	-0.5045	-0.50408
0.124316	0.000558	1.35E-08	0.070067	3.83E-15	8.25E-13	4.58E-13	1.47E-09	1.52E-09
-0.02961	-0.11758	-0.27643	-0.32429	-0.3803	-0.71901	-0.3919	-0.32793	-0.26623
0.741031	0.187979	0.001655	0.0002	1.03E-05	1.74E-21	5.2E-06	0.000167	0.002483
-0.43494	-0.07045	-0.26257	-0.40185	-0.2835	-0.08355	-0.4423	0.071408	-0.26504
3.22E-07	0.431231	0.002861	2.83E-06	0.001239	0.350381	1.93E-07	0.424989	0.002601

CD83	CD84	CD86	CD8A	CD8B	CD96	CDK14	CDK16	CDKN1A
brown	brown	brown	brown	brown	brown	brown	brown	brown
0.406467	0.218075	0.361924	0.489962	0.363353	0.314775	-0.23062	-0.14506	0.111999
2.12E-06	0.013778	2.9E-05	5.02E-09	2.68E-05	0.000313	0.009092	0.103688	0.209975
0.325418	0.066281	0.214704	0.32616	0.186066	0.120339	-0.16078	-0.07402	0.104788
0.000189	0.459068	0.01535	0.000182	0.03622	0.177774	0.070963	0.408231	0.241013
-0.53048	-0.34485	-0.37197	-0.38231	-0.40893	-0.07695	0.283647	0.143613	-0.21455
1.41E-10	7.18E-05	1.66E-05	9.19E-06	1.81E-06	0.389872	0.001231	0.107225	0.015425
-0.33938	-0.14416	-0.00565	-0.2032	-0.21012	0.212537	0.323852	-0.20575	-0.09654
9.49E-05	0.105871	0.949696	0.021947	0.017738	0.016441	0.000204	0.020307	0.280286
0.006908	-0.13957	0.224234	0.28994	0.305992	0.082663	-0.1484	-0.1219	-0.02942
0.938557	0.117592	0.011265	0.000945	0.000468	0.355519	0.095901	0.172157	0.742655
-0.10093	-0.06333	0.335567	0.163051	0.153583	0.357556	0.13879	-0.36711	0.011473
0.258878	0.479332	0.000115	0.067011	0.084722	3.68E-05	0.11967	2.18E-05	0.898131
-0.06692	-0.0967	0.206986	0.092906	0.097045	0.231062	0.114518	-0.06323	0.176543
0.454751	0.279449	0.019549	0.298854	0.27775	0.008958	0.199838	0.480038	0.04709
0.240109	0.221258	0.317133	0.082962	0.170199	0.187621	-0.1518	-0.24152	0.115719
0.006547	0.012424	0.00028	0.353779	0.05574	0.034663	0.088443	0.006228	0.195129
-0.17949	0.206108	0.272159	0.138583	0.123066	0.523762	-0.16424	-0.05398	0.473268
0.043466	0.020084	0.001965	0.120228	0.168073	2.64E-10	0.065023	0.546677	1.92E-08
0.423212	0.792818	0.911605	0.558611	0.689221	0.793827	-0.64194	-0.48893	0.624472
7.15E-07	1.17E-28	4.28E-50	8.82E-12	3.31E-19	8.95E-29	4.19E-16	5.47E-09	4.33E-15
0.344455	0.526207	0.725111	0.517764	0.561134	0.716392	-0.33181	-0.47778	0.352059
7.32E-05	2.1E-10	5.46E-22	4.56E-10	6.79E-12	2.84E-21	0.000138	1.35E-08	4.93E-05
-0.16964	-0.11736	-0.22227	-0.17001	-0.16801	-0.14717	0.013093	0.159329	-0.0197
0.056555	0.188811	0.012019	0.056013	0.059009	0.098724	0.883843	0.073575	0.826029
-0.30792	-0.02801	-0.39689	-0.39217	-0.35023	-0.16804	-0.11409	0.327297	0.004318
0.000429	0.754629	3.84E-06	5.12E-06	5.43E-05	0.058959	0.201548	0.000173	0.96157
-0.13954	0.033654	-0.18953	-0.09654	0.03322	-0.18874	-0.41811	0.175903	0.115627
0.117674	0.707201	0.032827	0.280244	0.710807	0.033576	1E-06	0.047909	0.195489
0.105892	0.551143	0.291148	0.103613	0.252828	0.301172	-0.68748	-0.07214	0.471631
0.236064	1.89E-11	0.000897	0.246361	0.004133	0.00058	4.4E-19	0.420219	2.18E-08
0.32336	0.321299	0.056266	0.041497	0.114394	-0.2162	-0.34791	-0.09388	-0.02688
0.000209	0.00023	0.529797	0.643203	0.200328	0.014635	6.12E-05	0.293799	0.764233
-0.16699	-0.3943	-0.72773	-0.50533	-0.54514	-0.74615	0.312494	0.42173	-0.45461
0.060592	4.5E-06	3.29E-22	1.37E-09	3.44E-11	7.81E-24	0.000348	7.89E-07	7.92E-08
-0.04662	-0.48707	-0.4454	-0.18581	-0.32403	-0.47176	0.747481	0.101986	-0.53416
0.602738	6.37E-09	1.55E-07	0.036484	0.000202	2.16E-08	5.9E-24	0.253898	9.96E-11
0.151849	-0.38249	-0.4573	-0.10534	-0.23825	-0.60913	0.455747	0.366353	-0.40808
0.088338	9.09E-06	6.49E-08	0.238514	0.00699	2.99E-14	7.28E-08	2.28E-05	1.91E-06
-0.40088	-0.38966	-0.45423	-0.20054	-0.37285	-0.25296	0.116849	0.362933	-0.37854
3.01E-06	5.95E-06	8.14E-08	0.023783	1.58E-05	0.004113	0.190777	2.75E-05	1.14E-05

CEACAM1	CEACAM2	CEACAM4	CEBPA	CEBPB	CEBPE	CEP97	CERK	CFB
brown	brown	brown	brown	brown	brown	brown	brown	brown
0.147082	0.081385	-0.07172	0.223066	0.077652	0.209972	-0.16523	-0.1626	0.246228
0.098917	0.363029	0.42294	0.011708	0.385532	0.017819	0.063397	0.067786	0.005263
-0.01654	0.013283	-0.05613	0.113861	0.020904	0.157824	-0.14487	-0.10607	0.158201
0.853578	0.88217	0.530779	0.202445	0.815554	0.076374	0.104156	0.235287	0.075664
-0.04885	-0.06155	-0.25416	-0.44858	-0.29909	-0.27196	0.225353	0.247846	-0.36666
0.58551	0.491816	0.003933	1.23E-07	0.000636	0.001981	0.010855	0.004963	2.24E-05
0.263319	0.026637	-0.22013	-0.47104	-0.131	-0.29051	0.163498	-0.12215	-0.12308
0.00278	0.766266	0.012889	2.28E-08	0.142097	0.000922	0.066256	0.171283	0.168019
0.064495	0.408438	0.138788	0.013516	0.068771	0.138162	-0.28897	0.084998	-0.11529
0.471285	1.87E-06	0.119675	0.880121	0.442335	0.121369	0.000984	0.342046	0.19679
0.351656	0.302802	-0.02997	-0.32409	0.043444	-0.09737	-0.09613	-0.22645	-0.0255
5.03E-05	0.00054	0.737981	0.000202	0.627695	0.276155	0.282326	0.010463	0.775928
0.293629	0.155841	0.009185	-0.23375	-0.11629	-0.0585	-0.23817	-0.14193	0.059001
0.000807	0.080191	0.918368	0.00817	0.192917	0.513585	0.00701	0.11144	0.509957
0.107737	0.155854	0.026265	0.06426	0.093767	-0.0314	0.079184	-0.39502	0.176897
0.227953	0.080166	0.769434	0.472909	0.29438	0.725986	0.376194	4.31E-06	0.046641
0.439545	0.180029	0.251389	-0.12586	0.459512	0.042678	-0.42626	-0.09201	0.456245
2.34E-07	0.042833	0.004359	0.158544	5.5E-08	0.63378	5.83E-07	0.303556	7.02E-08
0.602968	0.640291	0.502852	0.538721	0.684022	0.476069	-0.51372	-0.49606	0.678906
6.32E-14	5.26E-16	1.7E-09	6.43E-11	7.74E-19	1.54E-08	6.55E-10	3.02E-09	1.76E-18
0.55832	0.375995	0.077088	0.317393	0.375892	0.327638	-0.31994	-0.57503	0.569874
9.09E-12	1.32E-05	0.389003	0.000277	1.33E-05	0.00017	0.000246	1.55E-12	2.7E-12
0.022579	-0.18877	-0.0502	0.122216	0.053697	-0.04258	-0.14577	0.168121	0.391535
0.801069	0.033552	0.575186	0.171055	0.548781	0.634526	0.102002	0.058843	5.32E-06
-0.1578	-0.17081	-0.06769	0.044936	-0.07966	-0.0353	0.113698	0.229471	-0.03873
0.076412	0.054848	0.449547	0.615912	0.373318	0.693575	0.203099	0.009455	0.665505
-0.17749	0.16365	0.173665	0.383878	0.247996	0.270154	-0.11783	0.163434	-0.06322
0.045901	0.066	0.050865	8.38E-06	0.004936	0.002128	0.187066	0.066363	0.480086
0.186294	0.308026	0.354607	0.581991	0.488333	0.414347	-0.28511	-0.16704	0.428204
0.035988	0.000427	4.31E-05	7.17E-13	5.74E-09	1.28E-06	0.001158	0.06052	5.11E-07
-0.25976	-0.0908	0.036976	0.544661	-0.13031	0.31422	0.261596	-0.06001	-0.04768
0.003186	0.30999	0.679812	3.6E-11	0.144238	0.000321	0.00297	0.502748	0.594535
-0.62265	-0.48453	-0.33695	-0.02689	-0.57393	-0.16466	0.47483	0.515288	-0.55939
5.48E-15	7.83E-09	0.000107	0.764075	1.74E-12	0.064327	1.7E-08	5.69E-10	8.15E-12
-0.33024	-0.49997	-0.38093	-0.45813	-0.52548	-0.38723	0.480163	0.176833	-0.48818
0.00015	2.17E-09	9.96E-06	6.1E-08	2.25E-10	6.88E-06	1.11E-08	0.046722	5.81E-09
-0.50863	-0.49961	-0.20525	-0.12766	-0.42815	-0.20316	0.258263	0.319478	-0.33528
1.03E-09	2.24E-09	0.020621	0.152635	5.13E-07	0.021976	0.003372	0.000251	0.000117
-0.24557	-0.15339	-0.12592	-0.27613	-0.38649	-0.21208	0.226747	0.702624	-0.40626
0.005389	0.085126	0.158357	0.001676	7.19E-06	0.016678	0.010362	3.38E-20	2.15E-06

CFD	CFI	CFLAR	CH25H	CHI3L1	CHI3L2	CHIT1	CHML	CHRM3
brown	brown	brown	brown	brown	brown	brown	brown	brown
0.130832	0.173307	0.193934	0.31027	0.039634	0.154287	0.317235	-0.14273	-0.15823
0.142612	0.051351	0.02891	0.000385	0.658194	0.083288	0.000279	0.109431	0.075616
-0.00846	0.060639	0.058494	0.192246	-0.04607	0.137698	0.248294	-0.14741	-0.1242
0.924828	0.49826	0.513606	0.030363	0.607004	0.122637	0.004883	0.098152	0.164153
-0.33745	0.074614	-0.2173	-0.37633	-0.08186	-0.20579	-0.32663	0.344741	0.212634
0.000105	0.404444	0.014128	1.3E-05	0.360254	0.020282	0.000178	7.22E-05	0.016391
-0.39143	0.40357	0.156598	-0.13808	0.241209	-0.01364	-0.27051	0.327826	0.084706
5.35E-06	2.54E-06	0.078717	0.121587	0.006298	0.878992	0.002099	0.000168	0.343716
0.01478	0.158701	-0.25356	-0.07358	-0.06496	0.130316	0.104529	-0.17022	0.019685
0.869005	0.074734	0.004022	0.411038	0.468057	0.144207	0.242184	0.055712	0.82613
-0.2789	0.553206	0.118563	-0.01951	0.274871	0.150663	-0.06658	0.137469	-0.00043
0.001497	1.53E-11	0.184307	0.827637	0.001763	0.090882	0.457052	0.123267	0.996209
-0.33217	0.354623	0.226504	-0.02218	0.220637	0.13896	-0.00901	-0.05006	-0.04919
0.000136	4.3E-05	0.010446	0.804503	0.012679	0.119212	0.919922	0.576206	0.582904
0.072913	0.290503	0.271774	0.247357	0.264648	0.277135	0.127177	-0.00946	-0.20287
0.415274	0.000922	0.001996	0.005052	0.002641	0.001609	0.154204	0.915934	0.022171
0.247645	0.571418	0.542987	0.09541	0.49883	0.244246	0.013188	-0.12972	-0.27806
0.004999	2.29E-12	4.25E-11	0.28596	2.39E-09	0.005652	0.883004	0.146061	0.001549
0.647008	0.740145	0.715323	0.543596	0.53628	0.621256	0.570728	-0.54076	-0.68215
2.07E-16	2.74E-23	3.46E-21	4E-11	8.14E-11	6.55E-15	2.46E-12	5.28E-11	1.05E-18
0.333038	0.643118	0.614489	0.492166	0.497093	0.419871	0.372613	-0.29631	-0.49423
0.00013	3.56E-16	1.54E-14	4.18E-09	2.77E-09	8.92E-07	1.6E-05	0.000718	3.52E-09
0.067027	-0.15747	0.050993	-0.01632	0.062194	-0.05897	-0.1308	-0.2179	-0.13928
0.454019	0.07705	0.569121	0.855495	0.487283	0.510184	0.14271	0.013855	0.118354
0.161376	-0.2396	0.041167	-0.11626	-0.1123	-0.10547	-0.07304	-0.05456	-0.07866
0.069903	0.006665	0.645849	0.193046	0.208739	0.237935	0.414467	0.542352	0.379402
0.575022	-0.28539	-0.16576	-0.06761	-0.14904	0.057203	0.150881	-0.39568	-0.16036
1.55E-12	0.001145	0.062532	0.450118	0.094452	0.522956	0.090411	4.14E-06	0.07171
0.765202	0.180272	0.456621	0.280499	0.210329	0.346624	0.399588	-0.57512	-0.5826
1.15E-25	0.042547	6.82E-08	0.001402	0.017622	6.55E-05	3.26E-06	1.53E-12	6.7E-13
0.245599	-0.33306	-0.12092	0.106606	-0.2553	0.057437	0.282004	-0.36698	-0.21002
0.005383	0.00013	0.17568	0.232905	0.00377	0.521257	0.001318	2.2E-05	0.017792
-0.26025	-0.76302	-0.64871	-0.36659	-0.58567	-0.4456	-0.26166	0.219827	0.41458
0.003128	1.9E-25	1.63E-16	2.25E-05	4.74E-13	1.52E-07	0.002962	0.013018	1.26E-06
-0.67413	-0.44011	-0.52761	-0.30302	-0.34332	-0.48201	-0.39415	0.627333	0.652309
3.73E-18	2.25E-07	1.85E-10	0.000534	7.76E-05	9.6E-09	4.54E-06	2.98E-15	9.75E-17
-0.38842	-0.68471	-0.55025	-0.17142	-0.45115	-0.39201	-0.21772	0.32483	0.524984
6.41E-06	6.93E-19	2.07E-11	0.053986	1.02E-07	5.17E-06	0.013938	0.000195	2.36E-10
-0.28668	-0.32114	-0.44493	-0.45422	-0.51183	-0.43989	-0.32143	0.299488	0.358897
0.001084	0.000232	1.6E-07	8.15E-08	7.75E-10	2.28E-07	0.000229	0.000625	3.42E-05

CHST15	CIB1	CIITA	CKAP5	CLDN23	CLEC10A	CLEC12A	CLEC12B	CLEC2B
brown	brown	brown	brown	brown	brown	brown	brown	brown
0.236985	0.19378	0.164009	-0.27993	0.257209	0.306567	0.289331	0.365804	0.26081
0.007305	0.029041	0.0654	0.001435	0.003509	0.000456	0.00097	2.35E-05	0.003061
0.201485	0.214143	-0.04612	-0.22884	0.150294	0.169641	0.212055	0.280618	0.155066
0.023115	0.015627	0.606634	0.009658	0.091685	0.056559	0.016693	0.001395	0.081723
-0.52865	-0.36558	-0.15934	0.519562	-0.20648	-0.19384	-0.13102	-0.29119	-0.03844
1.68E-10	2.37E-05	0.073553	3.87E-10	0.019857	0.028991	0.142033	0.000896	0.667907
-0.299	-0.24015	0.040817	0.346467	0.093817	0.055983	0.192574	-0.06248	0.337189
0.000638	0.006537	0.648658	6.6E-05	0.294119	0.531877	0.030076	0.48531	0.000106
-0.25435	0.597917	-0.14425	-0.56843	0.120687	0.197613	0.384985	0.158211	0.448733
0.003907	1.15E-13	0.105667	3.15E-12	0.176512	0.025949	7.85E-06	0.075646	1.22E-07
-0.20019	0.261526	0.039582	-0.17993	0.310358	0.288208	0.536361	0.213554	0.688531
0.024031	0.002978	0.658616	0.042954	0.000384	0.001017	8.07E-11	0.015922	3.7E-19
0.016939	0.197714	0.04949	-0.13821	0.179314	0.226005	0.339757	0.07453	0.48354
0.85008	0.025871	0.580577	0.121232	0.043679	0.010621	9.31E-05	0.404976	8.48E-09
0.007335	0.356089	0.310102	-0.16097	0.237608	0.186733	0.231116	0.106791	0.216853
0.934769	3.98E-05	0.000388	0.07062	0.007148	0.035545	0.008941	0.232089	0.014331
0.236698	0.160999	0.386935	-0.03407	0.378914	0.394445	0.321059	0.154102	0.491348
0.007378	0.070569	7E-06	0.70371	1.12E-05	4.46E-06	0.000233	0.083664	4.48E-09
0.502832	0.690415	0.82415	-0.60883	0.77337	0.779936	0.714727	0.526647	0.708682
1.7E-09	2.71E-19	1.18E-32	3.11E-14	1.66E-26	3.3E-27	3.86E-21	2.02E-10	1.16E-20
0.329254	0.330961	0.67874	-0.29818	0.706749	0.537536	0.521555	0.368641	0.599963
0.000157	0.000144	1.81E-18	0.000662	1.63E-20	7.21E-11	3.23E-10	2E-05	9.05E-14
0.145277	-0.15662	-0.09209	0.254496	-0.13203	-0.23449	-0.31315	-0.28473	-0.27342
0.103174	0.078681	0.303162	0.003885	0.138966	0.007966	0.000337	0.001177	0.001869
-0.11895	-0.35489	0.078579	0.467698	-0.2294	-0.22358	-0.44181	-0.34583	-0.47326
0.182865	4.24E-05	0.379868	2.96E-08	0.009476	0.011513	2E-07	6.82E-05	1.92E-08
-0.21098	0.243655	0.056701	-0.22758	-0.0503	-0.07144	-0.23627	-0.19779	-0.28474
0.017266	0.005772	0.526616	0.010077	0.574408	0.424756	0.00749	0.025816	0.001176
0.276432	0.349113	0.588425	-0.22426	0.336548	0.312969	0.083925	0.111	0.015284
0.001655	5.75E-05	3.47E-13	0.011254	0.000109	0.00034	0.348195	0.214094	0.864582
0.218783	0.051504	-0.00739	-0.13917	-0.05663	-0.06207	-0.10509	0.123598	-0.38155
0.013466	0.565247	0.934238	0.118643	0.527132	0.488168	0.239635	0.16623	9.6E-06
-0.29804	-0.60076	-0.49638	0.523636	-0.72406	-0.65194	-0.72462	-0.51011	-0.86943
0.000666	8.23E-14	2.94E-09	2.67E-10	6.69E-22	1.03E-16	6.01E-22	9.02E-10	4.32E-40
-0.19157	-0.60104	-0.60371	0.342501	-0.4762	-0.4512	-0.31751	-0.18245	-0.34284
0.030966	7.96E-14	5.78E-14	8.1E-05	1.52E-08	1.02E-07	0.000275	0.04007	7.96E-05
0.03453	-0.4067	-0.5644	0.167618	-0.5478	-0.50068	-0.49013	-0.19615	-0.59765
0.699937	2.09E-06	4.83E-12	0.059615	2.64E-11	2.04E-09	4.95E-09	0.027093	1.19E-13
-0.41529	-0.36085	-0.36644	0.267997	-0.49832	-0.24297	-0.2524	-0.2491	-0.22296
1.2E-06	3.08E-05	2.26E-05	0.002317	2.5E-09	0.005914	0.004199	0.004742	0.011751

CLEC4A	CLEC4D	CLEC7A	CLIC1	CLIC6	CLN6	CMKLR1	CNTNAP5	COA7
brown	brown	brown	brown	brown	brown	brown	brown	brown
0.242849	0.055289	0.249278	0.010519	0.200679	0.218844	0.2935	-0.19138	-0.15695
0.005941	0.536977	0.00471	0.906567	0.023683	0.01344	0.000811	0.03113	0.07804
0.135425	0.003975	0.108945	-0.0747	0.080063	0.166661	0.119547	-0.10106	-0.1685
0.128995	0.964627	0.222752	0.403912	0.370906	0.061107	0.180664	0.258244	0.058265
-0.06492	-0.24702	-0.33839	0.102648	-0.1489	-0.20006	-0.1806	0.149158	0.401452
0.468335	0.005113	9.98E-05	0.250808	0.094773	0.024127	0.042167	0.094192	2.9E-06
0.263201	-0.0715	0.0897	0.371018	0.072583	-0.3269	0.01384	-0.17374	0.338836
0.002793	0.424425	0.315911	1.75E-05	0.417394	0.000176	0.877266	0.05076	9.75E-05
0.423885	0.220443	0.07538	0.326743	0.032706	-0.17006	0.019625	-0.17663	-0.06721
6.84E-07	0.012759	0.399626	0.000177	0.715087	0.055937	0.826651	0.046974	0.452811
0.598072	0.189891	0.334981	0.585835	0.204843	-0.34752	0.146432	-0.38293	0.170052
1.13E-13	0.032493	0.000118	4.66E-13	0.020878	6.25E-05	0.100433	8.86E-06	0.055955
0.421778	0.078496	0.234093	0.393174	0.165623	-0.19507	0.047114	-0.24793	0.069298
7.87E-07	0.38037	0.008075	4.82E-06	0.062759	0.027965	0.598896	0.004947	0.438836
0.263344	0.086778	0.39167	0.32132	0.313301	-0.18164	0.119492	-0.38631	-0.14044
0.002777	0.331995	5.28E-06	0.00023	0.000335	0.040973	0.180866	7.26E-06	0.115296
0.372931	0.290689	0.327477	0.60488	0.501173	0.275184	0.508801	-0.47522	-0.05387
1.57E-05	0.000915	0.000171	5.02E-14	1.96E-09	0.001741	1.01E-09	1.65E-08	0.547477
0.745474	0.517824	0.845555	0.711248	0.563524	0.488727	0.827822	-0.79276	-0.4569
9.02E-24	4.53E-10	7.17E-36	7.28E-21	5.29E-12	5.56E-09	3.57E-33	1.19E-28	6.69E-08
0.569358	0.248771	0.682803	0.612131	0.431724	0.345172	0.628523	-0.63079	-0.19621
2.85E-12	0.004798	9.43E-19	2.07E-14	4.02E-07	7.06E-05	2.55E-15	1.89E-15	0.027048
-0.23847	-0.24271	-0.22543	-0.23297	-0.07722	0.438738	-0.06821	0.172918	-0.29322
0.006936	0.005971	0.010827	0.008394	0.388176	2.48E-07	0.446066	0.051885	0.000821
-0.38069	-0.25689	-0.39245	-0.2514	-0.13122	0.326619	-0.05566	0.200741	0.01314
1.01E-05	0.003551	5.03E-06	0.004357	0.141427	0.000178	0.534252	0.023638	0.883429
-0.21749	-0.00611	-0.32139	-0.02413	-0.14857	0.393725	-0.00266	0.013039	-0.17332
0.014042	0.945603	0.000229	0.787692	0.095517	4.66E-06	0.976294	0.884317	0.05133
0.117385	0.173391	0.209151	0.233691	0.251449	0.685599	0.513378	-0.377	-0.43373
0.188734	0.051237	0.018281	0.008187	0.00435	5.99E-19	6.75E-10	1.25E-05	3.51E-07
-0.23943	-0.12453	-0.0036	-0.42787	-0.17727	0.193141	-0.01288	0.222676	-0.31586
0.006705	0.163022	0.967991	5.23E-07	0.046169	0.029586	0.885759	0.01186	0.000298
-0.79314	-0.52371	-0.71733	-0.7739	-0.58965	-0.14279	-0.61491	0.812235	0.235552
1.08E-28	2.65E-10	2.38E-21	1.46E-26	3.01E-13	0.109276	1.46E-14	4.79E-31	0.007678
-0.43357	-0.28065	-0.31113	-0.51327	-0.39201	-0.61732	-0.58841	0.565188	0.441295
3.55E-07	0.001394	0.00037	6.82E-10	5.17E-06	1.08E-14	3.47E-13	4.44E-12	2.07E-07
-0.61087	-0.2405	-0.4029	-0.74937	-0.40919	-0.32006	-0.58811	0.667949	0.166694
2.42E-14	0.006458	2.65E-06	3.94E-24	1.78E-06	0.000244	3.59E-13	9.67E-18	0.061055
-0.18699	-0.1893	-0.5019	-0.42957	-0.2686	-0.1302	-0.25002	0.510329	0.170124
0.035285	0.033044	1.84E-09	4.66E-07	0.002263	0.144571	0.004583	8.84E-10	0.055849

COCH	COL25A1	COL4A6	COL8A1	COL8A2	COLEC12	COPZ2	CORO1A	CP
brown	brown	brown	brown	brown	brown	brown	brown	brown
-0.19109	-0.13589	0.053849	0.05728	0.177769	0.449786	0.2064	0.256459	0.167288
0.03139	0.12767	0.547651	0.522393	0.045554	1.13E-07	0.019905	0.00361	0.060125
-0.09261	-0.12086	-0.04495	-0.04172	0.022712	0.285501	0.167321	0.15076	0.127119
0.300392	0.175883	0.61577	0.641413	0.799916	0.001139	0.060075	0.090671	0.154396
0.167443	0.329358	-0.17339	-0.13292	-0.09691	-0.44949	-0.22481	-0.62849	-0.32144
0.059886	0.000156	0.051244	0.136282	0.278418	1.15E-07	0.011053	2.56E-15	0.000229
0.02981	0.244395	-0.11041	0.116867	0.192438	-0.10171	0.099705	-0.68089	0.019315
0.739363	0.005621	0.216543	0.190708	0.030195	0.255198	0.264725	1.28E-18	0.829349
0.052793	-0.29123	-0.15722	-0.02926	0.028919	-0.07691	0.278835	0.087497	0.137986
0.555543	0.000894	0.077528	0.744008	0.746887	0.390092	0.001501	0.327991	0.121849
-0.00783	-0.08819	-0.10289	0.212023	0.29161	0.064948	0.398122	-0.39348	0.253535
0.93039	0.324144	0.249699	0.01671	0.00088	0.468175	3.56E-06	4.73E-06	0.004026
-0.05223	-0.0277	-0.06485	0.175686	0.247487	0.06521	0.214522	-0.31505	0.249942
0.559774	0.757232	0.468879	0.048189	0.005028	0.466375	0.01544	0.000309	0.004597
-0.26705	-0.3171	0.307424	0.294996	0.373827	0.398798	0.317379	0.04916	0.482484
0.002405	0.000281	0.000438	0.00076	1.5E-05	3.42E-06	0.000277	0.583098	9.24E-09
-0.06554	0.043594	0.284671	0.56433	0.455068	0.213388	0.382936	-0.1119	0.296376
0.46415	0.626507	0.00118	4.86E-12	7.65E-08	0.016005	8.86E-06	0.210372	0.000716
-0.51088	-0.53178	0.553099	0.747354	0.765894	0.675332	0.722932	0.541533	0.749721
8.42E-10	1.25E-10	1.55E-11	6.06E-24	9.76E-26	3.09E-18	8.3E-22	4.9E-11	3.65E-24
-0.41084	-0.32864	0.320202	0.520814	0.68768	0.633559	0.555409	0.295518	0.486669
1.61E-06	0.000162	0.000243	3.46E-10	4.26E-19	1.31E-15	1.23E-11	0.000743	6.58E-09
0.235664	0.134795	-0.15465	-0.21297	-0.12561	-0.12982	-0.07684	-0.04916	-0.12668
0.007648	0.130801	0.082561	0.016217	0.159393	0.145741	0.390523	0.583066	0.155851
-0.05522	0.25973	0.154208	-0.08589	-0.07825	-0.20725	-0.32965	-0.13687	-0.24089
0.537505	0.00319	0.083447	0.336997	0.381873	0.019389	0.000154	0.124913	0.00637
-0.09834	-0.06852	0.198314	-0.04471	-0.03899	-0.15049	-0.05442	0.397102	-0.11831
0.27134	0.44398	0.025415	0.617713	0.663432	0.091268	0.54341	3.79E-06	0.185248
-0.41987	-0.27295	0.563214	0.424607	0.409576	0.323661	0.265886	0.492135	0.314983
8.93E-07	0.001905	5.47E-12	6.52E-07	1.74E-06	0.000206	0.002517	4.19E-09	0.00031
-0.1706	-0.30927	0.019328	-0.15133	-0.18546	0.153594	-0.09625	0.504194	0.05186
0.055154	0.000403	0.829235	0.089434	0.036844	0.0847	0.281721	1.51E-09	0.562557
0.278761	0.28843	-0.3832	-0.66365	-0.70552	-0.51075	-0.71259	-0.08227	-0.66771
0.001506	0.001007	8.72E-06	1.85E-17	2.03E-20	8.52E-10	5.7E-21	0.357795	1E-17
0.432994	0.395794	-0.55602	-0.52075	-0.5557	-0.3508	-0.45971	-0.31775	-0.49431
3.69E-07	4.11E-06	1.15E-11	3.48E-10	1.19E-11	5.27E-05	5.42E-08	0.000272	3.5E-09
0.379815	0.211372	-0.41352	-0.56122	-0.64092	-0.28963	-0.54055	0.113877	-0.43572
1.06E-05	0.017056	1.35E-06	6.73E-12	4.82E-16	0.000957	5.39E-11	0.202384	3.06E-07
0.240939	0.309802	-0.38468	-0.44473	-0.47338	-0.57423	-0.55234	-0.42577	-0.47949
0.006358	0.000394	7.99E-06	1.62E-07	1.9E-08	1.69E-12	1.67E-11	6.03E-07	1.17E-08

CPD	CPNE4	CPQ	CPVL	CR1	CRB2	CREG1	CRTAM	CRYBB1
brown	brown	brown	brown	brown	brown	brown	brown	brown
-0.00309	-0.12829	0.330902	0.158886	0.300066	0.135086	0.516952	0.580228	0.431399
0.972514	0.150617	0.000145	0.07439	0.000609	0.129965	4.9E-10	8.73E-13	4.12E-07
-0.04822	-0.09759	0.268215	0.006415	0.201007	0.050301	0.441584	0.548411	0.37237
0.590365	0.275021	0.002297	0.942939	0.023451	0.574375	2.03E-07	2.48E-11	1.63E-05
-0.05733	0.275828	-0.49634	-0.06675	-0.36874	-0.40304	-0.55477	-0.31579	-0.32207
0.522005	0.001696	2.95E-09	0.455914	1.99E-05	2.63E-06	1.31E-11	0.000298	0.000222
0.285748	0.072178	-0.06858	0.293525	-0.11616	-0.29866	-0.19753	-0.05314	-0.31684
0.001128	0.420003	0.443637	0.00081	0.193413	0.000648	0.02601	0.55296	0.000284
-0.19203	-0.14316	0.23419	0.242598	0.0453	-0.32498	0.270041	0.360398	0.333714
0.030552	0.108339	0.008048	0.005995	0.613058	0.000193	0.002138	3.15E-05	0.000126
0.269349	-0.16033	0.312207	0.502621	0.074983	-0.3154	0.232791	0.32798	0.026199
0.002197	0.071763	0.000352	1.73E-09	0.40212	0.000304	0.008444	0.000167	0.769997
0.218547	-0.19086	0.281532	0.349718	0.112453	-0.28024	0.154681	0.301915	0.054481
0.013569	0.0316	0.001344	5.57E-05	0.20812	0.001417	0.082493	0.000561	0.542951
0.219513	-0.34119	0.327266	0.378767	0.080677	0.381312	0.265492	0.110009	-0.04782
0.013151	8.65E-05	0.000173	1.13E-05	0.367232	9.74E-06	0.002555	0.218241	0.593441
0.543877	-0.26277	0.263106	0.443258	0.419538	0.101651	0.148836	0.213859	0.004956
3.89E-11	0.002839	0.002803	1.8E-07	9.12E-07	0.255467	0.094913	0.015768	0.9559
0.480036	-0.65136	0.720058	0.813088	0.755533	0.438348	0.821846	0.608056	0.601104
1.12E-08	1.12E-16	1.43E-21	3.71E-31	1.03E-24	2.54E-07	2.47E-32	3.41E-14	7.9E-14
0.373555	-0.36999	0.529761	0.698996	0.448466	0.395141	0.561994	0.473011	0.365925
1.52E-05	1.86E-05	1.51E-10	6.35E-20	1.24E-07	4.28E-06	6.21E-12	1.96E-08	2.33E-05
-0.06782	0.117102	-0.07627	-0.1802	0.020963	0.046659	-0.23876	-0.14156	-0.13899
0.448666	0.189809	0.394047	0.042628	0.815041	0.602432	0.006866	0.112393	0.119122
-0.13442	0.190713	-0.51404	-0.26542	-0.13672	0.122118	-0.48996	-0.38543	-0.20103
0.131895	0.031735	6.36E-10	0.002563	0.125343	0.171401	5.02E-09	7.65E-06	0.023433
-0.32542	0.069381	-0.21946	-0.1124	0.004069	0.203308	-0.21735	-0.15562	0.189767
0.000189	0.438286	0.013174	0.208322	0.96379	0.021877	0.014105	0.080626	0.032608
0.095951	-0.3324	0.142988	0.269526	0.430712	0.541836	0.220715	0.116961	0.337826
0.283229	0.000135	0.108778	0.002182	4.31E-07	4.75E-11	0.012647	0.190348	0.000103
-0.30368	-0.12484	0.124124	-0.24729	0.062441	0.252894	0.293564	0.051057	0.300796
0.000519	0.161993	0.16442	0.005064	0.485555	0.004123	0.000809	0.568633	0.00059
-0.63182	0.507417	-0.67537	-0.76839	-0.59108	-0.20372	-0.66523	-0.64235	-0.29807
1.65E-15	1.14E-09	3.08E-18	5.44E-26	2.56E-13	0.021601	1.46E-17	3.96E-16	0.000665
-0.16428	0.46243	-0.26735	-0.48327	-0.48269	-0.40641	-0.33799	-0.31916	-0.4121
0.064958	4.42E-08	0.002377	8.67E-09	9.09E-09	2.13E-06	0.000102	0.000255	1.48E-06
-0.34618	0.381985	-0.27628	-0.67944	-0.39387	-0.16755	-0.24393	-0.3205	-0.21167
6.7E-05	9.36E-06	0.001665	1.62E-18	4.62E-06	0.059719	0.005716	0.000239	0.016897
-0.38486	0.315467	-0.62398	-0.40908	-0.26033	-0.67262	-0.44257	-0.21021	-0.13606
7.91E-06	0.000303	4.62E-15	1.8E-06	0.003118	4.72E-18	1.89E-07	0.01769	0.127201

CSF1R	CSF2RA	CSF2RB	CSF3R	CSPG5	CSRNP2	CST7	CSTA	CSTB
brown	brown	brown	brown	brown	brown	brown	brown	brown
0.198029	0.259465	0.217565	0.136779	-0.26959	-0.03532	0.238358	0.21914	0.248626
0.025631	0.003222	0.014006	0.125178	0.002177	0.693448	0.006963	0.013311	0.004824
0.04445	0.161055	0.024163	0.0381	-0.1636	0.053249	0.063956	0.11154	0.238774
0.619741	0.07047	0.787429	0.670635	0.066086	0.552125	0.475007	0.21186	0.006862
-0.43587	-0.4761	-0.2479	-0.46396	0.341077	0.185921	-0.20994	-0.0697	-0.2561
3.02E-07	1.54E-08	0.004952	3.93E-08	8.71E-05	0.036368	0.017837	0.436198	0.003659
-0.32355	-0.22739	0.022152	-0.37892	0.013719	0.171479	-0.10455	0.289807	-0.09336
0.000207	0.01014	0.804752	1.12E-05	0.878332	0.053897	0.242101	0.00095	0.296503
-0.15035	0.21833	-0.1561	-0.022	-0.11312	-0.11982	0.204001	0.41449	0.467865
0.091569	0.013665	0.079678	0.806086	0.205423	0.179663	0.02142	1.27E-06	2.92E-08
-0.22225	0.112739	0.086731	-0.20963	-0.22455	0.027055	0.114828	0.625477	0.28914
0.012028	0.206959	0.332262	0.018011	0.01115	0.762703	0.198613	3.8E-15	0.000977
-0.17429	0.092106	-0.0062	-0.10124	-0.13157	0.032309	0.064279	0.40874	0.305689
0.050025	0.303055	0.94487	0.257387	0.140358	0.718397	0.472776	1.83E-06	0.000474
0.238943	0.288095	0.251175	0.1541	-0.29408	-0.28023	0.030844	0.340823	0.171934
0.006822	0.001022	0.004394	0.083666	0.000791	0.001418	0.730669	8.82E-05	0.053254
0.211281	-0.0474	0.407053	0.120559	-0.41202	-0.10398	0.405268	0.455241	0.279103
0.017104	0.596652	2.04E-06	0.176976	1.49E-06	0.244702	2.29E-06	7.56E-08	0.001485
0.752108	0.703053	0.844137	0.758493	-0.70773	-0.58669	0.753615	0.82158	0.735543
2.18E-24	3.14E-20	1.21E-35	5.3E-25	1.37E-20	4.23E-13	1.57E-24	2.69E-32	7E-23
0.493488	0.442533	0.656178	0.408364	-0.43337	-0.37708	0.538161	0.616912	0.419772
3.75E-09	1.9E-07	5.58E-17	1.88E-06	3.59E-07	1.24E-05	6.79E-11	1.14E-14	8.98E-07
-0.00434	-0.21127	-0.21263	-0.08793	0.025896	0.188663	-0.01659	-0.27494	-0.12414
0.961346	0.017109	0.016395	0.32558	0.772586	0.033652	0.853092	0.001758	0.164359
0.011962	-0.3591	-0.07096	-0.00193	0.243533	-0.03315	-0.08747	-0.40255	-0.2388
0.893813	3.38E-05	0.427889	0.982813	0.005798	0.711404	0.328115	2.71E-06	0.006856
0.116769	-0.08534	-0.065	0.244937	0.127176	-0.30073	0.163533	-0.18488	0.158554
0.191083	0.340102	0.467788	0.005513	0.15421	0.000592	0.066197	0.037451	0.075007
0.588777	0.255246	0.490678	0.636748	-0.27398	-0.52821	0.466026	0.170144	0.39428
3.33E-13	0.003777	4.73E-09	8.52E-16	0.001827	1.75E-10	3.36E-08	0.05582	4.51E-06
0.276964	0.32131	0.019942	0.360593	0.146199	-0.10285	-0.06023	-0.25545	0.037253
0.00162	0.00023	0.8239	3.12E-05	0.100981	0.249854	0.501171	0.003749	0.677543
-0.33646	-0.42306	-0.58748	-0.31994	0.775881	0.292254	-0.55332	-0.85473	-0.59969
0.00011	7.22E-07	3.86E-13	0.000246	8.99E-27	0.000856	1.52E-11	2.1E-37	9.34E-14
-0.51311	-0.30703	-0.46678	-0.56597	0.415971	0.571796	-0.56968	-0.48007	-0.64735
6.91E-10	0.000446	3.17E-08	4.09E-12	1.15E-06	2.2E-12	2.76E-12	1.12E-08	1.97E-16
-0.23699	-0.18096	-0.4947	-0.27583	0.387384	0.409051	-0.4788	-0.67945	-0.52115
0.007305	0.041748	3.39E-09	0.001696	6.81E-06	1.8E-06	1.24E-08	1.62E-18	3.35E-10
-0.43677	-0.38217	-0.40085	-0.3635	0.299527	0.330812	-0.16856	-0.31516	-0.22396
2.84E-07	9.26E-06	3.01E-06	2.66E-05	0.000624	0.000145	0.058182	0.000307	0.011366

CSTF2T	CTLA4	CTNNBIP1	CTSA	CTSB	CTSC	CTSH	CTSL	CTSO
brown	brown	brown	brown	brown	brown	brown	brown	brown
0.114889	0.271593	-0.0939	0.327945	0.20775	0.198016	0.372659	0.260952	0.034408
0.198375	0.00201	0.293701	0.000167	0.019093	0.025641	1.6E-05	0.003044	0.700952
0.156054	0.180487	0.029356	0.227168	0.117073	0.044343	0.222788	0.140621	-0.07944
0.079774	0.042297	0.743199	0.010216	0.18992	0.620582	0.011816	0.114821	0.374677
0.054699	-0.17787	0.036486	-0.24647	-0.29264	-0.21743	-0.54273	-0.20165	-0.01631
0.541336	0.045436	0.683823	0.005217	0.000842	0.014066	4.36E-11	0.023003	0.855618
-0.16138	0.071198	-0.36536	-0.12604	-0.08017	-0.00175	-0.34303	0.12764	0.395194
0.069902	0.426358	2.4E-05	0.157966	0.370244	0.984422	7.88E-05	0.1527	4.26E-06
-0.15669	0.187239	0.133717	-0.08087	0.000132	0.110202	0.194936	0.183829	0.052514
0.078543	0.03504	0.133937	0.366103	0.998827	0.217429	0.028077	0.038562	0.557637
-0.28706	0.297661	-0.35203	-0.0551	0.068242	0.200856	0.002472	0.370259	0.51994
0.001067	0.000677	4.94E-05	0.53839	0.445862	0.023557	0.977993	1.83E-05	3.74E-10
-0.32788	0.172816	-0.16816	0.121867	0.082704	0.09194	-0.00784	0.259587	0.311836
0.000168	0.052024	0.058781	0.172289	0.355281	0.303927	0.930252	0.003207	0.000358
-0.23191	0.19994	-0.39002	-0.05288	0.193089	0.202087	0.311864	0.266031	0.582032
0.008703	0.024213	5.82E-06	0.554878	0.02963	0.0227	0.000358	0.002502	7.14E-13
-0.41961	0.353495	-0.48082	0.563801	0.50806	0.542976	0.092834	0.518281	0.40836
9.08E-07	4.57E-05	1.06E-08	5.14E-12	1.08E-09	4.25E-11	0.299232	4.35E-10	1.88E-06
-0.34531	0.657518	-0.66243	0.707375	0.838342	0.913715	0.79624	0.88874	0.651313
7.01E-05	4.58E-17	2.22E-17	1.46E-20	9.81E-35	1.01E-50	4.65E-29	3.63E-44	1.12E-16
-0.31057	0.501669	-0.61388	0.504643	0.484157	0.598515	0.60508	0.674845	0.619448
0.00038	1.88E-09	1.66E-14	1.45E-09	8.07E-09	1.07E-13	4.9E-14	3.34E-18	8.25E-15
-0.07079	-0.14331	0.264729	0.30652	-0.07886	-0.15571	-0.13405	-0.19463	-0.33905
0.429036	0.107966	0.002632	0.000457	0.378161	0.080451	0.132957	0.028331	9.65E-05
0.171138	-0.2469	0.081201	0.104647	-0.06167	-0.13796	-0.26164	-0.23811	-0.31584
0.054383	0.005137	0.36412	0.241651	0.490956	0.121908	0.002965	0.007024	0.000298
0.062131	-0.17619	0.181303	0.093461	0.082528	0.032324	0.156652	-0.04774	-0.39386
0.487727	0.047542	0.041358	0.295965	0.356306	0.718276	0.078612	0.594056	4.62E-06
-0.0595	0.201731	-0.27635	0.570235	0.544634	0.510344	0.476293	0.388774	0.065824
0.506368	0.022945	0.001661	2.6E-12	3.61E-11	8.83E-10	1.51E-08	6.27E-06	0.462177
0.444767	-0.14126	0.30917	-0.03753	0.030364	-0.03639	0.269693	-0.17935	-0.31847
1.62E-07	0.113169	0.000405	0.675289	0.7347	0.684629	0.002168	0.04364	0.000263
0.479416	-0.64052	0.714967	-0.53787	-0.61077	-0.68835	-0.48255	-0.81079	-0.73132
1.18E-08	5.1E-16	3.69E-21	6.98E-11	2.45E-14	3.82E-19	9.19E-09	7.37E-31	1.63E-22
0.239674	-0.35488	0.387945	-0.62714	-0.60365	-0.61955	-0.5149	-0.56904	-0.24934
0.006648	4.24E-05	6.59E-06	3.06E-15	5.83E-14	8.15E-15	5.89E-10	2.95E-12	0.004699
0.291261	-0.4193	0.607039	-0.45725	-0.50475	-0.58731	-0.29201	-0.6388	-0.51765
0.000893	9.27E-07	3.87E-14	6.51E-08	1.44E-09	3.94E-13	0.000865	6.45E-16	4.6E-10
0.370004	-0.14543	0.512394	-0.23193	-0.40815	-0.29413	-0.53332	-0.45446	-0.50296
1.86E-05	0.102815	7.37E-10	0.008696	1.9E-06	0.00079	1.08E-10	8.01E-08	1.68E-09

CTSS	CTSW	CTSZ	CX3CR1	CXCL12	CXCL13	CXCL16	CXCL2	CXCL3
brown	brown	brown	brown	brown	brown	brown	brown	brown
0.364893	0.150425	0.340804	0.216594	0.330114	0.019442	0.183	-0.00641	-0.04202
2.47E-05	0.091398	8.83E-05	0.014451	0.000151	0.828245	0.039462	0.942943	0.639029
0.193744	-0.01635	0.226452	0.090088	0.170842	-0.03985	0.022624	0.012585	-0.04806
0.029071	0.855267	0.010464	0.313816	0.054808	0.656481	0.800674	0.88832	0.591543
-0.37501	0.01093	-0.37816	-0.28647	-0.4091	-0.02455	-0.26123	-0.10555	0.012608
1.4E-05	0.902927	1.17E-05	0.001094	1.79E-06	0.784071	0.003012	0.237602	0.888114
-0.04465	0.14107	-0.25131	-0.19598	-0.17385	0.10361	-0.03992	0.18325	0.365638
0.618177	0.113653	0.004372	0.027228	0.050617	0.246375	0.655919	0.039189	2.37E-05
0.18739	0.124024	0.048295	-0.08739	0.031182	0.10441	-0.11525	0.084486	0.094015
0.034891	0.164761	0.589755	0.328564	0.727834	0.242725	0.19697	0.344971	0.293099
0.267538	0.235351	-0.04078	-0.12906	0.034747	0.223733	0.015639	0.32737	0.466424
0.002359	0.007732	0.648956	0.148158	0.698148	0.011453	0.861464	0.000172	3.26E-08
0.156094	0.124218	0.005565	-0.10159	0.068819	0.194647	0.044672	0.188378	0.301681
0.079696	0.164098	0.95049	0.255732	0.442017	0.028315	0.617989	0.033926	0.000567
0.315895	0.182598	0.163398	0.219144	0.20739	0.091394	0.150981	0.000189	0.125398
0.000297	0.039904	0.066424	0.01331	0.019307	0.306822	0.090195	0.998317	0.160096
0.317598	0.444651	0.405761	-0.28177	0.393957	0.465896	0.433097	0.254789	0.387363
0.000274	1.63E-07	2.22E-06	0.001331	4.59E-06	3.39E-08	3.66E-07	0.003843	6.82E-06
0.938328	0.658556	0.901486	0.224046	0.761125	0.482872	0.851352	0.386798	0.424464
1.68E-59	3.94E-17	2.72E-47	0.011335	2.93E-25	8.95E-09	7.93E-37	7.06E-06	6.58E-07
0.713238	0.626355	0.565668	0.322768	0.518856	0.358783	0.65471	0.316204	0.373593
5.07E-21	3.39E-15	4.22E-12	0.000215	4.13E-10	3.44E-05	6.9E-17	0.000293	1.52E-05
-0.26381	-0.05318	-0.03956	-0.08641	-0.04108	-0.01229	0.076985	-0.0698	-0.11264
0.002728	0.552655	0.658817	0.334058	0.646573	0.890932	0.389637	0.435536	0.207357
-0.34383	-0.01433	-0.03696	-0.14294	-0.16156	-0.10098	0.036234	-0.27392	-0.24564
7.56E-05	0.872947	0.679938	0.108907	0.06958	0.25864	0.685892	0.001832	0.005376
-0.09605	0.04797	0.165665	-0.10859	-0.06926	-0.04444	0.085256	-0.24335	-0.24594
0.282703	0.592262	0.062691	0.22426	0.43909	0.619852	0.340578	0.005837	0.005318
0.378466	0.382926	0.658414	0.067407	0.407086	0.193953	0.615489	-0.023	-0.0251
1.15E-05	8.86E-06	4.02E-17	0.451459	2.04E-06	0.028894	1.36E-14	0.797401	0.779372
0.095882	-0.21845	0.184639	0.407156	0.10081	-0.35682	0.039805	-0.23121	-0.45565
0.283576	0.013614	0.037699	2.03E-06	0.259439	3.83E-05	0.656815	0.008912	7.33E-08
-0.69648	-0.55496	-0.5746	0.084719	-0.55993	-0.5147	-0.5224	-0.45588	-0.60211
9.76E-20	1.28E-11	1.62E-12	0.343637	7.7E-12	6E-10	2.99E-10	7.21E-08	7.01E-14
-0.48201	-0.52445	-0.69859	0.041423	-0.46924	-0.35698	-0.62338	-0.09619	-0.15615
9.6E-09	2.48E-10	6.81E-20	0.643794	2.63E-08	3.79E-05	4.99E-15	0.282007	0.07958
-0.47159	-0.57164	-0.47704	0.154059	-0.3229	-0.348	-0.54668	-0.23106	-0.39498
2.19E-08	2.23E-12	1.43E-08	0.083749	0.000213	6.1E-05	2.95E-11	0.008959	4.32E-06
-0.47076	-0.18703	-0.38317	-0.29914	-0.27196	-0.20435	-0.42721	-0.32962	-0.30147
2.34E-08	0.035244	8.74E-06	0.000635	0.001981	0.021191	5.47E-07	0.000154	0.000573

CXCL8	CXCL9	CXCR3	CXCR4	CXCR6	CXorf21	CXorf65	CYB561A3	CYB5A
brown	brown	brown	brown	brown	brown	brown	brown	brown
-0.05413	0.404696	0.36402	0.19316	0.317862	0.241539	0.191778	0.242463	0.185216
0.545551	2.37E-06	2.59E-05	0.029569	0.000271	0.006224	0.030777	0.006023	0.037096
-0.05146	0.20232	0.188477	0.090646	0.129519	0.056265	0.100037	0.090991	0.173827
0.565586	0.022541	0.03383	0.310811	0.146699	0.529807	0.26313	0.30897	0.050647
0.045949	-0.10811	-0.24983	-0.16886	-0.11194	-0.17537	-0.14171	-0.34204	-0.14242
0.607972	0.226335	0.004616	0.057721	0.210232	0.048606	0.11202	8.29E-05	0.110214
0.325049	0.207163	-0.17523	0.191512	0.175699	0.102355	-0.01276	-0.21774	-0.10845
0.000192	0.019442	0.04878	0.031014	0.048172	0.252175	0.886816	0.013929	0.224858
0.196963	0.104443	0.040304	0.21821	0.193762	0.035038	0.235271	-0.11338	0.600449
0.026453	0.242575	0.652784	0.013718	0.029056	0.695739	0.007753	0.20438	8.54E-14
0.457947	0.37506	-0.02925	0.448921	0.393782	0.259335	0.211737	-0.1274	0.346503
6.18E-08	1.4E-05	0.744073	1.2E-07	4.64E-06	0.003238	0.016861	0.153473	6.59E-05
0.370214	0.260688	-0.04455	0.29989	0.224099	0.076776	0.188449	-0.08271	0.103331
1.83E-05	0.003075	0.618952	0.000614	0.011316	0.390927	0.033857	0.355262	0.247656
0.061453	0.24358	0.005196	0.329171	0.179838	0.311139	0.060781	0.321825	0.170128
0.492498	0.005788	0.95377	0.000158	0.043057	0.00037	0.497248	0.000225	0.055844
0.444944	0.407215	0.388009	0.508791	0.410782	0.283567	0.292257	0.361435	0.084943
1.6E-07	2.02E-06	6.57E-06	1.01E-09	1.61E-06	0.001235	0.000856	2.98E-05	0.34236
0.307315	0.731999	0.778308	0.773236	0.748025	0.775557	0.640266	0.796424	0.54422
0.000441	1.42E-22	4.94E-27	1.71E-26	5.25E-24	9.74E-27	5.28E-16	4.42E-29	3.76E-11
0.206771	0.73799	0.603298	0.55516	0.653801	0.636873	0.484819	0.614768	0.281604
0.019679	4.26E-23	6.08E-14	1.26E-11	7.87E-17	8.38E-16	7.65E-09	1.49E-14	0.00134
-0.10971	-0.19837	0.073151	-0.15003	-0.19285	-0.27427	-0.11493	0.091982	-0.28121
0.219494	0.025375	0.413747	0.092254	0.029835	0.001807	0.19823	0.303707	0.001362
-0.26745	-0.25545	0.030467	-0.33016	-0.25955	-0.19965	-0.14939	0.091245	-0.34571
0.002368	0.003748	0.733836	0.00015	0.003211	0.024425	0.093683	0.307611	6.87E-05
-0.21127	-0.29291	0.206308	-0.2193	-0.18312	-0.16813	0.020471	0.22928	0.187779
0.017111	0.000832	0.019961	0.013244	0.039335	0.058831	0.819304	0.009516	0.034509
-0.10967	0.170864	0.595587	0.204527	0.223886	0.310276	0.300555	0.702921	0.205027
0.21965	0.054776	1.52E-13	0.02108	0.011396	0.000385	0.000596	3.21E-20	0.020761
-0.51605	-0.17232	0.046849	-0.24602	-0.14417	-0.01711	-0.05543	0.101312	0.0481
5.32E-10	0.052713	0.600958	0.005302	0.105848	0.848542	0.535962	0.257062	0.591258
-0.51008	-0.67294	-0.50041	-0.78597	-0.72553	-0.56856	-0.4937	-0.50438	-0.46765
9.03E-10	4.49E-18	2.09E-09	7.12E-28	5.04E-22	3.11E-12	3.68E-09	1.49E-09	2.97E-08
-0.10153	-0.32566	-0.62466	-0.42436	-0.39315	-0.40772	-0.46122	-0.68713	-0.44484
0.25602	0.000187	4.23E-15	6.62E-07	4.83E-06	1.96E-06	4.84E-08	4.66E-19	1.61E-07
-0.30772	-0.47218	-0.48372	-0.56198	-0.54203	-0.47926	-0.42178	-0.48142	-0.3816
0.000433	2.09E-08	8.36E-09	6.22E-12	4.67E-11	1.2E-08	7.86E-07	1.01E-08	9.57E-06
-0.16901	-0.2759	-0.22339	-0.36817	-0.21259	-0.29816	-0.11973	-0.50096	-0.16821
0.057499	0.001691	0.011583	2.06E-05	0.016416	0.000662	0.180001	1.99E-09	0.058705

CYBB	CYP1B1	CYP27A1	CYP2E1	CYP2S1	CYP4V2	CYSLTR1	CYTH4	CYTIP
brown	brown	brown	brown	brown	brown	brown	brown	brown
0.262557	0.178948	0.373187	0.188325	0.021681	0.241215	0.174765	0.301575	0.144178
0.002863	0.044117	1.55E-05	0.033977	0.808821	0.006296	0.049393	0.00057	0.105835
0.064992	0.0923	0.324717	0.267353	-0.05631	0.190169	0.006319	0.12315	0.036791
0.46787	0.302028	0.000196	0.002377	0.529461	0.032235	0.943786	0.167783	0.681327
-0.27461	-0.28551	-0.61784	-0.12868	-0.04611	-0.14482	-0.10497	-0.3102	-0.06519
0.001781	0.001139	1.01E-14	0.149341	0.606721	0.104274	0.2402	0.000386	0.466487
0.010435	0.080486	-0.37595	-0.26994	-0.09635	0.217371	0.233957	-0.16084	0.26444
0.907306	0.368372	1.33E-05	0.002147	0.281239	0.014094	0.008113	0.070844	0.002662
-0.09195	0.067395	0.088748	-0.18886	-0.23436	0.102325	0.239018	0.074781	0.205556
0.303862	0.451538	0.321094	0.033459	0.008001	0.252315	0.006804	0.40339	0.020427
0.123612	0.302492	-0.09197	-0.4106	-0.20696	0.386011	0.489702	0.022661	0.474997
0.166179	0.000547	0.303768	1.63E-06	0.019562	7.39E-06	5.13E-09	0.800356	1.68E-08
0.049356	0.264101	-0.00353	-0.10628	-0.04717	0.156246	0.224534	0.009312	0.272014
0.581598	0.002697	0.968593	0.234335	0.598481	0.079398	0.011154	0.917242	0.001977
0.38822	0.155071	0.252266	-0.32379	-0.08192	0.312868	0.315787	0.191604	0.157163
6.48E-06	0.081713	0.00422	0.000204	0.3599	0.000342	0.000299	0.030931	0.077629
0.359613	0.50725	0.043738	-0.42146	0.388101	0.110121	0.194991	0.350384	0.534034
3.29E-05	1.16E-09	0.625367	8.04E-07	6.53E-06	0.217767	0.028032	5.38E-05	1.01E-10
0.85471	0.61117	0.64136	-0.48206	0.445497	0.419939	0.586371	0.903059	0.81342
2.12E-37	2.33E-14	4.54E-16	9.56E-09	1.53E-07	8.88E-07	4.38E-13	1.04E-47	3.36E-31
0.736221	0.497335	0.419451	-0.32618	0.249324	0.484119	0.559731	0.642135	0.611087
6.1E-23	2.71E-09	9.18E-07	0.000182	0.004702	8.1E-09	7.86E-12	4.08E-16	2.35E-14
-0.17252	-0.16149	0.003176	0.383843	0.120453	-0.20534	-0.42364	-0.07628	-0.2066
0.052439	0.069706	0.971734	8.4E-06	0.17736	0.020562	6.95E-07	0.393996	0.019784
-0.13859	-0.38095	-0.18957	0.229437	0.295654	-0.38682	-0.46011	-0.0387	-0.25318
0.120201	9.94E-06	0.032791	0.009465	0.000739	7.04E-06	5.26E-08	0.665743	0.00408
-0.12021	-0.30241	0.183166	0.052647	0.257054	-0.32089	-0.33449	0.179843	-0.16077
0.178244	0.000549	0.03928	0.556634	0.00353	0.000235	0.000121	0.043051	0.070981
0.443083	0.063223	0.483136	-0.1183	0.556438	-0.06057	-0.04796	0.632792	0.262628
1.82E-07	0.480097	8.77E-09	0.185275	1.1E-11	0.49875	0.592371	1.45E-15	0.002855
0.074875	-0.21447	0.451404	0.358289	0.01459	0.051937	-0.09558	0.142819	-0.3029
0.402799	0.015464	1E-07	3.53E-05	0.870675	0.561976	0.285101	0.109201	0.000537
-0.57519	-0.70603	-0.43929	0.509393	-0.17078	-0.48585	-0.58683	-0.52529	-0.79805
1.52E-12	1.85E-20	2.38E-07	9.6E-10	0.054899	7.03E-09	4.16E-13	2.29E-10	2.83E-29
-0.4772	-0.14448	-0.46435	0.266358	-0.49788	-0.03424	-0.08949	-0.69904	-0.47841
1.41E-08	0.105093	3.82E-08	0.002471	2.59E-09	0.70231	0.31703	6.3E-20	1.28E-08
-0.45737	-0.2564	-0.22759	0.457265	-0.35277	-0.25699	-0.32463	-0.53184	-0.65271
6.45E-08	0.003617	0.010073	6.5E-08	4.75E-05	0.003539	0.000196	1.24E-10	9.21E-17
-0.5126	-0.43636	-0.61078	0.333378	-0.12137	-0.47527	-0.30741	-0.3524	-0.28618
7.23E-10	2.92E-07	2.44E-14	0.000128	0.174074	1.64E-08	0.000439	4.84E-05	0.001107

DAB1	DAB2	DAP3	DAPP1	DAZAP2	DBNL	DCANP1	DCTD	DCUN1D2
brown	brown	brown	brown	brown	brown	brown	brown	brown
-0.23028	0.34405	0.122998	0.282576	0.256442	-0.00273	0.340693	0.200409	-0.04778
0.009199	7.48E-05	0.168313	0.001287	0.003612	0.975727	8.88E-05	0.023875	0.593722
-0.20959	0.189777	0.109178	0.093463	0.265346	-0.06918	0.201335	0.157625	0.09404
0.018035	0.032598	0.22176	0.295954	0.00257	0.439606	0.02322	0.076751	0.292971
0.289493	-0.22477	0.021224	-0.12987	-0.28308	-0.00902	-0.13643	-0.3552	0.017604
0.000963	0.011066	0.812776	0.145605	0.001261	0.919853	0.126151	4.17E-05	0.844264
-0.06204	0.070865	0.153907	0.17146	0.094604	-0.18029	-0.06077	-0.00658	-0.02689
0.488373	0.428529	0.08406	0.053923	0.290069	0.042528	0.497337	0.94148	0.764133
-0.12315	0.050074	0.560569	0.146195	0.222192	0.307604	-0.08815	0.044648	0.29214
0.167784	0.576106	7.21E-12	0.100991	0.01205	0.000435	0.324378	0.618182	0.00086
-0.28339	0.249062	0.503529	0.360898	0.360435	0.012089	-0.06303	0.226801	0.102041
0.001244	0.004748	1.6E-09	3.07E-05	3.15E-05	0.892693	0.481457	0.010343	0.253638
-0.13175	0.160038	0.420643	0.205404	0.349248	-0.04633	0.008122	0.154526	0.148639
0.139803	0.072287	8.48E-07	0.020522	5.71E-05	0.604983	0.927787	0.082806	0.095357
-0.35578	0.25481	0.262683	0.267206	0.383322	-0.04408	-0.04888	0.222091	-0.17115
4.04E-05	0.00384	0.002849	0.00239	8.66E-06	0.622649	0.585225	0.01209	0.054373
-0.18422	0.550466	0.357238	0.362687	0.085496	0.307443	0.192592	0.311012	-0.52181
0.038146	2.02E-11	3.74E-05	2.78E-05	0.339213	0.000438	0.03006	0.000372	3.15E-10
-0.64832	0.918813	0.60654	0.846844	0.562533	0.554631	0.469949	0.517849	-0.53469
1.72E-16	2.64E-52	4.11E-14	4.43E-36	5.87E-12	1.33E-11	2.49E-08	4.52E-10	9.48E-11
-0.557	0.673266	0.40152	0.723785	0.458703	0.340517	0.521072	0.534615	-0.45908
1.04E-11	4.27E-18	2.89E-06	7.05E-22	5.84E-08	8.96E-05	3.37E-10	9.54E-11	5.68E-08
0.083767	-0.12015	-0.08399	-0.24498	-0.06744	0.035662	0.204777	-0.01779	-0.06276
0.349106	0.178446	0.347849	0.005504	0.451233	0.690601	0.02092	0.842613	0.483312
0.29342	-0.14479	-0.27496	-0.22129	-0.37362	0.055584	0.097933	-0.36452	-0.25551
0.000814	0.104351	0.001757	0.012411	1.51E-05	0.534805	0.273352	2.52E-05	0.003741
0.085869	-0.10793	-0.0089	-0.14313	-0.25898	0.501093	0.06085	-0.21158	-0.16975
0.337103	0.227118	0.920877	0.108422	0.003282	1.97E-09	0.496762	0.016943	0.0564
-0.24391	0.481983	0.153185	0.319506	0.08084	0.52103	0.374712	0.107538	-0.56205
0.005721	9.62E-09	0.085541	0.000251	0.366263	3.39E-10	1.42E-05	0.228822	6.18E-12
-0.07431	-0.00752	-0.23883	-0.07625	0.170266	-0.02944	0.089383	-0.10082	0.188078
0.406345	0.933172	0.006848	0.394198	0.055642	0.742512	0.317633	0.259398	0.034216
0.638726	-0.75399	-0.61197	-0.6686	-0.50742	-0.26079	-0.24263	-0.57817	0.344388
6.51E-16	1.44E-24	2.11E-14	8.76E-18	1.14E-09	0.003063	0.005987	1.1E-12	7.35E-05
0.323691	-0.62252	-0.53185	-0.47463	-0.25501	-0.6088	-0.37752	-0.17747	0.550663
0.000205	5.57E-15	1.24E-10	1.73E-08	0.003812	3.12E-14	1.21E-05	0.045922	1.98E-11
0.426322	-0.64024	-0.55284	-0.58159	-0.32323	-0.44031	-0.29217	-0.20608	0.461452
5.81E-07	5.3E-16	1.59E-11	7.5E-13	0.00021	2.22E-07	0.000859	0.020098	4.76E-08
0.532904	-0.34372	-0.11314	-0.26905	-0.39435	-0.18547	-0.15852	-0.70016	0.414057
1.12E-10	7.61E-05	0.205332	0.002223	4.49E-06	0.036827	0.075075	5.19E-20	1.3E-06

DDAH1	DDB2	DDR2	DDX43	DEF6	DENND1C	DENND2D	DENND3	DENND5B
brown	brown	brown	brown	brown	brown	brown	brown	brown
-0.20383	0.24755	-0.03839	0.378567	0.258959	0.241695	0.332866	0.135771	-0.09985
0.021532	0.005016	0.668299	1.14E-05	0.003284	0.00619	0.000131	0.128012	0.264011
-0.09673	0.209105	-0.1569	0.359082	0.072609	0.084018	0.209308	0.045373	-0.03595
0.279306	0.018307	0.078134	3.39E-05	0.417225	0.347661	0.018192	0.612481	0.688247
0.150978	-0.14645	0.191813	-0.50311	-0.31996	-0.3162	-0.27534	-0.55545	0.339062
0.0902	0.100399	0.030746	1.66E-09	0.000245	0.000293	0.00173	1.22E-11	9.64E-05
0.034279	0.154162	0.480488	-0.25897	-0.24775	-0.25101	0.019343	-0.46028	0.251924
0.702015	0.083541	1.08E-08	0.003283	0.00498	0.004421	0.829109	5.19E-08	0.004274
-0.19394	0.258177	-0.11743	0.054005	-0.03629	-0.13546	0.149985	-0.2556	-0.11943
0.028907	0.003383	0.188551	0.546487	0.685459	0.128888	0.092362	0.003727	0.181089
-0.14636	0.397787	0.396604	0.000546	-0.13423	-0.18777	0.255273	-0.39771	0.046024
0.100609	3.64E-06	3.91E-06	0.995141	0.132433	0.034519	0.003774	3.65E-06	0.607385
-0.16042	0.415702	0.242109	0.181101	-0.12552	-0.13619	0.141966	-0.17424	-0.02799
0.071594	1.17E-06	0.0061	0.041589	0.159698	0.126822	0.111355	0.050085	0.754726
-0.01507	0.152263	0.390999	0.096062	0.170516	0.045751	0.332619	0.130515	-0.20527
0.866444	0.087463	5.49E-06	0.28267	0.055279	0.609519	0.000133	0.143591	0.020606
-0.56269	0.377006	0.414886	0.048658	0.315069	0.363962	0.373925	0.119013	-0.40352
5.78E-12	1.25E-05	1.24E-06	0.586956	0.000309	2.6E-05	1.49E-05	0.182633	2.55E-06
-0.70479	0.639929	0.413832	0.486671	0.857592	0.828274	0.908924	0.589579	-0.79216
2.31E-20	5.53E-16	1.32E-06	6.58E-09	6.66E-38	3.07E-33	2.54E-49	3.04E-13	1.4E-28
-0.41569	0.535526	0.479053	0.282054	0.634876	0.539295	0.726918	0.337098	-0.43125
1.17E-06	8.75E-11	1.22E-08	0.001315	1.1E-15	6.09E-11	3.85E-22	0.000106	4.16E-07
-0.16443	-0.13296	-0.38614	-0.02066	-0.00476	0.077886	-0.13386	0.072442	-0.06072
0.064707	0.136167	7.33E-06	0.817632	0.957664	0.384093	0.133518	0.418299	0.49767
0.019634	-0.24666	-0.05224	-0.28897	0.090678	0.156708	-0.19761	0.045063	-0.02768
0.826576	0.005182	0.559722	0.000985	0.310644	0.078503	0.025953	0.61492	0.757409
-0.15399	-0.05739	-0.29412	-0.21171	0.314733	0.249892	-0.01261	0.154434	-0.33254
0.083897	0.521564	0.00079	0.016875	0.000314	0.004606	0.888144	0.082991	0.000134
-0.47326	0.233499	0.069259	0.15323	0.749584	0.75324	0.462426	0.558023	-0.68182
1.92E-08	0.008242	0.439095	0.085448	3.76E-24	1.7E-24	4.42E-08	9.38E-12	1.1E-18
0.113884	-0.10172	-0.47974	0.376345	0.192992	0.224932	0.052396	0.371338	-0.12947
0.202356	0.255142	1.15E-08	1.3E-05	0.029714	0.011008	0.558526	1.72E-05	0.146864
0.609484	-0.68254	-0.52556	-0.35974	-0.43126	-0.41654	-0.70692	-0.18164	0.530094
2.87E-14	9.83E-19	2.23E-10	3.27E-05	4.15E-07	1.11E-06	1.58E-20	0.04097	1.46E-10
0.640617	-0.43642	-0.21424	-0.16751	-0.72664	-0.72191	-0.60647	-0.38272	0.772385
5.03E-16	2.91E-07	0.015581	0.059776	4.07E-22	1.01E-21	4.14E-14	8.97E-06	2.1E-26
0.549733	-0.55269	-0.48897	-0.02219	-0.50306	-0.48522	-0.59653	-0.01925	0.504102
2.18E-11	1.62E-11	5.45E-09	0.804403	1.67E-09	7.4E-09	1.36E-13	0.829945	1.52E-09
0.055285	-0.37205	-0.34638	-0.2532	-0.37353	-0.27537	-0.48878	-0.45042	0.365766
0.537006	1.66E-05	6.63E-05	0.004076	1.52E-05	0.001728	5.53E-09	1.08E-07	2.35E-05

DHRS3	DHRS7	DIAPH2	DIP2C	DLX1	DNAJC5B	DNASE1L1	DNASE2	DOCK10
brown	brown	brown	brown	brown	brown	brown	brown	brown
0.240215	0.432393	0.1777	0.128244	-0.00149	0.265625	0.365618	0.293992	0.246881
0.006523	3.84E-07	0.045639	0.150749	0.986751	0.002542	2.37E-05	0.000794	0.00514
0.165992	0.437451	0.075421	0.194622	0.077975	0.195512	0.320687	0.152333	0.186891
0.062167	2.71E-07	0.399369	0.028336	0.383548	0.027607	0.000237	0.087317	0.035387
-0.45928	-0.47672	-0.11257	-0.05721	0.245433	-0.19958	-0.63493	-0.27667	-0.32514
5.6E-08	1.46E-08	0.207642	0.52287	0.005416	0.024476	1.09E-15	0.001639	0.000192
-0.26266	-0.3486	0.163395	-0.4032	0.198613	0.003958	-0.50573	-0.07616	-0.16181
0.002851	5.91E-05	0.066428	2.6E-06	0.02519	0.96477	1.32E-09	0.394756	0.069153
0.329375	0.263726	-0.00029	-0.18134	-0.09804	0.147235	-0.01607	0.154472	-0.15815
0.000156	0.002737	0.997411	0.041314	0.272808	0.098563	0.857682	0.082915	0.07576
0.111781	-0.01621	0.278525	-0.55015	0.061904	0.201138	-0.26769	0.133681	-0.10623
0.210869	0.856486	0.00152	2.09E-11	0.489325	0.023359	0.002345	0.134044	0.234551
0.05902	0.064901	0.206466	-0.4106	-0.05998	0.138843	-0.07518	0.165879	0.021863
0.50982	0.468493	0.019864	1.63E-06	0.502956	0.119528	0.400865	0.062349	0.807245
0.389721	-0.07183	0.36538	-0.3598	-0.30853	0.183707	0.098103	0.216239	0.201838
5.93E-06	0.422281	2.4E-05	3.26E-05	0.000417	0.038694	0.27252	0.014616	0.022871
0.139259	0.103749	0.362348	-0.46351	-0.16115	0.221273	0.215583	0.381818	-0.00882
0.118414	0.245735	2.84E-05	4.07E-08	0.070307	0.012418	0.014926	9.45E-06	0.92158
0.753603	0.588955	0.597152	-0.42264	-0.51631	0.608217	0.616613	0.84768	0.450338
1.57E-24	3.26E-13	1.26E-13	7.43E-07	5.19E-10	3.35E-14	1.18E-14	3.23E-36	1.08E-07
0.480413	0.304653	0.418722	-0.48014	-0.17964	0.3537	0.32689	0.63481	0.27329
1.09E-08	0.000497	9.63E-07	1.12E-08	0.043295	4.52E-05	0.000176	1.11E-15	0.001879
-0.14778	0.211709	-0.35931	0.26164	0.041705	-0.26063	0.341633	-0.01904	-0.06015
0.097314	0.016876	3.34E-05	0.002965	0.641538	0.003081	8.46E-05	0.831713	0.501706
-0.2972	-0.2027	-0.20583	0.358323	-0.03498	-0.14834	-0.09503	-0.0626	-0.07596
0.000691	0.022284	0.020255	3.53E-05	0.69622	0.09604	0.287902	0.484431	0.395977
0.219875	0.107392	-0.30776	0.260396	-0.18882	-0.00283	0.159761	0.158665	-0.17531
0.012997	0.229457	0.000432	0.00311	0.0335	0.974848	0.072789	0.074801	0.048679
0.414907	0.348155	0.133649	0.095405	-0.4502	0.304083	0.515166	0.564944	0.262661
1.23E-06	6.05E-05	0.134137	0.28599	1.09E-07	0.00051	5.75E-10	4.56E-12	0.002851
0.130721	0.324319	-0.13122	0.546898	-0.12604	0.078263	0.315708	-0.01109	0.515717
0.142953	0.000199	0.141424	2.89E-11	0.15795	0.381789	0.0003	0.901513	5.48E-10
-0.53581	-0.31762	-0.56131	0.646964	0.330886	-0.48909	-0.38601	-0.61283	-0.13849
8.51E-11	0.000274	6.67E-12	2.08E-16	0.000145	5.39E-09	7.39E-06	1.9E-14	0.120476
-0.52097	-0.44429	-0.26475	0.090342	0.512129	-0.41393	-0.462	-0.69371	-0.20443
3.41E-10	1.67E-07	0.00263	0.312443	7.54E-10	1.32E-06	4.56E-08	1.56E-19	0.021144
-0.32614	-0.17066	-0.33274	0.386908	0.289129	-0.4218	-0.09983	-0.58036	-0.07823
0.000183	0.055065	0.000132	7.01E-06	0.000978	7.86E-07	0.264119	8.6E-13	0.381968
-0.58913	-0.18287	-0.27597	0.529765	0.107384	-0.21551	-0.37877	-0.41578	-0.16827
3.2E-13	0.039601	0.001687	1.51E-10	0.229493	0.014961	1.13E-05	1.17E-06	0.058619

DOCK2	DOCK8	DOK2	DOK3	DOK4	DPEP2	DPYD	DRAM1	DSC2
brown	brown	brown	brown	brown	brown	brown	brown	brown
0.254094	0.212519	0.256524	0.131506	-0.23341	0.261807	0.129473	0.122428	0.287389
0.003943	0.016451	0.003601	0.140549	0.008268	0.002946	0.146844	0.170306	0.001053
0.067365	0.030671	0.103762	-0.02872	-0.15317	0.166784	0.008831	0.000934	0.188286
0.45174	0.732119	0.245677	0.748572	0.085569	0.060914	0.921505	0.991685	0.034015
-0.36526	-0.31002	0.013651	-0.10532	0.310228	-0.42557	-0.11532	0.003312	-0.44915
2.42E-05	0.00039	0.878932	0.238598	0.000386	6.11E-07	0.196701	0.970521	1.18E-07
-0.12358	0.004587	0.06193	-0.06041	-0.06497	-0.32751	0.3072	0.406319	-0.15444
0.166308	0.959182	0.489142	0.499858	0.468004	0.000171	0.000443	2.14E-06	0.082976
-0.11896	-0.17133	0.124075	-0.08879	0.051597	0.232513	0.106555	0.07622	0.117679
0.182834	0.054112	0.164587	0.320883	0.564548	0.008525	0.233131	0.394382	0.187622
-0.01711	0.065408	0.166972	-0.05618	-0.17106	-0.04116	0.485078	0.511643	0.142376
0.848566	0.46502	0.060618	0.530434	0.054496	0.64587	7.49E-09	7.87E-10	0.110314
0.00447	0.017378	0.13413	-0.08878	-0.06789	0.036524	0.311452	0.403026	0.042261
0.960219	0.846242	0.132728	0.320913	0.448221	0.683514	0.000365	2.63E-06	0.637101
0.284542	0.340226	0.031065	0.166418	-0.51585	0.15054	0.400631	0.337899	0.277955
0.001186	9.09E-05	0.728808	0.06149	5.41E-10	0.09115	3.05E-06	0.000102	0.001556
0.322307	0.312153	0.621062	0.477755	-0.05589	0.110903	0.545243	0.688336	0.230097
0.00022	0.000353	6.72E-15	1.35E-08	0.532572	0.214498	3.4E-11	3.83E-19	0.009256
0.877999	0.799243	0.738079	0.820452	-0.54483	0.729112	0.774519	0.778104	0.75369
8.16E-42	2.03E-29	4.18E-23	3.84E-32	3.54E-11	2.51E-22	1.25E-26	5.2E-27	1.54E-24
0.665991	0.704216	0.517855	0.556145	-0.49091	0.35708	0.617156	0.703517	0.39523
1.3E-17	2.56E-20	4.52E-10	1.14E-11	4.64E-09	3.77E-05	1.1E-14	2.89E-20	4.25E-06
-0.06444	-0.04806	-0.06138	-0.00553	0.166453	-0.03725	-0.1931	-0.10037	-0.32536
0.4717	0.591607	0.493	0.950807	0.061434	0.677609	0.029623	0.261542	0.00019
-0.05605	-0.10934	0.068678	0.227481	0.190613	-0.1212	-0.29821	-0.18891	-0.33257
0.531358	0.221052	0.442951	0.01011	0.031827	0.174682	0.000661	0.033413	0.000133
-0.02092	-0.16067	0.195437	0.32466	0.086383	0.234856	-0.21607	-0.22193	-0.07752
0.815422	0.071162	0.027668	0.000196	0.334208	0.007865	0.014694	0.012155	0.386333
0.55317	0.422842	0.529899	0.775297	-0.25902	0.525427	0.211699	0.257815	0.303345
1.54E-11	7.33E-07	1.49E-10	1.04E-26	0.003276	2.26E-10	0.016881	0.00343	0.000527
0.166974	0.134171	-0.24085	0.021312	-0.1613	0.363738	-0.31963	-0.44684	0.121678
0.060615	0.132611	0.006379	0.812016	0.070044	2.63E-05	0.000249	1.39E-07	0.172961
-0.51809	-0.51603	-0.59142	-0.46282	0.467935	-0.39718	-0.87014	-0.84474	-0.62507
4.43E-10	5.33E-10	2.46E-13	4.29E-08	2.9E-08	3.77E-06	3.14E-40	9.69E-36	4.01E-15
-0.55128	-0.41253	-0.67915	-0.79906	0.283611	-0.55703	-0.38918	-0.49047	-0.33753
1.86E-11	1.44E-06	1.69E-18	2.14E-29	0.001233	1.04E-11	6.12E-06	4.81E-09	0.000104
-0.42241	-0.3963	-0.66319	-0.66999	0.33999	-0.33426	-0.60756	-0.71853	-0.2827
7.55E-07	3.98E-06	1.98E-17	7.08E-18	9.2E-05	0.000123	3.63E-14	1.9E-21	0.001281
-0.45157	-0.50096	-0.06953	-0.21651	0.623941	-0.21388	-0.51376	-0.41103	-0.40167
9.89E-08	1.99E-09	0.437308	0.014491	4.64E-15	0.015758	6.53E-10	1.59E-06	2.86E-06

DSE	DUSP1	DUSP23	DZIP1	EARS2	EBI3	ECM2	EFEMP1	EHBP1L1
brown	brown	brown	brown	brown	brown	brown	brown	brown
0.196283	0.024921	0.194981	-0.3196	-0.22281	0.150983	0.039318	0.163774	0.075621
0.026988	0.780929	0.02804	0.00025	0.011806	0.090191	0.660749	0.065793	0.398114
0.02445	0.005586	0.149572	-0.24669	-0.15796	0.11158	-0.04146	0.059405	-0.08231
0.784963	0.950297	0.093272	0.005174	0.076116	0.211697	0.643526	0.50706	0.357611
-0.0257	-0.35969	-0.44861	0.168001	0.41158	-0.42753	-0.0936	-0.19924	-0.25284
0.774238	3.28E-05	1.23E-07	0.059026	1.53E-06	5.35E-07	0.295243	0.024722	0.004131
0.339118	-0.16351	-0.46454	0.165446	0.420501	-0.50876	0.339009	0.173126	-0.25987
9.62E-05	0.06624	3.76E-08	0.063044	8.56E-07	1.01E-09	9.67E-05	0.051599	0.003173
0.06246	-0.06363	0.52469	-0.17881	-0.16197	0.14151	0.051011	-0.05456	-0.2349
0.485423	0.477301	2.42E-10	0.044278	0.068863	0.11252	0.568982	0.542396	0.007854
0.467817	-0.02371	-0.00477	-0.03742	0.18124	-0.26461	0.462727	0.248486	-0.30212
2.93E-08	0.791298	0.957523	0.67622	0.04143	0.002645	4.32E-08	0.004849	0.000556
0.291734	-0.01195	-0.07428	0.14525	0.232972	-0.09963	0.285573	0.259444	-0.18393
0.000875	0.893881	0.406534	0.103239	0.008392	0.265078	0.001136	0.003225	0.038456
0.384914	0.101595	0.06278	-0.0105	-0.08017	-0.02662	0.635854	0.371345	0.118948
7.89E-06	0.255729	0.483184	0.906758	0.370264	0.766419	9.62E-16	1.72E-05	0.182875
0.611782	0.30326	0.064722	-0.05902	0.125345	0.102564	0.307215	0.448508	0.443887
2.16E-14	0.000529	0.469724	0.509825	0.160277	0.251201	0.000443	1.24E-07	1.72E-07
0.776592	0.488381	0.556082	-0.5264	-0.52696	0.617239	0.582269	0.722414	0.689402
7.55E-27	5.72E-09	1.14E-11	2.07E-10	1.96E-10	1.09E-14	6.95E-13	9.16E-22	3.21E-19
0.68332	0.241245	0.220674	-0.46385	-0.24794	0.231877	0.565667	0.633468	0.411317
8.68E-19	0.00629	0.012663	3.97E-08	0.004947	0.008712	4.22E-12	1.33E-15	1.56E-06
-0.25162	-0.00635	-0.00712	-0.05214	0.071149	0.000427	-0.22369	-0.03548	0.049721
0.004322	0.943499	0.936689	0.560456	0.426679	0.996198	0.011471	0.692121	0.578805
-0.2521	-0.18386	-0.2687	0.06934	0.077495	-0.00086	-0.31829	-0.12327	0.284604
0.004246	0.038526	0.002254	0.438557	0.386491	0.992314	0.000266	0.167382	0.001183
-0.29351	-0.08197	0.447352	-0.24444	-0.33687	0.419879	-0.36654	-0.13809	0.380208
0.000811	0.359593	1.34E-07	0.005613	0.000108	8.92E-07	2.25E-05	0.121554	1.04E-05
0.193255	0.212165	0.392254	-0.39531	-0.48298	0.598306	0.05287	0.329185	0.780958
0.029488	0.016636	5.09E-06	4.23E-06	8.88E-09	1.1E-13	0.554963	0.000158	2.55E-27
-0.40937	-0.03612	0.214494	-0.21172	-0.45923	0.342896	-0.18798	-0.11276	0.054703
1.76E-06	0.686869	0.015453	0.01687	5.62E-08	7.93E-05	0.034311	0.206867	0.54131
-0.80299	-0.35484	-0.32344	0.26207	0.122018	-0.1874	-0.61903	-0.66144	-0.34136
7.1E-30	4.25E-05	0.000208	0.002917	0.171754	0.034885	8.7E-15	2.57E-17	8.58E-05
-0.38216	-0.19357	-0.49151	0.435033	0.408335	-0.54138	-0.20988	-0.44213	-0.66894
9.27E-06	0.029219	4.42E-09	3.2E-07	1.88E-06	4.97E-11	0.017871	1.95E-07	8.31E-18
-0.59721	-0.08957	-0.18986	0.345549	0.147916	-0.16603	-0.44067	-0.50805	-0.42695
1.25E-13	0.316607	0.032523	6.92E-05	0.096998	0.0621	2.16E-07	1.08E-09	5.57E-07
-0.36287	-0.39106	-0.21934	0.258776	0.186509	-0.19412	-0.59165	-0.57134	-0.26003
2.76E-05	5.47E-06	0.013224	0.003307	0.035771	0.028754	2.4E-13	2.31E-12	0.003154

EID2	ELF1	ELL2	ELOVL2	EMB	EMILIN3	EMP2	EMP3	ENOSF1
brown	brown	brown	brown	brown	brown	brown	brown	brown
0.023064	0.161026	0.262738	-0.36376	0.209022	-0.37648	0.230354	0.088627	0.361423
0.79688	0.070521	0.002843	2.62E-05	0.018355	1.29E-05	0.009176	0.321756	2.98E-05
0.166405	0.008255	0.257534	-0.27158	0.032072	-0.21568	0.127211	0.035519	0.312779
0.061511	0.926606	0.003466	0.002011	0.720381	0.014879	0.154094	0.691776	0.000343
0.113627	0.045226	-0.44663	0.498705	-0.21271	0.470564	-0.18601	-0.03122	-0.2752
0.203383	0.613635	1.41E-07	2.42E-09	0.016354	2.37E-08	0.036278	0.727539	0.00174
0.039368	0.397075	-0.04332	0.481242	0.15799	0.32161	0.01419	0.280178	-0.05493
0.66034	3.8E-06	0.628714	1.02E-08	0.076061	0.000227	0.874191	0.001421	0.539616
-0.04073	-0.23257	0.000995	-0.15238	0.048187	-0.02024	0.125617	0.116182	0.268753
0.649386	0.008507	0.991142	0.087228	0.590586	0.821284	0.159362	0.193338	0.002249
-0.03291	0.301745	0.176106	0.199357	0.345786	0.126587	0.22101	0.400806	0.218121
0.713368	0.000566	0.047648	0.024638	6.84E-05	0.156141	0.012525	3.02E-06	0.013758
-0.00388	0.231797	0.173964	0.019302	0.19965	-0.00226	0.204042	0.307884	0.148592
0.965498	0.008736	0.050461	0.829466	0.024423	0.979902	0.021393	0.000429	0.095462
-0.215	0.106389	0.096127	-0.16344	0.324772	-0.257	0.163693	0.296762	0.138132
0.015206	0.23386	0.28234	0.066361	0.000195	0.003537	0.065929	0.000704	0.121451
-0.38144	0.529054	0.157231	-0.05066	0.455031	-0.07301	0.421351	0.531526	-0.04129
9.66E-06	1.61E-10	0.077499	0.571635	7.67E-08	0.41463	8.09E-07	1.28E-10	0.644828
-0.61388	0.62082	0.580556	-0.51879	0.764356	-0.4947	0.493394	0.644006	0.44137
1.66E-14	6.93E-15	8.41E-13	4.15E-10	1.39E-25	3.39E-09	3.78E-09	3.15E-16	2.06E-07
-0.40942	0.592366	0.407947	-0.18986	0.617617	-0.3241	0.3155	0.57018	0.331883
1.76E-06	2.21E-13	1.93E-06	0.032525	1.04E-14	0.000201	0.000303	2.61E-12	0.000138
-0.0188	-0.05342	-0.09572	-0.00849	-0.31287	0.039588	-0.13784	-0.0664	-0.00145
0.833863	0.550828	0.284402	0.924522	0.000342	0.658565	0.122256	0.458292	0.987101
-0.08663	-0.02597	-0.3683	0.124259	-0.3401	0.141418	-0.24284	-0.14516	-0.30016
0.332846	0.771984	2.04E-05	0.163957	9.15E-05	0.112756	0.005943	0.103464	0.000607
-0.25322	-0.32656	-0.26564	-0.10682	-0.30088	0.010376	-0.12468	-0.02763	-0.19587
0.004074	0.000179	0.002541	0.231941	0.000588	0.90783	0.162506	0.757771	0.027319
-0.55024	0.233628	0.094595	-0.36832	0.169901	-0.26561	0.163315	0.292192	0.065751
2.07E-11	0.008205	0.290118	2.04E-05	0.056177	0.002544	0.066563	0.000858	0.462676
0.08458	-0.2273	0.124632	-0.32537	-0.20233	-0.19394	-0.1134	-0.29144	0.322157
0.344436	0.01017	0.162686	0.00019	0.022533	0.028901	0.204287	0.000886	0.000221
0.437852	-0.52437	-0.53961	0.244986	-0.72196	0.320128	-0.55968	-0.69012	-0.34695
2.63E-07	2.49E-10	5.9E-11	0.005503	9.97E-22	0.000243	7.9E-12	2.85E-19	6.44E-05
0.638199	-0.28238	-0.10136	0.400907	-0.26152	0.275913	-0.26937	-0.48663	-0.22515
7E-16	0.001298	0.256854	3E-06	0.002979	0.001691	0.002196	6.6E-09	0.010927
0.537364	-0.55121	-0.13336	0.020489	-0.40545	0.022912	-0.30856	-0.64127	-0.19302
7.33E-11	1.88E-11	0.134992	0.819148	2.26E-06	0.79819	0.000416	4.6E-16	0.029686
0.202564	-0.18219	-0.48599	0.024262	-0.394	0.173001	-0.20724	-0.55618	0.021219
0.022375	0.040355	6.95E-09	0.78658	4.58E-06	0.05177	0.019394	1.13E-11	0.812821

ENTPD1	EOMES	EPB41L2	EPHA1	EPM2AIP1	ERMAP	ERP29	ESR1	ESRRG
brown	brown	brown	brown	brown	brown	brown	brown	brown
-0.06801	0.197305	0.177377	0.25035	-0.08646	0.159421	0.213852	0.168858	-0.18326
0.447441	0.026186	0.046041	0.004529	0.333762	0.073409	0.015772	0.057726	0.03918
-0.13747	0.054497	0.093514	0.118349	-0.03263	0.052089	0.156949	0.02427	-0.10828
0.123269	0.542837	0.295691	0.185105	0.715749	0.560833	0.07804	0.786508	0.225603
-0.06486	-0.0713	-0.25959	-0.15383	0.135824	-0.24357	-0.17022	-0.06058	0.197699
0.468796	0.425672	0.003207	0.084218	0.127862	0.00579	0.055704	0.498687	0.025883
0.153618	-0.01414	-0.21229	0.026512	0.237168	0.159123	-0.03208	0.308398	0.155659
0.084651	0.874654	0.016571	0.767331	0.007259	0.073954	0.720297	0.00042	0.080548
-0.02585	0.150118	-0.40155	0.202013	-0.25214	-0.03391	0.526122	0.036196	0.068068
0.772981	0.09207	2.88E-06	0.022751	0.00424	0.705048	2.12E-10	0.686205	0.447021
0.213322	0.119077	-0.33116	0.243832	0.018728	0.248751	0.356761	0.432471	0.106823
0.016039	0.182398	0.000143	0.005736	0.834456	0.004802	3.84E-05	3.82E-07	0.231948
0.229948	0.026296	-0.19918	0.156578	0.110759	0.220519	0.433199	0.374306	0.097293
0.009303	0.769165	0.024768	0.078755	0.215097	0.012728	3.64E-07	1.46E-05	0.276514
0.273302	0.009233	0.030476	0.192809	0.147344	0.511377	0.084183	0.367816	-0.13585
0.001878	0.917944	0.733758	0.029872	0.09831	8.06E-10	0.346712	2.1E-05	0.127774
0.496997	0.273655	0.089419	0.341755	-0.2777	0.323685	0.308499	0.459614	-0.36481
2.79E-09	0.001851	0.317435	8.41E-05	0.001572	0.000206	0.000418	5.46E-08	2.48E-05
0.645289	0.503285	0.385157	0.69748	-0.54551	0.667591	0.592936	0.613515	-0.66133
2.63E-16	1.63E-09	7.77E-06	8.23E-20	3.32E-11	1.02E-17	2.06E-13	1.74E-14	2.62E-17
0.451677	0.45112	0.292874	0.506394	-0.295	0.621023	0.399472	0.545438	-0.42748
9.82E-08	1.02E-07	0.000833	1.25E-09	0.00076	6.75E-15	3.28E-06	3.34E-11	5.37E-07
-0.15253	-0.00452	0.160634	-0.22315	-0.08632	0.05693	0.02505	-0.26279	-0.15367
0.086905	0.959739	0.071218	0.011677	0.334553	0.524946	0.779824	0.002837	0.084553
-0.08326	0.001818	0.184014	-0.15974	0.008663	-0.15131	-0.30895	-0.29397	-0.14695
0.352071	0.983815	0.038364	0.072821	0.922996	0.089491	0.000409	0.000795	0.099226
-0.11693	0.170514	-0.03744	0.048008	-0.40456	-0.18619	0.027547	-0.34995	-0.20648
0.190467	0.055282	0.676048	0.59197	2.39E-06	0.03609	0.758514	5.51E-05	0.019857
0.341489	0.36264	0.447947	0.354161	-0.48823	0.286972	0.168148	0.062085	-0.62702
8.53E-05	2.79E-05	1.29E-07	4.41E-05	5.79E-09	0.001071	0.058802	0.488048	3.11E-15
-0.04767	-0.13294	0.490865	-0.04923	-0.05893	-0.07456	-0.15879	-0.26952	-0.18831
0.594574	0.136219	4.66E-09	0.58254	0.510434	0.404812	0.074574	0.002182	0.033988
-0.46357	-0.44159	0.027824	-0.62571	0.256737	-0.60921	-0.58235	-0.69842	0.350722
4.05E-08	2.03E-07	0.756166	3.69E-15	0.003572	2.96E-14	6.89E-13	7.01E-20	5.29E-05
-0.41994	-0.46535	-0.27501	-0.47851	0.546986	-0.41549	-0.44206	-0.21154	0.675558
8.88E-07	3.54E-08	0.001754	1.27E-08	2.86E-11	1.19E-06	1.96E-07	0.016966	2.99E-18
-0.49326	-0.44152	-0.09555	-0.51071	0.356534	-0.47889	-0.39472	-0.44417	0.471778
3.82E-09	2.04E-07	0.285229	8.55E-10	3.88E-05	1.23E-08	4.39E-06	1.69E-07	2.16E-08
-0.31437	-0.09391	-0.20247	-0.28343	0.282864	-0.54387	-0.10901	-0.32279	0.261557
0.000319	0.293629	0.022437	0.001242	0.001272	3.9E-11	0.222456	0.000215	0.002975

ESYT1	ETV7	EVI2B	F11R	F13A1	F5	FABP4	FAH	FAM117B
brown	brown	brown	brown	brown	brown	brown	brown	brown
0.285536	0.294464	0.297129	0.113044	0.165801	0.249393	0.267944	0.26388	-0.18839
0.001138	0.000778	0.000693	0.205728	0.062473	0.004691	0.002322	0.00272	0.03391
0.197756	0.167988	0.173047	-0.01117	0.027606	0.229182	0.180265	0.26993	-0.11852
0.025839	0.059047	0.051707	0.900775	0.758016	0.009547	0.042556	0.002147	0.184472
-0.19071	-0.08056	-0.42348	-0.20224	-0.18487	-0.55356	0.029477	-0.49675	0.227092
0.031737	0.367938	7.02E-07	0.022597	0.037459	1.48E-11	0.742175	2.85E-09	0.010243
-0.02125	0.193278	-0.06902	0.115814	0.17935	-0.39128	0.21856	-0.18924	-0.0298
0.812567	0.029468	0.440698	0.19476	0.043636	5.4E-06	0.013564	0.033102	0.739466
-0.18535	0.218909	0.181426	-0.20868	0.101356	0.101612	0.289013	0.377428	-0.08873
0.036956	0.013411	0.041219	0.01855	0.256854	0.255653	0.000983	1.22E-05	0.3212
-0.0161	0.423381	0.248189	0.1144	0.367986	-0.12031	0.415633	0.256995	-0.17256
0.857408	7.07E-07	0.004901	0.200305	2.08E-05	0.177867	1.18E-06	0.003537	0.052382
-0.02215	0.312682	0.161747	0.137267	0.33953	0.027838	0.344267	0.242363	-0.2452
0.804781	0.000345	0.069254	0.123825	9.42E-05	0.756046	7.4E-05	0.006045	0.005461
0.337246	0.173536	0.293816	0.348764	0.271155	0.339955	0.003157	0.240311	-0.38665
0.000106	0.05104	0.0008	5.86E-05	0.002046	9.22E-05	0.971898	0.006501	7.12E-06
0.436603	0.397226	0.197094	0.563148	0.611779	0.053672	0.414152	0.097765	-0.3531
2.87E-07	3.76E-06	0.02635	5.51E-12	2.16E-14	0.54897	1.3E-06	0.274182	4.66E-05
0.628521	0.707081	0.867885	0.740745	0.723978	0.547714	0.504462	0.736815	-0.70508
2.55E-15	1.54E-20	8.55E-40	2.42E-23	6.79E-22	2.66E-11	1.48E-09	5.41E-23	2.19E-20
0.586668	0.769462	0.594972	0.654631	0.524551	0.245445	0.388855	0.491015	-0.5645
4.24E-13	4.22E-26	1.63E-13	6.98E-17	2.45E-10	0.005413	6.24E-06	4.6E-09	4.78E-12
0.058918	-0.14285	-0.21453	-0.02018	-0.09774	0.015137	-0.08309	-0.1195	-0.04756
0.51055	0.10912	0.015434	0.82179	0.274325	0.86587	0.353023	0.180847	0.595407
0.090075	-0.29994	-0.35945	0.017001	-0.28427	-0.16182	-0.2281	-0.43002	0.057542
0.313884	0.000613	3.32E-05	0.849533	0.0012	0.069134	0.0099	4.52E-07	0.520493
0.038157	-0.12351	-0.14795	-0.05062	-0.2642	0.055967	-0.23667	-0.01637	-0.04747
0.670176	0.166539	0.096923	0.571931	0.002687	0.531988	0.007385	0.855092	0.596141
0.492278	0.18183	0.333172	0.50455	0.174381	0.353977	0.059621	0.264473	-0.45373
4.14E-09	0.040762	0.000129	1.46E-09	0.049903	4.45E-05	0.505509	0.002659	8.44E-08
-0.05104	-0.168	0.216608	-0.13243	-0.34086	0.303833	-0.1685	0.157439	-0.05646
0.568739	0.059027	0.014444	0.13776	8.8E-05	0.000515	0.058272	0.077103	0.528404
-0.53826	-0.67076	-0.64084	-0.65833	-0.77785	-0.39298	-0.52203	-0.64033	0.55986
6.73E-11	6.29E-18	4.88E-16	4.07E-17	5.54E-27	4.87E-06	3.09E-10	5.23E-16	7.76E-12
-0.53558	-0.35147	-0.42549	-0.55479	-0.3622	-0.40202	-0.28557	-0.43372	0.627793
8.7E-11	5.08E-05	6.14E-07	1.31E-11	2.86E-05	2.8E-06	0.001136	3.51E-07	2.81E-15
-0.45667	-0.51159	-0.41105	-0.57875	-0.45043	-0.0831	-0.43177	-0.32246	0.549371
6.8E-08	7.91E-10	1.58E-06	1.03E-12	1.08E-07	0.352967	4.01E-07	0.000218	2.26E-11
-0.48113	-0.38566	-0.41581	-0.55609	-0.33597	-0.30183	0.09235	-0.50677	0.436295
1.03E-08	7.55E-06	1.16E-06	1.14E-11	0.000113	0.000564	0.301765	1.21E-09	2.94E-07

FAM161A	FAM168B	FAM177B	FAM196A	FAM198B	FAM20A	FAM214B	FAM84A	FAS
brown	brown	brown	brown	brown	brown	brown	brown	brown
-0.27172	-0.03266	0.03665	-0.41283	0.1423	0.196067	0.339126	-0.11169	0.364792
0.002	0.715492	0.68248	1.41E-06	0.110506	0.027161	9.61E-05	0.211254	2.48E-05
-0.27877	0.0105	-0.04573	-0.36205	0.070041	0.007249	0.373694	-0.0025	0.32632
0.001505	0.906733	0.609678	2.88E-05	0.433934	0.93553	1.51E-05	0.977763	0.000181
0.426492	0.016246	-0.09951	0.545825	-0.20238	-0.07603	-0.49205	0.102451	-0.27088
5.74E-07	0.85614	0.265666	3.21E-11	0.022497	0.395564	4.22E-09	0.251728	0.002068
0.469294	-0.15696	0.102855	0.455639	0.23162	0.107358	-0.46272	0.078419	0.190814
2.61E-08	0.078022	0.249851	7.34E-08	0.008789	0.229606	4.32E-08	0.380841	0.031643
-0.11909	-0.58614	0.250529	-0.15203	-0.06308	-0.00224	0.114287	-0.08435	0.153025
0.182349	4.5E-13	0.004499	0.087945	0.481071	0.98007	0.200751	0.345781	0.085871
0.226911	-0.53032	0.333883	0.121046	0.354231	0.185126	-0.20268	-0.0146	0.460655
0.010305	1.43E-10	0.000125	0.175218	4.39E-05	0.037189	0.022299	0.870587	5.05E-08
-0.05037	-0.29466	0.249298	0.118272	0.118519	0.105767	-0.07799	0.004621	0.451751
0.573856	0.000772	0.004707	0.185392	0.184468	0.236624	0.383433	0.958878	9.76E-08
0.180139	-0.1972	0.288747	-0.40164	0.41493	0.163427	0.094175	-0.3892	0.38145
0.042703	0.026265	0.000994	2.87E-06	1.23E-06	0.066375	0.292274	6.12E-06	9.66E-06
-0.28651	-0.32312	0.184198	0.172519	0.162508	0.708808	-0.03207	-0.11091	0.387016
0.001092	0.000211	0.038167	0.052436	0.067938	1.13E-20	0.720435	0.214476	6.97E-06
-0.52158	-0.60866	0.530322	-0.49776	0.40553	0.820031	0.533661	-0.5153	0.664981
3.22E-10	3.17E-14	1.43E-10	2.62E-09	2.25E-06	4.38E-32	1.04E-10	5.69E-10	1.52E-17
-0.25396	-0.41914	0.301793	-0.35673	0.490583	0.593949	0.241546	-0.34392	0.538271
0.003963	9.36E-07	0.000564	3.84E-05	4.77E-09	1.84E-13	0.006223	7.53E-05	6.72E-11
-0.15621	0.19955	-0.47041	0.224492	-0.30508	-0.01237	0.044039	-0.00872	-0.24546
0.079473	0.024496	2.4E-08	0.011169	0.000487	0.890243	0.622986	0.922497	0.005411
0.013804	0.343627	-0.29577	0.248529	-0.36762	0.004661	-0.07311	-0.09307	-0.43554
0.877586	7.64E-05	0.000735	0.004841	2.12E-05	0.958523	0.41402	0.298	3.09E-07
-0.2949	-0.14417	-0.124	-0.09977	-0.32886	0.004022	0.23458	-0.13383	-0.36759
0.000764	0.105862	0.164829	0.264425	0.00016	0.964206	0.00794	0.133599	2.12E-05
-0.51771	-0.17756	0.0913	-0.30678	-0.01058	0.502487	0.490494	-0.45335	0.045002
4.58E-10	0.045818	0.307324	0.000451	0.905982	1.75E-09	4.81E-09	8.68E-08	0.615395
-0.28006	0.251328	-0.06038	-0.38962	-0.08385	-0.2408	0.537358	-0.12345	-0.11848
0.001428	0.004369	0.500106	5.97E-06	0.348633	0.006389	7.34E-11	0.166738	0.184628
0.222149	0.653812	-0.46503	0.309225	-0.55789	-0.6842	-0.18803	0.338994	-0.78408
0.012067	7.85E-17	3.63E-08	0.000404	9.51E-12	7.53E-19	0.034266	9.68E-05	1.16E-27
0.536481	0.45749	-0.22423	0.296696	-0.00194	-0.62448	-0.50411	0.571789	-0.23161
7.98E-11	6.4E-08	0.011265	0.000706	0.9827	4.32E-15	1.52E-09	2.2E-12	0.008791
0.15346	0.578056	-0.31268	0.005064	-0.28016	-0.64313	-0.15588	0.421919	-0.3926
0.084974	1.11E-12	0.000345	0.954944	0.001422	3.56E-16	0.080108	7.79E-07	4.99E-06
0.229968	0.199355	-0.17794	0.454363	-0.67136	-0.22255	-0.27881	0.109293	-0.49454
0.009297	0.02464	0.045346	8.06E-08	5.74E-18	0.011909	0.001503	0.221271	3.43E-09

FASLG	FAT3	FBLN5	FBP1	FBXO21	FBXO32	FBXW12	FCER1A	FCER1G
brown	brown	brown	brown	brown	brown	brown	brown	brown
0.358402	-0.19972	0.130106	0.123988	-0.20013	0.168169	-0.11693	0.215808	0.333339
3.51E-05	0.024372	0.14486	0.164885	0.024076	0.058769	0.190477	0.014819	0.000128
0.162778	-0.16423	0.061719	0.054082	-0.13874	0.100309	-0.06832	0.083841	0.209276
0.067476	0.06503	0.490625	0.545921	0.119793	0.261826	0.445312	0.348681	0.01821
-0.05643	0.22082	-0.12698	-0.16614	0.081941	-0.36842	0.296474	-0.1191	-0.25876
0.528601	0.012603	0.154862	0.061929	0.359751	2.03E-05	0.000713	0.182314	0.003309
0.160792	0.085385	0.128542	0.008548	0.091038	0.024423	0.019276	0.167415	-0.00792
0.070935	0.339843	0.149795	0.924016	0.308717	0.785197	0.829686	0.059929	0.929575
0.12436	-0.41057	-0.16581	0.231858	-0.25632	-0.18406	-0.15205	0.224634	0.201919
0.163613	1.63E-06	0.062461	0.008718	0.003628	0.038313	0.087911	0.011117	0.022815
0.308818	-0.28883	0.130815	0.238284	-0.09254	0.080094	-0.23378	0.411896	0.269251
0.000412	0.00099	0.142666	0.006981	0.300774	0.370718	0.008162	1.5E-06	0.002206
0.125925	-0.13345	0.064868	0.185336	-0.06058	0.161843	-0.10667	0.269594	0.206443
0.158335	0.134715	0.468719	0.036971	0.498674	0.069087	0.232615	0.002176	0.019878
0.161158	0.030486	0.147585	0.175335	-0.05826	0.417106	-0.22157	0.28289	0.245551
0.070288	0.733676	0.097756	0.048645	0.515292	1.07E-06	0.012299	0.00127	0.005393
0.329344	-0.33647	0.551591	0.428758	-0.29324	0.361397	-0.32067	0.242271	0.423119
0.000156	0.00011	1.81E-11	4.93E-07	0.00082	2.99E-05	0.000237	0.006065	7.2E-07
0.606723	-0.60068	0.509835	0.827375	-0.63468	0.653341	-0.51991	0.536206	0.941719
4.02E-14	8.31E-14	9.23E-10	4.14E-33	1.13E-15	8.41E-17	3.75E-10	8.2E-11	5.44E-61
0.671901	-0.38396	0.458089	0.454537	-0.42106	0.481565	-0.3758	0.383942	0.67245
5.27E-18	8.34E-06	6.12E-08	7.96E-08	8.25E-07	9.95E-09	1.34E-05	8.35E-06	4.85E-18
-0.04168	0.011053	0.050339	-0.13227	0.089724	0.061492	0.027227	-0.38705	-0.15511
0.641758	0.901839	0.574091	0.138245	0.31578	0.492223	0.761237	6.95E-06	0.081636
-0.16671	0.327581	-0.01268	-0.09538	-0.05372	-0.07462	0.315756	-0.36299	-0.21227
0.061032	0.00017	0.887476	0.286137	0.548597	0.404391	0.000299	2.74E-05	0.016582
-0.12628	-0.09717	-0.08925	0.14003	-0.35391	-0.15309	0.10082	-0.2318	-0.06313
0.157148	0.277135	0.31834	0.116369	4.47E-05	0.085732	0.259394	0.008734	0.480738
0.203137	-0.2396	0.293543	0.508909	-0.5536	0.38277	-0.15201	0.029837	0.457126
0.02199	0.006665	0.00081	1E-09	1.47E-11	8.94E-06	0.087993	0.73914	6.57E-08
-0.13891	-0.01264	-0.29233	-0.0408	-0.08548	0.056297	0.088166	-0.12535	0.036702
0.11934	0.887841	0.000853	0.648757	0.339295	0.529571	0.324293	0.160264	0.682053
-0.5587	0.544632	-0.5472	-0.62218	0.394981	-0.60326	0.565812	-0.58814	-0.70562
8.74E-12	3.61E-11	2.8E-11	5.82E-15	4.32E-06	6.11E-14	4.16E-12	3.59E-13	2E-20
-0.33912	0.403779	-0.35348	-0.69406	0.692992	-0.42032	0.245715	-0.18722	-0.64814
9.61E-05	2.51E-06	4.57E-05	1.47E-19	1.76E-19	8.66E-07	0.005361	0.035061	1.76E-16
-0.46616	0.384877	-0.40855	-0.64353	0.567007	-0.40149	0.26335	-0.34222	-0.58865
3.33E-08	7.9E-06	1.86E-06	3.36E-16	3.66E-12	2.89E-06	0.002777	8.21E-05	3.38E-13
-0.1535	0.241691	-0.45684	-0.25215	0.060245	-0.50454	0.485102	-0.2347	-0.34563
0.084885	0.006191	6.71E-08	0.004239	0.50106	1.47E-09	7.48E-09	0.007908	6.89E-05

FCGBP	FCGR1A	FCGR1B	FCGR2A	FCGR2B	FCGR2C	FCGR3A	FCGRT	FCN1
brown	brown	brown	brown	brown	brown	brown	brown	brown
-0.04115	0.359136	0.422089	0.300626	0.271123	0.303294	0.361691	0.114205	0.084971
0.645981	3.38E-05	7.71E-07	0.000594	0.002048	0.000528	2.94E-05	0.201079	0.342201
-0.09627	0.264349	0.327237	0.175325	0.150059	0.211956	0.2157	-0.01651	-0.03934
0.281637	0.002671	0.000173	0.048659	0.0922	0.016745	0.01487	0.853853	0.660609
0.195047	-0.34626	-0.31611	-0.2769	-0.09887	-0.05008	-0.27046	-0.22183	-0.34761
0.027986	6.67E-05	0.000294	0.001624	0.268787	0.576097	0.002103	0.012193	6.22E-05
0.153538	-0.04884	-0.03552	0.056696	0.288403	0.299631	-0.00268	-0.17504	-0.29412
0.084813	0.585547	0.691741	0.526654	0.001008	0.000621	0.976182	0.049036	0.00079
-0.07038	0.408509	0.369515	0.174943	0.289523	0.183909	0.075324	0.03291	0.034564
0.431704	1.86E-06	1.91E-05	0.049158	0.000962	0.038477	0.399975	0.713387	0.699659
0.062657	0.372185	0.345453	0.327144	0.541575	0.471797	0.210451	-0.0799	-0.13541
0.484044	1.64E-05	6.96E-05	0.000174	4.88E-11	2.15E-08	0.017555	0.371903	0.129028
0.100221	0.236085	0.277364	0.214056	0.366528	0.415031	0.138326	-0.03256	-0.02544
0.262247	0.007537	0.001594	0.01567	2.25E-05	1.22E-06	0.120923	0.716287	0.776447
0.170294	0.267324	0.293584	0.307978	0.43879	0.239422	0.314666	0.072509	-0.02372
0.055601	0.002379	0.000808	0.000428	2.47E-07	0.006708	0.000315	0.417867	0.791262
0.501457	0.093144	0.000766	0.478262	0.450175	0.308144	0.405839	0.534235	0.350513
1.91E-09	0.297614	0.99318	1.3E-08	1.1E-07	0.000424	2.2E-06	9.89E-11	5.35E-05
0.42913	0.775341	0.6736	0.926854	0.796384	0.590207	0.894868	0.836674	0.617537
4.8E-07	1.03E-26	4.05E-18	5.01E-55	4.47E-29	2.83E-13	1.28E-45	1.76E-34	1.05E-14
0.288628	0.597142	0.554495	0.653044	0.676813	0.551614	0.698669	0.514754	0.354632
0.000999	1.26E-13	1.35E-11	8.77E-17	2.45E-18	1.8E-11	6.71E-20	5.97E-10	4.3E-05
-0.00607	-0.30267	-0.34819	-0.15984	-0.26851	-0.26497	-0.1849	0.093429	-0.07686
0.945991	0.000543	6.04E-05	0.072637	0.002271	0.002608	0.037429	0.296129	0.390384
0.212386	-0.48574	-0.44237	-0.27621	-0.32049	-0.25731	-0.20522	0.167547	-0.0265
0.01652	7.1E-09	1.92E-07	0.001671	0.000239	0.003496	0.020641	0.059724	0.767434
0.128898	-0.1222	-0.1542	-0.122	-0.17276	-0.27871	-0.08477	0.44351	0.260449
0.148662	0.171097	0.083471	0.171814	0.052109	0.001509	0.343349	1.77E-07	0.003103
0.380302	0.161458	0.11473	0.388118	0.206131	0.109109	0.431911	0.80398	0.48998
1.03E-05	0.06976	0.199001	6.52E-06	0.02007	0.222053	3.97E-07	5.35E-30	5.01E-09
-0.30993	0.153031	0.209175	-0.05244	-0.26055	-0.08563	0.077707	0.005669	0.037659
0.000391	0.085861	0.018268	0.558198	0.003091	0.338471	0.385191	0.949564	0.674231
-0.27521	-0.58444	-0.51228	-0.77953	-0.83013	-0.59091	-0.64458	-0.48874	-0.35327
0.001739	5.45E-13	7.44E-10	3.65E-27	1.66E-33	2.61E-13	2.91E-16	5.55E-09	4.62E-05
-0.4824	-0.33738	-0.2869	-0.5659	-0.47349	-0.33159	-0.53792	-0.83473	-0.45962
9.3E-09	0.000105	0.001074	4.12E-12	1.89E-08	0.00014	6.95E-11	3.46E-34	5.46E-08
-0.45051	-0.34013	-0.29284	-0.58385	-0.65549	-0.53448	-0.51161	-0.63237	-0.234
1.07E-07	9.14E-05	0.000835	5.83E-13	6.16E-17	9.66E-11	7.9E-10	1.53E-15	0.008102
-0.13854	-0.41562	-0.35977	-0.37833	-0.39048	-0.14237	-0.44095	-0.32485	-0.3139
0.120336	1.18E-06	3.26E-05	1.16E-05	5.67E-06	0.110336	2.12E-07	0.000194	0.000326

FCRL3	FCRL5	FCRL6	FERMT3	FES	FFAR4	FGD1	FGD2	FGD3
brown	brown	brown	brown	brown	brown	brown	brown	brown
0.247934	0.341644	0.317727	0.18663	0.119948	0.354223	-0.38363	0.155759	0.001891
0.004947	8.46E-05	0.000273	0.035648	0.179196	4.39E-05	8.5E-06	0.080351	0.98317
0.105614	0.225581	0.186564	0.036282	0.025888	0.27143	-0.34305	0.029746	-0.12414
0.237304	0.010772	0.035715	0.685504	0.772653	0.002023	7.87E-05	0.739905	0.16435
-0.22379	-0.31214	-0.22741	-0.26411	-0.37312	-0.436	0.564054	-0.34378	-0.2123
0.011431	0.000353	0.010133	0.002696	1.56E-05	3E-07	5.01E-12	7.58E-05	0.016565
-0.11826	-0.1263	-0.00602	-0.17958	-0.21214	-0.32519	0.063054	-0.25353	-0.29027
0.185454	0.157082	0.946453	0.043356	0.016649	0.000191	0.481275	0.004028	0.000932
0.019686	0.060369	0.065402	0.09639	0.105544	0.006386	-0.20611	-0.14628	0.001812
0.826122	0.500177	0.465064	0.281017	0.237616	0.943196	0.020085	0.100798	0.983869
0.010106	0.054961	0.149791	0.000736	0.017726	-0.11871	-0.30928	-0.19276	-0.20463
0.910218	0.5394	0.092788	0.993449	0.843202	0.183741	0.000403	0.029912	0.021012
0.114218	0.063666	0.190847	-0.0261	0.063073	-0.18978	-0.22283	-0.23048	-0.21634
0.201025	0.477016	0.031613	0.770828	0.48114	0.032594	0.011799	0.009137	0.01457
0.109663	0.034108	0.117962	0.087408	0.178695	0.088021	-0.47837	0.104145	0.161544
0.219699	0.703435	0.186555	0.328484	0.044422	0.325088	1.28E-08	0.243929	0.06961
0.211442	0.314887	0.324457	0.443067	0.485129	-0.11325	0.037544	0.235624	0.225368
0.017018	0.000311	0.000198	1.82E-07	7.46E-09	0.20491	0.675165	0.007659	0.010849
0.551652	0.683639	0.59002	0.897315	0.788293	0.462985	-0.67261	0.765462	0.685366
1.79E-11	8.24E-19	2.89E-13	3.18E-46	3.89E-28	4.24E-08	4.73E-18	1.08E-25	6.23E-19
0.461581	0.517231	0.487455	0.603878	0.457032	0.322021	-0.57585	0.506722	0.358132
4.71E-08	4.78E-10	6.17E-09	5.67E-14	6.62E-08	0.000223	1.41E-12	1.21E-09	3.56E-05
-0.07691	-0.02501	-0.04715	-0.00997	-0.0938	-0.13054	0.178515	0.006756	-0.02641
0.390115	0.78014	0.598651	0.911403	0.294216	0.143512	0.04464	0.939905	0.768203
-0.07317	-0.1029	-0.15941	0.010114	-0.10167	-0.24003	0.555339	0.086068	0.178336
0.413621	0.249624	0.073437	0.910147	0.25537	0.006566	1.23E-11	0.335981	0.044858
-0.02425	0.032032	-0.11682	0.331388	0.25242	-0.0305	0.288343	0.224196	0.41872
0.786683	0.720716	0.190872	0.000141	0.004196	0.733551	0.001011	0.011279	9.63E-07
0.302109	0.402997	0.247939	0.701099	0.577193	0.201973	-0.06516	0.669567	0.720391
0.000557	2.63E-06	0.004946	4.41E-20	1.22E-12	0.022778	0.466697	7.55E-18	1.34E-21
0.020998	0.066756	0.011682	0.054969	-0.02222	0.434684	-0.29852	0.299531	0.177617
0.814739	0.455853	0.896291	0.53934	0.804144	3.28E-07	0.000652	0.000624	0.045742
-0.35619	-0.50844	-0.50255	-0.52497	-0.63581	-0.15391	0.624043	-0.33776	-0.2611
3.96E-05	1.04E-09	1.74E-09	2.36E-10	9.68E-16	0.084053	4.58E-15	0.000103	0.003027
-0.33798	-0.44788	-0.32753	-0.74253	-0.60822	-0.09258	0.154743	-0.56639	-0.68483
0.000102	1.29E-07	0.000171	1.67E-23	3.34E-14	0.300541	0.082369	3.91E-12	6.79E-19
-0.23015	-0.36125	-0.33143	-0.57662	-0.44542	0.05106	0.192812	-0.37027	-0.42197
0.00924	3.01E-05	0.000141	1.3E-12	1.54E-07	0.568613	0.029869	1.83E-05	7.77E-07
-0.16304	-0.18858	-0.23092	-0.35909	-0.46527	-0.3501	0.642039	-0.33894	-0.22176
0.067034	0.033734	0.009002	3.38E-05	3.56E-08	5.46E-05	4.14E-16	9.7E-05	0.012221

FGF20	FGL2	FGR	FIGNL2	FKBP15	FKBP4	FLI1	FLRT3	FMNL1
brown	brown	brown	brown	brown	brown	brown	brown	brown
0.153714	0.304408	0.084155	-0.19346	0.25477	-0.11353	0.181366	-0.03452	0.299381
0.084454	0.000502	0.346875	0.029309	0.003845	0.203786	0.041286	0.700008	0.000628
0.133692	0.131487	0.059274	-0.14978	0.13096	0.025392	0.011959	0.008466	0.174061
0.13401	0.140607	0.507993	0.092809	0.142219	0.77689	0.893846	0.924738	0.05033
-0.30786	-0.3309	-0.22199	0.452745	-0.04084	0.007511	-0.27559	0.290561	-0.68198
0.00043	0.000145	0.01213	9.08E-08	0.648468	0.933206	0.001713	0.00092	1.08E-18
-0.1076	0.021	-0.07634	0.244955	0.12089	-0.22531	-0.02867	0.131447	-0.5952
0.228561	0.814722	0.39362	0.00551	0.175779	0.010872	0.748956	0.140728	1.59E-13
0.132112	0.138695	0.12034	-0.08239	-0.24401	0.142803	0.013904	0.068471	-0.26425
0.138712	0.119927	0.177768	0.357116	0.005699	0.109241	0.876706	0.444335	0.002682
0.116469	0.311056	0.094139	-0.00102	0.032448	-0.20353	0.132296	0.064899	-0.48757
0.192233	0.000372	0.292461	0.990893	0.717241	0.021729	0.138159	0.468511	6.11E-09
0.020338	0.061467	0.16641	-0.02612	0.140988	0.016075	0.073368	-0.02342	-0.32898
0.820458	0.4924	0.061503	0.770657	0.113865	0.857643	0.41236	0.7938	0.000159
0.13251	0.316925	-0.00447	-0.44602	0.160165	-0.32101	0.179461	-0.1722	0.081775
0.137516	0.000283	0.960255	1.48E-07	0.072057	0.000234	0.043503	0.052885	0.360731
-0.21332	0.171323	0.461194	0.047929	0.464578	-0.38425	0.41927	-0.28774	0.065006
0.01604	0.054118	4.85E-08	0.592578	3.75E-08	8.2E-06	9.29E-07	0.001037	0.467776
0.394465	0.733061	0.737789	-0.56938	0.68011	-0.63693	0.769155	-0.56677	0.562747
4.46E-06	1.15E-22	4.44E-23	2.85E-12	1.45E-18	8.32E-16	4.54E-26	3.76E-12	5.74E-12
0.318145	0.694434	0.337064	-0.32453	0.611225	-0.53291	0.562427	-0.42905	0.343454
0.000267	1.38E-19	0.000107	0.000197	2.31E-14	1.12E-10	5.94E-12	4.83E-07	7.71E-05
-0.182	-0.39935	-0.06447	0.330326	0.089627	0.292242	-0.17287	-0.17765	0.148247
0.04057	3.3E-06	0.471479	0.000149	0.316305	0.000856	0.051953	0.045696	0.096243
-0.32588	-0.47164	-0.03255	0.236783	0.20793	0.022768	-0.16383	-0.04626	0.003074
0.000185	2.18E-08	0.716418	0.007357	0.018987	0.799432	0.065695	0.605573	0.972642
-0.12632	-0.21354	0.157401	0.005219	-0.06479	0.10908	-0.05778	-0.16875	0.175322
0.157021	0.015928	0.077176	0.953562	0.469278	0.222177	0.518735	0.057894	0.048662
0.050806	0.126946	0.516033	-0.30297	0.536558	-0.34076	0.423626	-0.53787	0.537858
0.570541	0.154961	5.32E-10	0.000536	7.92E-11	8.85E-05	6.96E-07	6.99E-11	6.99E-11
0.376429	0.005021	-0.02334	-0.32325	-0.00358	0.125117	0.108288	-0.1966	0.377159
1.29E-05	0.955319	0.794544	0.00021	0.968154	0.161043	0.225574	0.026739	1.24E-05
-0.16229	-0.61489	-0.55705	0.311909	-0.43809	0.504726	-0.54582	0.3623	-0.18365
0.068315	1.47E-14	1.04E-11	0.000357	2.59E-07	1.44E-09	3.22E-11	2.84E-05	0.03875
-0.03075	-0.13942	-0.60905	0.320938	-0.61418	0.359215	-0.45398	0.545438	-0.3208
0.73143	0.117994	3.02E-14	0.000234	1.6E-14	3.36E-05	8.29E-08	3.34E-11	0.000236
-0.04484	-0.27232	-0.50819	0.142279	-0.56922	0.514291	-0.44246	0.405404	0.097996
0.616708	0.001953	1.07E-09	0.110562	2.9E-12	6.22E-10	1.9E-07	2.27E-06	0.273043
-0.32785	-0.58735	-0.1785	0.484968	-0.26209	0.282405	-0.33158	0.476508	-0.43901
0.000168	3.92E-13	0.044654	7.56E-09	0.002914	0.001296	0.00014	1.49E-08	2.43E-07

FOLR2	FOXG1	FPR1	FPR2	FPR3	FRAS1	FRZB	FTL	FUCA1
brown	brown	brown	brown	brown	brown	brown	brown	brown
0.514641	-0.1691	0.230689	0.050958	0.375686	-0.06623	0.34647	0.387075	0.464673
6.03E-10	0.057371	0.009072	0.569385	1.35E-05	0.459449	6.6E-05	6.94E-06	3.73E-08
0.40696	-0.10551	0.141209	-0.02862	0.200263	-0.03704	0.188691	0.323755	0.373163
2.05E-06	0.237758	0.113294	0.749448	0.023979	0.679273	0.033625	0.000205	1.55E-05
-0.46521	0.349694	-0.31702	-0.13012	-0.13915	0.149814	-0.30847	-0.42962	-0.43472
3.58E-08	5.58E-05	0.000282	0.144805	0.118713	0.092739	0.000418	4.65E-07	3.27E-07
-0.33864	0.238526	-0.12203	0.076303	0.093081	-0.16973	-0.03273	-0.26943	-0.09668
9.85E-05	0.006922	0.171699	0.393861	0.297941	0.056433	0.714899	0.00219	0.279587
0.241147	-0.14118	0.202964	0.079845	0.128506	-0.34154	0.058615	0.250852	0.219443
0.006311	0.113364	0.022106	0.372211	0.14991	8.5E-05	0.512729	0.004446	0.013181
0.011343	0.005782	0.172323	0.215183	0.291403	-0.47691	0.157936	0.041336	0.251302
0.89928	0.948563	0.052708	0.015118	0.000888	1.44E-08	0.076163	0.64449	0.004373
0.026169	-0.12127	0.128474	0.113063	0.15404	-0.28472	0.088686	0.14316	0.202728
0.770249	0.174405	0.150014	0.20565	0.083788	0.001177	0.321433	0.108347	0.022264
0.054239	-0.27799	0.217615	0.055074	0.229253	-0.34394	0.39533	0.210566	0.275957
0.544752	0.001554	0.013984	0.538568	0.009525	7.52E-05	4.23E-06	0.017492	0.001688
0.158933	-0.19166	0.262566	0.487998	0.466431	-0.2047	0.340773	0.188556	0.277285
0.074304	0.030886	0.002862	5.9E-09	3.26E-08	0.02097	8.84E-05	0.033755	0.001599
0.776336	-0.55712	0.744742	0.632472	0.850331	-0.66204	0.607789	0.804271	0.859609
8.04E-27	1.03E-11	1.05E-23	1.51E-15	1.18E-36	2.35E-17	3.53E-14	4.93E-30	2.92E-38
0.459669	-0.25434	0.536902	0.41561	0.679734	-0.5507	0.457682	0.465456	0.612608
5.44E-08	0.003908	7.67E-11	1.18E-06	1.54E-18	1.97E-11	6.31E-08	3.51E-08	1.95E-14
-0.06967	-0.00147	-0.20206	-0.09127	-0.21509	0.289519	-0.17032	-0.12697	-0.17647
0.436352	0.986931	0.022715	0.307457	0.015164	0.000962	0.055567	0.154871	0.047182
-0.24457	0.152942	-0.29599	-0.14881	-0.15977	0.356137	-0.23314	-0.15773	-0.35144
0.005586	0.086045	0.000728	0.09497	0.072775	3.97E-05	0.008345	0.07655	5.09E-05
0.046478	0.02989	-0.05603	-0.039	-0.07442	0.060206	-0.14264	0.169425	-0.16046
0.603845	0.738687	0.531544	0.663362	0.405674	0.501335	0.109652	0.05688	0.071536
0.400228	-0.28841	0.269824	0.259904	0.399056	-0.18054	0.260157	0.519961	0.320889
3.13E-06	0.001008	0.002156	0.003169	3.36E-06	0.042235	0.003138	3.73E-10	0.000235
0.302585	-0.13211	-6.3E-05	-0.25247	-0.05101	0.05576	0.078135	0.30638	0.155714
0.000545	0.138718	0.99944	0.004188	0.568969	0.533512	0.38257	0.00046	0.080439
-0.44734	0.379457	-0.51658	-0.55436	-0.68453	0.669384	-0.53153	-0.48045	-0.71432
1.34E-07	1.08E-05	5.07E-10	1.36E-11	7.13E-19	7.77E-18	1.28E-10	1.09E-08	4.15E-21
-0.4768	0.398493	-0.37627	-0.35369	-0.55543	0.380817	-0.33074	-0.62583	-0.4619
1.45E-08	3.48E-06	1.3E-05	4.52E-05	1.22E-11	1E-05	0.000146	3.63E-15	4.6E-08
-0.23714	0.152467	-0.27904	-0.38973	-0.60804	0.536118	-0.32481	-0.39365	-0.40839
0.007266	0.087037	0.001489	5.92E-06	3.42E-14	8.27E-11	0.000195	4.68E-06	1.88E-06
-0.22124	0.167937	-0.37498	-0.22725	-0.24258	0.499445	-0.30091	-0.31272	-0.41334
0.012432	0.059125	1.4E-05	0.01019	0.005999	2.27E-09	0.000587	0.000344	1.37E-06

FUOM	FUT10	FXYD5	GAB3	GALC	GALM	GAPT	GATA3	GBA
brown	brown	brown	brown	brown	brown	brown	brown	brown
0.114083	-0.168	0.353874	0.064709	0.372016	0.265114	0.201092	0.193477	0.381801
0.201563	0.059024	4.48E-05	0.469815	1.66E-05	0.002593	0.02339	0.029298	9.46E-06
0.011909	-0.04118	0.24517	-0.02385	0.299668	0.156334	0.028485	0.067128	0.261472
0.894282	0.645711	0.005467	0.790092	0.00062	0.079227	0.75056	0.453337	0.002984
-0.13746	0.199543	-0.31725	0.038831	-0.33121	-0.11733	-0.0426	-0.17418	-0.37305
0.123298	0.024502	0.000279	0.664693	0.000143	0.188941	0.634366	0.050175	1.56E-05
-0.12552	0.389845	-0.1853	0.356716	0.161558	0.21131	0.308968	-0.01422	-0.28775
0.159683	5.89E-06	0.037006	3.85E-05	0.069584	0.017089	0.000409	0.873882	0.001037
0.477558	-0.01763	0.261316	0.07224	0.052824	0.178535	0.149596	0.099576	0.071416
1.37E-08	0.844024	0.003002	0.419603	0.555311	0.044616	0.09322	0.265346	0.424941
0.226288	0.3015	0.121902	0.410198	0.38071	0.43244	0.486813	0.145497	-0.06041
0.010522	0.000572	0.172166	1.67E-06	1.01E-05	3.83E-07	6.5E-09	0.102648	0.499868
0.10716	0.206386	0.180669	0.37631	0.270311	0.225932	0.275178	0.135948	-0.14007
0.230469	0.019913	0.042086	1.3E-05	0.002115	0.010647	0.001741	0.127511	0.116258
-0.01734	0.092863	0.14174	0.170622	0.243157	0.282374	0.469365	0.084719	0.330562
0.846561	0.299077	0.111931	0.055125	0.005876	0.001298	2.6E-08	0.34364	0.000147
0.206464	-0.21839	0.390488	0.366203	0.122002	0.448467	0.404502	0.429024	0.247717
0.019866	0.01364	5.66E-06	2.29E-05	0.171812	1.24E-07	2.4E-06	4.84E-07	0.004986
0.613997	-0.43072	0.878191	0.635984	0.57836	0.799475	0.779033	0.697508	0.658941
1.64E-14	4.31E-07	7.44E-42	9.45E-16	1.07E-12	1.91E-29	4.13E-27	8.19E-20	3.72E-17
0.408143	-0.27875	0.590028	0.559921	0.575848	0.707843	0.707155	0.548312	0.42802
1.91E-06	0.001506	2.89E-13	7.71E-12	1.41E-12	1.34E-20	1.52E-20	2.51E-11	5.18E-07
-0.2044	-0.08424	-0.05889	-0.0547	-0.30641	-0.21111	-0.28548	-0.01493	-0.0427
0.02116	0.346409	0.510717	0.541311	0.000459	0.017196	0.001141	0.867651	0.633636
-0.2246	-0.14595	-0.14017	-0.12627	-0.45124	-0.25025	-0.28046	-0.07247	-0.08617
0.011131	0.101558	0.116006	0.157174	1.01E-07	0.004546	0.001405	0.418107	0.335416
0.364037	-0.3923	0.130197	-0.23981	-0.37305	-0.12898	-0.21481	0.165871	0.155961
2.59E-05	5.08E-06	0.144577	0.006617	1.56E-05	0.148394	0.015296	0.062361	0.079955
0.354504	-0.52744	0.544256	0.203077	-0.00414	0.294917	0.223776	0.460028	0.470229
4.33E-05	1.88E-10	3.75E-11	0.02203	0.963126	0.000763	0.011437	5.29E-08	2.43E-08
-0.06814	-0.23584	0.10527	-0.09908	0.079757	-0.221	-0.17782	-0.13375	0.074687
0.446555	0.007602	0.238846	0.267742	0.372744	0.01253	0.045489	0.133849	0.403987
-0.43557	0.078468	-0.60274	-0.53748	-0.57984	-0.81087	-0.74714	-0.57517	-0.51472
3.09E-07	0.380543	6.5E-14	7.25E-11	9.11E-13	7.19E-31	6.35E-24	1.52E-12	5.99E-10
-0.46784	0.453482	-0.70229	-0.36497	-0.04807	-0.47716	-0.40933	-0.51497	-0.51905
2.92E-08	8.6E-08	3.58E-20	2.46E-05	0.591528	1.41E-08	1.77E-06	5.85E-10	4.06E-10
-0.44004	0.144199	-0.52774	-0.58345	-0.2678	-0.63564	-0.63397	-0.51519	-0.29031
2.26E-07	0.105784	1.82E-10	6.09E-13	0.002335	9.9E-16	1.24E-15	5.74E-10	0.00093
-0.25142	0.066884	-0.28395	-0.14636	-0.51158	-0.43472	-0.41739	-0.37229	-0.40253
0.004354	0.454984	0.001215	0.100611	7.92E-10	3.27E-07	1.05E-06	1.63E-05	2.71E-06

GBGT1	GBP1	GBP2	GBP3	GBP5	GCNT1	GCSAM	GEM	GFPT2
brown	brown	brown	brown	brown	brown	brown	brown	brown
0.122724	0.203795	0.230822	0.28688	0.420296	0.385509	0.248328	0.049928	0.081356
0.16927	0.021555	0.009031	0.001075	8.68E-07	7.61E-06	0.004877	0.577223	0.363201
0.001703	0.051935	0.103879	0.194992	0.227788	0.266115	0.153635	-0.04421	0.018077
0.984841	0.561992	0.245143	0.028031	0.010006	0.002494	0.084615	0.621614	0.840136
-0.28932	0.012414	-0.06096	-0.01896	-0.08579	-0.34426	-0.14646	-0.0026	-0.26734
0.00097	0.88983	0.495968	0.832431	0.337561	7.4E-05	0.100372	0.976873	0.002378
-0.16931	0.399403	0.210683	0.327148	0.276871	0.053893	0.045238	0.23367	0.035556
0.057057	3.29E-06	0.017428	0.000174	0.001626	0.547322	0.613539	0.008193	0.691475
0.104483	0.070129	0.01608	0.254213	0.199961	0.059125	0.205639	0.021855	-0.08844
0.242392	0.433356	0.857599	0.003926	0.024197	0.509066	0.020375	0.80732	0.322779
0.020684	0.500029	0.27562	0.569994	0.491644	0.301285	0.27053	0.297774	0.106857
0.817458	2.16E-09	0.001711	2.67E-12	4.37E-09	0.000577	0.002097	0.000674	0.231799
0.029554	0.31578	0.201271	0.313159	0.320249	0.192467	0.132049	0.120869	0.113326
0.741523	0.000299	0.023265	0.000337	0.000242	0.03017	0.138902	0.175856	0.20459
0.239134	0.383787	0.223253	0.393612	0.32023	0.272277	0.225196	0.318208	0.251581
0.006776	8.43E-06	0.011636	4.69E-06	0.000242	0.001956	0.010911	0.000267	0.004328
0.437409	0.46002	0.545585	0.359585	0.469053	0.411033	0.083782	0.416116	0.402903
2.72E-07	5.3E-08	3.29E-11	3.3E-05	2.66E-08	1.59E-06	0.34902	1.14E-06	2.65E-06
0.876201	0.683641	0.796505	0.675618	0.805229	0.701963	0.582372	0.475765	0.50725
1.92E-41	8.24E-19	4.32E-29	2.96E-18	3.74E-30	3.79E-20	6.87E-13	1.58E-08	1.16E-09
0.522636	0.838121	0.669879	0.730155	0.818611	0.600525	0.453931	0.409429	0.380562
2.92E-10	1.06E-34	7.2E-18	2.05E-22	6.83E-32	8.47E-14	8.32E-08	1.76E-06	1.02E-05
-0.0302	-0.19735	-0.02606	-0.2899	-0.20769	-0.07673	-0.33304	-0.05503	0.165641
0.736109	0.026149	0.771163	0.000946	0.01913	0.391184	0.00013	0.538906	0.062731
-0.00332	-0.23041	-0.01407	-0.39424	-0.30244	-0.36616	-0.21248	-0.09216	-0.08543
0.970434	0.009158	0.875227	4.52E-06	0.000548	2.3E-05	0.016469	0.302768	0.339599
0.260335	-0.21973	-0.03764	-0.33142	-0.24876	-0.32971	-0.10533	-0.09021	-0.13025
0.003117	0.01306	0.674359	0.000141	0.0048	0.000154	0.238576	0.313168	0.144424
0.667226	0.170688	0.452531	0.03971	0.191964	0.129393	0.206378	0.196313	0.24779
1.08E-17	0.05503	9.22E-08	0.657576	0.030612	0.147094	0.019918	0.026964	0.004973
0.028978	-0.3549	-0.19074	-0.23252	-0.26651	-0.17755	0.050265	-0.37599	-0.19717
0.746385	4.24E-05	0.031715	0.008523	0.002456	0.045826	0.574653	1.32E-05	0.026294
-0.57494	-0.70985	-0.65297	-0.70773	-0.80865	-0.78641	-0.50373	-0.53222	-0.56138
1.56E-12	9.37E-21	8.86E-17	1.37E-20	1.39E-30	6.34E-28	1.57E-09	1.2E-10	6.62E-12
-0.75354	-0.35737	-0.62338	-0.2808	-0.41095	-0.2613	-0.32913	-0.35595	-0.35647
1.59E-24	3.71E-05	4.99E-15	0.001385	1.59E-06	0.003004	0.000158	4.01E-05	3.9E-05
-0.56964	-0.62089	-0.68222	-0.50902	-0.60547	-0.3166	-0.37999	-0.4762	-0.29763
2.77E-12	6.87E-15	1.04E-18	9.92E-10	4.68E-14	0.000287	1.05E-05	1.53E-08	0.000678
-0.36189	-0.52084	-0.35247	-0.4006	-0.34349	-0.43912	-0.17265	-0.34564	-0.43938
2.91E-05	3.45E-10	4.82E-05	3.06E-06	7.7E-05	2.41E-07	0.052251	6.89E-05	2.37E-07

GGA2	GIMAP1	GIMAP2	GIMAP4	GIMAP6	GIMAP7	GIMAP8	GIPC2	GLA
brown	brown	brown	brown	brown	brown	brown	brown	brown
0.457222	0.096586	0.117987	0.342784	0.265039	0.275802	0.160747	0.299088	0.248204
6.53E-08	0.280036	0.18646	7.98E-05	0.002601	0.001698	0.071016	0.000636	0.004899
0.313969	-0.05124	-0.05405	0.18582	0.087144	0.112717	0.027954	0.240593	0.194975
0.000325	0.567246	0.546184	0.036471	0.329952	0.20705	0.755061	0.006436	0.028045
-0.25882	-0.314	-0.06401	-0.40094	-0.26642	-0.39947	-0.21426	-0.34792	0.14909
0.003302	0.000324	0.474662	2.99E-06	0.002465	3.28E-06	0.01557	6.12E-05	0.094344
-0.10984	-0.25313	0.316312	-0.11885	0.027367	-0.16106	0.039549	-0.09726	0.2862
0.218952	0.004088	0.000291	0.183232	0.760051	0.070457	0.658881	0.276664	0.001107
0.00745	-0.00088	0.338708	0.109361	0.067611	0.18068	-0.14616	0.283464	0.459049
0.933748	0.992155	9.82E-05	0.220981	0.450091	0.042074	0.101072	0.00124	5.7E-08
0.015762	-0.12698	0.601503	0.1568	0.222748	0.137	0.075976	0.272107	0.554525
0.860383	0.154864	7.54E-14	0.078326	0.011832	0.124564	0.395899	0.00197	1.34E-11
0.148155	-0.17543	0.316434	0.049406	0.121701	0.071605	0.077103	0.198883	0.430053
0.096453	0.048517	0.00029	0.581215	0.172877	0.423715	0.388908	0.024989	4.51E-07
0.133024	0.117646	0.315765	0.269942	0.191551	0.171877	0.186568	0.229067	0.146827
0.135983	0.187745	0.000299	0.002146	0.030978	0.053334	0.035711	0.009584	0.099511
0.311074	0.367741	0.350405	0.305152	0.397492	0.273822	0.483584	0.071324	0.449506
0.000371	2.11E-05	5.38E-05	0.000486	3.7E-06	0.001839	8.45E-09	0.425538	1.15E-07
0.635074	0.752648	0.747948	0.858543	0.721581	0.744959	0.714272	0.498604	0.585101
1.07E-15	1.93E-24	5.34E-24	4.52E-38	1.07E-21	1.01E-23	4.19E-21	2.44E-09	5.06E-13
0.49894	0.479292	0.688037	0.669986	0.640246	0.524812	0.534798	0.281132	0.37851
2.37E-09	1.19E-08	4.02E-19	7.08E-18	5.29E-16	2.39E-10	9.38E-11	0.001366	1.14E-05
-0.02952	0.019165	-0.30666	-0.18715	-0.2013	-0.27003	-0.03705	-0.34766	-0.18404
0.741805	0.830657	0.000454	0.035127	0.023245	0.002139	0.679228	6.21E-05	0.038335
-0.0153	0.05964	-0.44283	-0.29367	-0.28619	-0.30046	-0.0321	-0.48611	-0.22621
0.864452	0.505372	1.86E-07	0.000805	0.001107	0.000599	0.720135	6.89E-09	0.010548
-0.10575	0.354697	-0.20406	-0.08356	-0.13306	-0.02774	-0.12626	-0.23609	-0.15978
0.23672	4.28E-05	0.02138	0.350316	0.135865	0.756899	0.157224	0.007535	0.072753
0.388802	0.702455	0.092527	0.397274	0.273372	0.340275	0.436418	-0.03642	0.096506
6.26E-06	3.48E-20	0.300836	3.75E-06	0.001872	9.07E-05	2.91E-07	0.684337	0.280436
0.078924	0.182715	-0.23596	0.208499	0.026407	0.186786	0.087361	0.047778	-0.33462
0.377771	0.039775	0.007571	0.018655	0.768219	0.035492	0.328747	0.593748	0.00012
-0.45186	-0.4152	-0.74457	-0.61827	-0.58161	-0.55762	-0.53199	-0.52304	-0.6308
9.69E-08	1.21E-06	1.09E-23	9.59E-15	7.48E-13	9.78E-12	1.22E-10	2.82E-10	1.89E-15
-0.48611	-0.65878	-0.28861	-0.43963	-0.30026	-0.38132	-0.45891	-0.06294	-0.45065
6.89E-09	3.81E-17	0.001	2.33E-07	0.000604	9.73E-06	5.75E-08	0.482094	1.06E-07
-0.33689	-0.46215	-0.56696	-0.39295	-0.37193	-0.31526	-0.45879	-0.07116	-0.59019
0.000108	4.51E-08	3.68E-12	4.88E-06	1.67E-05	0.000306	5.81E-08	0.42662	2.84E-13
-0.00465	-0.31601	-0.39556	-0.43156	-0.40586	-0.30864	-0.24835	-0.26371	0.131697
0.958615	0.000295	4.17E-06	4.07E-07	2.2E-06	0.000415	0.004873	0.002738	0.139968

GLIPR1	GLMP	GLRX	GM2A	GMFG	GNA15	GNB1	GNGT2	GNLY
brown	brown	brown	brown	brown	brown	brown	brown	brown
0.245861	0.167601	0.303827	0.410757	0.210881	0.278769	-0.1311	0.307778	0.070763
0.005333	0.059641	0.000516	1.61E-06	0.01732	0.001505	0.141794	0.000431	0.4292
0.131889	0.058718	0.296952	0.312395	0.10549	0.16037	-0.09461	0.18984	-0.07442
0.139386	0.511993	0.000698	0.000349	0.237861	0.071689	0.290024	0.03254	0.405644
-0.16527	-0.09241	-0.34655	-0.4867	-0.29645	-0.32166	0.201163	-0.20503	-0.17024
0.063328	0.301452	6.57E-05	6.57E-09	0.000714	0.000226	0.023341	0.020759	0.055678
0.246679	0.002761	-0.00175	-0.22156	-0.10575	-0.17445	-0.14075	-0.13408	-0.15546
0.005177	0.975425	0.984412	0.012302	0.236713	0.049814	0.114476	0.132887	0.080934
0.13115	0.170048	0.507308	0.070531	0.528787	0.161393	-0.4923	0.337225	0.059231
0.141635	0.055961	1.15E-09	0.430718	1.65E-10	0.069875	4.14E-09	0.000106	0.508307
0.471689	0.170416	0.481383	0.037346	0.358085	0.082691	-0.5229	0.153965	-0.04421
2.17E-08	0.055423	1.01E-08	0.676784	3.57E-05	0.355357	2.86E-10	0.083941	0.6216
0.305247	0.15152	0.379043	0.072501	0.232769	0.061114	-0.29715	0.136614	-0.18556
0.000484	0.089037	1.11E-05	0.417924	0.008451	0.494892	0.000692	0.12564	0.03674
0.374318	0.061473	0.194871	0.230175	0.225246	0.1124	-0.37983	0.105607	-0.10198
1.46E-05	0.492358	0.02813	0.009232	0.010893	0.208335	1.06E-05	0.237336	0.253917
0.533864	0.529287	0.181899	0.08741	0.332791	0.397762	-0.07767	0.213285	0.199131
1.02E-10	1.58E-10	0.040684	0.328475	0.000132	3.64E-06	0.385436	0.016058	0.024805
0.743074	0.750744	0.660713	0.807243	0.846831	0.812619	-0.60081	0.7545	0.528168
1.49E-23	2.93E-24	2.87E-17	2.09E-30	4.45E-36	4.27E-31	8.18E-14	1.29E-24	1.75E-10
0.608912	0.506001	0.461679	0.603476	0.517579	0.500773	-0.45737	0.549148	0.411685
3.08E-14	1.29E-09	4.68E-08	5.95E-14	4.63E-10	2.03E-09	6.45E-08	2.31E-11	1.52E-06
-0.24749	0.101086	-0.25867	-0.14291	-0.25596	-0.06358	0.259799	-0.04228	0.040366
0.005028	0.258133	0.003321	0.108961	0.003678	0.477612	0.003181	0.636919	0.65229
-0.36812	0.002817	-0.60141	-0.20391	-0.41005	-0.14424	0.467396	-0.12052	-0.03358
2.06E-05	0.974925	7.62E-14	0.021482	1.69E-06	0.105674	3.03E-08	0.177101	0.707854
-0.30266	0.230934	-0.20735	0.022698	0.081373	0.150723	0.023526	0.174777	0.197219
0.000543	0.008997	0.019328	0.800042	0.363103	0.090752	0.792909	0.049377	0.026253
0.122279	0.542166	-0.02674	0.478712	0.325049	0.481839	-0.0791	0.451435	0.391264
0.170832	4.6E-11	0.765383	1.25E-08	0.000192	9.73E-09	0.376692	9.99E-08	5.41E-06
-0.29319	-0.17599	-0.0851	0.37153	-0.00751	-0.0431	0.064836	0.139628	0.103697
0.000822	0.047796	0.341479	1.7E-05	0.933193	0.630415	0.468944	0.11743	0.245975
-0.84956	-0.59807	-0.68732	-0.48653	-0.68097	-0.58748	0.650716	-0.45534	-0.24121
1.58E-36	1.13E-13	4.52E-19	6.66E-09	1.27E-18	3.86E-13	1.22E-16	7.5E-08	0.006297
-0.30527	-0.68042	-0.21041	-0.5034	-0.54743	-0.58394	0.296926	-0.61533	-0.34735
0.000483	1.38E-18	0.017575	1.62E-09	2.74E-11	5.76E-13	0.000699	1.39E-14	6.31E-05
-0.48819	-0.64087	-0.24437	-0.35678	-0.48452	-0.43087	0.466038	-0.47487	-0.24698
5.81E-09	4.86E-16	0.005627	3.83E-05	7.84E-09	4.27E-07	3.36E-08	1.69E-08	0.005122
-0.48206	-0.28492	-0.37667	-0.54513	-0.28958	-0.30202	0.402387	-0.12988	-0.18818
9.56E-09	0.001167	1.27E-05	3.44E-11	0.000959	0.000559	2.74E-06	0.14557	0.034121

GPBAR1	GPHN	GPLD1	GPNMB	GPR132	GPR141	GPR160	GPR171	GPR174
brown	brown	brown	brown	brown	brown	brown	brown	brown
0.164535	-0.1548	-0.05803	0.369422	0.286914	0.260015	0.149974	0.207183	0.409778
0.064531	0.082253	0.516972	1.92E-05	0.001074	0.003155	0.092387	0.01943	1.72E-06
0.076367	0.004387	0.039102	0.234919	0.155734	0.141414	0.014381	0.050549	0.201617
0.393465	0.960956	0.662498	0.007848	0.080401	0.112767	0.872506	0.572493	0.023024
0.046777	0.125309	-0.00512	-0.24238	-0.31898	-0.16592	0.063535	-0.06684	-0.06838
0.601513	0.160397	0.954483	0.006041	0.000257	0.062284	0.47793	0.455298	0.444957
-0.09971	-0.05521	-0.0081	-0.01111	-0.1621	0.156908	0.344679	0.195107	0.179567
0.264707	0.537529	0.927977	0.901357	0.068644	0.078119	7.24E-05	0.027936	0.043377
-0.09932	0.056802	-0.24324	-0.00495	0.00491	0.027024	0.115998	0.165621	0.019011
0.26658	0.525877	0.005858	0.95593	0.95631	0.762967	0.194048	0.062763	0.831993
-0.16744	-0.08142	-0.19078	0.111679	-0.01382	0.296986	0.486704	0.390408	0.288394
0.059884	0.362811	0.031676	0.211289	0.877407	0.000697	6.56E-09	5.69E-06	0.001009
-0.04576	-0.1378	0.033076	0.053652	-0.01036	0.22732	0.192717	0.143374	0.088935
0.609457	0.122345	0.712008	0.549119	0.907999	0.010165	0.029952	0.107817	0.320071
-0.16963	-0.30341	-0.10321	0.273686	0.190882	0.309792	0.095228	0.11894	0.168013
0.056573	0.000525	0.24819	0.001849	0.031581	0.000394	0.286885	0.182903	0.059008
0.48655	-0.57223	-0.30821	0.333046	0.314759	0.346343	0.337472	0.428536	0.478428
6.64E-09	2.1E-12	0.000423	0.00013	0.000313	6.64E-05	0.000104	5E-07	1.28E-08
0.455749	-0.70462	-0.64645	0.744149	0.751086	0.691931	0.583071	0.694603	0.61325
7.28E-08	2.38E-20	2.24E-16	1.19E-23	2.72E-24	2.11E-19	6.36E-13	1.34E-19	1.8E-14
0.345153	-0.52219	-0.38699	0.562087	0.513456	0.564752	0.503085	0.627958	0.66645
7.07E-05	3.05E-10	6.98E-06	6.15E-12	6.7E-10	4.65E-12	1.66E-09	2.75E-15	1.21E-17
0.139522	0.035344	0.267945	-0.16285	-0.05288	-0.18863	-0.37004	-0.26751	-0.05968
0.117712	0.693218	0.002322	0.067348	0.554916	0.033687	1.85E-05	0.002362	0.505092
0.302266	-0.07438	0.052825	-0.053	-0.04957	-0.2271	-0.31882	-0.25989	-0.15555
0.000553	0.405951	0.5553	0.554025	0.579933	0.010239	0.000259	0.003171	0.080771
0.325634	-0.08719	-0.28922	0.032381	0.129892	-0.23908	-0.25398	-0.13672	-0.22049
0.000187	0.329695	0.000974	0.717796	0.145528	0.006788	0.00396	0.125343	0.012741
0.552935	-0.5087	-0.46376	0.489414	0.50428	0.217778	0.02782	0.189823	0.208258
1.58E-11	1.02E-09	3.99E-08	5.25E-09	1.5E-09	0.013911	0.756203	0.032556	0.018795
-0.05249	0.252657	0.041536	0.139665	0.028554	-0.06984	-0.23545	-0.25273	-0.20603
0.557802	0.00416	0.642892	0.117333	0.749973	0.435246	0.007706	0.004149	0.020132
-0.17505	0.596433	0.396634	-0.54013	-0.49879	-0.60966	-0.56495	-0.70705	-0.65472
0.049012	1.37E-13	3.9E-06	5.61E-11	2.4E-09	2.81E-14	4.56E-12	1.55E-20	6.89E-17
-0.54086	0.582879	0.548437	-0.54171	-0.52887	-0.34286	-0.10448	-0.31583	-0.31648
5.23E-11	6.49E-13	2.48E-11	4.81E-11	1.64E-10	7.95E-05	0.242402	0.000298	0.000289
-0.38567	0.537644	0.567993	-0.52732	-0.39479	-0.43394	-0.44987	-0.50424	-0.46853
7.54E-06	7.14E-11	3.3E-12	1.9E-10	4.37E-06	3.46E-07	1.12E-07	1.5E-09	2.77E-08
0.030398	0.261985	0.105337	-0.3742	-0.33001	-0.43057	-0.18828	-0.30443	-0.19449
0.734414	0.002926	0.238542	1.46E-05	0.000151	4.36E-07	0.034019	0.000502	0.028444

GPR18	GPR183	GPR34	GPR45	GPR65	GPR84	GPRC5A	GPRIN3	GPSM3
brown	brown	brown	brown	brown	brown	brown	brown	brown
0.453298	0.042054	0.132279	-0.26549	0.164257	0.152982	0.237201	0.222137	0.218587
8.72E-08	0.638754	0.138209	0.002556	0.064989	0.085961	0.00725	0.012072	0.013552
0.244141	-0.06879	0.056618	-0.20884	-0.00585	0.029571	0.139167	0.045785	0.077679
0.005673	0.442179	0.527223	0.018461	0.947981	0.741379	0.118658	0.609258	0.385362
-0.1502	-0.20419	-0.34153	0.321602	0.039152	-0.00082	0.016057	-0.14163	-0.35105
0.091888	0.021297	8.51E-05	0.000227	0.662092	0.992657	0.857797	0.1122	5.2E-05
0.024128	0.109656	-0.07088	0.083421	0.33977	0.204098	0.276806	0.273081	-0.20765
0.787727	0.219729	0.428438	0.351107	9.3E-05	0.021357	0.001631	0.001894	0.019154
0.162175	0.121197	0.227259	-0.14889	0.173636	-0.0288	0.117879	-0.10416	0.252419
0.068512	0.174678	0.010185	0.094794	0.050904	0.747911	0.18687	0.243847	0.004196
0.227633	0.335205	0.238381	-0.13974	0.496585	0.233171	0.386991	0.350467	0.083312
0.010059	0.000117	0.006957	0.117127	2.89E-09	0.008335	6.98E-06	5.36E-05	0.351742
0.10221	0.139712	0.019247	-0.13081	0.243986	0.19358	0.265486	0.127998	0.086241
0.252848	0.117209	0.829937	0.142682	0.005705	0.02921	0.002556	0.151541	0.335008
0.182219	0.247966	0.354377	-0.30513	0.331434	0.185669	0.219604	0.383542	0.156515
0.040325	0.004941	4.36E-05	0.000486	0.000141	0.036627	0.013112	8.55E-06	0.078876
0.321746	0.236108	-0.06684	-0.25815	0.482381	0.533423	0.489645	0.198981	0.334792
0.000225	0.007531	0.455308	0.003386	9.32E-09	1.07E-10	5.15E-09	0.024915	0.000119
0.676953	0.550941	0.58339	-0.63966	0.831441	0.678779	0.546201	0.570198	0.908195
2.4E-18	1.93E-11	6.13E-13	5.73E-16	1.07E-33	1.8E-18	3.09E-11	2.61E-12	4.09E-49
0.643878	0.392085	0.364993	-0.45583	0.729288	0.622134	0.462549	0.588876	0.581888
3.21E-16	5.14E-06	2.45E-05	7.24E-08	2.43E-22	5.86E-15	4.38E-08	3.29E-13	7.25E-13
-0.141	-0.32201	-0.3926	0.077159	-0.271	-0.00616	-0.05756	-0.53375	-0.0308
0.113823	0.000223	4.99E-06	0.388565	0.002058	0.945183	0.520353	1.04E-10	0.731043
-0.16798	-0.40065	-0.44787	0.133754	-0.20665	0.008505	-0.13997	-0.32811	-0.12988
0.059058	3.05E-06	1.29E-07	0.13383	0.019755	0.924395	0.116517	0.000166	0.145556
-0.07299	-0.29815	-0.12685	-0.10542	-0.12472	-0.0455	-0.13946	-0.39836	0.223981
0.41481	0.000663	0.155287	0.238168	0.16238	0.611454	0.117871	3.51E-06	0.01136
0.296895	-0.00342	0.093239	-0.41076	0.306443	0.370006	0.166203	0.013851	0.599687
0.0007	0.969565	0.297118	1.61E-06	0.000458	1.86E-05	0.061832	0.877167	9.35E-14
-0.04763	-0.20295	0.286626	-0.03265	-0.23043	-0.31464	-0.34031	-0.15898	0.15245
0.594928	0.022117	0.001087	0.715521	0.009154	0.000315	9.05E-05	0.074226	0.087071
-0.56532	-0.54373	-0.38418	0.615939	-0.70912	-0.55694	-0.61521	-0.55861	-0.54751
4.38E-12	3.95E-11	8.23E-06	1.29E-14	1.07E-20	1.05E-11	1.41E-14	8.82E-12	2.72E-11
-0.42864	-0.08034	-0.09878	0.530552	-0.50747	-0.53174	-0.39505	-0.01878	-0.7137
4.97E-07	0.369267	0.269202	1.4E-10	1.14E-09	1.25E-10	4.3E-06	0.834016	4.66E-21
-0.44438	-0.21739	-0.1526	0.439638	-0.73031	-0.56736	-0.53307	-0.29362	-0.52563
1.66E-07	0.014086	0.086754	2.32E-07	1.98E-22	3.53E-12	1.11E-10	0.000807	2.22E-10
-0.18453	-0.34257	-0.45254	0.399111	-0.30239	-0.29652	-0.2731	-0.47193	-0.30586
0.037819	8.07E-05	9.22E-08	3.35E-06	0.00055	0.000712	0.001893	2.13E-08	0.00047

GRAP2	GRB2	GRHL1	GRN	GSAP	GSDMA	GSDMD	GSK3B	GSTA4
brown	brown	brown	brown	brown	brown	brown	brown	brown
0.255647	0.314642	-0.13891	0.172985	0.149178	0.120171	-0.02695	-0.19853	-0.0309
0.003721	0.000315	0.119355	0.051792	0.094149	0.178385	0.76358	0.025249	0.730158
0.098755	0.242226	-0.09192	0.020901	0.093046	0.067315	-0.17884	-0.19356	0.086863
0.269329	0.006074	0.304039	0.815574	0.298126	0.452081	0.044248	0.02923	0.331523
-0.09923	-0.52087	0.229969	-0.15354	-0.19795	0.046167	-0.09694	0.481549	0.197067
0.267032	3.44E-10	0.009296	0.084806	0.02569	0.606266	0.278287	9.96E-09	0.026371
0.094127	-0.53427	0.102938	-0.23557	0.323897	0.080695	0.085305	0.307742	0.017822
0.292519	9.86E-11	0.249467	0.007674	0.000203	0.367128	0.340298	0.000432	0.842362
0.13796	-0.08107	-0.07225	-0.12708	0.064533	0.105919	0.137768	-0.50547	0.388529
0.121921	0.364878	0.419561	0.154524	0.471024	0.235945	0.122445	1.35E-09	6.37E-06
0.247069	-0.39169	-0.0063	-0.23763	0.485274	0.180756	0.22966	-0.14471	0.163664
0.005105	5.27E-06	0.943952	0.007142	7.37E-09	0.041986	0.009394	0.104537	0.065977
0.162757	-0.11757	0.033365	-0.17036	0.401168	0.025949	0.07057	-0.09437	0.115252
0.067511	0.18804	0.709605	0.055501	2.95E-06	0.772136	0.430459	0.291258	0.19695
0.107928	-0.1068	-0.16158	-0.04296	0.329494	-0.01427	0.2752	-0.27873	-0.01061
0.227129	0.232065	0.069549	0.631528	0.000155	0.873509	0.00174	0.001508	0.905746
0.473004	0.030446	-0.27561	0.552271	0.359831	0.365216	0.458299	0.037753	-0.5576
1.96E-08	0.734008	0.001711	1.69E-11	3.25E-05	2.42E-05	6.02E-08	0.673463	9.79E-12
0.731204	0.416431	-0.52867	0.769402	0.622077	0.553581	0.725258	-0.58802	-0.60215
1.66E-22	1.12E-06	1.67E-10	4.28E-26	5.9E-15	1.48E-11	5.31E-22	3.63E-13	6.98E-14
0.571798	0.231806	-0.25806	0.484185	0.59726	0.268065	0.64169	-0.32803	-0.44546
2.19E-12	0.008734	0.003398	8.05E-09	1.25E-13	0.002311	4.34E-16	0.000167	1.54E-07
0.00581	0.109621	0.01235	0.181125	-0.078	-0.12604	-0.07796	0.032107	-0.12657
0.948314	0.219878	0.89039	0.041562	0.383419	0.157957	0.383671	0.72009	0.156196
-0.08054	-0.00521	-0.00742	0.313653	-0.38096	-0.02975	-0.08802	0.356674	-0.26916
0.36805	0.953673	0.933987	0.00033	9.94E-06	0.739863	0.325079	3.86E-05	0.002213
-0.04506	0.185639	-0.18835	0.45888	-0.41818	0.064629	0.240961	-0.29393	-0.23045
0.614956	0.036657	0.033951	5.77E-08	9.97E-07	0.470364	0.006353	0.000796	0.009147
0.380948	0.391122	-0.44018	0.859427	0.008563	0.333844	0.480657	-0.313	-0.65795
9.94E-06	5.45E-06	2.24E-07	3.14E-38	0.923878	0.000125	1.07E-08	0.00034	4.3E-17
-0.08695	0.367457	-0.15598	0.063281	-0.1693	0.008618	-0.23811	-0.23025	0.111996
0.331063	2.14E-05	0.079919	0.479695	0.05706	0.92339	0.007023	0.009209	0.209987
-0.63026	-0.03413	0.348028	-0.36676	-0.73933	-0.37077	-0.62092	0.473964	0.468933
2.03E-15	0.703258	6.09E-05	2.22E-05	3.24E-23	1.78E-05	6.84E-15	1.82E-08	2.69E-08
-0.54245	-0.25899	0.469683	-0.81562	-0.17617	-0.42632	-0.54864	0.444788	0.541376
4.48E-11	0.00328	2.54E-08	1.72E-31	0.047572	5.81E-07	2.43E-11	1.61E-07	4.97E-11
-0.58915	0.144349	0.375543	-0.56951	-0.41479	-0.46671	-0.61668	0.251928	0.491042
3.19E-13	0.105418	1.36E-05	2.81E-12	1.24E-06	3.19E-08	1.17E-14	0.004273	4.59E-09
-0.07709	-0.27983	0.196554	-0.20858	-0.54883	-0.0294	-0.50635	0.471428	0.374001
0.388974	0.001441	0.026774	0.018611	2.38E-11	0.742813	1.25E-09	2.22E-08	1.48E-05

GSTK1	GULP1	GYPC	GZMA	GZMB	GZMH	GZMK	H2AFJ	HAAO
brown	brown	brown	brown	brown	brown	brown	brown	brown
0.053013	-0.24127	0.33129	0.422301	0.326616	0.372229	0.413998	0.11451	0.176008
0.553896	0.006285	0.000142	7.6E-07	0.000178	1.64E-05	1.31E-06	0.199868	0.047774
-0.01956	-0.22251	0.235692	0.238937	0.225935	0.213131	0.236896	0.115537	0.105045
0.827259	0.011925	0.007641	0.006823	0.010646	0.016136	0.007328	0.19584	0.239856
0.02203	0.180083	-0.38039	-0.17005	-0.11424	-0.20413	-0.22062	-0.31384	-0.29418
0.805807	0.042768	1.03E-05	0.05596	0.200933	0.021336	0.012684	0.000327	0.000788
0.289404	0.153002	-0.31808	0.092153	0.063399	0.000652	0.019526	-0.28193	-0.39809
0.000967	0.085919	0.000268	0.302803	0.478874	0.994192	0.82751	0.001322	3.57E-06
0.31732	-0.14523	0.253599	0.284645	0.129738	0.293963	0.206235	0.52885	0.041436
0.000278	0.103282	0.004017	0.001181	0.14601	0.000795	0.020006	1.64E-10	0.643696
0.490739	0.011415	-0.00509	0.396905	0.226668	0.30465	0.273549	0.157723	-0.26518
4.71E-09	0.898647	0.954688	3.84E-06	0.010389	0.000497	0.001859	0.076564	0.002587
0.487174	-0.01756	0.017703	0.228953	0.126249	0.222832	0.186394	0.064209	-0.09022
6.31E-09	0.844642	0.843402	0.009621	0.15726	0.011799	0.035887	0.473262	0.313087
0.294804	-0.17289	0.086616	0.24446	-0.06082	0.174051	0.119159	0.089888	-0.02113
0.000767	0.051917	0.332905	0.005608	0.496972	0.050345	0.182094	0.314894	0.813575
0.46226	-0.17497	0.207527	0.368842	0.468328	0.34183	0.393326	0.171205	0.283165
4.47E-08	0.049122	0.019225	1.98E-05	2.82E-08	8.38E-05	4.77E-06	0.054287	0.001256
0.715634	-0.53593	0.800279	0.792604	0.602285	0.7599	0.774181	0.551946	0.622162
3.26E-21	8.41E-11	1.52E-29	1.24E-28	6.86E-14	3.86E-25	1.36E-26	1.74E-11	5.83E-15
0.594468	-0.41957	0.440258	0.725022	0.528712	0.625126	0.655278	0.216193	0.252487
1.73E-13	9.11E-07	2.23E-07	5.56E-22	1.67E-10	3.98E-15	6.35E-17	0.014638	0.004186
-0.17165	-0.23775	-0.12728	-0.25136	0.000112	-0.20144	-0.02644	-0.14568	0.084081
0.053659	0.007112	0.153859	0.004364	0.998999	0.023144	0.767942	0.1022	0.347301
-0.19873	-0.03834	-0.12091	-0.3294	-0.17175	-0.27409	-0.22565	-0.28987	0.157786
0.025106	0.66871	0.175709	0.000156	0.053508	0.00182	0.010749	0.000948	0.076446
-0.04328	-0.16834	0.263184	-0.16494	-0.07421	-0.04891	-0.11034	0.379762	0.414379
0.629009	0.058503	0.002794	0.063863	0.406983	0.584997	0.216836	1.07E-05	1.28E-06
0.262824	-0.43188	0.580013	0.230535	0.227404	0.290405	0.308814	0.360091	0.685306
0.002833	3.98E-07	8.94E-13	0.00912	0.010136	0.000926	0.000412	3.21E-05	6.29E-19
-0.2632	-0.18393	0.319404	-0.08814	-0.20861	-0.02899	-0.02706	0.112883	0.285057
0.002793	0.038456	0.000252	0.324422	0.01859	0.746282	0.762682	0.206375	0.001161
-0.66875	0.264797	-0.4162	-0.72212	-0.54582	-0.6636	-0.66776	-0.45941	-0.22108
8.56E-18	0.002625	1.14E-06	9.68E-22	3.21E-11	1.86E-17	9.95E-18	5.54E-08	0.012495
-0.53526	0.592249	-0.65414	-0.43899	-0.33977	-0.46116	-0.47782	-0.49006	-0.65772
8.97E-11	2.24E-13	7.49E-17	2.43E-07	9.3E-05	4.86E-08	1.34E-08	4.98E-09	4.45E-17
-0.67109	0.362809	-0.41661	-0.51858	-0.36744	-0.47843	-0.49456	-0.34479	-0.30649
5.98E-18	2.77E-05	1.11E-06	4.23E-10	2.14E-05	1.28E-08	3.43E-09	7.2E-05	0.000457
-0.34094	0.168567	-0.29942	-0.2725	-0.17194	-0.23023	-0.2103	-0.26948	-0.16125
8.77E-05	0.058165	0.000627	0.001939	0.05324	0.009215	0.017638	0.002185	0.070132

HAMP	HAVCR2	HBEGF	HCK	HCLS1	HCN3	HCST	HDAC4	HERPUD1
brown	brown	brown	brown	brown	brown	brown	brown	brown
0.194724	0.268407	0.181356	0.294214	0.114549	-0.1265	0.272757	-0.14319	0.29294
0.028252	0.00228	0.041298	0.000787	0.199716	0.156412	0.001919	0.108271	0.000831
0.036847	0.127553	0.119411	0.158383	0.011735	0.003702	0.205706	-0.05565	0.256774
0.680866	0.152981	0.181164	0.075325	0.895821	0.967053	0.020333	0.534319	0.003567
-0.1415	-0.37677	-0.19026	-0.37407	-0.42234	0.33701	-0.34183	0.059138	-0.39507
0.112545	1.27E-05	0.032151	1.48E-05	7.58E-07	0.000107	8.38E-05	0.508971	4.29E-06
-0.02405	-0.10578	0.031712	-0.21727	-0.33121	-0.13071	-0.25257	-0.18127	0.011437
0.788382	0.23655	0.723387	0.014139	0.000143	0.142982	0.004172	0.041396	0.898453
0.128341	0.163604	-0.01529	0.129801	-0.01508	-0.12761	0.176232	-0.31501	0.301076
0.150439	0.066078	0.864565	0.145813	0.866402	0.152791	0.047487	0.00031	0.000583
0.116395	0.176529	0.137314	0.025206	-0.15521	-0.38394	-0.016	-0.43893	0.38828
0.192516	0.047108	0.123694	0.778484	0.081443	8.35E-06	0.85828	2.44E-07	6.46E-06
0.106516	0.086103	0.157337	0.068586	-0.10728	-0.22964	0.076137	-0.31069	0.315906
0.233302	0.335787	0.077297	0.443567	0.229954	0.009401	0.394897	0.000378	0.000297
0.107185	0.250246	0.007069	0.163661	0.175347	-0.37367	-0.06156	-0.28994	0.179452
0.230361	0.004546	0.93713	0.065981	0.04863	1.51E-05	0.491773	0.000945	0.043514
0.342668	0.203493	0.419885	0.335071	0.328359	-0.27062	0.336936	-0.37411	0.30273
8.03E-05	0.021754	8.92E-07	0.000118	0.000164	0.002089	0.000107	1.47E-05	0.000541
0.652761	0.882473	0.559268	0.919262	0.833801	-0.64195	0.718205	-0.54046	0.633809
9.14E-17	9.12E-43	8.25E-12	1.89E-52	4.77E-34	4.18E-16	2.02E-21	5.43E-11	1.27E-15
0.497668	0.667057	0.397692	0.618446	0.434692	-0.57893	0.468276	-0.43199	0.458243
2.64E-09	1.11E-17	3.66E-06	9.37E-15	3.28E-07	1.01E-12	2.83E-08	3.95E-07	6.05E-08
-0.01808	-0.13969	0.106785	-0.04071	-0.06144	0.235011	0.419818	0.210115	-0.0702
0.84012	0.117262	0.232115	0.649494	0.492607	0.007823	8.96E-07	0.017741	0.432909
-0.07739	-0.27502	-0.10696	-0.10411	-0.00832	0.476078	-0.04618	0.322158	-0.51091
0.387161	0.001752	0.231359	0.244089	0.926071	1.54E-08	0.60615	0.000221	8.4E-10
0.099238	-0.02023	-0.13877	0.151656	0.243531	0.26983	0.214511	0.143827	-0.29462
0.266982	0.821415	0.119717	0.088748	0.005798	0.002156	0.015445	0.106696	0.000773
0.356422	0.412294	0.252425	0.598767	0.677592	-0.057	0.524495	-0.07326	0.020684
3.91E-05	1.46E-06	0.004195	1.04E-13	2.17E-18	0.524401	2.47E-10	0.413051	0.817455
-0.11444	0.208498	-0.09531	0.175566	0.25833	0.093609	0.105219	0.204657	-0.1463
0.200162	0.018656	0.286451	0.048344	0.003364	0.295196	0.239076	0.020997	0.100754
-0.4117	-0.55326	-0.44992	-0.52736	-0.43068	0.652685	-0.45358	0.574008	-0.6974
1.52E-06	1.53E-11	1.12E-07	1.89E-10	4.32E-07	9.24E-17	8.54E-08	1.73E-12	8.34E-20
-0.44244	-0.48813	-0.36641	-0.68111	-0.64348	0.130038	-0.62916	0.290763	-0.20507
1.91E-07	5.84E-09	2.27E-05	1.24E-18	3.39E-16	0.145073	2.35E-15	0.000912	0.020736
-0.38373	-0.42041	-0.3483	-0.47118	-0.37389	0.307501	-0.40453	0.426751	-0.25212
8.46E-06	8.61E-07	6E-05	2.26E-08	1.49E-05	0.000437	2.39E-06	5.64E-07	0.004243
-0.23337	-0.45041	-0.18118	-0.37407	-0.39174	0.612822	-0.19725	0.281546	-0.3743
0.008279	1.08E-07	0.041497	1.48E-05	5.25E-06	1.9E-14	0.026228	0.001343	1.46E-05

HES7	HEXB	HFE	HGF	HHEX	HIF3A	HK3	HLA-B	HLA-C
brown	brown	brown	brown	brown	brown	brown	brown	brown
-0.2465	0.246005	0.067836	0.471363	0.1453	-0.37393	0.081903	0.266398	0.350088
0.005211	0.005305	0.448575	2.23E-08	0.10312	1.49E-05	0.359972	0.002467	5.47E-05
-0.09727	0.120833	-0.03724	0.362337	0.02939	-0.22235	-0.04228	0.11293	0.275123
0.276645	0.175984	0.677669	2.84E-05	0.742906	0.011988	0.636954	0.206187	0.001745
0.410555	-0.05337	0.051146	-0.2832	-0.24459	-0.09045	0.012501	-0.03806	-0.18452
1.63E-06	0.551253	0.56796	0.001254	0.005581	0.311855	0.889062	0.67095	0.037824
0.020153	0.34329	0.508117	0.048823	0.080775	-0.2746	0.061863	0.284069	0.029999
0.822064	7.78E-05	1.07E-09	0.585689	0.366651	0.001782	0.489609	0.00121	0.737769
0.060589	0.282223	0.122517	0.154852	0.31335	-0.30191	-0.00535	0.183472	0.202314
0.498611	0.001306	0.169994	0.082151	0.000334	0.000562	0.952405	0.038947	0.022545
-0.17305	0.609583	0.637759	0.320004	0.412043	-0.49091	0.093074	0.468256	0.279003
0.051699	2.83E-14	7.43E-16	0.000245	1.49E-06	4.64E-09	0.297976	2.83E-08	0.001491
-0.05154	0.385915	0.401782	0.16914	0.231054	-0.16326	0.021749	0.379715	0.150523
0.564976	7.43E-06	2.84E-06	0.057304	0.00896	0.066664	0.808237	1.07E-05	0.091186
-0.37494	0.345159	0.361771	0.211013	0.310856	-0.21694	0.01801	0.333989	0.142205
1.41E-05	7.06E-05	2.93E-05	0.017248	0.000375	0.014293	0.840721	0.000124	0.110747
-0.14498	0.58635	0.442552	0.160726	0.19465	-0.30512	0.645354	0.52893	0.305506
0.103892	4.39E-13	1.89E-07	0.071053	0.028313	0.000486	2.61E-16	1.63E-10	0.000478
-0.53672	0.804687	0.57763	0.637298	0.751751	-0.47816	0.782475	0.822087	0.650832
7.8E-11	4.37E-30	1.16E-12	7.91E-16	2.35E-24	1.31E-08	1.74E-27	2.29E-32	1.2E-16
-0.53297	0.673545	0.63204	0.512995	0.546889	-0.51959	0.475448	0.803908	0.63287
1.12E-10	4.09E-18	1.6E-15	6.98E-10	2.89E-11	3.86E-10	1.62E-08	5.47E-30	1.44E-15
0.131767	-0.25057	-0.31662	-0.39883	-0.31122	0.298564	-0.04043	-0.2041	-0.16666
0.139756	0.004492	0.000287	3.41E-06	0.000369	0.000651	0.651802	0.021357	0.0611
0.407946	-0.36308	-0.34739	-0.41299	-0.40291	0.325848	0.143553	-0.22011	-0.23876
1.93E-06	2.72E-05	6.29E-05	1.4E-06	2.65E-06	0.000185	0.107373	0.0129	0.006865
0.366844	-0.28289	-0.32771	-0.2683	-0.16105	0.184034	0.240712	-0.1389	0.025117
2.21E-05	0.00127	0.000169	0.00229	0.070485	0.038342	0.006409	0.119379	0.779249
-0.03559	0.150328	0.009978	0.087507	0.200351	0.017711	0.630606	0.299223	0.26856
0.691154	0.091611	0.911346	0.327937	0.023916	0.843327	1.94E-15	0.000632	0.002267
-0.10059	-0.36875	-0.40147	0.109281	0.088134	0.237031	-0.20277	-0.26722	-0.11917
0.260496	1.99E-05	2.9E-06	0.221321	0.324467	0.007293	0.022239	0.002389	0.182062
0.507479	-0.91858	-0.78374	-0.54942	-0.61492	0.528371	-0.55862	-0.77886	-0.62946
1.14E-09	3.13E-52	1.26E-27	2.25E-11	1.46E-14	1.72E-10	8.82E-12	4.31E-27	2.26E-15
0.022486	-0.4377	-0.19568	-0.14978	-0.36987	0.179796	-0.71601	-0.51836	-0.37823
0.801868	2.66E-07	0.027472	0.092816	1.87E-05	0.043106	3.04E-21	4.32E-10	1.16E-05
0.085012	-0.67265	-0.60065	-0.27063	-0.45974	0.421384	-0.67522	-0.68993	-0.43452
0.341964	4.7E-18	8.34E-14	0.002089	5.41E-08	8.08E-07	3.15E-18	2.94E-19	3.32E-07
0.600324	-0.30816	-0.51961	-0.35498	-0.29964	0.14027	-0.19099	-0.40322	-0.46739
8.67E-14	0.000424	3.85E-10	4.22E-05	0.000621	0.115736	0.031484	2.6E-06	3.03E-08

HLA-DMA	HLA-DMB	HLA-DOA	HLA-DPA1	HLA-DPB1	HLA-DQB1	HLA-DRA	HLA-DRB1	HLA-E
brown	brown	brown	brown	brown	brown	brown	brown	brown
0.383294	0.305041	0.371221	0.393891	0.378048	0.32403	0.368438	0.436481	0.283339
8.67E-06	0.000488	1.73E-05	4.61E-06	1.18E-05	0.000202	2.03E-05	2.9E-07	0.001247
0.229178	0.162456	0.215593	0.219652	0.269855	0.206793	0.220511	0.316423	0.135882
0.009549	0.068027	0.014921	0.013092	0.002154	0.019666	0.012731	0.00029	0.127698
-0.38917	-0.38277	-0.32945	-0.40028	-0.37644	-0.1782	-0.33682	-0.3192	-0.39235
6.13E-06	8.94E-06	0.000156	3.12E-06	1.29E-05	0.045022	0.000108	0.000254	5.06E-06
-0.14195	-0.14046	-0.06335	-0.11486	-0.16879	0.063433	-0.00459	-0.13068	-0.08489
0.111395	0.115249	0.479232	0.198488	0.057826	0.478633	0.959123	0.143078	0.342688
0.124085	0.280756	0.154133	0.053545	0.099372	0.228825	0.241715	0.193214	0.020809
0.164554	0.001388	0.083599	0.549914	0.266332	0.009663	0.006186	0.029523	0.816374
0.122982	0.201753	0.196808	0.127301	0.091159	0.332235	0.323645	0.154897	0.106938
0.168367	0.02293	0.026574	0.153801	0.30807	0.000136	0.000206	0.082062	0.231442
0.125134	0.140355	0.172253	0.107519	0.115763	0.22222	0.243593	0.193567	0.100274
0.160987	0.115514	0.052807	0.228902	0.19496	0.012039	0.005785	0.029221	0.261997
0.295958	0.31214	0.30745	0.355562	0.320796	0.13782	0.392641	0.244678	0.440426
0.000729	0.000354	0.000438	4.09E-05	0.000236	0.122303	4.98E-06	0.005565	2.2E-07
0.293665	0.182628	0.284292	0.253271	0.239002	0.218438	0.247835	0.140024	0.281001
0.000805	0.039871	0.001199	0.004066	0.006807	0.013617	0.004965	0.116385	0.001374
0.928665	0.857958	0.902681	0.84446	0.788009	0.633466	0.87029	0.644978	0.844198
1.11E-55	5.74E-38	1.32E-47	1.08E-35	4.19E-28	1.33E-15	2.93E-40	2.75E-16	1.18E-35
0.682882	0.60428	0.679379	0.691936	0.601424	0.460476	0.675674	0.471681	0.746334
9.31E-19	5.4E-14	1.63E-18	2.1E-19	7.61E-14	5.12E-08	2.93E-18	2.17E-08	7.52E-24
-0.08976	-0.1539	-0.1537	-0.16625	-0.17924	-0.29572	-0.26435	-0.23256	-0.16772
0.315612	0.084066	0.084484	0.061749	0.04377	0.000737	0.002671	0.008511	0.059465
-0.2031	-0.32905	-0.23336	-0.25798	-0.21408	-0.30078	-0.3951	-0.2597	-0.17373
0.022012	0.000159	0.00828	0.003408	0.015658	0.00059	4.29E-06	0.003194	0.050778
-0.02062	0.001088	-0.05806	-0.10621	-0.0324	-0.13463	-0.1747	-0.10641	0.037773
0.818004	0.990311	0.516762	0.234671	0.717606	0.13129	0.049481	0.233768	0.6733
0.493658	0.366931	0.427659	0.390147	0.403049	0.159044	0.265319	0.239217	0.503768
3.69E-09	2.2E-05	5.31E-07	5.78E-06	2.63E-06	0.074099	0.002573	0.006756	1.57E-09
0.17364	0.17437	0.128437	0.173585	0.170137	-0.03619	0.084367	0.170142	0.129221
0.050899	0.049917	0.150132	0.050973	0.055831	0.68625	0.345653	0.055823	0.147636
-0.60908	-0.56131	-0.61985	-0.58463	-0.54638	-0.58479	-0.6624	-0.46583	-0.59072
3.01E-14	6.67E-12	7.84E-15	5.33E-13	3.04E-11	5.24E-13	2.23E-17	3.41E-08	2.67E-13
-0.58739	-0.4717	-0.54168	-0.44177	-0.46862	-0.30391	-0.41087	-0.36372	-0.54193
3.9E-13	2.17E-08	4.83E-11	2E-07	2.75E-08	0.000514	1.6E-06	2.63E-05	4.71E-11
-0.46188	-0.3847	-0.47662	-0.3647	-0.34401	-0.37942	-0.41706	-0.26968	-0.47831
4.61E-08	7.99E-06	1.48E-08	2.49E-05	7.5E-05	1.09E-05	1.07E-06	0.002169	1.29E-08
-0.43182	-0.42276	-0.4075	-0.55674	-0.50286	-0.25644	-0.48365	-0.29164	-0.63128
4E-07	7.37E-07	1.99E-06	1.07E-11	1.69E-09	0.003612	8.41E-09	0.000879	1.77E-15

HLA-F	HMGCL	HMGCR	HMGCS1	HMOX1	HNMT	HP	HPD	HPGDS
brown	brown	brown	brown	brown	brown	brown	brown	brown
0.324114	0.117159	-0.18199	-0.1998	0.154565	0.087459	0.011749	0.004953	0.299586
0.000201	0.189592	0.040579	0.024311	0.082727	0.328202	0.895692	0.955927	0.000622
0.126184	0.095248	-0.0599	-0.11836	0.056069	0.052479	-0.05953	-0.04876	0.220072
0.157475	0.286783	0.503501	0.185065	0.531242	0.557902	0.506168	0.586208	0.012914
-0.32073	-0.05696	0.25666	0.274815	-0.07673	-0.11571	0.012137	-0.00993	-0.06286
0.000237	0.52471	0.003582	0.001767	0.391194	0.195151	0.892268	0.911799	0.48262
-0.17318	0.204599	0.162497	0.164286	0.152502	0.107916	0.19815	0.235322	0.112131
0.051526	0.021033	0.067958	0.064941	0.086963	0.22718	0.025539	0.007739	0.209436
0.178304	0.555116	0.018891	-0.23557	0.017108	0.320997	-0.05614	0.225301	0.364297
0.044896	1.26E-11	0.833043	0.007672	0.848601	0.000234	0.5307	0.010873	2.55E-05
0.083303	0.578166	0.08159	-0.06683	0.218655	0.392844	0.191756	0.398736	0.392404
0.351796	1.1E-12	0.361818	0.45535	0.013522	4.91E-06	0.030796	3.43E-06	5.05E-06
0.021713	0.380386	0.085421	-0.03763	0.175878	0.131462	0.108051	0.129561	0.246555
0.808549	1.03E-05	0.339638	0.674492	0.047941	0.140683	0.226594	0.146566	0.005201
0.097565	0.336597	-0.21358	-0.23673	0.111497	0.487004	0.128216	0.40332	0.134405
0.275169	0.000109	0.015907	0.00737	0.21204	6.4E-09	0.150839	2.58E-06	0.131928
0.346878	0.259587	-0.37977	-0.25045	0.594932	-0.09335	0.610581	0.29369	0.030437
6.46E-05	0.003207	1.06E-05	0.004513	1.64E-13	0.296537	2.5E-14	0.000805	0.734088
0.870018	0.600333	-0.72949	-0.64888	0.76618	0.448241	0.549632	0.556922	0.553932
3.31E-40	8.66E-14	2.33E-22	1.59E-16	9.14E-26	1.26E-07	2.2E-11	1.05E-11	1.42E-11
0.728334	0.484213	-0.46441	-0.33666	0.541462	0.380509	0.395098	0.477761	0.457812
2.93E-22	8.03E-09	3.8E-08	0.000109	4.93E-11	1.02E-05	4.29E-06	1.35E-08	6.25E-08
-0.07969	-0.17139	-0.04199	0.129757	0.059194	-0.46395	0.023763	-0.13855	-0.26996
0.373122	0.054028	0.639288	0.145952	0.508567	3.94E-08	0.790869	0.120311	0.002145
-0.13398	-0.37155	-0.14446	0.081618	0.014369	-0.36731	0.041049	-0.16918	-0.33086
0.133171	1.7E-05	0.105149	0.361654	0.872615	2.16E-05	0.646797	0.057242	0.000145
0.238698	-0.00294	-0.38556	-0.30987	0.047301	-0.08407	0.059139	0.034714	-0.13658
0.006881	0.973846	7.59E-06	0.000392	0.597447	0.34735	0.508963	0.69842	0.125743
0.575234	0.112104	-0.72457	-0.4779	0.483746	0.04339	0.366362	0.22845	0.066944
1.51E-12	0.209547	6.07E-22	1.33E-08	8.34E-09	0.62812	2.27E-05	0.009786	0.454578
0.074238	-0.24542	-0.12452	-0.1147	-0.18959	0.0156	-0.2779	-0.27052	0.157927
0.406826	0.005418	0.16308	0.199103	0.032777	0.861809	0.00156	0.002097	0.07618
-0.578	-0.70508	0.48346	0.491972	-0.60873	-0.41418	-0.56236	-0.56563	-0.37197
1.12E-12	2.19E-20	8.54E-09	4.25E-09	3.14E-14	1.29E-06	5.98E-12	4.24E-12	1.66E-05
-0.61797	-0.45779	0.738266	0.556909	-0.63991	-0.18292	-0.46246	-0.43888	-0.25353
9.96E-15	6.26E-08	4.03E-23	1.05E-11	5.54E-16	0.039552	4.41E-08	2.45E-07	0.004027
-0.51462	-0.57374	0.57313	0.429195	-0.65181	-0.302	-0.53704	-0.56793	-0.34642
6.04E-10	1.78E-12	1.9E-12	4.78E-07	1.05E-16	0.000559	7.57E-11	3.32E-12	6.62E-05
-0.37785	-0.35534	0.414672	0.31667	-0.27726	-0.44065	-0.37785	-0.46401	-0.1654
1.19E-05	4.14E-05	1.25E-06	0.000286	0.001601	2.17E-07	1.19E-05	3.92E-08	0.063121

HPS5	HPSE	HSDL1	HSH2D	HTATIP2	HTR2B	HTRA4	HUNK	ICAM1
brown	brown	brown	brown	brown	brown	brown	brown	brown
0.059166	0.283524	-0.22997	0.422437	0.396656	0.333813	0.407083	-0.27851	0.306425
0.508769	0.001237	0.009295	7.53E-07	3.9E-06	0.000125	2.04E-06	0.001521	0.000459
-0.03036	0.112972	-0.12693	0.265246	0.406137	0.145707	0.235901	-0.23421	0.190512
0.734768	0.206017	0.155024	0.00258	2.16E-06	0.102148	0.007586	0.008043	0.031919
-0.03465	-0.21789	0.349261	-0.21371	-0.58394	-0.0229	-0.16304	0.364586	-0.22766
0.698919	0.013861	5.71E-05	0.015845	5.77E-13	0.798311	0.067036	2.51E-05	0.010051
0.360199	-0.01149	0.098113	-0.05221	-0.2684	0.217805	0.096105	0.03612	0.074824
3.19E-05	0.897962	0.272469	0.559885	0.002281	0.013898	0.282451	0.686831	0.403118
-0.14166	0.119751	0.030831	0.094077	0.387063	0.12613	0.093188	-0.12051	-0.03601
0.112132	0.179916	0.730774	0.292776	6.95E-06	0.157653	0.297386	0.177135	0.687706
0.365261	0.183391	-0.03534	0.121698	0.237191	0.350316	0.243265	-0.20103	0.186097
2.42E-05	0.039036	0.693211	0.17289	0.007253	5.4E-05	0.005854	0.023436	0.036188
0.231861	0.023313	-0.04395	0.129039	0.251536	0.268028	0.169518	-0.1148	0.254028
0.008717	0.794737	0.623708	0.148212	0.004336	0.002314	0.056741	0.198732	0.003953
0.322104	0.244315	-0.20672	0.130979	0.288392	0.093806	0.263117	-0.46374	0.173335
0.000222	0.005638	0.019713	0.14216	0.001009	0.294178	0.002802	4E-08	0.051313
0.497538	0.155307	-0.42846	0.363589	-0.00111	0.491849	0.301128	-0.06783	0.508944
2.67E-09	0.081244	5.03E-07	2.65E-05	0.990111	4.3E-09	0.000581	0.448612	9.99E-10
0.709732	0.728418	-0.78611	0.752576	0.645801	0.618223	0.730689	-0.64865	0.733805
9.57E-21	2.88E-22	6.86E-28	1.97E-24	2.45E-16	9.64E-15	1.84E-22	1.64E-16	9.92E-23
0.684438	0.662027	-0.60422	0.725174	0.334256	0.591384	0.609605	-0.49975	0.637245
7.24E-19	2.36E-17	5.44E-14	5.4E-22	0.000123	2.47E-13	2.82E-14	2.21E-09	7.97E-16
-0.03761	-0.20413	-0.06814	0.066299	-0.34534	-0.10628	-0.14096	0.37179	0.110556
0.674659	0.021335	0.446524	0.458946	7E-05	0.234331	0.11394	1.68E-05	0.215948
-0.10454	-0.19159	0.028601	-0.06034	-0.5686	-0.16533	-0.12328	0.279454	-0.10034
0.242142	0.03094	0.74958	0.500378	3.09E-12	0.063227	0.167346	0.001464	0.261666
-0.26723	0.036234	-0.16455	0.015917	-0.16847	-0.18412	-0.05557	-0.00861	-0.11893
0.002388	0.685891	0.064505	0.859025	0.058306	0.038255	0.534914	0.923486	0.182956
0.298364	0.379947	-0.60001	0.445275	0.113913	0.193559	0.35875	-0.31024	0.357842
0.000657	1.05E-05	9E-14	1.56E-07	0.20224	0.029228	3.45E-05	0.000386	3.62E-05
-0.14973	0.18315	-0.06702	0.007886	0.412085	-0.23819	0.017768	-0.23828	-0.19012
0.092926	0.039297	0.454034	0.929883	1.48E-06	0.007005	0.842831	0.006981	0.032281
-0.69131	-0.4464	0.609388	-0.58384	-0.55368	-0.61933	-0.59225	0.56156	-0.63301
2.33E-19	1.44E-07	2.9E-14	5.83E-13	1.46E-11	8.37E-15	2.24E-13	6.5E-12	1.41E-15
-0.41966	-0.42291	0.654753	-0.55937	-0.23799	-0.33426	-0.46985	0.337251	-0.50007
9.05E-07	7.3E-07	6.86E-17	8.16E-12	0.007052	0.000123	2.5E-08	0.000106	2.15E-09
-0.6449	-0.44948	0.542224	-0.51114	-0.13328	-0.49683	-0.54017	0.375651	-0.47713
2.78E-16	1.15E-07	4.58E-11	8.23E-10	0.135225	2.83E-09	5.59E-11	1.35E-05	1.42E-08
-0.48375	-0.36456	0.540414	-0.20383	-0.37154	-0.12478	-0.24136	0.613369	-0.39826
8.34E-09	2.51E-05	5.46E-11	0.021531	1.7E-05	0.162182	0.006265	1.77E-14	3.53E-06

ICAM4	ICOS	IER3	IFI16	IFITM1	IFITM2	IFITM3	IFNG	IFNGR2
brown	brown	brown	brown	brown	brown	brown	brown	brown
0.193413	0.419155	0.23098	0.262667	0.43672	0.292741	0.308985	0.413376	0.170056
0.029353	9.36E-07	0.008983	0.002851	2.85E-07	0.000838	0.000408	1.36E-06	0.055949
0.171556	0.255327	0.112579	0.125457	0.332386	0.279193	0.25946	0.243311	0.09497
0.053787	0.003766	0.207611	0.159899	0.000135	0.001479	0.003222	0.005844	0.288201
-0.34413	-0.23738	-0.47163	0.078653	-0.41394	-0.4294	-0.41481	-0.09698	0.088098
7.45E-05	0.007204	2.18E-08	0.379416	1.31E-06	4.72E-07	1.24E-06	0.27807	0.324664
-0.21802	0.027329	-0.22135	0.460704	-0.23871	-0.25079	-0.33778	0.116631	0.305667
0.013804	0.760374	0.012386	5.03E-08	0.006878	0.004456	0.000103	0.191611	0.000475
0.040941	0.155657	-0.02804	0.195503	0.24367	0.336562	0.203307	0.161365	0.375853
0.647663	0.080552	0.754313	0.027614	0.005769	0.000109	0.021877	0.069924	1.33E-05
-0.06347	0.283367	-0.03561	0.622822	0.093864	0.146387	-0.03276	0.305379	0.528788
0.478388	0.001246	0.691021	5.36E-15	0.293879	0.100539	0.714658	0.000481	1.65E-10
0.133716	0.215499	0.024314	0.381771	0.133759	0.259275	0.018465	0.205797	0.485888
0.133941	0.014966	0.786136	9.48E-06	0.133813	0.003245	0.836754	0.020277	7.01E-09
-0.01963	0.248843	0.20559	0.340531	0.015091	0.147964	0.124955	0.074662	0.038356
0.826629	0.004786	0.020405	8.95E-05	0.866268	0.096887	0.161592	0.404145	0.668556
0.303479	0.37	0.353452	0.464994	0.256672	0.333982	0.336143	0.297651	0.559735
0.000524	1.86E-05	4.58E-05	3.64E-08	0.003581	0.000124	0.000112	0.000677	7.86E-12
0.531281	0.789683	0.719304	0.721461	0.646546	0.716765	0.712992	0.544996	0.604848
1.31E-10	2.7E-28	1.65E-21	1.1E-21	2.21E-16	2.65E-21	5.3E-21	3.49E-11	5.04E-14
0.243576	0.676571	0.496198	0.762212	0.647406	0.452088	0.540061	0.546249	0.447383
0.005789	2.55E-18	2.99E-09	2.28E-25	1.96E-16	9.53E-08	5.65E-11	3.08E-11	1.34E-07
0.241943	-0.22583	0.15043	-0.15394	0.053569	-0.12399	-0.02396	-0.12706	0.067034
0.006136	0.010682	0.091389	0.083996	0.549736	0.164863	0.789139	0.154599	0.453976
-0.02746	-0.27947	-0.1466	-0.30156	-0.2908	-0.31351	-0.15223	-0.22874	-0.23498
0.759236	0.001463	0.100049	0.00057	0.000911	0.000332	0.087528	0.009691	0.007831
0.12646	-0.14617	0.020115	-0.38158	-0.00994	0.011985	0.242719	-0.22421	-0.21706
0.156559	0.10104	0.822395	9.58E-06	0.911654	0.893613	0.005969	0.011273	0.014237
0.389058	0.284149	0.433931	0.074751	0.270334	0.332069	0.508996	0.111326	0.067
6.17E-06	0.001206	3.46E-07	0.403585	0.002113	0.000137	9.94E-10	0.212746	0.454201
0.00706	-0.07807	0.088876	-0.3186	0.100289	0.086269	0.157967	-0.14775	-0.42914
0.93721	0.382996	0.320394	0.000262	0.261925	0.334852	0.076105	0.097366	4.8E-07
-0.36596	-0.69144	-0.49473	-0.76264	-0.51222	-0.5971	-0.48584	-0.50494	-0.6533
2.33E-05	2.29E-19	3.38E-09	2.07E-25	7.48E-10	1.27E-13	7.04E-09	1.42E-09	8.45E-17
-0.45073	-0.41467	-0.46487	-0.35802	-0.368	-0.50896	-0.57731	-0.27612	-0.40762
1.05E-07	1.25E-06	3.67E-08	3.59E-05	2.08E-05	9.97E-10	1.2E-12	0.001676	1.97E-06
-0.24026	-0.45355	-0.26943	-0.65486	-0.22468	-0.29748	-0.31891	-0.35398	-0.54093
0.006512	8.56E-08	0.00219	6.75E-17	0.0111	0.000683	0.000258	4.45E-05	5.19E-11
-0.19179	-0.30659	-0.48941	-0.25732	-0.28766	-0.32908	-0.42927	-0.0345	0.082073
0.030765	0.000455	5.26E-09	0.003494	0.001041	0.000158	4.76E-07	0.700164	0.358976

IGDCC3	IGFBP7	IGFLR1	IGLL5	IGSF6	IKBKE	IKZF1	IKZF3	IL10
brown	brown	brown	brown	brown	brown	brown	brown	brown
-0.36232	-0.00141	-0.00451	0.50055	0.269735	0.144384	0.289928	0.382335	0.182141
2.84E-05	0.987431	0.959859	2.06E-09	0.002164	0.105332	0.000945	9.17E-06	0.040412
-0.26071	-0.00419	-0.12026	0.388968	0.142515	0.048884	0.100035	0.190844	0.096552
0.003072	0.962745	0.178063	6.2E-06	0.109966	0.585224	0.263139	0.031616	0.280209
0.478937	-0.01096	-0.02689	-0.40827	-0.25573	-0.01022	-0.35677	-0.26216	-0.30278
1.23E-08	0.902645	0.764136	1.89E-06	0.00371	0.90925	3.84E-05	0.002907	0.00054
0.150279	0.158856	-0.1143	-0.18338	-0.07861	0.08983	-0.10385	0.032188	-0.03352
0.091718	0.074447	0.200681	0.039047	0.379662	0.31521	0.245271	0.719413	0.708292
-0.19559	0.20177	-0.06668	0.038541	0.212383	0.056853	-0.04152	0.073557	0.022235
0.027542	0.022918	0.456367	0.667051	0.016521	0.525504	0.643044	0.411157	0.804031
-0.19201	0.331404	-0.16787	0.025382	0.173897	0.171021	0.058883	0.233607	0.142358
0.030572	0.000141	0.059231	0.776975	0.050552	0.05455	0.510806	0.008211	0.110361
-0.22617	0.240742	-0.16907	0.081506	0.100971	0.133397	-0.01282	0.112859	0.100802
0.010564	0.006402	0.057408	0.362316	0.258677	0.134878	0.886247	0.206473	0.259477
-0.40196	0.128003	-0.03344	0.08452	0.278617	0.1232	0.279128	0.139052	0.146769
2.81E-06	0.151525	0.708973	0.344781	0.001515	0.167608	0.001483	0.118967	0.099645
-0.13301	0.606242	0.414999	0.38391	0.012919	0.465009	0.268824	0.378998	0.435672
0.136035	4.26E-14	1.23E-06	8.36E-06	0.885381	3.63E-08	0.002243	1.11E-05	3.07E-07
-0.56036	0.674697	0.615362	0.754922	0.674775	0.798927	0.865192	0.78713	0.778169
7.37E-12	3.42E-18	1.38E-14	1.17E-24	3.37E-18	2.22E-29	2.77E-39	5.27E-28	5.12E-27
-0.36862	0.409073	0.455731	0.54832	0.469907	0.602156	0.683901	0.687316	0.550437
2.01E-05	1.8E-06	7.29E-08	2.51E-11	2.49E-08	6.97E-14	7.9E-19	4.52E-19	2.03E-11
0.264138	0.042376	0.368869	0.170194	-0.3548	-0.00341	-0.13977	-0.08284	0.121606
0.002693	0.636181	1.98E-05	0.055747	4.26E-05	0.969672	0.117045	0.354473	0.173216
0.446311	-0.12323	0.345257	-0.10723	-0.24778	0.066092	-0.15184	-0.2312	-0.17561
1.45E-07	0.167494	7.03E-05	0.230169	0.004975	0.460356	0.088352	0.008916	0.048283
0.260904	-0.00101	0.532626	-0.00174	-0.00892	0.138951	-0.0574	-0.18148	-0.06915
0.00305	0.991036	1.15E-10	0.984534	0.920725	0.119237	0.521514	0.041153	0.439815
-0.05034	0.3555	0.758209	0.464047	0.281385	0.573207	0.474385	0.303289	0.373242
0.574098	4.11E-05	5.65E-25	3.91E-08	0.001352	1.88E-12	1.76E-08	0.000528	1.55E-05
-0.08009	-0.14262	-0.04624	0.116651	0.26336	-0.04707	0.191407	-0.01157	-0.03341
0.370722	0.109698	0.605729	0.191535	0.002776	0.599241	0.031108	0.897299	0.709259
0.565703	-0.57019	-0.25132	-0.5868	-0.37036	-0.53091	-0.53297	-0.68217	-0.57109
4.21E-12	2.61E-12	0.00437	4.17E-13	1.82E-05	1.36E-10	1.12E-10	1.04E-18	2.37E-12
0.160881	-0.57307	-0.74424	-0.5397	-0.34844	-0.70233	-0.48163	-0.39723	-0.4541
0.070778	1.91E-12	1.17E-23	5.85E-11	5.96E-05	3.56E-20	9.9E-09	3.76E-06	8.22E-08
0.070314	-0.62037	-0.57278	-0.39267	-0.3048	-0.6959	-0.41252	-0.44441	-0.41314
0.432138	7.33E-15	1.97E-12	4.97E-06	0.000494	1.08E-19	1.44E-06	1.66E-07	1.38E-06
0.297214	-0.25195	-0.19671	-0.24324	-0.2917	-0.18635	-0.42194	-0.30126	-0.3767
0.00069	0.00427	0.026655	0.005859	0.000877	0.035933	7.78E-07	0.000578	1.27E-05

IL10RA	IL12RB1	IL13RA1	IL15	IL15RA	IL16	IL17RA	IL18	IL1R1
brown	brown	brown	brown	brown	brown	brown	brown	brown
0.399456	0.304241	0.170701	0.309828	0.160974	0.282988	0.162704	0.272578	0.247551
3.28E-06	0.000506	0.055011	0.000393	0.070613	0.001265	0.067601	0.001933	0.005016
0.255387	0.118941	0.089288	0.210173	0.049308	0.091914	-0.01232	0.173121	0.149461
0.003758	0.182901	0.318146	0.017708	0.581968	0.304066	0.890623	0.051606	0.093518
-0.41717	-0.33474	-0.29617	-0.19331	-0.14524	-0.36771	-0.35343	-0.38307	-0.20451
1.07E-06	0.00012	0.000723	0.029439	0.103266	2.11E-05	4.58E-05	8.79E-06	0.02109
-0.1747	-0.23701	0.164637	0.226647	0.010666	-0.10113	-0.18458	-0.11083	0.130372
0.049481	0.007298	0.064362	0.010396	0.905267	0.25792	0.03776	0.214818	0.144035
0.003704	0.035665	0.02913	0.374862	0.113744	0.149558	-0.24436	0.272184	0.021997
0.967038	0.690573	0.745106	1.41E-05	0.202912	0.093305	0.005629	0.001964	0.806094
0.032528	-0.05558	0.368426	0.608491	0.170983	0.143186	-0.16051	0.245398	0.299306
0.716571	0.534849	2.03E-05	3.24E-14	0.054604	0.108285	0.071442	0.005422	0.00063
0.033051	-0.07699	0.251565	0.353933	0.170209	0.138831	-0.1292	0.132236	0.227135
0.712211	0.389582	0.004331	4.46E-05	0.055725	0.119559	0.147703	0.138338	0.010228
0.247873	0.045432	0.440757	0.319509	0.075901	0.340026	0.185801	0.305218	0.126519
0.004958	0.612019	2.15E-07	0.000251	0.396368	9.18E-05	0.036491	0.000484	0.156366
0.349786	0.188067	0.39882	0.357038	0.524848	0.335177	0.339999	0.131875	0.650739
5.55E-05	0.034227	3.41E-06	3.78E-05	2.39E-10	0.000117	9.2E-05	0.139429	1.22E-16
0.905747	0.731563	0.803471	0.762642	0.830019	0.890487	0.723772	0.833113	0.752528
1.96E-48	1.55E-22	6.19E-30	2.07E-25	1.72E-33	1.43E-44	7.07E-22	6.04E-34	1.99E-24
0.635943	0.641704	0.646836	0.655479	0.575396	0.663971	0.548373	0.511324	0.543514
9.5E-16	4.33E-16	2.12E-16	6.17E-17	1.49E-12	1.76E-17	2.49E-11	8.1E-10	4.03E-11
-0.07908	-0.03989	-0.13173	-0.34405	0.002474	-0.04742	0.077618	-0.25334	-0.02293
0.376805	0.656141	0.139864	7.48E-05	0.977981	0.596547	0.385741	0.004056	0.798031
-0.14124	-0.07476	-0.35137	-0.53223	-0.04359	-0.16013	0.043651	-0.36546	-0.2083
0.113206	0.403509	5.11E-05	1.2E-10	0.626537	0.072115	0.626054	2.39E-05	0.018768
-0.02626	0.150587	-0.35195	-0.26416	0.112128	0.026616	0.037931	-0.08358	-0.21739
0.769443	0.091047	4.96E-05	0.002691	0.209446	0.766441	0.672009	0.350201	0.014083
0.530079	0.497057	0.217778	0.058183	0.518518	0.500674	0.563638	0.316734	0.286223
1.47E-10	2.78E-09	0.013911	0.515846	4.26E-10	2.04E-09	5.23E-12	0.000286	0.001105
0.151489	0.160197	-0.02001	-0.22791	-0.12037	0.048535	0.177931	0.231442	-0.24205
0.089104	0.072	0.82328	0.009965	0.177661	0.587909	0.045354	0.008843	0.006113
-0.60267	-0.40642	-0.74426	-0.86886	-0.62396	-0.64162	-0.38496	-0.57792	-0.77807
6.55E-14	2.13E-06	1.16E-23	5.55E-40	4.62E-15	4.38E-16	7.86E-06	1.13E-12	5.25E-27
-0.57841	-0.4684	-0.36058	-0.28341	-0.64342	-0.63854	-0.49236	-0.42752	-0.43611
1.07E-12	2.8E-08	3.12E-05	0.001244	3.41E-16	6.68E-16	4.12E-09	5.36E-07	2.98E-07
-0.43087	-0.32263	-0.47271	-0.51637	-0.5994	-0.51018	-0.31475	-0.37871	-0.50581
4.27E-07	0.000216	2.01E-08	5.17E-10	9.68E-14	8.96E-10	0.000313	1.13E-05	1.31E-09
-0.37633	-0.36667	-0.59724	-0.42522	-0.21857	-0.3781	-0.41978	-0.386	-0.31964
1.3E-05	2.24E-05	1.25E-13	6.26E-07	0.013561	1.17E-05	8.98E-07	7.4E-06	0.000249

IL1RN	IL21R	IL23A	IL27	IL2RA	IL2RB	IL2RG	IL32	IL4I1
brown	brown	brown	brown	brown	brown	brown	brown	brown
0.19263	0.379701	0.14001	0.22276	0.114095	0.240975	0.424561	0.237253	0.308711
0.030028	1.07E-05	0.116421	0.011827	0.201515	0.00635	6.54E-07	0.007237	0.000414
0.052025	0.210415	0.124919	0.137025	-0.04125	-0.014	0.254271	0.134639	0.132236
0.561314	0.017575	0.161713	0.124495	0.645222	0.875834	0.003918	0.131251	0.138339
0.022029	-0.25853	-0.05535	-0.15368	-0.0002	-0.0055	-0.19173	-0.2057	-0.07959
0.805814	0.003338	0.536523	0.084524	0.998216	0.951069	0.030821	0.020336	0.373769
0.16883	-0.11524	0.150782	-0.13552	0.2024	0.160643	0.049017	0.01826	-0.0399
0.057768	0.196987	0.090624	0.128739	0.022486	0.071201	0.584196	0.838543	0.656033
0.130672	-0.03638	0.407113	0.016376	-0.02045	-0.11177	0.174012	0.189536	-0.1191
0.143105	0.684726	2.03E-06	0.85501	0.819463	0.210934	0.050397	0.032824	0.182319
0.284714	-0.00452	0.4263	-0.04645	0.24166	0.151197	0.273674	0.253439	-0.05364
0.001178	0.959745	5.82E-07	0.604092	0.006198	0.08973	0.00185	0.004041	0.549235
0.178098	0.019759	0.433375	-0.04726	0.147912	0.083227	0.215443	0.17627	-0.09874
0.045148	0.825491	3.59E-07	0.5978	0.097005	0.352235	0.014993	0.047438	0.269384
0.119115	0.094246	0.050081	0.022879	0.113796	0.151336	0.138224	0.127217	-0.00237
0.182258	0.291909	0.576058	0.798479	0.202706	0.089431	0.121202	0.154075	0.978923
0.425421	0.464641	0.1929	0.19123	0.696786	0.628586	0.442525	0.558074	0.496587
6.17E-07	3.74E-08	0.029793	0.031267	9.27E-20	2.53E-15	1.9E-07	9.33E-12	2.89E-09
0.530166	0.784021	0.473186	0.557082	0.691327	0.775637	0.887076	0.810212	0.700208
1.45E-10	1.17E-27	1.93E-08	1.03E-11	2.33E-19	9.55E-27	8.71E-44	8.75E-31	5.15E-20
0.481559	0.622278	0.259834	0.446262	0.505838	0.691053	0.693318	0.625259	0.606386
9.95E-09	5.75E-15	0.003177	1.45E-07	1.31E-09	2.44E-19	1.67E-19	3.91E-15	4.18E-14
-0.12106	0.114586	-0.17192	-0.01358	-0.02356	-0.00621	-0.06129	-0.01144	0.091072
0.175163	0.19957	0.05328	0.879549	0.792654	0.944762	0.493621	0.898434	0.308535
-0.11461	-0.00947	-0.27203	0.063549	-0.00869	0.079876	-0.16175	-0.16748	0.180048
0.199466	0.91587	0.001975	0.477832	0.922729	0.372027	0.069243	0.059825	0.04281
-0.10444	0.07836	-0.03725	0.245045	-0.04066	-0.03766	-0.08849	0.084614	0.244202
0.242572	0.3812	0.677589	0.005492	0.649928	0.67425	0.322489	0.344243	0.005661
0.155077	0.524918	0.075205	0.471497	0.370731	0.499268	0.413184	0.439582	0.617016
0.081702	2.37E-10	0.400722	2.2E-08	1.78E-05	2.3E-09	1.38E-06	2.33E-07	1.12E-14
-0.37322	0.003921	-0.20196	0.090795	-0.41058	-0.21122	-0.02919	-0.18281	-0.08631
1.55E-05	0.965106	0.02279	0.310013	1.63E-06	0.017136	0.744624	0.039667	0.334601
-0.486	-0.50336	-0.51469	-0.28228	-0.6743	-0.61178	-0.69919	-0.7189	-0.41494
6.95E-09	1.62E-09	6.01E-10	0.001303	3.63E-18	2.16E-14	6.14E-20	1.78E-21	1.23E-06
-0.35041	-0.56008	-0.30523	-0.46452	-0.49274	-0.59108	-0.59492	-0.59556	-0.62275
5.38E-05	7.58E-12	0.000484	3.77E-08	3.99E-09	2.56E-13	1.64E-13	1.52E-13	5.41E-15
-0.39818	-0.42874	-0.40165	-0.35693	-0.58736	-0.64247	-0.59545	-0.5785	-0.54239
3.55E-06	4.93E-07	2.87E-06	3.8E-05	3.92E-13	3.9E-16	1.54E-13	1.06E-12	4.5E-11
-0.08895	-0.26083	0.004788	-0.21185	-0.20389	-0.1801	-0.20027	-0.45228	-0.21895
0.319974	0.003059	0.957397	0.016801	0.021494	0.042753	0.023978	9.4E-08	0.013393

IL4R	IL6	IL6R	IL7R	ILDR1	INAFM2	INMT	INPP5D	IRAK1
brown	brown	brown	brown	brown	brown	brown	brown	brown
0.115306	0.130257	0.268005	0.064443	0.083116	0.038239	0.191685	0.121653	-0.06215
0.196741	0.144391	0.002317	0.471649	0.35288	0.669501	0.03086	0.17305	0.487601
-0.00628	0.025499	0.126707	-0.07211	0.040519	-0.00716	0.110749	-0.02155	-0.14253
0.944138	0.775978	0.155746	0.420414	0.651051	0.936279	0.215139	0.809967	0.109938
-0.22708	0.068883	-0.41649	0.00754	-0.2052	-0.14004	-0.15654	-0.41005	-0.05158
0.010248	0.441592	1.11E-06	0.93295	0.020654	0.116348	0.078826	1.69E-06	0.564709
-0.10359	0.276434	-0.19912	0.250899	0.002721	0.125706	-0.03148	-0.31333	-0.17311
0.246456	0.001655	0.02481	0.004439	0.975775	0.159066	0.725291	0.000335	0.051618
-0.06221	0.043547	-0.11975	0.017908	-0.00211	0.048847	0.172443	-0.23054	-0.10093
0.487192	0.626879	0.179932	0.841608	0.981221	0.585502	0.052542	0.009119	0.258849
-0.02176	0.342793	-0.12039	0.324278	0.090368	0.264759	0.141966	-0.28676	-0.21529
0.808176	7.98E-05	0.177592	0.0002	0.312304	0.002629	0.111355	0.001081	0.015065
0.08174	0.238046	-0.06911	0.182668	0.12228	0.255931	0.031088	-0.17859	-0.1993
0.360936	0.007039	0.440088	0.039827	0.170829	0.003682	0.728618	0.04455	0.024682
0.075225	0.092249	0.185092	0.168906	0.089463	0.179097	0.031313	0.274849	0.135179
0.400599	0.302297	0.037224	0.057654	0.317196	0.043937	0.726731	0.001765	0.129698
0.655289	0.498257	0.210007	0.639636	0.135161	0.308132	0.406092	0.171062	0.413076
6.34E-17	2.51E-09	0.0178	5.75E-16	0.129749	0.000425	2.17E-06	0.054492	1.39E-06
0.800724	0.521227	0.787874	0.706073	0.500304	0.530052	0.624155	0.685143	0.60315
1.34E-29	3.33E-10	4.34E-28	1.84E-20	2.11E-09	1.47E-10	4.51E-15	6.46E-19	6.19E-14
0.529476	0.455084	0.587044	0.530763	0.33603	0.39648	0.462876	0.507979	0.35198
1.55E-10	7.65E-08	4.06E-13	1.37E-10	0.000112	3.94E-06	4.27E-08	1.09E-09	4.95E-05
0.051785	-0.08084	0.062249	-0.10655	-0.10047	-0.10717	-0.00697	0.051063	-0.01881
0.563122	0.366256	0.486899	0.233134	0.261072	0.230419	0.937984	0.568589	0.833724
0.075235	-0.11637	0.045871	-0.10561	-0.07252	-0.24548	-0.08832	0.116028	0.341842
0.400534	0.192603	0.608581	0.237316	0.417791	0.005406	0.323443	0.193934	8.37E-05
0.157376	-0.19314	0.118183	-0.16027	-0.01788	-0.29624	0.169141	0.191326	0.55102
0.077223	0.029584	0.185724	0.071871	0.841849	0.00072	0.057302	0.03118	1.91E-11
0.638053	0.123316	0.61955	0.285775	0.264406	0.06365	0.401256	0.657041	0.793634
7.14E-16	0.167205	8.15E-15	0.001126	0.002665	0.477126	2.94E-06	4.92E-17	9.42E-29
-0.11134	-0.41291	0.284618	-0.35799	0.065765	-0.16376	-0.04194	0.323271	-0.02718
0.212687	1.41E-06	0.001182	3.59E-05	0.462581	0.065814	0.639673	0.00021	0.761663
-0.56858	-0.53374	-0.41449	-0.71419	-0.35814	-0.46587	-0.42639	-0.23089	-0.25468
3.1E-12	1.04E-10	1.27E-06	4.26E-21	3.56E-05	3.4E-08	5.78E-07	0.009009	0.003858
-0.68906	-0.2991	-0.5968	-0.42502	-0.28509	-0.19078	-0.49718	-0.54251	-0.7586
3.4E-19	0.000636	1.32E-13	6.34E-07	0.001159	0.031673	2.75E-09	4.45E-11	5.18E-25
-0.53631	-0.44372	-0.40957	-0.57337	-0.2914	-0.24869	-0.46464	-0.2685	-0.52532
8.11E-11	1.74E-07	1.74E-06	1.85E-12	0.000888	0.004813	3.74E-08	0.002271	2.28E-10
-0.28322	-0.13586	-0.38813	-0.20382	-0.28284	-0.27326	-0.35907	-0.46273	-0.30369
0.001253	0.127764	6.52E-06	0.021536	0.001273	0.001881	3.39E-05	4.32E-08	0.000519

IRAK3	IRF1	IRF2	IRF4	IRF5	IRF7	IRF8	ISLR	ITGA3
brown	brown	brown	brown	brown	brown	brown	brown	brown
0.019624	0.292314	0.082472	0.269792	0.311987	0.191442	0.243542	0.39199	0.084638
0.826665	0.000854	0.356637	0.002159	0.000356	0.031077	0.005796	5.17E-06	0.344102
0.080337	0.118208	-0.0313	0.127614	0.170534	0.010095	0.068427	0.326607	0.016089
0.369263	0.185631	0.726804	0.152782	0.055252	0.910313	0.444629	0.000179	0.857523
-0.30964	-0.07361	-0.08611	-0.26441	-0.43132	-0.11773	-0.38109	-0.49388	-0.1219
0.000396	0.410828	0.335755	0.002665	4.14E-07	0.187448	9.86E-06	3.63E-09	0.172166
0.048352	0.194889	0.269942	-0.04688	-0.25661	-0.14513	-0.1858	-0.30985	0.054377
0.589314	0.028115	0.002146	0.600738	0.003589	0.103523	0.036489	0.000393	0.543722
0.100821	0.14686	0.146999	-0.12524	0.188774	0.070715	0.144314	0.164291	-0.05466
0.259388	0.099434	0.09911	0.160623	0.033546	0.429511	0.105503	0.064933	0.541642
0.278999	0.355278	0.433941	-0.02239	0.031835	-0.04716	0.064097	0.013294	0.112455
0.001491	4.15E-05	3.46E-07	0.802679	0.722359	0.598522	0.474032	0.882073	0.208114
0.311915	0.263356	0.233755	0.078152	0.011971	-0.14263	-0.00024	0.094768	0.242854
0.000357	0.002776	0.008169	0.382466	0.89374	0.109665	0.997843	0.28923	0.00594
0.197488	0.16698	0.300093	-0.00545	0.243061	0.120054	0.192104	0.00516	0.020354
0.026045	0.060606	0.000609	0.951481	0.005896	0.17881	0.030488	0.954091	0.820322
0.322421	0.478701	0.326502	0.376385	0.151132	0.300666	0.075931	0.30591	0.587728
0.000218	1.25E-08	0.000179	1.29E-05	0.08987	0.000593	0.396182	0.00047	3.76E-13
0.588407	0.773064	0.63112	0.613401	0.864789	0.668733	0.757565	0.55947	0.536627
3.48E-13	1.78E-26	1.81E-15	1.77E-14	3.29E-39	8.58E-18	6.53E-25	8.08E-12	7.87E-11
0.279582	0.788214	0.632973	0.504811	0.60628	0.687705	0.547166	0.331062	0.355704
0.001456	3.97E-28	1.42E-15	1.43E-09	4.24E-14	4.24E-19	2.81E-11	0.000144	4.06E-05
-0.18512	-0.0326	-0.10121	0.172912	-0.09764	-0.04834	-0.14576	0.016483	0.07278
0.037197	0.715932	0.257522	0.051893	0.274783	0.589396	0.102032	0.854066	0.416125
-0.34168	-0.15071	-0.25527	0.057871	-0.17167	0.129736	-0.22547	-0.32446	-0.0098
8.44E-05	0.090775	0.003773	0.518109	0.053625	0.146018	0.010811	0.000198	0.912911
-0.28842	-0.04409	-0.11668	-0.01038	0.188341	0.46874	0.068153	-0.06729	-0.06817
0.001008	0.622565	0.19141	0.907761	0.033962	2.73E-08	0.446456	0.452214	0.446359
0.120593	0.337337	0.189618	0.434422	0.552689	0.653499	0.41141	0.220384	0.316648
0.176853	0.000105	0.032747	3.34E-07	1.62E-11	8.22E-17	1.55E-06	0.012784	0.000287
0.014201	-0.27365	-0.20364	0.012225	0.286865	-0.03152	0.303745	0.177439	-0.22789
0.874088	0.001852	0.021655	0.891493	0.001076	0.725017	0.000517	0.045963	0.009971
-0.59904	-0.67587	-0.65106	-0.42123	-0.49968	-0.38291	-0.37232	-0.46471	-0.5383
1.01E-13	2.84E-18	1.17E-16	8.16E-07	2.22E-09	8.87E-06	1.63E-05	3.72E-08	6.7E-11
-0.22259	-0.53466	-0.33373	-0.46527	-0.60297	-0.66304	-0.41988	-0.24403	-0.43423
0.011893	9.5E-11	0.000126	3.56E-08	6.32E-14	2.03E-17	8.92E-07	0.005695	3.39E-07
-0.27261	-0.62372	-0.55592	-0.38585	-0.41538	-0.53102	-0.31313	-0.08867	-0.39141
0.001931	4.77E-15	1.16E-11	7.46E-06	1.2E-06	1.34E-10	0.000338	0.321506	5.36E-06
-0.29112	-0.32241	-0.38881	-0.11413	-0.43207	-0.3311	-0.34055	-0.25611	-0.1768
0.000898	0.000218	6.26E-06	0.201384	3.93E-07	0.000143	8.94E-05	0.003658	0.04676

ITGAD	ITGAL	ITGAM	ITGAX	ITGB2	ITGB5	ITGB7	ITK	ITSN2
brown	brown	brown	brown	brown	brown	brown	brown	brown
0.543082	0.30149	0.144866	0.201477	0.194638	0.053531	0.254339	0.207524	0.255995
4.21E-11	0.000572	0.104163	0.023121	0.028323	0.55002	0.003908	0.019227	0.003673
0.434337	0.070923	-0.03764	0.082237	0.033764	-0.04376	0.097687	0.010886	0.086541
3.36E-07	0.428155	0.674381	0.358012	0.706289	0.625217	0.274564	0.903316	0.333321
-0.33374	-0.2344	-0.2844	-0.27421	-0.35193	-0.04598	-0.16699	0.01364	-0.38245
0.000126	0.007991	0.001193	0.00181	4.96E-05	0.607753	0.060598	0.879027	9.11E-06
-0.11583	-0.1436	-0.06352	-0.214	-0.29003	0.213539	-0.10307	0.384355	-0.03018
0.19471	0.107268	0.478044	0.015696	0.000941	0.015929	0.248871	8.15E-06	0.73625
0.137666	-0.19864	-0.20136	-0.19413	-0.05991	0.054822	-0.14506	0.060143	-0.16315
0.122725	0.025174	0.023203	0.028749	0.503424	0.540428	0.1037	0.501788	0.066848
0.100649	-0.1634	-0.03559	-0.2263	-0.17974	0.333564	-0.11575	0.479611	0.028194
0.260206	0.066419	0.691221	0.010519	0.043176	0.000127	0.195004	1.16E-08	0.753026
0.093827	-0.13764	-0.03006	-0.13011	-0.17389	0.25385	-0.12688	0.321056	-0.00509
0.294066	0.122785	0.737253	0.144852	0.050567	0.003979	0.155175	0.000233	0.954723
0.204644	0.084997	0.212565	0.034805	0.129785	0.335297	-0.00677	0.13931	0.30507
0.021005	0.342054	0.016427	0.697668	0.145864	0.000116	0.939786	0.118277	0.000488
0.136404	0.315964	0.442158	0.074204	0.360896	0.508798	0.456568	0.496735	0.069075
0.126227	0.000296	1.95E-07	0.407041	3.07E-05	1.01E-09	6.85E-08	2.85E-09	0.440315
0.681541	0.743765	0.842603	0.60349	0.859466	0.627058	0.622444	0.641108	0.497864
1.16E-18	1.29E-23	2.12E-35	5.94E-14	3.09E-38	3.09E-15	5.63E-15	4.7E-16	2.59E-09
0.55922	0.655949	0.62615	0.424227	0.550227	0.559214	0.523622	0.685402	0.407106
8.29E-12	5.76E-17	3.48E-15	6.68E-07	2.07E-11	8.29E-12	2.67E-10	6.19E-19	2.04E-06
-0.10909	0.144856	-0.06835	0.029228	0.020564	0.004725	0.357113	-0.12538	-0.36811
0.222118	0.104188	0.445144	0.744278	0.818501	0.957951	3.77E-05	0.160162	2.06E-05
-0.15364	0.179573	0.048937	0.184025	0.096749	-0.1403	0.207002	-0.25589	-0.17113
0.084595	0.04337	0.584818	0.038351	0.279221	0.11566	0.019539	0.003687	0.054398
-0.01436	0.137985	0.072386	0.203665	0.344643	-0.1539	0.219306	-0.35303	-0.19949
0.872702	0.121851	0.418663	0.02164	7.25E-05	0.084065	0.01324	4.68E-05	0.024543
0.366687	0.654279	0.621013	0.614233	0.768032	0.26884	0.608647	0.061283	0.208036
2.23E-05	7.34E-17	6.76E-15	1.59E-14	5.92E-26	0.002242	3.18E-14	0.493697	0.018925
0.251422	0.185245	0.039651	0.360981	0.200029	-0.27865	-0.03888	-0.34303	0.180068
0.004354	0.037065	0.658052	3.06E-05	0.024148	0.001513	0.664305	7.88E-05	0.042786
-0.45411	-0.33324	-0.54603	-0.17713	-0.4156	-0.63902	-0.41739	-0.69388	-0.4081
8.22E-08	0.000129	3.15E-11	0.046346	1.18E-06	6.26E-16	1.05E-06	1.52E-19	1.91E-06
-0.44785	-0.61094	-0.60372	-0.51761	-0.7182	-0.45333	-0.61389	-0.22385	-0.13374
1.3E-07	2.4E-14	5.78E-14	4.62E-10	2.02E-21	8.7E-08	1.66E-14	0.01141	0.133868
-0.36906	-0.44806	-0.5022	-0.32746	-0.46446	-0.55621	-0.49643	-0.51575	-0.15876
1.96E-05	1.28E-07	1.79E-09	0.000171	3.79E-08	1.13E-11	2.93E-09	5.46E-10	0.074623
-0.2377	-0.21591	-0.46395	-0.23829	-0.40973	-0.37138	-0.16836	-0.25511	-0.35379
0.007125	0.014772	3.94E-08	0.006981	1.72E-06	1.72E-05	0.058479	0.003797	4.5E-05

JAK2	JAK3	JCHAIN	JMY	JPH2	KCNAB3	KCNE3	KCNIP1	KCNJ5
brown	brown	brown	brown	brown	brown	brown	brown	brown
0.124787	0.331768	0.520351	-0.05667	-0.02136	-0.31438	0.06131	-0.17672	0.327708
0.16216	0.000139	3.6E-10	0.526844	0.811619	0.000319	0.493507	0.046865	0.000169
0.016084	0.179327	0.351068	-0.03439	-0.11142	-0.22249	-0.05493	-0.08467	0.193501
0.857567	0.043663	5.19E-05	0.701058	0.212377	0.011932	0.539621	0.343892	0.029277
-0.32509	-0.3454	-0.33822	0.174093	-0.18703	0.584186	0.029812	0.314828	-0.18059
0.000192	6.98E-05	0.000101	0.050288	0.035245	5.61E-13	0.739346	0.000312	0.042183
0.154821	-0.18293	-0.00993	0.318045	-0.10291	0.303567	0.239201	0.216477	-0.0717
0.082212	0.039542	0.911763	0.000269	0.249586	0.000522	0.00676	0.014505	0.423097
-0.08749	-0.03353	0.241333	-0.33999	-0.12642	-0.123	0.096551	0.048857	0.08065
0.328009	0.708199	0.00627	9.2E-05	0.15669	0.168289	0.280211	0.58543	0.367394
0.314892	-0.02372	0.317594	0.05545	-0.06801	-0.00295	0.330525	0.104024	0.050367
0.000311	0.791276	0.000274	0.535796	0.447382	0.973712	0.000148	0.24448	0.573878
0.084982	-0.0122	0.188597	-0.01774	-0.09813	-0.04409	0.282965	0.211681	0.047809
0.34214	0.891703	0.033716	0.843113	0.272371	0.622548	0.001266	0.016891	0.593506
0.460022	0.164485	0.256833	-0.05022	0.376376	-0.4055	0.109032	-0.47539	0.064952
5.3E-08	0.064613	0.003559	0.574978	1.29E-05	2.25E-06	0.22238	1.63E-08	0.468146
0.122592	0.443539	0.329442	-0.23263	0.376077	-0.08066	0.655723	-0.12157	0.00323
0.169731	1.76E-07	0.000156	0.00849	1.32E-05	0.367347	5.96E-17	0.17335	0.971251
0.51552	0.770181	0.832756	-0.48533	0.591873	-0.70852	0.735122	-0.48654	0.525953
5.57E-10	3.56E-26	6.83E-34	7.34E-09	2.33E-13	1.19E-20	7.62E-23	6.65E-09	2.15E-10
0.58812	0.579495	0.68481	-0.2821	0.374672	-0.51282	0.496054	-0.28842	0.43287
3.59E-13	9.47E-13	6.81E-19	0.001312	1.43E-05	7.1E-10	3.02E-09	0.001008	3.72E-07
-0.4077	0.005158	-0.20717	-0.14713	-0.08112	0.08746	0.017165	0.205076	-0.13202
1.96E-06	0.954108	0.01944	0.098816	0.364611	0.328197	0.848101	0.020729	0.138987
-0.48643	-0.09213	-0.34916	-0.0031	0.098308	0.311793	-0.08593	0.014988	-0.08863
6.71E-09	0.302909	5.74E-05	0.972434	0.271514	0.000359	0.336739	0.867176	0.321717
-0.43462	-0.0086	-0.13369	-0.43882	0.212394	0.024475	-0.11351	-0.18786	0.016511
3.3E-07	0.92354	0.134024	2.46E-07	0.016516	0.784748	0.203855	0.034427	0.853821
-0.073	0.464722	0.286765	-0.47728	0.563835	-0.34346	0.338965	-0.41928	0.284839
0.414739	3.71E-08	0.001081	1.4E-08	5.12E-12	7.71E-05	9.69E-05	9.28E-07	0.001171
-0.05163	0.012275	0.022303	-0.12829	0.029311	-0.29722	-0.17032	-0.08308	0.304469
0.564264	0.891053	0.80345	0.150595	0.743574	0.00069	0.05556	0.353084	0.000501
-0.55418	-0.54214	-0.74955	0.197374	-0.43515	0.450815	-0.5944	0.421271	-0.18661
1.39E-11	4.61E-11	3.78E-24	0.026133	3.18E-07	1.05E-07	1.74E-13	8.14E-07	0.035667
0.106579	-0.481	-0.44077	0.623679	-0.56487	0.351695	-0.5177	0.389205	-0.28677
0.233024	1.04E-08	2.15E-07	4.8E-15	4.6E-12	5.02E-05	4.58E-10	6.11E-06	0.00108
-0.12021	-0.33348	-0.4661	0.301499	-0.43334	0.162009	-0.62878	0.224831	-0.26103
0.178251	0.000127	3.34E-08	0.000572	3.6E-07	0.068799	2.47E-15	0.011045	0.003036
-0.65569	-0.30153	-0.33874	0.188396	-0.4585	0.586971	-0.11707	0.397415	-0.16555
5.98E-17	0.000571	9.8E-05	0.033908	5.93E-08	4.09E-13	0.189923	3.72E-06	0.062881

KCNJ6	KCNK13	KCNK6	KCNMB1	KCNQ1	KCTD12	KDSR	KIAA1211	KIAA1549
brown	brown	brown	brown	brown	brown	brown	brown	brown
-0.05637	0.137587	0.115444	0.008443	0.071336	-0.04434	0.314373	-0.32443	-0.38705
0.529055	0.122941	0.196203	0.924945	0.425462	0.620629	0.000319	0.000198	6.95E-06
0.023817	0.024273	-0.03569	-0.02367	-0.0754	-0.18978	0.269175	-0.26661	-0.33541
0.790401	0.786482	0.690379	0.791673	0.399497	0.032599	0.002212	0.002446	0.000116
0.115143	-0.22696	-0.06491	-0.18198	-0.11986	-0.08928	-0.46856	0.447661	0.49498
0.197379	0.010287	0.468432	0.040597	0.179523	0.318195	2.77E-08	1.31E-07	3.31E-09
0.116454	-0.10204	0.047767	-0.02533	-0.11301	0.205316	-0.02462	0.173338	0.171637
0.192289	0.253655	0.593833	0.777421	0.205879	0.020578	0.783522	0.051309	0.053673
0.053497	0.003199	-0.06816	0.021746	-0.06666	-0.19709	-0.08383	-0.37166	-0.46187
0.550272	0.971529	0.446393	0.80826	0.45649	0.026354	0.348727	1.69E-05	4.61E-08
0.105175	0.006626	0.081281	0.091755	-0.08492	0.168652	0.143543	-0.25062	-0.33266
0.239271	0.941064	0.363647	0.304906	0.342472	0.058037	0.107397	0.004484	0.000133
0.185097	-0.12808	0.017502	0.162419	-0.04717	0.013966	0.138553	-0.2796	-0.22425
0.037219	0.151291	0.845158	0.068091	0.598471	0.87616	0.12031	0.001455	0.011258
-0.20153	0.153944	0.13596	0.418972	0.066576	0.227939	0.326734	-0.31158	-0.23372
0.023085	0.083984	0.127478	9.47E-07	0.457066	0.009956	0.000177	0.000363	0.008179
-0.26005	0.075374	0.527485	0.406929	0.500188	0.309033	0.117798	-0.05662	-0.12227
0.003152	0.399666	1.87E-10	2.06E-06	2.13E-09	0.000408	0.187172	0.527182	0.170854
-0.49338	0.546503	0.746515	0.666151	0.792064	0.519796	0.530226	-0.62369	-0.73847
3.78E-09	3E-11	7.24E-24	1.27E-17	1.44E-28	3.79E-10	1.45E-10	4.79E-15	3.86E-23
-0.26094	0.386332	0.464157	0.444274	0.506905	0.469946	0.418703	-0.38307	-0.48513
0.003046	7.25E-06	3.88E-08	1.67E-07	1.19E-09	2.49E-08	9.64E-07	8.79E-06	7.46E-09
-0.20128	-0.12674	-0.2439	-0.09819	0.003225	-0.27463	-0.07368	0.497549	0.307834
0.02326	0.155633	0.005723	0.272079	0.971294	0.00178	0.41039	2.66E-09	0.00043
-0.16608	-0.10972	0.078246	-0.0048	0.197142	-0.10095	-0.27047	0.448107	0.51958
0.062024	0.219443	0.381895	0.957313	0.026313	0.258786	0.002102	1.27E-07	3.87E-10
-0.17659	0.051924	0.088272	0.130339	0.342407	-0.10514	-0.23818	0.052778	-0.01912
0.047027	0.562078	0.32371	0.144136	8.14E-05	0.239411	0.007006	0.555654	0.831068
-0.50839	0.291022	0.553433	0.498525	0.751999	0.24151	0.194608	-0.17076	-0.21361
1.05E-09	0.000902	1.5E-11	2.45E-09	2.23E-24	0.006231	0.028348	0.054919	0.015895
-0.14836	0.177091	-0.07523	-0.04068	0.029297	-0.06973	0.237385	-0.27631	-0.13312
0.095986	0.046398	0.400592	0.649775	0.743692	0.436013	0.007204	0.001664	0.1357
0.221637	-0.25433	-0.5414	-0.50982	-0.39748	-0.43889	-0.57015	0.533414	0.746418
0.012271	0.003909	4.96E-11	9.24E-10	3.71E-06	2.45E-07	2.62E-12	1.07E-10	7.39E-24
0.532733	-0.25863	-0.59454	-0.61004	-0.75778	-0.13976	-0.21805	0.313757	0.364446
1.14E-10	0.003325	1.71E-13	2.68E-14	6.23E-25	0.117071	0.013789	0.000328	2.53E-05
0.367487	-0.20708	-0.59005	-0.50734	-0.59994	-0.35931	-0.24048	0.226098	0.325264
2.14E-05	0.019489	2.88E-13	1.15E-09	9.08E-14	3.35E-05	0.006461	0.010588	0.000191
0.043059	-0.29925	-0.15559	-0.5044	-0.27071	-0.51365	-0.57108	0.323135	0.444078
0.630748	0.000632	0.080685	1.48E-09	0.002082	6.59E-10	2.37E-12	0.000211	1.7E-07

KIF1B	KIF21A	KIF2A	KIF5B	KIR2DL4	KLF15	KLHDC7B	KLHL42	KLHL6
brown	brown	brown	brown	brown	brown	brown	brown	brown
-0.09642	-0.27353	-0.09255	-0.04297	0.187859	-0.38105	0.332202	-0.12515	0.23155
0.280848	0.001861	0.300737	0.631421	0.03443	9.88E-06	0.000136	0.160948	0.00881
-0.02923	-0.2442	-0.05923	-0.04092	0.086669	-0.24848	0.144968	-0.0312	0.116572
0.74424	0.005662	0.508343	0.647802	0.332609	0.00485	0.103918	0.727678	0.191838
0.013804	0.096397	0.216135	0.193666	-0.10995	0.06113	-0.26034	0.305944	-0.25575
0.877584	0.280983	0.014665	0.029137	0.218488	0.494776	0.003116	0.000469	0.003707
-0.12451	-0.02748	0.129625	0.024908	-0.0713	-0.04319	-0.08596	0.28028	-0.00228
0.163105	0.759058	0.146364	0.781039	0.425723	0.629716	0.336575	0.001415	0.979693
-0.56159	-0.42756	-0.3878	-0.27336	-0.09349	-0.13829	-0.07144	-0.28437	-0.08084
6.48E-12	5.34E-07	6.65E-06	0.001873	0.295801	0.121034	0.424785	0.001195	0.366266
-0.50919	-0.37167	-0.16718	-0.22832	-0.06065	-0.2057	0.006986	0.004265	0.097498
9.77E-10	1.69E-05	0.060295	0.009829	0.498193	0.020336	0.937869	0.962046	0.275499
-0.29547	-0.2881	-0.19566	-0.32572	-0.11884	-0.17664	-0.0291	-0.04547	-0.0305
0.000745	0.001021	0.027486	0.000186	0.18329	0.046967	0.745344	0.611748	0.733522
-0.1224	-0.17047	-0.11838	-0.27788	0.050223	-0.08854	0.270968	-0.18311	0.197473
0.170398	0.05535	0.184976	0.001561	0.57497	0.322215	0.002061	0.039342	0.026057
-0.5101	-0.35538	-0.40684	-0.26483	0.24495	-0.34821	0.368191	-0.29138	0.274078
9.02E-10	4.13E-05	2.07E-06	0.002622	0.005511	6.03E-05	2.05E-05	0.000888	0.00182
-0.67493	-0.61129	-0.55779	-0.30976	0.513833	-0.46631	0.758804	-0.6202	0.784848
3.29E-18	2.3E-14	9.6E-12	0.000394	6.48E-10	3.29E-08	4.95E-25	7.5E-15	9.49E-28
-0.41887	-0.38705	-0.28953	-0.29563	0.510224	-0.35506	0.691275	-0.37197	0.572575
9.53E-07	6.95E-06	0.000961	0.00074	8.92E-10	4.2E-05	2.35E-19	1.66E-05	2.02E-12
0.181802	0.093408	0.061544	-0.087	0.094498	0.133943	-0.07244	-0.00538	-0.10664
0.040793	0.296239	0.491856	0.330741	0.290613	0.133275	0.418316	0.952167	0.232733
0.33685	0.27278	0.140159	0.257738	0.096309	0.162292	0.022254	0.044403	-0.06435
0.000108	0.001917	0.116029	0.00344	0.281425	0.06831	0.80387	0.620108	0.47226
-0.09047	0.037781	-0.32373	0.069524	0.112207	0.229178	0.106445	-0.34009	-0.04637
0.31176	0.673235	0.000205	0.437343	0.209125	0.009549	0.233615	9.16E-05	0.604714
-0.19426	-0.17945	-0.37701	-0.00788	0.413361	-0.10598	0.555381	-0.52376	0.470369
0.028635	0.043517	1.25E-05	0.929917	1.36E-06	0.235682	1.23E-11	2.64E-10	2.41E-08
0.38355	0.184797	0.203432	0.296455	-0.06203	0.124903	0.007264	0.02654	0.130336
8.54E-06	0.037534	0.021794	0.000714	0.488433	0.161766	0.935403	0.767084	0.144145
0.728876	0.609639	0.563933	0.429972	-0.3254	0.411473	-0.56998	0.406777	-0.55544
2.63E-22	2.81E-14	5.07E-12	4.54E-07	0.000189	1.54E-06	2.67E-12	2.08E-06	1.22E-11
0.489747	0.466101	0.549162	0.222968	-0.38839	0.276148	-0.58429	0.667081	-0.4743
5.11E-09	3.34E-08	2.3E-11	0.011746	6.42E-06	0.001675	5.55E-13	1.1E-17	1.77E-08
0.573066	0.43234	0.417346	0.130432	-0.31486	0.266467	-0.4904	0.374353	-0.49626
1.91E-12	3.86E-07	1.05E-06	0.143847	0.000312	0.00246	4.84E-09	1.45E-05	2.97E-09
0.110064	0.09657	0.263794	0.403565	-0.20392	-0.08768	-0.43062	0.214939	-0.33933
0.218009	0.280119	0.002729	2.54E-06	0.021475	0.32696	4.34E-07	0.015236	9.51E-05

KLKB1	KLRB1	KLRC1	KLRF1	KMO	KRT36	KYNU	LACTB	LAG3
brown	brown	brown	brown	brown	brown	brown	brown	brown
0.348058	0.255948	0.170813	0.239531	0.289771	0.408241	0.10093	0.10464	0.425931
6.08E-05	0.00368	0.05485	0.006682	0.000952	1.89E-06	0.258872	0.241683	5.96E-07
0.274962	0.086832	0.064579	0.180197	0.160994	0.323107	-0.02895	-0.00388	0.283115
0.001757	0.331696	0.470709	0.042636	0.070578	0.000211	0.746638	0.965471	0.001259
-0.20383	-0.07806	0.050722	-0.23842	-0.10423	-0.21373	0.02245	0.029104	-0.00824
0.021531	0.383044	0.571173	0.006947	0.243526	0.015834	0.802179	0.745322	0.926786
0.070069	0.261596	0.165577	-0.02197	0.227525	-0.10473	0.32699	0.301807	-0.12545
0.433747	0.00297	0.062833	0.806298	0.010095	0.24126	0.000175	0.000564	0.15993
0.178222	0.255926	0.202355	0.261071	0.073834	0.140318	0.177289	0.228229	0.09825
0.044997	0.003682	0.022517	0.003031	0.409388	0.115613	0.04615	0.009859	0.271797
0.280607	0.519739	0.333789	0.259187	0.385464	0.084545	0.504554	0.487424	-0.04392
0.001396	3.81E-10	0.000126	0.003256	7.63E-06	0.344633	1.46E-09	6.19E-09	0.623953
0.180829	0.308689	0.113938	0.243513	0.180618	0.036145	0.334182	0.300826	-0.00222
0.041901	0.000414	0.202139	0.005802	0.042145	0.686626	0.000123	0.000589	0.98024
0.408003	0.276914	0.124039	0.160991	0.298708	-0.005	0.261792	0.029707	-0.07712
1.92E-06	0.001623	0.164711	0.070582	0.000647	0.955484	0.002948	0.740231	0.388815
0.087058	0.37611	0.359893	0.065424	0.479958	0.139615	0.538804	0.362709	0.258787
0.330433	1.31E-05	3.24E-05	0.464914	1.13E-08	0.117464	6.38E-11	2.78E-05	0.003306
0.453973	0.726346	0.489287	0.479708	0.666305	0.538953	0.823298	0.607915	0.501673
8.3E-08	4.31E-22	5.31E-09	1.15E-08	1.24E-17	6.29E-11	1.55E-32	3.47E-14	1.88E-09
0.435808	0.636071	0.455434	0.328856	0.620131	0.398939	0.621214	0.435749	0.449648
3.04E-07	9.34E-16	7.45E-08	0.00016	7.57E-15	3.39E-06	6.59E-15	3.05E-07	1.14E-07
-0.14119	-0.25645	-0.16978	-0.27445	-0.13277	-0.12722	-0.34619	-0.26505	0.020075
0.113341	0.003611	0.056351	0.001793	0.136734	0.154067	6.7E-05	0.0026	0.822745
-0.29058	-0.37158	-0.1875	-0.34382	-0.26427	-0.1022	-0.22661	-0.25676	0.180156
0.000919	1.7E-05	0.034782	7.57E-05	0.002679	0.252897	0.010409	0.003569	0.042683
-0.23756	-0.3126	-0.13615	-0.1327	-0.23552	0.088161	-0.17187	-0.19708	0.189161
0.007161	0.000346	0.126954	0.136951	0.007686	0.32432	0.053347	0.026363	0.033177
0.057306	0.094065	0.109979	0.068425	0.145607	0.339122	0.269839	0.108747	0.47059
0.522207	0.292838	0.218367	0.444637	0.102386	9.61E-05	0.002155	0.2236	2.37E-08
0.141423	-0.19379	-0.24538	0.074487	-0.27444	0.177854	-0.30061	-0.25689	0.022253
0.112744	0.029035	0.005426	0.405249	0.001794	0.04545	0.000595	0.003552	0.803882
-0.46716	-0.73549	-0.50854	-0.46079	-0.72516	-0.39318	-0.78057	-0.63099	-0.26687
3.08E-08	7.07E-23	1.03E-09	5E-08	5.41E-22	4.82E-06	2.81E-27	1.84E-15	0.002422
-0.22165	-0.31886	-0.29175	-0.15294	-0.29165	-0.37445	-0.48393	-0.29172	-0.57565
0.012266	0.000259	0.000875	0.086046	0.000878	1.44E-05	8.22E-09	0.000875	1.45E-12
-0.26951	-0.5287	-0.388	-0.20513	-0.46056	-0.33487	-0.69482	-0.52885	-0.40948
0.002183	1.67E-10	6.57E-06	0.020695	5.09E-08	0.000119	1.29E-19	1.64E-10	1.75E-06
-0.27485	-0.22605	-0.13412	-0.23192	-0.3692	-0.11015	-0.25474	0.021999	0.15335
0.001765	0.010605	0.132761	0.008698	1.94E-05	0.217638	0.003849	0.806077	0.0852

LAIR1	LAMP1	LAMP3	LAPTM5	LARS2	LAT2	LAX1	LAYN	LCK
brown	brown	brown	brown	brown	brown	brown	brown	brown
0.266974	0.056272	0.248729	0.258993	-0.2351	0.181236	0.289674	0.311116	0.342789
0.002412	0.529755	0.004806	0.00328	0.007799	0.041435	0.000956	0.000371	7.98E-05
0.11856	-0.05284	0.091635	0.128974	-0.14218	0.050596	0.118928	0.253016	0.155485
0.184315	0.555165	0.30554	0.14842	0.110807	0.572132	0.182948	0.004105	0.080892
-0.39316	-0.25338	0.0199	-0.29954	0.34841	-0.36292	-0.10882	-0.34887	-0.20372
4.82E-06	0.004049	0.824263	0.000624	5.97E-05	2.75E-05	0.223294	5.83E-05	0.021602
-0.17283	-0.18718	0.373277	-0.07486	0.153635	-0.14183	0.143274	-0.05727	-0.01869
0.052003	0.035098	1.54E-05	0.402885	0.084615	0.111705	0.108065	0.522464	0.834762
0.060443	-0.39066	0.056642	0.057854	-0.24251	0.245995	-0.07855	0.358746	0.19096
0.499651	5.61E-06	0.527045	0.518226	0.006013	0.005307	0.380053	3.45E-05	0.03151
0.052531	-0.30927	0.470389	0.118131	-0.12765	0.161563	0.18129	0.33599	0.215785
0.557506	0.000403	2.4E-08	0.185922	0.152664	0.069576	0.041373	0.000113	0.01483
0.030871	-0.17379	0.330774	0.068006	-0.00355	0.07492	0.11462	0.294965	0.147417
0.730443	0.050702	0.000146	0.447439	0.968415	0.402518	0.199435	0.000761	0.098143
0.266654	0.076303	0.152157	0.247263	-0.08528	0.322738	0.149705	0.253196	0.174273
0.002442	0.393866	0.087688	0.005069	0.340422	0.000215	0.092979	0.004077	0.050047
0.344022	0.396282	0.549376	0.393629	-0.21746	0.056724	0.509043	0.227742	0.429161
7.49E-05	3.99E-06	2.26E-11	4.69E-06	0.014054	0.526448	9.9E-10	0.010022	4.79E-07
0.935301	0.637723	0.655634	0.931666	-0.53572	0.767075	0.676196	0.628532	0.790619
3.05E-58	7.47E-16	6.03E-17	8.29E-57	8.59E-11	7.41E-26	2.7E-18	2.55E-15	2.11E-28
0.629947	0.416746	0.682251	0.655722	-0.24513	0.620316	0.669192	0.387084	0.634595
2.12E-15	1.1E-06	1.03E-18	5.96E-17	0.005475	7.39E-15	8E-18	6.94E-06	1.14E-15
-0.11602	0.257882	-0.03706	-0.08389	0.073622	-0.18453	0.16956	-0.23283	-0.09619
0.193968	0.003421	0.679135	0.348389	0.410743	0.037815	0.05668	0.008433	0.282018
-0.13846	0.315251	-0.23288	-0.11804	0.254365	-0.29962	-0.00939	-0.43488	-0.16169
0.120549	0.000306	0.008419	0.186266	0.003904	0.000621	0.916538	3.24E-07	0.069352
0.073212	0.241703	-0.35731	0.047568	-0.10456	0.073025	-0.07441	-0.10274	0.012006
0.413361	0.006188	3.73E-05	0.595371	0.242063	0.414557	0.405707	0.250379	0.893426
0.567368	0.760398	0.094596	0.560428	-0.26442	0.356325	0.389281	0.164467	0.398369
3.53E-12	3.45E-25	0.290111	7.31E-12	0.002664	3.93E-05	6.09E-06	0.064642	3.51E-06
0.140609	0.228584	-0.31615	0.119456	-0.09775	0.235163	-0.22108	0.170385	-0.07919
0.114851	0.009742	0.000294	0.180999	0.274265	0.007782	0.012498	0.055469	0.376168
-0.5988	-0.25083	-0.67896	-0.59416	0.471202	-0.43433	-0.635	-0.62021	-0.65722
1.04E-13	0.00445	1.74E-18	1.79E-13	2.26E-08	3.37E-07	1.08E-15	7.49E-15	4.79E-17
-0.62496	-0.62907	-0.28931	-0.63764	0.309186	-0.42096	-0.47033	-0.34393	-0.54028
4.07E-15	2.38E-15	0.00097	7.55E-16	0.000405	8.31E-07	2.41E-08	7.53E-05	5.53E-11
-0.46995	-0.42836	-0.54698	-0.54552	0.255668	-0.33713	-0.55448	-0.34108	-0.51764
2.49E-08	5.06E-07	2.86E-11	3.31E-11	0.003718	0.000106	1.35E-11	8.71E-05	4.61E-10
-0.44972	-0.37128	-0.21094	-0.41795	0.188588	-0.51314	-0.27216	-0.24542	-0.25583
1.13E-07	1.73E-05	0.017287	1.01E-06	0.033724	6.89E-10	0.001965	0.005418	0.003696

LCP1	LCP2	LEFTY1	LEP	LGALS2	LGALS3	LGALS3BP	LGALS9	LGALSL
brown	brown	brown	brown	brown	brown	brown	brown	brown
0.227316	0.307414	-0.12788	0.226739	0.313564	0.279049	0.229543	0.291213	-0.09029
0.010166	0.000439	0.151937	0.010364	0.000331	0.001488	0.009432	0.000895	0.312709
0.077567	0.138213	-0.06255	0.173064	0.172131	0.249015	0.09966	0.106695	0.03057
0.386054	0.12123	0.48478	0.051684	0.052977	0.004756	0.264945	0.232511	0.732967
-0.26207	-0.28458	0.296566	-0.15031	-0.15666	-0.11485	-0.13765	-0.34985	0.159419
0.002917	0.001184	0.00071	0.091645	0.078596	0.198523	0.122759	5.54E-05	0.073412
-0.00489	-0.00764	0.071102	-0.0153	-0.09763	0.248612	-0.10259	-0.34957	0.159722
0.956515	0.932092	0.426984	0.864419	0.274831	0.004826	0.251098	5.62E-05	0.072858
0.079985	0.021781	-0.0585	0.120673	0.234834	0.296496	-0.12121	-0.06081	0.110914
0.37137	0.80796	0.513595	0.176564	0.007871	0.000712	0.174631	0.49704	0.214454
0.179666	0.182642	-0.13164	0.145382	0.141845	0.498802	-0.11139	-0.24741	0.144644
0.04326	0.039856	0.140153	0.102921	0.111662	2.4E-09	0.212462	0.005042	0.104699
0.135351	0.114555	-0.11108	0.163394	0.141382	0.387648	-0.02538	-0.17856	0.208679
0.129205	0.19969	0.213745	0.066431	0.112849	6.71E-06	0.776966	0.044591	0.018552
0.164027	0.286313	-0.41851	-0.01648	0.034337	0.101812	-0.01241	0.044573	-0.2293
0.06537	0.001101	9.76E-07	0.854077	0.701535	0.254711	0.88983	0.618769	0.00951
0.471475	0.48502	-0.17949	0.405563	0.075636	0.377604	0.55594	0.227708	-0.25842
2.21E-08	7.53E-09	0.043463	2.24E-06	0.398023	1.21E-05	1.16E-11	0.010033	0.003352
0.901815	0.917266	-0.53619	0.540609	0.51402	0.517584	0.741915	0.783294	-0.61667
2.23E-47	8.17E-52	8.21E-11	5.36E-11	6.37E-10	4.63E-10	1.9E-23	1.41E-27	1.17E-14
0.628726	0.737711	-0.35299	0.379245	0.475981	0.457453	0.618696	0.596035	-0.46015
2.49E-15	4.51E-23	4.69E-05	1.1E-05	1.55E-08	6.41E-08	9.08E-15	1.44E-13	5.25E-08
-0.02621	-0.16554	0.326164	0.089772	-0.29336	-0.10229	0.287716	0.102135	0.03243
0.769876	0.062898	0.000182	0.315519	0.000816	0.252472	0.001038	0.253198	0.717392
-0.13481	-0.18519	0.30244	-0.06307	-0.22989	-0.36024	0.27085	0.137329	-0.2156
0.130753	0.037127	0.000548	0.481137	0.009321	3.18E-05	0.002071	0.123651	0.014918
-0.06886	-0.06359	0.193055	0.053532	0.027501	-0.18409	0.351779	0.330103	-0.37798
0.441736	0.477548	0.02966	0.550015	0.758907	0.038278	5E-05	0.000151	1.18E-05
0.46457	0.463247	-0.13569	0.319874	0.168792	0.017402	0.781626	0.741735	-0.68867
3.76E-08	4.15E-08	0.12825	0.000246	0.057826	0.846034	2.16E-27	1.97E-23	3.62E-19
-0.00712	-0.03626	-0.03702	-0.14441	0.007225	-0.2425	0.025391	0.325862	-0.06406
0.93668	0.685695	0.679491	0.105276	0.935748	0.006016	0.776901	0.000185	0.474256
-0.63808	-0.67327	0.435269	-0.53702	-0.30669	-0.6686	-0.44904	-0.27917	0.341684
7.11E-16	4.26E-18	3.15E-07	7.58E-11	0.000453	8.75E-18	1.19E-07	0.001481	8.44E-05
-0.59909	-0.53592	0.165806	-0.441	-0.21686	-0.23604	-0.81237	-0.68655	0.673579
1E-13	8.43E-11	0.062465	2.11E-07	0.014328	0.007549	4.6E-31	5.13E-19	4.07E-18
-0.53532	-0.52153	0.130678	-0.39699	-0.19618	-0.44345	-0.64328	-0.39013	0.498522
8.92E-11	3.24E-10	0.143088	3.82E-06	0.027067	1.78E-07	3.48E-16	5.78E-06	2.45E-09
-0.25527	-0.45489	0.491708	-0.16338	-0.12994	-0.32773	-0.27583	-0.27096	0.408526
0.003775	7.76E-08	4.35E-09	0.066461	0.145373	0.000169	0.001696	0.002062	1.86E-06

LGMN	LILRA1	LILRA2	LILRA5	LILRA6	LILRB1	LILRB2	LILRB3	LILRB4
brown	brown	brown	brown	brown	brown	brown	brown	brown
0.430485	0.155603	0.200356	0.111535	0.18352	0.2493	0.355047	0.227093	0.265473
4.38E-07	0.080659	0.023913	0.211883	0.038896	0.004707	4.21E-05	0.010242	0.002557
0.244622	-0.00965	0.135972	0.016141	0.115604	0.047719	0.209782	0.128578	0.140044
0.005576	0.914263	0.127443	0.857061	0.195577	0.594207	0.017926	0.149682	0.116331
-0.34562	-0.21677	-0.27683	-0.08797	-0.07764	-0.29403	-0.19255	-0.1133	-0.36767
6.9E-05	0.014369	0.001629	0.325371	0.385607	0.000793	0.030095	0.204711	2.12E-05
-0.24119	0.019287	-0.15612	0.019752	0.169769	-0.15914	-0.0207	0.012527	-0.31069
0.006302	0.829591	0.079649	0.825551	0.05637	0.073928	0.817278	0.888833	0.000378
-0.0346	-0.03776	0.270922	0.247301	0.118446	-0.10655	0.087382	0.145608	0.062648
0.699328	0.67337	0.002065	0.005062	0.18474	0.233151	0.328627	0.102383	0.484108
-0.09589	0.108428	0.108723	0.244358	0.307963	-0.08267	0.145252	0.17104	-0.12101
0.283521	0.224968	0.223704	0.005629	0.000428	0.355478	0.103233	0.054523	0.175335
-0.10776	0.052494	0.066715	0.219552	0.337774	-0.10569	0.16994	0.162676	-0.07772
0.227867	0.557786	0.456125	0.013135	0.000103	0.236977	0.05612	0.067651	0.385094
-0.01049	0.332144	0.209335	-0.04351	0.166891	0.202135	0.127525	0.043302	0.125171
0.906825	0.000136	0.018177	0.627198	0.060745	0.022667	0.153072	0.62882	0.160863
0.346157	0.213187	0.017019	0.440036	0.491984	0.333949	0.510425	0.507214	0.115174
6.71E-05	0.016107	0.84938	2.26E-07	4.25E-09	0.000125	8.77E-10	1.16E-09	0.197256
0.8045	0.704056	0.627773	0.591651	0.747501	0.844899	0.863506	0.783506	0.785366
4.61E-30	2.63E-20	2.82E-15	2.4E-13	5.87E-24	9.14E-36	5.69E-39	1.34E-27	8.31E-28
0.611186	0.66396	0.483442	0.398178	0.540139	0.678651	0.630416	0.479459	0.499383
2.32E-14	1.77E-17	8.55E-09	3.55E-06	5.61E-11	1.83E-18	1.99E-15	1.18E-08	2.28E-09
0.102752	-0.08455	-0.13666	-0.17574	-0.13612	-0.08655	-0.10138	-0.09336	-0.09231
0.250327	0.34459	0.125519	0.048114	0.127013	0.33326	0.256749	0.296496	0.301979
-0.01691	-0.05051	-0.14678	-0.1721	-0.08953	0.061691	-0.04798	0.000715	-0.00149
0.850309	0.572775	0.099623	0.053017	0.316839	0.49082	0.592201	0.993638	0.986708
0.105323	-0.00202	0.20781	0.090778	-0.00247	0.189117	0.052944	0.149247	0.255706
0.238605	0.981998	0.019058	0.310107	0.977977	0.03322	0.554413	0.093995	0.003713
0.564325	0.432512	0.411648	0.257182	0.391887	0.670254	0.523994	0.517515	0.635384
4.87E-12	3.81E-07	1.52E-06	0.003513	5.21E-06	6.8E-18	2.58E-10	4.66E-10	1.02E-15
0.200735	0.106096	0.293825	-0.28334	-0.12525	0.140119	-0.08233	-0.13957	0.367312
0.023643	0.235158	0.0008	0.001247	0.160603	0.116133	0.357489	0.117577	2.16E-05
-0.41447	-0.46355	-0.37355	-0.52798	-0.65441	-0.44254	-0.64514	-0.63918	-0.31411
1.27E-06	4.06E-08	1.52E-05	1.78E-10	7.21E-17	1.89E-07	2.69E-16	6.13E-16	0.000323
-0.53815	-0.48479	-0.49936	-0.39992	-0.56383	-0.64844	-0.6488	-0.66224	-0.62777
6.8E-11	7.67E-09	2.29E-09	3.19E-06	5.13E-12	1.69E-16	1.61E-16	2.29E-17	2.82E-15
-0.36689	-0.46472	-0.3935	-0.39999	-0.63525	-0.4844	-0.58512	-0.61976	-0.37153
2.21E-05	3.71E-08	4.72E-06	3.18E-06	1.04E-15	7.92E-09	5.05E-13	7.93E-15	1.7E-05
-0.24276	-0.37184	-0.32553	-0.12801	-0.29322	-0.38942	-0.22931	-0.14326	-0.31198
0.00596	1.67E-05	0.000188	0.151506	0.000821	6.04E-06	0.009506	0.10811	0.000356

LILRB5	LIMK2	LIPA	LITAF	LMO2	LNP1	LPAR5	LPCAT2	LPXN
brown	brown	brown	brown	brown	brown	brown	brown	brown
0.433125	0.20677	0.444411	0.266859	0.018261	0.112109	0.163599	0.086246	0.289565
3.66E-07	0.01968	1.66E-07	0.002423	0.83853	0.209523	0.066085	0.334978	0.00096
0.380144	0.09924	0.372286	0.17342	0.003005	0.189903	0.017723	-0.03942	0.209914
1.04E-05	0.266971	1.63E-05	0.051197	0.973248	0.032482	0.843227	0.659897	0.017852
-0.36404	-0.26624	-0.36004	-0.05679	-0.02474	0.060231	-0.34219	-0.26702	-0.1644
2.58E-05	0.002482	3.21E-05	0.525995	0.782461	0.501157	8.23E-05	0.002408	0.064756
-0.17264	-0.1772	-0.09194	0.150658	0.287407	-0.03969	-0.23405	0.057304	0.212359
0.052264	0.046263	0.303937	0.090892	0.001052	0.657737	0.008086	0.522225	0.016534
0.149587	-0.25944	0.090033	0.139369	0.167791	0.269485	-0.09481	-0.00729	0.326889
0.093239	0.003225	0.31411	0.118119	0.059349	0.002185	0.289042	0.935141	0.000176
0.083407	-0.20436	0.142145	0.308608	0.418275	0.086778	-0.1472	0.207449	0.520613
0.351192	0.021189	0.1109	0.000416	9.91E-07	0.331996	0.098635	0.019272	3.52E-10
0.190529	-0.11319	0.095563	0.181643	0.451992	0.150913	-0.18095	0.002211	0.419215
0.031904	0.205135	0.285188	0.040973	9.59E-08	0.09034	0.041763	0.980317	9.32E-07
0.110299	0.270648	0.11665	0.193892	0.188641	0.04892	0.182301	0.315808	0.357603
0.217023	0.002087	0.191538	0.028946	0.033673	0.584945	0.040234	0.000298	3.67E-05
0.26345	0.27719	0.024268	0.394756	0.142335	-0.5387	0.051248	0.011502	0.320375
0.002766	0.001605	0.786529	4.38E-06	0.11042	6.45E-11	0.567182	0.897873	0.000241
0.657757	0.593422	0.629435	0.80241	0.409071	-0.58638	0.669642	0.525355	0.824388
4.43E-17	1.95E-13	2.26E-15	8.36E-30	1.8E-06	4.38E-13	7.47E-18	2.28E-10	1.1E-32
0.41949	0.495076	0.441204	0.625323	0.42371	-0.40207	0.538142	0.483378	0.655432
9.15E-07	3.28E-09	2.08E-07	3.88E-15	6.92E-07	2.79E-06	6.8E-11	8.6E-09	6.21E-17
-0.18216	0.044775	-0.20422	-0.09173	-0.18953	-0.17879	-0.04527	-0.30734	-0.24754
0.040388	0.617182	0.021275	0.305046	0.032834	0.044309	0.613325	0.00044	0.005018
-0.18777	0.171697	-0.27632	-0.07549	-0.26346	-0.22857	8.15E-06	-0.33934	-0.3462
0.03452	0.053588	0.001663	0.398948	0.002765	0.009745	0.999927	9.51E-05	6.69E-05
0.009502	0.161388	-0.14845	0.008679	-0.256	-0.24583	0.15682	-0.21766	-0.21733
0.915564	0.069882	0.095786	0.922853	0.003673	0.005338	0.078288	0.013963	0.014112
0.33872	0.599219	0.243685	0.477972	0.001859	-0.61912	0.533375	0.097344	0.229884
9.81E-05	9.89E-14	0.005766	1.33E-08	0.983447	8.6E-15	1.07E-10	0.276264	0.009323
0.152065	0.183711	0.41672	0.048566	0.015976	0.048901	0.364393	0.229996	-0.06486
0.087882	0.03869	1.1E-06	0.587671	0.858508	0.585091	2.54E-05	0.009288	0.468762
-0.51072	-0.30534	-0.40007	-0.60838	-0.34794	0.340225	-0.22669	-0.32962	-0.7796
8.54E-10	0.000482	3.16E-06	3.28E-14	6.12E-05	9.09E-05	0.010383	0.000154	3.58E-27
-0.41465	-0.5417	-0.2477	-0.65583	-0.16888	0.534075	-0.45008	-0.04963	-0.50443
1.26E-06	4.82E-11	0.00499	5.86E-17	0.057699	1E-10	1.1E-07	0.579531	1.48E-09
-0.30751	-0.34301	-0.2567	-0.72041	-0.36316	0.517512	-0.27102	-0.18538	-0.63297
0.000437	7.89E-05	0.003577	1.34E-21	2.71E-05	4.66E-10	0.002056	0.03692	1.42E-15
-0.25874	-0.52506	-0.30044	-0.24934	-0.27422	0.366569	-0.42145	-0.49752	-0.28738
0.003312	2.34E-10	0.000599	0.0047	0.00181	2.25E-05	8.04E-07	2.67E-09	0.001053

LRAT	LRCH2	LRG1	LRMP	LRP4	LRP8	LRRC25	LRRC47	LRRC75A
brown	brown	brown	brown	brown	brown	brown	brown	brown
0.254712	-0.23354	-0.05271	0.450333	-0.19784	-0.36326	0.165093	-0.0905	-0.1781
0.003854	0.00823	0.556138	1.08E-07	0.025775	2.7E-05	0.063616	0.311602	0.045152
0.236439	-0.19432	-0.12916	0.37984	-0.1141	-0.23825	0.006115	0.032867	-0.12867
0.007445	0.028591	0.147821	1.06E-05	0.201477	0.006989	0.945602	0.713746	0.149379
-0.41136	0.354376	-0.18441	-0.61358	0.26377	0.336848	-0.19322	0.008143	0.414601
1.55E-06	4.36E-05	0.037946	1.73E-14	0.002732	0.000108	0.029519	0.927606	1.26E-06
-0.14145	0.317753	-0.2079	-0.31774	0.149924	0.101735	-0.05213	-0.36151	0.290266
0.11268	0.000272	0.019006	0.000273	0.092497	0.255074	0.560492	2.97E-05	0.000932
0.115465	0.122114	0.131484	0.32047	-0.33952	-0.23247	0.015548	-0.03095	-0.07742
0.196118	0.171415	0.140616	0.00024	9.42E-05	0.008537	0.862265	0.729811	0.38693
0.100839	0.274022	-0.04728	0.146965	-0.19285	-0.19147	0.066046	-0.44002	0.064278
0.259304	0.001824	0.597569	0.099189	0.029839	0.031054	0.460668	2.26E-07	0.472782
-0.01366	-0.01376	0.026992	0.178585	-0.06962	0.036203	0.003674	-0.36382	0.008258
0.878853	0.877988	0.763236	0.044556	0.436713	0.686153	0.967297	2.62E-05	0.926586
0.26138	-0.07933	-0.08298	0.215245	-0.34167	-0.22031	0.136355	-0.19641	-0.20496
0.002995	0.375291	0.353691	0.015088	8.45E-05	0.012815	0.126363	0.02689	0.020801
-0.00933	-0.35724	0.328984	-0.24212	-0.2577	-0.2804	0.548344	-0.32611	-0.28777
0.917115	3.74E-05	0.000159	0.006096	0.003445	0.001408	2.5E-11	0.000183	0.001036
0.610424	-0.601	0.552384	0.503637	-0.58464	-0.72181	0.887424	-0.44875	-0.66074
2.55E-14	8E-14	1.67E-11	1.58E-09	5.33E-13	1.03E-21	7.26E-44	1.21E-07	2.86E-17
0.419035	-0.3592	0.218985	0.34676	-0.34394	-0.55415	0.577637	-0.41186	-0.28316
9.43E-07	3.37E-05	0.013378	6.5E-05	7.52E-05	1.39E-11	1.16E-12	1.5E-06	0.001256
-0.1556	-0.24026	-0.07594	-0.26428	0.233893	0.089186	-0.0347	0.271913	-0.00731
0.080667	0.006511	0.396093	0.002679	0.008131	0.318703	0.698518	0.001985	0.934986
-0.24072	-0.18779	0.007291	-0.61966	0.303079	0.281288	0.0393	0.296143	0.089777
0.006407	0.0345	0.935157	8.03E-15	0.000533	0.001357	0.660893	0.000723	0.315496
0.047025	-0.22211	0.292241	-0.30689	-0.05374	-0.11973	0.228863	0.391292	-0.12964
0.599583	0.012084	0.000856	0.000449	0.548473	0.179999	0.009651	5.4E-06	0.146332
0.322684	-0.62467	0.449909	-0.08677	-0.21342	-0.35942	0.665214	0.059658	-0.47463
0.000216	4.22E-15	1.12E-07	0.332017	0.01599	3.33E-05	1.46E-17	0.505244	1.73E-08
0.295373	-0.1914	-0.06423	0.427491	0.065098	-0.06935	-0.08222	0.280913	-0.17821
0.000748	0.031118	0.47309	5.37E-07	0.467142	0.438511	0.358129	0.001379	0.045012
-0.43	0.310411	-0.31625	-0.33379	0.532918	0.587069	-0.61334	0.531748	0.465375
4.53E-07	0.000383	0.000292	0.000126	1.12E-10	4.05E-13	1.78E-14	1.25E-10	3.53E-08
-0.33335	0.632092	-0.46669	0.04826	0.350803	0.415853	-0.73455	0.048347	0.518089
0.000128	1.59E-15	3.19E-08	0.590025	5.27E-05	1.16E-06	8.55E-23	0.589351	4.42E-10
-0.2915	0.28156	-0.28931	0.167766	0.260795	0.421756	-0.64118	0.332687	0.266668
0.000884	0.001342	0.00097	0.059387	0.003063	7.88E-07	4.65E-16	0.000133	0.002441
-0.50994	0.257549	-0.09778	-0.30944	0.201845	0.412005	-0.34828	0.225927	0.240115
9.15E-10	0.003464	0.274102	0.0004	0.022866	1.49E-06	6.01E-05	0.010649	0.006546

LRRFIP1	LRRFIP2	LRRTM2	LSP1	LST1	LTA4H	LTB	LTBR	LTF
brown	brown	brown	brown	brown	brown	brown	brown	brown
0.128895	0.243436	-0.15646	0.135362	0.319662	0.298558	0.295434	0.054374	0.039712
0.148671	0.005818	0.078981	0.129176	0.000249	0.000651	0.000746	0.543747	0.657561
-0.03809	0.191982	-0.07998	0.025845	0.224613	0.214764	0.107833	-0.04358	-0.04685
0.670715	0.030596	0.371431	0.773022	0.011125	0.015321	0.227537	0.626627	0.600931
-0.19355	-0.27491	0.180863	-0.15484	-0.36493	-0.17695	-0.30854	-0.24431	-0.10931
0.029235	0.001761	0.041863	0.082182	2.46E-05	0.046574	0.000417	0.005638	0.221199
0.01516	-0.0975	0.045892	-0.13865	-0.197	0.124651	-0.292	-0.2648	0.200483
0.86567	0.275483	0.60842	0.120061	0.026423	0.162623	0.000865	0.002626	0.023822
-0.29413	-0.05675	-0.3216	0.03807	0.553213	0.279058	0.119871	-0.08269	0.067706
0.000789	0.526271	0.000227	0.67088	1.53E-11	0.001488	0.179477	0.35538	0.449448
-0.04837	-0.01938	-0.24265	-0.06037	0.294799	0.403172	-0.10428	-0.20416	0.313506
0.589174	0.828765	0.005983	0.500144	0.000767	2.61E-06	0.243325	0.021318	0.000332
0.003987	0.053186	-0.04217	-0.11527	0.246983	0.214419	-0.1072	-0.05724	0.155993
0.964514	0.5526	0.637795	0.19687	0.005121	0.01549	0.230279	0.522706	0.079893
0.157756	0.351691	-0.41642	0.008619	0.217795	0.23927	0.032241	0.012358	0.420092
0.076502	5.02E-05	1.12E-06	0.92338	0.013903	0.006744	0.718964	0.890322	8.8E-07
0.592826	0.104817	-0.28238	0.483919	0.048753	0.237416	0.281584	0.530397	0.410455
2.09E-13	0.240885	0.001297	8.23E-09	0.586226	0.007196	0.001341	1.42E-10	1.64E-06
0.618487	0.511061	-0.72231	0.637408	0.77967	0.632299	0.706732	0.650491	0.655654
9.32E-15	8.29E-10	9.34E-22	7.79E-16	3.52E-27	1.55E-15	1.64E-20	1.26E-16	6.02E-17
0.453464	0.346797	-0.506	0.338011	0.519588	0.494763	0.497353	0.344455	0.581169
8.61E-08	6.49E-05	1.29E-09	0.000102	3.86E-10	3.37E-09	2.71E-09	7.32E-05	7.86E-13
-0.00384	-0.02513	0.247205	0.092677	-0.24134	-0.23433	0.001127	0.138574	0.003822
0.965806	0.779124	0.00508	0.300053	0.006269	0.00801	0.989965	0.120253	0.96598
0.114474	0.035132	0.160884	0.111602	-0.44678	-0.29718	0.017426	0.19534	-0.14131
0.200011	0.694968	0.070772	0.211604	1.4E-07	0.000691	0.845822	0.027746	0.113023
-0.06047	-0.02	-0.26246	0.371455	0.056932	-0.16647	0.366257	0.337895	-0.06607
0.499457	0.823361	0.002874	1.71E-05	0.524933	0.061406	2.29E-05	0.000102	0.460515
0.473346	0.419074	-0.45552	0.614913	0.240641	0.168632	0.610646	0.710342	0.29552
1.91E-08	9.41E-07	7.4E-08	1.46E-14	0.006426	0.058067	2.48E-14	8.58E-21	0.000743
-0.20156	0.333553	0.094978	-0.05271	0.166104	-0.08086	0.108426	0.013543	-0.20961
0.023066	0.000127	0.288161	0.55618	0.061988	0.36616	0.224979	0.879878	0.018021
-0.52402	-0.32474	0.665919	-0.39415	-0.5145	-0.66513	-0.40243	-0.37036	-0.61421
2.58E-10	0.000195	1.32E-17	4.54E-06	6.11E-10	1.48E-17	2.73E-06	1.82E-05	1.6E-14
-0.45145	-0.51425	0.618267	-0.62783	-0.44721	-0.36374	-0.60851	-0.70397	-0.46205
9.98E-08	6.24E-10	9.59E-15	2.8E-15	1.36E-07	2.63E-05	3.23E-14	2.67E-20	4.55E-08
-0.39478	-0.3597	0.565644	-0.48055	-0.32037	-0.47637	-0.40144	-0.43607	-0.55061
4.37E-06	3.28E-05	4.23E-12	1.08E-08	0.000241	1.5E-08	2.9E-06	2.98E-07	1.99E-11
-0.25569	-0.12524	0.406752	-0.25152	-0.32584	-0.22326	-0.24046	-0.24497	-0.5271
0.003715	0.160642	2.08E-06	0.004338	0.000185	0.011633	0.006467	0.005506	1.94E-10

LY86	LY9	LY96	LYN	LYSMD1	LYVE1	LYZ	M1AP	M6PR
brown	brown	brown	brown	brown	brown	brown	brown	brown
0.438156	0.379136	0.311715	0.249961	-0.18132	0.021014	0.237556	0.373058	0.241605
2.58E-07	1.1E-05	0.000361	0.004594	0.04134	0.814598	0.007161	1.56E-05	0.00621
0.339071	0.203893	0.194776	0.07149	-0.02975	0.018057	0.055074	0.33451	0.178048
9.64E-05	0.021491	0.028209	0.424457	0.739854	0.840311	0.538569	0.000121	0.045211
-0.54479	-0.10611	-0.09606	-0.25632	0.277608	-0.33239	-0.16428	-0.52226	-0.23093
3.56E-11	0.235082	0.282693	0.003629	0.001578	0.000135	0.064946	3.03E-10	0.008997
-0.30802	0.11864	0.268113	0.08625	0.238254	-0.05442	0.138799	-0.29833	0.102901
0.000427	0.184019	0.002307	0.334956	0.006988	0.543444	0.119645	0.000657	0.249638
0.291441	0.223381	0.46448	-0.04134	0.478762	0.150218	0.121579	0.336887	-0.01051
0.000886	0.011587	3.78E-08	0.644463	1.24E-08	0.091852	0.173313	0.000108	0.906614
0.103015	0.313271	0.645948	0.221322	0.40035	0.216021	0.328682	0.120657	0.226614
0.24911	0.000336	2.4E-16	0.012398	3.11E-06	0.014718	0.000161	0.176621	0.010408
0.102555	0.223508	0.390432	0.207812	0.260097	0.188389	0.151299	0.121754	0.281749
0.251245	0.011539	5.68E-06	0.019056	0.003145	0.033915	0.08951	0.172691	0.001332
0.274682	0.098676	0.287108	0.252104	0.017026	0.058989	0.20211	0.221262	0.119831
0.001777	0.269715	0.001065	0.004246	0.849319	0.51004	0.022684	0.012423	0.179623
0.01177	0.358397	0.443764	0.42714	-0.36293	0.385178	0.544908	-0.31177	0.409168
0.895507	3.51E-05	1.74E-07	5.5E-07	2.75E-05	7.76E-06	3.52E-11	0.00036	1.79E-06
0.829521	0.690619	0.821886	0.843242	-0.58269	0.485181	0.85918	0.406375	0.647981
2.03E-33	2.62E-19	2.44E-32	1.68E-35	6.63E-13	7.43E-09	3.48E-38	2.13E-06	1.8E-16
0.567656	0.586174	0.665571	0.743766	-0.44415	0.182685	0.678922	0.213422	0.585009
3.42E-12	4.48E-13	1.39E-17	1.29E-23	1.69E-07	0.039808	1.76E-18	0.015988	5.11E-13
-0.17767	-0.07564	-0.29715	-0.12769	-0.14088	-0.13697	-0.06129	-0.32847	0.296479
0.045673	0.398025	0.000692	0.152532	0.114144	0.124642	0.493654	0.000163	0.000713
-0.3686	-0.17036	-0.46461	-0.20448	-0.31967	-0.37376	-0.20873	-0.52756	-0.18627
2.01E-05	0.055501	3.75E-08	0.021113	0.000249	1.5E-05	0.018521	1.86E-10	0.036009
-0.00863	-0.09986	-0.21208	-0.18169	-0.20575	-0.20945	-0.04978	-0.195	-0.27705
0.92328	0.263998	0.016681	0.040922	0.020306	0.018112	0.578318	0.028021	0.001614
0.367156	0.243969	0.115421	0.350246	-0.71878	0.041896	0.382393	-0.06092	0.208907
2.18E-05	0.005708	0.19629	5.42E-05	1.82E-21	0.640013	9.14E-06	0.496257	0.018421
0.412065	-0.13784	-0.27282	-0.05553	-0.22043	-0.14572	-0.17405	0.479187	-0.0532
1.48E-06	0.122243	0.001915	0.535197	0.012765	0.102111	0.050342	1.2E-08	0.552465
-0.48662	-0.60509	-0.86551	-0.62503	0.178598	-0.56464	-0.74267	-0.26474	-0.57345
6.61E-09	4.9E-14	2.41E-39	4.03E-15	0.04454	4.71E-12	1.63E-23	0.002631	1.84E-12
-0.45558	-0.43665	-0.42103	-0.41353	0.509272	-0.14346	-0.51214	0.042273	-0.33035
7.37E-08	2.86E-07	8.26E-07	1.35E-06	9.7E-10	0.107605	7.54E-10	0.637005	0.000149
-0.25425	-0.50136	-0.63322	-0.45559	0.294531	-0.13595	-0.61676	0.133272	-0.37362
0.003921	1.93E-09	1.37E-15	7.36E-08	0.000776	0.127491	1.16E-14	0.135248	1.51E-05
-0.42681	-0.09087	-0.28769	-0.49761	0.303592	-0.31221	-0.40639	-0.25511	-0.32756
5.62E-07	0.309594	0.001039	2.65E-09	0.000521	0.000352	2.13E-06	0.003797	0.00017

MAF	MAFB	MAGI1	MAGI2	MAGI3	MAMSTR	MAN1C1	MAN2A1	MAN2B1
brown	brown	brown	brown	brown	brown	brown	brown	brown
0.208123	0.30975	-0.29341	-0.15446	-0.05536	-0.04083	0.259136	0.365274	0.031215
0.018874	0.000394	0.000814	0.082938	0.536428	0.648548	0.003262	2.42E-05	0.727554
0.030315	0.185704	-0.26394	-0.03514	-0.04226	0.021238	0.192214	0.313453	-0.13858
0.735113	0.036591	0.002714	0.694924	0.637096	0.812656	0.030392	0.000333	0.120232
-0.24271	-0.3842	0.341073	0.085686	0.192392	0.051652	-0.38278	-0.3885	-0.09822
0.005972	8.22E-06	8.71E-05	0.338138	0.030235	0.56413	8.94E-06	6.38E-06	0.271935
0.064185	-0.2013	0.079977	0.062914	0.108978	-0.29051	-0.17882	-0.00264	-0.14033
0.473426	0.023247	0.371417	0.482251	0.222612	0.000922	0.044273	0.976486	0.115588
-0.09882	-0.04786	-0.51593	-0.2584	-0.30682	-0.08322	-0.12257	-0.0932	-0.25157
0.268994	0.593147	5.38E-10	0.003355	0.000451	0.352299	0.169805	0.297334	0.00433
0.140518	-0.03934	-0.3889	-0.13288	-0.15244	-0.40119	-0.06933	0.149131	-0.25522
0.115089	0.66054	6.23E-06	0.136405	0.087084	2.95E-06	0.438606	0.094252	0.003781
-0.03678	-0.11766	-0.28399	-0.07892	-0.15199	-0.27108	-0.09312	0.133377	-0.1816
0.681416	0.187713	0.001213	0.377769	0.088049	0.002052	0.297732	0.134938	0.041017
0.233504	0.153689	-0.28813	-0.00098	-0.04705	-0.24554	0.260247	0.188251	-0.00709
0.00824	0.084505	0.00102	0.991299	0.599394	0.005395	0.003127	0.034049	0.936938
0.268622	0.398355	-0.28711	-0.44373	-0.40565	-0.31113	0.252419	0.155311	0.640652
0.002261	3.51E-06	0.001065	1.74E-07	2.23E-06	0.00037	0.004196	0.081237	5.01E-16
0.646704	0.844458	-0.71748	-0.57596	-0.6958	-0.43417	0.652361	0.631663	0.736663
2.16E-16	1.08E-35	2.32E-21	1.4E-12	1.1E-19	3.4E-07	9.68E-17	1.69E-15	5.58E-23
0.496516	0.55549	-0.40325	-0.35099	-0.32328	-0.39673	0.469129	0.446748	0.469779
2.91E-09	1.22E-11	2.59E-06	5.21E-05	0.00021	3.88E-06	2.65E-08	1.4E-07	2.52E-08
-0.18048	-0.04047	0.109096	0.08591	-0.01335	0.009555	0.029596	-0.22072	0.272033
0.04231	0.651423	0.22211	0.336873	0.881566	0.915091	0.741168	0.012644	0.001975
-0.16352	-0.06365	0.441603	0.028215	0.093848	0.277449	-0.05007	-0.24394	0.422769
0.066226	0.477159	2.02E-07	0.752846	0.293961	0.001588	0.576165	0.005714	7.37E-07
-0.099	0.129938	0.02575	-0.28283	-0.29859	0.403816	0.082995	-0.23721	0.462392
0.268143	0.145384	0.773834	0.001274	0.00065	2.5E-06	0.353584	0.007248	4.43E-08
0.298378	0.582597	-0.18331	-0.42966	-0.49938	0.031075	0.46558	0.26559	0.897278
0.000656	6.7E-13	0.039118	4.63E-07	2.28E-09	0.728725	3.48E-08	0.002546	3.24E-46
0.042586	0.094832	0.09644	0.187616	-0.07216	0.10159	0.13757	0.341226	-0.07575
0.634512	0.288902	0.280768	0.034668	0.42011	0.255753	0.122988	8.64E-05	0.397297
-0.48574	-0.58024	0.754956	0.497794	0.517205	0.439586	-0.45851	-0.5537	-0.38123
7.1E-09	8.72E-13	1.17E-24	2.61E-09	4.79E-10	2.33E-07	5.93E-08	1.46E-11	9.78E-06
-0.26165	-0.56839	0.462363	0.547747	0.632174	0.138545	-0.4495	-0.24192	-0.84649
0.002964	3.16E-12	4.44E-08	2.65E-11	1.58E-15	0.120331	1.15E-07	0.006142	5.06E-36
-0.37452	-0.42788	0.384297	0.474516	0.532637	0.317906	-0.30886	-0.32074	-0.62825
1.44E-05	5.23E-07	8.18E-06	1.74E-08	1.15E-10	0.00027	0.000411	0.000236	2.65E-15
-0.37238	-0.44323	0.282812	0.036474	0.198204	0.177807	-0.58757	-0.36199	-0.2277
1.62E-05	1.8E-07	0.001275	0.683929	0.025498	0.045507	3.83E-13	2.89E-05	0.010036

MAN2B2	MANEAL	MAP3K8	MAP4K1	MAPK11	MAPKAPK	44256	44261	MARCO
brown	brown	brown	brown	brown	brown	brown	brown	brown
0.243379	-0.27755	0.196338	0.217325	-0.12023	0.076622	0.309425	-0.0754	0.007817
0.00583	0.001582	0.026945	0.014115	0.178155	0.391883	0.0004	0.399507	0.930498
0.177303	-0.13321	0.091299	0.045879	-0.02328	0.015209	0.216699	-0.02232	-0.06291
0.046133	0.135431	0.307325	0.60852	0.795008	0.86524	0.014402	0.803326	0.482271
-0.51801	0.437427	-0.28334	-0.15077	-0.02086	-0.02092	-0.41042	0.238159	-0.10356
4.46E-10	2.71E-07	0.001247	0.090646	0.815925	0.815451	1.65E-06	0.007012	0.246616
-0.37093	0.137932	0.067433	-0.09777	-0.49447	-0.0268	-0.18943	0.270864	0.067983
1.76E-05	0.121998	0.451287	0.274153	3.45E-09	0.7649	0.032927	0.002069	0.447596
-0.35845	0.030016	0.103935	-0.15461	0.000266	0.203932	0.08103	-0.41405	0.077808
3.5E-05	0.73763	0.244886	0.082642	0.997633	0.021465	0.365132	1.31E-06	0.384571
-0.37176	-0.06106	0.300819	-0.13264	-0.54506	0.09918	0.081749	-0.0599	0.18526
1.68E-05	0.495287	0.000589	0.137132	3.46E-11	0.267265	0.360882	0.503489	0.03705
-0.0609	0.001742	0.261978	-0.07231	-0.33523	0.088484	-0.09459	-0.11865	0.177417
0.496405	0.984496	0.002927	0.419121	0.000117	0.322544	0.290145	0.183987	0.045991
0.063787	-0.39881	0.246847	0.044495	-0.40226	0.183798	0.27372	-0.00283	0.044086
0.476178	3.41E-06	0.005146	0.619383	2.76E-06	0.038596	0.001847	0.974795	0.622609
0.290752	-0.20162	0.484513	0.462735	-0.44726	0.219969	-0.19529	-0.31534	0.595866
0.000913	0.023024	7.84E-09	4.32E-08	1.35E-07	0.012958	0.02779	0.000305	1.47E-13
0.532208	-0.65013	0.78377	0.760059	-0.54988	0.583366	0.485575	-0.65195	0.649398
1.2E-10	1.33E-16	1.25E-27	3.73E-25	2.14E-11	6.15E-13	7.19E-09	1.03E-16	1.48E-16
0.312735	-0.46793	0.532396	0.589098	-0.59531	0.41328	0.41252	-0.37018	0.351848
0.000344	2.91E-08	1.18E-10	3.21E-13	1.57E-13	1.37E-06	1.44E-06	1.84E-05	4.98E-05
0.164908	0.366929	-0.17356	0.175877	-0.00855	-0.00325	-0.44115	-0.10011	-0.09869
0.063918	2.2E-05	0.051006	0.047942	0.924001	0.971089	2.09E-07	0.262765	0.269631
0.124046	0.227046	-0.31355	0.283888	0.265503	0.07569	-0.38622	0.085383	-0.05946
0.164689	0.010258	0.000331	0.001219	0.002554	0.397684	7.3E-06	0.339858	0.506665
0.036717	0.081419	-0.22323	0.314887	0.45384	0.2839	-0.16027	-0.39713	0.053648
0.68193	0.36283	0.011644	0.000311	8.38E-08	0.001218	0.071867	3.79E-06	0.54915
0.535144	-0.35362	0.240684	0.770367	-0.00601	0.493008	0.088609	-0.51198	0.361561
9.07E-11	4.54E-05	0.006416	3.41E-26	0.946508	3.9E-09	0.321854	7.64E-10	2.96E-05
0.240668	-0.18667	-0.16279	0.004457	0.297797	-0.01001	0.362739	-0.04592	-0.29129
0.006419	0.035604	0.067454	0.960339	0.000673	0.911026	2.78E-05	0.608182	0.000892
-0.30483	0.535519	-0.71517	-0.45089	0.668237	-0.30959	-0.28136	0.391083	-0.60045
0.000493	8.75E-11	3.55E-21	1.04E-07	9.26E-18	0.000397	0.001353	5.46E-06	8.54E-14
-0.40955	0.34421	-0.36732	-0.76761	0.203392	-0.63763	-0.02284	0.697834	-0.48623
1.74E-06	7.42E-05	2.16E-05	6.54E-26	0.02182	7.56E-16	0.798823	7.75E-20	6.82E-09
-0.09528	0.289039	-0.41892	-0.61537	0.541886	-0.53079	-0.00158	0.424875	-0.48059
0.286598	0.000982	9.5E-07	1.38E-14	4.73E-11	1.37E-10	0.985909	6.4E-07	1.08E-08
-0.38527	0.398378	-0.31766	-0.20905	0.357436	-0.06694	-0.39357	0.260318	-0.24532
7.72E-06	3.51E-06	0.000273	0.018337	3.7E-05	0.454634	4.7E-06	0.003119	0.005439

MARK1	MAX	MBNL1	MCOLN2	MDFIC	MED12L	MERTK	MFNG	MFSD1
brown	brown	brown	brown	brown	brown	brown	brown	brown
-0.30515	0.378701	0.22557	0.308299	-0.04501	-0.14019	0.245877	-0.01624	0.192449
0.000486	1.13E-05	0.010776	0.000421	0.615323	0.115936	0.00533	0.8562	0.030185
-0.23734	0.296795	0.072753	0.127491	-0.13192	-0.0974	0.218575	-0.08669	0.036305
0.007214	0.000703	0.416303	0.153184	0.139296	0.275975	0.013557	0.332462	0.68531
0.501873	-0.33796	-0.05412	-0.08172	-0.05585	0.095599	-0.45857	-0.16931	-0.10556
1.84E-09	0.000102	0.545655	0.361035	0.532849	0.285004	5.9E-08	0.057055	0.237536
0.336049	-0.15597	0.392749	0.183271	0.356939	0.313871	-0.20858	-0.24198	0.246401
0.000112	0.079935	4.94E-06	0.039166	3.8E-05	0.000326	0.018608	0.006129	0.00523
-0.3014	0.142794	-0.05294	0.054236	0.046153	-0.26346	-0.09665	0.164392	0.07792
0.000574	0.109262	0.554476	0.544772	0.606376	0.002764	0.279714	0.064765	0.38389
-0.07573	0.067271	0.471161	0.312764	0.494744	0.117528	-0.04442	-0.0829	0.418653
0.397431	0.452377	2.26E-08	0.000344	3.37E-09	0.188193	0.619972	0.354118	9.67E-07
0.013167	0.007501	0.175745	0.206267	0.177273	0.105578	-0.02757	-0.10577	0.2189
0.883191	0.933298	0.048113	0.019986	0.04617	0.237466	0.758329	0.236627	0.013415
-0.26723	-0.07938	0.380524	0.300931	0.525666	-0.01425	0.21149	0.055189	0.358828
0.002388	0.375011	1.02E-05	0.000586	2.21E-10	0.873687	0.016992	0.537717	3.43E-05
-0.18365	0.21788	0.320754	0.425528	0.274845	-0.26263	0.01789	0.240839	0.535949
0.038752	0.013865	0.000236	6.13E-07	0.001765	0.002855	0.841767	0.006381	8.4E-11
-0.82834	0.620739	0.548384	0.760287	0.461325	-0.45829	0.563234	0.671376	0.889454
3.01E-33	7E-15	2.49E-11	3.54E-25	4.8E-08	6.03E-08	5.46E-12	5.72E-18	2.48E-44
-0.52056	0.497354	0.607237	0.734048	0.527732	-0.10763	0.359643	0.354271	0.731072
3.54E-10	2.71E-09	3.77E-14	9.45E-23	1.83E-10	0.228422	3.29E-05	4.38E-05	1.71E-22
0.132393	0.131279	-0.41154	-0.17716	-0.34529	-0.20055	-0.14763	-0.01168	-0.29581
0.137867	0.141241	1.53E-06	0.046306	7.02E-05	0.023773	0.097663	0.896267	0.000734
0.294699	-0.13371	-0.37126	-0.1527	-0.38572	-0.1096	-0.20811	0.095606	-0.22877
0.00077	0.133967	1.73E-05	0.086543	7.52E-06	0.219948	0.01888	0.284971	0.009682
-0.15624	0.152922	-0.46023	-0.12254	-0.28667	-0.37456	-0.15297	0.428076	-0.17635
0.07941	0.086087	5.21E-08	0.169903	0.001085	1.44E-05	0.085978	5.16E-07	0.047332
-0.48859	0.38685	-0.05295	0.309764	-0.03165	-0.50862	0.267511	0.655812	0.361304
5.62E-09	7.03E-06	0.554358	0.000394	0.723944	1.03E-09	0.002362	5.88E-17	3E-05
-0.24252	0.118855	-0.19513	-0.21969	-0.35507	-0.14445	0.366709	0.150937	-0.17331
0.006011	0.183219	0.027919	0.013074	4.2E-05	0.105169	2.23E-05	0.090289	0.051341
0.612834	-0.49435	-0.63504	-0.68304	-0.66741	0.184728	-0.36481	-0.27279	-0.80371
1.9E-14	3.49E-09	1.07E-15	9.07E-19	1.05E-17	0.037606	2.48E-05	0.001917	5.78E-30
0.581654	-0.40081	0.015767	-0.43638	-0.04302	0.615944	-0.18797	-0.69757	-0.50623
7.45E-13	3.02E-06	0.86034	2.92E-07	0.631037	1.29E-14	0.034323	8.11E-20	1.27E-09
0.364042	-0.35085	-0.36818	-0.55344	-0.39546	0.30492	-0.1061	-0.45657	-0.68285
2.58E-05	5.25E-05	2.06E-05	1.5E-11	4.19E-06	0.000491	0.235154	6.85E-08	9.36E-19
0.486733	-0.24089	-0.38382	-0.3637	-0.66131	-0.09879	-0.40539	-0.23389	-0.42147
6.55E-09	0.006368	8.41E-06	2.63E-05	2.62E-17	0.269134	2.27E-06	0.008133	8.03E-07

MFSD5	MGAT1	MGAT4A	MGST1	MGST2	MILR1	MITF	MKNK1	MLKL
brown	brown	brown	brown	brown	brown	brown	brown	brown
0.234495	0.061423	0.285227	0.253688	0.224387	0.319711	0.321161	0.337212	0.201941
0.007964	0.492708	0.001153	0.004004	0.011208	0.000248	0.000232	0.000106	0.0228
0.271875	-0.09393	0.178577	0.301543	0.204332	0.211315	0.230214	0.242376	0.075413
0.001988	0.293531	0.044565	0.000571	0.021205	0.017086	0.00922	0.006042	0.399419
-0.1824	-0.20605	-0.40851	-0.55117	-0.37254	-0.11996	-0.22051	-0.55963	-0.08432
0.040123	0.02012	1.86E-06	1.88E-11	1.61E-05	0.179164	0.012732	7.94E-12	0.345936
-0.11438	-0.21335	-0.05481	-0.3482	-0.1613	0.238024	0.050036	-0.41823	0.149451
0.2004	0.016027	0.540554	6.03E-05	0.070044	0.007045	0.576403	9.94E-07	0.093541
0.520027	0.062703	0.025806	0.215243	0.597267	0.369711	0.156695	-0.1349	0.044334
3.71E-10	0.483724	0.773357	0.015089	1.25E-13	1.89E-05	0.07853	0.130513	0.620657
0.27367	-0.09566	0.176355	0.022826	0.372868	0.542893	0.266531	-0.27192	0.27956
0.00185	0.284694	0.04733	0.798936	1.58E-05	4.29E-11	0.002454	0.001984	0.001457
0.203891	-0.11387	0.159108	0.044396	0.302777	0.665993	0.084797	-0.12046	0.170795
0.021493	0.202393	0.073982	0.620164	0.00054	1.3E-17	0.343196	0.177332	0.054875
0.117867	0.04616	0.341527	0.375455	0.209059	0.247183	0.216168	0.212884	0.119239
0.186912	0.606326	8.51E-05	1.36E-05	0.018334	0.005084	0.014649	0.016262	0.1818
0.141408	0.500887	0.034619	-0.13116	0.111566	0.309229	0.04877	0.089526	0.570713
0.112783	2.01E-09	0.699207	0.141591	0.211754	0.000404	0.586095	0.316856	2.47E-12
0.568431	0.790787	0.58749	0.535067	0.69035	0.717685	0.565415	0.676978	0.810798
3.15E-12	2.02E-28	3.86E-13	9.14E-11	2.74E-19	2.23E-21	4.34E-12	2.39E-18	7.36E-31
0.387028	0.441311	0.433127	0.247186	0.397727	0.605473	0.377471	0.407425	0.668132
6.96E-06	2.07E-07	3.66E-07	0.005083	3.65E-06	4.67E-14	1.22E-05	1.99E-06	9.41E-18
-0.05972	-0.04532	-0.3087	-0.22901	-0.27206	-0.22856	-0.28123	0.00288	-0.10795
0.504808	0.612859	0.000414	0.009602	0.001974	0.009751	0.001361	0.97436	0.227031
-0.27071	0.137455	-0.42226	-0.35941	-0.53168	-0.37984	-0.30355	-0.007	-0.09059
0.002082	0.123306	7.62E-07	3.33E-05	1.26E-10	1.06E-05	0.000522	0.937765	0.311108
0.245252	0.506921	-0.38229	-0.00227	-0.00771	-0.28728	-0.16637	0.093802	-0.09664
0.005451	1.19E-09	9.2E-06	0.979774	0.931447	0.001057	0.061567	0.2942	0.279779
0.276587	0.776907	0.019478	0.213939	0.151127	0.08417	0.143804	0.565084	0.429661
0.001645	6.99E-27	0.827931	0.015728	0.089879	0.34679	0.106753	4.49E-12	4.63E-07
0.061183	-0.0368	0.23745	0.386127	0.126631	-0.10381	0.273037	0.433359	-0.08458
0.494404	0.681293	0.007188	7.34E-06	0.155996	0.245471	0.001898	3.6E-07	0.344414
-0.46164	-0.47235	-0.43926	-0.39067	-0.58039	-0.69546	-0.40909	-0.31519	-0.70205
4.69E-08	2.06E-08	2.39E-07	5.6E-06	8.57E-13	1.16E-19	1.79E-06	0.000307	3.74E-20
-0.47613	-0.7646	-0.01172	-0.27241	-0.36786	-0.37547	-0.20813	-0.48239	-0.58044
1.53E-08	1.32E-25	0.895923	0.001946	2.09E-05	1.36E-05	0.018871	9.31E-09	8.52E-13
-0.41132	-0.57363	-0.04618	-0.02444	-0.30104	-0.51579	-0.32676	-0.15659	-0.65371
1.56E-06	1.8E-12	0.606138	0.785087	0.000584	5.44E-10	0.000177	0.078723	7.97E-17
-0.30763	-0.29109	-0.37469	-0.5606	-0.35352	-0.19862	-0.11735	-0.38469	-0.20949
0.000434	0.0009	1.43E-05	7.18E-12	4.56E-05	0.025184	0.188869	7.99E-06	0.018092

MLPH	MNDA	MOB3C	MPEG1	MPPED2	MR1	MRC1	MS4A1	MS4A14
brown	brown	brown	brown	brown	brown	brown	brown	brown
0.319176	0.414322	0.299314	0.461721	-0.32351	0.267048	0.210352	0.236866	0.285772
0.000255	1.28E-06	0.00063	4.66E-08	0.000207	0.002405	0.017609	0.007336	0.001127
0.24873	0.265753	0.177616	0.298271	-0.20348	0.156006	0.189221	0.154421	0.181364
0.004806	0.00253	0.045743	0.000659	0.021766	0.079868	0.033121	0.083017	0.041289
-0.21562	-0.37228	-0.36168	-0.34683	0.363003	-0.22275	-0.24333	-0.11069	-0.25776
0.014909	1.63E-05	2.94E-05	6.48E-05	2.74E-05	0.011833	0.005841	0.215374	0.003437
-0.0523	-0.06524	-0.17366	-0.0716	0.112255	0.271265	0.067727	0.005393	-0.0444
0.559248	0.466173	0.050875	0.423739	0.208927	0.002037	0.449306	0.952019	0.620112
0.11506	0.208713	0.079644	0.099915	-0.07098	0.169283	0.165114	0.185585	-0.04471
0.197702	0.018532	0.37342	0.263716	0.427799	0.05709	0.063582	0.036713	0.6177
0.128256	0.273147	0.024115	0.173732	-0.10165	0.533099	0.325709	0.195645	0.062979
0.150713	0.001889	0.787839	0.050774	0.255461	1.1E-10	0.000186	0.027499	0.481794
0.098371	0.100772	0.056564	0.092511	-0.04972	0.334385	0.242796	0.132941	0.039972
0.271203	0.259622	0.527618	0.300921	0.578839	0.000122	0.005952	0.13623	0.655461
0.049319	0.276502	0.297767	0.327336	-0.28059	0.526364	0.215946	-0.04102	0.303965
0.581884	0.001651	0.000674	0.000172	0.001397	2.07E-10	0.014754	0.647006	0.000512
0.359192	0.21385	0.282592	0.221674	-0.41714	0.358011	0.381201	0.285989	0.2993
3.37E-05	0.015773	0.001286	0.012256	1.07E-06	3.59E-05	9.8E-06	0.001116	0.00063
0.536945	0.860842	0.761443	0.82147	-0.77947	0.770846	0.594269	0.534591	0.785953
7.63E-11	1.75E-38	2.72E-25	2.78E-32	3.71E-27	3.04E-26	1.77E-13	9.56E-11	7.14E-28
0.373054	0.68749	0.623891	0.66812	-0.51736	0.756067	0.328391	0.382323	0.499282
1.56E-05	4.4E-19	4.67E-15	9.42E-18	4.72E-10	9.12E-25	0.000164	9.18E-06	2.3E-09
-0.0495	-0.29847	-0.05321	-0.21036	-0.01061	-0.27665	-0.21715	-0.09299	-0.14966
0.580483	0.000654	0.552392	0.017605	0.905779	0.001641	0.014194	0.29844	0.093081
-0.11154	-0.4022	-0.04899	-0.24364	0.117522	-0.44285	-0.35125	-0.14768	-0.02779
0.211877	2.77E-06	0.584411	0.005776	0.188216	1.85E-07	5.14E-05	0.097539	0.756425
-0.01644	-0.16328	0.139039	-0.15798	-0.04841	-0.35304	-0.29157	-0.04037	-0.0289
0.854473	0.06662	0.119002	0.076077	0.588849	4.68E-05	0.000881	0.65224	0.747032
0.278713	0.27711	0.551203	0.351848	-0.48611	0.108245	0.075463	0.229837	0.507571
0.001509	0.00161	1.88E-11	4.98E-05	6.89E-09	0.225756	0.399105	0.009338	1.13E-09
-0.07005	0.187398	0.083157	0.177498	0.02408	-0.20836	-0.10349	-0.05654	0.194258
0.433854	0.034883	0.352642	0.04589	0.788143	0.018738	0.246928	0.527812	0.028639
-0.52697	-0.62955	-0.58061	-0.59908	0.706308	-0.84149	-0.65885	-0.42693	-0.54679
1.96E-10	2.23E-15	8.37E-13	1.01E-13	1.77E-20	3.18E-35	3.77E-17	5.57E-07	2.92E-11
-0.3866	-0.37922	-0.63035	-0.45046	0.61114	-0.31479	-0.23144	-0.33514	-0.56488
7.14E-06	1.1E-05	2.01E-15	1.07E-07	2.34E-14	0.000313	0.008844	0.000117	4.59E-12
-0.34553	-0.37526	-0.46167	-0.40765	0.476281	-0.52492	-0.30452	-0.34857	-0.4864
6.93E-05	1.38E-05	4.68E-08	1.97E-06	1.52E-08	2.37E-10	0.0005	5.92E-05	6.73E-09
-0.17148	-0.39139	-0.44117	-0.33103	0.371082	-0.6427	-0.20839	-0.09638	-0.27479
0.053899	5.36E-06	2.09E-07	0.000144	1.75E-05	3.77E-16	0.018716	0.281081	0.001769

MS4A4A	MS4A6A	MS4A7	MSANTD4	MSLN	MSR1	MSRB3	MTCL1	MTMR14
brown	brown	brown	brown	brown	brown	brown	brown	brown
0.317591	0.315709	0.325957	-0.24555	0.150814	0.201794	0.349487	-0.22917	0.150614
0.000274	0.0003	0.000184	0.005394	0.090554	0.022901	5.64E-05	0.009551	0.090988
0.214012	0.168159	0.181436	-0.16203	0.060705	0.061855	0.309699	-0.20713	0.099261
0.015692	0.058784	0.041207	0.068763	0.497786	0.489665	0.000395	0.01946	0.266872
-0.30589	-0.24471	-0.30833	0.524156	-0.35686	-0.04611	-0.43983	0.373647	-0.15452
0.00047	0.005559	0.000421	2.54E-10	3.82E-05	0.606685	2.29E-07	1.51E-05	0.082827
-0.05495	0.052438	-0.06791	0.549073	-0.3832	0.289087	-0.20815	0.187473	-0.17195
0.539481	0.558206	0.448111	2.33E-11	8.72E-06	0.00098	0.018861	0.034808	0.053235
0.228613	0.269295	-0.00033	0.137671	0.012179	0.157639	0.076312	-0.23207	0.322225
0.009732	0.002202	0.997039	0.122712	0.8919	0.076724	0.393805	0.008655	0.00022
0.257725	0.364315	0.10681	0.439148	-0.26322	0.470923	0.040817	-0.08426	0.064281
0.003442	2.55E-05	0.232006	2.41E-07	0.00279	2.31E-08	0.648657	0.34629	0.472761
0.145315	0.23433	0.003459	0.27057	-0.15748	0.254747	0.159596	-0.06785	0.10027
0.103083	0.008009	0.969212	0.002094	0.077022	0.003849	0.073088	0.448505	0.262014
0.228283	0.334146	0.273153	-0.09322	0.04332	0.30344	0.157451	-0.29362	-0.0496
0.009841	0.000123	0.001889	0.297214	0.628675	0.000525	0.077081	0.000807	0.579758
0.376508	0.413222	0.31541	-0.13355	-0.02019	0.513292	0.363049	0.067871	0.22931
1.28E-05	1.38E-06	0.000304	0.134419	0.821729	6.8E-10	2.73E-05	0.448345	0.009506
0.889269	0.915443	0.859953	-0.49452	0.457229	0.835116	0.579612	-0.47374	0.603934
2.74E-44	3.01E-51	2.53E-38	3.44E-09	6.52E-08	3.03E-34	9.34E-13	1.85E-08	5.63E-14
0.549863	0.654687	0.589034	-0.30647	0.224274	0.675414	0.315931	-0.34304	0.319952
2.15E-11	6.92E-17	3.24E-13	0.000458	0.01125	3.05E-18	0.000297	7.88E-05	0.000246
-0.23278	-0.25307	-0.21125	-0.20738	-0.07613	-0.26013	-0.02319	0.139889	0.00723
0.008447	0.004096	0.017123	0.019313	0.394936	0.003142	0.795798	0.116741	0.935702
-0.28208	-0.32839	-0.15812	-0.11177	0.071083	-0.25681	-0.24839	0.270433	-0.01203
0.001314	0.000164	0.07582	0.210915	0.42711	0.003562	0.004865	0.002105	0.893223
-0.03132	-0.0961	-0.05347	-0.3263	0.343001	-0.1457	-0.17246	-0.05427	0.304966
0.726691	0.282485	0.550509	0.000181	7.89E-05	0.102154	0.052512	0.544524	0.00049
0.401271	0.345412	0.465768	-0.61917	0.519875	0.287889	0.253187	-0.22039	0.474001
2.93E-06	6.97E-05	3.43E-08	8.55E-15	3.76E-10	0.001031	0.004079	0.012783	1.81E-08
0.07133	-0.01327	0.166091	-0.37343	0.39154	-0.19665	0.193258	-0.29805	0.123043
0.425499	0.882253	0.06201	1.53E-05	5.32E-06	0.026697	0.029485	0.000666	0.168155
-0.68371	-0.73761	-0.56201	0.120652	-0.05693	-0.75263	-0.4894	0.229086	-0.34502
8.15E-19	4.6E-23	6.2E-12	0.176638	0.52497	1.94E-24	5.26E-09	0.009578	7.12E-05
-0.53858	-0.52311	-0.48904	0.507308	-0.42578	-0.45838	-0.31727	0.248236	-0.61695
6.52E-11	2.8E-10	5.42E-09	1.15E-09	6.03E-07	5.99E-08	0.000279	0.004893	1.13E-14
-0.50689	-0.55928	-0.45063	0.066627	-0.14396	-0.6735	-0.16167	0.151411	-0.44447
1.19E-09	8.24E-12	1.06E-07	0.456724	0.106377	4.12E-18	0.069382	0.089272	1.65E-07
-0.32869	-0.37521	-0.36123	0.447265	-0.18331	-0.36751	-0.26906	0.385735	0.0388
0.000161	1.38E-05	3.01E-05	1.35E-07	0.039118	2.13E-05	0.002223	7.51E-06	0.664948

MVP	MYBPC3	MYBPH	MYL12A	MYLK	MYO1F	MYO1G	MYO7A	MYOZ3
brown	brown	brown	brown	brown	brown	brown	brown	brown
0.230098	0.174625	-0.00934	0.179829	0.339017	0.113309	0.133308	0.051436	-0.18139
0.009256	0.049579	0.917034	0.043067	9.66E-05	0.204657	0.135142	0.565763	0.041264
0.13593	0.071211	-0.11625	0.111519	0.311537	-0.04566	-0.05129	-0.0887	-0.15533
0.127562	0.426272	0.193094	0.211948	0.000363	0.61025	0.566887	0.321345	0.081193
-0.30338	-0.12879	-0.08129	-0.10191	-0.58893	-0.33797	-0.0108	-0.2373	0.357996
0.000526	0.149006	0.363584	0.254251	3.27E-13	0.000102	0.904083	0.007224	3.59E-05
-0.25335	-0.08295	0.248742	0.238115	-0.33176	-0.3013	0.090005	-0.21108	0.207141
0.004055	0.353859	0.004803	0.007022	0.000139	0.000577	0.314263	0.017212	0.019455
-0.09091	0.075708	0.022925	0.219451	-0.12012	-0.12257	0.007482	-0.21096	0.217461
0.309416	0.397571	0.79808	0.013178	0.178551	0.169796	0.93347	0.017279	0.014053
-0.17757	0.027962	0.315155	0.471767	-0.13352	-0.23809	0.116959	-0.25688	0.188202
0.045795	0.754997	0.000307	2.16E-08	0.134505	0.007027	0.190354	0.003553	0.034097
-0.08961	-0.0092	0.207375	0.409522	-0.03231	-0.18857	0.056405	-0.18506	0.027298
0.316424	0.918265	0.019316	1.75E-06	0.718421	0.03374	0.528783	0.03726	0.760637
0.030695	-0.00501	0.403395	0.254648	0.378126	0.129247	0.124469	0.27474	0.023301
0.731921	0.955411	2.57E-06	0.003863	1.17E-05	0.147557	0.163242	0.001772	0.794846
0.442244	0.257019	0.322993	0.647059	0.10948	0.365185	0.651582	0.410859	-0.12183
1.93E-07	0.003534	0.000212	2.05E-16	0.220474	2.43E-05	1.08E-16	1.6E-06	0.172419
0.774663	0.632578	0.543393	0.775347	0.580922	0.830063	0.738299	0.685881	-0.48132
1.21E-26	1.49E-15	4.08E-11	1.02E-26	8.08E-13	1.69E-33	4E-23	5.72E-19	1.01E-08
0.483922	0.432486	0.52725	0.617021	0.353623	0.484687	0.583553	0.390328	-0.26205
8.23E-09	3.82E-07	1.91E-10	1.12E-14	4.54E-05	7.73E-09	6.02E-13	5.72E-06	0.002919
0.073659	0.059972	-0.03554	0.004748	-0.08704	0.030226	0.01788	0.106276	-0.1508
0.410506	0.503005	0.691596	0.957746	0.33051	0.735861	0.841857	0.23436	0.090579
0.18212	0.043954	-0.12542	-0.27967	-0.20416	0.164768	0.118115	0.274159	-0.03622
0.040436	0.623655	0.160031	0.001451	0.021317	0.064148	0.185983	0.001814	0.686032
0.394905	0.195799	-0.14253	-0.21932	-0.05473	0.3609	0.213386	0.335374	-0.00955
4.34E-06	0.027375	0.109915	0.013235	0.541074	3.07E-05	0.016006	0.000116	0.915156
0.805636	0.494061	0.19531	0.23807	0.37909	0.8078	0.565514	0.753082	-0.43084
3.33E-30	3.57E-09	0.027771	0.007033	1.11E-05	1.78E-30	4.29E-12	1.76E-24	4.28E-07
0.182205	0.05307	-0.19447	-0.2159	0.487058	0.237837	-0.31729	0.061555	-0.40524
0.040341	0.553465	0.02846	0.014775	6.37E-09	0.007091	0.000278	0.491778	2.29E-06
-0.45638	-0.39775	-0.59075	-0.78017	-0.37292	-0.36908	-0.57758	-0.31123	0.139679
6.95E-08	3.65E-06	2.66E-13	3.11E-27	1.58E-05	1.96E-05	1.17E-12	0.000369	0.117295
-0.77978	-0.52558	-0.36396	-0.4977	-0.30933	-0.73554	-0.67714	-0.70713	0.322805
3.42E-27	2.23E-10	2.6E-05	2.63E-09	0.000402	7E-23	2.33E-18	1.53E-20	0.000214
-0.54607	-0.45774	-0.47459	-0.62291	-0.10271	-0.46069	-0.64768	-0.44755	0.165375
3.13E-11	6.28E-08	1.73E-08	5.3E-15	0.250527	5.03E-08	1.88E-16	1.32E-07	0.063158
-0.37527	-0.15099	-0.45069	-0.3676	-0.60648	-0.35721	-0.23695	-0.30919	0.27779
1.38E-05	0.090169	1.05E-07	2.12E-05	4.14E-14	3.75E-05	0.007314	0.000405	0.001567

MZB1	NAAA	NABP1	NAGA	NAGK	NAMPT	NANOS3	NANS	NBAS
brown	brown	brown	brown	brown	brown	brown	brown	brown
0.325257	0.337464	0.330359	0.145394	0.29417	-0.05724	-0.20395	0.273703	-0.14782
0.000191	0.000105	0.000149	0.102894	0.000788	0.522687	0.021452	0.001848	0.097215
0.187685	0.257737	0.169357	-0.02114	0.149079	-0.13091	-0.08866	0.251438	-0.12379
0.0346	0.00344	0.05698	0.813538	0.094369	0.142364	0.321557	0.004351	0.165554
-0.30783	-0.55648	-0.21293	-0.03797	-0.2735	0.189768	0.152435	-0.18587	0.364827
0.000431	1.1E-11	0.016241	0.67166	0.001863	0.032607	0.087103	0.036424	2.48E-05
-0.03474	-0.1584	0.138602	0.094216	-0.33104	0.536052	-0.28393	-0.29883	0.342505
0.698222	0.075296	0.120176	0.292065	0.000144	8.32E-11	0.001217	0.000643	8.1E-05
0.162385	0.205682	0.062258	0.034413	0.155687	-0.08289	0.183397	0.514959	-0.36873
0.06815	0.020348	0.486839	0.700912	0.080493	0.354213	0.039029	5.86E-10	1.99E-05
0.209851	0.208741	0.329296	0.159221	-0.14091	0.470991	-0.32059	0.064813	-0.0225
0.017887	0.018516	0.000157	0.073773	0.114077	2.29E-08	0.000238	0.469101	0.801721
0.184155	0.228872	0.11436	0.158988	-0.12492	0.297054	-0.19617	0.119781	0.066065
0.038213	0.009648	0.200463	0.074203	0.161712	0.000695	0.027079	0.179808	0.460538
0.25062	0.372728	0.320972	0.18926	0.024322	0.214038	-0.40556	-0.14855	-0.13957
0.004484	1.59E-05	0.000234	0.033084	0.786066	0.015679	2.24E-06	0.09555	0.117591
0.279742	0.157698	0.427028	0.592806	0.162946	0.594485	-0.40329	0.292263	0.17313
0.001447	0.076612	5.54E-07	2.1E-13	0.067189	1.72E-13	2.59E-06	0.000856	0.051593
0.725302	0.715175	0.860477	0.82909	0.723402	0.46385	-0.62875	0.532262	-0.45743
5.27E-22	3.55E-21	2.04E-38	2.34E-33	7.58E-22	3.97E-08	2.48E-15	1.19E-10	6.42E-08
0.580894	0.482334	0.686707	0.636359	0.521868	0.482987	-0.61245	0.233627	-0.17006
8.1E-13	9.35E-09	5E-19	8.98E-16	3.14E-10	8.87E-09	1.99E-14	0.008205	0.055938
-0.24072	-0.26576	-0.25641	0.083223	0.003712	-0.07812	0.151969	0.156346	0.340824
0.006407	0.002529	0.003616	0.352261	0.966965	0.382682	0.088083	0.079204	8.82E-05
-0.20746	-0.45855	-0.25408	0.08211	0.079305	-0.08005	0.204271	-0.11538	0.303944
0.019266	5.91E-08	0.003946	0.35876	0.375468	0.37098	0.021245	0.196457	0.000513
-0.00508	-0.18909	-0.2089	0.061953	0.423833	-0.24211	0.406941	0.308296	-0.29397
0.954766	0.033245	0.018422	0.488978	6.86E-07	0.0061	2.06E-06	0.000421	0.000795
0.350461	0.176248	0.309959	0.568264	0.676116	0.074685	-0.18057	0.352673	-0.26517
5.36E-05	0.047465	0.000391	3.21E-12	2.73E-18	0.403997	0.042199	4.77E-05	0.002587
0.005518	0.16765	-0.10231	-0.16394	0.282503	-0.57699	0.040709	-0.03162	-0.38622
0.950907	0.059565	0.25239	0.065512	0.001291	1.25E-12	0.649526	0.724129	7.3E-06
-0.63645	-0.65647	-0.76379	-0.571	-0.27179	-0.63585	0.647711	-0.341	0.181231
8.87E-16	5.34E-17	1.59E-25	2.39E-12	0.001995	9.63E-16	1.87E-16	8.74E-05	0.041441
-0.45843	-0.26437	-0.42542	-0.74104	-0.68081	-0.23523	0.252434	-0.567	0.230479
5.97E-08	0.002669	6.17E-07	2.28E-23	1.3E-18	0.007765	0.004194	3.67E-12	0.009137
-0.4554	-0.19933	-0.54125	-0.68117	-0.41483	-0.56768	0.457318	-0.28714	0.088396
7.47E-08	0.024659	5.03E-11	1.23E-18	1.24E-06	3.41E-12	6.48E-08	0.001064	0.323024
-0.31884	-0.46992	-0.33116	-0.17345	-0.19056	-0.37446	0.449363	0.117988	0.293268
0.000259	2.49E-08	0.000143	0.051158	0.031873	1.44E-05	1.16E-07	0.186459	0.000819

NCF1	NCF2	NCF4	NCKAP1L	NCOA4	NEU1	NFAM1	NFATC1	NFATC2
brown	brown	brown	brown	brown	brown	brown	brown	brown
0.327137	0.152392	0.259986	0.239636	0.467127	0.246363	0.082504	0.110402	0.253874
0.000174	0.087192	0.003159	0.006657	3.09E-08	0.005237	0.356447	0.216591	0.003976
0.196624	0.024104	0.140534	0.07005	0.387402	0.211593	-0.06736	0.027508	0.07107
0.026719	0.787933	0.115047	0.433871	6.81E-06	0.016938	0.451747	0.758846	0.427192
-0.27207	-0.25858	-0.27421	-0.28467	-0.47338	-0.12727	-0.19074	-0.10264	-0.19801
0.001973	0.003332	0.00181	0.00118	1.9E-08	0.153907	0.031708	0.250848	0.025643
-0.1198	0.028957	-0.07075	-0.04716	-0.30023	0.148321	-0.13163	-0.15936	0.151122
0.179732	0.746564	0.429312	0.59854	0.000605	0.096075	0.140163	0.073511	0.08989
0.109885	0.15717	0.298154	-0.00386	0.123482	0.524077	-0.10522	-0.16632	-0.06973
0.218761	0.077617	0.000663	0.96568	0.166632	2.56E-10	0.239088	0.061642	0.436003
0.058692	0.254528	0.237675	0.110525	-0.01388	0.554581	-0.09088	-0.22793	0.226427
0.512178	0.00388	0.007131	0.216075	0.876921	1.33E-11	0.30954	0.00996	0.010473
0.059236	0.161341	0.179525	0.048409	-0.00456	0.438296	-0.08711	-0.16284	0.175889
0.508267	0.069966	0.043427	0.588878	0.959385	2.55E-07	0.330127	0.067363	0.047926
0.072741	0.278683	0.259806	0.277858	0.152551	0.145949	0.082047	0.158302	0.399756
0.416375	0.001511	0.00318	0.001562	0.08686	0.101572	0.359126	0.075475	3.22E-06
0.336187	0.384986	0.401424	0.342655	-0.13471	0.328422	0.425285	0.25291	0.363919
0.000111	7.85E-06	2.91E-06	8.03E-05	0.131034	0.000163	6.23E-07	0.004121	2.6E-05
0.808896	0.874056	0.943471	0.907191	0.538052	0.523601	0.815469	0.521448	0.774403
1.29E-30	5.25E-41	8.52E-62	7.82E-49	6.86E-11	2.68E-10	1.8E-31	3.26E-10	1.29E-26
0.608623	0.586071	0.613771	0.694914	0.359309	0.455008	0.565816	0.317532	0.706313
3.19E-14	4.53E-13	1.69E-14	1.27E-19	3.35E-05	7.69E-08	4.16E-12	0.000275	1.76E-20
0.024732	-0.19662	-0.11239	-0.15482	-0.24133	-0.06663	-0.06878	0.102968	-0.11303
0.782545	0.026725	0.208367	0.082219	0.00627	0.456685	0.442255	0.24933	0.2058
-0.01323	-0.18858	-0.19003	-0.12393	-0.2922	-0.49107	0.15386	0.339077	-0.12218
0.882618	0.033729	0.032365	0.165102	0.000858	4.58E-09	0.084156	9.64E-05	0.171189
0.212212	0.031113	0.108223	-0.03427	-0.10089	-0.13349	0.293508	0.343197	-0.18258
0.016611	0.728406	0.225853	0.70213	0.259084	0.134605	0.000811	7.81E-05	0.039924
0.587812	0.460155	0.519911	0.508484	0.214244	-0.05461	0.72925	0.689258	0.368539
3.72E-13	5.24E-08	3.75E-10	1.04E-09	0.015576	0.542003	2.45E-22	3.29E-19	2.02E-05
0.104542	0.031181	0.026929	0.129891	0.509242	-0.35109	0.038836	0.106661	-0.05114
0.242129	0.727835	0.763777	0.145532	9.73E-10	5.19E-05	0.664656	0.232661	0.567983
-0.50903	-0.66732	-0.67482	-0.56949	-0.23231	-0.68089	-0.44434	-0.20677	-0.59058
9.92E-10	1.06E-17	3.35E-18	2.81E-12	0.008584	1.28E-18	1.67E-07	0.019682	2.71E-13
-0.67157	-0.59249	-0.70462	-0.56309	-0.18764	-0.24079	-0.6954	-0.66093	-0.45659
5.55E-18	2.17E-13	2.38E-20	5.54E-12	0.034647	0.006391	1.17E-19	2.77E-17	6.84E-08
-0.54077	-0.59364	-0.60983	-0.49502	-0.04162	-0.37289	-0.56009	-0.44975	-0.53596
5.27E-11	1.9E-13	2.75E-14	3.3E-09	0.642195	1.58E-05	7.57E-12	1.13E-07	8.39E-11
-0.32212	-0.41281	-0.30895	-0.41175	-0.1973	-0.32023	-0.30717	-0.08621	-0.38791
0.000221	1.41E-06	0.000409	1.51E-06	0.026191	0.000242	0.000444	0.335208	6.6E-06

NFKBIA	NFKBIE	NFKBIZ	NINJ1	NKG7	NKX2-8	NLE1	NLGN4X	NLRC4
brown	brown	brown	brown	brown	brown	brown	brown	brown
0.145905	0.16172	0.059855	0.296545	0.423475	0.181851	-0.23977	-0.40642	0.116016
0.101677	0.069301	0.503838	0.000711	7.03E-07	0.040738	0.006626	2.13E-06	0.19398
0.093524	0.08619	-0.03379	0.240258	0.248399	0.082385	-0.18339	-0.35273	-0.01796
0.295635	0.335295	0.706063	0.006513	0.004864	0.357143	0.039039	4.76E-05	0.841157
-0.15414	-0.02858	-0.03342	-0.52254	-0.21848	-0.37818	0.52635	0.656545	0.009641
0.08358	0.74979	0.709146	2.95E-10	0.013597	1.17E-05	2.08E-10	5.28E-17	0.914327
0.047979	-0.02082	0.371633	-0.51526	-0.02947	-0.39697	0.146242	0.491288	0.247761
0.592194	0.816255	1.69E-05	5.71E-10	0.742216	3.82E-06	0.10088	4.5E-09	0.004978
0.111411	0.464458	-0.04907	0.210499	0.254721	0.01028	0.164759	-0.3812	0.147915
0.212393	3.79E-08	0.583821	0.017529	0.003852	0.908673	0.064162	9.8E-06	0.097
0.180762	0.290183	0.415926	-0.19812	0.248898	-0.26164	0.009578	0.027791	0.383381
0.041979	0.000935	1.16E-06	0.025564	0.004776	0.002965	0.914885	0.756445	8.63E-06
0.185205	0.246782	0.334237	-0.04791	0.162353	-0.33742	0.014749	-0.03158	0.176238
0.037106	0.005158	0.000123	0.592696	0.068204	0.000105	0.869272	0.724458	0.047479
0.057402	0.061896	0.164826	0.058493	0.074058	0.020259	-0.37699	-0.31869	0.177197
0.521511	0.489379	0.064052	0.513613	0.407966	0.821143	1.25E-05	0.000261	0.046266
0.432999	0.186803	0.583974	-0.00964	0.406333	-0.1364	-0.08127	0.120292	0.495761
3.69E-07	0.035474	5.74E-13	0.914339	2.14E-06	0.126227	0.363687	0.177944	3.1E-09
0.611061	0.50788	0.579851	0.666085	0.798599	0.428758	-0.55286	-0.60206	0.821204
2.36E-14	1.1E-09	9.1E-13	1.28E-17	2.43E-29	4.93E-07	1.59E-11	7.05E-14	3.03E-32
0.416015	0.373824	0.511171	0.375054	0.613018	0.284896	-0.43363	-0.30805	0.611951
1.15E-06	1.5E-05	8.21E-10	1.4E-05	1.85E-14	0.001169	3.53E-07	0.000426	2.11E-14
0.022831	-0.21615	-0.03744	0.00561	-0.11226	0.043041	0.084863	0.124905	-0.19563
0.798889	0.014655	0.676002	0.950084	0.208905	0.630891	0.342816	0.161762	0.027512
-0.06372	-0.17617	-0.20785	-0.08209	-0.21585	0.021957	0.243797	0.35414	-0.11765
0.476643	0.047562	0.019036	0.358856	0.0148	0.806434	0.005743	4.41E-05	0.187728
0.06673	0.17922	-0.38193	0.347517	-0.00779	0.349977	0.180094	-0.19325	-0.04442
0.456025	0.04379	9.39E-06	6.25E-05	0.930763	5.5E-05	0.042756	0.029488	0.620001
0.352995	0.227966	0.065614	0.568918	0.356942	0.480102	-0.27166	-0.33817	0.386814
4.69E-05	0.009947	0.463615	2.99E-12	3.8E-05	1.12E-08	0.002005	0.000101	7.05E-06
-0.23452	-0.21453	-0.43073	0.390903	-0.03328	0.488641	-0.29435	-0.38906	-0.17402
0.007958	0.015433	4.31E-07	5.52E-06	0.710297	5.6E-09	0.000782	6.17E-06	0.050392
-0.54087	-0.43025	-0.67031	-0.24817	-0.6527	-0.06469	0.422211	0.427481	-0.67693
5.22E-11	4.45E-07	6.74E-18	0.004905	9.22E-17	0.469938	7.64E-07	5.37E-07	2.41E-18
-0.49214	-0.46634	-0.23488	-0.58759	-0.51785	-0.3525	0.188434	0.394079	-0.56294
4.19E-09	3.28E-08	0.00786	3.82E-13	4.52E-10	4.82E-05	0.033872	4.56E-06	5.63E-12
-0.46234	-0.41249	-0.45693	-0.18252	-0.4975	-0.12662	0.13607	0.089822	-0.70285
4.45E-08	1.44E-06	6.67E-08	0.039995	2.68E-09	0.156023	0.127165	0.315249	3.25E-20
-0.30524	-0.00652	-0.27562	-0.38419	-0.18909	-0.28997	0.554933	0.34825	-0.19931
0.000484	0.942003	0.001711	8.23E-06	0.033246	0.000944	1.29E-11	6.02E-05	0.02467

NLRP1	NLRP3	NME8	NMI	NNT	NOD2	NPC2	NPL	NR1H3
brown	brown	brown	brown	brown	brown	brown	brown	brown
0.274156	0.281474	0.414821	0.144315	-0.29303	0.133296	0.316649	0.248288	0.257432
0.001815	0.001347	1.24E-06	0.1055	0.000828	0.135177	0.000287	0.004884	0.00348
0.1462	0.15297	0.331888	-0.0019	-0.22096	-0.02674	0.22132	0.171191	0.188473
0.10098	0.085987	0.000138	0.98309	0.012544	0.765357	0.012399	0.054307	0.033835
-0.33162	-0.39327	-0.28239	0.112343	0.347934	-0.02032	-0.36453	-0.4294	-0.0436
0.00014	4.79E-06	0.001297	0.208569	6.12E-05	0.820622	2.52E-05	4.72E-07	0.626437
-0.07602	-0.09041	0.054383	0.447008	0.192025	0.239746	-0.11536	-0.07767	0.05418
0.395639	0.312092	0.543683	1.38E-07	0.030558	0.006632	0.196517	0.385443	0.545186
0.120998	-0.08026	0.173881	0.2354	-0.29194	-0.09511	0.218735	0.091476	0.535908
0.175392	0.369708	0.050573	0.007719	0.000867	0.287495	0.013487	0.306386	8.43E-11
0.17022	0.044154	0.328225	0.627339	-0.09129	0.250104	0.198737	0.179312	0.382455
0.055709	0.622075	0.000165	2.98E-15	0.307361	0.00457	0.025097	0.043681	9.11E-06
0.118024	0.088799	0.2205	0.315114	-0.19415	0.184762	0.124995	0.089461	0.259841
0.186324	0.320817	0.012735	0.000308	0.02873	0.03757	0.161457	0.317209	0.003176
0.370406	0.190124	0.253113	0.333422	-0.13163	0.20337	0.231518	0.355007	0.012887
1.82E-05	0.032276	0.00409	0.000128	0.140156	0.021835	0.00882	4.21E-05	0.885658
0.098517	0.22926	0.158985	0.500118	-0.30366	0.616776	0.418427	0.121073	0.20794
0.270491	0.009522	0.074208	2.14E-09	0.000519	1.16E-14	9.81E-07	0.175121	0.018981
0.667198	0.670721	0.646008	0.750665	-0.62045	0.81565	0.945365	0.797142	0.573925
1.08E-17	6.33E-18	2.38E-16	2.98E-24	7.26E-15	1.7E-31	1.07E-62	3.63E-29	1.74E-12
0.446669	0.530391	0.483198	0.820722	-0.39002	0.682754	0.641496	0.567961	0.43734
1.41E-07	1.42E-10	8.72E-09	3.53E-32	5.82E-06	9.51E-19	4.46E-16	3.31E-12	2.73E-07
-0.57267	-0.01243	-0.1835	-0.27285	-0.20092	-0.06158	-0.0305	-0.19899	-0.10698
2E-12	0.889705	0.038921	0.001912	0.023509	0.491598	0.733582	0.02491	0.231266
-0.26631	-0.17682	-0.36179	-0.28038	0.115485	0.027564	-0.22769	-0.2869	-0.22987
0.002476	0.046736	2.92E-05	0.001409	0.19604	0.758374	0.010038	0.001075	0.009327
-0.1161	-0.17981	-0.2993	-0.18	-0.16766	-0.07483	0.075774	-0.06267	0.161812
0.193669	0.043084	0.00063	0.042861	0.05955	0.403058	0.397157	0.483978	0.069141
0.285186	0.291628	0.12828	0.181332	-0.41731	0.477837	0.528486	0.379719	0.219024
0.001155	0.000879	0.150636	0.041326	1.06E-06	1.34E-08	1.7E-10	1.07E-05	0.013362
0.202829	0.04441	0.176399	-0.39792	-0.07461	-0.25985	0.078556	0.274446	-0.14446
0.022196	0.620054	0.047272	3.61E-06	0.404476	0.003175	0.380004	0.001794	0.105156
-0.52424	-0.45437	-0.58842	-0.78995	0.493727	-0.6841	-0.71566	-0.55831	-0.51924
2.52E-10	8.06E-08	3.47E-13	2.51E-28	3.67E-09	7.65E-19	3.25E-21	9.11E-12	3.99E-10
-0.33388	-0.31307	-0.27886	-0.42457	0.619857	-0.61545	-0.66957	-0.41194	-0.45038
0.000125	0.000339	0.0015	6.53E-07	7.83E-15	1.37E-14	7.55E-18	1.5E-06	1.08E-07
-0.31571	-0.22565	-0.35367	-0.7463	0.3995	-0.70061	-0.56469	-0.39635	-0.50773
0.0003	0.010747	4.53E-05	7.58E-24	3.27E-06	4.8E-20	4.68E-12	3.97E-06	1.11E-09
-0.31941	-0.38477	-0.24533	-0.39864	0.155532	-0.24883	-0.46857	-0.58288	-0.03475
0.000252	7.95E-06	0.005436	3.45E-06	0.080799	0.004788	2.76E-08	6.49E-13	0.698101

NRN1	NRROS	NT5M	NUB1	NUMB	NUPR1	ODF3B	OLFML3	OLR1
brown	brown	brown	brown	brown	brown	brown	brown	brown
-0.12042	0.311025	-0.16561	0.017737	0.148354	0.318224	0.174802	0.192989	0.23124
0.177472	0.000372	0.062776	0.8431	0.096	0.000266	0.049345	0.029716	0.008904
-0.03477	0.175529	-0.01491	-0.10693	0.038245	0.244792	-0.00766	0.004487	0.121076
0.697919	0.048392	0.867889	0.23146	0.669454	0.005542	0.931846	0.960068	0.175112
-0.01845	-0.43448	0.157078	0.062838	-0.08474	-0.3677	-0.318	-0.02022	-0.29477
0.836925	3.33E-07	0.077793	0.482777	0.343525	2.11E-05	0.000269	0.821474	0.000768
0.023291	-0.41554	-0.18531	0.412284	0.316282	-0.29265	-0.26952	0.134056	-0.13858
0.794927	1.18E-06	0.037	1.46E-06	0.000292	0.000841	0.002182	0.132944	0.120235
0.148726	-0.21217	0.362017	0.009316	-0.10065	0.398537	-0.00943	0.098077	-0.0505
0.095161	0.016632	2.89E-05	0.917212	0.260193	3.47E-06	0.91624	0.272648	0.57285
0.09407	-0.39133	-0.05864	0.42092	0.332121	0.074334	-0.15386	0.258636	-0.00897
0.292814	5.38E-06	0.51252	8.33E-07	0.000136	0.406219	0.084158	0.003325	0.92024
0.172217	-0.28368	-0.21744	0.280922	0.282471	0.015098	-0.18319	0.164718	-0.03168
0.052857	0.001229	0.014064	0.001378	0.001293	0.866212	0.03925	0.064229	0.723619
-0.07536	-0.03592	-0.15774	0.295949	0.134963	0.200787	0.368524	0.091297	0.114099
0.399725	0.6885	0.076527	0.00073	0.130317	0.023606	2.02E-05	0.307338	0.201498
-0.09644	0.128148	-0.49824	0.450156	0.514211	0.034668	0.247981	0.624899	-0.11141
0.280761	0.151059	2.51E-09	1.1E-07	6.27E-10	0.698798	0.004939	4.1E-15	0.212416
-0.46623	0.599387	-0.49874	0.490578	0.559281	0.671469	0.698806	0.742578	0.478795
3.31E-08	9.69E-14	2.41E-09	4.77E-09	8.23E-12	5.64E-18	6.56E-20	1.66E-23	1.24E-08
-0.34159	0.423754	-0.4208	0.636485	0.542989	0.448095	0.567792	0.663025	0.434233
8.48E-05	6.9E-07	8.4E-07	8.83E-16	4.25E-11	1.27E-07	3.37E-12	2.03E-17	3.39E-07
0.020816	0.231153	-0.12848	0.048378	0.09377	-0.18348	-0.0783	0.003683	-0.19659
0.816316	0.00893	0.150003	0.589116	0.294363	0.038936	0.381595	0.967223	0.026748
-0.22587	0.198781	-0.04627	-0.12936	-0.12486	-0.1812	0.081667	-0.07558	-0.17559
0.010668	0.025065	0.6055	0.147191	0.161903	0.041481	0.361364	0.398343	0.048309
-0.1767	0.333556	0.348707	-0.22863	-0.35168	0.364435	0.42004	-0.01635	-0.11184
0.046891	0.000127	5.88E-05	0.009726	5.03E-05	2.53E-05	8.83E-07	0.855201	0.210609
-0.51334	0.696728	-0.25826	0.082606	0.134161	0.471309	0.67489	0.399273	0.196887
6.77E-10	9.36E-20	0.003372	0.355851	0.13264	2.24E-08	3.31E-18	3.32E-06	0.026512
-0.26872	0.385284	0.087127	-0.44637	-0.32211	0.17905	0.069442	-0.21426	0.349401
0.002252	7.72E-06	0.330047	1.44E-07	0.000222	0.043994	0.437884	0.01557	5.67E-05
0.113028	-0.11885	0.411449	-0.5914	-0.62969	-0.43294	-0.40952	-0.60063	-0.16272
0.20579	0.183234	1.54E-06	2.47E-13	2.19E-15	3.7E-07	1.75E-06	8.36E-14	0.06758
0.443564	-0.54548	0.262089	-0.25576	-0.2822	-0.59083	-0.63215	-0.53626	-0.13484
1.76E-07	3.32E-11	0.002915	0.003705	0.001308	2.63E-13	1.58E-15	8.15E-11	0.130663
0.427111	-0.22853	0.318677	-0.49941	-0.46209	-0.38328	-0.42294	-0.59934	-0.08706
5.51E-07	0.009758	0.000261	2.28E-09	4.53E-08	8.68E-06	7.28E-07	9.75E-14	0.330401
-0.00606	-0.26457	0.14666	-0.41471	-0.22835	-0.38877	-0.43035	-0.24139	-0.32227
0.946061	0.002648	0.099899	1.25E-06	0.00982	6.28E-06	4.42E-07	0.006256	0.00022

OSCAR	OSGIN1	OSM	OSMR	OSTF1	P2RX4	P2RY10	P2RY13	P2RY6
brown	brown	brown	brown	brown	brown	brown	brown	brown
0.150622	0.34762	0.091673	0.009559	0.466544	0.253098	0.32971	0.335428	0.076936
0.090971	6.22E-05	0.305343	0.91506	3.23E-08	0.004092	0.000154	0.000116	0.389938
0.02752	0.306392	0.050301	-0.05397	0.413949	0.145549	0.126688	0.241479	-0.0766
0.75875	0.000459	0.574379	0.546777	1.31E-06	0.102524	0.155807	0.006238	0.392023
-0.19471	-0.31325	-0.29589	0.089928	-0.68612	-0.23523	-0.20873	-0.45478	-0.00965
0.028262	0.000336	0.000731	0.314676	5.51E-19	0.007765	0.018522	7.82E-08	0.914237
-0.07603	-0.22213	-0.14276	0.446568	-0.40541	-0.12964	0.023922	-0.17993	0.081342
0.395574	0.012073	0.109361	1.42E-07	2.27E-06	0.146325	0.789503	0.042946	0.363289
0.115047	0.255765	0.098963	-0.18392	0.278082	0.261735	0.134159	0.036575	-0.11225
0.197756	0.003705	0.268314	0.038461	0.001548	0.002954	0.132644	0.683096	0.208969
0.075933	0.054663	0.053654	0.368547	0.088476	0.121835	0.232178	0.042451	0.060856
0.396169	0.541609	0.549103	2.01E-05	0.322589	0.172404	0.008623	0.635581	0.496716
0.04466	0.075557	0.027365	0.315218	0.016437	0.13957	0.175965	0.04848	-0.07984
0.618089	0.398519	0.760068	0.000307	0.854475	0.117586	0.047829	0.588326	0.372258
0.123394	0.105029	0.156576	0.227006	0.195162	0.003344	0.241876	0.259213	0.1135
0.166934	0.239926	0.078759	0.010272	0.027892	0.970238	0.00615	0.003253	0.203893
0.431286	0.148043	0.197576	0.685057	0.01942	0.314008	0.385044	-0.11376	0.397217
4.15E-07	0.096707	0.025977	6.55E-19	0.828438	0.000324	7.83E-06	0.202859	3.77E-06
0.834784	0.53873	0.55227	0.554841	0.701272	0.781388	0.762872	0.559676	0.629098
3.4E-34	6.43E-11	1.69E-11	1.3E-11	4.28E-20	2.29E-27	1.96E-25	7.9E-12	2.37E-15
0.564822	0.345147	0.297959	0.511965	0.426303	0.477731	0.655594	0.466846	0.66117
4.62E-12	7.07E-05	0.000668	7.65E-10	5.82E-07	1.35E-08	6.07E-17	3.16E-08	2.68E-17
-0.03951	-0.12738	-0.06751	0.093434	-0.25797	0.005961	-0.22067	-0.17367	-0.05738
0.659163	0.15353	0.450794	0.296105	0.00341	0.946972	0.012665	0.050857	0.52165
0.035258	-0.12246	-0.17427	-0.02025	-0.55642	-0.07618	-0.19999	-0.30843	0.099028
0.693925	0.170204	0.050049	0.821231	1.11E-11	0.394649	0.024173	0.000419	0.268001
0.288242	0.264879	0.033707	-0.31059	-0.08063	0.199955	-0.1027	-0.18093	0.18217
0.001015	0.002617	0.706758	0.00038	0.367515	0.024202	0.250591	0.041783	0.04038
0.633366	0.407928	0.263451	0.198331	0.200336	0.526256	0.308942	0.179252	0.497033
1.34E-15	1.93E-06	0.002766	0.025402	0.023927	2.09E-10	0.000409	0.043752	2.78E-09
-0.04878	0.152374	0.000461	-0.44505	0.44387	0.110175	-0.13235	0.435859	-0.1272
0.58603	0.087231	0.995894	1.58E-07	1.72E-07	0.217541	0.138001	3.03E-07	0.154136
-0.5704	-0.42718	-0.34708	-0.66548	-0.53278	-0.48382	-0.63509	-0.28843	-0.42978
2.55E-12	5.48E-07	6.39E-05	1.4E-17	1.14E-10	8.29E-09	1.07E-15	0.001007	4.59E-07
-0.73001	-0.51934	-0.32523	-0.37211	-0.20633	-0.65525	-0.4471	-0.14551	-0.49933
2.11E-22	3.95E-10	0.000191	1.65E-05	0.019947	6.38E-17	1.37E-07	0.102621	2.29E-09
-0.61547	-0.3491	-0.19417	-0.59923	-0.04291	-0.53692	-0.43797	-0.06377	-0.52271
1.36E-14	5.76E-05	0.028712	9.88E-14	0.631911	7.65E-11	2.61E-07	0.476272	2.9E-10
-0.33488	-0.27066	-0.34336	-0.35982	-0.51101	-0.12346	-0.25661	-0.42021	-0.43355
0.000119	0.002086	7.75E-05	3.25E-05	8.32E-10	0.16669	0.00359	8.73E-07	3.55E-07

P2RY8	P3H2	P4HA2	PALMD	PAM	PANK1	PANK2	PAQR9	PARN
brown	brown	brown	brown	brown	brown	brown	brown	brown
0.321736	0.2165	0.200443	0.270748	0.119008	-0.21951	0.38613	-0.2876	0.240952
0.000226	0.014494	0.023851	0.002079	0.182651	0.013151	7.34E-06	0.001043	0.006355
0.178104	0.137795	0.150666	0.194068	0.050221	-0.1142	0.299546	-0.23191	0.167505
0.045142	0.122371	0.090875	0.028798	0.574988	0.201085	0.000623	0.008702	0.05979
-0.21584	-0.07623	-0.22218	-0.49966	-0.09799	0.386395	-0.19834	0.226417	-0.07594
0.014803	0.394341	0.012055	2.23E-09	0.273056	7.23E-06	0.025394	0.010477	0.396141
-0.10858	0.376409	0.07808	-0.10456	0.370826	0.157197	0.073366	-0.05115	0.219603
0.224311	1.29E-05	0.38291	0.242059	1.77E-05	0.077564	0.412372	0.567923	0.013113
-0.0179	0.104142	0.06236	0.155414	-0.07241	-0.01812	0.031088	-0.19385	-0.18494
0.841642	0.243943	0.486123	0.081032	0.418536	0.839795	0.728618	0.028982	0.037388
0.003072	0.517549	0.25595	0.222087	0.45059	0.025027	0.251438	-0.29282	0.216168
0.972656	4.65E-10	0.003679	0.012091	1.06E-07	0.780015	0.004351	0.000835	0.014649
0.006642	0.450414	0.189526	0.250414	0.341744	-0.04273	0.113775	-0.15615	0.218875
0.94092	1.08E-07	0.032833	0.004518	8.42E-05	0.633348	0.202789	0.07959	0.013426
0.078918	0.471026	0.218641	0.644245	0.292943	-0.3827	0.164223	-0.36947	0.381246
0.377806	2.29E-08	0.013528	3.05E-16	0.000831	8.98E-06	0.065046	1.91E-05	9.77E-06
0.463212	0.465407	0.56173	0.125999	0.447414	-0.3065	0.185143	-0.17883	0.372371
4.16E-08	3.52E-08	6.39E-12	0.158089	1.34E-07	0.000457	0.037172	0.044264	1.63E-05
0.792028	0.662966	0.50944	0.671119	0.405532	-0.71754	0.486481	-0.6737	0.482211
1.45E-28	2.05E-17	9.56E-10	5.95E-18	2.25E-06	2.29E-21	6.68E-09	3.99E-18	9.44E-09
0.504904	0.647774	0.396642	0.504249	0.500708	-0.53377	0.474645	-0.55804	0.489312
1.42E-09	1.86E-16	3.9E-06	1.5E-09	2.04E-09	1.03E-10	1.72E-08	9.36E-12	5.3E-09
-0.05781	-0.09229	0.107121	-0.21462	-0.03833	-0.15984	-0.1505	0.199328	0.025076
0.518561	0.302067	0.23064	0.015391	0.668785	0.072641	0.091228	0.024659	0.779602
0.049948	-0.27157	-0.22444	-0.42029	-0.36706	-0.01561	-0.27153	0.228461	-0.04603
0.577068	0.002012	0.011189	8.68E-07	2.19E-05	0.86172	0.002016	0.009782	0.607326
0.104332	-0.34048	-0.17829	-0.25954	-0.56587	-0.19748	-0.37575	0.055908	-0.39771
0.243078	8.98E-05	0.04492	0.003212	4.13E-12	0.026051	1.34E-05	0.532425	3.65E-06
0.594357	0.129285	0.155495	0.170287	-0.15593	-0.5844	0.020746	-0.30927	0.159687
1.75E-13	0.147435	0.080872	0.055612	0.080024	5.48E-13	0.816919	0.000403	0.072922
0.068	-0.20968	-0.30744	0.167275	-0.34591	-0.11225	-0.05632	-0.05004	-0.15195
0.447477	0.01798	0.000438	0.060147	6.79E-05	0.208932	0.529389	0.576383	0.088116
-0.53651	-0.74011	-0.6932	-0.61766	-0.62699	0.511184	-0.56267	0.624132	-0.56081
7.96E-11	2.76E-23	1.7E-19	1.04E-14	3.12E-15	8.2E-10	5.79E-12	4.53E-15	7.03E-12
-0.64631	-0.37477	-0.31844	-0.2995	0.030018	0.674337	-0.1114	0.466347	-0.30365
2.28E-16	1.42E-05	0.000264	0.000625	0.737614	3.61E-18	0.21245	3.28E-08	0.000519
-0.53339	-0.59673	-0.34639	-0.22652	-0.25293	0.474941	-0.20494	0.498148	-0.3809
1.07E-10	1.33E-13	6.63E-05	0.01044	0.004118	1.68E-08	0.020818	2.53E-09	9.97E-06
-0.17268	-0.47743	-0.37593	-0.53713	-0.51703	0.427333	-0.18143	0.418605	-0.2892
0.052215	1.38E-08	1.33E-05	7.5E-11	4.87E-10	5.42E-07	0.041217	9.7E-07	0.000975

PARP10	PARP15	PARP2	PARVG	PBX1	PCED1B	PCK2	PDCD1	PDCD1LG2
brown	brown	brown	brown	brown	brown	brown	brown	brown
0.070595	0.220712	0.060813	0.244925	-0.28689	0.308014	0.29179	0.264288	0.079756
0.430294	0.012648	0.497024	0.005515	0.001075	0.000427	0.000873	0.002678	0.37275
-0.10951	0.063991	0.175496	0.117952	-0.28442	0.150375	0.189086	0.106565	-0.033
0.220346	0.474768	0.048436	0.186593	0.001192	0.091508	0.033248	0.233082	0.71268
-0.28584	-0.0624	0.185252	-0.35948	0.390084	-0.27206	-0.25931	-0.21329	0.084577
0.001123	0.485856	0.037058	3.31E-05	5.8E-06	0.001973	0.003241	0.016053	0.344454
-0.28867	0.242755	0.095549	-0.21706	0.334911	-0.10558	-0.05253	-0.20247	0.467437
0.000997	0.005961	0.285259	0.014237	0.000119	0.237458	0.557502	0.022442	3.02E-08
-0.18146	-0.04228	0.143239	0.098433	-0.43077	0.072146	0.341388	0.014238	0.221794
0.041175	0.636965	0.108152	0.270902	4.3E-07	0.420211	8.57E-05	0.873771	0.012208
-0.2891	0.280222	0.090757	-0.00162	-0.06323	0.084217	0.275199	-0.0736	0.63345
0.000979	0.001418	0.31022	0.985538	0.480064	0.346518	0.00174	0.410906	1.33E-15
-0.30982	0.190947	0.02405	-0.02888	-0.13508	0.028972	0.099596	-0.13825	0.342481
0.000393	0.031523	0.788404	0.747241	0.129993	0.746437	0.26525	0.12113	8.1E-05
0.129619	0.243497	-0.25853	0.19247	0.135192	0.32183	0.180968	0.04899	0.349305
0.146385	0.005805	0.003338	0.030167	0.12966	0.000225	0.041742	0.584403	5.7E-05
0.25313	0.415676	-0.41692	0.263054	-0.18233	0.316541	0.168872	0.335368	0.448584
0.004087	1.17E-06	1.08E-06	0.002808	0.040202	0.000288	0.057705	0.000116	1.23E-07
0.59892	0.779822	-0.56898	0.895166	-0.60085	0.881762	0.665076	0.680071	0.685587
1.02E-13	3.39E-27	2.97E-12	1.08E-45	8.14E-14	1.3E-42	1.49E-17	1.46E-18	6.01E-19
0.573275	0.661535	-0.35722	0.576379	-0.19842	0.666961	0.589126	0.495936	0.720747
1.87E-12	2.54E-17	3.74E-05	1.33E-12	0.025333	1.12E-17	3.2E-13	3.05E-09	1.26E-21
0.084524	-0.04365	0.024205	-0.03972	-0.01778	-0.12939	-0.1334	-0.03486	-0.21585
0.344755	0.626067	0.787068	0.657492	0.842717	0.147109	0.134857	0.697197	0.014798
0.217371	-0.03152	-0.10286	-0.06195	0.222393	-0.06006	-0.32589	0.044419	-0.28576
0.014094	0.725013	0.249816	0.488992	0.01197	0.502375	0.000185	0.619985	0.001127
0.516107	-0.17567	-0.24686	0.187418	-0.22457	0.125572	0.085262	0.269847	-0.19353
5.29E-10	0.048209	0.005144	0.034863	0.01114	0.159515	0.340545	0.002154	0.029254
0.744584	0.400021	-0.56498	0.636361	-0.35571	0.593225	0.231487	0.560338	0.132821
1.09E-23	3.17E-06	4.54E-12	8.98E-16	4.06E-05	2E-13	0.008829	7.38E-12	0.136586
0.084396	-0.07322	-0.01098	0.25009	-0.23959	0.133234	-0.03615	0.029511	-0.41582
0.345489	0.413276	0.902451	0.004572	0.006669	0.13536	0.68662	0.741888	1.16E-06
-0.269	-0.63631	0.343228	-0.50259	0.430684	-0.58077	-0.55998	-0.42462	-0.77796
0.002228	9.04E-16	7.8E-05	1.73E-09	4.32E-07	8.21E-13	7.66E-12	6.51E-07	5.39E-27
-0.59168	-0.53215	0.51076	-0.67262	0.5003	-0.68064	-0.36431	-0.54319	-0.38281
2.39E-13	1.21E-10	8.51E-10	4.72E-18	2.11E-09	1.34E-18	2.55E-05	4.17E-11	8.92E-06
-0.37892	-0.65537	0.407359	-0.49364	0.245559	-0.56662	-0.3655	-0.41381	-0.70561
1.12E-05	6.27E-17	2E-06	3.7E-09	0.005391	3.82E-12	2.38E-05	1.33E-06	2E-20
-0.51548	-0.17142	0.371006	-0.35813	0.018384	-0.37871	-0.45329	-0.19026	-0.45061
5.59E-10	0.053981	1.76E-05	3.56E-05	0.837454	1.13E-05	8.73E-08	0.032147	1.06E-07

PDE4D	PDE6G	PDE7B	PDLIM3	PDP2	PDZK1IP1	PEG10	PERP	PGAM5
brown	brown	brown	brown	brown	brown	brown	brown	brown
-0.17269	0.163647	-0.27619	0.027236	-0.21799	0.076234	-0.16669	0.341207	-0.34162
0.052195	0.066005	0.001672	0.761159	0.013817	0.394293	0.061065	8.65E-05	8.47E-05
-0.06289	0.106319	-0.16698	0.014376	-0.15405	0.064588	-0.08876	0.228553	-0.23823
0.482439	0.23417	0.060604	0.872553	0.083765	0.470646	0.321022	0.009752	0.006994
0.329819	-0.06849	0.131935	-0.00354	0.165783	-0.3634	0.383945	-0.31858	0.388341
0.000153	0.444198	0.139248	0.968527	0.062502	2.68E-05	8.35E-06	0.000262	6.44E-06
0.243099	-0.12245	0.085056	0.270259	0.397448	-0.30416	0.196969	0.059421	0.105487
0.005888	0.170238	0.341718	0.00212	3.71E-06	0.000508	0.026447	0.506939	0.237874
0.022487	0.289082	-0.16725	0.294554	-0.01656	0.171442	-0.02941	0.038465	0.253251
0.801856	0.00098	0.060192	0.000775	0.853351	0.053949	0.742729	0.667663	0.004069
0.119662	0.077831	-0.06722	0.49881	0.32441	-0.04405	0.043505	0.256605	0.092779
0.180243	0.38443	0.45275	2.39E-09	0.000198	0.622895	0.627212	0.00359	0.299519
0.185012	0.063362	-0.06071	0.246085	0.143751	0.03747	0.105772	0.125561	0.011857
0.037308	0.479132	0.497758	0.00529	0.106884	0.675773	0.236598	0.159549	0.894739
-0.36387	-0.03493	-0.07023	0.461488	0.200428	0.281682	-0.12698	0.157756	-0.3111
2.61E-05	0.696615	0.432669	4.74E-08	0.023862	0.001336	0.154862	0.076502	0.000371
-0.03773	0.302479	-0.11241	0.188377	-0.32657	0.089697	-0.20014	0.40059	-0.19627
0.673614	0.000547	0.208279	0.033927	0.000179	0.315925	0.024068	3.06E-06	0.027
-0.50788	0.535201	-0.55436	0.397197	-0.51649	0.526535	-0.60186	0.477992	-0.5487
1.1E-09	9.02E-11	1.36E-11	3.77E-06	5.11E-10	2.04E-10	7.22E-14	1.32E-08	2.41E-11
-0.46291	0.335877	-0.38259	0.397371	-0.13527	0.214635	-0.4067	0.458561	-0.46418
4.26E-08	0.000113	9.04E-06	3.73E-06	0.129435	0.015384	2.09E-06	5.91E-08	3.87E-08
0.034634	-0.01975	0.001486	-0.29324	-0.14746	-0.22137	-0.0371	0.1262	-0.13496
0.699079	0.825604	0.986774	0.00082	0.098046	0.012378	0.678761	0.157422	0.130332
0.008889	-0.00645	-0.00807	-0.33893	-0.25431	-0.15146	0.045127	-0.34532	-0.02239
0.920989	0.942583	0.928221	9.71E-05	0.003912	0.089156	0.614414	7E-05	0.802692
-0.2351	0.355696	-0.14807	-0.10638	-0.40686	0.231091	-0.23315	-0.32934	0.153573
0.007798	4.06E-05	0.096641	0.233897	2.07E-06	0.008949	0.008342	0.000156	0.084743
-0.49641	0.44063	-0.3757	0.006357	-0.65228	0.379951	-0.47657	0.023653	-0.41741
2.93E-09	2.17E-07	1.35E-05	0.943448	9.8E-17	1.05E-05	1.48E-08	0.791817	1.05E-06
-0.30383	-0.07399	-0.07285	-0.20265	-0.25282	0.217781	-0.1096	-0.12099	-0.2289
0.000516	0.408401	0.415676	0.022314	0.004135	0.013909	0.219974	0.175407	0.009639
0.235038	-0.36765	0.347008	-0.51275	0.069373	-0.34324	0.460897	-0.61205	0.377418
0.007816	2.12E-05	6.42E-05	7.14E-10	0.438339	7.8E-05	4.96E-08	2.09E-14	1.22E-05
0.464364	-0.53584	0.498142	-0.22262	0.632001	-0.41961	0.447037	-0.11294	0.405946
3.82E-08	8.49E-11	2.53E-09	0.011881	1.61E-15	9.08E-07	1.37E-07	0.206131	2.19E-06
0.280914	-0.44241	0.366021	-0.43136	0.2531	-0.17847	0.322947	-0.20417	0.284951
0.001379	1.91E-07	2.32E-05	4.13E-07	0.004092	0.044691	0.000213	0.021311	0.001166
0.466155	-0.10146	0.051177	-0.45374	-0.05289	-0.48017	0.458965	-0.35325	0.324324
3.33E-08	0.256365	0.56772	8.44E-08	0.554834	1.11E-08	5.73E-08	4.63E-05	0.000199

PGBD1	PHF11	PICALM	PIGT	PIK3AP1	PIK3CD	PIK3CG	PIK3R5	PILRA
brown	brown	brown	brown	brown	brown	brown	brown	brown
-0.111	0.43544	0.158401	0.256556	0.322898	0.399647	0.129522	0.240675	0.369325
0.214087	3.12E-07	0.07529	0.003596	0.000213	3.24E-06	0.146687	0.006418	1.93E-05
0.004914	0.38065	0.10917	0.201124	0.135698	0.246177	-0.03817	0.124948	0.233925
0.956269	1.01E-05	0.221791	0.023368	0.128218	0.005272	0.670029	0.161615	0.008122
0.542037	-0.54953	-0.1145	-0.1796	-0.21226	-0.50921	-0.15376	-0.34316	-0.41469
4.66E-11	2.22E-11	0.199912	0.04334	0.016585	9.76E-10	0.084367	7.83E-05	1.25E-06
0.43715	-0.1125	0.209421	-0.0259	0.094951	-0.43367	0.219571	-0.13814	-0.19259
2.77E-07	0.207925	0.018128	0.772587	0.288298	3.52E-07	0.013127	0.121437	0.030063
-0.14276	0.224035	-0.26938	0.06084	0.078637	-0.17441	0.000915	0.005548	0.145123
0.109361	0.01134	0.002195	0.496833	0.379512	0.049869	0.991854	0.950638	0.103544
0.147889	0.273756	0.170125	0.108153	0.289219	-0.32593	0.341688	0.02767	0.061168
0.09706	0.001844	0.055848	0.226155	0.000974	0.000184	8.44E-05	0.757476	0.494512
0.055891	0.278629	0.161561	0.254876	0.145898	-0.19402	0.148524	0.046533	0.122965
0.532551	0.001514	0.069579	0.00383	0.101693	0.028836	0.095616	0.603412	0.168427
-0.28376	0.376302	0.16797	0.026814	0.281585	0.063	0.303634	0.180452	0.2215
0.001225	1.3E-05	0.059074	0.764754	0.001341	0.481651	0.00052	0.042338	0.012326
-0.0622	0.084299	0.408911	0.545709	0.394761	0.224508	0.440683	0.367728	0.249188
0.487253	0.346044	1.82E-06	3.25E-11	4.38E-06	0.011163	2.16E-07	2.11E-05	0.004726
-0.5171	0.725992	0.55728	0.674252	0.891556	0.688601	0.788694	0.900794	0.872192
4.84E-10	4.61E-22	1.01E-11	3.66E-18	8E-45	3.66E-19	3.5E-28	4.11E-47	1.24E-40
-0.33524	0.662557	0.430107	0.415727	0.750256	0.49252	0.698358	0.557186	0.615223
0.000117	2.18E-17	4.49E-07	1.17E-06	3.25E-24	4.06E-09	7.08E-20	1.02E-11	1.41E-14
0.108871	-0.17947	0.018	0.138769	-0.19407	0.09843	-0.02556	-0.0529	-0.05961
0.22307	0.043489	0.840812	0.119726	0.028798	0.270917	0.775458	0.554711	0.505564
0.230645	-0.47932	-0.05925	0.023731	-0.241	0.062601	-0.24452	-0.04938	-0.11431
0.009086	1.19E-08	0.508152	0.791145	0.006344	0.484431	0.005596	0.581437	0.200665
-0.2461	-0.24138	-0.384	0.097991	-0.14571	0.138787	-0.21673	0.080363	0.10213
0.005288	0.006259	8.32E-06	0.273068	0.102131	0.119677	0.014388	0.369108	0.253224
-0.35902	0.166999	0.242774	0.489634	0.358132	0.618362	0.267931	0.612269	0.553753
3.4E-05	0.060577	0.005957	5.16E-09	3.56E-05	9.47E-15	0.002323	2.03E-14	1.45E-11
-0.19282	0.255205	0.129512	-0.06906	-0.02366	0.355113	-0.13504	0.177446	0.163331
0.029866	0.003783	0.146719	0.440396	0.791791	4.19E-05	0.130091	0.045954	0.066537
0.275065	-0.6587	-0.45965	-0.61254	-0.66192	-0.29608	-0.65837	-0.56833	-0.58112
0.001749	3.86E-17	5.44E-08	1.97E-14	2.4E-17	0.000725	4.05E-17	3.18E-12	7.91E-13
0.29653	-0.29371	-0.26623	-0.63045	-0.46866	-0.52356	-0.36474	-0.65729	-0.66359
0.000711	0.000804	0.002483	1.98E-15	2.75E-08	2.69E-10	2.49E-05	4.74E-17	1.87E-17
0.027343	-0.26098	-0.39755	-0.53537	-0.5318	-0.1835	-0.53098	-0.52724	-0.47209
0.760251	0.003042	3.69E-06	8.88E-11	1.25E-10	0.038918	1.35E-10	1.91E-10	2.11E-08
0.48076	-0.57339	-0.21904	-0.21927	-0.37758	-0.25801	-0.43101	-0.31663	-0.36766
1.06E-08	1.85E-12	0.013355	0.013257	1.21E-05	0.003405	4.23E-07	0.000287	2.12E-05

PIM1	PLA2G15	PLA2G2A	PLA2G2D	PLA2G4A	PLA2G7	PLAUR	PLB1	PLBD1
brown	brown	brown	brown	brown	brown	brown	brown	brown
0.182908	0.428956	0.008968	0.351064	0.013211	0.240807	0.21937	0.213432	0.316324
0.039563	4.86E-07	0.920293	5.19E-05	0.882809	0.006388	0.013212	0.015983	0.000291
0.04697	0.293036	-0.04123	0.196052	-0.12261	0.19266	0.11755	0.063285	0.196857
0.60001	0.000828	0.645321	0.027172	0.169654	0.030002	0.188111	0.479666	0.026535
-0.10967	-0.33611	-0.00614	-0.19042	0.131406	-0.25041	-0.02215	-0.24008	-0.07972
0.219657	0.000112	0.945354	0.032007	0.140852	0.004519	0.804796	0.006554	0.372937
0.041075	-0.27154	0.200732	-0.07859	0.504836	-0.21861	0.130967	0.109938	0.254617
0.646588	0.002015	0.023645	0.379783	1.43E-09	0.01354	0.142195	0.21854	0.003867
-0.03095	0.126168	0.049289	0.170675	0.158249	0.092201	0.109216	0.060323	0.314957
0.729756	0.157528	0.582113	0.055049	0.075575	0.302553	0.221599	0.500501	0.00031
0.096552	-0.0398	0.260964	0.08859	0.605271	-0.0524	0.262555	0.284655	0.528809
0.280207	0.656871	0.003043	0.321962	4.79E-14	0.558487	0.002863	0.00118	1.65E-10
0.059646	0.007373	0.091163	0.089172	0.324133	-0.13698	0.213553	0.235548	0.408492
0.50533	0.934432	0.308053	0.31878	0.000201	0.12461	0.015922	0.007679	1.86E-06
0.029152	-0.02411	0.368616	-0.05691	0.527442	-0.04716	0.107656	0.406935	0.272221
0.744917	0.787912	2.01E-05	0.52512	1.88E-10	0.598508	0.228307	2.06E-06	0.001961
0.429335	0.243361	0.515421	0.161502	0.308048	-0.19443	0.610029	0.326753	0.53334
4.74E-07	0.005833	5.62E-10	0.069683	0.000426	0.028493	2.68E-14	0.000177	1.08E-10
0.62936	0.793954	0.56698	0.538098	0.382987	0.378348	0.753422	0.72794	0.825815
2.29E-15	8.65E-29	3.68E-12	6.83E-11	8.83E-06	1.16E-05	1.63E-24	3.16E-22	6.89E-33
0.515422	0.567366	0.435639	0.544504	0.516615	0.194607	0.555388	0.555498	0.655639
5.62E-10	3.53E-12	3.07E-07	3.66E-11	5.05E-10	0.028348	1.23E-11	1.21E-11	6.03E-17
0.051711	0.097343	-0.06447	0.057105	-0.34159	-0.21564	-0.04608	-0.28268	-0.21483
0.563684	0.276266	0.47148	0.523669	8.48E-05	0.0149	0.60697	0.001281	0.015291
0.017315	-0.04512	-0.04877	-0.11107	-0.35837	-0.08225	-0.03803	-0.22796	-0.29638
0.846794	0.61447	0.586114	0.213806	3.52E-05	0.357926	0.671236	0.009949	0.000716
0.075233	0.183921	0.051198	0.109339	-0.38429	0.090563	0.034446	-0.17464	-0.17652
0.400547	0.038463	0.567567	0.221073	8.18E-06	0.311261	0.700636	0.049564	0.047113
0.412757	0.572604	0.32375	0.282416	-0.17193	0.265179	0.42601	0.254023	0.255016
1.42E-06	2.01E-12	0.000205	0.001296	0.053254	0.002587	5.93E-07	0.003954	0.00381
-0.22141	0.232023	-0.24519	0.033244	-0.40415	0.459347	-0.30623	-0.18271	-0.19693
0.012364	0.008669	0.005463	0.710604	2.45E-06	5.57E-08	0.000463	0.039779	0.026476
-0.45713	-0.41717	-0.55553	-0.33519	-0.55491	-0.11073	-0.64188	-0.71318	-0.81024
6.57E-08	1.07E-06	1.21E-11	0.000117	1.29E-11	0.215221	4.22E-16	5.12E-21	8.67E-31
-0.47111	-0.61648	-0.46624	-0.32714	-0.03283	-0.21774	-0.6116	-0.38139	-0.52709
2.27E-08	1.2E-14	3.31E-08	0.000174	0.714061	0.013928	2.21E-14	9.69E-06	1.94E-10
-0.45221	-0.41465	-0.54779	-0.29565	-0.40245	-0.13577	-0.63312	-0.43652	-0.68944
9.44E-08	1.26E-06	2.64E-11	0.000739	2.73E-06	0.128014	1.39E-15	2.89E-07	3.19E-19
-0.29665	-0.19844	-0.45251	-0.14109	-0.43763	-0.03331	-0.19404	-0.4326	-0.26686
0.000708	0.025321	9.24E-08	0.113607	2.68E-07	0.71008	0.028821	3.79E-07	0.002423

PLCB2	PLCB4	PLCG2	PLCXD1	PLD6	PLEK	PLEKHO2	PLIN2	PLK3
brown	brown	brown	brown	brown	brown	brown	brown	brown
0.219253	-0.03555	0.191472	-0.19386	0.099797	0.387726	0.266326	0.069304	0.108355
0.013263	0.691523	0.031049	0.028974	0.264281	6.68E-06	0.002474	0.438797	0.225281
0.079534	0.033872	0.034656	-0.17309	0.15747	0.208254	0.13029	0.031516	0.032059
0.374081	0.705389	0.6989	0.051646	0.077044	0.018798	0.144288	0.725032	0.720488
-0.40459	0.065986	-0.33623	0.43322	-0.16355	-0.3168	-0.3688	0.073714	-0.26666
2.39E-06	0.461078	0.000111	3.63E-07	0.066172	0.000285	1.99E-05	0.410153	0.002442
-0.33766	-0.07248	-0.12935	0.161432	-0.23502	-0.01243	-0.33103	0.297734	-0.08606
0.000104	0.418076	0.147227	0.069805	0.00782	0.889663	0.000144	0.000675	0.336016
-0.25426	-0.13009	-0.10589	-0.2051	-0.22979	0.099134	-0.11437	0.234409	0.010413
0.003919	0.144922	0.236088	0.020714	0.009355	0.267487	0.200428	0.007987	0.907502
-0.32838	-0.1839	-0.03408	-0.1629	-0.37444	0.228226	-0.23526	0.44085	0.042196
0.000164	0.038487	0.703682	0.067276	1.45E-05	0.00986	0.007757	2.13E-07	0.637618
-0.19115	-0.08424	-0.04696	-0.08796	0.023031	0.149244	-0.16596	0.328059	0.092952
0.031341	0.346371	0.600118	0.325414	0.797166	0.094001	0.062219	0.000166	0.298614
0.120913	0.0168	0.265324	-0.48189	-0.2352	0.291828	0.082438	0.036586	0.167833
0.175697	0.851293	0.002572	9.69E-09	0.007773	0.000872	0.356834	0.683007	0.059285
0.183162	-0.31156	0.316276	-0.07611	-0.30061	0.294854	0.292087	0.597417	0.51093
0.039284	0.000363	0.000292	0.395042	0.000595	0.000765	0.000862	1.22E-13	8.39E-10
0.706413	-0.5184	0.828225	-0.563	-0.42143	0.86997	0.774576	0.558922	0.783251
1.73E-20	4.3E-10	3.12E-33	5.59E-12	8.05E-07	3.38E-40	1.24E-26	8.55E-12	1.43E-27
0.475723	-0.43622	0.609258	-0.41345	-0.36365	0.735578	0.531811	0.319797	0.475789
1.58E-08	2.95E-07	2.95E-14	1.36E-06	2.64E-05	6.95E-23	1.25E-10	0.000247	1.58E-08
0.12401	-0.09736	-0.0455	0.147344	0.397319	-0.20023	0.005254	-0.05356	0.058805
0.164812	0.276194	0.611468	0.098311	3.74E-06	0.024006	0.953248	0.549832	0.511367
0.156093	0.042381	-0.03404	0.370386	0.177244	-0.27108	0.111352	-0.13683	0.012176
0.079698	0.636143	0.704039	1.82E-05	0.046207	0.002051	0.212635	0.125033	0.891924
0.1856	-0.19517	0.013182	0.069802	-0.06995	-0.16135	0.281856	-0.09998	0.211365
0.036697	0.027883	0.883063	0.435506	0.434506	0.069943	0.001326	0.263381	0.017059
0.676668	-0.36124	0.558963	-0.18698	-0.1323	0.341821	0.712099	0.176057	0.605772
2.51E-18	3.01E-05	8.51E-12	0.0353	0.138133	8.38E-05	6.24E-21	0.047711	4.51E-14
0.363366	0.073297	0.183578	-0.17288	0.285368	0.048581	0.239238	-0.41047	-0.09043
2.68E-05	0.412815	0.038833	0.051939	0.001146	0.58755	0.006751	1.64E-06	0.31195
-0.22229	0.411325	-0.46637	0.438211	0.437255	-0.61376	-0.34435	-0.63913	-0.58331
0.012011	1.56E-06	3.27E-08	2.57E-07	2.75E-07	1.69E-14	7.36E-05	6.17E-16	6.19E-13
-0.54758	0.463693	-0.55242	0.27484	0.25187	-0.45701	-0.65914	-0.44255	-0.68155
2.7E-11	4.01E-08	1.66E-11	0.001765	0.004282	6.63E-08	3.61E-17	1.89E-07	1.15E-18
-0.25317	0.485133	-0.4214	0.188798	0.432415	-0.43775	-0.37383	-0.57789	-0.55166
0.004082	7.46E-09	8.07E-07	0.033523	3.84E-07	2.65E-07	1.5E-05	1.13E-12	1.79E-11
-0.35881	0.368304	-0.39793	0.516927	0.392255	-0.38778	-0.36456	-0.0116	-0.40505
3.44E-05	2.04E-05	3.61E-06	4.91E-10	5.09E-06	6.66E-06	2.51E-05	0.897043	2.32E-06

PLSCR1	PLTP	PLXDC2	PLXNA2	PML	PMP22	PNP	PNRC1	PODNL1
brown	brown	brown	brown	brown	brown	brown	brown	brown
0.165955	0.006579	0.295858	-0.25644	0.177408	0.269113	-0.12112	0.155652	0.149241
0.062227	0.941481	0.000732	0.003612	0.046002	0.002218	0.174947	0.080563	0.094009
-0.00759	-0.08935	0.157199	-0.16731	0.047977	0.278803	-0.08893	0.104878	0.101191
0.932467	0.317816	0.077561	0.060084	0.592214	0.001503	0.320085	0.240608	0.257635
0.062993	-0.29625	-0.42731	0.07196	-0.18892	-0.20731	0.155929	-0.18092	-0.29615
0.481695	0.00072	5.43E-07	0.421414	0.033407	0.019356	0.080019	0.041796	0.000723
0.43617	-0.08866	-0.18707	-0.23985	-0.19154	0.129416	0.276019	0.091073	-0.06865
2.96E-07	0.321567	0.035207	0.006607	0.030991	0.147022	0.001683	0.308529	0.443163
0.01575	-0.163	-0.11007	-0.51191	-0.25287	-0.0242	0.291578	0.046776	0.128781
0.860492	0.067103	0.217999	7.69E-10	0.004128	0.787082	0.000881	0.601519	0.149034
0.491095	-0.05931	-0.04017	-0.60396	-0.24361	0.224957	0.418157	0.221155	0.146421
4.57E-09	0.507721	0.65388	5.61E-14	0.005781	0.010998	9.99E-07	0.012466	0.100459
0.264468	0.082082	-0.12711	-0.31209	-0.13901	0.278337	0.463941	0.017948	0.160448
0.002659	0.358925	0.154421	0.000354	0.119075	0.001532	3.94E-08	0.841265	0.07155
0.302304	0.351722	0.340533	-0.22002	-0.10791	0.062023	0.11836	0.120937	0.175183
0.000552	5.02E-05	8.95E-05	0.012937	0.227198	0.488485	0.185062	0.175612	0.048844
0.490327	0.515563	0.143199	-0.11674	0.551036	0.269288	0.421366	0.34959	0.435747
4.87E-09	5.55E-10	0.10825	0.19121	1.91E-11	0.002202	8.09E-07	5.61E-05	3.05E-07
0.664407	0.772464	0.716662	-0.54441	0.65611	0.507452	0.378217	0.55738	0.66596
1.65E-17	2.06E-26	2.7E-21	3.7E-11	5.63E-17	1.14E-09	1.16E-05	1E-11	1.31E-17
0.832607	0.502365	0.543428	-0.4623	0.602807	0.418259	0.237228	0.354093	0.411258
7.18E-34	1.77E-09	4.07E-11	4.46E-08	6.45E-14	9.92E-07	0.007243	4.42E-05	1.56E-06
-0.1563	0.095814	-0.19481	0.229551	0.284283	0.074875	-0.03215	-0.05314	0.025013
0.079288	0.283916	0.028183	0.009429	0.001199	0.402798	0.719755	0.552942	0.780141
-0.22164	0.094842	-0.14917	0.472806	0.294446	-0.1404	-0.16372	-0.1638	-0.14996
0.012271	0.288854	0.094165	1.99E-08	0.000779	0.115401	0.065878	0.065755	0.092423
-0.25998	0.144712	-0.04125	0.162185	0.322179	-0.12547	-0.10337	-0.03894	0.115315
0.00316	0.104535	0.645222	0.068494	0.000221	0.159869	0.247459	0.663778	0.196706
0.127916	0.633402	0.441786	0.042236	0.733652	0.242193	0.036227	0.238639	0.419532
0.151806	1.34E-15	2E-07	0.637301	1.02E-22	0.006081	0.68595	0.006895	9.13E-07
-0.37697	-0.09058	0.292938	0.139463	-0.06624	0.150294	-0.36043	-0.20003	0.002634
1.25E-05	0.311192	0.000831	0.117869	0.459331	0.091685	3.15E-05	0.024149	0.976553
-0.67947	-0.54246	-0.4407	0.643472	-0.40928	-0.451	-0.37272	-0.56965	-0.60963
1.61E-18	4.47E-11	2.16E-07	3.39E-16	1.77E-06	1.03E-07	1.59E-05	2.76E-12	2.82E-14
-0.27155	-0.65645	-0.37102	0.218517	-0.68207	-0.31591	-0.30507	-0.29561	-0.52507
0.002014	5.36E-17	1.75E-05	0.013582	1.06E-18	0.000297	0.000488	0.00074	2.34E-10
-0.57849	-0.49892	-0.2779	0.45102	-0.46725	-0.42497	-0.40961	-0.39257	-0.46642
1.06E-12	2.37E-09	0.001559	1.03E-07	3.06E-08	6.36E-07	1.74E-06	5E-06	3.26E-08
-0.48992	-0.52906	-0.55714	0.193595	-0.29249	-0.38272	-0.05138	-0.30442	-0.33335
5.04E-09	1.61E-10	1.03E-11	0.029197	0.000847	8.97E-06	0.566154	0.000502	0.000128

POLD4	POLR3B	POMGNT2	POU2AF1	PPARG	PPIF	PPM1M	PPP1R14C	PRDM10
brown	brown	brown	brown	brown	brown	brown	brown	brown
0.215925	-0.10541	-0.2373	0.388364	0.38614	0.2097	0.289939	-0.16517	-0.19967
0.014763	0.238203	0.007224	6.43E-06	7.34E-06	0.017972	0.000945	0.063499	0.02441
0.216624	0.013949	-0.12456	0.339221	0.270406	0.248115	0.19519	-0.05646	-0.18989
0.014437	0.876305	0.162932	9.57E-05	0.002107	0.004914	0.027869	0.528371	0.032491
-0.35211	0.348259	0.148564	-0.48602	-0.27596	-0.33005	-0.39439	0.195166	0.432131
4.92E-05	6.01E-05	0.095526	6.93E-09	0.001687	0.000151	4.47E-06	0.027889	3.91E-07
-0.36717	0.319265	-0.24898	-0.30452	0.001585	-0.39808	-0.04984	0.040982	0.515427
2.17E-05	0.000254	0.004762	0.0005	0.985891	3.57E-06	0.577916	0.647331	5.62E-10
0.259633	-0.04397	0.114717	0.11834	0.317132	0.311366	0.441173	0.180991	-0.26731
0.003201	0.623559	0.199052	0.185137	0.00028	0.000366	2.09E-07	0.041716	0.00238
-0.09407	0.181453	-0.31331	-0.01407	0.335707	-0.06563	0.380469	0.077789	0.226873
0.2928	0.041188	0.000335	0.875283	0.000114	0.463507	1.02E-05	0.384691	0.010318
0.045839	0.031267	-0.1147	-0.03056	0.248526	-0.04631	0.323043	0.101915	0.090061
0.608834	0.727121	0.199105	0.733014	0.004841	0.605148	0.000212	0.25423	0.313961
-0.16298	0.028465	-0.14171	0.181835	0.133218	-0.0318	0.414443	-0.03505	-0.07831
0.06713	0.750733	0.112008	0.040756	0.135407	0.722647	1.27E-06	0.695658	0.381513
0.324702	-0.27336	-0.39147	-0.00814	0.220167	-0.16621	0.129898	-0.26958	-0.03103
0.000196	0.001873	5.34E-06	0.92765	0.012874	0.061823	0.14551	0.002177	0.729082
0.64687	-0.60638	-0.62381	0.627995	0.661847	0.425307	0.684797	-0.50404	-0.51763
2.11E-16	4.19E-14	4.72E-15	2.74E-15	2.42E-17	6.22E-07	6.83E-19	1.53E-09	4.61E-10
0.295225	-0.23329	-0.5897	0.421895	0.532585	0.20422	0.559641	-0.50788	-0.22839
0.000753	0.008302	3E-13	7.81E-07	1.16E-10	0.021278	7.93E-12	1.1E-09	0.009807
0.471002	-0.05851	0.113863	-0.17836	-0.1418	-0.06528	-0.27449	-0.18389	-0.05226
2.29E-08	0.513473	0.202439	0.04483	0.11179	0.465921	0.001791	0.038499	0.559514
-0.00647	-0.07892	0.223469	-0.21666	-0.37382	-0.19	-0.49554	-0.13722	0.04566
0.942471	0.377791	0.011554	0.014419	1.5E-05	0.032394	3.16E-09	0.123944	0.610234
0.342614	-0.34149	0.301708	0.088322	-0.17006	0.207172	-0.06991	-0.16791	-0.44704
8.05E-05	8.52E-05	0.000567	0.323432	0.055937	0.019437	0.434811	0.059158	1.37E-07
0.563397	-0.61232	-0.16843	0.390954	0.142439	0.284714	0.129007	-0.51053	-0.5395
5.36E-12	2.02E-14	0.058374	5.51E-06	0.110156	0.001178	0.148317	8.69E-10	5.97E-11
0.157876	-0.16532	0.06983	0.397336	0.100781	0.45255	0.002659	-0.12178	-0.39078
0.076275	0.063243	0.43532	3.74E-06	0.259576	9.21E-08	0.976334	0.172614	5.56E-06
-0.3733	0.295327	0.663795	-0.45187	-0.59067	-0.08284	-0.68417	0.352913	0.097663
1.54E-05	0.00075	1.81E-17	9.68E-08	2.68E-13	0.354503	7.57E-19	4.71E-05	0.274686
-0.66955	0.590132	0.194627	-0.37398	-0.30685	-0.33953	-0.32341	0.454992	0.574099
7.57E-18	2.85E-13	0.028332	1.48E-05	0.00045	9.42E-05	0.000208	7.7E-08	1.71E-12
-0.34597	0.315296	0.442373	-0.21991	-0.36719	-0.05803	-0.33628	0.419316	0.138086
6.77E-05	0.000305	1.92E-07	0.012981	2.17E-05	0.516988	0.000111	9.26E-07	0.121575
-0.14304	0.088063	0.479522	-0.38621	-0.18981	-0.20389	-0.45145	0.41918	0.278666
0.108656	0.32486	1.17E-08	7.31E-06	0.032569	0.021495	9.98E-08	9.34E-07	0.001512

PRDM11	PRDM12	PRDX6	PRKCH	PROCR	PROS1	PRR13	PRR3	PRRG4
brown	brown	brown	brown	brown	brown	brown	brown	brown
0.000135	-0.00019	0.193653	0.115513	-0.05844	0.096037	0.470003	0.052613	0.077345
0.998802	0.998303	0.029148	0.195933	0.513963	0.282793	2.48E-08	0.556895	0.387414
0.036325	0.0782	0.198423	0.024014	-0.15188	-0.02038	0.472731	0.168132	-0.06715
0.685149	0.382175	0.025333	0.788714	0.088265	0.820116	2E-08	0.058826	0.453195
0.016211	-0.00323	-0.32744	-0.27192	0.021792	0.198681	-0.61647	0.461126	-0.10894
0.856449	0.971278	0.000171	0.001985	0.807866	0.025139	1.2E-14	4.87E-08	0.222781
-0.16495	-0.12987	0.075431	-0.11226	0.267772	0.57356	-0.44313	0.313209	0.132616
0.063852	0.145611	0.399306	0.208922	0.002338	1.81E-12	1.82E-07	0.000337	0.1372
-0.42349	-0.04601	0.203177	-0.19422	0.434459	0.091655	0.448344	0.32225	0.082026
7.02E-07	0.60746	0.021964	0.028668	3.33E-07	0.305436	1.25E-07	0.00022	0.359253
-0.46032	-0.19496	0.366476	-0.08019	0.555311	0.614447	0.066738	0.332134	0.288246
5.18E-08	0.028055	2.26E-05	0.370153	1.24E-11	1.55E-14	0.455973	0.000136	0.001015
-0.19888	0.013017	0.29685	0.033403	0.399086	0.423595	0.151252	0.293955	-0.06917
0.024991	0.88451	0.000702	0.709289	3.36E-06	6.97E-07	0.089612	0.000795	0.439672
-0.29552	-0.25835	0.60859	-0.02435	0.197252	0.36209	0.084689	-0.14949	0.29685
0.000743	0.003361	3.2E-14	0.785805	0.026228	2.88E-05	0.343809	0.093458	0.000702
-0.38912	-0.2427	0.130167	0.48466	0.499673	0.62408	0.060512	-0.18829	0.239617
6.15E-06	0.005972	0.14467	7.75E-09	2.23E-09	4.56E-15	0.499162	0.034013	0.006662
-0.63282	-0.49072	0.577505	0.64154	0.567427	0.62354	0.696368	-0.47273	0.619883
1.45E-15	4.72E-09	1.18E-12	4.43E-16	3.51E-12	4.89E-15	9.95E-20	2E-08	7.81E-15
-0.35558	-0.38742	0.455633	0.409273	0.438367	0.596339	0.395937	-0.24075	0.507143
4.09E-05	6.8E-06	7.34E-08	1.77E-06	2.54E-07	1.39E-13	4.07E-06	0.0064	1.17E-09
0.224111	0.111156	-0.1874	0.08148	-0.18855	-0.17569	0.093907	0.044704	-0.34717
0.011311	0.213449	0.034876	0.36247	0.033757	0.048184	0.293658	0.617738	6.36E-05
0.290231	0.094973	-0.41898	0.024469	-0.34871	-0.17942	-0.39801	-0.05288	-0.24949
0.000933	0.288183	9.47E-07	0.784799	5.88E-05	0.043552	3.59E-06	0.554918	0.004675
-0.10504	0.057744	-0.29886	-0.05735	-0.04168	-0.35954	0.089854	-0.26213	-0.02801
0.239892	0.519024	0.000643	0.521914	0.641768	3.3E-05	0.315076	0.00291	0.754622
-0.22499	-0.20703	0.049616	0.478095	0.082542	0.076387	0.307832	-0.52724	0.222426
0.010985	0.019522	0.57961	1.31E-08	0.356228	0.393344	0.00043	1.91E-10	0.011957
0.220077	0.025643	-0.04595	0.115686	-0.40142	-0.50216	0.335414	-0.24309	-0.09844
0.012912	0.774747	0.607982	0.195257	2.91E-06	1.8E-09	0.000116	0.005891	0.270878
0.626332	0.345306	-0.65319	-0.40097	-0.68581	-0.74624	-0.50687	0.200818	-0.57626
3.4E-15	7.01E-05	8.59E-17	2.99E-06	5.79E-19	7.67E-24	1.2E-09	0.023584	1.35E-12
0.452508	0.275112	-0.23424	-0.44533	-0.34604	-0.36669	-0.4529	0.268847	-0.21702
9.24E-08	0.001746	0.008034	1.55E-07	6.75E-05	2.23E-05	8.98E-08	0.002241	0.014253
0.560313	0.350094	-0.28033	-0.33612	-0.5298	-0.701	-0.16196	0.089924	-0.41448
7.4E-12	5.46E-05	0.001412	0.000112	1.51E-10	4.48E-20	0.068881	0.314701	1.27E-06
0.252062	0.194613	-0.59367	-0.18354	-0.26939	-0.22073	-0.28144	0.553327	-0.41609
0.004252	0.028344	1.9E-13	0.038877	0.002193	0.012641	0.001349	1.52E-11	1.14E-06

PRSS23	PSD4	PSMB10	PSMB8	PSMB9	PSME1	PSME2	PSTPIP1	PTAFR
brown	brown	brown	brown	brown	brown	brown	brown	brown
0.211086	0.306313	0.210681	0.204884	0.183889	0.387162	0.298048	0.205298	0.172403
0.017209	0.000461	0.017429	0.020851	0.038498	6.9E-06	0.000666	0.020589	0.052598
0.127588	0.185817	0.155218	0.089209	0.037583	0.309415	0.207935	0.132909	0.020099
0.152867	0.036475	0.081421	0.318579	0.674848	0.000401	0.018984	0.136326	0.822531
-0.00105	-0.61323	-0.46558	0.007272	0.028334	-0.14353	0.028928	-0.41759	-0.29451
0.99061	1.8E-14	3.48E-08	0.93533	0.751842	0.107437	0.746813	1.04E-06	0.000776
0.270755	-0.48871	-0.54224	0.293368	0.198754	0.024851	0.197708	-0.27384	-0.1385
0.002078	5.57E-09	4.57E-11	0.000816	0.025085	0.781522	0.025876	0.001838	0.120446
0.076912	-0.0053	0.379996	0.433177	0.351397	0.537677	0.588393	0.132123	0.00615
0.390088	0.952835	1.05E-05	3.64E-07	5.1E-05	7.12E-11	3.48E-13	0.138679	0.945291
0.397884	-0.26709	-0.16044	0.608508	0.442591	0.43337	0.576984	-0.02956	-0.0065
3.62E-06	0.002401	0.071567	3.23E-14	1.89E-07	3.59E-07	1.25E-12	0.741463	0.942206
0.303712	-0.13023	-0.12021	0.369493	0.203966	0.329119	0.401499	0.035405	-0.02419
0.000518	0.144487	0.178255	1.91E-05	0.021443	0.000158	2.89E-06	0.692717	0.787176
0.145534	0.207903	-0.02488	0.276203	0.21038	0.078957	0.163023	0.240902	0.197922
0.102559	0.019003	0.781259	0.001671	0.017594	0.377573	0.067058	0.006366	0.025713
0.710251	0.025024	0.085685	0.383175	0.382122	0.294127	0.30129	0.275015	0.236
8.72E-21	0.78004	0.338147	8.73E-06	9.29E-06	0.00079	0.000577	0.001753	0.00756
0.701326	0.69668	0.604695	0.730604	0.734408	0.752019	0.666505	0.807214	0.825604
4.24E-20	9.44E-20	5.14E-14	1.87E-22	8.79E-23	2.22E-24	1.2E-17	2.11E-30	7.38E-33
0.529268	0.450978	0.364549	0.767966	0.750245	0.658492	0.684816	0.464549	0.585939
1.58E-10	1.03E-07	2.51E-05	6.01E-26	3.26E-24	3.97E-17	6.81E-19	3.76E-08	4.6E-13
-0.0062	-0.05794	0.213754	-0.23778	-0.23045	-0.05394	-0.22593	-0.02929	-0.08003
0.944829	0.517642	0.015821	0.007105	0.009145	0.546977	0.010649	0.743748	0.371096
-0.1677	-0.05586	-0.13794	-0.38169	-0.2038	-0.33428	-0.36017	-0.09715	-0.02156
0.059487	0.532811	0.121986	9.53E-06	0.02155	0.000123	3.19E-05	0.27721	0.809852
-0.22632	0.265519	0.529603	-0.07521	0.151859	0.0179	-0.02176	0.20214	0.158363
0.010511	0.002553	1.53E-10	0.400678	0.088317	0.841676	0.808142	0.022664	0.075361
0.244542	0.594707	0.533595	0.159655	0.346418	0.240386	0.117121	0.560119	0.599559
0.005592	1.68E-13	1.05E-10	0.072981	6.62E-05	0.006484	0.189737	7.55E-12	9.5E-14
-0.3077	0.381839	0.223338	-0.25448	-0.22983	-0.09286	-0.26942	0.148824	0.220944
0.000433	9.44E-06	0.011604	0.003887	0.009341	0.299105	0.002191	0.09494	0.012552
-0.72313	-0.34874	-0.27279	-0.73332	-0.65079	-0.64622	-0.6643	-0.53132	-0.43032
7.99E-22	5.86E-05	0.001916	1.09E-22	1.21E-16	2.31E-16	1.68E-17	1.3E-10	4.43E-07
-0.46187	-0.51239	-0.55049	-0.43138	-0.54341	-0.53442	-0.45273	-0.61797	-0.61029
4.61E-08	7.37E-10	2.02E-11	4.12E-07	4.07E-11	9.72E-11	9.09E-08	9.96E-15	2.6E-14
-0.60018	-0.1604	-0.18351	-0.66107	-0.69085	-0.53081	-0.58192	-0.37714	-0.47191
8.82E-14	0.071629	0.038906	2.72E-17	2.52E-19	1.37E-10	7.23E-13	1.24E-05	2.14E-08
-0.20945	-0.48732	-0.33288	-0.39143	-0.35951	-0.17179	-0.17381	-0.42656	-0.36839
0.018114	6.24E-09	0.000131	5.35E-06	3.31E-05	0.053461	0.050672	5.72E-07	2.03E-05

PTBP3	PTCH1	PTER	PTGDR	PTGER2	PTGER4	PTGS1	PTPN18	PTPN2
brown	brown	brown	brown	brown	brown	brown	brown	brown
0.053377	-0.27169	0.372521	0.180816	0.236276	0.296222	0.069754	0.236549	0.124587
0.551172	0.002003	1.61E-05	0.041916	0.007488	0.000721	0.435826	0.007417	0.16284
-0.06073	-0.18461	0.307958	0.038588	0.099551	0.101517	-0.08194	0.15269	0.008711
0.497587	0.03773	0.000428	0.666668	0.26547	0.256097	0.359734	0.086569	0.922565
0.212117	0.365622	-0.38009	-0.27241	-0.05308	-0.13636	0.024728	-0.45055	-0.06184
0.016661	2.37E-05	1.04E-05	0.001946	0.553429	0.126336	0.782577	1.07E-07	0.489739
0.491406	0.075992	-0.06455	-0.09831	0.135104	0.161395	0.303651	-0.40427	0.298861
4.46E-09	0.395802	0.470888	0.271479	0.129915	0.069871	0.00052	2.43E-06	0.000642
-0.00131	-0.31705	0.185509	0.01791	0.203694	0.061411	-0.0828	-0.03522	0.226436
0.988375	0.000282	0.036791	0.841595	0.021621	0.492791	0.354729	0.694271	0.01047
0.510082	-0.27416	0.264762	0.047247	0.330126	0.322738	0.274893	-0.2591	0.537563
9.04E-10	0.001814	0.002629	0.597862	0.00015	0.000215	0.001762	0.003267	7.19E-11
0.259807	-0.14283	0.094174	0.08226	0.254549	0.18853	0.241349	-0.06644	0.392868
0.00318	0.109182	0.292277	0.357876	0.003877	0.033779	0.006267	0.457974	4.91E-06
0.182456	-0.29914	0.164758	0.088284	0.069724	0.234414	0.298396	0.159183	0.306916
0.040062	0.000635	0.064163	0.323641	0.436022	0.007986	0.000656	0.073844	0.000449
0.547333	-0.26786	0.108502	0.347745	0.572768	0.509229	0.400282	0.254667	0.502171
2.77E-11	0.002329	0.224649	6.18E-05	1.98E-12	9.74E-10	3.12E-06	0.00386	1.8E-09
0.531653	-0.6425	0.665712	0.568084	0.670036	0.810013	0.639364	0.711018	0.708189
1.26E-10	3.88E-16	1.36E-17	3.27E-12	7.03E-18	9.28E-31	5.97E-16	7.59E-21	1.26E-20
0.478826	-0.52072	0.494681	0.389365	0.540392	0.691842	0.623871	0.346396	0.591656
1.24E-08	3.48E-10	3.39E-09	6.06E-06	5.47E-11	2.14E-19	4.68E-15	6.63E-05	2.39E-13
-0.35219	0.158454	-0.41612	-0.04265	-0.10837	-0.20376	-0.08705	0.008705	-0.29099
4.89E-05	0.075192	1.14E-06	0.633978	0.225219	0.021576	0.330471	0.922625	0.000903
-0.19975	0.389645	-0.4368	-0.12918	-0.17917	-0.21056	-0.01219	0.100513	-0.34584
0.024351	5.96E-06	2.83E-07	0.147773	0.043845	0.017498	0.891829	0.260853	6.82E-05
-0.35672	-0.05011	-0.19709	-0.06725	-0.07272	-0.17636	-0.13391	0.302816	-0.3192
3.85E-05	0.575799	0.026356	0.452541	0.416538	0.047329	0.133362	0.000539	0.000254
0.01262	-0.22507	0.13759	0.294085	0.246618	0.308612	0.309377	0.701604	0.087059
0.88801	0.010959	0.122934	0.000791	0.005189	0.000415	0.000401	4.04E-20	0.330429
-0.46738	0.034267	0.199282	0.002414	-0.30692	-0.20041	-0.1559	0.284167	-0.39921
3.03E-08	0.702116	0.024693	0.978514	0.000449	0.023878	0.080083	0.001205	3.33E-06
-0.65851	0.630112	-0.56832	-0.43873	-0.67186	-0.69735	-0.51776	-0.37418	-0.82989
3.96E-17	2.07E-15	3.19E-12	2.48E-07	5.31E-18	8.41E-20	4.56E-10	1.47E-05	1.79E-33
-0.12984	0.33599	-0.17824	-0.31391	-0.43334	-0.43315	-0.40373	-0.67046	-0.34477
0.145691	0.000113	0.044979	0.000326	3.6E-07	3.65E-07	2.52E-06	6.59E-18	7.21E-05
-0.51709	0.335774	-0.23568	-0.24577	-0.51072	-0.55732	-0.56639	-0.32325	-0.5268
4.84E-10	0.000114	0.007643	0.00535	8.54E-10	1.01E-11	3.91E-12	0.00021	1.99E-10
-0.11656	0.586601	-0.38416	-0.1778	-0.15331	-0.3011	-0.37752	-0.34249	-0.29954
0.191875	4.27E-13	8.24E-06	0.045515	0.085275	0.000582	1.21E-05	8.1E-05	0.000623

PTPN22	PTPN6	PTPN7	PTPRC	PURG	PVRIG	PXMP2	PYHIN1	QPRT
brown	brown	brown	brown	brown	brown	brown	brown	brown
0.192614	0.284921	0.095568	0.185933	-0.19382	0.24941	-0.42705	0.375797	0.237104
0.030041	0.001167	0.285162	0.036356	0.029003	0.004688	5.53E-07	1.34E-05	0.007275
0.042912	0.134793	-0.10147	-0.0115	-0.11235	0.099051	-0.29142	0.191616	0.143682
0.631918	0.130808	0.256313	0.897935	0.208531	0.26789	0.000887	0.030921	0.107054
0.02009	-0.41209	0.093852	-0.32718	0.35614	-0.07156	0.494119	-0.18537	-0.27747
0.822614	1.48E-06	0.293938	0.000174	3.97E-05	0.424011	3.56E-09	0.036938	0.001587
0.387736	-0.3148	0.251938	0.067891	0.335739	-0.08533	0.126554	0.039524	0.009602
6.67E-06	0.000313	0.004272	0.448207	0.000114	0.340179	0.156252	0.659085	0.914679
0.074465	0.032331	-0.03551	0.013152	0.081188	-0.10904	0.163096	0.104141	0.223917
0.405386	0.71822	0.691852	0.883328	0.364197	0.222343	0.066935	0.243948	0.011384
0.500218	-0.1227	0.233682	0.267378	0.225986	-0.11223	-0.00342	0.241466	0.311745
2.12E-09	0.169353	0.00819	0.002374	0.010628	0.20903	0.969603	0.00624	0.00036
0.254144	-0.10175	0.145063	0.116491	0.064107	-0.19704	-0.11537	0.086256	0.164855
0.003936	0.254982	0.103688	0.192149	0.473964	0.026393	0.196481	0.334924	0.064005
0.253371	0.161684	0.183682	0.500391	0.110739	-0.12356	-0.23663	0.134933	0.336535
0.004051	0.069365	0.03872	2.09E-09	0.215184	0.166358	0.007396	0.130405	0.000109
0.576707	0.265477	0.628641	0.290845	-0.44473	0.325826	-0.07104	0.417404	0.156223
1.29E-12	0.002557	2.51E-15	0.000909	1.62E-07	0.000185	0.427375	1.05E-06	0.079443
0.780958	0.880469	0.796268	0.833245	-0.60391	0.497197	-0.52997	0.763672	0.586448
2.55E-27	2.46E-42	4.61E-29	5.78E-34	5.64E-14	2.75E-09	1.48E-10	1.63E-25	4.34E-13
0.668347	0.57295	0.696743	0.711422	-0.26546	0.436683	-0.43247	0.624843	0.579108
9.1E-18	1.94E-12	9.34E-20	7.05E-21	0.002559	2.86E-07	3.83E-07	4.13E-15	9.88E-13
-0.22772	0.001043	0.037898	-0.33656	-0.12114	0.144195	0.466457	-0.17703	-0.26524
0.010029	0.990712	0.67228	0.000109	0.174882	0.105794	3.25E-08	0.04647	0.002581
-0.20105	-0.00911	0.117026	-0.33725	-0.09256	0.232438	0.247707	-0.19047	-0.37812
0.023423	0.919045	0.1901	0.000106	0.300664	0.008547	0.004988	0.031959	1.17E-05
-0.1922	0.242198	0.067545	-0.2266	-0.2606	0.309523	0.23987	-0.11369	-0.06723
0.030399	0.00608	0.450534	0.010413	0.003086	0.000399	0.006603	0.203147	0.45265
0.263773	0.684427	0.535619	0.266398	-0.58518	0.566812	-0.20792	0.324839	0.171404
0.002732	7.25E-19	8.67E-11	0.002467	5.01E-13	3.74E-12	0.01899	0.000194	0.054003
-0.30989	0.256625	-0.32193	-0.03327	-0.0596	0.009338	-0.34818	-0.07899	-0.03882
0.000392	0.003587	0.000223	0.710373	0.505649	0.917016	6.04E-05	0.377396	0.664785
-0.81542	-0.42976	-0.6167	-0.67566	0.374455	-0.29834	0.388543	-0.66564	-0.59573
1.83E-31	4.6E-07	1.17E-14	2.94E-18	1.44E-05	0.000657	6.36E-06	1.37E-17	1.49E-13
-0.42785	-0.68875	-0.68216	-0.30981	0.56093	-0.52899	0.120101	-0.43572	-0.26398
5.24E-07	3.57E-19	1.05E-18	0.000393	6.94E-12	1.62E-10	0.178639	3.06E-07	0.00271
-0.72928	-0.41873	-0.76527	-0.39161	0.257233	-0.45623	0.091634	-0.47573	-0.34998
2.43E-22	9.62E-07	1.13E-25	5.29E-06	0.003506	7.03E-08	0.305549	1.58E-08	5.5E-05
-0.30495	-0.3858	-0.23416	-0.58186	0.239582	-0.09119	0.408591	-0.22133	-0.5386
0.00049	7.49E-06	0.008055	7.28E-13	0.00667	0.307913	1.85E-06	0.012396	6.51E-11

RAB11FIP1	RAB20	RAB32	RAB38	RAC2	RAC3	RAP1A	RAP2B	RARRES2
brown	brown	brown	brown	brown	brown	brown	brown	brown
0.356336	0.136462	0.181736	0.338144	0.221508	-0.30789	0.210713	0.208654	0.064208
3.93E-05	0.126065	0.040868	0.000101	0.012323	0.000429	0.017412	0.018566	0.473268
0.233199	-0.00222	0.091294	0.304655	0.032463	-0.18826	0.115055	0.015022	0.048817
0.008327	0.98021	0.307355	0.000497	0.717116	0.034043	0.197722	0.866881	0.585738
-0.39446	-0.03134	-0.03704	-0.04945	-0.10068	0.38335	-0.09321	-0.15851	-0.33847
4.46E-06	0.726493	0.679275	0.58091	0.260056	8.64E-06	0.297255	0.075087	9.94E-05
-0.11463	0.170057	0.283787	0.147844	0.04018	-0.09292	0.20384	0.021118	-0.13073
0.19941	0.055948	0.001224	0.097162	0.653784	0.298794	0.021525	0.813694	0.142918
-0.09466	0.176873	0.486703	0.376602	0.095507	0.193123	0.186235	-0.15333	0.271158
0.289785	0.046672	6.56E-09	1.28E-05	0.285473	0.029601	0.036048	0.085239	0.002045
-0.00618	0.333412	0.639498	0.476367	0.173154	-0.15691	0.427671	0.037155	0.192173
0.945058	0.000128	5.86E-16	1.51E-08	0.051561	0.078114	5.3E-07	0.678349	0.030427
0.08202	0.18717	0.377304	0.324707	0.097857	-0.14895	0.107822	-0.05225	0.073001
0.359284	0.035109	1.23E-05	0.000196	0.273729	0.094668	0.227585	0.559647	0.414707
-0.03246	0.06074	0.263682	0.048157	0.125363	-0.40374	0.209274	0.085827	0.321887
0.717105	0.497538	0.002741	0.590816	0.160214	2.52E-06	0.018211	0.337341	0.000224
0.276512	0.507019	0.386385	0.292905	0.577854	-0.32	0.22396	0.499105	0.247118
0.00165	1.18E-09	7.23E-06	0.000832	1.13E-12	0.000245	0.011368	2.34E-09	0.005096
0.545248	0.796112	0.719182	0.607884	0.87415	-0.69172	0.638136	0.709008	0.502863
3.4E-11	4.81E-29	1.69E-21	3.49E-14	5.02E-41	2.18E-19	7.06E-16	1.09E-20	1.69E-09
0.477955	0.639312	0.580757	0.404266	0.684306	-0.61859	0.526726	0.558792	0.357817
1.33E-08	6.01E-16	8.23E-13	2.43E-06	7.4E-19	9.2E-15	2.01E-10	8.66E-12	3.63E-05
0.315419	-0.05937	-0.24032	-0.28681	-0.00902	-0.01536	-0.39828	-0.17359	-0.21959
0.000304	0.507329	0.006498	0.001079	0.919825	0.863944	3.53E-06	0.050973	0.013117
-0.14735	-0.11671	-0.40502	-0.37197	0.000413	0.174226	-0.32745	0.016765	-0.34174
0.098308	0.191313	2.32E-06	1.66E-05	0.99632	0.05011	0.000171	0.851601	8.42E-05
-0.20302	0.053116	-0.09625	-0.27431	0.129611	0.350803	-0.1309	0.106613	0.135979
0.022067	0.55312	0.281701	0.001803	0.146408	5.27E-05	0.14239	0.232871	0.127424
0.219812	0.396781	0.141129	0.026588	0.573336	-0.27758	0.190271	0.494349	0.211945
0.013024	3.87E-06	0.113502	0.766677	1.86E-12	0.00158	0.032141	3.49E-09	0.016751
0.052655	-0.26117	-0.25467	-0.0981	-0.14197	-0.09634	0.113081	-0.11836	-0.05767
0.556577	0.003019	0.00386	0.272525	0.11135	0.28126	0.205575	0.185062	0.519534
-0.43933	-0.63177	-0.78635	-0.63543	-0.64046	0.64659	-0.54419	-0.51271	-0.56303
2.37E-07	1.66E-15	6.45E-28	1.02E-15	5.14E-16	2.19E-16	3.77E-11	7.16E-10	5.57E-12
-0.22656	-0.55407	-0.45344	-0.28494	-0.71448	0.318686	-0.27516	-0.43348	-0.28974
0.010428	1.41E-11	8.63E-08	0.001167	4.04E-21	0.000261	0.001743	3.57E-07	0.000953
-0.15297	-0.65823	-0.65934	-0.40524	-0.66958	0.369693	-0.5157	-0.46982	-0.24834
0.085981	4.13E-17	3.51E-17	2.29E-06	7.54E-18	1.89E-05	5.49E-10	2.51E-08	0.004874
-0.18821	-0.26558	-0.32129	0.018767	-0.25778	0.508438	-0.32475	-0.33743	-0.66952
0.034088	0.002547	0.00023	0.834123	0.003435	1.04E-09	0.000195	0.000105	7.61E-18

RARRES3	RASAL3	RASGRP4	RASL10B	RASSF3	RASSF4	RASSF5	RBFOX2	RBM47
brown	brown	brown	brown	brown	brown	brown	brown	brown
0.440257	0.103415	0.247013	-0.29395	0.457873	-0.01019	0.415615	-0.10879	0.119958
2.23E-07	0.247266	0.005115	0.000796	6.22E-08	0.909458	1.18E-06	0.223434	0.179159
0.359836	-0.08264	0.098061	-0.1737	0.383116	-0.02887	0.283108	-0.091	-0.05123
3.25E-05	0.355652	0.272724	0.050818	8.76E-06	0.747315	0.001259	0.308913	0.567341
-0.46994	-0.27336	-0.30661	0.204858	-0.39523	-0.16468	-0.57936	0.253935	-0.06221
2.49E-08	0.001873	0.000455	0.020868	4.25E-06	0.064299	9.61E-13	0.003967	0.487161
-0.169	-0.29226	-0.18687	-0.16804	-0.07255	-0.12455	-0.33458	0.027604	0.283467
0.057517	0.000856	0.03541	0.058966	0.417635	0.162961	0.000121	0.758036	0.00124
0.393112	-0.1172	-0.07477	-0.09997	0.142392	-0.08353	-0.08031	-0.35865	0.097759
4.84E-06	0.189442	0.403458	0.26347	0.110275	0.350486	0.369412	3.47E-05	0.274211
0.283713	-0.25748	-0.09465	-0.34997	0.241543	-0.12092	-0.11261	-0.31841	0.431373
0.001228	0.003473	0.289836	5.5E-05	0.006223	0.17567	0.207503	0.000264	4.12E-07
0.192532	-0.27425	-0.0401	-0.29584	0.263453	-0.06175	-0.00871	-0.1042	0.189789
0.030113	0.001808	0.654421	0.000733	0.002766	0.490425	0.92258	0.243666	0.032587
0.401882	0.133369	0.080571	-0.26266	0.11105	0.02433	0.192959	-0.37643	0.317643
2.82E-06	0.134963	0.367864	0.002851	0.213889	0.785992	0.029742	1.29E-05	0.000274
-0.00479	0.318357	0.322978	-0.44193	0.308558	0.153828	0.126815	-0.07551	0.514478
0.957374	0.000265	0.000213	1.98E-07	0.000416	0.084221	0.155392	0.39883	6.12E-10
0.7762	0.766969	0.839564	-0.76985	0.719798	0.528285	0.636025	-0.71105	0.871179
8.32E-27	7.6E-26	6.36E-35	3.85E-26	1.5E-21	1.73E-10	9.4E-16	7.55E-21	1.96E-40
0.713497	0.501985	0.546407	-0.60963	0.497625	0.305908	0.487467	-0.47701	0.715634
4.83E-21	1.83E-09	3.03E-11	2.82E-14	2.65E-09	0.00047	6.16E-09	1.43E-08	3.26E-21
-0.21779	0.062972	0.068298	0.128131	-0.26241	0.16749	-0.08437	0.381825	-0.19101
0.013905	0.481843	0.445484	0.151113	0.002879	0.059813	0.345626	9.45E-06	0.031466
-0.48269	0.219997	0.082674	0.195034	-0.3874	0.1939	-0.24766	0.319143	-0.20405
9.09E-09	0.012946	0.355455	0.027996	6.81E-06	0.02894	0.004997	0.000255	0.021388
-0.04058	0.416419	0.15492	0.180399	-0.2765	0.183882	-0.15673	-0.14331	-0.10631
0.65059	1.12E-06	0.082014	0.0424	0.001651	0.038506	0.078461	0.107982	0.234213
0.223831	0.805758	0.671429	-0.3193	0.191367	0.556788	0.280665	-0.33894	0.354666
0.011416	3.22E-30	5.67E-18	0.000253	0.031144	1.06E-11	0.001393	9.7E-05	4.29E-05
0.199406	0.187461	0.2228	0.099816	0.151002	0.200589	0.19508	-0.14562	-0.23676
0.024602	0.03482	0.011811	0.26419	0.090148	0.023747	0.027959	0.102344	0.007364
-0.61207	-0.32004	-0.42802	0.772569	-0.63811	-0.22162	-0.4197	0.58005	-0.7797
2.08E-14	0.000244	5.18E-07	2.01E-26	7.09E-16	0.012279	9.03E-07	8.9E-13	3.5E-27
-0.37735	-0.73022	-0.67867	0.498769	-0.26929	-0.57091	-0.2233	0.486288	-0.51483
1.22E-05	2.02E-22	1.83E-18	2.4E-09	0.002202	2.42E-12	0.011618	6.79E-09	5.93E-10
-0.30009	-0.46391	-0.48416	0.587557	-0.28837	-0.38111	-0.01873	0.500372	-0.71445
0.000609	3.95E-08	8.07E-09	3.83E-13	0.00101	9.85E-06	0.834401	2.1E-09	4.06E-21
-0.63667	-0.31123	-0.27642	0.322421	-0.24927	-0.16377	-0.38382	0.506762	-0.43502
8.61E-16	0.000369	0.001656	0.000218	0.004712	0.065793	8.41E-06	1.21E-09	3.21E-07

RBP1	RBSN	RCCD1	RCSD1	REM2	RET	RFTN1	RGS1	RGS10
brown	brown	brown	brown	brown	brown	brown	brown	brown
-0.05722	-0.06536	-0.37384	0.141686	-0.01418	-0.30201	0.36094	0.022903	0.300126
0.522835	0.46534	1.5E-05	0.112068	0.874311	0.000559	3.06E-05	0.798267	0.000608
-0.11415	0.03365	-0.26103	0.012689	0.062515	-0.26883	0.288602	-0.00516	0.187676
0.201278	0.707237	0.003036	0.887407	0.485038	0.002242	0.001	0.954073	0.034609
-0.08661	0.161679	0.334172	-0.36029	0.227707	0.370687	-0.29934	-0.01791	-0.46392
0.332912	0.069373	0.000123	3.17E-05	0.010034	1.79E-05	0.000629	0.841633	3.95E-08
0.054153	0.149694	0.000648	-0.1758	0.232179	0.15184	-0.10895	0.297819	-0.2367
0.545392	0.093002	0.994235	0.048047	0.008623	0.088358	0.22275	0.000672	0.007378
0.188806	-0.09589	-0.06706	-0.0071	0.157477	-0.21207	-0.09305	0.179881	0.392014
0.033515	0.283549	0.453813	0.93689	0.077031	0.016683	0.298125	0.043007	5.17E-06
0.251592	0.008865	-0.20855	0.000708	0.215886	-0.15235	0.014215	0.492276	0.20477
0.004327	0.921199	0.018623	0.993695	0.014782	0.087286	0.873968	4.15E-09	0.020924
0.174557	0.111141	-0.20262	0.019851	0.107628	-0.17536	0.090082	0.235706	0.099959
0.049669	0.213512	0.022335	0.824686	0.228428	0.04861	0.313848	0.007637	0.263506
0.237565	0.026068	-0.50421	0.163898	-0.1257	-0.23518	0.242482	0.229735	0.295124
0.007159	0.771118	1.51E-09	0.065585	0.159093	0.007777	0.006019	0.00937	0.000756
0.363116	-0.42877	-0.0888	0.390855	-0.17099	-0.21536	0.376605	0.409154	-0.04412
2.72E-05	4.92E-07	0.320814	5.54E-06	0.054588	0.015031	1.28E-05	1.79E-06	0.622338
0.463993	-0.50279	-0.57644	0.813178	-0.41263	-0.56092	0.600727	0.561965	0.751507
3.92E-08	1.7E-09	1.33E-12	3.61E-31	1.43E-06	6.95E-12	8.27E-14	6.23E-12	2.48E-24
0.317923	-0.23002	-0.55132	0.517676	-0.19597	-0.30038	0.497855	0.414495	0.492543
0.00027	0.009282	1.86E-11	4.59E-10	0.02724	0.000601	2.6E-09	1.27E-06	4.05E-09
-0.23574	0.06504	0.14035	-0.05238	0.020341	0.112125	-0.08076	-0.25037	-0.27156
0.007628	0.467543	0.115527	0.558627	0.820437	0.209459	0.366759	0.004526	0.002013
-0.2211	-0.01008	0.318433	-0.12752	-0.0694	0.275281	-0.08993	-0.37105	-0.44193
0.012489	0.910455	0.000264	0.153078	0.438194	0.001734	0.314672	1.75E-05	1.98E-07
0.078888	-0.37948	0.297874	0.02016	-0.11807	0.106038	-0.1013	-0.31269	-0.02508
0.377986	1.08E-05	0.000671	0.822005	0.186133	0.235416	0.257101	0.000345	0.779564
0.20485	-0.46144	-0.11556	0.501967	-0.40265	-0.23742	0.342974	-0.00724	0.253517
0.020873	4.76E-08	0.195761	1.83E-09	2.69E-06	0.007195	7.9E-05	0.935647	0.004029
-0.19987	0.224162	-0.13884	0.179587	-0.2803	-0.17675	0.047806	-0.29639	0.332713
0.024263	0.011292	0.119541	0.043353	0.001414	0.046829	0.593529	0.000716	0.000132
-0.47613	0.462986	0.439478	-0.47438	0.116607	0.5159	-0.46339	-0.61293	-0.47262
1.53E-08	4.24E-08	2.35E-07	1.76E-08	0.191703	5.39E-10	4.11E-08	1.87E-14	2.02E-08
-0.28147	0.451717	0.23365	-0.49709	0.313625	0.368171	-0.3996	-0.15874	-0.36504
0.001348	9.79E-08	0.008199	2.77E-09	0.00033	2.06E-05	3.25E-06	0.074663	2.45E-05
-0.35927	0.360675	0.225711	-0.36955	0.118621	0.274219	-0.27851	-0.40663	-0.24038
3.35E-05	3.11E-05	0.010726	1.9E-05	0.184089	0.00181	0.001521	2.1E-06	0.006486
-0.49203	0.319901	0.429966	-0.35387	0.252621	0.251379	-0.44494	-0.34251	-0.36662
4.23E-09	0.000246	4.54E-07	4.48E-05	0.004165	0.004361	1.6E-07	8.09E-05	2.24E-05

RGS18	RGS8	RHBDF2	RHBDL3	RHOG	RHOH	RHOQ	RILPL2	RIN2
brown	brown	brown	brown	brown	brown	brown	brown	brown
0.05265	-0.07109	0.125958	-0.23971	0.308855	0.071215	0.221338	0.267364	0.166746
0.556616	0.427064	0.158225	0.00664	0.000411	0.426247	0.012392	0.002375	0.060974
-0.07495	-0.05754	0.025042	-0.14261	0.264602	-0.05333	0.171315	0.152133	0.080889
0.402304	0.520528	0.779892	0.109732	0.002645	0.551528	0.05413	0.087738	0.365973
-0.08049	0.259344	-0.47159	0.218546	-0.46935	-0.07152	-0.11831	-0.51528	-0.32557
0.368353	0.003237	2.19E-08	0.01357	2.6E-08	0.424262	0.185263	5.7E-10	0.000188
0.265844	0.256332	-0.40507	-0.17818	-0.44352	0.146574	0.173254	-0.28733	-0.04047
0.002521	0.003627	2.31E-06	0.045052	1.77E-07	0.100101	0.051424	0.001055	0.651459
0.283188	-0.065	-0.14292	-0.15103	0.119051	0.270379	-0.02618	0.222438	0.071165
0.001255	0.467831	0.108942	0.090092	0.182495	0.002109	0.770157	0.011953	0.426577
0.535657	0.157774	-0.29218	-0.41458	-0.19074	0.379178	0.249475	0.08048	0.183147
8.64E-11	0.076468	0.000858	1.26E-06	0.031709	1.1E-05	0.004677	0.368411	0.039301
0.316961	0.059954	-0.15469	-0.34959	-0.07848	0.247752	0.150323	0.111553	0.097238
0.000283	0.503135	0.082468	5.61E-05	0.380484	0.00498	0.091622	0.211808	0.276791
0.35051	0.034345	0.126436	-0.26704	-0.01368	0.165249	0.085078	0.23427	0.284208
5.35E-05	0.701471	0.156639	0.002405	0.878687	0.063363	0.341592	0.008026	0.001203
0.345992	-0.23725	0.280974	-0.41199	0.121367	0.46264	0.318515	0.245938	0.170479
6.77E-05	0.007238	0.001375	1.49E-06	0.174071	4.35E-08	0.000263	0.005318	0.055333
0.726936	-0.49988	0.757651	-0.67104	0.741707	0.766336	0.585922	0.744706	0.692296
3.84E-22	2.19E-09	6.41E-25	6.03E-18	1.98E-23	8.81E-26	4.61E-13	1.06E-23	1.98E-19
0.519977	-0.23129	0.408367	-0.59306	0.417572	0.591569	0.434269	0.506496	0.478817
3.73E-10	0.00889	1.88E-06	2.04E-13	1.04E-06	2.42E-13	3.38E-07	1.24E-09	1.24E-08
-0.42247	-0.31277	0.08583	0.130947	0.09857	-0.13333	-0.03843	-0.2478	-0.2449
7.51E-07	0.000343	0.337323	0.142258	0.27023	0.135064	0.667976	0.00497	0.005521
-0.43048	-0.11899	0.088927	0.348646	-0.0032	-0.18956	-0.15138	-0.35013	-0.28748
4.38E-07	0.182716	0.320116	5.89E-05	0.9715	0.032801	0.089339	5.45E-05	0.001048
-0.29247	-0.36483	0.277425	0.258251	0.378783	0.032658	-0.21898	0.042344	-0.14528
0.000848	2.47E-05	0.00159	0.003374	1.13E-05	0.715486	0.013383	0.63644	0.103165
0.063376	-0.54647	0.726936	-0.11934	0.689764	0.328097	0.223814	0.339429	0.259348
0.479032	3.01E-11	3.84E-22	0.181416	3.02E-19	0.000166	0.011423	9.47E-05	0.003236
-0.18381	-0.16068	0.302814	0.221131	0.48614	-0.31356	0.095019	0.053864	0.098293
0.038581	0.07114	0.000539	0.012476	6.87E-09	0.000331	0.287948	0.54754	0.271585
-0.69743	0.23493	-0.35283	0.755806	-0.30792	-0.70652	-0.43483	-0.5747	-0.54781
8.31E-20	0.007845	4.73E-05	9.66E-25	0.000429	1.7E-20	3.25E-07	1.6E-12	2.64E-11
-0.25245	0.549585	-0.6361	0.279814	-0.68897	-0.53453	-0.28601	-0.39324	-0.29005
0.004192	2.21E-11	9.3E-16	0.001442	3.44E-19	9.62E-11	0.001115	4.8E-06	0.00094
-0.4684	0.308277	-0.30357	0.425964	-0.34623	-0.60986	-0.43055	-0.22357	-0.29691
2.8E-08	0.000422	0.000521	5.95E-07	6.69E-05	2.74E-14	4.36E-07	0.011516	0.0007
-0.28953	0.175841	-0.39356	0.382928	-0.31933	-0.25722	-0.15677	-0.45246	-0.43091
0.000962	0.047988	4.71E-06	8.86E-06	0.000253	0.003508	0.078393	9.27E-08	4.26E-07

RIN3	RIPK2	RIPK3	RNASE2	RNASE6	RNASET2	RNF13	RNF130	RNF135
brown	brown	brown	brown	brown	brown	brown	brown	brown
0.059757	0.240689	0.240495	0.083276	0.375959	0.159751	0.415555	0.401973	0.19812
0.504536	0.006414	0.006459	0.351954	1.33E-05	0.072806	1.18E-06	2.81E-06	0.025562
-0.04815	0.1589	0.113572	0.041739	0.291051	0.040316	0.370283	0.330959	0.090391
0.590874	0.074365	0.203604	0.641264	0.000901	0.652688	1.83E-05	0.000144	0.312185
-0.24029	-0.02759	-0.21869	-0.02149	-0.3073	-0.43318	-0.39234	-0.38355	-0.14779
0.006506	0.75814	0.013508	0.810473	0.000441	3.64E-07	5.07E-06	8.54E-06	0.097293
-0.29387	0.147517	-0.05301	0.25488	0.007603	-0.41366	-1.2E-07	-0.1979	0.128804
0.000798	0.097913	0.553884	0.00383	0.932394	1.34E-06	0.999999	0.025731	0.148961
-0.24757	0.086895	0.165978	0.287345	0.344022	0.013355	0.224902	0.251778	0.179741
0.005013	0.331345	0.062189	0.001055	7.49E-05	0.881535	0.011019	0.004297	0.043172
-0.35437	0.245721	0.150266	0.509668	0.405798	-0.25552	0.345122	0.117187	0.328535
4.36E-05	0.00536	0.091747	9.37E-10	2.21E-06	0.00374	7.08E-05	0.189488	0.000163
-0.23292	0.239543	0.093279	0.378735	0.23578	-0.26466	0.21822	0.051531	0.220391
0.008406	0.006679	0.296911	1.13E-05	0.007618	0.002639	0.013714	0.565045	0.012781
0.013813	0.075701	0.082837	0.304951	0.312584	0.090443	0.324813	0.220976	0.323203
0.877504	0.397614	0.354506	0.00049	0.000346	0.311903	0.000195	0.012539	0.00021
0.422214	0.31589	0.387503	0.455236	0.272544	0.234967	-0.11311	0.134933	0.469555
7.64E-07	0.000297	6.77E-06	7.56E-08	0.001936	0.007835	0.205462	0.130404	2.56E-08
0.711726	0.529932	0.880135	0.680531	0.861527	0.749745	0.591713	0.793041	0.869685
6.68E-21	1.49E-10	2.9E-42	1.36E-18	1.31E-38	3.63E-24	2.38E-13	1.11E-28	3.84E-40
0.421647	0.358183	0.605825	0.503176	0.61605	0.422206	0.500094	0.497015	0.668306
7.94E-07	3.56E-05	4.48E-14	1.65E-09	1.27E-14	7.65E-07	2.15E-09	2.79E-09	9.16E-18
0.164604	-0.12847	0.063532	-0.23824	-0.22906	0.17536	-0.3032	-0.04937	-0.09706
0.064417	0.15004	0.477948	0.006992	0.009588	0.048612	0.00053	0.581473	0.277664
0.370683	-0.06237	-0.06801	-0.31665	-0.44522	0.08337	-0.51374	-0.19206	-0.13478
1.79E-05	0.48606	0.44739	0.000287	1.57E-07	0.351404	6.54E-10	0.030528	0.130849
0.46776	-0.13822	0.142221	-0.19683	-0.21547	0.430637	-0.29313	0.071119	0.020945
2.94E-08	0.121212	0.110707	0.02656	0.014979	4.34E-07	0.000824	0.426877	0.815196
0.888361	0.216627	0.556823	0.123706	0.209638	0.751672	0.031462	0.476335	0.49359
4.44E-44	0.014435	1.06E-11	0.165857	0.018006	2.39E-24	0.72548	1.51E-08	3.72E-09
0.145404	-0.15206	0.074422	-0.28256	0.043388	0.314415	0.426528	0.361778	-0.04824
0.102869	0.087898	0.405659	0.001288	0.628138	0.000318	5.73E-07	2.93E-05	0.590204
-0.26228	-0.43976	-0.58123	-0.70308	-0.75237	-0.30414	-0.4578	-0.50542	-0.71646
0.002893	2.3E-07	7.8E-13	3.12E-20	2.06E-24	0.000508	6.25E-08	1.36E-09	2.8E-21
-0.7893	-0.39	-0.67301	-0.40282	-0.4187	-0.70268	-0.10295	-0.60243	-0.69377
2.99E-28	5.83E-06	4.44E-18	2.66E-06	9.64E-07	3.35E-20	0.249395	6.74E-14	1.55E-19
-0.48961	-0.4226	-0.59949	-0.53043	-0.445	-0.36092	-0.21661	-0.44159	-0.72662
5.17E-09	7.45E-07	9.58E-14	1.42E-10	1.59E-07	3.06E-05	0.014445	2.03E-07	4.09E-22
-0.31683	0.024088	-0.23558	-0.28941	-0.35641	-0.36025	-0.42822	-0.22701	-0.41095
0.000284	0.788076	0.00767	0.000966	3.91E-05	3.18E-05	5.11E-07	0.010272	1.59E-06

RNF144A	RNF144B	RNF149	RNF19A	RNF19B	RNF217	RNFT2	ROBO2	RPGRIP1
brown	brown	brown	brown	brown	brown	brown	brown	brown
-0.03195	0.154672	0.26367	0.061643	0.279158	0.187528	-0.09534	-0.12944	0.257154
0.721396	0.082511	0.002742	0.491158	0.001482	0.034755	0.286321	0.146955	0.003516
0.044351	0.064187	0.18248	-0.02822	0.093689	0.074766	0.03829	-0.06807	0.153082
0.620517	0.473415	0.040035	0.752777	0.294781	0.403486	0.66909	0.447017	0.085753
0.098212	-0.31555	-0.30859	-0.0599	-0.27082	-0.16279	0.043971	0.416527	-0.25611
0.271985	0.000302	0.000416	0.503483	0.002073	0.067447	0.623524	1.11E-06	0.003657
-0.17692	0.022413	0.090378	0.279167	-0.07465	0.141647	-0.28501	0.311395	-0.14235
0.04661	0.802496	0.312251	0.001481	0.404234	0.11217	0.001163	0.000366	0.110387
-0.05235	0.074604	0.214986	-0.25094	-0.02491	0.053296	0.020303	-0.27294	0.219201
0.558857	0.404507	0.015213	0.004433	0.781059	0.55178	0.82076	0.001905	0.013285
-0.26933	0.248939	0.413598	0.202708	0.033534	0.31618	-0.33215	-0.03441	0.075208
0.002199	0.004769	1.34E-06	0.022278	0.708196	0.000293	0.000136	0.700948	0.400705
-0.22671	0.278341	0.223669	0.016079	0.059358	0.122459	-0.29619	-0.08317	0.152886
0.010373	0.001532	0.011478	0.85761	0.507395	0.170199	0.000722	0.352549	0.086161
-0.31803	0.165567	0.212771	0.465534	0.103079	0.225273	-0.23828	-0.22947	0.148385
0.000269	0.06285	0.01632	3.49E-08	0.248814	0.010883	0.006983	0.009453	0.09593
-0.33132	0.472165	0.366724	0.33226	0.190757	0.196437	-0.52581	-0.24906	0.106383
0.000142	2.09E-08	2.23E-05	0.000135	0.031695	0.026866	2.18E-10	0.004748	0.23389
-0.45058	0.768105	0.691772	0.530101	0.474803	0.500605	-0.57726	-0.66019	0.577973
1.06E-07	5.82E-26	2.16E-19	1.46E-10	1.7E-08	2.06E-09	1.21E-12	3.09E-17	1.12E-12
-0.46824	0.490168	0.516261	0.543354	0.466258	0.323983	-0.5271	-0.26363	0.368928
2.84E-08	4.94E-09	5.22E-10	4.1E-11	3.3E-08	0.000203	1.94E-10	0.002747	1.97E-05
0.161981	-0.16469	-0.25807	-0.02467	9.14E-05	-0.47645	0.053098	0.07894	-0.02708
0.068848	0.064279	0.003397	0.783101	0.999186	1.5E-08	0.553258	0.377676	0.762456
0.161909	-0.29679	-0.48282	-0.02011	-0.12671	-0.32545	0.105576	0.224784	-0.09875
0.068972	0.000703	8.99E-09	0.822457	0.155748	0.000189	0.237476	0.011062	0.269335
0.072532	-0.18402	-0.30194	-0.19077	-0.07324	-0.30419	0.201653	-0.13225	0.119845
0.41772	0.038359	0.000561	0.031682	0.413149	0.000507	0.022999	0.138302	0.179573
-0.12067	0.268032	0.056394	0.254611	0.191189	0.038407	-0.18944	-0.39339	0.359148
0.176579	0.002314	0.528862	0.003868	0.031303	0.668138	0.032918	4.76E-06	3.37E-05
0.406324	-0.11098	-0.17669	-0.20083	-0.12781	-0.00535	0.428082	-0.14257	0.16836
2.14E-06	0.214166	0.046905	0.023574	0.152161	0.952418	5.16E-07	0.109832	0.058479
0.536493	-0.68522	-0.78127	-0.51728	-0.31771	-0.55461	0.638033	0.489564	-0.35809
7.97E-11	6.37E-19	2.36E-27	4.76E-10	0.000273	1.33E-11	7.16E-16	5.19E-09	3.57E-05
0.230566	-0.36376	-0.19521	-0.29998	-0.21901	-0.08495	0.335273	0.502425	-0.44833
0.00911	2.63E-05	0.027852	0.000612	0.013369	0.342321	0.000117	1.76E-09	1.25E-07
0.316656	-0.38492	-0.32803	-0.45893	-0.14259	-0.27217	0.475854	0.256668	-0.31726
0.000287	7.88E-06	0.000167	5.75E-08	0.109788	0.001965	1.57E-08	0.003581	0.000279
0.523024	-0.32133	-0.34764	-0.56931	-0.27124	-0.18315	0.338656	0.17509	-0.14834
2.82E-10	0.00023	6.21E-05	2.87E-12	0.002038	0.0393	9.84E-05	0.048965	0.096039

RPRD2	RPS6KA1	RPUSD4	RRAD	RRAS	RSBN1	RTP4	RUNX3	S100A10
brown	brown	brown	brown	brown	brown	brown	brown	brown
-0.14622	0.243217	-0.08561	0.092004	0.104155	-0.21391	0.36725	0.255805	0.182787
0.100943	0.005863	0.338567	0.303592	0.243883	0.015744	2.16E-05	0.003699	0.039695
-0.14344	0.166498	0.019505	0.03975	0.089162	-0.20195	0.249421	0.072114	0.106198
0.107652	0.061363	0.827695	0.657255	0.318833	0.022791	0.004686	0.420416	0.234706
0.111982	-0.41626	0.280265	-0.36784	-0.36311	0.359114	-0.0637	0.036829	-0.07247
0.210044	1.13E-06	0.001416	2.1E-05	2.72E-05	3.38E-05	0.476809	0.681018	0.418143
0.021317	-0.38566	0.14495	-0.2652	-0.21091	0.458789	0.262047	0.062236	0.23077
0.811972	7.55E-06	0.10396	0.002585	0.017302	5.81E-08	0.002919	0.486988	0.009047
-0.63048	0.210971	0.407084	0.191816	0.382014	0.16181	0.453082	-0.05269	0.233617
1.97E-15	0.017271	2.04E-06	0.030743	9.35E-06	0.069145	8.86E-08	0.556348	0.008208
-0.43678	-0.10706	0.268143	-0.03021	0.158254	0.410284	0.623757	0.051225	0.436161
2.84E-07	0.230917	0.002304	0.735961	0.075567	1.66E-06	4.75E-15	0.567363	2.96E-07
-0.23976	-0.04045	0.20892	0.079761	0.259511	0.12187	0.393823	-0.01127	0.280448
0.006628	0.651639	0.018413	0.372715	0.003216	0.172279	4.63E-06	0.899903	0.001405
0.025992	0.118632	-0.26754	0.299112	0.197445	-0.0105	0.280077	-0.01714	0.370726
0.771762	0.184049	0.00236	0.000635	0.026078	0.906692	0.001427	0.848283	1.78E-05
-0.21905	0.102545	-0.2228	0.06604	0.407296	-0.1468	0.1977	0.621491	0.466527
0.013348	0.25129	0.011813	0.460706	2.01E-06	0.09958	0.025882	6.36E-15	3.23E-08
-0.58419	0.760312	-0.46839	0.503985	0.705767	-0.44435	0.692374	0.726857	0.758651
5.6E-13	3.52E-25	2.8E-08	1.54E-09	1.94E-20	1.66E-07	1.95E-19	3.9E-22	5.12E-25
-0.31581	0.384934	-0.34645	0.288435	0.340169	-0.20046	0.807822	0.579114	0.623482
0.000298	7.88E-06	6.61E-05	0.001007	9.12E-05	0.023842	1.77E-30	9.87E-13	4.92E-15
0.245578	-0.01898	0.072455	-0.09494	-0.08443	-0.22242	-0.32448	0.060993	-0.02512
0.005387	0.8323	0.418218	0.288346	0.34528	0.01196	0.000198	0.495745	0.77926
0.429256	-0.06958	-0.19665	-0.08466	-0.22743	-0.22311	-0.46957	0.198187	-0.20302
4.76E-07	0.436976	0.026702	0.343976	0.010127	0.011689	2.56E-08	0.025511	0.022069
-0.16051	0.324956	-0.16586	0.254229	0.259075	-0.30416	-0.20155	0.236416	-0.08335
0.071437	0.000193	0.062375	0.003924	0.00327	0.000508	0.023068	0.007451	0.351528
-0.14994	0.606337	-0.57777	0.406732	0.432806	-0.62353	0.041044	0.636984	0.301243
0.092452	4.21E-14	1.15E-12	2.08E-06	3.74E-07	4.89E-15	0.646836	8.26E-16	0.000578
0.030331	0.40171	-0.22604	0.070832	-0.04361	-0.42061	-0.12075	-0.19328	-0.22276
0.734976	2.85E-06	0.010611	0.428748	0.626376	8.5E-07	0.176286	0.029469	0.011825
0.523088	-0.32459	0.195567	-0.326	-0.61146	0.048553	-0.70701	-0.49212	-0.72175
2.81E-10	0.000197	0.027562	0.000184	2.25E-14	0.587766	1.56E-20	4.2E-09	1.04E-21
0.361473	-0.64044	0.408243	-0.482	-0.61048	0.565078	-0.3074	-0.70535	-0.5749
2.97E-05	5.15E-16	1.89E-06	9.61E-09	2.54E-14	4.49E-12	0.000439	2.09E-20	1.57E-12
0.409084	-0.33134	0.269538	-0.24362	-0.41624	0.130898	-0.54899	-0.6916	-0.65847
1.8E-06	0.000142	0.002181	0.00578	1.13E-06	0.14241	2.35E-11	2.23E-19	3.99E-17
0.189949	-0.21682	0.525559	-0.3225	-0.36035	0.203506	-0.3357	-0.07772	-0.42111
0.032438	0.014347	2.23E-10	0.000217	3.16E-05	0.021745	0.000114	0.3851	8.22E-07

S100A11	S100A16	S100A4	S100A6	S100A8	S100A9	S100Z	SAA1	SAA2
brown	brown	brown	brown	brown	brown	brown	brown	brown
0.272742	-0.05855	0.193308	0.163268	0.047314	0.098922	0.318611	0.009911	0.005336
0.00192	0.513222	0.029443	0.066643	0.597344	0.268515	0.000262	0.911942	0.952526
0.178096	0.024538	0.079862	0.152173	0.024684	0.046674	0.251146	-0.02495	-0.0581
0.045152	0.784213	0.372111	0.087654	0.782962	0.602317	0.004398	0.780708	0.516461
-0.0886	0.05965	-0.00295	-0.0329	-0.20776	-0.23558	-0.27204	-0.07884	-0.06723
0.3219	0.505303	0.973703	0.7135	0.019086	0.007671	0.001975	0.378253	0.45264
0.214897	0.192052	0.272096	0.110901	-0.04885	-0.06518	-0.19747	0.133568	0.186944
0.015256	0.030534	0.00197	0.214507	0.58545	0.466548	0.026059	0.134375	0.035334
0.259337	0.333553	0.398269	0.442563	0.387264	0.401447	-0.0675	0.081059	-0.00748
0.003238	0.000127	3.53E-06	1.89E-07	6.86E-06	2.9E-06	0.450852	0.364961	0.933471
0.459435	0.392905	0.568043	0.387633	0.28288	0.279557	-0.10536	0.264339	0.255398
5.53E-08	4.9E-06	3.28E-12	6.71E-06	0.001271	0.001458	0.238442	0.002672	0.003756
0.377079	0.36749	0.42318	0.363585	0.287032	0.264889	-0.12856	0.20292	0.182501
1.24E-05	2.14E-05	7.17E-07	2.65E-05	0.001069	0.002616	0.149743	0.022135	0.040012
0.211094	0.148506	0.300837	0.271152	0.062628	0.041057	0.006274	0.249308	0.282767
0.017205	0.095657	0.000589	0.002046	0.484243	0.646731	0.944191	0.004705	0.001277
0.586137	0.236982	0.5438	0.295601	0.370648	0.414095	0.12116	0.483441	0.463684
4.5E-13	0.007306	3.92E-11	0.000741	1.79E-05	1.3E-06	0.17481	8.55E-09	4.02E-08
0.860905	0.387978	0.771799	0.612139	0.692681	0.707033	0.553188	0.560966	0.539239
1.7E-38	6.58E-06	2.42E-26	2.07E-14	1.86E-19	1.55E-20	1.54E-11	6.91E-12	6.12E-11
0.689501	0.279102	0.607932	0.361562	0.330287	0.363428	0.302519	0.38199	0.412337
3.16E-19	0.001485	3.47E-14	2.96E-05	0.000149	2.67E-05	0.000546	9.36E-06	1.46E-06
0.000172	-0.07379	-0.22376	-0.15846	-0.1746	-0.09033	0.035871	-0.12383	-0.09575
0.998466	0.409663	0.011445	0.075185	0.049617	0.312485	0.688875	0.165417	0.284258
-0.2388	-0.19717	-0.33295	-0.19104	-0.2554	-0.27402	-0.00031	-0.11911	-0.10237
0.006855	0.026294	0.000131	0.03144	0.003756	0.001824	0.997249	0.182257	0.252103
-0.17381	-0.0032	-0.1525	0.055083	0.07578	0.10367	0.069927	-0.01504	-0.09977
0.050672	0.971483	0.08696	0.538496	0.39712	0.246097	0.434682	0.86673	0.264403
0.309564	0.084126	0.173814	0.246593	0.304461	0.304399	0.425923	0.253657	0.211437
0.000398	0.34704	0.050663	0.005193	0.000501	0.000502	5.97E-07	0.004008	0.017021
-0.18614	-0.1889	-0.33207	-0.10359	-0.05445	-0.10216	0.353848	-0.3077	-0.32164
0.036147	0.033423	0.000137	0.246461	0.543173	0.253059	4.48E-05	0.000433	0.000227
-0.77912	-0.41137	-0.79338	-0.51858	-0.55358	-0.58349	-0.24188	-0.62897	-0.62357
4.04E-27	1.55E-06	1.01E-28	4.23E-10	1.48E-11	6.06E-13	0.006149	2.41E-15	4.86E-15
-0.59514	-0.31213	-0.49187	-0.54002	-0.51773	-0.50884	-0.35232	-0.41271	-0.34571
1.6E-13	0.000354	4.29E-09	5.67E-11	4.57E-10	1.01E-09	4.86E-05	1.42E-06	6.87E-05
-0.68052	-0.40191	-0.64393	-0.52721	-0.43105	-0.42496	-0.23782	-0.43813	-0.41278
1.36E-18	2.82E-06	3.18E-16	1.92E-10	4.22E-07	6.36E-07	0.007096	2.58E-07	1.42E-06
-0.29965	-0.25771	-0.28426	-0.12912	-0.17666	-0.22758	-0.14958	-0.4046	-0.38497
0.000621	0.003443	0.0012	0.147947	0.046938	0.010077	0.093247	2.38E-06	7.86E-06

SAMD3	SAMHD1	SAMSN1	SAP130	SASH3	SAT1	SATB2	SCAMP2	SCIMP
brown	brown	brown	brown	brown	brown	brown	brown	brown
0.456007	0.150616	0.200479	-0.28431	0.200682	0.23397	-0.11654	0.13648	0.357997
7.14E-08	0.090984	0.023825	0.001198	0.02368	0.008109	0.191947	0.126015	3.59E-05
0.268582	-0.06497	0.01628	-0.22752	0.046433	0.153597	-0.06609	0.052365	0.235025
0.002265	0.468001	0.855843	0.010096	0.604191	0.084693	0.46036	0.558755	0.007819
-0.26209	-0.16476	-0.12476	0.604259	-0.34351	-0.22384	0.336731	-0.27816	-0.17871
0.002914	0.064163	0.162259	5.41E-14	7.69E-05	0.011413	0.000108	0.001543	0.04441
-0.07047	0.123722	0.188791	0.280527	-0.21556	0.098913	0.315462	-0.28237	0.028053
0.43114	0.165802	0.03353	0.001401	0.014937	0.26856	0.000303	0.001298	0.754225
0.166979	-0.28076	0.123286	-0.31685	0.052997	0.161452	-0.43261	-0.14255	0.166825
0.060607	0.001388	0.16731	0.000284	0.554013	0.069771	3.79E-07	0.109864	0.060849
0.161734	0.08618	0.384523	-0.17059	-0.04011	0.342058	-0.09537	-0.26901	0.246161
0.069278	0.335349	8.07E-06	0.055177	0.654339	8.28E-05	0.286172	0.002227	0.005275
0.103375	-0.06802	0.186691	-0.03303	-0.04394	0.266949	0.01408	-0.11958	0.131481
0.247454	0.447346	0.035587	0.71235	0.623743	0.002414	0.875152	0.180554	0.140623
0.083085	0.235652	0.261368	-0.26716	0.158614	0.239101	-0.231	0.094305	0.128561
0.353059	0.007651	0.002996	0.002394	0.074894	0.006784	0.008976	0.291602	0.149735
0.285623	0.368713	0.329475	0.047312	0.325813	0.503525	-0.02678	0.247159	0.384063
0.001134	2E-05	0.000155	0.597359	0.000186	1.6E-09	0.765001	0.005088	8.29E-06
0.569344	0.683299	0.842787	-0.59374	0.893537	0.844437	-0.53738	0.69229	0.858931
2.86E-12	8.71E-19	1.99E-35	1.88E-13	2.69E-45	1.08E-35	7.32E-11	1.98E-19	3.85E-38
0.572696	0.697728	0.718823	-0.40687	0.624077	0.552244	-0.31628	0.42683	0.661768
1.99E-12	7.89E-20	1.8E-21	2.07E-06	4.56E-15	1.69E-11	0.000292	5.61E-07	2.45E-17
0.006763	-0.16464	-0.34932	0.303648	0.029128	-0.21602	0.074304	0.121373	-0.01893
0.93985	0.064363	5.69E-05	0.00052	0.745124	0.014718	0.406406	0.174049	0.832684
-0.24114	-0.05923	-0.27409	0.593211	-0.02472	-0.27098	0.29366	0.266145	-0.15271
0.006312	0.508347	0.001819	2E-13	0.782665	0.00206	0.000806	0.002491	0.086524
-0.14159	-0.20626	-0.16211	0.055949	0.230509	-0.15226	-0.19111	0.383863	-0.01576
0.112307	0.019988	0.068622	0.532121	0.009128	0.087475	0.031377	8.39E-06	0.86038
0.163247	0.339918	0.288464	-0.08135	0.660282	0.325317	-0.28886	0.788356	0.425645
0.066678	9.23E-05	0.001006	0.363211	3.05E-17	0.00019	0.000989	3.82E-28	6.08E-07
-0.03769	-0.02388	-0.05411	-0.29846	0.199005	-0.11204	-0.17254	0.29118	0.032552
0.673997	0.789868	0.545717	0.000654	0.024898	0.209804	0.052407	0.000896	0.716368
-0.52683	-0.47038	-0.6492	0.46085	-0.46976	-0.75092	0.354391	-0.24178	-0.61441
1.99E-10	2.4E-08	1.52E-16	4.98E-08	2.52E-08	2.82E-24	4.36E-05	0.006172	1.56E-14
-0.26265	-0.27505	-0.40236	0.076591	-0.68006	-0.48407	0.418897	-0.74197	-0.57543
0.002853	0.00175	2.74E-06	0.392073	1.46E-18	8.13E-09	9.52E-07	1.88E-23	1.48E-12
-0.23811	-0.37627	-0.55978	0.012388	-0.48846	-0.5494	0.204325	-0.44519	-0.57431
0.007022	1.3E-05	7.82E-12	0.890062	5.68E-09	2.25E-11	0.02121	1.57E-07	1.67E-12
-0.15455	-0.4064	-0.35925	0.633052	-0.37797	-0.30626	0.319923	-0.31752	-0.27453
0.08275	2.13E-06	3.36E-05	1.4E-15	1.18E-05	0.000462	0.000246	0.000275	0.001787

SCIN	SCML4	SCN3A	SCPEP1	SCRN1	SECTM1	SELE	SELL	SELP
brown	brown	brown	brown	brown	brown	brown	brown	brown
0.069419	0.184341	-0.24038	0.380669	-0.09188	0.052117	0.203958	0.284353	0.277877
0.438036	0.038015	0.006486	1.01E-05	0.304239	0.560625	0.021449	0.001195	0.001561
-0.10287	0.066223	-0.2308	0.239518	-0.0551	-0.10837	0.020629	0.182091	0.154739
0.24979	0.459465	0.009037	0.006685	0.538389	0.225229	0.817932	0.040469	0.082377
-0.19631	0.02767	0.305511	-0.46516	0.129493	0.091417	-0.2926	-0.49735	-0.24795
0.026968	0.757469	0.000478	3.59E-08	0.14678	0.306699	0.000843	2.71E-09	0.004943
-0.0567	0.246863	0.111329	-0.15129	0.214715	0.16898	-0.04537	-0.25709	-0.06248
0.526605	0.005143	0.212733	0.089526	0.015345	0.057544	0.61249	0.003525	0.485276
-0.14466	0.050964	-0.4512	0.114829	-0.45877	0.108682	-0.09266	0.111586	0.025162
0.104668	0.569336	1.02E-07	0.198611	5.81E-08	0.223878	0.300138	0.211671	0.778858
-0.03601	0.323317	-0.29839	0.162632	-0.1109	0.214753	0.072074	0.032778	0.088817
0.68774	0.000209	0.000656	0.067724	0.214519	0.015326	0.420672	0.714486	0.320715
-0.11476	0.204078	-0.25997	0.115588	0.033295	0.108184	0.055326	0.021154	0.092977
0.19889	0.02137	0.003161	0.195638	0.710186	0.226018	0.536706	0.813381	0.298483
0.23396	0.039477	-0.07472	0.291326	-0.12174	-0.05362	0.175682	0.332587	0.084893
0.008112	0.659466	0.403773	0.000891	0.172749	0.549389	0.048194	0.000133	0.342646
0.255792	0.302912	-0.25506	0.360763	-0.16178	0.592754	0.236644	0.147954	0.363244
0.003701	0.000537	0.003803	3.09E-05	0.069189	2.11E-13	0.007392	0.09691	2.7E-05
0.697931	0.366704	-0.61565	0.880485	-0.48583	0.605558	0.519456	0.722742	0.552265
7.62E-20	2.23E-05	1.33E-14	2.44E-42	7.04E-09	4.63E-14	3.91E-10	8.6E-22	1.69E-11
0.493066	0.475329	-0.39019	0.645325	-0.10912	0.566523	0.465732	0.542696	0.427231
3.88E-09	1.63E-08	5.76E-06	2.62E-16	0.222019	3.86E-12	3.44E-08	4.37E-11	5.46E-07
-0.13033	-0.01836	0.033005	-0.12996	0.19191	0.060613	-0.10345	0.02945	0.00927
0.144173	0.837661	0.712598	0.145312	0.030659	0.49844	0.247105	0.742402	0.91762
0.01594	-0.20422	0.328426	-0.32899	0.140538	0.033609	-0.18843	-0.25645	-0.14782
0.858823	0.02128	0.000163	0.000159	0.115036	0.707577	0.033877	0.003611	0.097221
0.064826	-0.30421	-0.07107	-0.142	-0.32437	0.150981	-0.19885	-0.04196	-0.10462
0.469007	0.000507	0.427221	0.111263	0.000199	0.090194	0.025016	0.639464	0.241789
0.490884	-0.03156	-0.23823	0.379077	-0.31508	0.370005	0.170368	0.346017	0.247197
4.65E-09	0.724683	0.006994	1.11E-05	0.000309	1.86E-05	0.055493	6.76E-05	0.005081
0.168534	-0.16225	-0.00901	0.141166	0.030323	-0.43063	-0.06388	0.162489	-0.01287
0.058216	0.068389	0.919957	0.113406	0.735041	4.34E-07	0.475564	0.067971	0.885848
-0.31834	-0.40587	0.621413	-0.69189	0.362187	-0.51222	-0.40814	-0.54105	-0.46031
0.000265	2.2E-06	6.42E-15	2.12E-19	2.86E-05	7.48E-10	1.91E-06	5.13E-11	5.18E-08
-0.42933	-0.07787	0.468007	-0.46391	0.446009	-0.48574	-0.17615	-0.41648	-0.29252
4.74E-07	0.384177	2.89E-08	3.95E-08	1.48E-07	7.1E-09	0.047596	1.11E-06	0.000846
-0.37169	-0.28417	0.386931	-0.37382	0.282824	-0.54394	-0.15599	-0.20994	-0.26063
1.69E-05	0.001205	7E-06	1.5E-05	0.001274	3.87E-11	0.07989	0.017839	0.003082
-0.37255	-0.14626	0.20702	-0.46646	-0.08059	-0.15895	-0.27783	-0.50091	-0.19293
1.61E-05	0.100848	0.019528	3.25E-08	0.367756	0.07428	0.001564	2E-09	0.029771

SELPLG	SEMA4G	SEMA6D	44440	SERPINA1	SERPINA5	SERPINB1	SERPINB6	SERPINB8
brown	brown	brown	brown	brown	brown	brown	brown	brown
0.311967	-0.05954	-0.31462	0.259665	0.091156	0.014689	0.442091	0.191795	0.105011
0.000356	0.506119	0.000315	0.003197	0.30809	0.869801	1.96E-07	0.030761	0.24001
0.251173	0.010293	-0.2487	0.120772	-0.06623	0.001195	0.361387	0.168187	0.069493
0.004394	0.908558	0.004811	0.176207	0.459396	0.989365	2.99E-05	0.058743	0.437545
-0.63952	0.229264	0.463477	-0.31183	-0.20817	-0.06029	-0.42065	-0.2151	0.176249
5.84E-16	0.009521	4.08E-08	0.000359	0.018848	0.500723	8.48E-07	0.015156	0.047464
-0.4983	-0.10466	0.542647	-0.35412	0.002161	0.131657	-0.01866	0.109856	0.484855
2.5E-09	0.241607	4.39E-11	4.42E-05	0.980767	0.140089	0.835075	0.218883	7.63E-09
0.181362	-0.14029	-0.24132	0.146162	0.065433	0.23548	0.352044	0.357751	0.184987
0.041291	0.115693	0.006272	0.101068	0.464852	0.007697	4.93E-05	3.64E-05	0.037334
-0.14562	-0.33804	0.218031	-0.1812	0.162629	0.307932	0.416423	0.436237	0.606692
0.102345	0.000102	0.013798	0.041474	0.067731	0.000428	1.12E-06	2.95E-07	4.03E-14
-0.05636	-0.15521	0.192551	-0.09458	0.142679	0.327722	0.370525	0.358738	0.465718
0.529076	0.081428	0.030097	0.2902	0.109552	0.000169	1.8E-05	3.45E-05	3.44E-08
0.226139	-0.49973	-0.1154	-0.10321	0.239553	0.269116	0.341678	0.368058	0.190931
0.010574	2.21E-09	0.196359	0.248232	0.006677	0.002217	8.44E-05	2.07E-05	0.031537
-0.12793	-0.27793	-0.02384	0.215559	0.537818	0.280937	0.268252	0.309928	0.567408
0.151766	0.001558	0.790197	0.014937	7.02E-11	0.001377	0.002294	0.000391	3.51E-12
0.691423	-0.66653	-0.50424	0.572261	0.856268	0.550291	0.838902	0.716557	0.58925
2.29E-19	1.2E-17	1.5E-09	2.09E-12	1.14E-37	2.06E-11	8.04E-35	2.75E-21	3.16E-13
0.405167	-0.50134	-0.19738	0.307913	0.618135	0.268093	0.610124	0.575623	0.470392
2.3E-06	1.93E-09	0.026125	0.000429	9.75E-15	0.002309	2.65E-14	1.45E-12	2.4E-08
-0.0799	0.384175	0.099321	0.041626	-0.08161	-0.24028	-0.22746	-0.12821	-0.1949
0.371907	8.24E-06	0.26658	0.642172	0.361702	0.006509	0.010118	0.150858	0.028103
-0.23481	0.366818	0.106074	0.086609	-0.09372	-0.13478	-0.52658	-0.34618	-0.23656
0.007877	2.22E-05	0.235257	0.332943	0.294599	0.130859	2.03E-10	6.7E-05	0.007413
0.157013	0.128932	-0.34457	0.452434	0.050092	0.044847	-0.26927	-0.05198	-0.34051
0.077916	0.148553	7.28E-05	9.29E-08	0.575974	0.616613	0.002204	0.56166	8.96E-05
0.450635	-0.18644	-0.43743	0.596432	0.490112	0.290277	0.165529	0.248229	0.030219
1.06E-07	0.035841	2.71E-07	1.37E-13	4.96E-09	0.000931	0.06291	0.004894	0.735921
0.507729	-0.02517	-0.26161	0.165282	-0.17625	0.042811	0.144915	-0.01848	-0.41834
1.11E-09	0.778836	0.002968	0.06331	0.047469	0.632721	0.104044	0.836643	9.87E-07
-0.32429	0.614294	0.268343	-0.31622	-0.64929	-0.44103	-0.77125	-0.68208	-0.70207
0.0002	1.58E-14	0.002286	0.000293	1.5E-16	2.11E-07	2.76E-26	1.06E-18	3.73E-20
-0.44175	0.264241	0.428014	-0.56897	-0.61508	-0.47823	-0.38099	-0.48516	-0.31737
2E-07	0.002683	5.18E-07	2.97E-12	1.43E-14	1.3E-08	9.92E-06	7.44E-09	0.000277
-0.09906	0.387508	0.039317	-0.33298	-0.5369	-0.51813	-0.394	-0.55498	-0.60791
0.26784	6.76E-06	0.660755	0.000131	7.67E-11	4.41E-10	4.58E-06	1.28E-11	3.48E-14
-0.4834	0.561006	0.17988	0.000248	-0.40673	-0.18446	-0.41947	-0.57092	-0.12537
8.58E-09	6.89E-12	0.043007	0.997789	2.08E-06	0.037885	9.16E-07	2.41E-12	0.160183

SERPINB9	SERPINF2	SERPING1	SERTAD3	SFMBT2	SFRP2	SFRP4	SFXN1	SFXN2
brown	brown	brown	brown	brown	brown	brown	brown	brown
0.263659	0.109664	0.272738	0.159356	0.198201	0.183957	0.054397	-0.43242	0.169795
0.002744	0.219697	0.001921	0.073527	0.025501	0.038424	0.543579	3.84E-07	0.056332
0.135336	0.031491	0.179759	0.119586	0.123515	0.098656	-0.0603	-0.38391	0.049627
0.12925	0.725242	0.04315	0.180521	0.166517	0.269809	0.500692	8.37E-06	0.579525
-0.37356	-0.16618	-0.30273	-0.17899	-0.25812	-0.35051	-0.06102	0.583706	0.077574
1.52E-05	0.061871	0.000541	0.044071	0.003391	5.35E-05	0.495578	5.92E-13	0.386012
-0.09017	-0.18677	-0.0045	-0.06571	-0.07349	-0.13261	0.223253	0.510713	0.151997
0.313392	0.035505	0.959986	0.462934	0.411587	0.137229	0.011636	8.55E-10	0.088026
-0.11091	0.061081	0.066849	0.489075	-0.29629	0.046743	-0.0553	0.012252	0.057794
0.214485	0.495123	0.455225	5.4E-09	0.000719	0.601777	0.536913	0.891258	0.518662
0.050433	-0.10628	0.186668	0.282395	-0.11325	0.07683	0.238859	0.30619	0.172895
0.573375	0.234329	0.03561	0.001297	0.204898	0.390597	0.006842	0.000464	0.051916
0.027887	-0.1152	0.185116	0.249589	-0.07708	0.027216	0.168403	0.064177	0.140322
0.755634	0.197141	0.037199	0.004657	0.38907	0.761335	0.058413	0.473484	0.115602
0.208105	0.012503	0.326078	-0.02152	0.166777	0.052045	0.326747	-0.12245	0.030022
0.018884	0.889045	0.000183	0.810213	0.060924	0.561167	0.000177	0.170216	0.737575
0.219458	0.298504	0.531261	0.326735	0.048409	0.281366	0.51373	-0.11094	0.225176
0.013175	0.000653	1.31E-10	0.000177	0.588875	0.001353	6.54E-10	0.214331	0.010919
0.462313	0.582288	0.81882	0.634786	0.443237	0.515694	0.621249	-0.63804	0.57225
4.46E-08	6.94E-13	6.41E-32	1.11E-15	1.8E-07	5.49E-10	6.56E-15	7.15E-16	2.09E-12
0.449771	0.277618	0.691379	0.373098	0.342579	0.346787	0.565129	-0.35458	0.435068
1.13E-07	0.001578	2.31E-19	1.56E-05	8.06E-05	6.49E-05	4.47E-12	4.31E-05	3.2E-07
-0.11574	0.038365	0.086467	0.053622	-0.06263	0.018832	-0.1101	-0.10162	-0.04364
0.195032	0.668481	0.333739	0.54934	0.484232	0.833556	0.217851	0.255614	0.626149
-0.2992	0.137868	-0.14368	-0.20028	0.055181	-0.22404	-0.03669	-0.02114	0.100135
0.000633	0.122172	0.107069	0.023968	0.537778	0.011338	0.682142	0.813499	0.262661
-0.27613	0.426681	-0.0072	0.261201	-0.1057	-0.12778	0.014222	-0.25127	-0.0063
0.001676	5.67E-07	0.935928	0.003016	0.236913	0.152248	0.873904	0.004378	0.943978
0.081341	0.608079	0.444744	0.334846	0.358339	0.198062	0.378725	-0.62662	0.386907
0.36329	3.41E-14	1.62E-07	0.000119	3.53E-05	0.025606	1.13E-05	3.28E-15	7.01E-06
-0.00131	0.068394	-0.14763	-0.126	0.287796	0.025436	-0.23741	-0.47378	0.057805
0.988377	0.444845	0.097641	0.158077	0.001035	0.776514	0.007197	1.84E-08	0.518588
-0.38371	-0.31517	-0.74873	-0.54399	-0.22903	-0.49771	-0.56343	0.308082	-0.35587
8.46E-06	0.000307	4.51E-24	3.85E-11	0.009597	2.63E-09	5.35E-12	0.000426	4.03E-05
-0.03034	-0.59073	-0.58542	-0.56646	-0.27897	-0.24617	-0.42416	0.577662	-0.51578
0.734919	2.66E-13	4.88E-13	3.88E-12	0.001493	0.005273	6.71E-07	1.16E-12	5.45E-10
-0.02174	-0.42094	-0.52488	-0.49812	-0.23052	-0.19548	-0.56641	0.185859	-0.55344
0.808311	8.32E-07	2.38E-10	2.54E-09	0.009125	0.02763	3.9E-12	0.036431	1.5E-11
-0.39912	-0.20741	-0.5354	-0.14307	-0.19462	-0.2169	-0.51485	0.31531	0.083588
3.35E-06	0.019298	8.85E-11	0.108573	0.028341	0.014308	5.92E-10	0.000305	0.350143

SGK1	SH2B3	SH2D1A	SH2D3A	SH3BGRL	SH3BGRL3	SH3BP1	SH3TC1	SHF
brown	brown	brown	brown	brown	brown	brown	brown	brown
0.217439	0.141683	0.362031	0.290926	0.18473	0.156536	0.273283	-0.00165	-0.18934
0.014063	0.112077	2.89E-05	0.000906	0.037604	0.078835	0.001879	0.985349	0.033005
0.223276	-0.01276	0.230687	0.185378	0.114382	0.204225	0.128317	-0.11744	-0.10839
0.011627	0.886751	0.009073	0.036927	0.200375	0.021275	0.150514	0.188528	0.225136
-0.36888	0.067143	-0.16665	-0.23278	-0.08701	-0.3078	-0.34537	-0.2888	0.211414
1.98E-05	0.453238	0.061119	0.008448	0.330687	0.000431	6.99E-05	0.000991	0.017033
-0.21474	0.266549	0.129113	-0.18476	0.208174	-0.40316	-0.39797	-0.30338	-0.2858
0.015331	0.002452	0.14798	0.037577	0.018844	2.61E-06	3.6E-06	0.000526	0.001125
-2.5E-05	-0.19763	0.282978	0.08418	0.120881	0.279647	-0.13388	-0.11497	-0.01936
0.999781	0.025934	0.001266	0.346729	0.175815	0.001452	0.133458	0.198062	0.82899
-0.05559	0.185798	0.412161	-0.02466	0.412343	-0.13722	-0.35649	-0.25032	-0.46406
0.534786	0.036494	1.47E-06	0.783137	1.46E-06	0.12395	3.89E-05	0.004533	3.9E-08
0.015621	0.194417	0.275741	-0.01539	0.127106	0.09994	-0.20098	-0.2327	-0.29391
0.861622	0.028506	0.001702	0.863619	0.154439	0.263595	0.023469	0.008471	0.000797
0.04261	0.068383	0.166561	-0.04522	0.239809	-0.14494	-0.11534	0.011084	-0.58008
0.634314	0.444923	0.061265	0.613671	0.006617	0.103985	0.196608	0.901569	8.87E-13
0.215426	0.664808	0.243889	0.266202	0.051128	0.304379	0.287806	0.378735	-0.33295
0.015001	1.56E-17	0.005724	0.002486	0.568098	0.000503	0.001034	1.13E-05	0.000131
0.529496	0.617485	0.649323	0.619926	0.468928	0.559443	0.622307	0.701717	-0.68951
1.55E-10	1.06E-14	1.49E-16	7.77E-15	2.69E-08	8.1E-12	5.73E-15	3.96E-20	3.15E-19
0.20874	0.534645	0.606304	0.49606	0.461076	0.180205	0.433081	0.399241	-0.65584
0.018516	9.51E-11	4.23E-14	3.02E-09	4.89E-08	0.042626	3.67E-07	3.33E-06	5.86E-17
0.036797	0.070041	-0.15268	0.076406	-0.43583	0.339003	0.331657	-0.02702	0.313601
0.681276	0.433932	0.08659	0.393222	3.03E-07	9.67E-05	0.00014	0.763016	0.00033
-0.12908	0.059263	-0.33536	-0.01741	-0.41864	0.034475	0.227309	0.172113	0.383364
0.148077	0.508073	0.000116	0.845979	9.68E-07	0.700395	0.010168	0.053003	8.64E-06
0.009826	-0.25771	-0.1676	0.190024	-0.33624	0.357699	0.349049	0.44748	0.400085
0.91269	0.003444	0.059635	0.032369	0.000111	3.65E-05	5.77E-05	1.33E-07	3.16E-06
0.335902	0.30941	0.136976	0.462968	-0.05177	0.539746	0.703313	0.76366	-0.11033
0.000113	0.000401	0.124631	4.24E-08	0.563221	5.83E-11	3E-20	1.64E-25	0.216876
0.289099	-0.25564	-0.0705	0.069917	0.022297	0.167503	0.204656	0.121608	0.052521
0.000979	0.003723	0.430925	0.434747	0.803504	0.059792	0.020997	0.173211	0.557586
-0.26862	-0.48797	-0.64614	-0.40029	-0.43215	-0.28306	-0.19418	-0.32189	0.769756
0.002261	5.91E-09	2.34E-16	3.12E-06	3.91E-07	0.001262	0.028706	0.000224	3.94E-26
-0.32603	-0.41336	-0.32489	-0.48531	0.036452	-0.66934	-0.63172	-0.64807	0.252795
0.000184	1.37E-06	0.000194	7.35E-09	0.684105	7.83E-18	1.67E-15	1.78E-16	0.004138
-0.16453	-0.5153	-0.45681	-0.35127	-0.25105	-0.27778	-0.27505	-0.41537	0.484149
0.064541	5.69E-10	6.73E-08	5.14E-05	0.004414	0.001567	0.00175	1.2E-06	8.08E-09
-0.24943	-0.00168	-0.24786	-0.1881	-0.38736	-0.02108	-0.16868	-0.36453	0.643191
0.004684	0.985025	0.004961	0.0342	6.83E-06	0.814049	0.057996	2.52E-05	3.53E-16

SHISA5	SHROOM2	SIGLEC1	SIGLEC10	SIGLEC11	SIGLEC16	SIGLEC7	SIGLEC9	SIL1
brown	brown	brown	brown	brown	brown	brown	brown	brown
0.196024	-0.2988	0.492398	0.047756	0.217772	0.129509	0.242188	0.116003	0.156312
0.027195	0.000644	4.1E-09	0.593921	0.013913	0.146731	0.006083	0.194028	0.079271
0.099391	-0.17502	0.307693	0.039561	0.077032	0.054429	0.105154	0.005299	0.072505
0.266239	0.049062	0.000433	0.658784	0.389347	0.543337	0.239368	0.952853	0.417897
-0.0737	0.472083	-0.31911	-0.32547	-0.27633	-0.19359	-0.05006	-0.05513	-0.27424
0.41023	2.11E-08	0.000255	0.000189	0.001662	0.029203	0.576215	0.538126	0.001808
-0.18902	0.324826	-0.11791	-0.30474	-0.23679	-0.17426	0.103039	0.126	-0.18387
0.033308	0.000195	0.186744	0.000495	0.007356	0.050071	0.248999	0.158085	0.038513
-0.00201	-0.18158	-0.03706	-0.07396	-0.03233	-0.06979	0.174976	0.117419	0.268816
0.982098	0.041038	0.67913	0.408603	0.718245	0.435602	0.049115	0.188605	0.002244
-0.1455	0.035685	0.022207	-0.20755	-0.15791	-0.13726	0.250004	0.262833	0.119849
0.102632	0.690408	0.804274	0.019209	0.076207	0.123841	0.004587	0.002832	0.179558
-0.01414	0.020779	0.027325	-0.06396	0.002315	-0.08806	0.1495	0.122854	0.015632
0.874663	0.816636	0.760403	0.474994	0.979396	0.324858	0.093433	0.168814	0.861527
0.004562	-0.38352	0.269894	0.044017	-0.0159	0.032914	0.112868	0.190219	0.17176
0.959406	8.56E-06	0.00215	0.623154	0.859212	0.713352	0.206438	0.032188	0.053499
0.47909	-0.07291	0.308378	0.300273	0.142276	0.261151	0.533992	0.552295	0.423297
1.21E-08	0.415292	0.00042	0.000604	0.110569	0.003021	1.01E-10	1.68E-11	7.11E-07
0.610161	-0.71844	0.796011	0.637425	0.604904	0.617305	0.842713	0.871469	0.747718
2.64E-14	1.94E-21	4.95E-29	7.78E-16	5.01E-14	1.08E-14	2.04E-35	1.72E-40	5.6E-24
0.523709	-0.4181	0.79332	0.234684	0.410593	0.352038	0.597391	0.615067	0.502589
2.65E-10	1E-06	1.03E-28	0.007912	1.63E-06	4.93E-05	1.23E-13	1.43E-14	1.73E-09
0.159596	0.193186	-0.05572	0.060563	0.036281	0.0125	0.043467	-0.1153	0.089322
0.073088	0.029547	0.533824	0.498795	0.685512	0.88907	0.627511	0.196777	0.317963
0.250245	0.146704	-0.05479	0.074808	0.093375	0.185531	0.005015	-0.04371	-0.14536
0.004546	0.099796	0.540702	0.403222	0.296409	0.036769	0.955375	0.625624	0.102982
0.375841	-0.19221	0.01367	0.206455	0.249955	0.286148	0.150872	0.114914	0.28393
1.33E-05	0.030398	0.878762	0.019871	0.004595	0.001109	0.09043	0.198277	0.001217
0.664501	-0.49791	0.502622	0.574693	0.55903	0.641192	0.545706	0.52827	0.520994
1.63E-17	2.58E-09	1.73E-09	1.6E-12	8.45E-12	4.65E-16	3.25E-11	1.74E-10	3.4E-10
-0.0475	-0.23634	0.135751	0.240672	0.296651	0.185616	-0.12872	-0.16876	-0.06376
0.595864	0.00747	0.128068	0.006418	0.000708	0.036681	0.149226	0.05787	0.476347
-0.32569	0.483889	-0.55392	-0.27216	-0.24297	-0.31692	-0.62914	-0.65343	-0.60789
0.000187	8.25E-09	1.43E-11	0.001966	0.005916	0.000283	2.35E-15	8.3E-17	3.49E-14
-0.72361	0.547103	-0.54271	-0.5385	-0.51567	-0.60865	-0.73608	-0.67489	-0.63897
7.29E-22	2.83E-11	4.37E-11	6.58E-11	5.5E-10	3.17E-14	6.28E-23	3.31E-18	6.3E-16
-0.48182	0.299334	-0.44764	-0.25565	-0.3104	-0.44403	-0.7431	-0.70906	-0.51747
9.75E-09	0.000629	1.32E-07	0.003721	0.000383	1.7E-07	1.48E-23	1.08E-20	4.68E-10
-0.10219	0.3555	-0.36631	-0.25517	-0.19277	-0.21335	-0.17054	-0.32674	-0.43212
0.252954	4.11E-05	2.28E-05	0.003789	0.029902	0.016026	0.055244	0.000177	3.92E-07

SIRPB2	SIRPG	SIRT4	SIT1	SKAP1	SKIDA1	SLA	SLA2	SLAMF1
brown	brown	brown	brown	brown	brown	brown	brown	brown
0.176096	0.225621	-0.12667	0.342206	0.37877	-0.18476	0.167526	0.265809	0.31953
0.04766	0.010758	0.155854	8.22E-05	1.13E-05	0.037573	0.059756	0.002524	0.00025
0.068976	0.081116	-0.01454	0.165806	0.246491	-0.112	0.104841	0.056201	0.137827
0.440974	0.364624	0.871138	0.062464	0.005213	0.209986	0.240775	0.530278	0.122284
-0.19333	-0.16722	0.312818	-0.28275	-0.23175	0.396264	-0.39369	-0.09657	-0.09725
0.029423	0.060229	0.000343	0.001278	0.00875	3.99E-06	4.67E-06	0.280111	0.276724
0.049797	-0.05899	0.193804	-0.08241	0.034767	0.234388	-0.11879	0.104008	0.180338
0.578225	0.510005	0.02902	0.357	0.697981	0.007993	0.183464	0.244555	0.042471
0.082054	0.067443	0.415852	0.246509	0.328081	-0.05614	0.060822	0.065246	0.200998
0.359085	0.451215	1.16E-06	0.005209	0.000166	0.530686	0.496961	0.466129	0.023457
0.226082	0.086686	0.314262	0.216265	0.378475	0.039159	0.120729	0.217307	0.403524
0.010594	0.332512	0.000321	0.014604	1.15E-05	0.662033	0.176361	0.014123	2.55E-06
0.160824	0.143502	0.17099	0.143557	0.209083	-0.16716	0.071337	0.172502	0.318987
0.070879	0.107498	0.054594	0.107362	0.01832	0.060322	0.425451	0.052459	0.000257
0.159731	0.101419	-0.13476	0.240186	0.1819	-0.025	0.184195	0.134874	0.234667
0.072842	0.256559	0.130903	0.00653	0.040682	0.780251	0.038171	0.130574	0.007917
0.358827	0.450032	-0.21195	0.347937	0.301479	-0.29961	0.410399	0.541043	0.458438
3.43E-05	1.11E-07	0.016747	6.12E-05	0.000572	0.000622	1.65E-06	5.14E-11	5.96E-08
0.822019	0.726695	-0.44945	0.8144	0.705923	-0.60039	0.83238	0.752529	0.75484
2.34E-32	4.02E-22	1.15E-07	2.49E-31	1.89E-20	8.6E-14	7.76E-34	1.99E-24	1.2E-24
0.568183	0.511282	-0.35774	0.660644	0.620288	-0.3051	0.488776	0.637506	0.688684
3.23E-12	8.13E-10	3.64E-05	2.89E-17	7.42E-15	0.000487	5.54E-09	7.69E-16	3.61E-19
-0.20933	-0.05016	-0.0578	-0.2047	-0.16949	-0.02577	-0.09748	-0.09584	-0.2216
0.01818	0.575455	0.518632	0.02097	0.056789	0.773628	0.275575	0.283791	0.012287
-0.1459	-0.0133	-0.14911	-0.24325	-0.3812	0.147311	-0.22307	-0.04598	-0.25645
0.101685	0.881984	0.094297	0.005857	9.8E-06	0.098386	0.011707	0.607714	0.00361
-0.0158	0.053174	-0.00148	0.072722	-0.17226	0.01621	-0.04531	-0.00811	-0.1774
0.860065	0.552687	0.986851	0.416499	0.052796	0.856464	0.612986	0.927898	0.046009
0.424386	0.477771	-0.45185	0.402594	0.150252	-0.3353	0.414746	0.423017	0.228222
6.61E-07	1.35E-08	9.7E-08	2.7E-06	0.091776	0.000116	1.25E-06	7.25E-07	0.009861
0.010164	-0.04256	-0.21415	-0.01266	-0.09277	-0.12412	0.032108	-0.18724	-0.2383
0.909699	0.634749	0.015621	0.887695	0.299556	0.164421	0.720075	0.035039	0.006978
-0.59464	-0.57151	0.105861	-0.66703	-0.70484	0.336015	-0.67	-0.65489	-0.7507
1.69E-13	2.26E-12	0.236206	1.11E-17	2.29E-20	0.000112	7.07E-18	6.72E-17	2.95E-24
-0.51999	-0.58044	0.296668	-0.52277	-0.35095	0.383282	-0.47424	-0.54685	-0.42403
3.72E-10	8.52E-13	0.000707	2.89E-10	5.22E-05	8.68E-06	1.78E-08	2.9E-11	6.77E-07
-0.5373	-0.48943	0.097684	-0.48694	-0.40622	0.154809	-0.38724	-0.59111	-0.54929
7.38E-11	5.25E-09	0.27458	6.44E-09	2.15E-06	0.082237	6.87E-06	2.55E-13	2.28E-11
-0.34133	-0.17609	0.398344	-0.39945	-0.27557	0.246508	-0.45063	-0.12175	-0.26648
8.59E-05	0.047662	3.52E-06	3.28E-06	0.001714	0.00521	1.06E-07	0.172696	0.002459

SLAMF6	SLAMF7	SLAMF8	SLC10A4	SLC11A1	SLC12A7	SLC15A3	SLC15A4	SLC16A5
brown	brown	brown	brown	brown	brown	brown	brown	brown
0.318984	0.432178	0.143121	-0.28965	-0.03988	0.006748	0.294806	0.273209	0.180487
0.000257	3.9E-07	0.108447	0.000956	0.65618	0.939977	0.000767	0.001885	0.042297
0.141224	0.217082	0.047633	-0.24686	-0.08541	-0.15732	0.102874	0.238233	0.064124
0.113257	0.014226	0.594872	0.005143	0.339721	0.077322	0.249764	0.006994	0.47385
-0.0963	-0.04816	-0.01005	0.44795	-0.16899	-0.11795	-0.35322	-0.15477	-0.17129
0.281492	0.590812	0.910695	1.29E-07	0.057534	0.186596	4.64E-05	0.082306	0.054166
0.152925	0.183104	0.175695	0.371584	-0.11185	-0.0795	-0.23933	0.182881	-0.08382
0.08608	0.039348	0.048178	1.7E-05	0.210586	0.374318	0.006729	0.039593	0.348829
0.094789	0.149227	0.131771	-0.14271	-0.05357	-0.2772	-0.00749	0.123217	-0.01535
0.289123	0.094039	0.139742	0.109478	0.549751	0.001605	0.933433	0.167549	0.863957
0.302444	0.338984	0.304702	0.126942	-0.0632	-0.187	-0.09122	0.364905	-0.01689
0.000548	9.68E-05	0.000496	0.154973	0.48024	0.035277	0.307722	2.46E-05	0.850504
0.205572	0.229694	0.192819	-0.0685	-0.0658	-0.09429	-0.0659	0.206757	0.02712
0.020417	0.009383	0.029863	0.444129	0.462348	0.291703	0.46165	0.019687	0.762152
0.112979	0.178978	0.193556	-0.2522	0.087728	0.054713	0.134381	0.124605	0.149332
0.20599	0.044081	0.029231	0.00423	0.326708	0.541235	0.132	0.162777	0.093805
0.566996	0.440927	0.626645	0.014805	0.450163	0.491793	0.303068	0.403878	0.47365
3.67E-12	2.12E-07	3.27E-15	0.868783	1.1E-07	4.32E-09	0.000533	2.49E-06	1.86E-08
0.823731	0.766201	0.836809	-0.47717	0.705639	0.503557	0.864773	0.656265	0.702955
1.35E-32	9.09E-26	1.68E-34	1.41E-08	1.99E-20	1.6E-09	3.31E-39	5.5E-17	3.19E-20
0.659576	0.70046	0.531847	-0.18039	0.338389	0.391518	0.648352	0.519528	0.462812
3.39E-17	4.93E-20	1.24E-10	0.042412	9.98E-05	5.32E-06	1.71E-16	3.88E-10	4.29E-08
-0.08676	-0.16498	-0.05245	0.218168	0.025272	0.103497	0.005313	-0.01619	0.046248
0.332082	0.063794	0.558136	0.013737	0.777921	0.24689	0.952723	0.856607	0.60564
-0.11556	-0.13365	-0.03212	0.133495	0.136915	0.271283	0.036392	-0.21214	0.139375
0.19574	0.134138	0.720015	0.13459	0.124799	0.002035	0.6846	0.016649	0.118103
-0.10893	-0.10561	0.036674	-0.08107	0.313563	0.290946	0.255622	-0.15718	0.192976
0.222817	0.237318	0.682286	0.364898	0.000331	0.000905	0.003725	0.077605	0.029727
0.389157	0.321949	0.482943	-0.33245	0.654307	0.61748	0.703319	0.236434	0.620678
6.13E-06	0.000223	8.9E-09	0.000134	7.31E-17	1.06E-14	2.99E-20	0.007446	7.05E-15
-0.16186	-0.164	-0.21574	-0.40641	-0.00588	-0.19902	0.201373	-0.10989	-0.0452
0.069051	0.065422	0.014854	2.13E-06	0.947689	0.02489	0.023194	0.218752	0.613844
-0.73276	-0.66455	-0.71304	0.228906	-0.42524	-0.34277	-0.47121	-0.68001	-0.51628
1.22E-22	1.62E-17	5.25E-21	0.009637	6.24E-07	7.99E-05	2.25E-08	1.48E-18	5.21E-10
-0.54986	-0.50332	-0.67224	0.372886	-0.66434	-0.5328	-0.70346	-0.42175	-0.6841
2.15E-11	1.63E-09	5.01E-18	1.58E-05	1.67E-17	1.13E-10	2.92E-20	7.88E-07	7.64E-19
-0.62478	-0.60327	-0.73053	0.058657	-0.51928	-0.40964	-0.50056	-0.52845	-0.57405
4.16E-15	6.1E-14	1.9E-22	0.51243	3.97E-10	1.73E-06	2.06E-09	1.71E-10	1.72E-12
-0.20784	-0.18045	-0.2022	0.149516	-0.28697	-0.20983	-0.35293	-0.34906	-0.18386
0.01904	0.042339	0.022622	0.093398	0.001071	0.017899	4.71E-05	5.77E-05	0.038533

SLC25A19	SLC25A45	SLC29A1	SLC29A3	SLC29A4	SLC2A5	SLC2A9	SLC35D2	SLC35F1
brown	brown	brown	brown	brown	brown	brown	brown	brown
0.143402	0.367816	0.251599	0.460559	-0.42702	0.084989	0.186884	0.306854	-0.38149
0.107747	2.1E-05	0.004325	5.09E-08	5.54E-07	0.342097	0.035394	0.00045	9.63E-06
0.082581	0.295245	0.166792	0.379868	-0.35727	0.027265	0.047142	0.281342	-0.28852
0.355999	0.000752	0.060901	1.06E-05	3.73E-05	0.760918	0.598677	0.001354	0.001004
0.064563	-0.30583	-0.26553	-0.52994	0.525971	-0.30966	-0.0117	-0.12415	0.498269
0.470819	0.000471	0.002552	1.48E-10	2.15E-10	0.000396	0.896129	0.164316	2.51E-09
0.047289	-0.29642	-0.20848	-0.45625	0.074415	-0.14637	0.25449	0.125749	0.33212
0.59754	0.000715	0.018668	7.01E-08	0.405703	0.100581	0.003886	0.158923	0.000136
0.069742	0.097365	-0.00137	0.273494	-0.10938	0.052232	0.005385	0.254833	-0.34111
0.435904	0.276161	0.987794	0.001863	0.220902	0.559754	0.952085	0.003836	8.69E-05
0.080601	-0.14371	-0.0662	-0.08488	-0.24319	0.029668	0.325723	0.373056	-0.06035
0.367689	0.106987	0.459609	0.342716	0.005869	0.740565	0.000186	1.56E-05	0.500311
0.109146	-0.02639	0.107029	-0.05734	-0.20252	0.038251	0.202709	0.226692	-0.07461
0.221893	0.768367	0.231043	0.521988	0.022404	0.669411	0.022277	0.010381	0.404467
-0.16966	0.015443	0.021915	0.034382	-0.48714	0.151235	0.244031	0.230895	-0.33345
0.056527	0.863187	0.806801	0.701165	6.33E-09	0.089648	0.005695	0.009009	0.000128
0.503909	0.057112	0.323424	-0.17409	-0.0234	0.309609	0.467505	0.239058	0.01497
1.55E-09	0.523619	0.000208	0.050286	0.793985	0.000397	3E-08	0.006794	0.867335
0.546	0.462999	0.50572	0.573432	-0.6591	0.748135	0.74804	0.64784	-0.67183
3.16E-11	4.23E-08	1.32E-09	1.84E-12	3.63E-17	5.13E-24	5.23E-24	1.84E-16	5.34E-18
0.453986	0.331847	0.387195	0.341234	-0.53115	0.411725	0.659718	0.454321	-0.45553
8.29E-08	0.000138	6.89E-06	8.64E-05	1.33E-10	1.52E-06	3.32E-17	8.09E-08	7.4E-08
0.289092	0.014825	-0.08857	-0.0625	0.224343	-0.0568	-0.147	-0.05998	0.120366
0.000979	0.868612	0.32207	0.48515	0.011225	0.525881	0.099116	0.502948	0.177674
0.145928	0.052134	-0.08751	-0.25726	0.458962	-0.08705	-0.07129	-0.24443	0.283852
0.101623	0.560491	0.327934	0.003503	5.73E-08	0.330496	0.425733	0.005614	0.001221
0.236764	0.277615	-0.01154	0.115081	0.350155	0.118307	-0.12007	-0.12104	-0.1441
0.007361	0.001578	0.897501	0.197621	5.45E-05	0.185263	0.178738	0.175244	0.106031
0.450382	0.460359	0.295991	0.325798	-0.11027	0.501298	0.365121	0.253001	-0.35112
1.08E-07	5.16E-08	0.000728	0.000186	0.217144	1.94E-09	2.44E-05	0.004107	5.18E-05
-0.21569	0.202924	0.037908	0.510587	-0.29117	0.124992	-0.21983	0.18912	-0.18919
0.014875	0.022132	0.672202	8.64E-10	0.000897	0.161464	0.013015	0.033216	0.033151
-0.35993	-0.27895	-0.28125	-0.23826	0.657441	-0.4633	-0.66327	-0.54709	0.501382
3.23E-05	0.001494	0.00136	0.006988	4.64E-17	4.14E-08	1.96E-17	2.83E-11	1.92E-09
-0.57689	-0.45945	-0.28196	-0.32176	0.183593	-0.54961	-0.4963	-0.46239	0.46096
1.26E-12	5.53E-08	0.00132	0.000225	0.038817	2.2E-11	2.96E-09	4.43E-08	4.94E-08
-0.51621	-0.25804	-0.12872	-0.06747	0.195419	-0.39559	-0.6459	-0.54645	0.217281
5.24E-10	0.003401	0.149212	0.451034	0.027682	4.16E-06	2.42E-16	3.02E-11	0.014135
-0.06922	-0.09512	-0.17595	-0.19031	0.500745	-0.3742	-0.33278	-0.22216	0.404891
0.439367	0.287412	0.04785	0.032107	2.03E-09	1.47E-05	0.000132	0.012063	2.34E-06

SLC35F6	SLC37A2	SLC39A14	SLC40A1	SLC46A3	SLC52A1	SLC6A6	SLC7A7	SLC8B1
brown	brown	brown	brown	brown	brown	brown	brown	brown
0.158063	0.223313	-0.11464	0.303979	0.285623	0.295292	0.404924	0.250621	0.131364
0.075924	0.011613	0.199348	0.000512	0.001134	0.000751	2.34E-06	0.004484	0.140982
0.09145	0.054995	-0.11641	0.181189	0.24506	0.193359	0.316077	0.111991	0.032063
0.306524	0.539153	0.192476	0.041488	0.005489	0.029399	0.000295	0.210007	0.720452
-0.12112	-0.2254	0.244626	-0.1316	-0.43234	-0.24613	-0.31282	-0.24519	-0.25653
0.174938	0.010837	0.005575	0.140271	3.86E-07	0.005281	0.000343	0.005463	0.0036
-0.05366	-0.1644	0.481841	0.050075	-0.01351	-0.07427	-0.06992	-0.04854	-0.17605
0.549073	0.064754	9.73E-09	0.576103	0.880204	0.406653	0.434702	0.587884	0.047724
0.00448	-0.07918	-0.08894	0.000292	0.075764	0.121838	-0.10521	0.141199	-0.17791
0.960135	0.376203	0.320035	0.997399	0.397222	0.172392	0.239099	0.113321	0.045386
0.015056	-0.10898	0.380687	0.123677	0.252712	0.117628	-0.00885	0.16927	-0.17804
0.866578	0.222615	1.01E-05	0.165957	0.004151	0.187816	0.921319	0.05711	0.045218
-0.06957	-0.11633	0.239879	0.003469	0.261366	0.165398	0.149051	0.073564	-0.05266
0.437021	0.192786	0.006601	0.969126	0.002997	0.063121	0.094433	0.411108	0.556554
0.241162	0.147346	0.081945	0.187149	0.280994	0.142393	0.000601	0.253954	0.010973
0.006308	0.098305	0.359729	0.03513	0.001374	0.110273	0.994651	0.003964	0.902554
0.307976	0.271112	0.541568	0.351265	0.179044	0.336809	0.431318	0.376124	0.419925
0.000428	0.002049	4.88E-11	5.14E-05	0.044001	0.000108	4.14E-07	1.31E-05	8.89E-07
0.660177	0.778637	0.247412	0.621354	0.621214	0.602595	0.547786	0.931751	0.75586
3.1E-17	4.56E-27	0.005042	6.47E-15	6.59E-15	6.61E-14	2.64E-11	7.69E-57	9.54E-25
0.46099	0.514911	0.296027	0.465134	0.427814	0.567862	0.364663	0.661843	0.509693
4.92E-08	5.89E-10	0.000727	3.6E-08	5.25E-07	3.35E-12	2.5E-05	2.42E-17	9.35E-10
-0.00951	-0.09147	0.074746	-0.12059	-0.28457	0.023966	0.179492	-0.11026	0.125996
0.915526	0.30644	0.403611	0.176847	0.001185	0.789126	0.043466	0.217205	0.158099
0.101687	0.152228	0.000471	-0.0303	-0.43994	-0.14086	0.004126	-0.11235	0.240645
0.2553	0.087538	0.995805	0.735255	2.28E-07	0.114197	0.963278	0.208561	0.006425
0.287232	0.286322	-0.21931	0.032645	-0.37507	0.067834	-0.09666	0.088374	0.342265
0.00106	0.001101	0.013237	0.715595	1.39E-05	0.448592	0.279685	0.323145	8.19E-05
0.600747	0.715369	-0.01069	0.406197	0.065926	0.326643	0.354364	0.566484	0.791004
8.25E-14	3.43E-21	0.905014	2.16E-06	0.461482	0.000178	4.36E-05	3.87E-12	1.9E-28
0.071106	0.226616	-0.60211	0.05883	0.205253	-0.05695	0.05664	0.105557	0.088206
0.42696	0.010407	7.01E-14	0.511182	0.020617	0.52478	0.527061	0.237561	0.32407
-0.42212	-0.38711	-0.50435	-0.43333	-0.56422	-0.49278	-0.47437	-0.61233	-0.42629
7.69E-07	6.92E-06	1.49E-09	3.6E-07	4.92E-12	3.98E-09	1.76E-08	2.02E-14	5.82E-07
-0.64864	-0.69596	-0.16024	-0.43298	-0.10878	-0.41669	-0.43466	-0.68101	-0.73921
1.64E-16	1.07E-19	0.071922	3.69E-07	0.223472	1.1E-06	3.29E-07	1.26E-18	3.32E-23
-0.57248	-0.5389	-0.41856	-0.47216	-0.17414	-0.35293	-0.31593	-0.6129	-0.54573
2.04E-12	6.33E-11	9.73E-07	2.09E-08	0.050222	4.71E-05	0.000297	1.88E-14	3.24E-11
-0.37386	-0.25995	-0.21085	-0.31725	-0.42342	-0.35954	-0.05567	-0.35392	-0.36623
1.49E-05	0.003163	0.017339	0.000279	7.05E-07	3.3E-05	0.534159	4.47E-05	2.29E-05

SLC9A9	SLCO2B1	SLFN11	SLFN12L	SLFN5	SLN	SLPI	SMCO4	SMIM10
brown	brown	brown	brown	brown	brown	brown	brown	brown
0.473581	0.158124	0.113656	0.249333	0.222698	0.272538	0.000982	0.266245	0.273985
1.87E-08	0.07581	0.203265	0.004701	0.011851	0.001936	0.991254	0.002482	0.001827
0.415179	0.053439	-0.03699	0.095215	0.047312	0.133961	-0.04078	0.188262	0.216804
1.21E-06	0.550709	0.679714	0.286951	0.597354	0.133221	0.648949	0.034038	0.014353
-0.56861	-0.45382	-0.00484	-0.10733	-0.10238	-0.01513	-0.02502	-0.12077	-0.28808
3.09E-12	8.39E-08	0.956909	0.229714	0.252043	0.865953	0.78011	0.176227	0.001022
-0.27639	-0.37814	0.371719	0.184278	0.313027	0.189204	0.10371	-0.02771	0.110764
0.001658	1.17E-05	1.69E-05	0.038082	0.000339	0.033137	0.245916	0.757169	0.215079
0.063734	-0.17289	0.058806	-0.09726	-0.11761	0.109596	0.228616	0.391634	0.263366
0.476545	0.051924	0.511359	0.27667	0.187877	0.219984	0.009731	5.29E-06	0.002775
0.003447	-0.29835	0.483654	0.205415	0.353466	0.30528	0.27158	0.288507	0.425694
0.96932	0.000657	8.41E-09	0.020516	4.57E-05	0.000483	0.002011	0.001004	6.06E-07
0.108952	-0.18078	0.23972	0.129674	0.213347	0.164226	0.18704	0.142018	0.267663
0.222723	0.041953	0.006638	0.14621	0.016026	0.06504	0.035238	0.111222	0.002348
0.202814	0.161316	0.350042	0.182388	0.339158	0.324276	0.178673	0.021611	0.398961
0.022206	0.07001	5.48E-05	0.040138	9.6E-05	0.0002	0.044449	0.809429	3.38E-06
0.016315	0.173021	0.415463	0.381139	0.52017	0.448301	0.425826	0.383955	0.201345
0.855541	0.051743	1.19E-06	9.83E-06	3.66E-10	1.25E-07	6E-07	8.34E-06	0.023213
0.708679	0.72124	0.570271	0.584	0.738075	0.489163	0.554303	0.788895	0.553303
1.16E-20	1.14E-21	2.59E-12	5.73E-13	4.19E-23	5.36E-09	1.37E-11	3.32E-28	1.52E-11
0.490395	0.409923	0.610976	0.576084	0.734647	0.41484	0.306232	0.489835	0.498303
4.85E-09	1.7E-06	2.39E-14	1.38E-12	8.38E-23	1.24E-06	0.000463	5.07E-09	2.5E-09
-0.0622	-0.02507	-0.21028	-0.06535	-0.29515	-0.14303	-0.08068	-0.12631	-0.23855
0.487248	0.779645	0.017648	0.465406	0.000755	0.108673	0.367232	0.157051	0.006916
-0.29035	0.071636	-0.30931	-0.07211	-0.16577	-0.14793	-0.10469	-0.1731	-0.43507
0.000928	0.423514	0.000402	0.420423	0.06252	0.096957	0.241461	0.051641	3.2E-07
-0.14021	0.233924	-0.34044	-0.22798	-0.27932	-0.17142	0.018901	0.13779	-0.21396
0.115886	0.008122	8.99E-05	0.009941	0.001472	0.053979	0.832953	0.122386	0.015716
0.310869	0.668542	0.045151	0.237749	0.275381	0.125473	0.224462	0.43332	0.0658
0.000375	8.84E-18	0.614225	0.007113	0.001727	0.159845	0.01118	3.61E-07	0.462345
0.488076	0.407379	-0.26245	-0.14017	-0.16938	-0.3001	-0.29946	0.001844	-0.00751
5.86E-09	2E-06	0.002875	0.115999	0.056949	0.000608	0.000626	0.983582	0.933244
-0.41937	-0.24814	-0.6355	-0.55326	-0.72638	-0.55429	-0.5506	-0.58644	-0.67213
9.23E-07	0.00491	1.01E-15	1.53E-11	4.28E-22	1.37E-11	2E-11	4.35E-13	5.09E-18
-0.32761	-0.5367	-0.18573	-0.30554	-0.37733	-0.31903	-0.43969	-0.63993	-0.25505
0.00017	7.82E-11	0.036567	0.000477	1.23E-05	0.000256	2.32E-07	5.53E-16	0.003805
-0.142	-0.23788	-0.48991	-0.43165	-0.58894	-0.39996	-0.42966	-0.60038	-0.37973
0.111275	0.00708	5.04E-09	4.05E-07	3.27E-13	3.18E-06	4.63E-07	8.61E-14	1.07E-05
-0.42986	-0.41107	-0.40104	-0.19082	-0.44495	-0.20524	-0.17474	-0.08863	-0.56429
4.57E-07	1.58E-06	2.98E-06	0.031635	1.6E-07	0.020627	0.049424	0.321764	4.88E-12

SNAI3	SNRK	SNX17	SNX2	SNX20	SNX32	SNX9	SOAT1	SOBP
brown	brown	brown	brown	brown	brown	brown	brown	brown
0.18172	-0.34308	0.190615	0.377328	0.141201	-0.0131	0.322478	0.015333	-0.04917
0.040885	7.86E-05	0.031824	1.23E-05	0.113316	0.883801	0.000218	0.864151	0.58299
0.131375	-0.31167	0.176363	0.210485	-0.07093	0.097402	0.245155	-0.14271	0.01474
0.140946	0.000361	0.047319	0.017536	0.428136	0.275973	0.00547	0.109478	0.869359
-0.41251	0.475513	-0.22173	-0.15481	-0.07311	-0.07058	-0.48751	0.187806	0.003711
1.44E-06	1.61E-08	0.012232	0.082235	0.413996	0.430403	6.14E-09	0.034481	0.966973
-0.57955	0.312065	-0.32599	0.246246	0.161424	-0.36135	-0.1223	0.520846	-0.28348
9.4E-13	0.000355	0.000184	0.005259	0.069819	2.99E-05	0.170754	3.45E-10	0.001239
0.051426	-0.22878	0.402833	0.238525	0.056768	-0.08872	-0.00023	-0.01816	-0.2371
0.565835	0.009679	2.66E-06	0.006922	0.526128	0.321229	0.997992	0.839414	0.007276
-0.42167	-0.01435	0.010921	0.552706	0.273523	-0.47525	0.117388	0.513977	-0.49575
7.93E-07	0.872782	0.903014	1.61E-11	0.001861	1.64E-08	0.188722	6.4E-10	3.1E-09
-0.2634	-0.04548	0.029929	0.219392	0.084261	-0.2403	0.128563	0.232246	-0.36016
0.002772	0.61166	0.738366	0.013203	0.346263	0.006503	0.149728	0.008603	3.19E-05
-0.12093	-0.29956	0.024358	0.389591	0.263112	-0.33739	0.177898	0.251987	-0.37753
0.175649	0.000623	0.785754	5.98E-06	0.002802	0.000105	0.045394	0.004264	1.21E-05
-0.08421	-0.0268	0.146645	0.213774	0.497775	-0.62638	0.251864	0.650997	-0.53871
0.346532	0.764869	0.099934	0.015811	2.61E-09	3.38E-15	0.004283	1.18E-16	6.44E-11
0.400436	-0.58469	0.467851	0.676709	0.860029	-0.72393	0.628393	0.747678	-0.73757
3.09E-06	5.3E-13	2.92E-08	2.49E-18	2.45E-38	6.86E-22	2.6E-15	5.65E-24	4.64E-23
0.09247	-0.36502	0.247467	0.699138	0.695661	-0.61184	0.461718	0.694708	-0.51706
0.301136	2.45E-05	0.005032	6.19E-20	1.12E-19	2.14E-14	4.66E-08	1.32E-19	4.85E-10
0.025891	0.045958	0.09569	-0.4819	-0.27757	0.176974	0.047738	-0.22585	0.273387
0.772624	0.607902	0.284543	9.68E-09	0.00158	0.046545	0.594058	0.010675	0.001871
0.118401	0.218361	-0.14948	-0.49954	-0.09816	0.161744	-0.33798	-0.076	0.241552
0.184909	0.013651	0.093471	2.25E-09	0.272262	0.069259	0.000102	0.395771	0.006221
0.480061	-0.17179	0.323268	-0.43067	0.004658	0.127407	-0.2103	-0.15923	0.14205
1.12E-08	0.053461	0.00021	4.33E-07	0.958552	0.153457	0.017637	0.073753	0.11114
0.562582	-0.36529	0.328457	-0.01815	0.447748	-0.30025	0.208735	0.301912	-0.24012
5.84E-12	2.41E-05	0.000163	0.83953	1.31E-07	0.000604	0.018519	0.000561	0.006546
0.475926	-0.28152	0.135209	-0.0873	-0.20003	0.344777	0.178826	-0.44733	0.2872
1.56E-08	0.001344	0.129613	0.329075	0.024149	7.2E-05	0.044263	1.34E-07	0.001061
0.028849	0.458379	-0.2064	-0.72799	-0.67396	0.79379	-0.54755	-0.76112	0.798919
0.747477	5.99E-08	0.019904	3.13E-22	3.83E-18	9.04E-29	2.71E-11	2.93E-25	2.22E-29
-0.4236	0.457338	-0.45344	-0.17234	-0.548	0.487491	-0.24484	-0.45571	0.492659
6.97E-07	6.47E-08	8.63E-08	0.052687	2.59E-11	6.15E-09	0.005532	7.3E-08	4.02E-09
-0.0127	0.219259	-0.19098	-0.41057	-0.6324	0.731415	-0.19539	-0.81276	0.6429
0.887301	0.01326	0.031493	1.63E-06	1.53E-15	1.6E-22	0.027706	4.09E-31	3.67E-16
-0.1319	0.563075	-0.19805	-0.4153	-0.35215	0.411743	-0.59375	-0.32466	0.299617
0.139347	5.55E-12	0.025617	1.2E-06	4.9E-05	1.51E-06	1.88E-13	0.000196	0.000621

SOCS3	SOD2	SP100	SP140	SP140L	SPATC1	SPATS2	SPI1	SPIN2B
brown	brown	brown	brown	brown	brown	brown	brown	brown
-0.02316	0.116672	0.260386	0.367495	0.130976	0.14036	-0.24375	0.1625	0.00183
0.79604	0.191452	0.003111	2.14E-05	0.142169	0.115502	0.005753	0.067951	0.983712
-0.1408	0.0315	0.100665	0.18514	0.000193	0.061769	-0.16644	0.021247	0.156227
0.11435	0.725168	0.260131	0.037175	0.998282	0.490269	0.061461	0.812575	0.079436
0.008533	-0.0981	-0.23795	-0.20638	0.057486	-0.22013	0.476511	-0.36459	0.134099
0.924149	0.272529	0.007064	0.019917	0.520897	0.012892	1.49E-08	2.51E-05	0.132821
0.218244	0.20469	0.185233	0.045847	0.408073	-0.10307	0.292173	-0.4138	-0.02637
0.013703	0.020975	0.037077	0.60877	1.91E-06	0.248841	0.000859	1.33E-06	0.768563
-0.0652	-0.05352	0.10051	-0.00629	0.051702	-0.21391	0.137854	-0.04022	0.506381
0.466425	0.550101	0.260868	0.944007	0.563749	0.015741	0.12221	0.653434	1.25E-09
0.215978	0.266469	0.399732	0.168388	0.490095	-0.13241	0.193887	-0.30316	0.184538
0.014739	0.00246	3.23E-06	0.058436	4.97E-09	0.137827	0.02895	0.000531	0.037806
0.155893	0.081405	0.276309	0.0866	0.296056	-0.00308	0.080152	-0.26574	0.123498
0.080089	0.362915	0.001664	0.332992	0.000726	0.972615	0.37037	0.002531	0.166577
0.110648	0.299215	0.399333	0.189948	0.320389	0.012157	-0.30965	0.036202	-0.0867
0.215561	0.000632	3.31E-06	0.03244	0.00024	0.892093	0.000396	0.686156	0.332417
0.63848	0.432849	0.510043	0.331547	0.605925	0.370673	-0.05013	0.276211	-0.47299
6.74E-16	3.73E-07	9.07E-10	0.00014	4.43E-14	1.79E-05	0.575714	0.00167	1.96E-08
0.592688	0.630773	0.88341	0.839142	0.782656	0.652486	-0.66936	0.775825	-0.43735
2.12E-13	1.9E-15	5.7E-43	7.39E-35	1.66E-27	9.5E-17	7.8E-18	9.12E-27	2.73E-07
0.436842	0.540166	0.832829	0.789314	0.763514	0.465754	-0.47554	0.455283	-0.33723
2.83E-07	5.59E-11	6.66E-34	2.97E-28	1.69E-25	3.43E-08	1.61E-08	7.53E-08	0.000106
0.048879	-0.20382	-0.11417	-0.07879	-0.11633	0.081784	0.039341	0.075417	-0.02195
0.585261	0.021538	0.201198	0.378588	0.192754	0.360675	0.660564	0.399392	0.806525
0.001183	-0.11743	-0.29598	-0.05279	-0.13631	0.145973	-0.02416	0.173215	-0.18095
0.989467	0.188563	0.000729	0.555533	0.126494	0.101515	0.787443	0.051477	0.041761
-0.01075	-0.08416	-0.22898	-0.03623	-0.21193	0.126822	-0.1858	0.521989	0.041617
0.904501	0.346857	0.009611	0.685931	0.016757	0.155368	0.036489	3.1E-10	0.642244
0.325439	0.285536	0.31165	0.494224	0.302355	0.564031	-0.60283	0.830209	-0.41427
0.000189	0.001138	0.000362	3.52E-09	0.00055	5.02E-12	6.43E-14	1.61E-33	1.29E-06
-0.36409	-0.26811	-0.17413	0.048813	-0.3579	0.10704	-0.45402	0.261341	0.01345
2.58E-05	0.002307	0.050243	0.585764	3.61E-05	0.230997	8.27E-08	0.002999	0.880704
-0.53151	-0.62707	-0.81847	-0.62587	-0.79007	-0.35578	0.292845	-0.28094	0.222794
1.28E-10	3.09E-15	7.15E-32	3.61E-15	2.44E-28	4.05E-05	0.000834	0.001377	0.011814
-0.4279	-0.37977	-0.48826	-0.58793	-0.5234	-0.4956	0.538592	-0.74036	0.257974
5.22E-07	1.06E-05	5.78E-09	3.67E-13	2.73E-10	3.14E-09	6.51E-11	2.62E-23	0.003409
-0.52158	-0.48449	-0.60326	-0.59224	-0.76326	-0.38138	0.307015	-0.40509	0.237643
3.22E-10	7.86E-09	6.1E-14	2.24E-13	1.79E-25	9.7E-06	0.000447	2.31E-06	0.007139
-0.29998	-0.56456	-0.53115	-0.33234	-0.37228	-0.29987	0.537591	-0.33642	0.371483
0.000612	4.75E-12	1.33E-10	0.000135	1.63E-05	0.000615	7.18E-11	0.00011	1.71E-05

SPIN3	SPINT1	SPN	SPNS3	SPTLC3	SQLE	SQSTM1	SRA1	SRGN
brown	brown	brown	brown	brown	brown	brown	brown	brown
-0.0609	0.196097	0.198654	0.211857	0.23946	-0.17202	0.310034	0.19363	0.198755
0.496389	0.027137	0.025159	0.016797	0.006699	0.053129	0.000389	0.029168	0.025084
-0.01118	0.03605	0.034926	0.141634	0.112842	-0.12558	0.211241	0.148323	0.104782
0.900734	0.687407	0.696666	0.112203	0.206541	0.159486	0.017126	0.096071	0.241041
0.227498	-0.04823	-0.143	-0.27766	-0.20506	0.403891	-0.49442	-0.06181	-0.32533
0.010104	0.590228	0.108753	0.001575	0.020737	2.49E-06	3.47E-09	0.489965	0.00019
0.259992	-0.00408	-0.02536	-0.37765	0.129603	0.372749	-0.35238	-0.0451	-0.07406
0.003158	0.963666	0.777136	1.2E-05	0.146433	1.59E-05	4.84E-05	0.614654	0.407944
-0.26684	0.10108	-0.10349	0.220177	0.039906	-0.05388	-0.09449	0.49769	0.204467
0.002425	0.258162	0.246912	0.01287	0.655995	0.547423	0.290644	2.63E-09	0.021118
-0.00986	0.118157	-0.01423	-0.15644	0.293775	0.226417	-0.22487	0.275302	0.24001
0.912354	0.185822	0.873816	0.079027	0.000802	0.010476	0.011031	0.001733	0.00657
0.068185	-0.08079	-0.12885	-0.13375	0.116272	0.109217	-0.15605	0.24022	0.14426
0.446245	0.366541	0.148799	0.133853	0.19299	0.221594	0.079776	0.006522	0.105635
0.070207	0.076653	0.129621	0.10617	0.319961	-0.04126	0.239565	0.21439	0.2002
0.432841	0.39169	0.146377	0.23483	0.000245	0.645112	0.006674	0.015504	0.024025
-0.3541	0.469939	0.227841	-0.01459	0.241312	-0.1521	0.20784	0.24065	0.371973
4.42E-05	2.49E-08	0.009988	0.870639	0.006275	0.087817	0.01904	0.006423	1.66E-05
-0.60385	0.710062	0.658388	0.423535	0.700851	-0.51692	0.608052	0.524221	0.785088
5.69E-14	9.02E-21	4.04E-17	7E-07	4.6E-20	4.92E-10	3.42E-14	2.53E-10	8.92E-28
-0.26262	0.461036	0.584718	0.270336	0.623496	-0.26984	0.417401	0.390002	0.515603
0.002855	4.91E-08	5.28E-13	0.002113	4.91E-15	0.002155	1.05E-06	5.83E-06	5.53E-10
-0.00575	0.009587	-0.04217	0.021046	-0.23069	-0.09301	0.204467	-0.02592	-0.28538
0.948832	0.914811	0.637803	0.814321	0.009071	0.29833	0.021119	0.77237	0.001145
0.081972	0.043538	0.077629	-0.02109	-0.22566	-0.05593	0.035827	-0.14157	-0.3522
0.359567	0.626946	0.385672	0.813942	0.010743	0.532242	0.689243	0.112365	4.89E-05
-0.44171	0.171255	0.136823	0.390548	-0.17526	-0.44569	0.292798	0.244561	-0.075
2.01E-07	0.054216	0.125056	5.64E-06	0.048748	1.51E-07	0.000836	0.005588	0.402005
-0.51518	0.513561	0.519343	0.413947	0.252468	-0.55601	0.605487	0.30748	0.273176
5.75E-10	6.64E-10	3.95E-10	1.31E-06	0.004189	1.15E-11	4.67E-14	0.000437	0.001887
-0.02137	-0.143	0.144959	0.195187	-0.12358	-0.19656	0.230451	-0.10753	-0.07653
0.811547	0.108738	0.103939	0.027871	0.166292	0.026765	0.009146	0.228837	0.392474
0.420839	-0.58013	-0.35405	-0.15221	-0.69669	0.295925	-0.3914	-0.4203	-0.63958
8.37E-07	8.82E-13	4.44E-05	0.08758	9.43E-20	0.00073	5.36E-06	8.68E-07	5.8E-16
0.559688	-0.62714	-0.47942	-0.41138	-0.34652	0.499356	-0.55403	-0.58769	-0.36786
7.89E-12	3.06E-15	1.18E-08	1.55E-06	6.58E-05	2.29E-09	1.41E-11	3.77E-13	2.09E-05
0.394023	-0.57681	-0.46157	-0.13833	-0.44857	0.223851	-0.27164	-0.47316	-0.34418
4.58E-06	1.27E-12	4.71E-08	0.120922	1.23E-07	0.011409	0.002007	1.94E-08	7.43E-05
0.282912	-0.13441	-0.30809	-0.23383	-0.4887	0.404779	-0.51842	-0.18447	-0.40186
0.001269	0.131904	0.000425	0.008149	5.57E-09	2.36E-06	4.29E-10	0.037877	2.83E-06

SRSF12	SS18L1	SSH2	ST14	ST3GAL1	ST3GAL3	ST6GAL1	ST6GAL2	ST6GALNA
brown	brown	brown	brown	brown	brown	brown	brown	brown
-0.31277	-0.03539	0.131812	-0.03346	0.280807	-0.13666	0.169931	-0.36423	0.194536
0.000343	0.692843	0.139618	0.708807	0.001385	0.125498	0.056133	2.56E-05	0.028407
-0.1967	0.097109	0.083759	-0.16692	0.156015	-0.0334	0.039743	-0.28457	0.153323
0.02666	0.277431	0.349155	0.060698	0.07985	0.709315	0.657315	0.001185	0.085256
0.507149	0.295558	-0.28969	0.097834	-0.45077	0.125832	-0.46144	0.366829	-0.38585
1.17E-09	0.000742	0.000955	0.273841	1.05E-07	0.158644	4.76E-08	2.22E-05	7.46E-06
0.299246	0.283579	-0.24358	0.083531	-0.37314	-0.27923	-0.41066	0.050493	-0.31997
0.000632	0.001235	0.005787	0.350471	1.56E-05	0.001477	1.62E-06	0.572916	0.000245
0.024086	0.041261	-0.37603	0.038879	-0.29805	0.098203	-0.23194	-0.22347	-0.14702
0.788091	0.645094	1.32E-05	0.66431	0.000666	0.272027	0.008694	0.011555	0.099059
0.148017	0.198757	-0.33626	0.093737	-0.35295	-0.33199	-0.35734	-0.24229	-0.28748
0.096766	0.025082	0.000111	0.294533	4.7E-05	0.000137	3.72E-05	0.006061	0.001049
-0.02505	0.090405	-0.16026	-0.03205	-0.18254	-0.13076	-0.22313	-0.22481	-0.17236
0.779837	0.312107	0.071893	0.720588	0.039973	0.142839	0.011685	0.011052	0.052653
-0.2713	-0.15613	-0.01806	0.083069	-0.14047	-0.40684	0.13197	-0.28038	0.416631
0.002034	0.079629	0.840287	0.353153	0.115218	2.07E-06	0.139142	0.001409	1.1E-06
-0.23198	-0.3769	0.171919	0.656579	0.353466	-0.3719	-0.09467	-0.35684	0.04965
0.008681	1.26E-05	0.053275	5.26E-17	4.57E-05	1.67E-05	0.289739	3.82E-05	0.579347
-0.72372	-0.63218	0.385232	0.735932	0.523702	-0.70191	0.469711	-0.71035	0.456802
7.13E-22	1.57E-15	7.74E-06	6.47E-23	2.65E-10	3.83E-20	2.53E-08	8.56E-21	6.73E-08
-0.502	-0.34753	0.100857	0.41543	0.315266	-0.58578	0.349396	-0.53432	0.318493
1.82E-09	6.25E-05	0.259217	1.19E-06	0.000306	4.69E-13	5.67E-05	9.82E-11	0.000263
-0.09241	-0.16471	0.127846	-0.04113	0.229781	0.191996	0.00015	-0.03918	0.24723
0.301431	0.064246	0.152032	0.646183	0.009356	0.030584	0.998661	0.661828	0.005075
0.010826	-0.11559	0.17356	0.202446	0.0975	0.160417	0.018629	0.266261	0.166922
0.903846	0.195646	0.051007	0.022455	0.275492	0.071605	0.835322	0.00248	0.060697
-0.19409	-0.34795	-0.08874	0.339701	0.06628	0.250249	0.077629	0.076737	0.266381
0.02878	6.11E-05	0.321152	9.34E-05	0.459079	0.004546	0.38567	0.391172	0.002468
-0.60368	-0.64339	0.398342	0.654469	0.493409	-0.29312	0.442331	-0.28975	0.567031
5.8E-14	3.43E-16	3.52E-06	7.14E-17	3.77E-09	0.000825	1.92E-07	0.000953	3.66E-12
-0.28479	-0.07889	0.370613	-0.26681	0.111299	-0.00695	0.532524	0.020029	0.285361
0.001174	0.377955	1.79E-05	0.002428	0.212856	0.938172	1.16E-10	0.823142	0.001146
0.42344	0.276273	-0.09106	-0.50431	-0.27676	0.668567	0.009785	0.716014	-0.20322
7.04E-07	0.001666	0.308606	1.49E-09	0.001634	8.8E-18	0.913055	3.04E-21	0.021934
0.599593	0.623687	-0.2588	-0.75412	-0.36431	0.371718	-0.25083	0.47786	-0.51171
9.46E-14	4.79E-15	0.003304	1.4E-24	2.55E-05	1.69E-05	0.004449	1.34E-08	7.83E-10
0.334209	0.344899	-0.03967	-0.72874	-0.0676	0.582302	0.036514	0.410685	-0.23369
0.000123	7.16E-05	0.657908	2.7E-22	0.450133	6.93E-13	0.683601	1.62E-06	0.008187
0.45595	0.362733	0.014013	-0.09336	-0.2667	0.350944	-0.35497	0.326404	-0.36268
7.17E-08	2.78E-05	0.875747	0.296482	0.002438	5.23E-05	4.22E-05	0.00018	2.79E-05

ST8SIA4	ST8SIA5	STAB1	STAC3	STAT5A	STAT6	STOM	STRIP1	STX11
brown	brown	brown	brown	brown	brown	brown	brown	brown
0.044432	-0.07661	0.095022	0.199655	0.255243	0.365407	0.394815	-0.28343	0.27486
0.61988	0.391929	0.287934	0.02442	0.003778	2.4E-05	4.36E-06	0.001242	0.001764
-0.08423	-0.01963	-0.00524	0.048225	0.116319	0.27335	0.389218	-0.24344	0.141264
0.346464	0.826585	0.953386	0.590292	0.19281	0.001874	6.11E-06	0.005816	0.113152
0.084989	0.256205	-0.14201	-0.00457	-0.33502	-0.62571	-0.63992	0.371813	-0.13993
0.342099	0.003644	0.111238	0.959314	0.000118	3.69E-15	5.53E-16	1.68E-05	0.116644
0.465811	0.109484	-0.14001	0.048805	-0.20192	-0.48321	-0.25847	0.07336	0.080976
3.42E-08	0.220459	0.116409	0.585831	0.022815	8.71E-09	0.003346	0.41241	0.365454
-0.01931	-0.07142	-0.177	0.08674	-0.02698	-0.03228	0.150532	-0.41814	-0.00098
0.829356	0.424943	0.046507	0.332207	0.763327	0.71866	0.091168	1E-06	0.991235
0.5165	-0.04619	-0.16948	0.129292	-0.0937	-0.23584	0.133013	-0.37445	0.194542
5.11E-10	0.606119	0.056804	0.147413	0.294715	0.007602	0.136015	1.44E-05	0.028402
0.21332	-0.05634	-0.12518	0.086771	0.001741	-0.05158	0.173003	-0.17781	0.09567
0.01604	0.529238	0.160828	0.332037	0.984505	0.56464	0.051768	0.045499	0.284647
0.133983	-0.43666	0.093963	0.052306	0.128679	0.129784	0.354821	-0.30901	0.10473
0.133159	2.86E-07	0.293367	0.5592	0.149358	0.145865	4.26E-05	0.000408	0.241276
0.556076	-0.11237	0.575543	0.47691	0.361289	0.179747	0.122573	-0.1645	0.433703
1.15E-11	0.208446	1.46E-12	1.44E-08	3E-05	0.043164	0.169799	0.064583	3.51E-07
0.595539	-0.51229	0.750155	0.819057	0.819932	0.625678	0.664839	-0.66904	0.728624
1.52E-13	7.44E-10	3.32E-24	5.95E-32	4.52E-32	3.7E-15	1.55E-17	8.18E-18	2.76E-22
0.560097	-0.29821	0.411775	0.598118	0.581926	0.377181	0.47248	-0.46405	0.608376
7.57E-12	0.000661	1.51E-06	1.13E-13	7.22E-13	1.24E-05	2.04E-08	3.91E-08	3.28E-14
-0.20863	0.221478	0.099753	-0.04767	0.044499	-0.04483	-0.11635	0.318975	-0.00254
0.01858	0.012335	0.264497	0.59457	0.619357	0.616757	0.192685	0.000257	0.97742
-0.26801	0.090105	0.266288	0.136285	0.12544	-0.15618	-0.51198	0.521965	-0.11196
0.002316	0.313721	0.002477	0.12656	0.159957	0.079536	7.64E-10	3.11E-10	0.210142
-0.36257	-0.05242	0.319181	0.228593	0.286692	0.063409	-0.25618	0.017399	-0.07057
2.8E-05	0.558333	0.000255	0.009739	0.001084	0.478805	0.003648	0.846054	0.430468
0.03262	-0.30753	0.772393	0.650669	0.748431	0.451932	0.134925	-0.16395	0.352502
0.715807	0.000436	2.1E-26	1.23E-16	4.81E-24	9.64E-08	0.130428	0.065502	4.81E-05
-0.38704	0.024103	0.033366	-0.04379	0.151792	0.330581	0.280204	-0.09899	-0.17473
6.96E-06	0.787947	0.709594	0.624937	0.088459	0.000147	0.001419	0.2682	0.049433
-0.66979	0.457046	-0.41009	-0.53302	-0.50291	-0.38504	-0.58241	0.668032	-0.57446
7.3E-18	6.61E-08	1.68E-06	1.11E-10	1.69E-09	7.83E-06	6.85E-13	9.55E-18	1.65E-12
-0.1545	0.364565	-0.75947	-0.77475	-0.77926	-0.36717	-0.18666	0.303041	-0.42315
0.082853	2.51E-05	4.26E-25	1.18E-26	3.9E-27	2.17E-05	0.035617	0.000534	7.18E-07
-0.51834	0.244732	-0.5594	-0.73496	-0.56603	-0.0555	-0.05214	0.360625	-0.48971
4.32E-10	0.005554	8.13E-12	7.87E-23	4.07E-12	0.535408	0.560413	3.12E-05	5.13E-09
-0.34425	0.322932	-0.22885	-0.05897	-0.32141	-0.37146	-0.68358	0.450925	-0.25545
7.4E-05	0.000213	0.009654	0.510199	0.000229	1.71E-05	8.32E-19	1.04E-07	0.003748

STX4	STXBP2	SUDS3	SULF1	SULT1C2	SUMF1	SUSD3	SUSD6	SWAP70
brown	brown	brown	brown	brown	brown	brown	brown	brown
0.257731	0.09928	-0.18674	0.122624	0.48432	0.277043	0.081324	0.01754	0.16128
0.003441	0.266779	0.035536	0.16962	7.97E-09	0.001615	0.363394	0.844824	0.070073
0.164421	-0.05036	-0.13907	0.029277	0.434086	0.237161	-0.04765	-0.11398	0.086799
0.064718	0.573923	0.11893	0.743865	3.42E-07	0.00726	0.594734	0.201988	0.331878
-0.21695	-0.22478	0.312061	-0.25432	-0.3507	-0.14687	-0.15752	-0.00532	-0.27153
0.014287	0.011063	0.000355	0.00391	5.29E-05	0.099402	0.076953	0.95267	0.002016
-0.27855	-0.29187	0.340559	-0.08836	-0.27232	0.190866	-0.14249	0.142111	0.213656
0.001519	0.00087	8.94E-05	0.323202	0.001953	0.031596	0.110029	0.110987	0.01587
-0.02006	-0.161	-0.25069	-0.10771	0.224303	0.423375	0.117569	-0.3026	-0.13322
0.822875	0.070569	0.004472	0.228089	0.011239	7.08E-07	0.188036	0.000544	0.135393
-0.2149	-0.31911	0.059142	0.007046	0.031576	0.575037	-0.03741	0.009981	0.308477
0.015255	0.000255	0.50894	0.937331	0.724532	1.54E-12	0.676276	0.911321	0.000418
-0.07063	-0.27268	0.05165	-0.00243	-0.02666	0.377625	-0.02345	0.032648	0.286302
0.430055	0.001925	0.564141	0.978365	0.766072	1.2E-05	0.793563	0.715571	0.001102
-0.14151	-0.09522	-0.08037	0.310459	0.166729	0.326275	-0.00655	0.060443	0.391705
0.112517	0.286942	0.369052	0.000382	0.060999	0.000181	0.941752	0.49965	5.26E-06
0.347015	0.435034	-0.15704	0.420362	-0.13369	0.300964	0.043845	0.706089	0.409896
6.42E-05	3.2E-07	0.077873	8.64E-07	0.134005	0.000586	0.624521	1.84E-20	1.7E-06
0.663216	0.727642	-0.50956	0.561598	0.512561	0.685643	0.401385	0.677737	0.642173
1.97E-17	3.35E-22	9.46E-10	6.47E-12	7.26E-10	5.95E-19	2.91E-06	2.12E-18	4.06E-16
0.358316	0.421549	-0.23782	0.417252	0.3599	0.465014	0.314835	0.505711	0.596135
3.53E-05	7.99E-07	0.007096	1.06E-06	3.24E-05	3.63E-08	0.000312	1.32E-09	1.42E-13
0.091804	0.302728	-0.0075	0.031177	-0.32388	-0.31967	0.162434	0.114329	-0.15258
0.304649	0.000541	0.93333	0.727875	0.000204	0.000249	0.068064	0.200587	0.086796
0.264115	0.347772	0.10145	-0.05273	-0.23018	-0.47259	-0.03751	0.222164	-0.30156
0.002696	6.17E-05	0.256411	0.556051	0.009229	2.02E-08	0.675469	0.012061	0.00057
0.443378	0.490325	-0.38447	-0.06575	0.06144	-0.27557	0.127006	0.037242	-0.37781
1.78E-07	4.87E-09	8.1E-06	0.462684	0.492589	0.001714	0.154765	0.677635	1.19E-05
0.773731	0.871493	-0.45782	0.35631	0.265801	0.052027	0.254674	0.554411	0.145648
1.52E-26	1.7E-40	6.24E-08	3.93E-05	0.002525	0.561297	0.003859	1.36E-11	0.102287
0.237531	0.137924	-0.19432	-0.13222	0.407085	-0.13387	0.126424	-0.31434	-0.02672
0.007167	0.122019	0.028591	0.138389	2.04E-06	0.133503	0.15668	0.000319	0.765589
-0.29007	-0.27391	0.244385	-0.47733	-0.27605	-0.7641	-0.09391	-0.53313	-0.66799
0.000939	0.001832	0.005624	1.39E-08	0.001681	1.48E-25	0.293665	1.1E-10	9.61E-18
-0.77631	-0.79657	0.511921	-0.39579	-0.30438	-0.3421	-0.27087	-0.56854	-0.18706
8.09E-27	4.25E-29	7.68E-10	4.11E-06	0.000503	8.26E-05	0.002069	3.11E-12	0.035223
-0.50754	-0.50592	0.255416	-0.29053	-0.16494	-0.50299	-0.13	-0.56969	-0.3715
1.13E-09	1.3E-09	0.003753	0.000921	0.063859	1.67E-09	0.145205	2.75E-12	1.71E-05
-0.04087	-0.1401	0.293813	-0.44757	-0.2191	-0.24593	-0.12657	-0.25895	-0.67554
0.648238	0.116193	0.0008	1.32E-07	0.013329	0.00532	0.156201	0.003286	2.99E-18

SYK	SYNGR2	SYTL3	TAB2	TACSTD2	TAGAP	TAP1	TAPBP	TAPBPL
brown	brown	brown	brown	brown	brown	brown	brown	brown
0.238074	0.365615	0.067017	0.111266	0.428135	0.289706	0.154143	0.200449	0.246394
0.007032	2.37E-05	0.454088	0.212991	5.14E-07	0.000954	0.083579	0.023846	0.005231
0.083132	0.298789	-0.07832	-0.00325	0.426309	0.076526	0.037251	0.047387	0.163765
0.352784	0.000644	0.381429	0.971044	5.81E-07	0.392477	0.677559	0.596778	0.065807
-0.39654	-0.60762	-0.08018	0.087515	-0.25186	-0.37442	0.13672	-0.34331	-0.22887
3.93E-06	3.6E-14	0.370176	0.327892	0.004285	1.45E-05	0.125342	7.77E-05	0.009648
-0.15417	-0.55423	0.233693	0.394854	0.015091	-0.12789	0.367795	-0.25551	-0.04577
0.083534	1.38E-11	0.008187	4.35E-06	0.866272	0.151895	2.1E-05	0.00374	0.609396
0.028017	0.144462	0.049085	0.137759	0.287829	-0.00335	0.115922	-0.24417	0.384418
0.754527	0.105143	0.583676	0.12247	0.001033	0.970147	0.194343	0.005667	8.12E-06
0.042089	-0.23245	0.328476	0.501968	0.339289	0.034249	0.426987	-0.24731	0.313849
0.638475	0.008544	0.000163	1.83E-09	9.53E-05	0.702267	5.55E-07	0.00506	0.000327
0.023669	-0.12533	0.2252	0.255716	0.264347	0.016266	0.329228	-0.15731	0.222631
0.791673	0.160341	0.01091	0.003712	0.002672	0.85597	0.000157	0.077344	0.011877
0.272174	0.105528	0.42091	0.317269	0.144146	0.222693	0.080008	0.167126	0.217565
0.001964	0.237689	8.33E-07	0.000279	0.105912	0.011853	0.371231	0.060378	0.014006
0.296496	0.036359	0.499372	0.241978	0.234711	0.033222	0.529084	0.440056	0.186065
0.000712	0.684866	2.28E-09	0.006128	0.007905	0.710793	1.61E-10	2.26E-07	0.03622
0.90552	0.747427	0.801899	0.502684	0.626988	0.688688	0.612185	0.729173	0.736741
2.26E-48	5.96E-24	9.67E-30	1.72E-09	3.12E-15	3.61E-19	2.05E-14	2.48E-22	5.49E-23
0.613905	0.409519	0.684232	0.544177	0.428079	0.624918	0.770779	0.598067	0.601107
1.66E-14	1.75E-06	7.49E-19	3.78E-11	5.16E-07	4.09E-15	3.09E-26	1.13E-13	7.9E-14
-0.04993	-0.02377	-0.07612	-0.13887	-0.13297	-0.13686	0.030929	0.112241	-0.19497
0.577224	0.790842	0.395026	0.119467	0.136132	0.124952	0.729955	0.208985	0.028048
-0.13539	-0.12455	-0.0658	-0.26199	-0.32967	-0.17271	-0.07407	0.1867	-0.28031
0.129094	0.162974	0.462318	0.002926	0.000154	0.052176	0.407872	0.035578	0.001413
0.038156	0.362668	-0.03526	-0.35684	-0.12514	-0.04824	-0.02253	0.308559	0.071279
0.67018	2.79E-05	0.693879	3.82E-05	0.160981	0.590216	0.801527	0.000416	0.425831
0.549881	0.633035	0.433819	-0.01532	0.189346	0.354771	0.27338	0.752033	0.320117
2.14E-11	1.4E-15	3.49E-07	0.864253	0.033003	4.27E-05	0.001872	2.21E-24	0.000244
0.208233	0.542261	-0.27669	-0.27742	0.137184	0.231312	-0.34328	0.030676	-0.0367
0.01881	4.56E-11	0.001638	0.00159	0.124054	0.008882	7.78E-05	0.732078	0.682075
-0.53982	-0.31352	-0.73896	-0.50892	-0.60507	-0.35008	-0.59588	-0.45124	-0.62792
5.79E-11	0.000332	3.5E-23	1E-09	4.91E-14	5.47E-05	1.47E-13	1.01E-07	2.76E-15
-0.5897	-0.57552	-0.60674	-0.20056	-0.37554	-0.34974	-0.46233	-0.66315	-0.52487
3E-13	1.47E-12	4.01E-14	0.023771	1.36E-05	5.57E-05	4.45E-08	1.99E-17	2.38E-10
-0.44875	-0.19824	-0.7054	-0.45403	-0.40895	-0.26291	-0.6877	-0.43108	-0.48321
1.21E-07	0.025471	2.07E-20	8.26E-08	1.81E-06	0.002824	4.25E-19	4.21E-07	8.71E-09
-0.44999	-0.44325	-0.38181	-0.24884	-0.24653	-0.36475	-0.31307	-0.50798	-0.38813
1.11E-07	1.8E-07	9.46E-06	0.004786	0.005205	2.49E-05	0.000339	1.09E-09	6.52E-06

TBC1D1	TBC1D10C	TBC1D2B	TBXAS1	TC2N	TCAF1	TCIRG1	TCN2	TDRP
brown	brown	brown	brown	brown	brown	brown	brown	brown
-0.03111	0.186513	0.109265	0.199309	0.265434	-0.29192	-0.1133	0.050492	-0.32613
0.728446	0.035766	0.221389	0.024674	0.002561	0.000868	0.204712	0.572925	0.000183
-0.10711	0.009077	-0.0319	0.079508	0.172381	-0.17839	-0.26294	-0.01676	-0.25421
0.230708	0.91933	0.721788	0.37424	0.052628	0.044787	0.002821	0.851636	0.003926
-0.06016	-0.16351	-0.16013	-0.49258	-0.21201	0.273061	-0.01515	-0.14073	0.360933
0.501659	0.066236	0.072118	4.04E-09	0.016716	0.001896	0.865768	0.114529	3.06E-05
0.013576	-0.13163	0.013743	-0.32809	0.059757	0.053234	-0.04691	-0.05266	0.384222
0.879592	0.140184	0.878124	0.000166	0.504539	0.552237	0.600505	0.556564	8.21E-06
-0.25943	0.196821	-0.40979	0.065458	-0.01368	-0.31572	-0.13377	0.103319	-0.15453
0.003226	0.026564	1.72E-06	0.464678	0.878656	0.000299	0.133787	0.247711	0.082805
-0.07767	0.063574	-0.13712	-0.0778	0.185083	-0.26651	-0.11182	0.081051	0.123617
0.385447	0.477654	0.124241	0.38463	0.037234	0.002456	0.210694	0.36501	0.166165
-0.05729	-0.04028	-0.04341	-0.04861	-0.02414	-0.11473	-0.16382	0.194649	0.113455
0.522296	0.652975	0.627987	0.587354	0.787667	0.198995	0.065716	0.028313	0.204072
0.284899	0.135309	0.167635	0.234459	0.291988	-0.26653	0.003157	-0.08839	0.220953
0.001168	0.129328	0.059589	0.007974	0.000866	0.002455	0.971898	0.323049	0.012549
0.521773	0.399749	0.59914	0.223131	0.24982	-0.00755	0.640691	0.541399	-0.15902
3.17E-10	3.22E-06	9.98E-14	0.011683	0.004618	0.932831	4.98E-16	4.96E-11	0.07414
0.545772	0.774411	0.664559	0.858778	0.547258	-0.56082	0.722443	0.604501	-0.50374
3.23E-11	1.29E-26	1.61E-17	4.1E-38	2.79E-11	7.02E-12	9.11E-22	5.26E-14	1.57E-09
0.390543	0.531499	0.553096	0.486346	0.468923	-0.45592	0.442539	0.407363	-0.34667
5.64E-06	1.28E-10	1.55E-11	6.76E-09	2.69E-08	7.19E-08	1.89E-07	2E-06	6.53E-05
0.07087	-0.09695	0.284501	-0.05433	-0.25358	0.377011	0.095471	0.221668	-0.13822
0.428499	0.278208	0.001188	0.544068	0.004019	1.25E-05	0.285651	0.012259	0.121217
0.236632	-0.01052	0.267328	-0.13155	-0.19013	0.324579	0.322298	0.003674	0.13039
0.007395	0.906557	0.002379	0.140423	0.032273	0.000197	0.00022	0.967299	0.143977
0.108318	0.318697	0.006411	0.152758	-0.05809	-0.11478	0.48184	0.123476	-0.26458
0.225442	0.00026	0.942973	0.086428	0.516547	0.198795	9.73E-09	0.166651	0.002648
0.571896	0.582246	0.639708	0.584191	0.21781	-0.21203	0.821053	0.448384	-0.38082
2.17E-12	6.97E-13	5.7E-16	5.6E-13	0.013896	0.016709	3.18E-32	1.25E-07	1E-05
-0.11657	-0.06219	-0.0497	0.277788	-0.05118	-0.1139	-0.15453	-0.12967	-0.19941
0.191833	0.487331	0.578996	0.001567	0.567682	0.202279	0.082806	0.14621	0.024597
-0.40654	-0.53504	-0.45054	-0.46144	-0.55507	0.457838	-0.4434	-0.44224	0.274331
2.11E-06	9.16E-11	1.07E-07	4.76E-08	1.27E-11	6.23E-08	1.78E-07	1.93E-07	0.001802
-0.57918	-0.65926	-0.6022	-0.58572	-0.23983	0.319802	-0.79178	-0.56014	0.35401
9.8E-13	3.55E-17	6.94E-14	4.72E-13	0.006612	0.000247	1.55E-28	7.53E-12	4.44E-05
-0.49379	-0.54367	-0.51905	-0.33094	-0.34326	0.335077	-0.68244	-0.45903	0.113048
3.66E-09	3.97E-11	4.06E-10	0.000145	7.79E-05	0.000118	1E-18	5.7E-08	0.205712
-0.31313	-0.22983	-0.33084	-0.44267	-0.55285	0.369792	-0.2226	-0.18866	0.308333
0.000338	0.009339	0.000145	1.88E-07	1.59E-11	1.88E-05	0.011891	0.033659	0.000421

TEC	TFEC	TFR2	TGFBR2	TGM2	TGM5	THEMIS2	THNSL1	TIFA
brown	brown	brown	brown	brown	brown	brown	brown	brown
0.271869	0.1955	-0.15354	0.17719	0.084779	-0.02603	0.327205	0.016759	0.107524
0.001988	0.027617	0.084804	0.046274	0.343298	0.771421	0.000173	0.85165	0.228881
0.165639	0.054021	-0.03499	0.027663	0.050187	-0.06687	0.223125	0.111281	0.014378
0.062733	0.546368	0.696163	0.757536	0.575245	0.455058	0.011685	0.21293	0.872536
-0.16001	-0.18464	0.24703	-0.21989	-0.43919	-0.18762	-0.424	0.298455	-0.11271
0.07233	0.037696	0.005112	0.012993	2.4E-07	0.034666	6.79E-07	0.000654	0.20707
0.143495	0.242893	-0.00684	0.151449	-0.35864	-0.06848	-0.21709	0.150415	0.257122
0.107516	0.005932	0.939182	0.08919	3.47E-05	0.444282	0.01422	0.091422	0.003521
0.038231	0.154186	0.093601	-0.05757	-0.11272	0.03039	0.039759	0.263879	0.309489
0.669566	0.083493	0.295238	0.52032	0.20704	0.734481	0.657186	0.00272	0.000399
0.309436	0.479201	-0.06402	0.274969	-0.2479	0.029406	0.018422	0.182109	0.562137
0.0004	1.2E-08	0.474562	0.001756	0.004953	0.742769	0.83713	0.040449	6.12E-12
0.176761	0.224326	-0.06009	0.206211	-0.01315	-0.07804	-0.02301	0.036512	0.278374
0.046813	0.011231	0.502153	0.020021	0.88338	0.383155	0.797377	0.683611	0.00153
0.094604	0.400195	-0.34555	0.2253	-0.15369	0.406948	0.131322	-0.08634	0.394353
0.290073	3.14E-06	6.92E-05	0.010874	0.084502	2.06E-06	0.141111	0.334441	4.49E-06
0.508824	0.249715	-0.02652	0.612147	0.369643	0.130008	0.354706	-0.50032	0.239603
1.01E-09	0.004636	0.767301	2.06E-14	1.89E-05	0.145165	4.28E-05	2.11E-09	0.006665
0.734076	0.798156	-0.51174	0.849193	0.487756	0.493754	0.880826	-0.60923	0.60691
9.4E-23	2.75E-29	7.81E-10	1.82E-36	6.02E-09	3.67E-09	2.06E-42	2.96E-14	3.93E-14
0.555236	0.681781	-0.4025	0.672645	0.198335	0.361866	0.581664	-0.42402	0.55581
1.25E-11	1.11E-18	2.72E-06	4.7E-18	0.025399	2.91E-05	7.44E-13	6.78E-07	1.18E-11
-0.10623	-0.40546	0.15995	-0.08916	0.216459	-0.0066	-0.07066	-0.17012	-0.4212
0.23455	2.26E-06	0.072446	0.318822	0.014513	0.941257	0.429885	0.055859	8.17E-07
-0.20629	-0.41964	0.081968	-0.20639	-0.01166	0.00219	-0.14682	-0.11724	-0.47565
0.019972	9.06E-07	0.359591	0.019911	0.896486	0.980506	0.099527	0.189295	1.59E-08
-0.18204	-0.28027	0.105263	-0.26025	0.107064	0.185976	0.075756	-0.16823	-0.17781
0.040521	0.001415	0.238875	0.003128	0.230891	0.036311	0.397271	0.058671	0.045503
0.282803	0.141912	-0.30077	0.344922	0.423279	0.420893	0.553097	-0.59238	-0.00137
0.001275	0.111493	0.000591	7.15E-05	7.12E-07	8.34E-07	1.55E-11	2.2E-13	0.98779
-0.13095	-0.03252	-0.26179	-0.11883	0.146576	0.089398	0.17676	-0.02621	-0.29159
0.142264	0.716659	0.002948	0.183315	0.100095	0.317549	0.046814	0.769936	0.00088
-0.72099	-0.71124	0.303406	-0.73508	-0.27303	-0.32405	-0.59276	0.371664	-0.74321
1.2E-21	7.3E-21	0.000525	7.68E-23	0.001898	0.000202	2.11E-13	1.69E-05	1.45E-23
-0.39722	-0.24564	0.271261	-0.44178	-0.33001	-0.4722	-0.57725	0.484733	-0.1673
3.77E-06	0.005376	0.002037	2E-07	0.000151	2.09E-08	1.21E-12	7.7E-09	0.060103
-0.53029	-0.49316	0.292559	-0.52655	-0.06025	-0.37622	-0.43284	0.34075	-0.41607
1.44E-10	3.85E-09	0.000845	2.04E-10	0.501011	1.31E-05	3.73E-07	8.85E-05	1.15E-06
-0.26067	-0.47524	0.261603	-0.41268	-0.19908	-0.52971	-0.40583	0.520204	-0.56163
0.003077	1.65E-08	0.002969	1.43E-06	0.024842	1.52E-10	2.21E-06	3.65E-10	6.45E-12

TIGD3	TIGIT	TIMD4	TIMMDC1	TK2	TLR1	TLR2	TLR3	TLR4
brown	brown	brown	brown	brown	brown	brown	brown	brown
-0.23791	0.296735	0.314911	0.11301	0.203224	0.24364	0.226286	0.132053	0.415927
0.007072	0.000705	0.000311	0.205861	0.021932	0.005776	0.010522	0.13889	1.16E-06
-0.11496	0.106558	0.184211	0.085224	0.158453	0.082438	0.129185	-0.00416	0.335905
0.198077	0.233116	0.038153	0.340758	0.075195	0.356836	0.14775	0.96297	0.000113
0.226282	-0.1815	-0.26616	-0.09501	-0.44248	-0.12775	-0.34533	0.012028	-0.52563
0.010524	0.041133	0.00249	0.287985	1.9E-07	0.152333	7E-05	0.893235	2.22E-10
-0.02918	0.030433	0.062099	0.075814	-0.23517	0.240623	0.042307	0.382435	-0.18537
0.744654	0.734116	0.487953	0.396909	0.007781	0.006429	0.636735	9.12E-06	0.036934
0.23848	0.007321	0.238852	0.526544	-0.3407	0.076213	0.043329	0.100158	0.051848
0.006933	0.93489	0.006843	2.04E-10	8.87E-05	0.394422	0.628606	0.262551	0.562653
-0.03466	0.161891	0.349761	0.452968	-0.26067	0.404212	0.26369	0.52477	0.114538
0.698886	0.069003	5.56E-05	8.93E-08	0.003077	2.44E-06	0.00274	2.4E-10	0.19976
-0.00179	0.008633	0.239626	0.244759	-0.13121	0.244206	0.188177	0.260619	-0.01692
0.984029	0.923258	0.00666	0.005548	0.141463	0.00566	0.034121	0.003083	0.850243
0.025081	0.211336	0.228613	0.367718	0.219522	0.376487	0.376937	0.300978	0.339588
0.77956	0.017075	0.009733	2.11E-05	0.013147	1.29E-05	1.25E-05	0.000585	9.39E-05
-0.48628	0.360229	0.148369	0.111331	0.068649	0.411608	0.390189	0.256397	-0.01008
6.79E-09	3.18E-05	0.095966	0.212723	0.443145	1.53E-06	5.77E-06	0.003618	0.910424
-0.53888	0.719733	0.601413	0.511442	0.467821	0.82377	0.884828	0.575151	0.680428
6.34E-11	1.52E-21	7.62E-14	8.01E-10	2.93E-08	1.34E-32	2.78E-43	1.53E-12	1.38E-18
-0.43516	0.642379	0.592484	0.377369	0.369938	0.761404	0.656387	0.67858	0.563584
3.18E-07	3.95E-16	2.18E-13	1.22E-05	1.86E-05	2.75E-25	5.41E-17	1.85E-18	5.26E-12
-0.15423	-0.16723	-0.27689	-0.13979	0.244879	-0.2223	-0.18691	-0.32836	-0.21023
0.083411	0.060223	0.001625	0.116995	0.005525	0.012008	0.035365	0.000164	0.017675
0.034755	-0.0957	-0.39111	-0.37281	0.08918	-0.2451	-0.28239	-0.3805	-0.42388
0.698081	0.284508	5.46E-06	1.59E-05	0.318734	0.005481	0.001297	1.02E-05	6.84E-07
0.206959	0.000996	-0.19384	-0.01189	-0.04539	-0.23226	-0.19094	-0.39976	-0.27346
0.019565	0.991137	0.028993	0.894485	0.612348	0.008599	0.031531	3.22E-06	0.001866
-0.31333	0.411354	0.06418	0.099196	0.444572	0.274842	0.361715	-0.05603	0.169177
0.000335	1.55E-06	0.473462	0.267185	1.64E-07	0.001765	2.94E-05	0.531553	0.057249
0.036603	-0.10521	-0.01444	0.016731	0.329162	-0.18804	0.042835	-0.21889	0.329576
0.682871	0.239118	0.872029	0.851901	0.000158	0.03425	0.632526	0.013422	0.000155
0.480108	-0.59999	-0.57098	-0.48532	-0.2651	-0.7217	-0.73819	-0.5723	-0.47981
1.12E-08	9.02E-14	2.4E-12	7.34E-09	0.002595	1.05E-21	4.1E-23	2.08E-12	1.15E-08
0.304766	-0.47365	-0.21349	-0.37093	-0.3306	-0.44171	-0.45493	-0.08248	-0.16219
0.000494	1.86E-08	0.015956	1.76E-05	0.000147	2.01E-07	7.73E-08	0.356566	0.068486
0.297904	-0.50943	-0.29458	-0.41757	-0.14576	-0.58736	-0.49165	-0.39183	-0.10742
0.00067	9.57E-10	0.000774	1.04E-06	0.102022	3.92E-13	4.37E-09	5.22E-06	0.229334
0.320662	-0.38367	-0.33253	-0.20347	-0.44239	-0.43286	-0.49409	-0.42479	-0.5782
0.000237	8.48E-06	0.000134	0.021767	1.92E-07	3.72E-07	3.56E-09	6.44E-07	1.09E-12

TLR5	TLR6	TLR7	TLR8	TM6SF1	TMBIM1	TMBIM4	TMC8	TMCO4
brown	brown	brown	brown	brown	brown	brown	brown	brown
0.230033	0.080943	0.357708	0.177983	0.228647	0.253356	0.215505	0.226377	0.174253
0.009276	0.365653	3.65E-05	0.04529	0.009721	0.004053	0.014963	0.01049	0.050074
0.126713	-0.09063	0.174914	0.002539	0.164634	0.259733	0.219847	0.044296	0.079894
0.155726	0.310903	0.049197	0.977398	0.064368	0.003189	0.013009	0.620954	0.371921
-0.31494	-0.12904	-0.34159	-0.2239	-0.551	-0.49152	-0.21996	-0.33814	-0.12052
0.000311	0.148203	8.48E-05	0.011391	1.92E-11	4.41E-09	0.01296	0.000101	0.177107
0.03693	0.190609	-0.04834	0.068761	-0.32854	-0.17879	0.081485	-0.23967	0.201064
0.680187	0.03183	0.589419	0.442401	0.000163	0.044312	0.362437	0.006649	0.02341
0.178086	-0.21537	0.046299	0.11587	-0.16322	0.058881	0.119669	-0.13225	0.170645
0.045164	0.015027	0.605237	0.194546	0.066727	0.510814	0.180218	0.138288	0.055092
0.304037	0.168018	0.151548	0.284461	-0.17453	0.078332	0.277242	-0.18593	0.385745
0.000511	0.059	0.088979	0.00119	0.049702	0.381372	0.001602	0.036357	7.51E-06
0.202637	0.010935	0.023881	0.126664	-0.14528	0.100296	0.257894	-0.16261	0.265094
0.022326	0.902889	0.789853	0.155887	0.103168	0.261889	0.003419	0.067755	0.002595
0.397305	0.315999	0.399549	0.241717	0.118185	0.263546	0.080031	0.180637	0.382124
3.75E-06	0.000296	3.26E-06	0.006185	0.18572	0.002756	0.371098	0.042124	9.29E-06
0.209569	0.360773	0.043965	0.376733	-0.0406	0.138423	0.361414	0.287969	0.381506
0.018045	3.09E-05	0.62357	1.27E-05	0.650416	0.120662	2.98E-05	0.001027	9.63E-06
0.76875	0.754341	0.726654	0.801289	0.496359	0.607259	0.528134	0.823314	0.751696
5E-26	1.33E-24	4.06E-22	1.15E-29	2.95E-09	3.76E-14	1.76E-10	1.55E-32	2.38E-24
0.52291	0.66864	0.671891	0.57617	0.339584	0.461971	0.460481	0.584288	0.677727
2.85E-10	8.7E-18	5.28E-18	1.36E-12	9.39E-05	4.57E-08	5.12E-08	5.54E-13	2.12E-18
-0.22588	-0.10228	-0.25335	-0.24455	0.045656	-0.07273	0.340172	0.00698	-0.11289
0.010665	0.252503	0.004054	0.00559	0.610262	0.41645	9.12E-05	0.937918	0.206333
-0.33039	-0.01748	-0.26886	-0.2647	-0.22206	-0.28279	-0.24731	0.136977	-0.21641
0.000148	0.845337	0.00224	0.002635	0.012101	0.001276	0.00506	0.124628	0.014538
-0.22712	-0.18119	-0.13984	-0.13768	-0.10811	0.019246	-0.14588	0.267068	-0.04828
0.010233	0.041492	0.11688	0.122673	0.226344	0.829953	0.101735	0.002403	0.589846
0.244682	0.408729	0.284111	0.294999	0.253738	0.318989	0.156884	0.750982	0.308463
0.005564	1.84E-06	0.001208	0.00076	0.003996	0.000257	0.078165	2.78E-24	0.000418
0.150459	-0.04449	0.249592	-0.09899	0.440888	0.24834	-0.07545	0.2707	-0.16467
0.091325	0.619459	0.004657	0.268165	2.13E-07	0.004874	0.399184	0.002083	0.064307
-0.63102	-0.56105	-0.45638	-0.66852	-0.20265	-0.502	-0.52433	-0.38761	-0.70709
1.84E-15	6.85E-12	6.94E-08	8.86E-18	0.022317	1.82E-09	2.5E-10	6.72E-06	1.54E-20
-0.37858	-0.44035	-0.29942	-0.38564	-0.0714	-0.33893	-0.28475	-0.69553	-0.47541
1.14E-05	2.21E-07	0.000627	7.55E-06	0.425019	9.71E-05	0.001176	1.15E-19	1.62E-08
-0.41344	-0.53654	-0.30323	-0.45987	0.06545	-0.24307	-0.36013	-0.47896	-0.59314
1.36E-06	7.93E-11	0.000529	5.36E-08	0.464732	0.005895	3.2E-05	1.23E-08	2.02E-13
-0.38262	-0.38954	-0.46531	-0.3163	-0.44544	-0.75473	-0.36907	-0.34175	-0.55401
9.02E-06	5.99E-06	3.55E-08	0.000291	1.54E-07	1.22E-24	1.96E-05	8.41E-05	1.41E-11

TMEM106	TMEM108	TMEM109	TMEM119	TMEM140	TMEM150	TMEM156	TMEM173	TMEM176
brown	brown	brown	brown	brown	brown	brown	brown	brown
0.190935	-0.4254	0.214093	0.104635	0.479189	0.273387	-0.01235	0.245157	0.27728
0.031533	6.18E-07	0.015651	0.241706	1.2E-08	0.001871	0.890434	0.00547	0.001599
-0.00988	-0.39579	0.074	0.026889	0.409022	0.138523	-0.08489	0.083452	0.2282
0.912172	4.11E-06	0.408333	0.764113	1.8E-06	0.120391	0.342637	0.350933	0.009869
-0.10371	0.197124	-0.3026	-0.33367	-0.32191	-0.18582	-0.13128	-0.28481	-0.38939
0.245932	0.026327	0.000544	0.000126	0.000224	0.036474	0.141237	0.001173	6.05E-06
0.15245	-0.11107	-0.17723	-0.32089	-0.1308	-0.11998	0.047609	-0.18387	-0.17553
0.08707	0.213803	0.046228	0.000235	0.142702	0.179077	0.595054	0.038515	0.048389
-0.07279	-0.31091	-0.003	0.072456	0.180892	0.008237	0.144922	0.006151	0.204163
0.416039	0.000374	0.97333	0.418213	0.041829	0.926774	0.104029	0.945279	0.021315
0.195521	-0.42223	-0.01529	-0.13072	0.123717	-0.00685	0.204629	-0.03865	0.112849
0.0276	7.63E-07	0.864542	0.14296	0.165818	0.939081	0.021014	0.666132	0.206515
0.06556	-0.20585	0.02294	-0.11794	0.133566	0.000288	0.107783	0.049287	0.022264
0.463978	0.020247	0.797952	0.186628	0.134382	0.997434	0.227756	0.582132	0.803782
0.200359	-0.3456	0.233063	0.100824	0.050237	0.082053	0.188407	0.10135	0.271228
0.023911	6.91E-05	0.008366	0.259376	0.574866	0.359092	0.033898	0.256883	0.00204
0.538523	-0.08021	0.49292	0.018826	0.150797	0.505594	-0.09734	0.515975	0.226426
6.56E-11	0.370026	3.93E-09	0.833603	0.090593	1.34E-09	0.276287	5.35E-10	0.010473
0.871502	-0.55417	0.77421	0.515001	0.666522	0.809848	0.430369	0.808105	0.737777
1.69E-40	1.39E-11	1.35E-26	5.84E-10	1.2E-17	9.75E-31	4.42E-07	1.63E-30	4.45E-23
0.750223	-0.54797	0.630608	0.345953	0.570014	0.566027	0.357309	0.562086	0.542343
3.27E-24	2.6E-11	1.94E-15	6.78E-05	2.66E-12	4.07E-12	3.73E-05	6.15E-12	4.52E-11
0.008158	0.152666	0.013583	-0.00654	-0.05233	-0.00762	-0.22074	0.055734	-0.02609
0.927468	0.086619	0.879528	0.941789	0.559036	0.932232	0.012635	0.533701	0.770933
0.007301	0.42493	-0.03839	-0.08713	-0.16329	0.08743	-0.19591	-0.01391	-0.21427
0.935068	6.38E-07	0.668245	0.330004	0.066603	0.32836	0.027288	0.876657	0.015562
0.011832	0.236421	0.174242	0.206693	0.090269	0.244682	-0.0102	0.105358	0.154991
0.894968	0.00745	0.050089	0.019726	0.312838	0.005564	0.909357	0.238449	0.081873
0.552459	-0.01444	0.575223	0.412368	0.416491	0.654532	0.143886	0.584713	0.442074
1.65E-11	0.871984	1.51E-12	1.45E-06	1.11E-06	7.08E-17	0.10655	5.28E-13	1.96E-07
-0.11447	-0.0855	-0.00867	0.31973	0.321419	-0.02197	0.179583	0.080129	0.130449
0.200022	0.339212	0.922969	0.000248	0.000229	0.806284	0.043358	0.370508	0.143794
-0.64995	0.581912	-0.54038	-0.15371	-0.44532	-0.55252	-0.25704	-0.46978	-0.51946
1.37E-16	7.23E-13	5.48E-11	0.084455	1.55E-07	1.64E-11	0.003532	2.52E-08	3.91E-10
-0.62995	0.197133	-0.59849	-0.37248	-0.50601	-0.70937	-0.17532	-0.62348	-0.5515
2.12E-15	0.02632	1.08E-13	1.61E-05	1.29E-09	1.02E-20	0.048665	4.92E-15	1.82E-11
-0.6997	0.329024	-0.44675	-0.14051	-0.42868	-0.57138	-0.27175	-0.43203	-0.39906
5.62E-20	0.000159	1.4E-07	0.115097	4.95E-07	2.3E-12	0.001998	3.94E-07	3.36E-06
-0.34599	0.392062	-0.4862	-0.35719	-0.27443	-0.26881	-0.26163	-0.26294	-0.58917
6.77E-05	5.15E-06	6.83E-09	3.75E-05	0.001795	0.002244	0.002966	0.002821	3.19E-13

TMEM176	TMEM177	TMEM178	TMEM189	TMEM198	TMEM26	TMEM50A	TMEM52B	TMEM86A
brown	brown	brown	brown	brown	brown	brown	brown	brown
0.257367	-0.20802	-0.35416	0.377589	-0.37525	0.185612	0.207048	0.238856	0.3631
0.003488	0.018932	4.41E-05	1.21E-05	1.38E-05	0.036685	0.019511	0.006843	2.72E-05
0.17277	-0.04163	-0.2708	0.437861	-0.26536	0.052969	0.198339	0.184441	0.21393
0.052089	0.642133	0.002075	2.63E-07	0.002569	0.554223	0.025396	0.037908	0.015733
-0.33805	0.134468	0.386874	-0.21613	0.329786	-0.02627	-0.1974	-0.22885	-0.39594
0.000101	0.131746	7.02E-06	0.014666	0.000153	0.769365	0.02611	0.009654	4.07E-06
-0.07282	-0.06245	0.140647	-0.16868	-0.16216	0.321326	0.05802	-0.03112	-0.30818
0.415855	0.485484	0.114752	0.057989	0.068537	0.00023	0.517025	0.728374	0.000424
0.199074	0.393944	-0.46042	0.385084	-0.02864	-0.10125	0.304071	0.089065	-0.06864
0.024847	4.6E-06	5.14E-08	7.81E-06	0.749283	0.257377	0.00051	0.319364	0.443206
0.201045	0.0976	-0.31005	0.171469	-0.35338	0.348393	0.399878	0.151358	-0.17933
0.023424	0.274998	0.000389	0.053911	4.59E-05	5.97E-05	3.2E-06	0.089384	0.04366
0.10335	0.047222	-0.179	0.207341	-0.29809	0.220143	0.329008	0.183481	-0.1192
0.247567	0.598057	0.044056	0.019336	0.000665	0.012884	0.000159	0.038938	0.181926
0.312966	-0.1372	-0.24838	-0.10082	-0.49361	0.182691	0.097246	0.1845	0.086624
0.00034	0.124017	0.004866	0.259393	3.71E-09	0.039801	0.276749	0.037847	0.332861
0.340077	-0.3738	-0.20546	0.05445	-0.25333	0.580712	0.400144	0.051039	0.064858
9.16E-05	1.5E-05	0.020488	0.543183	0.004057	8.27E-13	3.15E-06	0.568766	0.468789
0.805471	-0.56392	-0.75095	0.491446	-0.68022	0.599499	0.698457	0.592613	0.674013
3.49E-30	5.07E-12	2.8E-24	4.44E-09	1.43E-18	9.56E-14	6.96E-20	2.14E-13	3.8E-18
0.6165	-0.51169	-0.49535	0.217053	-0.58633	0.563525	0.444859	0.450672	0.446993
1.2E-14	7.84E-10	3.21E-09	0.014239	4.4E-13	5.29E-12	1.61E-07	1.06E-07	1.38E-07
-0.04331	-0.1442	0.125864	-0.05319	0.134835	-0.13378	-0.14357	-0.12962	-0.0498
0.628766	0.105784	0.158539	0.552573	0.130685	0.133746	0.107338	0.146391	0.578184
-0.23705	-0.22379	0.398016	-0.19867	0.340235	-0.16747	-0.3718	-0.19878	0.007791
0.007289	0.011432	3.59E-06	0.025145	9.09E-05	0.059849	1.68E-05	0.025064	0.930727
0.068597	0.053434	-0.05129	0.129766	0.472675	-0.27935	-0.1373	-0.16235	0.127582
0.443491	0.550749	0.566894	0.145924	2.01E-08	0.00147	0.123721	0.068216	0.152887
0.417932	-0.50805	-0.29533	0.288397	-0.11375	0.16293	0.205731	0.197309	0.546224
1.01E-06	1.08E-09	0.00075	0.001009	0.202873	0.067216	0.020318	0.026184	3.09E-11
0.015159	-0.06861	-0.1127	0.351814	-0.04418	-0.28454	0.028114	0.203993	0.393445
0.865677	0.443422	0.207123	4.99E-05	0.621861	0.001186	0.753703	0.021426	4.74E-06
-0.61251	0.317119	0.702517	-0.33839	0.698537	-0.64837	-0.63331	-0.34794	-0.28941
1.97E-14	0.000281	3.44E-20	9.98E-05	6.87E-20	1.71E-16	1.35E-15	6.12E-05	0.000966
-0.55792	0.46232	0.485103	-0.45393	0.272084	-0.23585	-0.38756	-0.29162	-0.46269
9.47E-12	4.45E-08	7.48E-09	8.32E-08	0.001971	0.007598	6.74E-06	0.000879	4.33E-08
-0.46988	0.434131	0.416463	-0.35241	0.375493	-0.4846	-0.46271	-0.24222	-0.26079
2.5E-08	3.41E-07	1.12E-06	4.84E-05	1.36E-05	7.79E-09	4.32E-08	0.006076	0.003063
-0.58421	0.182843	0.249242	-0.0262	0.372306	-0.38045	-0.29983	-0.2598	-0.26104
5.59E-13	0.039635	0.004717	0.769951	1.63E-05	1.02E-05	0.000616	0.003181	0.003034

TMIGD3	TMPRSS13	TNF	TNFAIP2	TNFAIP3	TNFAIP8	TNFAIP8L2	TNFRSF10	TNFRSF11
brown	brown	brown	brown	brown	brown	brown	brown	brown
0.268695	0.314454	0.321338	0.249878	0.141767	0.178231	0.336716	0.12016	0.161983
0.002255	0.000318	0.00023	0.004608	0.111864	0.044987	0.000109	0.178421	0.068844
0.239853	0.189924	0.237263	0.140287	0.031761	0.00285	0.25721	-0.01991	0.056848
0.006607	0.032462	0.007235	0.115693	0.722981	0.974631	0.003509	0.82418	0.525544
-0.36148	-0.23412	-0.44004	-0.2668	-0.06294	0.034075	-0.26114	-0.12066	-0.10438
2.97E-05	0.008069	2.26E-07	0.002429	0.48204	0.703709	0.003023	0.176625	0.242846
-0.18461	-0.23105	-0.267	-0.05376	0.263119	0.340561	-0.07097	0.100113	0.147073
0.037732	0.008963	0.002409	0.548283	0.002801	8.94E-05	0.427874	0.262767	0.098938
0.317177	0.088772	0.089687	-0.05777	-0.03156	0.232596	0.441523	-0.13679	0.103833
0.00028	0.320966	0.31598	0.518863	0.724685	0.008501	2.04E-07	0.12515	0.245351
0.159112	-0.09921	-0.05068	0.055729	0.33052	0.54609	0.319158	0.126853	0.334254
0.073975	0.267127	0.5715	0.533736	0.000148	3.13E-11	0.000255	0.155267	0.000123
0.172467	-0.04715	0.134509	-0.0238	0.275614	0.318704	0.236648	0.074119	0.221121
0.052509	0.598639	0.131629	0.790577	0.001711	0.00026	0.007391	0.407577	0.01248
0.039788	-0.07798	0.273025	0.240883	0.132105	0.286059	0.252834	0.009536	0.16336
0.656946	0.383506	0.001899	0.006371	0.138734	0.001113	0.004132	0.915258	0.066488
0.096663	0.208116	-0.0027	0.385529	0.660552	0.581173	0.139767	0.467108	0.333423
0.279653	0.018878	0.975964	7.61E-06	2.93E-17	7.86E-13	0.117062	3.09E-08	0.000128
0.72087	0.571736	0.526115	0.815043	0.716151	0.780333	0.785674	0.577141	0.431065
1.23E-21	2.21E-12	2.12E-10	2.05E-31	2.97E-21	2.98E-27	7.68E-28	1.23E-12	4.21E-07
0.405136	0.339456	0.342842	0.58611	0.593054	0.641813	0.550714	0.535339	0.272748
2.3E-06	9.45E-05	7.96E-05	4.51E-13	2.04E-13	4.27E-16	1.97E-11	8.91E-11	0.00192
-0.13043	0.07619	-0.08519	-0.08482	-0.01828	-0.345	-0.18459	-0.04049	-0.34696
0.143865	0.394565	0.340946	0.343043	0.838381	7.12E-05	0.037755	0.651286	6.43E-05
-0.25988	0.070391	-0.16353	-0.02451	-0.11441	-0.27254	-0.34669	-0.04959	-0.3439
0.003171	0.431637	0.066209	0.784441	0.200261	0.001936	6.53E-05	0.579786	7.53E-05
0.048553	0.201345	0.02194	0.060301	-0.20056	-0.1697	-0.00292	-0.07256	-0.34328
0.587767	0.023213	0.806587	0.500658	0.023769	0.056478	0.974002	0.417531	7.78E-05
0.35422	0.486175	0.296032	0.532851	0.285887	0.216637	0.275116	0.320304	-0.08025
4.4E-05	6.85E-09	0.000727	1.13E-10	0.001121	0.01443	0.001746	0.000241	0.369779
0.345214	0.14679	0.316735	-0.00699	-0.4	-0.41468	0.17331	-0.15427	-0.23234
7.04E-05	0.099596	0.000286	0.93786	3.17E-06	1.25E-06	0.051348	0.08332	0.008577
-0.43028	-0.28043	-0.25222	-0.61456	-0.72422	-0.82613	-0.54268	-0.50773	-0.55082
4.44E-07	0.001406	0.004227	1.53E-14	6.49E-22	6.23E-33	4.38E-11	1.11E-09	1.95E-11
-0.486	-0.52715	-0.35245	-0.5744	-0.45094	-0.44795	-0.48986	-0.34242	-0.03944
6.95E-09	1.93E-10	4.83E-05	1.66E-12	1.04E-07	1.29E-07	5.06E-09	8.13E-05	0.659793
-0.33576	-0.32216	-0.10469	-0.49431	-0.57748	-0.68768	-0.42479	-0.42201	-0.189
0.000114	0.000221	0.241464	3.5E-09	1.18E-12	4.26E-19	6.44E-07	7.75E-07	0.033333
-0.2145	0.051984	-0.27414	-0.41107	-0.27833	-0.24762	-0.28636	-0.31603	-0.15541
0.015449	0.561627	0.001816	1.58E-06	0.001532	0.005004	0.001099	0.000295	0.08104

TNFRSF14	TNFRSF17	TNFRSF1B	TNFRSF8	TNFRSF9	TNFSF10	TNFSF13	TNFSF13B	TNFSF14
brown	brown	brown	brown	brown	brown	brown	brown	brown
0.301806	0.191573	0.214701	0.120797	0.142823	0.425332	0.279659	0.289542	0.269058
0.000564	0.030959	0.015352	0.176115	0.10919	6.21E-07	0.001451	0.000961	0.002223
0.189181	0.105464	0.055697	-0.01295	-0.00138	0.219823	0.173664	0.064759	0.194518
0.033159	0.237976	0.533976	0.885092	0.98772	0.013019	0.050866	0.46947	0.028423
-0.39632	-0.10416	-0.36754	-0.2165	0.030053	-0.30262	-0.46091	-0.09289	-0.37454
3.98E-06	0.243866	2.13E-05	0.014496	0.737321	0.000544	4.95E-08	0.298953	1.44E-05
-0.26225	0.041317	-0.25903	-0.15979	0.24264	0.068121	-0.45449	0.209099	-0.15052
0.002897	0.644645	0.003276	0.072729	0.005985	0.446671	7.98E-08	0.018311	0.091197
0.198245	0.186238	-0.06921	0.01705	0.24933	0.196943	-0.17912	0.102114	-0.03498
0.025467	0.036045	0.439405	0.849105	0.004701	0.026468	0.043913	0.253297	0.696181
0.024045	0.246806	-0.12811	-0.05428	0.41701	0.370874	-0.38771	0.373289	-0.02474
0.788444	0.005154	0.151187	0.544447	1.08E-06	1.77E-05	6.68E-06	1.54E-05	0.782503
0.028091	0.090388	-0.0696	-0.06197	0.261383	0.247973	-0.22403	0.127172	0.134049
0.753901	0.312196	0.436832	0.488873	0.002995	0.00494	0.011343	0.15422	0.132966
0.175106	0.105634	0.126487	0.02149	0.18974	0.316137	-0.01583	0.241192	0.338985
0.048944	0.237218	0.15647	0.810478	0.032633	0.000294	0.859761	0.006301	9.68E-05
0.269445	0.238224	0.446888	0.372015	0.389641	0.32258	0.082892	0.377941	0.33726
0.002189	0.006996	1.39E-07	1.66E-05	5.96E-06	0.000217	0.354181	1.18E-05	0.000106
0.752848	0.489405	0.841983	0.610247	0.569097	0.78684	0.459874	0.748656	0.748003
1.85E-24	5.26E-09	2.66E-35	2.61E-14	2.93E-12	5.68E-28	5.35E-08	4.59E-24	5.27E-24
0.547895	0.363742	0.538104	0.402912	0.48689	0.805316	0.264208	0.811283	0.521194
2.62E-11	2.63E-05	6.83E-11	2.65E-06	6.46E-09	3.65E-30	0.002686	6.37E-31	3.34E-10
-0.11625	-0.22627	-0.01044	0.212778	-0.21432	-0.26923	0.026253	-0.27634	0.017604
0.193063	0.010529	0.907255	0.016317	0.015541	0.002207	0.76954	0.001662	0.844269
-0.13458	-0.20389	0.034342	0.039403	-0.20481	-0.41237	0.1012	-0.24914	0.012233
0.131418	0.021492	0.701492	0.660064	0.020899	1.45E-06	0.257593	0.004735	0.891427
0.300498	-0.02148	0.193166	0.119507	-0.07528	-0.25296	0.304582	-0.10125	0.11441
0.000598	0.8106	0.029564	0.180812	0.400224	0.004113	0.000498	0.257356	0.200266
0.551289	0.172532	0.665621	0.460204	0.137225	0.168253	0.530014	0.269508	0.588726
1.86E-11	0.052418	1.38E-17	5.22E-08	0.12394	0.058641	1.47E-10	0.002183	3.35E-13
0.057711	-0.04932	0.061471	-0.06382	-0.40421	-0.07191	0.22011	-0.18903	0.183162
0.519265	0.581861	0.492373	0.47595	2.44E-06	0.421748	0.012898	0.033306	0.039284
-0.56036	-0.46002	-0.50484	-0.39432	-0.61964	-0.73566	-0.17606	-0.64593	-0.53778
7.37E-12	5.3E-08	1.43E-09	4.5E-06	8.06E-15	6.84E-23	0.047703	2.41E-16	7.04E-11
-0.59392	-0.25837	-0.65745	-0.5069	-0.33676	-0.31598	-0.34387	-0.35107	-0.65537
1.84E-13	0.003358	4.63E-17	1.19E-09	0.000108	0.000296	7.55E-05	5.19E-05	6.27E-17
-0.42891	-0.34145	-0.4271	-0.3263	-0.48809	-0.39169	-0.04739	-0.55634	-0.46403
4.87E-07	8.54E-05	5.51E-07	0.000181	5.86E-09	5.27E-06	0.596783	1.11E-11	3.91E-08
-0.41739	-0.09093	-0.33041	-0.18281	-0.17942	-0.42722	-0.26956	-0.44367	-0.47855
1.05E-06	0.309275	0.000148	0.039673	0.043551	5.47E-07	0.002179	1.75E-07	1.27E-08

TNFSF8	TNNI2	TOR3A	TP53BP2	TPD52L2	TPM3	TPP1	TPST2	TRAF1
brown	brown	brown	brown	brown	brown	brown	brown	brown
0.303032	0.130424	0.017294	-0.184	0.187335	0.329823	0.354382	0.257157	0.307671
0.000534	0.143873	0.84697	0.038381	0.034945	0.000153	4.36E-05	0.003516	0.000434
0.156923	-0.00084	-0.07633	-0.07685	0.151184	0.220087	0.296442	0.170154	0.288441
0.07809	0.992524	0.393681	0.390455	0.089757	0.012908	0.000714	0.055806	0.001007
-0.16718	-0.07342	0.025844	0.087592	-0.21374	-0.28172	-0.31303	-0.11201	-0.44189
0.060298	0.412045	0.773026	0.327465	0.015826	0.001334	0.000339	0.209926	1.98E-07
0.174087	-0.16419	0.289772	0.129221	0.033961	-0.10344	0.049106	0.03172	-0.13285
0.050296	0.065106	0.000952	0.147636	0.704652	0.24717	0.583514	0.723327	0.136501
0.151695	0.188962	0.483836	-0.22898	0.282418	0.207339	-0.20305	0.393018	0.087348
0.088665	0.033367	8.28E-09	0.009613	0.001296	0.019337	0.022049	4.86E-06	0.32882
0.377313	-0.03791	0.592961	-0.06923	0.303266	0.186187	0.094784	0.344417	0.106971
1.23E-05	0.672183	2.06E-13	0.439278	0.000529	0.036096	0.289151	7.34E-05	0.231297
0.270935	-0.00206	0.399879	0.043767	0.392649	0.092203	0.193123	0.016498	0.300862
0.002064	0.981672	3.2E-06	0.625137	4.97E-06	0.302543	0.029601	0.853934	0.000588
0.228896	-0.07367	0.46618	0.054035	0.130885	0.149645	0.207118	0.066573	0.262827
0.00964	0.410407	3.32E-08	0.546266	0.142449	0.093111	0.019469	0.45709	0.002833
0.437982	0.273433	0.256761	-0.39831	0.338937	0.358027	0.392645	0.216251	0.19579
2.61E-07	0.001868	0.003569	3.52E-06	9.7E-05	3.59E-05	4.97E-06	0.014611	0.027382
0.891458	0.597596	0.548958	-0.5493	0.652841	0.66131	0.7085	0.442448	0.680726
8.44E-45	1.2E-13	2.35E-11	2.27E-11	9.03E-17	2.62E-17	1.19E-20	1.91E-07	1.32E-18
0.761837	0.276137	0.532911	-0.28199	0.458963	0.533088	0.60121	0.352894	0.433696
2.49E-25	0.001675	1.12E-10	0.001318	5.73E-08	1.1E-10	7.8E-14	4.72E-05	3.51E-07
-0.05661	0.032261	-0.23296	0.046637	-0.098	-0.07303	0.125045	-0.18023	-0.05909
0.527245	0.718798	0.008395	0.602603	0.273037	0.414496	0.161285	0.042597	0.50929
-0.20413	0.148429	-0.3749	0.069307	-0.22924	-0.31445	-0.03442	-0.3144	-0.21463
0.021336	0.095832	1.41E-05	0.438777	0.009529	0.000318	0.700824	0.000318	0.015387
-0.16184	0.378954	-0.06277	-0.21118	-0.0068	-0.05179	-0.22228	0.045489	-0.13724
0.069086	1.12E-05	0.483261	0.017157	0.939519	0.563116	0.012017	0.611576	0.123885
0.369406	0.584826	0.036267	-0.35533	0.255516	0.21907	0.419767	0.100533	0.322975
1.92E-05	5.22E-13	0.685623	4.14E-05	0.00374	0.013342	8.99E-07	0.26076	0.000213
-0.0736	0.082825	-0.35127	0.028551	-0.15671	-0.01435	0.105561	-0.17401	0.240761
0.41089	0.354573	5.14E-05	0.75	0.078507	0.872806	0.237541	0.050398	0.006398
-0.74681	-0.27695	-0.62625	0.386738	-0.66181	-0.53297	-0.59684	-0.47826	-0.55463
6.8E-24	0.001621	3.44E-15	7.08E-06	2.43E-17	1.12E-10	1.31E-13	1.3E-08	1.33E-11
-0.57372	-0.65206	-0.33509	0.427019	-0.44862	-0.3071	-0.49778	-0.27165	-0.45032
1.78E-12	1.01E-16	0.000118	5.54E-07	1.23E-07	0.000445	2.61E-09	0.002006	1.08E-07
-0.66203	-0.47416	-0.53178	0.341807	-0.44292	-0.26436	-0.47881	-0.34493	-0.29079
2.36E-17	1.79E-08	1.25E-10	8.39E-05	1.84E-07	0.00267	1.24E-08	7.15E-05	0.000911
-0.31675	0.066506	-0.35665	0.004163	-0.37404	-0.2676	-0.4296	-0.0884	-0.39356
0.000285	0.457546	3.86E-05	0.962947	1.48E-05	0.002353	4.65E-07	0.32298	4.71E-06

TRAF3IP3	TRAT1	TREM1	TREM2	TREML1	TREML2	TRIM14	TRIM21	TRIM38
brown	brown	brown	brown	brown	brown	brown	brown	brown
0.306512	0.293939	-0.00661	0.27222	0.240239	-0.03072	0.214719	0.223894	0.335814
0.000457	0.000796	0.941234	0.001961	0.006517	0.731669	0.015343	0.011393	0.000114
0.118274	0.133684	-0.03475	0.203684	0.153967	-0.10753	0.041572	0.060788	0.176051
0.185384	0.134033	0.698147	0.021628	0.083937	0.228857	0.642604	0.497197	0.047719
-0.2928	-0.13597	0.127372	-0.31165	-0.22738	0.057878	-0.10968	0.028838	-0.2658
0.000836	0.127442	0.153569	0.000362	0.010144	0.518057	0.219648	0.747574	0.002525
-0.02433	0.105379	0.378848	-0.22022	-0.0246	0.138561	0.057608	0.357803	0.070396
0.785988	0.238355	1.12E-05	0.012852	0.783658	0.120288	0.520012	3.63E-05	0.431601
0.196809	0.189476	0.24938	0.395405	0.056402	0.053727	-0.26581	0.251158	0.095648
0.026573	0.032881	0.004693	4.21E-06	0.528801	0.548557	0.002524	0.004396	0.284754
0.226416	0.332925	0.53673	0.14492	0.11229	0.177149	-0.00594	0.562822	0.284201
0.010477	0.000131	7.79E-11	0.104031	0.208787	0.046325	0.947175	5.7E-12	0.001203
0.131732	0.166608	0.364347	0.084636	0.124841	0.103098	-0.05833	0.400544	0.2015
0.139861	0.061191	2.54E-05	0.344116	0.161976	0.248728	0.514819	3.07E-06	0.023105
0.293624	0.145059	0.196764	0.195056	0.27493	-0.0131	0.241561	0.279487	0.261225
0.000807	0.103697	0.026608	0.027978	0.001759	0.883759	0.006219	0.001462	0.003013
0.262103	0.333759	0.62052	-0.05577	0.069833	0.488187	0.461151	0.482083	0.464539
0.002913	0.000126	7.2E-15	0.533427	0.435305	5.81E-09	4.86E-08	9.54E-09	3.76E-08
0.874696	0.705142	0.551517	0.666363	0.657398	0.464689	0.752074	0.756076	0.867157
3.9E-41	2.17E-20	1.82E-11	1.23E-17	4.67E-17	3.72E-08	2.19E-24	9.1E-25	1.18E-39
0.710768	0.662945	0.357	0.424171	0.501584	0.351754	0.754587	0.857865	0.748728
7.94E-21	2.06E-17	3.79E-05	6.71E-07	1.89E-09	5.01E-05	1.26E-24	5.96E-38	4.52E-24
-0.1259	-0.26814	-0.13599	-0.21837	-0.11056	0.034536	0.021818	-0.20106	-0.1191
0.158427	0.002304	0.127402	0.013647	0.215918	0.699895	0.807636	0.023414	0.182314
-0.20848	-0.27527	-0.20538	-0.28595	-0.09468	0.025628	0.11644	-0.27885	-0.23343
0.018669	0.001735	0.02054	0.001118	0.289677	0.774877	0.192342	0.0015	0.008262
0.006397	-0.09688	-0.16501	0.178923	-0.03245	0.049811	0.000506	-0.15724	-0.1245
0.9431	0.278574	0.063744	0.044146	0.717216	0.57812	0.995498	0.077486	0.163141
0.44427	0.2222	0.087688	0.335142	0.375731	0.273882	0.550388	0.196922	0.400868
1.68E-07	0.012046	0.326932	0.000117	1.34E-05	0.001835	2.04E-11	0.026485	3.01E-06
0.116131	-0.11517	-0.53962	0.360401	0.286677	-0.35484	-0.01726	-0.32009	0.0142
0.193536	0.197254	5.9E-11	3.15E-05	0.001085	4.25E-05	0.847258	0.000244	0.874099
-0.62226	-0.63998	-0.70596	-0.32174	-0.35142	-0.43331	-0.45992	-0.7575	-0.73262
5.76E-15	5.49E-16	1.88E-20	0.000225	5.1E-05	3.61E-07	5.34E-08	6.63E-25	1.26E-22
-0.56661	-0.32967	-0.38644	-0.42712	-0.44919	-0.4149	-0.53779	-0.44909	-0.51667
3.82E-12	0.000154	7.21E-06	5.5E-07	1.18E-07	1.23E-06	7.04E-11	1.18E-07	5.03E-10
-0.53732	-0.46456	-0.58728	-0.29619	-0.41149	-0.41903	-0.51234	-0.69002	-0.5768
7.37E-11	3.76E-08	3.95E-13	0.000722	1.54E-06	9.43E-07	7.4E-10	2.9E-19	1.27E-12
-0.36246	-0.26033	-0.13781	-0.32911	-0.25837	-0.07714	-0.43649	-0.35235	-0.36429
2.82E-05	0.003117	0.122327	0.000158	0.003359	0.388711	2.9E-07	4.85E-05	2.55E-05

TRIM50	TRPM2	TRPV2	TSHZ1	TSPAN32	TSPAN5	TTC16	TTC19	TTC3
brown	brown	brown	brown	brown	brown	brown	brown	brown
0.256027	0.39425	0.155102	-0.24798	0.028113	0.060452	0.116093	-0.02222	-0.02493
0.003669	4.51E-06	0.081652	0.004938	0.75371	0.499583	0.193682	0.804205	0.780835
0.152242	0.314731	0.021663	-0.27871	-0.10502	0.17655	0.025989	0.127955	-0.00929
0.087509	0.000314	0.808982	0.001509	0.239973	0.047081	0.771791	0.151681	0.917472
-0.28313	-0.71495	-0.36083	0.32038	-0.21565	-0.01794	-0.28881	0.168803	0.185399
0.001258	3.7E-21	3.08E-05	0.000241	0.014893	0.841357	0.000991	0.057809	0.036906
-0.20772	-0.67472	-0.26106	-0.02558	-0.04608	-0.04097	-0.12242	0.086222	0.030751
0.019109	3.4E-18	0.003031	0.775286	0.606912	0.647444	0.170349	0.335118	0.731443
-0.11009	-0.0532	0.004742	-0.3702	0.123613	0.096706	-0.081	0.098842	-0.412
0.217885	0.552486	0.957802	1.84E-05	0.166179	0.279439	0.365329	0.268903	1.49E-06
-0.14829	-0.42543	-0.08919	-0.40451	0.136694	0.011505	-0.05693	0.071373	-0.32142
0.096136	6.16E-07	0.318708	2.4E-06	0.125416	0.897852	0.524923	0.42522	0.000229
-0.13472	-0.30776	-0.0214	-0.2886	0.051436	0.047715	0.062727	0.069926	-0.0873
0.131026	0.000432	0.811292	0.001	0.565762	0.594236	0.483554	0.43469	0.329091
-0.05703	0.105951	0.032863	-0.25897	0.26065	-0.25191	0.434655	-0.23804	-0.15921
0.524204	0.235802	0.71378	0.003283	0.00308	0.004276	3.29E-07	0.007041	0.073797
0.316968	-0.13441	0.476189	-0.156	0.203904	-0.53332	0.170049	-0.46641	-0.08795
0.000283	0.131917	1.53E-08	0.079872	0.021484	1.08E-10	0.055959	3.26E-08	0.325497
0.596859	0.559719	0.707455	-0.61206	0.636918	-0.59575	0.519234	-0.68444	-0.51956
1.31E-13	7.87E-12	1.44E-20	2.09E-14	8.33E-16	1.49E-13	3.99E-10	7.24E-19	3.87E-10
0.42753	0.318021	0.443114	-0.47328	0.427313	-0.36806	0.387779	-0.44055	-0.38739
5.35E-07	0.000269	1.82E-07	1.92E-08	5.43E-07	2.07E-05	6.66E-06	2.18E-07	6.81E-06
0.098015	-0.05983	0.00702	0.207491	-0.17923	0.053194	-0.03799	-0.03442	0.245268
0.272948	0.504047	0.937569	0.019247	0.043784	0.552536	0.671567	0.700867	0.005448
0.086539	-0.14033	-0.08421	0.465427	-0.14314	-0.22592	0.020947	-0.18116	0.365912
0.333336	0.115571	0.34653	3.52E-08	0.108404	0.010653	0.815177	0.04152	2.33E-05
0.193141	0.253865	0.186722	0.133009	0.104221	-0.24838	0.054354	-0.32302	-0.25436
0.029585	0.003977	0.035556	0.136027	0.243581	0.004868	0.543899	0.000212	0.003905
0.554783	0.478776	0.501839	-0.07781	0.378374	-0.58484	0.418795	-0.69554	-0.18796
1.31E-11	1.24E-08	1.85E-09	0.384557	1.15E-05	5.21E-13	9.58E-07	1.15E-19	0.034334
0.161133	0.559411	0.075861	-0.07502	0.032874	0.179234	0.171068	-0.00311	0.027325
0.070333	8.12E-12	0.396616	0.401865	0.713689	0.043774	0.054483	0.972285	0.760404
-0.34888	-0.13864	-0.41744	0.636996	-0.50741	0.418379	-0.36614	0.475158	0.44989
5.82E-05	0.120065	1.05E-06	8.24E-16	1.14E-09	9.84E-07	2.3E-05	1.66E-08	1.12E-07
-0.48959	-0.27328	-0.46456	0.279428	-0.4133	0.641098	-0.43997	0.701214	0.27847
5.18E-09	0.001879	3.76E-08	0.001465	1.37E-06	4.71E-16	2.27E-07	4.32E-20	0.001524
-0.33145	0.143939	-0.28372	0.317334	-0.4139	0.551513	-0.31258	0.576927	0.356157
0.000141	0.106422	0.001227	0.000278	1.32E-06	1.82E-11	0.000346	1.26E-12	3.96E-05
-0.25075	-0.47859	-0.35685	0.512068	-0.36842	0.175558	-0.3037	0.32552	0.48528
0.004462	1.26E-08	3.82E-05	7.58E-10	2.03E-05	0.048355	0.000518	0.000188	7.37E-09

TTC7A	TXK	TXNDC11	TYMP	TYROBP	UBASH3A	UBD	UBE2L6	UBE2Z
brown	brown	brown	brown	brown	brown	brown	brown	brown
0.028013	0.170833	0.167584	0.159233	0.329913	0.374128	0.22747	0.33437	0.438424
0.754564	0.05482	0.059668	0.073752	0.000152	1.47E-05	0.010113	0.000122	2.53E-07
-0.03002	0.070196	0.085961	-0.05042	0.216191	0.196759	0.191769	0.231233	0.398911
0.737622	0.432915	0.336588	0.573501	0.014638	0.026613	0.030784	0.008906	3.39E-06
-0.35477	-0.09089	-0.06565	-0.24677	-0.44581	-0.22328	-0.18617	-0.14255	-0.37149
4.27E-05	0.309507	0.463364	0.00516	1.5E-07	0.011628	0.036112	0.109868	1.71E-05
-0.19308	0.060311	0.185491	-0.191	-0.32564	-0.05934	0.030962	-0.17171	-0.22712
0.029641	0.500591	0.03681	0.03147	0.000187	0.507554	0.729675	0.053569	0.010233
-0.20157	-0.1378	0.082358	-0.08794	0.331618	0.162757	0.01346	0.261438	0.177407
0.02306	0.122361	0.357306	0.325558	0.00014	0.067511	0.880612	0.002988	0.046004
-0.16824	0.079124	0.302244	-0.12867	0.060088	0.162293	0.132066	0.049269	0.04516
0.058667	0.376557	0.000553	0.149381	0.502176	0.068307	0.138851	0.582267	0.614157
-0.08329	0.02431	0.295311	-0.20542	0.024366	0.042757	0.20311	0.013781	0.174147
0.351851	0.786171	0.00075	0.020515	0.785683	0.633147	0.022008	0.877787	0.050216
0.278972	0.132386	0.094866	0.206589	0.225998	0.05799	0.126396	-0.00764	0.029171
0.001493	0.137887	0.28873	0.019789	0.010624	0.517242	0.156772	0.93208	0.744756
0.176944	0.451966	0.517289	0.430397	0.207853	0.36114	0.421406	0.200464	0.244158
0.046583	9.61E-08	4.76E-10	4.41E-07	0.019032	3.03E-05	8.06E-07	0.023835	0.005669
0.671142	0.584841	0.565209	0.792802	0.89843	0.795277	0.654736	0.630681	0.516948
5.93E-18	5.21E-13	4.43E-12	1.18E-28	1.66E-46	6.04E-29	6.87E-17	1.92E-15	4.9E-10
0.454148	0.43284	0.462529	0.633848	0.554812	0.656855	0.529164	0.628698	0.252742
8.19E-08	3.73E-07	4.38E-08	1.26E-15	1.3E-11	5.05E-17	1.6E-10	2.49E-15	0.004147
0.05944	-0.05687	0.140448	0.000191	-0.13886	0.002182	0.333675	0.071376	0.045272
0.506806	0.525403	0.11527	0.998299	0.119479	0.980572	0.000126	0.425198	0.613278
0.106128	0.021764	-0.10081	0.133386	-0.22919	-0.12538	-0.04321	-0.02291	-0.21473
0.235017	0.808109	0.259459	0.134913	0.009544	0.160157	0.62952	0.798176	0.015337
0.189393	-0.05701	-0.15089	0.386425	0.26687	0.063661	-0.04938	0.274954	-0.09222
0.032958	0.524347	0.09039	7.21E-06	0.002422	0.477054	0.581438	0.001757	0.302454
0.646693	0.383482	0.219892	0.735501	0.562938	0.448983	0.362669	0.443988	0.195254
2.16E-16	8.58E-06	0.01299	7.06E-23	5.63E-12	1.19E-07	2.79E-05	1.71E-07	0.027816
0.294438	-0.13056	-0.38893	0.006244	0.232897	0.027944	-0.0804	-0.02746	0.163638
0.000779	0.143454	6.22E-06	0.944457	0.008414	0.755146	0.368905	0.75925	0.066021
-0.34245	-0.53582	-0.61204	-0.45042	-0.54007	-0.60685	-0.51254	-0.3722	-0.45425
8.12E-05	8.5E-11	2.09E-14	1.08E-07	5.65E-11	3.96E-14	7.27E-10	1.64E-05	8.13E-08
-0.57784	-0.40009	-0.41854	-0.69696	-0.65546	-0.55484	-0.49273	-0.56068	-0.3277
1.14E-12	3.16E-06	9.74E-07	8.99E-20	6.19E-17	1.3E-11	3.99E-09	7.12E-12	0.000169
-0.40091	-0.43155	-0.48152	-0.52384	-0.4222	-0.51519	-0.43109	-0.39029	-0.12967
3E-06	4.07E-07	9.98E-09	2.62E-10	7.65E-07	5.74E-10	4.2E-07	5.73E-06	0.146233
-0.53837	-0.24339	-0.1829	-0.41496	-0.41318	-0.25459	-0.30248	-0.16189	-0.12199
6.66E-11	0.005827	0.039571	1.23E-06	1.38E-06	0.003871	0.000547	0.069007	0.17185

UCP2	UGCG	UNC13D	UNC93B1	UPP1	VAMP3	VAMP5	VAMP8	VAV1
brown	brown	brown	brown	brown	brown	brown	brown	brown
0.186657	0.203827	0.055502	0.120463	0.06687	0.212367	0.084304	0.226154	0.28216
0.035621	0.021534	0.53541	0.177323	0.455081	0.01653	0.346018	0.010569	0.001309
0.030542	0.148653	-0.05568	-0.04179	0.042421	0.177998	0.063431	0.101574	0.091229
0.733206	0.095325	0.534134	0.640873	0.635821	0.045272	0.478651	0.255827	0.307701
-0.17323	-0.0371	-0.12506	-0.34445	-0.04577	-0.24143	-0.26885	-0.29757	-0.35603
0.051459	0.678776	0.161253	7.33E-05	0.609411	0.006249	0.002241	0.00068	3.99E-05
-0.23747	0.384583	-0.14075	-0.29814	0.188204	0.186728	-0.07898	-0.11097	-0.18536
0.007182	8.04E-06	0.114496	0.000663	0.034095	0.03555	0.377438	0.214211	0.036946
-0.06703	0.223014	-0.08539	-0.08369	0.463241	0.171127	0.595981	0.4058	-0.01609
0.453994	0.011728	0.339834	0.349535	4.15E-08	0.054398	1.45E-13	2.21E-06	0.85753
-0.20324	0.62095	-0.18082	-0.2227	0.51743	0.441692	0.39577	0.274481	-0.03284
0.021921	6.81E-15	0.041909	0.01185	4.7E-10	2.01E-07	4.11E-06	0.001791	0.714008
-0.20334	0.405273	-0.14993	-0.17854	0.372443	0.325518	0.267106	0.178832	-0.0599
0.021853	2.28E-06	0.092479	0.044612	1.62E-05	0.000188	0.0024	0.044256	0.503551
0.046285	0.258058	-0.01886	0.076865	0.140318	0.45794	0.202856	0.234513	0.215722
0.605344	0.003398	0.833348	0.390377	0.115612	6.19E-08	0.022178	0.007959	0.01486
0.268877	0.419718	0.348046	0.325537	0.438992	0.289734	0.269718	0.357687	0.310359
0.002238	9.02E-07	6.08E-05	0.000188	2.43E-07	0.000953	0.002165	3.65E-05	0.000384
0.721385	0.554759	0.65597	0.764774	0.589068	0.735791	0.701328	0.912881	0.900526
1.11E-21	1.31E-11	5.75E-17	1.27E-25	3.22E-13	6.66E-23	4.24E-20	1.8E-50	4.83E-47
0.477801	0.525589	0.410391	0.589185	0.411824	0.590454	0.460904	0.5828	0.665918
1.34E-08	2.23E-10	1.65E-06	3.18E-13	1.51E-06	2.75E-13	4.96E-08	6.55E-13	1.32E-17
0.071858	-0.35166	0.170537	0.121976	-0.14064	-0.21138	-0.17904	-0.16954	-0.03926
0.422075	5.03E-05	0.055248	0.171902	0.114761	0.017049	0.044004	0.056714	0.661239
0.232837	-0.47321	0.34499	0.152318	-0.34755	-0.36504	-0.44663	-0.29806	-0.03555
0.008431	1.93E-08	7.13E-05	0.087348	6.24E-05	2.45E-05	1.41E-07	0.000665	0.691502
0.371515	-0.38599	0.489127	0.473214	0.010684	-0.24881	0.181687	0.151069	0.135596
1.71E-05	7.4E-06	5.38E-09	1.93E-08	0.905101	0.004792	0.040923	0.090004	0.12851
0.762729	-0.12345	0.802444	0.789763	0.118222	0.208046	0.232684	0.450004	0.629958
2.03E-25	0.166745	8.29E-30	2.64E-28	0.18558	0.018919	0.008475	1.11E-07	2.11E-15
0.28214	-0.43199	0.074697	0.177841	-0.39966	0.083769	-0.11477	0.022525	0.196821
0.001311	3.95E-07	0.403925	0.045465	3.24E-06	0.349095	0.198824	0.801527	0.026564
-0.21596	-0.76753	-0.31863	-0.35967	-0.7007	-0.69563	-0.63231	-0.6676	-0.49478
0.014748	6.67E-26	0.000261	3.28E-05	4.73E-20	1.13E-19	1.55E-15	1.02E-17	3.36E-09
-0.72901	-0.07472	-0.79265	-0.70257	-0.40874	-0.39893	-0.45636	-0.62837	-0.63709
2.57E-22	0.40379	1.23E-28	3.41E-20	1.83E-06	3.39E-06	6.96E-08	2.6E-15	8.14E-16
-0.49541	-0.39844	-0.59117	-0.45971	-0.52093	-0.53367	-0.43307	-0.54446	-0.47523
3.19E-09	3.5E-06	2.53E-13	5.42E-08	3.42E-10	1.04E-10	3.67E-07	3.68E-11	1.65E-08
-0.17697	-0.39164	-0.13688	-0.46025	-0.28805	-0.55464	-0.42903	-0.36593	-0.39497
0.046555	5.28E-06	0.124887	5.2E-08	0.001024	1.33E-11	4.84E-07	2.33E-05	4.32E-06

VENTX	VNN1	VNN2	VPS37D	VRK2	VSIG4	VWA5A	WAS	WBP1L
brown	brown	brown	brown	brown	brown	brown	brown	brown
0.181107	0.217942	0.327195	-0.34306	0.418726	0.138675	0.19575	0.161998	0.148943
0.041583	0.013837	0.000174	7.87E-05	9.62E-07	0.11998	0.027415	0.068817	0.094673
0.096574	0.157917	0.265327	-0.23186	0.366867	0.138981	0.135513	-0.01212	0.11609
0.280095	0.076199	0.002572	0.008719	2.21E-05	0.119156	0.128746	0.892388	0.193692
-0.17161	-0.02085	-0.21936	0.38338	-0.45312	-0.27064	-0.19868	-0.30033	-0.41751
0.053715	0.815983	0.013217	8.63E-06	8.83E-08	0.002088	0.025142	0.000602	1.04E-06
0.119063	0.234587	0.086193	-0.03335	-0.10946	-0.09338	0.260619	-0.23769	-0.33836
0.182448	0.007938	0.33528	0.709754	0.220581	0.296391	0.003083	0.007127	9.99E-05
0.04745	0.287407	0.39921	0.279785	0.136888	0.236364	0.147875	-0.10002	0.149872
0.596288	0.001052	3.33E-06	0.001444	0.124874	0.007465	0.097091	0.263232	0.09261
0.247703	0.486491	0.45557	-0.06624	0.214422	0.189059	0.464479	-0.16814	-0.06298
0.004989	6.68E-09	7.38E-08	0.459323	0.015489	0.033274	3.78E-08	0.058817	0.481804
0.22081	0.319401	0.373499	-0.13027	0.16781	0.160411	0.379226	-0.12317	-0.02123
0.012607	0.000252	1.52E-05	0.144362	0.05932	0.071616	1.1E-05	0.167708	0.812722
0.286869	0.101349	0.132858	-0.50197	0.281479	0.184872	0.483071	0.117495	0.049966
0.001076	0.25689	0.136478	1.83E-09	0.001347	0.037454	8.81E-09	0.18832	0.576936
0.480076	0.354388	0.330535	-0.1757	0.119312	0.369088	0.270031	0.362281	0.206462
1.12E-08	4.36E-05	0.000147	0.048176	0.181529	1.95E-05	0.002139	2.85E-05	0.019867
0.709159	0.686017	0.741383	-0.53873	0.689934	0.793377	0.635859	0.84169	0.685255
1.06E-20	5.6E-19	2.12E-23	6.43E-11	2.94E-19	1.01E-28	9.61E-16	2.96E-35	6.34E-19
0.428744	0.435757	0.459957	-0.50904	0.505156	0.401897	0.560698	0.563985	0.361947
4.93E-07	3.05E-07	5.32E-08	9.91E-10	1.39E-09	2.82E-06	7.11E-12	5.04E-12	2.9E-05
-0.00164	-0.28967	-0.23423	0.135922	-0.23935	-0.10655	-0.09213	0.062539	-0.02503
0.985357	0.000956	0.008037	0.127584	0.006724	0.233134	0.3029	0.484866	0.780014
-0.11405	-0.30413	-0.41649	0.187973	-0.39825	-0.21092	-0.37952	0.154313	-0.1734
0.201694	0.000509	1.11E-06	0.034319	3.54E-06	0.0173	1.08E-05	0.083235	0.051221
-0.10904	-0.2088	-0.21212	0.49373	-0.22535	0.047158	-0.38895	0.293315	0.180705
0.22235	0.018483	0.016658	3.67E-09	0.010855	0.598554	6.21E-06	0.000818	0.042045
0.336737	0.14042	0.132722	-0.14664	0.217536	0.409439	0.019492	0.756397	0.458815
0.000108	0.115344	0.136882	0.099944	0.01402	1.76E-06	0.827808	8.47E-25	5.8E-08
-0.19916	-0.13057	-0.0455	-0.22641	0.389725	0.104294	-0.12663	0.137144	0.24263
0.024786	0.143413	0.611516	0.010479	5.93E-06	0.24325	0.155992	0.124164	0.005987
-0.70304	-0.66332	-0.68455	0.439511	-0.58129	-0.54704	-0.67967	-0.41345	-0.38304
3.14E-20	1.94E-17	7.11E-19	2.35E-07	7.76E-13	2.85E-11	1.56E-18	1.36E-06	8.8E-06
-0.48953	-0.3341	-0.37651	0.154307	-0.28825	-0.55785	-0.23576	-0.73695	-0.44323
5.2E-09	0.000124	1.28E-05	0.083247	0.001015	9.54E-12	0.007624	5.27E-23	1.8E-07
-0.53308	-0.53325	-0.43997	0.146754	-0.27936	-0.4479	-0.40238	-0.49593	-0.22986
1.1E-10	1.09E-10	2.27E-07	0.099679	0.001469	1.29E-07	2.74E-06	3.06E-09	0.009332
-0.22155	-0.12844	-0.17012	0.422085	-0.36884	-0.29865	-0.45324	-0.30232	-0.41315
0.012306	0.150133	0.055851	7.71E-07	1.98E-05	0.000648	8.76E-08	0.000551	1.38E-06

WDFY4	WDR82	WIPF1	WIPI1	WNT3	WNT7A	WRNIP1	XCL1	XCL2
brown	brown	brown	brown	brown	brown	brown	brown	brown
0.218093	-0.26657	0.22949	0.141314	-0.35278	-0.07334	-0.20974	0.360763	0.377632
0.01377	0.00245	0.009448	0.113025	4.74E-05	0.412537	0.01795	3.09E-05	1.2E-05
0.047258	-0.28151	0.094355	0.086585	-0.27613	0.030107	-0.11857	0.177369	0.213984
0.597778	0.001345	0.291346	0.333078	0.001676	0.736863	0.184291	0.046051	0.015705
-0.33027	0.480513	-0.07449	-0.14033	0.540862	0.133436	0.163206	-0.14015	-0.09834
0.000149	1.08E-08	0.405245	0.115593	5.23E-11	0.134764	0.066748	0.116044	0.271363
-0.17201	0.367822	0.190594	0.203814	0.379968	0.051427	0.042811	0.044907	0.083684
0.05315	2.1E-05	0.031844	0.021543	1.05E-05	0.565833	0.632718	0.616144	0.349586
-0.30085	-0.52108	0.025366	-0.1232	-0.07393	0.036057	0.224275	0.374037	0.322745
0.000588	3.37E-10	0.777119	0.167624	0.408758	0.687353	0.01125	1.48E-05	0.000215
-0.203	-0.1176	0.288163	0.231805	0.108037	-0.00055	0.109421	0.347459	0.347679
0.022081	0.187939	0.001019	0.008734	0.226656	0.995121	0.220727	6.27E-05	6.2E-05
-0.11945	-0.00512	0.158455	0.343862	0.05004	0.049463	0.103705	0.273232	0.258422
0.181025	0.954457	0.07519	7.55E-05	0.576367	0.580782	0.245937	0.001883	0.003352
0.204908	-0.18898	0.149568	0.031132	0.069118	-0.31754	-0.04163	0.116604	0.165061
0.020836	0.033351	0.093283	0.728252	0.440029	0.000275	0.642168	0.191714	0.063668
0.274002	0.15998	0.506115	0.699839	-0.123	-0.21058	-0.22786	0.155331	0.227828
0.001826	0.072391	1.28E-09	5.49E-20	0.168289	0.017484	0.009981	0.081197	0.009993
0.76078	-0.52716	0.806177	0.649434	-0.52947	-0.4488	-0.46494	0.50659	0.607688
3.16E-25	1.93E-10	2.85E-30	1.47E-16	1.55E-10	1.21E-07	3.65E-08	1.23E-09	3.57E-14
0.601838	-0.24039	0.619019	0.522484	-0.35184	-0.19215	-0.36772	0.507459	0.617627
7.24E-14	0.006483	8.71E-15	2.97E-10	4.98E-05	0.030444	2.11E-05	1.14E-09	1.04E-14
0.084144	0.242612	-0.10135	0.164006	-0.18245	0.053345	-0.12872	-0.24943	-0.25574
0.346934	0.005991	0.256872	0.065405	0.040071	0.551413	0.149241	0.004684	0.003708
0.14458	0.393955	-0.07742	-0.04894	0.249413	-0.01679	-0.13289	-0.34974	-0.25856
0.104856	4.6E-06	0.386938	0.584801	0.004687	0.851352	0.136388	5.57E-05	0.003335
0.066287	-0.29817	-0.06411	-0.18612	-0.00967	0.027084	-0.02731	-0.09969	-0.07373
0.459032	0.000662	0.473914	0.036164	0.914097	0.762455	0.76052	0.264812	0.410063
0.644944	-0.26751	0.457784	0.33102	-0.28878	-0.31407	-0.39334	0.045982	0.1559
2.76E-16	0.002362	6.26E-08	0.000144	0.000992	0.000323	4.77E-06	0.607711	0.080075
0.24355	-0.35289	0.042863	-0.25563	-0.39211	-0.08993	-0.07817	-0.11585	-0.13528
0.005794	4.72E-05	0.632307	0.003723	5.14E-06	0.314678	0.382358	0.19461	0.129412
-0.35463	0.38382	-0.63008	-0.69081	0.280075	0.33578	0.216378	-0.48021	-0.53291
4.3E-05	8.41E-06	2.08E-15	2.54E-19	0.001427	0.000114	0.014551	1.11E-08	1.12E-10
-0.55647	0.358487	-0.56983	-0.43785	0.246982	0.348872	0.358925	-0.21062	-0.35779
1.1E-11	3.5E-05	2.71E-12	2.63E-07	0.005121	5.83E-05	3.42E-05	0.017461	3.63E-05
-0.38235	0.166206	-0.69637	-0.54181	0.033931	0.264215	0.261485	-0.27682	-0.41021
9.16E-06	0.061827	9.95E-20	4.77E-11	0.704905	0.002685	0.002983	0.001629	1.67E-06
-0.40692	0.381079	-0.23325	-0.35361	0.266673	-0.03672	0.197463	-0.14994	-0.15255
2.06E-06	9.87E-06	0.008311	4.54E-05	0.002441	0.68193	0.026064	0.092451	0.08687

YPEL1	YWHAE	YWHAQ	ZADH2	ZAP70	ZBED9	ZBTB32	ZC3H12A	ZC3H12D
brown	brown	brown	brown	brown	brown	brown	brown	brown
-0.07255	-0.29879	-0.05652	-0.1535	0.139948	-0.19486	0.176682	0.094473	0.327228
0.417622	0.000644	0.527969	0.084892	0.116585	0.028143	0.046913	0.290741	0.000173
0.013214	-0.201	0.040855	-0.09906	0.009389	-0.16011	0.05946	0.029677	0.202333
0.882781	0.023455	0.648353	0.267838	0.916558	0.072158	0.506662	0.740483	0.022532
0.009261	0.305029	0.386208	0.299282	-0.12062	0.325223	-0.09015	0.011203	-0.3272
0.917697	0.000488	7.31E-06	0.000631	0.17675	0.000191	0.313498	0.900514	0.000173
-0.34531	0.108993	0.229435	0.202494	-0.12921	0.393332	0.023312	0.151198	-0.10952
7.01E-05	0.222547	0.009466	0.022422	0.147663	4.77E-06	0.794746	0.089727	0.220307
-0.18371	-0.23358	0.027226	-0.06111	0.014807	-0.20283	0.16521	-0.00633	0.045454
0.038695	0.008219	0.761247	0.494953	0.868768	0.022195	0.063427	0.943665	0.611845
-0.51613	-0.17704	0.123667	0.037251	-0.07342	0.134234	0.18694	0.173305	0.081635
5.28E-10	0.04646	0.165992	0.677563	0.412011	0.132426	0.035337	0.051355	0.361556
-0.36091	-0.17727	-0.06563	0.105971	-0.04665	0.013558	0.108833	0.208707	0.026247
3.07E-05	0.046171	0.463492	0.235715	0.602476	0.879747	0.223231	0.018536	0.769585
-0.38702	-0.1785	-0.16671	-0.16669	-0.11543	-0.1654	0.052601	0.03616	0.197798
6.96E-06	0.044654	0.061023	0.061067	0.196264	0.063118	0.556984	0.686502	0.025807
-0.4482	-0.34163	-0.36469	-0.32563	0.425661	0.094139	0.275745	0.57432	0.315517
1.26E-07	8.46E-05	2.49E-05	0.000187	6.07E-07	0.292461	0.001702	1.67E-12	0.000302
-0.75953	-0.69766	-0.57704	-0.67063	0.652634	-0.43975	0.505048	0.580713	0.808686
4.2E-25	7.99E-20	1.24E-12	6.42E-18	9.3E-17	2.31E-07	1.4E-09	8.27E-13	1.37E-30
-0.64583	-0.43771	-0.33624	-0.4384	0.430695	-0.17452	0.38854	0.428272	0.577695
2.44E-16	2.66E-07	0.000111	2.54E-07	4.32E-07	0.049712	6.36E-06	5.09E-07	1.15E-12
0.393188	0.195546	-0.05385	-0.0357	0.050413	0.309091	-0.23191	0.087772	-0.06104
4.81E-06	0.02758	0.547653	0.690264	0.573528	0.000407	0.008702	0.326466	0.495403
0.241472	0.20833	-0.03472	0.017894	0.188602	0.07403	-0.13374	0.086588	-0.13947
0.006239	0.018753	0.698361	0.841732	0.03371	0.408143	0.133876	0.333063	0.11785
0.114362	-0.00763	-0.16746	-0.34099	0.346236	-0.20217	0.014296	0.052253	0.036008
0.200455	0.93216	0.059851	8.75E-05	6.68E-05	0.022644	0.873261	0.5596	0.687754
-0.27105	-0.33331	-0.47382	-0.59028	0.658101	-0.38173	0.212769	0.396284	0.465601
0.002054	0.000129	1.84E-08	2.81E-13	4.21E-17	9.5E-06	0.016321	3.99E-06	3.47E-08
0.223005	0.079157	0.081665	-0.15743	0.04984	-0.37025	-0.206	-0.39233	0.095361
0.011732	0.376362	0.361379	0.077115	0.577896	1.83E-05	0.020153	5.07E-06	0.28621
0.757957	0.67273	0.459743	0.422718	-0.39258	0.143299	-0.45205	-0.5603	-0.60138
5.98E-25	4.64E-18	5.41E-08	7.39E-07	4.99E-06	0.108001	9.55E-08	7.41E-12	7.65E-14
0.4653	0.481365	0.516939	0.598932	-0.67458	0.41866	-0.32351	-0.55137	-0.49431
3.55E-08	1.01E-08	4.91E-10	1.02E-13	3.48E-18	9.67E-07	0.000207	1.85E-11	3.5E-09
0.699515	0.380479	0.271708	0.443039	-0.55082	0.098241	-0.32032	-0.55975	-0.43853
5.8E-20	1.02E-05	0.002001	1.83E-07	1.95E-11	0.271839	0.000241	7.84E-12	2.51E-07
0.462542	0.19741	0.294235	0.527374	-0.07915	0.102309	-0.13345	-0.1221	-0.33195
4.38E-08	0.026105	0.000786	1.89E-10	0.376391	0.252388	0.134735	0.171466	0.000138

ZDHHC2	ZFP14	ZFP36	ZFP69B	ZMYND11	ZMYND15	ZNF280B	ZNF346	ZNF382
brown	brown	brown	brown	brown	brown	brown	brown	brown
0.326463	-0.08813	0.017871	-0.25044	0.023659	0.169214	-0.03036	-0.22295	0.087876
0.00018	0.324493	0.84193	0.004514	0.79176	0.057194	0.734709	0.011753	0.325894
0.277009	-0.01956	-0.05335	-0.19898	0.101877	0.049544	0.000366	-0.22081	0.165652
0.001617	0.827245	0.551337	0.024917	0.254404	0.580161	0.996745	0.012607	0.062712
-0.39801	0.307233	-0.29154	0.533131	-0.01618	-0.29184	0.199722	0.454489	0.197804
3.59E-06	0.000442	0.000882	1.1E-10	0.856696	0.000871	0.024371	7.99E-08	0.025803
-0.09088	0.264897	-0.17593	0.228734	-0.23507	-0.11738	0.239869	0.153586	0.159873
0.309549	0.002615	0.047877	0.009693	0.007808	0.188765	0.006603	0.084715	0.072584
0.098509	-0.11263	-0.23827	-0.16402	-0.23701	0.039674	-0.3061	-0.13369	-0.1331
0.27053	0.207416	0.006984	0.065387	0.007298	0.657871	0.000465	0.134004	0.135772
0.177268	0.03646	-0.19984	-0.06461	-0.40136	0.003413	-0.02734	-0.13269	-0.00619
0.046176	0.684037	0.024288	0.470518	2.92E-06	0.969619	0.760267	0.136968	0.944916
0.092459	0.020541	-0.13695	-0.12119	-0.33095	0.043064	0.05323	-0.17394	-0.00197
0.301197	0.818695	0.124708	0.17471	0.000144	0.630711	0.552273	0.050492	0.982465
0.238047	-0.03886	0.093728	-0.28552	-0.22234	0.240591	-0.10344	-0.27855	-0.14482
0.007039	0.664474	0.294581	0.001139	0.011993	0.006437	0.24717	0.001519	0.104282
-0.20199	-0.38124	0.38465	-0.03217	-0.61565	0.154762	-0.22862	-0.18934	-0.2333
0.022768	9.77E-06	8.01E-06	0.719597	1.33E-14	0.08233	0.00973	0.03301	0.008297
0.384141	-0.5574	0.546754	-0.60988	-0.58375	0.725409	-0.58384	-0.62443	-0.54727
8.25E-06	1E-11	2.93E-11	2.73E-14	5.89E-13	5.16E-22	5.83E-13	4.35E-15	2.78E-11
0.304943	-0.32461	0.304688	-0.4172	-0.50738	0.514034	-0.24018	-0.39275	-0.3166
0.00049	0.000197	0.000496	1.06E-06	1.14E-09	6.37E-10	0.006532	4.94E-06	0.000287
-0.3786	-0.07238	0.116507	0.022504	-0.00291	-0.00804	0.216878	0.141312	-0.11049
1.14E-05	0.418708	0.192087	0.801715	0.974071	0.928496	0.01432	0.113028	0.216219
-0.50687	0.128138	0.113928	0.310264	0.16942	0.001163	0.072748	0.339382	0.048539
1.2E-09	0.15109	0.202181	0.000385	0.056887	0.989647	0.416332	9.49E-05	0.587878
-0.36842	-0.1846	0.139882	-0.06349	-0.01579	0.170531	-0.42175	0.030426	-0.28174
2.03E-05	0.037739	0.116758	0.47826	0.860103	0.055256	7.88E-07	0.734183	0.001333
-0.14257	-0.38415	0.505225	-0.3005	-0.22272	0.543144	-0.50716	-0.27632	-0.44912
0.109833	8.25E-06	1.38E-09	0.000598	0.011841	4.18E-11	1.17E-09	0.001663	1.18E-07
0.366376	0.119154	0.020081	-0.28509	0.470052	0.185477	-0.11229	-0.22468	-0.08914
2.27E-05	0.182112	0.82269	0.001159	2.47E-08	0.036825	0.208798	0.011102	0.318942
-0.24485	0.407857	-0.26205	0.444876	0.670151	-0.40897	0.347795	0.495436	0.233212
0.005531	1.94E-06	0.002919	1.6E-07	6.91E-18	1.81E-06	6.16E-05	3.18E-09	0.008323
0.17995	0.428867	-0.43778	0.297832	0.464199	-0.55523	0.575791	0.302216	0.458707
0.042925	4.89E-07	2.65E-07	0.000672	3.86E-08	1.25E-11	1.42E-12	0.000554	5.84E-08
0.126769	0.211009	-0.24965	0.190819	0.558778	-0.4539	0.386755	0.254422	0.345938
0.155542	0.017251	0.004646	0.031639	8.67E-12	8.34E-08	7.07E-06	0.003896	6.79E-05
-0.38028	0.365642	-0.40242	0.603982	0.361769	-0.31027	0.301782	0.521517	0.319542
1.03E-05	2.37E-05	2.73E-06	5.6E-14	2.93E-05	0.000385	0.000565	3.24E-10	0.00025

ZNF420	ZNF425	ZNF48	ZNF506	ZNF519	ZNF529	ZNF569	ZNF675	ZNF683
brown	brown	brown	brown	brown	brown	brown	brown	brown
-0.17843	-0.38721	-0.21023	-0.07881	-0.27602	-0.02827	-0.1518	-0.22555	0.342141
0.04474	6.88E-06	0.017676	0.378452	0.001683	0.752396	0.088448	0.010785	8.25E-05
-0.12503	-0.30915	-0.11315	-0.0303	-0.21418	0.039126	-0.09385	-0.20522	0.160668
0.161343	0.000405	0.205303	0.735229	0.01561	0.662303	0.293943	0.020641	0.071156
0.524707	0.47591	0.258252	0.423733	0.599401	0.194131	0.498916	0.494906	-0.09377
2.42E-10	1.56E-08	0.003374	6.91E-07	9.68E-14	0.028746	2.37E-09	3.33E-09	0.294371
0.570966	0.399537	-0.22039	0.469659	0.426096	0.336677	0.540559	0.470948	-0.02355
2.4E-12	3.27E-06	0.012782	2.54E-08	5.9E-07	0.000109	5.38E-11	2.3E-08	0.792672
0.109894	-0.05672	0.16445	-0.14775	0.031901	-0.10145	0.020408	0.106955	-0.00638
0.218726	0.526501	0.06467	0.097372	0.721813	0.256427	0.819848	0.231367	0.94326
0.456538	0.130837	-0.27758	0.241389	0.227603	0.173268	0.391126	0.365613	0.035484
6.87E-08	0.142596	0.00158	0.006258	0.010068	0.051405	5.45E-06	2.37E-05	0.692063
0.30574	0.107487	-0.17955	0.136075	0.027865	0.07603	0.139828	0.143815	-0.07199
0.000473	0.229041	0.043396	0.127153	0.755819	0.395562	0.116902	0.106725	0.421228
0.012121	-0.02208	-0.39441	-0.29673	-0.28589	0.029899	0.125907	-0.30143	0.02297
0.892417	0.805414	4.47E-06	0.000705	0.001121	0.738617	0.158394	0.000573	0.797691
-0.08801	-0.0877	-0.36741	0.064224	-0.2527	-0.3033	-0.16719	-0.12163	0.353812
0.325153	0.326867	2.15E-05	0.473155	0.004153	0.000528	0.060284	0.173122	4.49E-05
-0.4717	-0.533	-0.65799	-0.41326	-0.72412	-0.53433	-0.46797	-0.49612	0.621929
2.17E-08	1.11E-10	4.28E-17	1.37E-06	6.62E-22	9.81E-11	2.9E-08	3.01E-09	6.01E-15
-0.22895	-0.30398	-0.62403	-0.19691	-0.47798	-0.31339	-0.172	-0.29424	0.596092
0.009622	0.000512	4.58E-15	0.026496	1.32E-08	0.000334	0.053165	0.000786	1.43E-13
-0.35325	0.089556	0.080308	0.0073	-0.19208	-0.1009	-0.38251	-0.24966	0.016642
4.63E-05	0.316693	0.369436	0.935079	0.030505	0.259012	9.08E-06	0.004645	0.852677
-0.11184	0.21799	0.2196	0.081595	0.037787	-0.07941	-0.05434	-0.0868	0.060636
0.210646	0.013816	0.013114	0.361793	0.673184	0.374838	0.543977	0.331865	0.498277
-0.38245	0.002952	0.37794	-0.36086	-0.22337	-0.40829	-0.27357	-0.22126	0.104883
9.11E-06	0.973721	1.18E-05	3.08E-05	0.011593	1.89E-06	0.001858	0.012425	0.240584
-0.63459	-0.3112	-0.21979	-0.41987	-0.63262	-0.57377	-0.52775	-0.56927	0.460644
1.14E-15	0.000369	0.013033	8.93E-07	1.49E-15	1.77E-12	1.82E-10	2.88E-12	5.05E-08
-0.44386	-0.37808	0.000918	-0.2825	-0.2939	-0.06778	-0.32134	-0.3882	-0.00299
1.72E-07	1.17E-05	0.991831	0.001291	0.000797	0.448944	0.00023	6.49E-06	0.973386
0.085337	0.268805	0.624669	0.066669	0.406286	0.232309	0.100039	0.1658	-0.4189
0.340117	0.002245	4.22E-15	0.456442	2.14E-06	0.008585	0.263122	0.062474	9.51E-07
0.486546	0.256512	0.277247	0.373416	0.623245	0.57985	0.433832	0.521752	-0.49044
6.65E-09	0.003602	0.001602	1.53E-05	5.07E-15	9.1E-13	3.48E-07	3.17E-10	4.83E-09
0.070644	0.021024	0.440076	0.022743	0.2469	0.293495	0.019736	0.101674	-0.46385
0.429976	0.814513	2.25E-07	0.799647	0.005136	0.000811	0.825691	0.255358	3.97E-08
0.2763	0.184222	0.524609	0.399093	0.585008	0.271212	0.136866	0.361085	-0.16868
0.001664	0.038142	2.44E-10	3.36E-06	5.11E-13	0.002041	0.124936	3.04E-05	0.057994

ZNF704	ZNF714	ZNF737	ZNF774	ZNF793	ZNF853	ZNF91	ZNRF1	ZNRF2
brown	brown	brown	brown	brown	brown	brown	brown	brown
-0.18018	-0.48582	-0.21156	-0.10128	-0.23113	-0.36917	-0.22392	-0.13166	0.077304
0.042656	7.05E-09	0.016953	0.257217	0.008937	1.95E-05	0.011383	0.140067	0.38767
-0.14365	-0.46581	-0.15442	-0.08517	-0.26668	-0.30276	-0.25154	-0.04559	-0.05463
0.107136	3.42E-08	0.083023	0.341054	0.00244	0.000541	0.004336	0.610752	0.541886
0.375289	0.587798	0.398217	0.409592	0.475695	0.487905	0.381023	0.082886	-0.03994
1.38E-05	3.73E-13	3.54E-06	1.74E-06	1.59E-08	5.95E-09	9.9E-06	0.354217	0.655697
0.176806	0.364794	0.270542	0.337329	0.47968	0.103422	0.357712	-0.39929	0.328086
0.046757	2.48E-05	0.002096	0.000105	1.16E-08	0.247236	3.65E-05	3.32E-06	0.000166
-0.25705	-0.32867	-0.28043	-0.11267	-0.51913	-0.17356	-0.14438	-0.02242	0.10637
0.00353	0.000162	0.001406	0.207252	4.03E-10	0.051001	0.105354	0.802461	0.233945
-0.17167	-0.0904	-0.07028	0.111705	-0.02679	-0.26228	0.119698	-0.47881	0.500047
0.053627	0.31214	0.432386	0.21118	0.764957	0.002893	0.180109	1.24E-08	2.16E-09
-0.15935	-0.21904	-0.09902	-0.02899	-0.14607	-0.17036	-0.01843	-0.26449	0.084486
0.07353	0.013353	0.268045	0.746315	0.101292	0.055511	0.837059	0.002656	0.344975
-0.10779	-0.28443	-0.25341	-0.07076	-0.16631	-0.35858	-0.11748	-0.45605	0.313956
0.227721	0.001192	0.004044	0.4292	0.061657	3.48E-05	0.188375	7.12E-08	0.000325
-0.32696	0.000794	-0.19927	-0.28658	0.040004	-0.14939	-0.20593	-0.27273	0.26767
0.000176	0.992934	0.024704	0.001089	0.655207	0.093674	0.020194	0.001921	0.002347
-0.6167	-0.59773	-0.70497	-0.51699	-0.51407	-0.57751	-0.4852	-0.57118	0.590667
1.17E-14	1.18E-13	2.24E-20	4.88E-10	6.35E-10	1.18E-12	7.41E-09	2.35E-12	2.68E-13
-0.39596	-0.37915	-0.47667	-0.20759	-0.14213	-0.39131	-0.30935	-0.6056	0.601717
4.07E-06	1.1E-05	1.47E-08	0.019185	0.11093	5.39E-06	0.000402	4.6E-14	7.34E-14
0.110186	0.198812	0.137069	-0.23463	0.179954	0.232506	-0.36688	0.336756	-0.30951
0.217494	0.025041	0.124372	0.007927	0.04292	0.008527	2.21E-05	0.000108	0.000399
0.317078	0.430628	0.234877	0.106263	0.375261	0.524048	0.066014	0.304711	-0.38088
0.000281	4.34E-07	0.00786	0.23442	1.38E-05	2.57E-10	0.460883	0.000495	9.99E-06
-0.08248	0.096758	-0.17018	-0.1782	-0.18638	0.359252	-0.20012	0.326095	-0.19018
0.356574	0.279179	0.055767	0.045027	0.035897	3.36E-05	0.024083	0.000183	0.032226
-0.26985	-0.19177	-0.41623	-0.40318	-0.23124	-0.00447	-0.43011	-0.04736	0.052262
0.002154	0.030782	1.13E-06	2.61E-06	0.008905	0.960243	4.49E-07	0.596996	0.559531
0.117864	-0.34067	-0.07206	-0.20553	-0.27925	-0.10142	-0.23595	0.149645	-0.2447
0.186923	8.89E-05	0.420747	0.020445	0.001476	0.256534	0.007572	0.093112	0.005561
0.623471	0.431609	0.465991	0.290823	0.287262	0.651196	0.249108	0.636158	-0.64012
4.93E-15	4.06E-07	3.37E-08	0.00091	0.001058	1.14E-16	0.00474	9.23E-16	5.38E-16
0.374984	0.33404	0.531697	0.440244	0.365857	0.085391	0.509746	0.143	-0.12166
1.4E-05	0.000124	1.26E-10	2.23E-07	2.34E-05	0.33981	9.31E-10	0.108747	0.173012
0.263857	0.068539	0.300878	0.167795	0.024862	0.103944	0.183488	0.471201	-0.47463
0.002723	0.443878	0.000588	0.059343	0.781428	0.244847	0.03893	2.26E-08	1.73E-08
0.445327	0.3546	0.490072	0.252457	0.162864	0.305427	0.27638	0.512966	-0.5243
1.55E-07	4.31E-05	4.98E-09	0.004191	0.067329	0.00048	0.001659	7E-10	2.51E-10

ZNRF3	ZXDB	AAED1	AASDH	ABCA3	ABCC8	ABCF3	ABHD13	ABHD3
brown	brown	cyan	cyan	cyan	cyan	cyan	cyan	cyan
-0.15021	0.016499	0.095756	-0.03428	-0.14051	0.18728	0.053053	-0.08894	-0.13705
0.091875	0.853929	0.284212	0.70205	0.115102	0.035	0.553594	0.320028	0.124416
-0.12636	0.06241	0.04656	-0.00225	-0.08034	0.216968	0.062071	-0.09549	-0.18296
0.156888	0.485773	0.603202	0.979939	0.369237	0.014278	0.48815	0.285572	0.039508
0.114197	0.342194	0.274146	0.306531	-0.05331	-0.31546	-0.0582	0.280124	0.327317
0.201111	8.22E-05	0.001815	0.000457	0.551708	0.000303	0.515713	0.001424	0.000173
0.153186	0.430894	0.640383	0.643395	-0.47102	-0.54058	-0.42769	0.479597	0.627519
0.085539	4.26E-07	5.19E-16	3.43E-16	2.29E-08	5.37E-11	5.3E-07	1.16E-08	2.91E-15
-0.39061	-0.1147	0.380583	0.203681	-0.49179	-0.30584	-0.2929	0.226685	0.195869
5.62E-06	0.199114	1.02E-05	0.02163	4.32E-09	0.000471	0.000833	0.010383	0.027319
-0.14354	0.287081	0.851403	0.674268	-0.82333	-0.66694	-0.62702	0.56595	0.692622
0.107415	0.001066	7.77E-37	3.65E-18	1.54E-32	1.13E-17	3.11E-15	4.1E-12	1.88E-19
-0.07128	0.118067	0.497615	0.465172	-0.47266	-0.3487	-0.29102	0.303714	0.303761
0.425796	0.18616	2.65E-09	3.59E-08	2.01E-08	5.88E-05	0.000902	0.000518	0.000517
-0.04065	0.016872	0.328093	0.420668	-0.40627	-0.34209	-0.36954	-0.01618	0.189757
0.650024	0.850665	0.000166	8.47E-07	2.15E-06	8.27E-05	1.91E-05	0.856705	0.032617
-0.29088	-0.18598	0.377138	0.012706	-0.24247	-0.36143	0.07851	-0.10247	0.183894
0.000908	0.036305	1.24E-05	0.887253	0.006021	2.98E-05	0.380287	0.251636	0.038493
-0.58318	-0.44334	0.457702	-0.04429	-0.42608	-0.32225	-0.06897	-0.22892	0.168945
6.28E-13	1.79E-07	6.3E-08	0.620983	5.9E-07	0.00022	0.441	0.009631	0.057596
-0.28033	-0.16245	0.520154	0.117374	-0.46841	-0.33262	-0.20467	-0.00037	0.337173
0.001412	0.068044	3.67E-10	0.188777	2.8E-08	0.000133	0.02099	0.996678	0.000106
0.125163	-0.28796	-0.37228	-0.31897	0.402644	0.382057	0.436794	-0.43816	-0.30385
0.160888	0.001027	1.63E-05	0.000257	2.69E-06	9.32E-06	2.84E-07	2.58E-07	0.000515
0.127224	-0.08913	-0.43396	-0.31071	0.618183	0.278859	0.540556	-0.45383	-0.3348
0.154052	0.319024	3.45E-07	0.000378	9.69E-15	0.0015	5.39E-11	8.38E-08	0.000119
-0.22579	-0.44504	-0.44837	-0.56039	0.4919	0.168154	0.461815	-0.52905	-0.36313
0.010697	1.59E-07	1.25E-07	7.34E-12	4.28E-09	0.058792	4.63E-08	1.61E-10	2.72E-05
-0.37124	-0.55075	-0.22838	-0.5026	0.325865	0.099771	0.46146	-0.70709	-0.29301
1.73E-05	1.97E-11	0.009807	1.73E-09	0.000185	0.26441	4.75E-08	1.54E-20	0.000828
-0.08942	-0.33079	-0.50857	-0.41829	0.380392	0.487402	0.22586	-0.38918	-0.41669
0.31741	0.000146	1.03E-09	9.9E-07	1.03E-05	6.2E-09	0.010673	6.12E-06	1.1E-06
0.413224	0.070891	-0.70149	-0.37778	0.758164	0.542136	0.382916	-0.09973	-0.43431
1.38E-06	0.42836	4.12E-20	1.19E-05	5.71E-25	4.62E-11	8.87E-06	0.264593	3.37E-07
0.517323	0.537151	-0.12114	0.224023	0.005143	0.141382	-0.3011	0.620321	0.116267
4.74E-10	7.49E-11	0.174883	0.011344	0.954236	0.112849	0.000582	7.38E-15	0.193011
0.384506	0.15596	-0.59701	-0.22679	0.410192	0.558727	0.106313	0.134243	-0.41537
8.08E-06	0.079957	1.28E-13	0.010345	1.67E-06	8.72E-12	0.234199	0.132399	1.2E-06
0.028364	0.205396	-0.1513	0.015186	0.162274	0.157593	0.235413	0.125705	-0.20306
0.751586	0.020527	0.089511	0.865435	0.06834	0.076811	0.007715	0.159068	0.022039

ABHD8	ABRACL	ACAP3	ACO2	ACOT9	ACP6	ACTR5	ADAL	ADAMTS1
cyan	cyan	cyan	cyan	cyan	cyan	cyan	cyan	cyan
-0.04518	0.227343	-0.23029	0.101735	0.093751	-0.01158	-0.001	0.089743	-0.30206
0.613996	0.010157	0.009196	0.255072	0.294463	0.897199	0.991065	0.315676	0.000558
-0.01191	0.172249	-0.20516	0.167314	0.036496	0.017625	-0.00174	0.135294	-0.34149
0.894319	0.052813	0.020679	0.060086	0.683744	0.844085	0.984536	0.129369	8.53E-05
-0.41934	0.009777	0.164442	-0.33885	0.270737	0.25671	0.258722	0.153381	0.118499
9.24E-07	0.913124	0.064683	9.74E-05	0.00208	0.003576	0.003314	0.085137	0.184544
-0.67543	0.20089	-0.39773	-0.6479	0.558193	0.550118	0.329809	0.459909	-0.24176
3.05E-18	0.023533	3.65E-06	1.82E-16	9.21E-12	2.09E-11	0.000153	5.34E-08	0.006177
-0.20366	0.596	-0.29893	-0.31955	0.280121	0.340422	0.376334	0.354641	-0.57715
0.021645	1.44E-13	0.000641	0.00025	0.001424	9E-05	1.3E-05	4.3E-05	1.23E-12
-0.70142	0.644271	-0.7186	-0.78561	0.698484	0.681682	0.451092	0.676287	-0.70719
4.17E-20	3.03E-16	1.88E-21	7.79E-28	6.93E-20	1.13E-18	1.02E-07	2.66E-18	1.51E-20
-0.53088	0.277256	-0.55851	-0.44249	0.455029	0.579789	0.218256	0.479917	-0.43298
1.36E-10	0.001601	8.92E-12	1.9E-07	7.68E-08	9.16E-13	0.013698	1.14E-08	3.69E-07
-0.24063	0.096889	-0.34875	-0.32386	0.232555	0.304047	0.098564	0.245292	-0.34027
0.006427	0.278523	5.86E-05	0.000204	0.008513	0.00051	0.270259	0.005443	9.07E-05
-0.37632	0.27008	-0.19136	-0.4159	0.532954	0.281283	-0.02839	-0.15521	0.231331
1.3E-05	0.002135	0.031147	1.16E-06	1.12E-10	0.001358	0.751403	0.081439	0.008876
-0.35333	0.403481	-0.35767	-0.33844	0.480825	0.310089	-0.02591	-0.04457	-0.15198
4.61E-05	2.56E-06	3.65E-05	9.95E-05	1.06E-08	0.000388	0.772499	0.618785	0.088058
-0.35488	0.363109	-0.44819	-0.36333	0.51855	0.326382	0.014965	0.127421	-0.31972
4.24E-05	2.72E-05	1.26E-07	2.69E-05	4.24E-10	0.00018	0.867375	0.153411	0.000248
0.222446	-0.37716	0.227295	0.528055	-0.14162	-0.27881	-0.18518	-0.40108	0.345858
0.011949	1.24E-05	0.010173	1.77E-10	0.112246	0.001503	0.037137	2.97E-06	6.81E-05
0.159721	-0.60582	0.698881	0.344985	-0.28541	-0.20934	-0.15363	-0.50142	0.782015
0.072861	4.48E-14	6.47E-20	7.13E-05	0.001144	0.018177	0.084627	1.92E-09	1.95E-27
0.521182	-0.23089	0.70329	0.33437	-0.39503	-0.25638	-0.14109	-0.55687	0.526497
3.34E-10	0.009012	3.01E-20	0.000122	4.31E-06	0.00362	0.113602	1.06E-11	2.05E-10
0.149528	-0.24063	0.455014	0.210222	-0.1068	-0.09042	-0.25826	-0.58264	0.569123
0.093371	0.006427	7.68E-08	0.017681	0.232037	0.312019	0.003372	6.67E-13	2.93E-12
0.329859	-0.31218	0.326349	0.57921	-0.57383	-0.38153	-0.3159	-0.26669	0.027508
0.000152	0.000353	0.000181	9.77E-13	1.76E-12	9.61E-06	0.000297	0.002439	0.758851
0.502611	-0.61536	0.709274	0.682242	-0.68201	-0.58842	-0.29326	-0.37007	0.436706
1.73E-09	1.38E-14	1.04E-20	1.03E-18	1.07E-18	3.47E-13	0.00082	1.85E-05	2.85E-07
0.209018	-0.01006	-0.18902	0.068903	-0.22942	-0.26908	0.01289	0.317064	-0.27977
0.018357	0.910645	0.033315	0.441455	0.009472	0.00222	0.88563	0.000281	0.001445
0.613954	-0.27119	0.16664	0.570988	-0.57937	-0.59323	-0.22337	-0.07096	0.011664
1.65E-14	0.002043	0.06114	2.4E-12	9.59E-13	1.99E-13	0.011591	0.427929	0.896446
-0.19136	-0.12808	0.42067	0.067145	-0.04192	-0.04237	0.293106	-0.03627	0.295038
0.03115	0.151285	8.47E-07	0.453221	0.639811	0.636246	0.000825	0.685621	0.000759

ADAMTSL:	ADCY9	ADD1	ADH4	ADH5	ADK	ADM5	ADPRM	ADRA2C
cyan	cyan	cyan	cyan	cyan	cyan	cyan	cyan	cyan
-0.09788	-0.1933	-0.03553	0.104331	-0.15012	0.030761	-0.20189	-0.017	0.077098
0.273597	0.029452	0.691654	0.243085	0.092074	0.731362	0.022835	0.849561	0.388939
-0.06173	-0.18707	0.000421	0.146418	-0.17898	0.058616	-0.19278	0.024821	0.112515
0.490549	0.035204	0.996255	0.100465	0.044072	0.512722	0.029894	0.781786	0.207871
-0.39241	0.12507	-0.19569	0.045939	0.41033	0.244372	-0.03144	0.403194	-0.28566
5.05E-06	0.161201	0.027461	0.608054	1.66E-06	0.005626	0.725649	2.6E-06	0.001132
-0.68179	-0.08658	-0.38872	0.351973	0.609831	0.276705	-0.37677	0.57746	-0.57124
1.11E-18	0.333099	6.29E-06	4.95E-05	2.75E-14	0.001637	1.27E-05	1.18E-12	2.33E-12
-0.26256	-0.55943	-0.59784	0.327005	0.440016	0.340616	-0.31518	0.516368	-0.05233
0.002862	8.11E-12	1.16E-13	0.000175	2.26E-07	8.91E-05	0.000307	5.17E-10	0.55904
-0.74689	-0.46456	-0.73981	0.570739	0.769363	0.446105	-0.64284	0.764558	-0.57939
6.69E-24	3.76E-08	2.94E-23	2.46E-12	4.32E-26	1.47E-07	3.7E-16	1.33E-25	9.58E-13
-0.37579	-0.19106	-0.33841	0.406838	0.38898	0.191271	-0.29781	0.460768	-0.37309
1.34E-05	0.031422	9.97E-05	2.07E-06	6.2E-06	0.03123	0.000673	5.01E-08	1.56E-05
-0.32435	-0.31643	-0.31098	0.177399	0.294233	0.017402	-0.34484	-0.00142	-0.408
0.000199	0.00029	0.000373	0.046013	0.000786	0.846032	7.18E-05	0.987332	1.92E-06
-0.10305	0.190177	-0.18501	0.145856	0.12827	-0.23475	0.040238	0.150426	-0.39412
0.248967	0.032227	0.037314	0.101792	0.150666	0.007895	0.653322	0.091398	4.55E-06
-0.02177	-0.33472	-0.32964	0.271743	0.074376	-0.17847	-0.14784	-0.02053	-0.36355
0.808064	0.00012	0.000154	0.001998	0.405954	0.044694	0.097171	0.818759	2.65E-05
-0.27563	-0.32479	-0.26082	0.331257	0.219367	0.016186	-0.36977	0.040984	-0.40582
0.00171	0.000195	0.00306	0.000142	0.013214	0.856666	1.88E-05	0.647321	2.21E-06
0.315639	0.262321	0.627203	-0.09053	-0.34222	-0.40105	0.399474	-0.19803	0.228346
0.000301	0.002889	3.04E-15	0.311459	8.21E-05	2.97E-06	3.28E-06	0.025627	0.00982
0.328439	0.451284	0.514625	-0.3783	-0.39857	-0.32731	0.604031	-0.35032	0.180024
0.000163	1.01E-07	6.04E-10	1.16E-05	3.47E-06	0.000173	5.56E-14	5.4E-05	0.042838
0.543287	0.056544	0.197979	-0.33025	-0.31106	-0.25014	0.544692	-0.32359	0.410067
4.12E-11	0.527767	0.025669	0.00015	0.000372	0.004564	3.59E-11	0.000206	1.69E-06
0.485875	0.077817	0.26783	-0.20872	-0.42072	-0.45021	0.486561	-0.47503	0.073242
7.02E-09	0.384518	0.002332	0.018527	8.44E-07	1.09E-07	6.64E-09	1.67E-08	0.413164
0.516302	-0.15337	0.455997	-0.18203	-0.54502	-0.1603	0.188599	-0.53329	0.370026
5.2E-10	0.085164	7.15E-08	0.040538	3.48E-11	0.071818	0.033714	1.08E-10	1.85E-05
0.453388	0.321915	0.61175	-0.49469	-0.36775	-0.00615	0.430055	-0.37944	0.551083
8.66E-08	0.000224	2.17E-14	3.39E-09	2.11E-05	0.945334	4.51E-07	1.09E-05	1.9E-11
-0.17766	0.131707	0.036937	-0.08353	0.13199	0.291241	-0.26834	0.108396	0.156365
0.045689	0.139938	0.680128	0.350474	0.139081	0.000894	0.002286	0.225108	0.079167
0.322745	0.25922	0.382375	-0.32874	-0.34966	0.014883	0.085739	-0.31821	0.537278
0.000215	0.003252	9.15E-06	0.000161	5.59E-05	0.868095	0.337839	0.000266	7.39E-11
-0.05399	0.248601	0.035929	-0.17757	-0.04055	0.055117	0.330683	0.273885	0.182691
0.546613	0.004828	0.688399	0.045806	0.650777	0.538252	0.000146	0.001834	0.039802

AES	AGA	AGAP3	AGBL3	AGGF1	AGPAT3	AGPS	AGT	AHDC1
cyan	cyan	cyan	cyan	cyan	cyan	cyan	cyan	cyan
-0.10945	0.270723	-0.04931	-0.15877	-0.13339	0.070011	0.024634	-0.10448	-0.24757
0.220585	0.002081	0.581964	0.0746	0.13489	0.434127	0.783386	0.242389	0.005014
-0.03896	0.225396	0.019421	-0.10582	-0.16033	0.09151	-0.04605	-0.05816	-0.27463
0.663621	0.010839	0.828427	0.236373	0.07177	0.306203	0.607181	0.51598	0.00178
-0.10517	-0.18844	-0.35146	0.507142	0.28769	-0.50155	0.304594	-0.34055	-0.06887
0.239305	0.033871	5.09E-05	1.17E-09	0.001039	1.89E-09	0.000498	8.94E-05	0.441677
-0.57289	0.246311	-0.73745	0.689069	0.641507	-0.57073	0.618212	-0.46244	-0.41474
1.95E-12	0.005247	4.76E-23	3.39E-19	4.45E-16	2.46E-12	9.65E-15	4.41E-08	1.25E-06
-0.07216	0.394623	-0.05383	0.319048	0.03221	-0.48781	-0.03165	-0.27264	-0.52119
0.42013	4.41E-06	0.547787	0.000256	0.719226	5.99E-09	0.723891	0.001928	3.34E-10
-0.67752	0.628917	-0.72752	0.711697	0.63405	-0.68994	0.585247	-0.54117	-0.76363
2.19E-18	2.42E-15	3.43E-22	6.71E-21	1.23E-15	2.93E-19	4.98E-13	5.07E-11	1.65E-25
-0.36915	0.348079	-0.41898	0.434694	0.289649	-0.35204	0.213884	-0.26622	-0.58133
1.95E-05	6.07E-05	9.46E-07	3.28E-07	0.000957	4.93E-05	0.015756	0.002484	7.72E-13
-0.5502	0.435245	-0.30534	0.295425	0.423944	0.002732	0.101305	0.225401	-0.08318
2.08E-11	3.16E-07	0.000482	0.000746	6.81E-07	0.975679	0.257097	0.010837	0.352486
-0.24495	-0.02541	-0.52691	-0.06812	0.107251	-0.16397	0.324296	-0.32602	-0.00603
0.005511	0.776763	1.97E-10	0.446679	0.230072	0.065463	0.0002	0.000184	0.946375
-0.51528	0.507578	-0.48862	-0.22432	0.053754	0.019961	0.30267	-0.05161	-0.20058
5.7E-10	1.13E-09	5.61E-09	0.011232	0.548357	0.823734	0.000543	0.564478	0.023753
-0.56952	0.44979	-0.59928	0.007386	0.217552	-0.04894	0.421421	-0.11574	-0.23357
2.8E-12	1.13E-07	9.82E-14	0.934315	0.014012	0.58482	8.06E-07	0.195034	0.00822
0.412093	-0.44896	0.142594	-0.46019	-0.47761	0.318673	-0.3266	0.050305	0.299635
1.48E-06	1.2E-07	0.109766	5.23E-08	1.36E-08	0.000261	0.000179	0.574347	0.000621
0.363853	-0.62172	0.191612	-0.26839	-0.35377	0.254102	-0.2381	0.201601	0.657228
2.61E-05	6.17E-15	0.030924	0.002282	4.5E-05	0.003942	0.007026	0.023035	4.78E-17
0.580653	-0.3613	0.527477	-0.37181	-0.5675	0.189557	-0.45789	0.322392	0.657803
8.32E-13	3E-05	1.87E-10	1.68E-05	3.48E-12	0.032804	6.21E-08	0.000219	4.4E-17
0.120113	-0.16468	0.087985	-0.56278	-0.44091	0.390948	-0.15177	0.296699	0.55863
0.178595	0.064298	0.325292	5.72E-12	2.13E-07	5.51E-06	0.088498	0.000706	8.81E-12
0.255993	0.066514	0.505036	-0.44377	-0.46024	0.538134	-0.23384	0.462192	0.099462
0.003673	0.457493	1.4E-09	1.74E-07	5.21E-08	6.81E-11	0.008144	4.5E-08	0.2659
0.684386	-0.63096	0.705728	-0.16664	-0.41642	0.274643	-0.44427	0.390047	0.440012
7.3E-19	1.85E-15	1.96E-20	0.061138	1.12E-06	0.001779	1.67E-07	5.81E-06	2.26E-07
0.127495	-0.02743	0.205763	0.292518	0.312245	-0.09072	0.060054	-0.07896	-0.23522
0.15317	0.759476	0.020298	0.000846	0.000352	0.31043	0.502416	0.377533	0.007767
0.545304	-0.32922	0.700562	-0.22266	-0.21762	0.337425	-0.47309	0.285545	0.13915
3.38E-11	0.000157	4.84E-20	0.011867	0.013983	0.000105	1.95E-08	0.001137	0.118703
0.349219	-0.42372	0.166098	0.07409	-0.284	-0.35057	-0.08539	-0.37397	-0.10368
5.72E-05	6.91E-07	0.061998	0.407764	0.001213	5.33E-05	0.33979	1.48E-05	0.246035

AIMP1	AK3	AK6	ALDOA	ALG13	ALG14	ALG5	ALG8	ALKBH1
cyan	cyan	cyan	cyan	cyan	cyan	cyan	cyan	cyan
0.035501	0.102583	-0.02618	0.092668	0.094871	0.230501	0.125031	-0.05361	0.014306
0.691923	0.251113	0.77019	0.300098	0.288707	0.00913	0.161334	0.54945	0.873166
-0.01731	0.111393	-0.03175	0.154124	0.088054	0.27407	0.175998	-0.01206	0.084659
0.846876	0.212469	0.723034	0.083618	0.324911	0.001821	0.047786	0.892943	0.343985
0.235625	-0.19778	0.362923	-0.39411	0.07181	0.063945	0.116895	0.428591	0.386761
0.007659	0.025823	2.75E-05	4.55E-06	0.422384	0.475088	0.1906	4.98E-07	7.07E-06
0.532528	0.157177	0.566497	-0.5698	0.440106	0.318914	0.414982	0.639707	0.53824
1.16E-10	0.077603	3.87E-12	2.72E-12	2.25E-07	0.000258	1.23E-06	5.7E-16	6.74E-11
0.431945	0.357008	0.433753	-0.01936	0.297574	0.724889	0.694516	0.387617	0.487734
3.96E-07	3.79E-05	3.5E-07	0.828963	0.00068	5.7E-22	1.36E-19	6.72E-06	6.03E-09
0.739165	0.510359	0.753107	-0.47822	0.651804	0.788685	0.822932	0.793088	0.71778
3.35E-23	8.82E-10	1.75E-24	1.3E-08	1.05E-16	3.51E-28	1.75E-32	1.09E-28	2.19E-21
0.511991	0.203751	0.342315	-0.20756	0.367706	0.500031	0.658891	0.586018	0.454963
7.63E-10	0.021584	8.17E-05	0.019205	2.11E-05	2.16E-09	3.75E-17	4.56E-13	7.71E-08
0.329069	0.427448	0.169693	-0.06302	0.470584	0.275776	0.185499	0.256345	-0.10119
0.000158	5.38E-07	0.056483	0.48154	2.37E-08	0.0017	0.036802	0.003625	0.257652
0.288079	0.020477	0.006211	-0.18306	-0.11068	-0.04399	0.152844	0.335847	0.145035
0.001022	0.819254	0.94475	0.039391	0.21541	0.623334	0.086248	0.000113	0.103756
0.434374	0.451846	0.002615	-0.19549	0.157281	0.275039	0.311701	0.254593	0.02345
3.35E-07	9.7E-08	0.976719	0.027624	0.077404	0.001751	0.000361	0.003871	0.793558
0.374181	0.320504	0.1353	-0.31196	0.239549	0.236323	0.232059	0.271812	0.138668
1.47E-05	0.000239	0.129354	0.000357	0.006678	0.007475	0.008658	0.001993	0.119998
-0.41802	-0.39087	-0.43881	0.119893	-0.53869	-0.43885	-0.29698	-0.275	-0.02644
1.01E-06	5.53E-06	2.46E-07	0.179395	6.46E-11	2.46E-07	0.000698	0.001754	0.767905
-0.32475	-0.59998	-0.42333	0.059868	-0.51524	-0.64839	-0.59147	-0.29907	-0.31458
0.000195	9.03E-14	7.1E-07	0.503749	5.72E-10	1.7E-16	2.44E-13	0.000636	0.000316
-0.26968	-0.32102	-0.39068	0.358003	-0.5193	-0.32549	-0.40393	-0.43332	-0.29464
0.002169	0.000233	5.6E-06	3.59E-05	3.97E-10	0.000188	2.48E-06	3.61E-07	0.000772
-0.09885	-0.17632	-0.48828	0.118524	-0.40965	-0.38066	-0.38733	-0.28261	-0.41602
0.268883	0.047369	5.77E-09	0.184451	1.73E-06	1.01E-05	6.84E-06	0.001285	1.15E-06
-0.38706	0.041062	-0.40087	0.222818	-0.10681	-0.13409	-0.27523	-0.48392	-0.44893
6.95E-06	0.646691	3.01E-06	0.011804	0.231988	0.132832	0.001738	8.23E-09	1.2E-07
-0.63991	-0.53395	-0.36072	0.208617	-0.40026	-0.49625	-0.54834	-0.56438	-0.36574
5.54E-16	1.02E-10	3.1E-05	0.018587	3.12E-06	2.97E-09	2.5E-11	4.84E-12	2.35E-05
-0.24442	0.01426	0.206429	0.024514	0.223452	-0.00051	-0.03614	-0.11774	0.067448
0.005615	0.87357	0.019887	0.784412	0.01156	0.995435	0.686653	0.18738	0.451186
-0.60692	-0.19097	-0.27161	0.43663	-0.1985	-0.28988	-0.33731	-0.52429	-0.34121
3.92E-14	0.031505	0.002009	2.87E-07	0.025275	0.000947	0.000105	2.51E-10	8.65E-05
-0.02424	-0.45087	0.127838	-0.1748	-0.10235	-0.06972	0.049777	0.057296	0.227288
0.786762	1.04E-07	0.152056	0.049351	0.252185	0.43608	0.578375	0.522281	0.010175

ALKBH3	AMBRA1	ANAPC10	ANAPC13	ANAPC4	ANK1	ANKRA2	ANKRD11	ANKRD13I
cyan	cyan	cyan	cyan	cyan	cyan	cyan	cyan	cyan
0.288593	0.005621	-0.01742	0.161605	0.179789	0.235404	-0.01906	-0.17131	-0.15381
0.001	0.949992	0.845901	0.069503	0.043115	0.007718	0.831546	0.054132	0.084267
0.324871	-0.00316	0.028661	0.192713	0.196954	0.233646	0.01122	-0.26621	-0.11865
0.000194	0.971893	0.749074	0.029955	0.02646	0.0082	0.900371	0.002485	0.18398
-0.02398	-0.14162	0.270066	-0.04031	0.188685	-0.25951	0.317478	-0.04851	0.049089
0.789029	0.112234	0.002136	0.652767	0.033631	0.003216	0.000276	0.588127	0.58365
0.260096	-0.40553	0.544531	0.190387	0.577089	-0.49575	0.619492	-0.32216	-0.47185
0.003145	2.25E-06	3.65E-11	0.032034	1.23E-12	3.1E-09	8.21E-15	0.000221	2.14E-08
0.644381	-0.52635	0.60659	0.651376	0.289919	-0.26254	0.514171	-0.6707	-0.35591
2.99E-16	2.08E-10	4.08E-14	1.11E-16	0.000946	0.002865	6.29E-10	6.35E-18	4.02E-05
0.701799	-0.69048	0.860241	0.674949	0.726647	-0.59759	0.840062	-0.76497	-0.79663
3.9E-20	2.68E-19	2.24E-38	3.28E-18	4.06E-22	1.2E-13	5.32E-35	1.21E-25	4.18E-29
0.532293	-0.48197	0.548822	0.493674	0.561269	-0.26059	0.485521	-0.59487	-0.57268
1.19E-10	9.63E-09	2.38E-11	3.69E-09	6.7E-12	0.003087	7.23E-09	1.65E-13	2E-12
0.259109	-0.28121	0.130107	0.278606	0.287233	-0.40014	0.237012	-0.17467	-0.43094
0.003266	0.001362	0.144857	0.001515	0.00106	3.15E-06	0.007298	0.04952	4.25E-07
0.068232	-0.16125	-0.01332	-0.09459	0.100851	-0.11924	0.020007	0.164805	-0.31267
0.445928	0.070123	0.881856	0.290124	0.259246	0.181809	0.823337	0.064086	0.000345
0.356369	-0.2481	-0.01519	0.191815	0.262838	-0.21192	0.022656	-0.05877	-0.49063
3.92E-05	0.004918	0.865363	0.030744	0.002832	0.016762	0.8004	0.511645	4.75E-09
0.370934	-0.19331	0.070551	0.239087	0.347159	-0.27441	0.087174	-0.1663	-0.54127
1.76E-05	0.029444	0.430583	0.006787	6.37E-05	0.001796	0.329788	0.061685	5.02E-11
-0.25736	0.414912	-0.40017	-0.51289	-0.29924	0.387046	-0.41193	0.433569	0.420201
0.00349	1.23E-06	3.14E-06	7.05E-10	0.000632	6.95E-06	1.5E-06	3.55E-07	8.73E-07
-0.55227	0.470453	-0.60673	-0.71648	-0.37941	0.279705	-0.49046	0.73437	0.661243
1.69E-11	2.39E-08	4.01E-14	2.79E-21	1.09E-05	0.001449	4.82E-09	8.86E-23	2.65E-17
-0.32672	0.286488	-0.48247	-0.43091	-0.5967	0.16423	-0.43712	0.516826	0.612225
0.000178	0.001093	9.25E-09	4.26E-07	1.33E-13	0.065033	2.77E-07	4.96E-10	2.04E-14
-0.27225	0.29391	-0.6481	-0.47781	-0.34082	0.142721	-0.53978	0.654488	0.345654
0.001958	0.000797	1.78E-16	1.34E-08	8.82E-05	0.109445	5.81E-11	7.12E-17	6.89E-05
-0.21488	0.313249	-0.45135	-0.09942	-0.2041	0.302432	-0.39969	0.194909	0.451577
0.015264	0.000336	1.01E-07	0.266103	0.021359	0.000549	3.24E-06	0.028099	9.89E-08
-0.62567	0.484529	-0.40075	-0.39354	-0.52874	0.384478	-0.45403	0.357718	0.823103
3.71E-15	7.83E-09	3.03E-06	4.71E-06	1.66E-10	8.09E-06	8.26E-08	3.65E-05	1.65E-32
-0.13398	0.026062	0.295683	0.161651	0.021719	0.04661	0.217664	-0.30188	-0.04632
0.13318	0.771171	0.000738	0.069422	0.808493	0.602811	0.013962	0.000562	0.605049
-0.33239	0.322973	-0.16949	-0.05235	-0.38788	0.461942	-0.2923	-0.00411	0.343006
0.000135	0.000213	0.056786	0.558903	6.62E-06	4.58E-08	0.000854	0.963399	7.89E-05
-0.1662	-0.01138	0.078384	-0.10428	0.074794	0.210246	0.121971	0.088712	0.425511
0.061836	0.898971	0.381054	0.243305	0.403311	0.017668	0.17192	0.321295	6.13E-07

ANKRD23	ANKRD49	ANKS1A	AP1S2	AP2A1	AP2A2	APBA2	APC2	APIP
cyan	cyan	cyan	cyan	cyan	cyan	cyan	cyan	cyan
-0.13421	0.017588	0.023419	-0.06665	-0.19894	-0.01712	-0.10203	-0.15084	0.189873
0.132503	0.844408	0.793829	0.456558	0.024946	0.848519	0.253675	0.090503	0.032509
-0.10202	0.032189	-0.04162	-0.0746	-0.1636	-0.02122	-0.0587	-0.15663	0.145809
0.253722	0.719405	0.642204	0.40452	0.066091	0.812843	0.512095	0.078647	0.101905
0.138846	0.241458	-0.33171	0.39439	-0.20604	-0.42626	-0.13799	-0.0586	-0.1431
0.11952	0.006242	0.000139	4.48E-06	0.020127	5.83E-07	0.121837	0.512823	0.10851
-0.14124	0.528784	-0.44451	0.53965	-0.63363	-0.70007	-0.53352	-0.41448	0.206595
0.113224	1.66E-10	1.65E-07	5.88E-11	1.3E-15	5.27E-20	1.06E-10	1.27E-06	0.019786
-0.36726	0.63887	-0.53006	0.388641	-0.44729	-0.33437	-0.33904	-0.51553	0.526035
2.16E-05	6.39E-16	1.47E-10	6.32E-06	1.35E-07	0.000122	9.65E-05	5.57E-10	2.14E-10
-0.51059	0.838627	-0.64134	0.687193	-0.89504	-0.79793	-0.7588	-0.77051	0.627682
8.64E-10	8.87E-35	4.55E-16	4.62E-19	1.16E-45	2.93E-29	4.95E-25	3.29E-26	2.85E-15
-0.27023	0.517036	-0.43589	0.342135	-0.55203	-0.51057	-0.48041	-0.5617	0.362008
0.002122	4.87E-10	3.02E-07	8.25E-05	1.73E-11	8.66E-10	1.09E-08	6.41E-12	2.89E-05
-0.32719	0.191377	0.048126	0.132423	-0.40536	-0.25331	-0.4144	-0.21452	0.434109
0.000174	0.031135	0.591057	0.137778	2.27E-06	0.004059	1.28E-06	0.01544	3.42E-07
-0.09377	-0.03622	-0.04646	0.036917	-0.07576	-0.19017	-0.46364	-0.19149	0.024183
0.294371	0.686015	0.603951	0.680298	0.397218	0.03223	4.03E-08	0.031036	0.787258
-0.32047	0.092111	0.046186	0.03688	-0.32243	-0.13557	-0.51868	-0.24784	0.568757
0.00024	0.303024	0.606123	0.680597	0.000218	0.128586	4.2E-10	0.004963	3.04E-12
-0.3717	0.148688	0.015732	0.130159	-0.47071	-0.25506	-0.47597	-0.25363	0.470974
1.69E-05	0.095246	0.860651	0.144696	2.34E-08	0.003803	1.55E-08	0.004011	2.3E-08
0.471318	-0.38672	0.221689	-0.57211	0.498377	0.269022	0.358113	0.264044	-0.53248
2.24E-08	7.09E-06	0.01225	2.12E-12	2.48E-09	0.002226	3.57E-05	0.002703	1.17E-10
0.628597	-0.56973	0.343059	-0.36863	0.562574	0.322881	0.40604	0.60961	-0.54907
2.53E-15	2.74E-12	7.87E-05	2.01E-05	5.85E-12	0.000214	2.18E-06	2.82E-14	2.33E-11
0.362623	-0.36291	0.196599	-0.29688	0.615713	0.479722	0.411667	0.601382	-0.17748
2.79E-05	2.75E-05	0.026738	0.000701	1.32E-14	1.15E-08	1.52E-06	7.65E-14	0.045913
0.311964	-0.52783	0.434124	-0.4133	0.427005	0.369806	0.12755	0.485043	-0.03588
0.000356	1.81E-10	3.41E-07	1.37E-06	5.55E-07	1.88E-05	0.15299	7.51E-09	0.688833
0.230046	-0.34764	0.355955	-0.40903	0.307737	0.380229	0.47114	0.410749	0.064402
0.009272	6.21E-05	4.01E-05	1.8E-06	0.000432	1.04E-05	2.27E-08	1.61E-06	0.471931
0.478209	-0.45416	0.230172	-0.29181	0.630662	0.441947	0.84835	0.661059	-0.65145
1.3E-08	8.19E-08	0.009233	0.000872	1.93E-15	1.98E-07	2.51E-36	2.72E-17	1.1E-16
-0.1437	0.202181	-0.13274	0.208145	-0.06426	-0.03012	0.201305	-0.11337	-0.21533
0.107005	0.022635	0.136822	0.018861	0.472884	0.736742	0.023241	0.204418	0.015048
0.042673	-0.26775	0.208837	-0.27245	0.442249	0.481191	0.592379	0.209108	-0.44851
0.633818	0.00234	0.01846	0.001943	1.93E-07	1.03E-08	2.2E-13	0.018306	1.24E-07
0.487661	0.0849	-0.18005	0.036154	0.062686	-0.16195	0.151231	-0.04389	-0.32119
6.07E-09	0.342607	0.042806	0.686556	0.483841	0.068895	0.089656	0.62418	0.000232

APTX	ARF4	ARFIP1	ARHGAP1	ARHGAP3:	ARHGAP3!	ARHGEF2	ARHGEF4	ARIH2OS
cyan	cyan	cyan	cyan	cyan	cyan	cyan	cyan	cyan
0.132858	0.056735	-0.11298	0.211011	-0.17625	-0.19966	0.131564	0.011848	-0.0433
0.136476	0.526371	0.20597	0.01725	0.047464	0.024415	0.140371	0.894823	0.62887
0.198987	0.039967	-0.20081	0.215414	-0.15634	-0.21755	0.112445	0.087853	-0.00755
0.024912	0.655502	0.023586	0.015007	0.079226	0.014013	0.208153	0.326021	0.932891
0.096948	0.344214	0.339371	-0.34565	0.138707	-0.02282	-0.07932	-0.32199	0.254279
0.27823	7.42E-05	9.49E-05	6.89E-05	0.119893	0.798948	0.375392	0.000223	0.003916
0.246465	0.629045	0.668683	-0.42989	-0.39457	-0.37733	-0.38896	-0.51475	0.352229
0.005218	2.38E-15	8.65E-18	4.56E-07	4.43E-06	1.23E-05	6.21E-06	5.97E-10	4.88E-05
0.606374	0.275392	0.162938	-0.40404	-0.44545	-0.48033	-0.23281	-0.34218	0.638688
4.19E-14	0.001726	0.067203	2.47E-06	1.54E-07	1.1E-08	0.00844	8.23E-05	6.55E-16
0.604538	0.745504	0.716201	-0.55333	-0.7824	-0.73275	-0.57179	-0.65206	0.619673
5.23E-14	8.97E-24	2.94E-21	1.52E-11	1.77E-27	1.23E-22	2.2E-12	1.01E-16	8.02E-15
0.340785	0.49585	0.370434	-0.29858	-0.57661	-0.59267	-0.32306	-0.37766	0.425086
8.84E-05	3.07E-09	1.81E-05	0.00065	1.3E-12	2.13E-13	0.000212	1.2E-05	6.31E-07
0.174015	0.228262	0.152834	-0.00498	-0.39307	-0.15197	-0.37111	-0.09988	0.114768
0.050392	0.009848	0.086269	0.95571	4.85E-06	0.088087	1.74E-05	0.263875	0.198853
-0.10374	0.516272	0.36208	0.105187	-0.26257	-0.11686	-0.03669	-0.44742	-0.10752
0.245771	5.21E-10	2.88E-05	0.239218	0.002861	0.190719	0.682191	1.34E-07	0.228891
0.09371	0.331294	0.36354	0.336112	-0.47889	-0.25029	-0.00924	-0.35062	-0.03484
0.294676	0.000142	2.66E-05	0.000112	1.23E-08	0.004538	0.917914	5.32E-05	0.697368
0.146504	0.378801	0.469361	0.120592	-0.45298	-0.24051	-0.14589	-0.33488	0.05932
0.100264	1.13E-05	2.6E-08	0.176856	8.92E-08	0.006455	0.101722	0.000119	0.507663
-0.1764	-0.22128	-0.36284	0.307549	0.319988	0.387907	0.338656	0.253236	-0.16622
0.047273	0.012415	2.76E-05	0.000436	0.000245	6.61E-06	9.84E-05	0.004071	0.061806
-0.53013	-0.30732	-0.28058	0.393034	0.706637	0.648983	0.531201	0.238926	-0.3412
1.46E-10	0.000441	0.001398	4.86E-06	1.67E-20	1.57E-16	1.32E-10	0.006826	8.65E-05
-0.34248	-0.46011	-0.28287	0.333363	0.591587	0.633253	0.533609	0.241604	-0.05626
8.1E-05	5.26E-08	0.001272	0.000128	2.41E-13	1.36E-15	1.05E-10	0.00621	0.529827
-0.42889	-0.21834	-0.13035	0.692319	0.354107	0.52561	0.54513	0.115501	-0.3483
4.88E-07	0.013662	0.144093	1.97E-19	4.42E-05	2.22E-10	3.44E-11	0.19598	6E-05
-0.05124	-0.52099	-0.47512	0.517632	0.347567	0.359456	0.474794	0.526057	-0.30189
0.567282	3.4E-10	1.66E-08	4.61E-10	6.24E-05	3.32E-05	1.7E-08	2.13E-10	0.000562
-0.23242	-0.59072	-0.56916	0.056469	0.812352	0.595761	0.37212	0.591174	-0.22994
0.008552	2.67E-13	2.92E-12	0.528312	4.63E-31	1.49E-13	1.65E-05	2.53E-13	0.009305
0.097355	-0.11496	-0.03357	-0.50457	-0.0364	-0.21165	-0.3876	0.146974	0.013695
0.276209	0.198107	0.707883	1.46E-09	0.684524	0.016907	6.73E-06	0.099167	0.878543
-0.10232	-0.53717	-0.59794	-0.13068	0.30104	0.102057	-0.02878	0.531419	-0.24379
0.252344	7.47E-11	1.15E-13	0.143096	0.000584	0.253564	0.748054	1.29E-10	0.005744
0.097124	0.000929	-0.19785	-0.26464	0.365856	0.059184	0.323329	-0.14291	0.1192
0.277355	0.991727	0.025767	0.002641	2.34E-05	0.50864	0.000209	0.108977	0.181943

ARL5A	ARMC10	ARMT1	ASB1	ASB3	ASB6	ASB9	ASNSD1	ASTE1
cyan	cyan	cyan	cyan	cyan	cyan	cyan	cyan	cyan
-0.1156	0.196241	-0.01215	0.089665	-0.05066	0.033853	-0.08293	0.021732	-0.0496
0.195577	0.027022	0.892153	0.316101	0.571666	0.705551	0.353946	0.808385	0.579767
-0.20851	0.276351	-0.04698	0.092886	0.02809	0.090108	-0.06253	0.09831	-0.05746
0.018652	0.001661	0.599901	0.298958	0.753904	0.313704	0.484942	0.271505	0.521104
0.07377	-0.00697	0.146508	-0.15921	0.335909	-0.10896	0.278457	0.173954	0.404026
0.409798	0.938035	0.100255	0.073791	0.000113	0.22267	0.001525	0.050474	2.47E-06
0.340357	0.428257	0.468952	-0.34889	0.363413	-0.45669	0.493578	0.46947	0.676133
9.03E-05	5.1E-07	2.68E-08	5.82E-05	2.68E-05	6.79E-08	3.72E-09	2.58E-08	2.73E-18
0.120535	0.43407	0.272843	-0.39862	0.392501	-0.18109	0.220344	0.580396	0.291152
0.177062	3.43E-07	0.001913	3.46E-06	5.02E-06	0.041603	0.0128	8.57E-13	0.000897
0.461776	0.750924	0.690188	-0.56869	0.491473	-0.58612	0.576809	0.814638	0.747883
4.64E-08	2.81E-24	2.82E-19	3.06E-12	4.43E-09	4.51E-13	1.27E-12	2.32E-31	5.41E-24
0.0742	0.546404	0.303336	-0.18772	0.203187	-0.25694	0.429393	0.513331	0.443352
0.407065	3.03E-11	0.000527	0.034563	0.021957	0.003545	4.72E-07	6.78E-10	1.79E-07
0.259363	0.251175	0.196845	-0.31875	-0.10607	-0.33839	0.032136	0.207617	0.322659
0.003234	0.004394	0.026544	0.00026	0.235294	9.97E-05	0.719845	0.019172	0.000216
0.050311	0.018214	0.069141	-0.128	-0.19764	-0.10293	0.420978	-0.0797	0.194079
0.574301	0.838938	0.43988	0.151548	0.025931	0.249509	8.3E-07	0.373063	0.028789
0.299082	0.125575	0.048626	-0.24484	-0.23953	-0.198	0.192974	-0.06717	0.181927
0.000636	0.159503	0.587208	0.005532	0.006682	0.025651	0.029729	0.453033	0.040652
0.366002	0.251073	0.176237	-0.32349	-0.09754	-0.33778	0.126911	0.037277	0.362418
2.32E-05	0.00441	0.04748	0.000207	0.275294	0.000103	0.155077	0.677352	2.83E-05
-0.51899	-0.22742	-0.62359	0.241393	-0.14915	0.32643	-0.0947	-0.44075	-0.12787
4.08E-10	0.010129	4.85E-15	0.006257	0.094206	0.00018	0.289572	2.15E-07	0.15195
-0.38812	-0.67105	-0.60965	0.395606	-0.25055	0.446088	-0.2417	-0.69663	-0.25524
6.52E-06	6.01E-18	2.81E-14	4.16E-06	0.004496	1.47E-07	0.006189	9.53E-20	0.003779
-0.18172	-0.62197	-0.54122	0.113378	-0.20621	0.512737	-0.34348	-0.56556	-0.42752
0.040881	5.98E-15	5.05E-11	0.204381	0.020023	7.15E-10	7.7E-05	4.27E-12	5.36E-07
-0.1409	-0.58172	-0.57271	0.212251	-0.46593	0.358189	-0.20701	-0.74679	-0.3043
0.114101	7.39E-13	1.99E-12	0.01659	3.39E-08	3.55E-05	0.019536	6.83E-24	0.000505
-0.20023	-0.22585	-0.37966	0.409858	-0.26458	0.218843	-0.41983	-0.34983	-0.44993
0.024005	0.010675	1.07E-05	1.71E-06	0.002648	0.01344	8.95E-07	5.54E-05	1.11E-07
-0.34343	-0.5229	-0.4483	0.431647	-0.05857	0.399273	-0.41834	-0.36628	-0.49019
7.72E-05	2.85E-10	1.25E-07	4.05E-07	0.513023	3.32E-06	9.87E-07	2.28E-05	4.93E-09
0.166899	0.291684	0.446069	-0.02701	0.269524	-0.20137	-0.02943	0.429889	-0.04265
0.060733	0.000877	1.47E-07	0.763068	0.002182	0.023196	0.742565	4.56E-07	0.633988
-0.26008	-0.1019	-0.06249	0.282274	-0.06082	0.204577	-0.38297	0.007865	-0.50218
0.003148	0.254307	0.48519	0.001303	0.496984	0.021048	8.84E-06	0.930072	1.8E-09
-0.41447	-0.23713	-0.13711	0.349079	0.281212	0.241107	0.087424	-0.02826	0.01835
1.27E-06	0.007268	0.124258	5.76E-05	0.001362	0.00632	0.328393	0.752463	0.837759

ATF1	ATG10	ATG12	ATG3	ATG4A	ATG4C	ATG5	ATG9A	ATL3
cyan	cyan	cyan	cyan	cyan	cyan	cyan	cyan	cyan
-0.03555	0.106907	-0.03901	0.23104	0.123916	0.220863	0.124277	0.047407	0.035352
0.691525	0.231579	0.66322	0.008965	0.165135	0.012585	0.163896	0.596625	0.693151
-0.15583	0.078234	0.034869	0.199931	0.092809	0.214066	0.151191	0.104614	0.011265
0.080218	0.381969	0.697139	0.024219	0.299362	0.015665	0.089742	0.241801	0.899972
0.302286	0.172025	0.407858	-0.05294	0.104883	-0.02675	0.083128	-0.43903	0.190437
0.000552	0.053126	1.94E-06	0.554422	0.240587	0.76526	0.352808	2.43E-07	0.031988
0.565453	0.342322	0.642673	0.19429	0.429325	0.312404	0.453429	-0.71115	0.567537
4.32E-12	8.17E-05	3.79E-16	0.028612	4.74E-07	0.000349	8.64E-08	7.41E-21	3.46E-12
0.325096	0.458151	0.404093	0.668172	0.569579	0.234242	0.578264	-0.3926	0.15455
0.000192	6.09E-08	2.46E-06	9.35E-18	2.79E-12	0.008034	1.08E-12	4.99E-06	0.082757
0.697918	0.647191	0.808973	0.678313	0.790473	0.521699	0.854861	-0.84299	0.663582
7.64E-20	2.02E-16	1.26E-30	1.93E-18	2.19E-28	3.19E-10	2E-37	1.85E-35	1.87E-17
0.281297	0.25867	0.420436	0.361681	0.530474	0.251204	0.519494	-0.45613	0.336183
0.001357	0.003321	8.6E-07	2.94E-05	1.41E-10	0.004389	3.9E-10	7.08E-08	0.000111
0.203547	0.369314	0.102039	0.338111	0.413057	0.196833	0.231936	-0.25755	0.274931
0.021718	1.93E-05	0.253648	0.000101	1.39E-06	0.026554	0.008695	0.003465	0.001759
0.368215	0.130537	-0.01106	-0.00156	0.284063	-0.22538	-0.00646	-0.16808	0.507446
2.05E-05	0.143522	0.901781	0.986152	0.00121	0.010845	0.942572	0.058902	1.14E-09
0.294921	0.291234	-0.10284	0.517957	0.500396	0.155292	0.104071	-0.17308	0.552093
0.000763	0.000894	0.249942	4.48E-10	2.09E-09	0.081274	0.244267	0.051663	1.72E-11
0.332578	0.266387	0.070005	0.434254	0.424494	0.255306	0.234409	-0.33065	0.553116
0.000133	0.002468	0.434171	3.38E-07	6.57E-07	0.003769	0.007987	0.000147	1.55E-11
-0.40514	-0.53895	-0.41497	-0.49262	-0.39799	-0.42857	-0.52474	0.466577	-0.21515
2.3E-06	6.29E-11	1.23E-06	4.03E-09	3.59E-06	4.99E-07	2.41E-10	3.22E-08	0.015132
-0.29567	-0.43768	-0.49034	-0.58864	-0.50973	-0.47115	-0.78924	0.411974	-0.26352
0.000738	2.67E-07	4.87E-09	3.38E-13	9.32E-10	2.26E-08	3.04E-28	1.49E-06	0.002758
-0.16845	-0.25951	-0.50794	-0.16125	-0.31524	-0.41599	-0.59888	0.48555	-0.26744
0.058342	0.003217	1.09E-09	0.070119	0.000306	1.15E-06	1.03E-13	7.21E-09	0.002368
-0.16509	-0.22594	-0.63446	-0.10317	-0.15819	-0.31536	-0.69342	0.443073	0.050658
0.06362	0.010644	1.16E-15	0.248402	0.075683	0.000305	1.64E-19	1.82E-07	0.57166
-0.58668	-0.27471	-0.35444	-0.00413	-0.30286	0.215852	-0.37221	0.483626	-0.38249
4.23E-13	0.001774	4.34E-05	0.96326	0.000538	0.014798	1.64E-05	8.43E-09	9.09E-06
-0.61832	-0.54136	-0.30483	-0.58289	-0.71163	-0.29728	-0.51353	0.456593	-0.7394
9.52E-15	4.98E-11	0.000493	6.48E-13	6.8E-21	0.000689	6.66E-10	6.84E-08	3.19E-23
-0.05811	-0.05535	0.381732	-0.21249	-0.22491	0.20001	0.395665	-0.1337	-0.28267
0.516354	0.536513	9.5E-06	0.016464	0.011017	0.024162	4.14E-06	0.133984	0.001282
-0.5295	-0.36668	-0.19142	-0.41766	-0.54194	-0.15103	-0.05669	0.404586	-0.70612
1.55E-10	2.23E-05	0.031095	1.03E-06	4.71E-11	0.090084	0.526722	2.39E-06	1.83E-20
-0.04426	-0.0594	0.107288	-0.15983	-0.10651	-0.15382	-0.18066	-0.08685	-0.38958
0.621266	0.507127	0.22991	0.072656	0.233335	0.084228	0.042092	0.331601	5.98E-06

ATN1	ATP13A2	ATP13A3	ATP5S	ATP6V0E2	ATRAID	AVEN	B2M	B3GNT5
cyan	cyan	cyan	cyan	cyan	cyan	cyan	cyan	cyan
-0.14714	-0.06372	-0.1953	-0.06715	-0.08256	0.002137	0.099129	0.167013	-0.151
0.098777	0.476655	0.027781	0.45317	0.356144	0.980976	0.267508	0.060554	0.090155
-0.12042	-0.05038	-0.23312	-0.00285	-0.0016	0.011664	0.145281	0.015177	-0.22506
0.177474	0.573796	0.008349	0.974615	0.985771	0.896449	0.103163	0.865521	0.010961
-0.0584	-0.35844	0.329115	0.217421	-0.24364	0.1899	0.116926	0.191302	0.223488
0.514249	3.51E-05	0.000158	0.014072	0.005775	0.032484	0.190482	0.031202	0.011546
-0.46839	-0.74285	0.694276	0.337297	-0.65949	0.425198	0.319513	0.500138	0.591104
2.8E-08	1.56E-23	1.42E-19	0.000105	3.43E-17	6.26E-07	0.000251	2.14E-09	2.55E-13
-0.50195	-0.41511	-0.08811	0.454306	-0.09329	0.65081	0.333657	0.294018	-0.16116
1.83E-09	1.22E-06	0.324605	8.1E-08	0.296841	1.21E-16	0.000126	0.000793	0.070287
-0.80739	-0.91599	0.571134	0.536902	-0.70078	0.75374	0.522018	0.657515	0.482989
2.01E-30	2.04E-51	2.36E-12	7.67E-11	4.66E-20	1.52E-24	3.1E-10	4.59E-17	8.87E-09
-0.50782	-0.60321	0.439018	0.265743	-0.38799	0.559346	0.447159	0.461741	0.226155
1.1E-09	6.14E-14	2.43E-07	0.002531	6.57E-06	8.18E-12	1.36E-07	4.65E-08	0.010568
-0.2271	-0.31387	0.119861	0.007885	-0.24371	0.256124	-0.14847	0.328446	0.371465
0.010239	0.000326	0.179514	0.929892	0.005761	0.003655	0.095745	0.000163	1.71E-05
-0.19265	-0.23931	0.444405	-0.39389	-0.4989	0.328075	0.296755	0.335172	0.427572
0.03001	0.006735	1.66E-07	4.61E-06	2.38E-09	0.000166	0.000704	0.000117	5.34E-07
-0.27978	-0.2901	0.155369	-0.25073	-0.38078	0.405874	0.280216	0.603207	0.318356
0.001444	0.000939	0.081121	0.004466	1E-05	2.2E-06	0.001419	6.14E-14	0.000265
-0.30686	-0.36743	0.218727	-0.07847	-0.44848	0.335444	0.169191	0.659624	0.464516
0.00045	2.14E-05	0.013491	0.380505	1.24E-07	0.000116	0.057228	3.37E-17	3.77E-08
0.327362	0.376956	-0.22531	-0.42319	0.214778	-0.12574	0.039838	-0.37604	-0.07496
0.000172	1.25E-05	0.01087	7.16E-07	0.015314	0.158959	0.656545	1.32E-05	0.40226
0.663049	0.437636	-0.14847	-0.45302	0.284047	-0.39431	-0.26135	-0.29622	-0.12281
2.02E-17	2.67E-07	0.095742	8.9E-08	0.001211	4.5E-06	0.002999	0.000721	0.168975
0.561093	0.568201	-0.48242	-0.27049	0.545458	-0.11632	-0.29715	-0.3	-0.35818
6.82E-12	3.23E-12	9.29E-09	0.0021	3.33E-11	0.192788	0.000692	0.000611	3.56E-05
0.473338	0.388838	-0.2614	-0.60054	0.200873	-0.1186	-0.12922	0.005826	-0.04288
1.91E-08	6.25E-06	0.002992	8.46E-14	0.023545	0.184152	0.147637	0.94817	0.632206
0.280189	0.447862	-0.64757	-0.10562	0.538736	-0.43605	-0.19513	-0.31411	-0.47114
0.00142	1.29E-07	1.91E-16	0.237286	6.42E-11	2.99E-07	0.027917	0.000323	2.27E-08
0.60153	0.613618	-0.50603	0.042736	0.717282	-0.6222	-0.47058	-0.61485	-0.50218
7.51E-14	1.72E-14	1.29E-09	0.633313	2.4E-21	5.81E-15	2.37E-08	1.47E-14	1.8E-09
-0.15973	0.008034	0.10557	0.461194	0.057341	-0.30687	-0.15386	-0.272	-0.06353
0.072848	0.928567	0.2375	4.85E-08	0.52195	0.00045	0.084164	0.001978	0.477943
0.259265	0.548034	-0.38095	0.085035	0.528318	-0.56576	-0.37681	-0.61869	-0.48973
0.003246	2.58E-11	9.94E-06	0.341836	1.73E-10	4.18E-12	1.26E-05	9.09E-15	5.12E-09
0.036523	-0.05949	-0.01258	0.07009	0.078735	-0.11292	0.195398	-0.04492	-0.44953
0.68352	0.506457	0.888371	0.433612	0.378915	0.20624	0.027699	0.616069	1.15E-07

B4GALNT4	BAG2	BAHCC1	BAHD1	BBS10	BCAP29	BCL10	BCR	BDH2
cyan	cyan	cyan	cyan	cyan	cyan	cyan	cyan	cyan
-0.30351	0.21346	-0.24693	-0.02586	-0.14073	-0.13392	-0.02176	-0.2001	0.13436
0.000523	0.015969	0.005131	0.772854	0.114539	0.133334	0.808102	0.024093	0.132061
-0.27665	0.202477	-0.28259	-0.01234	-0.10927	-0.06312	-0.03977	-0.16357	0.1638
0.001641	0.022434	0.001286	0.890455	0.221379	0.48083	0.657093	0.066131	0.065749
0.10099	-0.03543	0.054474	0.041283	0.332236	0.108271	0.392625	-0.19311	-0.17308
0.258588	0.692469	0.543008	0.644922	0.000136	0.225645	4.98E-06	0.029615	0.05166
-0.37488	0.264883	-0.4141	-0.22518	0.618639	0.480366	0.573024	-0.52408	0.219837
1.41E-05	0.002617	1.3E-06	0.010917	9.15E-15	1.1E-08	1.92E-12	2.56E-10	0.013013
-0.41243	0.50381	-0.53506	-0.39647	0.207706	0.091095	0.207151	-0.47267	0.31147
1.45E-06	1.56E-09	9.14E-11	3.94E-06	0.019119	0.308415	0.019449	2.01E-08	0.000365
-0.7463	0.663682	-0.83633	-0.51363	0.650391	0.568656	0.63231	-0.80488	0.513153
7.57E-24	1.84E-17	1.99E-34	6.6E-10	1.28E-16	3.07E-12	1.55E-15	4.14E-30	6.89E-10
-0.56374	0.312873	-0.57942	-0.25117	0.28643	0.331778	0.270764	-0.4364	0.380652
5.17E-12	0.000342	9.55E-13	0.004394	0.001096	0.000139	0.002078	2.92E-07	1.01E-05
-0.41717	0.392866	-0.31919	-0.31654	0.174749	0.196875	0.039747	-0.36792	0.340554
1.07E-06	4.91E-06	0.000254	0.000288	0.049414	0.026521	0.657282	2.09E-05	8.94E-05
-0.21308	-0.03716	-0.03518	-0.08098	-0.09282	-0.05773	0.470639	-0.25237	-0.07312
0.016161	0.678295	0.6946	0.365436	0.299297	0.519117	2.36E-08	0.004203	0.413924
-0.51038	0.17355	-0.23549	-0.24857	-0.25423	-0.17322	0.276014	-0.45038	0.274307
8.81E-10	0.051021	0.007694	0.004834	0.003924	0.05147	0.001684	1.08E-07	0.001804
-0.48081	0.258779	-0.32796	-0.20077	-0.02064	0.081903	0.245334	-0.46205	0.294247
1.06E-08	0.003307	0.000167	0.023618	0.817815	0.359973	0.005435	4.54E-08	0.000785
0.384822	-0.47625	0.353321	0.445337	-0.31101	-0.32969	-0.23739	0.420747	-0.15498
7.93E-06	1.52E-08	4.61E-05	1.55E-07	0.000372	0.000154	0.007203	8.42E-07	0.081888
0.657854	-0.64335	0.802241	0.508576	-0.41253	-0.53266	-0.17575	0.48383	-0.51415
4.36E-17	3.45E-16	8.77E-30	1.03E-09	1.44E-06	1.15E-10	0.0481	8.29E-09	6.3E-10
0.643934	-0.42101	0.692775	0.279467	-0.48187	-0.58326	-0.26357	0.370338	-0.40923
3.18E-16	8.28E-07	1.83E-19	0.001463	9.71E-09	6.22E-13	0.002754	1.82E-05	1.78E-06
0.331565	-0.43451	0.615103	0.255888	-0.66826	-0.66601	-0.12809	0.216421	-0.23854
0.00014	3.32E-07	1.43E-14	0.003688	9.22E-18	1.3E-17	0.151259	0.014531	0.006919
0.223897	-0.19178	0.272819	0.10834	-0.36463	-0.31687	-0.61163	0.307841	-0.02406
0.011391	0.030773	0.001914	0.22535	2.5E-05	0.000284	2.2E-14	0.00043	0.788295
0.778699	-0.49578	0.613498	0.411154	-0.18874	-0.24246	-0.54688	0.68369	-0.44233
4.49E-27	3.09E-09	1.74E-14	1.57E-06	0.033577	0.006023	2.89E-11	8.17E-19	1.92E-07
-0.00529	0.178418	-0.28204	-0.11506	0.554104	0.601485	-0.12207	0.11533	0.031569
0.952924	0.044759	0.001316	0.197698	1.4E-11	7.55E-14	0.171563	0.196645	0.72459
0.300897	-0.12851	0.111231	0.140101	-0.02373	0.114386	-0.53751	0.54152	-0.18072
0.000587	0.149892	0.213136	0.116182	0.791135	0.20036	7.23E-11	4.9E-11	0.04203
0.257222	-0.18273	0.267795	0.222476	-0.02451	-0.3812	0.11372	0.038811	-0.36665
0.003507	0.039756	0.002336	0.011938	0.784422	9.8E-06	0.20301	0.66486	2.24E-05

BET1	BFAR	BIRC2	BNIP1	BNIP3	BNIP3L	BOD1	BPGM	BPNT1
cyan	cyan	cyan	cyan	cyan	cyan	cyan	cyan	cyan
-0.00826	-0.08256	-0.08758	-0.11567	0.076727	0.211116	-0.12764	-0.05383	-0.23515
0.926555	0.356138	0.327511	0.195339	0.391231	0.017193	0.152709	0.547811	0.007787
-0.0245	-0.05802	-0.15305	-0.06138	0.181326	0.212444	-0.09343	0.010805	-0.23665
0.784512	0.517029	0.085829	0.493004	0.041333	0.01649	0.296138	0.904034	0.007391
0.196161	0.435997	0.459109	0.326976	0.002829	0.096309	0.430183	0.355419	0.263488
0.027085	3E-07	5.67E-08	0.000175	0.97482	0.281425	4.47E-07	4.12E-05	0.002762
0.611875	0.639188	0.743855	0.376158	0.165431	0.490335	0.501339	0.507437	0.34089
2.13E-14	6.12E-16	1.27E-23	1.31E-05	0.063069	4.87E-09	1.93E-09	1.14E-09	8.79E-05
0.431088	0.411404	0.195938	0.743626	0.342113	0.166362	0.678011	0.305607	0.470563
4.2E-07	1.55E-06	0.027263	1.33E-23	8.26E-05	0.061579	2.03E-18	0.000476	2.37E-08
0.871009	0.793753	0.767138	0.703503	0.394007	0.605774	0.74795	0.58519	0.565376
2.12E-40	9.13E-29	7.3E-26	2.9E-20	4.58E-06	4.51E-14	5.33E-24	5.01E-13	4.36E-12
0.538732	0.586122	0.405086	0.3976	0.178355	0.345967	0.471499	0.457376	0.189458
6.43E-11	4.51E-13	2.31E-06	3.68E-06	0.044834	6.78E-05	2.2E-08	6.45E-08	0.032898
0.336389	0.032723	0.177056	0.020941	0.005041	0.170115	0.116242	-0.09274	0.27038
0.00011	0.714946	0.046443	0.815226	0.955145	0.055863	0.193105	0.299723	0.002109
0.262913	0.378964	0.429581	-0.06351	-0.16713	0.176738	0.09287	0.096867	-0.10317
0.002824	1.12E-05	4.66E-07	0.478101	0.06037	0.046842	0.299042	0.278632	0.248404
0.264295	0.098776	0.269168	-0.06064	-0.25361	0.102063	-0.07324	-0.07062	-0.10454
0.002677	0.269227	0.002213	0.498261	0.004015	0.253537	0.413194	0.430141	0.242141
0.385525	0.170331	0.33696	-0.02435	-0.17088	0.191688	0.002101	0.09358	-0.07554
7.61E-06	0.055548	0.000107	0.78583	0.054755	0.030857	0.981296	0.295346	0.398615
-0.42308	-0.14882	-0.35647	-0.15432	-0.25344	-0.18952	-0.15544	-0.02471	-0.55146
7.21E-07	0.094946	3.9E-05	0.083211	0.00404	0.032839	0.080983	0.782694	1.83E-11
-0.57606	-0.36956	-0.25244	-0.45649	-0.46446	-0.4029	-0.37561	-0.27137	-0.44142
1.38E-12	1.9E-05	0.004192	6.89E-08	3.79E-08	2.65E-06	1.35E-05	0.002028	2.05E-07
-0.46023	-0.45308	-0.39345	-0.15801	-0.27428	-0.52349	-0.17359	-0.36759	-0.1506
5.21E-08	8.86E-08	4.74E-06	0.07602	0.001805	2.71E-10	0.05097	2.12E-05	0.091016
-0.4273	-0.44801	-0.24292	-0.50492	-0.54432	-0.41071	-0.48049	-0.43212	-0.45334
5.44E-07	1.28E-07	0.005927	1.42E-09	3.73E-11	1.62E-06	1.08E-08	3.92E-07	8.69E-08
-0.55401	-0.62665	-0.64191	-0.4242	-0.15239	-0.35504	-0.52453	-0.36717	-0.25324
1.41E-11	3.26E-15	4.21E-16	6.69E-07	0.087189	4.21E-05	2.46E-10	2.17E-05	0.004071
-0.6805	-0.47995	-0.57913	-0.23742	-0.10923	-0.47161	-0.27785	-0.13627	-0.12849
1.37E-18	1.13E-08	9.86E-13	0.007195	0.221541	2.19E-08	0.001563	0.126593	0.149948
0.129875	0.089146	-0.01077	0.149729	0.405055	0.218545	0.095717	0.167835	0.285949
0.14558	0.31892	0.904314	0.092925	2.32E-06	0.01357	0.284408	0.059281	0.001118
-0.39294	-0.36319	-0.55879	-0.16362	0.195272	-0.19484	-0.26191	-0.17721	-0.06821
4.89E-06	2.71E-05	8.67E-12	0.066055	0.027802	0.028155	0.002935	0.046245	0.446043
-0.29096	0.131276	0.041551	0.264954	-0.09615	-0.10109	0.238399	0.065437	-0.02027
0.000905	0.14125	0.642771	0.00261	0.2822	0.258133	0.006953	0.464823	0.821062

BRD9	BRIX1	BRSK1	BSG	BTF3L4	C11orf54	C11orf58	C11orf65	C12orf45
cyan	cyan	cyan	cyan	cyan	cyan	cyan	cyan	cyan
-0.01246	-0.04039	0.050834	-0.32261	0.025179	-0.05532	-0.05988	-0.02616	0.125082
0.88939	0.652079	0.570327	0.000216	0.778715	0.536772	0.503681	0.770366	0.161163
-0.04732	-0.02421	0.113417	-0.26592	0.107222	0.049052	-0.04613	-0.00491	0.178982
0.59731	0.787005	0.204223	0.002514	0.230198	0.583929	0.606527	0.956301	0.044075
-0.02382	0.557271	-0.36722	0.034004	0.356071	0.283528	0.178917	0.309559	0.274145
0.790364	1.01E-11	2.17E-05	0.704298	3.98E-05	0.001237	0.044154	0.000398	0.001815
-0.40077	0.663416	-0.67964	-0.41403	0.47617	0.436773	0.471377	0.428167	0.310045
3.03E-06	1.92E-17	1.57E-18	1.31E-06	1.53E-08	2.84E-07	2.23E-08	5.13E-07	0.000389
-0.14304	0.489992	-0.24734	-0.25128	0.518979	0.440249	0.461691	0.308576	0.712978
0.108646	5.01E-09	0.005054	0.004377	4.08E-10	2.23E-07	4.67E-08	0.000416	5.31E-21
-0.55599	0.805563	-0.78296	-0.66359	0.709395	0.602918	0.752037	0.536687	0.671154
1.16E-11	3.4E-30	1.54E-27	1.87E-17	1.02E-20	6.36E-14	2.21E-24	7.83E-11	5.92E-18
-0.36731	0.501598	-0.37857	-0.37024	0.40697	0.363675	0.387381	0.358227	0.455076
2.16E-05	1.89E-09	1.14E-05	1.83E-05	2.05E-06	2.64E-05	6.82E-06	3.55E-05	7.65E-08
-0.32895	0.026468	-0.35772	-0.45805	0.121337	0.004257	0.340728	0.22046	-0.00438
0.000159	0.767705	3.64E-05	6.14E-08	0.174178	0.962113	8.86E-05	0.012752	0.961007
0.004182	0.227656	-0.50609	0.025056	-0.22866	-0.08217	-0.03291	0.077959	0.106024
0.962786	0.010051	1.28E-09	0.779768	0.009716	0.358408	0.713408	0.383647	0.23548
-0.08295	0.034974	-0.50774	-0.36426	-0.2668	-0.07437	0.033864	0.049447	0.193962
0.353869	0.696272	1.11E-09	2.55E-05	0.002428	0.405989	0.705456	0.580906	0.028887
-0.23695	0.114817	-0.55968	-0.46085	-0.06473	-0.04226	0.164872	0.081645	0.133181
0.007314	0.198659	7.9E-12	4.98E-08	0.469674	0.637085	0.063976	0.361494	0.135519
0.241169	-0.3311	0.327092	0.467467	-0.43926	-0.08375	-0.54165	-0.30187	-0.33165
0.006307	0.000143	0.000174	3.01E-08	2.39E-07	0.349229	4.84E-11	0.000562	0.00014
0.502302	-0.28051	0.280408	0.550444	-0.53571	-0.32189	-0.62974	-0.21752	-0.37396
1.78E-09	0.001402	0.001408	2.03E-11	8.6E-11	0.000224	2.17E-15	0.014027	1.49E-05
0.540813	-0.33264	0.350786	0.709854	-0.50271	-0.32607	-0.42154	-0.28891	-0.14855
5.25E-11	0.000133	5.27E-05	9.37E-21	1.72E-09	0.000183	7.99E-07	0.000987	0.095548
0.467613	-0.41003	0.079385	0.368118	-0.77002	-0.43878	-0.57471	-0.27561	-0.25785
2.98E-08	1.69E-06	0.374984	2.06E-05	3.7E-26	2.47E-07	1.6E-12	0.001712	0.003425
0.217261	-0.511	0.637067	0.078272	-0.26593	-0.18064	-0.31035	-0.29533	-0.3159
0.014144	8.33E-10	8.16E-16	0.381735	0.002512	0.042115	0.000384	0.000749	0.000297
0.334209	-0.37553	0.765174	0.580983	-0.06961	-0.18263	-0.39251	-0.28828	-0.42724
0.000123	1.36E-05	1.15E-25	8.02E-13	0.436793	0.03987	5.02E-06	0.001014	5.46E-07
-0.31239	0.023106	0.225446	-0.10709	0.502189	0.164757	0.378401	0.002262	-0.1385
0.000349	0.796522	0.010821	0.230769	1.79E-09	0.064165	1.15E-05	0.979866	0.120439
0.034683	-0.43679	0.706843	0.241646	0.074576	-0.18323	-0.12163	-0.30099	-0.36699
0.698676	2.84E-07	1.61E-20	0.006201	0.404684	0.039214	0.17312	0.000585	2.2E-05
0.406552	0.370323	0.215969	0.174767	0.186278	0.298767	-0.19508	0.205309	0.279291
2.11E-06	1.82E-05	0.014743	0.049391	0.036004	0.000645	0.027958	0.020582	0.001473

C12orf60	C12orf66	C12orf75	C14orf119	C14orf28	C15orf40	C16orf72	C17orf58	C18orf21
cyan	cyan	cyan	cyan	cyan	cyan	cyan	cyan	cyan
0.051992	0.152444	-0.34138	0.220798	0.20197	0.171849	-0.09548	0.066878	-0.00024
0.561566	0.087084	8.57E-05	0.012612	0.02278	0.053373	0.285624	0.455026	0.997877
0.107433	0.172879	-0.34219	0.265349	0.261403	0.223194	-0.12083	0.144601	0.067211
0.22928	0.051938	8.23E-05	0.00257	0.002992	0.011659	0.176014	0.104803	0.452776
0.214211	-0.01513	0.435901	0.161921	0.102471	0.031694	0.386568	0.264564	0.399695
0.015593	0.865887	3.02E-07	0.068952	0.251632	0.723543	7.15E-06	0.002649	3.23E-06
0.408975	0.408326	0.472434	0.41011	0.318448	0.399182	0.686868	0.408184	0.462276
1.81E-06	1.88E-06	2.05E-08	1.68E-06	0.000264	3.34E-06	4.87E-19	1.9E-06	4.47E-08
0.531801	0.2478	0.361031	0.668265	0.32001	0.56554	0.205941	0.497404	0.68297
1.25E-10	0.004971	3.05E-05	9.22E-18	0.000245	4.28E-12	0.020187	2.7E-09	9.18E-19
0.690622	0.576208	0.573693	0.797153	0.492923	0.78041	0.762347	0.639648	0.762129
2.62E-19	1.36E-12	1.79E-12	3.62E-29	3.93E-09	2.93E-27	2.21E-25	5.74E-16	2.33E-25
0.419162	0.416943	0.258413	0.586557	0.231087	0.548192	0.460459	0.336609	0.480013
9.35E-07	1.08E-06	0.003353	4.29E-13	0.00895	2.54E-11	5.12E-08	0.000109	1.13E-08
0.185306	0.263295	0.087128	0.105915	-0.16411	0.292753	0.094341	0.056188	0.004737
0.037002	0.002782	0.330043	0.235963	0.065235	0.000838	0.291418	0.530366	0.957851
-0.04757	-0.12305	0.138711	0.188306	-0.07312	-0.07717	0.405923	0.038353	0.050989
0.595365	0.168133	0.119883	0.033996	0.413956	0.388525	2.19E-06	0.66858	0.569148
0.105604	0.058793	-0.04794	0.342632	-0.04768	0.160273	0.190767	-0.13328	0.016409
0.237349	0.511447	0.5925	8.04E-05	0.594544	0.071863	0.031686	0.135237	0.854717
0.14086	0.169947	-0.01094	0.335252	-0.01232	0.274579	0.266141	-0.0245	-0.0218
0.114197	0.056108	0.90282	0.000117	0.890615	0.001784	0.002492	0.784534	0.807775
-0.33506	-0.23751	-0.32926	-0.20612	-0.20197	-0.44597	-0.35855	-0.28504	-0.35101
0.000118	0.007173	0.000157	0.020076	0.02278	1.48E-07	3.48E-05	0.001162	5.21E-05
-0.42707	-0.45428	-0.27182	-0.51676	-0.34571	-0.63973	-0.34523	-0.40909	-0.41684
5.52E-07	8.11E-08	0.001992	4.99E-10	6.86E-05	5.68E-16	7.04E-05	1.79E-06	1.09E-06
-0.27777	-0.4593	-0.19899	-0.36648	-0.28753	-0.47609	-0.51627	-0.26724	-0.27262
0.001568	5.59E-08	0.024912	2.26E-05	0.001046	1.54E-08	5.21E-10	0.002387	0.00193
-0.37635	-0.42968	-0.35648	-0.31216	-0.38252	-0.49119	-0.37648	-0.50262	-0.46887
1.3E-05	4.63E-07	3.9E-05	0.000353	9.07E-06	4.54E-09	1.29E-05	1.73E-09	2.7E-08
-0.23791	-0.04344	-0.44718	-0.28938	-0.07984	-0.18492	-0.66501	-0.4016	-0.4042
0.007072	0.627764	1.36E-07	0.000967	0.372249	0.037403	1.51E-17	2.87E-06	2.44E-06
-0.37978	-0.35902	-0.21448	-0.57381	-0.28233	-0.53044	-0.58317	-0.30634	-0.34628
1.06E-05	3.4E-05	0.015462	1.76E-12	0.0013	1.42E-10	6.29E-13	0.00046	6.67E-05
0.050847	0.238752	0.159793	-0.11161	0.243911	0.165449	0.118705	0.207324	0.081725
0.570229	0.006867	0.07273	0.211578	0.00572	0.063039	0.183778	0.019346	0.361024
-0.27091	-0.1355	-0.22559	-0.41182	-0.11717	-0.19716	-0.41867	-0.13283	-0.27572
0.002066	0.128772	0.010769	1.51E-06	0.189559	0.026303	9.66E-07	0.136553	0.001704
-0.01499	-0.11317	0.141542	0.039379	0.188307	-0.19979	0.002615	0.090731	0.340286
0.867147	0.205199	0.112438	0.660258	0.033994	0.02432	0.976724	0.310356	9.06E-05

C1GALT1	C1GALT1C	C1GALT1C	C1orf174	C1orf53	C1orf54	C1QTNF4	C2orf76	C3orf38
cyan	cyan	cyan	cyan	cyan	cyan	cyan	cyan	cyan
0.058029	0.194166	-0.08937	0.031815	-0.15162	0.140911	-0.11973	0.08472	-0.02587
0.516961	0.028715	0.317688	0.722527	0.08882	0.114067	0.179985	0.343634	0.772803
0.043319	0.193501	-0.07087	0.104874	-0.09454	0.128207	-0.07182	0.168512	-0.08143
0.62868	0.029277	0.42853	0.240624	0.290393	0.15087	0.422324	0.058248	0.362785
0.093999	0.051748	0.352363	0.366525	0.221667	0.037676	-0.32701	0.247687	0.309974
0.293182	0.563406	4.85E-05	2.25E-05	0.012259	0.67409	0.000175	0.004991	0.00039
0.44576	0.447446	0.402558	0.438245	0.305597	0.343331	-0.69195	0.416837	0.54397
1.51E-07	1.33E-07	2.71E-06	2.56E-07	0.000476	7.76E-05	2.1E-19	1.09E-06	3.86E-11
-0.00361	0.607524	0.401972	0.489997	0.529497	0.63769	-0.10161	0.72324	0.446639
0.96788	3.64E-14	2.81E-06	5.01E-09	1.55E-10	7.5E-16	0.255656	7.82E-22	1.41E-07
0.480736	0.875064	0.515538	0.605648	0.539346	0.75389	-0.70479	0.791055	0.774621
1.06E-08	3.28E-41	5.57E-10	4.58E-14	6.06E-11	1.47E-24	2.31E-20	1.88E-28	1.22E-26
0.20075	0.606328	0.450288	0.33277	0.211538	0.585045	-0.52406	0.54315	0.421827
0.023632	4.21E-14	1.09E-07	0.000132	0.016967	5.09E-13	2.57E-10	4.18E-11	7.84E-07
0.374555	0.446645	0.224773	0.03117	0.300603	0.247879	-0.17728	0.258005	0.145918
1.44E-05	1.41E-07	0.011066	0.727929	0.000595	0.004957	0.046157	0.003405	0.101645
0.061592	0.22832	0.045343	0.064638	0.02642	0.278262	-0.5216	-0.12444	0.168514
0.491517	0.009829	0.61272	0.470303	0.768116	0.001537	3.22E-10	0.163343	0.058245
0.268862	0.462867	-0.00526	-0.03287	0.108431	0.539454	-0.48384	0.030353	0.17911
0.00224	4.27E-08	0.953158	0.713725	0.224957	5.99E-11	8.28E-09	0.734794	0.043922
0.410066	0.426924	-0.00872	0.042308	0.065951	0.456274	-0.52883	0.063491	0.25503
1.69E-06	5.58E-07	0.92253	0.636727	0.461315	7E-08	1.65E-10	0.478232	0.003808
-0.44179	-0.50292	-0.17033	-0.14514	-0.36238	-0.36956	0.165434	-0.44821	-0.502
2E-07	1.69E-09	0.055546	0.103504	2.83E-05	1.9E-05	0.063064	1.26E-07	1.82E-09
-0.25073	-0.67083	-0.20229	-0.23149	-0.29903	-0.536	0.172521	-0.53791	-0.50013
0.004466	6.23E-18	0.022559	0.008827	0.000638	8.36E-11	0.052433	6.96E-11	2.14E-09
-0.26695	-0.46726	-0.27485	-0.21542	0.057591	-0.23955	0.516081	-0.3259	-0.34423
0.002414	3.06E-08	0.001764	0.015002	0.520139	0.006679	5.3E-10	0.000185	7.41E-05
-0.07953	-0.3273	-0.33412	-0.35236	-0.16447	-0.1153	0.073542	-0.5034	-0.4279
0.37408	0.000173	0.000123	4.85E-05	0.06464	0.196776	0.41125	1.62E-09	5.22E-07
-0.07927	-0.31339	-0.26112	-0.3679	-0.29343	-0.26409	0.447897	-0.24837	-0.52595
0.375685	0.000334	0.003025	2.09E-05	0.000814	0.002699	1.29E-07	0.004869	2.16E-10
-0.42728	-0.75765	-0.13478	-0.25417	-0.29138	-0.67736	0.687238	-0.33477	-0.45174
5.44E-07	6.4E-25	0.130847	0.003933	0.000888	2.25E-18	4.58E-19	0.00012	9.77E-08
0.008372	-0.0598	0.018538	0.023155	-0.08167	-0.25549	0.254374	0.095055	0.187841
0.925575	0.504252	0.836113	0.796103	0.36137	0.003743	0.003903	0.287767	0.034447
-0.40143	-0.42507	-0.19626	-0.26981	-0.33057	-0.51845	0.687309	-0.22955	-0.29419
2.9E-06	6.32E-07	0.027005	0.002158	0.000147	4.28E-10	4.53E-19	0.009429	0.000788
-0.46859	-0.20561	0.276746	0.304788	-0.11623	-0.18958	0.042798	0.096397	0.013142
2.76E-08	0.020394	0.001634	0.000494	0.193148	0.032779	0.632821	0.280982	0.883415

C5orf15	C6orf203	C6orf52	C6orf62	C8orf48	C8orf59	C8orf76	C9orf135	C9orf50
cyan	cyan	cyan	cyan	cyan	cyan	cyan	cyan	cyan
0.00303	0.138225	-0.14487	0.034986	0.069633	0.166767	-0.06551	0.267129	-0.10767
0.973031	0.121197	0.104153	0.696171	0.436621	0.06094	0.464321	0.002397	0.228242
-0.06685	0.211371	-0.09501	0.002575	0.068719	0.204108	-0.02078	0.289004	-0.04878
0.455231	0.017056	0.288	0.977082	0.442681	0.021351	0.816609	0.000983	0.586039
0.220779	0.142072	0.363248	0.140027	0.190928	0.057401	0.295709	-0.17132	0.205024
0.01262	0.111085	2.7E-05	0.116376	0.031539	0.521517	0.000737	0.054126	0.020762
0.61061	0.263001	0.315746	0.526112	0.432799	0.292546	0.43676	0.142651	0.264988
2.5E-14	0.002814	0.000299	2.12E-10	3.74E-07	0.000845	2.84E-07	0.109623	0.002606
0.238148	0.675185	0.565345	0.206052	0.329965	0.633316	0.625332	0.354972	0.470322
0.007014	3.17E-18	4.37E-12	0.020119	0.000152	1.35E-15	3.87E-15	4.22E-05	2.42E-08
0.774784	0.643756	0.508446	0.662012	0.589082	0.654989	0.718317	0.455842	0.455592
1.18E-26	3.26E-16	1.04E-09	2.36E-17	3.22E-13	6.62E-17	1.98E-21	7.23E-08	7.36E-08
0.426209	0.377597	0.234423	0.303693	0.347471	0.631491	0.392069	0.248492	0.353103
5.85E-07	1.21E-05	0.007984	0.000519	6.27E-05	1.72E-15	5.15E-06	0.004847	4.66E-05
0.268856	0.177952	0.391386	0.389258	0.225208	0.075614	0.176437	0.383251	0.369947
0.00224	0.045328	5.37E-06	6.1E-06	0.010907	0.398161	0.047225	8.69E-06	1.86E-05
0.455021	-0.15968	-0.22978	0.215628	0.212106	0.085712	0.138159	0.010205	0.067958
7.68E-08	0.072937	0.009357	0.014904	0.016667	0.337994	0.121378	0.909338	0.44776
0.416694	0.0255	-0.17544	0.397746	0.149617	0.138758	0.097178	0.371284	0.169929
1.1E-06	0.775969	0.048507	3.65E-06	0.093173	0.119756	0.277087	1.73E-05	0.056136
0.477297	0.059044	-0.13347	0.552712	0.18096	0.152312	0.114208	0.258944	0.014668
1.4E-08	0.509647	0.134678	1.61E-11	0.041751	0.08736	0.201066	0.003286	0.869985
-0.30997	-0.39268	-0.30239	-0.2625	-0.13435	0.066793	-0.06886	-0.28475	-0.27995
0.000391	4.96E-06	0.00055	0.002869	0.132097	0.455598	0.441759	0.001176	0.001434
-0.44671	-0.49926	-0.23879	-0.386	-0.29807	-0.49147	-0.38545	-0.45281	-0.1528
1.41E-07	2.3E-09	0.006858	7.39E-06	0.000665	4.43E-09	7.64E-06	9.04E-08	0.086346
-0.45627	-0.26362	-0.04095	-0.34186	-0.24307	-0.37637	-0.18511	-0.27622	0.023948
7E-08	0.002748	0.647571	8.36E-05	0.005895	1.29E-05	0.037204	0.00167	0.789277
-0.23256	-0.43366	-0.36066	-0.14989	-0.2462	-0.40413	-0.33705	-0.11825	-0.05593
0.008512	3.52E-07	3.11E-05	0.092562	0.005269	2.45E-06	0.000107	0.185456	0.532283
-0.50661	-0.1366	-0.19601	-0.28782	-0.32862	-0.25424	-0.42889	0.125638	-0.2919
1.22E-09	0.125686	0.027208	0.001033	0.000162	0.003922	4.88E-07	0.159292	0.000869
-0.71999	-0.31436	-0.03624	-0.60423	-0.406	-0.40001	-0.43598	-0.45894	-0.28829
1.45E-21	0.000319	0.685828	5.43E-14	2.18E-06	3.17E-06	3E-07	5.74E-08	0.001013
0.002681	0.096872	0.064754	-0.038	-0.01568	0.010423	-0.03129	-0.09481	-0.25239
0.976139	0.278609	0.469503	0.671419	0.861083	0.907417	0.726922	0.288999	0.004202
-0.51181	-0.14617	-0.16542	-0.47832	-0.33135	-0.20025	-0.37431	-0.25042	-0.37255
7.76E-10	0.101048	0.063082	1.29E-08	0.000142	0.023988	1.46E-05	0.004517	1.61E-05
-0.27271	0.132034	0.245255	-0.47044	-0.0453	0.192228	0.15804	-0.21323	0.053871
0.001923	0.138946	0.00545	2.39E-08	0.613063	0.030379	0.075966	0.016086	0.547491

C9orf85	CA13	CABIN1	CACNA1A	CACNA1H	CADM4	CALCOCO	CAMTA2	CAPZA1
cyan	cyan	cyan	cyan	cyan	cyan	cyan	cyan	cyan
0.064301	-0.01498	-0.17823	0.052755	-0.21142	-0.30217	-0.02106	0.148313	-0.00066
0.472623	0.867264	0.044986	0.555831	0.01703	0.000555	0.814241	0.096093	0.994084
0.084192	-0.05749	-0.23356	0.044577	-0.18757	-0.22199	-0.00733	0.181319	-0.06718
0.346659	0.520876	0.008223	0.618743	0.03471	0.01213	0.934828	0.041341	0.452955
0.346302	0.337444	0.11154	-0.39526	0.117971	0.105204	-0.22336	-0.45306	0.289142
6.66E-05	0.000105	0.211863	4.25E-06	0.186522	0.239142	0.011595	8.87E-08	0.000977
0.50619	0.668164	-0.22146	-0.60526	-0.28789	-0.27998	-0.39393	-0.65432	0.630297
1.27E-09	9.36E-18	0.012341	4.8E-14	0.001031	0.001432	4.6E-06	7.3E-17	2.02E-15
0.470588	0.058657	-0.65781	-0.31725	-0.3647	-0.24056	-0.29801	-0.50663	0.251277
2.37E-08	0.512427	4.4E-17	0.000279	2.49E-05	0.006444	0.000667	1.22E-09	0.004377
0.720277	0.637384	-0.72434	-0.68462	-0.60673	-0.53909	-0.60109	-0.85532	0.772052
1.37E-21	7.82E-16	6.34E-22	7.02E-19	4.01E-14	6.21E-11	7.92E-14	1.66E-37	2.28E-26
0.370744	0.346012	-0.46069	-0.4346	-0.29725	-0.25415	-0.19481	-0.44276	0.39374
1.78E-05	6.76E-05	5.04E-08	3.3E-07	0.000689	0.003935	0.028184	1.86E-07	4.66E-06
0.052668	0.22248	-0.22083	-0.2142	-0.40746	-0.22368	-0.13232	-0.3949	0.285878
0.556477	0.011936	0.012597	0.0156	1.99E-06	0.011475	0.138098	4.34E-06	0.001122
0.10569	0.209159	0.16544	-0.2509	0.229905	-0.24704	-0.29683	-0.20959	0.532309
0.236964	0.018277	0.063054	0.004438	0.009317	0.00511	0.000702	0.018036	1.19E-10
0.245477	0.034481	-0.20624	-0.15453	-0.34856	-0.34718	-0.2838	-0.28417	0.564698
0.005407	0.700348	0.02	0.082788	5.92E-05	6.36E-05	0.001223	0.001204	4.68E-12
0.210399	0.221533	-0.21061	-0.30283	-0.4331	-0.43086	-0.3528	-0.34392	0.53497
0.017583	0.012313	0.017468	0.000539	3.66E-07	4.27E-07	4.74E-05	7.53E-05	9.22E-11
-0.412	-0.17059	0.521707	0.240625	0.440725	0.178695	0.361886	0.534162	-0.42457
1.49E-06	0.055169	3.18E-10	0.006429	2.15E-07	0.044421	2.91E-05	9.96E-11	6.53E-07
-0.31856	-0.25874	0.801304	0.225265	0.574709	0.502322	0.396002	0.37888	-0.34346
0.000262	0.003311	1.14E-29	0.010886	1.6E-12	1.77E-09	4.06E-06	1.12E-05	7.71E-05
-0.2599	-0.50531	0.462067	0.219167	0.379124	0.485507	0.243148	0.26843	-0.39854
0.00317	1.37E-09	4.54E-08	0.0133	1.11E-05	7.23E-09	0.005878	0.002278	3.47E-06
-0.2149	-0.40803	0.536001	0.206869	0.263391	0.281757	0.196769	0.259621	-0.06713
0.015256	1.92E-06	8.36E-11	0.019619	0.002772	0.001331	0.026604	0.003203	0.453353
-0.20433	-0.51721	0.085934	0.43564	-0.07349	0.393569	0.385376	0.480286	-0.43101
0.02121	4.79E-10	0.336739	3.07E-07	0.411591	4.7E-06	7.67E-06	1.1E-08	4.23E-07
-0.43997	-0.38621	0.506474	0.431792	0.472829	0.704142	0.489406	0.528999	-0.75099
2.27E-07	7.31E-06	1.24E-09	4.01E-07	1.99E-08	2.59E-20	5.26E-09	1.62E-10	2.78E-24
-0.07919	0.216365	-0.27929	0.071791	-0.07835	-0.09273	0.000974	0.094815	-0.20917
0.376161	0.014557	0.001474	0.422508	0.381288	0.299775	0.991327	0.288992	0.018271
-0.49841	-0.30742	0.028755	0.5274	0.246586	0.180299	0.362873	0.582219	-0.67725
2.48E-09	0.000438	0.748278	1.88E-10	0.005195	0.042516	2.76E-05	6.99E-13	2.29E-18
0.190664	-0.03803	0.13301	0.072652	0.406171	0.24968	0.223575	0.056968	-0.15038
0.03178	0.671237	0.136026	0.416948	2.16E-06	0.004642	0.011513	0.524671	0.0915

CASKIN2	CBFA2T3	CBWD1	CBWD2	CBWD5	CBWD6	CBX4	CBX6	CC2D1A
cyan	cyan	cyan	cyan	cyan	cyan	cyan	cyan	cyan
-0.3149	-0.0059	0.230834	0.110241	0.056248	0.062178	-0.185	0.147231	-0.24828
0.000311	0.947538	0.009028	0.217265	0.52993	0.487394	0.03732	0.098571	0.004886
-0.24812	0.030876	0.150545	0.05072	0.087057	0.105334	-0.20626	0.138487	-0.25058
0.004914	0.7304	0.091138	0.57119	0.330441	0.238558	0.019992	0.120488	0.004491
0.055036	-0.20117	-0.09893	0.227645	0.197668	0.224117	-0.03295	-0.54389	-0.0035
0.538845	0.023334	0.268464	0.010054	0.025907	0.011309	0.713084	3.89E-11	0.968818
-0.41508	-0.59622	0.269801	0.448105	0.42212	0.377097	-0.44434	-0.75407	-0.41741
1.22E-06	1.41E-13	0.002158	1.27E-07	7.69E-07	1.24E-05	1.67E-07	1.42E-24	1.05E-06
-0.43597	-0.1355	0.423686	0.352288	0.402153	0.490162	-0.40492	-0.35506	-0.48763
3E-07	0.128782	6.93E-07	4.87E-05	2.78E-06	4.94E-09	2.34E-06	4.2E-05	6.08E-09
-0.76946	-0.68319	0.649576	0.647674	0.635915	0.633583	-0.74788	-0.8118	-0.81024
4.23E-26	8.86E-19	1.44E-16	1.88E-16	9.54E-16	1.31E-15	5.42E-24	5.46E-31	8.69E-31
-0.49108	-0.4116	0.336053	0.319679	0.391963	0.41748	-0.58232	-0.55357	-0.53376
4.58E-09	1.53E-06	0.000112	0.000249	5.18E-06	1.04E-06	6.92E-13	1.48E-11	1.03E-10
-0.35446	-0.38164	0.417062	0.154107	0.106238	0.164217	-0.40912	-0.20635	-0.31167
4.34E-05	9.55E-06	1.07E-06	0.083653	0.23453	0.065055	1.79E-06	0.019935	0.000361
-0.07015	-0.39047	0.158872	0.097783	-0.079	-0.08376	-0.05438	-0.443	-0.02214
0.433212	5.67E-06	0.074417	0.274095	0.377282	0.34916	0.54374	1.83E-07	0.804883
-0.32747	-0.48895	0.493786	0.14297	0.035193	0.04521	-0.24703	-0.30353	-0.24019
0.000171	5.46E-09	3.66E-09	0.108823	0.694461	0.613761	0.005112	0.000522	0.006528
-0.4349	-0.52042	0.507356	0.264402	0.091046	0.083256	-0.35515	-0.34381	-0.30642
3.23E-07	3.58E-10	1.15E-09	0.002666	0.308675	0.352064	4.18E-05	7.57E-05	0.000459
0.391943	0.257891	-0.56499	-0.57874	-0.51893	-0.57096	0.339487	0.162025	0.458476
5.19E-06	0.00342	4.54E-12	1.03E-12	4.1E-10	2.4E-12	9.44E-05	0.068772	5.94E-08
0.719021	0.299211	-0.60051	-0.39485	-0.42825	-0.41773	0.653894	0.159338	0.759117
1.74E-21	0.000633	8.48E-14	4.35E-06	5.1E-07	1.03E-06	7.76E-17	0.07356	4.61E-25
0.693982	0.41546	-0.3921	-0.36088	-0.38172	-0.37019	0.712832	0.306565	0.727297
1.49E-19	1.19E-06	5.14E-06	3.07E-05	9.51E-06	1.84E-05	5.46E-21	0.000456	3.58E-22
0.533049	0.068632	-0.20014	-0.36151	-0.42967	-0.42126	0.546773	0.159489	0.585798
1.11E-10	0.443257	0.024069	2.97E-05	4.63E-07	8.14E-07	2.92E-11	0.073283	4.68E-13
0.321526	0.292496	-0.18434	-0.40665	-0.18143	-0.15685	0.285896	0.624738	0.202903
0.000228	0.000847	0.038019	2.09E-06	0.041211	0.078236	0.001121	4.18E-15	0.022147
0.639279	0.64771	-0.66827	-0.45875	-0.32357	-0.26407	0.549259	0.572686	0.579065
6.04E-16	1.87E-16	9.21E-18	5.82E-08	0.000207	0.002701	2.28E-11	1.99E-12	9.93E-13
-0.24187	0.187783	-0.06387	0.112574	0.19153	0.104913	-0.22561	0.237863	-0.2718
0.006152	0.034504	0.475582	0.207631	0.030997	0.240452	0.010763	0.007085	0.001994
0.140423	0.608338	-0.31497	-0.25695	-0.19021	-0.18283	0.126199	0.710098	0.127908
0.115336	3.3E-14	0.00031	0.003544	0.032201	0.039652	0.157424	8.96E-21	0.15183
0.182728	0.355005	-0.37109	-0.03676	0.183214	0.180489	0.263493	-0.10516	0.096074
0.039761	4.22E-05	1.75E-05	0.681555	0.039228	0.042296	0.002761	0.239322	0.282606

CCDC121	CCDC126	CCDC149	CCDC160	CCDC58	CCDC59	CCDC66	CCDC85C	CCDC90B
cyan	cyan	cyan	cyan	cyan	cyan	cyan	cyan	cyan
0.102437	-0.19761	0.156863	-0.09042	0.014519	0.069122	0.020053	0.046573	0.169664
0.251793	0.025949	0.078205	0.312015	0.871293	0.44	0.822931	0.603106	0.056526
0.062105	-0.16546	0.188147	-0.04049	-0.00309	0.066602	0.018961	0.073926	0.20567
0.487907	0.063023	0.034149	0.651266	0.972498	0.456891	0.83243	0.408809	0.020356
0.060527	0.299643	-0.37019	0.171216	0.420083	0.178012	0.191023	-0.09316	0.182003
0.499053	0.000621	1.84E-05	0.054271	8.8E-07	0.045254	0.031453	0.297503	0.040568
0.346692	0.534933	-0.48073	0.398562	0.572103	0.367457	0.45895	-0.42628	0.405164
6.53E-05	9.26E-11	1.06E-08	3.47E-06	2.12E-12	2.14E-05	5.74E-08	5.82E-07	2.3E-06
0.228006	0.196159	-0.3267	0.317416	0.590603	0.660655	0.213911	-0.34444	0.640584
0.009933	0.027087	0.000178	0.000277	2.7E-13	2.89E-17	0.015742	7.33E-05	5.05E-16
0.488038	0.598606	-0.61617	0.540501	0.807533	0.71745	0.528168	-0.65978	0.775721
5.88E-09	1.06E-13	1.25E-14	5.41E-11	1.92E-30	2.33E-21	1.75E-10	3.29E-17	9.35E-27
0.26909	0.211026	-0.20632	0.300649	0.544144	0.371265	0.401316	-0.48385	0.465084
0.00222	0.017242	0.019951	0.000594	3.79E-11	1.73E-05	2.93E-06	8.28E-09	3.61E-08
0.475703	0.064283	-0.26226	0.384426	0.135416	0.138965	0.460584	-0.39662	0.182956
1.59E-08	0.472749	0.002895	8.11E-06	0.12902	0.119199	5.08E-08	3.91E-06	0.03951
-0.21167	-0.03484	-0.21771	-0.24084	0.211199	0.163624	0.020459	-0.46672	0.0246
0.016895	0.697339	0.013939	0.00638	0.017148	0.066044	0.819404	3.19E-08	0.783678
0.04219	-0.28339	-0.32148	-0.02361	0.182392	0.241812	0.12102	-0.54296	0.280763
0.637666	0.001244	0.000228	0.79218	0.040133	0.006164	0.175312	4.26E-11	0.001387
0.195931	-0.06956	-0.33382	0.127509	0.217811	0.203156	0.150164	-0.33895	0.256393
0.027269	0.437125	0.000125	0.153122	0.013896	0.021977	0.091969	9.7E-05	0.003619
-0.37888	-0.4343	0.399972	-0.40183	-0.34876	-0.29195	-0.38328	0.430247	-0.42915
1.12E-05	3.37E-07	3.18E-06	2.83E-06	5.86E-05	0.000867	8.68E-06	4.45E-07	4.8E-07
-0.35098	-0.4476	0.260414	-0.36575	-0.33256	-0.44036	-0.20227	0.384208	-0.54019
5.22E-05	1.32E-07	0.003107	2.35E-05	0.000133	2.21E-07	0.022572	8.22E-06	5.58E-11
-0.28395	-0.40526	0.097386	-0.27905	-0.29618	-0.07349	-0.395	0.292246	-0.33363
0.001215	2.29E-06	0.276053	0.001488	0.000722	0.411583	4.31E-06	0.000856	0.000127
-0.29438	-0.67859	0.069622	-0.38331	-0.32235	-0.22535	-0.22086	0.048891	-0.32798
0.000781	1.85E-18	0.436697	8.66E-06	0.000219	0.010857	0.012585	0.58517	0.000167
-0.12168	-0.47148	0.324017	-0.0934	-0.49007	-0.41291	-0.1451	0.372922	-0.11346
0.17297	2.21E-08	0.000202	0.29626	4.98E-09	1.4E-06	0.103611	1.58E-05	0.204071
-0.24432	-0.15416	0.43066	-0.12533	-0.49088	-0.53461	-0.36611	0.717714	-0.46085
0.005636	0.083549	4.33E-07	0.160338	4.65E-09	9.55E-11	2.31E-05	2.22E-21	4.98E-08
0.146418	0.617777	0.131982	0.201989	-0.10939	-0.10676	-0.04849	0.236522	-0.01998
0.100466	1.02E-14	0.139103	0.022767	0.220855	0.232213	0.588231	0.007424	0.823587
-0.16757	0.071023	0.521788	-0.14672	-0.48733	-0.42658	-0.33288	0.527337	-0.36902
0.059689	0.427502	3.16E-10	0.099757	6.23E-09	5.71E-07	0.000131	1.89E-10	1.96E-05
-0.20139	-0.09212	0.250207	-0.15578	0.277817	0.056334	0.155701	0.171238	0.045511
0.023184	0.302957	0.004553	0.080302	0.001565	0.5293	0.080465	0.054239	0.6114

CCNB1IP1	CCNC	CCNG1	CCT4	CCT6B	CCZ1	CCZ1B	CD164	CD226
cyan	cyan	cyan	cyan	cyan	cyan	cyan	cyan	cyan
0.055942	0.035282	0.10573	-0.06774	0.138271	0.075706	0.162373	-0.01362	-0.0044
0.532177	0.693732	0.236787	0.449234	0.121073	0.397587	0.06817	0.879165	0.960824
0.190982	0.071434	0.104489	0.01096	0.205522	0.065836	0.192552	-0.09655	-0.16519
0.031491	0.424824	0.242368	0.902663	0.020448	0.4621	0.030095	0.280235	0.063466
0.410713	0.213776	0.094627	0.352988	0.102226	0.143251	0.253171	0.128843	0.168059
1.62E-06	0.01581	0.289955	4.69E-05	0.252773	0.108121	0.004081	0.148835	0.058938
0.375717	0.506621	0.485716	0.456406	0.409861	0.417744	0.420363	0.573826	0.490774
1.34E-05	1.22E-09	7.11E-09	6.93E-08	1.71E-06	1.03E-06	8.64E-07	1.76E-12	4.7E-09
0.475156	0.533011	0.45338	0.637416	0.605525	0.467265	0.43646	-0.00818	0.139498
1.66E-08	1.11E-10	8.67E-08	7.79E-16	4.65E-14	3.06E-08	2.9E-07	0.927251	0.117775
0.50627	0.828423	0.800687	0.751499	0.769412	0.687643	0.635051	0.620778	0.575092
1.26E-09	2.93E-33	1.36E-29	2.48E-24	4.27E-26	4.29E-19	1.07E-15	6.97E-15	1.54E-12
0.378407	0.477934	0.52628	0.428779	0.537319	0.448491	0.450571	0.248714	0.319644
1.15E-05	1.33E-08	2.09E-10	4.92E-07	7.37E-11	1.24E-07	1.06E-07	0.004808	0.000249
-0.21857	0.135946	0.229676	0.061902	0.367022	0.16307	-0.0515	0.378677	0.393617
0.013561	0.127515	0.009389	0.489335	2.19E-05	0.066978	0.565306	1.13E-05	4.69E-06
-0.11133	-0.07188	0.249695	-0.02985	-0.02363	0.133629	0.065797	0.447127	0.338594
0.212734	0.421899	0.004639	0.739015	0.792044	0.134196	0.462366	1.37E-07	9.87E-05
-0.33667	-0.05268	0.394742	-0.11519	0.219132	0.173657	0.079482	0.496291	0.590208
0.000109	0.556409	4.38E-06	0.197191	0.013315	0.050876	0.374399	2.96E-09	2.83E-13
-0.26199	0.10091	0.348722	0.007486	0.162363	0.223369	0.184295	0.521978	0.598713
0.002926	0.258968	5.87E-05	0.933433	0.068187	0.011592	0.038064	3.11E-10	1.05E-13
-0.15676	-0.53964	-0.44767	-0.23388	-0.40929	-0.2732	-0.27952	-0.374	-0.45047
0.078403	5.89E-11	1.31E-07	0.008135	1.77E-06	0.001886	0.00146	1.48E-05	1.07E-07
-0.21192	-0.72176	-0.56834	-0.51566	-0.53811	-0.46499	-0.36133	-0.31447	-0.21355
0.016763	1.04E-21	3.18E-12	5.5E-10	6.83E-11	3.64E-08	3E-05	0.000317	0.015922
-0.27067	-0.62208	-0.48012	-0.3656	-0.41866	-0.35757	-0.3282	-0.36184	-0.22668
0.002085	5.9E-15	1.12E-08	2.37E-05	9.66E-07	3.67E-05	0.000165	2.91E-05	0.010386
-0.54918	-0.76624	-0.31283	-0.61988	-0.38855	-0.36143	-0.37852	-0.02888	0.081645
2.3E-11	9.02E-26	0.000342	7.81E-15	6.36E-06	2.98E-05	1.14E-05	0.747237	0.361497
-0.29468	-0.35101	-0.29858	-0.36483	-0.19996	-0.33524	-0.27104	-0.39963	-0.38591
0.000771	5.21E-05	0.00065	2.47E-05	0.024201	0.000117	0.002055	3.25E-06	7.43E-06
0.016694	-0.34925	-0.69752	-0.22291	-0.52964	-0.45672	-0.33265	-0.7278	-0.65863
0.852226	5.71E-05	8.17E-20	0.011767	1.53E-10	6.77E-08	0.000133	3.25E-22	3.9E-17
0.261042	0.511115	0.017548	0.267415	-0.00113	0.054956	0.07119	-0.06582	-0.27558
0.003034	8.25E-10	0.844757	0.002371	0.989938	0.539437	0.426412	0.462174	0.001714
-0.00924	0.004012	-0.40753	-0.10273	-0.31987	-0.27342	-0.25409	-0.57722	-0.63095
0.917917	0.964293	1.98E-06	0.250413	0.000246	0.001869	0.003944	1.22E-12	1.85E-15
0.526128	0.007171	-0.14757	0.214375	0.034983	-0.04441	0.07004	-0.45922	-0.25908
2.12E-10	0.936229	0.097786	0.015512	0.696198	0.620051	0.433936	5.62E-08	0.00327

CD46	CD58	CDC16	CDC42	CDC42BPA	CDC42BPB	CDC5L	CDH4	CDK7
cyan	cyan	cyan	cyan	cyan	cyan	cyan	cyan	cyan
0.102319	-0.0416	-0.0111	0.080175	0.012855	0.05281	-0.214	-0.30556	0.098316
0.252339	0.642351	0.90145	0.370231	0.885944	0.555414	0.015699	0.000477	0.271471
0.070817	-0.04203	0.051114	0.100594	0.050406	0.062557	-0.20343	-0.26051	0.100502
0.428842	0.638928	0.568202	0.260468	0.573577	0.484743	0.021797	0.003096	0.260908
0.045652	0.284361	0.3387	0.372915	-0.19537	-0.19484	0.497794	0.264359	0.181918
0.610292	0.001195	9.82E-05	1.58E-05	0.027723	0.028157	2.61E-09	0.00267	0.040663
0.512986	0.656266	0.586306	0.610574	-0.03807	-0.40902	0.545374	-0.06854	0.428503
6.99E-10	5.5E-17	4.41E-13	2.51E-14	0.670864	1.8E-06	3.36E-11	0.443893	5.01E-07
0.297203	0.369345	0.358207	0.476669	-0.66665	-0.68607	0.3301	-0.42331	0.646598
0.000691	1.93E-05	3.55E-05	1.47E-08	1.18E-17	5.55E-19	0.000151	7.1E-07	2.19E-16
0.729367	0.830306	0.691182	0.830928	-0.37656	-0.80235	0.593461	-0.46996	0.779802
2.39E-22	1.56E-33	2.39E-19	1.27E-33	1.28E-05	8.5E-30	1.94E-13	2.48E-08	3.41E-27
0.510522	0.500349	0.56037	0.516548	-0.10753	-0.44051	0.192051	-0.33718	0.512599
8.69E-10	2.1E-09	7.36E-12	5.08E-10	0.228867	2.19E-07	0.030535	0.000106	7.23E-10
0.391469	0.38839	0.145982	0.186248	0.13098	-0.40065	0.16422	-0.35924	0.178854
5.34E-06	6.42E-06	0.101495	0.036034	0.142156	3.05E-06	0.065051	3.36E-05	0.04423
0.265184	0.326075	0.109266	0.210629	-0.08352	0.00793	-0.10685	-0.18535	0.094312
0.002586	0.000183	0.221383	0.017457	0.350531	0.929489	0.231831	0.036953	0.291571
0.424832	0.382265	0.04617	0.196812	-0.26451	-0.222	-0.30067	-0.5087	0.118864
6.42E-07	9.21E-06	0.606248	0.02657	0.002655	0.012127	0.000593	1.02E-09	0.183188
0.41802	0.437719	0.154301	0.272117	-0.13814	-0.22779	-0.05735	-0.36197	0.164605
1.01E-06	2.66E-07	0.083258	0.001969	0.121441	0.010006	0.521888	2.9E-05	0.064415
-0.37956	-0.37222	-0.16144	-0.31033	0.217369	0.694235	-0.34625	0.188451	-0.18363
1.08E-05	1.64E-05	0.069789	0.000384	0.014095	1.43E-19	6.68E-05	0.033855	0.038775
-0.49953	-0.38055	-0.28774	-0.48028	0.251655	0.607259	-0.31744	0.555294	-0.5482
2.25E-09	1.02E-05	0.001037	1.1E-08	0.004317	3.76E-14	0.000276	1.24E-11	2.54E-11
-0.54702	-0.34636	-0.50735	-0.50844	-0.29279	0.245555	-0.29948	0.371056	-0.41211
2.85E-11	6.64E-05	1.15E-09	1.04E-09	0.000836	0.005392	0.000625	1.75E-05	1.48E-06
-0.26581	-0.19545	-0.42226	-0.45872	-0.01573	0.404424	-0.60388	0.124925	-0.51385
0.002524	0.027656	7.62E-07	5.84E-08	0.860705	2.41E-06	5.67E-14	0.161693	6.47E-10
-0.24826	-0.49877	-0.30161	-0.40229	0.262887	0.391781	-0.41849	0.033592	-0.48599
0.004889	2.4E-09	0.000569	2.75E-06	0.002826	5.24E-06	9.77E-07	0.707716	6.95E-09
-0.69642	-0.67156	-0.349	-0.4392	0.191432	0.503855	-0.02923	0.594591	-0.4883
9.86E-20	5.56E-18	5.79E-05	2.4E-07	0.031085	1.55E-09	0.744224	1.7E-13	5.76E-09
0.000627	-0.13271	0.070926	0.11813	0.242324	-0.07203	0.442146	0.136611	0.118462
0.994414	0.136925	0.428131	0.185923	0.006053	0.420931	1.95E-07	0.125648	0.184684
-0.40534	-0.61324	-0.30497	-0.34657	0.294127	0.293385	-0.03827	0.199045	-0.21089
2.27E-06	1.8E-14	0.00049	6.57E-05	0.00079	0.000815	0.669292	0.024868	0.017315
-0.12876	-0.24247	0.189944	0.077515	-0.13018	0.09941	0.102108	0.233098	0.163048
0.149093	0.006022	0.032443	0.386373	0.14463	0.266151	0.253325	0.008356	0.067016

CDK9	CDKL3	CDKN2AIP	CEBPZ	CELSR2	CELSR3	CENPQ	CEP120	CEP170B
cyan	cyan	cyan	cyan	cyan	cyan	cyan	cyan	cyan
-0.01251	0.090794	-0.05572	-0.13442	-0.11266	-0.11995	-0.1261	0.102693	0.021777
0.888959	0.310019	0.533842	0.131875	0.207295	0.179203	0.157759	0.250601	0.807994
-0.03551	0.152216	-0.08059	-0.1021	-0.09036	-0.08812	-0.10174	0.045229	0.059594
0.691833	0.087563	0.367756	0.253342	0.312335	0.32455	0.255067	0.613615	0.505706
-0.23573	0.170213	0.219192	0.558639	-0.06215	0.06316	0.478945	0.088934	-0.37617
0.007631	0.05572	0.013289	8.8E-12	0.487582	0.480532	1.23E-08	0.320077	1.31E-05
-0.44066	0.409962	0.458308	0.737392	-0.37945	-0.37857	0.636023	0.535583	-0.68085
2.16E-07	1.7E-06	6.02E-08	4.81E-23	1.08E-05	1.14E-05	9.4E-16	8.7E-11	1.29E-18
-0.08838	0.352863	0.497086	0.242037	-0.60536	-0.42204	0.524179	0.256214	-0.28351
0.323117	4.72E-05	2.77E-09	0.006115	4.74E-14	7.73E-07	2.54E-10	0.003643	0.001238
-0.47721	0.570383	0.715805	0.686262	-0.78001	-0.70975	0.830066	0.709389	-0.77493
1.41E-08	2.56E-12	3.16E-21	5.38E-19	3.23E-27	9.54E-21	1.69E-33	1.02E-20	1.13E-26
-0.26017	0.427146	0.34063	0.47613	-0.46727	-0.52143	0.398184	0.476996	-0.51719
0.003137	5.49E-07	8.91E-05	1.53E-08	3.06E-08	3.27E-10	3.55E-06	1.43E-08	4.8E-10
0.155999	0.29151	0.182577	0.093824	-0.13569	-0.23142	0.188334	0.310304	-0.33511
0.079881	0.000884	0.039928	0.294085	0.128243	0.008851	0.033969	0.000385	0.000118
-0.2455	-0.08827	-0.13449	0.325013	-0.32881	-0.24768	-0.02958	0.193325	-0.36322
0.005403	0.323706	0.131696	0.000193	0.00016	0.004994	0.741332	0.029428	2.7E-05
0.070939	-0.09225	0.000675	0.046282	-0.47908	-0.43427	-0.07519	0.25912	-0.45049
0.428049	0.302318	0.993988	0.605374	1.21E-08	3.38E-07	0.400827	0.003264	1.07E-07
-0.08632	0.064458	0.148403	0.20216	-0.42268	-0.42007	0.087358	0.33832	-0.47431
0.334592	0.471544	0.095891	0.02265	7.41E-07	8.81E-07	0.328763	0.0001	1.77E-08
0.195267	0.157943	-0.511	0.07073	0.266967	0.270633	-0.45372	-0.3746	0.271323
0.027806	0.076149	8.34E-10	0.429413	0.002413	0.002088	8.45E-08	1.43E-05	0.002032
0.315736	-0.32971	-0.55127	-0.0795	0.587771	0.56823	-0.45469	-0.4673	0.245911
0.000299	0.000154	1.87E-11	0.374274	3.74E-13	3.22E-12	7.87E-08	3.05E-08	0.005323
0.377892	-0.44003	-0.30006	-0.32764	0.332511	0.403017	-0.33377	-0.54392	0.380031
1.19E-05	2.26E-07	0.000609	0.00017	0.000134	2.63E-06	0.000126	3.88E-11	1.05E-05
0.41906	-0.46626	-0.52911	-0.27945	0.240991	0.232074	-0.55937	-0.36629	0.095688
9.41E-07	3.3E-08	1.61E-10	0.001464	0.006346	0.008654	8.16E-12	2.28E-05	0.284553
0.357225	-0.25251	-0.33483	-0.57836	0.431406	0.298361	-0.42098	-0.37293	0.395257
3.74E-05	0.004183	0.000119	1.07E-12	4.11E-07	0.000657	8.29E-07	1.57E-05	4.25E-06
0.242481	-0.26621	-0.28533	-0.35111	0.71903	0.710291	-0.29768	-0.63846	0.581994
0.006019	0.002485	0.001148	5.18E-05	1.73E-21	8.66E-21	0.000677	6.75E-16	7.17E-13
-0.32194	0.152466	0.312716	-0.06359	0.104992	0.035983	0.273357	0.119137	0.245114
0.000223	0.087038	0.000344	0.477575	0.240096	0.687956	0.001874	0.182174	0.005478
0.080932	-0.14095	-0.12576	-0.51106	0.421006	0.376257	-0.27667	-0.32986	0.701561
0.365717	0.113953	0.158897	8.29E-10	8.28E-07	1.3E-05	0.00164	0.000152	4.07E-20
0.053813	0.133997	-0.03107	0.268681	0.112838	0.244555	0.087138	-0.16413	0.082938
0.547918	0.133118	0.728799	0.002256	0.206559	0.005589	0.329985	0.065201	0.353913

CEP44	CEP57	CEP57L1	CEP70	CEP76	CEPT1	CERS5	CES2	CETN3
cyan	cyan	cyan	cyan	cyan	cyan	cyan	cyan	cyan
-0.11067	-0.19131	-0.19043	-0.09668	-0.18737	0.063365	-0.16783	-0.02514	0.036041
0.215474	0.031194	0.031998	0.279557	0.034912	0.479105	0.059295	0.779041	0.687481
-0.09321	-0.20267	-0.17104	-0.04394	-0.1366	0.085469	-0.03833	-0.11844	0.007018
0.297281	0.0223	0.054524	0.623749	0.125673	0.339367	0.668759	0.184758	0.937586
0.447954	0.639268	0.554302	0.395429	0.552764	0.078683	0.393659	-0.26432	0.147186
1.29E-07	6.05E-16	1.37E-11	4.2E-06	1.6E-11	0.379236	4.68E-06	0.002675	0.098675
0.680135	0.618569	0.697221	0.509697	0.605571	0.51309	0.326195	-0.43967	0.345104
1.45E-18	9.23E-15	8.6E-20	9.35E-10	4.62E-14	6.93E-10	0.000182	2.32E-07	7.08E-05
0.310316	0.30336	0.401259	0.37844	0.305456	0.254126	0.41699	-0.45178	0.64532
0.000384	0.000526	2.94E-06	1.15E-05	0.000479	0.003939	1.08E-06	9.74E-08	2.62E-16
0.744412	0.599115	0.788035	0.619624	0.614484	0.706151	0.41216	-0.6626	0.704287
1.13E-23	1E-13	4.16E-28	8.07E-15	1.54E-14	1.82E-20	1.47E-06	2.17E-17	2.53E-20
0.259999	0.238707	0.412529	0.345136	0.312081	0.382631	0.308191	-0.38603	0.395943
0.003157	0.006878	1.44E-06	7.07E-05	0.000354	9.02E-06	0.000423	7.38E-06	4.07E-06
0.094343	0.031702	0.107833	0.336191	-0.02822	0.345677	-0.20827	-0.10395	0.465436
0.291409	0.723474	0.22754	0.000111	0.752788	6.88E-05	0.018791	0.244808	3.52E-08
0.054729	0.052958	0.091478	-0.22733	-0.02192	0.212018	0.094773	0.207886	-0.09783
0.541118	0.554302	0.306376	0.01016	0.806786	0.016713	0.289205	0.019013	0.273846
-0.0555	-0.24771	-0.09408	-0.27846	-0.38315	0.372708	-0.17559	0.163099	0.166714
0.53546	0.004988	0.292777	0.001524	8.75E-06	1.59E-05	0.048319	0.06693	0.061023
0.123448	-0.07245	0.023369	-0.05419	-0.1592	0.466398	-0.19346	0.029943	0.118131
0.16675	0.418224	0.794257	0.545111	0.073809	3.27E-08	0.029312	0.738246	0.185921
-0.2662	-0.4108	-0.48581	-0.50583	-0.19905	-0.23628	0.09481	0.423905	-0.58662
0.002486	1.61E-06	7.06E-09	1.31E-09	0.024863	0.007486	0.289015	6.83E-07	4.26E-13
-0.33055	-0.14387	-0.32008	-0.33515	-0.24063	-0.4528	-0.09487	0.524549	-0.52129
0.000147	0.106581	0.000244	0.000117	0.006429	9.05E-08	0.2887	2.45E-10	3.31E-10
-0.37492	-0.29831	-0.38713	-0.41827	-0.37128	-0.40191	-0.08599	0.501666	-0.24834
1.41E-05	0.000658	6.92E-06	9.91E-07	1.73E-05	2.82E-06	0.336413	1.88E-09	0.004875
-0.46978	-0.50104	-0.52541	-0.60414	-0.6673	-0.22472	-0.30463	0.68444	-0.3579
2.52E-08	1.98E-09	2.27E-10	5.49E-14	1.07E-17	0.011086	0.000497	7.24E-19	3.61E-05
-0.3997	-0.56445	-0.54907	-0.32355	-0.54263	-0.29349	-0.34309	0.21557	-0.1869
3.23E-06	4.8E-12	2.33E-11	0.000207	4.4E-11	0.000812	7.86E-05	0.014932	0.035377
-0.32856	-0.11654	-0.32549	-0.07511	-0.07425	-0.65261	-0.07173	0.117132	-0.42061
0.000162	0.191958	0.000188	0.401349	0.40673	9.34E-17	0.42291	0.189698	8.5E-07
0.236691	0.276447	0.231347	0.328177	0.446758	-0.00675	0.036019	-0.44283	0.040971
0.00738	0.001654	0.008871	0.000165	1.4E-07	0.939942	0.687662	1.86E-07	0.64742
-0.32688	-0.18248	-0.3201	-0.04122	-0.03584	-0.43054	-0.14563	-0.04796	-0.26599
0.000176	0.04003	0.000244	0.645422	0.689136	4.36E-07	0.102319	0.592319	0.002506
0.126447	0.461484	0.246632	0.191961	0.386024	-0.35736	0.407474	-0.14819	0.076816
0.156604	4.74E-08	0.005186	0.030614	7.39E-06	3.72E-05	1.99E-06	0.096366	0.390682

CFL1	CHCHD4	CHD3	CHMP2B	CHODL	CHPT1	CHST5	CIC	CIZ1
cyan	cyan	cyan	cyan	cyan	cyan	cyan	cyan	cyan
-0.07492	0.192532	-0.11257	0.277182	-0.12182	0.12975	-0.11341	-0.16117	-0.16517
0.402519	0.030112	0.207643	0.001606	0.172454	0.145974	0.204253	0.070267	0.063492
-0.06154	0.225209	-0.13272	0.242969	-0.12639	0.198507	-0.09078	-0.17882	-0.15464
0.491853	0.010907	0.136882	0.005916	0.156799	0.02527	0.310077	0.044265	0.082577
-0.04339	-0.0215	-0.08512	-0.21263	0.192497	-0.11617	-0.06239	-0.06191	0.151259
0.628111	0.810422	0.341364	0.016396	0.030143	0.193371	0.48594	0.489256	0.089596
-0.52694	0.148434	-0.42912	0.232309	0.534927	0.336141	-0.25036	-0.48492	-0.32246
1.97E-10	0.095819	4.81E-07	0.008585	9.26E-11	0.000112	0.004528	7.59E-09	0.000218
-0.20856	0.514313	-0.53473	0.311746	0.242905	0.184053	-0.43676	-0.54292	-0.38929
0.018619	6.21E-10	9.44E-11	0.00036	0.005929	0.038322	2.84E-07	4.27E-11	6.08E-06
-0.6706	0.492651	-0.76963	0.58511	0.646345	0.566589	-0.5447	-0.87618	-0.71009
6.44E-18	4.02E-09	4.06E-26	5.05E-13	2.27E-16	3.83E-12	3.59E-11	1.94E-41	8.98E-21
-0.38617	0.418617	-0.461	0.197768	0.457863	0.366507	-0.33057	-0.51188	-0.32882
7.32E-06	9.69E-07	4.92E-08	0.02583	6.22E-08	2.26E-05	0.000147	7.71E-10	0.00016
-0.34845	0.264168	-0.40936	0.335237	0.27503	0.187079	0.012259	-0.35125	-0.46842
5.96E-05	0.00269	1.76E-06	0.000117	0.001752	0.035199	0.891194	5.14E-05	2.8E-08
0.089546	-0.13928	-0.10126	-0.07451	0.136765	0.094511	-0.24166	0.030691	0.047731
0.316748	0.118359	0.25733	0.405127	0.125218	0.290545	0.006199	0.731952	0.594111
-0.0008	0.02073	-0.46115	0.321858	0.110825	0.253687	-0.38131	-0.21159	-0.33417
0.992904	0.817054	4.86E-08	0.000224	0.214824	0.004004	9.74E-06	0.016938	0.000123
-0.19993	0.052815	-0.46367	0.371218	0.216731	0.374433	-0.24537	-0.36096	-0.39948
0.024222	0.555378	4.02E-08	1.73E-05	0.014387	1.45E-05	0.005428	3.06E-05	3.28E-06
0.428749	-0.39077	0.338412	-0.51798	-0.26674	-0.19976	0.37659	0.40176	0.549601
4.93E-07	5.57E-06	9.96E-05	4.47E-10	0.002435	0.024342	1.28E-05	2.85E-06	2.21E-11
0.527432	-0.47116	0.506316	-0.68735	-0.40284	-0.55425	0.423354	0.776642	0.774877
1.88E-10	2.26E-08	1.26E-09	4.5E-19	2.66E-06	1.38E-11	7.08E-07	7.46E-27	1.15E-26
0.63145	-0.38432	0.244196	-0.41717	-0.43734	-0.49111	0.28728	0.686013	0.547107
1.73E-15	8.17E-06	0.005662	1.07E-06	2.73E-07	4.57E-09	0.001057	5.6E-19	2.83E-11
0.57354	-0.4315	0.153625	-0.3317	-0.4003	-0.3603	0.166523	0.62294	0.436232
1.82E-12	4.09E-07	0.084636	0.000139	3.12E-06	3.17E-05	0.061325	5.28E-15	2.95E-07
0.285262	-0.04967	0.17091	0.019849	-0.41211	-0.28246	0.233456	0.240585	0.074932
0.001151	0.579195	0.05471	0.824708	1.48E-06	0.001293	0.008254	0.006438	0.402442
0.447922	-0.29484	0.620168	-0.51989	-0.42159	-0.57787	0.484698	0.569851	0.597286
1.29E-07	0.000765	7.53E-15	3.76E-10	7.97E-07	1.13E-12	7.72E-09	2.71E-12	1.24E-13
-0.37678	0.117916	0.180766	0.23766	0.188268	0.216635	0.086069	-0.29651	-0.2451
1.26E-05	0.186727	0.041975	0.007135	0.034032	0.014432	0.335979	0.000712	0.005481
0.088428	0.001267	0.536788	-0.11506	-0.23896	-0.12225	0.275524	0.161734	0.142412
0.322848	0.988716	7.75E-11	0.197687	0.006816	0.170926	0.001717	0.069278	0.110223
0.211949	0.149725	0.24922	-0.45672	-0.23082	-0.52398	-0.00331	0.191859	0.429471
0.016749	0.092935	0.00472	6.78E-08	0.009033	2.59E-10	0.970571	0.030705	4.69E-07

CLCN6	CLDN1	CLEC16A	CLEC5A	CLECL1	CLIC2	CLIP3	CLK1	CLK4
cyan	cyan	cyan	cyan	cyan	cyan	cyan	cyan	cyan
0.220088	0.031414	-0.24761	-0.18916	0.062399	0.424707	0.020279	-0.00862	-0.10472
0.012908	0.725888	0.005005	0.03318	0.485848	6.47E-07	0.820967	0.923332	0.241302
0.231575	-0.05904	-0.25201	-0.12496	-0.0157	0.345347	0.098806	-0.01901	-0.10323
0.008803	0.509687	0.004261	0.16159	0.860893	7E-05	0.269078	0.831995	0.248099
-0.33978	0.206564	-0.02726	0.24529	0.152034	-0.19865	-0.28789	0.153329	0.203396
9.3E-05	0.019805	0.760925	0.005444	0.087946	0.025159	0.00103	0.085243	0.021818
-0.41104	0.516839	-0.35543	0.536458	0.393499	0.164439	-0.63664	0.473318	0.516809
1.58E-06	4.95E-10	4.12E-05	8E-11	4.72E-06	0.064688	8.65E-16	1.91E-08	4.97E-10
-0.49629	0.115508	-0.66052	0.054515	0.401373	0.449337	-0.38055	0.172852	0.241028
2.96E-09	0.19595	2.95E-17	0.5427	2.92E-06	1.16E-07	1.02E-05	0.051975	0.006338
-0.63004	0.551698	-0.79552	0.520452	0.623117	0.583655	-0.83744	0.532414	0.612876
2.09E-15	1.79E-11	5.66E-29	3.57E-10	5.16E-15	5.95E-13	1.35E-34	1.18E-10	1.89E-14
-0.29371	0.4391	-0.50658	0.411481	0.42108	0.396559	-0.42274	0.436741	0.400633
0.000804	2.41E-07	1.23E-09	1.54E-06	8.24E-07	3.92E-06	7.38E-07	2.85E-07	3.05E-06
-0.1254	0.19497	-0.14595	0.30921	0.215013	0.253013	-0.33062	0.292418	0.318669
0.160089	0.028049	0.101567	0.000404	0.0152	0.004105	0.000147	0.00085	0.000261
-0.43457	0.40321	-0.11248	0.502435	0.267762	0.226492	-0.44727	0.038454	-0.08403
3.31E-07	2.6E-06	0.207995	1.76E-09	0.002339	0.01045	1.35E-07	0.667757	0.347582
-0.30244	0.23802	-0.3498	0.241616	0.426953	0.607113	-0.48722	0.19646	-0.00458
0.000548	0.007046	5.55E-05	0.006207	5.57E-07	3.83E-14	6.29E-09	0.026848	0.959282
-0.19365	0.303499	-0.34305	0.197625	0.362791	0.472588	-0.49704	0.182171	0.096112
0.029154	0.000523	7.87E-05	0.02594	2.77E-05	2.02E-08	2.78E-09	0.040379	0.282418
0.346996	-0.27801	0.384547	-0.12761	-0.29139	-0.43849	0.437739	-0.29528	-0.45897
6.42E-05	0.001553	8.06E-06	0.15281	0.000888	2.52E-07	2.66E-07	0.000751	5.73E-08
0.297117	-0.24611	0.69871	-0.14103	-0.36249	-0.5961	0.411847	-0.2196	-0.34195
0.000693	0.005284	6.66E-20	0.113758	2.81E-05	1.43E-13	1.5E-06	0.013114	8.33E-05
-0.02601	-0.37291	0.401512	-0.30927	-0.25949	-0.39328	0.345519	-0.40614	-0.43939
0.771593	1.58E-05	2.89E-06	0.000403	0.003218	4.79E-06	6.93E-05	2.16E-06	2.37E-07
0.086233	-0.21107	0.418815	-0.10203	-0.13228	-0.12359	0.153068	-0.19935	-0.40958
0.335052	0.01722	9.57E-07	0.253692	0.138197	0.166274	0.085784	0.024646	1.74E-06
0.55504	-0.49672	0.231058	-0.54311	-0.39339	-0.12358	0.541724	-0.16399	-0.23933
1.27E-11	2.86E-09	0.008959	4.2E-11	4.76E-06	0.166304	4.81E-11	0.065427	0.006728
0.530463	-0.52536	0.583565	-0.52073	-0.55123	-0.69388	0.775584	-0.38615	-0.34504
1.41E-10	2.27E-10	6.01E-13	3.48E-10	1.87E-11	1.52E-19	9.67E-27	7.33E-06	7.11E-05
0.16336	0.014836	-0.09374	-0.13595	-0.17211	-0.12867	0.153879	-0.02229	0.208258
0.066488	0.868514	0.294522	0.12751	0.053013	0.149401	0.084117	0.803563	0.018795
0.491382	-0.33695	0.251603	-0.44626	-0.43657	-0.31987	0.657139	-0.3442	-0.2111
4.46E-09	0.000107	0.004325	1.45E-07	2.88E-07	0.000246	4.85E-17	7.42E-05	0.017202
0.102449	-0.01073	0.118752	-0.24508	-0.09939	-0.18085	0.140306	0.083533	0.054434
0.251734	0.904676	0.183603	0.005484	0.26624	0.041875	0.115644	0.350463	0.543301

CLN5	CLNS1A	CLPTM1	CLRN3	CMPK1	CMTM6	CMTR2	CNBP	CNEP1R1
cyan	cyan	cyan	cyan	cyan	cyan	cyan	cyan	cyan
-0.03013	0.022806	-0.19375	0.144594	0.033865	-0.10787	-0.13492	0.038238	0.009679
0.736671	0.799108	0.029063	0.104821	0.705446	0.227357	0.130432	0.669512	0.913996
-0.0155	0.081228	-0.13876	0.14875	0.010199	-0.2181	-0.20942	0.003396	0.000516
0.862725	0.363958	0.119739	0.095107	0.909391	0.013765	0.018132	0.969772	0.99541
0.046272	0.239365	-0.14446	-0.00475	0.141929	0.336511	0.41449	0.105139	0.233485
0.605447	0.006721	0.105159	0.957735	0.11145	0.00011	1.27E-06	0.239434	0.008246
0.464965	0.336761	-0.50716	0.255452	0.457704	0.653734	0.722825	0.40507	0.619459
3.64E-08	0.000108	1.17E-09	0.003749	6.3E-08	7.94E-17	8.47E-22	2.31E-06	8.24E-15
0.289053	0.578789	-0.44345	0.296591	0.178276	0.055161	0.169723	0.460605	0.275536
0.000981	1.02E-12	1.78E-07	0.000709	0.044931	0.537925	0.056439	5.07E-08	0.001716
0.645485	0.625764	-0.80669	0.475268	0.60905	0.636441	0.73932	0.671043	0.783846
2.56E-16	3.66E-15	2.45E-30	1.64E-08	3.02E-14	8.89E-16	3.25E-23	6.02E-18	1.23E-27
0.511463	0.446448	-0.41168	0.353367	0.272405	0.39537	0.372691	0.445437	0.440027
8E-10	1.43E-07	1.52E-06	4.6E-05	0.001946	4.22E-06	1.6E-05	1.54E-07	2.26E-07
0.337291	0.333809	-0.39028	0.186298	0.283695	0.316679	0.363614	0.433593	0.066035
0.000105	0.000125	5.73E-06	0.035984	0.001229	0.000286	2.65E-05	3.54E-07	0.460745
0.332354	-0.07569	-0.05676	-0.01363	0.25951	0.625727	0.245323	0.093236	0.148775
0.000135	0.397681	0.52622	0.879125	0.003216	3.68E-15	0.005437	0.297134	0.09505
0.357131	0.13681	-0.28443	0.096486	0.303062	0.554406	0.28674	0.285797	0.064515
3.76E-05	0.125091	0.001191	0.280538	0.000533	1.36E-11	0.001082	0.001125	0.471151
0.394278	0.161491	-0.40318	0.22893	0.330465	0.530417	0.427555	0.350125	0.22942
4.51E-06	0.069702	2.6E-06	0.009629	0.000148	1.42E-10	5.34E-07	5.46E-05	0.009471
0.093446	-0.28383	0.575569	-0.12724	-0.56438	-0.29549	-0.47883	-0.3393	-0.35736
0.29604	0.001222	1.46E-12	0.154	4.84E-12	0.000744	1.24E-08	9.53E-05	3.72E-05
-0.39159	-0.3728	0.601058	-0.44386	-0.40675	-0.13958	-0.26324	-0.43203	-0.54836
5.3E-06	1.59E-05	7.95E-14	1.73E-07	2.08E-06	0.117554	0.002788	3.94E-07	2.5E-11
-0.38885	-0.27625	0.56264	-0.42093	-0.42363	-0.33696	-0.35784	-0.26227	-0.60236
6.24E-06	0.001668	5.81E-12	8.32E-07	6.96E-07	0.000107	3.62E-05	0.002895	6.8E-14
-0.19195	-0.3022	0.434935	-0.39231	-0.23434	0.061662	-0.20123	-0.22915	-0.59193
0.030623	0.000554	3.23E-07	5.08E-06	0.008007	0.491026	0.023292	0.009559	2.32E-13
-0.3436	-0.18168	0.290205	-0.21055	-0.31628	-0.54454	-0.45283	-0.30657	-0.46675
7.65E-05	0.04093	0.000934	0.017502	0.000292	3.65E-11	9.02E-08	0.000456	3.18E-08
-0.60195	-0.2764	0.595759	-0.31366	-0.56733	-0.66223	-0.52127	-0.51628	-0.48048
7.14E-14	0.001658	1.49E-13	0.00033	3.54E-12	2.29E-17	3.31E-10	5.21E-10	1.09E-08
-0.10097	-0.05243	-0.14292	0.172927	0.101342	-0.28003	-0.00035	-0.04807	0.401143
0.258682	0.558278	0.108941	0.051872	0.256924	0.001429	0.996906	0.5915	2.96E-06
-0.41506	-0.26666	0.317037	-0.03636	-0.32256	-0.71175	-0.56071	-0.36972	-0.17342
1.22E-06	0.002442	0.000282	0.684837	0.000217	6.65E-21	7.1E-12	1.89E-05	0.051197
-0.26507	0.065229	0.072164	-0.14516	-0.27994	-0.12515	-0.12916	-0.07543	-0.05179
0.002598	0.466244	0.420093	0.103453	0.001435	0.160942	0.147843	0.399331	0.563115

CNIH1	CNIH3	CNIH4	CNNM3	CNOT10	CNOT11	CNOT7	CNOT8	CNTNAP1
cyan	cyan	cyan	cyan	cyan	cyan	cyan	cyan	cyan
-0.00537	0.128156	-0.02766	-0.06249	-0.20058	0.076425	0.047892	-0.03193	0.111488
0.95222	0.151032	0.757539	0.485226	0.02375	0.393107	0.592866	0.721595	0.212077
0.057237	0.118875	0.018251	-0.03447	-0.1876	0.101502	0.028569	-0.02003	0.134011
0.522711	0.183145	0.838615	0.700444	0.034679	0.25617	0.749846	0.82316	0.133076
0.195292	0.146168	0.304713	-0.00766	0.614563	0.324127	0.341631	0.319834	-0.35217
0.027786	0.101054	0.000495	0.931927	1.53E-14	0.000201	8.46E-05	0.000247	4.9E-05
0.468911	0.410986	0.50826	-0.31669	0.685604	0.603781	0.592208	0.506728	-0.37063
2.69E-08	1.59E-06	1.06E-09	0.000286	5.99E-19	5.73E-14	2.25E-13	1.21E-09	1.79E-05
0.506611	0.228249	0.696101	-0.42397	0.261364	0.215523	0.405494	0.482323	-0.57954
1.22E-09	0.009852	1.04E-19	6.8E-07	0.002997	0.014955	2.25E-06	9.36E-09	9.42E-13
0.774173	0.531923	0.847411	-0.64795	0.629982	0.689525	0.773334	0.73902	-0.62106
1.36E-26	1.23E-10	3.58E-36	1.81E-16	2.11E-15	3.14E-19	1.67E-26	3.45E-23	6.72E-15
0.436653	0.39705	0.534102	-0.35448	0.378976	0.519361	0.452799	0.343169	-0.28177
2.86E-07	3.81E-06	1E-10	4.33E-05	1.11E-05	3.94E-10	9.04E-08	7.82E-05	0.001331
0.051624	-0.00415	0.293949	-0.29481	0.184245	0.254552	0.17804	0.033653	-0.00365
0.564342	0.96311	0.000796	0.000767	0.038117	0.003877	0.04522	0.70721	0.96753
-0.04638	0.20794	0.24009	-0.18975	0.249227	0.302048	-0.00901	0.060657	-0.12465
0.604569	0.018981	0.006552	0.032619	0.004719	0.000558	0.919887	0.498129	0.162641
-0.05544	-0.04163	0.232368	-0.31455	-0.08355	0.247826	-0.13356	-0.0055	-0.2388
0.535855	0.642133	0.008568	0.000316	0.350372	0.004966	0.134394	0.951038	0.006856
0.026123	0.136194	0.197262	-0.34492	0.102711	0.305483	0.120144	0.077124	-0.17932
0.77065	0.126815	0.02622	7.15E-05	0.250519	0.000479	0.178483	0.388776	0.043666
-0.50499	-0.04588	-0.36986	0.564197	-0.03636	-0.19523	-0.44369	-0.33295	0.39374
1.41E-09	0.608533	1.87E-05	4.93E-12	0.684831	0.027833	1.75E-07	0.000131	4.66E-06
-0.65472	-0.3691	-0.4838	0.623735	-0.04746	-0.29405	-0.55192	-0.49975	0.26723
6.88E-17	1.95E-05	8.31E-09	4.76E-15	0.596214	0.000792	1.75E-11	2.21E-09	0.002388
-0.46821	-0.45586	-0.23839	0.364591	-0.29832	-0.54375	-0.62678	-0.43987	-0.00051
2.84E-08	7.22E-08	0.006954	2.51E-05	0.000658	3.94E-11	3.21E-15	2.29E-07	0.995452
-0.67193	-0.47048	-0.35019	0.357137	-0.33424	-0.31077	-0.75156	-0.54999	0.096958
5.25E-18	2.39E-08	5.44E-05	3.76E-05	0.000123	0.000376	2.45E-24	2.12E-11	0.278181
-0.35858	-0.45196	-0.53359	0.333448	-0.57315	-0.40176	-0.49382	-0.37102	0.278265
3.48E-05	9.62E-08	1.05E-10	0.000128	1.9E-12	2.85E-06	3.65E-09	1.75E-05	0.001537
-0.34201	-0.34436	-0.58928	0.518764	-0.23108	-0.49725	-0.26973	-0.32967	0.339811
8.3E-05	7.36E-05	3.14E-13	4.16E-10	0.008953	2.73E-09	0.002164	0.000154	9.28E-05
0.454266	0.253299	-0.07921	-0.16283	-0.00622	0.000802	0.482633	0.301024	0.186277
8.12E-08	0.004062	0.376041	0.067387	0.944701	0.992857	9.13E-09	0.000584	0.036005
-0.03033	-0.09262	-0.44053	0.166342	-0.40611	-0.40261	-0.01785	-0.15748	0.489905
0.735015	0.300371	2.18E-07	0.061611	2.17E-06	2.7E-06	0.842074	0.077033	5.05E-09
-0.05343	0.070168	0.019049	0.310671	0.337741	-0.01243	0.091496	0.203795	-0.24966
0.550763	0.433096	0.831661	0.000378	0.000103	0.889672	0.30628	0.021555	0.004645

COA1	COIL	COL9A3	COMMD1(	COMMD2	COMMD8	COPB1	COPS2	COPS8
cyan	cyan	cyan	cyan	cyan	cyan	cyan	cyan	cyan
-0.02165	-0.15255	-0.20896	-0.13228	-0.22457	-0.01115	-0.09103	0.222065	-0.0159
0.809124	0.086865	0.01839	0.138202	0.011141	0.900976	0.308784	0.0121	0.859141
0.047969	-0.11421	-0.16979	-0.10698	-0.2084	-0.03544	-0.1292	0.230628	0.090728
0.592274	0.201077	0.056341	0.231266	0.018714	0.692463	0.147711	0.009091	0.310372
0.257032	0.508551	0.104601	0.350352	0.630764	0.104352	0.395613	-0.11941	0.178684
0.003533	1.03E-09	0.241861	5.39E-05	1.9E-15	0.24299	4.15E-06	0.181183	0.044435
0.439662	0.584754	-0.22516	0.555539	0.739887	0.383949	0.795737	0.364442	0.364405
2.32E-07	5.26E-13	0.010925	1.21E-11	2.89E-23	8.35E-06	5.33E-29	2.53E-05	2.53E-05
0.679951	0.416861	-0.28685	0.481234	0.329445	0.574206	0.210042	0.379116	0.519405
1.49E-18	1.09E-06	0.001077	1.02E-08	0.000156	1.69E-12	0.017781	1.11E-05	3.93E-10
0.786538	0.656566	-0.49965	0.764867	0.72558	0.757788	0.854189	0.732939	0.649856
6.14E-28	5.27E-17	2.23E-09	1.24E-25	4.99E-22	6.21E-25	2.61E-37	1.18E-22	1.38E-16
0.641889	0.341894	-0.36885	0.441208	0.414079	0.316589	0.52876	0.476053	0.551931
4.22E-16	8.35E-05	1.98E-05	2.08E-07	1.3E-06	0.000288	1.66E-10	1.54E-08	1.75E-11
0.14619	-0.00168	-0.37637	0.096468	0.013386	0.32326	0.329024	0.241254	0.199155
0.101003	0.985087	1.29E-05	0.28063	0.881265	0.00021	0.000159	0.006288	0.024787
-0.01179	0.036026	-0.11211	-0.01145	0.114739	-0.04043	0.485011	-0.02173	-0.10965
0.89532	0.687605	0.209507	0.898378	0.198966	0.651801	7.53E-09	0.808442	0.219749
-0.00201	-0.22977	-0.26175	-0.12203	-0.21225	0.150608	0.273241	0.237441	-0.22829
0.982083	0.009358	0.002952	0.171715	0.016589	0.091	0.001882	0.00719	0.009839
0.040402	-0.11858	-0.36799	0.026473	0.026867	0.17824	0.425373	0.286181	-0.11718
0.651998	0.184239	2.08E-05	0.767661	0.764299	0.044975	6.19E-07	0.001107	0.18951
-0.43606	-0.20945	0.221882	-0.42278	-0.18436	-0.6289	-0.28031	-0.45781	-0.32129
2.98E-07	0.01811	0.012173	7.36E-07	0.037992	2.43E-15	0.001413	6.25E-08	0.00023
-0.58355	-0.26572	0.481666	-0.53595	-0.24183	-0.67436	-0.35619	-0.7487	-0.56708
6.02E-13	0.002533	9.87E-09	8.4E-11	0.006161	3.6E-18	3.96E-05	4.54E-24	3.64E-12
-0.3991	-0.32964	0.462725	-0.42831	-0.39513	-0.30053	-0.54935	-0.63426	-0.51143
3.35E-06	0.000154	4.32E-08	5.08E-07	4.28E-06	0.000597	2.26E-11	1.19E-15	8.02E-10
-0.60211	-0.56084	0.31914	-0.66395	-0.57863	-0.47879	-0.34209	-0.53374	-0.73509
7.01E-14	7.01E-12	0.000255	1.77E-17	1.04E-12	1.24E-08	8.27E-05	1.04E-10	7.66E-23
-0.36876	-0.47644	0.404232	-0.48267	-0.56801	-0.27413	-0.6541	-0.1613	-0.37481
1.99E-05	1.5E-08	2.44E-06	9.1E-09	3.29E-12	0.001817	7.53E-17	0.070035	1.41E-05
-0.32959	-0.16628	0.581091	-0.25631	-0.10321	-0.4384	-0.66554	-0.5903	-0.19877
0.000154	0.061712	7.93E-13	0.00363	0.248195	2.54E-07	1.39E-17	2.8E-13	0.025071
0.229482	0.285526	-0.08005	0.441028	0.306858	0.276609	0.020616	0.317209	0.422943
0.009451	0.001138	0.370964	2.11E-07	0.00045	0.001644	0.818043	0.000279	7.28E-07
-0.11682	-0.15584	0.096225	-0.07865	-0.23405	-0.165	-0.54269	-0.08692	0.13423
0.190893	0.080195	0.281847	0.379455	0.008087	0.063774	4.37E-11	0.331188	0.132439
0.105194	0.311564	0.267333	0.081108	0.303655	-0.18769	-0.17349	-0.27734	0.078575
0.239187	0.000363	0.002378	0.36467	0.000519	0.034591	0.051103	0.001595	0.379893

COPZ1	COQ5	COQ7	COX18	CPED1	CPT1C	CRAT	CROT	CRTC1
cyan	cyan	cyan	cyan	cyan	cyan	cyan	cyan	cyan
-0.18174	0.036415	-0.11273	-0.05926	0.04778	-0.28795	-0.20379	-0.16907	-0.01427
0.040862	0.684408	0.206994	0.508128	0.593736	0.001028	0.021559	0.057408	0.873454
-0.11483	0.127456	-0.04029	0.030145	-0.05269	-0.17016	-0.13687	-0.17752	0.023264
0.198621	0.153296	0.652922	0.73654	0.556279	0.055802	0.124913	0.04586	0.79516
0.478949	0.275567	0.168573	0.226976	-0.01576	0.148042	0.082612	0.219689	-0.35435
1.23E-08	0.001714	0.058157	0.010282	0.860446	0.09671	0.355818	0.013076	4.37E-05
0.503589	0.459414	0.359393	0.43093	0.405375	-0.26974	-0.42527	0.548296	-0.65174
1.59E-09	5.54E-08	3.33E-05	4.25E-07	2.27E-06	0.002164	6.23E-07	2.51E-11	1.06E-16
0.514195	0.73731	0.312358	0.335389	0.143025	-0.22986	-0.2369	-0.04343	-0.38086
6.28E-10	4.89E-23	0.00035	0.000116	0.108685	0.009331	0.007326	0.627791	1E-05
0.656137	0.830923	0.483368	0.571365	0.561379	-0.55573	-0.66718	0.50186	-0.83179
5.61E-17	1.27E-33	8.6E-09	2.3E-12	6.62E-12	1.19E-11	1.09E-17	1.85E-09	9.49E-34
0.551564	0.526512	0.383142	0.256487	0.358155	-0.18264	-0.40324	0.276111	-0.51844
1.81E-11	2.05E-10	8.75E-06	0.003606	3.56E-05	0.03986	2.6E-06	0.001677	4.29E-10
0.082088	0.193359	0.441804	0.040469	0.324814	-0.5425	-0.48389	0.344105	-0.25323
0.358886	0.029399	2E-07	0.651457	0.000195	4.46E-11	8.25E-09	7.46E-05	0.004073
0.272016	-0.07833	-0.1995	-0.17291	0.330181	-0.21127	-0.01023	0.364355	-0.4655
0.001977	0.381384	0.024535	0.051893	0.00015	0.01711	0.909083	2.54E-05	3.5E-08
0.08605	-0.01479	-0.24433	-0.1606	0.402163	-0.63842	-0.27668	0.265787	-0.52584
0.336085	0.868942	0.005635	0.071273	2.78E-06	6.79E-16	0.001639	0.002526	2.18E-10
0.128436	0.031699	-0.14547	-0.03905	0.476286	-0.64406	-0.45464	0.388473	-0.49351
0.150133	0.723501	0.102723	0.662899	1.51E-08	3.13E-16	7.9E-08	6.39E-06	3.74E-09
-0.04945	-0.33336	-0.25951	-0.33075	-0.2487	0.341664	0.464512	-0.14854	0.283692
0.580871	0.000128	0.003217	0.000146	0.004811	8.45E-05	3.77E-08	0.09558	0.001229
-0.20174	-0.5799	-0.3463	-0.41847	-0.44326	0.467256	0.626981	-0.14044	0.32717
0.022938	9.05E-13	6.66E-05	9.79E-07	1.8E-07	3.06E-08	3.12E-15	0.115287	0.000174
-0.21123	-0.34827	-0.34772	-0.34056	-0.48007	0.349812	0.627255	-0.27664	0.386507
0.017131	6.01E-05	6.19E-05	8.94E-05	1.12E-08	5.55E-05	3.01E-15	0.001641	7.18E-06
-0.27667	-0.58084	-0.54741	-0.5253	-0.20561	-0.0228	0.434031	-0.05512	0.09302
0.001639	8.15E-13	2.74E-11	2.29E-10	0.020395	0.799185	3.43E-07	0.538216	0.298261
-0.49492	-0.29434	-0.22483	-0.09058	-0.32461	0.114	0.104594	-0.44449	0.439259
3.32E-09	0.000782	0.011044	0.311146	0.000197	0.201895	0.241893	1.65E-07	2.39E-07
-0.29196	-0.36444	-0.13832	-0.13684	-0.58779	0.74593	0.539958	-0.50874	0.704686
0.000867	2.53E-05	0.120939	0.125017	3.73E-13	8.19E-24	5.71E-11	1.02E-09	2.35E-20
-0.12405	0.182424	0.335445	0.398888	0.041907	0.212854	-0.24732	-0.09603	0.284176
0.164658	0.040097	0.000116	3.4E-06	0.639924	0.016278	0.005058	0.282831	0.001204
-0.40054	-0.19925	0.051482	-0.04558	-0.30254	0.477873	0.163788	-0.47153	0.735054
3.07E-06	0.024717	0.565417	0.610824	0.000546	1.34E-08	0.065769	2.2E-08	7.72E-23
0.204437	0.119048	0.014473	0.072216	-0.34429	0.437506	0.33911	-0.48078	0.009945
0.021138	0.182505	0.871697	0.419757	7.39E-05	2.7E-07	9.62E-05	1.06E-08	0.911643

CRYZ	CSNK1D	CSNK1G3	CSTF1	CTBS	CTH	CTIF	CTNNAL1	CTNND2
cyan	cyan	cyan	cyan	cyan	cyan	cyan	cyan	cyan
-0.06503	-0.23614	-0.12211	-0.0657	0.154326	-0.07212	0.100341	-0.01888	-0.0658
0.467642	0.007524	0.171435	0.463003	0.083209	0.420372	0.261674	0.833144	0.46231
-0.19891	-0.28428	-0.1238	-0.02058	0.132157	-0.01459	0.159737	-0.03976	-0.03355
0.024971	0.001199	0.165546	0.818389	0.138575	0.870636	0.072831	0.657202	0.708085
0.2662	-0.05171	0.382092	0.44169	-0.17128	0.253126	-0.38105	0.497555	-0.14752
0.002486	0.563713	9.3E-06	2.01E-07	0.054181	0.004088	9.88E-06	2.66E-09	0.097901
0.57951	-0.35871	0.638207	0.562613	0.29228	0.532073	-0.75498	0.683217	-0.38353
9.45E-13	3.46E-05	6.99E-16	5.82E-12	0.000855	1.21E-10	1.16E-24	8.82E-19	8.56E-06
0.004578	-0.60333	0.183304	0.303846	0.364248	0.145344	-0.31915	0.487602	-0.44291
0.959263	6.05E-14	0.03913	0.000515	2.56E-05	0.103013	0.000255	6.1E-09	1.85E-07
0.564535	-0.76747	0.679435	0.625776	0.652112	0.554751	-0.86853	0.853861	-0.64774
4.76E-12	6.75E-26	1.62E-18	3.66E-15	1E-16	1.31E-11	6.43E-40	2.97E-37	1.87E-16
0.217167	-0.48306	0.327725	0.375446	0.396322	0.378724	-0.53123	0.525151	-0.43846
0.014187	8.82E-09	0.000169	1.36E-05	3.98E-06	1.13E-05	1.31E-10	2.32E-10	2.52E-07
0.255479	-0.16987	0.093478	-0.00998	0.331339	0.005499	-0.28035	0.183566	-0.09061
0.003745	0.056224	0.295875	0.911331	0.000142	0.951077	0.001411	0.038845	0.310992
0.406723	0.215305	0.056796	0.153339	0.279073	-0.09941	-0.46215	0.274499	-0.48414
2.09E-06	0.015059	0.525924	0.085222	0.001487	0.266143	4.51E-08	0.00179	8.08E-09
0.336652	-0.01293	-0.22913	-0.02252	0.567286	-0.30698	-0.39386	0.110442	-0.37432
0.000109	0.885267	0.009563	0.801533	3.56E-12	0.000447	4.62E-06	0.216423	1.46E-05
0.461217	-0.10254	0.016048	0.014084	0.502491	0.030323	-0.49316	0.218422	-0.27522
4.84E-08	0.251305	0.857875	0.875118	1.75E-09	0.735045	3.85E-09	0.013624	0.001739
-0.29637	0.528703	-0.38524	-0.32703	-0.14946	-0.07726	0.289446	-0.37539	0.169389
0.000716	1.67E-10	7.74E-06	0.000175	0.09352	0.387938	0.000965	1.37E-05	0.056934
-0.20706	0.739716	-0.42963	-0.18092	-0.58754	-0.34109	0.360904	-0.38652	0.365482
0.019506	2.99E-23	4.64E-07	0.041796	3.83E-13	8.7E-05	3.07E-05	7.17E-06	2.39E-05
-0.33733	0.589288	-0.54365	-0.27098	-0.39573	-0.52146	0.400765	-0.44165	0.248179
0.000105	3.14E-13	3.98E-11	0.00206	4.13E-06	3.26E-10	3.03E-06	2.02E-07	0.004903
-0.10317	0.693386	-0.68941	-0.33288	-0.12591	-0.65224	0.237345	-0.4667	0.149966
0.248373	1.65E-19	3.2E-19	0.000131	0.158376	9.85E-17	0.007214	3.19E-08	0.092404
-0.54805	0.141203	-0.52951	-0.41419	-0.21886	-0.3871	0.652303	-0.64125	0.50179
2.58E-11	0.11331	1.55E-10	1.29E-06	0.013434	6.93E-06	9.76E-17	4.61E-16	1.86E-09
-0.56991	0.367916	-0.25395	-0.33516	-0.7516	-0.11372	0.716527	-0.48139	0.69287
2.69E-12	2.09E-05	0.003964	0.000117	2.43E-24	0.203002	2.77E-21	1.01E-08	1.8E-19
-0.01881	-0.38986	0.558354	0.079209	-0.12688	0.488547	0.095423	0.07098	0.171094
0.83375	5.88E-06	9.06E-12	0.376045	0.155174	5.64E-09	0.285898	0.427783	0.054445
-0.46798	-0.02092	-0.03631	-0.34438	-0.39917	0.044183	0.640864	-0.4055	0.453629
2.89E-08	0.815369	0.685282	7.35E-05	3.34E-06	0.621849	4.86E-16	2.25E-06	8.51E-08
-0.26156	-0.02727	0.004477	0.247405	-0.40851	-0.03452	0.189098	0.192899	-0.09022
0.002974	0.76085	0.960155	0.005043	1.86E-06	0.699983	0.033237	0.029794	0.313103

CTSV	CWC22	CYB5R3	CYB5R4	CYP2R1	CYP39A1	D2HGDH	DAB2IP	DAGLA
cyan	cyan	cyan	cyan	cyan	cyan	cyan	cyan	cyan
0.074875	-0.04969	0.150638	0.052263	0.115108	0.145201	-0.23571	-0.05969	-0.20584
0.402801	0.579022	0.090937	0.559523	0.197516	0.103355	0.007637	0.505003	0.02025
0.070084	-0.07652	0.161212	0.004021	0.168179	0.148499	-0.24276	-0.07504	-0.15463
0.433651	0.392503	0.070192	0.964212	0.058754	0.095673	0.00596	0.40177	0.082591
0.2955	0.494912	-0.34375	0.083963	0.225963	-0.24746	0.043728	-0.14514	-0.10694
0.000744	3.33E-09	7.6E-05	0.347981	0.010636	0.005034	0.625447	0.103497	0.231455
0.330542	0.801832	-0.6425	0.368003	0.457211	0.02677	-0.37456	-0.58277	-0.47124
0.000147	9.85E-30	3.88E-16	2.08E-05	6.53E-08	0.765131	1.44E-05	6.58E-13	2.25E-08
0.319522	0.236763	-0.18805	0.422233	0.541017	0.352482	-0.27128	-0.47132	-0.53936
0.000251	0.007362	0.03424	7.63E-07	5.15E-11	4.82E-05	0.002036	2.23E-08	6.05E-11
0.458129	0.813934	-0.68668	0.683006	0.728633	0.382548	-0.64323	-0.87676	-0.80979
6.1E-08	2.87E-31	5.02E-19	9.13E-19	2.76E-22	9.06E-06	3.51E-16	1.47E-41	9.9E-31
0.246589	0.528615	-0.31494	0.378051	0.507268	0.284839	-0.46444	-0.51901	-0.52448
0.005194	1.68E-10	0.000311	1.18E-05	1.16E-09	0.001171	3.79E-08	4.07E-10	2.47E-10
-0.01595	0.202759	-0.27113	0.172677	0.146408	0.457851	-0.19548	-0.30938	-0.32284
0.858756	0.022243	0.002048	0.052217	0.100491	6.23E-08	0.027637	0.000401	0.000214
0.021	0.258056	0.112204	0.188961	0.239054	-0.03895	-0.06271	-0.14619	-0.25308
0.814719	0.003399	0.209135	0.033367	0.006795	0.663732	0.483644	0.100993	0.004095
0.136893	0.062445	0.254353	0.414935	0.30564	0.372959	-0.09889	-0.30378	-0.50893
0.124862	0.485522	0.003906	1.23E-06	0.000475	1.57E-05	0.268647	0.000517	1E-09
0.131484	0.235599	-0.04657	0.266943	0.243684	0.192664	-0.2352	-0.38323	-0.4443
0.140614	0.007666	0.603095	0.002415	0.005766	0.029998	0.007771	8.7E-06	1.67E-07
-0.17268	-0.38348	0.554453	-0.5764	-0.22825	-0.41191	0.242595	0.403659	0.422756
0.052219	8.58E-06	1.35E-11	1.33E-12	0.009851	1.5E-06	0.005995	2.53E-06	7.37E-07
-0.1624	-0.26783	0.463424	-0.55215	-0.39167	-0.48105	0.66847	0.605322	0.511707
0.068129	0.002333	4.1E-08	1.71E-11	5.27E-06	1.04E-08	8.93E-18	4.76E-14	7.83E-10
-0.19695	-0.5636	0.593555	-0.39854	-0.31421	-0.25347	0.715839	0.531344	0.388764
0.026459	5.25E-12	1.92E-13	3.47E-06	0.000321	0.004036	3.14E-21	1.3E-10	6.28E-06
-0.12648	-0.44584	0.739543	-0.28182	-0.25718	-0.10842	0.618772	0.433311	0.184024
0.15648	1.5E-07	3.1E-23	0.001328	0.003513	0.225024	8.99E-15	3.61E-07	0.038353
0.005739	-0.56899	0.44518	-0.26716	-0.31615	0.079672	0.223181	0.353919	0.23267
0.948939	2.97E-12	1.57E-07	0.002395	0.000293	0.373255	0.011664	4.47E-05	0.008479
-0.20307	-0.47618	0.242681	-0.56735	-0.54655	-0.47693	0.431858	0.674329	0.718956
0.022038	1.53E-08	0.005977	3.54E-12	2.99E-11	1.44E-08	3.99E-07	3.62E-18	1.76E-21
-0.09704	0.108158	-0.60074	0.063209	-0.13495	-0.06464	-0.41475	-0.09844	0.191372
0.277763	0.226133	8.25E-14	0.480195	0.130342	0.470315	1.25E-06	0.270848	0.031139
-0.36274	-0.4407	-0.01646	-0.29026	-0.43361	-0.12518	-0.05082	0.3936	0.553927
2.78E-05	2.16E-07	0.854274	0.000932	3.53E-07	0.160844	0.570436	4.7E-06	1.43E-11
0.352177	0.205043	0.025349	-0.04207	0.074159	-0.32146	0.288936	0.165842	0.065205
4.9E-05	0.020751	0.777264	0.638615	0.407326	0.000229	0.000986	0.062407	0.466409

DAPL1	DARS	DBN1	DBNDD1	DCAKD	DCK	DCTN1	DDIT3	DDO
cyan	cyan	cyan	cyan	cyan	cyan	cyan	cyan	cyan
-0.02219	-0.09657	-0.33932	0.068161	-0.04133	-0.14555	0.176201	0.439587	0.215345
0.804428	0.280101	9.52E-05	0.446402	0.64455	0.102516	0.047526	2.33E-07	0.01504
0.021885	-0.13428	-0.33866	0.139817	-0.01196	-0.16409	0.21684	0.41934	0.207838
0.807055	0.132304	9.84E-05	0.116931	0.893808	0.065266	0.014337	9.24E-07	0.019041
0.076149	0.366974	0.280446	-0.24706	-0.13294	0.205876	-0.45192	-0.23978	-0.13952
0.394824	2.2E-05	0.001405	0.005107	0.136237	0.020228	9.64E-08	0.006624	0.117728
0.251367	0.667051	-0.331	-0.50922	-0.42489	0.425928	-0.68443	0.051317	0.272421
0.004363	1.11E-17	0.000144	9.75E-10	6.39E-07	5.96E-07	7.25E-19	0.566664	0.001945
0.480505	0.200651	-0.25178	-0.28633	-0.15323	0.263474	-0.49411	0.620347	0.467685
1.08E-08	0.023702	0.004296	0.0011	0.085451	0.002763	3.56E-09	7.36E-15	2.96E-08
0.545415	0.707248	-0.65086	-0.63705	-0.54968	0.568465	-0.86441	0.532816	0.666289
3.35E-11	1.49E-20	1.2E-16	8.18E-16	2.19E-11	3.14E-12	3.87E-39	1.13E-10	1.24E-17
0.388471	0.351809	-0.50431	-0.36271	-0.34277	0.177769	-0.47095	0.481227	0.4827
6.39E-06	4.99E-05	1.5E-09	2.78E-05	7.99E-05	0.045554	2.3E-08	1.02E-08	9.08E-09
0.20082	0.311305	-0.43108	-0.42188	-0.0627	0.132412	-0.3054	0.184351	0.453033
0.023583	0.000367	4.21E-07	7.82E-07	0.483753	0.13781	0.00048	0.038004	8.89E-08
-0.17374	0.242118	-0.02772	-0.22999	-0.15103	-0.15582	-0.29115	0.066553	-0.00767
0.050759	0.006098	0.757031	0.009289	0.090093	0.080232	0.000897	0.457227	0.93177
0.057328	0.19905	-0.47357	-0.37251	-0.03346	-0.1906	-0.23421	0.439638	0.477584
0.522051	0.024865	1.88E-08	1.61E-05	0.708798	0.03184	0.008043	2.32E-07	1.37E-08
0.122876	0.36452	-0.50702	-0.39229	-0.15403	0.009436	-0.31982	0.347661	0.514896
0.168736	2.52E-05	1.18E-09	5.08E-06	0.083817	0.916146	0.000247	6.21E-05	5.9E-10
-0.29208	-0.31824	0.318627	0.393018	0.456223	-0.43152	0.504504	-0.15614	-0.34097
0.000862	0.000266	0.000261	4.86E-06	7.03E-08	4.08E-07	1.47E-09	0.079603	8.75E-05
-0.46455	-0.25029	0.64469	0.281126	0.444619	-0.53006	0.377242	-0.54832	-0.58335
3.76E-08	0.004539	2.86E-16	0.001367	1.63E-07	1.47E-10	1.23E-05	2.51E-11	6.16E-13
-0.34846	-0.34598	0.665791	0.311151	0.529877	-0.39852	0.258074	-0.19805	-0.34101
5.95E-05	6.77E-05	1.34E-17	0.00037	1.49E-10	3.48E-06	0.003396	0.025618	8.74E-05
-0.4002	-0.23636	0.301237	0.128553	0.479497	-0.64707	0.305493	-0.13574	-0.17326
3.14E-06	0.007465	0.000578	0.149762	1.17E-08	2.05E-16	0.000478	0.1281	0.051417
-0.11111	-0.57002	-0.00323	0.334571	0.349996	-0.3146	0.653741	-0.08783	-0.09864
0.213624	2.66E-12	0.971292	0.000121	5.49E-05	0.000315	7.93E-17	0.326129	0.269893
-0.21885	-0.48868	0.72974	0.551718	0.377041	-0.12439	0.563647	-0.58699	-0.6592
0.013436	5.58E-09	2.22E-22	1.78E-11	1.25E-05	0.163515	5.23E-12	4.08E-13	3.58E-17
0.133391	-0.0348	-0.06247	0.128199	-0.37379	0.598591	0.048181	-0.19804	-0.12711
0.134898	0.697698	0.485381	0.150896	1.5E-05	1.07E-13	0.590637	0.025624	0.154424
-0.08027	-0.49833	0.244785	0.477906	0.047437	0.10823	0.550298	-0.2503	-0.36788
0.369642	2.49E-09	0.005543	1.33E-08	0.596392	0.225823	2.06E-11	0.004538	2.09E-05
0.065303	-0.23558	0.440114	0.104093	-0.05212	-0.08859	0.061205	-0.11976	-0.42945
0.465742	0.00767	2.25E-07	0.244165	0.560633	0.321952	0.494252	0.179879	4.7E-07

DDX50	DDX51	DECR1	DEF8	DEGS1	DENND1B	DENND4B	DENND6B	DEPDC4
cyan	cyan	cyan	cyan	cyan	cyan	cyan	cyan	cyan
0.254185	-0.10442	0.100528	0.001664	-0.00183	-0.01626	0.050509	0.084242	-0.08051
0.00393	0.242664	0.260783	0.985187	0.983672	0.856059	0.572798	0.346373	0.368228
0.26917	-0.01971	0.128241	0.04822	0.001422	-0.07001	-0.00486	0.063155	-0.04208
0.002213	0.825907	0.15076	0.590333	0.987339	0.434109	0.95678	0.480572	0.638506
0.069163	0.101009	0.102174	-0.41169	0.128484	0.132292	-0.14125	-0.28083	0.448172
0.439729	0.258496	0.253018	1.52E-06	0.149982	0.138171	0.113202	0.001383	1.27E-07
0.24311	-0.22311	0.225402	-0.67914	0.528183	0.463906	-0.45748	-0.50941	0.574749
0.005886	0.01169	0.010837	1.7E-18	1.75E-10	3.95E-08	6.4E-08	9.58E-10	1.59E-12
0.580705	-0.23604	0.468907	-0.05134	0.325526	0.107855	-0.53924	-0.24004	0.272333
8.28E-13	0.007549	2.69E-08	0.56646	0.000188	0.227444	6.12E-11	0.006564	0.001952
0.631469	-0.49091	0.533106	-0.65652	0.733545	0.522723	-0.81071	-0.63727	0.600951
1.73E-15	4.64E-09	1.1E-10	5.31E-17	1.05E-22	2.9E-10	7.55E-31	7.94E-16	8.05E-14
0.394164	-0.20843	0.268807	-0.30498	0.474831	0.319834	-0.47205	-0.40233	0.400727
4.54E-06	0.018692	0.002245	0.00049	1.7E-08	0.000247	2.11E-08	2.75E-06	3.03E-06
0.17028	-0.29509	0.190959	-0.14542	0.365103	0.32995	-0.33167	0.08299	0.032981
0.055622	0.000757	0.031511	0.102822	2.44E-05	0.000152	0.000139	0.353612	0.712797
0.022367	-0.20298	-0.00173	-0.4285	0.357867	0.223519	0.01749	-0.41832	0.011163
0.802899	0.022093	0.984569	5.01E-07	3.62E-05	0.011535	0.845262	9.88E-07	0.900869
0.239989	-0.36316	0.291711	-0.1109	0.3419	0.225907	-0.04088	-0.34563	-0.21597
0.006575	2.71E-05	0.000876	0.214509	8.35E-05	0.010656	0.648189	6.89E-05	0.014741
0.190524	-0.30416	0.208666	-0.32212	0.386445	0.286495	-0.15379	-0.31045	-0.00616
0.031908	0.000508	0.018559	0.000221	7.2E-06	0.001093	0.0843	0.000382	0.945165
-0.45747	0.349894	-0.3871	0.1723	-0.17683	-0.31735	0.452142	0.212027	-0.2524
6.41E-08	5.52E-05	6.93E-06	0.052741	0.046725	0.000277	9.49E-08	0.016708	0.004199
-0.5317	0.518116	-0.39133	0.282154	-0.46154	-0.36545	0.718433	0.305972	-0.22306
1.26E-10	4.41E-10	5.38E-06	0.00131	4.72E-08	2.39E-05	1.94E-21	0.000468	0.01171
-0.39253	0.256961	-0.26432	0.543392	-0.39869	-0.44843	0.521839	0.355133	-0.34387
5.01E-06	0.003542	0.002674	4.08E-11	3.44E-06	1.24E-07	3.15E-10	4.19E-05	7.55E-05
-0.37021	0.150878	-0.1499	0.390946	-0.25948	-0.31166	0.638727	0.13941	-0.51062
1.83E-05	0.090417	0.092552	5.51E-06	0.00322	0.000361	6.51E-16	0.118009	8.62E-10
-0.20052	0.191667	-0.0287	0.625529	-0.42502	-0.34351	0.328617	0.271686	-0.44986
0.023796	0.030876	0.748782	3.78E-15	6.34E-07	7.69E-05	0.000162	0.002003	1.12E-07
-0.47887	0.523921	-0.34236	0.453812	-0.61389	-0.40679	0.410919	0.488938	-0.17407
1.23E-08	2.6E-10	8.15E-05	8.4E-08	1.66E-14	2.08E-06	1.6E-06	5.46E-09	0.050325
0.031937	-0.07302	-0.1099	-0.20508	-0.03834	0.19076	-0.35372	0.075829	0.278592
0.721504	0.414569	0.218709	0.020724	0.668701	0.031693	4.51E-05	0.396816	0.001516
-0.16677	0.228137	-0.30341	0.317542	-0.42243	-0.18405	0.088208	0.482682	-0.16384
0.060933	0.00989	0.000525	0.000275	7.53E-07	0.03833	0.324058	9.09E-09	0.065679
0.055253	0.374871	0.039027	0.068292	-0.36014	-0.2674	0.26371	0.049365	0.239462
0.537248	1.41E-05	0.663104	0.445526	3.2E-05	0.002372	0.002738	0.58153	0.006698

DERA	DGCR2	DGKQ	DGKZ	DHTKD1	DHX32	DIMT1	DIRAS3	DLD
cyan	cyan	cyan	cyan	cyan	cyan	cyan	cyan	cyan
0.054382	-0.29186	0.022553	0.069667	-0.23185	0.2345	-0.19215	-0.00613	-0.19372
0.54369	0.000871	0.801285	0.436397	0.00872	0.007962	0.030451	0.945492	0.029091
0.127322	-0.31669	0.014419	0.086723	-0.17232	0.278328	-0.18371	-0.07022	-0.13044
0.153734	0.000286	0.872178	0.332303	0.052711	0.001533	0.038692	0.432787	0.143836
0.212178	0.057644	-0.33736	-0.50382	0.319725	0.107452	0.459249	0.134573	0.371953
0.016629	0.519751	0.000105	1.56E-09	0.000248	0.229195	5.61E-08	0.131442	1.66E-05
0.347741	-0.33287	-0.64749	-0.7565	-0.04501	0.415151	0.665381	0.460872	0.585231
6.18E-05	0.000131	1.93E-16	8.28E-25	0.615345	1.22E-06	1.43E-17	4.97E-08	4.98E-13
0.55533	-0.51368	-0.20164	-0.28498	-0.39556	0.498974	0.449748	0.161081	0.155513
1.24E-11	6.57E-10	0.023006	0.001165	4.17E-06	2.36E-09	1.13E-07	0.070424	0.080836
0.668582	-0.72427	-0.69571	-0.79633	-0.44596	0.747863	0.821324	0.544134	0.624473
8.78E-18	6.43E-22	1.11E-19	4.53E-29	1.48E-07	5.43E-24	2.92E-32	3.8E-11	4.33E-15
0.236789	-0.47475	-0.42643	-0.4895	-0.41673	0.521259	0.391485	0.383061	0.292131
0.007355	1.71E-08	5.77E-07	5.22E-09	1.1E-06	3.32E-10	5.33E-06	8.79E-06	0.00086
0.172425	-0.34786	-0.38344	-0.25924	-0.4343	0.119748	0.152661	0.35271	0.160767
0.052567	6.14E-05	8.6E-06	0.003249	3.37E-07	0.179927	0.08663	4.76E-05	0.070981
-0.02219	-0.01561	-0.21484	-0.44382	-0.21352	0.267578	0.111486	0.374114	0.071166
0.804443	0.861735	0.015285	1.73E-07	0.015936	0.002356	0.212086	1.47E-05	0.426566
0.24206	-0.33826	-0.16211	-0.36388	-0.42958	0.35516	0.044089	0.295324	-0.10995
0.00611	0.0001	0.068615	2.61E-05	4.66E-07	4.18E-05	0.622586	0.00075	0.218469
0.191256	-0.32331	-0.26792	-0.40909	-0.38262	0.264911	0.131816	0.393151	0.075445
0.031243	0.000209	0.002324	1.79E-06	9.02E-06	0.002614	0.139606	4.82E-06	0.39922
-0.29423	0.446481	0.196306	0.178004	0.384953	-0.16939	-0.50375	-0.0536	-0.50062
0.000786	1.43E-07	0.02697	0.045265	7.87E-06	0.056933	1.57E-09	0.549513	2.05E-09
-0.44656	0.634016	0.261507	0.153554	0.594748	-0.50581	-0.41079	-0.27656	-0.37198
1.42E-07	1.23E-15	0.00298	0.084781	1.67E-13	1.31E-09	1.61E-06	0.001647	1.66E-05
-0.22354	0.514401	0.453798	0.357033	0.2422	-0.45332	-0.33547	-0.30637	-0.47556
0.011526	6.16E-10	8.4E-08	3.78E-05	0.00608	8.7E-08	0.000115	0.00046	1.6E-08
-0.26133	0.38176	0.284124	0.11208	0.160698	-0.29233	-0.46148	-0.14593	-0.54694
0.003	9.48E-06	0.001207	0.209645	0.071104	0.000853	4.75E-08	0.10161	2.88E-11
-0.20987	0.172007	0.35629	0.547034	0.156423	-0.25797	-0.50047	-0.43267	-0.45788
0.017874	0.053151	3.94E-05	2.85E-11	0.079056	0.00341	2.08E-09	3.77E-07	6.21E-08
-0.39731	0.65134	0.426611	0.590133	0.632056	-0.6476	-0.36817	-0.52449	-0.27392
3.75E-06	1.12E-16	5.7E-07	2.85E-13	1.6E-15	1.91E-16	2.06E-05	2.47E-10	0.001832
-0.02825	-0.07791	0.031357	0.279309	0.025225	-0.08339	0.191622	-0.07499	0.41231
0.752592	0.383959	0.72636	0.001472	0.778325	0.351281	0.030916	0.402099	1.46E-06
-0.32875	0.229362	0.453244	0.755849	0.117884	-0.39713	-0.35297	-0.3955	-0.0918
0.000161	0.009489	8.75E-08	9.57E-25	0.186851	3.79E-06	4.7E-05	4.18E-06	0.304669
-0.11007	0.12269	0.163526	-0.07385	0.446229	0.017849	0.051497	-0.27291	-0.18012
0.21798	0.169388	0.066208	0.409272	1.46E-07	0.842129	0.565301	0.001907	0.04273

DLL1	DMRTA1	DMWD	DNAAF2	DNAJB9	DNAJC22	DNAJC24	DNAJC25	DNAJC28
cyan	cyan	cyan	cyan	cyan	cyan	cyan	cyan	cyan
-0.14093	0.226686	-0.34284	-0.18129	0.065366	-0.135	-0.14947	0.211278	0.052124
0.114017	0.010383	7.96E-05	0.041376	0.465309	0.130209	0.093502	0.017106	0.560568
-0.12388	0.145028	-0.36482	-0.10177	0.113581	-0.14677	-0.0778	0.180886	0.092362
0.165263	0.103772	2.48E-05	0.254899	0.203566	0.099631	0.384639	0.041837	0.301702
0.341019	0.072753	0.058125	0.495422	-0.04552	0.33369	0.303258	-0.02728	0.166619
8.73E-05	0.416299	0.516268	3.19E-09	0.611321	0.000126	0.000529	0.76079	0.061173
-0.10787	0.38292	-0.3103	0.51365	0.352134	0.636747	0.607167	0.393291	0.323908
0.227398	8.86E-06	0.000385	6.59E-10	4.91E-05	8.52E-16	3.81E-14	4.78E-06	0.000203
-0.22028	0.244766	-0.64325	0.344802	0.30875	0.065946	0.259857	0.372198	0.34854
0.012826	0.005547	3.5E-16	7.19E-05	0.000413	0.461351	0.003174	1.64E-05	5.93E-05
-0.418	0.575574	-0.81255	0.541482	0.600967	0.593846	0.730464	0.686024	0.477397
1.01E-06	1.46E-12	4.36E-31	4.92E-11	8.03E-14	1.86E-13	1.93E-22	5.59E-19	1.39E-08
-0.3898	0.399289	-0.49387	0.270038	0.424467	0.346392	0.354441	0.506688	0.2315
5.9E-06	3.32E-06	3.63E-09	0.002138	6.58E-07	6.63E-05	4.34E-05	1.22E-09	0.008825
-0.39309	0.260961	-0.28172	-0.08424	0.08835	0.339132	0.131246	0.591142	0.036058
4.84E-06	0.003043	0.001333	0.346395	0.323276	9.61E-05	0.14134	2.54E-13	0.687337
-0.20864	0.17403	0.076227	-0.11034	0.114328	0.367622	0.081416	0.158727	-0.29177
0.018575	0.050372	0.394337	0.216843	0.200591	2.12E-05	0.36285	0.074685	0.000874
-0.40439	0.217516	-0.33058	-0.40112	0.096221	0.248486	-0.06041	0.425536	-0.26097
2.41E-06	0.014029	0.000147	2.96E-06	0.281867	0.004849	0.499876	6.12E-07	0.003043
-0.31651	0.338031	-0.36447	-0.19594	0.160564	0.349154	0.103811	0.425408	-0.14463
0.000289	0.000102	2.52E-05	0.027261	0.071343	5.74E-05	0.245455	6.18E-07	0.104726
0.19888	-0.3149	0.488867	-0.17249	-0.21366	-0.18767	-0.23304	-0.44697	-0.29596
0.024991	0.000311	5.5E-09	0.052475	0.015867	0.034611	0.008374	1.38E-07	0.000729
0.534733	-0.43175	0.80417	-0.221	-0.56677	-0.101	-0.46179	-0.50797	-0.37103
9.44E-11	4.02E-07	5.07E-30	0.012531	3.76E-12	0.258538	4.63E-08	1.09E-09	1.75E-05
0.413614	-0.48397	0.600551	-0.21226	-0.41946	-0.16757	-0.50114	-0.43385	-0.37233
1.34E-06	8.19E-09	8.44E-14	0.016584	9.17E-07	0.059684	1.96E-09	3.48E-07	1.63E-05
0.193843	-0.32855	0.511802	-0.59146	-0.47019	-0.02269	-0.5631	-0.22714	-0.58518
0.028988	0.000162	7.76E-10	2.45E-13	2.44E-08	0.800127	5.53E-12	0.010225	5.01E-13
0.145495	-0.26698	0.070643	-0.43741	-0.34831	-0.42103	-0.39564	-0.31233	-0.10413
0.102653	0.002412	0.429982	2.72E-07	6E-05	8.27E-07	4.15E-06	0.00035	0.243989
0.659789	-0.49712	0.582933	0.084861	-0.4476	-0.54306	-0.36594	-0.68087	-0.11659
3.28E-17	2.76E-09	6.45E-13	0.342831	1.32E-07	4.22E-11	2.33E-05	1.29E-18	0.191771
-0.02675	0.092984	-0.16806	0.404742	0.29076	-0.16996	0.366937	-0.07793	0.421307
0.765291	0.298447	0.058939	2.36E-06	0.000912	0.05609	2.2E-05	0.383837	8.12E-07
0.098534	-0.21041	0.165402	-0.01414	-0.05871	-0.62375	-0.18865	-0.38299	0.10075
0.270405	0.017577	0.063116	0.874627	0.512041	4.75E-15	0.033664	8.83E-06	0.259728
0.497522	-0.13625	0.132798	0.29301	-0.33447	-0.28559	-0.11101	-0.24514	0.197731
2.67E-09	0.126662	0.136657	0.000828	0.000121	0.001135	0.214038	0.005473	0.025858

DNAJC5	DNAJC8	DNTTIP2	DPH1	DPH3	DPH5	DPH6	DPM1	DR1
cyan	cyan	cyan	cyan	cyan	cyan	cyan	cyan	cyan
0.086867	0.049173	0.172702	-0.28004	0.180385	-0.0139	-0.1231	0.1194	-0.12888
0.3315	0.583005	0.052182	0.001429	0.042416	0.87674	0.167964	0.181203	0.148706
0.090198	0.060756	0.134538	-0.2567	0.177817	0.057758	-0.0934	0.133021	-0.17671
0.31322	0.497426	0.131544	0.003577	0.045495	0.518927	0.296256	0.135993	0.046881
-0.55264	0.244935	0.125553	0.152544	0.01573	0.412241	0.445933	0.168101	0.286428
1.62E-11	0.005514	0.159578	0.086875	0.860671	1.47E-06	1.49E-07	0.058873	0.001096
-0.71336	0.298099	0.395309	-0.1154	0.465571	0.527787	0.650488	0.475079	0.44179
4.95E-21	0.000664	4.23E-06	0.196383	3.48E-08	1.82E-10	1.26E-16	1.67E-08	2E-07
-0.42025	0.774171	0.445108	-0.35559	0.410566	0.714579	0.368183	0.609815	0.198346
8.71E-07	1.36E-26	1.58E-07	4.09E-05	1.63E-06	3.96E-21	2.06E-05	2.75E-14	0.025391
-0.7766	0.677434	0.675467	-0.47199	0.776233	0.83614	0.742024	0.834888	0.532387
7.54E-27	2.22E-18	3.03E-18	2.12E-08	8.25E-27	2.13E-34	1.86E-23	3.28E-34	1.18E-10
-0.41781	0.427222	0.502276	-0.26659	0.628766	0.51185	0.481586	0.594516	0.156086
1.02E-06	5.47E-07	1.78E-09	0.002448	2.47E-15	7.73E-10	9.93E-09	1.72E-13	0.079711
-0.16976	0.235601	0.262156	-0.45865	0.445256	0.183322	0.151619	0.17856	0.020504
0.056389	0.007665	0.002907	5.87E-08	1.56E-07	0.03911	0.088827	0.044586	0.819021
-0.29979	-0.05424	0.139775	-0.13583	0.1511	0.009206	-0.03268	0.086127	-0.03918
0.000617	0.544774	0.117041	0.127836	0.089937	0.91818	0.715322	0.33565	0.661871
-0.15711	0.072284	0.296308	-0.55188	0.397475	0.027669	-0.09655	0.148046	-0.15543
0.077724	0.419317	0.000718	1.75E-11	3.71E-06	0.75748	0.280222	0.096701	0.081003
-0.19746	0.085877	0.306999	-0.43878	0.437803	0.087045	0.063514	0.15519	0.066489
0.026063	0.33706	0.000447	2.47E-07	2.64E-07	0.330505	0.47807	0.081476	0.457656
0.206433	-0.27307	-0.43362	0.297514	-0.42655	-0.3056	-0.34034	-0.51465	-0.45777
0.019884	0.001895	3.53E-07	0.000681	5.72E-07	0.000476	9.04E-05	6.03E-10	6.26E-08
0.166619	-0.45866	-0.45682	0.479004	-0.59447	-0.45937	-0.33037	-0.62317	-0.41565
0.061172	5.86E-08	6.72E-08	1.22E-08	1.73E-13	5.56E-08	0.000149	5.12E-15	1.18E-06
0.234011	-0.1839	-0.4441	0.365746	-0.58956	-0.33343	-0.45143	-0.47138	-0.37764
0.008098	0.038489	1.7E-07	2.35E-05	3.05E-13	0.000128	9.99E-08	2.22E-08	1.2E-05
0.225012	-0.39892	-0.30775	0.031619	-0.35801	-0.49639	-0.5467	-0.54513	-0.55413
0.010978	3.39E-06	0.000432	0.724169	3.59E-05	2.94E-09	2.94E-11	3.44E-11	1.4E-11
0.505173	-0.2324	-0.32506	-0.07816	-0.21708	-0.38944	-0.43995	-0.42988	-0.40551
1.39E-09	0.008559	0.000192	0.3824	0.014228	6.03E-06	2.27E-07	4.56E-07	2.25E-06
0.419688	-0.26767	-0.51686	0.553152	-0.62717	-0.3483	-0.22556	-0.57013	-0.14427
9.03E-07	0.002347	4.94E-10	1.54E-11	3.05E-15	6E-05	0.01078	2.63E-12	0.10562
0.155678	0.001369	-0.02031	0.209577	0.024561	0.060965	0.244719	0.202433	0.518437
0.080511	0.987815	0.820695	0.018041	0.784017	0.495949	0.005556	0.022464	4.29E-10
0.623246	-0.20896	-0.29476	0.31697	-0.33397	-0.31555	-0.24835	-0.21532	0.024738
5.07E-15	0.01839	0.000768	0.000282	0.000124	0.000302	0.004873	0.01505	0.782493
-0.29245	0.210222	0.068792	0.225115	-0.2254	0.201071	0.228586	0.09439	0.002932
0.000849	0.017681	0.442192	0.010941	0.010839	0.023405	0.009741	0.291169	0.973898

DTD2	DTWD1	DTX1	DUS4L	DUSP12	DUSP8	DYNC1H1	DYNC2LI1	DYNLT1
cyan	cyan	cyan	cyan	cyan	cyan	cyan	cyan	cyan
-0.03064	0.118553	-0.16026	0.000351	-0.16854	0.029901	0.062962	0.188184	-0.12021
0.732374	0.184344	0.071881	0.996879	0.0582	0.738597	0.481914	0.034114	0.178235
0.038892	0.138481	-0.11114	0.050971	-0.14469	0.080866	0.075929	0.231609	-0.14656
0.664198	0.120504	0.21353	0.569286	0.104583	0.366112	0.396194	0.008793	0.100142
0.229405	0.08357	-0.02926	0.300731	0.696322	-0.33092	-0.01898	-0.07055	0.335354
0.009476	0.35025	0.744021	0.000592	1E-19	0.000145	0.832254	0.430614	0.000116
0.391383	0.468151	-0.44418	0.663242	0.705496	-0.64491	-0.19023	0.346612	0.438429
5.37E-06	2.86E-08	1.69E-07	1.97E-17	2.04E-20	2.78E-16	0.032177	6.55E-05	2.53E-07
0.40079	0.343963	-0.22619	0.405921	0.436257	-0.30557	-0.72389	0.504266	0.604546
3.02E-06	7.51E-05	0.010555	2.19E-06	2.94E-07	0.000477	6.91E-22	1.5E-09	5.23E-14
0.573067	0.715318	-0.63306	0.85611	0.728117	-0.77086	-0.66324	0.726927	0.712127
1.91E-12	3.46E-21	1.4E-15	1.21E-37	3.05E-22	3.03E-26	1.97E-17	3.85E-22	6.21E-21
0.203617	0.424135	-0.44035	0.57916	0.373927	-0.47876	-0.3925	0.606652	0.360383
0.021672	6.73E-07	2.21E-07	9.82E-13	1.49E-05	1.24E-08	5.02E-06	4.05E-14	3.16E-05
0.00842	0.232455	-0.24877	0.275268	0.043813	-0.38875	-0.36596	0.529259	0.350463
0.92515	0.008542	0.004798	0.001735	0.624771	6.28E-06	2.33E-05	1.58E-10	5.36E-05
-0.26124	-0.08879	-0.39226	0.062813	0.199903	-0.47734	-0.12073	-0.17444	0.325388
0.003011	0.320872	5.09E-06	0.482955	0.02424	1.39E-08	0.176364	0.049829	0.000189
-0.1947	-0.02438	-0.49967	0.098851	-0.04906	-0.53249	-0.41165	0.07927	0.446974
0.028268	0.78553	2.23E-09	0.268861	0.583832	1.17E-10	1.52E-06	0.375679	1.38E-07
-0.03495	0.177744	-0.38738	0.296481	0.062882	-0.54213	-0.2404	0.182042	0.383943
0.696432	0.045585	6.81E-06	0.000713	0.482474	4.62E-11	0.006479	0.040524	8.35E-06
-0.2405	-0.44621	0.244174	-0.35374	-0.23443	0.219343	0.567427	-0.55512	-0.30409
0.006459	1.46E-07	0.005666	4.51E-05	0.007982	0.013224	3.5E-12	1.26E-11	0.00051
-0.45236	-0.60175	0.408318	-0.49192	-0.10291	0.262117	0.566234	-0.72002	-0.28928
9.34E-08	7.31E-14	1.88E-06	4.27E-09	0.2496	0.002911	3.98E-12	1.44E-21	0.000972
-0.29608	-0.60266	0.490084	-0.55859	-0.19239	0.313709	0.031154	-0.63745	-0.00012
0.000726	6.56E-14	4.97E-09	8.85E-12	0.030237	0.000329	0.728063	7.75E-16	0.998902
-0.54668	-0.63229	0.142249	-0.51713	-0.33498	0.033564	0.144051	-0.63114	0.001865
2.95E-11	1.55E-15	0.110637	4.82E-10	0.000118	0.707947	0.106147	1.81E-15	0.983401
-0.04658	-0.30261	0.230715	-0.4144	-0.57424	0.491593	0.313373	-0.1513	-0.4713
0.603046	0.000544	0.009064	1.28E-06	1.69E-12	4.39E-09	0.000334	0.089515	2.24E-08
-0.03285	-0.43123	0.675644	-0.48197	-0.24237	0.737665	0.573827	-0.46702	-0.56091
0.713884	4.16E-07	2.95E-18	9.63E-09	0.006043	4.55E-23	1.76E-12	3.11E-08	6.95E-12
0.413713	0.40488	0.119992	0.16075	-0.01792	0.309076	0.166605	0.319128	-0.38419
1.33E-06	2.34E-06	0.179037	0.07101	0.84153	0.000407	0.061195	0.000255	8.23E-06
-0.01688	-0.04873	0.445327	-0.33685	-0.48224	0.739793	0.356573	0.005433	-0.6585
0.850628	0.586441	1.55E-07	0.000108	9.42E-09	2.95E-23	3.88E-05	0.95166	3.97E-17
0.017798	-0.11896	0.093268	-0.11146	0.393589	0.167981	0.17863	-0.20184	-0.068
0.842572	0.182845	0.296968	0.212188	4.7E-06	0.059057	0.0445	0.022869	0.447476

DYRK1B	E2F6	EAF2	EBPL	ECHDC1	ECI2	EEA1	EED	EEF1E1
cyan	cyan	cyan	cyan	cyan	cyan	cyan	cyan	cyan
0.009873	-0.12992	0.164864	-0.01969	0.190832	-0.17988	-0.06769	-0.0542	0.093634
0.912278	0.145437	0.06399	0.826105	0.031627	0.043007	0.449543	0.545058	0.295066
-0.02345	-0.06747	0.158513	0.047892	0.178163	-0.0721	-0.22211	-0.02516	0.143355
0.793555	0.451008	0.075083	0.592867	0.045069	0.420502	0.012084	0.778888	0.107863
-0.27108	0.58112	-0.12011	0.411845	0.004753	0.285932	0.220151	0.42301	0.183303
0.002051	7.9E-13	0.178613	1.5E-06	0.957707	0.001119	0.012881	7.25E-07	0.039131
-0.65319	0.783284	0.222586	0.477201	0.325212	0.382875	0.574795	0.619453	0.358892
8.59E-17	1.42E-27	0.011895	1.41E-08	0.000191	8.89E-06	1.59E-12	8.25E-15	3.42E-05
-0.45487	0.323343	0.464983	0.547233	0.33758	0.360161	-0.3544	0.449541	0.505519
7.77E-08	0.000209	3.64E-08	2.79E-11	0.000104	3.19E-05	4.35E-05	1.15E-07	1.35E-09
-0.89406	0.808488	0.627123	0.667425	0.637425	0.487803	0.349668	0.795463	0.659171
2.01E-45	1.45E-30	3.07E-15	1.05E-17	7.78E-16	6E-09	5.59E-05	5.74E-29	3.6E-17
-0.61851	0.514269	0.331148	0.514921	0.268226	0.395731	0.031445	0.318163	0.367735
9.3E-15	6.23E-10	0.000143	5.88E-10	0.002297	4.12E-06	0.725628	0.000267	2.11E-05
-0.2965	0.089394	0.334637	-0.08403	0.308436	0.000315	0.206983	0.247096	0.058305
0.000712	0.31757	0.00012	0.3476	0.000419	0.997198	0.019551	0.0051	0.514966
-0.14379	0.161517	0.038028	0.187653	0.112226	0.203041	0.401942	0.149128	-0.08544
0.106798	0.069656	0.671221	0.034632	0.209048	0.022054	2.81E-06	0.09426	0.339515
-0.18829	-0.07399	0.436693	-0.03165	0.476663	0.076188	0.26903	0.146902	0.00395
0.034013	0.408382	2.86E-07	0.723934	1.47E-08	0.394576	0.002225	0.099334	0.964846
-0.2827	0.17173	0.415526	0.005219	0.502406	0.07153	0.371334	0.254866	0.0718
0.001281	0.053541	1.19E-06	0.953563	1.76E-09	0.424197	1.72E-05	0.003832	0.422451
0.450355	-0.16383	-0.45942	-0.12379	-0.42579	0.193582	-0.31479	-0.42207	-0.41973
1.08E-07	0.065694	5.54E-08	0.165584	6.02E-07	0.029209	0.000313	7.72E-07	9.01E-07
0.562752	-0.28057	-0.58168	-0.27735	-0.53709	-0.12831	-0.02392	-0.38334	-0.51518
5.74E-12	0.001398	7.42E-13	0.001595	7.53E-11	0.150541	0.789521	8.65E-06	5.75E-10
0.606658	-0.46573	-0.31968	-0.28995	-0.37823	-0.11875	-0.27238	-0.33739	-0.36748
4.05E-14	3.44E-08	0.000249	0.000944	1.16E-05	0.183623	0.001948	0.000105	2.14E-05
0.511703	-0.52107	-0.18618	-0.42239	-0.14509	-0.13811	0.008748	-0.37086	-0.49965
7.83E-10	3.38E-10	0.036103	7.55E-07	0.103617	0.121508	0.92224	1.77E-05	2.23E-09
0.392432	-0.52325	-0.16985	-0.48748	-0.04063	-0.29812	-0.44487	-0.4401	-0.18657
5.04E-06	2.77E-10	0.056247	6.16E-09	0.650157	0.000664	1.6E-07	2.25E-07	0.035713
0.543302	-0.27899	-0.61308	-0.28972	-0.57226	-0.25038	-0.4699	-0.47598	-0.32661
4.12E-11	0.001492	1.84E-14	0.000954	2.09E-12	0.004524	2.5E-08	1.55E-08	0.000178
-0.14322	0.19408	-0.07109	0.050233	-0.05543	-0.17	0.067383	0.066391	0.264325
0.108209	0.028788	0.42707	0.574897	0.535918	0.056031	0.451623	0.45832	0.002674
0.354358	-0.36848	-0.30966	-0.26956	-0.39414	-0.36668	-0.43059	-0.43211	-0.1213
4.36E-05	2.02E-05	0.000396	0.002179	4.54E-06	2.23E-05	4.35E-07	3.92E-07	0.174326
0.054005	0.155657	-0.32195	0.368102	-0.41256	-0.01022	-0.3388	0.055899	0.094205
0.546491	0.080553	0.000223	2.06E-05	1.44E-06	0.909248	9.77E-05	0.532488	0.292117

EFCAB11	EGF	EHMT2	EIF2A	EIF2B2	EIF2B5	EIF2D	EIF2S1	EIF3E
cyan	cyan	cyan	cyan	cyan	cyan	cyan	cyan	cyan
0.125092	0.144078	-0.1064	0.042781	-0.05025	0.023196	0.004639	-0.21508	0.1205
0.16113	0.10608	0.233828	0.632958	0.574731	0.795745	0.958715	0.015169	0.177187
0.115207	0.100327	-0.09993	0.051556	0.030078	0.066083	-0.02157	-0.16116	0.121376
0.197126	0.261743	0.26363	0.564853	0.737105	0.460413	0.809821	0.070276	0.174036
0.131248	0.038167	0.079476	0.335472	0.38506	0.413343	0.142789	0.630642	0.236875
0.141335	0.670088	0.374433	0.000115	7.82E-06	1.37E-06	0.109276	1.93E-15	0.007333
0.374893	0.431458	-0.45509	0.545741	0.35308	0.350575	0.403	0.750223	0.439197
1.41E-05	4.1E-07	7.64E-08	3.24E-11	4.67E-05	5.33E-05	2.63E-06	3.27E-24	2.4E-07
0.353186	-0.01707	-0.40552	0.556553	0.484562	0.348351	0.536818	0.254611	0.635185
4.64E-05	0.848914	2.25E-06	1.09E-11	7.81E-09	5.99E-05	7.73E-11	0.003868	1.05E-15
0.557181	0.494938	-0.80613	0.795404	0.488827	0.374537	0.648171	0.706472	0.772149
1.02E-11	3.32E-09	2.89E-30	5.84E-29	5.51E-09	1.44E-05	1.76E-16	1.72E-20	2.22E-26
0.30144	0.352364	-0.55857	0.549636	0.3895	0.403662	0.630289	0.390496	0.482883
0.000573	4.85E-05	8.86E-12	2.2E-11	6.01E-06	2.53E-06	2.02E-15	5.66E-06	8.95E-09
0.285893	0.327505	-0.38698	0.06597	-0.1719	-0.08684	0.377252	-0.12037	0.170924
0.001121	0.000171	6.98E-06	0.461182	0.053299	0.331667	1.23E-05	0.177678	0.05469
-0.11221	0.097408	-0.20393	0.192691	0.213803	0.094285	0.201081	0.29131	0.074283
0.209127	0.275945	0.021467	0.029974	0.015796	0.291709	0.023398	0.000891	0.406541
0.071904	0.253952	-0.37627	0.040447	-0.02322	-0.06355	0.33857	-0.16806	0.21685
0.421774	0.003964	1.3E-05	0.651635	0.795523	0.477846	9.89E-05	0.058935	0.014332
0.163917	0.487627	-0.36466	0.085925	0.019329	-0.02841	0.277312	0.060951	0.159179
0.065554	6.08E-09	2.5E-05	0.336791	0.82923	0.751192	0.001597	0.496047	0.07385
-0.36099	-0.17696	0.392092	-0.35461	0.119264	0.01505	-0.22689	-0.1233	-0.37929
3.05E-05	0.046557	5.14E-06	4.31E-05	0.181707	0.86663	0.010312	0.167264	1.09E-05
-0.36985	-0.30378	0.735254	-0.4569	-0.09723	0.047288	-0.28888	-0.18437	-0.48896
1.87E-05	0.000517	7.42E-23	6.68E-08	0.276846	0.597547	0.000988	0.037986	5.45E-09
-0.29496	-0.45073	0.667298	-0.43558	-0.01398	-0.11829	-0.1842	-0.37726	-0.43154
0.000762	1.05E-07	1.07E-17	3.09E-07	0.876058	0.185308	0.038161	1.23E-05	4.07E-07
-0.35211	-0.18743	0.472858	-0.51554	-0.19635	-0.17243	-0.11735	-0.51236	-0.40821
4.92E-05	0.034848	1.98E-08	5.56E-10	0.026935	0.052566	0.188863	7.39E-10	1.9E-06
-0.18727	-0.24854	0.330935	-0.49482	-0.37693	-0.23043	-0.38042	-0.65627	-0.32059
0.035011	0.004838	0.000145	3.35E-09	1.25E-05	0.009152	1.03E-05	5.5E-17	0.000238
-0.32106	-0.49125	0.710566	-0.41859	-0.18269	-0.05125	-0.5529	-0.21815	-0.45383
0.000233	4.51E-09	8.24E-21	9.71E-07	0.039801	0.567188	1.58E-11	0.013743	8.39E-08
0.137428	0.035873	-0.17985	0.135291	-0.15236	-0.15385	-0.30285	0.242138	0.009215
0.123379	0.688858	0.043041	0.129378	0.08727	0.084185	0.000538	0.006093	0.918102
-0.19927	-0.30156	0.219676	-0.26805	-0.33415	-0.28217	-0.4862	-0.29672	-0.308
0.024702	0.00057	0.013082	0.002312	0.000123	0.001309	6.84E-09	0.000705	0.000427
-0.13638	-0.44837	0.288937	0.311549	0.241293	0.547162	-0.00595	0.223117	0.249027
0.126306	1.25E-07	0.000986	0.000363	0.006279	2.81E-11	0.947097	0.011688	0.004754

EIF3M	ELAVL3	ELF2	ELK3	ELOVL6	ELP4	EMC8	ENKUR	ENTHD1
cyan	cyan	cyan	cyan	cyan	cyan	cyan	cyan	cyan
0.049278	0.080349	0.044518	-0.12381	-0.00769	-0.15485	-0.24707	-0.16759	0.126102
0.582196	0.369192	0.619203	0.165484	0.931613	0.082149	0.005104	0.05966	0.157746
0.092668	0.105332	0.007714	-0.23978	0.058835	-0.04671	-0.13269	-0.13034	0.117221
0.3001	0.238565	0.931408	0.006623	0.511146	0.602003	0.136968	0.144118	0.189358
0.29634	-0.22159	0.320395	0.350442	0.368262	0.297552	0.506355	0.283477	0.079205
0.000717	0.012291	0.00024	5.37E-05	2.05E-05	0.00068	1.25E-09	0.00124	0.37607
0.579266	-0.53132	0.626454	0.640462	0.573245	0.484138	0.443813	0.509316	0.388984
9.71E-13	1.3E-10	3.35E-15	5.14E-16	1.88E-12	8.08E-09	1.73E-07	9.67E-10	6.2E-06
0.463133	-0.34303	0.10895	0.093374	0.098819	0.27715	0.600294	0.308987	0.34652
4.19E-08	7.88E-05	0.22273	0.296416	0.269014	0.001608	8.7E-14	0.000408	6.58E-05
0.825336	-0.73892	0.615527	0.632741	0.571341	0.577517	0.620874	0.602419	0.609676
8.05E-33	3.52E-23	1.35E-14	1.46E-15	2.31E-12	1.18E-12	6.88E-15	6.75E-14	2.8E-14
0.469408	-0.43731	0.464203	0.379469	0.380649	0.305866	0.350171	0.325141	0.462319
2.59E-08	2.74E-07	3.86E-08	1.08E-05	1.01E-05	0.00047	5.44E-05	0.000192	4.45E-08
0.198875	-0.42534	0.266338	0.184814	-0.16581	0.149953	-0.0638	0.551721	0.196525
0.024994	6.2E-07	0.002473	0.037516	0.062457	0.092431	0.476069	1.78E-11	0.026797
0.217466	-0.32701	0.197908	0.728693	0.096875	-0.18982	0.029022	-0.15635	0.38356
0.014051	0.000175	0.025723	2.73E-22	0.278595	0.032557	0.746015	0.079205	8.54E-06
0.220879	-0.36469	0.184382	0.54272	-0.14323	-0.21994	-0.09298	-0.06396	0.333589
0.012579	2.49E-05	0.037972	4.36E-11	0.108164	0.01297	0.298482	0.475009	0.000127
0.310507	-0.39401	0.263189	0.515103	0.040864	0.061046	-0.03439	0.113181	0.273962
0.000381	4.58E-06	0.002794	5.79E-10	0.648285	0.495375	0.701139	0.205175	0.001829
-0.30298	0.441934	-0.23449	-0.18671	-0.20525	-0.16283	-0.11379	-0.34076	-0.10982
0.000535	1.98E-07	0.007965	0.035568	0.020619	0.067394	0.202744	8.85E-05	0.219032
-0.46776	0.428459	-0.17575	-0.10739	-0.27575	-0.34204	-0.21391	-0.30958	-0.3463
2.94E-08	5.03E-07	0.0481	0.229463	0.001702	8.29E-05	0.015744	0.000398	6.66E-05
-0.47807	0.39454	-0.5252	-0.21929	-0.48439	-0.39447	-0.01674	-0.30938	-0.31882
1.31E-08	4.43E-06	2.31E-10	0.013246	7.92E-09	4.45E-06	0.851823	0.000401	0.000259
-0.39808	0.248048	-0.24057	0.104642	-0.49983	-0.55102	-0.32642	-0.40509	-0.17577
3.57E-06	0.004926	0.006442	0.241676	2.2E-09	1.91E-11	0.00018	2.31E-06	0.048083
-0.42704	0.615509	-0.27082	-0.68519	-0.31202	-0.27946	-0.40448	-0.2971	-0.46843
5.53E-07	1.36E-14	0.002073	6.41E-19	0.000356	0.001463	2.4E-06	0.000694	2.79E-08
-0.56078	0.717702	-0.43533	-0.73017	-0.22267	-0.10092	-0.10069	-0.2128	-0.64401
7.05E-12	2.22E-21	3.14E-07	2.04E-22	0.011864	0.258908	0.25999	0.016308	3.14E-16
0.028629	0.039446	-0.02099	-0.33934	0.352172	0.339903	-0.0066	0.150541	-0.168
0.749338	0.659716	0.814773	9.51E-05	4.9E-05	9.24E-05	0.94127	0.091147	0.059028
-0.40781	0.437025	-0.42975	-0.75796	-0.13007	-0.05088	-0.30461	-0.22539	-0.40312
1.95E-06	2.79E-07	4.61E-07	5.98E-25	0.144982	0.56994	0.000498	0.010841	2.61E-06
-0.06089	0.233534	0.124561	-0.16961	0.130014	-0.16245	0.224086	-0.1471	-0.07646
0.496493	0.008232	0.162926	0.056612	0.145148	0.068043	0.01132	0.098882	0.39286

EPHB1	EPN1	EPS15L1	ERCC8	ERF	ERGIC2	ERH	ERICH2	ERLIN1
cyan	cyan	cyan	cyan	cyan	cyan	cyan	cyan	cyan
-0.09941	-0.13653	-0.20549	-0.17635	-0.27352	0.370101	0.034967	0.044472	-0.06996
0.266167	0.125878	0.020469	0.04734	0.001862	1.85E-05	0.69633	0.619568	0.434469
-0.0834	-0.14729	-0.13153	-0.15971	-0.27894	0.340243	0.122826	0.098894	-0.06814
0.35125	0.098435	0.140469	0.072874	0.001495	9.08E-05	0.168913	0.268651	0.446554
0.01012	-0.21867	0.032346	0.627644	0.091981	0.035908	0.424022	0.086468	0.412762
0.910091	0.013515	0.718091	2.87E-15	0.303713	0.688577	6.78E-07	0.333735	1.42E-06
-0.39238	-0.69958	-0.24269	0.74379	-0.33321	0.276343	0.38565	0.368373	0.544986
5.05E-06	5.74E-20	0.005975	1.29E-23	0.000129	0.001661	7.55E-06	2.03E-05	3.49E-11
-0.25528	-0.31203	-0.48761	0.271466	-0.3643	0.50309	0.595274	0.36797	0.184903
0.003773	0.000355	6.09E-09	0.002021	2.55E-05	1.66E-09	1.57E-13	2.08E-05	0.037422
-0.59888	-0.87526	-0.63082	0.723149	-0.67327	0.644447	0.613386	0.574792	0.55546
1.03E-13	2.99E-41	1.89E-15	7.96E-22	4.27E-18	2.96E-16	1.77E-14	1.59E-12	1.22E-11
-0.37712	-0.65696	-0.28896	0.428168	-0.46648	0.336015	0.300888	0.450594	0.363871
1.24E-05	4.98E-17	0.000985	5.13E-07	3.25E-08	0.000112	0.000588	1.06E-07	2.61E-05
-0.27588	-0.42033	-0.39459	-0.06384	-0.30104	0.049591	-0.12027	0.307041	-0.06985
0.001693	8.66E-07	4.42E-06	0.475803	0.000584	0.5798	0.178032	0.000446	0.43519
-0.25037	-0.12004	-0.07532	0.127347	-0.02509	0.017422	0.028933	-0.02686	0.429105
0.004525	0.178865	0.400021	0.153651	0.779455	0.845855	0.746769	0.764336	4.81E-07
-0.35082	-0.19408	-0.52468	-0.17376	-0.24004	0.275606	-0.10648	-0.07398	0.049749
5.26E-05	0.02879	2.42E-10	0.050741	0.006564	0.001712	0.233461	0.408437	0.578588
-0.41166	-0.36658	-0.45239	0.010705	-0.34007	0.242965	0.006965	0.09167	0.130319
1.52E-06	2.25E-05	9.32E-08	0.904922	9.16E-05	0.005916	0.93805	0.305357	0.144199
0.14535	0.347173	0.596678	-0.19523	0.220094	-0.54595	-0.1056	-0.16121	0.052312
0.102999	6.36E-05	1.33E-13	0.027835	0.012905	3.17E-11	0.237348	0.070199	0.559156
0.419961	0.540737	0.574852	-0.21952	0.681568	-0.57828	-0.31625	-0.42206	-0.14973
8.87E-07	5.29E-11	1.58E-12	0.013149	1.15E-18	1.08E-12	0.000292	7.72E-07	0.092926
0.326537	0.788119	0.304021	-0.45931	0.723439	-0.42013	-0.16834	-0.36424	-0.29478
0.000179	4.07E-28	0.000511	5.58E-08	7.53E-22	8.77E-07	0.058514	2.56E-05	0.000768
0.18114	0.548607	0.138181	-0.55857	0.53099	-0.36434	-0.43897	-0.45877	-0.28333
0.041545	2.44E-11	0.121317	8.86E-12	1.35E-10	2.54E-05	2.44E-07	5.81E-08	0.001247
0.294604	0.356146	0.043568	-0.53413	0.168462	-0.09987	-0.34072	-0.30247	-0.61986
0.000773	3.97E-05	0.62671	9.99E-11	0.058324	0.26395	8.86E-05	0.000548	7.83E-15
0.657759	0.591546	0.589477	-0.15377	0.565774	-0.51292	-0.12718	-0.32276	-0.41093
4.43E-17	2.42E-13	3.08E-13	0.084332	4.18E-12	7.03E-10	0.154192	0.000215	1.6E-06
0.016071	-0.19011	0.095119	0.28683	-0.24777	0.129742	0.132445	0.194515	0.044564
0.857676	0.032285	0.287443	0.001078	0.004977	0.145998	0.13771	0.028425	0.61884
0.366331	0.330972	0.364426	-0.25313	0.050673	-0.18313	-0.17087	-0.09818	-0.33094
2.28E-05	0.000144	2.53E-05	0.004087	0.571549	0.039325	0.05477	0.272116	0.000144
0.317922	0.105956	0.194007	0.326245	0.24525	0.052441	0.295039	-0.02392	0.097386
0.00027	0.23578	0.02885	0.000182	0.005451	0.558183	0.000759	0.78951	0.276055

ERMARD	ERO1A	ESCO1	ESD	ETFA	EVI5	EVL	EXOC3	EXOG
cyan	cyan	cyan	cyan	cyan	cyan	cyan	cyan	cyan
0.111403	0.173637	-0.09549	0.187561	-0.13731	0.008424	0.211813	0.145961	-0.19672
0.212425	0.050903	0.285552	0.034722	0.123703	0.925108	0.016821	0.101544	0.026639
0.130195	0.168467	-0.10478	0.255259	-0.07529	-0.05391	0.191758	0.092831	-0.14941
0.144584	0.058316	0.24104	0.003776	0.400201	0.54717	0.030795	0.299247	0.093625
0.047333	0.066677	0.457466	-0.03476	0.143699	-0.03261	-0.36256	-0.5558	0.435294
0.597195	0.456385	6.41E-08	0.698035	0.107012	0.715922	2.8E-05	1.18E-11	3.15E-07
0.375581	0.393144	0.683	0.229072	0.346209	0.453897	-0.75673	-0.67561	0.590925
1.35E-05	4.83E-06	9.14E-19	0.009583	6.69E-05	8.34E-08	7.87E-25	2.96E-18	2.6E-13
0.455173	0.078455	0.248856	0.637861	0.619242	-0.06499	-0.12678	-0.30489	0.170134
7.6E-08	0.380619	0.004783	7.33E-16	8.47E-15	0.467861	0.1555	0.000492	0.055835
0.662179	0.469905	0.70382	0.657934	0.740792	0.503006	-0.7683	-0.70649	0.547024
2.3E-17	2.49E-08	2.74E-20	4.31E-17	2.4E-23	1.67E-09	5.56E-26	1.71E-20	2.85E-11
0.427638	0.345505	0.461355	0.575418	0.37185	0.235199	-0.52829	-0.41062	0.342115
5.31E-07	6.94E-05	4.79E-08	1.48E-12	1.67E-05	0.007772	1.73E-10	1.63E-06	8.26E-05
0.504083	0.015852	0.000304	0.32897	0.132507	0.570142	-0.3991	-0.06286	0.223319
1.52E-09	0.859598	0.997289	0.000159	0.137526	2.62E-12	3.36E-06	0.482633	0.011611
-0.05175	0.466678	0.131952	-0.15071	0.014453	0.126673	-0.41497	-0.06236	0.128279
0.563372	3.2E-08	0.139195	0.09079	0.871875	0.155857	1.23E-06	0.48614	0.150637
0.296571	0.2771	-0.08807	0.152151	0.198351	0.323026	-0.16372	0.160341	0.004786
0.00071	0.001611	0.324822	0.0877	0.025387	0.000212	0.065889	0.071742	0.957408
0.284596	0.262474	0.052694	0.132708	0.164344	0.405372	-0.26301	-0.0393	0.175105
0.001183	0.002872	0.556282	0.136923	0.064845	2.27E-06	0.002814	0.660913	0.048946
-0.37191	-0.10391	-0.3027	-0.36421	-0.41942	-0.34268	0.264734	0.331823	-0.04082
1.67E-05	0.245012	0.000542	2.56E-05	9.19E-07	8.02E-05	0.002632	0.000138	0.648674
-0.44641	-0.23661	-0.27865	-0.62575	-0.65086	-0.39184	0.300605	0.311527	-0.08979
1.44E-07	0.007401	0.001512	3.67E-15	1.2E-16	5.22E-06	0.000595	0.000364	0.315432
-0.36666	-0.3681	-0.50357	-0.5125	-0.30617	-0.52899	0.495098	0.497523	-0.34235
2.24E-05	2.07E-05	1.59E-09	7.3E-10	0.000464	1.62E-10	3.28E-09	2.67E-09	8.16E-05
-0.2433	-0.1476	-0.51116	-0.51368	-0.4406	-0.19814	0.339641	0.60258	-0.25469
0.005846	0.097723	8.22E-10	6.57E-10	2.17E-07	0.025545	9.36E-05	6.62E-14	0.003857
-0.11172	-0.47929	-0.46536	-0.08151	-0.28533	-0.11181	0.646265	0.479272	-0.27882
0.211132	1.19E-08	3.54E-08	0.362288	0.001148	0.210737	2.3E-16	1.2E-08	0.001502
-0.54717	-0.58692	-0.26719	-0.36355	-0.40333	-0.5046	0.615035	0.144216	-0.23841
2.81E-11	4.12E-13	0.002392	2.66E-05	2.58E-06	1.46E-09	1.44E-14	0.105742	0.00695
-0.07703	-0.0618	0.260831	0.134566	0.180751	0.144802	-0.04779	-0.33117	-0.00117
0.38935	0.49003	0.003058	0.131464	0.041992	0.104317	0.593691	0.000143	0.98954
-0.36216	-0.32791	-0.26369	-0.03467	-0.17855	-0.27426	0.477547	0.165255	-0.37683
2.86E-05	0.000168	0.00274	0.698803	0.044602	0.001807	1.37E-08	0.063353	1.26E-05
-0.09654	-0.1006	0.298518	0.026355	-0.1548	-0.48548	0.240563	-0.14062	0.11605
0.280244	0.260453	0.000652	0.76867	0.082254	7.25E-09	0.006443	0.114823	0.193848

EXOSC2	EXOSC8	EXOSC9	F2RL1	FAIM	FAM114A2	FAM120A(	FAM151B	FAM155B
cyan	cyan	cyan	cyan	cyan	cyan	cyan	cyan	cyan
-0.14347	-0.10102	-0.04143	-0.11797	-0.19374	0.018718	0.230379	-0.05597	-0.01806
0.107566	0.258423	0.643722	0.186511	0.029074	0.834551	0.009168	0.531983	0.840315
-0.07847	-0.0519	-0.00217	-0.17859	-0.2373	-0.04795	0.238497	-0.13614	0.066149
0.380522	0.562231	0.980647	0.044549	0.007226	0.592406	0.006929	0.126975	0.45997
0.586048	0.573724	0.511333	0.352913	0.203753	0.369389	-0.03439	0.216324	-0.06965
4.54E-13	1.78E-12	8.09E-10	4.71E-05	0.021582	1.92E-05	0.70107	0.014576	0.436541
0.465291	0.630497	0.546335	0.565755	0.492608	0.740356	0.368838	0.489092	-0.40068
3.56E-08	1.97E-15	3.05E-11	4.18E-12	4.03E-09	2.62E-23	1.98E-05	5.39E-09	3.04E-06
0.31805	0.509723	0.572334	0.113631	0.226884	0.298926	0.488386	0.235001	-0.17184
0.000269	9.33E-10	2.07E-12	0.203366	0.010314	0.000641	5.72E-09	0.007826	0.053391
0.438358	0.725164	0.736809	0.571407	0.600089	0.86188	0.749956	0.602051	-0.53899
2.54E-07	5.41E-22	5.42E-23	2.29E-12	8.92E-14	1.13E-38	3.47E-24	7.06E-14	6.27E-11
0.324535	0.479224	0.518611	0.19515	0.231047	0.531679	0.553686	0.308866	-0.26592
0.000197	1.2E-08	4.22E-10	0.027901	0.008962	1.26E-10	1.46E-11	0.000411	0.002514
-0.08238	0.003785	0.033664	0.261585	0.491629	0.313252	0.393436	0.413181	-0.18206
0.357182	0.966312	0.707116	0.002972	4.37E-09	0.000336	4.74E-06	1.38E-06	0.040503
-0.00699	0.096654	0.142939	0.333956	0.057964	0.432833	0.075189	0.081462	-0.52569
0.93781	0.2797	0.108899	0.000125	0.517433	3.73E-07	0.400824	0.362578	2.21E-10
-0.4273	-0.02251	0.02888	0.228502	0.218981	0.39534	0.362797	0.186459	-0.50456
5.44E-07	0.801669	0.747214	0.009769	0.01338	4.22E-06	2.77E-05	0.035821	1.46E-09
-0.2227	0.042063	0.060211	0.399112	0.34033	0.460907	0.448064	0.269539	-0.44306
0.011852	0.638679	0.501302	3.35E-06	9.04E-05	4.95E-08	1.28E-07	0.002181	1.83E-07
-0.07893	-0.2011	-0.37042	-0.1356	-0.33498	-0.26936	-0.3036	-0.42202	0.139115
0.377754	0.023385	1.81E-05	0.128492	0.000118	0.002196	0.000521	7.74E-07	0.118796
-0.07265	-0.18832	-0.28862	-0.1941	-0.29211	-0.36635	-0.63435	-0.33018	0.286788
0.416976	0.033979	0.000999	0.028773	0.000861	2.28E-05	1.18E-15	0.00015	0.00108
-0.24516	-0.23916	-0.28011	-0.27238	-0.24851	-0.53095	-0.51916	-0.35033	0.25968
0.00547	0.00677	0.001425	0.001948	0.004844	1.35E-10	4.02E-10	5.4E-05	0.003196
-0.56143	-0.37147	-0.40452	-0.13937	-0.1624	-0.26988	-0.3561	-0.26979	-4.9E-05
6.59E-12	1.71E-05	2.4E-06	0.118107	0.068126	0.002152	3.98E-05	0.002159	0.99956
-0.47305	-0.40925	-0.45829	-0.4835	-0.25759	-0.55017	-0.15861	-0.33909	0.501056
1.95E-08	1.78E-06	6.03E-08	8.51E-09	0.003459	2.08E-11	0.074907	9.63E-05	1.98E-09
0.125253	-0.20482	-0.30629	-0.39386	-0.40134	-0.68786	-0.61923	-0.44913	0.736551
0.160586	0.020894	0.000462	4.62E-06	2.92E-06	4.14E-19	8.49E-15	1.18E-07	5.71E-23
0.320346	0.000934	0.011516	-0.0507	-0.04874	-0.08163	0.019523	0.088194	0.206715
0.000241	0.991684	0.897754	0.571369	0.58632	0.361575	0.827535	0.32414	0.019713
0.023825	-0.41218	-0.34458	-0.47004	-0.42618	-0.60184	-0.30007	-0.33755	0.498362
0.790337	1.47E-06	7.28E-05	2.47E-08	5.86E-07	7.24E-14	0.000609	0.000104	2.49E-09
0.528526	0.4078	0.385688	-0.20703	-0.31259	-0.05902	-0.33675	-0.05485	0.265412
1.7E-10	1.95E-06	7.53E-06	0.019522	0.000346	0.509801	0.000108	0.540232	0.002563

FAM162A	FAM172A	FAM173B	FAM185A	FAM189B	FAM200A	FAM206A	FAM210A	FAM213B
cyan	cyan	cyan	cyan	cyan	cyan	cyan	cyan	cyan
0.23808	0.028649	-0.07179	-0.0654	-0.03371	-0.20266	0.039489	-0.18228	-0.06763
0.007031	0.749174	0.422498	0.465052	0.706768	0.022307	0.659366	0.040254	0.449944
0.248767	0.026905	-0.01991	0.041135	0.021859	-0.1328	0.045051	-0.14684	0.002527
0.004799	0.76398	0.824203	0.646104	0.807288	0.136648	0.615008	0.099482	0.97751
-0.0802	0.207596	0.312406	0.301541	-0.08006	0.543605	0.091184	0.41608	-0.12945
0.370057	0.019184	0.000349	0.000571	0.37092	4E-11	0.307939	1.14E-06	0.146906
0.189873	0.584483	0.529216	0.511283	-0.48392	0.768938	0.518104	0.61245	-0.55488
0.032509	5.42E-13	1.59E-10	8.13E-10	8.23E-09	4.78E-26	4.42E-10	1.99E-14	1.29E-11
0.621791	0.134984	0.307665	0.241062	-0.20878	0.3363	0.348081	0.337499	-0.19445
6.12E-15	0.130259	0.000434	0.006331	0.018493	0.000111	6.07E-05	0.000104	0.028482
0.611161	0.658216	0.651345	0.598226	-0.6451	0.816271	0.759156	0.69379	-0.66241
2.33E-14	4.14E-17	1.12E-16	1.11E-13	2.7E-16	1.41E-31	4.57E-25	1.54E-19	2.23E-17
0.424738	0.304958	0.396328	0.379425	-0.33635	0.41937	0.526524	0.374879	-0.47564
6.46E-07	0.00049	3.98E-06	1.09E-05	0.000111	9.22E-07	2.04E-10	1.41E-05	1.59E-08
0.207219	0.429843	0.368614	-0.02743	-0.50058	0.105507	0.574783	-0.00503	-0.27282
0.019409	4.58E-07	2.01E-05	0.759485	2.06E-09	0.237784	1.59E-12	0.955278	0.001915
0.05079	0.06989	-0.14296	-0.0467	0.008038	0.119651	0.110638	0.146908	-0.41524
0.570661	0.434925	0.108847	0.602085	0.928538	0.180284	0.215605	0.09932	1.21E-06
0.161434	-0.00121	-0.13454	-0.27815	-0.19367	-0.17092	0.248249	-0.16092	-0.39494
0.069802	0.989243	0.131527	0.001544	0.029132	0.054702	0.004891	0.070706	4.33E-06
0.151775	0.140629	0.008894	-0.0868	-0.37629	0.037688	0.386712	-0.03077	-0.40173
0.088495	0.114798	0.920951	0.331849	1.3E-05	0.673997	7.09E-06	0.73125	2.85E-06
-0.29375	-0.44281	-0.47226	-0.18829	0.395923	-0.25348	-0.28643	-0.19595	0.122006
0.000802	1.86E-07	2.08E-08	0.034007	4.08E-06	0.004035	0.001096	0.027257	0.171798
-0.58229	-0.43052	-0.41659	-0.35733	0.51977	-0.34734	-0.5393	-0.33126	0.305998
6.94E-13	4.37E-07	1.11E-06	3.72E-05	3.8E-10	6.31E-05	6.08E-11	0.000142	0.000468
-0.30144	-0.56453	-0.49339	-0.4844	0.623666	-0.46822	-0.59348	-0.33863	0.469389
0.000573	4.76E-12	3.78E-09	7.92E-09	4.81E-15	2.84E-08	1.94E-13	9.86E-05	2.6E-08
-0.39678	-0.51422	-0.56691	-0.6446	0.446869	-0.62139	-0.42462	-0.55159	0.154817
3.87E-06	6.26E-10	3.7E-12	2.9E-16	1.39E-07	6.44E-15	6.51E-07	1.81E-11	0.082221
-0.29125	-0.39656	-0.20516	-0.36229	0.235112	-0.60249	-0.33072	-0.61691	0.378003
0.000893	3.92E-06	0.020676	2.85E-05	0.007796	6.69E-14	0.000146	1.14E-14	1.18E-05
-0.50326	-0.39983	-0.18987	-0.18724	0.385658	-0.29988	-0.5704	-0.31244	0.674156
1.64E-09	3.21E-06	0.032511	0.035035	7.55E-06	0.000614	2.55E-12	0.000349	3.72E-18
0.054299	0.330615	0.362979	0.434339	-0.23722	0.336662	0.084427	0.303446	0.130646
0.544303	0.000147	2.74E-05	3.36E-07	0.007247	0.000109	0.345314	0.000524	0.143186
-0.11178	-0.16459	-0.10414	0.010272	0.142421	-0.26028	-0.28346	-0.19768	0.507093
0.21087	0.06444	0.243963	0.908752	0.110201	0.003123	0.001241	0.0259	1.17E-09
-0.04539	-0.23284	0.054644	0.111539	0.360272	0.072948	-0.34223	0.15075	0.113866
0.612371	0.008432	0.541747	0.211864	3.18E-05	0.415048	8.21E-05	0.090693	0.202428

FAM220A	FAM221A	FAM227B	FAM228B	FAM35A	FAM91A1	FANCF	FANCL	FARP2
cyan	cyan	cyan	cyan	cyan	cyan	cyan	cyan	cyan
0.024474	-0.01352	0.079683	0.290044	0.017034	-0.09299	-0.16327	0.081518	-0.01365
0.784761	0.880106	0.373187	0.000941	0.849248	0.298436	0.066644	0.362246	0.878955
0.097075	0.055139	0.093846	0.354607	0.00195	-0.22747	-0.14242	0.154014	0.037444
0.277597	0.538083	0.293968	4.31E-05	0.982643	0.010114	0.110192	0.083841	0.67599
0.2934	-0.05182	0.188825	-0.25319	0.406749	0.252582	0.454455	0.095539	-0.03994
0.000815	0.562845	0.033497	0.004078	2.08E-06	0.004171	8.01E-08	0.28531	0.65571
0.457407	0.336537	0.502891	0.055293	0.611942	0.666179	0.641848	0.313884	-0.30728
6.44E-08	0.000109	1.69E-09	0.536952	2.12E-14	1.26E-17	4.25E-16	0.000326	0.000441
0.39534	0.337248	0.397439	0.468596	0.319884	-0.11256	0.261925	0.351121	-0.18453
4.22E-06	0.000106	3.72E-06	2.76E-08	0.000246	0.207681	0.002933	5.18E-05	0.037814
0.628034	0.595146	0.714679	0.412441	0.726368	0.590759	0.675991	0.532692	-0.46487
2.72E-15	1.6E-13	3.89E-21	1.45E-06	4.29E-22	2.65E-13	2.79E-18	1.15E-10	3.67E-08
0.333837	0.421139	0.455194	0.416842	0.399153	0.24647	0.272032	0.302772	-0.1857
0.000125	8.21E-07	7.58E-08	1.09E-06	3.34E-06	0.005217	0.001975	0.00054	0.036598
-0.08915	0.226853	0.424294	0.273892	0.080946	0.368859	0.254115	0.210263	-0.16412
0.318919	0.010325	6.65E-07	0.001834	0.365634	1.98E-05	0.00394	0.017658	0.065221
-0.06937	-0.09527	-0.09562	-0.10296	0.122976	0.443316	0.150824	-0.23625	-0.28387
0.438379	0.286697	0.28489	0.249351	0.16839	1.79E-07	0.090533	0.007496	0.00122
-0.30336	0.12331	0.080714	0.256044	0.081228	0.399372	-0.00472	-0.00815	-0.24624
0.000526	0.167225	0.367017	0.003666	0.36396	3.3E-06	0.957982	0.927497	0.005261
-0.03288	0.243789	0.149134	0.241903	0.178786	0.493667	0.159949	0.122976	-0.40515
0.713668	0.005745	0.094247	0.006145	0.044312	3.69E-09	0.072448	0.168389	2.3E-06
-0.18662	-0.32408	-0.51597	0.124972	-0.51124	-0.30663	-0.22374	-0.15855	0.011789
0.035657	0.000202	5.36E-10	0.161532	8.16E-10	0.000454	0.01145	0.075019	0.895344
-0.46875	-0.55196	-0.43728	-0.44147	-0.35424	-0.23176	-0.24933	-0.43563	0.367442
2.73E-08	1.74E-11	2.74E-07	2.04E-07	4.39E-05	0.008748	0.004701	3.08E-07	2.14E-05
-0.44014	-0.35305	-0.4281	-0.21968	-0.48923	-0.4217	-0.24527	-0.43737	0.284096
2.24E-07	4.68E-05	5.15E-07	0.013081	5.34E-09	7.91E-07	0.005448	2.72E-07	0.001208
-0.7251	-0.38631	-0.42892	-0.16859	-0.44656	-0.10588	-0.3487	-0.44997	0.207107
5.48E-22	7.26E-06	4.87E-07	0.058133	1.42E-07	0.236127	5.88E-05	1.11E-07	0.019476
-0.4089	-0.0401	-0.27638	0.14298	-0.42453	-0.50683	-0.44064	0.077057	0.408518
1.82E-06	0.654393	0.001659	0.108798	6.55E-07	1.2E-09	2.17E-07	0.389194	1.86E-06
-0.15075	-0.3622	-0.43082	-0.38379	-0.38695	-0.58995	-0.336	-0.23224	0.468985
0.0907	2.86E-05	4.28E-07	8.42E-06	6.99E-06	2.91E-13	0.000112	0.008605	2.68E-08
0.514252	0.218246	0.127206	-0.11632	0.170558	0.035919	0.130191	0.232536	-0.07178
6.24E-10	0.013702	0.15411	0.192788	0.055218	0.68848	0.144596	0.008518	0.422577
0.086232	-0.13173	-0.28156	-0.14511	-0.29904	-0.51416	-0.38739	-0.06699	0.209523
0.335061	0.139862	0.001342	0.103585	0.000637	6.3E-10	6.81E-06	0.454264	0.018071
0.040897	-0.44837	-0.0379	-0.22331	0.139617	-0.37947	-0.05964	0.032143	0.431942
0.648016	1.25E-07	0.672233	0.011615	0.117461	1.08E-05	0.505395	0.719783	3.97E-07

FASN	FASTKD1	FASTKD3	FBRSL1	FBXL14	FBXL18	FBXL19	FBXL5	FBXO22
cyan	cyan	cyan	cyan	cyan	cyan	cyan	cyan	cyan
-0.22142	-0.06241	-0.00766	-0.18029	-0.06259	-0.25813	-0.20733	-0.05553	-0.20651
0.012357	0.485796	0.931925	0.042532	0.484533	0.003389	0.019341	0.535216	0.019835
-0.2091	-0.00305	0.062909	-0.20742	-0.06307	-0.19532	-0.20755	0.018791	-0.15195
0.01831	0.972874	0.482285	0.019288	0.481183	0.02776	0.019212	0.833907	0.088126
-0.03733	0.398727	0.438444	-0.06717	-0.16875	-0.01278	-0.00421	0.087679	0.259165
0.676943	3.43E-06	2.53E-07	0.453044	0.057889	0.886642	0.96256	0.326983	0.003259
-0.49947	0.598961	0.635906	-0.51772	-0.42435	-0.38719	-0.50071	0.455963	0.618528
2.26E-09	1.02E-13	9.55E-16	4.57E-10	6.63E-07	6.89E-06	2.04E-09	7.16E-08	9.28E-15
-0.54041	0.265156	0.428969	-0.40242	-0.26007	-0.51751	-0.4735	0.165171	0.225534
5.46E-11	0.002589	4.86E-07	2.73E-06	0.003148	4.66E-10	1.89E-08	0.06349	0.010789
-0.88421	0.642025	0.770145	-0.81221	-0.54464	-0.74352	-0.85857	0.568489	0.732181
3.8E-43	4.14E-16	3.59E-26	4.83E-31	3.61E-11	1.36E-23	4.47E-38	3.13E-12	1.37E-22
-0.59954	0.426648	0.523748	-0.65556	-0.29302	-0.50512	-0.59028	0.364629	0.52508
9.51E-14	5.68E-07	2.64E-10	6.1E-17	0.000828	1.39E-09	2.81E-13	2.5E-05	2.34E-10
-0.40584	0.068306	0.056296	-0.29081	-0.415	-0.34427	-0.50964	0.230623	0.199265
2.2E-06	0.445433	0.529577	0.000911	1.23E-06	7.39E-05	9.39E-10	0.009092	0.024706
-0.11246	-0.08689	-0.03771	-0.10012	-0.21507	-0.10085	-0.09492	-0.09673	0.274362
0.208095	0.331373	0.673843	0.262756	0.015175	0.25927	0.288462	0.279338	0.0018
-0.41899	-0.12326	-0.16288	-0.18379	-0.28319	-0.48388	-0.43856	-0.006	0.101622
9.46E-07	0.167396	0.067308	0.038606	0.001255	8.25E-09	2.51E-07	0.946582	0.255606
-0.45503	0.021863	-0.02802	-0.31741	-0.28722	-0.45721	-0.48118	0.175929	0.223598
7.68E-08	0.807247	0.754529	0.000277	0.00106	6.53E-08	1.03E-08	0.047874	0.011505
0.43044	-0.44507	-0.3145	0.280054	0.162083	0.501329	0.479869	-0.24726	-0.25286
4.39E-07	1.58E-07	0.000317	0.001428	0.068671	1.93E-09	1.14E-08	0.00507	0.004129
0.701674	-0.30537	-0.4013	0.670133	0.274091	0.546547	0.679025	-0.48611	-0.42762
3.99E-20	0.000481	2.93E-06	6.93E-18	0.001819	2.99E-11	1.73E-18	6.89E-09	5.32E-07
0.643575	-0.49737	-0.52278	0.742159	0.402424	0.447823	0.614583	-0.52912	-0.4828
3.34E-16	2.71E-09	2.89E-10	1.81E-23	2.73E-06	1.3E-07	1.52E-14	1.6E-10	9.01E-09
0.455098	-0.53025	-0.6553	0.600381	0.18283	0.23295	0.385846	-0.51512	-0.43516
7.64E-08	1.44E-10	6.33E-17	8.61E-14	0.039649	0.008398	7.46E-06	5.78E-10	3.18E-07
0.289196	-0.19385	-0.3516	0.297059	0.191249	0.199048	0.197691	-0.11811	-0.49287
0.000975	0.028978	5.05E-05	0.000695	0.03125	0.024866	0.025889	0.185999	3.95E-09
0.706157	-0.16812	-0.24319	0.541803	0.435049	0.631892	0.699389	-0.22139	-0.51867
1.81E-20	0.058844	0.005869	4.77E-11	3.2E-07	1.64E-15	5.93E-20	0.01237	4.2E-10
-0.07493	0.319608	0.343204	-0.26914	0.097944	0.10989	-0.03689	0.407768	0.209688
0.402425	0.00025	7.81E-05	0.002216	0.273297	0.218743	0.680541	1.95E-06	0.017978
0.327431	-0.15385	-0.14878	0.147116	0.359759	0.402385	0.369773	-0.02151	-0.30038
0.000172	0.084177	0.095033	0.098839	3.26E-05	2.74E-06	1.88E-05	0.81033	0.000601
0.221916	0.239808	0.308237	0.225831	0.070439	0.080733	0.343029	-0.31072	-0.26153
0.012159	0.006617	0.000423	0.010683	0.431317	0.366903	7.88E-05	0.000377	0.002978

FBXO28	FBXO36	FBXO38	FBXO48	FBXO8	FCHO2	FDX1	FEM1C	FGF17
cyan	cyan	cyan	cyan	cyan	cyan	cyan	cyan	cyan
-0.16274	-0.05501	-0.1026	0.001875	0.089523	-0.07141	-0.06842	-0.18897	0.0873
0.067539	0.53904	0.251024	0.983306	0.31687	0.424988	0.444692	0.033361	0.329087
-0.09597	-0.01011	-0.10505	-0.0103	0.076258	-0.18838	-0.0873	-0.17924	0.109226
0.283145	0.910201	0.239816	0.908474	0.394142	0.033925	0.329083	0.043763	0.221554
0.286805	0.114597	0.289663	0.359966	-0.00488	0.186765	0.303987	0.499197	-0.32645
0.001079	0.199524	0.000956	3.23E-05	0.95657	0.035513	0.000512	2.32E-09	0.00018
0.489763	0.527183	0.636814	0.520709	0.337633	0.55469	0.495434	0.723282	-0.62662
5.1E-09	1.92E-10	8.45E-16	3.49E-10	0.000104	1.32E-11	3.18E-09	7.76E-22	3.28E-15
0.093957	0.333799	0.239581	0.322698	0.51551	-0.011	0.489136	-0.18953	-0.1952
0.293396	0.000125	0.00667	0.000215	5.58E-10	0.90233	5.38E-09	0.032827	0.027857
0.483292	0.733696	0.712173	0.612317	0.72438	0.527085	0.754148	0.469036	-0.69658
8.66E-09	1.01E-22	6.15E-21	2.02E-14	6.29E-22	1.94E-10	1.39E-24	2.67E-08	9.6E-20
0.21573	0.439623	0.486204	0.305277	0.343393	0.237885	0.275601	0.286117	-0.43854
0.014856	2.33E-07	6.83E-09	0.000483	7.74E-05	0.007079	0.001712	0.00111	2.51E-07
0.190355	0.427688	0.19939	0.0557	0.365413	0.225868	0.238675	-0.05957	-0.24157
0.032063	5.3E-07	0.024614	0.533956	2.4E-05	0.01067	0.006886	0.505846	0.006217
-0.13437	-0.09454	0.118171	0.063288	0.043493	0.425404	0.063286	0.395914	-0.46619
0.132021	0.290378	0.185769	0.47964	0.627307	6.18E-07	0.479659	4.08E-06	3.32E-08
-0.33742	0.018538	0.029036	0.055389	0.416121	0.382273	0.164402	-0.07143	-0.39545
0.000105	0.836114	0.745903	0.536241	1.14E-06	9.21E-06	0.06475	0.424853	4.2E-06
-0.11635	0.200293	0.116351	0.074618	0.402942	0.361953	0.207056	0.082541	-0.4509
0.192674	0.023958	0.192685	0.404422	2.64E-06	2.9E-05	0.019507	0.356234	1.04E-07
-0.3945	-0.32737	-0.27073	-0.44617	-0.45844	-0.31299	-0.5135	-0.1584	0.222754
4.45E-06	0.000172	0.00208	1.46E-07	5.96E-08	0.00034	6.68E-10	0.075295	0.011829
-0.31691	-0.55749	-0.40085	-0.25332	-0.62749	-0.19911	-0.4854	0.046168	0.267779
0.000283	9.91E-12	3.01E-06	0.004058	2.92E-15	0.024818	7.3E-09	0.606259	0.002337
-0.42905	-0.52433	-0.60121	-0.29248	-0.32629	-0.29936	-0.25643	-0.33369	0.350387
4.83E-07	2.5E-10	7.8E-14	0.000848	0.000181	0.000628	0.003613	0.000126	5.38E-05
-0.62359	-0.55785	-0.53492	-0.33767	-0.26514	-0.04739	-0.38109	-0.24534	0.121033
4.85E-15	9.55E-12	9.27E-11	0.000103	0.002591	0.596723	9.86E-06	0.005434	0.175266
-0.3202	-0.35133	-0.37589	-0.3292	-0.23694	-0.43119	-0.46745	-0.64176	0.480986
0.000243	5.12E-05	1.33E-05	0.000157	0.007317	4.18E-07	3.01E-08	4.3E-16	1.04E-08
-0.08231	-0.4144	-0.39478	-0.3434	-0.56049	-0.58648	-0.439	-0.28431	0.574986
0.357599	1.28E-06	4.37E-06	7.73E-05	7.26E-12	4.33E-13	2.43E-07	0.001198	1.55E-12
0.548662	0.290651	0.288564	0.112251	-0.00776	-0.0155	0.133479	0.152286	0.147629
2.42E-11	0.000917	0.001002	0.208944	0.930987	0.862667	0.134637	0.087416	0.097654
0.098862	-0.14783	-0.21116	-0.31237	-0.32832	-0.49309	-0.32268	-0.36658	0.592625
0.268805	0.097183	0.017168	0.00035	0.000164	3.88E-09	0.000216	2.25E-05	2.14E-13
-0.03112	-0.32083	0.141329	0.198597	-0.38011	-0.14641	-0.04436	0.077922	0.169061
0.728358	0.000235	0.112986	0.025202	1.04E-05	0.100479	0.620421	0.383874	0.057422

FGGY	FKRP	FMO3	FNTA	FOPNL	FOXK2	FOXP4	FPGT	FRMD4A
cyan	cyan	cyan	cyan	cyan	cyan	cyan	cyan	cyan
0.058088	-0.25841	-0.00172	0.230435	0.070579	-0.35994	0.033897	-0.14417	0.080648
0.516538	0.003354	0.984724	0.009151	0.430399	3.23E-05	0.705185	0.105854	0.367408
0.107983	-0.27451	-0.00639	0.268203	0.087619	-0.27279	0.025589	-0.15181	0.070245
0.226889	0.001789	0.943181	0.002299	0.327314	0.001917	0.775208	0.088423	0.432594
-0.00018	-0.09133	-0.07475	-0.15143	0.231043	0.347013	-0.17399	0.457764	-0.10945
0.998428	0.30718	0.403567	0.089222	0.008964	6.42E-05	0.050425	6.27E-08	0.220585
0.215008	-0.36888	0.206411	0.184588	0.591037	-0.1011	-0.44834	0.800534	-0.39514
0.015202	1.98E-05	0.019898	0.037754	2.57E-13	0.258082	1.25E-07	1.42E-29	4.28E-06
0.617568	-0.49447	0.280551	0.51319	0.473204	-0.43498	-0.41412	0.225615	-0.43621
1.05E-14	3.45E-09	0.001399	6.86E-10	1.93E-08	3.22E-07	1.3E-06	0.01076	2.95E-07
0.611664	-0.69132	0.458667	0.573112	0.864213	-0.55708	-0.66564	0.840013	-0.61556
2.19E-14	2.33E-19	5.86E-08	1.9E-12	4.21E-39	1.03E-11	1.37E-17	5.41E-35	1.35E-14
0.418962	-0.47929	0.391075	0.354208	0.542872	-0.3493	-0.43931	0.38104	-0.40387
9.48E-07	1.19E-08	5.47E-06	4.4E-05	4.3E-11	5.7E-05	2.38E-07	9.89E-06	2.5E-06
0.461389	-0.21207	0.343921	0.268548	0.280601	-0.47237	-0.32226	0.373058	-0.38435
4.78E-08	0.016686	7.53E-05	0.002268	0.001396	2.06E-08	0.00022	1.56E-05	8.15E-06
-0.24698	0.078653	0.283364	-0.09776	0.111843	0.058678	-0.05134	0.138746	-0.04157
0.005121	0.379414	0.001246	0.274208	0.210615	0.512278	0.566512	0.119788	0.642657
0.071258	-0.14887	0.380655	0.353652	0.131192	-0.62717	-0.15482	0.069388	-0.08767
0.425968	0.094841	1.01E-05	4.53E-05	0.141508	3.05E-15	0.082209	0.438241	0.327056
0.104417	-0.22917	0.244821	0.348007	0.251262	-0.62438	-0.20418	0.281132	-0.1779
0.242694	0.009551	0.005536	6.09E-05	0.00438	4.38E-15	0.021304	0.001366	0.045387
-0.38904	0.34256	-0.35476	-0.04347	-0.40622	0.47533	0.372348	-0.46133	0.460482
6.18E-06	8.07E-05	4.27E-05	0.627478	2.15E-06	1.63E-08	1.63E-05	4.8E-08	5.12E-08
-0.5646	0.57514	-0.40778	-0.55785	-0.58098	0.697283	0.462317	-0.37461	0.437992
4.73E-12	1.53E-12	1.95E-06	9.54E-12	8.02E-13	8.51E-20	4.46E-08	1.43E-05	2.61E-07
-0.25691	0.575368	-0.28243	-0.30951	-0.53829	0.392686	0.450286	-0.48405	0.133649
0.003549	1.49E-12	0.001295	0.000399	6.71E-11	4.96E-06	1.09E-07	8.14E-09	0.134137
-0.42364	0.492321	-0.11033	-0.21116	-0.54212	0.147077	0.40314	-0.45858	0.324317
6.95E-07	4.13E-09	0.216912	0.017168	4.62E-11	0.098928	2.61E-06	5.9E-08	0.000199
-0.17585	0.102582	-0.22744	0.17247	-0.45492	-0.06742	0.231098	-0.4976	0.351875
0.047978	0.251115	0.010124	0.052505	7.74E-08	0.451358	0.008947	2.65E-09	4.98E-05
-0.36124	0.381927	-0.55078	-0.40528	-0.5716	0.6365	0.348178	-0.45901	0.399286
3.01E-05	9.39E-06	1.96E-11	2.28E-06	2.24E-12	8.81E-16	6.04E-05	5.71E-08	3.32E-06
0.126122	-0.19628	-0.06845	-0.06007	0.220692	0.053182	-0.11842	0.233486	-0.10367
0.157679	0.026995	0.444502	0.502339	0.012656	0.552628	0.184823	0.008245	0.246094
-0.09492	0.1304	-0.25156	-0.24737	-0.27571	0.222205	0.218095	-0.42915	0.234401
0.288446	0.143945	0.004331	0.00505	0.001704	0.012045	0.013769	4.8E-07	0.00799
-0.15143	-0.15988	-0.15759	-0.24418	-0.09313	0.502991	0.040132	-0.14178	0.370081
0.089233	0.07257	0.076819	0.005664	0.297685	1.68E-09	0.654168	0.11182	1.85E-05

FURIN	FXR2	FXYD1	GAB2	GALK2	GALNT1	GAS2	GATAD2B	GCFC2
cyan	cyan	cyan	cyan	cyan	cyan	cyan	cyan	cyan
-0.1843	-0.07159	0.120388	0.107662	0.233871	-0.21888	0.164842	-0.2814	-0.13605
0.038055	0.423788	0.177593	0.228283	0.008137	0.013425	0.064026	0.001351	0.127232
-0.23085	0.028416	0.247498	0.090673	0.260525	-0.31479	0.181811	-0.2863	-0.0705
0.009023	0.751146	0.005026	0.310669	0.003094	0.000313	0.040783	0.001102	0.430951
-0.16533	-0.12538	-0.4835	-0.05825	-0.13279	0.54382	0.160005	0.276984	0.452459
0.06323	0.160173	8.51E-09	0.515342	0.13669	3.91E-11	0.072347	0.001619	9.27E-08
-0.37343	-0.54491	-0.65758	-0.27777	0.150796	0.696484	0.273121	-0.06491	0.702319
1.53E-05	3.52E-11	4.55E-17	0.001568	0.090593	9.76E-20	0.001891	0.468414	3.57E-20
-0.43279	-0.13159	-0.10757	-0.3608	0.414053	0.004434	0.44742	-0.62809	0.280878
3.74E-07	0.140288	0.228696	3.09E-05	1.31E-06	0.960538	1.34E-07	2.7E-15	0.001381
-0.57606	-0.65868	-0.60759	-0.53187	0.499153	0.588295	0.51162	-0.62039	0.747183
1.38E-12	3.87E-17	3.61E-14	1.24E-10	2.33E-09	3.52E-13	7.89E-10	7.32E-15	6.28E-24
-0.32902	-0.32481	-0.32702	-0.27616	0.293969	0.237797	0.311134	-0.46525	0.412235
0.000159	0.000195	0.000175	0.001674	0.000795	0.007101	0.00037	3.57E-08	1.47E-06
-0.30001	-0.51498	-0.07921	-0.28819	0.069727	0.16011	0.027036	-0.27538	0.086299
0.000611	5.85E-10	0.376036	0.001018	0.436002	0.072157	0.762859	0.001727	0.334683
0.468609	-0.39507	-0.38947	-0.06051	0.019194	0.570137	-0.1173	-0.15565	0.087644
2.76E-08	4.29E-06	6.02E-06	0.499191	0.830406	2.62E-12	0.189066	0.080563	0.327175
0.182087	-0.5596	-0.2231	0.058806	0.299074	0.319997	-0.08982	-0.57094	-0.07542
0.040473	7.97E-12	0.011695	0.511354	0.000636	0.000245	0.315259	2.41E-12	0.399393
-0.06096	-0.63981	-0.27883	-0.04803	0.274	0.302677	-0.0185	-0.41951	0.069788
0.495977	5.61E-16	0.001502	0.591823	0.001826	0.000543	0.836426	9.14E-07	0.4356
0.337887	0.284412	0.457492	0.402458	-0.00722	-0.32419	-0.2067	0.385913	-0.28891
0.000102	0.001193	6.4E-08	2.72E-06	0.935775	0.000201	0.019721	7.43E-06	0.000987
0.458762	0.350029	0.132362	0.556201	-0.48416	-0.0313	-0.41052	0.747971	-0.27598
5.82E-08	5.48E-05	0.137961	1.13E-11	8.07E-09	0.726844	1.64E-06	5.31E-24	0.001686
0.476424	0.44993	0.326826	0.33989	-0.29049	-0.30823	-0.338	0.30823	-0.38192
1.5E-08	1.11E-07	0.000177	9.25E-05	0.000923	0.000423	0.000102	0.000423	9.39E-06
0.609845	0.054676	0.181481	0.540315	-0.20927	-0.04076	-0.46923	0.204068	-0.43591
2.74E-14	0.541512	0.041156	5.51E-11	0.018215	0.649079	2.63E-08	0.021377	3.02E-07
-0.07757	0.378037	0.533969	0.424848	-0.08465	-0.6211	-0.0637	0.125309	-0.42803
0.386045	1.18E-05	1.01E-10	6.41E-07	0.344037	6.68E-15	0.47681	0.160396	5.18E-07
0.031247	0.712028	0.387297	0.358167	-0.49555	-0.47327	-0.14073	0.710105	-0.34508
0.727281	6.32E-21	6.85E-06	3.56E-05	3.15E-09	1.92E-08	0.114539	8.95E-21	7.09E-05
-0.36315	0.161178	0.005258	-0.39289	-0.03805	-0.13686	0.239036	0.08448	0.185421
2.71E-05	0.070253	0.95322	4.9E-06	0.671076	0.124941	0.006799	0.345009	0.036883
-0.03711	0.56781	0.476459	-0.08668	-0.21193	-0.6203	-0.03737	0.20216	-0.37481
0.678712	3.37E-12	1.49E-08	0.332538	0.016759	7.4E-15	0.676625	0.02265	1.42E-05
-0.10868	0.368926	-0.11446	0.251283	-0.10083	0.024983	0.325071	0.431522	0.119059
0.223897	1.97E-05	0.200071	0.004376	0.259352	0.780398	0.000192	4.08E-07	0.182464

GDAP2	GDF9	GDI1	GEMIN2	GEMIN6	GGCT	GGPS1	GGT7	GIN1
cyan	cyan	cyan	cyan	cyan	cyan	cyan	cyan	cyan
-0.03547	0.011796	-0.01398	-0.02555	-0.0047	-0.01804	0.06688	-0.10187	0.179872
0.69221	0.895277	0.876073	0.775517	0.958168	0.840433	0.455013	0.254439	0.043017
-0.04465	0.028193	0.081118	0.061561	0.00433	0.086625	0.086496	-0.02727	0.211608
0.618126	0.753034	0.364613	0.491738	0.961462	0.332851	0.333574	0.760852	0.016929
0.455123	0.42192	-0.27415	0.447781	0.374973	0.357756	0.1906	-0.0621	0.088193
7.62E-08	7.79E-07	0.001815	1.3E-07	1.4E-05	3.64E-05	0.031839	0.487916	0.324143
0.73913	0.615443	-0.62445	0.488479	0.54042	0.407396	0.403647	-0.37382	0.461371
3.38E-23	1.37E-14	4.34E-15	5.67E-09	5.46E-11	2E-06	2.53E-06	1.5E-05	4.78E-08
0.251795	0.23324	-0.31175	0.626862	0.637861	0.297261	0.530676	-0.16299	0.518061
0.004294	0.008315	0.00036	3.17E-15	7.33E-16	0.000689	1.39E-10	0.067113	4.44E-10
0.758594	0.612203	-0.77524	0.734349	0.838945	0.517504	0.721097	-0.54247	0.799003
5.18E-25	2.05E-14	1.05E-26	8.9E-23	7.92E-35	4.66E-10	1.18E-21	4.47E-11	2.17E-29
0.444991	0.381236	-0.40592	0.337712	0.516261	0.261967	0.369729	-0.20693	0.478309
1.59E-07	9.78E-06	2.19E-06	0.000103	5.22E-10	0.002928	1.89E-05	0.01958	1.29E-08
0.158048	0.190616	-0.22383	-0.10012	0.285638	-0.09775	0.429194	-0.44747	0.315642
0.075952	0.031824	0.011418	0.262735	0.001133	0.274236	4.78E-07	1.33E-07	0.000301
0.333558	-0.03862	-0.51257	-0.07962	0.168391	0.195212	0.015103	-0.27876	-0.18163
0.000127	0.666435	7.26E-10	0.373536	0.058432	0.027851	0.866168	0.001506	0.040988
0.18736	-0.09091	-0.49364	-0.18394	0.263275	-0.02076	0.129681	-0.52051	0.072182
0.034921	0.309399	3.7E-09	0.038439	0.002785	0.816796	0.146188	3.55E-10	0.419978
0.268177	0.150601	-0.46532	-0.04645	0.255631	0.110903	0.236388	-0.53824	0.212059
0.002301	0.091016	3.55E-08	0.604042	0.003723	0.214499	0.007459	6.74E-11	0.016691
-0.19771	-0.14259	0.31144	-0.34214	-0.50688	0.059879	-0.49067	0.358173	-0.5567
0.025878	0.109767	0.000365	8.25E-05	1.2E-09	0.503671	4.73E-09	3.56E-05	1.07E-11
-0.19607	-0.16196	0.324002	-0.41515	-0.42818	-0.22204	-0.50145	0.34677	-0.65886
0.027161	0.068884	0.000202	1.22E-06	5.12E-07	0.01211	1.91E-09	6.5E-05	3.77E-17
-0.40341	-0.37813	0.320827	-0.26883	-0.2457	-0.22794	-0.35206	0.317637	-0.55125
2.57E-06	1.17E-05	0.000236	0.002242	0.005364	0.009954	4.93E-05	0.000274	1.87E-11
-0.27653	-0.40608	0.097666	-0.59102	-0.30364	-0.30528	-0.381	-0.00785	-0.59051
0.001649	2.17E-06	0.274668	2.58E-13	0.00052	0.000483	9.91E-06	0.930193	2.73E-13
-0.60336	-0.39329	0.598182	-0.38783	-0.44906	-0.35562	-0.24737	0.15822	-0.12571
6.03E-14	4.78E-06	1.12E-13	6.64E-06	1.19E-07	4.08E-05	0.00505	0.07563	0.15905
-0.54667	-0.25474	0.783134	-0.19235	-0.54268	-0.24168	-0.41928	0.555628	-0.46193
2.95E-11	0.003849	1.47E-27	0.03027	4.38E-11	0.006194	9.28E-07	1.2E-11	4.59E-08
-0.03794	0.121847	0.200783	0.273915	-0.10194	0.062114	0.050401	0.183118	0.294638
0.671968	0.172362	0.023609	0.001832	0.254093	0.487843	0.573617	0.039333	0.000772
-0.55666	-0.30374	0.666037	-0.14459	-0.4874	-0.2498	-0.27871	0.499089	-0.14768
1.08E-11	0.000518	1.29E-17	0.104828	6.2E-09	0.004621	0.001509	2.34E-09	0.097545
0.219394	0.131989	0.08469	0.299569	0.053528	0.026749	-0.10811	0.418938	-0.09421
0.013202	0.139084	0.343804	0.000623	0.550043	0.765309	0.226344	9.49E-07	0.292079

GINM1	GIT1	GK	GLMN	GLO1	GLT8D1	GLT8D2	GMPS	GNAO1
cyan	cyan	cyan	cyan	cyan	cyan	cyan	cyan	cyan
0.12723	0.05391	-0.05522	-0.08087	0.10825	-0.01489	0.141058	-0.20104	0.052068
0.154032	0.547197	0.53748	0.36608	0.225736	0.86804	0.113684	0.023425	0.560994
0.076585	0.112825	-0.11279	0.016159	0.216026	-0.02422	0.114128	-0.22095	0.117879
0.392111	0.206611	0.206745	0.856904	0.014716	0.786945	0.201384	0.012549	0.186868
-0.0355	-0.38562	0.062058	0.372323	0.118441	0.261388	0.013137	0.610753	-0.35834
0.691898	7.56E-06	0.488238	1.63E-05	0.18476	0.002994	0.883455	2.45E-14	3.53E-05
0.346938	-0.77035	0.470868	0.529713	0.409586	0.641792	0.231321	0.683693	-0.62193
6.44E-05	3.42E-26	2.32E-08	1.52E-10	1.74E-06	4.28E-16	0.008879	8.17E-19	6.01E-15
0.323787	-0.20828	0.138093	0.399394	0.547318	0.312319	0.230802	0.210877	-0.3161
0.000205	0.018783	0.121557	3.29E-06	2.77E-11	0.000351	0.009037	0.017322	0.000294
0.649221	-0.83097	0.618668	0.682865	0.773152	0.784346	0.444164	0.641728	-0.73642
1.51E-16	1.25E-33	9.11E-15	9.34E-19	1.75E-26	1.08E-27	1.69E-07	4.32E-16	5.86E-23
0.295774	-0.43306	0.330769	0.33128	0.476092	0.561543	0.258156	0.375278	-0.41192
0.000735	3.67E-07	0.000146	0.000142	1.54E-08	6.51E-12	0.003386	1.38E-05	1.5E-06
0.225916	-0.29487	0.526171	0.068712	0.272283	0.473404	-0.09214	0.139858	-0.13053
0.010653	0.000764	2.11E-10	0.442727	0.001956	1.9E-08	0.30289	0.116822	0.143558
0.271067	-0.49408	0.249638	-0.19002	-0.18428	0.425343	0.37723	0.416663	-0.61949
0.002053	3.57E-09	0.004649	0.032374	0.038085	6.2E-07	1.23E-05	1.1E-06	8.21E-15
0.388389	-0.43118	0.386908	-0.37615	-0.08836	0.338063	0.133278	0.000266	-0.40931
6.42E-06	4.18E-07	7.01E-06	1.31E-05	0.323218	0.000101	0.135231	0.997633	1.77E-06
0.383349	-0.55238	0.415528	-0.12557	0.07079	0.40262	0.171857	0.106577	-0.38889
8.64E-06	1.67E-11	1.19E-06	0.15953	0.429019	2.7E-06	0.053362	0.23303	6.23E-06
-0.28287	0.221914	-0.48348	-0.40977	-0.39846	-0.21932	-0.05964	-0.40367	0.162081
0.001272	0.01216	8.53E-09	1.72E-06	3.49E-06	0.013234	0.505392	2.53E-06	0.068674
-0.5547	0.294896	-0.44611	-0.49835	-0.72825	-0.33851	-0.41323	-0.07522	0.248143
1.32E-11	0.000764	1.47E-07	2.49E-09	2.97E-22	9.91E-05	1.38E-06	0.400617	0.004909
-0.41318	0.467002	-0.44194	-0.51863	-0.62955	-0.4838	-0.42299	-0.26967	0.274401
1.38E-06	3.12E-08	1.98E-07	4.21E-10	2.23E-15	8.31E-09	7.26E-07	0.002169	0.001797
-0.23279	0.176566	-0.23089	-0.82034	-0.75863	-0.23127	-0.33201	-0.27286	0.110594
0.008444	0.047061	0.009011	3.98E-32	5.14E-25	0.008894	0.000137	0.001911	0.215788
-0.25708	0.613988	-0.42628	-0.40733	-0.22502	-0.52377	-0.40381	-0.71963	0.739546
0.003526	1.64E-14	5.82E-07	2.01E-06	0.010977	2.64E-10	2.5E-06	1.55E-21	3.1E-23
-0.62672	0.702215	-0.65288	-0.12347	-0.32814	-0.69801	-0.45875	-0.39365	0.739334
3.23E-15	3.63E-20	8.98E-17	0.166669	0.000166	7.51E-20	5.82E-08	4.68E-06	3.24E-23
-0.00481	0.143396	0.080931	0.620947	0.465645	-0.15451	0.160287	-0.01723	0.209885
0.957211	0.10776	0.365723	6.82E-15	3.46E-08	0.082845	0.071838	0.847569	0.017868
-0.32272	0.686928	-0.32162	0.122619	0.061233	-0.55553	-0.06985	-0.45597	0.634306
0.000215	4.82E-19	0.000227	0.169636	0.494051	1.21E-11	0.435171	7.16E-08	1.18E-15
-0.266	0.162207	-0.33607	0.12457	-0.12163	-0.13296	-0.02683	0.28187	0.014706
0.002506	0.068457	0.000112	0.162897	0.17313	0.136166	0.76463	0.001325	0.869653

GNAZ	GNG11	GNL3	GNPAT	GNPDA1	GNPDA2	GOLGA5	GOLGA7	GOLPH3L
cyan	cyan	cyan	cyan	cyan	cyan	cyan	cyan	cyan
0.090842	0.002451	-0.15961	-0.22652	0.175726	-0.02281	0.178106	0.109249	0.138439
0.309767	0.978183	0.073055	0.010442	0.048137	0.799085	0.04514	0.221456	0.120619
0.187219	0.044688	-0.17976	-0.18805	0.143188	0.022198	0.17249	0.139343	0.103378
0.03506	0.617866	0.043144	0.034239	0.108278	0.804352	0.052477	0.11819	0.247437
-0.32476	0.039413	0.564212	0.548443	0.124916	0.25179	0.14399	-0.011	-0.08117
0.000195	0.659977	4.92E-12	2.48E-11	0.161722	0.004295	0.106296	0.902328	0.364308
-0.64506	0.31225	0.732036	0.561515	0.395769	0.55081	0.523849	0.201887	0.39254
2.72E-16	0.000352	1.41E-22	6.53E-12	4.12E-06	1.95E-11	2.62E-10	0.022837	5.01E-06
-0.08738	0.565545	0.343203	0.398628	0.305378	0.399966	0.320194	0.393344	0.213055
0.328623	4.28E-12	7.81E-05	3.45E-06	0.000481	3.18E-06	0.000243	4.77E-06	0.016175
-0.64956	0.671926	0.760264	0.606001	0.56527	0.772923	0.731163	0.462097	0.595713
1.44E-16	5.25E-18	3.56E-25	4.39E-14	4.4E-12	1.85E-26	1.68E-22	4.53E-08	1.49E-13
-0.37633	0.51647	0.503682	0.310525	0.431665	0.376548	0.476686	0.246809	0.363944
1.3E-05	5.12E-10	1.58E-09	0.000381	4.04E-07	1.28E-05	1.47E-08	0.005153	2.6E-05
-0.16815	0.023914	0.146077	0.302144	0.047565	0.239672	0.075806	0.047163	0.564081
0.058797	0.78957	0.10127	0.000556	0.595398	0.006649	0.39696	0.598517	4.99E-12
-0.63054	0.279955	0.436296	0.050675	0.120995	-0.13971	0.345775	-0.19308	0.099853
1.96E-15	0.001434	2.94E-07	0.571531	0.175402	0.117208	6.84E-05	0.029635	0.264014
-0.47027	0.225133	0.091386	-0.1149	0.243951	-0.16389	0.346172	0.083375	0.174474
2.42E-08	0.010934	0.306865	0.198319	0.005712	0.065603	6.7E-05	0.351375	0.049779
-0.51909	0.189131	0.195203	-0.00857	0.411236	0.079372	0.386514	0.11678	0.311619
4.04E-10	0.033206	0.027858	0.923787	1.56E-06	0.375062	7.18E-06	0.191041	0.000362
0.188536	-0.20227	-0.25863	-0.25112	0.023479	-0.53347	-0.20094	-0.10834	-0.24815
0.033773	0.022575	0.003325	0.004403	0.793307	1.06E-10	0.023497	0.225329	0.004908
0.176177	-0.57242	-0.18064	-0.12426	-0.33023	-0.61584	-0.44678	-0.4425	-0.52964
0.047556	2.05E-12	0.042124	0.163954	0.00015	1.3E-14	1.4E-07	1.9E-07	1.53E-10
0.344204	-0.32955	-0.33724	-0.25484	-0.32648	-0.54989	-0.51794	-0.19839	-0.56718
7.42E-05	0.000155	0.000106	0.003836	0.00018	2.14E-11	4.48E-10	0.02536	3.6E-12
0.034311	-0.35306	-0.32474	-0.34709	-0.24155	-0.7406	-0.30672	-0.28143	-0.44204
0.701753	4.67E-05	0.000195	6.39E-05	0.006221	2.5E-23	0.000453	0.001349	1.96E-07
0.711684	-0.31784	-0.71191	-0.37068	-0.27291	-0.4028	-0.35826	0.182262	-0.35838
6.73E-21	0.000271	6.45E-21	1.79E-05	0.001907	2.67E-06	3.54E-05	0.040278	3.52E-05
0.711417	-0.49218	-0.44852	-0.12686	-0.40221	-0.28965	-0.61791	-0.12959	-0.55355
7.06E-21	4.18E-09	1.23E-07	0.155252	2.77E-06	0.000956	1E-14	0.146479	1.48E-11
0.193321	0.062233	-0.04075	-0.00714	-0.0051	0.531801	-0.00288	0.136408	0.229369
0.029432	0.487013	0.649221	0.936458	0.95458	1.25E-10	0.974408	0.126216	0.009487
0.634331	-0.21374	-0.49922	-0.3254	-0.30773	-0.0037	-0.3923	-0.10756	-0.0962
1.18E-15	0.015828	2.31E-09	0.000189	0.000432	0.967089	5.08E-06	0.228729	0.281954
0.127326	-0.07764	0.275231	0.351493	-0.15636	-0.13423	-0.10576	-0.10879	-0.36314
0.153718	0.385612	0.001738	5.08E-05	0.079176	0.132428	0.236634	0.223409	2.72E-05

GOLT1B	GPALPP1	GPI	GPN1	GPR89A	GPR89B	GPSM1	GRAMD4	GREB1
cyan	cyan	cyan	cyan	cyan	cyan	cyan	cyan	cyan
0.090691	-0.13981	0.125246	0.107989	0.015776	-0.07663	-0.33976	-0.13293	-0.09872
0.310574	0.116942	0.160611	0.226864	0.860267	0.39186	9.31E-05	0.136273	0.26948
0.141493	-0.14178	0.171207	0.129068	0.012307	0.004378	-0.29198	-0.20684	-0.05409
0.112564	0.111827	0.054284	0.148122	0.890775	0.961042	0.000866	0.019637	0.545893
0.167758	0.310006	-0.27919	0.264973	0.331834	0.379815	0.21478	-0.15217	-0.01198
0.059399	0.00039	0.00148	0.002608	0.000138	1.06E-05	0.015313	0.087652	0.89363
0.477641	0.500351	-0.46875	0.460816	0.504562	0.491411	-0.38206	-0.41723	-0.23407
1.36E-08	2.1E-09	2.73E-08	4.99E-08	1.46E-09	4.45E-09	9.32E-06	1.06E-06	0.00808
0.346712	0.393778	-0.33314	0.570903	0.514754	0.539054	-0.27985	-0.52737	-0.51753
6.52E-05	4.64E-06	0.00013	2.42E-12	5.97E-10	6.23E-11	0.00144	1.89E-10	4.65E-10
0.710525	0.635161	-0.62451	0.745808	0.721124	0.694099	-0.71922	-0.72095	-0.60019
8.3E-21	1.06E-15	4.31E-15	8.41E-24	1.17E-21	1.46E-19	1.67E-21	1.21E-21	8.81E-14
0.394929	0.375864	-0.27154	0.606909	0.565383	0.598945	-0.47795	-0.59384	-0.31712
4.33E-06	1.33E-05	0.002014	3.93E-14	4.35E-12	1.02E-13	1.33E-08	1.86E-13	0.00028
0.000681	0.114301	-0.31607	0.072533	0.138319	0.191041	-0.51968	-0.12083	-0.44352
0.993936	0.200699	0.000295	0.417717	0.120943	0.031438	3.83E-10	0.176001	1.77E-07
0.059157	-0.1614	-0.04267	0.228318	0.145311	0.080946	-0.12568	-0.01583	-0.25623
0.508835	0.069862	0.633801	0.00983	0.103092	0.365633	0.159164	0.85975	0.003641
0.004707	-0.23837	-0.30961	0.179199	0.195701	0.094154	-0.50144	-0.03988	-0.55793
0.958113	0.00696	0.000397	0.043816	0.027454	0.29238	1.91E-09	0.656221	9.46E-12
0.103954	-0.11454	-0.2766	0.207772	0.213396	0.092106	-0.57509	-0.06389	-0.46839
0.2448	0.199752	0.001644	0.01908	0.016001	0.303053	1.54E-12	0.475476	2.8E-08
-0.35562	-0.53045	0.452731	-0.13985	-0.38327	-0.37857	0.422112	0.273092	0.325608
4.08E-05	1.41E-10	9.09E-08	0.116851	8.68E-06	1.14E-05	7.7E-07	0.001894	0.000187
-0.61049	-0.43766	0.304742	-0.44272	-0.31197	-0.28192	0.706919	0.573516	0.449155
2.53E-14	2.67E-07	0.000495	1.87E-07	0.000356	0.001322	1.58E-20	1.82E-12	1.18E-07
-0.54295	-0.40686	0.250554	-0.38206	-0.24699	-0.2502	0.681219	0.596622	0.150704
4.26E-11	2.07E-06	0.004495	9.32E-06	0.005119	0.004554	1.22E-18	1.34E-13	0.090793
-0.62831	-0.66304	0.135784	-0.40912	-0.25882	-0.30491	0.343666	0.602394	0.011182
2.63E-15	2.03E-17	0.127975	1.79E-06	0.003302	0.000491	7.63E-05	6.77E-14	0.900702
-0.34872	-0.32961	0.184973	-0.5062	-0.37985	-0.34523	0.132744	0.189494	0.274859
5.87E-05	0.000154	0.037349	1.27E-09	1.06E-05	7.04E-05	0.136816	0.032863	0.001764
-0.40022	-0.12124	0.371975	-0.44687	-0.47154	-0.36927	0.778158	0.360786	0.698309
3.13E-06	0.174529	1.66E-05	1.39E-07	2.2E-08	1.93E-05	5.13E-27	3.09E-05	7.14E-20
0.473333	0.474555	0.077339	0.001451	-0.10128	-0.06256	-0.09707	-0.25374	0.286272
1.91E-08	1.74E-08	0.387456	0.987084	0.257204	0.48474	0.277625	0.003996	0.001103
-0.03867	0.030576	0.464646	-0.29852	-0.45976	-0.39309	0.263615	0.078522	0.467273
0.666004	0.732918	3.73E-08	0.000652	5.4E-08	4.84E-06	0.002748	0.380213	3.06E-08
-0.06925	0.193691	-0.01525	0.135996	0.147673	0.210191	0.498682	-0.18175	0.2182
0.439154	0.029116	0.864894	0.127374	0.097552	0.017698	2.42E-09	0.040854	0.013723

GRID1	GRIK5	GRIN2D	GRINA	GRIPAP1	GRPEL2	GSK3A	GSR	GTF2B
cyan	cyan	cyan	cyan	cyan	cyan	cyan	cyan	cyan
0.118525	-0.33714	-0.28185	0.195613	0.155772	-0.18111	-0.10909	0.026135	0.270059
0.184448	0.000106	0.001326	0.027525	0.080326	0.041577	0.222138	0.770543	0.002136
0.186808	-0.30028	-0.18816	0.200633	0.156019	-0.13858	0.001803	-0.0459	0.3038
0.035469	0.000603	0.034136	0.023715	0.079842	0.120234	0.983947	0.608346	0.000516
-0.39226	0.207102	0.324991	-0.63193	-0.42532	0.397729	-0.16394	-0.00488	0.016899
5.09E-06	0.019479	0.000193	1.63E-15	6.21E-07	3.65E-06	0.065524	0.956582	0.85043
-0.59473	-0.24104	-0.13886	-0.82635	-0.6563	0.668408	-0.52941	0.257372	0.242737
1.67E-13	0.006336	0.119476	5.79E-33	5.48E-17	9.02E-18	1.56E-10	0.003488	0.005965
-0.36261	-0.39366	-0.15842	-0.15084	-0.38143	0.14924	-0.23627	0.27285	0.642592
2.8E-05	4.68E-06	0.075249	0.090509	9.67E-06	0.09401	0.007489	0.001912	3.83E-16
-0.7159	-0.63609	-0.42646	-0.721	-0.7775	0.660403	-0.70041	0.483058	0.675806
3.11E-21	9.32E-16	5.76E-07	1.2E-21	6.04E-27	3E-17	4.97E-20	8.82E-09	2.87E-18
-0.34747	-0.3463	-0.19777	-0.36921	-0.49779	0.404774	-0.26413	0.193478	0.52873
6.27E-05	6.66E-05	0.025825	1.94E-05	2.61E-09	2.36E-06	0.002694	0.029297	1.66E-10
-0.19927	-0.44582	-0.63072	-0.12354	-0.13927	0.094893	-0.45752	0.358573	0.229916
0.024699	1.5E-07	1.91E-15	0.166442	0.118385	0.288594	6.38E-08	3.48E-05	0.009313
-0.38992	-0.06347	0.035903	-0.21363	-0.29963	0.328062	-0.29167	0.0122	-0.10836
5.86E-06	0.47835	0.688616	0.015884	0.000621	0.000166	0.000878	0.891718	0.225272
-0.20443	-0.5238	-0.5216	0.16557	-0.15969	-0.12025	-0.56752	0.195575	0.141495
0.021141	2.63E-10	3.22E-10	0.062845	0.072924	0.17808	3.47E-12	0.027556	0.112558
-0.32928	-0.47441	-0.51632	-0.04502	-0.27433	0.068553	-0.61112	0.224325	0.132911
0.000157	1.76E-08	5.19E-10	0.615266	0.001802	0.443785	2.34E-14	0.011231	0.136319
0.228888	0.392802	0.434423	0.322286	0.189011	-0.20746	0.495145	-0.35741	-0.47622
0.009642	4.93E-06	3.34E-07	0.00022	0.03332	0.019267	3.26E-09	3.71E-05	1.52E-08
0.298252	0.650636	0.534614	0.174539	0.341934	-0.29679	0.372292	-0.46973	-0.64425
0.00066	1.24E-16	9.54E-11	0.049693	8.33E-05	0.000703	1.63E-05	2.53E-08	3.04E-16
0.252813	0.524292	0.414543	0.464658	0.297363	-0.43255	0.348232	-0.26875	-0.49239
0.004136	2.51E-10	1.26E-06	3.73E-08	0.000686	3.8E-07	6.02E-05	0.002249	4.11E-09
0.262989	0.214633	0.075858	0.512862	0.323704	-0.48961	0.027094	-0.27715	-0.52775
0.002815	0.015385	0.396634	7.07E-10	0.000205	5.17E-09	0.762373	0.001608	1.82E-10
0.696699	-0.0711	-0.14828	0.541036	0.561036	-0.55344	0.296224	-0.23512	-0.15769
9.41E-20	0.426983	0.096179	5.14E-11	6.86E-12	1.5E-11	0.000721	0.007794	0.076626
0.579124	0.66917	0.586263	0.251775	0.427103	-0.36149	0.714759	-0.37264	-0.36908
9.86E-13	8.03E-18	4.44E-13	0.004297	5.51E-07	2.97E-05	3.83E-21	1.6E-05	1.96E-05
0.027152	0.029121	0.032117	-0.27239	-0.02179	0.308685	0.196416	0.171827	0.179656
0.761873	0.745183	0.720007	0.001947	0.807856	0.000414	0.026883	0.053404	0.043272
0.457344	0.296936	0.220957	0.341816	0.469085	-0.24096	0.626592	-0.12987	-0.0315
6.47E-08	0.000699	0.012547	8.38E-05	2.66E-08	0.006354	3.29E-15	0.145589	0.725135
0.054485	0.246131	0.543627	-0.21591	0.089648	-0.00664	0.27241	-0.28531	0.120804
0.542923	0.005281	3.99E-11	0.01477	0.316193	0.940972	0.001946	0.001148	0.176089

GTF2E1	GTF2E2	GTF2F1	GTF2F2	GTF2H1	GTF3C3	GTPBP1	GTPBP10	HACD4
cyan	cyan	cyan	cyan	cyan	cyan	cyan	cyan	cyan
-0.12074	0.057981	-0.16927	-0.19718	0.147091	-0.1294	0.049691	-0.1244	0.33099
0.176329	0.517312	0.057106	0.026285	0.098895	0.14706	0.579033	0.163464	0.000144
-0.14317	0.047298	-0.16231	-0.15097	0.171147	-0.09098	-0.01373	-0.06986	0.240534
0.108316	0.59747	0.068272	0.090222	0.05437	0.30901	0.878261	0.435137	0.00645
0.472306	0.303076	-0.05304	0.465226	0.016448	0.420601	0.026851	0.356084	-0.26654
2.07E-08	0.000533	0.553667	3.57E-08	0.854373	8.51E-07	0.764436	3.98E-05	0.002453
0.577525	0.441575	-0.48735	0.430453	0.382225	0.762617	-0.34703	0.669094	0.145753
1.18E-12	2.03E-07	6.22E-09	4.39E-07	9.23E-06	2.08E-25	6.41E-05	8.12E-18	0.102038
0.316419	0.561402	-0.38808	0.261751	0.39631	0.316419	-0.47029	0.301172	0.388922
0.00029	6.61E-12	6.54E-06	0.002953	3.98E-06	0.00029	2.42E-08	0.00058	6.22E-06
0.615528	0.707287	-0.78973	0.429889	0.716973	0.824848	-0.70464	0.750675	0.572241
1.35E-14	1.48E-20	2.66E-28	4.56E-07	2.55E-21	9.43E-33	2.37E-20	2.97E-24	2.09E-12
0.268271	0.463047	-0.4044	0.301626	0.405794	0.630457	-0.4009	0.495012	0.29963
0.002292	4.22E-08	2.41E-06	0.000569	2.21E-06	1.98E-15	3E-06	3.3E-09	0.000621
0.039599	0.0782	-0.37598	-0.08107	0.220143	0.206545	-0.33747	0.145559	0.523213
0.658477	0.382173	1.32E-05	0.364913	0.012885	0.019816	0.000104	0.1025	2.77E-10
0.128447	0.329726	0.095907	0.031755	0.037599	0.127324	-0.12485	0.031032	0.082338
0.150098	0.000153	0.283448	0.723034	0.674717	0.153725	0.16193	0.729088	0.357423
-0.00406	0.180992	-0.17887	-0.11605	0.147466	-0.08556	-0.25973	-0.15614	0.686387
0.963883	0.041714	0.044213	0.193845	0.09803	0.338835	0.003189	0.079613	5.27E-19
0.088248	0.136939	-0.31809	-0.0777	0.291776	0.098406	-0.14816	0.057762	0.596531
0.323842	0.124734	0.000268	0.385217	0.000874	0.271033	0.096442	0.518894	1.36E-13
-0.21903	-0.19888	0.487587	-0.1798	-0.34503	-0.31117	0.448197	-0.30905	-0.60183
0.013357	0.024993	6.1E-09	0.043098	7.11E-05	0.00037	1.26E-07	0.000407	7.24E-14
-0.19676	-0.3408	0.705466	-0.02419	-0.68256	-0.39272	0.670311	-0.44406	-0.64017
0.026612	8.83E-05	2.05E-20	0.787231	9.81E-19	4.95E-06	6.74E-18	1.7E-07	5.34E-16
-0.23311	-0.24831	0.693937	-0.16343	-0.61945	-0.62398	0.400356	-0.49708	-0.37267
0.008351	0.004879	1.5E-19	0.066368	8.25E-15	4.61E-15	3.1E-06	2.77E-09	1.6E-05
-0.32607	-0.27549	0.580781	-0.2361	-0.58651	-0.63344	0.403036	-0.65182	-0.06643
0.000183	0.001719	8.21E-13	0.007534	4.31E-13	1.33E-15	2.63E-06	1.05E-16	0.45803
-0.38168	-0.48135	0.148973	-0.26283	-0.2931	-0.53063	0.251398	-0.42762	0.007878
9.53E-06	1.01E-08	0.094607	0.002833	0.000825	1.39E-10	0.004358	5.32E-07	0.929952
-0.22518	-0.50881	0.504091	-0.00981	-0.53539	-0.38347	0.583507	-0.29811	-0.73084
0.010917	1.01E-09	1.52E-09	0.912828	8.86E-11	8.58E-06	6.05E-13	0.000664	1.79E-22
0.114957	-0.11097	-0.33843	0.013844	0.340233	0.301763	-0.18346	0.433917	-0.12996
0.198108	0.214236	9.96E-05	0.877228	9.09E-05	0.000565	0.038955	3.46E-07	0.14532
-0.34525	-0.4216	0.140113	-0.24947	-0.04864	-0.24883	0.175063	-0.13858	-0.33037
7.03E-05	7.96E-07	0.116151	0.004678	0.587064	0.004788	0.049001	0.120224	0.000149
0.334716	0.27255	0.164	0.374094	-0.2489	0.167076	0.369774	-0.02215	-0.51591
0.00012	0.001935	0.065415	1.47E-05	0.004775	0.060456	1.88E-05	0.804774	5.39E-10

HACL1	HADHB	HAUS1	HAUS2	HAUS6	HBP1	HCFC2	HDAC5	HDAC9
cyan	cyan	cyan	cyan	cyan	cyan	cyan	cyan	cyan
0.155393	0.111922	-0.20252	0.000604	-0.1181	-0.01537	-0.18291	0.187347	-0.08817
0.081075	0.210289	0.022407	0.994626	0.186048	0.863856	0.039561	0.034933	0.324275
0.169202	0.156579	-0.1712	0.002538	-0.10783	-0.03578	-0.14328	0.182007	-0.09729
0.057212	0.078753	0.054292	0.977406	0.22754	0.68963	0.108044	0.040563	0.276537
-0.16425	0.084255	0.643566	0.289306	0.306324	0.322448	0.277494	-0.44754	0.329695
0.065008	0.346299	3.35E-16	0.000971	0.000461	0.000218	0.001586	1.33E-07	0.000154
0.185334	0.296426	0.617301	0.618399	0.572958	0.685464	0.560921	-0.80371	0.562974
0.036973	0.000715	1.08E-14	9.43E-15	1.94E-12	6.13E-19	6.95E-12	5.79E-30	5.61E-12
0.546175	0.315595	0.594397	0.074861	0.025691	0.22456	0.189635	-0.08462	0.062625
3.1E-11	0.000301	1.74E-13	0.402889	0.77434	0.011144	0.032732	0.344223	0.484265
0.620721	0.501558	0.764907	0.63366	0.551926	0.7347	0.610597	-0.77327	0.501041
7.02E-15	1.89E-09	1.23E-25	1.29E-15	1.75E-11	8.29E-23	2.5E-14	1.7E-26	1.98E-09
0.48173	0.286206	0.354606	0.388615	0.256913	0.532786	0.234928	-0.46525	0.316195
9.82E-09	0.001106	4.31E-05	6.33E-06	0.003548	1.14E-10	0.007845	3.57E-08	0.000293
0.527097	0.16055	0.121469	0.071507	0.320309	0.164634	0.063034	-0.24203	-0.01533
1.94E-10	0.071367	0.173706	0.424348	0.000241	0.064368	0.481413	0.006117	0.864209
-0.1058	0.025598	0.188632	0.109127	0.275916	0.43007	-0.16378	-0.39919	0.019086
0.23649	0.775129	0.033681	0.221978	0.00169	4.51E-07	0.065776	3.34E-06	0.831343
0.273802	0.333192	0.014176	0.015354	0.211124	0.262164	-0.30593	-0.13344	-0.26068
0.001841	0.000129	0.874316	0.863967	0.017189	0.002906	0.000469	0.134743	0.003076
0.288255	0.284425	0.03866	0.228904	0.254665	0.335718	-0.03774	-0.32935	0.079333
0.001015	0.001192	0.666086	0.009637	0.00386	0.000114	0.673555	0.000156	0.375294
-0.4074	-0.20436	-0.29607	-0.13199	-0.25684	0.04023	-0.31301	0.224837	-0.15903
2E-06	0.021188	0.000726	0.139095	0.003558	0.653386	0.00034	0.011042	0.074121
-0.64516	-0.29507	-0.22727	-0.33542	-0.20256	-0.25264	-0.46422	0.303871	-0.2322
2.68E-16	0.000758	0.010182	0.000116	0.022376	0.004162	3.86E-08	0.000515	0.008617
-0.40955	-0.25939	-0.14467	-0.5722	-0.40134	-0.44657	-0.48435	0.602117	-0.38186
1.74E-06	0.003231	0.104645	2.1E-12	2.92E-06	1.42E-07	7.94E-09	7E-14	9.43E-06
-0.36994	-0.06696	-0.33978	-0.46823	-0.17526	-0.24457	-0.71772	0.426814	-0.51242
1.86E-05	0.454447	9.3E-05	2.84E-08	0.048747	0.005587	2.22E-21	5.62E-07	7.35E-10
-0.08414	0.035519	-0.54217	-0.35754	-0.31345	-0.55788	-0.31561	0.657743	-0.40789
0.346938	0.691773	4.6E-11	3.68E-05	0.000333	9.52E-12	0.000301	4.44E-17	1.94E-06
-0.51567	-0.35071	-0.27903	-0.30822	-0.39682	-0.59542	-0.10549	0.50701	-0.0901
5.5E-10	5.29E-05	0.001489	0.000423	3.86E-06	1.55E-13	0.237847	1.18E-09	0.313772
0.068363	-0.18272	-0.0348	0.271557	0.000539	-0.06274	0.645567	-0.17641	0.419807
0.44505	0.039766	0.697738	0.002013	0.9952	0.483461	2.53E-16	0.047265	8.96E-07
-0.11809	-0.38667	-0.4599	-0.21617	-0.40781	-0.53172	0.075295	0.409764	-0.07175
0.186094	7.11E-06	5.34E-08	0.014647	1.95E-06	1.26E-10	0.400161	1.72E-06	0.422765
-0.15635	-0.08845	0.334104	-0.07957	-0.11415	0.000286	-0.08149	0.143079	-0.01148
0.079204	0.32271	0.000124	0.373856	0.201287	0.997458	0.3624	0.108551	0.898056

HEATR3	HEBP2	HECTD4	HELQ	HESX1	HGS	HHLA3	HIBADH	HIBCH
cyan	cyan	cyan	cyan	cyan	cyan	cyan	cyan	cyan
-0.19132	-0.01163	0.018835	0.165993	0.151287	-0.08496	0.271338	0.051229	0.007806
0.031186	0.896749	0.833527	0.062166	0.089537	0.342267	0.002031	0.567327	0.930595
-0.2262	0.01095	0.06028	0.130241	0.07165	-0.06629	0.308413	0.04993	0.074597
0.010552	0.902754	0.500811	0.144441	0.423423	0.458983	0.000419	0.577212	0.404551
0.38899	0.021514	-0.17096	0.150288	0.154086	-0.094	-0.1071	0.08917	0.177886
6.19E-06	0.810266	0.054631	0.091697	0.083695	0.293167	0.23075	0.318793	0.04541
0.627406	0.293602	-0.37227	0.543483	0.461175	-0.48559	0.139677	0.451503	0.34894
2.96E-15	0.000808	1.63E-05	4.05E-11	4.86E-08	7.19E-09	0.117301	9.94E-08	5.8E-05
0.162192	0.649851	-0.67332	0.489286	0.269663	-0.34103	0.539613	0.442086	0.192556
0.068483	1.38E-16	4.23E-18	5.31E-09	0.00217	8.73E-05	5.9E-11	1.96E-07	0.030092
0.651459	0.699168	-0.7599	0.820187	0.620643	-0.74887	0.519417	0.738536	0.450834
1.1E-16	6.16E-20	3.87E-25	4.17E-32	7.09E-15	4.38E-24	3.92E-10	3.81E-23	1.04E-07
0.335465	0.40005	-0.42278	0.581765	0.35286	-0.43114	0.455079	0.500887	0.210816
0.000116	3.16E-06	7.36E-07	7.35E-13	4.72E-05	4.19E-07	7.65E-08	2.01E-09	0.017356
0.228221	0.303104	-0.30183	0.27549	0.327934	-0.36	0.344127	0.349366	0.191654
0.009862	0.000532	0.000563	0.00172	0.000167	3.22E-05	7.45E-05	5.68E-05	0.030886
0.259085	0.23484	-0.27441	0.222788	0.230868	0.155598	-0.06319	0.163494	-0.18964
0.003269	0.00787	0.001796	0.011816	0.009017	0.080669	0.480344	0.066262	0.032727
0.234023	0.350154	-0.50386	0.379778	0.517175	-0.05828	0.329817	0.342657	-0.03377
0.008094	5.45E-05	1.55E-09	1.06E-05	4.8E-10	0.515144	0.000153	8.03E-05	0.706273
0.290811	0.234507	-0.43069	0.407908	0.591118	-0.26913	0.346963	0.420413	0.086794
0.00091	0.00796	4.32E-07	1.93E-06	2.55E-13	0.002216	6.43E-05	8.61E-07	0.331905
-0.45114	-0.28667	0.427664	-0.27275	-0.32616	0.675622	-0.09032	-0.34594	-0.39611
1.02E-07	0.001085	5.31E-07	0.00192	0.000182	2.96E-18	0.312587	6.79E-05	4.03E-06
-0.27245	-0.52402	0.478559	-0.47946	-0.26119	0.701195	-0.43173	-0.52558	-0.276
0.001943	2.57E-10	1.26E-08	1.18E-08	0.003016	4.34E-20	4.02E-07	2.23E-10	0.001685
-0.40617	-0.13032	0.035719	-0.46096	-0.24711	0.712092	-0.19874	-0.43284	-0.37446
2.16E-06	0.144186	0.690126	4.94E-08	0.005097	6.25E-21	0.025094	3.73E-07	1.44E-05
-0.27205	-0.17739	0.073076	-0.30585	0.018702	0.676409	-0.13343	-0.30868	-0.32391
0.001974	0.046022	0.414227	0.000471	0.834689	2.61E-18	0.134771	0.000414	0.000203
-0.43434	-0.36474	0.408242	-0.40581	-0.22561	0.184389	-0.03103	-0.354	-0.04622
3.36E-07	2.49E-05	1.89E-06	2.21E-06	0.010763	0.037965	0.729096	4.45E-05	0.605871
-0.41214	-0.63365	0.648637	-0.68636	-0.63105	0.365424	-0.46764	-0.62685	-0.15927
1.48E-06	1.29E-15	1.64E-16	5.29E-19	1.83E-15	2.4E-05	2.97E-08	3.18E-15	0.073681
0.083532	-0.17733	0.276459	-0.08129	-0.28229	-0.48302	-0.2146	-0.00199	0.177548
0.350467	0.0461	0.001654	0.3636	0.001302	8.85E-09	0.015402	0.982302	0.045827
-0.39891	-0.39933	0.575096	-0.47313	-0.62074	0.007592	-0.29755	-0.35071	-0.12952
3.39E-06	3.31E-06	1.53E-12	1.94E-08	7E-15	0.93249	0.00068	5.29E-05	0.146683
-0.00829	-0.23938	0.212381	-0.02351	-0.1221	0.212915	-0.25694	-0.34732	-0.03098
0.926307	0.006718	0.016523	0.793074	0.171473	0.016246	0.003545	6.32E-05	0.729535

HIF1AN	HINFP	HIST1H2B(	HIST1H2BI	HIST1H2BI	HMCES	HMGN4	HMX1	HNRNPC
cyan	cyan	cyan	cyan	cyan	cyan	cyan	cyan	cyan
0.324924	-0.15442	0.305889	0.223576	0.332917	-0.07645	-0.03937	-0.10057	-0.17433
0.000194	0.083011	0.00047	0.011513	0.000131	0.392952	0.660346	0.260605	0.049977
0.324653	-0.07154	0.287358	0.220042	0.38247	-0.07827	-0.03445	-0.11777	-0.11578
0.000196	0.424122	0.001054	0.012927	9.1E-06	0.381751	0.700567	0.187273	0.194888
-0.3304	0.439601	-0.11569	0.096135	0.016975	-0.12878	0.582551	0.089263	0.527335
0.000148	2.33E-07	0.195229	0.2823	0.84976	0.149049	6.74E-13	0.318283	1.89E-10
-0.3335	0.344172	0.164462	0.245352	0.158793	-0.40296	0.779743	-0.38972	0.651771
0.000127	7.43E-05	0.06465	0.005432	0.074564	2.64E-06	3.46E-27	5.93E-06	1.05E-16
-0.41833	0.398021	0.401095	0.533952	0.481794	-0.20516	0.187572	-0.11652	0.460674
9.88E-07	3.59E-06	2.97E-06	1.02E-10	9.77E-09	0.020678	0.034712	0.192052	5.04E-08
-0.46138	0.399159	0.510217	0.571788	0.515022	-0.50755	0.755535	-0.56117	0.719124
4.78E-08	3.34E-06	8.93E-10	2.2E-12	5.83E-10	1.13E-09	1.03E-24	6.77E-12	1.7E-21
-0.21941	0.314287	0.426503	0.353848	0.332402	-0.2435	0.454881	-0.36225	0.527748
0.013197	0.00032	5.74E-07	4.48E-05	0.000134	0.005805	7.76E-08	2.85E-05	1.82E-10
-0.10437	-0.05015	0.1733	0.193264	0.065536	-0.064	0.081808	-0.2996	0.061894
0.242894	0.5755	0.051361	0.02948	0.464144	0.474698	0.360534	0.000622	0.48939
-0.16428	-0.14746	-0.05058	0.126033	0.218837	-0.16085	0.435109	-0.11311	0.08948
0.064945	0.098048	0.572242	0.157974	0.013442	0.070841	3.19E-07	0.20544	0.317104
-0.02845	-0.36916	0.281945	0.321375	0.439977	-0.34905	0.157211	-0.18657	-0.13473
0.750817	1.95E-05	0.001321	0.000229	2.27E-07	5.77E-05	0.077538	0.035706	0.130985
-0.07736	-0.28631	0.258399	0.307927	0.338585	-0.31075	0.328963	-0.36953	0.021905
0.387301	0.001102	0.003355	0.000429	9.88E-05	0.000377	0.000159	1.91E-05	0.806885
0.15582	-0.19338	-0.43206	-0.28899	-0.09024	0.253144	-0.14975	0.226717	-0.11838
0.080232	0.02938	3.93E-07	0.000984	0.313012	0.004085	0.092868	0.010372	0.184998
0.181898	-0.07896	-0.5252	-0.35942	-0.38065	0.27697	-0.15787	0.497769	-0.24579
0.040685	0.377571	2.31E-10	3.33E-05	1.01E-05	0.00162	0.07629	2.62E-09	0.005346
-0.14761	-0.09346	-0.31883	-0.12266	-0.1832	0.236406	-0.45342	0.49456	-0.25317
0.097688	0.295963	0.000259	0.169484	0.039246	0.007454	8.64E-08	3.43E-09	0.004081
0.14071	-0.45188	-0.23435	-0.10339	-0.03149	0.076655	-0.2869	0.363671	-0.47113
0.114588	9.67E-08	0.008003	0.247391	0.725215	0.391675	0.001074	2.64E-05	2.27E-08
0.519246	-0.3696	0.012119	-0.21498	-0.08896	0.081174	-0.63001	0.295232	-0.47495
3.98E-10	1.9E-05	0.892431	0.015215	0.319955	0.364279	2.1E-15	0.000753	1.68E-08
0.188901	0.057008	-0.43319	-0.50283	-0.52662	0.365174	-0.47775	0.577641	-0.14504
0.033425	0.524377	3.64E-07	1.7E-09	2.03E-10	2.43E-05	1.35E-08	1.16E-12	0.103748
0.044512	0.198904	0.058024	-0.2227	-0.26053	0.067056	-0.01142	-0.23955	0.149736
0.619249	0.024973	0.516997	0.011852	0.003094	0.453827	0.898579	0.006677	0.09291
0.296524	-0.00063	-0.15511	-0.41254	-0.40065	0.405052	-0.55262	0.146239	-0.30278
0.000712	0.994383	0.081639	1.44E-06	3.05E-06	2.32E-06	1.63E-11	0.100888	0.00054
0.085815	0.4342	-0.12219	0.015092	-0.01988	0.177336	0.083884	0.546474	0.240808
0.337412	3.39E-07	0.171133	0.866262	0.824401	0.046091	0.348436	3.01E-11	0.006388

HNRNPLL	HORMAD2	HOXB9	HS3ST3B1	HS6ST1	HSCB	HSD11B1L	HSD17B11	HSD17B7
cyan	cyan	cyan	cyan	cyan	cyan	cyan	cyan	cyan
-0.29321	0.071734	0.127757	-0.08861	-0.10383	0.133857	-0.05618	0.233756	0.199018
0.000821	0.422876	0.152319	0.321825	0.245388	0.133527	0.53045	0.008169	0.024888
-0.30383	0.093465	0.201418	-0.06735	-0.08791	0.123039	-1.6E-05	0.207766	0.187131
0.000515	0.295943	0.023162	0.451816	0.325704	0.168168	0.999858	0.019084	0.035148
0.533067	0.012969	0.225396	0.304749	-0.13464	0.224366	-0.21639	-0.10677	0.085569
1.11E-10	0.884941	0.010839	0.000495	0.131236	0.011216	0.014547	0.23218	0.338804
0.614965	0.298658	0.456662	0.611614	-0.35332	0.338608	-0.63904	0.123057	0.282914
1.45E-14	0.000648	6.8E-08	2.2E-14	4.61E-05	9.87E-05	6.25E-16	0.168106	0.001269
0.241557	0.282132	0.322392	0.023064	-0.4175	0.67282	-0.08326	0.430544	0.445296
0.00622	0.001311	0.000219	0.796886	1.04E-06	4.58E-18	0.352018	4.36E-07	1.56E-07
0.605274	0.523832	0.59648	0.553838	-0.5782	0.678771	-0.70056	0.496701	0.534758
4.79E-14	2.62E-10	1.37E-13	1.44E-11	1.09E-12	1.8E-18	4.84E-20	2.86E-09	9.41E-11
0.242615	0.376042	0.467302	0.37214	-0.34389	0.348536	-0.45625	0.252308	0.333211
0.005991	1.32E-05	3.05E-08	1.65E-05	7.54E-05	5.93E-05	7.01E-08	0.004214	0.000129
0.042816	0.462126	-0.03713	0.043954	-0.24089	0.055829	-0.2738	0.110236	0.176966
0.632677	4.52E-08	0.678552	0.623654	0.006368	0.533003	0.00184	0.217284	0.046555
-0.01654	-0.06216	0.328478	0.453373	-0.11663	0.007907	-0.48937	0.081453	0.036385
0.85359	0.487545	0.000163	8.67E-08	0.191607	0.929695	5.27E-09	0.362631	0.684656
-0.3686	0.106956	0.147612	0.19021	-0.19311	0.182375	-0.52221	0.389887	0.267515
2.01E-05	0.231364	0.097694	0.032197	0.02961	0.040152	3.04E-10	5.87E-06	0.002361
-0.0886	0.22468	0.211817	0.361422	-0.12201	0.206087	-0.55882	0.147054	0.280672
0.321886	0.0111	0.016819	2.98E-05	0.171792	0.020097	8.64E-12	0.098983	0.001392
-0.43556	-0.33506	-0.04995	-0.02853	0.305078	-0.16689	0.234573	-0.44497	-0.14878
3.09E-07	0.000118	0.577025	0.750192	0.000487	0.060744	0.007942	1.59E-07	0.095035
-0.30349	-0.44813	-0.27587	-0.13605	0.369355	-0.40357	0.294867	-0.54315	-0.27193
0.000523	1.27E-07	0.001694	0.12722	1.93E-05	2.54E-06	0.000765	4.18E-11	0.001984
-0.36409	-0.34097	-0.29087	-0.29354	0.378581	-0.1384	0.55293	-0.32224	-0.135
2.58E-05	8.75E-05	0.000908	0.00081	1.14E-05	0.120715	1.58E-11	0.00022	0.130201
-0.67948	-0.33616	-0.23921	-0.14983	0.27682	-0.27883	0.085117	-0.21711	-0.09681
1.61E-18	0.000112	0.006759	0.092698	0.00163	0.001502	0.341367	0.014211	0.278898
-0.59476	-0.1295	-0.39236	-0.58898	0.144894	-0.29247	0.354323	-0.0735	-0.22687
1.67E-13	0.146746	5.06E-06	3.25E-13	0.104095	0.000848	4.37E-05	0.411498	0.010319
-0.02609	-0.35686	-0.45546	-0.49649	0.399681	-0.36936	0.72186	-0.48228	-0.4566
0.770898	3.82E-05	7.43E-08	2.91E-09	3.24E-06	1.93E-05	1.02E-21	9.39E-09	6.84E-08
0.540649	0.138471	-0.05501	-0.0293	0.010053	-0.09188	0.159872	0.041165	-0.22267
5.34E-11	0.120531	0.539005	0.743678	0.910682	0.304248	0.072586	0.645867	0.011862
0.015195	-0.14949	-0.36599	-0.4641	0.258166	-0.35054	0.622028	-0.14126	-0.40049
0.865358	0.093457	2.32E-05	3.89E-08	0.003385	5.34E-05	5.93E-15	0.113165	3.08E-06
0.160306	-0.32028	-0.01758	-0.26104	-0.19514	0.133782	0.19679	0.023661	0.011094
0.071804	0.000242	0.844465	0.003034	0.027907	0.133746	0.026588	0.791742	0.901478

HSDL2	HSPA12B	HSPB11	HSPD1	HSPE1	HUS1	ICE2	IER3IP1	IFNGR1
cyan	cyan	cyan	cyan	cyan	cyan	cyan	cyan	cyan
0.018984	-0.10075	0.076754	-0.19107	0.054861	-0.0805	-0.13751	-0.20862	0.168412
0.832231	0.259726	0.391068	0.031414	0.54014	0.368305	0.123153	0.018585	0.0584
0.085105	-0.131	0.098557	-0.15471	0.143613	-0.08502	-0.14724	-0.1896	0.186384
0.341438	0.142102	0.270295	0.082434	0.107224	0.34191	0.098541	0.032761	0.035896
-0.04588	-0.22729	0.147911	0.630131	0.137826	0.504441	0.376593	0.52548	-0.2702
0.608506	0.010175	0.097009	2.07E-15	0.122286	1.48E-09	1.28E-05	2.25E-10	0.002125
0.234868	-0.4803	0.30107	0.75166	0.287759	0.761587	0.762979	0.582372	0.171902
0.007862	1.1E-08	0.000583	2.4E-24	0.001036	2.63E-25	1.92E-25	6.87E-13	0.053298
0.16444	-0.27111	0.725485	0.071004	0.701307	0.129148	0.101251	0.380773	0.481925
0.064687	0.002049	5.08E-22	0.427622	4.25E-20	0.147869	0.257353	1E-05	9.66E-09
0.395338	-0.60918	0.740067	0.598463	0.681292	0.723434	0.726448	0.682564	0.622903
4.22E-06	2.98E-14	2.78E-23	1.08E-13	1.2E-18	7.54E-22	4.22E-22	9.8E-19	5.3E-15
0.192183	-0.44346	0.426547	0.455963	0.406104	0.404866	0.390302	0.359022	0.427812
0.030419	1.77E-07	5.72E-07	7.16E-08	2.17E-06	2.34E-06	5.73E-06	3.4E-05	5.25E-07
0.45116	-0.33545	0.34131	0.176578	0.229505	0.054734	0.258399	-0.00881	0.307776
1.02E-07	0.000116	8.6E-05	0.047045	0.009444	0.541082	0.003355	0.921688	0.000432
-0.3414	0.052188	-0.05345	0.284406	0.013222	0.306212	0.157167	0.357152	0.058103
8.56E-05	0.560089	0.550603	0.001193	0.88271	0.000463	0.077621	3.76E-05	0.516431
0.005631	-0.06357	0.145484	-0.09318	0.213461	0.02743	0.120888	0.020765	0.574937
0.949899	0.477655	0.102678	0.297407	0.015968	0.759513	0.175787	0.816754	1.56E-12
0.153717	-0.14561	0.105255	0.104202	0.196193	0.281578	0.354665	0.00953	0.431653
0.084447	0.102382	0.238914	0.24367	0.02706	0.001341	4.29E-05	0.915311	4.04E-07
-0.37848	0.340352	-0.59674	-0.22526	-0.25083	-0.10953	-0.22589	-0.34615	-0.31163
1.15E-05	9.03E-05	1.32E-13	0.010887	0.00445	0.220281	0.010661	6.71E-05	0.000362
-0.43747	0.364114	-0.60957	0.028709	-0.46028	-0.21911	-0.26833	-0.25584	-0.69062
2.71E-07	2.57E-05	2.84E-14	0.74866	5.19E-08	0.013322	0.002287	0.003694	2.62E-19
-0.38516	0.469407	-0.32982	-0.3573	-0.13242	-0.48871	-0.47398	-0.2521	-0.42512
7.77E-06	2.59E-08	0.000153	3.73E-05	0.137772	5.57E-09	1.82E-08	0.004247	6.3E-07
-0.36633	0.447409	-0.46034	-0.2773	-0.23429	-0.42328	-0.34597	-0.36528	-0.19141
2.28E-05	1.34E-07	5.17E-08	0.001598	0.008019	7.12E-07	6.77E-05	2.41E-05	0.031109
0.101575	0.331772	-0.26046	-0.56941	-0.19829	-0.64143	-0.39439	-0.65192	0.056465
0.255825	0.000139	0.003102	2.84E-12	0.025431	4.5E-16	4.47E-06	1.03E-16	0.528345
-0.11257	0.349931	-0.46747	-0.24569	-0.42315	-0.39572	-0.44987	-0.39196	-0.65277
0.20764	5.51E-05	3.01E-08	0.005366	7.18E-07	4.13E-06	1.12E-07	5.18E-06	9.12E-17
0.288121	-0.18585	0.097011	-0.01	-0.14987	0.164195	0.141639	0.116823	-0.05137
0.001021	0.036444	0.277916	0.911189	0.092614	0.065092	0.112191	0.190875	0.566295
0.02319	0.158531	-0.17881	-0.45924	-0.36816	-0.38145	-0.43646	-0.34516	-0.24726
0.795798	0.075049	0.044284	5.61E-08	2.06E-05	9.65E-06	2.9E-07	7.06E-05	0.005069
-0.43663	0.026111	0.096436	0.225461	0.01331	0.01202	-0.1698	0.255414	-0.3901
2.87E-07	0.770748	0.280789	0.010816	0.881934	0.8933	0.056329	0.003754	5.8E-06

IFT22	IFT52	IFT57	IFT74	IFT88	IGHMBP2	IGSF21	IKBIP	IL7
cyan	cyan	cyan	cyan	cyan	cyan	cyan	cyan	cyan
-0.06231	-0.16397	0.109252	0.187463	-0.03778	-0.07964	0.011619	-0.18203	0.078103
0.48649	0.065467	0.221443	0.034819	0.673278	0.373444	0.896846	0.040537	0.382769
-0.01505	-0.11418	0.065859	0.149937	-0.02486	-0.06085	0.086391	-0.24406	-0.00055
0.866654	0.201178	0.46194	0.092468	0.781447	0.496743	0.334165	0.00569	0.995093
0.081052	0.559359	0.020182	-0.00136	0.073927	-0.0976	-0.41541	0.526487	0.171805
0.365008	8.17E-12	0.821817	0.987861	0.408802	0.274987	1.2E-06	2.05E-10	0.053435
0.291996	0.551088	0.388102	0.354256	0.419116	-0.46666	-0.81594	0.706656	0.582127
0.000865	1.9E-11	6.53E-06	4.39E-05	9.38E-07	3.2E-08	1.56E-31	1.66E-20	7.06E-13
0.590285	0.440052	0.448374	0.240878	0.33815	-0.24098	-0.01631	0.172882	0.195006
2.8E-13	2.26E-07	1.25E-07	0.006372	0.000101	0.00635	0.855586	0.051934	0.028019
0.634667	0.63852	0.723121	0.574349	0.604897	-0.64102	-0.73227	0.690586	0.722538
1.13E-15	6.7E-16	8E-22	1.67E-12	5.01E-14	4.76E-16	1.35E-22	2.63E-19	8.94E-22
0.439763	0.373034	0.445088	0.275417	0.462385	-0.37625	-0.4517	0.352674	0.470993
2.3E-07	1.57E-05	1.58E-07	0.001725	4.43E-08	1.3E-05	9.8E-08	4.77E-05	2.29E-08
0.547116	0.035552	0.635145	0.589999	0.614732	-0.43055	-0.33261	0.081069	0.247593
2.83E-11	0.691502	1.06E-15	2.9E-13	1.5E-14	4.36E-07	0.000133	0.364903	0.005009
-0.08554	0.174398	0.044118	0.099248	-0.20697	-0.10815	-0.41419	0.763933	0.449609
0.338958	0.04988	0.622362	0.266935	0.019557	0.226166	1.29E-06	1.54E-25	1.14E-07
0.012693	-0.11898	0.247964	0.362413	-0.00404	-0.25959	-0.22102	0.432242	0.508061
0.887372	0.182761	0.004942	2.83E-05	0.964007	0.003206	0.012522	3.88E-07	1.08E-09
0.105662	-0.08587	0.307761	0.363985	0.114214	-0.35232	-0.47219	0.396137	0.562571
0.237093	0.337071	0.000432	2.59E-05	0.201042	4.86E-05	2.09E-08	4.02E-06	5.85E-12
-0.33476	-0.1694	-0.54486	-0.53818	-0.35328	0.35661	0.270961	-0.19358	-0.25135
0.00012	0.056924	3.53E-11	6.78E-11	4.62E-05	3.87E-05	0.002061	0.029209	0.004365
-0.55392	-0.17264	-0.64317	-0.41271	-0.4372	0.487462	0.201024	-0.07054	-0.39739
1.43E-11	0.052264	3.54E-16	1.42E-06	2.76E-07	6.17E-09	0.023438	0.430685	3.73E-06
-0.27626	-0.25815	-0.54837	-0.42623	-0.49625	0.496768	0.522803	-0.18188	-0.43611
0.001667	0.003387	2.5E-11	5.84E-07	2.97E-09	2.85E-09	2.88E-10	0.040707	2.97E-07
-0.49525	-0.41427	-0.47068	-0.16865	-0.47717	0.324957	0.263884	0.044068	-0.13615
3.23E-09	1.29E-06	2.35E-08	0.058046	1.41E-08	0.000193	0.00272	0.622757	0.126953
-0.37986	-0.56051	-0.36115	-0.13329	-0.11517	0.130992	0.582004	-0.71783	-0.43003
1.06E-05	7.25E-12	3.03E-05	0.135204	0.197276	0.142121	7.16E-13	2.17E-21	4.52E-07
-0.35655	-0.19519	-0.55608	-0.61259	-0.29084	0.411585	0.571803	-0.66113	-0.71356
3.88E-05	0.027868	1.14E-11	1.95E-14	0.000909	1.53E-06	2.19E-12	2.69E-17	4.77E-21
0.165657	0.084476	0.158009	-0.06285	0.201655	-0.13527	-0.03325	-0.32026	-0.10781
0.062705	0.345032	0.076025	0.48272	0.022998	0.129446	0.710555	0.000242	0.227626
-0.04896	-0.27018	-0.14979	-0.33452	-0.11387	0.265273	0.564149	-0.78628	-0.54952
0.584641	0.002126	0.092794	0.000121	0.20241	0.002577	4.96E-12	6.57E-28	2.22E-11
-0.28824	0.405501	-0.23744	-0.26511	-0.09008	0.301501	0.177072	-0.00559	-0.30889
0.001015	2.25E-06	0.00719	0.002594	0.313868	0.000572	0.046422	0.950264	0.00041

IMMP2L	ING3	INSIG2	INSL5	INTS10	IQCB1	IQCE	IRAK1BP1	IRAK4
cyan	cyan	cyan	cyan	cyan	cyan	cyan	cyan	cyan
-0.10157	-0.30989	0.117494	0.076043	-0.05643	-0.02014	-0.24727	-0.30835	0.006969
0.255831	0.000392	0.188324	0.395483	0.528584	0.822164	0.005068	0.00042	0.938014
-0.08677	-0.28761	0.149627	0.050882	-0.02781	-0.0801	-0.26463	-0.28946	-0.04807
0.332037	0.001043	0.093151	0.569963	0.756247	0.370688	0.002643	0.000964	0.591509
0.317762	0.626602	0.138124	0.099	0.283579	0.310791	0.073335	0.526186	0.2429
0.000272	3.28E-15	0.121474	0.268138	0.001235	0.000376	0.412574	2.11E-10	0.00593
0.548012	0.760573	0.465804	0.366699	0.500216	0.649505	-0.08454	0.638502	0.660279
2.59E-11	3.32E-25	3.42E-08	2.23E-05	2.12E-09	1.45E-16	0.344638	6.72E-16	3.06E-17
0.516035	0.295051	0.278038	0.209846	0.315369	0.39413	-0.45921	0.261047	0.209764
5.32E-10	0.000759	0.001551	0.01789	0.000304	4.55E-06	5.63E-08	0.003033	0.017935
0.763115	0.747209	0.634155	0.489988	0.591039	0.817219	-0.45802	0.618802	0.74962
1.86E-25	6.25E-24	1.21E-15	5.01E-09	2.57E-13	1.05E-31	6.15E-08	8.96E-15	3.73E-24
0.508861	0.411392	0.39466	0.440528	0.402356	0.566694	-0.18238	0.306145	0.496805
1.01E-09	1.55E-06	4.4E-06	2.18E-07	2.74E-06	3.79E-12	0.040145	0.000465	2.84E-09
0.120777	0.099552	0.033531	0.542228	0.119259	0.36626	0.138254	0.293233	0.318368
0.176187	0.265465	0.708219	4.58E-11	0.181724	2.29E-05	0.121118	0.000821	0.000265
0.162017	0.161489	0.241569	0.072246	0.272932	0.158688	-0.06931	-0.10434	0.477689
0.068786	0.069705	0.006218	0.419563	0.001906	0.074757	0.438754	0.243036	1.36E-08
0.097677	-0.1328	0.049257	0.212767	0.152613	0.114718	-0.30612	-0.37677	0.549701
0.274615	0.136637	0.582356	0.016323	0.08673	0.199049	0.000465	1.27E-05	2.18E-11
0.150018	0.094872	0.108641	0.281173	0.134688	0.307224	-0.15037	-0.07338	0.537
0.092288	0.2887	0.224053	0.001364	0.131112	0.000442	0.091521	0.412301	7.59E-11
-0.22335	-0.25714	-0.23402	-0.26823	0.027589	-0.39262	0.257425	-0.37967	-0.216
0.011598	0.003518	0.008094	0.002296	0.758164	4.98E-06	0.003481	1.07E-05	0.01473
-0.41381	-0.2394	-0.43312	-0.29154	-0.24438	-0.43352	0.486147	-0.27528	-0.29361
1.33E-06	0.006714	3.66E-07	0.000883	0.005625	3.56E-07	6.87E-09	0.001734	0.000807
-0.32854	-0.36161	-0.46675	-0.34474	-0.30341	-0.49127	0.230884	-0.3748	-0.38928
0.000163	2.95E-05	3.18E-08	7.22E-05	0.000525	4.51E-09	0.009012	1.42E-05	6.09E-06
-0.43946	-0.50915	-0.46223	-0.20199	-0.25583	-0.46497	0.205919	-0.65521	-0.04834
2.35E-07	9.81E-10	4.49E-08	0.022766	0.003696	3.64E-08	0.020201	6.41E-17	0.589429
-0.5595	-0.62722	-0.46604	-0.19443	-0.36233	-0.5228	0.038484	-0.51476	-0.39849
8.05E-12	3.03E-15	3.36E-08	0.028499	2.84E-05	2.88E-10	0.667513	5.97E-10	3.48E-06
-0.43119	-0.1863	-0.47512	-0.39809	-0.34172	-0.52407	0.440306	-0.01207	-0.73528
4.17E-07	0.035978	1.66E-08	3.57E-06	8.43E-05	2.56E-10	2.22E-07	0.892875	7.38E-23
0.076777	0.240442	0.207197	-0.01533	-0.03125	0.126997	-0.03534	0.458185	-0.23484
0.390924	0.006471	0.019422	0.864144	0.727286	0.154793	0.693221	6.07E-08	0.007871
-0.31805	-0.3129	-0.16453	-0.27129	-0.34733	-0.35979	0.141177	-0.03688	-0.69204
0.000269	0.000341	0.064541	0.002035	6.31E-05	3.26E-05	0.113378	0.680607	2.07E-19
0.045595	0.082271	-0.02205	-0.17888	0.107443	0.071536	-0.131	0.085547	-0.21011
0.610741	0.357812	0.805618	0.044203	0.229235	0.424161	0.142097	0.338925	0.017745

IRGQ	ISOC1	ITCH	ITGAE	ITGAV	ITGB3BP	JAG2	JKAMP	KANSL2
cyan	cyan	cyan	cyan	cyan	cyan	cyan	cyan	cyan
-0.29724	0.081008	0.000271	-0.02219	-0.2869	-0.12757	-0.11683	0.145188	-0.18255
0.00069	0.365264	0.997584	0.80443	0.001075	0.152929	0.190863	0.103387	0.039955
-0.24302	0.055192	0.004709	0.091075	-0.36506	-0.05562	-0.13035	0.16423	-0.10283
0.005905	0.537698	0.958096	0.308522	2.44E-05	0.53456	0.144099	0.065034	0.249967
0.290517	-0.11588	0.105775	0.20561	0.285512	0.533748	-0.33029	0.16918	0.540624
0.000922	0.194491	0.236585	0.020393	0.001139	1.04E-10	0.000149	0.057244	5.35E-11
-0.00907	0.245673	0.600101	0.281799	0.595574	0.617665	-0.62528	0.544499	0.600314
0.919369	0.005369	8.91E-14	0.001329	1.52E-13	1.03E-14	3.9E-15	3.66E-11	8.68E-14
-0.40209	0.271221	0.018888	0.781147	-0.2678	0.576932	-0.41486	0.416443	0.395022
2.79E-06	0.00204	0.833066	2.43E-27	0.002335	1.26E-12	1.24E-06	1.12E-06	4.31E-06
-0.43655	0.528121	0.61656	0.692422	0.39259	0.80141	-0.79624	0.822537	0.648184
2.89E-07	1.76E-10	1.19E-14	1.94E-19	4.99E-06	1.11E-29	4.65E-29	1.98E-32	1.75E-16
-0.21786	0.205977	0.393198	0.426197	0.221851	0.470084	-0.51916	0.481374	0.457495
0.013876	0.020165	4.81E-06	5.86E-07	0.012185	2.46E-08	4.02E-10	1.01E-08	6.39E-08
-0.2721	0.434512	0.097833	0.029336	0.151496	0.209284	-0.26918	0.154132	-0.11876
0.00197	3.32E-07	0.273845	0.743365	0.08909	0.018206	0.002212	0.083602	0.183583
-0.03794	-0.15577	0.084329	-0.17086	0.555858	0.168873	0.027198	0.052968	0.054573
0.671946	0.080322	0.345876	0.054788	1.17E-11	0.057704	0.761486	0.554229	0.54227
-0.49239	0.143399	0.045443	-0.04343	0.314066	-0.04767	-0.08811	0.141359	-0.27158
4.11E-09	0.107753	0.611935	0.627774	0.000323	0.594608	0.324627	0.112909	0.002012
-0.48512	0.333701	0.238918	-0.07782	0.305802	0.010426	-0.2654	0.2364	-0.11659
7.47E-09	0.000126	0.006828	0.384498	0.000472	0.907389	0.002565	0.007455	0.191783
0.27107	-0.40889	-0.12703	-0.32474	-0.16257	-0.38	0.262233	-0.45689	-0.09267
0.002053	1.82E-06	0.15468	0.000195	0.067839	1.05E-05	0.002899	6.69E-08	0.3001
0.584529	-0.59934	-0.41654	-0.57021	-0.02757	-0.33993	0.412889	-0.636	-0.22195
5.4E-13	9.75E-14	1.11E-06	2.6E-12	0.758318	9.23E-05	1.41E-06	9.43E-16	0.012144
0.199051	-0.3539	-0.61565	-0.23685	-0.30205	-0.25013	0.527604	-0.61192	-0.31262
0.024864	4.47E-05	1.33E-14	0.007339	0.000558	0.004566	1.85E-10	2.12E-14	0.000346
0.080661	-0.37797	-0.47808	-0.54069	0.019556	-0.46515	0.497603	-0.57086	-0.56246
0.367328	1.18E-05	1.31E-08	5.32E-11	0.827252	3.59E-08	2.65E-09	2.43E-12	5.92E-12
-0.02816	-0.07427	-0.22809	-0.18227	-0.46105	-0.56225	0.357106	-0.26526	-0.47175
0.753345	0.406609	0.009905	0.040271	4.9E-08	6.05E-12	3.77E-05	0.002579	2.16E-08
0.540962	-0.42647	-0.41404	-0.19807	-0.44586	-0.35273	0.423294	-0.4971	-0.08727
5.18E-11	5.75E-07	1.31E-06	0.025601	1.5E-07	4.76E-05	7.11E-07	2.77E-09	0.329228
0.067373	0.275216	0.380943	0.206378	-0.0297	0.089154	-0.13962	0.310618	0.272398
0.451687	0.001739	9.95E-06	0.019918	0.740293	0.318875	0.117462	0.000379	0.001947
0.179702	-0.06524	-0.16238	-0.06234	-0.4666	-0.3463	0.314486	-0.2011	-0.14529
0.043217	0.466203	0.068166	0.486253	3.22E-08	6.66E-05	0.000317	0.023386	0.10314
0.49503	-0.53855	-0.1923	0.147627	-0.3075	0.202877	-0.00519	-0.04756	0.335037
3.29E-09	6.54E-11	0.030319	0.097658	0.000437	0.022164	0.95379	0.595416	0.000118

KARS	KAT6B	KATNBL1	KAZN	KCNQ4	KCTD13	KCTD9	KDELR3	KIAA0895L
cyan	cyan	cyan	cyan	cyan	cyan	cyan	cyan	cyan
-0.12396	-0.0587	-0.16627	-0.02675	0.121218	-0.06747	0.162051	0.087798	-0.2132
0.164992	0.512108	0.061725	0.765302	0.174603	0.45105	0.068727	0.326321	0.016103
-0.08492	-0.07834	-0.1096	0.014832	0.133406	0.002832	0.106577	0.016601	-0.15724
0.34247	0.381296	0.21997	0.868548	0.134853	0.974796	0.23303	0.853037	0.077489
0.506378	0.178179	0.411386	-0.07723	-0.39581	-0.26121	0.089944	0.272424	0.293359
1.25E-09	0.045049	1.55E-06	0.38813	4.1E-06	0.003014	0.31459	0.001945	0.000816
0.498411	-0.09291	0.57364	-0.17498	-0.5229	-0.60555	0.588013	0.609815	-0.14141
2.48E-09	0.298857	1.8E-12	0.049114	2.86E-10	4.63E-14	3.64E-13	2.75E-14	0.112782
0.451706	-0.42271	0.263722	-0.46081	-0.40807	-0.17138	0.309341	0.222755	-0.18324
9.8E-08	7.4E-07	0.002737	4.99E-08	1.91E-06	0.054041	0.000402	0.011829	0.039199
0.598373	-0.47344	0.610927	-0.45346	-0.66165	-0.68297	0.821059	0.71901	-0.45205
1.09E-13	1.89E-08	2.4E-14	8.62E-08	2.49E-17	9.18E-19	3.17E-32	1.74E-21	9.55E-08
0.496015	-0.38244	0.320228	-0.28658	-0.40556	-0.40365	0.58952	0.484706	-0.21007
3.03E-09	9.12E-06	0.000242	0.001089	2.24E-06	2.53E-06	3.06E-13	7.72E-09	0.017764
0.059546	-0.28336	-0.14044	-0.06029	0.006154	-0.32158	0.401898	0.154719	-0.33822
0.506044	0.001246	0.115284	0.500768	0.945253	0.000227	2.82E-06	0.082418	0.000101
0.347331	-0.24554	-0.12323	-0.10152	-0.20143	-0.40003	0.326026	0.635517	-0.1372
6.31E-05	0.005394	0.167491	0.256077	0.023156	3.17E-06	0.000184	1.01E-15	0.124022
0.167786	-0.44805	-0.24987	-0.05111	-0.10801	-0.46956	0.373017	0.363692	-0.48823
0.059356	1.28E-07	0.00461	0.568213	0.22678	2.56E-08	1.57E-05	2.63E-05	5.79E-09
0.192147	-0.34537	-0.1158	-0.032	-0.0952	-0.53796	0.450585	0.431891	-0.50575
0.03045	6.99E-05	0.194831	0.720995	0.287023	6.92E-11	1.06E-07	3.98E-07	1.32E-09
0.014656	0.283432	-0.38673	0.38411	0.319207	0.177503	-0.31878	-0.08953	0.224467
0.870089	0.001242	7.09E-06	8.27E-06	0.000254	0.045884	0.00026	0.316829	0.011178
-0.07675	0.53145	-0.32154	0.430924	0.322971	0.30616	-0.56973	-0.30595	0.587556
0.391103	1.29E-10	0.000228	4.25E-07	0.000213	0.000464	2.74E-12	0.000469	3.83E-13
-0.19812	0.158622	-0.41338	0.22063	0.277383	0.534604	-0.67439	-0.45566	0.357961
0.025558	0.074881	1.36E-06	0.012682	0.001593	9.55E-11	3.59E-18	7.33E-08	3.6E-05
-0.15909	0.09728	-0.60331	0.381104	0.342673	0.162936	-0.40574	-0.1961	0.137656
0.074006	0.276582	6.07E-14	9.85E-06	8.03E-05	0.067207	2.22E-06	0.027134	0.122753
-0.53596	0.172141	-0.28226	0.409892	0.437911	0.374158	-0.46524	-0.62487	0.076146
8.39E-11	0.052964	0.001304	1.71E-06	2.62E-07	1.47E-05	3.57E-08	4.11E-15	0.39484
-0.32162	0.55262	-0.09268	0.33486	0.355363	0.561487	-0.75801	-0.71461	0.597456
0.000227	1.63E-11	0.300057	0.000119	4.14E-05	6.55E-12	5.91E-25	3.94E-21	1.22E-13
-0.27489	0.096065	0.46241	-0.20576	-0.10098	0.129346	0.093675	-0.11211	-0.05799
0.001762	0.282654	4.42E-08	0.020302	0.258657	0.147242	0.294856	0.20951	0.517261
-0.47973	0.207539	-0.05628	-0.02141	0.297197	0.524573	-0.37637	-0.54445	0.173208
1.15E-08	0.019218	0.529732	0.811184	0.000691	2.45E-10	1.29E-05	3.68E-11	0.051486
0.321469	0.378627	0.307897	-0.06214	-0.15456	0.15639	-0.14652	-0.03634	0.645383
0.000228	1.14E-05	0.000429	0.487656	0.082738	0.079119	0.100233	0.685002	2.6E-16

KIAA1586	KIAA2026	KIF13B	KIF1A	KIF21B	KIF26A	KIFC2	KLC2	KLF13
cyan	cyan	cyan	cyan	cyan	cyan	cyan	cyan	cyan
-0.19904	0.069277	0.147829	0.032731	-0.08029	-0.27287	-0.08262	-0.07109	0.249479
0.024875	0.438976	0.097195	0.714881	0.369529	0.00191	0.355773	0.427088	0.004676
-0.12993	-0.03465	0.097243	0.097582	-0.06485	-0.33927	-0.04359	-0.0192	0.242259
0.145409	0.698957	0.276765	0.275083	0.468823	9.54E-05	0.626533	0.830362	0.006067
0.562485	-0.36049	-0.38132	-0.35754	-0.15883	0.130701	-0.30067	-0.33474	-0.42197
5.9E-12	3.14E-05	9.73E-06	3.68E-05	0.074502	0.143014	0.000593	0.00012	7.77E-07
0.590268	-0.34737	-0.35361	-0.58477	-0.57637	-0.27872	-0.61158	-0.68116	-0.45739
2.81E-13	6.3E-05	4.54E-05	5.25E-13	1.34E-12	0.001508	2.22E-14	1.23E-18	6.44E-08
0.182249	-0.45488	-0.56531	-0.28147	-0.38674	-0.37449	-0.29994	-0.30733	-0.48443
0.040292	7.76E-08	4.39E-12	0.001348	7.08E-06	1.44E-05	0.000613	0.00044	7.89E-09
0.551085	-0.51291	-0.57599	-0.68377	-0.80036	-0.59882	-0.76094	-0.80662	-0.58983
1.9E-11	7.04E-10	1.39E-12	8.06E-19	1.49E-29	1.04E-13	3.05E-25	2.51E-30	2.95E-13
0.188962	-0.48421	-0.36174	-0.35407	-0.47851	-0.57478	-0.49438	-0.49291	-0.29956
0.033366	8.03E-09	2.93E-05	4.43E-05	1.27E-08	1.59E-12	3.48E-09	3.93E-09	0.000623
-0.12497	0.005057	-0.00271	-0.12395	-0.36052	-0.44744	-0.24516	-0.32418	-0.20517
0.161542	0.955001	0.975894	0.165029	3.13E-05	1.33E-07	0.005469	0.000201	0.020671
-0.07658	-0.07274	0.030111	-0.57663	-0.32504	-0.037	-0.41326	-0.40639	-0.22809
0.392124	0.416397	0.736832	1.3E-12	0.000193	0.679644	1.37E-06	2.13E-06	0.009906
-0.44563	0.065993	0.273744	-0.41109	-0.40171	-0.28487	-0.46289	-0.53437	0.006458
1.52E-07	0.46103	0.001845	1.58E-06	2.85E-06	0.00117	4.27E-08	9.77E-11	0.942554
-0.20391	0.005342	0.171054	-0.37762	-0.41269	-0.21813	-0.46687	-0.552	-0.05197
0.021482	0.95247	0.054503	1.2E-05	1.43E-06	0.013754	3.15E-08	1.73E-11	0.561716
-0.34172	0.221136	0.394992	0.160523	0.274647	0.305364	0.247928	0.303926	0.218452
8.42E-05	0.012474	4.31E-06	0.071416	0.001779	0.000481	0.004948	0.000513	0.013611
-0.24285	0.231841	0.39742	0.203465	0.447124	0.547516	0.322992	0.281716	0.210202
0.005941	0.008723	3.72E-06	0.021773	1.37E-07	2.72E-11	0.000212	0.001334	0.017692
-0.37586	0.145931	0.267921	0.234162	0.35259	0.493028	0.468548	0.406579	0.025238
1.33E-05	0.101615	0.002324	0.008056	4.79E-05	3.89E-09	2.77E-08	2.1E-06	0.778215
-0.68923	0.36129	0.64312	0.064014	0.230914	0.338551	0.14852	0.066207	0.28294
3.3E-19	3E-05	3.56E-16	0.47461	0.009003	9.89E-05	0.095625	0.459574	0.001268
-0.49188	0.451355	0.568552	0.686799	0.47499	0.117004	0.325947	0.392289	0.665932
4.28E-09	1.01E-07	3.11E-12	4.93E-19	1.68E-08	0.190185	0.000184	5.08E-06	1.31E-17
0.039416	0.170103	0.101916	0.723612	0.760488	0.584449	0.623228	0.692129	0.310268
0.659957	0.05588	0.254225	7.29E-22	3.38E-25	5.44E-13	5.09E-15	2.04E-19	0.000385
0.576441	-0.07792	-0.36272	0.222913	0.086565	-0.04966	0.19252	0.288999	0.005751
1.32E-12	0.38391	2.78E-05	0.011767	0.333189	0.579238	0.030124	0.000983	0.948838
0.051731	0.131661	-0.08645	0.632877	0.535502	0.164038	0.609427	0.75449	0.310426
0.56353	0.140078	0.333862	1.43E-15	8.77E-11	0.065352	2.89E-14	1.29E-24	0.000382
0.30494	-0.14393	-0.31774	-0.01281	0.232883	0.272871	0.039525	0.082683	-0.10464
0.00049	0.106441	0.000272	0.886377	0.008418	0.00191	0.659073	0.3554	0.241675

KLF16	KLHL26	KPNA4	KRBOX4	KRCC1	KRT10	LACTB2	LAMTOR3	LAMTOR5
cyan	cyan	cyan	cyan	cyan	cyan	cyan	cyan	cyan
-0.10484	0.056374	-0.03299	0.093822	-0.02646	-0.10789	0.110469	-0.02834	0.049072
0.240792	0.529007	0.712718	0.294093	0.767764	0.227299	0.216308	0.751822	0.583776
-0.13968	0.060585	-0.03272	0.180891	-0.04876	-0.0496	0.146384	0.020326	0.150952
0.117305	0.498639	0.714935	0.04183	0.586174	0.579717	0.100547	0.820566	0.090257
-0.23044	-0.53877	0.246551	0.049234	0.174067	0.283344	0.03875	0.260426	0.088587
0.009149	6.4E-11	0.005201	0.582534	0.050323	0.001247	0.665354	0.003106	0.321977
-0.60925	-0.58438	0.607553	0.379055	0.523156	0.384534	0.294017	0.578966	0.126763
2.95E-14	5.49E-13	3.63E-14	1.11E-05	2.79E-10	8.06E-06	0.000793	1E-12	0.155562
-0.33946	-0.3978	0.000762	0.549529	0.538849	0.606002	0.366572	0.459806	0.870524
9.45E-05	3.64E-06	0.993212	2.22E-11	6.35E-11	4.38E-14	2.25E-05	5.38E-08	2.64E-40
-0.77934	-0.64566	0.584169	0.733913	0.832576	0.630219	0.5588	0.82515	0.615327
3.83E-27	2.5E-16	5.62E-13	9.71E-23	7.25E-34	2.04E-15	8.65E-12	8.55E-33	1.39E-14
-0.5143	-0.38427	0.314016	0.576937	0.44903	0.335876	0.27074	0.535308	0.419746
6.22E-10	8.19E-06	0.000324	1.25E-12	1.19E-07	0.000113	0.00208	8.93E-11	9E-07
-0.32219	-0.07094	0.160896	0.25613	0.420403	0.061326	0.106025	0.201536	0.171534
0.000221	0.428072	0.070751	0.003654	8.62E-07	0.493397	0.235476	0.02308	0.053819
-0.11107	-0.18215	0.080678	-0.20168	0.106443	0.284705	-0.06117	-0.08716	-0.04465
0.213807	0.040403	0.367228	0.02298	0.233623	0.001178	0.494462	0.32988	0.618186
-0.19803	-0.0651	-0.05869	-0.07962	0.372441	0.17772	0.169987	-0.1114	0.12784
0.025631	0.467099	0.512197	0.37355	1.62E-05	0.045615	0.056051	0.212425	0.152051
-0.29528	-0.08666	0.262922	0.033772	0.354584	0.09442	0.198397	0.086284	0.012236
0.000751	0.332666	0.002823	0.706222	4.31E-05	0.291015	0.025352	0.334767	0.891399
0.189611	0.28513	-0.18001	-0.43358	-0.46371	0.004966	-0.31644	-0.46269	-0.14906
0.032754	0.001157	0.042854	3.54E-07	4.01E-08	0.95581	0.00029	4.33E-08	0.094408
0.438788	0.169051	-0.36017	-0.67737	-0.54375	-0.28293	-0.49106	-0.63079	-0.50331
2.47E-07	0.057438	3.19E-05	2.25E-18	3.94E-11	0.001268	4.59E-09	1.89E-15	1.63E-09
0.63844	0.321418	-0.57694	-0.60755	-0.33423	-0.04161	-0.24761	-0.62264	-0.01929
6.77E-16	0.000229	1.25E-12	3.63E-14	0.000123	0.64231	0.005005	5.49E-15	0.829581
0.415301	0.320737	-0.51864	-0.72376	-0.27802	-0.16079	-0.30544	-0.77056	-0.28722
1.2E-06	0.000237	4.21E-10	7.08E-22	0.001552	0.070941	0.00048	3.25E-26	0.00106
0.226257	0.422571	-0.4299	-0.19546	-0.26692	-0.37193	-0.02014	-0.48464	-0.18839
0.010533	7.46E-07	4.56E-07	0.027652	0.002417	1.67E-05	0.822151	7.76E-09	0.033918
0.483848	0.229623	-0.26355	-0.3494	-0.60083	-0.38115	-0.30064	-0.34472	-0.31845
8.28E-09	0.009406	0.002755	5.67E-05	8.16E-14	9.83E-06	0.000594	7.23E-05	0.000264
-0.06601	0.009359	0.417502	0.408315	-0.03097	-0.20249	0.173293	0.486716	-0.10808
0.460901	0.916824	1.04E-06	1.89E-06	0.729648	0.022426	0.051371	6.55E-09	0.226453
0.3647	0.37458	-0.10352	0.063598	-0.47779	-0.44093	-0.18241	-0.01915	-0.21013
2.49E-05	1.43E-05	0.246806	0.477487	1.34E-08	2.12E-07	0.040117	0.830793	0.01773
0.038443	-0.44979	-0.22964	-0.01889	-0.19304	0.078985	-0.25063	-0.05601	0.116526
0.667845	1.13E-07	0.0094	0.833035	0.029673	0.3774	0.004482	0.531691	0.192014

LAPTM4A	LARP4B	LCMT2	LDB1	LEKR1	LETM1	LIMK1	LIMS2	LIN52
cyan	cyan	cyan	cyan	cyan	cyan	cyan	cyan	cyan
-0.00031	0.092876	-0.22991	0.185301	-0.10065	-0.30307	-0.07145	-0.04189	-0.10654
0.997239	0.299011	0.009316	0.037007	0.260197	0.000533	0.424689	0.640094	0.233205
-0.06332	0.105512	-0.17112	0.168707	-0.18477	-0.30173	-0.06658	-0.04481	-0.09069
0.479449	0.237762	0.054405	0.057954	0.037565	0.000566	0.457058	0.616925	0.310576
0.230021	-0.01036	0.440936	-0.33594	0.284358	0.105823	0.00593	-0.42788	0.298436
0.00928	0.908003	2.12E-07	0.000113	0.001195	0.236371	0.947248	5.23E-07	0.000655
0.525833	-0.30149	0.503744	-0.66715	0.424843	-0.2542	-0.2577	-0.61638	0.432175
2.18E-10	0.000572	1.57E-09	1.09E-17	6.41E-07	0.003928	0.003445	1.22E-14	3.9E-07
0.223481	-0.36284	0.328379	-0.18782	0.361619	-0.65142	-0.27444	-0.28506	0.248788
0.011549	2.76E-05	0.000164	0.034469	2.95E-05	1.11E-16	0.001794	0.001161	0.004795
0.642098	-0.54875	0.545253	-0.70644	0.563828	-0.74371	-0.45083	-0.67479	0.497717
4.1E-16	2.4E-11	3.4E-11	1.72E-20	5.13E-12	1.31E-23	1.04E-07	3.36E-18	2.63E-09
0.373035	-0.44471	0.256678	-0.39328	0.331987	-0.51897	-0.25333	-0.36329	0.272104
1.57E-05	1.62E-07	0.00358	4.79E-06	0.000137	4.09E-10	0.004057	2.69E-05	0.00197
0.27223	-0.36508	0.1379	-0.23123	0.435916	-0.34514	-0.50061	-0.13559	-0.08153
0.00196	2.44E-05	0.122084	0.008907	3.01E-07	7.07E-05	2.05E-09	0.128517	0.362199
0.622036	-0.14618	-0.03096	-0.38918	0.052432	0.098175	0.03526	0.016736	-0.20756
5.93E-15	0.101035	0.729665	6.12E-06	0.558252	0.272165	0.693908	0.851858	0.019208
0.563706	-0.17267	-0.17092	-0.21972	0.132728	-0.26193	-0.34117	0.185568	-0.38366
5.19E-12	0.052223	0.0547	0.013065	0.136864	0.002932	8.67E-05	0.03673	8.49E-06
0.510858	-0.29485	-0.00149	-0.32221	0.136028	-0.26227	-0.27084	-0.08694	-0.12644
8.44E-10	0.000765	0.98676	0.000221	0.127286	0.002894	0.002071	0.331067	0.156635
-0.23386	0.274593	-0.14871	0.139153	-0.45207	0.502917	0.351838	0.272016	-0.33861
0.008139	0.001783	0.095201	0.118696	9.54E-08	1.69E-09	4.99E-05	0.001977	9.86E-05
-0.21886	0.46836	-0.1872	0.280627	-0.23942	0.741565	0.285118	0.349557	-0.38255
0.013434	2.81E-08	0.035076	0.001395	0.006709	2.04E-23	0.001158	5.62E-05	9.06E-06
-0.20678	0.226553	-0.15371	0.361755	-0.21988	0.480536	0.255119	0.467671	-0.4437
0.019671	0.010429	0.084464	2.93E-05	0.012995	1.08E-08	0.003796	2.96E-08	1.74E-07
0.0857	0.273609	-0.39076	0.238143	-0.22335	0.472294	0.042627	0.62924	-0.7343
0.338061	0.001855	5.57E-06	0.007016	0.011599	2.07E-08	0.634184	2.32E-15	8.99E-23
-0.56235	0.324651	-0.39782	0.487774	-0.37213	0.137427	-0.0406	0.540895	-0.3617
5.98E-12	0.000196	3.63E-06	6.01E-09	1.65E-05	0.123382	0.650415	5.21E-11	2.94E-05
-0.72788	0.415843	-0.12946	0.509082	-0.33893	0.560958	0.406004	0.238919	0.00921
3.2E-22	1.16E-06	0.146894	9.87E-10	9.71E-05	6.92E-12	2.18E-06	0.006827	0.918147
-0.34865	-0.05335	0.170299	0.013063	-0.04481	-0.14085	0.170262	-0.39396	0.629832
5.89E-05	0.551354	0.055595	0.884112	0.61692	0.114234	0.055648	4.59E-06	2.15E-15
-0.70194	0.182811	-0.21076	0.495566	-0.34281	0.128266	0.35342	0.097495	0.214741
3.81E-20	0.03967	0.017384	3.15E-09	7.97E-05	0.15068	4.59E-05	0.275517	0.015332
-0.22279	0.488708	0.103366	0.184383	0.118558	0.086384	0.161088	-0.07707	0.163231
0.011815	5.57E-09	0.247494	0.037971	0.184323	0.334202	0.070412	0.389132	0.066706

LIN7A	LINGO3	LIPT1	LIPT2	LLGL1	LLPH	LMAN2L	LMF1	LPAR6
cyan	cyan	cyan	cyan	cyan	cyan	cyan	cyan	cyan
-0.11517	-0.42412	0.156716	-0.11064	-0.06188	0.094227	0.007769	-0.06624	0.129607
0.19726	6.73E-07	0.078488	0.215603	0.489507	0.292006	0.930916	0.459339	0.146421
-0.10478	-0.41384	0.175685	-0.10433	-0.08438	0.054263	-0.00854	0.016594	-0.00743
0.241072	1.32E-06	0.04819	0.2431	0.345559	0.544574	0.924065	0.8531	0.93396
0.203458	0.088102	0.222683	0.381258	-0.19964	0.17731	0.417153	-0.04949	0.018341
0.021777	0.324642	0.011857	9.77E-06	0.02443	0.046124	1.07E-06	0.58058	0.837836
0.476485	-0.28384	0.47474	0.385007	-0.43829	0.409105	0.624986	-0.45055	0.45986
1.49E-08	0.001221	1.71E-08	7.84E-06	2.56E-07	1.79E-06	4.05E-15	1.07E-07	5.36E-08
0.177333	-0.32384	0.56374	0.393825	-0.37057	0.298278	0.375494	-0.27622	0.266381
0.046095	0.000204	5.17E-12	4.63E-06	1.8E-05	0.000659	1.36E-05	0.00167	0.002468
0.575824	-0.60209	0.759288	0.512509	-0.63383	0.598893	0.722467	-0.67556	0.684326
1.42E-12	7.03E-14	4.43E-25	7.29E-10	1.26E-15	1.03E-13	9.06E-22	2.99E-18	7.37E-19
0.357415	-0.45538	0.394552	0.264786	-0.46019	0.294805	0.500787	-0.32468	0.403727
3.7E-05	7.48E-08	4.43E-06	0.002626	5.23E-08	0.000767	2.02E-09	0.000196	2.52E-06
0.162806	-0.30625	0.268423	0.160057	0.018005	0.158779	0.181019	-0.52905	0.397816
0.067429	0.000462	0.002279	0.072253	0.840767	0.074589	0.041683	1.61E-10	3.63E-06
0.124007	-0.22765	0.0623	0.040085	-0.20658	0.444643	0.306107	-0.24802	0.370376
0.16482	0.010052	0.486539	0.65455	0.019793	1.63E-07	0.000465	0.004931	1.82E-05
-0.04775	-0.52368	0.244267	-0.08637	-0.00633	0.486882	0.190921	-0.32105	0.685701
0.593946	2.66E-10	0.005647	0.334303	0.943695	6.47E-09	0.031546	0.000233	5.89E-19
0.069967	-0.4927	0.315833	0.056236	-0.07359	0.436305	0.287363	-0.38579	0.66649
0.43442	4E-09	0.000298	0.530019	0.410947	2.93E-07	0.001054	7.49E-06	1.21E-17
-0.50187	0.197129	-0.24219	-0.33965	0.177827	-0.16276	-0.07382	0.575606	-0.38647
1.84E-09	0.026323	0.006082	9.36E-05	0.045483	0.067515	0.409454	1.45E-12	7.19E-06
-0.44954	0.487641	-0.40817	-0.2071	0.454191	-0.28633	-0.23054	0.5035	-0.45129
1.15E-07	6.08E-09	1.9E-06	0.019482	8.16E-08	0.001101	0.009117	1.6E-09	1.01E-07
-0.51347	0.551155	-0.22609	-0.09669	0.565431	-0.18293	-0.29876	0.387294	-0.37216
6.69E-10	1.89E-11	0.010591	0.279518	4.33E-12	0.039539	0.000645	6.85E-06	1.64E-05
-0.56317	0.173818	-0.25936	-0.32495	0.57799	0.020993	-0.26187	0.298213	-0.01081
5.49E-12	0.050658	0.003234	0.000193	1.12E-12	0.814782	0.002939	0.000661	0.904023
-0.43427	0.154435	-0.3107	-0.46143	0.53348	-0.34548	-0.55613	0.535353	-0.25507
3.38E-07	0.082989	0.000378	4.76E-08	1.06E-10	6.95E-05	1.14E-11	8.89E-11	0.003802
-0.32114	0.725692	-0.54065	-0.23821	0.408959	-0.60875	-0.4675	0.721464	-0.74257
0.000232	4.89E-22	5.34E-11	0.007	1.81E-06	3.14E-14	3E-08	1.1E-21	1.66E-23
0.423782	0.138861	-0.08887	0.0804	-0.29917	-0.26063	-0.1007	-0.08382	-0.20833
6.89E-07	0.119478	0.320425	0.368885	0.000634	0.003082	0.259941	0.348811	0.018751
-0.00633	0.346749	-0.46705	-0.20767	0.040285	-0.58384	-0.4994	0.305434	-0.57698
0.943672	6.51E-05	3.11E-08	0.019141	0.652936	5.83E-13	2.28E-09	0.00048	1.25E-12
-0.0684	0.158828	-0.00223	0.086986	-0.2007	-0.1588	0.007812	0.413681	-0.39723
0.444827	0.074499	0.980122	0.330836	0.023671	0.074557	0.930542	1.34E-06	3.76E-06

LRFN1	LRIF1	LRP3	LRRC28	LRRC42	LRRC4B	LRRC8A	LRRCC1	LRRIQ3
cyan	cyan	cyan	cyan	cyan	cyan	cyan	cyan	cyan
-0.30491	-0.1835	-0.27324	-0.0289	-0.10826	-0.28976	-0.22913	-0.02678	0.093342
0.000491	0.038922	0.001883	0.747089	0.225686	0.000952	0.009564	0.765062	0.296583
-0.27717	-0.14226	-0.26478	-0.03769	-0.07701	-0.23509	-0.16774	-0.12063	0.055951
0.001607	0.110618	0.002627	0.67401	0.389461	0.007802	0.059426	0.176735	0.532105
0.043764	0.343139	0.012008	0.037613	0.535025	-0.04741	-0.07914	0.327734	0.185071
0.625157	7.84E-05	0.89341	0.674606	9.18E-11	0.596585	0.376477	0.000169	0.037246
-0.42921	0.534608	-0.51704	0.28698	0.565884	-0.51777	-0.38861	0.63328	0.45495
4.78E-07	9.55E-11	4.86E-10	0.001071	4.13E-12	4.55E-10	6.34E-06	1.36E-15	7.72E-08
-0.42736	0.251731	-0.29509	0.381452	0.584254	-0.30201	-0.4432	0.163615	0.42665
5.41E-07	0.004304	0.000757	9.66E-06	5.56E-13	0.000559	1.81E-07	0.066059	5.68E-07
-0.78873	0.594481	-0.76467	0.534081	0.751618	-0.76677	-0.67021	0.645359	0.704563
3.47E-28	1.73E-13	1.3E-25	1E-10	2.42E-24	7.96E-26	6.85E-18	2.61E-16	2.41E-20
-0.59588	0.257962	-0.65472	0.38352	0.44055	-0.55237	-0.30333	0.347315	0.384398
1.47E-13	0.003411	6.88E-17	8.56E-06	2.18E-07	1.67E-11	0.000527	6.32E-05	8.13E-06
-0.48774	0.065587	-0.38284	0.558779	0.090712	-0.39795	-0.32346	0.426945	0.50831
6.03E-09	0.463795	8.91E-06	8.67E-12	0.310458	3.6E-06	0.000208	5.57E-07	1.06E-09
-0.12025	0.135991	-0.20591	0.014295	0.158665	-0.28885	0.021168	0.311368	-0.07878
0.17808	0.127389	0.020205	0.873262	0.0748	0.00099	0.813266	0.000366	0.378641
-0.51232	-0.16763	-0.4148	0.095352	-0.01959	-0.4443	-0.19938	0.340946	0.169667
7.41E-10	0.059602	1.24E-06	0.286254	0.826933	1.67E-07	0.024622	8.76E-05	0.056522
-0.50065	-0.08114	-0.49497	0.097178	0.074724	-0.51182	-0.32754	0.394635	0.365115
2.05E-09	0.364488	3.31E-09	0.277086	0.403752	7.75E-10	0.000171	4.41E-06	2.44E-05
0.527052	-0.19123	0.232044	-0.47422	-0.1661	0.318815	0.421931	-0.31426	-0.46416
1.95E-10	0.031262	0.008663	1.78E-08	0.061992	0.000259	7.79E-07	0.000321	3.87E-08
0.637323	-0.30633	0.545476	-0.42612	-0.28164	0.52762	0.531394	-0.18425	-0.50136
7.88E-16	0.000461	3.33E-11	5.89E-07	0.001338	1.85E-10	1.3E-10	0.038116	1.93E-09
0.641629	-0.40073	0.747384	-0.30763	-0.24403	0.695328	0.399321	-0.32336	-0.43828
4.38E-16	3.03E-06	6.02E-24	0.000434	0.005695	1.19E-19	3.31E-06	0.000209	2.56E-07
0.30851	-0.53276	0.359244	-0.34337	-0.42458	0.338649	0.391852	-0.08362	-0.42451
0.000417	1.14E-10	3.36E-05	7.74E-05	6.53E-07	9.85E-05	5.22E-06	0.349944	6.56E-07
0.149809	-0.52108	0.220994	-0.34357	-0.63128	0.388525	0.174593	-0.37598	-0.26364
0.092749	3.37E-10	0.012532	7.66E-05	1.77E-15	6.37E-06	0.049621	1.32E-05	0.002745
0.734957	-0.25354	0.698869	-0.3886	-0.32987	0.796237	0.460661	-0.51921	-0.44187
7.88E-23	0.004025	6.48E-20	6.34E-06	0.000152	4.65E-29	5.05E-08	4E-10	1.99E-07
0.038977	0.316786	-0.00508	0.09879	0.013595	0.011614	-0.15315	-0.1697	0.147005
0.663509	0.000285	0.954814	0.269157	0.879428	0.896888	0.085613	0.056466	0.099095
0.379308	-0.10127	0.365723	-0.13588	-0.35259	0.376472	0.228235	-0.59755	-0.2504
1.09E-05	0.257241	2.36E-05	0.127703	4.79E-05	1.29E-05	0.009857	1.2E-13	0.00452
0.263037	0.222226	0.168754	-0.12437	0.305401	0.17042	0.013634	-0.11884	-0.18491
0.00281	0.012036	0.057884	0.163577	0.00048	0.055418	0.879077	0.183286	0.037414

LRSAM1	LSM14B	LSM5	LSM6	LSS	LTBP4	LTV1	LUZP2	LYPLAL1
cyan	cyan	cyan	cyan	cyan	cyan	cyan	cyan	cyan
0.162819	-0.19647	-0.146	-0.23799	0.006992	-0.04789	0.098476	-0.28808	0.248779
0.067405	0.026837	0.101447	0.007053	0.937812	0.592888	0.270689	0.001022	0.004797
0.168859	-0.12992	-0.08105	-0.25409	0.001393	-0.0818	0.053119	-0.29953	0.265322
0.057725	0.145446	0.365	0.003944	0.9876	0.360596	0.553101	0.000624	0.002572
-0.30849	0.098389	0.547085	0.417153	-0.18372	0.004842	0.342437	-0.06713	-0.2633
0.000418	0.271117	2.84E-11	1.07E-06	0.038675	0.956915	8.12E-05	0.453359	0.002782
-0.64458	-0.28725	0.712585	0.441997	-0.41523	-0.39099	0.588575	-0.34295	0.124243
2.91E-16	0.001059	5.71E-21	1.97E-07	1.21E-06	5.5E-06	3.41E-13	7.91E-05	0.164013
-0.33698	-0.45729	0.521911	0.505708	-0.3562	-0.31655	0.203977	-0.38474	0.544313
0.000107	6.49E-08	3.13E-10	1.32E-09	3.95E-05	0.000288	0.021436	7.97E-06	3.73E-11
-0.82458	-0.65187	0.871268	0.629133	-0.61588	-0.63601	0.668872	-0.61099	0.585818
1.03E-32	1.04E-16	1.88E-40	2.36E-15	1.3E-14	9.41E-16	8.4E-18	2.38E-14	4.66E-13
-0.48614	-0.42752	0.511363	0.251416	-0.26643	-0.42891	0.367913	-0.3753	0.271002
6.87E-09	5.36E-07	8.07E-10	0.004355	0.002464	4.87E-07	2.09E-05	1.38E-05	0.002058
-0.41986	-0.3697	0.154594	0.028348	-0.28595	-0.28262	0.126173	-0.18785	0.526863
8.93E-07	1.89E-05	0.082667	0.75172	0.001118	0.001285	0.15751	0.034439	1.98E-10
-0.24382	-0.13479	0.181054	0.156223	-0.05474	0.226834	0.440456	-0.19387	-0.2497
0.005738	0.130812	0.041643	0.079444	0.541059	0.010331	2.19E-07	0.028962	0.004639
-0.19098	-0.551	0.011513	0.069619	0.075135	0.031986	0.328526	-0.27414	0.330957
0.031494	1.92E-11	0.897779	0.436716	0.401165	0.721097	0.000163	0.001815	0.000144
-0.28761	-0.53639	0.156553	0.078807	-0.04153	-0.21139	0.421952	-0.31064	0.340478
0.001043	8.05E-11	0.078803	0.378482	0.642924	0.017044	7.78E-07	0.000379	8.98E-05
0.535684	0.32811	-0.35368	-0.31695	0.417516	0.245712	-0.11477	0.286843	-0.45949
8.62E-11	0.000166	4.52E-05	0.000283	1.04E-06	0.005361	0.198857	0.001077	5.51E-08
0.516927	0.584215	-0.37711	-0.27169	0.532431	0.640762	-0.23615	0.410504	-0.69303
4.91E-10	5.59E-13	1.24E-05	0.002003	1.17E-10	4.93E-16	0.007521	1.64E-06	1.75E-19
0.526738	0.356198	-0.35185	-0.01452	0.457136	0.601478	-0.42407	0.274808	-0.29804
2E-10	3.95E-05	4.98E-05	0.871288	6.57E-08	7.56E-14	6.75E-07	0.001768	0.000666
0.44175	0.155359	-0.48549	-0.25046	0.619162	0.64344	-0.15297	0.201701	-0.27561
2E-07	0.081142	7.24E-09	0.004511	8.56E-15	3.41E-16	0.085995	0.022966	0.001711
0.455615	0.112553	-0.55988	-0.51838	0.497687	0.133061	-0.36269	0.283673	0.144834
7.35E-08	0.207714	7.74E-12	4.31E-10	2.63E-09	0.135872	2.78E-05	0.00123	0.10424
0.554621	0.603821	-0.3889	-0.32242	0.288806	0.291778	-0.523	0.627529	-0.46861
1.33E-11	5.71E-14	6.23E-06	0.000218	0.000991	0.000874	2.83E-10	2.91E-15	2.76E-08
-0.16653	0.126765	0.115637	0.020064	-0.43799	-0.46763	-0.14573	0.052919	0.057951
0.061319	0.155556	0.195448	0.822836	2.61E-07	2.97E-08	0.102088	0.554598	0.517526
0.361415	0.376496	-0.41225	-0.3586	-0.03343	-0.07617	-0.54745	0.361966	-0.15701
2.98E-05	1.29E-05	1.47E-06	3.48E-05	0.709077	0.39466	2.73E-11	2.9E-05	0.077915
0.213596	0.361038	0.089979	0.10508	0.069645	0.327465	0.057307	0.125289	-0.42945
0.0159	3.05E-05	0.3144	0.239698	0.43654	0.000171	0.522201	0.160463	4.7E-07

LYRM2	LYSMD3	LZIC	MACC1	MAFG	MAFK	MAGOH	MAGOHB	MAGT1
cyan	cyan	cyan	cyan	cyan	cyan	cyan	cyan	cyan
0.098457	-0.06981	-0.11725	0.169504	0.109461	0.078201	0.024369	-0.19014	-0.06404
0.270783	0.435468	0.189237	0.056762	0.220557	0.382169	0.785661	0.032259	0.474434
0.132702	-0.16685	-0.10008	0.064637	0.000852	0.026778	-0.00732	-0.17844	-0.11285
0.136943	0.06081	0.262934	0.470312	0.992411	0.765061	0.934932	0.044726	0.206512
0.09954	0.293999	0.466777	0.235248	-0.3882	-0.40326	0.382166	0.555047	0.286317
0.265523	0.000794	3.17E-08	0.007759	6.49E-06	2.59E-06	9.26E-06	1.27E-11	0.001101
0.453151	0.691461	0.689056	0.533382	-0.56178	-0.48237	0.420676	0.724633	0.666804
8.81E-08	2.28E-19	3.4E-19	1.07E-10	6.35E-12	9.33E-09	8.46E-07	5.99E-22	1.15E-17
0.478856	0.099644	0.331558	0.144006	-0.41638	-0.46896	0.532697	0.402562	0.159937
1.24E-08	0.265018	0.00014	0.106258	1.12E-06	2.68E-08	1.15E-10	2.71E-06	0.07247
0.762878	0.727924	0.756902	0.601994	-0.6975	-0.65749	0.650005	0.824415	0.731707
1.96E-25	3.17E-22	7.57E-25	7.11E-14	8.2E-20	4.6E-17	1.35E-16	1.09E-32	1.51E-22
0.478043	0.336856	0.416986	0.401588	-0.45119	-0.3945	0.308276	0.477915	0.394953
1.32E-08	0.000108	1.08E-06	2.88E-06	1.02E-07	4.45E-06	0.000422	1.33E-08	4.32E-06
0.378494	0.226808	0.344478	0.274535	-0.13266	-0.23543	0.208519	0.143991	0.23596
1.15E-05	0.01034	7.32E-05	0.001787	0.137063	0.007711	0.018644	0.106294	0.00757
-0.19598	0.327536	-0.01234	0.493129	0.021468	0.211469	0.348313	0.346819	0.412898
0.027232	0.000171	0.890469	3.86E-09	0.810662	0.017004	6E-05	6.48E-05	1.41E-06
0.015668	0.179574	-0.0874	0.535585	0.107563	0.195615	0.32876	0.121953	0.361162
0.861212	0.043368	0.328522	8.7E-11	0.228714	0.027524	0.000161	0.171983	3.03E-05
0.192865	0.331338	0.141667	0.5364	-0.0914	0.046805	0.269531	0.270507	0.441778
0.029824	0.000142	0.112117	8.04E-11	0.306763	0.601298	0.002181	0.002099	2E-07
-0.44818	-0.46851	-0.43189	-0.26074	0.176867	0.514075	-0.3507	-0.38408	-0.27971
1.27E-07	2.78E-08	3.98E-07	0.00307	0.04668	6.34E-10	5.29E-05	8.28E-06	0.001448
-0.68084	-0.41116	-0.33728	-0.20234	0.379663	0.414419	-0.20334	-0.27074	-0.33544
1.29E-18	1.57E-06	0.000105	0.022525	1.07E-05	1.27E-06	0.021857	0.002079	0.000116
-0.60242	-0.51467	-0.43048	-0.27263	0.449702	0.457139	-0.02108	-0.25962	-0.36076
6.75E-14	6.01E-10	4.38E-07	0.001929	1.13E-07	6.57E-08	0.814066	0.003203	3.09E-05
-0.66434	-0.40368	-0.51845	0.040704	0.562379	0.651039	-0.02536	-0.30695	-0.17041
1.67E-17	2.52E-06	4.28E-10	0.649568	5.97E-12	1.17E-16	0.77719	0.000448	0.055428
-0.17629	-0.555	-0.39983	-0.42716	0.339135	0.323538	-0.46721	-0.59346	-0.45617
0.047416	1.28E-11	3.21E-06	5.49E-07	9.61E-05	0.000207	3.07E-08	1.94E-13	7.06E-08
-0.35238	-0.55479	-0.25877	-0.64988	0.17526	0.103382	-0.54255	-0.48734	-0.59039
4.85E-05	1.31E-11	0.003308	1.38E-16	0.048743	0.247419	4.43E-11	6.23E-09	2.77E-13
0.384323	0.272333	0.229777	-0.25707	-0.25502	-0.36895	-0.33024	-0.02046	-0.02879
8.16E-06	0.001952	0.009357	0.003528	0.00381	1.97E-05	0.00015	0.819406	0.748013
-0.04366	-0.33973	-0.27486	-0.64253	0.151217	0.010752	-0.6035	-0.56102	-0.55899
0.62596	9.32E-05	0.001764	3.87E-16	0.089686	0.904501	5.93E-14	6.88E-12	8.49E-12
-0.20248	-0.16776	0.021009	-0.14944	-0.02875	-0.19161	0.158454	0.028566	-0.31753
0.022432	0.059399	0.814641	0.093571	0.748329	0.030926	0.075192	0.749879	0.000275

MALSU1	MAMDC2	MAP1LC3(	MAP2K7	MAP3K10	MAP3K12	MAP3K2	MAP3K3	MAP3K7
cyan	cyan	cyan	cyan	cyan	cyan	cyan	cyan	cyan
-0.12847	0.165193	0.026214	-0.08964	-0.05388	0.15526	0.062702	0.189118	-0.108
0.150022	0.063455	0.769868	0.316238	0.547397	0.081337	0.483729	0.033218	0.226819
-0.09317	0.136313	0.017806	-0.11442	-0.01607	0.177358	-0.04529	0.116218	-0.09239
0.297472	0.126481	0.8425	0.200226	0.857697	0.046064	0.613158	0.193197	0.301562
0.38041	0.005309	0.16741	-0.06643	-0.32612	-0.24048	0.140615	-0.32942	0.356509
1.03E-05	0.952758	0.059936	0.458041	0.000183	0.006462	0.114834	0.000156	3.89E-05
0.499505	0.363885	0.544773	-0.48751	-0.70157	-0.35414	0.651492	-0.43795	0.75385
2.26E-09	2.61E-05	3.56E-11	6.15E-09	4.06E-20	4.41E-05	1.1E-16	2.62E-07	1.49E-24
0.5972	0.140285	0.272474	-0.36978	-0.36081	-0.4385	-0.06327	-0.27664	0.204676
1.25E-13	0.115698	0.001941	1.88E-05	3.08E-05	2.52E-07	0.479738	0.001641	0.020984
0.724688	0.499932	0.690306	-0.79046	-0.88756	-0.59714	0.624331	-0.50494	0.816829
5.93E-22	2.18E-09	2.76E-19	2.2E-28	6.78E-44	1.26E-13	4.41E-15	1.42E-09	1.19E-31
0.382195	0.441154	0.5002	-0.46701	-0.55954	-0.1845	0.333941	-0.22463	0.517494
9.25E-06	2.09E-07	2.13E-09	3.12E-08	8.02E-12	0.037849	0.000125	0.011117	4.67E-10
0.070852	0.343548	0.372207	-0.41805	-0.40104	0.030398	0.367107	-0.20491	0.275328
0.428618	7.67E-05	1.64E-05	1.01E-06	2.98E-06	0.734414	2.18E-05	0.020838	0.001731
0.236366	0.259534	0.351678	0.05539	-0.39834	-0.08887	0.260709	0.301038	0.152327
0.007464	0.003213	5.03E-05	0.536234	3.52E-06	0.32044	0.003073	0.000584	0.087329
0.100224	0.377724	0.280178	-0.1195	-0.45594	-0.12486	0.351286	0.447507	0.054133
0.262233	1.2E-05	0.001421	0.180841	7.18E-08	0.161927	5.13E-05	1.33E-07	0.545535
0.189657	0.447415	0.387056	-0.28568	-0.5042	-0.17381	0.514151	0.103863	0.225779
0.03271	1.34E-07	6.95E-06	0.001131	1.51E-09	0.050673	6.3E-10	0.245213	0.010702
-0.14016	-0.23721	-0.25684	0.484624	0.365401	0.337449	-0.3725	0.313005	-0.40132
0.116018	0.007249	0.003559	7.77E-09	2.4E-05	0.000105	1.61E-05	0.00034	2.92E-06
-0.32793	-0.36187	-0.38288	0.745547	0.410838	0.43737	-0.31368	0.35046	-0.43333
0.000167	2.91E-05	8.89E-06	8.89E-24	1.6E-06	2.72E-07	0.000329	5.36E-05	3.61E-07
-0.06666	-0.44217	-0.36313	0.73988	0.530545	0.174714	-0.51757	0.297525	-0.63604
0.456522	1.94E-07	2.72E-05	2.89E-23	1.4E-10	0.049461	4.64E-10	0.000681	9.38E-16
-0.2762	-0.16438	-0.24041	0.669144	0.238715	0.302399	-0.18683	0.678302	-0.53483
0.001671	0.064782	0.006477	8.06E-18	0.006876	0.000549	0.035445	1.94E-18	9.35E-11
-0.50623	-0.19407	-0.50981	0.281445	0.517734	0.286821	-0.35977	0.353161	-0.43583
1.27E-09	0.028795	9.25E-10	0.001349	4.57E-10	0.001078	3.26E-05	4.65E-05	3.03E-07
-0.39568	-0.50986	-0.58668	0.47368	0.751425	0.275776	-0.60516	-0.06649	-0.47509
4.14E-06	9.21E-10	4.23E-13	1.86E-08	2.52E-24	0.0017	4.86E-14	0.457625	1.66E-08
-0.08286	-0.00817	-0.02902	-0.4251	0.150241	-0.1271	0.082244	-0.52859	0.303397
0.354392	0.927351	0.746024	6.3E-07	0.091802	0.154451	0.357973	1.69E-10	0.000525
-0.44233	-0.28907	-0.42502	0.041708	0.647128	0.229983	-0.4591	-0.14079	-0.31896
1.92E-07	0.00098	6.34E-07	0.641517	2.03E-16	0.009292	5.67E-08	0.114389	0.000257
0.041088	-0.28748	-0.32652	0.279574	0.093237	0.073624	-0.31812	0.056888	-0.06863
0.646482	0.001049	0.000179	0.001456	0.297127	0.410727	0.000268	0.525254	0.443273

MAP3K7CI	MAP4	MAPK8IP3	MAPKAPK	MAPT	44263	MARCKSL1	MARK2	MARK4
cyan	cyan	cyan	cyan	cyan	cyan	cyan	cyan	cyan
-0.00534	0.173103	-0.02939	-0.16791	0.032988	0.166779	-0.18767	-0.02861	-0.22325
0.952498	0.051631	0.742927	0.059167	0.712736	0.060921	0.034614	0.749532	0.011636
-0.10755	0.123	-0.00624	-0.17119	0.078406	0.139456	-0.11305	-0.0531	-0.2051
0.228751	0.168305	0.94453	0.054303	0.380921	0.117889	0.205716	0.55321	0.020716
0.207939	-0.34674	-0.3584	0.421459	-0.25801	-0.09035	0.298903	-0.20086	-0.01146
0.018982	6.51E-05	3.51E-05	8.04E-07	0.003405	0.31242	0.000641	0.023552	0.898228
0.464276	-0.33278	-0.72257	0.570397	-0.54648	-0.28399	-0.21034	-0.56168	-0.46441
3.84E-08	0.000132	8.89E-22	2.55E-12	3.01E-11	0.001213	0.017618	6.42E-12	3.8E-08
0.256305	-0.46091	-0.40448	0.420223	-0.33893	-0.27953	-0.09578	-0.51752	-0.48274
0.00363	4.95E-08	2.4E-06	8.72E-07	9.71E-05	0.001459	0.284074	4.66E-10	9.05E-09
0.590365	-0.52785	-0.91272	0.707882	-0.74292	-0.44874	-0.439	-0.85434	-0.83386
2.78E-13	1.81E-10	2.01E-50	1.33E-20	1.54E-23	1.22E-07	2.43E-07	2.46E-37	4.67E-34
0.450009	-0.27332	-0.54163	0.452562	-0.41622	-0.25368	-0.25498	-0.57187	-0.53822
1.11E-07	0.001876	4.85E-11	9.2E-08	1.13E-06	0.004004	0.003815	2.18E-12	6.75E-11
0.425234	0.138529	-0.31978	0.118035	-0.21295	-0.24004	-0.45577	-0.39289	-0.51106
6.25E-07	0.120373	0.000247	0.18628	0.016229	0.006564	7.27E-08	4.9E-06	8.29E-10
0.537617	-0.0162	-0.33141	0.216245	-0.57933	-0.20813	-0.18925	-0.09778	-0.11049
7.16E-11	0.856588	0.000141	0.014613	9.64E-13	0.018871	0.033098	0.274121	0.216236
0.520919	0.22959	-0.31892	0.070176	-0.5378	0.00719	-0.3851	-0.2392	-0.50146
3.42E-10	0.009417	0.000258	0.433046	7.03E-11	0.93606	7.8E-06	0.006759	1.91E-09
0.430263	0.227667	-0.46418	0.157984	-0.48297	-0.10913	-0.44144	-0.29484	-0.52972
4.45E-07	0.010047	3.87E-08	0.076073	8.88E-09	0.221967	2.05E-07	0.000766	1.52E-10
-0.3124	0.39878	0.289348	-0.31375	0.269035	0.211777	0.246477	0.447772	0.506958
0.000349	3.42E-06	0.000969	0.000328	0.002225	0.01684	0.005215	1.3E-07	1.19E-09
-0.20801	0.385197	0.467357	-0.2949	0.329058	0.402218	0.529705	0.586639	0.647173
0.018943	7.76E-06	3.04E-08	0.000764	0.000158	2.77E-06	1.52E-10	4.25E-13	2.02E-16
-0.23752	0.215917	0.559374	-0.19913	0.270946	0.179461	0.505982	0.511099	0.551324
0.007171	0.014767	8.16E-12	0.024808	0.002063	0.043503	1.29E-09	8.26E-10	1.86E-11
0.046395	0.571624	0.395945	-0.32284	0.041243	0.372378	0.258939	0.459075	0.29581
0.604488	2.24E-12	4.07E-06	0.000214	0.645244	1.62E-05	0.003287	5.68E-08	0.000734
-0.44471	0.481072	0.577778	-0.55152	0.604376	0.502886	0.347249	0.337996	0.160817
1.62E-07	1.03E-08	1.14E-12	1.82E-11	5.34E-14	1.69E-09	6.34E-05	0.000102	0.070891
-0.63624	0.140785	0.654023	-0.37358	0.821613	0.338603	0.705587	0.530599	0.737633
9.13E-16	0.114393	7.62E-17	1.52E-05	2.66E-32	9.87E-05	2.01E-20	1.4E-10	4.58E-23
-0.34652	-0.39198	0.000684	0.028076	0.262447	-0.23761	-0.13551	-0.10488	0.04262
6.58E-05	5.18E-06	0.993914	0.754024	0.002875	0.007149	0.128757	0.2406	0.634234
-0.6146	-0.06892	0.523261	-0.40108	0.662203	0.019083	0.072974	0.32866	0.4357
1.52E-14	0.441325	2.76E-10	2.97E-06	2.3E-17	0.831366	0.414882	0.000162	3.06E-07
-0.09495	-0.30083	0.128422	0.041885	0.127248	0.353466	0.527573	0.151278	0.296715
0.288283	0.000589	0.150179	0.640101	0.153973	4.57E-05	1.85E-10	0.089557	0.000706

MBD4	MBIP	MBOAT7	MCEE	MCF2L	MCM9	MCMBP	MCPH1	MCTS1
cyan	cyan	cyan	cyan	cyan	cyan	cyan	cyan	cyan
0.207606	0.086909	0.035973	0.140889	-0.08206	-0.17885	0.035955	-0.12309	0.210815
0.019178	0.331263	0.688043	0.114123	0.35907	0.044238	0.688185	0.168003	0.017356
0.170821	0.129498	0.093612	0.178915	-0.07029	-0.23803	0.028216	-0.12391	0.240594
0.054838	0.146764	0.295183	0.044157	0.432316	0.007044	0.752844	0.165145	0.006436
-0.01793	0.196681	-0.40871	0.16643	-0.25703	0.40265	0.404293	0.54032	0.052181
0.841419	0.026673	1.84E-06	0.061472	0.003533	2.69E-06	2.43E-06	5.51E-11	0.560141
0.373061	0.414512	-0.68351	0.457972	-0.5763	0.762636	0.599358	0.698513	0.306756
1.56E-05	1.27E-06	8.41E-19	6.17E-08	1.35E-12	2.07E-25	9.73E-14	6.89E-20	0.000452
0.302318	0.472627	-0.25298	0.701436	-0.43264	-0.0742	0.34489	0.267308	0.48619
0.000551	2.02E-08	0.00411	4.16E-20	3.78E-07	0.407036	7.16E-05	0.002381	6.84E-09
0.633668	0.657529	-0.7383	0.855863	-0.82239	0.644398	0.72137	0.69457	0.615604
1.29E-15	4.58E-17	4E-23	1.34E-37	2.08E-32	2.98E-16	1.12E-21	1.35E-19	1.34E-14
0.439123	0.292882	-0.37423	0.533657	-0.50821	0.322885	0.37579	0.464094	0.493559
2.41E-07	0.000833	1.46E-05	1.05E-10	1.07E-09	0.000214	1.34E-05	3.89E-08	3.73E-09
0.332357	0.036075	-0.21218	0.224409	-0.32651	0.30058	0.131727	-0.05093	0.261951
0.000135	0.687199	0.01663	0.0112	0.000179	0.000596	0.139875	0.569573	0.00293
0.22379	-0.20526	-0.16913	0.026593	-0.34268	0.333573	0.380464	0.116598	-0.1918
0.011432	0.020611	0.057319	0.766634	8.02E-05	0.000127	1.02E-05	0.191738	0.03076
0.509229	-0.10151	-0.23089	0.165342	-0.33405	0.209532	0.335631	-0.19306	-0.01554
9.74E-10	0.256136	0.00901	0.063213	0.000124	0.018066	0.000115	0.029658	0.86234
0.467667	0.035952	-0.36917	0.221626	-0.41735	0.368068	0.294692	-0.00515	0.073257
2.96E-08	0.688214	1.95E-05	0.012275	1.05E-06	2.07E-05	0.00077	0.95416	0.413071
-0.38616	-0.35083	0.222835	-0.36052	0.365677	-0.23567	-0.26934	-0.17283	-0.41346
7.33E-06	5.26E-05	0.011798	3.13E-05	2.36E-05	0.007646	0.002198	0.052012	1.36E-06
-0.44809	-0.5387	0.286201	-0.61402	0.46606	-0.15492	-0.23773	-0.24547	-0.55624
1.27E-07	6.45E-11	0.001107	1.63E-14	3.35E-08	0.082014	0.007118	0.005408	1.13E-11
-0.45778	-0.43653	0.495223	-0.38507	0.431709	-0.50629	-0.28266	-0.4451	-0.57302
6.26E-08	2.89E-07	3.24E-09	7.81E-06	4.03E-07	1.26E-09	0.001283	1.58E-07	1.92E-12
-0.14474	-0.63824	0.295313	-0.4883	0.327274	-0.21455	-0.14157	-0.5696	-0.59244
0.104458	6.96E-16	0.00075	5.76E-09	0.000173	0.015427	0.112373	2.78E-12	2.19E-13
-0.15729	-0.23989	0.294	-0.32117	0.627844	-0.47567	-0.42985	-0.54996	-0.10511
0.077379	0.006598	0.000794	0.000232	2.79E-15	1.59E-08	4.57E-07	2.13E-11	0.239564
-0.70457	-0.19961	0.384925	-0.5248	0.717188	-0.49088	-0.54991	-0.1673	-0.32173
2.4E-20	0.024452	7.88E-06	2.4E-10	2.45E-21	4.66E-09	2.14E-11	0.060102	0.000226
-0.12107	0.42167	-0.01573	0.102795	0.050425	0.050047	-0.17326	0.311043	0.300538
0.17512	7.92E-07	0.860654	0.25013	0.573435	0.576314	0.051422	0.000372	0.000597
-0.4132	0.014623	0.482801	-0.29919	0.46215	-0.51846	-0.58944	-0.20628	0.041104
1.38E-06	0.870383	9.01E-09	0.000633	4.51E-08	4.28E-10	3.09E-13	0.019978	0.646355
-0.14022	0.052837	-0.09042	0.039561	0.18443	-0.15222	0.063836	0.332194	0.163837
0.115856	0.55521	0.312015	0.658782	0.037921	0.087554	0.475837	0.000136	0.065688

MCUR1	MED14OS	MED18	MED20	MED28	MED30	MED7	MED8	MEGF8
cyan	cyan	cyan	cyan	cyan	cyan	cyan	cyan	cyan
0.017912	-0.08596	-0.02304	-0.13318	-0.04853	-0.02512	0.195464	0.230155	-0.29085
0.841573	0.336592	0.797097	0.135519	0.58794	0.779202	0.027646	0.009238	0.000909
0.063862	-0.10389	-0.04984	-0.12208	-0.01047	0.008509	0.221694	0.307539	-0.28177
0.47566	0.24508	0.577873	0.171544	0.906987	0.924355	0.012248	0.000436	0.00133
0.189517	0.323981	0.264713	0.698415	0.421254	0.299991	-0.00631	0.020764	0.020424
0.032842	0.000203	0.002634	7.01E-20	8.15E-07	0.000611	0.943883	0.81676	0.819711
0.398301	0.493797	0.44849	0.744269	0.513032	0.368046	0.339588	0.236548	-0.33164
3.52E-06	3.65E-09	1.24E-07	1.16E-23	6.96E-10	2.07E-05	9.39E-05	0.007417	0.00014
0.246747	0.519539	0.566907	0.176947	0.455675	0.661167	0.719736	0.553525	-0.62762
0.005165	3.88E-10	3.7E-12	0.046579	7.32E-08	2.68E-17	1.52E-21	1.49E-11	2.87E-15
0.524463	0.735272	0.720625	0.648672	0.647831	0.676866	0.825272	0.607581	-0.79552
2.47E-10	7.39E-23	1.29E-21	1.64E-16	1.84E-16	2.43E-18	8.22E-33	3.62E-14	5.66E-29
0.348031	0.318978	0.367688	0.32697	0.436408	0.270967	0.512842	0.517302	-0.41313
6.09E-05	0.000257	2.11E-05	0.000175	2.91E-07	0.002061	7.08E-10	4.75E-10	1.39E-06
0.148962	0.167123	0.178377	0.107005	0.083517	0.020791	0.344402	-0.02685	-0.29518
0.094631	0.060383	0.044807	0.231151	0.350555	0.816527	7.34E-05	0.764436	0.000754
-0.20189	0.155073	0.189494	0.323481	0.037211	-0.0015	-0.12317	0.271704	0.04341
0.022836	0.081709	0.032864	0.000208	0.67789	0.986603	0.167698	0.002001	0.62796
-0.24806	0.211157	0.206777	0.0237	-0.06069	0.090354	0.160186	0.268072	-0.36527
0.004924	0.017171	0.019675	0.791409	0.497899	0.31238	0.07202	0.00231	2.42E-05
0.05713	0.2127	0.183099	0.221573	-0.03176	0.035493	0.213371	0.191569	-0.44616
0.523489	0.016357	0.039353	0.012297	0.723025	0.691993	0.016014	0.030962	1.46E-07
-0.35617	-0.30185	-0.34791	-0.13506	-0.29011	-0.26121	-0.48171	0.036045	0.39611
3.96E-05	0.000563	6.13E-05	0.130029	0.000938	0.003015	9.83E-09	0.687448	4.03E-06
-0.44795	-0.39662	-0.39915	0.009447	-0.26457	-0.37864	-0.76622	-0.46119	0.737423
1.29E-07	3.91E-06	3.34E-06	0.916049	0.002649	1.14E-05	9.06E-26	4.85E-08	4.78E-23
-0.48017	-0.20476	-0.12087	-0.29418	-0.34461	-0.07427	-0.46507	-0.35229	0.468708
1.11E-08	0.020929	0.175839	0.000788	7.27E-05	0.406607	3.62E-08	4.87E-05	2.74E-08
-0.63687	-0.30358	-0.25687	-0.21734	-0.45462	-0.30578	-0.57948	-0.27815	0.420458
8.39E-16	0.000521	0.003554	0.014107	7.91E-08	0.000472	9.48E-13	0.001544	8.59E-07
-0.16093	-0.48939	-0.39836	-0.55684	-0.44027	-0.34591	-0.23916	-0.22686	0.124402
0.070696	5.26E-09	3.51E-06	1.06E-11	2.22E-07	6.79E-05	0.006769	0.010321	0.163471
-0.07639	-0.49166	-0.49037	-0.28585	-0.22997	-0.32641	-0.56366	-0.52798	0.59166
0.393353	4.36E-09	4.86E-09	0.001123	0.009295	0.00018	5.22E-12	1.78E-10	2.39E-13
0.484704	0.010883	-0.03714	-0.07943	0.135477	-0.01247	0.22561	-0.10214	-0.10775
7.72E-09	0.903343	0.678434	0.374696	0.128846	0.889327	0.010762	0.253196	0.22792
0.103545	-0.40508	-0.4187	-0.55189	-0.21991	-0.32781	-0.13067	-0.25823	0.24351
0.246673	2.31E-06	9.64E-07	1.75E-11	0.012984	0.000168	0.143099	0.003377	0.005803
-0.13198	-0.00362	0.037695	0.202318	0.365534	0.228641	-0.0893	0.085346	0.143076
0.139111	0.967753	0.673936	0.022542	2.38E-05	0.009723	0.318093	0.340069	0.108558

METTL15	METTL18	METTL23	METTL25	METTL2A	METTL2B	METTL4	METTL6	MFN2
cyan	cyan	cyan	cyan	cyan	cyan	cyan	cyan	cyan
-0.03017	0.035735	0.014087	-0.00547	-0.24161	-0.21305	-0.13327	0.002898	0.079009
0.736342	0.689996	0.875095	0.951375	0.006209	0.01618	0.135266	0.974202	0.377253
-0.02511	0.038112	0.032823	0.028739	-0.22182	-0.16948	-0.10975	0.058041	0.087056
0.779294	0.670535	0.714114	0.748407	0.012196	0.056802	0.219325	0.516875	0.330442
0.14159	0.321952	0.280401	0.289922	0.56875	0.56505	0.527508	0.307932	-0.27841
0.112316	0.000223	0.001408	0.000946	3.04E-12	4.51E-12	1.86E-10	0.000428	0.001527
0.528071	0.502876	0.474459	0.615001	0.569478	0.670847	0.772887	0.573865	-0.51555
1.77E-10	1.69E-09	1.75E-08	1.45E-14	2.82E-12	6.21E-18	1.86E-26	1.75E-12	5.56E-10
0.069882	0.453548	0.564466	0.496764	0.34952	0.254377	0.237815	0.446534	-0.43567
0.434981	8.56E-08	4.79E-12	2.85E-09	5.63E-05	0.003902	0.007096	1.42E-07	3.07E-07
0.587639	0.711811	0.726843	0.846566	0.581687	0.652591	0.771528	0.789527	-0.72618
3.79E-13	6.57E-21	3.91E-22	4.92E-36	7.42E-13	9.36E-17	2.58E-26	2.81E-28	4.45E-22
0.320817	0.369552	0.43569	0.546361	0.255429	0.363438	0.487687	0.579543	-0.36234
0.000236	1.9E-05	3.06E-07	3.05E-11	0.003752	2.67E-05	6.05E-09	9.41E-13	2.84E-05
0.3308	0.331689	0.172698	0.217132	-0.06559	0.036022	0.099236	0.180191	-0.11878
0.000146	0.000139	0.052188	0.014203	0.463803	0.68764	0.266991	0.042643	0.183512
0.008603	0.218736	0.34912	0.166641	0.086951	0.078028	0.115311	0.020515	-0.29906
0.923521	0.013487	5.75E-05	0.061138	0.331029	0.383226	0.196722	0.818925	0.000637
-0.03739	0.366389	0.349085	0.186243	-0.24782	-0.24012	-0.13499	-0.03982	-0.27437
0.676439	2.27E-05	5.76E-05	0.03604	0.004968	0.006544	0.130243	0.65667	0.001799
0.133989	0.334496	0.29155	0.241437	-0.11017	-0.01694	0.066679	0.148178	-0.22101
0.133141	0.000121	0.000882	0.006247	0.217571	0.850059	0.45637	0.0964	0.012526
-0.40171	-0.48783	-0.06668	-0.26969	-0.23384	-0.38877	-0.33733	-0.34592	0.21267
2.85E-06	5.98E-09	0.456338	0.002168	0.008144	6.27E-06	0.000105	6.79E-05	0.016373
-0.39992	-0.26039	-0.27991	-0.44551	-0.15347	-0.22372	-0.3125	-0.49627	0.358028
3.19E-06	0.00311	0.001437	1.53E-07	0.084949	0.011459	0.000348	2.97E-09	3.58E-05
-0.55396	-0.14117	-0.12743	-0.38637	-0.13534	-0.34475	-0.54435	-0.54787	0.241804
1.42E-11	0.113403	0.153379	7.24E-06	0.129243	7.21E-05	3.72E-11	2.62E-11	0.006166
-0.50037	-0.07096	-0.07968	-0.38737	-0.45273	-0.54886	-0.60663	-0.61531	0.225309
2.1E-09	0.427891	0.373231	6.82E-06	9.09E-08	2.38E-11	4.06E-14	1.39E-14	0.010871
-0.33844	-0.38025	-0.40825	-0.44602	-0.54938	-0.54005	-0.50715	-0.40432	0.491067
9.95E-05	1.04E-05	1.89E-06	1.48E-07	2.25E-11	5.66E-11	1.17E-09	2.43E-06	4.58E-09
-0.43025	-0.5965	-0.59217	-0.56912	-0.07567	-0.11849	-0.29377	-0.34611	0.548943
4.45E-07	1.36E-13	2.25E-13	2.93E-12	0.397826	0.184591	0.000802	6.72E-05	2.36E-11
0.371609	-0.26627	-0.34388	0.028529	0.215524	0.310777	0.363772	0.270067	0.073353
1.7E-05	0.002479	7.54E-05	0.750185	0.014954	0.000376	2.62E-05	0.002136	0.412454
-0.1258	-0.62193	-0.62687	-0.44145	-0.18946	-0.17646	-0.24746	-0.17119	0.47651
0.158758	6.01E-15	3.17E-15	2.05E-07	0.032896	0.047196	0.005033	0.054306	1.49E-08
-0.14159	-0.00768	0.031271	-0.00439	0.201392	0.126607	0.165987	0.080567	-0.06711
0.112311	0.931709	0.727083	0.960964	0.02318	0.156077	0.062175	0.367892	0.45346

MFSD8	MGAT3	MGAT5	MGLL	MGRN1	MICAL3	MICU2	MIER1	MINK1
cyan	cyan	cyan	cyan	cyan	cyan	cyan	cyan	cyan
0.013111	0.061606	0.289584	0.066904	-0.11435	0.095785	-0.03868	-0.08531	0.143021
0.883682	0.491421	0.000959	0.454854	0.200511	0.284063	0.665947	0.340294	0.108694
-0.01049	0.114883	0.238914	0.149655	-0.14595	0.102429	0.002358	-0.14211	0.107983
0.906833	0.1984	0.006828	0.093088	0.10156	0.25183	0.979005	0.110992	0.226888
-0.04478	-0.2913	-0.32791	-0.37129	-0.10343	-0.17557	0.258173	0.30123	-0.53802
0.61712	0.000892	0.000168	1.73E-05	0.247187	0.048345	0.003384	0.000579	6.88E-11
0.484183	-0.56876	-0.37075	-0.62633	-0.48332	-0.41121	0.498285	0.642919	-0.71551
8.05E-09	3.04E-12	1.78E-05	3.4E-15	8.64E-09	1.57E-06	2.5E-09	3.66E-16	3.34E-21
0.118558	-0.20796	-0.41208	-0.30395	-0.58774	-0.55174	0.453554	0.072004	-0.52906
0.184325	0.018969	1.48E-06	0.000513	3.75E-13	1.78E-11	8.56E-08	0.421131	1.61E-10
0.641432	-0.66618	-0.47511	-0.709	-0.86743	-0.7467	0.707269	0.621897	-0.88325
4.5E-16	1.26E-17	1.66E-08	1.09E-20	1.04E-39	6.97E-24	1.49E-20	6.04E-15	6.18E-43
0.372129	-0.34983	-0.21205	-0.33878	-0.56002	-0.39803	0.466374	0.287131	-0.54803
1.65E-05	5.54E-05	0.016698	9.78E-05	7.63E-12	3.58E-06	3.27E-08	0.001064	2.58E-11
0.338328	-0.46675	-0.01109	-0.45061	-0.43724	-0.21782	0.202683	0.13511	-0.09649
0.0001	3.18E-08	0.901513	1.06E-07	2.75E-07	0.013893	0.022295	0.129897	0.280504
0.019923	-0.50834	0.073172	-0.28558	0.040473	-0.19095	-0.12695	0.150841	-0.23324
0.824066	1.05E-09	0.413612	0.001136	0.651422	0.031524	0.154938	0.090497	0.008314
0.191285	-0.51266	0.229038	-0.2253	-0.18758	-0.24939	-0.03421	-0.00078	0.024367
0.031217	7.19E-10	0.009594	0.010873	0.034702	0.004691	0.702588	0.993067	0.785674
0.417073	-0.48258	0.10709	-0.34746	-0.27121	-0.2434	0.05536	0.203552	-0.11354
1.07E-06	9.16E-09	0.230775	6.27E-05	0.002041	0.005826	0.536454	0.021715	0.203717
-0.32324	0.361043	0.226607	0.269608	0.471633	0.602649	-0.44508	-0.42215	0.307374
0.00021	3.05E-05	0.01041	0.002175	2.18E-08	6.57E-14	1.58E-07	7.68E-07	0.000439
-0.57067	0.21878	0.275067	0.262786	0.727552	0.567285	-0.48138	-0.34358	0.411248
2.48E-12	0.013468	0.001749	0.002838	3.41E-22	3.56E-12	1.01E-08	7.66E-05	1.56E-06
-0.56759	0.251944	0.020504	0.339174	0.674723	0.276708	-0.46549	-0.40881	0.438513
3.44E-12	0.004271	0.819014	9.59E-05	3.4E-18	0.001637	3.5E-08	1.83E-06	2.52E-07
-0.42891	-0.02056	0.439273	0.251404	0.619871	0.384131	-0.55699	-0.43759	0.56426
4.88E-07	0.818573	2.39E-07	0.004357	7.82E-15	8.26E-06	1.04E-11	2.68E-07	4.9E-12
-0.23457	0.594695	0.468147	0.534333	0.267719	0.544047	-0.19885	-0.44624	0.641466
0.007943	1.68E-13	2.86E-08	9.8E-11	0.002343	3.83E-11	0.025015	1.45E-07	4.48E-16
-0.51421	0.76478	0.030089	0.56634	0.523994	0.597565	-0.23304	-0.34566	0.400397
6.27E-10	1.26E-25	0.737015	3.93E-12	2.58E-10	1.2E-13	0.008371	6.88E-05	3.1E-06
0.320276	0.296238	-0.28394	0.04681	-0.23277	-0.11343	0.307981	0.392093	-0.17304
0.000242	0.00072	0.001216	0.601261	0.008451	0.204163	0.000428	5.14E-06	0.051713
-0.15754	0.674396	0.027035	0.484864	0.160921	0.257946	-0.12348	-0.22436	0.349381
0.076911	3.58E-18	0.762871	7.62E-09	0.070707	0.003413	0.166631	0.011217	5.67E-05
-0.56366	0.287685	-0.07167	0.025731	0.108762	0.209673	0.112149	-0.06342	-0.24652
5.22E-12	0.00104	0.42326	0.773992	0.223534	0.017987	0.20936	0.478695	0.005207

MINPP1	MIPEP	MIS18BP1	MITD1	MKL1	MLH1	MLLT1	MLLT6	MMADHC
cyan	cyan	cyan	cyan	cyan	cyan	cyan	cyan	cyan
-0.09033	-0.1376	-0.05902	0.076728	-0.04514	-0.15861	-0.28906	0.062487	-0.01483
0.312536	0.1229	0.50979	0.391228	0.614343	0.07491	0.000981	0.485233	0.868576
-0.04733	-0.12242	-0.15198	0.051059	-0.08708	-0.14044	-0.29541	0.029638	-0.04736
0.597221	0.17032	0.088053	0.568617	0.330298	0.115301	0.000747	0.740818	0.59697
0.35952	0.291617	0.370778	0.24315	-0.04123	0.510929	0.100456	-0.24236	0.220921
3.31E-05	0.00088	1.78E-05	0.005878	0.64538	8.39E-10	0.261126	0.006045	0.012562
0.574997	0.539312	0.553589	0.607034	-0.42005	0.646404	-0.33614	-0.56173	0.456126
1.55E-12	6.08E-11	1.48E-11	3.87E-14	8.82E-07	2.25E-16	0.000112	6.39E-12	7.08E-08
0.323969	0.377711	0.13081	0.49721	-0.43871	0.389494	-0.46641	-0.4627	0.605928
0.000203	1.2E-05	0.142678	2.74E-09	2.48E-07	6.01E-06	3.26E-08	4.33E-08	4.42E-14
0.697943	0.64981	0.566109	0.859683	-0.7329	0.723466	-0.7275	-0.84938	0.828742
7.6E-20	1.39E-16	4.03E-12	2.83E-38	1.19E-22	7.49E-22	3.44E-22	1.7E-36	2.63E-33
0.387092	0.513216	0.264594	0.533382	-0.49135	0.493746	-0.45675	-0.41476	0.425161
6.93E-06	6.85E-10	0.002646	1.07E-10	4.48E-09	3.67E-09	6.76E-08	1.25E-06	6.28E-07
-0.03988	0.372062	0.109033	0.29672	-0.51859	0.295332	-0.23685	-0.16815	0.232213
0.656174	1.65E-05	0.222377	0.000706	4.23E-10	0.000749	0.00734	0.058795	0.008613
0.203808	0.110006	0.243605	0.204407	0.137415	0.276893	0.073258	-0.14509	0.176752
0.021547	0.218253	0.005783	0.021157	0.123417	0.001625	0.413062	0.103622	0.046825
-0.01292	0.070011	0.130189	0.394172	-0.12238	0.150992	-0.24018	-0.13226	0.190265
0.885406	0.434131	0.144602	4.54E-06	0.170481	0.090171	0.006532	0.138263	0.032146
0.018205	0.121769	0.176078	0.406018	-0.21104	0.212841	-0.34814	-0.23721	0.210868
0.839017	0.172638	0.047683	2.18E-06	0.017232	0.016285	6.05E-05	0.007248	0.017327
-0.29806	-0.19435	-0.4088	-0.43143	0.564105	-0.23374	0.274449	0.363783	-0.59774
0.000665	0.028565	1.83E-06	4.11E-07	4.98E-12	0.008174	0.001793	2.62E-05	1.18E-13
-0.37928	-0.28517	-0.2186	-0.43659	0.649026	-0.17954	0.782794	0.629628	-0.63653
1.1E-05	0.001156	0.013546	2.88E-07	1.56E-16	0.04341	1.6E-27	2.21E-15	8.78E-16
-0.41403	-0.29331	-0.38354	-0.37383	0.54923	-0.28961	0.706342	0.478832	-0.39996
1.31E-06	0.000818	8.55E-06	1.5E-05	2.29E-11	0.000958	1.76E-20	1.24E-08	3.18E-06
-0.47885	-0.34233	-0.27056	-0.22993	0.520474	-0.23904	0.590008	0.542604	-0.48196
1.24E-08	8.17E-05	0.002094	0.00931	3.56E-10	0.006799	2.89E-13	4.41E-11	9.63E-09
-0.44275	-0.39469	-0.4374	-0.33617	0.105586	-0.44753	0.088328	0.467984	-0.46264
1.87E-07	4.39E-06	2.72E-07	0.000111	0.237428	1.33E-07	0.323398	2.89E-08	4.35E-08
-0.37252	-0.33901	-0.39695	-0.66039	0.447071	-0.39518	0.4844	0.517133	-0.5447
1.61E-05	9.67E-05	3.83E-06	3.01E-17	1.37E-07	4.26E-06	7.91E-09	4.82E-10	3.59E-11
0.258573	0.038796	0.142061	-0.14034	-0.26854	-0.1402	-0.33436	-0.27263	0.162788
0.003333	0.664978	0.111113	0.11556	0.002268	0.115919	0.000122	0.001929	0.067459
-0.23303	-0.32291	-0.33736	-0.58775	0.125541	-0.53217	0.023035	0.249856	-0.25162
0.008375	0.000213	0.000105	3.75E-13	0.159619	1.2E-10	0.797136	0.004612	0.004322
0.168987	0.09877	0.100255	-0.03126	0.250575	0.220721	0.168298	0.216464	-0.04821
0.057533	0.269253	0.262087	0.727207	0.004492	0.012644	0.058574	0.014511	0.59041

MMP17	MNAT1	MNT	MON1A	MOSPD1	MOSPD2	MPHOSPH	MPHOSPH	MPLKIP
cyan	cyan	cyan	cyan	cyan	cyan	cyan	cyan	cyan
-0.13339	0.111228	0.051028	-0.03989	0.0525	0.238446	0.048388	0.091012	-0.22138
0.1349	0.213151	0.568857	0.656163	0.557744	0.006941	0.589035	0.308857	0.012376
-0.08522	0.186894	0.024774	0.029238	0.088896	0.190213	0.035412	-0.00533	-0.1541
0.340773	0.035384	0.782188	0.744194	0.320284	0.032194	0.692657	0.952569	0.083671
-0.14196	0.291924	-0.30131	-0.18073	0.330578	-0.0564	0.400003	-0.40754	0.426337
0.111363	0.000868	0.000577	0.042011	0.000147	0.528799	3.17E-06	1.98E-06	5.8E-07
-0.56762	0.409283	-0.55734	-0.59076	0.622237	0.405312	0.568201	-0.4964	0.537382
3.44E-12	1.77E-06	1.01E-11	2.66E-13	5.78E-15	2.28E-06	3.23E-12	2.94E-09	7.32E-11
-0.21455	0.600101	-0.56977	-0.0926	0.520871	0.048044	0.18107	-0.22119	0.540677
0.015426	8.9E-14	2.73E-12	0.300477	3.44E-10	0.591689	0.041624	0.012453	5.32E-11
-0.6909	0.694693	-0.83323	-0.68161	0.897876	0.530096	0.582799	-0.55649	0.738143
2.5E-19	1.32E-19	5.81E-34	1.14E-18	2.3E-46	1.46E-10	6.55E-13	1.1E-11	4.13E-23
-0.44197	0.41064	-0.5285	-0.41417	0.535899	0.236806	0.366427	-0.41362	0.317005
1.97E-07	1.63E-06	1.7E-10	1.3E-06	8.44E-11	0.007351	2.27E-05	1.34E-06	0.000282
-0.42569	-0.04236	-0.27813	-0.35604	0.363406	0.312999	0.073694	-0.12227	0.096815
6.06E-07	0.636318	0.001545	3.99E-05	2.68E-05	0.00034	0.410284	0.170848	0.278891
-0.27563	-0.12175	-0.15302	-0.3747	0.139991	-0.04153	0.266932	-0.17846	0.1524
0.00171	0.172717	0.085879	1.42E-05	0.116472	0.642911	0.002416	0.044703	0.087176
-0.44526	-0.08695	-0.25087	-0.36356	0.121181	0.281766	0.112875	0.014705	0.037661
1.56E-07	0.331041	0.004443	2.65E-05	0.174734	0.001331	0.20641	0.869662	0.674217
-0.50321	-0.00823	-0.30066	-0.38565	0.219581	0.425927	0.163057	-0.1083	0.113324
1.64E-09	0.926833	0.000593	7.55E-06	0.013122	5.96E-07	0.067001	0.225526	0.204597
0.28098	-0.32585	0.422506	0.415133	-0.54192	-0.28916	-0.29463	0.233816	-0.30162
0.001375	0.000185	7.5E-07	1.22E-06	4.72E-11	0.000976	0.000773	0.008152	0.000569
0.295742	-0.4479	0.504835	0.376986	-0.57018	-0.44737	-0.12784	0.212415	-0.33504
0.000736	1.29E-07	1.43E-09	1.25E-05	2.61E-12	1.34E-07	0.152043	0.016505	0.000118
0.422672	-0.37968	0.382099	0.598711	-0.55474	-0.5726	-0.41193	0.324889	-0.08353
7.41E-07	1.07E-05	9.3E-06	1.05E-13	1.31E-11	2.01E-12	1.5E-06	0.000194	0.350502
0.112872	-0.55022	0.375232	0.254349	-0.55701	-0.27487	-0.24001	0.344208	-0.31572
0.206421	2.07E-11	1.38E-05	0.003906	1.04E-11	0.001763	0.00657	7.42E-05	0.000299
0.343829	-0.2619	0.328658	0.449092	-0.46366	0.100207	-0.3828	0.457572	-0.48912
7.56E-05	0.002936	0.000162	1.18E-07	4.03E-08	0.262316	8.93E-06	6.36E-08	5.38E-09
0.720197	-0.22945	0.46299	0.665003	-0.5327	-0.45292	-0.39601	0.22935	-0.36054
1.39E-21	0.00946	4.23E-08	1.51E-17	1.14E-10	8.97E-08	4.05E-06	0.009493	3.13E-05
0.197739	0.205791	-0.0105	-0.02544	0.195471	0.168752	-0.04272	-0.08553	0.022174
0.025853	0.02028	0.906745	0.77649	0.02764	0.057886	0.633454	0.339008	0.804563
0.583549	-0.12926	0.405591	0.417215	-0.3111	-0.22443	-0.41381	0.235115	-0.41742
6.02E-13	0.147502	2.24E-06	1.06E-06	0.000371	0.011194	1.33E-06	0.007795	1.05E-06
0.271997	0.320186	0.078878	0.177787	-0.01035	-0.32204	0.291009	0.068839	-0.04793
0.001978	0.000243	0.378047	0.045531	0.908025	0.000222	0.000903	0.441881	0.592583

MPP5	MREG	MROH1	MRPL13	MRPL3	MRPL30	MRPL32	MRPL39	MRPL47
cyan	cyan	cyan	cyan	cyan	cyan	cyan	cyan	cyan
-0.06971	-0.02438	0.006794	0.073289	-0.30193	-0.04842	-0.07247	0.108176	0.118043
0.436145	0.785531	0.939569	0.412866	0.000561	0.588755	0.418127	0.226053	0.186251
0.001529	-0.02733	-0.02034	0.111895	-0.32504	0.095334	-0.03969	0.188361	0.173738
0.98639	0.760334	0.820448	0.210402	0.000193	0.286347	0.657748	0.033943	0.050766
0.234312	0.158876	-0.48105	0.2692	0.510092	0.325658	0.300139	0.257931	0.271221
0.008014	0.074409	1.04E-08	0.00221	9.03E-10	0.000187	0.000607	0.003415	0.00204
0.497265	0.45394	-0.71882	0.465412	0.591837	0.395393	0.469584	0.432065	0.396299
2.73E-09	8.32E-08	1.8E-21	3.52E-08	2.34E-13	4.21E-06	2.56E-08	3.93E-07	3.98E-06
0.081903	0.316492	-0.405	0.747115	0.456225	0.49015	0.710328	0.689593	0.694618
0.359974	0.000289	2.32E-06	6.37E-24	7.03E-08	4.94E-09	8.6E-21	3.11E-19	1.34E-19
0.488113	0.61409	-0.85215	0.870405	0.733515	0.60994	0.806246	0.800427	0.775016
5.85E-09	1.62E-14	5.82E-37	2.78E-40	1.05E-22	2.71E-14	2.79E-30	1.46E-29	1.11E-26
0.228963	0.434547	-0.55608	0.543934	0.362776	0.354879	0.52055	0.502652	0.508602
0.009618	3.31E-07	1.14E-11	3.87E-11	2.77E-05	4.24E-05	3.54E-10	1.72E-09	1.03E-09
0.010981	0.14186	-0.23263	0.226712	0.209395	-0.1303	0.090565	0.179108	0.177619
0.902479	0.111626	0.00849	0.010374	0.018143	0.144265	0.311249	0.043924	0.045739
-0.22316	0.196562	-0.12006	0.023077	0.226306	-0.23626	0.053662	-0.09328	-0.02468
0.011673	0.026767	0.178801	0.79677	0.010515	0.007491	0.549047	0.296928	0.783007
-0.24612	0.20688	-0.06036	0.145347	0.004247	-0.35957	0.037986	-0.01124	0.103875
0.005284	0.019613	0.500258	0.103006	0.962203	3.3E-05	0.671562	0.90016	0.24516
0.071415	0.25854	-0.24093	0.170605	0.122016	-0.16236	0.115807	0.06961	0.109991
0.424949	0.003337	0.006361	0.055149	0.171762	0.068185	0.19479	0.436776	0.218315
-0.19221	-0.12639	0.323796	-0.37018	-0.40954	-0.18192	-0.43967	-0.49409	-0.49765
0.030398	0.156787	0.000204	1.84E-05	1.74E-06	0.040659	2.32E-07	3.56E-09	2.64E-09
-0.33442	-0.36964	0.404425	-0.61633	-0.32403	-0.47086	-0.55581	-0.56154	-0.54593
0.000122	1.89E-05	2.41E-06	1.22E-14	0.000202	2.32E-08	1.18E-11	6.51E-12	3.18E-11
-0.40459	-0.30446	0.577809	-0.4241	-0.23266	-0.44158	-0.23601	-0.40084	-0.38035
2.39E-06	0.000501	1.14E-12	6.74E-07	0.008481	2.03E-07	0.007557	3.01E-06	1.03E-05
-0.5424	-0.29658	0.529395	-0.53651	-0.40797	-0.76212	-0.52287	-0.57721	-0.48676
4.5E-11	0.00071	1.56E-10	7.96E-11	1.93E-06	2.33E-25	2.86E-10	1.22E-12	6.53E-09
-0.10347	-0.48463	0.402833	-0.36173	-0.64607	-0.24347	-0.46002	-0.31939	-0.25499
0.247017	7.77E-09	2.66E-06	2.93E-05	2.36E-16	0.005811	5.3E-08	0.000252	0.003813
-0.00596	-0.43748	0.363206	-0.44544	-0.34042	0.000975	-0.37656	-0.3832	-0.36519
0.947019	2.7E-07	2.71E-05	1.54E-07	9E-05	0.991324	1.28E-05	8.72E-06	2.43E-05
0.498369	0.048879	-0.15662	0.10625	0.108069	0.510059	0.180119	0.181671	0.093333
2.49E-09	0.585258	0.07867	0.234477	0.226516	9.05E-10	0.042727	0.040941	0.296631
-0.00377	-0.29443	0.362212	-0.25345	-0.32766	0.133538	-0.2201	-0.1651	-0.22601
0.966441	0.000779	2.86E-05	0.004038	0.00017	0.134464	0.012902	0.06361	0.010618
-0.14848	-0.19111	-0.11736	0.089024	0.115623	0.263594	0.068215	0.135278	0.200592
0.095726	0.031372	0.188813	0.319588	0.195506	0.00275	0.446044	0.129415	0.023744

MRPL50	MRPS17	MRPS30	MRPS31	MRPS35	MRRF	MRS2	MRTO4	MTAP
cyan	cyan	cyan	cyan	cyan	cyan	cyan	cyan	cyan
0.01583	-0.01685	0.102958	0.081579	0.097129	0.17098	0.095988	-0.3038	0.299655
0.859793	0.850845	0.249377	0.361885	0.27733	0.05461	0.283038	0.000516	0.00062
0.069419	0.087039	0.124838	0.16362	0.200684	0.192544	0.143703	-0.22414	0.244553
0.438038	0.330538	0.161986	0.066051	0.023679	0.030102	0.107003	0.011301	0.00559
0.274009	0.315641	0.179412	0.172774	0.25025	0.27065	-0.03776	0.497716	-0.20433
0.001825	0.000301	0.043561	0.052084	0.004546	0.002087	0.673402	2.63E-09	0.021208
0.466015	0.397501	0.347805	0.312659	0.385568	0.507751	0.339993	0.310899	0.180498
3.36E-08	3.7E-06	6.16E-05	0.000345	7.59E-06	1.11E-09	9.2E-05	0.000374	0.042285
0.65047	0.447051	0.453371	0.629112	0.537398	0.511114	0.372686	0.603489	0.165692
1.27E-16	1.37E-07	8.67E-08	2.36E-15	7.31E-11	8.25E-10	1.6E-05	5.94E-14	0.062647
0.812637	0.565825	0.612028	0.632518	0.673788	0.754045	0.663892	0.477774	0.441204
4.25E-31	4.15E-12	2.09E-14	1.5E-15	3.94E-18	1.42E-24	1.78E-17	1.35E-08	2.08E-07
0.520691	0.458927	0.207883	0.478606	0.366419	0.585738	0.372205	0.291436	0.333402
3.49E-10	5.75E-08	0.019015	1.26E-08	2.27E-05	4.71E-13	1.64E-05	0.000886	0.000128
0.335147	-0.12692	0.113902	0.053434	-0.04434	0.247308	0.234799	-0.088	0.262018
0.000117	0.155032	0.202284	0.550748	0.620586	0.005061	0.007881	0.325227	0.002923
-0.0356	0.140793	-0.13983	-0.20536	-0.17873	0.142873	0.042306	-0.065	0.119793
0.691139	0.114371	0.116891	0.020549	0.044382	0.109065	0.636739	0.4678	0.179761
0.105249	-0.12947	-0.07927	-0.05413	-0.19463	0.250679	0.134795	-0.35512	0.42257
0.238938	0.146866	0.375672	0.545564	0.028328	0.004474	0.1308	4.19E-05	7.46E-07
0.160032	0.049157	0.075467	-0.03054	-0.06963	0.231124	0.247238	-0.27596	0.479394
0.072298	0.583125	0.399082	0.733186	0.436629	0.008939	0.005074	0.001687	1.18E-08
-0.60693	0.205912	-0.51548	-0.31725	-0.18483	-0.29142	-0.37375	-0.19806	-0.24425
3.92E-14	0.020205	5.6E-10	0.000279	0.037498	0.000887	1.5E-05	0.025604	0.00565
-0.55863	-0.29015	-0.50141	-0.48305	-0.57232	-0.36526	-0.66572	-0.21099	-0.47995
8.81E-12	0.000936	1.92E-09	8.83E-09	2.08E-12	2.42E-05	1.36E-17	0.017262	1.13E-08
-0.37771	-0.29829	-0.27348	-0.37804	-0.48502	-0.46249	-0.49761	0.045146	-0.49806
1.2E-05	0.000659	0.001865	1.18E-05	7.52E-09	4.4E-08	2.65E-09	0.614264	2.55E-09
-0.4989	-0.4678	-0.53079	-0.54271	-0.73595	-0.32494	-0.48211	-0.47755	-0.15778
2.38E-09	2.93E-08	1.37E-10	4.36E-11	6.45E-23	0.000193	9.52E-09	1.37E-08	0.076457
-0.29444	-0.44359	-0.3613	-0.11075	-0.24083	-0.30963	-0.18043	-0.45237	0.049475
0.000779	1.76E-07	3E-05	0.215152	0.006382	0.000397	0.042362	9.33E-08	0.580687
-0.40147	-0.1933	-0.30747	-0.17845	-0.14527	-0.51336	-0.50914	0.092047	-0.53578
2.9E-06	0.02945	0.000438	0.044721	0.103192	6.76E-10	9.81E-10	0.303365	8.54E-11
0.136898	0.136253	0.345401	0.210207	0.473842	-0.10349	0.292681	0.194905	-0.0074
0.124847	0.126649	6.98E-05	0.017689	1.84E-08	0.246939	0.00084	0.028102	0.934213
-0.23983	-0.13475	-0.04002	-0.04461	0.095081	-0.41317	-0.09122	-0.01314	-0.18785
0.006611	0.130936	0.655046	0.61848	0.287635	1.38E-06	0.307761	0.883401	0.034437
0.008887	0.192498	0.053806	0.303331	0.180184	0.232406	-0.37863	0.332734	-0.30506
0.921013	0.030142	0.547969	0.000527	0.042651	0.008556	1.14E-05	0.000132	0.000488

MTERF1	MTERF2	MTERF3	MTFMT	MTHFR	MTIF2	MTO1	MTRF1	MTRR
cyan	cyan	cyan	cyan	cyan	cyan	cyan	cyan	cyan
-0.06339	-0.09296	0.019656	-0.06	0.128388	0.083466	0.281401	0.066095	0.130724
0.478941	0.298594	0.826381	0.502814	0.150289	0.350847	0.001351	0.460334	0.142943
-0.02059	0.005239	0.054568	-0.00407	0.041348	0.063928	0.249875	0.063401	0.11335
0.818277	0.953386	0.54231	0.963758	0.644396	0.475204	0.004609	0.478854	0.204495
0.419754	0.22874	0.436558	0.22643	-0.1391	0.07482	-0.05341	0.2808	0.016655
8.99E-07	0.009691	2.88E-07	0.010472	0.118832	0.403149	0.550921	0.001385	0.852562
0.703257	0.426647	0.68372	0.45761	-0.37607	0.462305	0.3578	0.57111	0.433123
3.03E-20	5.68E-07	8.13E-19	6.34E-08	1.32E-05	4.46E-08	3.63E-05	2.36E-12	3.66E-07
0.301079	0.24278	0.553984	0.403127	-0.5872	0.213149	0.215299	0.361008	0.248241
0.000583	0.005956	1.42E-11	2.61E-06	3.99E-13	0.016127	0.015062	3.05E-05	0.004892
0.795345	0.481046	0.892729	0.648046	-0.72431	0.640923	0.594074	0.711343	0.619981
5.93E-29	1.04E-08	4.21E-45	1.79E-16	6.38E-22	4.82E-16	1.81E-13	7.16E-21	7.71E-15
0.455819	0.217693	0.56052	0.370217	-0.38689	0.406417	0.36181	0.505412	0.435246
7.24E-08	0.013949	7.24E-12	1.83E-05	7.02E-06	2.13E-06	2.92E-05	1.36E-09	3.16E-07
0.173388	0.249143	0.216418	0.071291	-0.21296	0.492857	0.251829	0.139562	0.223738
0.05124	0.004734	0.014532	0.425754	0.016223	3.95E-09	0.004289	0.117605	0.011451
0.111803	-0.29123	0.120227	0.096143	0.0711	0.186013	0.013782	0.056262	-0.042
0.210779	0.000894	0.178181	0.282261	0.426995	0.036274	0.877779	0.52983	0.63921
-0.02152	-0.35414	0.06431	-0.06907	0.070195	0.419716	0.164085	0.104116	0.206028
0.810249	4.41E-05	0.472564	0.440349	0.432923	9.02E-07	0.065274	0.244061	0.020134
0.188086	-0.06519	0.171371	-0.01063	-0.02994	0.379574	0.336493	0.160954	0.237601
0.034209	0.466485	0.054051	0.905577	0.73824	1.08E-05	0.00011	0.070649	0.00715
-0.24124	-0.16039	-0.26359	-0.08485	0.330419	-0.47861	-0.25734	-0.21921	-0.29057
0.006291	0.071648	0.002751	0.342864	0.000148	1.26E-08	0.003492	0.013281	0.00092
-0.38539	-0.30572	-0.44185	-0.40128	0.641129	-0.38187	-0.52961	-0.35183	-0.5012
7.67E-06	0.000473	1.99E-07	2.93E-06	4.69E-16	9.42E-06	1.53E-10	4.99E-05	1.95E-09
-0.54776	-0.34777	-0.50294	-0.37566	0.340342	-0.4892	-0.59716	-0.47415	-0.50947
2.65E-11	6.17E-05	1.68E-09	1.35E-05	9.04E-05	5.35E-09	1.26E-13	1.79E-08	9.53E-10
-0.55857	-0.58425	-0.54655	-0.5064	0.610382	-0.16297	-0.4361	-0.42256	-0.38195
8.86E-12	5.57E-13	2.99E-11	1.25E-09	2.57E-14	0.067151	2.98E-07	7.47E-07	9.38E-06
-0.49972	-0.19236	-0.48842	-0.42255	0.319218	-0.19174	-0.12873	-0.30684	-0.04385
2.22E-09	0.030264	5.7E-09	7.47E-07	0.000254	0.030815	0.149209	0.00045	0.62446
-0.38665	-0.05705	-0.4511	-0.37481	0.320064	-0.64852	-0.51605	-0.39118	-0.42924
7.12E-06	0.524097	1.02E-07	1.42E-05	0.000244	1.67E-16	5.32E-10	5.43E-06	4.77E-07
0.265663	0.434039	0.122946	0.228773	-0.33903	-0.11311	0.230729	0.128526	0.21592
0.002539	3.43E-07	0.168494	0.00968	9.66E-05	0.205458	0.00906	0.149848	0.014766
-0.27196	0.054082	-0.36031	-0.14703	0.039251	-0.43412	-0.10989	-0.29524	-0.19383
0.001981	0.545915	3.17E-05	0.099029	0.661293	3.41E-07	0.218724	0.000753	0.028999
-0.01972	-0.03226	0.196446	0.060096	0.15939	-0.22885	-0.23829	0.222938	-0.05562
0.82579	0.718834	0.026859	0.502119	0.073464	0.009653	0.006979	0.011758	0.534575

MTSS1L	MTTP	MUM1	MUT	MYBBP1A	MYH10	MYH7	MYH7B	MYNN
cyan	cyan	cyan	cyan	cyan	cyan	cyan	cyan	cyan
-0.1832	-0.08021	-0.13166	-0.01532	-0.18037	-0.28399	-0.08961	0.191352	-0.1129
0.039247	0.370046	0.140066	0.864275	0.042435	0.001214	0.316404	0.031157	0.206303
-0.10921	-0.02249	-0.14514	0.018757	-0.1707	-0.27279	-0.09174	0.233128	-0.08423
0.221643	0.801805	0.103503	0.834203	0.055016	0.001916	0.305008	0.008347	0.34646
-0.10602	0.156547	-0.03961	0.182636	0.087802	0.341894	-0.07832	-0.5011	0.536337
0.235482	0.078814	0.658376	0.039862	0.326301	8.35E-05	0.381418	1.97E-09	8.09E-11
-0.47212	0.521106	-0.41893	0.525889	-0.34769	0.020874	-0.2417	-0.55388	0.734974
2.1E-08	3.36E-10	9.49E-07	2.17E-10	6.2E-05	0.81581	0.006189	1.43E-11	7.85E-23
-0.49548	0.299331	-0.54146	0.24462	-0.58899	-0.63142	-0.33957	-0.2263	0.369563
3.17E-09	0.000629	4.93E-11	0.005576	3.25E-13	1.74E-15	9.4E-05	0.010517	1.9E-05
-0.82029	0.688752	-0.8243	0.659538	-0.81171	-0.51287	-0.50448	-0.57697	0.796692
4.05E-32	3.57E-19	1.13E-32	3.41E-17	5.61E-31	7.06E-10	1.47E-09	1.25E-12	4.11E-29
-0.44484	0.464056	-0.54365	0.301538	-0.52116	-0.38431	-0.21609	-0.31166	0.431762
1.61E-07	3.91E-08	3.98E-11	0.000571	3.35E-10	8.17E-06	0.014688	0.000361	4.01E-07
-0.29972	0.494672	-0.40185	0.290571	-0.42174	-0.22463	0.202526	-0.0921	0.045548
0.000619	3.39E-09	2.83E-06	0.00092	7.89E-07	0.011119	0.022401	0.303071	0.611111
-0.24804	0.072819	-0.14797	-0.21426	0.100892	-0.03001	-0.17727	-0.44901	0.116407
0.004928	0.415879	0.096867	0.015567	0.259051	0.737662	0.046171	1.19E-07	0.192472
-0.36508	0.165235	-0.33287	-0.19498	-0.27818	-0.63875	-0.20182	-0.12585	-0.17815
2.44E-05	0.063387	0.000131	0.028038	0.001542	6.49E-16	0.022884	0.158601	0.04509
-0.37605	0.246592	-0.33064	0.110915	-0.30949	-0.45362	-0.18468	-0.20506	0.058809
1.32E-05	0.005194	0.000147	0.21445	0.000399	8.52E-08	0.037655	0.020738	0.511336
0.544984	-0.36769	0.539761	-0.27928	0.525853	0.342819	0.158749	0.117986	-0.28091
3.49E-11	2.11E-05	5.82E-11	0.001474	2.17E-10	7.97E-05	0.074644	0.186465	0.001379
0.636486	-0.40255	0.706222	-0.52652	0.82404	0.601953	0.426748	0.180754	-0.36227
8.83E-16	2.71E-06	1.79E-20	2.04E-10	1.22E-32	7.14E-14	5.64E-07	0.041988	2.85E-05
0.507779	-0.38499	0.566981	-0.57857	0.621719	0.060365	0.236175	0.307572	-0.4477
1.11E-09	7.85E-06	3.68E-12	1.05E-12	6.17E-15	0.500204	0.007514	0.000436	1.31E-07
0.417925	-0.32351	0.465624	-0.69714	0.548879	-0.04263	0.236409	0.26133	-0.63797
1.01E-06	0.000207	3.47E-08	8.72E-20	2.37E-11	0.634168	0.007453	0.003001	7.22E-16
0.450656	-0.38007	0.374662	-0.29104	0.120756	-0.15545	0.180147	0.645564	-0.61461
1.06E-07	1.05E-05	1.43E-05	0.000901	0.176264	0.080955	0.042693	2.53E-16	1.52E-14
0.727949	-0.53689	0.676571	-0.24382	0.554783	0.618539	0.453421	0.302822	-0.25019
3.15E-22	7.67E-11	2.55E-18	0.005739	1.31E-11	9.26E-15	8.64E-08	0.000539	0.004556
-0.10301	0.055206	-0.11839	0.520744	-0.24665	0.290628	-0.09302	-0.02209	0.355279
0.249134	0.537589	0.184941	3.48E-10	0.005184	0.000917	0.298254	0.805286	4.15E-05
0.313143	-0.33975	0.246255	0.029392	0.120971	0.351598	0.201037	0.339503	-0.22924
0.000338	9.31E-05	0.005257	0.742889	0.17549	5.05E-05	0.02343	9.43E-05	0.009527
0.138543	-0.3018	0.182748	-0.20044	0.218803	0.316605	-0.0222	-0.01707	0.213985
0.120335	0.000564	0.039739	0.023855	0.013457	0.000287	0.804337	0.848968	0.015705

MYO15B	MYO18A	MYPOP	N4BP2L2	NACC1	NAE1	NANP	NAPA	NAT1
cyan	cyan	cyan	cyan	cyan	cyan	cyan	cyan	cyan
0.097055	0.128626	-0.05891	0.105979	-0.20775	-0.04952	-0.07944	0.157385	0.142368
0.277696	0.149528	0.510612	0.23568	0.019094	0.580367	0.374631	0.077205	0.110335
0.02914	0.069405	0.047258	0.068755	-0.22021	0.037667	-0.09537	0.189903	0.047153
0.745019	0.438131	0.597775	0.442443	0.012855	0.674162	0.286162	0.032481	0.598592
-0.37534	-0.38544	-0.16024	0.029183	-0.06173	0.488604	0.542738	-0.50597	0.105998
1.37E-05	7.65E-06	0.071923	0.74466	0.490537	5.62E-09	4.35E-11	1.29E-09	0.235595
-0.41569	-0.56453	-0.54729	0.427214	-0.48039	0.638433	0.713345	-0.78265	0.509208
1.17E-06	4.76E-12	2.78E-11	5.47E-07	1.09E-08	6.78E-16	4.97E-21	1.66E-27	9.76E-10
-0.32994	-0.59947	-0.06765	0.170967	-0.56375	0.341851	0.254488	-0.24153	0.293826
0.000152	9.6E-14	0.44983	0.054628	5.17E-12	8.37E-05	0.003886	0.006226	0.0008
-0.52399	-0.84517	-0.60126	0.524796	-0.85614	0.720016	0.731556	-0.81438	0.731234
2.58E-10	8.28E-36	7.76E-14	2.4E-10	1.2E-37	1.44E-21	1.55E-22	2.51E-31	1.65E-22
-0.21667	-0.45533	-0.38995	0.455076	-0.54266	0.412013	0.313534	-0.40947	0.475023
0.014415	7.51E-08	5.85E-06	7.65E-08	4.39E-11	1.49E-06	0.000331	1.75E-06	1.67E-08
-0.001	-0.19939	-0.48156	0.301756	-0.42525	0.249133	0.111237	-0.30637	0.343519
0.991084	0.024612	9.95E-09	0.000565	6.24E-07	0.004735	0.213114	0.00046	7.69E-05
0.064805	-0.12229	-0.42017	0.037291	0.065778	0.015773	0.304363	-0.21873	0.435321
0.469151	0.1708	8.75E-07	0.677234	0.462493	0.860292	0.000503	0.013491	3.14E-07
0.32588	0.038891	-0.58526	0.254221	-0.20314	-0.16424	0.059205	-0.10175	0.632997
0.000185	0.664205	4.97E-13	0.003925	0.021989	0.065025	0.508488	0.254998	1.41E-15
0.130766	-0.07375	-0.56572	0.277866	-0.30748	-0.01664	0.181484	-0.17255	0.600989
0.142817	0.409956	4.2E-12	0.001562	0.000437	0.852658	0.041152	0.052386	8.01E-14
0.174227	0.432891	0.267195	-0.29322	0.490984	-0.36603	-0.2021	0.573455	-0.37461
0.050109	3.71E-07	0.002391	0.000821	4.61E-09	2.32E-05	0.022693	1.83E-12	1.43E-05
0.382244	0.54359	0.221704	-0.30236	0.708224	-0.30904	-0.21025	0.307424	-0.42502
9.22E-06	4E-11	0.012244	0.00055	1.26E-20	0.000407	0.017667	0.000438	6.34E-07
0.385761	0.41008	0.43233	-0.48299	0.640246	-0.5337	-0.3772	0.504079	-0.36589
7.5E-06	1.68E-06	3.86E-07	8.87E-09	5.29E-16	1.04E-10	1.23E-05	1.53E-09	2.33E-05
0.680966	0.618197	-0.0504	-0.22426	0.572372	-0.57692	-0.36166	0.399414	-0.03926
1.27E-18	9.67E-15	0.573602	0.011254	2.06E-12	1.26E-12	2.94E-05	3.29E-06	0.661253
0.38795	0.622725	0.297233	-0.08101	0.235526	-0.40621	-0.63822	0.502041	-0.31826
6.59E-06	5.43E-15	0.00069	0.365252	0.007685	2.15E-06	6.98E-16	1.82E-09	0.000266
-0.00408	0.425894	0.662665	-0.42477	0.565205	-0.25595	-0.42573	0.459665	-0.75367
0.963651	5.98E-07	2.14E-17	6.45E-07	4.44E-12	0.003679	6.04E-07	5.44E-08	1.55E-24
-0.49295	-0.25971	0.293303	0.022664	-0.21169	0.258697	0.072924	-0.12101	-0.22242
3.92E-09	0.003192	0.000818	0.800336	0.016887	0.003317	0.415202	0.175335	0.01196
-0.13688	0.201804	0.646672	-0.28599	0.201134	-0.19199	-0.44769	0.472068	-0.62256
0.124903	0.022895	2.17E-16	0.001117	0.023361	0.030587	1.31E-07	2.11E-08	5.55E-15
-0.13893	-0.09578	0.265812	0.088339	0.092654	0.252902	0.163578	-0.09925	-0.33296
0.119294	0.284079	0.002524	0.323337	0.30017	0.004122	0.066121	0.266947	0.000131

NCAM1	NCKAP5L	NDUFAF7	NEMF	NEURL4	NF2	NFE2L1	NFE2L2	NFYC
cyan	cyan	cyan	cyan	cyan	cyan	cyan	cyan	cyan
-0.09793	-0.13865	-0.05542	0.098207	-0.17333	-0.07253	0.184058	0.115117	-0.03284
0.273376	0.120044	0.535979	0.272005	0.051315	0.417703	0.038317	0.197478	0.713998
-0.05776	-0.15824	0.006343	0.037332	-0.13993	-0.08549	0.134423	0.027579	0.028809
0.518886	0.075598	0.943573	0.676902	0.116642	0.339255	0.131878	0.758247	0.747817
0.006431	0.025692	0.356657	-0.25583	0.062022	-0.03901	-0.48605	0.170835	0.059344
0.942796	0.774325	3.86E-05	0.003696	0.488491	0.663277	6.92E-09	0.054817	0.507495
-0.31724	-0.39947	0.497697	-0.29447	-0.37341	-0.32101	-0.43091	0.547413	-0.39139
0.000279	3.28E-06	2.63E-09	0.000778	1.53E-05	0.000233	4.26E-07	2.74E-11	5.37E-06
-0.48224	-0.49379	0.396983	-0.38025	-0.58188	-0.46098	-0.48039	0.133977	0.07518
9.42E-09	3.66E-09	3.82E-06	1.04E-05	7.26E-13	4.93E-08	1.09E-08	0.133176	0.400879
-0.67991	-0.77579	0.616699	-0.45808	-0.8375	-0.62593	-0.54058	0.625541	-0.40775
1.5E-18	9.19E-27	1.17E-14	6.12E-08	1.32E-34	3.58E-15	5.37E-11	3.77E-15	1.95E-06
-0.37532	-0.48172	0.496215	-0.44927	-0.53106	-0.40929	-0.24437	0.402387	-0.29068
1.38E-05	9.83E-09	2.98E-09	1.17E-07	1.34E-10	1.77E-06	0.005627	2.74E-06	0.000915
-0.16904	-0.40004	-0.009	-0.19864	-0.45003	-0.39118	0.157921	0.455079	-0.32591
0.057449	3.17E-06	0.919986	0.025168	1.11E-07	5.43E-06	0.07619	7.65E-08	0.000185
-0.42618	0.147324	-0.04908	-0.10161	-0.08518	0.001672	0.13953	0.429969	-0.17432
5.86E-07	0.098356	0.583746	0.255679	0.340997	0.985117	0.11769	4.54E-07	0.049988
-0.52931	-0.03442	-0.21564	0.04667	-0.44089	-0.29162	0.315232	0.609256	-0.35274
1.57E-10	0.700892	0.014899	0.602347	2.13E-07	0.000879	0.000306	2.95E-14	4.75E-05
-0.45769	-0.21622	-0.15843	-0.02696	-0.45782	-0.28844	0.1585	0.580578	-0.39869
6.3E-08	0.014626	0.075232	0.763487	6.24E-08	0.001007	0.075107	8.39E-13	3.44E-06
0.251485	0.459029	-0.36157	0.253677	0.571618	0.507637	0.326799	-0.34295	0.233503
0.004344	5.7E-08	2.96E-05	0.004005	2.24E-12	1.12E-09	0.000177	7.91E-05	0.00824
0.512885	0.807467	-0.3356	0.260654	0.780653	0.504084	0.249875	-0.17774	0.304147
7.05E-10	1.96E-30	0.000115	0.003079	2.75E-27	1.52E-09	0.004609	0.045595	0.000508
0.246	0.716981	-0.41025	0.180611	0.546458	0.295476	0.12221	-0.28329	0.456228
0.005306	2.54E-21	1.67E-06	0.042154	3.02E-11	0.000745	0.171075	0.00125	7.03E-08
0.116844	0.755427	-0.598	0.352819	0.40908	0.277858	0.521292	0.120106	0.129262
0.190795	1.05E-24	1.14E-13	4.73E-05	1.8E-06	0.001562	3.31E-10	0.17862	0.147507
0.493895	0.237822	-0.35073	0.436108	0.217906	0.20983	0.350269	-0.3699	0.103301
3.62E-09	0.007095	5.29E-05	2.97E-07	0.013853	0.017899	5.41E-05	1.87E-05	0.247793
0.807472	0.427984	-0.1838	0.175954	0.689113	0.481825	-0.05071	-0.74694	0.44787
1.96E-30	5.19E-07	0.038589	0.047843	3.36E-19	9.74E-09	0.571263	6.62E-24	1.29E-07
0.170504	-0.46982	0.348294	-0.07724	-0.07553	-0.04285	-0.32033	-0.39745	-0.05884
0.055297	2.51E-08	6E-05	0.388091	0.398681	0.632378	0.000241	3.71E-06	0.511107
0.446742	-0.07413	-0.0462	0.07711	0.282585	0.245635	0.05005	-0.73134	0.263788
1.4E-07	0.407497	0.606008	0.388862	0.001287	0.005376	0.576296	1.62E-22	0.00273
0.23921	0.267404	0.364895	-0.02415	0.31326	0.21678	-0.429	-0.28283	0.394698
0.006758	0.002372	2.47E-05	0.787557	0.000336	0.014365	4.85E-07	0.001273	4.39E-06

NGDN	NGLY1	NIP7	NISCH	NKAIN3	NLGN2	NMD3	NME6	NME7
cyan	cyan	cyan	cyan	cyan	cyan	cyan	cyan	cyan
0.083757	-0.0777	-0.14853	-0.00745	-0.20234	-0.23979	-0.09655	-0.0819	0.037078
0.349166	0.385263	0.095608	0.933763	0.022524	0.006622	0.280224	0.359967	0.678981
0.159582	-0.07301	-0.14751	-0.00249	-0.19825	-0.20013	-0.12975	-0.12932	0.01053
0.073114	0.414652	0.09792	0.977847	0.025462	0.024079	0.145988	0.14734	0.906468
0.237814	0.365712	0.554293	-0.26067	0.264443	0.200179	0.377365	0.436771	0.22484
0.007097	2.36E-05	1.37E-11	0.003077	0.002662	0.02404	1.22E-05	2.84E-07	0.011041
0.35046	0.612829	0.752358	-0.56722	-0.07467	-0.27574	0.718448	0.614776	0.417058
5.36E-05	1.9E-14	2.06E-24	3.58E-12	0.404084	0.001702	1.93E-21	1.49E-14	1.07E-06
0.708143	0.302772	0.295978	-0.48335	-0.35066	-0.53683	0.251421	0.164913	0.394824
1.27E-20	0.00054	0.000729	8.62E-09	5.3E-05	7.72E-11	0.004354	0.06391	4.36E-06
0.68301	0.680744	0.795283	-0.83251	-0.40739	-0.74723	0.80317	0.570364	0.618921
9.12E-19	1.31E-18	6.03E-29	7.42E-34	2E-06	6.22E-24	6.74E-30	2.56E-12	8.82E-15
0.445106	0.538908	0.424847	-0.47347	-0.47203	-0.42654	0.45471	0.379799	0.2711
1.58E-07	6.32E-11	6.41E-07	1.89E-08	2.12E-08	5.72E-07	7.86E-08	1.06E-05	0.00205
-0.02719	0.128033	0.073315	-0.22919	-0.19446	-0.49004	0.211895	0.297719	0.626961
0.761551	0.15143	0.412697	0.009546	0.028468	4.99E-09	0.016777	0.000675	3.13E-15
-0.16917	0.378612	0.406289	-0.12913	-0.24668	-0.08896	0.238627	0.310431	0.013501
0.057266	1.14E-05	2.14E-06	0.147934	0.005177	0.319951	0.006898	0.000382	0.880248
-0.03327	0.204991	0.144075	-0.31042	-0.44522	-0.52355	0.140653	0.164912	0.082427
0.710426	0.020783	0.106086	0.000383	1.56E-07	2.69E-10	0.114737	0.063912	0.356899
0.011975	0.246557	0.248706	-0.37298	-0.29863	-0.52338	0.323628	0.328242	0.233994
0.893705	0.0052	0.00481	1.57E-05	0.000649	2.73E-10	0.000206	0.000165	0.008102
-0.3228	-0.2253	-0.24227	0.391113	0.150364	0.40329	-0.41466	-0.07908	-0.42113
0.000214	0.010875	0.006065	5.45E-06	0.091531	2.59E-06	1.25E-06	0.37682	8.21E-07
-0.45298	-0.18967	-0.24643	0.522761	0.460711	0.762869	-0.42088	-0.03752	-0.3687
8.93E-08	0.032699	0.005224	2.89E-10	5.03E-08	1.96E-25	8.35E-07	0.675355	2E-05
-0.19108	-0.32393	-0.33191	0.384999	0.292965	0.454537	-0.50311	-0.28244	-0.34138
0.031403	0.000203	0.000138	7.85E-06	0.00083	7.96E-08	1.66E-09	0.001295	8.58E-05
-0.46897	-0.21742	-0.32136	0.33841	0.096711	0.297319	-0.45115	-0.13153	-0.34317
2.68E-08	0.014073	0.00023	9.97E-05	0.279413	0.000687	1.02E-07	0.140477	7.82E-05
-0.21472	-0.57105	-0.67444	0.32002	0.056782	0.163367	-0.57822	-0.50821	-0.37183
0.015343	2.38E-12	3.55E-18	0.000245	0.526022	0.066476	1.09E-12	1.06E-09	1.68E-05
-0.23432	-0.51516	-0.48204	0.506157	0.570159	0.770857	-0.44073	-0.36486	-0.39265
0.008011	5.76E-10	9.58E-09	1.27E-09	2.62E-12	3.03E-26	2.15E-07	2.47E-05	4.97E-06
0.125973	-0.12855	0.029505	-0.03228	0.119705	-0.02065	0.203586	-0.16739	0.026057
0.158176	0.149768	0.741942	0.718627	0.180086	0.81777	0.021693	0.059971	0.771211
-0.17264	-0.49061	-0.52357	0.444416	0.18529	0.284331	-0.36034	-0.51129	-0.25539
0.052267	4.76E-09	2.68E-10	1.66E-07	0.037019	0.001197	3.16E-05	8.13E-10	0.003758
0.231013	0.126715	0.083769	0.174206	0.104216	0.458685	-0.13399	0.090555	-0.09905
0.008973	0.155719	0.349096	0.050137	0.243605	5.85E-08	0.133129	0.311299	0.267876

NMNAT1	NOC3L	NOL6	NOP16	NOXO1	NPHP4	NPM3	NPRL3	NR2C1
cyan	cyan	cyan	cyan	cyan	cyan	cyan	cyan	cyan
0.061246	-0.01587	-0.04512	-0.18881	-0.17373	-0.2305	-0.01472	-0.1364	0.031567
0.49396	0.859456	0.614447	0.033515	0.050777	0.00913	0.869503	0.126225	0.724603
0.105392	-0.01163	-0.07516	-0.15376	-0.11024	-0.23711	0.032588	-0.11759	0.088292
0.238296	0.896736	0.40099	0.084367	0.217267	0.007274	0.716068	0.187968	0.323597
0.349873	0.37593	-0.05873	0.505146	0.023798	-0.0607	0.399388	-0.05537	0.227941
5.53E-05	1.33E-05	0.511893	1.39E-09	0.790562	0.497852	3.3E-06	0.536392	0.009955
0.654977	0.709127	-0.26957	0.418489	-0.38658	-0.31092	0.228597	-0.5196	0.483473
6.64E-17	1.07E-20	0.002178	9.77E-07	7.15E-06	0.000374	0.009738	3.86E-10	8.53E-09
0.372559	0.316783	-0.41601	0.457266	-0.1166	-0.32938	0.55228	-0.36148	0.279858
1.61E-05	0.000285	1.15E-06	6.5E-08	0.191735	0.000156	1.68E-11	2.97E-05	0.00144
0.793415	0.832499	-0.52462	0.496774	-0.55087	-0.5307	0.414923	-0.80322	0.594077
1E-28	7.45E-34	2.44E-10	2.84E-09	1.94E-11	1.38E-10	1.23E-06	6.65E-30	1.81E-13
0.563514	0.465993	-0.2885	0.474495	-0.27023	-0.32572	0.237586	-0.4566	0.33779
5.3E-12	3.37E-08	0.001004	1.74E-08	0.002122	0.000186	0.007153	6.83E-08	0.000103
0.176442	0.155963	-0.13358	-0.15599	-0.26342	0.182756	-0.27957	-0.35422	0.190871
0.047218	0.079951	0.13433	0.079891	0.002769	0.03973	0.001457	4.4E-05	0.031591
0.174523	0.230341	0.262347	0.268911	-0.17225	-0.12295	0.115649	-0.01279	-0.08744
0.049714	0.00918	0.002886	0.002235	0.052805	0.168471	0.195403	0.886475	0.328284
0.0932	0.240515	0.08579	0.012093	-0.3935	-0.20251	-0.11732	-0.18283	-0.08288
0.297322	0.006454	0.337551	0.892664	4.72E-06	0.022414	0.188967	0.039654	0.354278
0.242612	0.345009	0.022477	0.008905	-0.45933	-0.29462	-0.09717	-0.28819	0.026364
0.005991	7.12E-05	0.801944	0.920852	5.58E-08	0.000773	0.277106	0.001018	0.768585
-0.15639	-0.36422	0.449845	-0.01366	0.343132	0.00184	-0.00324	0.52433	-0.42069
0.079115	2.56E-05	1.12E-07	0.878893	7.84E-05	0.983624	0.971139	2.5E-10	8.46E-07
-0.38221	-0.38509	0.487883	-0.01995	0.449884	0.43048	-0.13414	0.713884	-0.35789
9.24E-06	7.8E-06	5.96E-09	0.823849	1.12E-07	4.38E-07	0.132699	4.5E-21	3.61E-05
-0.52018	-0.46636	0.266605	0.0354	0.549508	0.391276	0.072809	0.751385	-0.40455
3.66E-10	3.28E-08	0.002447	0.692756	2.23E-11	5.4E-06	0.415942	2.55E-24	2.39E-06
-0.45699	-0.35371	0.475554	-0.16115	0.210929	0.308705	-0.26658	0.620782	-0.45282
6.64E-08	4.52E-05	1.6E-08	0.07031	0.017294	0.000414	0.002449	6.96E-15	9.03E-08
-0.39926	-0.42868	0.11258	-0.55202	0.130948	0.058582	-0.4362	0.255272	-0.16346
3.32E-06	4.95E-07	0.207605	1.73E-11	0.142254	0.512971	2.96E-07	0.003774	0.066317
-0.46806	-0.54909	0.214684	-0.18224	0.528798	0.289894	-0.08361	0.516039	-0.27893
2.88E-08	2.32E-11	0.01536	0.040303	1.65E-10	0.000947	0.350003	5.32E-10	0.001495
0.103053	0.055039	-0.35061	-0.16942	-0.05542	-0.11066	-0.01183	-0.3708	0.282586
0.248936	0.538825	5.32E-05	0.056891	0.536035	0.215496	0.895008	1.78E-05	0.001287
-0.36113	-0.48983	-0.00976	-0.3781	0.278358	0.187065	-0.1609	0.108173	-0.15141
3.03E-05	5.08E-09	0.913306	1.17E-05	0.001531	0.035213	0.070742	0.226069	0.089279
0.024232	-0.01932	0.063865	0.360042	0.292004	0.030307	0.358645	0.145277	0.111269
0.786835	0.829316	0.475641	3.22E-05	0.000865	0.735179	3.47E-05	0.103173	0.21298

NR2F1	NRDE2	NSA2	NSL1	NSMCE2	NSUN3	NTNG2	NUDCD1	NUDT12
cyan	cyan	cyan	cyan	cyan	cyan	cyan	cyan	cyan
-0.06335	0.097689	0.104488	-0.11258	0.074753	0.055683	0.047575	-0.08443	-0.01011
0.479198	0.274557	0.242371	0.207611	0.40357	0.534078	0.595317	0.34532	0.910148
-0.15795	0.005295	0.060726	-0.09859	0.095146	0.043157	0.093291	-0.04861	0.008275
0.076143	0.952891	0.497639	0.270127	0.287301	0.629966	0.296848	0.587296	0.926434
0.007933	-0.12062	0.186131	0.28607	0.17028	0.333815	-0.23728	0.433274	0.050459
0.929463	0.17675	0.036154	0.001113	0.055622	0.000125	0.007229	3.62E-07	0.573179
-0.23678	-0.19899	0.368608	0.416481	0.294935	0.529899	-0.53515	0.58021	0.405021
0.007358	0.024909	2.01E-05	1.11E-06	0.000762	1.49E-10	9.07E-11	8.74E-13	2.32E-06
-0.41666	-0.29191	0.648105	0.511025	0.788557	0.24349	-0.16926	0.41925	0.354023
1.1E-06	0.000868	1.77E-16	8.32E-10	3.63E-28	0.005807	0.057126	9.3E-07	4.44E-05
-0.54739	-0.36993	0.722739	0.644062	0.741131	0.634791	-0.6316	0.73091	0.66164
2.75E-11	1.86E-05	8.61E-22	3.12E-16	2.24E-23	1.11E-15	1.7E-15	1.76E-22	2.5E-17
-0.45547	-0.35749	0.448082	0.238959	0.37354	0.328016	-0.32394	0.391794	0.233551
7.43E-08	3.69E-05	1.27E-07	0.006818	1.52E-05	0.000167	0.000203	5.24E-06	0.008227
0.09013	-0.24185	0.121706	0.322882	0.048861	0.219797	-0.47838	-0.01353	0.322941
0.313588	0.006157	0.172859	0.000214	0.585396	0.013031	1.28E-08	0.880031	0.000213
-0.03066	0.091642	0.145348	-0.22053	-0.07907	0.272173	-0.44389	-0.00855	-0.29009
0.732219	0.305507	0.103003	0.012724	0.376864	0.001964	1.72E-07	0.923975	0.000939
-0.17321	0.14644	0.301766	-0.04627	0.072013	0.279702	-0.4377	-0.25939	-0.05269
0.051491	0.100415	0.000565	0.605439	0.421069	0.001449	2.66E-07	0.003231	0.556329
-0.10313	0.116246	0.239279	0.103756	0.088646	0.259745	-0.4083	-0.04108	0.195888
0.248598	0.19309	0.006741	0.245704	0.321653	0.003188	1.89E-06	0.646585	0.027303
0.11547	0.337283	-0.25892	-0.51841	-0.3214	-0.4326	0.45508	-0.35066	-0.45368
0.196099	0.000105	0.003289	4.3E-10	0.000229	3.79E-07	7.65E-08	5.3E-05	8.48E-08
0.521335	0.336976	-0.45007	-0.46445	-0.62912	-0.23138	0.261635	-0.47054	-0.66788
3.29E-10	0.000107	1.1E-07	3.79E-08	2.36E-15	0.008862	0.002966	2.38E-08	9.77E-18
0.474463	0.295911	-0.2587	-0.17105	-0.2136	-0.36586	0.304374	-0.48879	-0.47088
1.75E-08	0.000731	0.003317	0.054505	0.015896	2.34E-05	0.000503	5.53E-09	2.31E-08
0.39306	0.438165	-0.26365	-0.4497	-0.51313	-0.1703	0.060464	-0.75929	-0.62779
4.85E-06	2.58E-07	0.002745	1.13E-07	6.9E-10	0.055595	0.499503	4.43E-25	2.81E-15
0.029393	0.221963	-0.345	-0.1407	-0.33667	-0.35098	0.569036	-0.53777	-0.10727
0.742881	0.012141	7.12E-05	0.11461	0.000109	5.22E-05	2.95E-12	7.05E-11	0.229968
0.362816	0.063687	-0.52005	-0.13007	-0.34803	-0.52138	0.711522	-0.18473	-0.25775
2.76E-05	0.47687	3.7E-10	0.144983	6.09E-05	3.28E-10	6.93E-21	0.037599	0.003438
-0.13736	-0.24483	-0.10618	0.260225	0.188284	-0.09604	0.182451	0.512243	0.505628
0.123572	0.005534	0.234805	0.00313	0.034017	0.282777	0.040067	7.47E-10	1.33E-09
0.068484	-0.11748	-0.39197	-0.14986	-0.12918	-0.48915	0.559283	0.011566	0.032705
0.444244	0.18839	5.18E-06	0.092638	0.14776	5.37E-09	8.23E-12	0.897313	0.715096
0.003865	-0.02747	0.19897	-0.12406	0.093884	0.147761	0.281014	0.189561	-0.33325
0.965605	0.75919	0.024924	0.164626	0.293776	0.097351	0.001373	0.0328	0.000129

NUDT15	NUDT6	NUMBL	NUP54	NUP88	NXN	NXT2	NYAP1	OBSCN
cyan	cyan	cyan	cyan	cyan	cyan	cyan	cyan	cyan
-0.00861	-0.028	-0.14597	-0.13778	-0.18239	-0.23774	-0.11903	-0.11285	0.085697
0.923431	0.75464	0.101532	0.12241	0.040136	0.007116	0.182556	0.206501	0.338076
0.062633	0.043835	-0.14024	-0.15496	-0.11209	-0.24679	-0.188	-0.01803	0.010483
0.484212	0.624594	0.115817	0.081938	0.209603	0.005156	0.034291	0.840548	0.90688
0.308392	0.312714	-0.14445	0.563439	0.621214	0.219521	0.374459	-0.33127	-0.20753
0.00042	0.000344	0.105169	5.34E-12	6.59E-15	0.013148	1.44E-05	0.000142	0.019224
0.401089	0.356833	-0.43137	0.826734	0.574187	-0.25873	0.617111	-0.55902	-0.44603
2.97E-06	3.82E-05	4.12E-07	5.11E-33	1.69E-12	0.003314	1.11E-14	8.46E-12	1.48E-07
0.334251	0.388106	-0.44772	0.358609	0.427274	-0.12082	0.264114	-0.23615	-0.37457
0.000123	6.53E-06	1.31E-07	3.47E-05	5.45E-07	0.176025	0.002696	0.00752	1.43E-05
0.555566	0.471519	-0.70009	0.885488	0.601105	-0.45085	0.677966	-0.64002	-0.63855
1.21E-11	2.2E-08	5.25E-20	1.98E-43	7.9E-14	1.04E-07	2.04E-18	5.46E-16	6.67E-16
0.414095	0.210589	-0.46189	0.531057	0.385349	-0.38881	0.319607	-0.34481	-0.31688
1.3E-06	0.017479	4.6E-08	1.34E-10	7.69E-06	6.26E-06	0.00025	7.19E-05	0.000284
-0.00294	-0.06023	-0.23634	0.239643	-0.16687	-0.40835	0.409716	-0.05957	-0.23153
0.973821	0.501188	0.007471	0.006656	0.060783	1.88E-06	1.72E-06	0.505899	0.008815
0.155802	0.03329	0.018401	0.274731	0.046388	0.065684	0.15882	-0.56017	0.030696
0.080267	0.710226	0.837306	0.001773	0.604543	0.463136	0.074512	7.51E-12	0.731906
-0.1402	-0.01325	-0.22744	0.077983	-0.27524	-0.31404	0.168143	-0.53792	-0.18069
0.115924	0.882481	0.010122	0.3835	0.001737	0.000324	0.05881	6.95E-11	0.042064
-0.08523	0.075549	-0.24807	0.215545	-0.15876	-0.42384	0.340801	-0.51874	-0.17437
0.340717	0.398568	0.004922	0.014944	0.074629	6.86E-07	8.83E-05	4.17E-10	0.049922
-0.26554	0.261542	0.390603	-0.38741	-0.0729	0.247745	-0.44043	0.071437	0.34277
0.00255	0.002976	5.62E-06	6.8E-06	0.415332	0.004981	2.2E-07	0.424803	7.99E-05
-0.36628	-0.14227	0.485265	-0.30439	-0.15142	0.470012	-0.29785	0.146059	0.395152
2.28E-05	0.110582	7.38E-09	0.000503	0.08925	2.47E-08	0.000672	0.101311	4.27E-06
-0.40713	-0.12229	0.48631	-0.44768	-0.25832	0.48261	-0.26261	0.256892	0.229071
2.03E-06	0.170797	6.78E-09	1.31E-07	0.003365	9.15E-09	0.002857	0.003551	0.009583
-0.54695	-0.21193	0.358448	-0.44572	-0.52386	0.262506	-0.26553	-0.06397	0.27517
2.87E-11	0.016761	3.5E-05	1.51E-07	2.61E-10	0.002868	0.002552	0.474902	0.001742
-0.39528	-0.19369	0.06485	-0.64811	-0.46226	-0.00347	-0.47053	0.437382	0.15033
4.24E-06	0.029113	0.468848	1.77E-16	4.48E-08	0.969111	2.38E-08	2.72E-07	0.091607
-0.21148	-0.15892	0.427684	-0.48748	0.008983	0.446099	-0.35346	0.66926	0.320572
0.016998	0.074322	5.3E-07	6.16E-09	0.920161	1.47E-07	4.57E-05	7.92E-18	0.000238
0.328148	-0.05189	-0.07473	0.09528	0.207571	-0.11819	0.06183	0.342541	-0.06937
0.000166	0.562307	0.403688	0.286624	0.019199	0.185717	0.489838	8.08E-05	0.438387
-0.00938	-0.30661	0.306202	-0.50139	-0.16212	0.100534	-0.41784	0.717588	0.311278
0.916661	0.000455	0.000463	1.92E-09	0.068604	0.260753	1.02E-06	2.27E-21	0.000368
0.246353	0.222098	-0.12925	0.156068	0.489739	0.519903	-0.1851	-0.06209	0.21968
0.005239	0.012087	0.147552	0.079747	5.11E-09	3.75E-10	0.037213	0.48805	0.01308

OGDH	OMA1	OPN1SW	OR2B6	ORAI2	ORC3	ORC5	ORMDL1	OSBPL9
cyan	cyan	cyan	cyan	cyan	cyan	cyan	cyan	cyan
-0.12109	0.121161	0.069188	0.115593	-0.2951	0.140078	0.003461	0.038225	0.140645
0.17507	0.174807	0.439565	0.195622	0.000757	0.116241	0.969191	0.66962	0.114756
-0.13399	0.128303	0.20301	0.173902	-0.4064	0.219242	0.087244	0.088645	0.031117
0.13313	0.15056	0.022075	0.050544	2.13E-06	0.013267	0.329396	0.321659	0.728375
-0.1931	0.070579	0.160586	0.231587	0.075297	0.077656	0.341951	0.24463	0.129006
0.029623	0.4304	0.071303	0.008799	0.400149	0.385503	8.33E-05	0.005574	0.148319
-0.38489	0.474136	0.315517	0.309385	-0.2075	0.396947	0.582693	0.593015	0.486757
7.89E-06	1.79E-08	0.000302	0.000401	0.019243	3.83E-06	6.63E-13	2.05E-13	6.53E-09
-0.48421	0.392454	0.455111	0.413924	-0.441	0.562696	0.519743	0.458188	0.008637
8.03E-09	5.03E-06	7.63E-08	1.32E-06	2.11E-07	5.77E-12	3.81E-10	6.07E-08	0.923224
-0.63925	0.71022	0.549848	0.507772	-0.53862	0.768504	0.821542	0.825724	0.513153
6.06E-16	8.77E-21	2.15E-11	1.11E-09	6.5E-11	5.3E-26	2.72E-32	7.1E-33	6.89E-10
-0.37194	0.537704	0.359996	0.349225	-0.32836	0.489845	0.509972	0.612699	0.338084
1.67E-05	7.1E-11	3.22E-05	5.72E-05	0.000164	5.07E-09	9.12E-10	1.93E-14	0.000101
-0.01366	0.494462	0.259603	0.265217	-0.11735	0.430732	0.106868	0.27137	0.427585
0.878821	3.45E-09	0.003205	0.002583	0.188868	4.31E-07	0.231752	0.002028	5.33E-07
-0.00606	0.121008	-0.2285	0.011397	0.258534	-0.24427	-0.00042	0.165758	0.428936
0.946112	0.175357	0.009771	0.898803	0.003338	0.005647	0.996241	0.062542	4.87E-07
-0.09567	0.378167	-0.05858	0.131346	-0.10984	-0.04013	-0.13804	0.235055	0.511026
0.284622	1.17E-05	0.512997	0.141035	0.218946	0.654188	0.121692	0.007811	8.31E-10
-0.06705	0.356699	-0.01606	0.153504	-0.17673	0.105696	0.082778	0.256812	0.533577
0.453836	3.85E-05	0.857801	0.084883	0.046852	0.236941	0.354851	0.003562	1.05E-10
0.311807	-0.28486	-0.52709	-0.43856	0.230762	-0.50518	-0.25743	-0.39792	-0.15883
0.000359	0.001171	1.94E-10	2.51E-07	0.00905	1.39E-09	0.00348	3.61E-06	0.074486
0.423065	-0.39093	-0.39099	-0.272	0.519489	-0.71117	-0.56344	-0.46408	-0.14517
7.22E-07	5.51E-06	5.5E-06	0.001978	3.9E-10	7.39E-21	5.34E-12	3.9E-08	0.103421
0.371595	-0.37029	-0.20884	-0.17955	0.401167	-0.60586	-0.55039	-0.51499	-0.45598
1.7E-05	1.83E-05	0.01846	0.043396	2.95E-06	4.46E-14	2.04E-11	5.85E-10	7.16E-08
0.387595	-0.16279	-0.38941	-0.20599	0.385147	-0.71066	-0.74052	-0.39395	0.066106
6.73E-06	0.067448	6.04E-06	0.020159	7.78E-06	8.1E-21	2.53E-23	4.6E-06	0.46026
0.1582	-0.1855	-0.09026	-0.17837	-0.14493	-0.15116	-0.45326	-0.33895	-0.23975
0.075666	0.036797	0.312882	0.044821	0.104008	0.089819	8.74E-08	9.7E-05	0.00663
0.331915	-0.63957	-0.19553	-0.34104	0.31171	-0.36983	-0.32176	-0.56651	-0.62513
0.000138	5.8E-16	0.027596	8.72E-05	0.000361	1.88E-05	0.000225	3.86E-12	3.97E-15
-0.11052	-0.19158	0.152502	-0.03389	-0.14547	0.386819	0.400178	0.02386	-0.31798
0.216077	0.030956	0.086962	0.705273	0.102708	7.05E-06	3.14E-06	0.790031	0.000269
0.224253	-0.51073	-0.12347	-0.28012	0.090738	0.013679	-0.07508	-0.40351	-0.60466
0.011258	8.54E-10	0.166655	0.001424	0.310319	0.878682	0.401512	2.55E-06	5.16E-14
-0.33191	-0.12389	-0.05142	0.05192	-0.00604	-0.09592	0.035647	0.029747	-0.12724
0.000138	0.165219	0.565888	0.562106	0.946282	0.283404	0.690724	0.739894	0.153995

OSER1	OSGEPL1	OSTC	OSTM1	OSTN	OXCT2	OXNAD1	OXSM	PACRGL
cyan	cyan	cyan	cyan	cyan	cyan	cyan	cyan	cyan
0.240999	-0.02913	-0.03693	-0.05579	-0.15784	0.037571	0.106876	0.019222	-0.00167
0.006345	0.745128	0.680197	0.533276	0.076338	0.674951	0.231713	0.830159	0.985129
0.296792	0.028429	-0.07546	-0.11929	-0.16212	0.096832	0.134447	0.063574	0.063
0.000703	0.751036	0.399097	0.18162	0.068606	0.278806	0.131807	0.477655	0.481649
-0.04247	0.460722	0.397014	0.075446	0.115555	0.094294	0.145474	0.356696	0.15893
0.635469	5.02E-08	3.81E-06	0.399212	0.195768	0.29166	0.102702	3.85E-05	0.07431
0.278815	0.63313	0.620634	0.424419	0.363541	-0.23937	0.437623	0.563505	0.412589
0.001502	1.39E-15	7.1E-15	6.6E-07	2.66E-05	0.006721	2.68E-07	5.3E-12	1.43E-06
0.705587	0.455285	0.434848	0.260409	0.321311	-0.15404	0.430618	0.665335	0.401845
2.01E-20	7.53E-08	3.25E-07	0.003108	0.00023	0.083796	4.34E-07	1.44E-17	2.83E-06
0.735014	0.759293	0.810538	0.647847	0.553524	-0.42541	0.677793	0.842904	0.645528
7.79E-23	4.43E-25	7.95E-31	1.84E-16	1.49E-11	6.18E-07	2.1E-18	1.9E-35	2.55E-16
0.626192	0.442819	0.499613	0.240076	0.282635	-0.13857	0.501633	0.585146	0.330802
3.46E-15	1.86E-07	2.24E-09	0.006555	0.001284	0.120263	1.88E-09	5.03E-13	0.000146
0.1684	0.099118	0.105044	0.385964	0.417755	-0.45576	0.190649	0.245286	0.275023
0.058419	0.267565	0.239859	7.41E-06	1.03E-06	7.27E-08	0.031794	0.005444	0.001752
-0.05347	-0.05312	0.621564	0.229113	-0.23659	0.039055	0.007453	0.067408	-0.24897
0.550509	0.553105	6.3E-15	0.00957	0.007406	0.662875	0.933723	0.451451	0.004764
0.207165	-0.08601	0.435857	0.34008	-0.0629	-0.23291	0.114445	0.122015	-0.21254
0.019441	0.336302	3.03E-07	9.16E-05	0.482372	0.008409	0.200125	0.171763	0.016442
0.173043	0.065711	0.365576	0.344889	0.128491	-0.31588	0.167695	0.160315	-0.00377
0.051713	0.462949	2.38E-05	7.16E-05	0.149959	0.000297	0.059496	0.071789	0.966479
-0.29344	-0.2369	-0.25251	-0.64962	-0.51155	0.417411	-0.39604	-0.3392	-0.43212
0.000813	0.007327	0.004182	1.43E-16	7.94E-10	1.05E-06	4.05E-06	9.58E-05	3.92E-07
-0.64451	-0.31875	-0.31658	-0.52301	-0.51123	0.462001	-0.44903	-0.43462	-0.55585
2.93E-16	0.00026	0.000288	2.83E-10	8.16E-10	4.56E-08	1.19E-07	3.3E-07	1.17E-11
-0.37357	-0.35978	-0.29344	-0.29853	-0.4013	0.361876	-0.40893	-0.31056	-0.48945
1.52E-05	3.26E-05	0.000813	0.000652	2.93E-06	2.91E-05	1.81E-06	0.00038	5.24E-09
-0.45789	-0.49033	-0.13982	-0.23943	-0.54806	0.271544	-0.41364	-0.4276	-0.70848
6.21E-08	4.87E-09	0.116912	0.006706	2.57E-11	0.002014	1.34E-06	5.33E-07	1.2E-20
-0.25315	-0.29282	-0.66372	-0.34616	-0.24156	0.146741	-0.24497	-0.43584	-0.27656
0.004085	0.000835	1.83E-17	6.71E-05	0.00622	0.099711	0.005507	3.03E-07	0.001647
-0.53598	-0.23068	-0.71086	-0.5985	-0.20555	0.358687	-0.43126	-0.45377	-0.18292
8.38E-11	0.009075	7.81E-21	1.08E-13	0.02043	3.46E-05	4.15E-07	8.42E-08	0.039549
0.080076	0.169692	-0.22678	0.141243	0.369042	-0.1779	0.121374	0.003957	0.48775
0.370825	0.056484	0.010349	0.113206	1.96E-05	0.045398	0.174043	0.964779	6.02E-09
-0.16283	-0.32096	-0.65377	-0.32072	0.050372	0.062767	-0.24426	-0.39531	0.087056
0.067385	0.000234	7.9E-17	0.000237	0.573838	0.483276	0.005648	4.23E-06	0.330444
0.012349	0.257306	0.034026	-0.38045	-0.21603	0.53396	0.097587	0.189941	0.019618
0.890405	0.003496	0.704116	1.02E-05	0.014713	1.02E-10	0.275061	0.032446	0.826714

PAIP1	PAK1IP1	PALM	PAN3	PANK4	PBX3	PCDH1	PCGF6	PCID2
cyan	cyan	cyan	cyan	cyan	cyan	cyan	cyan	cyan
-0.05779	-0.04048	0.00014	-0.1687	-0.29759	-0.19034	-0.01928	-0.08898	-0.02469
0.518659	0.651391	0.998754	0.057959	0.000679	0.032081	0.829666	0.319806	0.782928
-0.01925	-0.05178	0.063279	-0.20726	-0.24349	-0.2482	0.019968	-0.05585	0.009043
0.829937	0.563199	0.479705	0.019386	0.005807	0.004898	0.823675	0.532828	0.91963
0.491787	0.417563	-0.32578	0.239156	0.187403	0.315011	-0.16022	0.460396	0.306102
4.32E-09	1.04E-06	0.000186	0.006771	0.034878	0.00031	0.071966	5.15E-08	0.000465
0.647978	0.616152	-0.68115	0.47699	-0.26963	0.619749	-0.33142	0.52293	0.444984
1.81E-16	1.25E-14	1.23E-18	1.43E-08	0.002173	7.94E-15	0.000141	2.85E-10	1.59E-07
0.435606	0.566993	-0.10185	-0.18107	-0.04335	0.068225	-0.43554	0.255965	0.602775
3.08E-07	3.67E-12	0.25455	0.041624	0.628438	0.445978	3.09E-07	0.003677	6.47E-14
0.769613	0.844569	-0.69461	0.276665	-0.44745	0.61247	-0.56459	0.585208	0.681783
4.07E-26	1.03E-35	1.34E-19	0.00164	1.33E-07	1.98E-14	4.73E-12	5E-13	1.11E-18
0.426562	0.48739	-0.39306	0.241826	-0.34749	0.3886	-0.10874	0.183003	0.546354
5.71E-07	6.2E-09	4.85E-06	0.006161	6.26E-05	6.34E-06	0.223628	0.039459	3.05E-11
0.072862	0.078544	-0.27525	0.120934	-0.45891	0.35636	-0.39133	-0.16989	-0.0003
0.4156	0.380079	0.001736	0.175623	5.76E-08	3.92E-05	5.39E-06	0.0562	0.997305
0.064134	0.113943	-0.55981	0.164488	-0.15123	0.382628	0.033642	0.123332	0.030511
0.473778	0.202121	7.8E-12	0.064608	0.089655	9.02E-06	0.7073	0.167149	0.733462
-0.14966	0.013265	-0.43791	0.120262	-0.57503	0.380725	-0.30042	-0.03455	-0.05838
0.093074	0.882329	2.62E-07	0.178054	1.55E-12	1.01E-05	0.0006	0.699768	0.514404
0.056254	0.20691	-0.49611	0.133091	-0.55251	0.40666	-0.34347	0.008313	-0.01738
0.529882	0.019594	3.01E-09	0.135785	1.65E-11	2.09E-06	7.71E-05	0.926097	0.84622
-0.15123	-0.30036	0.114937	-0.02994	0.335771	-0.18954	0.393645	-0.37602	-0.16618
0.089661	0.000601	0.198187	0.738244	0.000114	0.032818	4.68E-06	1.32E-05	0.061866
-0.36921	-0.48169	0.19163	0.075003	0.378506	-0.20824	0.30208	-0.22348	-0.39609
1.94E-05	9.85E-09	0.030908	0.401996	1.15E-05	0.018803	0.000557	0.011551	4.04E-06
-0.45771	-0.37913	0.394642	-0.33487	0.546215	-0.38574	0.043504	-0.21168	-0.30467
6.29E-08	1.1E-05	4.41E-06	0.000119	3.09E-11	7.51E-06	0.627218	0.016889	0.000496
-0.59898	-0.55106	0.075668	-0.04459	0.021738	-0.11031	0.048188	-0.31726	-0.51504
1.02E-13	1.91E-11	0.397823	0.618658	0.808328	0.21697	0.590584	0.000279	5.82E-10
-0.45583	-0.50687	0.613819	-0.16674	-0.0261	-0.4578	0.19854	-0.31314	-0.39998
7.23E-08	1.2E-09	1.68E-14	0.060984	0.770816	6.25E-08	0.025245	0.000338	3.18E-06
-0.22307	-0.37544	0.736956	-0.20474	0.603057	-0.51618	0.45045	-0.24194	-0.25355
0.011708	1.37E-05	5.26E-23	0.020943	6.26E-14	5.25E-10	1.07E-07	0.006137	0.004025
0.273062	0.203938	0.195604	0.00395	0.170554	-0.09331	0.17452	0.179184	0.167232
0.001896	0.021462	0.027532	0.964845	0.055223	0.296736	0.049718	0.043834	0.060212
-0.21068	-0.28998	0.657398	-0.32248	0.349428	-0.51789	0.470601	-0.31092	-0.16046
0.017427	0.000943	4.67E-17	0.000218	5.66E-05	4.51E-10	2.36E-08	0.000374	0.071536
0.270921	0.065884	0.195383	0.206792	0.404353	-0.30005	0.175724	0.218489	0.395976
0.002065	0.461773	0.027711	0.019666	2.42E-06	0.00061	0.04814	0.013595	4.06E-06

PCNP	PCNT	PDCD2	PDCL3	PDGFRL	PDK1	PDSS1	PDSS2	PECR
cyan	cyan	cyan	cyan	cyan	cyan	cyan	cyan	cyan
-0.0766	-0.2327	-0.0858	0.048581	0.101237	0.034115	-0.05682	0.020946	-0.09062
0.391993	0.008471	0.3375	0.58755	0.257418	0.70338	0.525736	0.815186	0.310926
-0.11602	-0.2471	-0.05051	0.055547	0.074762	0.041255	0.021265	-0.03477	-0.13312
0.193976	0.005098	0.57277	0.535079	0.40351	0.645145	0.812427	0.697959	0.135712
0.268079	0.044088	0.336607	0.217879	-0.00786	0.25149	0.491958	0.362161	0.179107
0.00231	0.622597	0.000109	0.013865	0.930087	0.004343	4.26E-09	2.87E-05	0.043925
0.61327	-0.19043	0.576046	0.282106	0.373967	0.554605	0.37922	0.644109	0.433289
1.8E-14	0.031991	1.38E-12	0.001312	1.48E-05	1.33E-11	1.1E-05	3.1E-16	3.61E-07
0.165746	-0.56137	0.634987	0.510934	0.215862	0.294157	0.494062	0.209567	0.426544
0.062562	6.63E-12	1.08E-15	8.38E-10	0.014794	0.000789	3.57E-09	0.018046	5.72E-07
0.68092	-0.58385	0.876275	0.558888	0.572592	0.679261	0.491724	0.719488	0.647726
1.28E-18	5.83E-13	1.86E-41	8.58E-12	2.01E-12	1.66E-18	4.34E-09	1.59E-21	1.87E-16
0.327663	-0.40807	0.582837	0.349548	0.419585	0.406251	0.27277	0.433773	0.42091
0.00017	1.91E-06	6.52E-13	5.62E-05	9.09E-07	2.15E-06	0.001918	3.5E-07	8.33E-07
0.215175	-0.05376	0.250123	0.120621	0.119848	0.110377	-0.17457	0.217417	0.240784
0.015122	0.548345	0.004567	0.17675	0.179561	0.216696	0.049652	0.014073	0.006393
0.028592	-0.12645	0.136875	0.265519	0.399622	0.335954	-0.14196	0.273929	0.164526
0.749652	0.156602	0.124911	0.002553	3.25E-06	0.000113	0.111378	0.001831	0.064545
-0.08697	-0.39218	0.134903	0.379848	0.357103	0.100588	-0.1658	0.190632	0.280927
0.330951	5.11E-06	0.130491	1.06E-05	3.77E-05	0.260495	0.062475	0.031809	0.001378
0.199331	-0.3931	0.231387	0.201169	0.394099	0.161632	-0.11133	0.438452	0.276664
0.024657	4.84E-06	0.008859	0.023336	4.56E-06	0.069456	0.212745	2.53E-07	0.00164
-0.34689	0.15727	-0.31513	-0.3792	-0.03486	-0.16621	-0.15315	-0.21192	-0.25012
6.46E-05	0.077425	0.000308	1.1E-05	0.697199	0.061821	0.085605	0.016766	0.004568
-0.49195	0.501088	-0.53072	-0.25041	-0.42963	-0.36187	-0.1313	-0.30992	-0.35514
4.26E-09	1.97E-09	1.38E-10	0.004519	4.64E-07	2.91E-05	0.14117	0.000391	4.19E-05
-0.52124	0.142681	-0.40678	-0.04573	-0.42273	-0.39364	-0.08553	-0.44829	-0.14658
3.32E-10	0.109547	2.08E-06	0.609722	7.39E-07	4.68E-06	0.339016	1.26E-07	0.100085
-0.61773	0.139377	-0.48807	0.001028	-0.22225	-0.38404	-0.36184	-0.32387	-0.16709
1.03E-14	0.118097	5.87E-09	0.990845	0.012027	8.3E-06	2.92E-05	0.000204	0.060435
-0.43098	0.106327	-0.44899	-0.20095	-0.4155	-0.57925	-0.27044	-0.56026	-0.39932
4.24E-07	0.234134	1.19E-07	0.023489	1.19E-06	9.72E-13	0.002105	7.44E-12	3.31E-06
-0.28124	0.451054	-0.46342	-0.48131	-0.6415	-0.50518	0.043154	-0.51932	-0.47383
0.00136	1.03E-07	4.1E-08	1.02E-08	4.45E-16	1.39E-09	0.629996	3.96E-10	1.84E-08
0.515128	0.117655	0.082268	-0.3286	0.019914	0.099803	0.10349	0.071091	-0.10445
5.77E-10	0.187712	0.35783	0.000162	0.824143	0.264257	0.246925	0.427058	0.242551
-0.07134	0.337637	-0.34287	-0.5021	-0.29982	-0.31501	-0.17859	-0.42454	-0.4429
0.425428	0.000104	7.94E-05	1.81E-09	0.000616	0.00031	0.044548	6.55E-07	1.85E-07
-0.18435	0.209798	0.005384	0.131097	-0.25016	0.002868	0.525998	-0.17791	-0.10666
0.038008	0.017917	0.952096	0.141797	0.00456	0.974474	2.15E-10	0.04538	0.232677

PELP1	PEX12	PEX2	PEX3	PFKP	PFN4	PGAP3	PHF5A	PI4K2B
cyan	cyan	cyan	cyan	cyan	cyan	cyan	cyan	cyan
-0.11261	-0.01473	0.047067	0.15213	0.035652	-0.03196	0.034953	-0.01854	-0.03608
0.207503	0.869479	0.599258	0.087743	0.690684	0.721327	0.696447	0.836137	0.687196
-0.11146	-0.00202	0.055225	0.168997	0.124311	-0.05787	0.038242	0.001595	-0.13695
0.21218	0.981977	0.537448	0.057518	0.163779	0.518119	0.669482	0.985802	0.124709
0.008729	0.103875	0.067606	0.158398	-0.15424	0.352762	-0.16947	0.30843	0.35667
0.922409	0.245162	0.450118	0.075296	0.083374	4.75E-05	0.056812	0.000419	3.86E-05
-0.49897	0.459894	0.460446	0.536961	-0.44215	0.573316	-0.50649	0.405071	0.712477
2.36E-09	5.35E-08	5.13E-08	7.62E-11	1.95E-07	1.86E-12	1.24E-09	2.31E-06	5.82E-21
-0.40759	0.185173	0.569849	0.451003	-0.15524	0.283328	-0.06625	0.701083	0.074191
1.97E-06	0.03714	2.71E-12	1.03E-07	0.081374	0.001248	0.459262	4.42E-20	0.407125
-0.83677	0.541235	0.822861	0.829728	-0.54447	0.642785	-0.56782	0.719148	0.698403
1.7E-34	5.04E-11	1.79E-32	1.9E-33	3.67E-11	3.73E-16	3.36E-12	1.7E-21	7.03E-20
-0.58601	0.402727	0.461212	0.470823	-0.3124	0.382346	-0.22884	0.404184	0.345072
4.56E-13	2.68E-06	4.84E-08	2.32E-08	0.000349	9.17E-06	0.009658	2.45E-06	7.1E-05
-0.44432	0.337201	0.379022	0.29284	-0.37371	0.411258	-0.3453	0.110593	0.268642
1.67E-07	0.000106	1.11E-05	0.000835	1.51E-05	1.56E-06	7.01E-05	0.21579	0.002259
-0.08309	0.047339	-0.08467	-0.04894	-0.31851	0.270719	-0.07095	-0.0391	0.494768
0.353053	0.597151	0.343922	0.584804	0.000263	0.002081	0.427993	0.662499	3.37E-09
-0.26313	0.079953	0.155462	0.008619	-0.46327	0.257718	-0.11339	0.041467	0.483912
0.0028	0.371563	0.080937	0.923385	4.14E-08	0.003442	0.204336	0.643442	8.23E-09
-0.35974	0.236623	0.284229	0.257285	-0.49353	0.289292	-0.26218	0.097195	0.585817
3.27E-05	0.007398	0.001202	0.003499	3.73E-09	0.000971	0.002904	0.277002	4.67E-13
0.429889	0.003156	-0.33797	-0.36304	0.243813	-0.24428	0.277363	-0.23549	-0.3458
4.56E-07	0.971911	0.000102	2.73E-05	0.00574	0.005644	0.001594	0.007694	6.83E-05
0.75032	-0.30383	-0.69784	-0.67686	0.259231	-0.18062	0.365726	-0.45434	-0.18195
3.21E-24	0.000515	7.74E-20	2.43E-18	0.003251	0.04214	2.36E-05	8.07E-08	0.040632
0.730646	-0.37168	-0.46587	-0.63823	0.303558	-0.26597	0.518125	-0.15769	-0.31226
1.86E-22	1.69E-05	3.4E-08	6.97E-16	0.000522	0.002508	4.41E-10	0.076629	0.000352
0.575656	-0.31917	-0.54265	-0.69378	0.017156	-0.13382	0.349104	-0.42322	-0.00861
1.44E-12	0.000255	4.39E-11	1.54E-19	0.848182	0.133639	5.76E-05	7.15E-07	0.923451
0.297956	-0.26702	-0.25126	-0.34894	0.303581	-0.4045	0.255857	-0.30771	-0.54146
0.000668	0.002408	0.004381	5.81E-05	0.000521	2.4E-06	0.003692	0.000433	4.93E-11
0.63033	-0.33829	-0.46411	-0.44821	0.531088	-0.50204	0.341935	-0.25749	-0.68083
2.01E-15	0.0001	3.89E-08	1.26E-07	1.33E-10	1.82E-09	8.33E-05	0.003473	1.29E-18
-0.28042	0.081547	0.240442	0.397881	0.165951	-0.17841	-0.20156	0.077483	-0.18411
0.001407	0.362075	0.006471	3.62E-06	0.062233	0.044774	0.023062	0.386571	0.038266
0.160824	-0.22433	-0.18889	-0.08962	0.512693	-0.52815	0.212742	-0.25341	-0.72617
0.070879	0.011229	0.033437	0.316331	7.17E-10	1.76E-10	0.016335	0.004045	4.45E-22
0.307148	-0.27879	-0.2715	-0.13987	0.246163	-0.0481	0.225513	0.170838	-0.15415
0.000444	0.001504	0.002018	0.116784	0.005275	0.591283	0.010797	0.054813	0.083563

PIBF1	PIGA	PIGB	PIGC	PIGF	PIGH	PIGK	PIGN	PIGP
cyan	cyan	cyan	cyan	cyan	cyan	cyan	cyan	cyan
-0.11625	0.165963	0.243084	0.237792	-0.05236	0.27147	0.222408	-0.103	0.349742
0.193062	0.062214	0.005891	0.007102	0.558784	0.00202	0.011964	0.249188	5.57E-05
-0.12152	0.161351	0.255383	0.235186	0.051633	0.367068	0.189844	-0.13961	0.353491
0.173532	0.069947	0.003758	0.007776	0.564274	2.19E-05	0.032537	0.11749	4.57E-05
0.389729	0.118045	-0.11251	0.043496	0.456315	0.040473	-0.05021	0.400953	-0.23948
5.93E-06	0.186244	0.207876	0.627281	6.98E-08	0.651422	0.575053	2.99E-06	0.006693
0.671476	0.463662	0.359292	0.277652	0.512171	0.245945	0.423992	0.765649	0.069283
5.63E-18	4.02E-08	3.35E-05	0.001575	7.51E-10	0.005317	6.79E-07	1.03E-25	0.438938
0.390338	0.165408	0.303859	0.612091	0.496191	0.544449	0.377789	0.092603	0.697062
5.71E-06	0.063105	0.000515	2.08E-14	2.99E-09	3.68E-11	1.19E-05	0.300438	8.84E-20
0.811262	0.586376	0.677817	0.680072	0.655702	0.559969	0.771484	0.756559	0.626614
6.41E-31	4.38E-13	2.09E-18	1.46E-18	5.97E-17	7.67E-12	2.61E-26	8.17E-25	3.28E-15
0.528168	0.392476	0.492384	0.527507	0.520959	0.325219	0.493185	0.463506	0.432213
1.75E-10	5.03E-06	4.11E-09	1.86E-10	3.41E-10	0.000191	3.84E-09	4.07E-08	3.89E-07
0.316618	0.137034	0.25468	0.416916	-0.03556	-0.07239	0.324176	0.224734	0.354198
0.000287	0.124468	0.003858	1.08E-06	0.691468	0.418631	0.000201	0.01108	4.4E-05
0.174033	0.038198	0.214089	0.18638	0.107501	-0.11007	0.009148	0.357674	-0.09301
0.050368	0.669838	0.015653	0.035901	0.228984	0.217991	0.918699	3.65E-05	0.298294
-0.01509	0.141776	0.475234	0.386408	-0.0075	0.014268	0.205743	0.184753	0.410528
0.866264	0.111838	1.65E-08	7.22E-06	0.933309	0.873501	0.02031	0.03758	1.64E-06
0.101975	0.223704	0.459712	0.323806	0.02204	0.136496	0.312013	0.294473	0.260696
0.253949	0.011464	5.42E-08	0.000204	0.805723	0.12597	0.000356	0.000778	0.003074
-0.39472	-0.37091	-0.20181	-0.36454	-0.01509	0.047919	-0.55204	-0.33241	-0.51835
4.39E-06	1.77E-05	0.022892	2.52E-05	0.866255	0.592659	1.73E-11	0.000134	4.32E-10
-0.43251	-0.38185	-0.59356	-0.47993	-0.20124	-0.48473	-0.7791	-0.30865	-0.71017
3.81E-07	9.43E-06	1.92E-13	1.13E-08	0.023285	7.7E-09	4.06E-27	0.000415	8.85E-21
-0.54793	-0.51609	-0.58484	-0.30239	-0.29445	-0.3265	-0.68959	-0.58338	-0.31011
2.61E-11	5.3E-10	5.21E-13	0.00055	0.000779	0.00018	3.11E-19	6.14E-13	0.000388
-0.58103	-0.37451	-0.28084	-0.20804	-0.35014	-0.47488	-0.59856	-0.36772	-0.27763
7.98E-13	1.44E-05	0.001383	0.01892	5.45E-05	1.69E-08	1.07E-13	2.11E-05	0.001577
-0.55814	-0.281	-0.19577	-0.30044	-0.33621	-0.14686	-0.17329	-0.52476	0.047393
9.26E-12	0.001374	0.027399	0.000599	0.000111	0.099436	0.051371	2.41E-10	0.596732
-0.41356	-0.42579	-0.75317	-0.61888	-0.2254	-0.25882	-0.56674	-0.55011	-0.61466
1.35E-06	6.02E-07	1.73E-24	8.87E-15	0.010837	0.003301	3.77E-12	2.1E-11	1.51E-14
0.241532	0.181782	0.019499	-0.20176	-0.04405	0.182602	0.390662	0.148513	-0.04255
0.006226	0.040816	0.827751	0.022927	0.622908	0.0399	5.6E-06	0.095641	0.634828
-0.25036	-0.20432	-0.30549	-0.36959	-0.34785	-0.03836	-0.07011	-0.43196	-0.17655
0.004528	0.021215	0.000479	1.9E-05	6.15E-05	0.668505	0.433479	3.96E-07	0.047081
0.174175	0.015291	-0.3539	-0.07762	0.418154	0.043206	-0.30009	-0.12914	-0.15235
0.050179	0.864515	4.47E-05	0.385739	9.99E-07	0.629579	0.000609	0.147883	0.087278

PIGU	PIGV	PIK3C3	PIP5K1C	PITPNM1	PITPNM2	PKD1	PKIB	PLA2G5
cyan	cyan	cyan	cyan	cyan	cyan	cyan	cyan	cyan
0.055812	0.237858	-0.01015	-0.10138	0.017301	-0.01057	-0.18124	-0.00944	0.131437
0.533131	0.007086	0.909792	0.256747	0.846915	0.906097	0.041427	0.916068	0.140759
0.131842	0.277615	-0.06365	-0.0895	-0.0306	0.008992	-0.20272	-0.06461	0.175803
0.139528	0.001578	0.477112	0.316993	0.7327	0.920081	0.022272	0.470464	0.048038
0.188542	-0.1899	0.223168	-0.20066	-0.46829	-0.24516	-0.06188	0.194656	-0.17049
0.033768	0.03248	0.011669	0.023693	2.83E-08	0.00547	0.489467	0.028308	0.055317
0.324548	0.255891	0.612074	-0.61479	-0.6849	-0.46316	-0.4169	0.387846	0.20902
0.000197	0.003687	2.08E-14	1.48E-14	6.71E-19	4.18E-08	1.08E-06	6.63E-06	0.018356
0.62466	0.417169	0.321178	-0.52065	-0.2843	-0.55287	-0.57394	0.371528	0.272368
4.23E-15	1.07E-06	0.000232	3.51E-10	0.001198	1.59E-11	1.74E-12	1.7E-05	0.001949
0.641025	0.606299	0.792899	-0.92166	-0.71521	-0.76038	-0.81813	0.558822	0.468894
4.76E-16	4.23E-14	1.15E-28	3.1E-53	3.53E-21	3.46E-25	7.95E-32	8.63E-12	2.7E-08
0.52008	0.458558	0.519571	-0.59835	-0.47689	-0.4601	-0.55596	0.356693	0.356049
3.69E-10	5.91E-08	3.87E-10	1.1E-13	1.44E-08	5.27E-08	1.16E-11	3.85E-05	3.99E-05
0.088841	0.555843	0.436116	-0.46999	0.01635	-0.28079	-0.26107	0.168854	0.228005
0.320585	1.17E-11	2.97E-07	2.48E-08	0.855232	0.001386	0.003031	0.057732	0.009934
0.041883	0.007757	0.077647	-0.13509	-0.07935	-0.29476	0.042444	0.13109	0.13237
0.640114	0.931023	0.385561	0.129955	0.375201	0.000768	0.635639	0.141818	0.137936
0.15777	0.305974	0.199795	-0.38215	0.055264	-0.43315	-0.25762	0.137115	0.315043
0.076475	0.000468	0.024318	9.27E-06	0.537162	3.65E-07	0.003456	0.124243	0.000309
0.055983	0.286593	0.318507	-0.43384	-0.17976	-0.37378	-0.32772	0.251668	0.379566
0.531876	0.001088	0.000263	3.48E-07	0.043146	1.5E-05	0.000169	0.004315	1.08E-05
-0.22981	-0.21606	-0.44971	0.471899	0.062789	0.437456	0.3707	-0.33948	-0.06351
0.009347	0.014701	1.13E-07	2.14E-08	0.483123	2.71E-07	1.79E-05	9.44E-05	0.478128
-0.38428	-0.54647	-0.5131	0.573329	0.263043	0.423084	0.718081	-0.3593	-0.4362
8.18E-06	3.01E-11	6.92E-10	1.86E-12	0.00281	7.21E-07	2.07E-21	3.35E-05	2.96E-07
-0.15578	-0.38069	-0.63239	0.537597	0.539819	0.200011	0.633898	-0.22805	-0.24422
0.080302	1.01E-05	1.53E-15	7.17E-11	5.79E-11	0.024161	1.25E-15	0.00992	0.005656
-0.26224	-0.26753	-0.49716	0.372509	0.489999	0.122686	0.544003	-0.30618	-0.13734
0.002898	0.00236	2.75E-09	1.61E-05	5.01E-09	0.169402	3.84E-11	0.000464	0.123612
-0.14999	-0.14107	-0.41172	0.348984	0.320916	0.294301	0.135443	-0.35624	-0.17444
0.09235	0.11366	1.52E-06	5.79E-05	0.000235	0.000784	0.128946	3.95E-05	0.049831
-0.40395	-0.65521	-0.52184	0.686188	0.219349	0.600952	0.47346	-0.33657	-0.55317
2.48E-06	6.42E-17	3.15E-10	5.44E-19	0.013221	8.05E-14	1.89E-08	0.000109	1.54E-11
-0.07689	-0.03312	0.191794	0.032568	-0.1868	0.20799	-0.20261	0.118479	-0.03783
0.390213	0.711672	0.030762	0.716236	0.035474	0.018952	0.022342	0.184619	0.672802
-0.33266	-0.24215	-0.27228	0.506024	0.296705	0.56765	0.182358	-0.20543	-0.24049
0.000133	0.00609	0.001956	1.29E-09	0.000706	3.42E-12	0.040171	0.020505	0.00646
0.112379	-0.37741	-0.09205	0.134188	-0.16459	0.086262	0.08214	-0.04883	-0.52531
0.208421	1.22E-05	0.303345	0.132559	0.064445	0.334888	0.358583	0.585641	2.29E-10

PLA2G6	PLAC1	PLAG1	PLBD2	PLCD3	PLEC	PLEKHM2	PLGRKT	PLK5
cyan	cyan	cyan	cyan	cyan	cyan	cyan	cyan	cyan
0.154995	-0.10141	0.208454	0.238686	-0.0492	-0.18868	0.083054	-0.0085	-0.09026
0.081865	0.256604	0.018681	0.006883	0.582826	0.033632	0.353241	0.924432	0.312865
0.16178	-0.04402	0.178555	0.143135	-0.08517	-0.25144	0.061523	0.001975	-0.02994
0.069196	0.623144	0.044591	0.10841	0.341068	0.004351	0.492002	0.982418	0.73824
-0.41728	0.445979	-0.12008	-0.4954	-0.16528	-0.14014	-0.42199	0.197869	-0.02698
1.06E-06	1.48E-07	0.178708	3.19E-09	0.063314	0.116088	7.76E-07	0.025753	0.763377
-0.55448	0.518463	0.325407	-0.51755	-0.37835	-0.4435	-0.73133	0.403182	-0.3728
1.35E-11	4.28E-10	0.000189	4.64E-10	1.16E-05	1.77E-07	1.62E-22	2.6E-06	1.59E-05
-0.26257	0.372194	0.149504	-0.37917	-0.33426	-0.63661	-0.35535	0.636937	-0.18059
0.002861	1.64E-05	0.093424	1.1E-05	0.000123	8.68E-16	4.14E-05	8.31E-16	0.042178
-0.65781	0.599957	0.51328	-0.53821	-0.58105	-0.82421	-0.85731	0.763043	-0.57627
4.39E-17	9.06E-14	6.81E-10	6.76E-11	7.96E-13	1.16E-32	7.47E-38	1.89E-25	1.35E-12
-0.32712	0.358353	0.321448	-0.23999	-0.4171	-0.59889	-0.45011	0.367029	-0.3026
0.000174	3.52E-05	0.000229	0.006575	1.07E-06	1.03E-13	1.1E-07	2.19E-05	0.000545
-0.05847	0.020876	0.138244	-0.1643	-0.04766	-0.13968	-0.34155	0.370726	-0.29573
0.513805	0.815792	0.121147	0.064925	0.594641	0.117286	8.5E-05	1.78E-05	0.000736
-0.36955	0.097459	0.098603	0.130995	-0.05842	0.11161	-0.18692	0.165517	-0.32109
1.9E-05	0.275693	0.27007	0.142111	0.514166	0.211574	0.035358	0.06293	0.000233
-0.1753	-0.09161	0.359484	0.311542	0.092872	0.013124	-0.14922	0.39679	-0.53792
0.048695	0.305666	3.31E-05	0.000363	0.299032	0.883567	0.094056	3.87E-06	6.95E-11
-0.26845	0.033678	0.360099	0.099909	-0.00027	-0.08333	-0.27588	0.361649	-0.44326
0.002276	0.706998	3.21E-05	0.263746	0.997612	0.351666	0.001693	2.95E-05	1.8E-07
0.348551	-0.1364	-0.23098	0.09001	0.379083	0.345119	0.326301	-0.43013	0.258482
5.92E-05	0.126227	0.008983	0.314235	1.11E-05	7.08E-05	0.000181	4.49E-07	0.003344
0.339757	-0.24116	-0.43156	0.16406	0.464568	0.706472	0.404293	-0.46646	0.40779
9.31E-05	0.006309	4.07E-07	0.065316	3.76E-08	1.72E-20	2.43E-06	3.25E-08	1.95E-06
0.367375	-0.28037	-0.43129	0.212037	0.515249	0.644595	0.525546	-0.10686	0.470386
2.15E-05	0.00141	4.15E-07	0.016703	5.71E-10	2.9E-16	2.24E-10	0.231767	2.4E-08
0.333367	-0.42054	-0.17852	0.47337	0.564609	0.745229	0.432915	-0.13542	0.076606
0.000128	8.54E-07	0.044635	1.9E-08	4.72E-12	9.5E-24	3.71E-07	0.129009	0.391984
0.55904	-0.39524	0.00115	0.288795	0.270886	0.242911	0.412188	-0.30921	0.212333
8.44E-12	4.25E-06	0.989762	0.000992	0.002068	0.005928	1.47E-06	0.000404	0.016548
0.396379	-0.17958	-0.56222	-0.04391	0.315814	0.379724	0.49696	-0.56692	0.63145
3.96E-06	0.04336	6.07E-12	0.624003	0.000298	1.07E-05	2.8E-09	3.7E-12	1.73E-15
-0.11735	0.116592	0.041522	-0.20789	-0.36004	-0.37991	-0.09944	-0.2256	0.149809
0.188854	0.19176	0.643004	0.01901	3.22E-05	1.06E-05	0.266011	0.010767	0.092749
0.312882	-0.24019	-0.28927	0.142179	-0.00147	0.017633	0.461238	-0.54083	0.422509
0.000342	0.006528	0.000972	0.110813	0.986925	0.844015	4.83E-08	5.24E-11	7.49E-07
0.115507	0.19659	-0.22136	-0.28703	-0.23662	-0.11475	0.026185	-0.20915	0.2258
0.195957	0.026745	0.012384	0.001069	0.007398	0.19891	0.770117	0.018281	0.010694

PLRG1	PLXNA1	PLXNB1	PLXNB3	PMPCB	PNO1	PNPLA7	PNPLA8	POC1B
cyan	cyan	cyan	cyan	cyan	cyan	cyan	cyan	cyan
-0.02613	-0.23024	-0.06664	-0.02113	-0.15687	-0.10097	0.229606	-0.14468	0.044203
0.770574	0.009212	0.456655	0.813561	0.078184	0.258659	0.009412	0.104619	0.62169
-0.08209	-0.30238	-0.0481	-0.00088	-0.08799	-0.0905	0.205681	-0.0799	0.141314
0.358881	0.00055	0.591229	0.992198	0.325261	0.311615	0.020349	0.371867	0.113025
0.228924	0.179035	-0.16272	-0.22289	0.435288	0.337603	-0.56268	0.114432	0.102143
0.009631	0.044012	0.067578	0.011778	3.15E-07	0.000104	5.79E-12	0.200178	0.25316
0.538114	-0.18182	-0.41492	-0.489	0.713529	0.605203	-0.55383	0.472522	0.499243
6.82E-11	0.040768	1.23E-06	5.43E-09	4.81E-21	4.83E-14	1.44E-11	2.04E-08	2.31E-09
0.360542	-0.56892	-0.34523	-0.43987	0.235075	0.490427	-0.37128	0.207215	0.35323
3.13E-05	2.99E-12	7.04E-05	2.29E-07	0.007806	4.83E-09	1.73E-05	0.019411	4.63E-05
0.704454	-0.6315	-0.62349	-0.7508	0.74018	0.821256	-0.62217	0.599567	0.721319
2.45E-20	1.72E-15	4.92E-15	2.89E-24	2.72E-23	2.98E-32	5.83E-15	9.49E-14	1.13E-21
0.438154	-0.43283	-0.38912	-0.42825	0.426865	0.496059	-0.3265	0.38428	0.43761
2.58E-07	3.73E-07	6.15E-06	5.1E-07	5.6E-07	3.02E-09	0.000179	8.18E-06	2.68E-07
0.467589	-0.4452	0.102582	-0.15038	0.181172	0.220525	0.068401	0.331227	0.157052
2.98E-08	1.57E-07	0.251118	0.091502	0.041508	0.012725	0.444796	0.000143	0.077842
-0.0116	0.311812	-0.31717	-0.09945	0.10963	0.21215	-0.18373	-0.08873	-0.10752
0.89703	0.000359	0.00028	0.265941	0.219841	0.016644	0.038668	0.321182	0.228903
0.227046	-0.2433	-0.10569	-0.0375	-0.14812	0.145987	0.165296	-0.0995	0.073798
0.010258	0.005847	0.236953	0.675494	0.096536	0.101481	0.063287	0.265702	0.409621
0.360168	-0.24477	-0.14468	-0.19441	0.133336	0.23629	0.018705	0.086421	0.168524
3.19E-05	0.005546	0.104619	0.028513	0.135059	0.007484	0.834656	0.333997	0.058231
-0.3788	0.485106	0.172815	0.470408	-0.14747	-0.24636	0.200945	-0.41341	-0.35321
1.13E-05	7.47E-09	0.052026	2.4E-08	0.098025	0.005238	0.023494	1.36E-06	4.64E-05
-0.40199	0.709732	0.473334	0.595209	-0.3525	-0.45392	0.253658	-0.51778	-0.55207
2.81E-06	9.58E-21	1.91E-08	1.58E-13	4.81E-05	8.33E-08	0.004008	4.55E-10	1.72E-11
-0.38058	0.449709	0.42418	0.533726	-0.56124	-0.37588	0.268324	-0.4862	-0.46828
1.02E-05	1.13E-07	6.7E-07	1.04E-10	6.72E-12	1.33E-05	0.002288	6.84E-09	2.83E-08
-0.32296	0.417112	0.467671	0.636748	-0.61243	-0.43893	0.46983	-0.5931	-0.47648
0.000213	1.07E-06	2.96E-08	8.52E-16	1.99E-14	2.44E-07	2.51E-08	2.03E-13	1.49E-08
-0.28416	-0.18783	0.578892	0.594951	-0.55016	-0.5178	0.530525	-0.34975	-0.05681
0.001205	0.034457	1.01E-12	1.63E-13	2.09E-11	4.54E-10	1.41E-10	5.56E-05	0.525829
-0.43962	0.368242	0.491099	0.46058	-0.31511	-0.47299	0.087955	-0.24586	-0.35064
2.33E-07	2.05E-05	4.57E-09	5.08E-08	0.000308	1.96E-08	0.325455	0.005333	5.31E-05
0.041443	-0.1415	-0.25509	-0.37524	0.345492	0.080528	-0.22716	0.453586	0.27789
0.64364	0.112549	0.0038	1.38E-05	6.94E-05	0.368124	0.01022	8.54E-08	0.00156
-0.35303	0.080255	0.121208	0.063475	-0.19033	-0.36139	0.186089	0.02836	-0.20463
4.68E-05	0.369755	0.17464	0.478346	0.032089	2.99E-05	0.036196	0.751622	0.021014
-0.23512	0.274824	-0.00259	0.073087	-0.04598	0.005041	-0.1966	-0.38335	-0.19507
0.007792	0.001766	0.976982	0.41416	0.607767	0.955148	0.026734	8.64E-06	0.027966

POC5	POLE3	POLR1C	POLR2H	POLR3C	POLR3H	PON2	POP5	POT1
cyan	cyan	cyan	cyan	cyan	cyan	cyan	cyan	cyan
0.106428	0.1174	-0.12987	-0.05128	-0.00609	0.115565	-0.23003	0.023672	-0.10061
0.233687	0.188677	0.145601	0.566979	0.945855	0.195731	0.009277	0.791651	0.260387
0.148069	0.200088	-0.02559	0.020345	-0.01105	0.136718	-0.12429	0.072755	-0.07596
0.096648	0.024105	0.775175	0.820397	0.901854	0.125349	0.163838	0.416289	0.395972
0.288269	0.353078	0.414338	0.309761	0.493678	-0.24	0.199247	0.205486	0.443188
0.001014	4.67E-05	1.28E-06	0.000394	3.69E-09	0.006573	0.024719	0.020471	1.81E-07
0.529787	0.485688	0.424277	0.402164	0.585354	-0.61864	0.459893	0.325702	0.783195
1.51E-10	7.13E-09	6.66E-07	2.78E-06	4.92E-13	9.14E-15	5.35E-08	0.000187	1.45E-27
0.558128	0.521825	0.553409	0.719476	0.417379	-0.16384	0.076716	0.75374	0.359188
9.28E-12	3.15E-10	1.5E-11	1.6E-21	1.05E-06	0.06569	0.391301	1.52E-24	3.37E-05
0.811895	0.712952	0.596825	0.728059	0.673504	-0.6975	0.466936	0.715427	0.892754
5.3E-31	5.34E-21	1.31E-13	3.09E-22	4.11E-18	8.2E-20	3.14E-08	3.39E-21	4.15E-45
0.506853	0.55307	0.435624	0.512695	0.372082	-0.41355	0.374719	0.481773	0.549743
1.2E-09	1.56E-11	3.08E-07	7.17E-10	1.65E-05	1.35E-06	1.42E-05	9.78E-09	2.17E-11
0.19088	0.194196	0.029873	0.18045	-0.05172	-0.16231	0.25309	0.325716	0.334127
0.031583	0.028691	0.738831	0.042341	0.563641	0.068284	0.004093	0.000186	0.000123
0.051824	0.12094	0.07351	0.139249	0.135305	-0.36323	-0.05155	0.099836	0.222519
0.562833	0.1756	0.411452	0.118439	0.129338	2.7E-05	0.564931	0.264094	0.011921
0.09667	0.069094	-0.16351	0.144333	-0.10986	-0.25296	-0.09731	0.316478	0.099677
0.279617	0.440186	0.066233	0.105456	0.218883	0.004113	0.276408	0.000289	0.26486
0.155443	0.172077	-0.06281	0.138486	0.095477	-0.2774	0.132603	0.266465	0.315939
0.080974	0.053053	0.48294	0.120491	0.285623	0.001592	0.137237	0.00246	0.000296
-0.3279	-0.20661	0.017195	-0.27814	-0.03193	0.431284	-0.12437	-0.27574	-0.37306
0.000168	0.019775	0.847838	0.001545	0.721549	4.15E-07	0.163565	0.001703	1.56E-05
-0.4793	-0.40279	-0.25593	-0.38416	-0.24296	0.376255	-0.26765	-0.41725	-0.40741
1.19E-08	2.67E-06	0.003682	8.24E-06	0.005918	1.3E-05	0.002349	1.06E-06	2E-06
-0.41921	-0.43456	-0.13686	-0.0602	-0.37486	0.419492	-0.41392	-0.07387	-0.47783
9.32E-07	3.31E-07	0.124952	0.501347	1.41E-05	9.15E-07	1.32E-06	0.40918	1.34E-08
-0.46546	-0.46466	-0.43589	-0.27861	-0.49652	0.283702	-0.38166	-0.16278	-0.47111
3.51E-08	3.73E-08	3.02E-07	0.001515	2.91E-09	0.001228	9.54E-06	0.067467	2.27E-08
-0.31905	-0.35611	-0.45685	-0.47615	-0.50389	0.465527	-0.21982	-0.29582	-0.60738
0.000256	3.97E-05	6.71E-08	1.53E-08	1.55E-09	3.49E-08	0.013022	0.000734	3.71E-14
-0.46102	-0.30531	-0.15382	-0.42213	-0.22561	0.590727	-0.10589	-0.49225	-0.54751
4.91E-08	0.000482	0.084233	7.69E-07	0.010762	2.66E-13	0.236056	4.15E-09	2.72E-11
0.089931	0.090285	0.090425	-0.12928	0.144776	-0.1048	0.244431	-0.25848	0.131124
0.314661	0.312754	0.312002	0.14744	0.104381	0.240959	0.005614	0.003345	0.141715
-0.32082	-0.23235	-0.18848	-0.40117	-0.25043	0.411487	-0.09324	-0.4631	-0.44772
0.000236	0.008572	0.033825	2.95E-06	0.004516	1.54E-06	0.29713	4.2E-08	1.31E-07
0.193241	0.171145	0.202496	0.113577	0.330212	0.162124	-0.33529	-0.06637	-0.13867
0.0295	0.054372	0.022421	0.203581	0.00015	0.0686	0.000117	0.458474	0.119999

PPHLN1	PPIE	PPIH	PPIL1	PPIL3	PPIL4	PPP1R26	PPP1R37	PPP1R8
cyan	cyan	cyan	cyan	cyan	cyan	cyan	cyan	cyan
0.101559	-0.0514	-0.0425	0.079242	0.03691	0.010191	-0.21987	-0.14493	-0.14988
0.255898	0.566065	0.635165	0.375847	0.680356	0.909469	0.013001	0.104002	0.092604
0.176183	-0.00544	0.025659	0.17488	0.052826	-0.04233	-0.21748	-0.12084	-0.04792
0.047548	0.951566	0.774614	0.049241	0.555293	0.636521	0.014043	0.17597	0.592655
0.148865	0.19457	0.367774	0.20675	0.293904	0.276008	0.089306	-0.22505	0.542695
0.094849	0.028379	2.1E-05	0.019692	0.000797	0.001684	0.318052	0.010963	4.37E-11
0.372858	0.306194	0.326247	0.259122	0.56238	0.62769	-0.32713	-0.63937	0.642646
1.58E-05	0.000464	0.000182	0.003264	5.97E-12	2.85E-15	0.000174	5.97E-16	3.8E-16
0.498548	0.726551	0.805879	0.496209	0.582595	0.349139	-0.54068	-0.19775	0.45539
2.45E-09	4.14E-22	3.11E-30	2.98E-09	6.7E-13	5.74E-05	5.32E-11	0.025843	7.47E-08
0.690965	0.641892	0.676619	0.540572	0.832783	0.823138	-0.7538	-0.77511	0.735657
2.47E-19	4.22E-16	2.53E-18	5.38E-11	6.76E-34	1.64E-32	1.5E-24	1.09E-26	6.84E-23
0.410308	0.556764	0.499915	0.298317	0.620749	0.392058	-0.47885	-0.54456	0.563537
1.66E-06	1.07E-11	2.18E-09	0.000658	6.99E-15	5.15E-06	1.24E-08	3.64E-11	5.29E-12
0.147765	0.353168	0.092314	0.210804	0.314432	0.385371	-0.28976	-0.45241	0.073068
0.097343	4.65E-05	0.301957	0.017362	0.000318	7.68E-06	0.000952	9.3E-08	0.414281
-0.08672	-0.04917	-0.00687	-0.12772	0.083964	0.252102	-0.14796	-0.19939	0.084929
0.332298	0.583051	0.938864	0.152438	0.347975	0.004246	0.09689	0.024611	0.342439
-0.02889	0.137279	-0.02599	0.006317	0.147252	0.315124	-0.52913	-0.3246	-0.1383
0.747173	0.123789	0.771778	0.943808	0.098522	0.000308	1.6E-10	0.000197	0.120989
0.01945	0.112687	-0.01938	0.129442	0.247666	0.410919	-0.41344	-0.49037	-0.0105
0.828177	0.207172	0.828754	0.14694	0.004995	1.6E-06	1.36E-06	4.85E-09	0.906759
-0.3641	-0.29309	-0.25396	-0.22432	-0.36854	-0.41418	0.389103	0.365149	-0.1251
2.58E-05	0.000825	0.003963	0.011234	2.02E-05	1.29E-06	6.15E-06	2.43E-05	0.161095
-0.60883	-0.3856	-0.3975	-0.4299	-0.46538	-0.44632	0.66873	0.447639	-0.23978
3.11E-14	7.57E-06	3.7E-06	4.56E-07	3.53E-08	1.45E-07	8.58E-18	1.32E-07	0.006625
-0.57639	-0.05675	-0.09076	-0.31412	-0.45015	-0.45509	0.450212	0.74242	-0.3293
1.33E-12	0.526229	0.310199	0.000323	1.1E-07	7.64E-08	1.09E-07	1.71E-23	0.000157
-0.64645	-0.26787	-0.42624	-0.42342	-0.45609	-0.30234	0.255978	0.381481	-0.47973
2.24E-16	0.002328	5.84E-07	7.05E-07	7.1E-08	0.000551	0.003675	9.64E-06	1.15E-08
-0.2217	-0.24475	-0.41824	-0.08049	-0.43405	-0.47536	0.110943	0.318917	-0.41947
0.012244	0.005549	9.94E-07	0.368373	3.43E-07	1.63E-08	0.214333	0.000258	9.17E-07
-0.31294	-0.31658	-0.22393	-0.12816	-0.48417	-0.62773	0.688648	0.577002	-0.18655
0.000341	0.000288	0.011381	0.151029	8.06E-09	2.83E-15	3.63E-19	1.25E-12	0.035725
0.365301	-0.12253	0.010959	0.143265	0.035374	-0.02147	0.060758	-0.06236	0.107646
2.41E-05	0.169934	0.902675	0.108087	0.692974	0.81068	0.49741	0.486099	0.228351
0.026048	-0.30426	-0.20364	-0.05486	-0.34911	-0.50798	0.364785	0.394896	-0.31087
0.771286	0.000506	0.021659	0.540114	5.75E-05	1.09E-09	2.48E-05	4.34E-06	0.000375
0.093471	0.054647	0.288701	-0.10327	0.132587	-0.13087	0.229442	0.15296	0.252702
0.29591	0.541721	0.000996	0.24792	0.137287	0.142486	0.009464	0.086007	0.004153

PPP1R9B	PPP2R1A	PPP2R3C	PPP2R5B	PPP5C	PPP6R1	PPP6R2	PPT1	PPWD1
cyan	cyan	cyan	cyan	cyan	cyan	cyan	cyan	cyan
-0.0431	-0.11376	0.182361	0.092029	-0.4393	-0.09516	-0.1018	0.431543	-0.0677
0.630422	0.202835	0.040167	0.303459	2.38E-07	0.287255	0.254771	4.08E-07	0.449461
-0.07506	-0.00159	0.293289	0.194185	-0.36277	-0.12566	-0.14266	0.351774	-0.09732
0.40165	0.985887	0.000819	0.0287	2.77E-05	0.159221	0.109611	5E-05	0.276385
-0.31823	-0.0691	0.227165	-0.39233	0.276942	-0.20636	-0.13125	-0.22341	0.369127
0.000266	0.440171	0.010218	5.07E-06	0.001622	0.019928	0.14134	0.011577	1.95E-05
-0.691	-0.57427	0.333673	-0.67432	-0.19355	-0.62474	-0.5456	0.198918	0.620833
2.46E-19	1.68E-12	0.000126	3.63E-18	0.029232	4.18E-15	3.28E-11	0.024963	6.92E-15
-0.50545	-0.20851	0.581092	-0.14951	-0.27261	-0.47693	-0.34261	0.349654	0.205822
1.35E-09	0.018651	7.93E-13	0.093418	0.00193	1.44E-08	8.05E-05	5.59E-05	0.020261
-0.94418	-0.75897	0.637985	-0.69661	-0.53427	-0.90389	-0.79156	0.561045	0.611421
3.95E-62	4.76E-25	7.21E-16	9.55E-20	9.87E-11	6.24E-48	1.64E-28	6.86E-12	2.26E-14
-0.62643	-0.32516	0.372269	-0.36241	-0.34078	-0.51972	-0.49959	0.383997	0.376942
3.36E-15	0.000191	1.63E-05	2.83E-05	8.84E-05	3.82E-10	2.24E-09	8.32E-06	1.25E-05
-0.31973	-0.46609	-0.18007	-0.36365	-0.32637	-0.43097	-0.41092	0.424017	0.170322
0.000248	3.35E-08	0.04278	2.64E-05	0.000181	4.24E-07	1.6E-06	6.78E-07	0.05556
-0.1391	-0.2407	-0.06486	-0.39656	-0.1434	0.050481	-0.18155	0.082534	0.152611
0.118844	0.006412	0.468795	3.92E-06	0.107763	0.573011	0.041079	0.356275	0.086734
-0.20698	-0.53888	-0.02942	-0.40159	-0.62968	-0.1086	-0.23067	0.582787	0.123976
0.019552	6.34E-11	0.742677	2.88E-06	2.19E-15	0.224228	0.009077	6.56E-13	0.164929
-0.33355	-0.6209	0.04927	-0.41759	-0.58072	-0.27263	-0.31788	0.655631	0.164054
0.000127	6.86E-15	0.582259	1.04E-06	8.26E-13	0.001929	0.000271	6.04E-17	0.065325
0.352935	0.454821	-0.07693	0.407137	0.360447	0.50522	0.426546	-0.25369	-0.25852
4.71E-05	7.8E-08	0.389987	2.03E-06	3.15E-05	1.38E-09	5.72E-07	0.004003	0.003339
0.540569	0.479155	-0.45631	0.226837	0.555031	0.656481	0.585123	-0.55219	-0.14827
5.38E-11	1.21E-08	6.98E-08	0.01033	1.27E-11	5.33E-17	5.05E-13	1.7E-11	0.096187
0.591672	0.471932	-0.37021	0.423167	0.460464	0.678287	0.581419	-0.46813	-0.39035
2.39E-13	2.13E-08	1.84E-05	7.17E-07	5.12E-08	1.94E-18	7.64E-13	2.86E-08	5.71E-06
0.502811	0.147395	-0.51426	0.142246	0.051381	0.634574	0.459399	-0.1249	-0.24267
1.7E-09	0.098194	6.24E-10	0.110644	0.566181	1.14E-15	5.55E-08	0.161784	0.005978
0.433828	0.305096	-0.16907	0.536535	-0.07042	0.305405	0.383102	-0.01978	-0.27285
3.48E-07	0.000487	0.057414	7.94E-11	0.431455	0.00048	8.77E-06	0.825265	0.001912
0.59608	0.777782	-0.20497	0.600698	0.714748	0.509096	0.6186	-0.63099	-0.36539
1.43E-13	5.63E-27	0.020796	8.29E-14	3.84E-21	9.85E-10	9.19E-15	1.84E-15	2.4E-05
-0.08955	0.072013	0.190278	0.093014	0.138016	-0.28905	-0.18078	-0.14132	-0.01776
0.316731	0.421067	0.032134	0.298289	0.121766	0.000981	0.041954	0.113014	0.842875
0.426783	0.564373	-0.09067	0.572193	0.360509	0.232571	0.286306	-0.29735	-0.43617
5.63E-07	4.84E-12	0.310661	2.1E-12	3.13E-05	0.008508	0.001102	0.000686	2.96E-07
0.035754	0.370741	0.265671	0.055478	0.279347	0.112891	0.287044	-0.45096	0.27422
0.689839	1.78E-05	0.002538	0.535584	0.00147	0.206343	0.001068	1.03E-07	0.00181

PRCP	PRDM5	PRDX2	PRDX4	PREX1	PRIM2	PRIMPOL	PRKACA	PRKRA
cyan	cyan	cyan	cyan	cyan	cyan	cyan	cyan	cyan
0.231978	-0.19425	-0.1581	-0.05614	-0.12739	-0.04522	-0.11879	0.025322	-0.00371
0.008683	0.028644	0.075861	0.530725	0.153521	0.613691	0.183475	0.777493	0.966977
0.237581	-0.23545	-0.02424	-0.06091	-0.14596	-0.03756	-0.12931	0.040478	0.114826
0.007155	0.007705	0.786784	0.49633	0.101541	0.675031	0.147345	0.651383	0.198624
0.040929	0.30608	-0.12431	0.388178	-0.10692	0.60964	0.439256	-0.28992	0.350526
0.647756	0.000466	0.163775	6.5E-06	0.231515	2.81E-14	2.39E-07	0.000946	5.34E-05
0.366172	0.648495	-0.59246	0.5981	-0.3227	0.763254	0.667098	-0.56715	0.406085
2.3E-05	1.68E-16	2.18E-13	1.13E-13	0.000215	1.8E-25	1.1E-17	3.61E-12	2.17E-06
0.360319	-0.01206	0.093868	0.517497	-0.52057	0.345915	0.434839	-0.45725	0.561365
3.17E-05	0.892959	0.293856	4.67E-10	3.53E-10	6.79E-05	3.25E-07	6.51E-08	6.63E-12
0.612411	0.56579	-0.58483	0.801829	-0.64723	0.804583	0.811447	-0.76596	0.653363
2E-14	4.17E-12	5.21E-13	9.86E-30	2.01E-16	4.51E-30	6.06E-31	9.62E-26	8.38E-17
0.435416	0.370682	-0.29839	0.476518	-0.3691	0.673328	0.397419	-0.31482	0.381767
3.12E-07	1.79E-05	0.000656	1.49E-08	1.95E-05	4.23E-18	3.72E-06	0.000312	9.48E-06
0.343168	0.374544	-0.31187	0.197583	-0.23706	0.102285	0.314748	-0.30285	0.004769
7.82E-05	1.44E-05	0.000358	0.025972	0.007285	0.252497	0.000313	0.000539	0.95756
0.11877	0.216114	-0.34615	0.53857	0.013595	0.344532	0.23271	0.044701	-0.13301
0.183534	0.014675	6.71E-05	6.53E-11	0.879419	7.3E-05	0.008468	0.617762	0.136012
0.343703	0.135406	-0.45284	0.400313	0.071014	0.069578	0.167326	-0.136	-0.24702
7.61E-05	0.129049	9.02E-08	3.11E-06	0.427558	0.436983	0.060067	0.12737	0.005114
0.424667	0.331015	-0.60437	0.342239	-0.04879	0.234372	0.27055	-0.28962	-0.12603
6.49E-07	0.000144	5.34E-14	8.21E-05	0.585973	0.007998	0.002095	0.000958	0.157981
-0.17304	-0.32128	0.321856	-0.19882	0.329835	-0.21305	-0.27875	0.419753	-0.27436
0.051722	0.000231	0.000224	0.025036	0.000153	0.016175	0.001506	8.99E-07	0.001799
-0.42172	-0.16831	0.271406	-0.27546	0.604053	-0.19992	-0.34075	0.437988	-0.43322
7.9E-07	0.058552	0.002025	0.001722	5.55E-14	0.024224	8.85E-05	2.61E-07	3.63E-07
-0.37503	-0.39589	0.562915	-0.16677	0.484784	-0.39804	-0.27395	0.325946	-0.37348
1.4E-05	4.08E-06	5.64E-12	0.060934	7.67E-09	3.58E-06	0.00183	0.000184	1.53E-05
-0.19958	-0.24659	0.099386	-0.09152	0.657176	-0.36624	-0.31982	0.365369	-0.6343
0.024476	0.005194	0.266265	0.30616	4.82E-17	2.29E-05	0.000247	2.4E-05	1.19E-15
-0.24298	-0.5242	0.298196	-0.6197	0.449374	-0.60702	-0.56078	0.284763	-0.28885
0.005913	2.53E-10	0.000661	7.99E-15	1.16E-07	3.87E-14	7.05E-12	0.001175	0.000989
-0.50715	-0.43807	0.626248	-0.67534	0.386831	-0.40273	-0.49061	0.392405	-0.10347
1.17E-09	2.59E-07	3.44E-15	3.09E-18	7.04E-06	2.68E-06	4.76E-09	5.05E-06	0.246997
-0.07921	0.088136	0.038589	-0.32476	-0.37406	-0.00935	-0.02089	-0.0918	0.309587
0.376062	0.324459	0.666658	0.000195	1.48E-05	0.916949	0.815677	0.304649	0.000397
-0.34689	-0.37081	0.507699	-0.69623	-0.06911	-0.51261	-0.50325	0.385025	-0.01909
6.46E-05	1.77E-05	1.11E-09	1.02E-19	0.440103	7.22E-10	1.64E-09	7.83E-06	0.831296
-0.28889	-0.34278	0.206566	0.018907	-0.02074	0.222542	-0.03751	0.020294	0.335245
0.000988	7.98E-05	0.019803	0.832905	0.817005	0.011912	0.675465	0.820841	0.000117

PRMT3	PRPF39	PRR12	PRRT3	PRRT4	PSMA3	PSMA4	PSMB2	PSMC2
cyan	cyan	cyan	cyan	cyan	cyan	cyan	cyan	cyan
-0.25183	-0.06398	-0.22067	0.061638	-0.21683	0.263547	0.164767	0.059391	-0.00856
0.004289	0.474823	0.012664	0.491195	0.014343	0.002755	0.06415	0.507156	0.923879
-0.1704	-0.00053	-0.27349	0.079848	-0.14635	0.252702	0.142927	0.128015	0.006606
0.055449	0.995292	0.001864	0.372197	0.10062	0.004153	0.10893	0.151486	0.941238
0.38461	0.409745	-0.04363	-0.46595	0.117445	0.153027	0.134556	0.290417	0.198308
8.03E-06	1.72E-06	0.626218	3.38E-08	0.188508	0.085868	0.131493	0.000926	0.02542
0.627672	0.675317	-0.43196	-0.66978	-0.28552	0.397145	0.228119	0.281716	0.518492
2.85E-15	3.1E-18	3.96E-07	7.31E-18	0.001138	3.78E-06	0.009896	0.001334	4.27E-10
0.175235	0.196773	-0.65928	-0.28114	-0.23498	0.641322	0.635795	0.504368	0.543717
0.048775	0.026602	3.54E-17	0.001366	0.007831	4.56E-16	9.7E-16	1.49E-09	3.95E-11
0.647662	0.658928	-0.88857	-0.70835	-0.52653	0.809247	0.645257	0.541938	0.851277
1.89E-16	3.73E-17	3.98E-44	1.23E-20	2.04E-10	1.16E-30	2.65E-16	4.71E-11	8.16E-37
0.328083	0.355389	-0.63016	-0.50685	-0.35735	0.495619	0.303487	0.504214	0.532922
0.000166	4.13E-05	2.06E-15	1.2E-09	3.72E-05	3.14E-09	0.000523	1.51E-09	1.12E-10
0.281322	-0.01008	-0.35786	-0.17684	-0.11816	0.171925	0.199567	0.039716	0.368998
0.001356	0.910417	3.62E-05	0.046711	0.185827	0.053266	0.024484	0.657528	1.96E-05
-0.04405	0.016006	0.070053	-0.44005	-0.21708	0.193966	0.169754	0.15633	0.207673
0.622935	0.858246	0.433856	2.26E-07	0.014225	0.028883	0.056393	0.079236	0.019139
-0.099	-0.15591	-0.22303	-0.37846	-0.39907	0.432522	0.416716	0.084976	0.319261
0.268146	0.080063	0.011721	1.15E-05	3.36E-06	3.81E-07	1.1E-06	0.342172	0.000254
0.158542	0.01453	-0.28545	-0.39007	-0.45243	0.462351	0.371706	0.053489	0.385053
0.075028	0.871197	0.001142	5.81E-06	9.29E-08	4.44E-08	1.69E-05	0.550333	7.82E-06
-0.32009	-0.25723	0.495971	0.209718	0.182856	-0.46439	-0.48866	-0.17146	-0.39839
0.000244	0.003507	3.04E-09	0.017962	0.03962	3.81E-08	5.59E-09	0.053924	3.51E-06
-0.30369	-0.25475	0.79257	0.129176	0.469981	-0.56014	-0.49905	-0.32704	-0.60048
0.000519	0.003848	1.25E-28	0.147779	2.48E-08	7.53E-12	2.35E-09	0.000175	8.52E-14
-0.3916	-0.46004	0.661111	0.305117	0.439169	-0.33741	-0.17337	-0.32021	-0.4065
5.3E-06	5.29E-08	2.7E-17	0.000487	2.4E-07	0.000105	0.051268	0.000243	2.11E-06
-0.44824	-0.51136	0.621346	0.065159	0.187301	-0.25446	-0.15613	-0.3354	-0.37866
1.26E-07	8.07E-10	6.48E-15	0.466727	0.034979	0.003891	0.079618	0.000116	1.13E-05
-0.33578	-0.32931	0.207367	0.468724	0.183606	-0.28105	-0.18737	-0.25112	-0.39094
0.000114	0.000157	0.01932	2.73E-08	0.038803	0.001371	0.034913	0.004403	5.51E-06
-0.20999	-0.24972	0.556573	0.498616	0.598602	-0.63629	-0.54178	-0.24723	-0.59799
0.017809	0.004635	1.09E-11	2.43E-09	1.06E-13	9.07E-16	4.78E-11	0.005075	1.14E-13
0.270289	0.316254	-0.22837	0.273741	-0.00513	-0.12906	-0.14653	-0.01078	0.033985
0.002117	0.000292	0.009814	0.001845	0.954396	0.148135	0.100205	0.904279	0.704454
-0.24411	-0.22717	0.155157	0.68603	0.26258	-0.45263	-0.36756	-0.16948	-0.38564
0.005679	0.010217	0.081543	5.59E-19	0.00286	9.16E-08	2.13E-05	0.056793	7.56E-06
-0.25269	0.229148	0.106697	-0.08682	0.192678	-0.04507	-0.04529	0.235501	-0.29746
0.004154	0.009558	0.232501	0.331783	0.029986	0.614856	0.613115	0.007692	0.000683

PSMD12	PSMD14	PSMD6	PSMG2	PSMG3	PSORS1C1	PSPH	PTCD2	PTGES3
cyan	cyan	cyan	cyan	cyan	cyan	cyan	cyan	cyan
-0.02956	0.087956	0.072427	0.172024	-0.10679	0.109322	-0.15234	0.08402	-0.0462
0.741515	0.325449	0.4184	0.053127	0.232111	0.221147	0.08731	0.347651	0.605981
-0.06365	0.155146	0.1003	0.189018	0.002809	0.118616	-0.13456	0.126826	0.008611
0.477144	0.081563	0.261871	0.033313	0.974994	0.184109	0.131468	0.155355	0.923453
0.35344	0.079153	0.077205	0.157874	0.316866	0.221774	0.434275	0.183789	0.322507
4.58E-05	0.376384	0.38828	0.07628	0.000284	0.012216	3.38E-07	0.038605	0.000217
0.633444	0.35096	0.268986	0.363299	0.291025	0.457049	0.551174	0.498729	0.605659
1.33E-15	5.22E-05	0.002229	2.69E-05	0.000902	6.61E-08	1.88E-11	2.41E-09	4.57E-14
0.277451	0.672937	0.526316	0.734069	0.689723	0.23237	0.35315	0.377039	0.237908
0.001588	4.49E-18	2.08E-10	9.41E-23	3.04E-19	0.008567	4.65E-05	1.25E-05	0.007074
0.73711	0.79991	0.609116	0.810858	0.586306	0.531688	0.62405	0.731686	0.69549
5.1E-23	1.69E-29	3E-14	7.23E-31	4.41E-13	1.26E-10	4.57E-15	1.51E-22	1.16E-19
0.409395	0.566429	0.443468	0.431413	0.37883	0.488971	0.348217	0.453296	0.297817
1.76E-06	3.9E-12	1.77E-07	4.11E-07	1.12E-05	5.45E-09	6.03E-05	8.72E-08	0.000673
0.224588	0.307847	0.324841	0.153879	-0.07159	0.129855	0.198234	0.349918	0.186512
0.011134	0.00043	0.000194	0.084116	0.423818	0.145643	0.025476	5.52E-05	0.035767
0.271322	-0.09344	0.059573	0.027964	-0.014	0.252367	0.13863	-0.10298	0.014712
0.002032	0.296087	0.505853	0.754979	0.875854	0.004204	0.120101	0.249277	0.869602
0.086439	0.106362	0.19459	0.259386	-0.09023	0.203091	-0.01252	-0.05443	-0.0909
0.333894	0.233982	0.028363	0.003232	0.313044	0.022021	0.888895	0.54332	0.309467
0.168854	0.183979	0.16181	0.225973	-0.0255	0.244169	0.149623	0.114275	0.202016
0.057733	0.038401	0.069145	0.010633	0.775937	0.005667	0.09316	0.2008	0.022748
-0.52901	-0.49226	-0.42569	-0.4092	-0.21442	-0.10728	-0.15803	-0.54808	-0.28002
1.62E-10	4.15E-09	6.06E-07	1.78E-06	0.015488	0.22996	0.07598	2.57E-11	0.00143
-0.39981	-0.74104	-0.4836	-0.60554	-0.38677	-0.16039	-0.20865	-0.54491	-0.41212
3.21E-06	2.28E-23	8.44E-09	4.64E-14	7.07E-06	0.071654	0.018569	3.52E-11	1.48E-06
-0.47899	-0.47528	-0.29008	-0.34195	0.020959	-0.26922	-0.1477	-0.53855	-0.45467
1.22E-08	1.64E-08	0.000939	8.33E-05	0.815075	0.002208	0.097495	6.54E-11	7.88E-08
-0.45954	-0.58832	-0.28493	-0.4039	-0.40334	-0.13837	-0.29713	-0.61323	-0.5278
5.49E-08	3.51E-13	0.001167	2.49E-06	2.58E-06	0.120793	0.000693	1.8E-14	1.81E-10
-0.54571	-0.18924	-0.11545	-0.27567	-0.34844	-0.35611	-0.44948	-0.33261	-0.34674
3.25E-11	0.0331	0.196178	0.001707	5.96E-05	3.97E-05	1.15E-07	0.000133	6.51E-05
-0.46432	-0.4224	-0.3669	-0.54354	-0.14016	-0.41999	-0.24949	-0.40535	-0.30568
3.83E-08	7.55E-07	2.21E-05	4.02E-11	0.11602	8.85E-07	0.004674	2.27E-06	0.000474
0.203636	0.241933	-0.02306	0.013478	0.116742	-0.14877	0.024499	0.348549	0.349634
0.021659	0.006138	0.796936	0.880451	0.191184	0.095059	0.784543	5.92E-05	5.6E-05
-0.27667	-0.08986	-0.22664	-0.31028	-0.13885	-0.41509	-0.35976	-0.09644	-0.18585
0.001639	0.315061	0.010399	0.000385	0.119513	1.22E-06	3.27E-05	0.28075	0.03644
0.014153	-0.19601	0.029157	0.080781	0.048377	-0.04225	-0.05406	-0.10347	-0.16232
0.874512	0.027204	0.744877	0.366616	0.589122	0.637172	0.546091	0.247009	0.06826

PTOV1	PTPRF	PTPRS	PTRH2	PUS10	PUS3	PUS7L	PWP1	PYGB
cyan	cyan	cyan	cyan	cyan	cyan	cyan	cyan	cyan
-0.21067	-0.15222	-0.23462	0.181047	-0.10803	-0.24821	-0.12796	-0.04924	-0.03413
0.017434	0.087552	0.007929	0.041651	0.226697	0.004898	0.151661	0.582473	0.70329
-0.09798	-0.14935	-0.23271	0.250293	-0.13504	-0.26599	-0.09526	-0.01914	-0.00998
0.27312	0.093763	0.008468	0.004538	0.13011	0.002507	0.28674	0.83089	0.911341
0.085252	0.046329	0.198821	0.09645	0.424752	0.65404	0.388391	0.345088	-0.31852
0.3406	0.605008	0.025035	0.280719	6.45E-07	7.6E-17	6.42E-06	7.09E-05	0.000263
-0.45248	-0.09602	-0.28701	0.316559	0.728868	0.616817	0.637776	0.623974	-0.5781
9.26E-08	0.282897	0.00107	0.000288	2.64E-22	1.15E-14	7.41E-16	4.62E-15	1.1E-12
-0.10956	-0.52946	-0.57837	0.74211	0.207823	0.302277	0.22959	0.439951	-0.39768
0.220121	1.55E-10	1.07E-12	1.83E-23	0.01905	0.000552	0.009416	2.27E-07	3.66E-06
-0.66962	-0.481	-0.78909	0.750131	0.753277	0.565917	0.683988	0.831057	-0.7345
7.5E-18	1.04E-08	3.16E-28	3.34E-24	1.69E-24	4.11E-12	7.79E-19	1.21E-33	8.64E-23
-0.31778	-0.29165	-0.57033	0.586391	0.396039	0.269021	0.347021	0.519851	-0.43882
0.000272	0.000878	2.57E-12	4.37E-13	4.05E-06	0.002226	6.42E-05	3.77E-10	2.46E-07
-0.43289	0.076512	-0.42114	0.136264	0.166473	-0.05772	0.10612	0.211451	-0.18263
3.71E-07	0.392566	8.21E-07	0.126621	0.061403	0.519226	0.235052	0.017013	0.039868
-0.18924	-0.07263	-0.05634	0.075058	0.189551	0.254403	-0.10328	0.166718	-0.09474
0.033102	0.417062	0.529241	0.401646	0.03281	0.003898	0.247879	0.061018	0.289375
-0.46204	-0.30518	-0.4666	0.208321	0.11728	-0.01555	-0.27489	0.015092	-0.04371
4.55E-08	0.000485	3.22E-08	0.018759	0.189132	0.862288	0.001762	0.866267	0.625605
-0.55011	-0.18265	-0.41477	0.142249	0.228633	0.102296	-0.04715	0.137772	-0.23105
2.1E-11	0.039844	1.25E-06	0.110637	0.009726	0.252449	0.598597	0.122435	0.008962
0.658437	0.25857	0.517402	-0.25363	-0.41827	-0.03829	-0.56877	-0.40009	0.264875
4.01E-17	0.003333	4.71E-10	0.004013	9.92E-07	0.669118	3.04E-12	3.16E-06	0.002617
0.613187	0.527113	0.826104	-0.57859	-0.27725	0.031408	-0.43535	-0.51458	0.418603
1.81E-14	1.93E-10	6.27E-33	1.05E-12	0.001602	0.725938	3.14E-07	6.07E-10	9.7E-07
0.654083	0.258598	0.566533	-0.33634	-0.40076	-0.07635	-0.54664	-0.52727	0.471804
7.55E-17	0.00333	3.85E-12	0.000111	3.03E-06	0.393573	2.96E-11	1.91E-10	2.15E-08
0.307977	0.247449	0.416805	-0.41738	-0.35469	-0.19747	-0.73459	-0.59526	0.487596
0.000428	0.005035	1.09E-06	1.05E-06	4.29E-05	0.026055	8.49E-23	1.57E-13	6.1E-09
0.219324	0.025241	0.167202	-0.23739	-0.47454	-0.5546	-0.39267	-0.54722	0.356297
0.013232	0.778183	0.060259	0.007203	1.74E-08	1.33E-11	4.97E-06	2.8E-11	3.93E-05
0.705172	0.350754	0.749357	-0.48252	-0.47378	-0.17939	-0.182	-0.46294	0.319712
2.16E-20	5.28E-05	3.95E-24	9.21E-09	1.84E-08	0.043588	0.040572	4.25E-08	0.000248
-0.16887	-0.06257	-0.10754	-0.00972	0.12929	-0.1057	0.58853	0.274739	-0.18866
0.057702	0.48468	0.228829	0.913621	0.14742	0.23693	3.43E-13	0.001772	0.033659
0.273403	0.100388	0.199781	-0.24561	-0.45038	-0.48241	0.01855	-0.2183	0.25259
0.00187	0.261449	0.024328	0.005381	1.08E-07	9.3E-09	0.836011	0.013679	0.00417
0.455885	-0.10473	0.330086	0.137805	0.046505	0.359333	0.088092	-0.03155	-0.06147
7.21E-08	0.241276	0.000151	0.122345	0.603636	3.34E-05	0.3247	0.724709	0.492388

QRSL1	R3HDM2	RAB11FIP3	RAB28	RAB33B	RAB35	RAB40C	RAB9A	RABGGTB
cyan	cyan	cyan	cyan	cyan	cyan	cyan	cyan	cyan
-0.03878	0.068718	-0.24139	0.095653	-0.03376	-0.06172	-0.16858	0.142311	0.316384
0.665142	0.442685	0.006258	0.28473	0.706314	0.490586	0.058145	0.11048	0.00029
-0.02644	0.081663	-0.25061	0.141325	-0.08832	-0.11575	-0.09289	0.172193	0.376849
0.767968	0.361388	0.004486	0.112997	0.323418	0.194992	0.298934	0.052891	1.26E-05
0.306834	-0.15547	0.011689	-0.05535	0.002076	0.093819	-0.21749	0.033893	-0.02618
0.00045	0.080915	0.89623	0.5365	0.981515	0.294108	0.01404	0.705216	0.770189
0.628981	-0.3738	-0.42262	0.393176	0.361903	-0.24072	-0.65899	0.34075	0.202906
2.4E-15	1.5E-05	7.44E-07	4.82E-06	2.91E-05	0.006409	3.69E-17	8.85E-05	0.022145
0.293649	-0.61638	-0.46495	0.524468	0.100069	-0.24744	-0.19981	0.472062	0.419072
0.000806	1.22E-14	3.65E-08	2.47E-10	0.262977	0.005036	0.024305	2.11E-08	9.41E-07
0.76482	-0.75136	-0.79491	0.784331	0.48163	-0.43505	-0.76733	0.664079	0.533304
1.25E-25	2.56E-24	6.67E-29	1.08E-27	9.9E-09	3.2E-07	6.99E-26	1.74E-17	1.08E-10
0.471471	-0.37822	-0.51663	0.513693	0.103717	-0.31275	-0.49887	0.331131	0.400186
2.21E-08	1.16E-05	5.05E-10	6.56E-10	0.245885	0.000344	2.38E-09	0.000143	3.14E-06
0.407169	-0.39452	-0.39754	0.501249	0.20761	-0.45798	-0.41044	0.324484	0.085413
2.03E-06	4.44E-06	3.69E-06	1.95E-09	0.019176	6.17E-08	1.65E-06	0.000198	0.339689
0.012485	-0.07212	-0.12195	-0.21584	-0.04731	0.219655	-0.34341	0.05726	0.056415
0.889206	0.420393	0.17201	0.014803	0.597371	0.013091	7.73E-05	0.522539	0.528706
0.088169	-0.35817	-0.41808	0.14606	-0.01454	-0.0993	-0.49435	0.41193	0.336554
0.324275	3.56E-05	1E-06	0.101309	0.871108	0.266685	3.49E-09	1.5E-06	0.000109
0.290485	-0.3263	-0.48308	0.235154	0.228923	-0.14877	-0.58517	0.381238	0.244247
0.000923	0.000181	8.81E-09	0.007784	0.009631	0.095072	5.02E-13	9.78E-06	0.005651
-0.34059	0.569192	0.303818	-0.62122	-0.30962	0.261692	0.261626	-0.23615	-0.35775
8.92E-05	2.9E-12	0.000516	6.59E-15	0.000397	0.002959	0.002967	0.00752	3.64E-05
-0.42416	0.583972	0.657548	-0.75467	-0.47497	0.417184	0.349377	-0.4681	-0.41876
6.71E-07	5.74E-13	4.57E-17	1.24E-24	1.68E-08	1.06E-06	5.67E-05	2.87E-08	9.6E-07
-0.53729	0.186089	0.610029	-0.55893	-0.30604	0.391292	0.606992	-0.25538	-0.35955
7.39E-11	0.036197	2.68E-14	8.54E-12	0.000467	5.4E-06	3.89E-14	0.003758	3.3E-05
-0.46271	0.260224	0.38356	-0.57533	-0.41178	0.297732	0.185605	-0.14273	-0.17148
4.32E-08	0.00313	8.54E-06	1.5E-12	1.51E-06	0.000675	0.036692	0.10942	0.053893
-0.32625	0.296803	0.203627	-0.09988	-0.23009	-0.11958	0.336689	-0.10055	0.020574
0.000182	0.000703	0.021665	0.263868	0.009259	0.180541	0.000109	0.260663	0.818411
-0.39679	0.501223	0.675221	-0.49323	-0.29261	0.263014	0.678241	-0.53178	-0.46722
3.87E-06	1.95E-09	3.15E-18	3.83E-09	0.000843	0.002813	1.96E-18	1.25E-10	3.07E-08
0.170631	0.027522	-0.03836	0.308733	0.420006	-0.1274	0.148413	-0.14826	-0.12327
[truncated: 513,217 more chars]
